# Iodine-DMSO mediated conversion of *N*-arylcyanothioformamides to *N*-arylcyanoformamides and the unexpected formation of 2-cyanobenzothiazoles†‡

**DOI:** 10.1039/d2ra00049k

**Published:** 2022-02-21

**Authors:** Ziad Moussa, Zaher M. A. Judeh, Ahmed Alzamly, Saleh A. Ahmed, Harbi Tomah Al-Masri, Bassam Al-Hindawi, Faisal Rasool, Sara Saada

**Affiliations:** Department of Chemistry, College of Science, United Arab Emirates University P. O. Box 15551 Al Ain United Arab Emirates zmoussa@uaeu.ac.ae; School of Chemical and Biomedical Engineering, Nanyang Technological University 62 Nanyang Drive, N1.2–B1-14 Singapore 637459 Singapore; Department of Chemistry, Faculty of Applied Sciences, Umm Al-Qura University Makkah 21955 Saudi Arabia; Department of Chemistry, Faculty of Science, Assiut University 71516 Assiut Egypt; Department of Chemistry, Faculty of Sciences, Al al-Bayt University P. O. Box 130040 Mafraq 25113 Jordan

## Abstract

Cyanoformamides are ubiquitous as useful components for assembling key intermediates and bioactive molecules. The development of an efficient and simple approach to this motif is a challenge. Herein, we demonstrate the effectiveness of the I_2_-DMSO oxidative system in the preparation of *N*-arylcyanoformamides from *N*-arylcyanothioformamides. The synthetic method features mild conditions, broad substrate scope, and high reaction efficiency. Furthermore, this method provides an excellent entry to exclusively afford 2-cyanobenzothiazoles which are useful substrates to access new luciferin analogs. The structures of all new products were elucidated by multinuclear NMR spectroscopy and high accuracy mass spectral analysis. Crystal-structure determination by means of single-crystal X-ray diffraction was carried out on (4-bromophenyl)carbamoyl cyanide, 5,6-dimethoxybenzo[*d*]thiazole-2-carbonitrile, 5-(benzyloxy)benzo[*d*]oxazole-2-carbonitrile, 4,7-dimethoxybenzo[*d*]thiazole-2-carbonitrile, and (5-iodo-2,4-dimethoxyphenyl)carbamoyl cyanide, a key intermediate with mechanistic implications.

## Abstract 2

Conversion of *N*-arylcyanothioformamides to *N*-arylcyanoformamides and 2-cyanobenzothiazoles has been achieved with I_2_-DMSO oxidative system.

## Graphical abstract

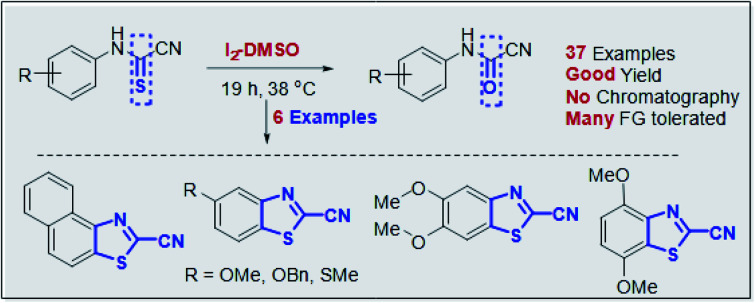

## Introduction

**Scheme 1:**
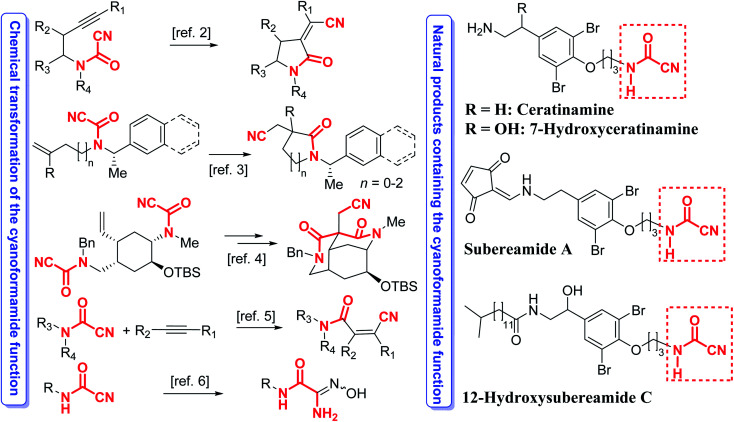
Examples of prevalence of the cyanoformamide motif in several natural products and its transformations for assembling useful intermediates and bioactive products.

Cyanoformamides are valuable and versatile building blocks used for constructing synthetically useful intermediates and many bioactive compounds. Cyanoformamides bearing an alkynyl tether undergo intramolecular cyanoamidation to produce five- to seven-membered ring α-alkylidene lactams whereas those possessing a 1,1-disubstituted alkenyl groups afford 3,3-disubstituted oxindoles having a quaternary carbon center.^1^ An enantioselective^2^ and a diastereoselective^3^ asymmetric version of the latter reaction has also been developed. The related di(cyanoformamide) precursors are also synthetically useful and their key role in the cascade cyanoamidation route to synthesize the madangamine core is noteworthy (Scheme 1).^4^ Cyanoformamides also add across alkynes by nickel/BPh_3_-catalyzed cyanocarbamoylation to give β-cyano-substituted acrylamides (Scheme 1).^5^ The synthesis of carbamoyl amidoximes from cyanoformamides^6^ and formation of β-keto Weinreb amides and unsymmetrical ketones has also been reported (Scheme 1).^7^ Likewise, cyanoformamides have been utilized in the preparation of 1,8-dihydroindeno[2,1-*b*]pyrrole-2-carboxamide and the carboxylate derivatives.^8^ Upon treatment with aluminum azide, cyanoformamides convert to the corresponding bioactive antiallergic tetrazole-5-carboxamides.^9^ Transformation of the cyanoformamide function into the tetrazol-5-carboxamide has also been achieved with Me_3_SiN_3_–Bu_2_SnO and was used to prepare 5-aryl-1,3,4-oxadiazoles used for glycogen phosphorylase *b* (RMGP*b*) inhibition.^10^ Interestingly, cyanoformamide is the nitrile derivative of formamide, a species responsible for the synthesis of nucleic acid precursors under prebiotic conditions in interstellar space.^11^ Furthermore, such a ubiquitous motif is also present in several natural products like ceratinamine,^12^ and its 7-hydroxyceratinamine derivative,^13^ subereamide A,^14^ and 12-hydroxysubereamide C (Scheme 1).^14^

**Scheme 2:**
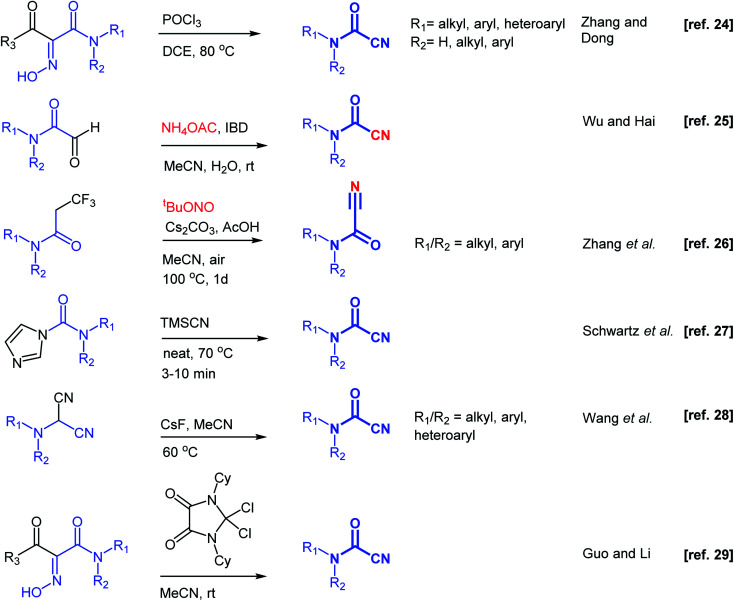
Overview of strategies towards the synthesis of cyanoformamides.

Substantial efforts have been directed toward the development of synthetic methodologies to prepare cyanoformamides. One early strategy described reacting primary and secondary amines with carbonyl cyanide. However, this method was deemed unsuitable for large scale preparation due to the production of toxic hydrogen cyanide.^15^ As an alternative, reacting the amines with triphosgene followed by substitution reaction of the resulting chlorocarbamates with cyanide ion provided acceptable yields.^15^ Several other earlier reports described the formation of cyanoformamides.^16–21^ For instance, hydration of cyanogen under high pressure using excess water gave 1-cyanoformamide,^16^ whereas reaction of 5-hydroxyimino-1,3-dioxine-4,6-dione (isonitroso Meldrum's acid) with carbodiimides (*N*,*N*′-dicyclohexylcarbodiimide and *N*,*N*′-diisopropylcarbodiimide) gave *N*-cyclohexylcyanoformamide and *N*-isopropylcyanoformamide, respectively.^17^ Some other reagents like tetracyanoethylene,^18^ 5-tosyloxyimino-2,2-dimethyl-1,3-dioxane-4,6-dione,^19^ 4-chloro-5*H*-1,2,3-dithiazol-5-one,^20^ tetraalkyl-cyanoformamidinium salt,^21^ or dichlorosulfenyl chlorides^22^ have been employed in the synthesis of similar types of synthetic compounds. Unfortunately, the structural complexity and toxicity associated with these reagents hampered their use. Recently, other more direct synthetic methods have been developed (Scheme 2).^23–29^ For instance, Muñoz reported that the reaction of primary amines with tetramethylphenylguanidine and cyanophosphonates at −10 °C under an atmosphere of CO_2_ furnishes cyanoformamides in good yields.^23^ Dong and co-workers^24^ employed phosphoryltrichloride (POCl_3_) to convert 1-acyl-1-carbamoyl oximes to cyanoformamides, while concurrently Wu and co-workers^25^ reported an eco-friendly method for the conversion of 2-oxoaldehydes into cyanoformamides using iodosobenzene diacetate (IBD) as oxidant. Zhang and co-workers described the transformation of trifluoropropanamide precursors into cyanoformamides *via* a sequence of C–CF_3_ bond breaking process and subsequent nitrogenation using *tert*-butyl nitrite as the source of nitrogen.^26^ At the same time, Schwartz's group described solvent-free access to secondary and tertiary cyanoformamides from TMSCN and carbamoyl imidazoles.^27^ Recently, cyanoformamides were prepared from *N*,*N*-disubstituted aminomalononitriles^28^ with CsF as the promoter and in another study, 4,5-dioxo-imidazolinium cation activation of 1-acyl-1-carbamoyl oximes was used.^29^ Electrochemical synthesis of cyanoformamides was also reported starting from trichloroacetonitrile and secondary amines mediated by heptamethyl cobyrinate, a B_12_ derivative.^30^ Therefore, efficient and convenient methods for the synthesis of cyanoformamides are still highly desirable.

## Results and discussion

**Scheme 3:**
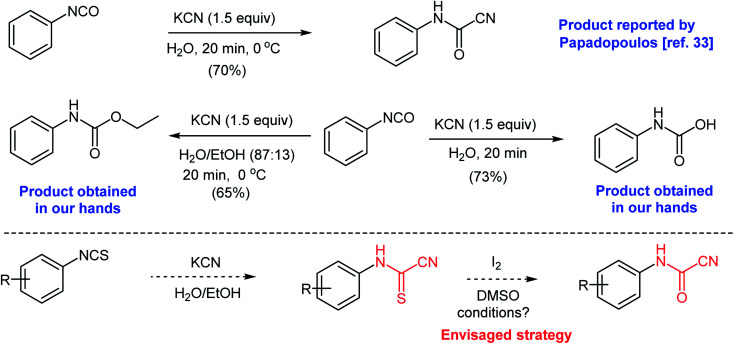
Synthesis of *N*-phenylcyanoformamide from phenyl isocyanate and potassium cyanide.

At the outset of our work, we were interested in preparing 5-imino-1,3-diphenyl-2-thioxoimidazolidin-4-ones and 5-imino-1,3-diphenyl-2-selenoxoimidazolidin-4-ones as an extension to our previous work.^31,32^ We attempted a previously reported procedure where Papadopoulos prepared *N*-phenylcyanoformamide by reacting phenyl isocyanate with potassium cyanide in water (Scheme 3).^33^ The arylcyanoformamide product was only characterized by melting point. The author noted the slow precipitation of *N*,*N*-diphenylurea upon standing of the alkaline reaction mixture due to the dissociation of the anion of *N*-phenylcyanoformamide to form phenyl isocyanate. However, in our hands, and after multiple attempts to duplicate the above method, phenyl isocyanate reacted competitively with water to produce phenylcarbamic acid (see ESI section‡). Attempts to run the same reaction using ethanol–water mixture (87 : 13) produced ethyl phenylcarbamate as the major product (Scheme 3). Clearly, the reactivity of the isocyanate group renders the preceding strategy impractical. On the contrary, isothiocyanates are less reactive and comprise more convenient precursors to prepare the cyanoformamide. Hence, we envisaged that cyanoformamides could be obtained directly from cyanothioformamides by converting the thione to the carbonyl function. Herein, we present an efficient method for the synthesis of *N*-arylcyanoformamides from *N*-arylcyanothioformamides using simple iodine-DMSO oxidative system and report unexpected formation of cyanobenzothiazoles.

Following the design in Scheme 3, several *N*-arylcyanothioformamides were prepared on large scale (20 mmol) from commercially available isothiocyanates and potassium cyanide in water–ethanol in good yields (see ESI S2–S253‡). Initially, *N*-4-tolylcyanothioformamide (1a) was selected as the model substrate (Table 1) to examine its reaction with I_2_. Ketcham and Schaumann reported that the oxidation of 1a at 80 °C using 16 mol% I_2_ produced cyanoformamide 2a in 86% yield, although they abandoned the method and opted to employ other more convenient procedures to prepare 2a.^34^ Indeed, the initial test to reproduce the formation of 2a at 80 °C using 16 mol% of the I_2_/DMSO oxidant system only resulted in partial conversion (30%) of 1a to the expected product 2a as indicated by ^1^HNMR (Table 1, entry 1). The product 2a could not be separated and purified by column chromatography from 1a as both exhibit the same R_*f*_ value. This was not surprising and presented a purification challenge for all *N*-arylcyanoformamide substrates as, they too, would likely have similar R_*f*_ values to their corresponding starting materials. Therefore, for this methodology to be useful, complete, and clean conversion of all starting material 1 to product 2 is required. Thus, further variation in the amount of iodine confirmed that 1.1 equivalent is optimal at 80 °C to completely transform 1a to 2a (Table 1, entry 4). Next, the reaction was carried out on different substrates using the optimal conditions (1.1 equiv. I_2_, 80 °C, 6 h) (Table 1, entries 5–10). Unfortunately, many substrates (1b–e) furnished the desired products 2b–e in low yields (entries 5–8), while others like 3-(fluorophenyl)carbamothioyl cyanide (1f) and 4-(nitrophenyl)carbamothioyl cyanide (1g) afforded complex mixtures (entries 9 and 10). *N*-Arylcyanothioformamides and the *N*-arylcyanoformamides products are temperature sensitive and may extrude HCN and undergo a reversible reaction to form the isothiocyanates and isocyanates, respectively, at elevated temperatures. Thus, from yield and safety perspectives, ambient conditions are better suited for both, the substrate, and product.

**Table 1:** Reaction condition studies at various temperatures using variations in the amount of I_2_ and several different *N*-arylcyanothioformamides

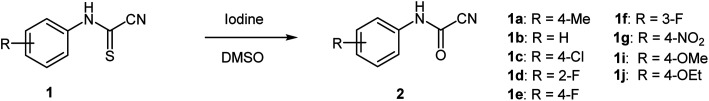						
Entry	Substrate	I_2_ (equiv.)	Temp. (°C)	Time (h)	Product	Conversion^a^ (%)/yield^b^
1	1a	0.16	80	6	2a	30^a^
2	1a	0.5	80	6	2a	38^a^
3	1a	1.0	80	6	2a	90^a^
4	1a	1.1	80	6	2a	>95^a^
5	1b	1.1	80	6	2b	39^b^
6	1c	1.1	80	6	2c	45^b^
7	1d	1.1	80	6	2d	37^b^
8	1e	1.1	80	6	2e	39^b^
9	1f	1.1	80	6	c—	d—
10	1g	1.1	80	6	c—	d—
11	1a	1.1	eRT	6	2a	10^a^
12	1a	1.1	^e^RT	19	2a	35^a^
13	1a	1.5	RT	19	2a	56^a^
14	1a	2	RT	19	2a	63^a^
15	1a	2.5	RT	19	2a	86^a^
16	1a	2.75	RT	19	2a	>95^a^
17	1i	2.75	RT	19	2i	25^a^
18	1i	3.0	RT	19	2i	36^a^
21	1i	3.5	RT	19	2i	44^a^
22	1j	3.5	RT	19	2j	27^a^
23	1j	3.5	29.5	19	2j	72^a^
24	1j	3.5	35.5	19	2j	85^a^
25	1j	3.5	37	19	2j	>95^a^
26	1j	3.5	38	19	2j	>95^a^

a Product was not isolated and percent conversion was measured by ^1^H NMR.

b Isolated yield.

c Complex mixture of products was obtained.

d Conversion could not be measured.

e RT was measured to be 20°.

Thus, with the above in mind, the best reaction conditions resulting in complete conversion of 1a at 80 °C (1.1 equiv. I_2_) were applied to *N*-4-tolylcyanothioformamide (1a) at ambient temperature (20 °C), resulting in a disappointing 10% conversion to 2a (Table 2, entry 11). Increasing reaction time from 6 h to 19 h improved conversion to 35%, (Table 2, entry 12) and additional variation in the amount of I_2_ (Table 2, entries 13–16) established that 2.75 equiv. I_2_ was required for complete conversion of 1a to 2a. With the enhanced reaction conditions in hand (2.75 equiv. I_2_, 20 °C, 19 h) (Table 2, entry 16), these were first applied to (4-methoxyphenyl)carbamothioyl cyanide (1i), resulting in 25% conversion to the target product 2i. Further increase in the amount of I_2_ to 3 and 3.5 equivalents resulted in 36% and 44% conversion to 2i, respectively. However, testing the latest conditions (3.5 equiv. I_2_, 20 °C, 19 h) on the related (4-ethoxyphenyl)carbamothioyl cyanide (1j) resulted in only 27% conversion to 2j. Potassium iodide (KI) was also explored as an alternative source of iodine in the optimization of conditions. Thus, treatment of 1j using the optimized conditions using KI (3.5 equiv. KI, 38 °C, 19 h) failed to give product 2j and the starting material was recovered unchanged. Potassium iodide (1 equimolar) was also used as a co-reagent with iodine (3.5 equiv. KI, RT, 19 h) in the preceding reaction but no enhancement in conversion was detected, suggesting that KI was not a suitable replacement for iodine. At this point, it became clear that investigating the impact of slight elevation in temperature was warranted to establish the optimal value suitable for a wide range of substrates. Thus, when 1j was treated with 3.5 equiv. I_2_ and heated at various low temperatures (29.5–38 °C) for 19 h (Table 1, entries 23–26), complete and clean conversion to 2j was observed at 37–38 °C (>95% based on the minimum detection limit of ^1^HNMR).

**Table 2:** Substrate scope investigation^a^

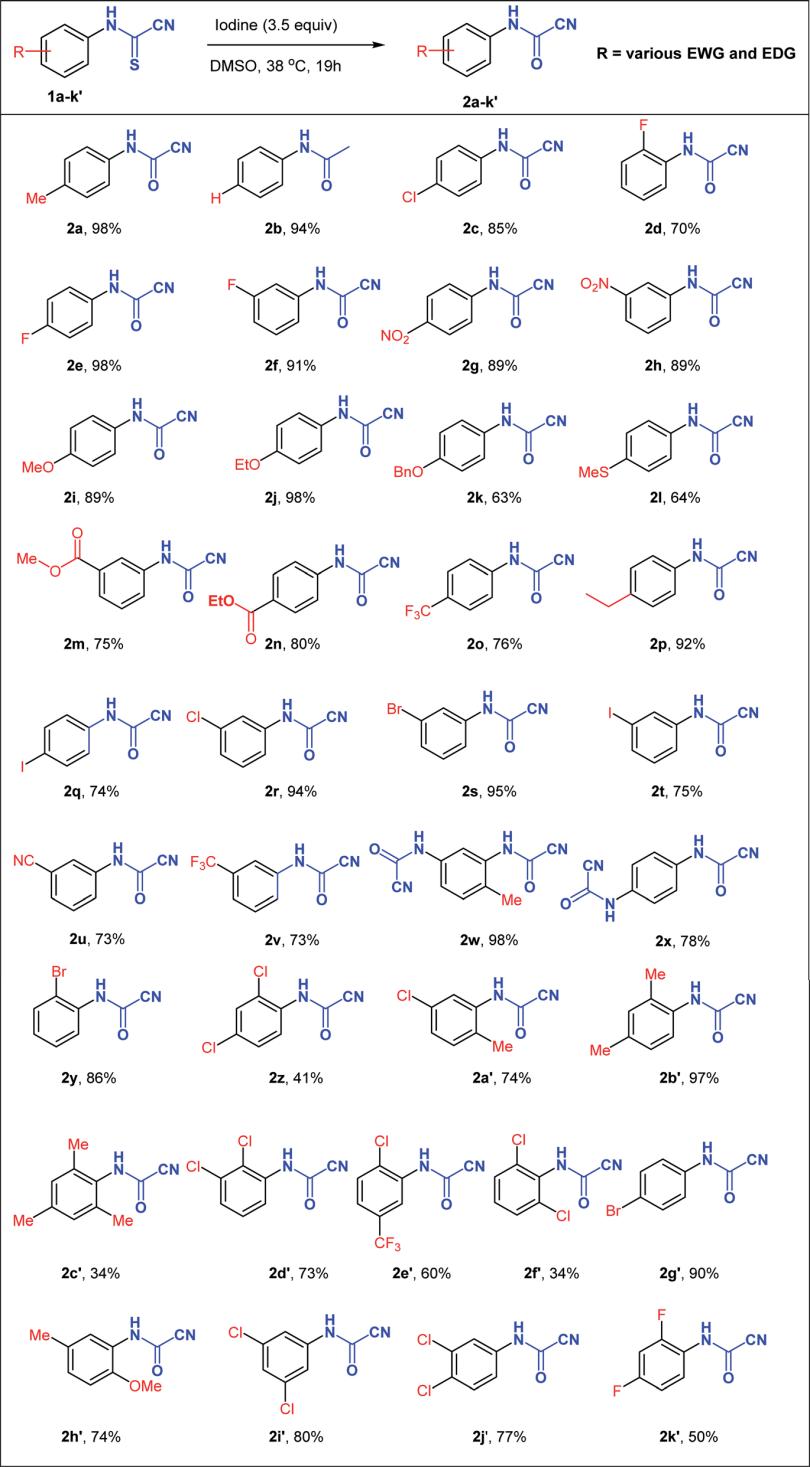

a Compound 1y was stirred for 2 d at 38 °C for complete conversion to 2y.

With the optimized reaction conditions in hand (3.5 equiv. I_2_, 38 °C, 19 h), the generality of this synthetic protocol was subsequently evaluated on a variety of *N*-arylcyanothioformamides 1a–k′ (Table 2) bearing substituents capable of displaying positive and negative mesomeric (+M, −M) and inductive (+I and –I) effects as well as imposing unfavorable sterics. Monohalogenated substrates (1c–f, 1q–t, 1y) afforded products (2c–f, 2q–t, 2y) in 70–98% yield, with the 2-F, 3-and 4-iodo-substituted starting materials generating the least yield among the series. Generally, unsubstituted, alkylated, and nitrated *N*-arylcyanothioformamides (1a, 1b, 1p, 1w, 1g, 1h, 1b′) consistently gave high yields (>89%), whereas alkoxylated ones (1i–k, 1h′) afforded variable yields (63–98%). Disubstituted substrates generated moderate to good yields (60–97%) in most cases (1a′, 1b′, 1d′, 1e′, 1h′) except for 2,4-dichloro 1z (41%), 2,4-difluoro-substituted 1k′ (50%), and 2,4-dichloro 1f′ (34%) species. The low yields are possibly due to the high solubility of the fluorinated compound in the aqueous medium and the congested environment of the chlorinated compounds, especially 1f′. Increasing the size of halogenated substituent next to the cyanothioformamide group (1d*vs.*1y; F → Br) did not reduce the yield, whereas 2,6-disubstitution (1c′ & 1f′) was detrimental. The current conditions also worked well on bis-*N*-arylcyanothioformamides (1w, 1x), producing the bis-*N*-arylcyanoformamides 2w, 2x in 98 and 78% yield, respectively. The synthesis of cyanoformamides was amenable to scale-up to gram quantities as demonstrated by the synthesis of 2a, 2e, 2f on a large scale from 1a, 1e, 1f (20 mmol scale). The products were isolated in 95%, 94%, and 87%, respectively, with yields comparable to those obtained during small scale preparation.

**Fig. 1:**
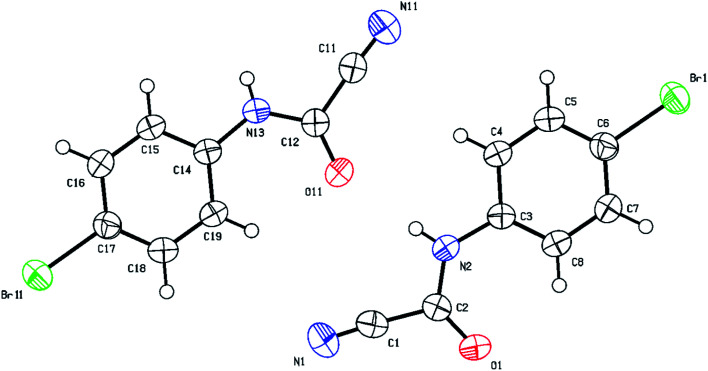
Thermal ellipsoid plots of (4-bromophenyl)carbamoyl cyanide (2g′)with ellipsoids drawn at 50% probability level. Selected bond distances (Å) and angles (deg) for compound 2g′: Br(11)–C(17) = 1.905(2), N(11)–C(11) = 1.141(4), O(1)–C(2) = 1.227(3), C(3)–C(4) = 1.393(3), O(1)–C(2)–N(2) = 127.6(2), N(2)–C(2)–C(1) = 113.2(2), C(7)–C(6)–Br(1) = 120.04(19), C(3)–C(8)–C(7) = 119.5(2), for N(11)–C(11)–C(12) = 176.3(3).

All new cyanothioformamides and cyanoformamides were characterized by standard spectroscopic and analytical techniques (mp, IR, 1D and 2D NMR, and HRMS). The physical and spectral data of known compounds matched those reported (see Experimental and ESI sections‡). The most distinctive signal to distinguish the formamide product from the cyanothioformamide starting material is that of the carbonyl (C=O) group which appears around 1700 cm^−1^ in the IR region and resonates around 140–144 ppm in the ^13^C NMR compared to approximately 160–165 ppm for the thiocarbonyl (C=S) group. Structural verification of (4-bromophenyl)carbamoyl cyanide (2g′) by single crystal X-ray crystallography, as a representative example of the cyanoformamide products, is shown in Fig. 1. Clearly, the nitrile function remained intact (exhibiting a typical linear bond angle = 176.3(3)° for N(11)–C(11)–C(12) and C(11)–N(11) bond length = 1.141(4) Å) while the thiocarbonyl has clearly been converted to the carbonyl where the bond length of O(1)–C(2) = 1.227(3) Å. Unlike their thiocarbonyl counterparts, the cyanoformamide products generally appear as one tautomer possibly due to strong intermolecular hydrogen bonding between O_11_ and N_2_–H (Fig. 1). The two molecules in the unit cell are arranged tail to tail to accommodate hydrogen bonding as shown in Fig. 1. The bond length between O_11_ and N_2_–H is 2.135 Å, indicating strong interaction.

An unexpected number of 2-cyanobenzothiazoles were formed exclusively and in good to very high yield (64–98%) when the *N*-arylcyanothioformamides 1l′–q′ (see ESI section‡) were treated in the usual way with iodine in DMSO using the optimized conditions (3.5 equiv. I_2_, 38 °C, 19 h) (Table 3). The resulting light yellow/orange/brown products could be isolated cleanly without the need for flash chromatography and are very stable at room temperature. High-resolution mass spectrometry (HRMS) and NMR measurements corroborated the suggested structures 3a–f (Table 3). Very few synthetic methods are available for the synthesis of 2-cyanobenzothiazoles which are themselves scarce in the literature. Thus, the iodine-DMSO system comprises a novel approach to access 3a–f. While 3c and 3d are unreported, the remaining analogues in the 3a–f series appear in the literature with partial (only ^1^H NMR) or even no reported NMR or physical properties data. Thus, 3a–f were extensively characterized (*vide infra* and see ESI‡).

**Table 3:** Unexpected formation of 2-cyanobenzothiazoles 3a–f from various *N*-arylcyanothioformamides and (3-iodo-4,6-dimethoxyphenyl)carbamoyl cyanide 3g

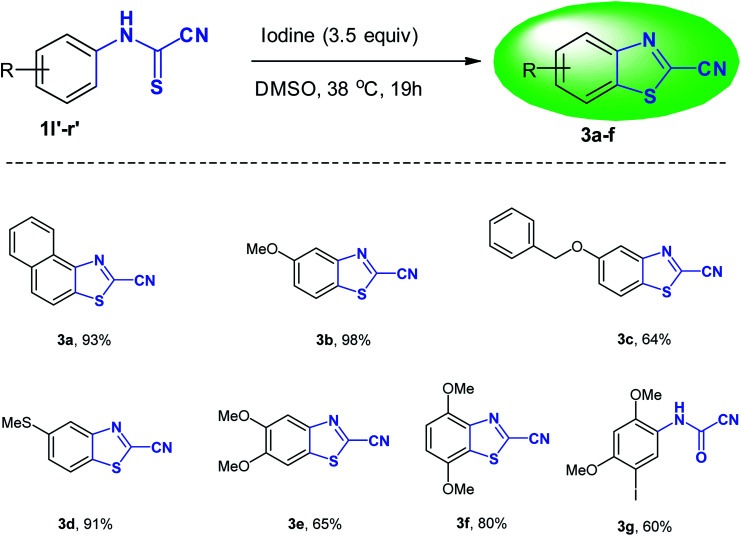

The benzothiazole nucleus has a wide profile of biological activities.^35^ In particular, the 2-cyanobenzothiazole derivatives have been recently used in self-fluorescent hyaluronic acid–based gel for dermal applications^36^ and as linkers in the development of single-molecule strategy to characterize the folded state of individual proteins during membrane translocation.^37^ Recently, 2-cyanobenzothiazole was incorporated into gold nanoparticles to enhance imaging and treatment of breast cancer^38^ and was also used in site-specific immobilization of biomolecules by reaction with terminal cysteine.^39^ Perhaps the most intriguing application of 2-cyanobenzothiazoles entails their use as precursors to access new luciferin analogs for bioluminescence imaging applications.^40–42^

On the other hand, the iodine/DMSO oxidation system has truly revolutionized synthetic practices in a plethora of reactions involving oxidation processes.^43–56^ This oxidant has been particularly used in C–N bond chemistry as a greener solution to existing conventional synthetic methodologies and to avoid employing harsh, toxic, and expensive metals and reagents. The wide and abundant availability of iodine and DMSO, ease of preparation, moisture and air stability, atom and step economy, as well as its environmentally benign nature render such system very convenient. Mechanistically, the I_2_/DMSO oxidant system has been largely described to involve prior iodination of substrates. Iodine in catalytic amount is often regenerated in the reaction from the oxidation of HI with DMSO with concurrent production of dimethyl sulfide (DMS) and is mainly reachable at higher temperatures. Notable biologically potent molecules that have been constructed through key C–N bond formation using I_2_/DMSO include α-ketoamides^46^ and α-ketoimides,^47^ imidazoles,^48^ quinoxalines,^49^ pyrazines,^49^ quinazolinones,^50^ isatin,^51^ amides,^52^ thioamides,^52^ thiazoles,^53^ triazoles,^54^ oxindoles,^55^ oxadiazoles^56^ and oxazoles.^56^

**Fig. 2:**
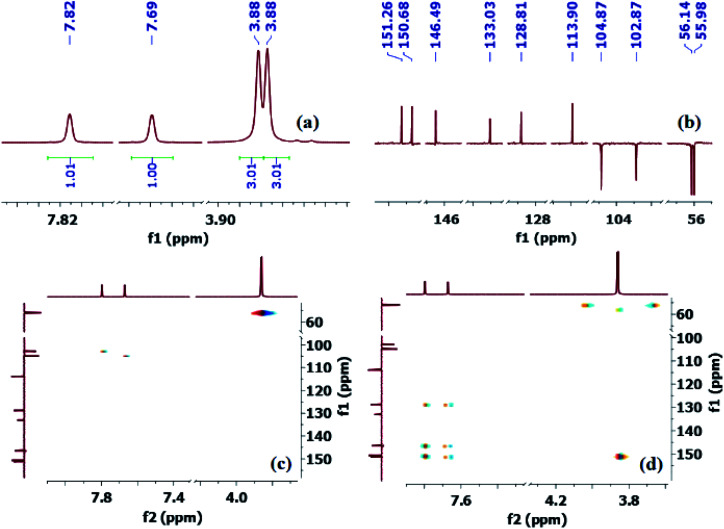
Truncated 1D and 2D NMR spectra of cyanobenzothiazole 3e: (a) ^1^H-NMR spectrum; (b) ^13^C-CRAPT NMR spectrum; (c) ^1^H-^13^C-gHSQC NMR spectrum; (d) 1H-^13^C-gHMBC NMR spectrum.

Thus, to fully confirm the chemical structures of heterocycles 3a–f (Table 2) and prove the formation of the new C–S quaternary center, extensive one-dimensional (1D) (^1^H-, ^13^C-, ^13^C-CRAPT NMR) and two-dimensional (2D) homonuclear (^1^H-^1^H-gDQCOSY) and heteronuclear (^1^H-^13^C-gHSQC, ^1^H-^13^C-gHMBC) correlation NMR spectrometry experiments were initially performed on all compounds (see ESI section‡). Hence, using 5,6-dimethoxybenzo[*d*]thiazole-2-carbonitrile (3e) as a representative model for the remaining structurally related cyanobenzothiazoles, the relevant NMR spectra that were used for structural proof and chemical shift assignment are shown in Fig. 2.

**Fig. 3:**
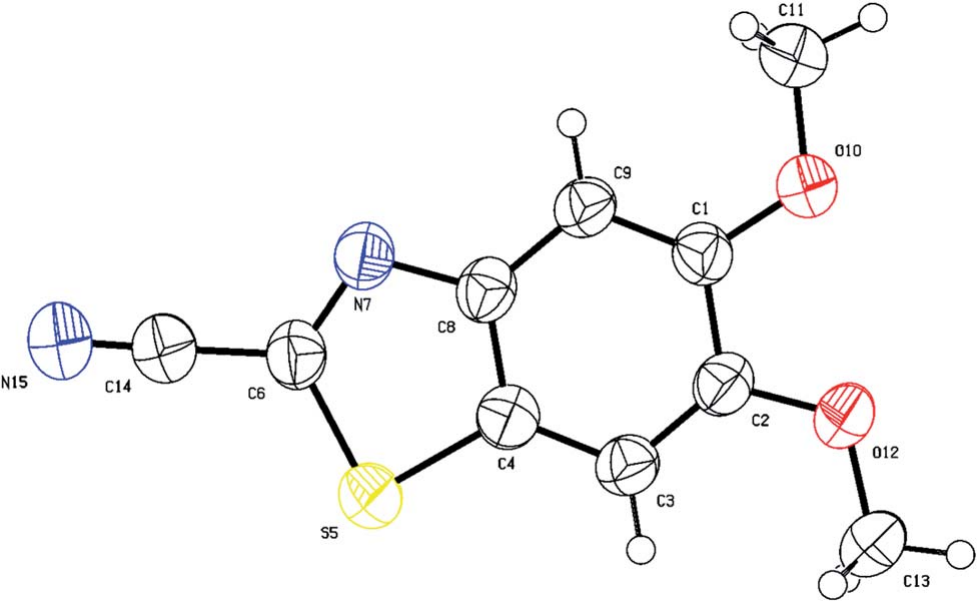
Thermal ellipsoid plots of 5,6-dimethoxybenzo[*d*]thiazole-2-carbonitrile (3e) with ellipsoids drawn at 50% probability level. Selected bond distances (Å) and angles (deg) for compound 3e: C(1)–C(2) = 1.433(3), O(10)–C(1) = 1.357(3), N(15)–C(14) = 1.138(4), S(5)–C(4) = 1.728(2), N(7)–C(6) = 1.303(3), O(12)–C(2) = 1.361(3), O(12)–C(13) = 1.429(3), C(1)–C(9)–C(8) = 118.4(2), O(10)–C(1)–C(9) = 125.3(2), N(7)–C(6)–C(14) = 121.8(2), C(6)–N(7)–C(8) = 109.2(2), C(14)–C(6)–S(5) = 120.5(2), C(4)–S(5)–C(6) = 87.95(12), N(15)–C(14)–C(6) = 178.7(3), C(3)–C(4)–S(5) = 128.2(2), N(7)–C(8)–C(4) = 115.1(2).

Analysis of the ^13^C-CRAPT NMR spectrum (Fig. 2, spectrum b) of 3e confirmed the presence of the expected 10 signals (2 aromatic CH's, 5 aromatic quaternary carbons, 1 cyano carbon, and 2 methoxy groups) which is consistent with all carbons being magnetically nonequivalent. The most striking feature of the ^13^C-CRAPT NMR of 3e, compared to the precursor (3,4-dimethoxyphenyl)carbamothioyl cyanide (1p′) (see ESI‡), is the presence of only 2 aromatic CH's (*δ* 104.9 & 102.9 ppm) as indicated by their negative phase and an additional quaternary carbon in the former (3e), suggesting that one proton has been removed from 1p′ and replaced with a quaternary center in product 3e. Evidence supporting the suggested regiochemistry of 3e cyclization at C_6_ (IUPAC numbering) rather than C_2_ is based on the presence of two singlets (*δ* 7.82 & 7.69 ppm) for the two aromatic CH's in the ^1^HNMR spectrum of 3e (Fig. 2, spectrum a). Further, the two CH's of 3e do not show any correlation in the ^1^H-^1^H-gDQCOSY NMR (see ESI‡), clearly indicating that they are isolated spin systems and are not coupled. These protons are attached to carbon atoms and could not be stemming from a NH group as indicated by the strong correlation contours with the carbons at *δ* 102.9 & 104.9 ppm in the ^1^H-^13^C-gHSQC NMR spectrum (Fig. 2, spectrum c). In fact, the ^1^HNMR spectrum of 3e is lacking the typical NH signal observed in products 2a–k′. Conclusive evidence supporting cyclization and the creation of the new ArC–S bond in 3e stems from the ^1^H-^13^C-gHMBC NMR spectrum (Fig. 2, spectrum d). The nitrile and C=N chemical shifts were easily identified at *δ* 113.9 (**C**N) and 133.0 (**C**=N) ppm, respectively, since they did not show any long-range ^1^H-^13^C heteronuclear multiple bond correlations with the CH protons. On the contrary, the two Ar**C**–OMe quaternary carbons were identified as the signals at *δ* 151.3 (C–O) and 150.7 (C–O) ppm due to strong ^1^H-^13^C long-range correlation cross peaks with the two methoxy groups at *δ* 3.88/3.87 ppm. The two remaining quaternary centers of the fused heterocycle, the C–S (*δ* 128.8) and C–N (*δ* 146.5), were instrumental proof of heterocyclization. Clearly, both protons at *δ* 7.82 & 7.69 ppm are totally correlated with the two adjacent carbon atoms of the fused ring (*δ* 128.8 & 146.5), as well as with the other two adjacent Ar**C**–OMe quaternary carbon atoms (*δ* 151.3 & 150.7) in the ^1^H–^13^C-gHMBC NMR spectrum (6 contour correlation squares in the aromatic region) (Fig. 2, spectrum d). Pleasingly, we were able to grow crystals suitable for X-ray diffraction analysis. Thus, structural verification of 3e was also carried out by single crystal X-ray crystallography (Fig. 3). Clearly, 3e comprises a 5-membered heterocyclic ring containing sulfur and C=N (characterized by short bond length of N(7)–C(6) = 1.303(3) Å and typical trigonal planar geometry where N(7)–C(8)–C(4) = 115.1(2)°), indicating that heterocyclization of the cyanothioformamide precursor 1p′ is faster than desulfurization. The nitrile group is also intact, displaying the typical linear bond angle = 178.7(3)° for N(15)–C(14)–C(6).

**Fig. 4:**
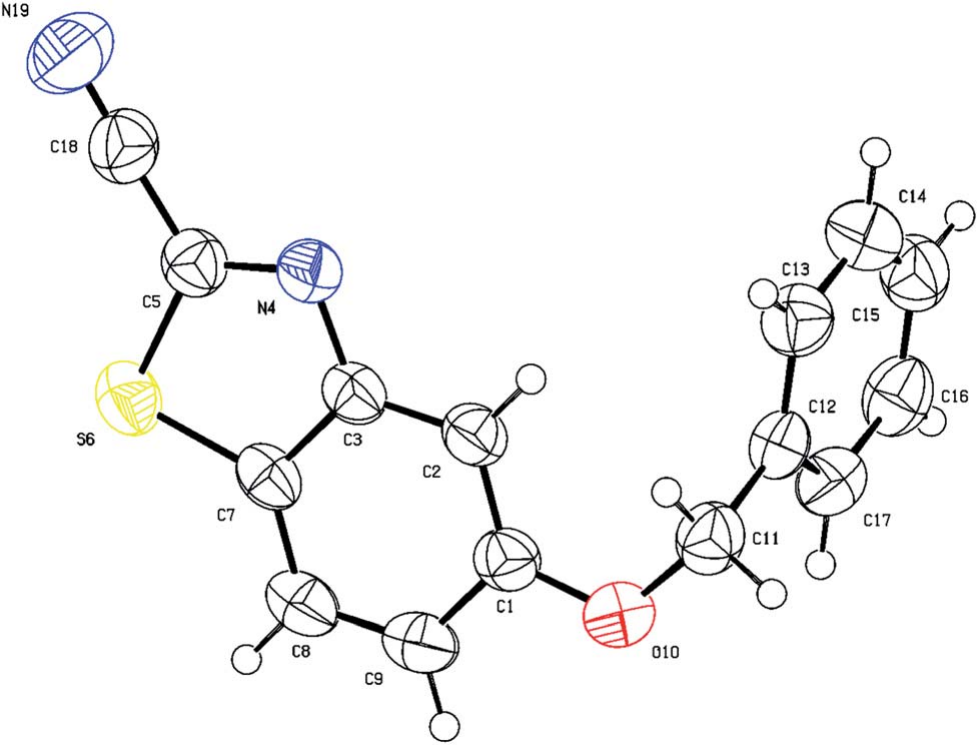
Thermal ellipsoid plots of compound 3c with ellipsoids drawn at 50% probability level. Selected bond distances (Å) and angles (deg) for compound 3c: S(6)–C(7) = 1.7299(17), C(7)–C(8) = 1.396(2), O(10)–C(1) = 1.362(2), N(4)–C(5) = 1.303(2), N(19)–C(18) = 1.136(2), C(5)–S(6)–C(7) = 87.96(7), C(2)–C(3)–C(7) = 121.11(14), C(1)–O(10)–C(11) = 118.23(13), N(19)–C(18)–C(5) = 177.1(2).

The structures of cyanobenzothiazoles 3c and 3f were also proven by single crystal X-ray crystallography, highlighting the role of the alkoxy group in cyclization. The single crystal X-ray structure of 5-(benzyloxy)benzo[*d*]oxazole-2-carbonitrile (3c) is shown in Fig. 4. The benzyloxy group clearly directs cyclization to the less hindered *para* position (C_7_ atom; Fig. 4 X-ray numbering).

**Fig. 5:**
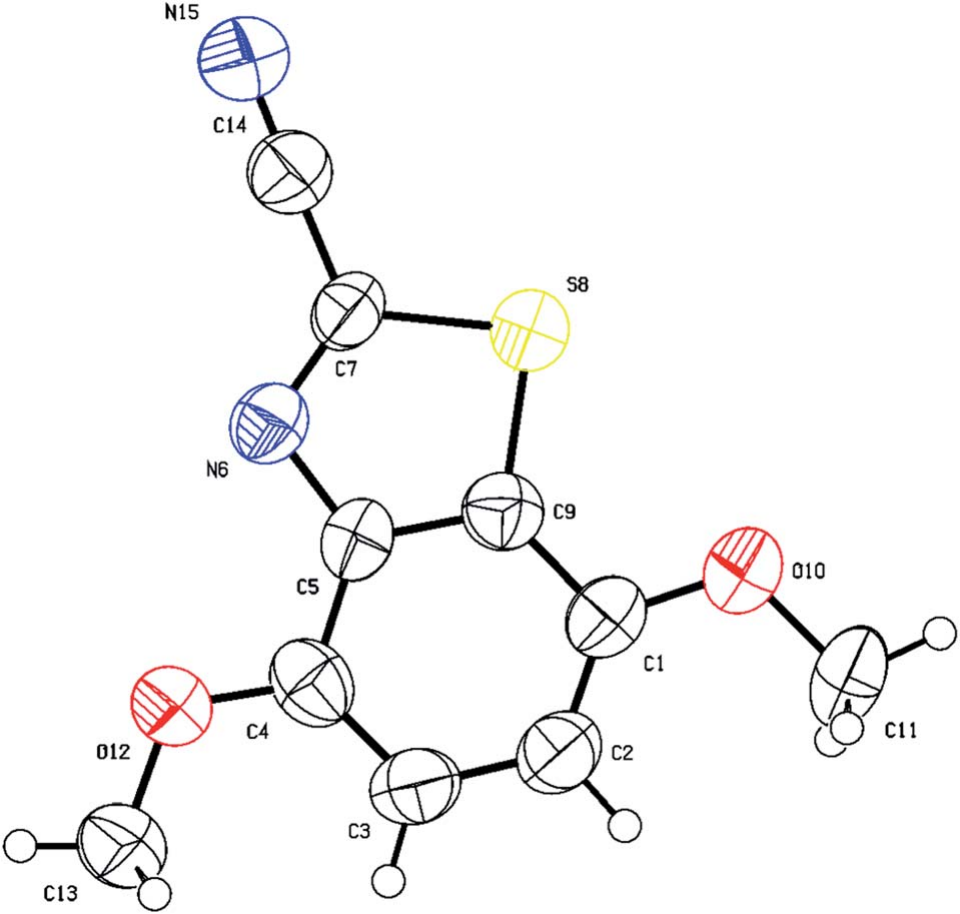
Thermal ellipsoid plots of compound 3f with ellipsoids drawn at 50% probability level. Selected bond distances (Å) and angles (deg) for compound 3f: N(15)–C(14) = 1.156(12), S(8)–C(7) = 1.729(7), C(7)–C(14) = 1.427(12), O(12)–C(4) = 1.345(10), O(12)–C(13) = 1.403(9), C(7)–S(8)–C(9) = 87.5(4), C(7)–N(6)–C(5) = 107.6(6), N(6)–C(7)–C(14) = 122.1(7), N(15)–C(14)–C(7) = 178.3(11), C(1)–C(9)–C(5) = 123.4(7), C(1)–O(10)–C(11) = 116.2(7).

The single crystal X-ray structure of 4,7-dimethoxybenzo[*d*]thiazole-2-carbonitrile (3f) is shown in Fig. 5. The C_1_ methoxy group directs cyclization to the more hindered C_9_ position since the *para* position (C_4_ atom in Fig. 5) is substituted.

**Scheme 4:**
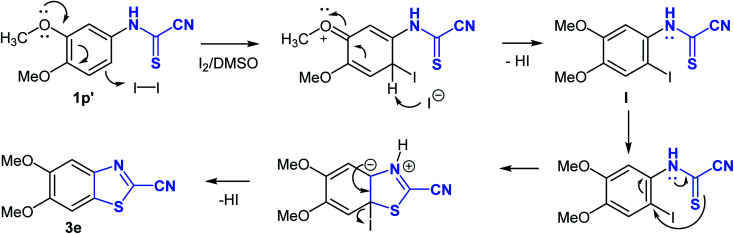
Proposed mechanism for the formation of 2-cyanobenzothiazoles from *N*-arylcyanothioformamide.

Interestingly, the cyclization reaction in all cases was completely regioselective, exclusively producing cyclized products 3b–e in which the CH *para* to the alkoxy or thiomethyl groups was the site of oxidative cyclization (Scheme 4). However, in case of product 3f, the more hindered *ortho*-CH was involved in the cyclization reaction due to the absence of a *para*-CH. Clearly, the alkoxy and thiomethyl groups direct the cyclization reaction and offer mechanistic implications. Though, it seems the presence of a substituent is not mandatory for annulation as suggested by the cyclization of naphthalen-1-ylcarbamothioyl cyanide (1l′) to the naphthyl derivative 3a. Using (3,4-dimethoxyphenyl)carbamothioyl cyanide (1p′) as a representative example, the proposed mechanism of heterocyclization is shown in Scheme 4. Mechanistically, it is conceivable that the cyanothioformanilide precursor 1 undergoes fast iodination and subsequent rearomatization to generate intermediate I. Indirect evidence for this mechanism is based on the isolation of (3-iodo-4,6-dimethoxyphenyl)carbamoyl cyanide (3g) (Table 3) from the reaction of its precursor (2,4-dimethoxyphenyl)carbamothioyl cyanide (1r′) (see ESI section‡) with I_2_-DMSO. Next, intramolecular nucleophilic attack by the thioformamide sulfur atom, followed by elimination of a HI molecule produces the cyanobenzothiazole 3e. To shed further light on the mechanism, free radical trapping control experiments were performed. Thus, the reaction of 1p′ with I_2_-DMSO was conducted in the presence of equimolar amounts of TEMPO (2,2,6,6-tetramethyl-1-piperidinyloxy) and BHT (2,6- ditertbutyl-4-methylphenol) as the radical inhibitors. We observed that product 3e was obtained in 63% and 62% yields, respectively, which suggested that a free radical pathway leading to free radical intermediates was not involved in the transformation process and formation of 3e.

**Fig. 6:**
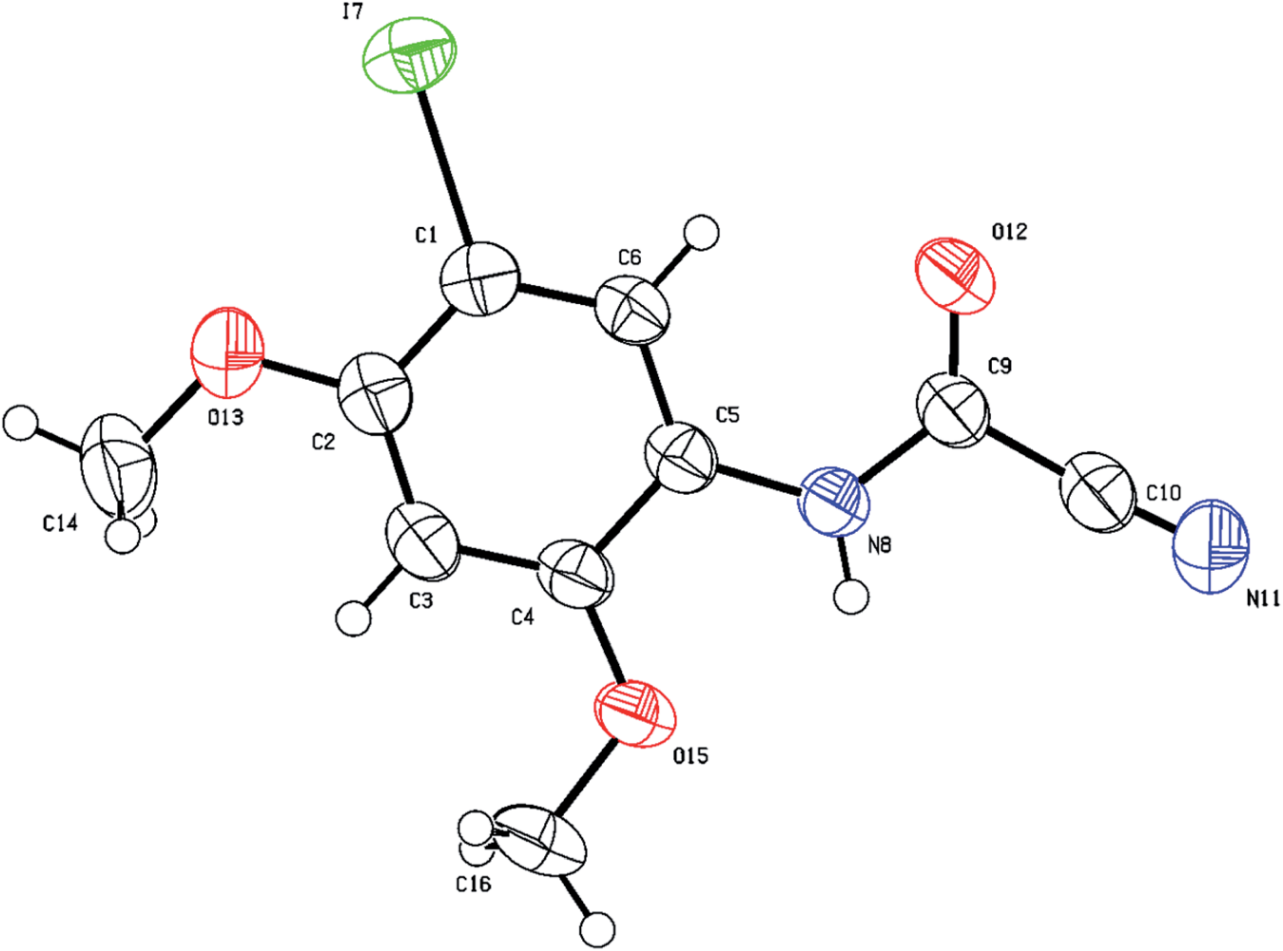
Thermal ellipsoid plots of compound 3g with ellipsoids drawn at 50% probability level. Selected bond distances (Å) and angles (deg) for compound 3g: I(7)–C(1) = 2.091(3), C(6)–C(1) = 1.384(5), O(13)–C(2) = 1.367(4), O(12)–C(9) = 1.203(4), N(11)–C(10) = 1.121(5), N(8)–C(5) = 1.418(4), N(8)–C(9) = 1.344(4), C(2)–O(13)–C(14) = 117.6(3), C(2)–C(1)–I(7) = 120.1(3), C(2)–C(3)–C(4) = 120.4(3), C(4)–O(15)–C(16) = 118.9(3), C(9)–N(8)–C(5) = 126.2(3), O(12)–C(9)–C(10) = 118.9(3), N(11)–C(10)–C(9) = 176.1(4).

The structure of (5-iodo-2,4-dimethoxyphenyl)carbamoyl cyanide (3g) could not be fully established based on 1D and 2D NMR, especially the position of iodine on the aromatic ring (position 2 (X-ray numbering C_6_) *vs.* 3 (X-ray numbering C_1_)). Thus, structural verification of 3g by single crystal X-ray crystallography was carried out as shown in Fig. 6. As expected, the iodination has been directed to the *o*/*p* position by the two methoxy groups, rendering 3g unsuitable species for cyclization. On the contrary, products 3b–f were all possible since iodination presumably occurs next to the cyanothioformamide group as directed by the alkoxy or thiomethyl groups. Finally, the preparation of cyanobenzothiazoles was amenable to scale-up to gram quantities as shown by the synthesis of 3a, 3b, 3d on a large scale from their precursors (10 mmol scale) 90%, 95%, and 87% isolated yields, respectively.

In conclusion, the I_2_-DMSO mediated desulfurization of 1 for the synthesis of cyanoformamides 2 at 38 °C has been successfully demonstrated. The reaction tolerated a range of functional groups including various halides, alkoxides, esters, cyano, nitro, thiomethyl, and trifluoromethyl functions and afforded a broad scope of products. It is expected that the current synthetic technique could become candidate for the synthesis of cyanoformamides because it is practical, scalable, uses a simple reagent system, and offers mild reaction conditions. The I_2_-DMSO oxidative system has also proven useful to access 2-cyanobenzothiazoles which may serve as useful precursors to access new luciferin analogs.

## Experimental section

### General information

Reactions were conducted with magnetic stirring in air-dried glassware. All reagents and reaction solvents were used as received without any further purification. Analytical thin-layer chromatography (TLC) was used to follow the progress of reactions and was carried out on precoated silica gel plates (HSGF 254) and visualized under UV irradiation (254 nm). Flash column chromatography was performed using silica gel (200−300 mesh) in cases where pure analytical samples were required. ^1^H and ^13^C NMR spectra were recorded in DMSO-d_6_ or CDCl_3_ on a Bruker DPX 300 and 75 MHz NMR spectrometer and on a Varian 400 and 100 MHz NMR spectrometer. The NMR chemical shifts (*δ*) are reported in parts per million (ppm) relative to the residual solvent peak (^1^H-NMR *δ* 7.26 for CDCl_3_, *δ* 2.50 for DMSO-d_6_; ^13^C-NMR *δ* 77.0 for CDCl_3_, *δ* 39.52 for DMSO-d_6_). The following abbreviations were used to explain NMR peak multiplicities: br s = broad signal, s = singlet, d = doublet, t = triplet, q = quartet, p = pentet, sept = septet, app = apparent, and m = multiplet. IR spectra were recorded using a Bruker FT-IR spectrometer and a Thermo Nicolet Nexus 470 FT-IR. High-resolution mass analyses (HRMS) were obtained using a Waters Q-TOF Premier mass spectrometer [electrospray ionization (ESI)]. Melting points were measured using a capillary melting point apparatus (MEL-TEMP) in degrees Celsius (°C).

### Procedures

The *N*-Arylcyanothioformamide 1 (0.5 mmol) was heated at 38 °C for 19 h in 2 mL of DMSO with 444 mg (1.75 mmol, 3.5 equiv.) of iodine. The reaction mixture was treated with 4 mL of sodium thiosulfate (1 M) and was extraction with ether (10 mL). The colorless or faint yellow ether extract was washed with a brine solution (2 × 10 mL), dried (Na_2_SO_4_), and concentrated *in vacuo* to afford the corresponding *N*-arylcyanoformamide 2 or cyanbenzoxazole 3 product in the specified chemical yield.

#### *p*-Tolylcarbamoyl cyanide (2a)^29^

Colorless solid (98% yield); mp 178–180 °C; IR (KBr) 3277 (NH), 2233 (CN), 1715 (C=O), 1678, 1611, 1555, 1509, 1407, 1323, 1261, 1181, 1122, 941, 827, 707, 512 cm^−1^; ^1^H NMR (DMSO-d_6_, 400 MHz) *δ* 11.75 (broad s, 1H, NH), 7.45 (d, *J* = 8.0 Hz, 2H, Ar–H), 7.20 (d, *J* = 8.0 Hz, 2H, Ar–H), 2.27 (s, 3H, CH_3_). ^13^C NMR (DMSO-d_6_, 100 MHz) *δ* 140.5 (C=O), 135.1 (C–N), 134.2 (**C**_**q**_–Me), 129.6 (2×CH), 120.2 (2×CH), 112.5 (CN), 20.6 (CH_3_); HRMS (ESI^+^): *m*/*z* [M + H]^+^ calcd for C_9_H_9_N_2_O: 161.0715; found: 161.0728.

#### Phenylcarbamoyl cyanide (2b)^29^

Colorless solid (94% yield); mp 123–124 °C; IR (KBr) 3277 (NH), 2234 (CN), 1681 (C=O), 1613, 1560, 1492, 1447, 1396, 1327, 1260, 937, 761, 707, 688, 509 cm^−1^; ^1^H NMR (DMSO-d_6_, 400 MHz) *δ* 11.82 (broad s, 1H, NH), 7.56 (d, *J* = 8.0 Hz, 2H, Ar–H), 7.40 (t, *J* = 8.0 Hz, 2H, Ar–H), 7.22 (t, *J* = 8.0 Hz, 1H, Ar–H); ^13^C NMR (DMSO-d_6_, 100 MHz) *δ* 140.7 (C=O), 136.6 (C–N), 129.3 (2×CH), 125.8 (CH), 120.3 (2×CH), 112.5 (CN); HRMS (ESI^+^): *m*/*z* [M + H]^+^ calcd for C_8_H_7_N_2_O: 147.0558; found: 147.0551.

#### (4-Chlorophenyl)carbamoyl cyanide (2c)^29^

Colorless solid (85% yield); mp 240–241 °C; IR (KBr) 3259 (NH), 2231 (CN), 1697 (C=O), 1612, 1553, 1489, 1401, 1318, 1254, 1093, 1012, 927, 831, 755, 713, 509, 492 cm^−1^; ^1^H NMR (DMSO-d_6_, 400 MHz) *δ* 11.96 (broad s, 1H, NH), 7.58 (d, *J* = 8.8 Hz, 2H, Ar–H), 7.47 (d, *J* = 8.8 Hz, 2H, Ar–H); ^13^C NMR (DMSO-d_6_, 100 MHz) *δ* 140.8 (C=O), 135.6 (C–N), 129.6 (C–Cl), 129.2 (2×CH), 122.0 (2×CH), 112.4 (CN). HRMS (ESI^+^): *m*/*z* [M + H]^+^ calcd for C_8_H_6_ClN_2_O: 181.0169; found: 181.0185.

#### (2-Fluorophenyl)carbamoyl cyanide (2d)

[CAS 199584-45-7]: light yellow solid (70% yield). mp 115–117 °C; IR (KBr) 3281 (NH), 2235 (CN), 1705 (C=O) cm^−1^; ^1^H NMR (DMSO-d_6_, 400 MHz) *δ* 11.84 (broad s, 1H, NH), 7.71 (t, 1H, *J* = 8.0 Hz, Ar–H), 7.38–7.29 (m, 2H, Ar–H), 7.28–7.19 (m, 1H, Ar–H). ^13^C NMR (DMSO-d_6_, 100 MHz) *δ* 154.3 (d, *J* = 247.0 Hz, C–F), 141.6 (C=O), 128.4 (d, *J* = 8.0 Hz, CH), 125.5 (d, *J* = 1.0 Hz, CH), 124.9 (d, *J* = 4.0 Hz, CH), 123.1 (d, *J* = 12.0 Hz, C–N), 116.2 (d, *J* = 19.0 Hz, CH), 112.3 (CN). HRMS (ESI^+^): *m*/*z* [M + H]^+^ calcd for C_8_H_6_FN_2_O: 165.0464; found: 165.0469.

#### (4-Fluorophenyl)carbamoyl cyanide (2e)^29^

Colorless solid (98% yield); mp 118–119 °C; IR (KBr) 3471 (NH), 2232 (CN), 1684 (C=O), 1507, 1411, 1228, 840, 779, 721, 564, 496 cm^−1^; ^1^H NMR (DMSO-d_6_, 400 MHz) *δ* 11.91 (broad s, 1H, NH), 7.58 (dd, *J* = 8.0, 4.0 Hz, 2H, Ar–H), 7.24 (t, *J* = 8.0 Hz, 2H, Ar–H); ^13^C NMR (DMSO-d_6_, 100 MHz) *δ* 159.4 (d, *J* = 242.0 Hz, C–F), 140.8 (C=O), 133.1 (d, *J* = 3.0 Hz, C–N), 122.5 (d, *J* = 8.0 Hz, 2×CH), 116.1 (d, *J* = 23.0 Hz, 2×CH), 112.5 (CN); HRMS (ESI^+^): *m*/*z* [M + H]^+^ calcd for C_8_H_6_FN_2_O: 165.0464; found: 165.0470.

#### (3-Fluorophenyl)carbamoyl cyanide (2f)

[CAS 199584-47-9] Light orange solid (91% yield); mp 101–103 °C; IR (KBr) 3292 (NH), 2232 (CN), 1701 (C=O) cm^−1^; ^1^H NMR (DMSO-d_6_, 400 MHz) *δ* 12.01 (broad s, 1H, NH), 7.48–7.40 (m, 2H, Ar–H), 7.32 (dddd, *J* = 8.4, 2.8, 2.0, 0.8 Hz, 1H, Ar–H), 7.05 (tdd, *J* = 8.8, 2.8, 0.8 Hz, 1H, Ar–H); ^13^C NMR (DMSO-d_6_, 100 MHz) *δ* 162.0 (d, *J* = 242.0 Hz, C–F), 140.9 (C=O), 138.2 (d, *J* = 11.0 Hz, C–N), 131.1 (d, *J* = 10.0 Hz, CH), 116.2 (d, *J* = 3.0 Hz, CH), 112.5 (d, *J* = 21.0 Hz, CH), 112.3 (CN), 107.4 (d, *J* = 27.0 Hz, CH); HRMS (ESI^+^): *m*/*z* [M + H]^+^ calcd for C_8_H_6_FN_2_O: 165.0464; found: 165.0473.

#### (4-Nitrophenyl)carbamoyl cyanide (2g)^57^

Light yellow solid (89% yield); mp 265–266 °C (decomp); IR (KBr) 3284 (NH), 2238 (CN), 1686 (C=O), 1621, 1573, 1513, 1409, 1340, 1256, 1202, 1111, 930, 857, 829, 752, 711, 685 cm^−1^; ^1^H NMR (DMSO-d_6_, 400 MHz) *δ* 12.35 (broad s, 1H, NH), 8.27 (d, *J* = 8.8 Hz, 2H, Ar–H), 7.78 (d, *J* = 8.8 Hz, 2H, Ar–H); ^13^C NMR (DMSO-d_6_, 100 MHz) *δ* 144.1 (C–NO_2_), 142.6 (C–N), 141.4 (C=O), 125.2 (2×CH), 120.6 (2×CH), 112.2 (CN); HRMS (ESI^+^): *m*/*z* [M + H]^+^ calcd for C_8_H_6_N_3_O_3_: 192.0409; found: 192.0502.

#### (3-Nitrophenyl)carbamoyl cyanide (2h)

[CAS 200422-14-6]: light yellow solid (89% yield); mp 143–145 °C; IR (KBr) 3308 (NH), 2237 (CN), 1702 (C=O), 1702 (C=O), 1596, 1480, 1434, 1350, 1283, 1237, 1089, 1063, 945, 921, 896, 817, 739, 670, 415 cm^−1^; ^1^H NMR (DMSO-d_6_, 400 MHz) *δ* 12.27 (broad s, 1H, NH), 8.47 (t, *J* = 2.5 Hz, 1H, Ar–H), 8.05 (dd, *J* = 8.0, 2.0 Hz, 1H, Ar–H), 7.90–7.85 (m, 1H, Ar–H), 7.61 (t, *J* = 8.0 Hz, 1H, Ar–H). ^13^C NMR (DMSO-d_6_, 100 MHz) *δ* 147.9 (C–NO_2_), 141.3 (C=O), 137.7 (C–N), 130.8 (CH), 126.3 (CH), 120.3 (CH), 114.7 (CH), 112.2 (CN); HRMS (ESI^+^): *m*/*z* [M + H]^+^ calcd for C_8_H_6_N_3_O_3_: 192.0409; found: 192.0498.

#### (4-Methoxyphenyl)carbamoyl cyanide (2i)^29^

Colorless solid (89% yield); mp 145–146 °C; IR (KBr) 3263 (NH), 2231 (CN), 1670 (C=O), 1616, 1558, 1509, 1417, 1305, 1262, 1172, 1038, 809, 766, 710, 568, 522; ^1^H NMR (DMSO-d_6_, 400 MHz) *δ* 11.70 (broad s, 1H, NH), 7.47 (d, *J* = 8.0 Hz, 2H, Ar–H), 6.93 (d, *J* = 8.0 Hz, 2H, Ar–H), 3.71 (s, 3H, OCH_3_); ^13^C NMR (DMSO-d_6_, 100 MHz) *δ* 157.0 (C–O), 140.3 (C=O), 129.8 (C–N), 121.9 (2×CH), 114.4 (2×CH), 112.7 (CN), 55.4 (OCH_3_); HRMS (ESI^+^): *m*/*z* [M + H]^+^ calcd for C_9_H_9_N_2_O_2_: 177.0664; found: 177.0660.

#### (4-Ethoxyphenyl)carbamoyl cyanide (2j)

[CAS 1904417-73-7]: light yellow solid (98% yield); mp 145–147 °C; IR (KBr) 3274 (NH), 2233 (CN), 1682 (C=O), 1618, 1558, 1509, 1475, 1391, 1252, 1173, 1117, 1049, 923, 836, 735, 705, 607, 523 cm^−1^; ^1^H NMR (DMSO-d_6_, 400 MHz) *δ* 11.72 (broad s, 1H, NH), 7.47 (d, *J* = 8.0 Hz, 2H, Ar–H), 6.91 (d, *J* = 8.0 Hz, 2H, Ar–H), 3.97 (q, *J* = 8.0 Hz, 2H, OCH_2_), 1.29 (t, *J* = 8.0 Hz, 2H, CH_3_); ^13^C NMR (DMSO-d_6_, 100 MHz) *δ* 156.4 (C–O), 140.4 (C=O), 129.7 (C–N), 122.0 (2×CH), 114.9 (2×CH), 112.8 (CN), 63.5 (OCH_2_), 14.8 (CH_3_). HRMS (ESI^+^): *m*/*z* [M + H]^+^ calcd for C_10_H_11_N_2_O_2_: 191.0821; found: 191.0833.

#### (4-(Benzyloxy)phenyl)carbamoyl cyanide (2k)

Light yellow orange (63% yield); mp 132–134 °C. IR (KBr) 2228 (CN), 1605 (C=N), 1561, 1509, 1237, 1175, 998, 833, 748, 698 cm^−1^; ^1^H NMR (DMSO-d_6_, 400 MHz) *δ* 11.75 (broad s, 1H, NH), 7.50 (d, *J* = 8.8 Hz, 2H, Ar–H), 7.45–7.29 (m, 5H, Ar–H), 7.03 (d, *J* = 8.8 Hz, 2H, Ar–H), 5.07 (s, 2H, OCH_2_); ^13^C NMR (DMSO-d_6_, 100 MHz) *δ* 156.1 (C–O), 140.4 (C=O), 137.0 (CH_2_**C**_q_), 130.0 (C–N), 128.6 (2×CH), 128.0 (CH), 127.9 (2×CH), 122.0 (2×CH), 115.0 (2×CH), 112.7 (CN), 69.5 (OCH_2_). HRMS (ESI^+^): *m*/*z* [M + H]^+^ calcd for C_15_H_13_N_2_O_2_: 253.0977; found: 253.0965.

#### (4-(Methylthio)phenyl)carbamoyl cyanide (2l)

[CAS 1893995-33-9]: light brown solid (64% yield); mp 123–125 °C; IR (KBr) 3309 (NH), 2230 (CN), 1690 (C=O), 1604, 1537, 1493, 1435, 1397, 1312, 1285, 1252, 1126, 1095, 927, 806, 667, 507 cm^−1^; ^1^H NMR (DMSO-d_6_, 400 MHz) *δ* 11.81 (broad s, 1H, NH), 7.50 (d, *J* = 8.0 Hz, 2H, Ar–H), 7.26 (d, *J* = 8.0 Hz, 2H, Ar–H), 2.44 (s, 3H, SCH_3_); ^13^C NMR (DMSO-d_6_, 100 MHz) *δ* 140.5 (C=O), 135.5 (**C_q_**–SMe), 133.8 (C–N), 126.7 (2×CH), 121.0 (2×CH), 112.6 (CN), 15.0 (SCH_3_); HRMS (ESI^+^): *m*/*z* [M + H]^+^ calcd for C_9_H_9_N_2_OS: 193.0436; found: 193.0444.

#### Methyl 3-((cyanocarbonyl)amino)benzoate (2m)

[CAS 1893995-33-9]: colorless solid (75% yield); mp 157–159 °C; IR (KBr) 3289 (NH), 2232 (CN), 1689 (C=O), 1605, 1545, 1432, 1412, 1320, 1250, 1201, 1179, 1120, 1016, 959, 927, 857, 772, 693, 515, 493 cm^−1^; ^1^H NMR (DMSO-d_6_, 400 MHz) *δ* 12.10 (broad s, 1H, NH), 7.95 (d, *J* = 8.0 Hz, 2H, Ar–H), 7.67 (d, *J* = 8.0 Hz, 2H, Ar–H), 3.81 (s, 3H, OCH_3_); ^13^C NMR (DMSO-d_6_, 100 MHz) *δ* 165.6 (C=O), 141.1 (C_q_), 140.9 (C=O), 130.6 (2×CH), 126.4 (C–N), 120.0 (2×CH), 112.3 (CN), 52.2 (OCH_3_); HRMS (ESI^+^): *m*/*z* [M + H]^+^ calcd for C_10_H_9_N_2_O_3_: 205.0613; found: 205.0601.

#### Ethyl 3-((cyanocarbonyl)amino)benzoate (2n)^29^

Colorless solid (80% yield); mp 209–210 °C. IR (KBr) 3263 (NH), 2231 (CN), 1724 (C=O), 1685 (C=O), 1605, 1552, 1475, 1413, 1295, 1178, 1114, 1021, 925, 857, 771, 713, 694 cm^−1^; ^1^H NMR (DMSO-d_6_, 400 MHz) *δ* 12.12 (broad s, 1H, NH), 7.97 (d, *J* = 8.0 Hz, 2H, Ar–H), 7.69 (d, *J* = 8.0 Hz, 2H, Ar–H), 4.28 (q, *J* = 8.0 Hz, 2H, OCH_2_), 1.30 (t, *J* = 8.0 Hz, 3H, CH_3_); ^13^C NMR (DMSO-d_6_, 100 MHz) *δ* 165.0 (C=O), 141.1 (C_q_), 140.9 (C=O), 130.5 (2×CH), 126.6 (C–N), 120.0 (2×CH), 112.3 (CN), 60.8 (OCH_2_), 14.2 (CH_3_); HRMS (ESI^+^): *m*/*z* [M + H]^+^ calcd for C_11_H_11_N_2_O_3_: 219.0770; found: 219.0763.

#### (4-(Trifluoromethyl)phenyl)carbamoyl cyanide (2o)^29^

Colorless solid (76% yield); mp 126–127 °C. IR (KBr) 3289 (NH), 2238 (CN), 1709 (C=O), 1616, 1556, 1411, 1323, 1256, 1147, 1066, 1013, 926, 846, 703, 596, 513, 459 cm^−1^; ^1^H NMR (DMSO-d_6_, 400 MHz) *δ* 12.16 (broad s, 1H, NH), 7.77 (coallesced AB quartet, 4H, Ar–H); ^13^C NMR (DMSO-d_6_, 100 MHz) *δ* 141.3 (C=O), 140.3 (q, *J* = 1.0 Hz, C–N), 126.6 (q, *J* = 4.0 Hz, 2×CH), 125.8 (q, *J* = 32.0 Hz, **C**–CF_3_), 124.1 (q, *J* = 270.0 Hz, **C**F_3_), 120.6 (2×CH), 112.3 (CN); HRMS (ESI^+^): *m*/*z* [M + H]^+^ calcd for C_9_H_6_F_3_N_2_O: 215.0432; found: 215.0439.

#### (4-Ethylphenyl)carbamoyl cyanide (2p)

[CAS 1903633-06-6]: light yellow solid (92% yield); mp 145–147 °C; lit. mp 145–146 °C. IR (KBr) 3274 (NH), 2232 (CN), 1713 (C=O), 1615, 1556, 1509, 1417, 1324, 1264, 1059, 943, 823, 752, 709, 532 cm^−1^; ^1^H NMR (DMSO-d_6_, 400 MHz) *δ* 11.76 (broad s, 1H, NH), 7.45 (d, *J* = 8.0 Hz, 2H, Ar–H), 7.19 (d, *J* = 8.0 Hz, 2H, Ar–H), 2.54 (q, *J* = 8.0 Hz, 2H, CH_2_), 1.12 (t, *J* = 8.0 Hz, 3H, CH_3_); ^13^C NMR (DMSO-d_6_, 100 MHz) *δ* 141.6 (**C**_q_–CH_2_), 140.6 (C=O), 134.5 (C–N), 128.5 (2×CH), 120.4 (2×CH), 112.6 (CN), 27.8 (CH_2_), 15.7 (CH_3_); HRMS (ESI^+^): *m*/*z* [M + H]^+^ calcd for C_10_H_11_N_2_O: 175.0871; found: 175.0867.

#### (4-Iodophenyl)carbamoyl cyanide (2q)

Light yellow solid (74% yield); mp 247–250 °C; IR (KBr) 3286 (NH), 2231 (CN), 1698 (C=O), 1670, 1602, 1542, 1483, 1396, 1313, 1289, 1249, 1062, 1006, 928, 829, 694, 505 cm^−1^; ^1^H NMR (DMSO-d_6_, 400 MHz) *δ* 11.93 (broad s, 1H, NH), 7.74 (d, *J* = 8.8 Hz, 2H, Ar–H), 7.37 (d, *J* = 8.8 Hz, 2H, Ar–H); ^13^C NMR (DMSO-d_6_, 100 MHz) *δ* 140.8 (C=O), 138.0 (2×CH), 136.5 (C–N), 122.4 (2×CH), 112.5 (CN), 90.4 (C–I); HRMS (ESI^+^): *m*/*z* [M + H]^+^ calcd for C_8_H_6_IN_2_O: 272.9525; found: 272.9514.

#### (3-Chlorophenyl)carbamoyl cyanide (2r)^29^

Colorless solid (94% yield); mp 116–118 °C; IR (KBr) 3290 (NH), 2139 (CN), 1702 (C=O), 1597, 1549, 1476, 1429, 1279, 1250, 1197, 1080, 997, 927, 867, 781, 675 cm^−1^; ^1^H NMR (DMSO-d_6_, 400 MHz) *δ* 12.06 (broad s, 1H, NH), 7.69–7.67 (m, 1H, Ar–H), 7.49–7.39 (m, 2H, Ar–H), 7.30–7.26 (m, 1H, Ar–H); ^13^C NMR (DMSO-d_6_, 100 MHz) *δ* 141.0 (C=O), 138.1 (C–N), 133.5 (C–Cl), 131.1 (CH), 125.7 (CH), 120.0 (CH), 118.9 (CH), 112.4 (CN); HRMS (ESI^+^): *m*/*z* [M + H]^+^ calcd for C_8_H_6_ClN_2_O: 181.0169; found: 181.0187.

#### (3-Bromophenyl)carbamoyl cyanide (2s)^58^

Orange solid (95% yield); mp 122–124 °C; IR (KBr) 3286 (NH), 2250 (CN), 1698 (C=O), 1602, 1542, 1472, 1430, 1279, 1248, 1192, 1072, 995, 925, 867, 780, 675 cm^−1^; ^1^H NMR (DMSO-d_6_, 400 MHz) *δ* 12.01 (broad s, 1H, NH), 7.83–7.80 (m, 1H, Ar–H), 7.52–7.48 (m, 1H, Ar–H), 7.43–7.33 (m, 2H, Ar–H). ^13^C NMR (DMSO-d_6_, 100 MHz) *δ* 141.0 (C=O), 138.2 (C–N), 131.3 (CH), 128.6 (CH), 122.8 (CH), 121.8 (C–Br), 119.3 (CH), 112.4 (CN); HRMS (ESI^+^): *m*/*z* [M + H]^+^ calcd for C_8_H_6_BrN_2_O: 224.9663; found: 224.9659.

#### (3-Iodophenyl)carbamoyl cyanide (2t)

Light yellow solid (75% yield); mp 134–136 °C; IR (KBr) 3254 (NH), 2230 (CN), 1695 (C=O), 1606, 1582, 1551, 1473, 1401, 1315, 1243, 1172, 1066, 994, 862, 784, 752, 715, 678, 434 cm^−1^; ^1^H NMR (DMSO-d_6_, 400 MHz) *δ* 11.91 (broad s, 1H, NH), 7.98 (t, *J* = 2.5 Hz, 1H, Ar–H), 7.59–7.55 (m, 1H, Ar–H), 7.51 (dddd, *J* = 8.4, 2.8, 2.0, 0.8 Hz, 1H, Ar–H), 7.19 (t, *J* = 8.0 Hz, 1H, Ar–H); ^13^C NMR (DMSO-d_6_, 100 MHz) *δ* 140.9 (C=O), 138.0 (C–N), 134.4 (CH), 131.3 (CH), 128.6 (CH), 119.7 (CH), 112.4 (CN), 94.9 (C–I); HRMS (ESI^+^): *m*/*z* [M + H]^+^ calcd for C_8_H_6_IN_2_O: 272.9525; found: 272.9519.

#### (3-Cyanophenyl)carbamoyl cyanide (2u)

Light yellow solid (73% yield); mp 232–234 °C. IR (KBr) 3250 (NH), 2246 (CN), 1706 (C=O), 1615, 1591, 1565, 1476, 1436, 1327, 1297, 1260, 1233, 950, 922, 891, 805, 720, 680, 478 cm^−1^; ^1^H NMR (DMSO-d_6_, 400 MHz) *δ* 12.17 (broad s, 1H, NH), 7.95 (t, *J* = 1.6 Hz, 1H, Ar–H), 7.82–7.78 (m, 1H, Ar–H), 7.68 (dt, *J* = 7.6, 1.2 Hz, 1H, Ar–H), 7.61 (t, *J* = 8.0 Hz, 1H, Ar–H); ^13^C NMR (DMSO-d_6_, 100 MHz) *δ* 141.3 (C=O), 137.5 (C–N), 130.8 (CH), 129.4 (CH), 125.1 (CH), 123.3 (CH), 118.3 (**C**CN), 112.3 (CN), 112.1 (CN); HRMS (ESI^+^): *m*/*z* [M + H]^+^ calcd for C_9_H_6_N_3_O: 172.0511; found: 172.0518.

#### (3-(Trifluoromethyl)phenyl)carbamoyl cyanide (2v)^59^

Light yellow solid (73% yield); mp 103–106. lit. mp > 100 °C (decomp).;^37^ IR (KBr) 3300 (NH), 2245 (CN), 1702 (C=O), 1602, 1566, 1451, 1337, 1291, 1255, 1193, 1137, 1069, 930, 886, 802, 698, 663 cm^−1^; ^1^H NMR (DMSO-d_6_, 400 MHz) *δ* 12.14 (broad s, 1H, NH), 7.96 (s, 1H), 7.77 (d, *J* = 8.0 Hz, 1H), 7.65 (t, *J* = 8.0 Hz, 1H), 7.57 (d, *J* = 8.0 Hz, 1H), 7.65 (t, *J* = 8.0 Hz, 1H); ^13^C NMR (DMSO-d_6_, 100 MHz) *δ* 141.2 (C=O), 137.5 (C–N), 130.6 (CH), 129.8 (q, *J* = 32.0 Hz, **C**–CF_3_), 124.0 (CH), 123.4 (q, *J* = 271.0 Hz, **C**F_3_), 122.2 (q, *J* = 3.0 Hz, CH), 116.6 (q, *J* = 5.0 Hz, CH), 112.3 (CN); HRMS (ESI^+^): *m*/*z* [M + H]^+^ calcd for C_9_H_6_F_3_N_2_O: 215.0432; found: 215.0444.

#### (4-Methyl-1,3-phenylene)dicarbamoyl cyanide (2w)^60^

Light orange solid (98% yield); mp 172–175 °C; IR (KBr) 3241 (NH), 2241 (CN), 1689 (C=O), 1560, 1538, 1496, 1255, 1038, 945, 889, 713 cm^−1^; ^1^H NMR (DMSO-d_6_, 400 MHz) *δ* 11.91 (broad s, 1H, NH), 11.39 (broad s, 1H, NH), 7.68 (d, *J* = 2.4 Hz, 1H, Ar–H), 7.40 (dd, *J* = 8.4, 2.4 Hz, 1H, Ar–H), 7.29 (d, *J* = 8.4 Hz, 1H, Ar–H), 2.20 (s, 3H, CH_3_); ^13^C NMR (DMSO-d_6_, 100 MHz) *δ* 141.7 (C=O), 140.7 (C=O), 134.9 (C–N), 133.7 (**C**–Me), 131.4 (CH), 129.9 (C–N), 119.0 (CH), 116.9 (CH), 112.6 (CN), 112.4 (CN), 17.4 (CH_3_); HRMS (ESI^+^): *m*/*z* [M + H]^+^ calcd for C_11_H_9_N_4_O_2_: 229.0726; found: 229.0728.

#### 1,4-Phenylenedicarbamoyl cyanide (2x)^29^

Light yellow solid (78% yield); mp > 290 °C; IR (KBr) 3268 (NH), 2233 (CN), 1689 (C=O), 1582, 1509, 1411, 1317, 1244, 1206, 1130, 920, 852, 742, 707, 536, 464 cm^−1^; ^1^H NMR (DMSO-d_6_, 400 MHz) *δ* 11.93 (broad s, 2H, NH), 7.59 (s, 4H, Ar–H); ^13^C NMR (DMSO-d_6_, 100 MHz) *δ* 140.6 (C=O), 134.1 (C–N), 121.0 (4xCH), 112.5 (CN); HRMS (ESI^+^): *m*/*z* [M + H]^+^ calcd for C_10_H_7_N_4_O_2_: 215.0569; found: 215.0565.

#### (2-Bromophenyl)carbamoyl cyanide (2y)

[1904417-81-7]: light yellow solid (86% yield); mp 77–79 °C; IR (KBr) 3263 (NH), 2236 (CN), 1695 (C=O), 1592, 1533, 1440, 1300, 1243, 1203, 1029, 930, 761, 659 cm^−1^; ^1^H NMR (DMSO-d_6_, 400 MHz) *δ* 11.75 (broad s, 1H, NH), 7.73 (dd, *J* = 8.0, 1.2 Hz, 1H, Ar–H), 7.50 (td, *J* = 8.0, 1.6 Hz, 1H, Ar–H), 7.45 (td, *J* = 7.6, 1.6 Hz, 1H, Ar–H), 7.29 (td, *J* = 7.6, 1.6 Hz, 1H, Ar–H); ^13^C NMR (DMSO-d_6_, 100 MHz) *δ* 142.0 (C=O), 133.3 (C–N), 133.2 (CH), 129.7 (CH), 128.7 (CH), 128.5 (CH), 119.3 (C–Br), 112.5 (CN); HRMS (ESI^+^): *m*/*z* [M + H]^+^ calcd for C_8_H_6_BrN_2_O: 224.9663; found: 224.9655.

#### (2,4-Dichlorophenyl)carbamoyl cyanide (2z)

[CAS 1903829-54-8]: light yellow solid (41% yield); mp 119–122 °C; IR (KBr) 3247 (NH), 2238 (CN), 1681 (C=O), 1586, 1532, 1472, 1382, 1297, 1207, 1055, 933, 821, 662, 559 cm^−1^; ^1^H NMR (DMSO-d_6_, 400 MHz) major tautomer: *δ* 11.83 (broad s, 1H, NH), 7.77 (d, *J* = 2.4 Hz, 1H, Ar–H), 7.62 (d, *J* = 8.8 Hz, 1H, Ar–H), 7.50 (dd, *J* = 8.8, 2.4 Hz, 1H, Ar–H). minor tautomer: *δ* 7.26 (d, *J* = 2.4 Hz, 0.24H, Ar–H), 7.05 (dd, *J* = 8.8, 2.4 Hz, 0.25H, Ar–H), 6.77 (d, *J* = 8.8 Hz, 0.26H, Ar–H), 6.36 (broad s, 0.25H, NH); ^13^C NMR (DMSO-d_6_, 100 MHz) major tautomer: *δ* 142.0 (C=O), 132.5 (C–Cl), 131.1 (C–N), 129.6 (CH), 129.4 (C–Cl), 128.8 (CH), 128.2 (CH), 112.3 (CN). Minor tautomer: *δ* 144.0 (C=O), 128.2 (CH), 127.8 (CH), 124.3 (C–Cl), 119.0 (C–N), 117.5 (C–Cl), 116.4 (CH), 113.9 (CN); HRMS (ESI^+^): *m*/*z* [M + H]^+^ calcd for C_8_H_5_Cl_2_N_2_O: 214.9779; found: 214.9770.

#### (5-Chloro-2-methylphenyl)carbamoyl cyanide (2a′)

[CAS 1904233-70-0]: light yellow solid (74% yield); mp 132–134 °C; IR (KBr) 3338 (NH), 2238 (CN), 1698 (C=O), 1584, 1535, 1478, 1445, 1411, 1299, 1253, 1203, 1182, 1130, 1087, 1009, 927, 903, 881, 817, 646, 448 cm^−1^; ^1^H NMR (DMSO-d_6_, 400 MHz) *δ* 11.41 (broad s, 1H, NH), 7.48 (d, *J* = 2.0 Hz, 1H, Ar–H), 7.29 (d, *J* = 8.0 Hz, 1H, Ar–H), 7.25 (dd, *J* = 8.0, 2.0 Hz, 1H, Ar–H), 2.19 (s, CH_3_). ^13^C NMR (DMSO-d_6_, 100 MHz) *δ* 142.0 (C=O), 134.7 (C–Cl), 132.4 (CH), 131.5 (**C**–CH_3_), 130.3 (C–N), 127.1 (CH), 125.1 (CH), 112.6 (CN), 17.3 (CH_3_); HRMS (ESI^+^): *m*/*z* [M + H]^+^ calcd for C_9_H_8_ClN_2_O: 195.0325; found: 195.0319.

#### (2,4-Dimethylphenyl)carbamoyl cyanide (2b′)^29^

Colorless solid (97% yield); mp 84–86 °C; IR (KBr) 3212 (NH), 2237 (CN), 1690 (C=O), 1544, 1450, 1300, 1280, 1234, 1035, 958, 943, 806, 698 563, 450 cm^−1^; ^1^H NMR (DMSO-d_6_, 400 MHz) *δ* 11.24 (broad s, 1H, NH), 7.23 (d, *J* = 8.0 Hz, 1H, Ar–H), 7.08 (s, 1H), 7.02 (d, *J* = 8.0 Hz, 1H, Ar–H), 2.25 (s, 3H, CH_3_), 2.17 (s, 3H, CH_3_); ^13^C NMR (DMSO-d_6_, 100 MHz) *δ* 141.7 (C=O), 136.8 (**C**–CH_3_), 132.4 (**C**–CH_3_), 131.4 (CH), 130.7 (C–N), 127.0 (CH), 125.4 (CH), 112.8 (CN), 20.6 (CH_3_), 17.7 (CH_3_); HRMS (ESI^+^): *m*/*z* [M + H]^+^ calcd for C_10_H_11_N_2_O: 175.0871; found: 175.0874.

#### Mesitylcarbamoyl cyanide (2c′)

[CAS 1903301-18-7]: light yellow solid (34% yield); mp 123–126 °C; IR (KBr) 3317 (NH), 2233 (CN), 1694 (C=O), 1611, 1579, 1472, 1443, 1376, 1204, 1066, 857, 792, 618, 475 cm^−1^; ^1^H NMR (CDCl_3_, 400 MHz) *δ* 6.91 (s, 2H, Ar–H), 2.29 (s, 3H, CH_3_), 2.08 (s, 6H, 2×CH_3_). ^13^C NMR (CDCl_3_, 100 MHz) *δ* 142.3 (C=O), 136.4 (**C**–CH_3_), 129.4 (2×CH), 125.4 (2×C–CH_3_), 109.3 (CN), 20.8 (CH_3_), 17.5 (2×CH_3_); HRMS (ESI^+^): *m*/*z* [M + H]^+^ calcd for C_11_H_13_N_2_O: 189.1028; found: 189.1037.

#### (2,3-Dichlorophenyl)carbamoyl cyanide (2d′)

[CAS 199736-11-3]: light brown solid (73% yield); mp 119–120 °C; IR (KBr) 3307 (NH), 2240 (CN), 1712 (C=O), 1590, 1531, 1454, 1413, 1217, 1191, 1051, 943, 782, 742, 700, 666 cm^−1^; ^1^H NMR (DMSO-d_6_, 400 MHz) *δ* 11.95 (broad s, 1H, NH), 7.62 (dd, *J* = 8.0, 1.2 Hz, 1H, Ar–H), 7.57 (dd, *J* = 8.0, 1.6 Hz, 1H, Ar–H), 7.43 (t, *J* = 8.0 Hz, 1H, Ar–H); ^13^C NMR (DMSO-d_6_, 100 MHz) *δ* 142.0 (C=O), 133.9 (C–Cl), 132.5 (C–N), 129.5 (CH), 128.6 (CH), 127.2 (C–Cl), 126.4 (CH), 112.3 (CN); HRMS (ESI^+^): *m*/*z* [M + H]^+^ calcd for C_8_H_5_Cl_2_N_2_O: 214.9779; found: 214.9774.

#### (2-Chloro-5-(trifluoromethyl)phenyl)carbamoyl cyanide (2e′)

[CAS 1903764-61-3]: Orange solid (60% yield); mp 83–85 °C; IR (KBr) 3212 (NH), 2279 (CN), 1702 (C=O), 1597, 1541, 1427, 1330, 1271, 1181, 1125, 1085, 935, 894, 827 cm^−1^; ^1^H NMR (DMSO-d_6_, 400 MHz) *δ* 12.03 (broad s, 1H, NH), 8.07 (d, *J* = 2.0 Hz, 1H), 7.86 (d, *J* = 8.4 Hz, 1H), 7.74 (dd, *J* = 8.4, 2.0 Hz, 1H); ^13^C NMR (DMSO-d_6_, 100 MHz) *δ* 142.3 (C=O), 133.0 (C–Cl), 132.4 (C–N), 131.3 (CH), 128.4 (q, *J* = 23.0 Hz, **C**–CF_3_), 125.4 (q, *J* = 3.0 Hz, CH), 124.3 (q, *J* = 4.0 Hz, CH), 123.4 (q, *J* = 271.0 Hz, **C**F_3_), 112.1 (CN); HRMS (ESI^+^): *m*/*z* [M + H]^+^ calcd for C_9_H_5_ClF_3_N_2_O: 249.0043; found: 249.0055.

#### (2,6-Dichlorophenyl)carbamoyl cyanide (2f′)

Light yellow solid (34% yield); mp 131–133 °C; IR (KBr) 3269 (NH), 2231 (CN), 1689 (C=O), ^1^H NMR (DMSO-d_6_, 400 MHz) *δ* 12.21 (broad s, 1H, NH), 7.64 (d, *J* = 8.4 Hz, 1H, Ar–H), 7.64 (d, *J* = 8.0 Hz, 1H, Ar–H), 7.47 (dd, *J* = 8.4, 8.0 Hz, 1H, Ar–H); ^13^C NMR (DMSO-d_6_, 100 MHz) *δ* 141.3 (C=O), 132.8 (2×C–Cl), 131.0 (CH), 129.3 (C–N), 129.0 (2×CH), 111.9 (CN); HRMS (ESI^+^): *m*/*z* [M + H]^+^ calcd for C_8_H_5_Cl_2_N_2_O: 214.9779; found: 214.9785.

#### (4-Bromophenyl)carbamoyl cyanide (2g′)^29^

Colorless solid (90% yield); mp 259–261 °C; lit mp^29^ 263–264 °C; IR (KBr) 3262 (NH), 2232 (CN), 1693 (C=O), 1609, 1549, 1486, 1398, 1320, 1253, 1074, 1010, 928, 831, 813, 751, 711, 506 cm^−1^; ^1^H NMR (DMSO-d_6_, 400 MHz) *δ* 11.96 (broad s, 1H, NH), 7.58 (d, *J* = 8.8, Hz, 1H, Ar–H), 7.51 (d, *J* = 8.8, Hz, 1H, Ar–H); ^13^C NMR (DMSO-d_6_, 100 MHz) *δ* 142.8 (C=O), 136.1 (C–N), 133.2 (2×CH), 122.3 (2×CH), 117.8 (C–Br), 112.5 (CN); HRMS (ESI^+^): *m*/*z* [M + H]^+^ calcd for C_8_H_6_BrN_2_O: 224.9663; found: 224.9667.

#### (2-Methoxy-5-methylphenyl)carbamoyl cyanide (2h′)

[CAS 1904138-13-1]: light yellow solid (74% yield); mp 80–82 °C; IR (KBr) 3302 (NH), 2226 (CN), 1696 (C=O), 1616, 1597, 1546, 1493, 1456, 1381, 1324, 1262, 1224, 1181, 1129, 1031, 927, 885, 807, 680, 455 cm^−1^. ^1^H NMR (DMSO-d_6_, 400 MHz) *δ* 11.25 (broad s, 1H, NH), 7.42 (d, *J* = 2.0 Hz, 1H, Ar–H), 7.04 (dd, *J* = 8.4, 2.0 Hz, 1H, Ar–H), 6.98 (d, *J* = 8.4 Hz, 1H, Ar–H), 3.78 (s, 3H, OCH_3_), 2.21 (s, 3H, CH_3_); ^13^C NMR (DMSO-d_6_, 100 MHz) *δ* 149.3 (C–O), 141.5 (C=O), 129.4 (**C**–CH_3_), 128.0 (CH), 124.8 (CH), 123.7 (C–N), 112.6 (CN), 111.9 (CH), 55.9 (OCH_3_), 20.3 (CH_3_). HRMS (ESI^+^): *m*/*z* [M + H]^+^ calcd for C_10_H_11_N_2_O_2_: 191.0821; found: 191.0815.

#### (3,5-Dichlorophenyl)carbamoyl cyanide (2i′)

[CAS 502173-47-9]: light yellow solid (80% yield); mp 138–140 °C; IR (KBr) 3260 (NH), 2237 (CN), 1702 (C=O), 1672, 1613, 1589, 1552, 1443, 1416, 1277, 1212, 1118, 1096, 929, 885, 852, 806, 749, 717, 668, 653 cm^−1^; ^1^H NMR (DMSO-d_6_, 400 MHz) *δ* 12.13 (broad s, 1H, NH), 7.55 (d, *J* = 1.6 Hz, 2H, Ar–H), 7.45 (t, *J* = 2.0 Hz, 1H, Ar–H); ^13^C NMR (DMSO-d_6_, 100 MHz) *δ* 141.2 (C=O), 138.9 (C–N), 134.5 (2×C–Cl), 125.1 (CH), 118.6 (CH), 112.1 (CN); HRMS (ESI^+^): *m*/*z* [M + H]^+^ calcd for C_8_H_5_Cl_2_N_2_O: 214.9779; found: 214.9771.

#### (3,4-Dichlorophenyl)carbamoyl cyanide (2j′)^59^

Light yellow solid (77% yield); mp 159–161 °C; IR (KBr) 3270 (NH), 2249 (CN), 1701 (C=O), 1606, 1590, 1541, 1472, 1383, 1301, 1242, 1201, 1149, 1129, 1026, 868, 819, 705 cm^−1^; ^1^H NMR (DMSO-d_6_, 400 MHz) *δ* 12.08 (broad s, 1H, NH), 7.81 (d, *J* = 2.4 Hz, 1H, Ar–H), 7.63 (d, *J* = 8.8 Hz, 1H, Ar–H), 7.46 (dd, *J* = 8.8, 2.8 Hz, 1H, Ar–H); ^13^C NMR (DMSO-d_6_, 100 MHz) *δ* 141.0 (C=O), 136.7 (C–Cl), 131.4 (C–N), 131.2 (CH), 127.6 (C–Cl), 121.7 (CH), 120.4 (CH), 112.2 (CN); HRMS (ESI^+^): *m*/*z* [M + H]^+^ calcd for C_8_H_5_Cl_2_N_2_O: 214.9779; found: 214.9773.

#### (2,4-Difluorophenyl)carbamoyl cyanide (2k′)

[CAS 1892850-82-6]: light peach solid (50% yield); mp 89–91 °C; IR (KBr) 3291 (NH), 2242 (CN), 1716 (C=O), 1613, 1558, 1502, 1438, 1292, 1263, 1229, 1147, 1098, 968, 849, 813, 728, 677, 603, 575, 536 cm^−1^; ^1^H NMR (DMSO-d_6_, 400 MHz) *δ* 11.82 (broad s, 1H, NH), 7.69 (td, *J* = 8.8, 6.0 Hz, 1H, Ar–H), 7.42 (ddd, *J* = 8.8, 6.0, 4.8, 2.4 Hz, 1H, Ar–H), 7.18–7.10 (m, 1H, Ar–H); ^13^C NMR (DMSO-d_6_, 100 MHz) *δ* 160.3 (dd, *J* = 245.0, 12.0 Hz, C–F), 154.7 (dd, *J* = 250.0, 12.0 Hz, C–F), 141.7 (C=O), 127.0 (dd, *J* = 10.0, 2.0 Hz, CH), 119.6 (dd, *J* = 13.0, 4.0 Hz, C–N), 111.9 (dd, *J* = 22.0, 3.0 Hz, CH), 112.2 (CN), 104.9 (dd, *J* = 24.0, 24.0 Hz, CH); HRMS (ESI^+^): *m*/*z* [M + H]^+^ calcd for C_8_H_5_F_2_N_2_O: 183.0370; found: 183.0359.

#### Naphtho[1,2-*d*]thiazole-2-carbonitrile (3a)^61^

Brown solid (93% yield); mp 146–148 °C; IR (KBr) 2222 (CN), 1622 (C=N), 1501, 1450, 1423, 1393, 1212, 1159, 1126, 810, 771, 752, 686, 549 cm^−1^; ^1^H NMR (DMSO-d_6_, 400 MHz) *δ* 8.65 (d, *J* = 8.4 Hz, 1H), 8.25 (d, *J* = 8.8 Hz, 1H), 8.17–8.09 (m, 2H), 7.78 (t, *J* = 7.2 Hz, 1H), 7.72 (t, *J* = 6.8 Hz, 1H); ^13^C NMR (DMSO-d_6_, 100 MHz) *δ* 148.5 (C–N), 135.4 (C=N), 134.19, 132.0, 129.8 (CH), 128.6 (CH), 128.4 (CH), 127.8 (C–S), 127.7 (CH), 123.2 (CH), 119.6 (CH), 113.9 (CN). HRMS (ESI^+^): *m*/*z* [M + H]^+^ calcd for C_12_H_7_N_2_S: 211.0330; found: 211.0321.

#### 5-Methoxybenzo[*d*]thiazole-2-carbonitrile (3b)^62^

Light yellow solid (98% yield); lit. mp 96–98 °C; IR (KBr) 2229 (CN), 1604 (C=N), 1473, 1414, 1339, 1277, 1205, 1168, 1065, 1020, 956, 833, 815 cm^−1^; ^1^H NMR (DMSO-d_6_, 400 MHz) *δ* 8.16 (d, *J* = 8.8 Hz, 1H, Ar–H), 7.71 (d, *J* = 2.4 Hz, 1H, Ar–H), 7.34 (dd, *J* = 8.8, 2.4 Hz, 1H, Ar–H), 3.87 (s, 3H, OCH_3_). ^13^C NMR (DMSO-d_6_, 100 MHz) *δ* 159.9 (C–O), 153.3 (C–N), 137.5 (C=N), 127.6 (C–S), 123.5 (CH), 119.8 (CH), 113.6 (CN), 105.8 (CH), 55.9 (OCH_3_); HRMS (ESI^+^): *m*/*z* [M + H]^+^ calcd for C_9_H_7_N_2_OS: 191.0279; found: 191.0271.

#### 5-(Benzyloxy)benzo[*d*]oxazole-2-carbonitrile (3c)

Light orange solid (64% yield); mp 88–92 °C; IR (KBr) 2227 (CN), 1604 (C=N), 1604, 1547, 1496, 1463, 1443, 1416, 1339, 1271, 1205, 1171, 1130, 1012, 949, 831, 813, 776, 741, 698, 457, 414 cm^−1^; ^1^H NMR (DMSO-d_6_, 400 MHz) *δ* 7.83 (d, *J* = 8.8 Hz, 1H, Ar–H), 7.69 (d, *J* = 2.0 Hz, 1H, Ar–H), 7.50–7.33 (m, 6H, Ar–H), 5.17 (s, 2H, OCH_2_); ^13^C NMR (DMSO-d_6_, 100 MHz) *δ* 159.1 (C–O), 153.7 (C–N), 137.1 (C=N), 135.9 (CH_2_C_q_), 128.7 (2×CH), 128.3 (CH), 127.6 (C–S), 127.5 (2×CH), 122.0 (CH), 120.5 (CH), 113.1 (CN), 107.3 (CH), 70.5 (OCH_2_); HRMS (ESI^+^): *m*/*z* [M + H]^+^ calcd for C_15_H_11_N_2_OS: 267.0592; found: 267.0581.

#### 5-(Methylthio)benzo[*d*]thiazole-2-carbonitrile (3d)

Light yellow solid (91% yield); mp 105–107 °C; IR (KBr) 2230 (CN), 1614 (C=N), 1585, 1434, 1402, 1311, 1228, 1151, 1133, 1044, 922, 841, 808, 777, 715 cm^−1^; ^1^H NMR (DMSO-d_6_, 400 MHz) *δ* 8.17 (d, *J* = 8.8 Hz, 1H, Ar–H), 7.99 (d, *J* = 2.4 Hz, 1H, Ar–H), 7.56 (dd, *J* = 8.8, 2.4 Hz, 1H, Ar–H), 2.56 (s, 3H, SCH_3_); ^13^C NMR (DMSO-d_6_, 100 MHz) *δ* 152.6 (C–N), 139.7 (**C**–SMe), 137.7 (C=N), 131.9 (C–S), 127.4 (CH), 123.2 (CH), 119.7 (CH), 113.5 (CN), 14.8 (SCH_3_); HRMS (ESI^+^): *m*/*z* [M + H]^+^ calcd for C_9_H_7_N_2_S_2_: 207.0051; found: 207.0045.

#### 5,6-Dimethoxybenzo[*d*]thiazole-2-carbonitrile (3e)^63^

Colorless solid (65% yield); mp 156–157 °C; IR (KBr) 2224 (CN), 1604 (C=N), 1547, 1497, 1442, 1420, 1354, 1283, 1207, 1170, 1059, 993, 848, 771 cm^−1^; ^1^H NMR (DMSO-d_6_, 400 MHz) *δ* 7.82 (s, 1H, Ar–H), 7.69 (s, 1H, Ar–H), 3.88 (s, 3H, OCH_3_), 3.87 (s, 3H, OCH_3_); ^13^C NMR (DMSO-d_6_, 100 MHz) *δ* 151.3 (C–O), 150.7 (C–O), 146.5 (C–N), 133.0 (C=N), 128.8 (C–S), 113.9 (CN), 104.9 (CH), 102.9 (CH), 56.1 (OCH_3_), 56.0 (OCH_3_). HRMS (ESI^+^): *m*/*z* [M + H]^+^ calcd for C_10_H_9_N_2_O_2_S: 221.0385; found: 221.0389.

#### 4,7-Dimethoxybenzo[*d*]thiazole-2-carbonitrile (3f)^64^

Light orange solid (80% yield); mp 147–148 °C; lit. mp 174 °C; ^1^H NMR (DMSO-d_6_, 400 MHz) *δ* 6.93 (d, *J* = 8.8 Hz, 1H, Ar–H), 6.91 (d, *J* = 8.8 Hz, 1H, Ar–H), 4.03 (s, 3H, OCH_3_), 3.96 (s, 3H, OCH_3_); ^13^C NMR (DMSO-d_6_, 100 MHz) *δ* 148.9 (C–O), 147.6 (C–O), 143.7 (C–N), 135.6 (C=N), 126.3 (C–O), 112.9 (CN), 108.4 (CH), 108.0 (CH), 56.4 (OCH_3_), 56.2 (OCH_3_); HRMS (ESI^+^): *m*/*z* [M + H]^+^ calcd for C_10_H_9_N_2_O_2_S: 221.0385; found: 221.0391.

#### (5-Iodo-2,4-dimethoxyphenyl)carbamoyl cyanide (3g)

Light brown solid (60% yield); mp 168–171 °C; IR (KBr) 3273 (NH), 2233 (CN), 1697 (C=O), 1593, 1528, 1495, 1463, 1434, 1386, 1329, 1289, 1207, 1163, 1026, 933, 887, 811, 678 cm^−1^; ^1^H NMR (DMSO-d_6_, 400 MHz) *δ* 11.25 (broad s, 1H, NH), 7.91 (s, 1H, Ar–H), 6.74 (s, 1H, Ar–H), 3.86 (s, 3H, OCH_3_), 3.85 (s, 3H, OCH_3_); ^13^C NMR (DMSO-d_6_, 100 MHz) *δ* 157.4 (C–O), 153.3 (C–O), 141.6 (C=O), 133.8 (CH), 118.2 (C–N), 112.5 (CN), 97.1 (CH), 73.1 (C–I), 56.9 (OCH_3_), 56.3 (OCH_3_); HRMS (ESI^+^): *m*/*z* [M + H]^+^ calcd for C_10_H_10_IN_2_O_3_: 332.9736; found: 332.9723.

## Funding information

Dr Ziad Moussa is grateful to the United Arab Emirates University (UAEU) of Al-Ain and to the Research Office for supporting the research developed in his laboratory (Grant no. G00003291/Fund no. 31S401/12S040/Project #852). The authors would like to acknowledge the Deanship of Scientific Research at Umm Al-Qura University for supporting this work by Grant code: 22UQU4320545DSR04.

## Author contributions

Ziad Moussa (Writing–original draft, Conceptualization, Investigation, Data curation), Zaher M. A. Judeh (Conceptualization, Writing – review & editing), Ahmed Alzamly (Data curation, Software, Validation), Saleh A. Ahmed (Conceptualization, Review & editing), Harbi T. Al-Masri (Data curation, Formal Analysis), Bassam Al-Hindawi (Data curation), Faisal Rasool (Investigation), Sara Saada (Data curation).

## Conflicts of interest

There are no conflicts to declare.

## Supplementary Material

RA-012-D2RA00049K-s001

RA-012-D2RA00049K-s002
