# Supplementary material for: Iodine-DMSO mediated conversion of N-arylcyanothioformamides to N-arylcyanoformamides and the unexpected formation of 2-cyanobenzothiazoles
Source: RSC Adv. 2022 Feb 21;12(10):6133–48. doi: 10.1039/d2ra00049k (PMC8981512; doi:10.1039/d2ra00049k)

Electronic Supplementary Material (ESI) for RSC Advances.
This journal is © The Royal Society of Chemistry 2022

Supporting Information

Iodine-DMSO Mediated Conversion of *N*-Arylcyanoformamides to *N*-Arylcyanothioformamides and the Unexpected Formation of 2-Cyanobenzothiazoles

Ziad Moussa*[a], Zaher M.A. Judeh[b], Ahmed Alzamly[a], Saleh A. Ahmed[c,d], Harbi T. Al-Masri[e] , Bassam Al-Hindawi[a], Faisal Rasool[a], Sara Saada[a]

[a]Department of Chemistry, College of Science, United Arab Emirates University, P. O. Box 15551, Al Ain, United Arab Emirates
zmoussa@uaeu.ac.ae
[b]School of Chemical and Biomedical Engineering, Nanyang Technological University, 62 Nanyang Drive, N1.2–B1-14, Singapore, 637459, Singapore
[c]Department of Chemistry, Faculty of Applied Sciences, Umm Al-Qura University, Makkah 21955, Saudi Arabia
[d]Department of Chemistry, Faculty of Science, Assiut University, 71516 Assiut, Egypt
[e]Department of Chemistry, Faculty of Sciences, Al al-Bayt University, Mafraq, Hashemite Kingdom of Jordan
Correspondence may be addressed to:
Ziad Moussa: E-mail address: zmoussa@uaeu.ac.ae
Tel.: + 971 509695321

Table of Contents	S1
General Information	S2
General Procedure and Characterization of Arylcarbamothioyl cyanides **1a-1t'**	S2
References	S17
1D and 2D NMR spectra of **1a-1t'**, **2a-2k'**, **3a-g**	S18
^{1}H NMR (DMSO-d$_6$) spectrum of ethyl phenylcarbamate	S504
^{13}C NMR (DMSO-d$_6$) spectrum of ethyl phenylcarbamate	S505
^{13}C DEPT-90 NMR (DMSO-d$_6$) spectrum of ethyl phenylcarbamate	S506
Single crystal X-ray diffraction data for compound **2g'**, **3c**, **3f**, **3e**, **3g**	S507
1D/2D spectra of (3-methoxy-[1,1'-biphenyl]-4-yl)carbamoyl cyanide	S526
1D/2D spectra of (2-iodophenyl)carbamothioyl cyanide	S531

General Information. Reactions were conducted with magnetic stirring in air-dried glassware. All reagents and reaction solvents were used as received without any further purification. Analytical thin-layer chromatography (TLC) was used to follow the progress of reactions and was carried out on precoated silica gel plates (HSGF 254) and visualized under UV irradiation (254 nm). Flash column chromatography was performed using silica gel (200−300 mesh) in cases where pure analytical samples were required. ^{1}H and ^{13}C NMR spectra were recorded in DMSO-d_6 or $CDCl_3$ on a Bruker DPX 300 and 75 MHz NMR spectrometer and on a Varian 400 and 100 MHz NMR spectrometer. The NMR chemical shifts (δ) are reported in parts per million (ppm) relative to the residual solvent peak (^{1}H-NMR δ 7.26 for $CDCl_3$, δ 2.50 for DMSO-d_6; ^{13}C-NMR δ 77.0 for $CDCl_3$, δ 39.52 for DMSO-d_6). The following abbreviations were used to explain NMR peak multiplicities: br s = broad signal, s = singlet, d = doublet, t = triplet, q = quartet, p = pentet, sept = septet, app = apparent, and m = multiplet. IR spectra were recorded using a Bruker FT-IR spectrometer and a Thermo Nicolet Nexus 470 FT-IR. High-resolution mass analyses (HRMS) were obtained using a Waters Q-TOF Premier mass spectrometer [electrospray ionization (ESI)]. Melting points were measured using a capillary melting point apparatus (MEL-TEMP) in degrees Celsius (°C).

General procedure for the preparation of arylcarbamothioyl cyanides 1a'-1t':

The starting materials arylcarbamothioyl cyanides **1a'-1t'** were prepared according to the following procedure on a 20 mmol scale.

Into a gently stirred room-temperature solution of potassium cyanide (1.30 g, 20 mmol) in water (15 mL) was added slowly an ethanolic solution of the isothiocyanate (20 mmol dissolved in 100 mL ethanol) over a period of 5 min. After the reaction mixture had been stirred for an additional 120 min, it was quenched dropwise over 15 min with dilute 5% hydrochloric acid solution (70 mL) using a dropping funnel *(Caution:* to be performed in a well-ventilated fume hood). Ice water (100-200 mL) was added, and the resulting precipitate was filtered using a 500 mL sintered glass funnel, followed by washing with ice water (3x50 mL) and petroleum ether (1x25mL). The product was then oven-dried at 60 °C for 6h or air-dried in the fume hood for 2d to yield arylcarbamothioyl cyanides **1a'-1t'** pure enough for all further uses. In the rare cases where no precipitate was observed following the addition of ice water (100-200 mL), ethyl acetate was used to extract the product from the aqueous solution (2x100mL). Subsequent drying of the organic layer with sodium sulfate and evaporation of the solvent under reduced pressure afforded the desired arylcarbamothioyl cyanides pure enough for further uses.

The starting materials arylcarbamothioyl cyanides 1a,c,i,q,g',j' [ref.1a], 1j [ref.1b], 1r' [ref.1c], 1e,f,o,p' [ref.1d], 1i' [ref.1e], 1b [ref.1f], 1q' [ref.1g], 1d [ref.1h], 1g [ref.1i], 1h [ref.1j], 1n,y [ref. 1k], 1r [ref.1l], 1v,z,a', 1l', [ref.m], 1w [ref.n], 1x [ref.o], 1b' [ref.p], 1d' [ref.q], and 1m' [ref.] have been previously reported.[1] Arylcarbamothioyl cyanides 1k,l,m,p,s,t,u,e',h',k',n', o',s',t' are novel compounds.

p-Tolylcarbamothioyl cyanide (1:0.6 tautomeric ratio) (1a): Bright yellow solid; 88% Yield; Mp 123-124, Lit[2] Mp 126.5-128.5; IR (KBr) 3272 (NH), 3071, 2227 (CN), 1611, 1545, 1507, 1403 (C=C), 1298, 1181, 1122, 1106, 1085, 809, 732 (C-H bend), 634, 607, 578, 520, 504 cm^{-1}; ^{1}H NMR ($CDCl_3$, 400 MHz) δ 9.97 (s, 1H, NH, major tautomer), 9.72 (s, 0.53H, NH, minor tautomer),

7.68 (d, J = 8.0 Hz, 2H, Ar-H, major tautomer), 7.26 (broad s, 2.32H, Ar-H, minor tautomer), 7.23 (d, J = 8.0 Hz, 2H, Ar-H, major tautomer), 2.38 (s, 1.84H, CH_3, minor tautomer), 2.36 (s, 3H, CH_3, major tautomer) ppm; ^{13}C NMR ($CDCl_3$, 100 MHz) δ 165.4 (C=S, minor tautomer), 161.0 (C=S, major tautomer), 139.2 (C_q-CH_3, minor tautomer), 138.5 (C_q-CH_3, major tautomer), 134.5 (C_q-N, major tautomer), 134.4 (C_q-N, minor tautomer), 130.4 (2xCH, minor tautomer), 129.8 (2xCH, major tautomer), 122.7 (2xCH, minor tautomer), 122.2 (2xCH, major tautomer), 113.5 (CN, major tautomer), 112.0 (CN, minor tautomer), 21.3 (CH_3, major tautomer), 21.2 (CH_3, minor tautomer) ppm; HRMS (ESI$^+$): m/z $[M + H]^+$ calcd for $C_9H_9N_2S$: 177.0486; found: 177.0492.

Phenylcarbamothioyl cyanide (1:0.59 tautomeric ratio) (1b): Dark orange solid; 87% Yield; Mp 79-81, Lit[3] Mp 80; IR (KBr) 3273 (NH), 3089, 2225 (CN), 1615, 1553, 1488, 1406 (C=C), 1205, 1093, 906, 759 (C-H bend), 737, 682, 634, 607, 522, 502 cm^{-1}; ^{1}H NMR ($CDCl_3$, 300 MHz) δ 9.95 (s, 1.7H, NH, both tautomers), 7.80 (d, J = 8.0 Hz, 2H, Ar-H, both tautomers), 7.51-7.31 (m, 6.48H, Ar-H, both tautomers) ppm; ^{13}C NMR ($CDCl_3$, 75 MHz) δ 165.6 (C=S, minor tautomer), 161.6 (C=S, major tautomer), 137.1 (C_q-N, major tautomer), 136.9 (C_q-N, minor tautomer), 129.9 (2xCH, minor tautomer), 129.3 (2xCH, major tautomer), 128.8 (CH, minor tautomer), 128.2 (CH, major tautomer) 122.7 (2xCH, minor tautomer), 122.3 (2xCH, major tautomer), 113.5 (CN, major tautomer), 112.0 (CN, minor tautomer) ppm; HRMS (ESI$^+$): m/z $[M + H]^+$ calcd for $C_8H_7N_2S$: 163.0330; found: 163.0328.

4-Chlorophenyl)carbamothioyl cyanide (1:0.47 tautomeric ratio) (1c): Orange solid; 92% Yield; Mp 114-116, Lit[3] Mp 120-122; IR (KBr) 3274 (NH), 3117, 2231 (CN), 1603, 1543, 1488 (C=C), 1388, 1092 , 1010, 824 (C-H bend), 747 (C-Cl) cm^{-1}; ^{1}H NMR ($CDCl_3$, 300 MHz) δ 9.87 (s, 1.35H, NH), 7.80 (d, J = 9.0 Hz, 1.87H, Ar-H), 7.53-7.33 (m, 3.64H, Ar-H) ppm; ^{13}C NMR ($CDCl_3$, 75 MHz) δ 165.6 (C=S, minor tautomer), 161.6 (C=S, major tautomer), 135.4 (C_q-N, major tautomer), 135.3 (C_q-N, minor tautomer), 134.7 (C-Cl, minor tautomer), 133.4 (C-Cl, major tautomer), 130.2 (2xCH, minor tautomer), 129.5 (2xCH, major tautomer), 124.1 (2xCH, minor tautomer), 123.6 (2xCH, major tautomer), 113.4 (CN, major tautomer), 111.8 (CN, minor tautomer) ppm; HRMS (ESI$^+$): m/z $[M + H]^+$ calcd for $C_8H_6ClN_2S$: 196.9940; found: 196.9958.

(2-Fluorophenyl)carbamothioyl cyanide (1:0.53 tautomeric ratio) (1d): orange solid; 79% Yield; Mp 86-87, Lit[4] Mp 86-87.5; IR (KBr) 3255 (NH), 2233 (CN), 1618, 1597, 1551, 1484, 1460, 1390, 1309, 1282, 1248, 1159, 1103, 751, 616, 545, 511, 453 cm^{-1}; ^{1}H NMR ($CDCl_3$, 400 MHz) δ 9.64 (br s, 1H, NH, major tautomer), 9.45 (br s, 0.53H, NH, minor tautomer), 8.44 (t, J = 8.0 Hz, 1H, Ar-H, major tautomer), 8.65 (t, J = 8.0 Hz, 0.53H, Ar-H, minor tautomer), 7.43-7.21 (m, 4.46H, Ar-H, major and minor tautomer) ppm; ^{13}C NMR ($CDCl_3$, 100 MHz) δ 166.1 (C=S, minor tautomer), 162.6 (C=S, major tautomer), 154.4 (d, J = 249.0 Hz, C-F, minor tautomer), 153.9 (d, J = 249.0 Hz, C-F, major tautomer), 130.2 (d, J = 8.0 Hz, CH, minor tautomer), 129.2 (d, J = 8.0 Hz, CH, major tautomer), 125.2 (d, J = 4.0 Hz, CH, minor tautomer), 125.0 (d, J = 11.0 Hz, C_q-N, both tautomers), 124.5 (CH, minor tautomer), 124.4 (d, J = 4.0 Hz, CH, major tautomer), 123.9 (CH, major tautomer), 116.7 (d, J = 19.0 Hz, CH, minor tautomer), 116.0 (d, J = 19.0 Hz, CH, major tautomer), 113.3 (CN, major tautomer), 111.9 (CN, minor tautomer) ppm; HRMS (ESI$^+$): m/z $[M + H]^+$ calcd for $C_8H_6FN_2S$: 181.0236; found: 181.0227.

(4-Fluorophenyl)carbamothioyl cyanide (1:0.2 tautomeric ratio) (1e): Dark brown solid: 81% Yield; Mp 104-106; IR (KBr) 3259 (NH), 3083, 2240 (CN), 1505, 1396 (C=C), 1231 (C-F), 1159, 1109, 1088, 834 (C-H bend), 735 cm^{-1}; ^{1}H NMR (DMSO-d6, 300 MHz) δ 13.50 (br s, 0.70H, NH), 7.91 (dd, *J* = 6.0, 6.0 Hz, 2H, Ar-H, major tautomer), 7.54 (dd, *J* = 6.0, 6.0 Hz, 0.2H, Ar-H, minor tautomer), 7.37-7.26 (m, 2.38H, Ar-H, major and minor tautomer) ppm; ^{13}C NMR (DMSO-d6, 75 MHz) δ 165.4 (C=S, minor tautomer), 161.7 (C=S, major tautomer), 161.5 (d, *J* = 247.5 Hz, C-F, minor tautomer), 161.0 (d, *J* = 245.3 Hz, C-F, major tautomer), 134.7 (d, *J* = 3.0 Hz, C_q-N, minor tautomer), 134.1 (d, *J* = 3.0 Hz, C_q-N, major tautomer), 126.0 (d, *J* = 8.3 Hz, 2xCH, minor tautomer), 125.3 (d, *J* = 9.0 Hz, 2xCH, major tautomer), 116.6 (d, *J* = 23.3 Hz, 2xCH, minor tautomer), 116.1 (d, *J* = 22.5 Hz, 2xCH, major tautomer), 113.9 (CN, major tautomer), 112.7 (CN, minor tautomer) ppm; HRMS (ESI$^+$): m/z $[M + H]^+$ calcd for $C_8H_6FN_2S$: 181.0236; found: 181.0223.

(3-Fluorophenyl)carbamothioyl cyanide (1:0.39 tautomeric ratio) (1f): Dark brown solid; 85% Yield; Mp 87-89; IR (KBr) 3271 (NH), 3087, 2228 (CN), 1610, 1556 (C=S), 1486, 1446, 1395 (C=C), 1308, 1280, 1246 (C-F), 1173, 1146, 1093, 964, 864, 788, 752, 676, 633, 613, 521, 447, 432 cm^{-1}; ^{1}H NMR ($CDCl_3$, 400 MHz) δ 10.15 (br s, 1H, NH, major tautomer), 9.68 (br s, 0.39H, NH, minor tautomer), 7.85 (dt, *J* = 10.0, 2.4 Hz, 1H, Ar-H, major tautomer), 7.49-7.37 (m, 2.39H, Ar-H, both tautomers), 7.24-7.20 (m, 0.39H, Ar-H, minor tautomer), 7.18-7.10 (m, 0.78H, Ar-H, minor tautomer), 7.08-7.02 (m, 1H, Ar-H, major tautomer) ppm; ^{13}C NMR ($CDCl_3$, 100 MHz) δ 165.4 (C=S, minor tautomer), 162.9 (d, *J* = 249.0 Hz, C-F, minor tautomer), 162.4 (d, *J* = 247.0 Hz, C-F, major tautomer), 161.4 (C=S, major tautomer), 138.3 (d, *J* = 10.0 Hz, C_q-N, major tautomer), 137.9 (d, *J* = 10.0 Hz, C_q-N, minor tautomer), 131.4 (d, *J* = 9.0 Hz, CH, minor tautomer), 130.6 (d, *J* = 10.0 Hz, CH, major tautomer), 118.4 (d, *J* = 4.0 Hz, CH, minor tautomer), 117.7 (d, *J* = 4.0 Hz, CH, major tautomer), 115.9 (d, *J* = 21.0 Hz, CH, minor tautomer), 115.1 (d, *J* = 21.0 Hz, CH, major tautomer), 113.2 (CN, major tautomer), 111.8 (CN, minor tautomer), 110.2 (d, *J* = 25.0 Hz, CH, minor tautomer), 109.5 (d, *J* = 26.0 Hz, CH, major tautomer) ppm; HRMS (ESI$^+$): m/z $[M + H]^+$ calcd for $C_8H_6FN_2S$: 181.0236; found: 181.0225.

4-Nitrophenyl)carbamothioyl cyanide (1:0.1 tautomeric ratio) (1g): Yellow solid; 75% Yield; Mp 123-125, Lit[5] Mp 61-62; IR (KBr) 3269 (NH), 3075, 2238 (CN), 1624, 1595, 1570, 1514, 1496, 1413, 1386 (C=C), 1344, 1326, 1107, 871, 849, 837, 746 (C-H bend), 716, 682, 610, 526, 495 cm^{-1}; ^{1}H NMR (CD_3OD, 400 MHz) δ 8.32 (d, *J* = 8.0 Hz, 0.17H, Ar-H, minor tautomer), 8.28 (d, *J* = 8.0 Hz, 2H, Ar-H, major tautomer), 8.19 (d, *J* = 8.0 Hz, 2H, Ar-H, major tautomer), 7.68 (d, *J* = 8.0 Hz, 0.22H, Ar-H, minor tautomer) ppm; ^{13}C NMR (CD_3OD, 100 MHz) δ 164.5 (C=S), 146.7 (C-NO_2), 144.4 (C_q-N), 125.6 (2xCH), 123.5 (2xCH), 114.6 (CN) ppm; HRMS (ESI$^+$): m/z $[M + H]^+$ calcd for $C_8H_6N_3O_2S$: 208.0181; found: 208.0166.

3-(Nitrophenyl)carbamothioyl cyanide (1:0.15 tautomeric ratio) (1h): Orange solid; 83% Yield; Mp 83-84, Lit[1m] Mp 99-102; IR (KBr) 32727 (NH), 2227 (CN), 1605 (NO_2), 1558, 1525, 1477, 1394 (NO_2), 1331, 1200, 1086, 1038, 999, 936, 891, 831, 799, 738, (C-H bend), 678, 666, 611 cm^{-1}; ^{1}H NMR (DMSO-d6, 300 MHz) major tautomer: δ 11.51 (br s, 1H, NH), 8.91 (t, *J* = 2.2 Hz, 1H, Ar-H), 8.22-8.17 (m, 2H), 7.78 (t, *J* = 8.4 Hz, 1H, Ar-H) ppm; ^{13}C NMR (DMSO-d6, 75 MHz) δ 162.7 (C=S, major tautomer), 147.7 (C-NO_2), 138.7 (C_q-N), 130.8 (CH, major tautomer),

129.0 (CH, major tautomer), 122.3 (CH, major tautomer), 117.1 (CH, major tautomer), 113.8 (CN, major tautomer) ppm; HRMS (ESI$^+$): m/z [M + H]$^+$ calcd for $C_8H_6N_3O_2S$: 208.0181; found: 208.0174.

(4-methoxyphenyl)carbamothioyl cyanide (1:0.21 tautomeric ratio) (1i): Bright yellow solid; 79% Yield; Mp 116-117, Lit[6] Mp 118-119; IR (KBr) 3257 (NH), 3068, 2239 (CN), 1614, 1548, 1442, 1398 (C=C), 1172 (C-O), 1113, 748 (C-H bend), 609 cm^{-1}; ^{1}H NMR (DMSO-d6, 400 MHz) δ 7.76 (d, *J* = 8.0 Hz, 2H, Ar-H, major tautomer), 7.31 (d, *J* = 8.0 Hz, 0.43H, Ar-H, minor tautomer), 6.95 (d, *J* = 8.0 Hz, 2.41H, Ar-H, both tautomers), 3.72 (s, 3.65H, OCH_3, both tautomers) ppm; ^{13}C NMR (DMSO-d6, 100 MHz) δ 160.7 (C=S, major tautomer), 159.1 (O-C_q), 131.5 (C_q-N, major tautomer), 125.8 (C_q-N, minor tautomer), 125.1 (2xCH, major tautomer), 115.6 (CN, minor tautomer), 115.0 (2xCH, major tautomer), 114.6 (CN, major tautomer), 56.3 (OMe, both tautomers) ppm; HRMS (ESI$^+$): m/z [M + H]$^+$ calcd for $C_9H_9N_2OS$: 193.0436; found: 193.0439.

(4-Ethoxyphenyl)carbamothioyl cyanide (1:0.59 tautomeric ratio) (1j): 88% Yield; Bright yellow solid Mp 113-114, Lit[6] Mp 111-112; IR (KBr) 3272 (NH), 3076, 2224 (CN), 608, 1509, 1410, 1392, 1307, 1263, 1178, 1130, 1114, 1092, 1042, 923, 830, 820, 794, 753, 734, 634, 606, 517 cm^{-1}; ^{1}H NMR ($CDCl_3$, 400 MHz) δ 9.78 (br s, 0.6H, NH, major tautomer), 9.62 (br s, 0.37H, NH, minor tautomer), 7.72 (d, *J* = 8.0 Hz, 1.17H, Ar-H, major tautomer), 7.29 (d, *J* = 8.0 Hz, 0.73H, Ar-H, minor tautomer), 6.95-6.91 (m, 2H, Ar-H, major and minor tautomer), 4.05 (q, *J* = 7.0 Hz, 2H, major and minor tautomer), 1.43 (t, *J* = 8.0 Hz, 1.14H, minor tautomer), 1.42 (t, *J* = 7.0 Hz, 1.86H, major tautomer); ^{13}C NMR ($CDCl_3$, 100 MHz) δ 165.7 (C=S, minor tautomer), 160.6 (C=S, major tautomer), 159.3 (O-C, minor tautomer), 158.3 (O-C, major tautomer), 129.9 (C-N, major tautomer), 129.7 (C-N, minor tautomer), 124.8 (CH, minor tautomer), 124.0 (CH, major tautomer), 115.5 (*o*-CH, minor tautomer), 114.8 (*o*-CH, major tautomer), 113.6 (CN, major tautomer), 112.0 (CN, minor tautomer), 64.0 (OCH_2, minor tautomer), 63.9 (OCH_2, major tautomer), 14.7 (CH_3, major tautomer), 14.6 (CH_3, minor tautomer).

(4-(Benzyloxy)phenyl)carbamothioyl cyanide (1:0.11 tautomeric ratio) (1k): Bright yellow solid; 91% Yield; Mp 136-137; IR (KBr) 3231 (NH), 3116, 2233 (CN), 1608, 1548, 1507, 1466, 1454, 1422, 1387, 1302, 1249, 1175, 1108, 1007, 920, 873, 818, 753, 739, 701, 635, 625, 515 cm^{-1}; ^{1}H NMR (DMSO-d6, 400 MHz) δ 7.87 (d, *J* = 8.0 Hz, 2H, Ar-H, major tautomer), 7.48-7.30 (m, 6.41H, Ar-H, major and minor tautomer), 7.11 (d, *J* = 8.0 Hz, 0.2H, Ar-H, major tautomer), 7.06 (d, *J* = 8.0 Hz, 0.26H, Ar-H, minor tautomer), 5.13 (s, 2H, OCH_2, major tautomer), 5.12 (s, 0.26H, OCH_2, minor tautomer). ^{13}C NMR (DMSO-d6, 100 MHz) δ 164.5 (C=S, minor tautomer), 159.7 (C=S, major tautomer), 158.2 (O-C, minor tautomer), 157.3 (O-C, major tautomer), 136.7 (C_{Bn}, major tautomer), 136.6 (C_{Bn}, minor tautomer), 131.4 (C-N, minor tautomer), 131.1 (C-N, major tautomer), 128.2 (2xCH, major tautomer), 128.1 (CH, major tautomer), 127.93 (2xCH, minor tautomer), 127.90 (2xCH, major tautomer), 127.5 (CH, minor tautomer), 125.1 (2xCH, minor tautomer), 124.2 (2xCH, major tautomer), 116.0 (2xCH, minor tautomer), 115.5 (2xCH, minor tautomer), 115.1 (2xCH, major tautomer), 114.0 (CN, major tautomer), 112.8 (CN, minor tautomer), 69.7 (OCH_2, minor tautomer), 69.6 (OCH_2, major tautomer); HRMS (ESI$^+$): m/z [M + H]$^+$ calcd for $C_{15}H_{13}N_2OS$: 269.0749; found: 269.0741.

(4-(Methylthio)phenyl)carbamothioyl cyanide (1:0.19 tautomeric ratio) (1l): Orange solid; 92% Yield; Mp 123-124; IR (KBr) 3261 (NH), 3105, 2231 (CN), 1606, 1590, 1536, 1492, 1437, 1419, 1386, 1281, 1187, 1094, 959, 813, 755, 730, 605, 503 cm^{-1}; ^{1}H NMR (DMSO-d6, 400 MHz) δ 13.48 (s, 1.19 H, NH, major and minor tautomer), 7.86 (d, J = 8.0 Hz, 2H, Ar-H, major tautomer), 7.31 (d, J = 8.0 Hz, 0.37H, Ar-H, minor tautomer), 7.33 (d, J = 8.0 Hz, 2.37H, Ar-H, major and minor tautomer), 2.48 (s, 1.11H, CH_3, minor tautomer), 2.47 (s, 3H, CH_3, major tautomer) ppm; ^{13}C NMR (DMSO-d6, 100 MHz) δ 164.4 (C=S, minor tautomer), 160.3 (C=S, major tautomer), 139.1 (C_q-SCH_3, minor tautomer), 138.3 (C_q-CH_3, major tautomer), 135.0 (C_q-N, minor tautomer), 134.7 (C_q-N, major tautomer), 126.4 (2xCH, minor tautomer), 125.9 (2xCH, major tautomer), 123.8 (2xCH, minor tautomer), 123.0 (2xCH, major tautomer), 113.9 (CN, major tautomer), 112.8 (CN, minor tautomer), 14.6 (CH_3, major tautomer) ppm; HRMS (ESI$^+$): m/z $[M + H]^+$ calcd for $C_9H_9N_2S_2$: 209.0207; found: 209.0217.

Methyl 4-((cyanocarbonothioyl)amino)benzoate (1:0.13 tautomeric ratio) (1m): Orange solid; 86% Yield; Mp 172-173; IR (KBr) 3282 (NH), 3074, 2230 (CN), 1696 (C=O), 1604, 1546, 1509, 1433, 1396, 1288, 1138, 988, 853, 821, 770, 746, 712, 690, 609, 511, 485 cm^{-1}; ^{1}H NMR (DMSO-d6, 400 MHz) δ 13.63 (s, 1.14 H, NH, major and minor tautomer), 8.06 (collapsed AB quartet, 4.51H, Ar-H, major and minor tautomer), 7.65 (d, J = 8.0 Hz, 0.51H, Ar-H, minor tautomer), 3.86 (s, 0.38H, OCH_3, minor tautomer), 3.86 (s, 3H, OCH_3, major tautomer) ppm; ^{13}C NMR (DMSO-d6, 100 MHz) δ 165.5 (C=O, minor tautomer), 165.4 (C=O, major tautomer), 165.2 (C=S, minor tautomer), 162.2 (C=S, major tautomer), 141.9 (O=C-C_q, minor tautomer), 141.7 (O=C-C_q, major tautomer), 130.7 (2xCH, minor tautomer), 130.3 (2xCH, major tautomer), 128.8 (C_q-N, minor tautomer), 128.2 (C_q-N, major tautomer), 123.0 (2xCH, minor tautomer), 122.4 (2xCH, major tautomer), 113.8 (CN, major tautomer), 112.7 (CN, minor tautomer), 52.5 (OMe, minor tautomer), 52.4 (OMe, major tautomer) ppm; HRMS (ESI$^+$): m/z $[M + H]^+$ calcd for $C_{10}H_9N_2O_2S$: 221.0385; found: 221.0394.

Ethyl 4-((cyanocarbonothioyl)amino)benzoate (1:0.09 tautomeric ratio) (1n): Orange solid; 94% Yield; Mp 174-176. Lit[1k] Mp 182; IR (KBr) 3266 (NH), 3073, 2227 (CN), 1697 (C=O), 1605, 1552, 1508, 1474, 1423, 1393, 1367, 1178, 1138 (C-O), 1102, 1017, 854, 772, 748 (C-H bend), 692, 616, 511, 500 cm^{-1}; ^{1}H NMR (DMSO-d6, 400 MHz) δ 8.05 (collapsed AB quartet, 4.35H, Ar-H, major and minor tautomer), 7.64 (d, J = 8.0 Hz, 0.35H, Ar-H, minor tautomer), 4.31 (q, J = 8.0 Hz, 2.18H, OCH_2, major and minor tautomer), 1.31 (t, J = 8.0 Hz, 3.26H, CH_3, major and minor tautomer) ppm; ^{13}C NMR (DMSO-d6, 100 MHz) δ 165.2 (C=O, minor tautomer), 165.0 (C=S, minor tautomer), 164.9 (C=O, major tautomer), 162.2 (C=S, major tautomer), 141.8 (O=C-C_q, minor tautomer), 141.6 (O=C-C_q, major tautomer), 130.6 (2xCH, minor tautomer), 130.3 (2xCH, major tautomer), 129.0 (C_q-N, minor tautomer), 128.5 (C_q-N, major tautomer), 123.0 (2xCH, minor tautomer), 122.4 (2xCH, major tautomer), 113.8 (CN, major tautomer), 112.7 (CN, minor tautomer), 61.1 (OCH_2, minor tautomer), 61.0 (OCH_2, major tautomer), 14.2 (CH_3) ppm.

4-(Trifluoromethyl)phenyl)carbamothioyl cyanide (1:0.37 tautomeric ratio) (1o): Dark orange solid; 84% Yield; Mp 101-102; IR (KBr) 3271 (NH), 3075, 2234 (CN), 1612, 1549, 1514, 1394, 1314, 1216, 1173, 1116, 1066, 1012, 839 (C-H bend), 741, 616, 590, 525, 509, 460 cm^{-1}; ^{1}H NMR ($CDCl_3$, 300 MHz) δ 10.3 (br s, 1.10H, NH), 7.90 (d, J = 9.0 Hz, 2H, Ar-H, major tautomer), 7.71-

7.56 (m, 2.70H, Ar-H, major and minor tautomer), 7.46 (d, J = 9.0 Hz, 0.74H, Ar-H, minor tautomer) ppm; ^{13}C NMR ($CDCl_3$, 75 MHz) δ 165.1 (C=S, minor tautomer), 161.8 (C=S, major tautomer), 139.9 (C_q-N, major tautomer), 139.6 (C_q-N, minor tautomer), 129.5 (q, J = 33 Hz, C-CF_3, major tautomer), 127.2 (q, J = 3.8 Hz, 2xCH, minor tautomer), 126.5 (q, J = 3.8 Hz, 2xCH, major tautomer), 123.4 (q, J = 270.8 Hz, CF_3, major tautomer), 122.5 (2xCH, minor tautomer), 121.1 (2xCH, major tautomer), 113.0 (CN, major tautomer), 111.8 (CN, minor tautomer) ppm; HRMS (ESI^+): m/z $[M + H]^+$ calcd for $C_9H_6F_3N_2S$: 231.0204; found: 231.0224.

(4-Ethylphenyl)carbamothioyl cyanide ($CDCl_3$: 1:0.25 tautomeric ratio) (1p): Light brown solid; 82% Yield; Mp 84-85; IR (KBr) 3278 (NH), 2229 (CN), 1610, 1544, 1508, 1403, 1088, 812, 724, 610, 581, 529, 519 cm^{-1}; ^{1}H NMR (DMSO-d6, 400 MHz) δ 7.81 (d, J = 8.0 Hz, 2H, Ar-H, major tautomer), 7.39 (d, J = 8.0 Hz, 0.50H, Ar-H, minor tautomer), 7.32 (d, J = 8.0 Hz, 2.49H, major and minor tautomer), 2.61 (q, J = 8.0 Hz, 2.51H, major and minor tautomer), 1.18 (t, J = 8.0 Hz, 0.75H, minor tautomer), 1.17 (t, J = 8.0 Hz, 3H, major tautomer); ^{13}C NMR ($CDCl_3$, 100 MHz) δ 164.9 (C=S, minor tautomer), 161.0 (C=S, major tautomer), 144.6 (C, minor tautomer), 144.2 (C, major tautomer), 136.3 (C-N, minor tautomer), 135.9 (C-N, major tautomer), 129.2 (2xCH, minor tautomer), 128.7 (2xCH, major tautomer), 123.6 (2xCH, minor tautomer), 122.9 (2xCH, major tautomer), 113.6 (CN, major tautomer), 112.0 (CN, minor tautomer), 28.4 (CH_2, major tautomer), 28.2 (CH_2, minor tautomer), 15.8 (CH_3, major & minor tautomer); HRMS (ESI^+): m/z $[M + H]^+$ calcd for $C_{10}H_{11}N_2S$: 191.0643; found: 191.0651.

4-(Iodophenyl)carbamothioyl cyanide (1:0.15 tautomeric ratio) (1q): Orange solid; 94% Yield; Mp 138-139, Lit[1k] Mp 151; IR (KBr) 3257 (NH), 3105, 2232 (CN), 1607, 1577, 1541, 1483, 1418, 1390, 1282, 1094, 1061, 1003, 849, 813, 761, 743, 610, 504 cm^{-1}; ^{1}H NMR (DMSO-d6, 400 MHz) δ 13.54 (s, 1H, NH, major tautomer), 11.14 (s, 0.15H, NH, minor tautomer), 7.84 (d, J = 8.0 Hz, 2.30H, Ar-H, major and minor tautomer), 7.71 (d, J = 8.0 Hz, 2H, Ar-H, major tautomer), 7.31 (d, J = 8.0 Hz, 0.30H, Ar-H, minor tautomer) ppm; ^{13}C NMR (DMSO-d6, 400 MHz)) δ 164.8 (C=S, minor tautomer), 161.4 (C=S, major tautomer), 138.4 (2xCH, minor tautomer), 137.9 (2xCH, major tautomer), 137.8 (C_q-N, minor tautomer), 137.4 (C_q-N, major tautomer), 125.3 (2xCH, minor tautomer), 124.5 (2xCH, major tautomer), 113.9 (CN, major tautomer), 112.6 (CN, minor tautomer), 94.2 (C-I, minor tautomer), 93.2 (C-I, major tautomer) ppm.

3-Chlorophenyl)carbamothioyl cyanide (1:0.28 tautomeric ratio) (1r): Orange solid; 83% Yield; Mp 86-87, Lit[1m] Mp 88-91.5; IR (KBr) 3265 (NH), 2233 (CN), 1608, 1591, 1547, 1472, 1403 (C=C), 1210, 1098, 996, 904, 869, 785 (C-H bend), 743 (C-Cl), 686, 672, 631, 613 cm^{-1}; ^{1}H NMR ($CDCl_3$, 300 MHz) δ 8.22 (s, 0.83H, NH), 7.97 (t, J = 3.0 Hz, 1.04H, Ar-H), 7.66 (d, J = 6.0 Hz, 1.07H, Ar-H), 7.47-7.24 (m, 3.45H, Ar-H) ppm; ^{13}C NMR ($CDCl_3$, 75 MHz) δ 165.3 (C=S, minor tautomer), 161.4 (C=S, major tautomer), 138.0 (C_q-N, major tautomer), 137.7 (C_q-N, minor tautomer), 135.5 (C-Cl, minor tautomer), 138.8 (C-Cl, major tautomer), 130.9 (CH, minor tautomer), 130.3 (CH, major tautomer), 128.9 (CH, minor tautomer), 128.1 (CH, major tautomer), 122.8 (CH, minor tautomer), 122.1 (CH, major tautomer), 120.9 (CH, minor tautomer), 120.3 (CH, major tautomer), 113.1 (CN, major tautomer), 111.7 (CN, minor tautomer) ppm; HRMS (ESI^+): m/z $[M + H]^+$ calcd for $C_8H_6ClN_2S$: 196.9940; found: 196.9942.

3-Bromophenyl)carbamothioyl cyanide (1:0.32 tautomeric ratio) (1s): Yellow solid; 94% Yield; Mp 97-98; IR (KBr) 3270 (NH), 3081, 2232 (CN), 1607, 1590, 1544, 1474, 1390 (C=C), 1298, 1202, 1100, 1089, 995, 888, 872, 856 (C-H bend), 780 (C-Br), 739, 671 cm^{-1}; ^{1}H NMR ($CDCl_3$, 300 MHz) δ 10.3 (br s, 0.95H, NH), 7.99 (t, J = 3.0 Hz, 0.95H, Ar-H), 7.64 (ddd, J = 6.0, 3.0, 3.0 Hz, 0.99H, Ar-H), 7.48-7.35 (m, 1.58H, Ar-H), 7.33-7.14 (m, 1.77H, Ar-H) ppm; ^{13}C NMR ($CDCl_3$, 75 MHz) δ 165.6 (C=S, minor tautomer), 161.8 (C=S, major tautomer), 138.2 (C_q-N, major tautomer), 138.0 (C_q-N, minor tautomer), 131.9 (CH, minor tautomer), 131.3 (CH, minor tautomer), 131.1 (CH, major tautomer), 130.6 (CH, major tautomer), 125.8 (CH, minor tautomer), 125.1 (CH, major tautomer), 123.4 (C-Br, minor tautomer), 122.7 (C-Br, major tautomer), 121.5 (CH, minor tautomer), 120.9 (CH, major tautomer), 113.3 (CN, major tautomer), 111.8 (CN, minor tautomer) ppm; HRMS (ESI^+): m/z $[M + H]^+$ calcd for $C_8H_6BrN_2S$: 240.9435; found: 240.9413.

3-Iodophenyl)carbamothioyl cyanide (1:0.28 tautomeric ratio) (1t): Yellow solid; 51% Yield; Mp 121-122; IR (KBr) 3266 (NH), 2233 (CN), 1605, 1585, 1542, 1471, 1421, 1390, 1297, 1111, 992, 871, 784, 748, 730, 673, 657, 608, 524 cm^{-1}; ^{1}H NMR (DMSO-d6, 400 MHz) δ 13.51 (br s, 1H, NH, major tautomer), 11.15 (br s, 0.28H, NH, minor tautomer), 8.32 (br s, 1H, CH, major tautomer), 7.88 (br s, 0.28H, CH, minor tautomer), 7.82 (dd, J = 8.0, 2.0 Hz, 1H, Ar-H, major tautomer), 7.78 (d, J = 8.0 Hz, 0.28H, Ar-H, minor tautomer), 7.72 (d, J = 8.0 Hz, 1H, Ar-H, major tautomer), 7.54 (dd, J = 8.0, 2.0 Hz, 0.28H, Ar-H, minor tautomer), 7.28 (d, J = 8.0 Hz, 1.28H, Ar-H, major and minor tautomer) ppm; ^{13}C NMR (DMSO-d6, 400 MHz) δ 165.2 (C=S, minor tautomer), 161.9 (C=S, major tautomer), 138.3 (C_q-N, minor tautomer), 138.8 (C_q-N, major tautomer), 136.9 (CH, minor tautomer), 136.4 (CH, major tautomer), 131.6 (CH, minor tautomer), 131.4 (CH, minor tautomer), 131.1 (CH, major tautomer), 130.8 (CH, major tautomer), 122.8 (CH, minor tautomer), 122.2 (CH, major tautomer), 113.7 (CN, major tautomer), 112.6 (CN, minor tautomer), 95.0 (C-I, minor tautomer), 94.5 (C-I, major tautomer) ppm; HRMS (ESI^+): m/z $[M + H]^+$ calcd for $C_8H_6IN_2S$: 288.9296; found: 288.9287.

3-(Cyanophenyl)carbamothioyl cyanide (1:0.31 tautomeric ratio) (1u): Orange solid; 85% Yield; Mp 144-45; IR (KBr) 3220 (NH), 2238 (CN), 1585, 1545, 1473,1434, 1375, 1249, 1112, 890, 801, 722, 678 cm^{-1}; ^{1}H NMR (DMSO-d6, 400 MHz) δ 13.63 (br s, 1H, NH, major tautomer), 11.36 (br s, 0.31H, NH, minor tautomer), 8.35-8.32 (m, 1H, CH, major tautomer), 8.10-8.04 (m, 1.31H, CH, major and minor tautomer), 7.91-7.82 (m, 1.62H, CH, major and minor tautomer), 7.72 (t, J = 8.0 Hz, 0.31H, Ar-H, minor tautomer), 7.70 (t, J = 8.0 Hz, 1H, Ar-H, major tautomer) ppm; ^{13}C NMR (DMSO-d6, 400 MHz) δ 165.8 (C=S, minor tautomer), 162.9 (C=S, major tautomer), 138.9 (C_q-N, minor tautomer), 138.2 (C_q-N, major tautomer), 131.9 (CH, minor tautomer), 131.4 (CH, major tautomer), 131.1 (CH, minor tautomer), 130.8 (CH, major tautomer), 128.4 (CH, minor tautomer), 127.9 (CH, major tautomer), 126.9 (CH, minor tautomer), 126.1 (CH, major tautomer), 118.1 (C, major tautomer), 117.9 (C, minor tautomer) 113.7 (CN, major tautomer), 112.5 (CN, minor tautomer), 112.4 (CN, minor tautomer), 112.0 (CN, major tautomer) ppm; HRMS (ESI^+): m/z $[M + H]^+$ calcd for $C_9H_6N_3S$: 188.0282; found: 188.0291.

3-(Trifluoromethyl)phenyl)carbamothioyl cyanide (1:0.19 tautomeric ratio) (1v): Orange solid; 87% Yield; Mp 103-104; IR (KBr) 3280 (NH), 2235 (CN), 1621, 1601, 1569, 1483, 1451, 1402,

1325, 1286, 1205, 1156, 1132, 1111, 1071, 999, 910, 892, 800, 736, 692, 658, 635, 612 cm^{-1}; ^{1}H NMR (DMSO-d6, 400 MHz) δ 13.72 (br s, 1H, NH, major tautomer), 11.36 (br s, 0.19H, NH, major tautomer), 8.34 (br s, 1H, Ar-H, major tautomer), 8.11-8.05 (m, 1H, Ar-H, major tautomer), 7.93 (br s, 1H, Ar-H, minor tautomer), 7.85-7.75 (m, 1H, Ar-H, minor tautomer), 7.73 (d, *J* = 5.2 Hz, 1H, Ar-H, major tautomer) ppm; ^{13}C NMR (DMSO-d6, 100 MHz) δ 165.7 (C=S, minor tautomer), 162.6 (C=S, major tautomer), 138.8 (C_q-N, minor tautomer), 138.3 (C_q-N, major tautomer), 130.9 (CH, minor tautomer), 130.6 (CH, major tautomer), 129.7 (q, *J* = 32 Hz, C-CF_3, major tautomer), 127.3 (CH, minor tautomer), 126.7 (CH, major tautomer), 124.7 (q, *J* = 4.0 Hz, C-H, minor tautomer), 124.3 (q, *J* = 4.0 Hz, C-H, major tautomer), 123.6 (q, *J* = 271.0 Hz, CF_3, major tautomer), 120.2 (q, *J* = 4.0 Hz, C-H, minor tautomer), 119.2 (q, *J* = 4.0 Hz, C-H, major tautomer), 113.8 (CN, major tautomer), 112.6 (CN, minor tautomer) ppm; HRMS (ESI^+): m/z $[M + H]^+$ calcd for $C_9H_6F_3N_2S$: 231.0204; found: 231.0224.

(4-Methyl-1,3-phenylene)dicarbamothioyl cyanide (1:0.29 tautomeric ratio) (1w): Bright orange solid; 85% Yield; Mp 120-122. Lit[1n] Mp 129; IR (KBr) 3263 (NH), 3049, 2232 (CN), 1602, 1541, 1494, 1391, 1245, 1122, 1104, 867, 819, 748, 670, 609, 568, 515 cm^{-1}; ^{1}H NMR (DMSO-d6, 400 MHz) δ 13.45 (br s, 1H, NH, major tautomer), 8.02 (d, *J* = 2.4 Hz, 1H, Ar-H, major tautomer), 7.96 (d, *J* = 2.4 Hz, 0.28H, Ar-H, minor tautomer), 7.80 (dd, *J* = 8.4, 2.4 Hz, 1H, Ar-H, major tautomer), 7.69 (dd, *J* = 8.4, 2.4 Hz, 0.28H, Ar-H, minor tautomer), 7.47 (d, *J* = 8.4 Hz, 1H, Ar-H, major tautomer), 7.43 (d, *J* = 8.4 Hz, 0.28H, Ar-H, minor tautomer), 2.35 (s, 0.84H, CH_3, minor tautomer), 2.21 (s, 3H, CH_3, major tautomer) ppm; ^{13}C NMR (DMSO-d6, 400 MHz) δ 164.5 (2xC=S, major tautomer), 161.3 (2xC=S, minor tautomer), 136.4 (C_q-N, minor tautomer), 136.1 (C_q-N, major tautomer), 131.9 (CH, minor tautomer), 135.3 (**C**-Me, major tautomer), 134.7 (**C**-Me, minor tautomer), 134.4 (C_q-N, minor tautomer), 133.7 (C_q-N, major tautomer), 131.6 (CH, major tautomer), 131.4 (CH, minor tautomer), 122.8 (CH, major tautomer), 122.1 (CH, minor tautomer), 120.0 (CH, major tautomer), 119.5 (CH, minor tautomer), 113.77 (CN, major tautomer), 113.75 (CN, minor tautomer), 113.73 (CN, minor tautomer), 113.72 (CN, major tautomer), 112.4 (CN, minor tautomer), 112.0 (CN, major tautomer), 17.9 (CH_3, minor tautomer), 17.4 (CH_3, major tautomer) ppm.

1,4-Phenylenedicarbamothioyl cyanide (1:0.22 tautomeric ratio) (1x): Bright orange solid; 87% Yield; Mp 160-162, Lit[1o] Mp 166-170; IR (KBr) 3261 (NH), 3074, 2232 (CN), 1587, 1509, 1426, 1367, 1310, 1097, 813, 750, 719, 609, 518, 463 cm^{-1}; ^{1}H NMR (DMSO-d6, 400 MHz) δ 13.64 (s, 1H, NH, major tautomer), 11.26 (s, 0.22H, NH, minor tautomer), 8.03 (br s, 4.88H, Ar-H, major and minor tautomer), 7.61 (d, *J* = 8.0 Hz, 0.88H, Ar-H, minor tautomer) ppm; ^{13}C NMR (DMSO-d6, 100 MHz) δ 164.7 (C=S, minor tautomer), 161.2 (C=S, major tautomer), 137.1 (C_q-N, minor tautomer), 136.4 (C_q-N, major tautomer), 123.8 (2xCH, minor tautomer), 123.6 (2xCH, minor tautomer), 123.1 (4xCH, major tautomer), 113.8 (CN, major tautomer), 112.7 (CN, minor tautomer) ppm.

2-(Bromophenyl)carbamothioyl cyanide (1:0.28 tautomeric ratio) (1y): Orange solid; 63% Yield; Mp 100-101, Lit[1k] Mp 101; IR (KBr) 3227 (NH), 2236 (CN), 1579, 1523, 1440, 1378, 1105, 1047, 1026, 852,765, 735, 670, 655, 607 cm^{-1}; ^{1}H NMR (DMSO-d6, 400 MHz) δ 13.45 (br s, 1H, NH, major tautomer), 7.84 (dd, *J* = 8.0, 1.2 Hz, 0.28H, Ar-H, minor tautomer), 7.79 (d, *J* = 8.0 Hz, 1H,

Ar-H, major tautomer), 7.70 (dd, J = 8.0, 1.2 Hz, 0.28H, Ar-H, minor tautomer), 7.55 (td, J = 8.0, 1.2 Hz, 0.28H, Ar-H, minor tautomer), 7.52-7.48 (m, 2H, Ar-H, major tautomer), 7.44 (td, J = 8.0, 1.6 Hz, 0.28H, Ar-H, minor tautomer), 7.40-7.33 (m, 1H, Ar-H, major tautomer); ^{13}C NMR (DMSO-d6, 100 MHz) δ 167.7 (C=S, minor tautomer), 165.1 (C=S, major tautomer), 137.3 (C_q-N, minor tautomer), 135.4 (C_q-N, major tautomer), 133.6 (CH, minor tautomer), 133.4 (CH, major tautomer), 131.4 (CH, minor tautomer), 130.6 (CH, major tautomer), 129.2 (CH, minor tautomer), 128.94 (CH, major tautomer), 128.91 (CH, major tautomer), 128.88 (CH, minor tautomer), 120.1 (C-Br, minor tautomer), 119.9 (C-Br, major tautomer), 113.7 (CN, major tautomer), 112.2 (CN, minor tautomer) ppm.

(2,4-dichlorophenyl)carbamothioyl cyanide (1:0.24 tautomeric ratio) (1z): Bright orange solid; 95% Yield; Mp 116-117, Lit[1m] Mp 121; IR (KBr) 3214 (NH), 3017, 2248 (CN), 1584, 1528, 1471, 1371, 1110, 1056, 867, 814, 776, 751, 686, 608, 556 cm^{-1}; ^{1}H NMR (DMSO-d6, 400 MHz) δ 7.92 (dd, J = 2.4 Hz, 0.24H, Ar-H, minor tautomer), 7.86 (dd, J = 2.0, 0.4 Hz, 1H, Ar-H, major tautomer), 7.61-7.60 (m, 0.38H, Ar-H, minor tautomer), 7.58 (d, J = 0.8 Hz, 1H, Ar-H, major tautomer), 7.57 (d, J = 2.0 Hz, 1H, Ar-H, major tautomer); ^{13}C NMR (DMSO-d6, 100 MHz) δ 168.0 (C=S, minor tautomer), 165.5 (C=S, major tautomer), 135.0 (C-Cl, minor tautomer), 134.9 (C-Cl, minor tautomer), 134.3 (C-Cl, major tautomer), 133.2 (C-Cl, major tautomer), 131.1 (C_q-N, major tautomer), 131.0 (C_q-N, minor tautomer), 130.26 (CH, minor tautomer), 130.22 (CH, major tautomer), 130.14 (CH, major tautomer), 130.09 (CH, minor tautomer), 129.0 (CH, minor tautomer), 128.8 (CH, major tautomer), 113.7 (CN, major tautomer), 112.2 (CN, minor tautomer) ppm.

(5-Chloro-2-methylphenyl)carbamothioyl cyanide (1:0.16 tautomeric ratio) (1a'): Bright orange solid; 86% Yield; Mp 102-103, Lit[1m] Mp 107; IR (KBr) 3272 (NH), 2232 (CN), 1577, 1529, 1398, 1099, 865, 800 cm^{-1}; ^{1}H NMR (DMSO-d6, 400 MHz) δ 7.92 (dd, J = 2.4 Hz, 0.24H, Ar-H, minor tautomer), 7.86 (dd, J = 2.0, 0.4 Hz, 1H, Ar-H, major tautomer), 7.61-7.60 (m, 0.38H, Ar-H, minor tautomer), 7.58 (d, J = 0.8 Hz, 1H, Ar-H, major tautomer), 7.57 (d, J = 2.0 Hz, 1H, Ar-H, major tautomer), 2.24 (s, 0.47H, CH_3, minor tautomer), 2.15 (s, 3H, CH_3, major tautomer); ^{13}C NMR (DMSO-d6, 100 MHz) δ 167.1 (C=S, minor tautomer), 164.8 (C=S, major tautomer), 138.4 (**C**-Me, minor tautomer), 136.4 (**C**-Me, major tautomer), 133.1 (C-Cl, major tautomer), 132.7 (C-Cl, major tautomer), 132.68 (CH, major tautomer), 132.63 (CH, minor tautomer), 131.0 (C_q-N, minor tautomer), 130.5 (C_q-N, major tautomer), 129.2 (CH, minor tautomer), 128.6 (CH, major tautomer), 126.4 (CH, minor tautomer), 126.1 (CH, major tautomer), 113.7 (CN, major tautomer), 112.3 (CN, minor tautomer), 17.1 (CH_3, minor tautomer), 17.0 (CH_3, major tautomer) ppm.

(2,4-dimethylphenyl)carbamothioyl cyanide (1:0.32 tautomeric ratio) (1b'): Yellow solid; 57% Yield; Mp 99-101; IR (KBr) 3286 (NH), 3081, 2229 (CN), 1499, 1377, 1216, 1133, 1105, 1039, 837, 752, 688, 636, 575, 537, 440 cm^{-1}; ^{1}H NMR (DMSO-d6, 400 MHz) δ 13.11 (br s, 1H, NH, major tautomer), 7.33 (d, J = 8.0 Hz, 0.32H, Ar-H, minor tautomer), 7.22-7.07 (m, 3.65H, Ar-H, major and minor tautomer), 2.30 (s, 0.97H, CH_3, minor tautomer), 2.28 (s, 3H, CH_3, major tautomer), 2.23 (s, 0.97H, CH_3, minor tautomer), 2.14 (s, 3H, CH_3, major tautomer); ^{13}C NMR (DMSO-d6, 100 MHz) δ 166.8 (C=S, minor tautomer), 164.3 (C=S, major tautomer), 139.1 (**C**-Me, minor tautomer), 138.2 (**C**-Me, major tautomer), 134.9 (**C**-Me, minor tautomer), 133.5 (**C**-

Me, major tautomer), 133.2 (C_q-N, minor tautomer), 132.7 (C_q-N, major tautomer), 131.7 (CH, minor tautomer), 131.6 (CH, major tautomer), 127.6 (CH, minor tautomer), 127.3 (CH, major tautomer), 126.3 (CH, minor tautomer), 125.9 (CH, major tautomer), 113.9 (CN, major tautomer), 112.6 (CN, minor tautomer), 20.77 (CH_3, major tautomer), 20.76 (CH_3, minor tautomer), 17.50 (CH_3, minor tautomer), 17.48 (CH_3, major tautomer) ppm.

Mesitylcarbamothioyl cyanide (1:0.6 tautomeric ratio) (1c'): Bright orange solid; 57% Yield; Mp 86-87; IR (KBr) 3256 (NH), 2989, 2234 (CN), 1608, 1510, 1397, 1304, 1212, 1110, 1030, 813, 819, 762, 689, 670, 618, 569 cm^{-1}; ^{1}H NMR ($CDCl_3$, 400 MHz) δ 9.10 (br s, 1H, NH, major tautomer), 9.03 (br s, 0.64H, NH, minor tautomer), 6.98 (s, 1.27H, CH, minor tautomer), 6.96 (s, 2H, CH, major tautomer), 2.31 (s, 1.91H, CH_3, minor tautomer), 2.30 (s, 3H, CH_3, major tautomer), 2.29 (s, 3.80H, 2xCH_3, minor tautomer), 2.20 (s, 6H, 2xCH_3, major tautomer); ^{13}C NMR ($CDCl_3$, 100 MHz) δ 169.5 (C=S, minor tautomer), 164.6 (C=S, major tautomer), 140.1 (**C**-Me, minor tautomer), 139.5 (**C**-Me, major tautomer), 135.3 (2x**C**-Me, minor tautomer), 134.8 (2x**C**-Me, major tautomer), 132.5 (C_q-N, minor tautomer), 130.2 (C_q-N, major tautomer), 129.7 (2xCH, minor tautomer), 129.4 (2xCH, major tautomer), 113.3 (CN, major tautomer), 111.6 (CN, minor tautomer), 21.04 (CH_3, minor tautomer), 21.06 (2xCH_3, minor tautomer), 18.2 (CH_3, major tautomer), 17.9 (2xCH_3, major tautomer) ppm.

(2,3-Dichlorophenyl)carbamothioyl cyanide (1:0.35 tautomeric ratio) (1d'): Orange solid; 94% Yield; Mp 141-143; IR (KBr) 3226 (NH), 2242 (CN), 1577, 1531, 1454, 1379, 1189, 1115, 1052, 911, 745, 699, 669 cm^{-1}; ^{1}H NMR ($CDCl_3$, 400 MHz) δ 7.80-7.73 (m, 0.35H, CH, minor tautomer), 7.70 (dd, J = 8.0, 2.0 Hz, 1H, Ar-H, major tautomer), 7.56-7.44 (m, 2.70H, Ar-H, major and minor tautomer); ^{13}C NMR ($CDCl_3$, 100 MHz) δ 167.7 (C=S, minor tautomer), 165.3 (C=S, major tautomer), 137.5 (C-Cl, minor tautomer), 135.9 (C-Cl, major tautomer), 132.9 (C-Cl, minor tautomer), 132.8 (C-Cl, major tautomer), 131.4 (CH, minor tautomer), 130.7 (CH, major tautomer), 129.2 (CH, minor tautomer), 129.0 (CH, major tautomer), 128.4 (C_q-N, minor tautomer), 128.3 (C_q-N, major tautomer), 127.5 (CH, major tautomer), 127.4 (CH, minor tautomer), 113.6 (CN, major tautomer), 112.1 (CN, minor tautomer) ppm.

(2-Chloro-5-(trifluoromethyl)phenyl)carbamothioyl cyanide (1:0.17 tautomeric ratio) (1e'): Orange solid; 81% Yield; Mp 69-70; IR (KBr) 3238 (NH), 2243 (CN), 1613, 1588, 1541, 1426, 1368, 1325, 1270, 1212, 1179, 1143, 1083, 1051, 923, 881, 810, 777, 713, 700, 616, 541 cm^{-1}; ^{1}H NMR (DMSO-d6, 400 MHz) δ 8.17 (s, 0.17H, Ar-H, minor tautomer), 8.04 (s, 1H, Ar-H, major tautomer), 7.95 (d, J = 8.4 Hz, 0.17H, Ar-H, minor tautomer), 7.90 (d, J = 8.8 Hz, 1.17H, Ar-H, major and minor tautomer), 7.81 (d, J = 8.8 Hz, 1H, Ar-H, major tautomer) ppm; ^{13}C NMR (DMSO-d6, 100 MHz) δ 168.1 (C=S, minor tautomer), 165.8 (C=S, major tautomer), 136.5 (C-Cl, minor tautomer), 135.1 (C-Cl, major tautomer), 134.4 (C_q-N, major tautomer), 134.2 (C_q-N, minor tautomer), 131.7 (CH, minor tautomer), 131.6 (CH, major tautomer), 129.2 (q, J = 30.0 Hz, C-CF_3, minor tautomer), 128.9 (q, J = 33.0 Hz, C-CF_3, major tautomer), 127.6 (q, J = 35.0 Hz, C-H, minor tautomer), 126.9 (q, J = 35.0 Hz, C-H, major tautomer), 126.0 (q, J = 38.0 Hz, C-H, major tautomer), 123.2 (q, J = 272.0 Hz, CF_3, minor tautomer), 123.4 (q, J = 271.0 Hz, CF_3, major

tautomer), 113.7 (CN, major tautomer), 112.1 (CN, minor tautomer) ppm; HRMS (ESI^+): m/z $[M + H]^+$ calcd for $C_9H_5ClF_3N_2S$: 264.9814; found: 264.9799.

2,6-(Dichlorophenyl)carbamothioyl cyanide (1:0.13 tautomeric ratio) (1f'): Light yellow solid: 73% Yield; Mp 157-159; IR (KBr) 3233 (NH), 3066, 2996, 2250 (CN), 1568, 1513, 1437, 1372, 1201, 1123, 1101, 794, 694, 656, 634, 603 cm^{-1}; ^1H NMR (DMSO-d6, 400 MHz) δ 7.74 (d, J = 8.0 Hz, 0.25H, Ar-H, minor tautomer), 7.65 (d, J = 8.0 Hz, 2H, Ar-H, major tautomer), 7.57 (t, J = 8.0 Hz, 0.25H, Ar-H, minor tautomer), 7.49 (t, J = 8.0 Hz, 1H, Ar-H, major tautomer) ppm; ^{13}C NMR (DMSO-d6, 100 MHz) δ 168.9 (C=S, minor tautomer), 165.0 (C=S, major tautomer), 133.4 (C_q-N, minor tautomer), 132.9 (C_q-N, major tautomer), 132.9 (CH, minor tautomer), 132.1 (C-Cl, major tautomer), 131.9 (C-Cl, minor tautomer), 131.3 (CH, major tautomer), 129.6 (2xCH, minor tautomer), 129.3 (2xCH, major tautomer), 113.5 (CN, major tautomer), 111.9 (CN, minor tautomer) ppm.

(4-Bromophenyl)carbamothioyl cyanide (1:0.45 tautomeric ratio) (1g'): Yellow solid; 94% Yield; Mp 127-128, Lit[1k] Mp 127; IR (KBr) 3260 (NH), 3064 (CH arom.), 2231 (CN), 1607, 1584, 1542, 1487, 1420, 1388, 1373, 1285, 1099, 1075, 1009, 852, 810, 739, 502 cm^{-1}; ^1H NMR ($CDCl_3$, 400 MHz) δ 9.76 (br s, 1H, NH, major tautomer), 9.47 (br s, 0.45H, NH, minor tautomer), 7.71 (d, J = 8.8 Hz, 2H, Ar-H, major tautomer), 7.61 (d, J = 8.8 Hz, 0.89H, Ar-H, minor), 7.57 (d, J = 8.8 Hz, 2H, Ar-H, major tautomer), 7.29 (d, J = 8.8 Hz, 0.90H, Ar-H, minor); ^{13}C NMR ($CDCl_3$, 100 MHz) δ 165.5 (C=S, major tautomer), 161.7 (C=N, minor), 135.8 (C-N, major tautomer), 135.7 (C-N, minor), 133.2 (*m*-CH, minor), 132.5 (*m*-CH, major tautomer), 124.3 (*o*-CH, minor), 123.8 (*o*-CH, major tautomer), 122.6 (C-Br, minor), 121.3 (C-Br, major tautomer), 113.4 (CN, major tautomer), 111.8 (CN, minor).

(2-Methoxy-5-methylphenyl)carbamothioyl cyanide (1:0.52 tautomeric ratio) (1h'): Bright yellow solid; 82% Yield; Mp 123-124; IR (KBr) 3243 (NH), 3017, 2227 (CN), 1617, 1593, 1545, 1492, 1457, 1436, 1396, 1372, 1309, 1259, 1188, 1127, 1100, 1031, 1005, 879, 805, 763, 700, 630, 611, 575, 517, 449 cm^{-1}; ^1H NMR (DMSO-d6, 400 MHz) δ 13.03 (br s, 1.52H, NH, major and minor tautomer), 7.34 (d, J = 2.0 Hz, 1H, Ar-H, major tautomer), 7.24 (ddd, J = 8.4, 2.4, 0.4 Hz, 1H, Ar-H, major tautomer), 7.20-7.16 (m, 1.52H, Ar-H, major and minor tautomer), 7.11 (d, J = 8.8 Hz, 0.52H, Ar-H, minor tautomer), 7.07 (d, J = 8.4 Hz, 1H, Ar-H, major tautomer), 3.81 (s, 1.55H, OCH_3, minor tautomer), 3.78 (s, 3H, OCH_3, major tautomer), 2.26 (s, 1.55H, CH_3, minor tautomer), 2.24 (s, 3H, CH_3, major tautomer); ^{13}C NMR (DMSO-d6, 100 MHz) δ 167.6 (C=S, minor tautomer), 163.6 (C=S, major tautomer), 151.5 (**C**-O, minor tautomer), 150.7 (**C**-O, major tautomer), 130.8 (CH, minor tautomer), 130.2 (CH, major tautomer), 130.1 (**C**-Me, minor tautomer), 129.5 (**C**-Me, major tautomer), 126.8 (CH, minor tautomer), 126.7 (CH, major tautomer), 126.5 (C_q-N, minor tautomer), 124.6 (C_q-N, major tautomer), 113.9 (CN, major tautomer), 112.8 (CN, minor tautomer), 112.7 (CH, minor tautomer), 112.5 (CH, major tautomer), 55.9 (OCH_3, minor tautomer), 55.8 (OCH_3, major tautomer), 20.1 (CH_3, major tautomer), 19.9 (CH_3, minor tautomer) ppm; HRMS (ESI^+): m/z $[M + H]^+$ calcd for $C_{10}H_{11}N_2OS$: 207.0592; found: 207.0581.

3,5-(Dichlorophenyl)carbamothioyl cyanide (1:0.23 tautomeric ratio) (1i'): Orange solid; 68% Yield; Mp 138-139; IR (KBr) 3265 (NH), 3080, 2241 (CN), 1611, 1589, 1550, 1447, 1383 (C=C),

1299, 1255, 1217, 1098, 852 (C-H bend), 815, 744 (C-Cl), 662 cm^{-1}; ^{1}H NMR (DMSO-d6, 400 MHz) δ 7.93 (d, *J* = 2.0 Hz, 2H, Ar-H, major tautomer), 7.64 (t, *J* = 2.0 Hz, 1H, Ar-H, major tautomer), 7.70 (t, *J* = 2.0 Hz, 1H, Ar-H, minor tautomer), 7.66 (d, *J* = 2.0 Hz, 2H, Ar-H, minor tautomer) ppm; ^{13}C NMR (DMSO-d6, 100 MHz) δ 165.7 (C=S, minor tautomer), 162.7 (C=S, major tautomer), 140.1 (C_q-N, minor tautomer), 139.7 (C_q-N, major tautomer), 134.7 (C-Cl, minor tautomer), 134.4 (C-Cl, major tautomer), 127.6 (CH, minor tautomer), 127.2 (CH, minor tautomer), 122.1 (2xCH, minor tautomer), 121.1 (2xCH, major tautomer), 113.6 (CN, major tautomer), 114.4 (CN, major tautomer) ppm; HRMS (ESI^+): m/z $[M + H]^+$ calcd for $C_8H_5Cl_2N_2S$: 230.9551; found: 230.9559.

3,4-(Dichlorophenyl)carbamothioyl cyanide (1:0.22 tautomeric ratio) (1j'): Orange solid: 80% Yield; Mp 161-162; IR (KBr) 3269 (NH), 2234 (CN), 1606, 1554, 1538, 1475, 1376, 1289, 1129, 1105, 1029, 873, 800, 742, 672, 614 cm^{-1}; ^{1}H NMR (DMSO-d6, 400 MHz) δ 8.27 (d, *J* = 2.4 Hz, 1H, Ar-H, major tautomer), 7.87 (d, *J* = 2.4 Hz, 0.22H, Ar-H, minor tautomer), 7.80 (dd, *J* = 8.8, 2.4 Hz, 1.22H, Ar-H, major and minor tautomer), 7.26 (d, *J* = 8.8 Hz, 1H, Ar-H, major tautomer), 7.53 (dd, *J* = 8.8, 2.4 Hz, 0.22H, Ar-H, minor tautomer) ppm; ^{13}C NMR (DMSO-d6, 100 MHz) δ 165.5 (C=S, minor tautomer), 162.2 (C=S, major tautomer), 137.9 (C_q-N, minor tautomer), 137.5 (C_q-N, major tautomer), 131.9 (C-Cl, minor tautomer), 131.5 (CH, minor tautomer), 131.3 (C-Cl, major tautomer), 131.1 (CH, major tautomer), 130.7 (C-Cl, minor tautomer), 129.6 (C-Cl, major tautomer), 125.1 (CH, minor tautomer), 124.0 (CH, major tautomer), 123.5 (CH, minor tautomer), 122.9 (CH, major tautomer), 113.7 (CN, major tautomer), 112.5 (CN, minor tautomer) ppm; HRMS (ESI^+): m/z $[M + H]^+$ calcd for $C_8H_5Cl_2N_2S$: 230.9551; found: 230.9528.

(2,4-Difluorophenyl)carbamothioyl cyanide (1:0.27 tautomeric ratio) (1k'): Orange solid; 82% Yield; Mp 99-100; IR (KBr) 3253 (NH), 2239 (CN), 1607, 1541 (C=S), 1499, 1443, 1391, 1296, 1264, 1192, 1148, 1100, 967, 854, 814, 753, 729, 667, 606, 590, 557, 499, 453 cm^{-1}; ^{1}H NMR (DMSO-d6, 400 MHz) δ 7.76 (td, *J* = 8.8, 4.0 Hz, 0.27H, Ar-H, minor tautomer), 7.66 (td, *J* = 8.8, 4.0 Hz, 1H, Ar-H, major tautomer), 7.62-7.55 (m, 0.27H, Ar-H, minor tautomer), 7.53-7.46 (m, 1H, Ar-H, major tautomer), 7.31-7.26 (m, 0.27H, Ar-H, minor tautomer), 7.25-7.19 (m, 1H, Ar-H, major tautomer); ^{13}C NMR (DMSO-d6, 100 MHz) δ 167.9 (C=S, minor tautomer), 165.2 (C=S, major tautomer), 162.0 (dd, *J* = 260.0, 11.0 Hz, C-F, minor tautomer), 161.8 (dd, *J* = 258.0, 11.0 Hz, C-F, major tautomer), 156.1 (dd, *J* = 250.0, 13.0 Hz, C-F, minor tautomer), 155.8 (dd, *J* = 252.0, 14.0 Hz, C-F, major tautomer), 129.2 (d, *J* = 10.0 Hz, C-H, minor tautomer), 128.9 (dd, *J* = 11.0, 3.0 Hz, C-H, major tautomer), 122.8 (dd, *J* = 12.0, 3.0 Hz, C_q-N, minor tautomer), 120.9 (dd, *J* = 12.0, 4.0 Hz, C_q-N, major tautomer), 113.6 (CN, major tautomer), 112.7 (dd, *J* = 23.0, 4.0 Hz, C-H, minor tautomer), 112.4 (dd, *J* = 23.0, 4.0 Hz, C-H, major tautomer), 112.2 (CN, minor tautomer), 105.6 (dd, *J* = 27.2, 23.9 Hz, C-H, minor tautomer), 105.4 (dd, *J* = 27.0, 24.0 Hz, C-H, major tautomer) ppm; HRMS (ESI^+): m/z $[M + H]^+$ calcd for $C_8H_5F_2N_2S$: 199.0142; found: 199.0131.

Naphthalen-1-ylcarbamothioyl cyanide (1l'): Red solid; 92% Yield; Mp 130-131, Lit[1m] Mp 136-137; IR (KBr) 3252 (NH), 2232 (CN), 1597, 1575, 1526, 1505, 1386, 1217, 1172, 1107, 963, 881, 791, 770, 752, 682, 608, 552, 536 cm^{-1}; ^{1}H NMR (DMSO-d6, 400 MHz) δ 13.70 (br s, H, NH), 8.10-7.55 (m, 7H, Ar-H) ppm; ^{13}C NMR (DMSO-d6, 100 MHz) δ 167.5 (C=S, minor tautomer),

165.6 (C=S, major tautomer), 134.6 (C_q, minor tautomer), 133.9 (C_q, major tautomer), 133.7 (C_q-N, minor tautomer), 132.7 (C_q-N, major tautomer), 129.7 (CH, minor tautomer), 129.0 (CH, major tautomer), 128.6 (CH, major tautomer), 128.5 (CH, minor tautomer), 128.0 (C_q, minor tautomer), 127.8 (CH, minor tautomer), 127.6 (C_q, major tautomer), 127.3 (CH, minor tautomer), 127.2 (CH, major tautomer), 126.9 (CH, major tautomer), 125.8 (CH, minor tautomer), 125.7 (CH, major tautomer), 124.4 (CH, major tautomer), 124.3 (CH, minor tautomer), 122.5 (CH, major tautomer), 122.3 (CH, minor tautomer), 114.0 (CN, major tautomer), 112.6 (CN, minor tautomer) ppm.

3-(Methoxyphenyl)carbamothioyl cyanide (1.91:1 tautomeric ratio) (1m'): yellow solid; 86% Yield; Mp 83-85, Lit[1m] Mp 83; IR (KBr) 3267 (NH), 3091, 2228 (CN), 1618, 1592, 1559, 1492, 1462, 1450, 1400 (C=C), 1266 (C-O), 1195, 1173, 1160, 1097 , 846, 789 (C-H bend) cm^{-1}; ^1H NMR ($CDCl_3$, 300 MHz) δ 7.57 (t, J = 6.0 Hz, 1.04H, Ar-H), 7.43-7.22 (m, 2.93H, Ar-H), 7.02-6.83 (m, 4.29H, Ar-H & NH), 3.86 (s, 1.57H, OCH_3, minor tautomer), 3.82 (s, 3H, OCH_3, major tautomer) ppm; ^{13}C NMR ($CDCl_3$, 75 MHz) δ 165.3 (C=S, minor tautomer), 161.2 (C=S, major tautomer), 160.5 (C-O, minor tautomer), 159.9 (C-O, major tautomer), 138.7 (C_q-N, major tautomer), 137.9 (C_q-N, minor tautomer), 130.8 (CH, minor tautomer), 130.1 (CH, major tautomer), 114.7 (CH, minor tautomer), 114.5 (CH, minor tautomer), 114.4 (CH, major tautomer), 113.8 (CH, major tautomer), 113.8 (CN, major tautomer), 113.6 (CN, minor tautomer), 108.3 (CH, major tautomer), 107.7 (CH, major tautomer), 55.6 (OCH_3, minor tautomer), 55.5 (OCH_3, major tautomer) ppm; HRMS (ESI^+): m/z $[M + H]^+$ calcd for $C_9H_9N_2OS$: 193.0436; found: 193.0448.

(3-(Benzyloxy)phenyl)carbamothioyl cyanide (1:0.24 tautomeric ratio) (1n'): Bright yellow solid; 92% Yield; Mp 80-82; IR (KBr) 3271 (NH), 3088, 2229 (CN), 166, 15, 1593, 1556, 1470, 1447, 1397, 1331, 1312, 1293, 1244, 1185, 1157, 1095, 1029, 912, 871, 776, 752, 734, 697, 682, 632, 616, 528, 454 cm^{-1}; ^1H NMR (DMSO-d6, 400 MHz) δ 13.48 (br s, 1.24H, NH, major and minor tautomer), 7.70 (t, J = 2.0 Hz, 1H, Ar-H, major tautomer), 7.47-7.30 (m, 7.24H, Ar-H, major and minor tautomer), 7.17 (t, J = 2.0 Hz, 0.24H, Ar-H, minor tautomer), 7.09 (d, J = 2.0 Hz, 0.24H, Ar-H, minor tautomer), 7.08-7.06 (m, 0.24H, Ar-H, minor tautomer), 7.03 (ddd, J = 8.0, 2.8, 1.2 Hz, 1H, Ar-H, major tautomer),5.13 (s, 0.48H, OCH_2, minor tautomer), 5.11 (s, 2H, OCH_2, major tautomer) ppm; ^{13}C NMR (DMSO-d6, 100 MHz) δ 164.9 (C=S, minor tautomer), 161.3 (C=S, major tautomer), 159.0 (C-O, minor tautomer), 158.5 (C-O, major tautomer), 139.2 ($\mathbf{C_q}$-CH_2, minor tautomer), 138.8 ($\mathbf{C_q}$-CH_2, major tautomer), 136.6 (C_q-N, major tautomer), 136.5 (C_q-N, minor tautomer), 128.6 (2xCH, minor tautomer), 128.5 (2xCH, major tautomer), 128.1 (CH, minor tautomer), 128.0 (CH, major tautomer), 127.9 (2xCH, minor tautomer), 127.8 (2xCH, major tautomer), 115.6 (CH, minor tautomer), 115.1 (CH, major tautomer), 114.6 (CH, minor tautomer), 114.2 (CH, major tautomer), 113.8 (CN, major tautomer), 112.8 (CN, minor tautomer), 110.0 (CH, minor tautomer), 109.0 (CH, major tautomer), 69.7 (OCH_2, minor tautomer), 69.5 (OCH_2, major tautomer) ppm; HRMS (ESI^+): m/z $[M + H]^+$ calcd for $C_{15}H_{13}N_2OS$: 269.0749; found: 269.0756.

3-(Methylthiophenyl)carbamothioyl cyanide (1:0.3 tautomeric ratio) (1o'): Bright yellow solid; 80% Yield; Mp 107-108; IR (KBr) 3271 (NH), 3125, 3080, 2227 (CN), 1609, 1579, 1550, 1472,

1430, 1391, 1332, 1305, 1168, 1098, 904, 855, 788, 759, 735, 678, 635, 607, 526 cm^{-1}; ^{1}H NMR (DMSO-d6, 400 MHz) δ 13.50 (br s, 1.3H, NH, major and minor tautomer), 7.85 (t, *J* = 2.0 Hz, 1H, Ar-H, major tautomer), 7.59 (ddd, *J* = 8.0, 2.0, 0.8 Hz, 1H, Ar-H, major tautomer), 7.41 (t, *J* = 8.0 Hz, 1.60H, Ar-H, major and minor tautomer), 7.28 (ddd, *J* = 8.0, 2.0, 0.8 Hz, 0.3H, Ar-H, minor tautomer), 7.24 (ddd, *J* = 8.0, 2.0, 0.8 Hz, 1H, Ar-H, major tautomer), 7.23 (ddd, *J* = 8.0, 2.0, 0.8 Hz, 0.3H, Ar-H, minor tautomer), 2.50 (s, 0.9H, SCH_3, minor tautomer), 2.47 (s, 3H, SCH_3, major tautomer) ppm; ^{13}C NMR (DMSO-d6, 100 MHz) δ 165.0 (C=S, minor tautomer), 161.6 (C=S, major tautomer), 140.3 (C_q-S, minor tautomer), 139.6 (C_q-S, major tautomer), 138.7 (C_q-N, minor tautomer), 138.3 (C_q-N, major tautomer), 130.0 (CH, minor tautomer), 129.7 (CH, major tautomer), 125.4 (CH, minor tautomer), 125.0 (CH, major tautomer), 120.0 (CH, minor tautomer), 119.5 (CH, minor tautomer), 119.2 (CH, major tautomer), 119.0 (CH, major tautomer), 113.8 (CN, major tautomer), 112.8 (CN, minor tautomer), 14.6 (SCH_3, major tautomer), 14.5 (SCH_3, minor tautomer) ppm; HRMS (ESI^+): m/z $[M + H]^+$ calcd for $C_9H_9N_2S_2$: 209.0207; found: 209.0201.

(3,4-dimethoxyphenyl)carbamothioyl cyanide (1:0.23 tautomeric ratio) (1p'): Bright orange-solid; 84% Yield; Mp 108-109; IR (KBr) 3268 (NH), 3085, 2228 (CN), 1613, 1602, 1555, 1510, 1463, 1409, 1272, 1241, 1164, 1144, 1090, 1010, 960, 851, 780, 775, 714, 612 cm^{-1}; ^{1}H NMR (DMSO-d6, 400 MHz) δ 12.90 (br s, 1.56H, NH, major and minor tautomer), 7.49 (d, *J* = 8.8 Hz, 1H, Ar-H, major tautomer), 7.31 (d, *J* = 8.8 Hz, 0.58H, Ar-H, minor tautomer), 6.74 (d, *J* = 2.4 Hz, 0.58H, Ar-H, minor tautomer), 6.71 (d, *J* = 2.4 Hz, 1H, Ar-H, major tautomer), 6.60 (dd, *J* = 8.8, 2.4 Hz, 0.58H, Ar-H, minor tautomer), 6.58 (dd, *J* = 8.8, 2.4 Hz, 1H, Ar-H, major tautomer), 3.84 (s, 1.74H, OCH_3, minor tautomer), 3.82 (s, 3H, OCH_3, major tautomer), 3.81 (s, 1.74H, OCH_3, minor tautomer), 3.79 (s, 3H, OCH_3, major tautomer) ppm; ^{13}C NMR (DMSO-d6, 100 MHz) δ 167.6 (C=S, minor tautomer), 163.2 (C=S, major tautomer), 161.0 (C-O, minor tautomer), 160.4 (C-O, major tautomer), 154.7 (C-O, minor tautomer), 153.9 (C-O, major tautomer), 127.5 (CH, minor tautomer), 127.2 (CH, major tautomer), 120.1 (C_q-N, minor tautomer), 118.0 (C_q-N, major tautomer), 114.0 (CN, major tautomer), 112.9 (CN, minor tautomer), 105.3 (CH, minor tautomer), 104.9 (CH, major tautomer), 99.6 (CH, minor tautomer), 99.3 (CH, major tautomer), 56.1 (OCH_3, minor tautomer), 56.0 (OCH_3, major tautomer), 55.7 (OCH_3, minor tautomer), 55.6 (OCH_3, major tautomer) ppm.

(2,5-Dimethoxyphenyl)carbamothioyl cyanide (1:0.52 tautomeric ratio) (1q'): Bright orange-yellow solid; 84% Yield; Mp 140-141; IR (KBr) 3242 (NH), 2227 (CN), 1598, 1541, 1491, 1434, 1397, 1320, 1284, 1223, 1165, 1129, 1097, 1050, 919, 939, 852, 799, 763, 746, 708, 627, 604 cm^{-1}; ^{1}H NMR (DMSO-d6, 400 MHz)) δ 13.07 (br s, 1.51H, NH, major and minor tautomer), 7.22 (d, *J* = 2.4 Hz, 1H, Ar-H, major tautomer), 7.15 (d, *J* = 9.2 Hz, 0.52H, Ar-H, minor tautomer), 7.11 (d, *J* = 9.2 Hz, 1H, Ar-H, major tautomer), 7.04 (dd, *J* = 2.8 Hz, 0.52H, Ar-H, minor tautomer), 7.00 (dd, *J* = 9.2, 3.2 Hz, 0.52H, Ar-H, minor tautomer), 6.96 (dd, *J* = 9.2, 3.2 Hz, 1H, Ar-H, major tautomer), 3.79 (s, 1.53H, OCH_3, minor tautomer), 3.77 (s, 3H, OCH_3, major tautomer), 3.73 (s, 1.53H, OCH_3, minor tautomer), 3.70 (s, 3H, OCH_3, major tautomer) ppm; ^{13}C NMR (DMSO-d6, 100 MHz) δ 167.7 (C=S, minor tautomer), 163.7 (C=S, major tautomer), 153.1 (C-O, minor tautomer), 152.7 (C-O, major tautomer), 147.6 (C-O, minor tautomer), 146.9 (C-O, major tautomer), 127.2 (C_q-N, minor tautomer), 125.4 (C_q-N, major tautomer), 115.2 (CH, minor

tautomer), 114.5 (CH, major tautomer), 113.9 (CN, major tautomer), 113.8 (CH, minor tautomer), 113.4 (CH, major tautomer), 112.8 (CN, minor tautomer), 112.5 (CH, minor tautomer), 112.2 (CH, major tautomer), 56.3 (OCH_3, major tautomer), 56.2 (OCH_3, minor tautomer), 55.8 (OCH_3, minor tautomer), 55.7 (OCH_3, major tautomer) ppm.

(2,4-Dimethoxyphenyl)carbamothioyl cyanide (1:0.58 tautomeric ratio) (1r'): Bright orange-solid; 84% Yield; Mp 139-140; IR (KBr) 3262 (NH), 3021, 2936, 2229 (CN), 1615, 1534, 1400, 1333, 1288, 1272, 1212, 1182, 1163, 1128, 1099, 1041, 1031, 922, 820, 784, 747, 627, 602, 579, 540 cm^{-1}; ^{1}H NMR (DMSO-d6, 400 MHz)) δ 12.90 (br s, 1.58H, NH, major and minor tautomer), 7.49 (d, J = 8.8 Hz, 1H, Ar-H, major tautomer), 7.31 (d, J = 8.8 Hz, 0.58H, Ar-H, minor tautomer), 6.74 (d, J = 2.4 Hz, 0.58H, Ar-H, minor tautomer), 6.71 (d, J = 2.4 Hz, 1H, Ar-H, major tautomer), 6.60 (dd, J = 8.4, 2.8 Hz, 0.58H, Ar-H, minor tautomer), 6.58 (dd, J = 8.4, 2.4 Hz, 1H, Ar-H, major tautomer), 3.84 (s, 1.74H, OCH_3, minor tautomer), 3.82 (s, 3H, OCH_3, major tautomer), 3.81 (s, 1.74H, OCH_3, minor tautomer), 3.79 (s, 3H, OCH_3, major tautomer) ppm; ^{13}C NMR (DMSO-d6, 100 MHz) δ 167.6 (C=S, minor tautomer), 163.2 (C=S, major tautomer), 161.0 (C-O, minor tautomer), 160.4 (C-O, major tautomer), 154.7 (C-O, minor tautomer), 153.9 (C-O, major tautomer), 127.5 (CH, minor tautomer), 127.2 (CH, major tautomer), 120.1 (C_q-N, minor tautomer), 118.0 (C_q-N, major tautomer), 114.0 (CN, major tautomer), 112.9 (CN, minor tautomer), 105.3 (CH, minor tautomer), 104.9 (CH, major tautomer), 99.6 (CH, minor tautomer), 99.3 (CH, major tautomer), 56.1 (OCH_3, minor tautomer), 56.0 (OCH_3, major tautomer), 55.7 (OCH_3, minor tautomer), 55.6 (OCH_3, major tautomer) ppm.

(3-Acetylphenyl)carbamothioyl cyanide (1:0.27 tautomeric ratio) (1s'): Yellow solid; 97% Yield; Mp 116-117; IR (KBr) 3264 (NH), 3077, 2227 (CN), 1653 (C=O), 1625, 1590, 1476, 1444, 1406, 1356, 1267, 1196, 1102, 983, 902, 843, 794, 765, 705, 677, 631, 591, 481 cm^{-1}; ^{1}H NMR (DMSO-d6, 400 MHz) δ 13.64 (s, 1H, NH, major and minor tautomer), 8.46 (t, J = 2.0 Hz, 1H, Ar-H, major tautomer), 8.09-8.04 (m, 1.27H, Ar-H, major and minor tautomer), 7.99 (d, J = 7.6 Hz, 0.27H, Ar-H, minor tautomer), 7.95 (d, J = 7.6 Hz, 1H, Ar-H, major tautomer), 7.79-7.75 (m, 0.27H, Ar-H, minor tautomer), 7.64 (t, J = 8.0 Hz, 1.28H, Ar-H, major and minor tautomer), 2.60 (s, 0.82H, CH_3, minor tautomer), 2.58 (s, 3H, CH_3, major tautomer) ppm; ^{13}C NMR (DMSO-d6, 100 MHz) δ 165.5 (C=O, minor tautomer), 197.1 (C=O, major tautomer), 165.3 (C=S, minor tautomer), 162.1 (C=S, major tautomer), 138.6 (C_q-N, minor tautomer), 138.0 (C_q-N, major tautomer), 137.9 (O=C-C_q, minor tautomer), 137.5 (O=C-C_q, major tautomer), 130.2 (CH, minor tautomer), 129.7 (CH, major tautomer), 127.9 (CH, minor tautomer), 127.7 (CH, minor tautomer), 127.6 (CH, major tautomer), 127.2 (CH, major tautomer), 122.6 (CH, minor tautomer), 122.0 (CH, major tautomer), 113.8 (CN, major tautomer), 112.7 (CN, minor tautomer), 27.0 (Me, minor tautomer), 26.9 (Me, major tautomer) ppm; HRMS (ESI^+): m/z $[M + H]^+$ calcd for $C_{10}H_9N_2OS$: 205.0436; found: 205.0444.

2-(Methylthiophenyl)carbamothioyl cyanide (1:0.60 tautomeric ratio) (1t'): Orange solid; 86% Yield; Mp 94-95; IR (KBr) 3223 (NH), 2235 (CN), 1586, 1510, 1460, 1442, 1379, 1273, 1210, 1109, 1070, 1038, 971, 845, 764, 732, 681, 666, 607, 516, 455 cm^{-1}; ^{1}H NMR (DMSO-d6, 400 MHz) δ 13.27 (br s, 1.6H, NH, major and minor tautomer), 7.53-7.38 (m, 3.37H, major and minor tautomer), 7.34-7.22 (m, 2.64H, major and minor tautomer), 2.50 (s, 1.8H, SCH_3, minor tautomer),

2.46 (s, 3H, SCH_3, major tautomer) ppm; ^{13}C NMR (DMSO-d6, 100 MHz) δ 168.1 (C=S, minor tautomer), 164.8 (C=S, major tautomer), 136.6 (C_q-S, minor tautomer), 135.8 (C_q-S, major tautomer), 135.6 (C_q-N, minor tautomer), 133.6 (C_q-N, major tautomer), 130.4 (CH, minor tautomer), 129.5 (CH, major tautomer), 127.4 (CH, minor tautomer), 127.2 (CH, major tautomer), 126.7 (CH, minor tautomer), 126.4 (CH, minor tautomer), 125.8 (CH, minor tautomer), 125.7 (CH, major tautomer), 113.7 (CN, major tautomer), 112.4 (CN, minor tautomer), 14.8 (SCH_3, major tautomer), 14.6 (SCH_3, minor tautomer) ppm; HRMS (ESI^+): m/z $[M + H]^+$ calcd for $C_9H_9N_2S_2$: 209.0207; found: 209.0199.

References:

[1] (a) A. M. Sh. El-Sharief, Z. Moussa, *Eur. J. Med. Chem.* **2009**, *44*, 4315–4334. (b) M. A. M. Sh. El-Sharief, El-Sharief, S. Y. Abbas, A. M. Sh. El-Sharief, N. M. Sabry, Z. Moussa, S. M. El-Messery, A. R. Elsheakh, G. S. Hassan, M. T. El Sayed, *Bioorg. Chem.* **2019**, *87*, 679–687. (c) Z. Moussa, M. A. M. Sh. El-Sharief, A. M. Sh. El-Sharief, *Eur. J. Med. Chem.* **2011**, *46*, 2280–2289. (d) M. A. M. Sh. El-Sharief, Z. Moussa, A. M. Sh. El-Sharief, *J. Fluorine Chem.* **2011**, *132*, 596–611. (e) M. A. M. Sh. El-Sharief, S. Y. Abbas, Z. Moussa, E. W. El-Gammal, A. M. Sh. El-Sharief, *Croat. Chem. Acta.* **2018**, *91*, 335–340. (f) M. A. M. Sh. El-Sharief, Z. Moussa, A. M. Sh. El-Sharief, *Arch. Pharm.Chem. Life Sci.* **2013**, *346*, 542–555. (g) Z. Moussa, M. A. M. Sh. El-Sharief, S. Y. Abbas, *Eur. J. Med. Chem.* **2016**, *122*, 419–428. (h) T. Besson, C. W. Rees, *J. Chem. Soc. Perkin Trans. 1*, **1995**, 1659–1662. (i) D. C. McCutcheon, W. B. Porterfield, J. A. Prescher, *Org. Biomol. Chem.* **2015**, *13*, 2117–2121. (j) H.-S. Lee, K. Kim, *Tetrahedron Lett.* **1996**, *37*, 869–872. (k) A. D. Grabendo, P. S. Pel'kis, *Zhurnal Obshchei Khimii*, **1961**, *31*, 2739-2743. (l) J. Huang, M. D. Graves, *J. Heterocycl. Chem.* **1987**, *24*, 1781–1785. (m) CIBA Ltd. NL. Patent 6500321, **1965**. (n) A. M. Sh. El-Sharief, A.M. Al-Amri, S.Y. Al-Raqa, *J. Sulfur Chem.*, **2006**, *27*, 245-263. (o) A. M. Sh. El-Sharief, Y. A. Ammar, M. A. Zahran, H. Kh Sabet, *J. Chem. Res., Synop.*, **2003**, 3, 162-167. (p) A. Reissert, K. Bruggemann, *Ber. Dtsch. Chem. Ges. B.,* **1924**, 57B, 981-989. (q) J.F. Olin, US Patent 3287102, **1966**. (r) E. Schiewald, H.D.Martin, K. Nadolski, C. Fieseler, W. Mueller, W. Kochmann, W. Steinke, Ger. Patent DD 252749 A1 19871230, **1987**. (s) Y. A. Ammar, S. Y. Abbas, A. M. Sh. El-Sharief, M. A. E. R. Salem, A. R. Mohamed, *Eur. J. Chem.* **2017**, 8, 76–81.
[2] M. T. Omar, *Helv. Chim. Acta*., **2008**, *91*, 1461-1470.
[3] Kumelj, B.; Vestn. Slov. Kem. Drus., **1958**, *5*, 69-73.
[4] Klopping, Hein L., US Patent 3287466, **1966**.
[5] K. Friedrich, Chem. Ber. **1979**, *112*, 1867-1872.
[6] A. D. Grabendo, *Zhurnal Obshchei Khimii*, **1960**, *30*, 1222-1226.

^{1}H NMR ($CDCl_3$) spectrum of *p*-tolylcarbamothioyl cyanide (1:0.41 tautomeric ratio) (1a)

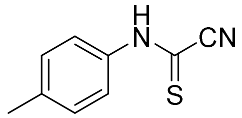

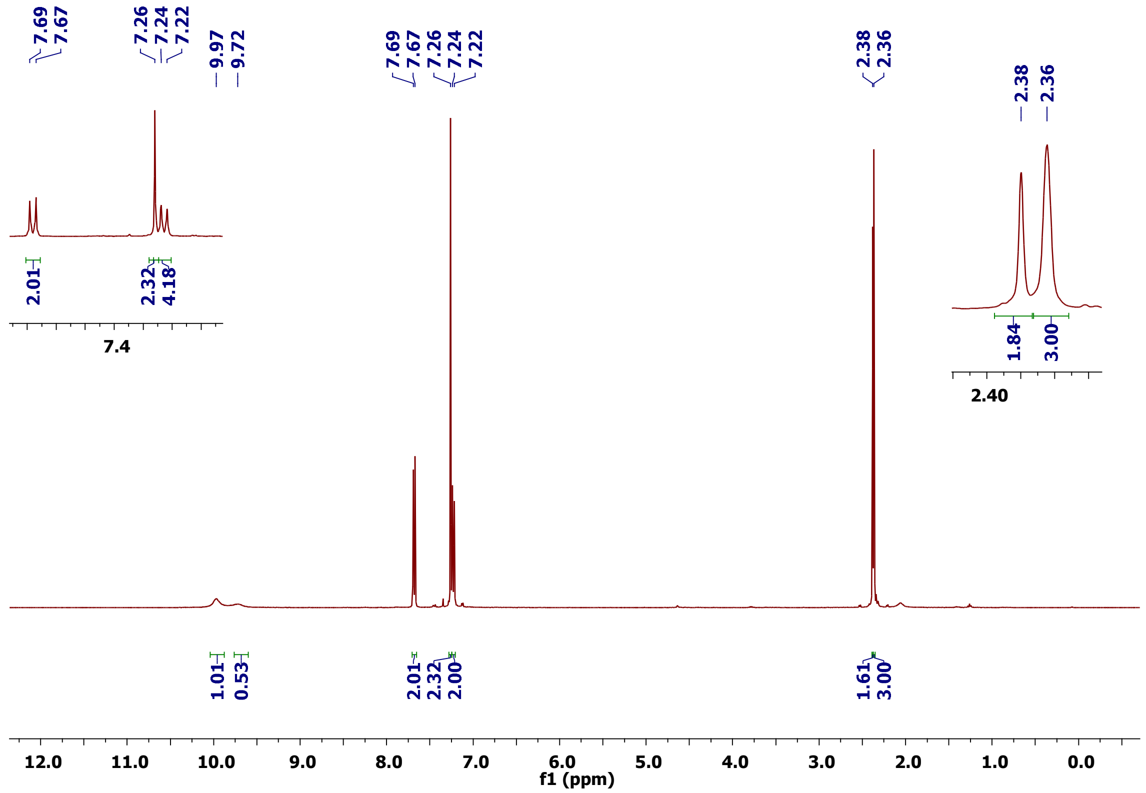

^{13}C NMR ($CDCl_3$) spectrum of *p*-tolylcarbamothioyl cyanide (1:0.41 tautomeric ratio) (1a)

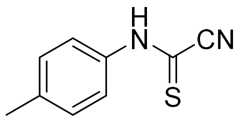

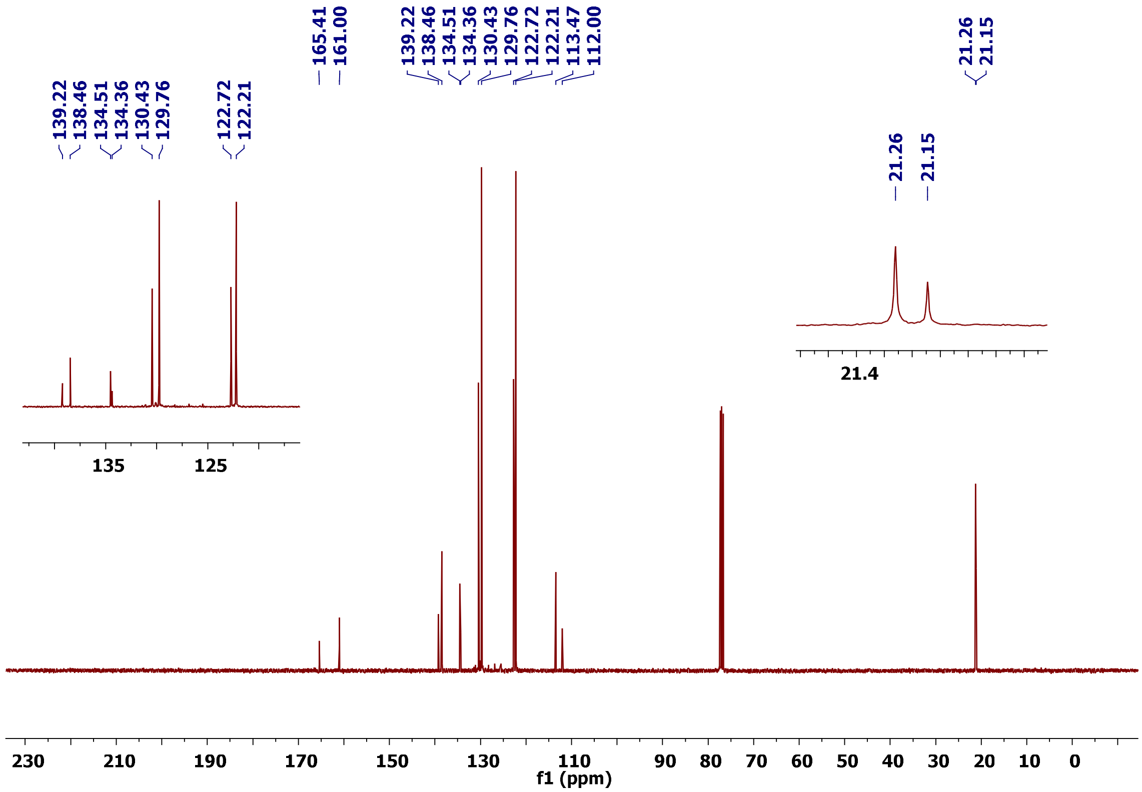

^{13}C-CRAPT NMR ($CDCl_3$) spectrum of *p*-tolylcarbamothioyl cyanide (1:0.41 tautomeric ratio) (1a)

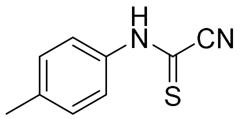

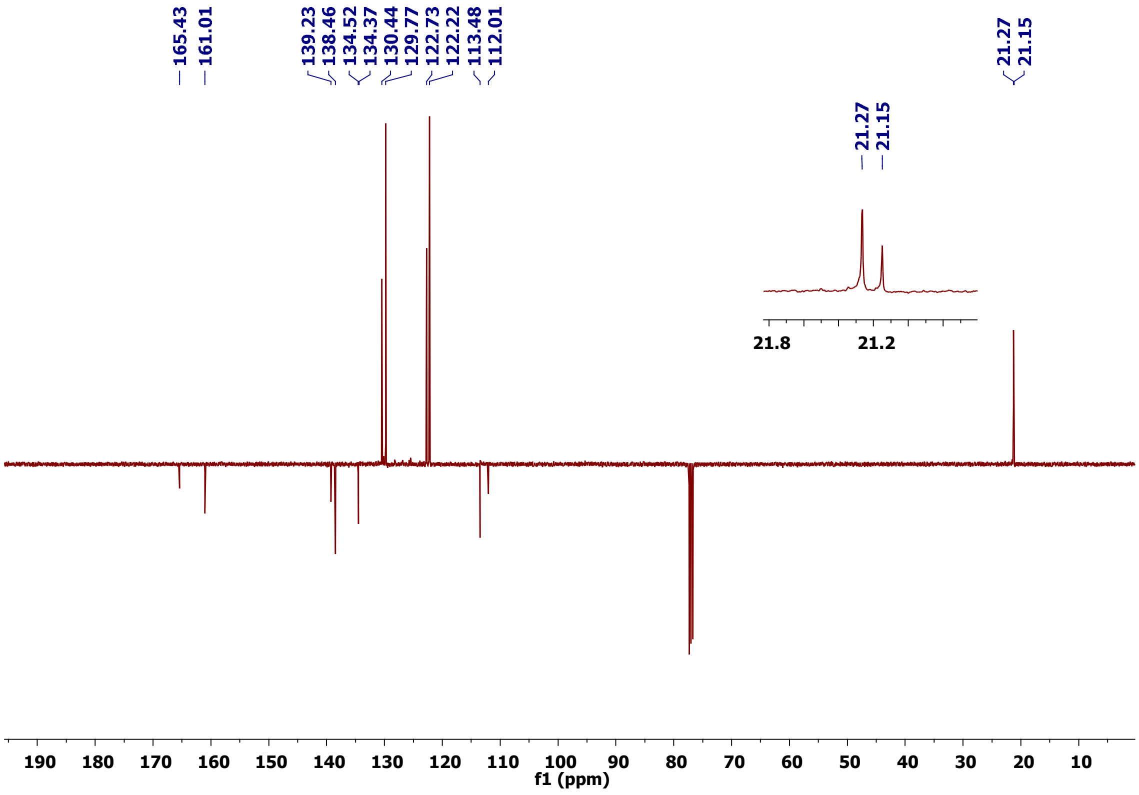

^{13}C DEPT-135 NMR ($CDCl_3$) spectrum of *p*-tolylcarbamothioyl cyanide (1:0.41 tautomeric ratio) (1a)

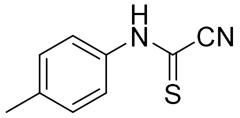

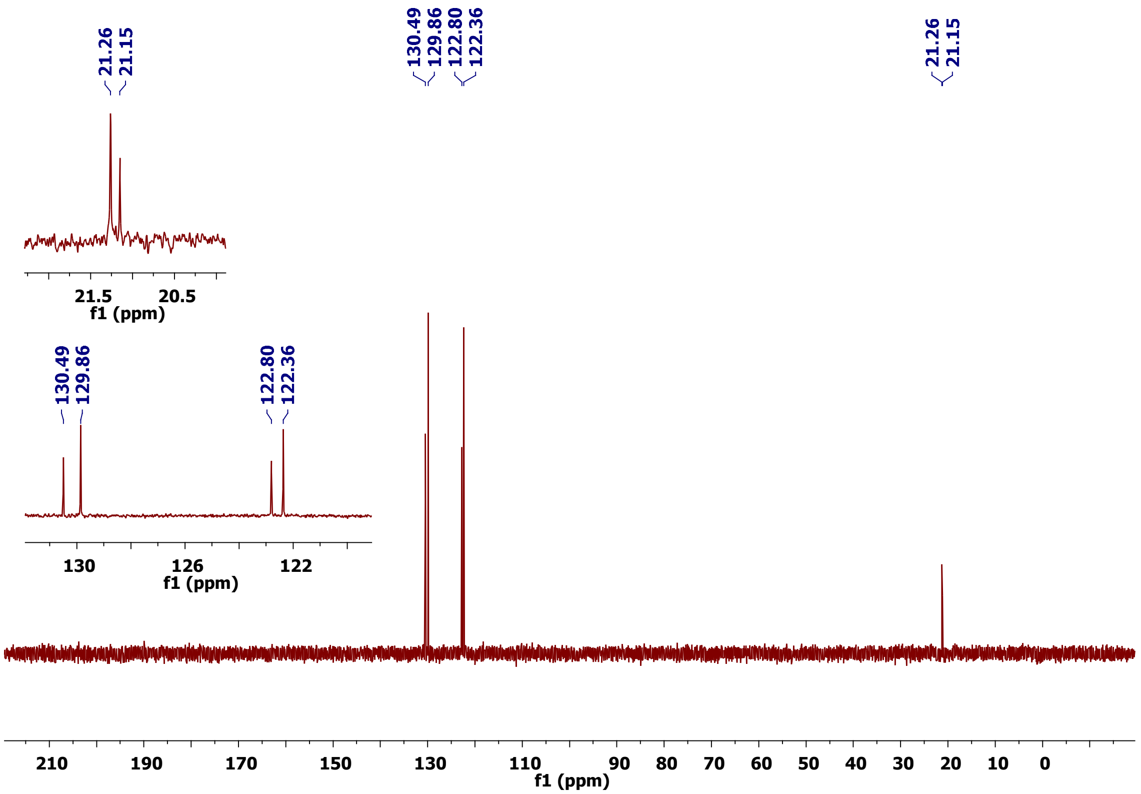

^{1}H-^{1}H -gDQFCOSY NMR ($CDCl_3$) spectrum of *p*-tolylcarbamothioyl cyanide (1:0.41 tautomeric ratio) (1a)

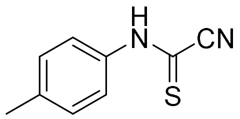

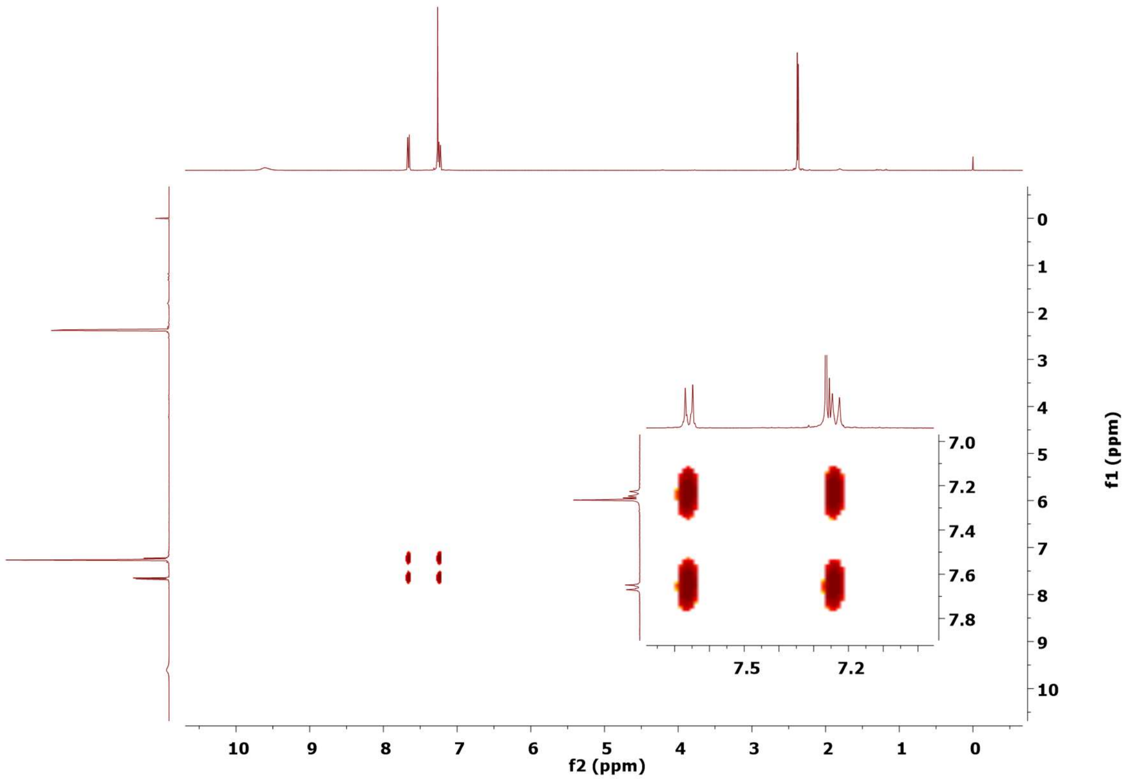

^{1}H-^{13}C-gHSQCAD NMR ($CDCl_3$) spectrum of *p*-tolylcarbamothioyl cyanide (1:0.41 tautomeric ratio) (1a)

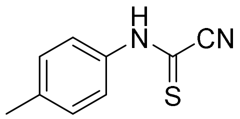

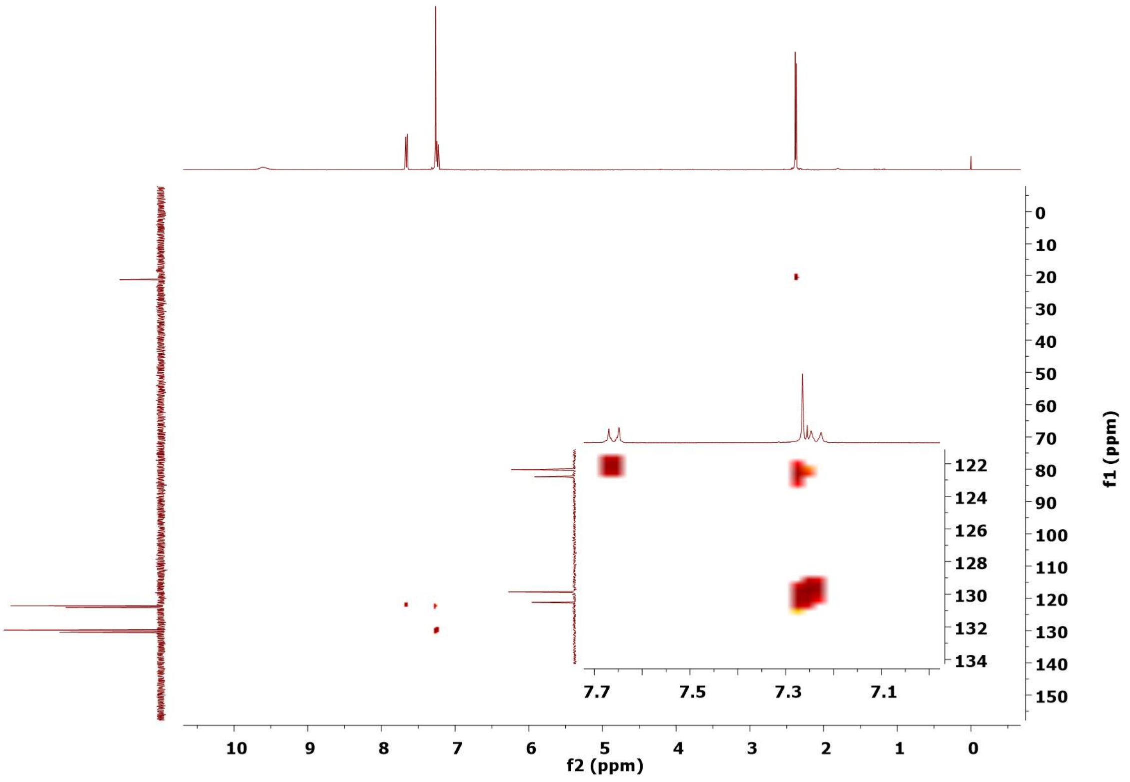

^{1}H NMR ($CDCl_3$) spectrum of phenylcarbamothioyl cyanide (1:0.59 tautomeric ratio) (1b)

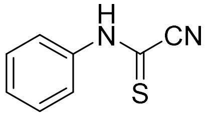

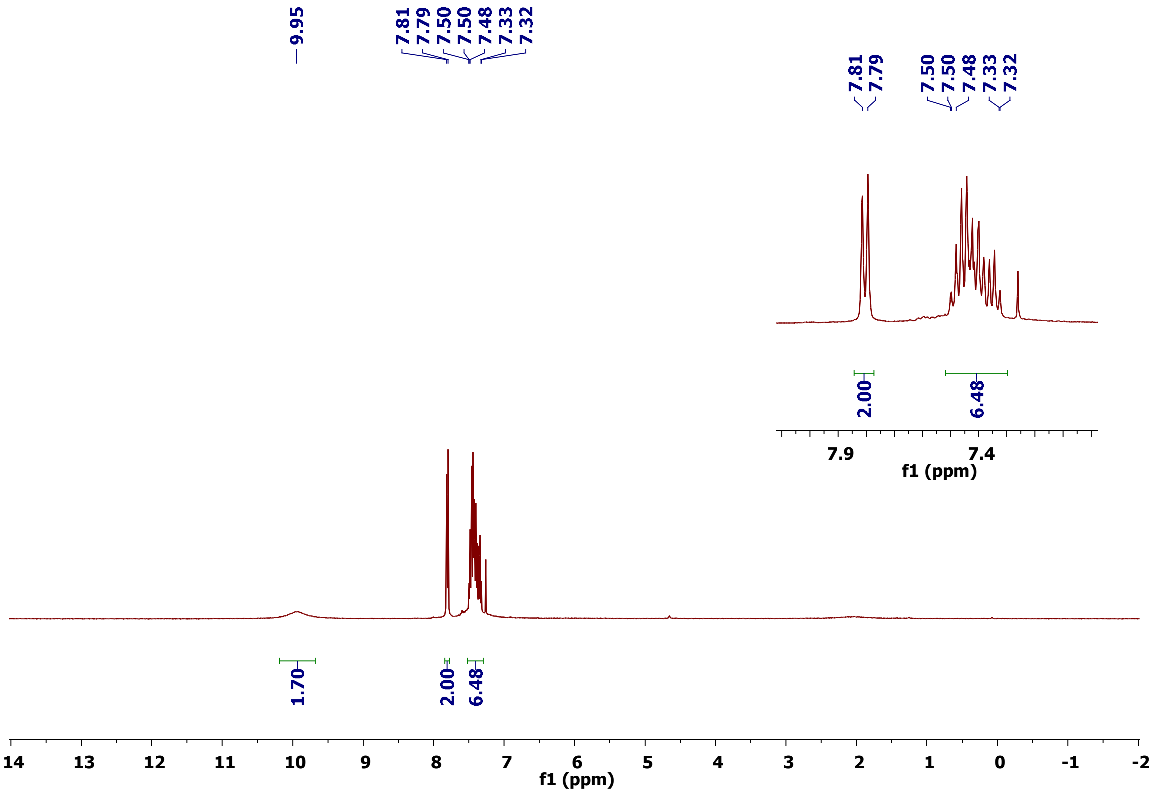

^{13}C-DEPT 90 NMR ($CDCl_3$) spectrum of phenylcarbamothioyl cyanide (1:0.59 tautomeric ratio) (1b)

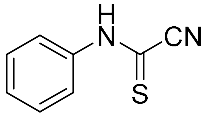

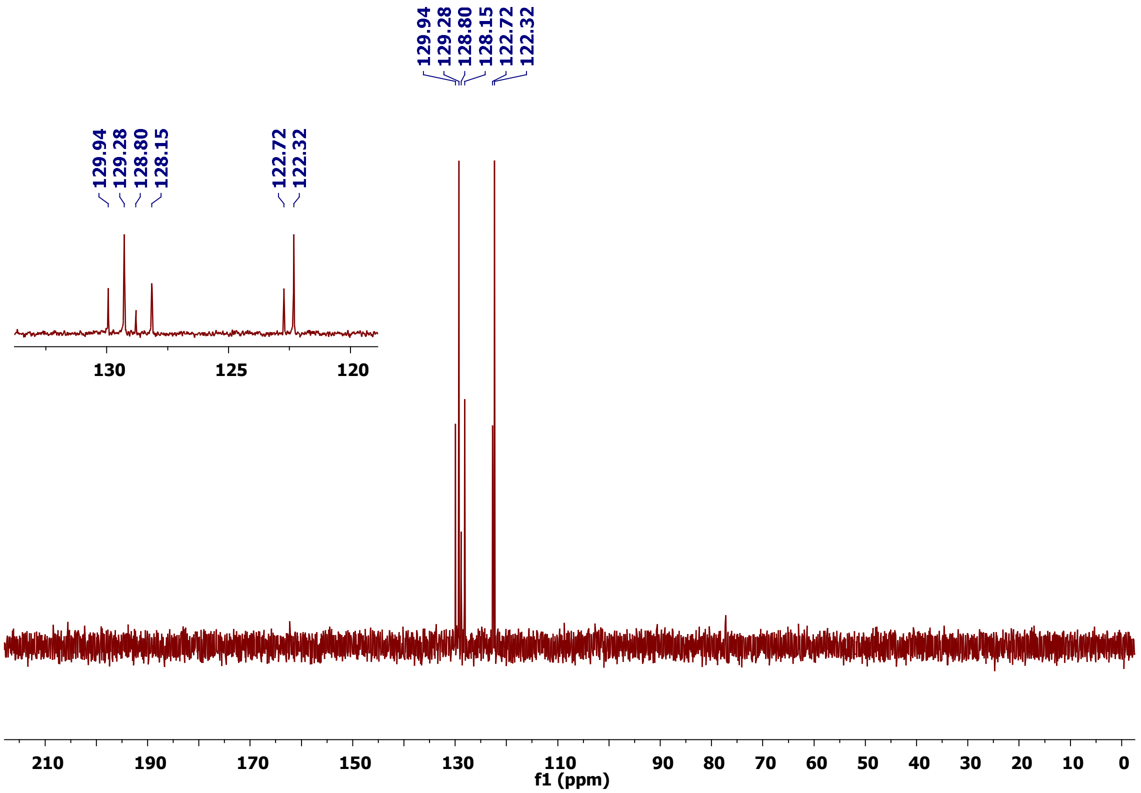

^{13}C CRAPT NMR ($CDCl_3$) spectrum of phenylcarbamothioyl cyanide (1:0.59 tautomeric ratio) (1b)

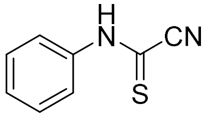

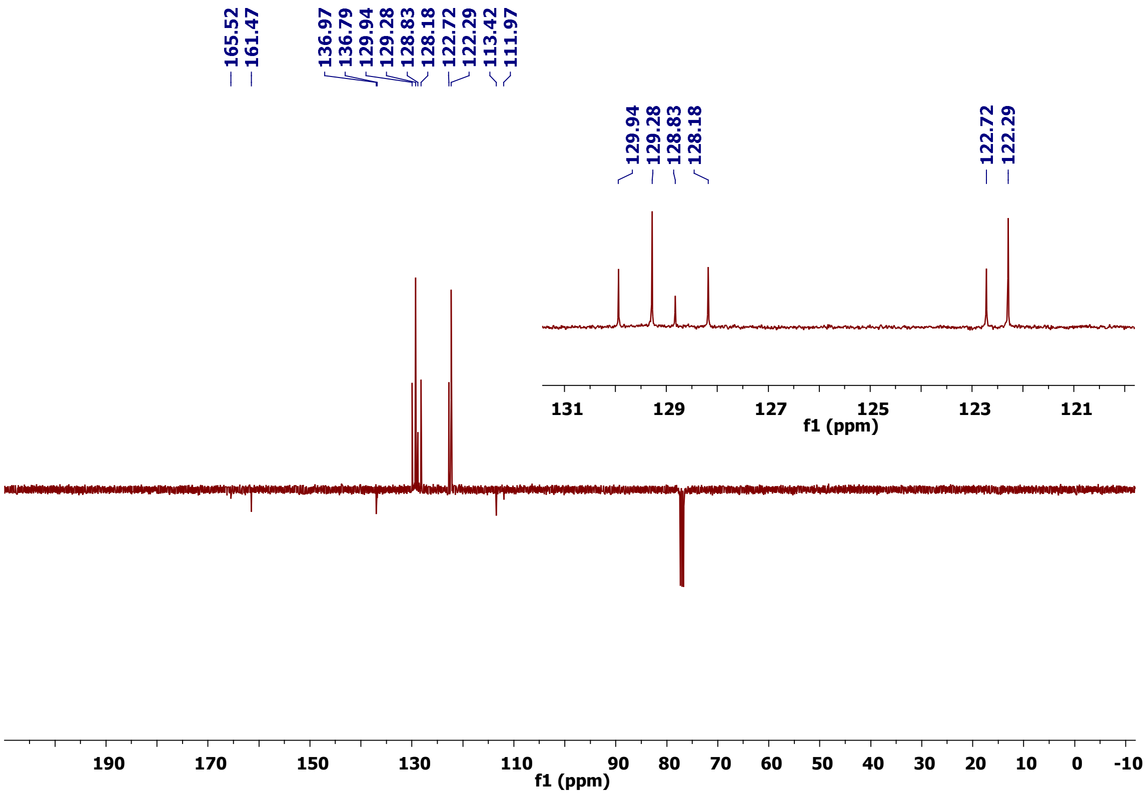

^{13}C NMR ($CDCl_3$) spectrum of phenylcarbamothioyl cyanide (1:0.59 tautomeric ratio) (1b)

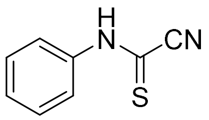

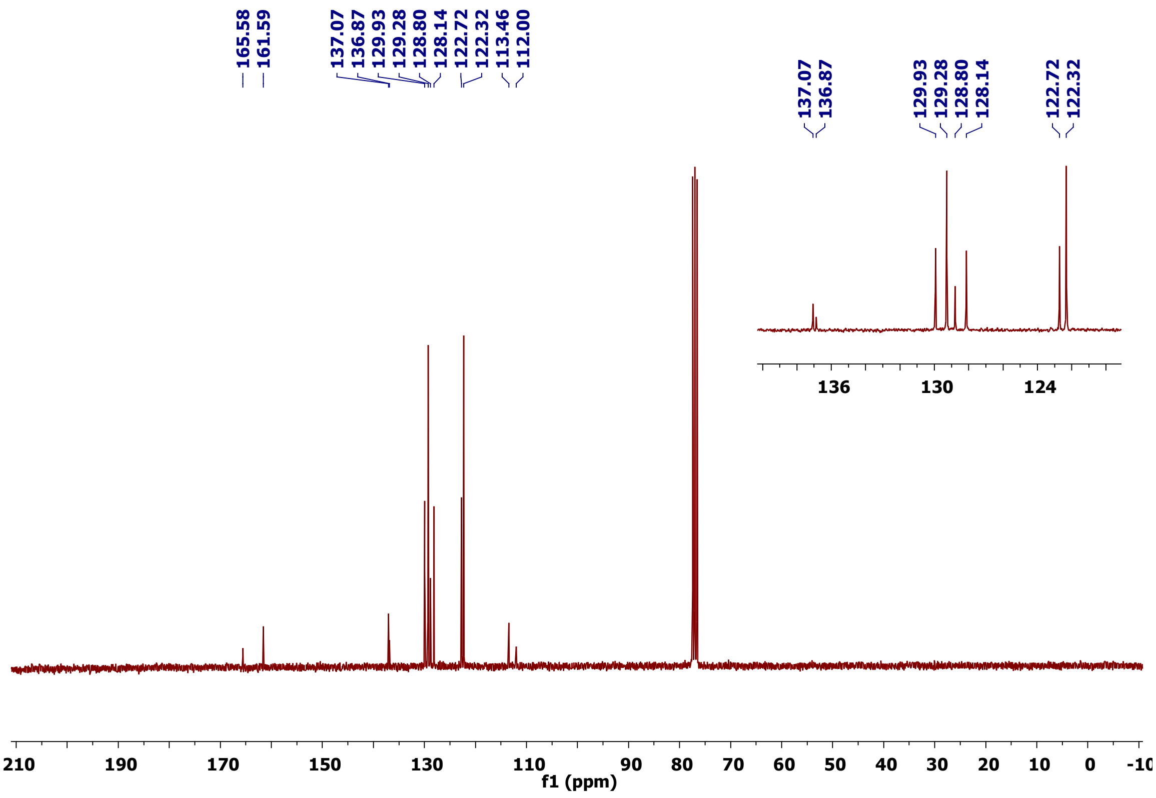

^{1}H-^{13}C-gHSQC NMR ($CDCl_3$) spectrum of phenylcarbamothioyl cyanide (1:0.59 tautomeric ratio) (1b)

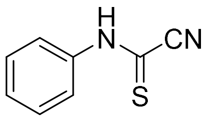

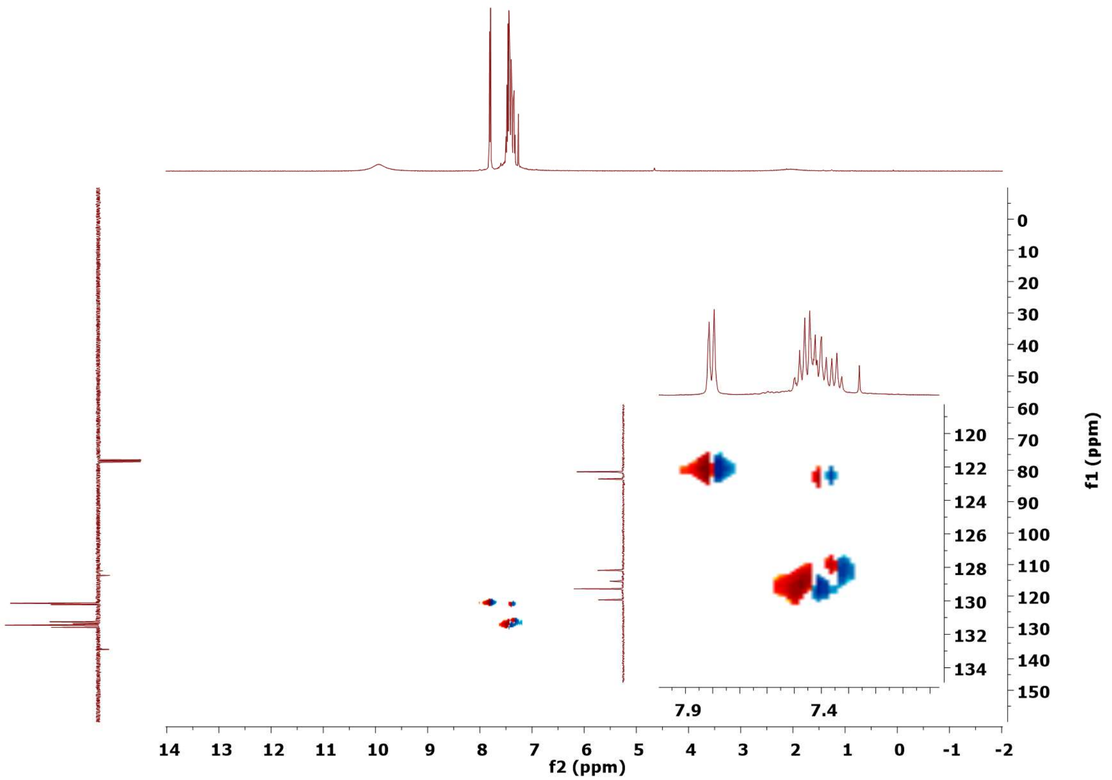

^{1}H NMR ($CDCl_3$) spectrum of (4-chlorophenyl)carbamothioyl cyanide (1:0.47 tautomeric ratio) (1c)

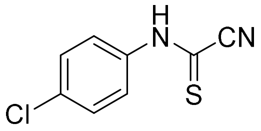

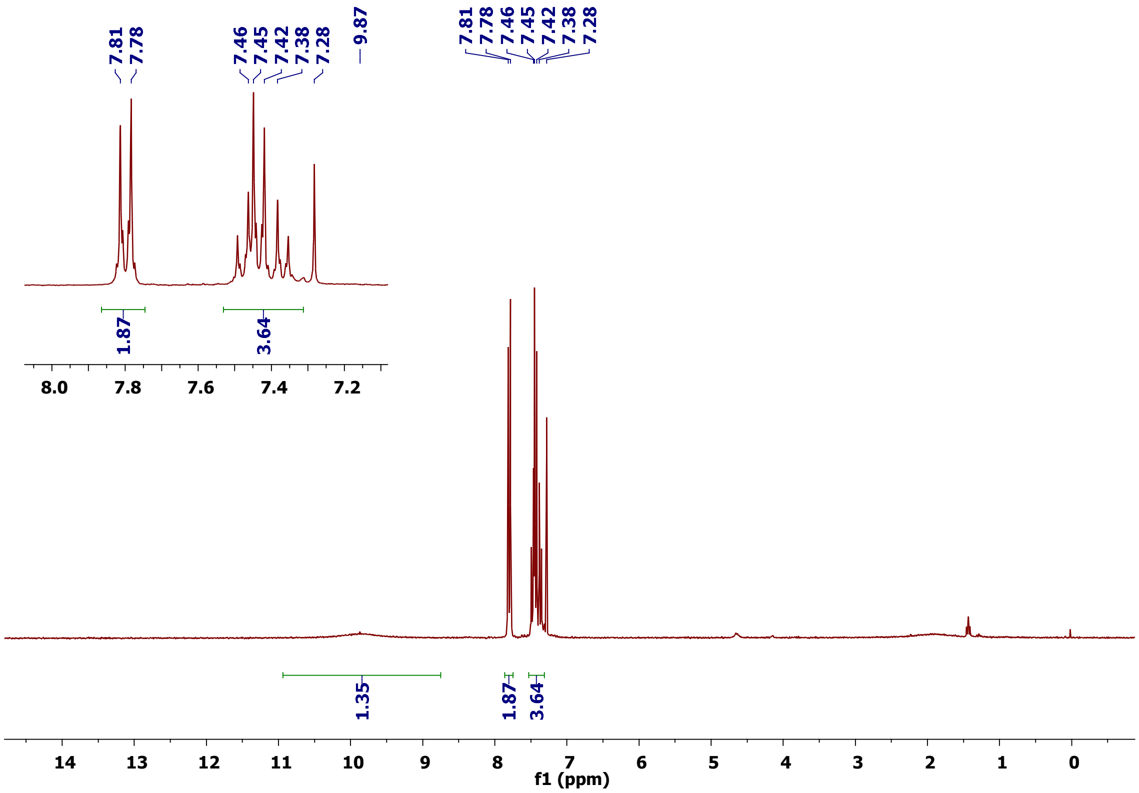

^{13}C DEPT-135 NMR ($CDCl_3$) spectrum of (4-chlorophenyl)carbamothioyl cyanide (1:0.47 tautomeric ratio) (1c)

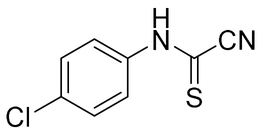

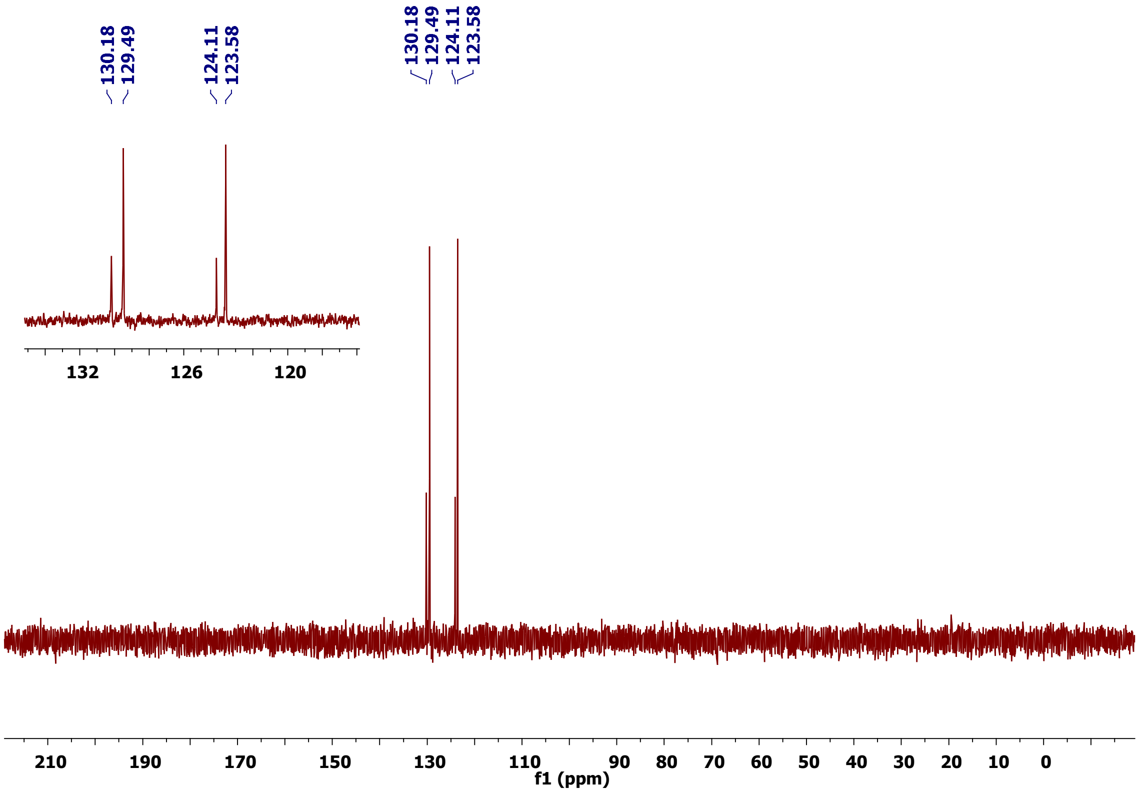

^{13}C NMR ($CDCl_3$) spectrum of (4-chlorophenyl)carbamothioyl cyanide (1:0.47 tautomeric ratio) (1c)

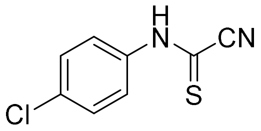

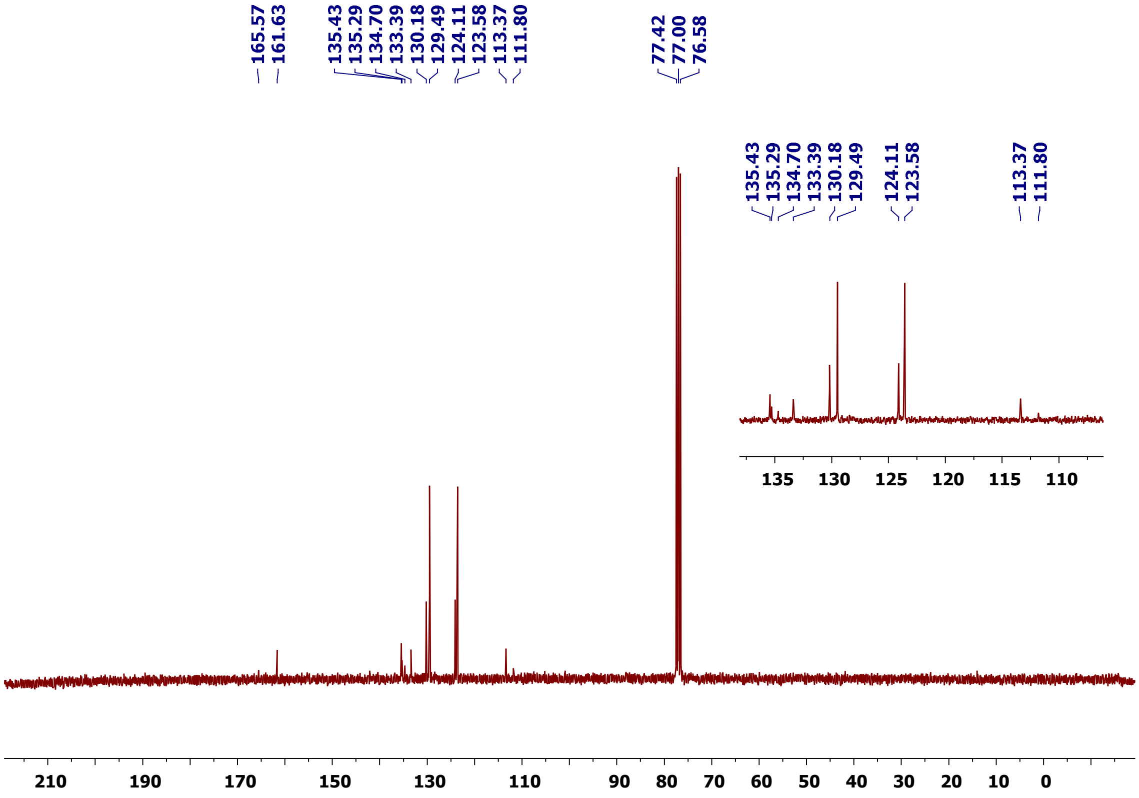

^{1}H-^{1}H-gDQFCOSY NMR ($CDCl_3$) spectrum of (4-chlorophenyl)carbamothioyl cyanide (1:0.47 tautomeric ratio) (1c)

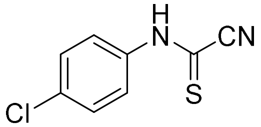

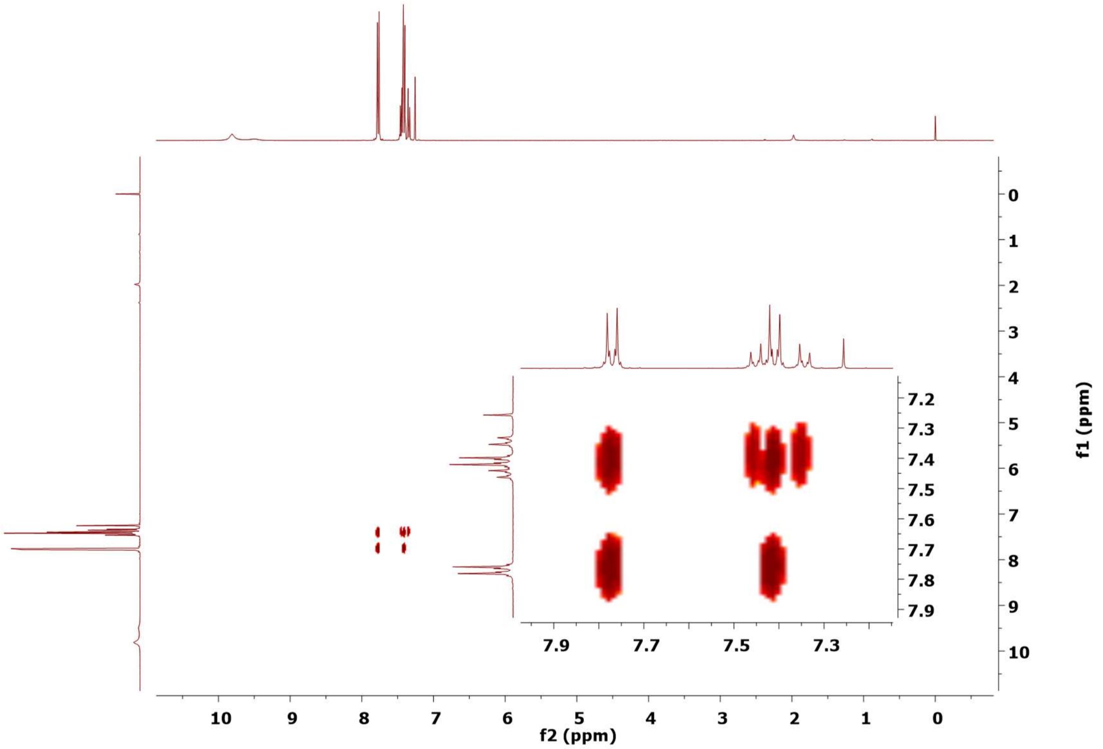

^{1}H-^{13}C-gHSQC NMR ($CDCl_3$) spectrum of (4-chlorophenyl)carbamothioyl cyanide (1:0.47 tautomeric ratio) (1c)

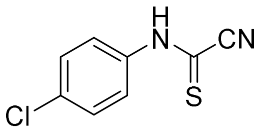

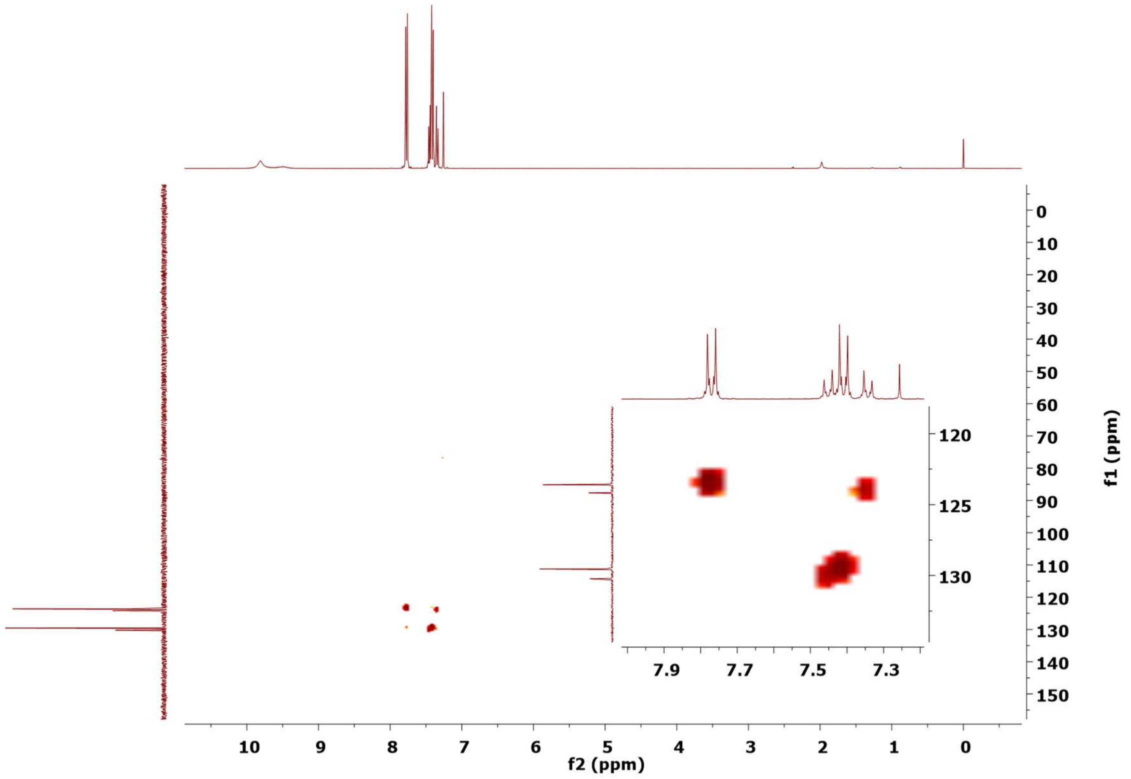

^{1}H NMR ($CDCl_3$) spectrum of (2-fluorophenyl)carbamothioyl cyanide (1:0.53 tautomeric ratio) (1d)

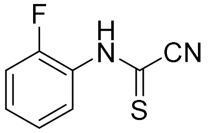

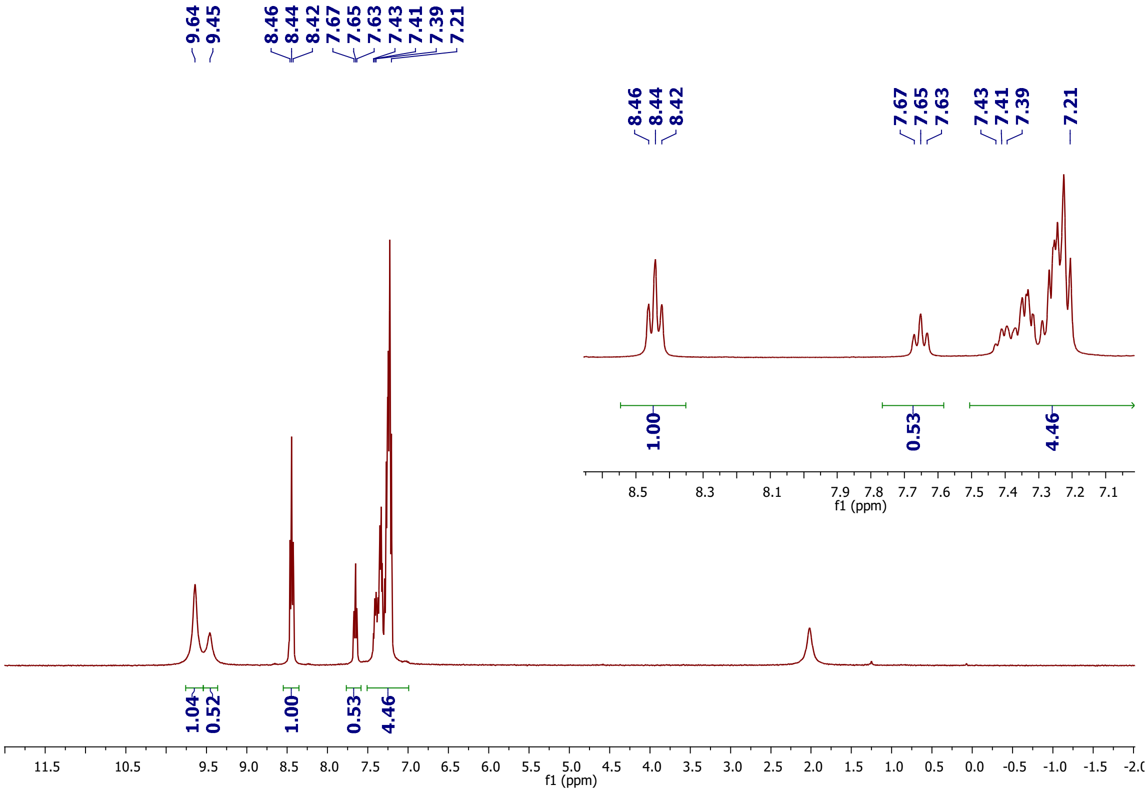

^{13}C NMR ($CDCl_3$) spectrum of (2-fluorophenyl)carbamothioyl cyanide (1:0.53 tautomeric ratio) (1d)

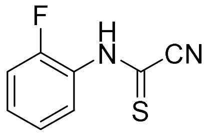

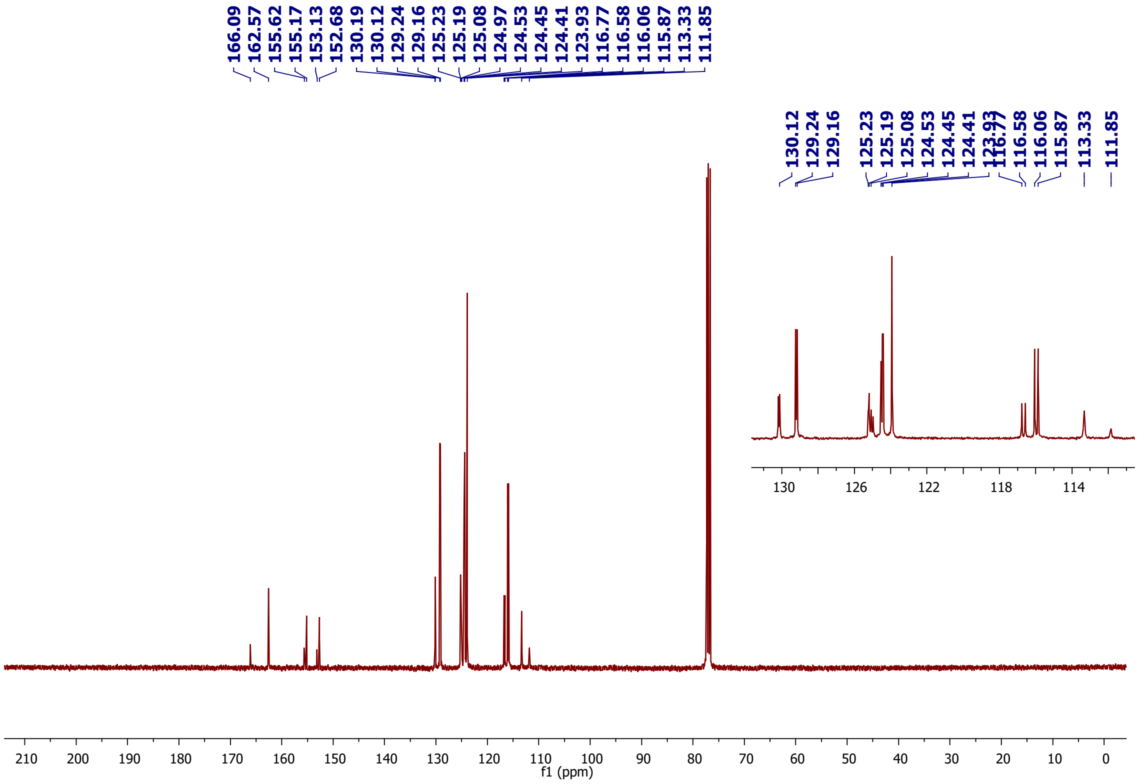

^{13}C CRAPT NMR ($CDCl_3$) spectrum of (2-fluorophenyl)carbamothioyl cyanide (1:0.53 tautomeric ratio) (1d)

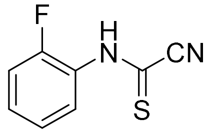

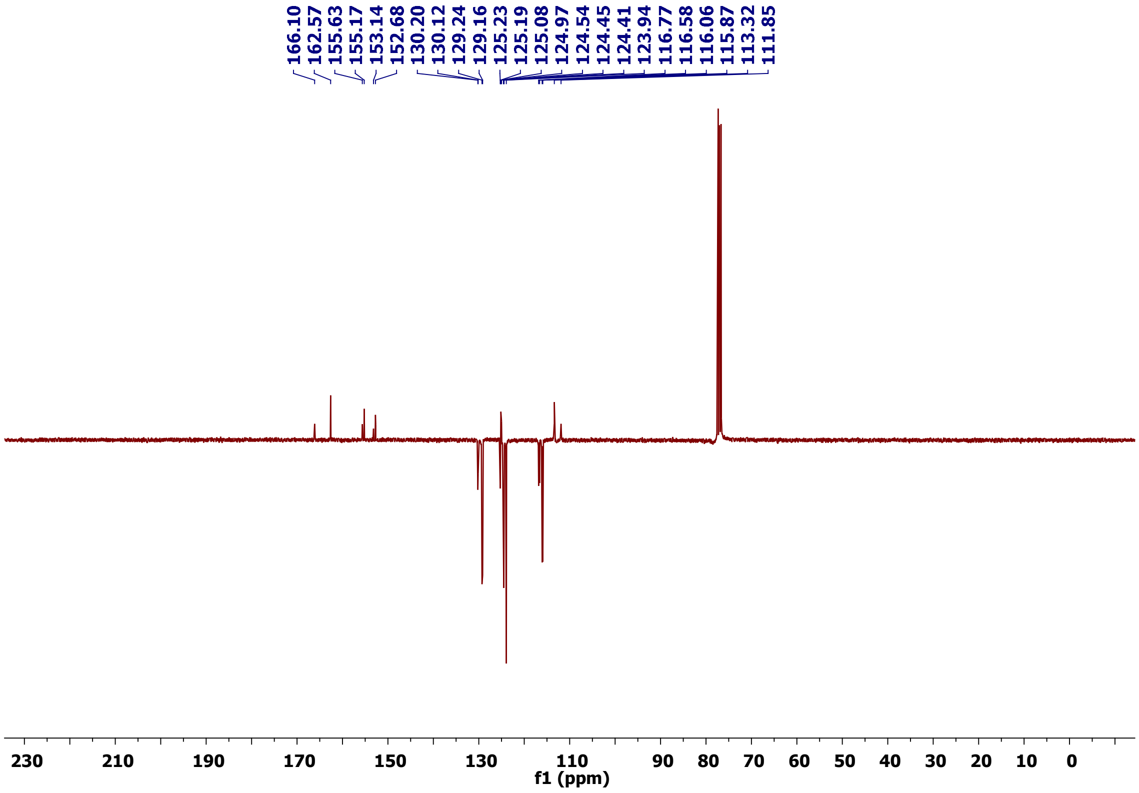

^{1}H-^{13}C gHMBCAD NMR ($CDCl_3$) spectrum of (2-fluorophenyl)carbamothioyl cyanide (1:0.53 tautomeric ratio) (1d)

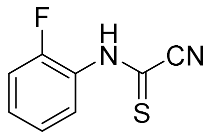

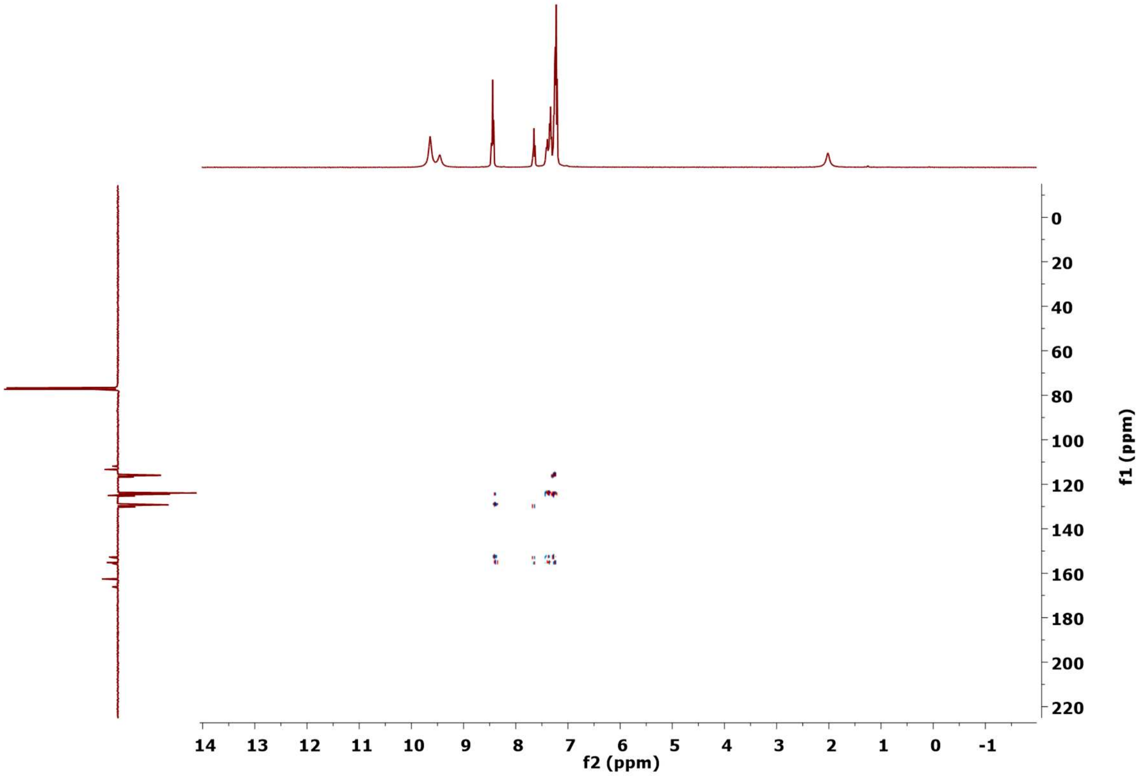

^{1}H NMR (DMSO-d6) spectrum of ((4-fluorophenyl)carbamothioyl cyanide (1:0.2 tautomeric ratio) (1e)

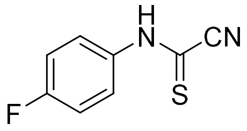

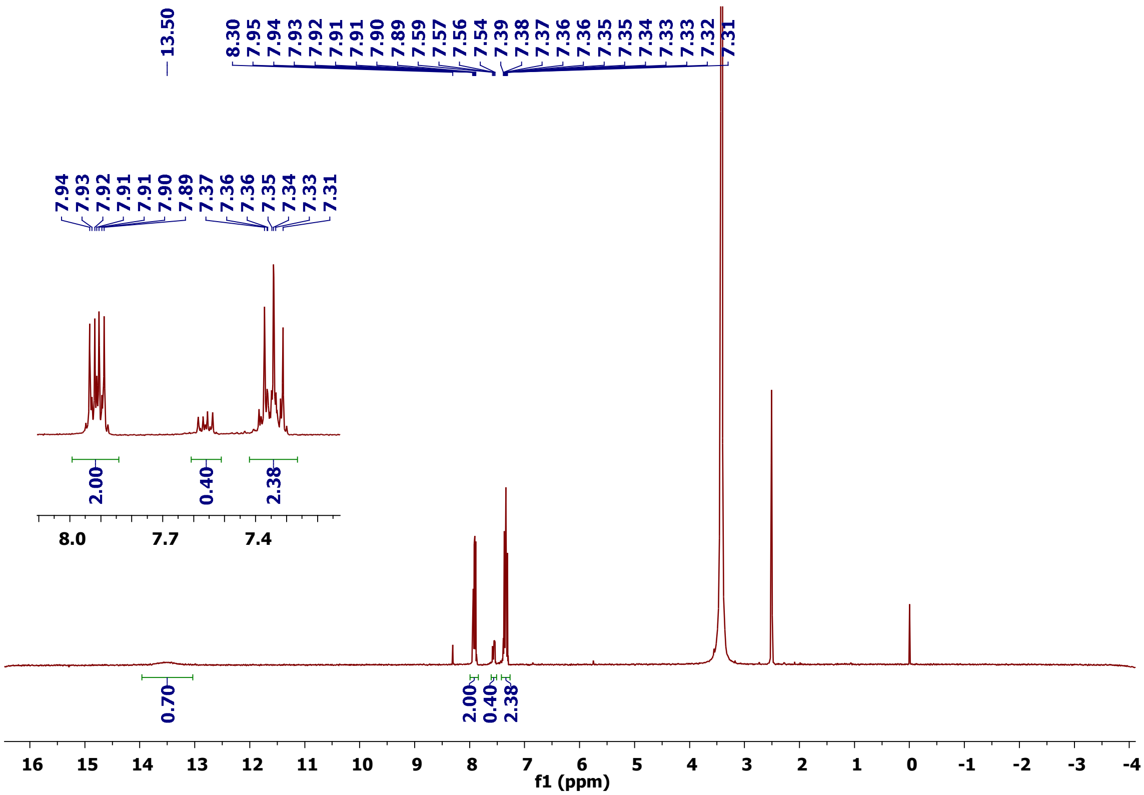

^{13}C-DEPT 90 spectrum of ((4-fluorophenyl)carbamothioyl cyanide (1:0.2 tautomeric ratio) (1e)

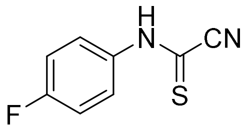

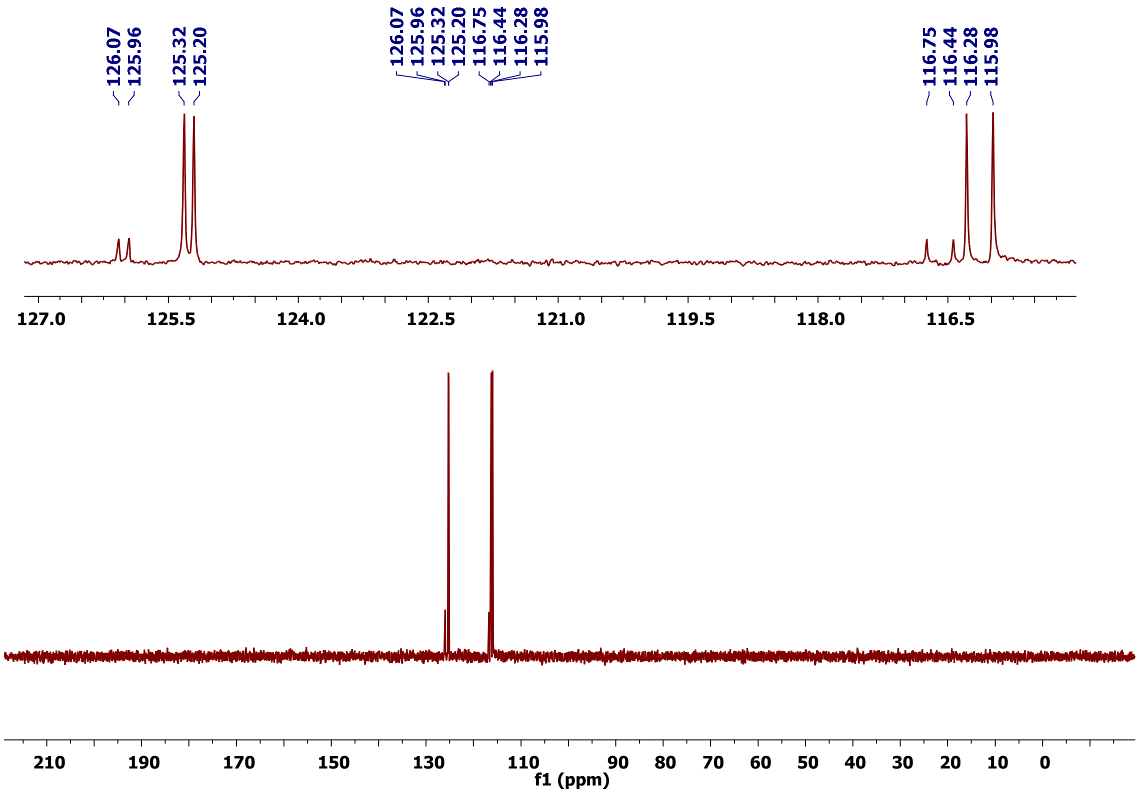

^{13}C NMR (DMSO-d6) spectrum of ((4-fluorophenyl)carbamothioyl cyanide (1:0.2 tautomeric ratio) (1e)

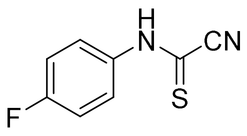

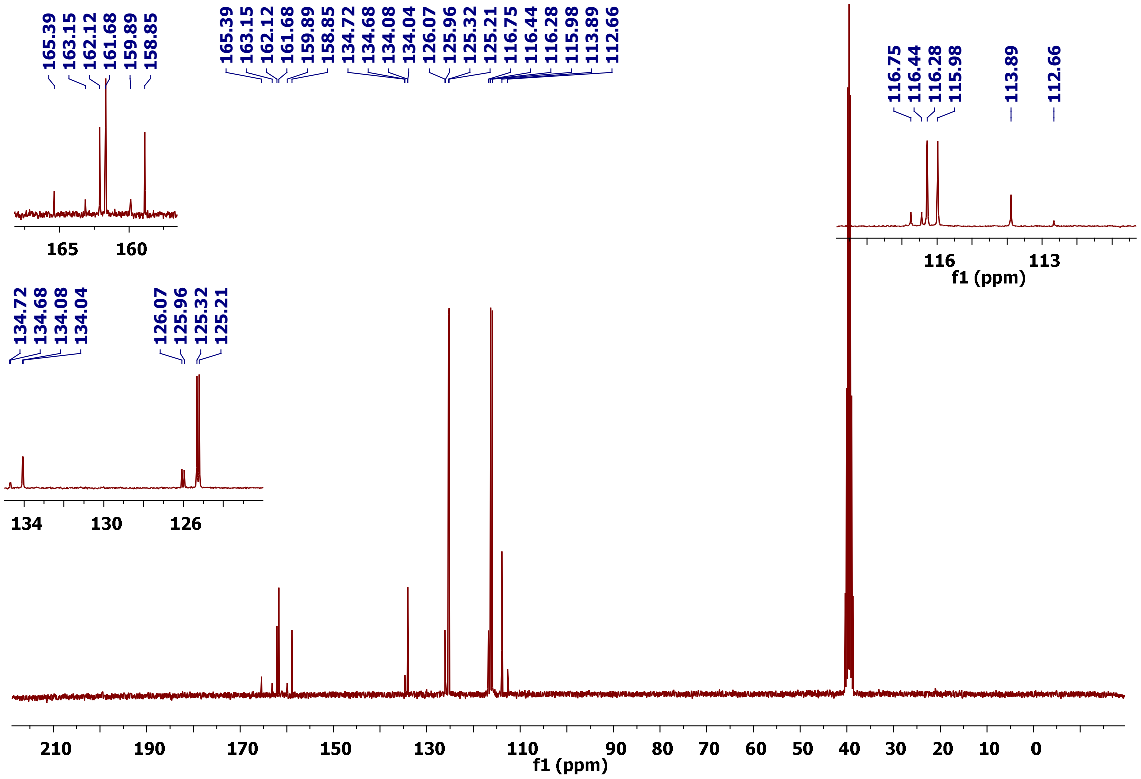

^{1}H NMR ($CDCl_3$) spectrum of (3-fluorophenyl)carbamothioyl cyanide (1:0.39 tautomeric ratio) (1f)

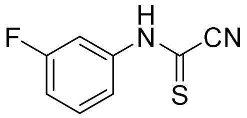

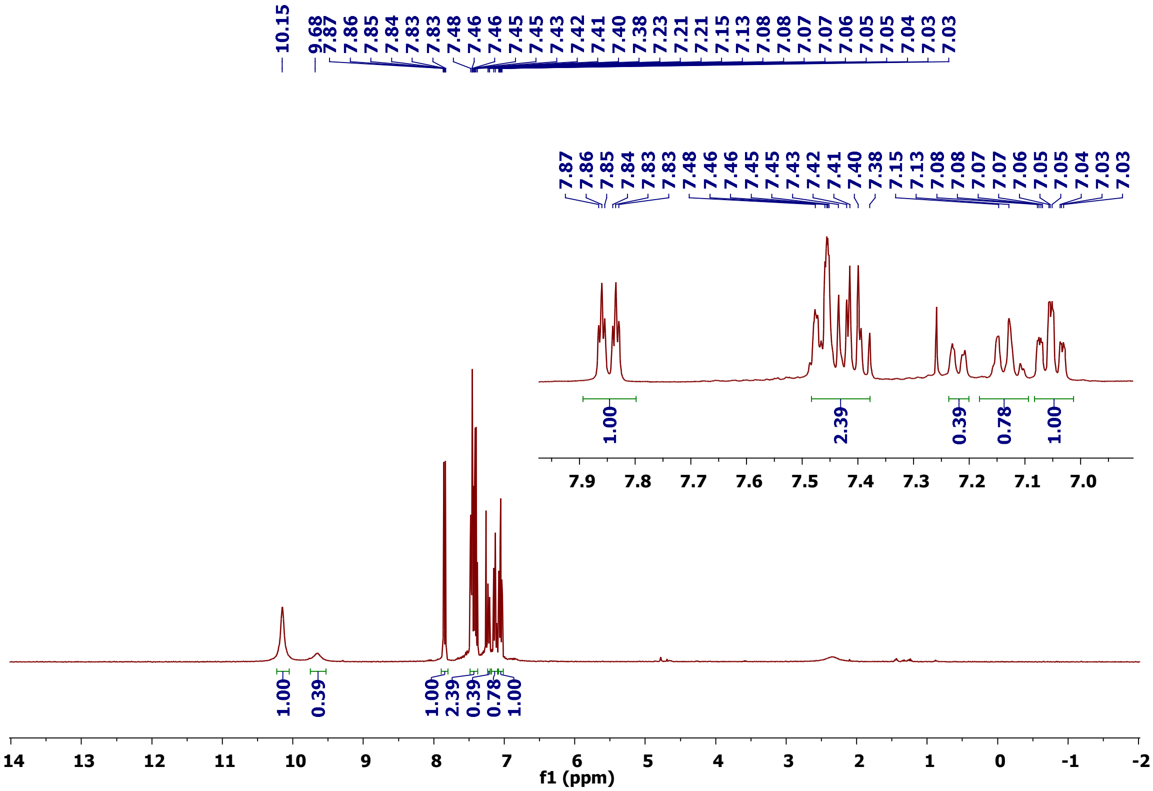

^{13}C NMR ($CDCl_3$) spectrum of (3-fluorophenyl)carbamothioyl cyanide (1:0.39 tautomeric ratio) (1f)

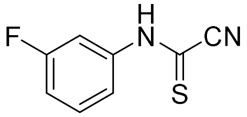

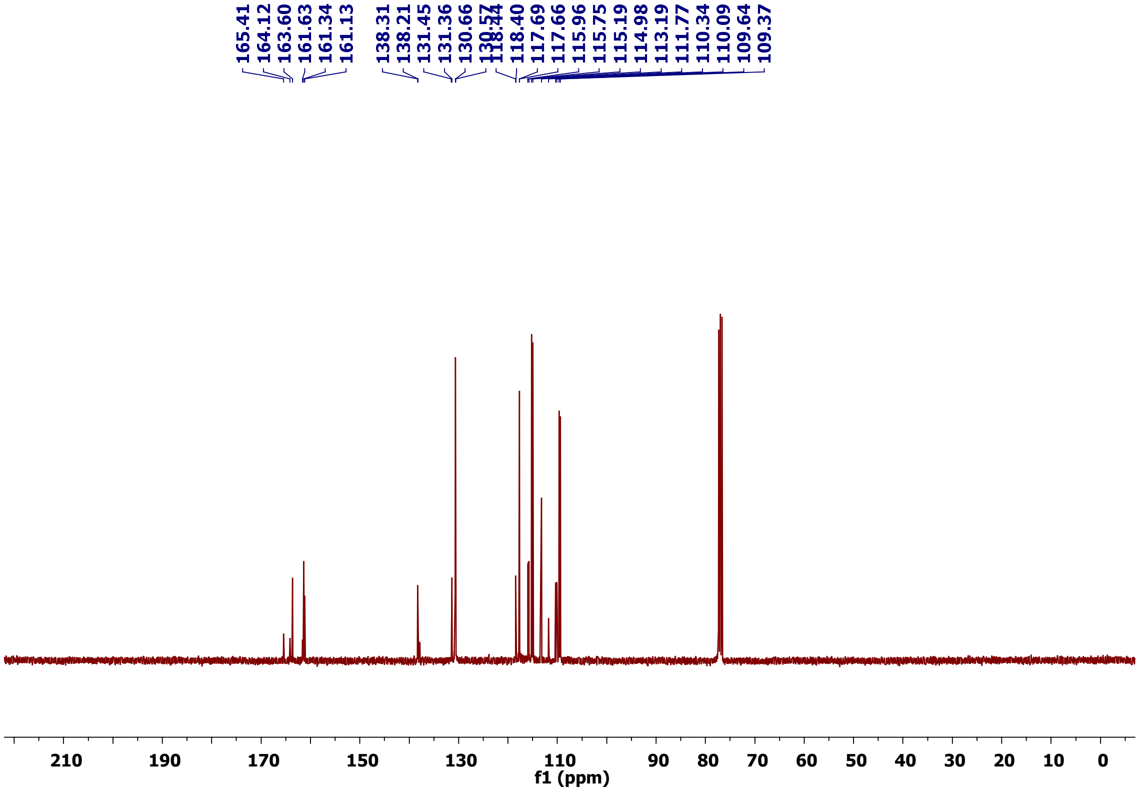

^{13}C CRAPT NMR ($CDCl_3$) spectrum (3-fluorophenyl)carbamothioyl cyanide (1:0.39 tautomeric ratio) (1f)

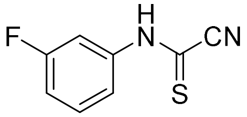

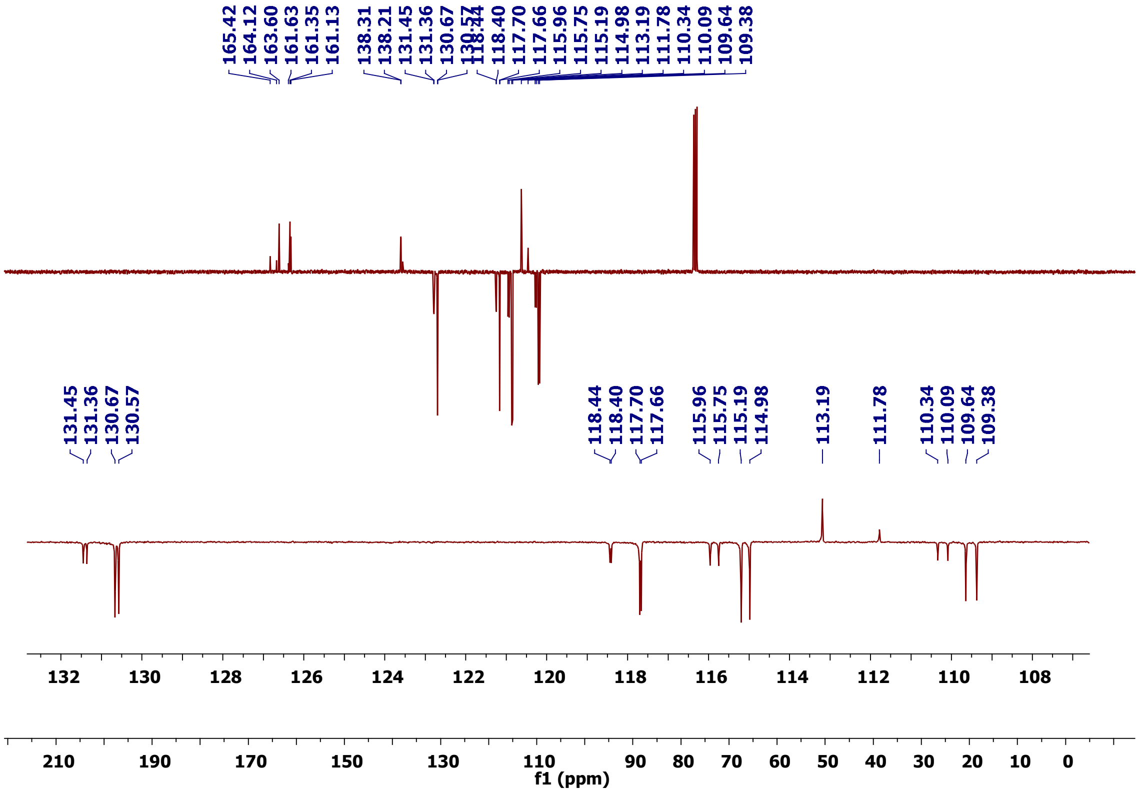

^{1}H-^{1}H gDQCOSY NMR ($CDCl_3$) spectrum (3-fluorophenyl)carbamothioyl cyanide (1:0.39 tautomeric ratio) (1f)

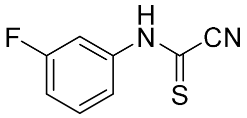

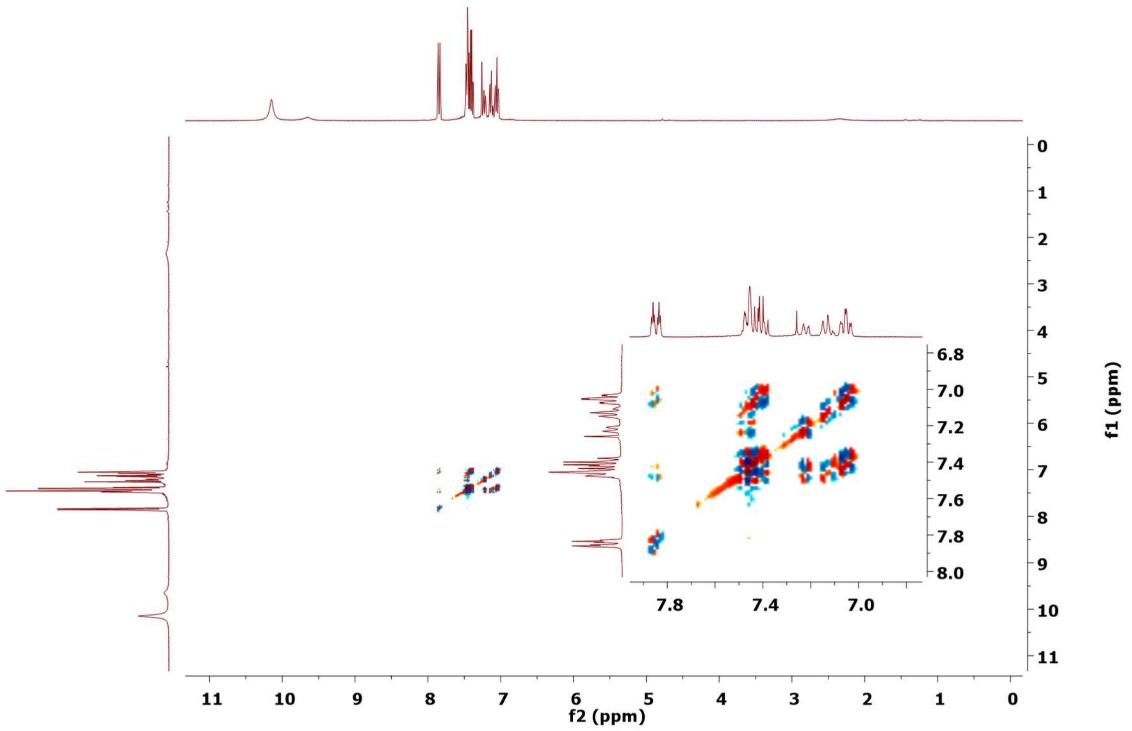

^{1}H-^{13}C gHSQC NMR ($CDCl_3$) spectrum of (3-fluorophenyl)carbamothioyl cyanide (1:0.39 tautomeric ratio) (1f)

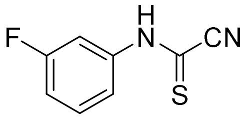

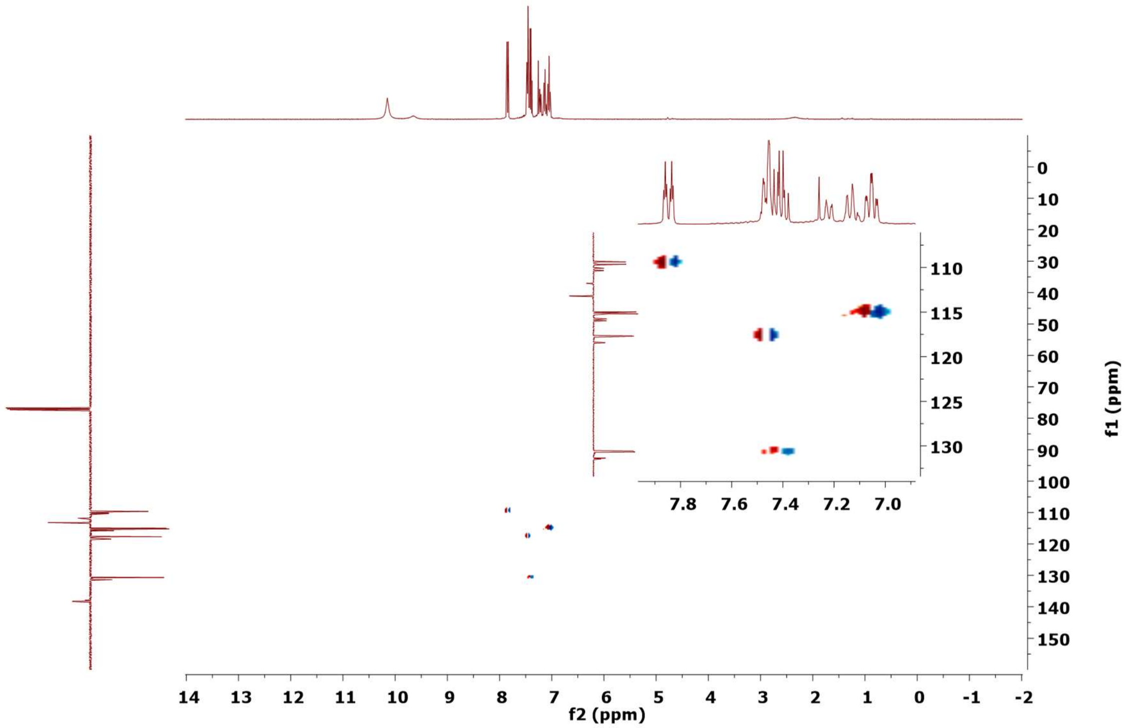

^{1}H-^{13}C gHMBCAD NMR ($CDCl_3$) spectrum of (3-fluorophenyl)carbamothioyl cyanide (1:0.39 tautomeric ratio) (1f)

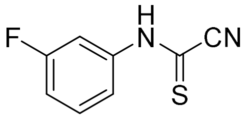

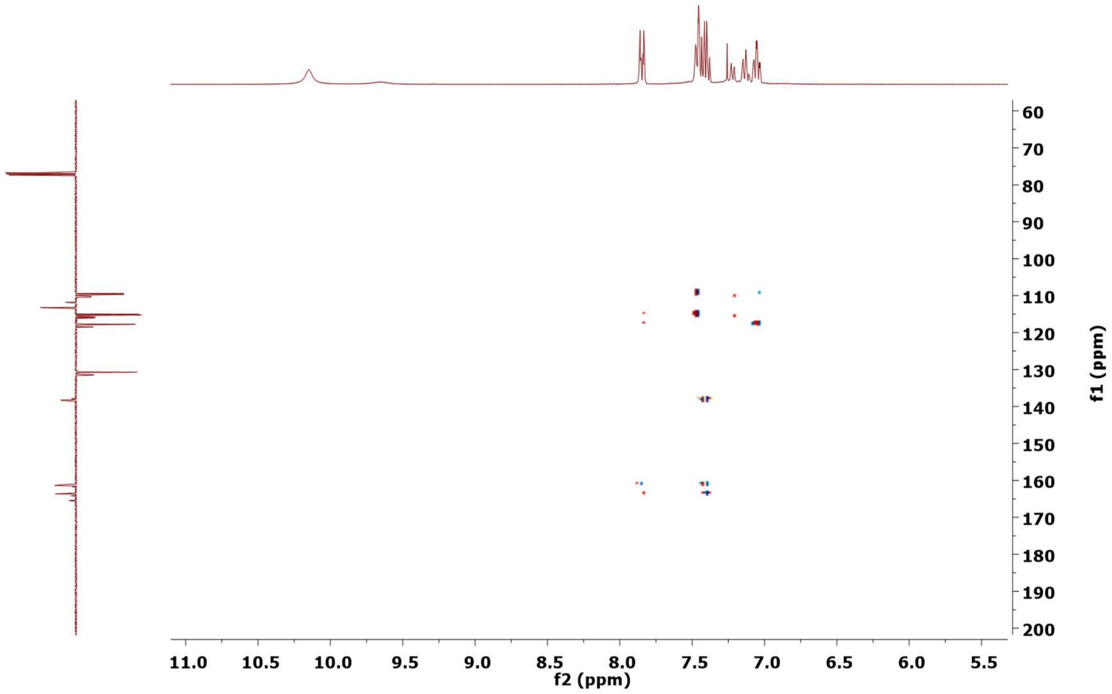

^{1}H NMR (CD_3OD) spectrum of (4-nitrophenyl)carbamothioyl cyanide (1g)

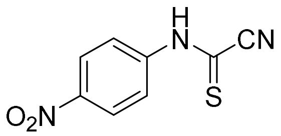

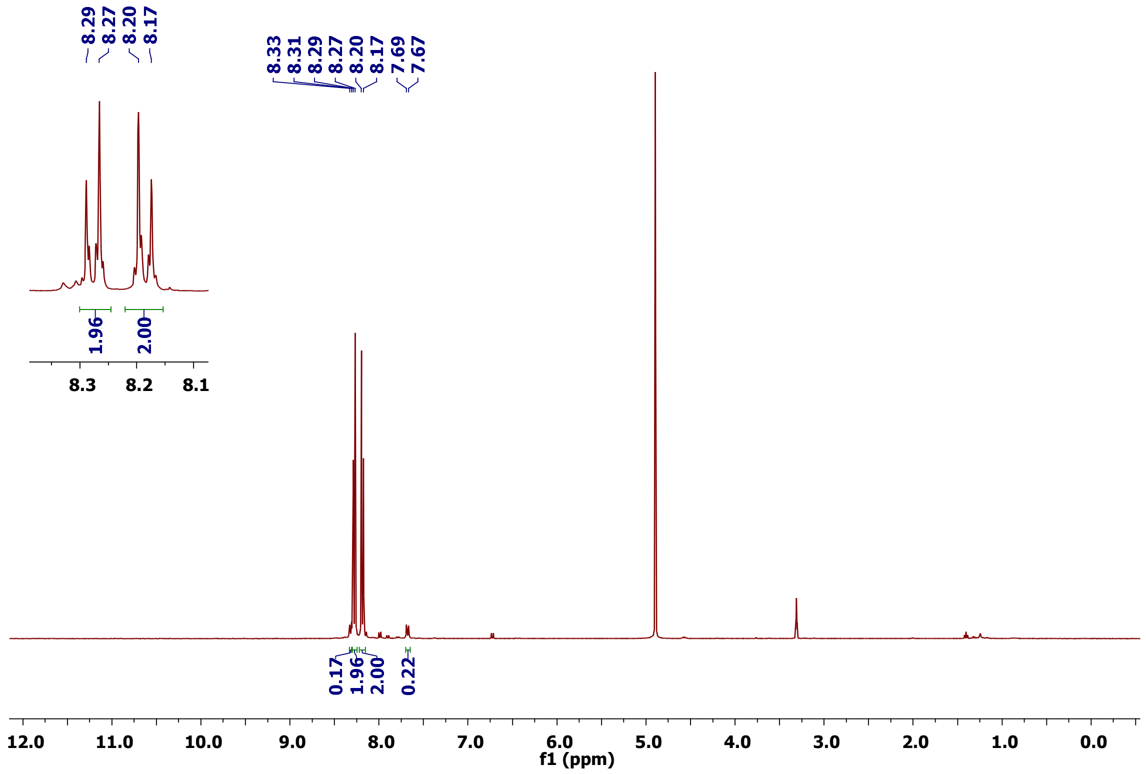

^{13}C CRAPT NMR (CD_3OD) spectrum of (4-nitrophenyl)carbamothioyl cyanide (1g)

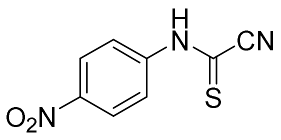

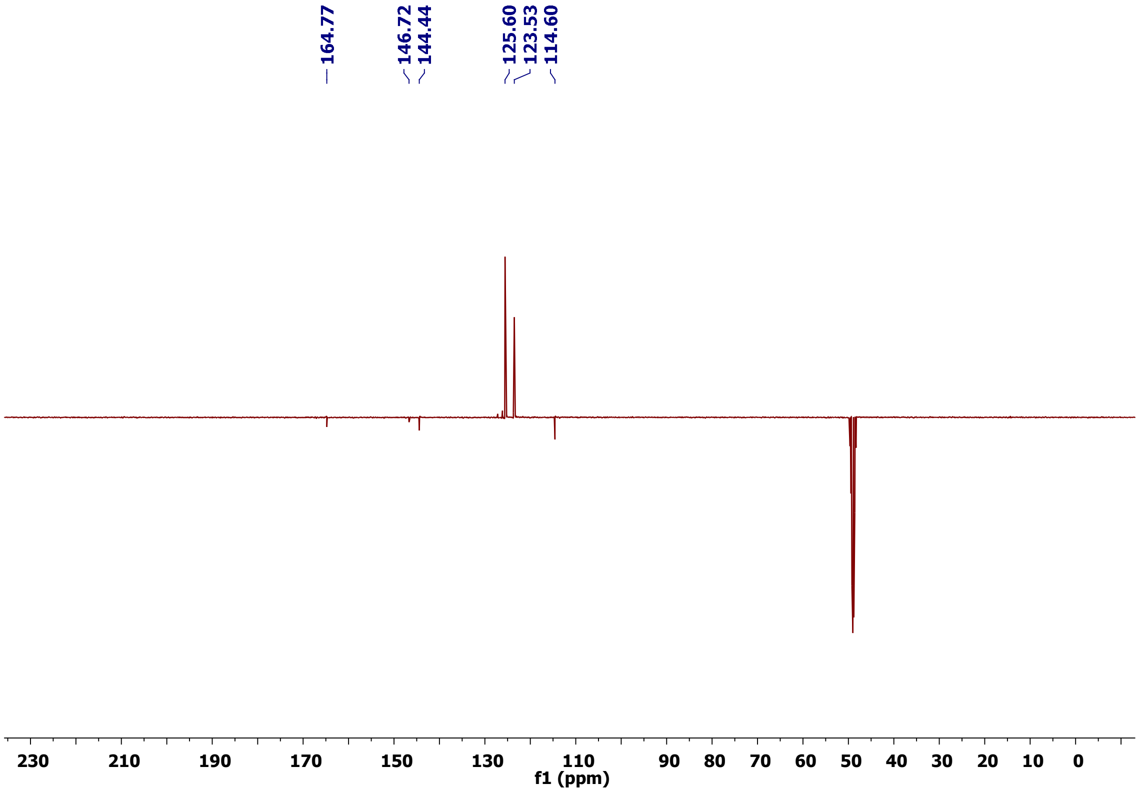

^{13}C NMR (CD_3OD) spectrum of (4-nitrophenyl)carbamothioyl cyanide (1g)

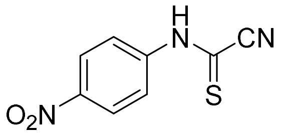

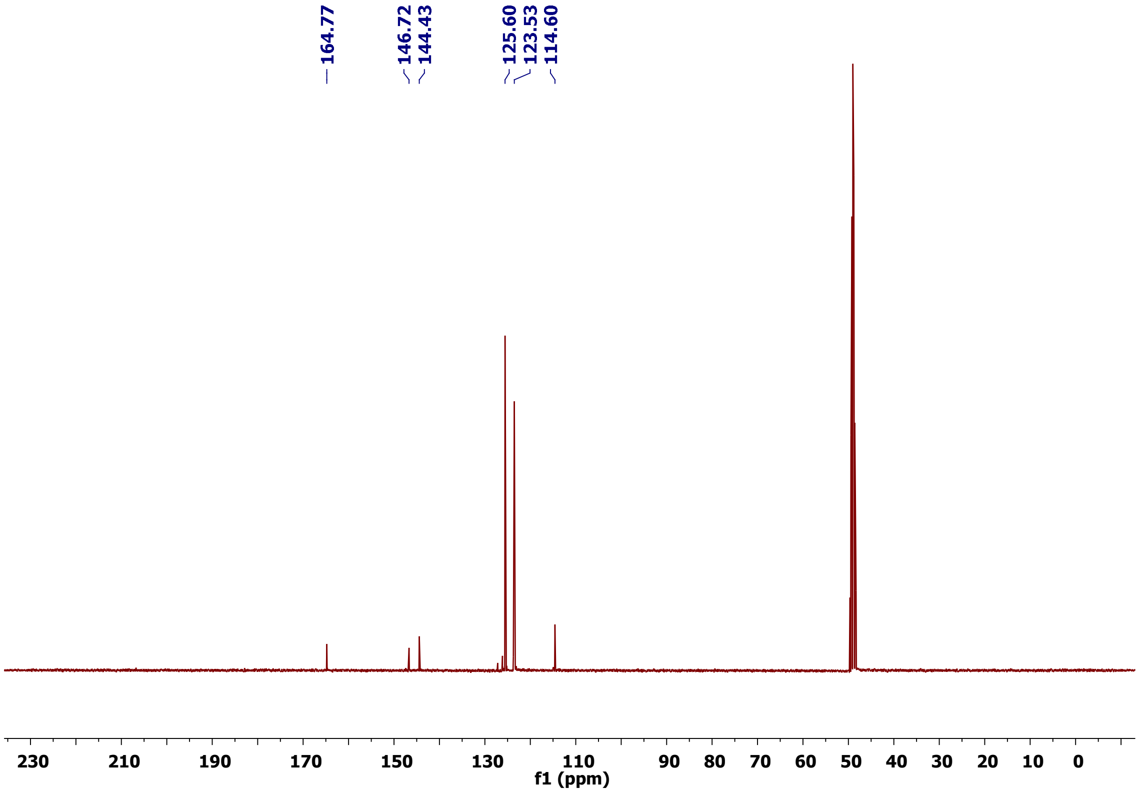

^{1}H NMR (DMSO-d6) spectrum of (3-nitrophenyl)carbamothioyl cyanide (1:0.22 tautomeric ratio) (1h)

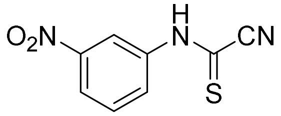

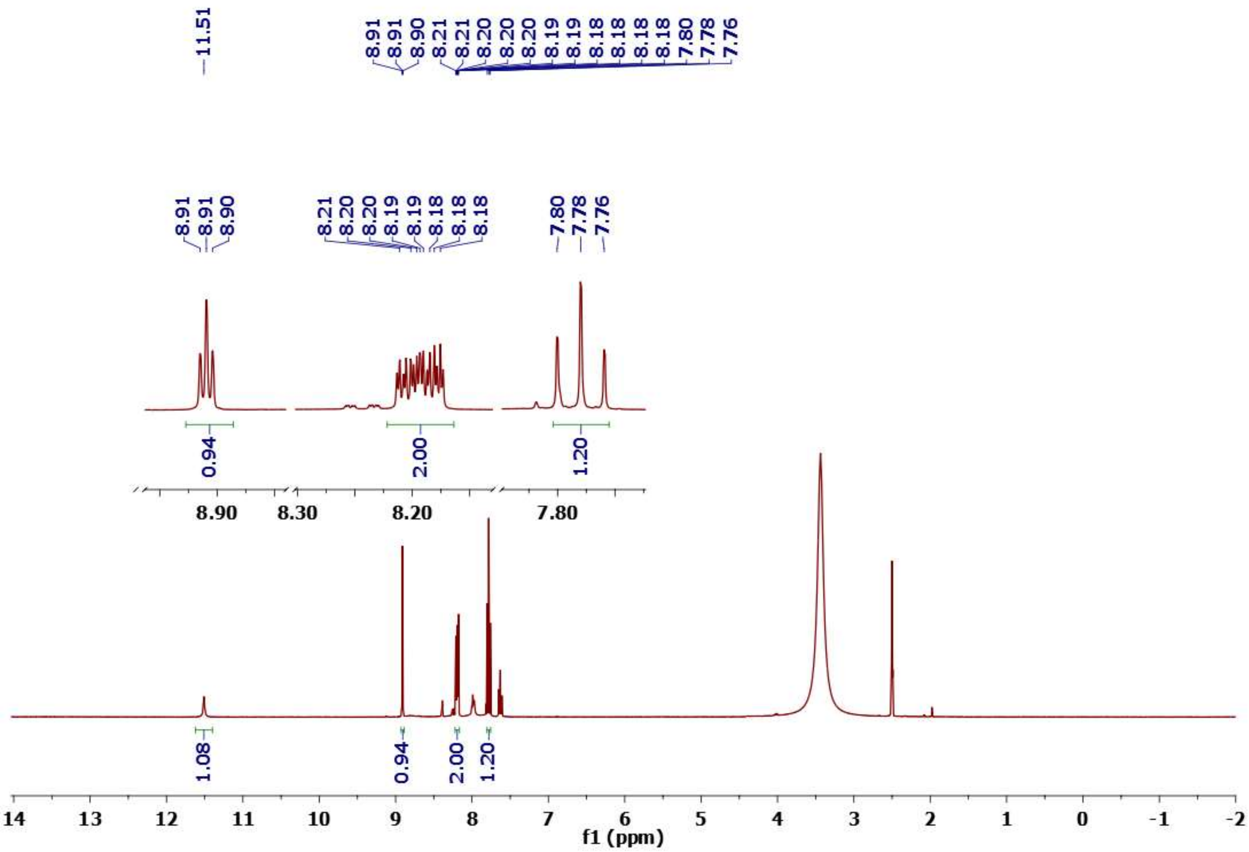

^{13}C NMR (DMSO-d6) spectrum of (3-nitrophenyl)carbamothioyl cyanide (1:0.22 tautomeric ratio) (1h)

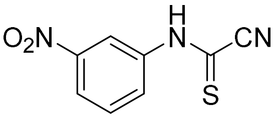

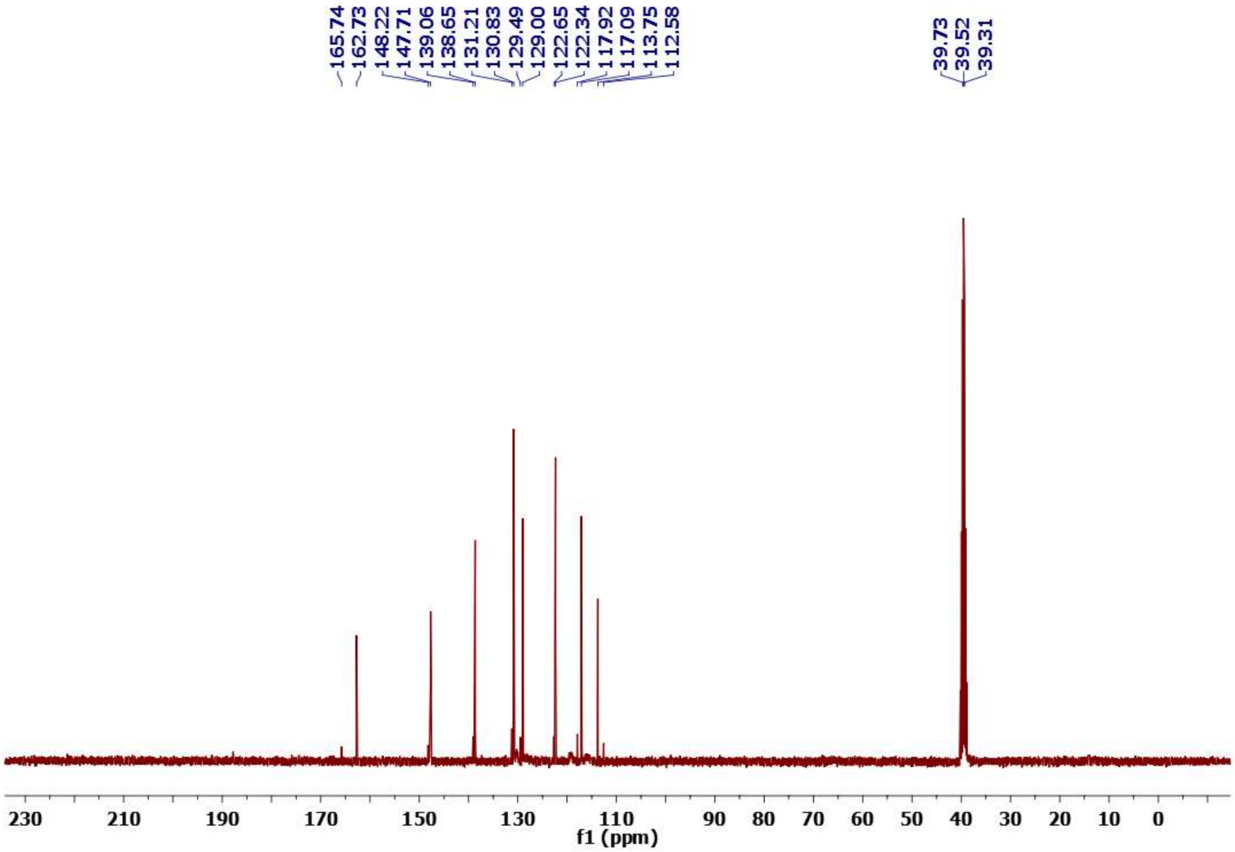

^{13}C CRAPT NMR (DMSO-d6) spectrum of (3-nitrophenyl)carbamothioyl cyanide (1:0.22 tautomeric ratio) (1h)

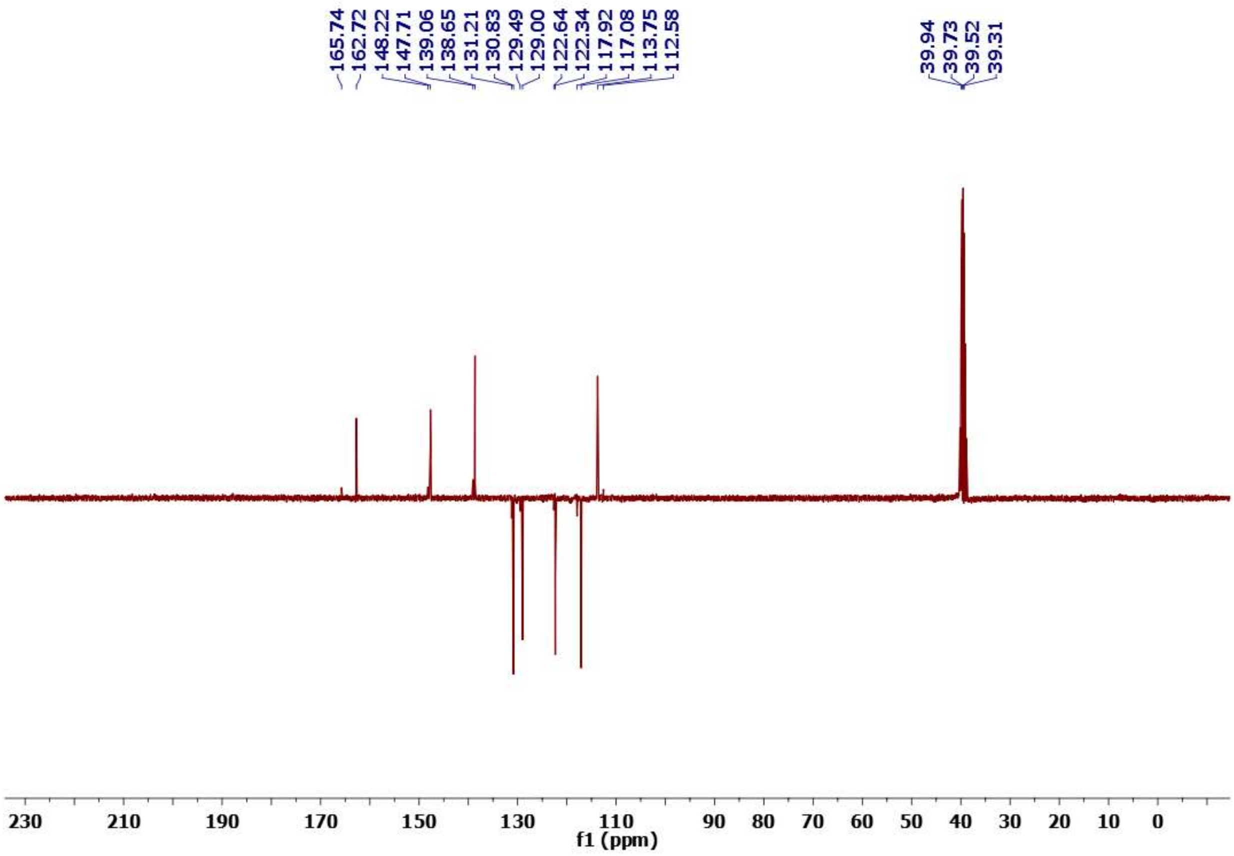

^{1}H NMR (DMSO-d6) spectrum of (4-methoxyphenyl)carbamothioyl cyanide (1:0.21 tautomeric ratio) (1i)

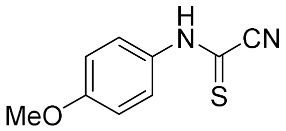

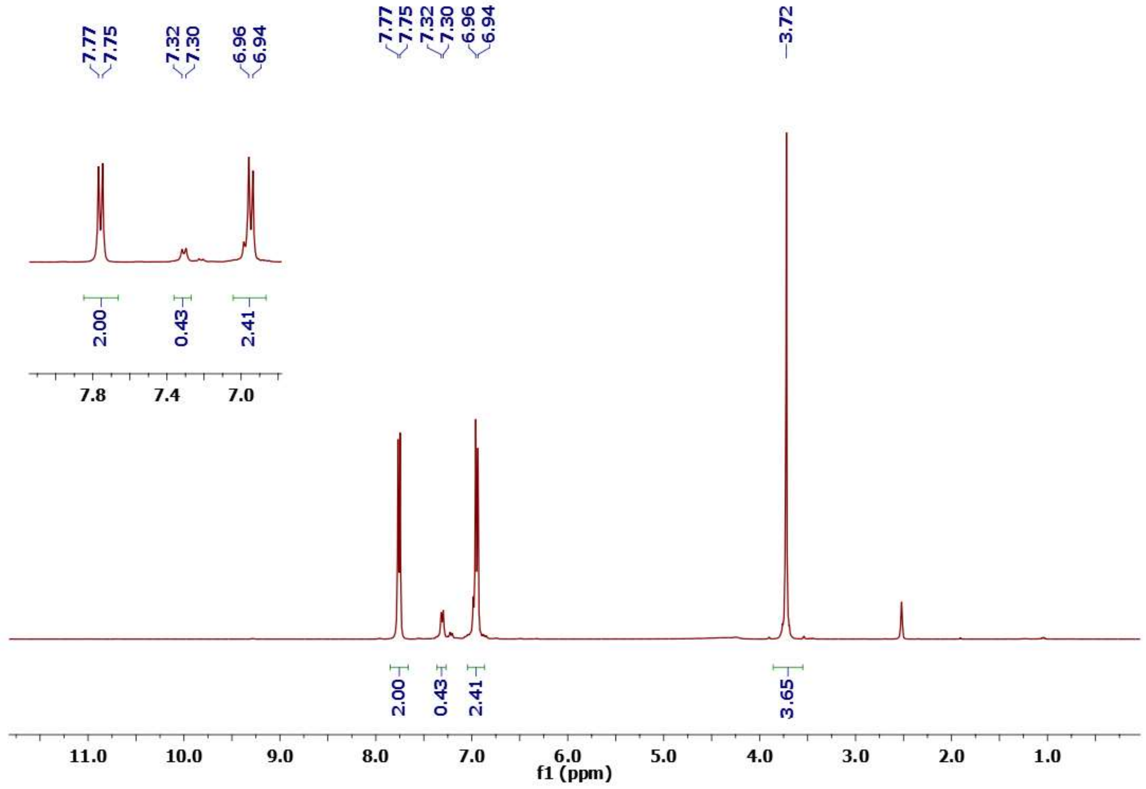

^{13}C NMR (DMSO-d6) spectrum of (4-methoxyphenyl)carbamothioyl cyanide (1:0.21 tautomeric ratio) (1i)

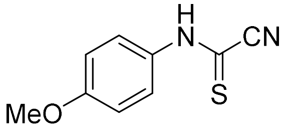

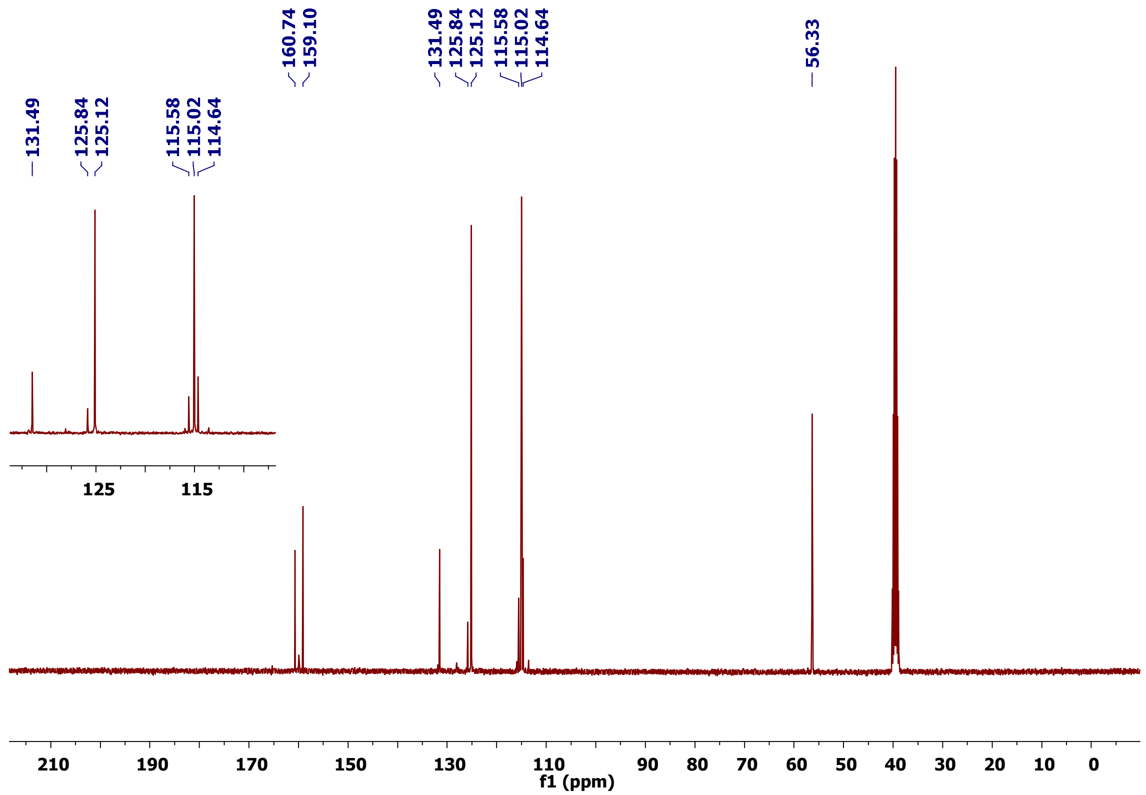

^{13}C-CRAPT NMR (DMSO-d6) spectrum of (4-methoxyphenyl)carbamothioyl cyanide (1:0.21 tautomeric ratio) (1i)

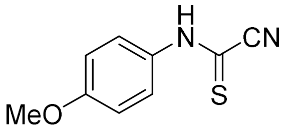

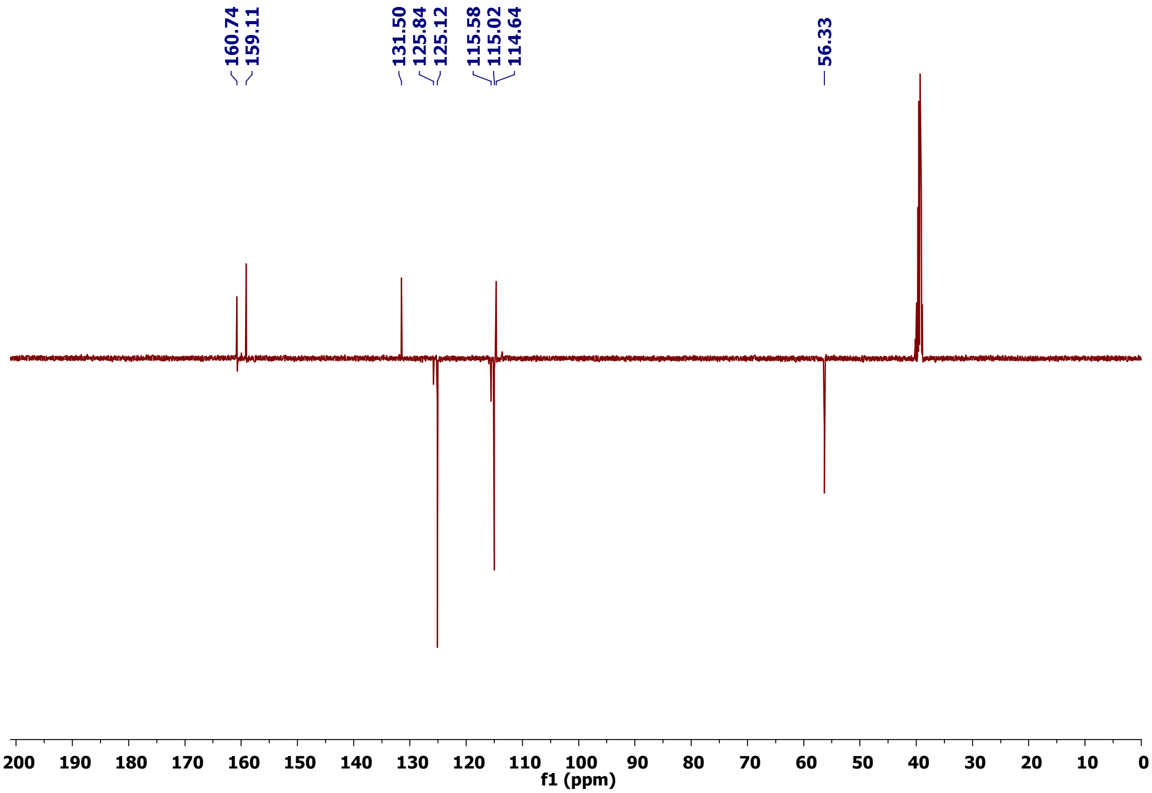

^1H NMR ($CDCl_3$) spectrum of (4-ethoxyphenyl)carbamothioyl cyanide (1:0.59 tautomeric ratio) (1j)

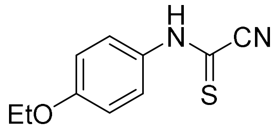

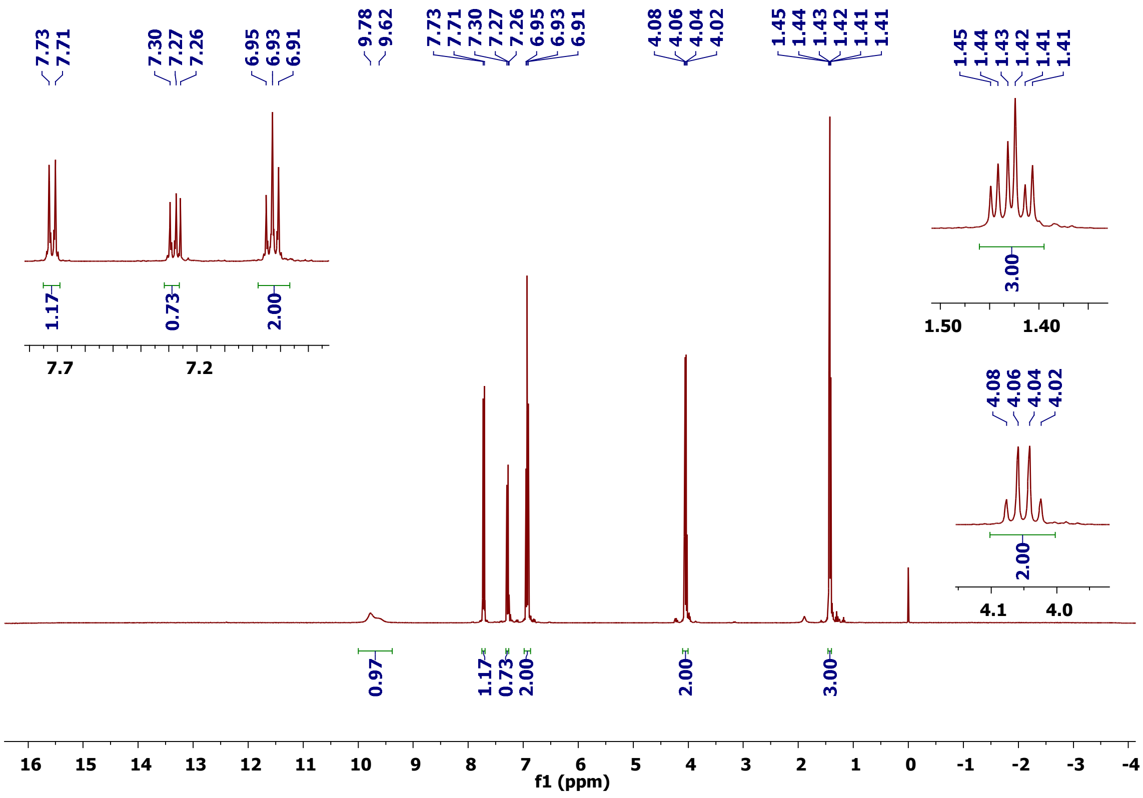

^{13}C NMR ($CDCl_3$) spectrum of (4-ethoxyphenyl)carbamothioyl cyanide (1:0.59 tautomeric ratio) (1j)

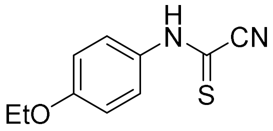

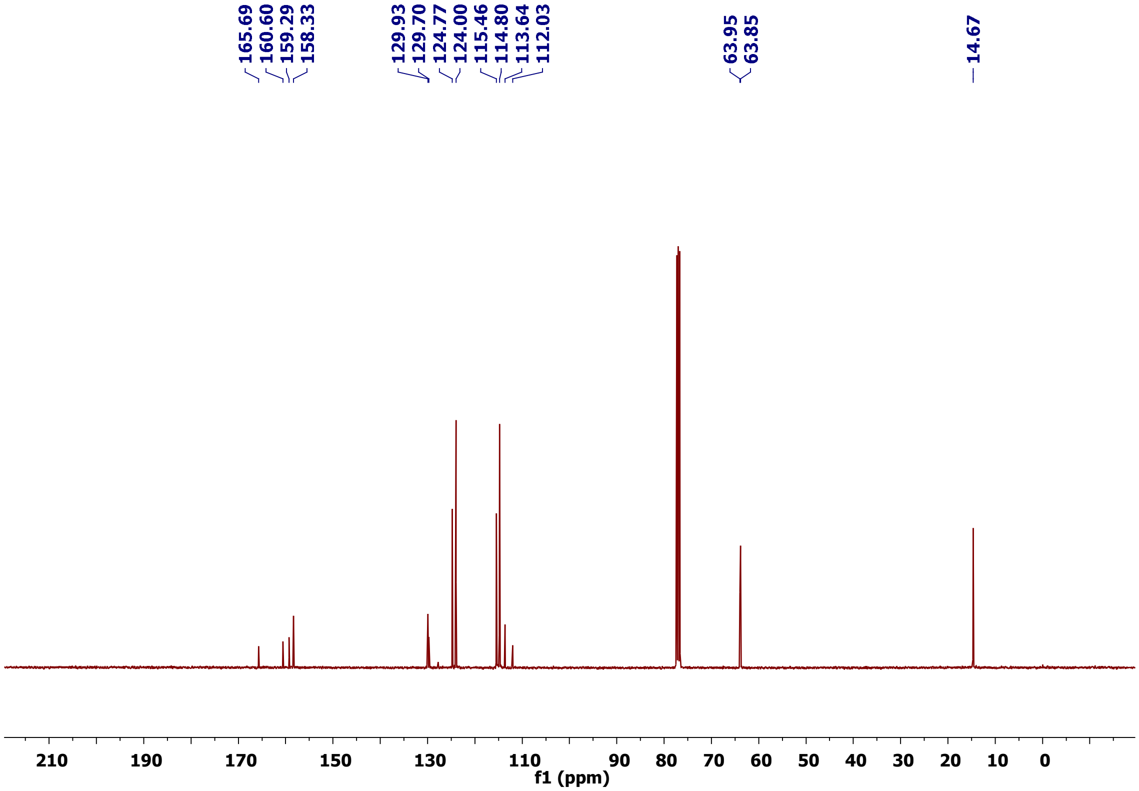

^{13}C DEPT-135 NMR ($CDCl_3$) spectrum of (4-ethoxyphenyl)carbamothioyl cyanide (1:0.59 tautomeric ratio) (1j)

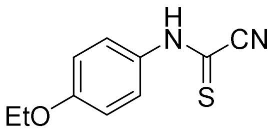

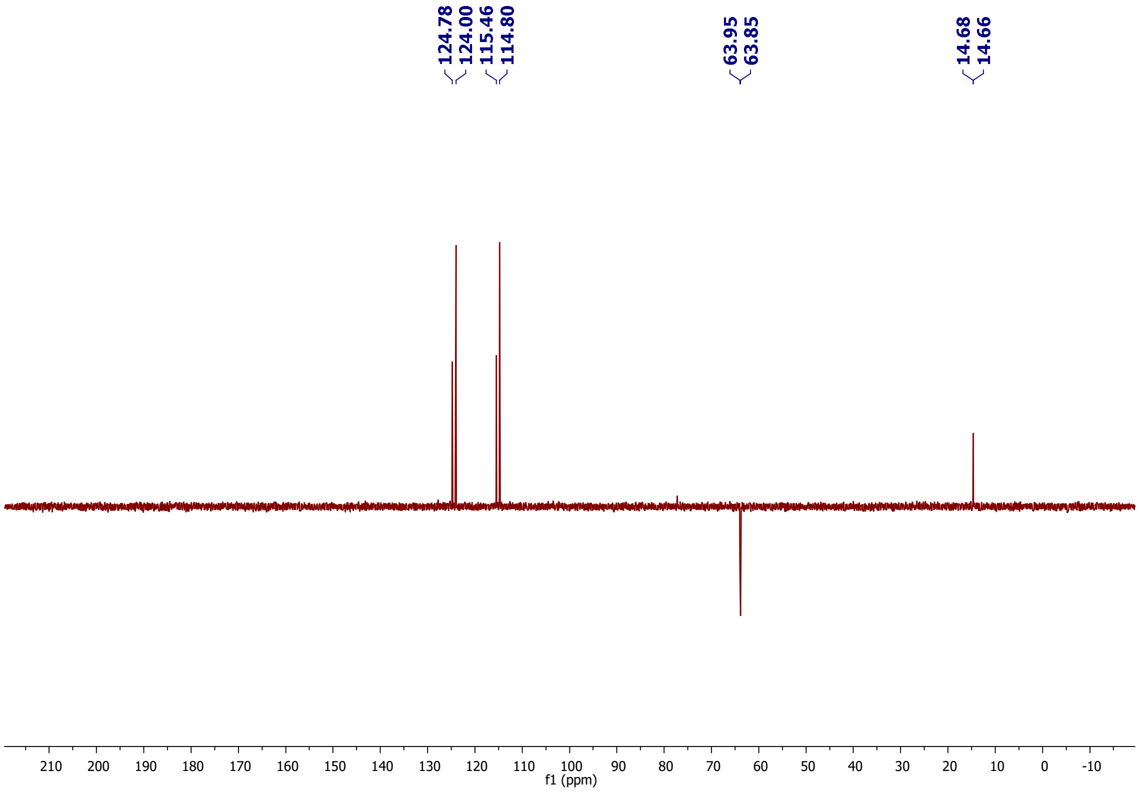

^1H-^1H-gDQCOSY NMR ($CDCl_3$) spectrum of (4-ethoxyphenyl)carbamothioyl cyanide (1:0.59 tautomeric ratio) (1j)

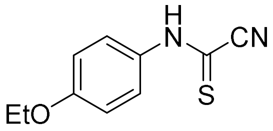

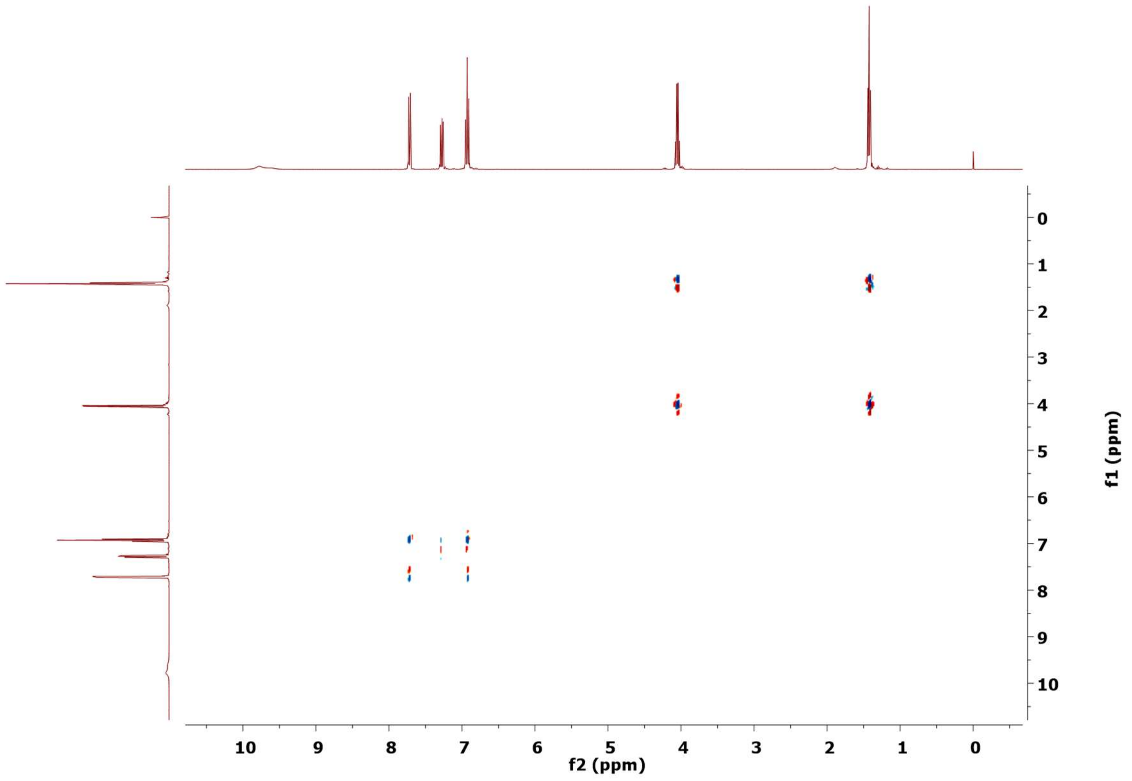

^{1}H-^{13}C-gHSQC NMR ($CDCl_3$) spectrum of (4-ethoxyphenyl)carbamothioyl cyanide (1:0.59 tautomeric ratio) (1j)

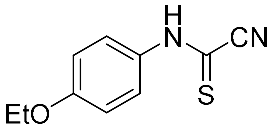

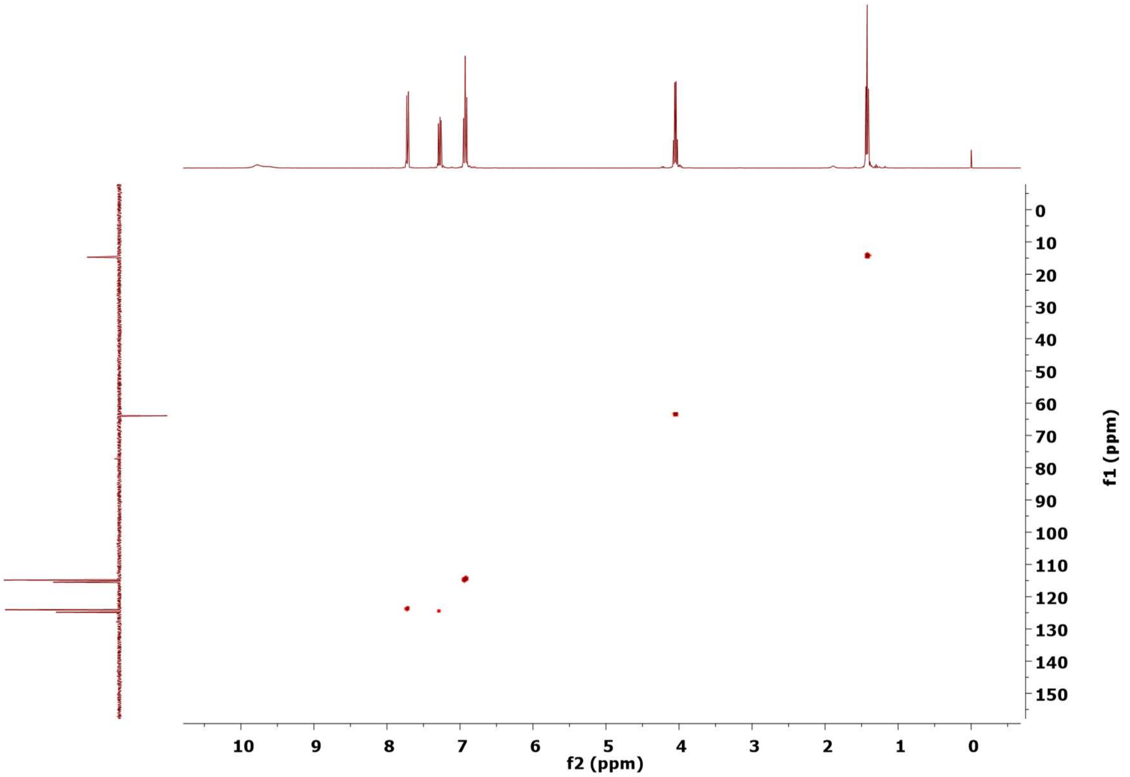

^{1}H NMR (DMSO-d6) spectrum of (4-(benzyloxy)phenyl)carbamothioyl cyanide (1k)

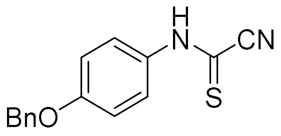

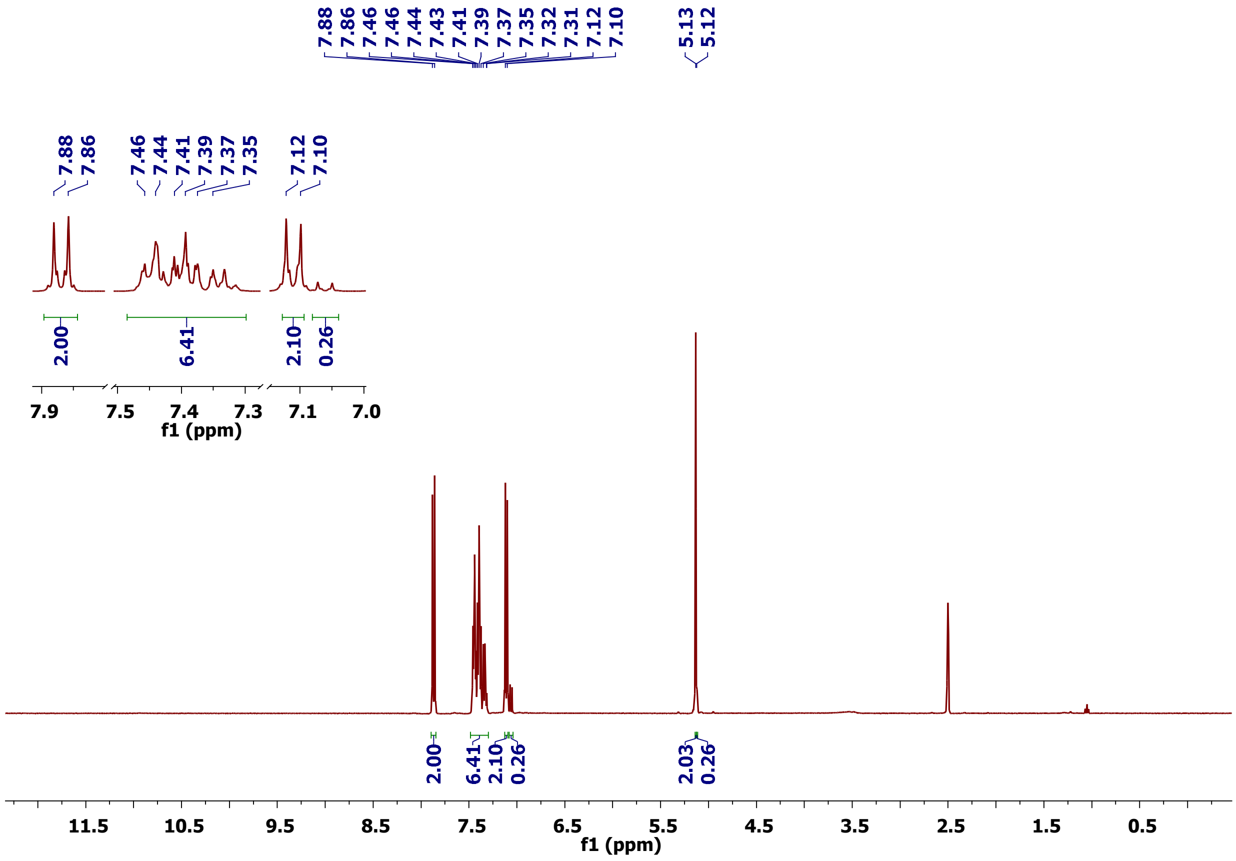

^{13}C NMR (DMSO-d6) spectrum of (4-(benzyloxy)phenyl)carbamothioyl cyanide (1k)

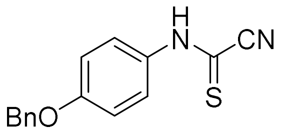

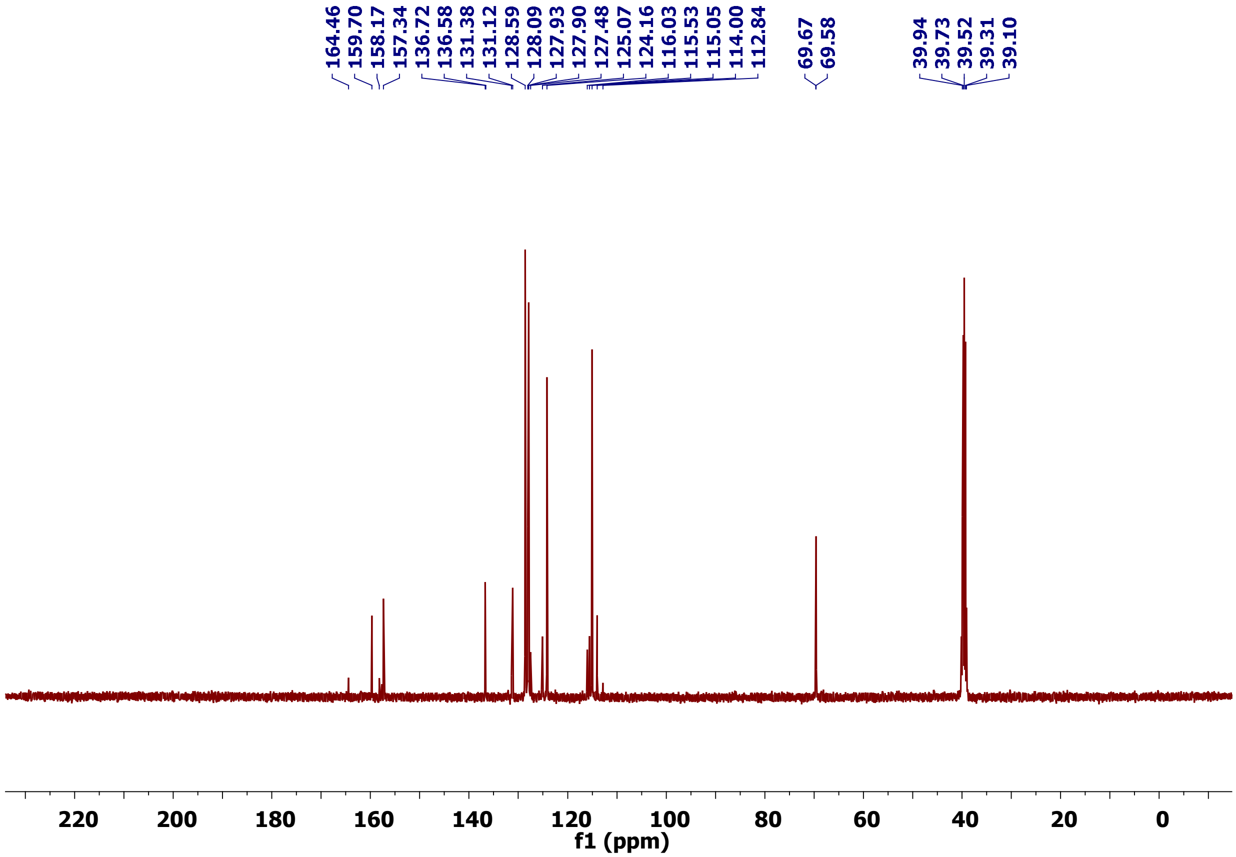

^{13}C CRAPT NMR (DMSO-d6) spectrum of (4-(benzyloxy)phenyl)carbamothioyl cyanide (1k)

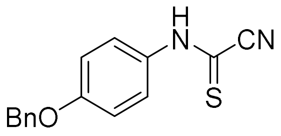

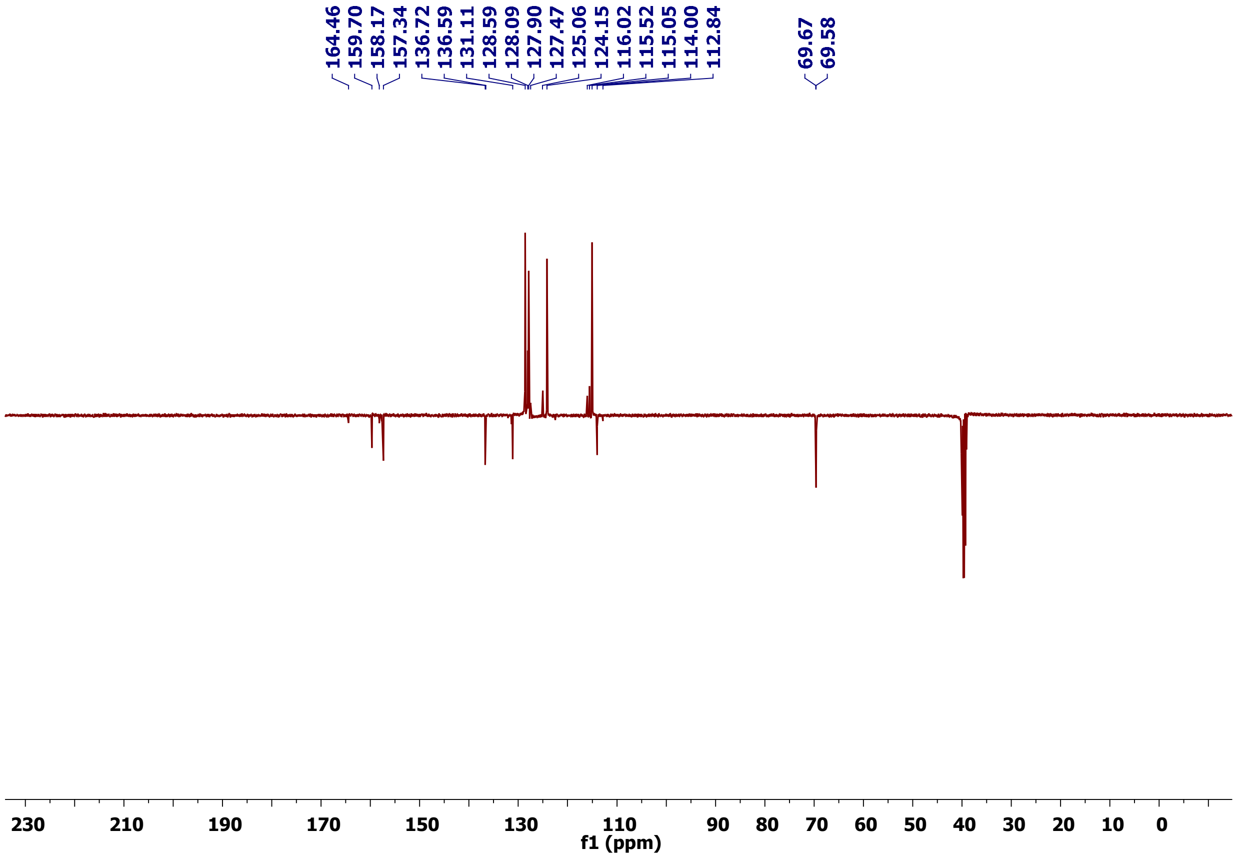

^{1}H-^{1}H gDQCOSY NMR (DMSO-d6) spectrum of (4-(benzyloxy)phenyl)carbamothioyl cyanide (1k)

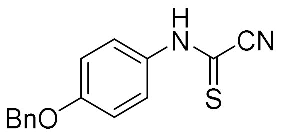

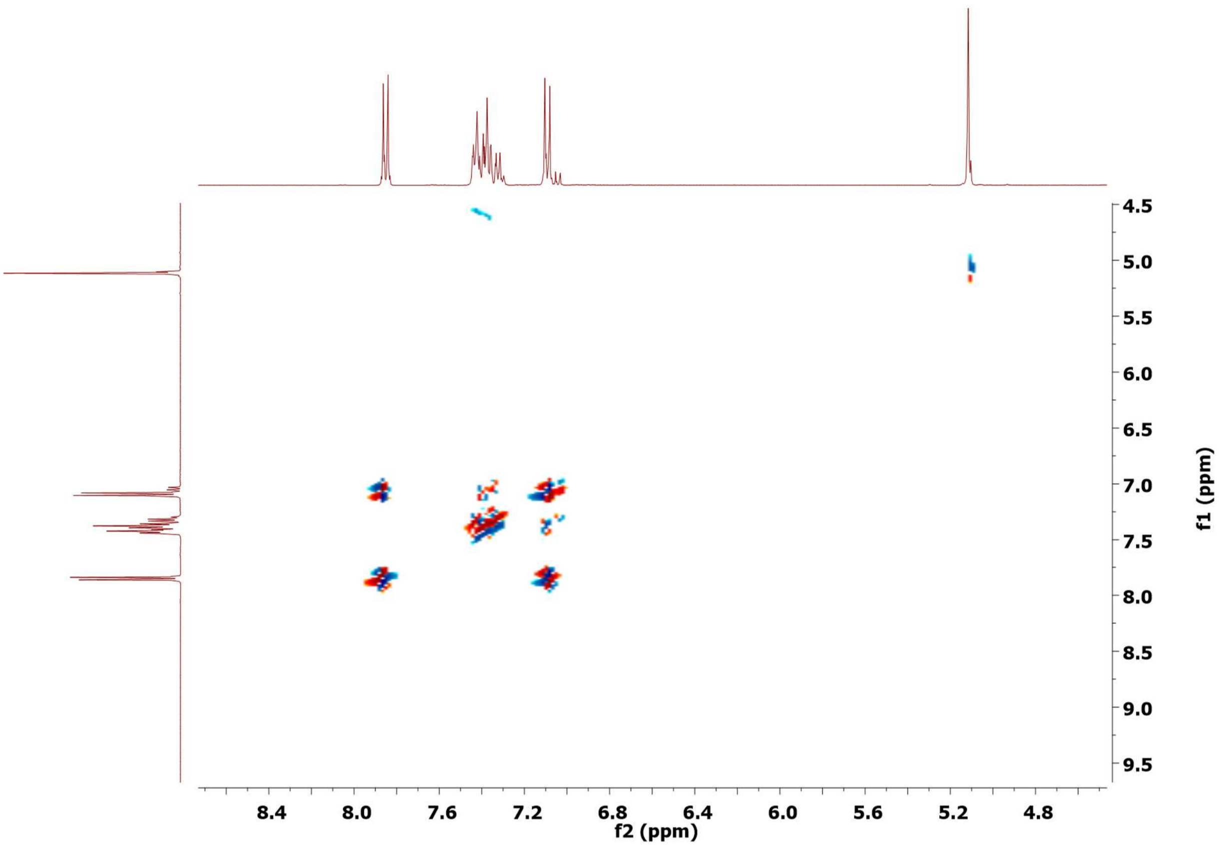

^{1}H-^{13}C-gHSQC NMR (DMSO-d6) spectrum of (4-(benzyloxy)phenyl)carbamothioyl cyanide (1k)

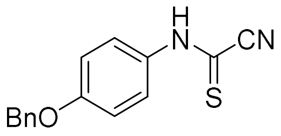

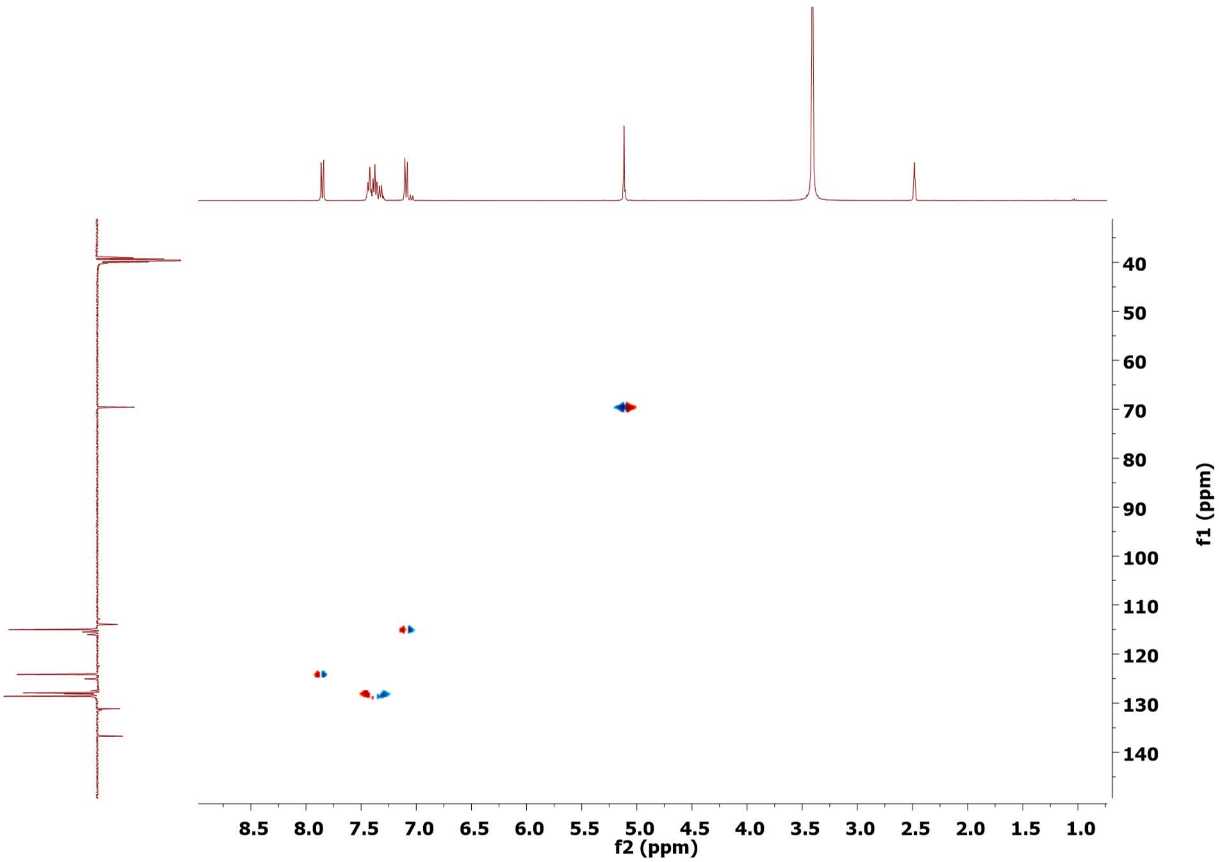

^{1}H-^{13}C-gHMBC NMR (DMSO-d6) spectrum of (4-(benzyloxy)phenyl)carbamothioyl cyanide (1k)

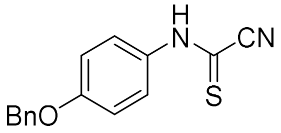

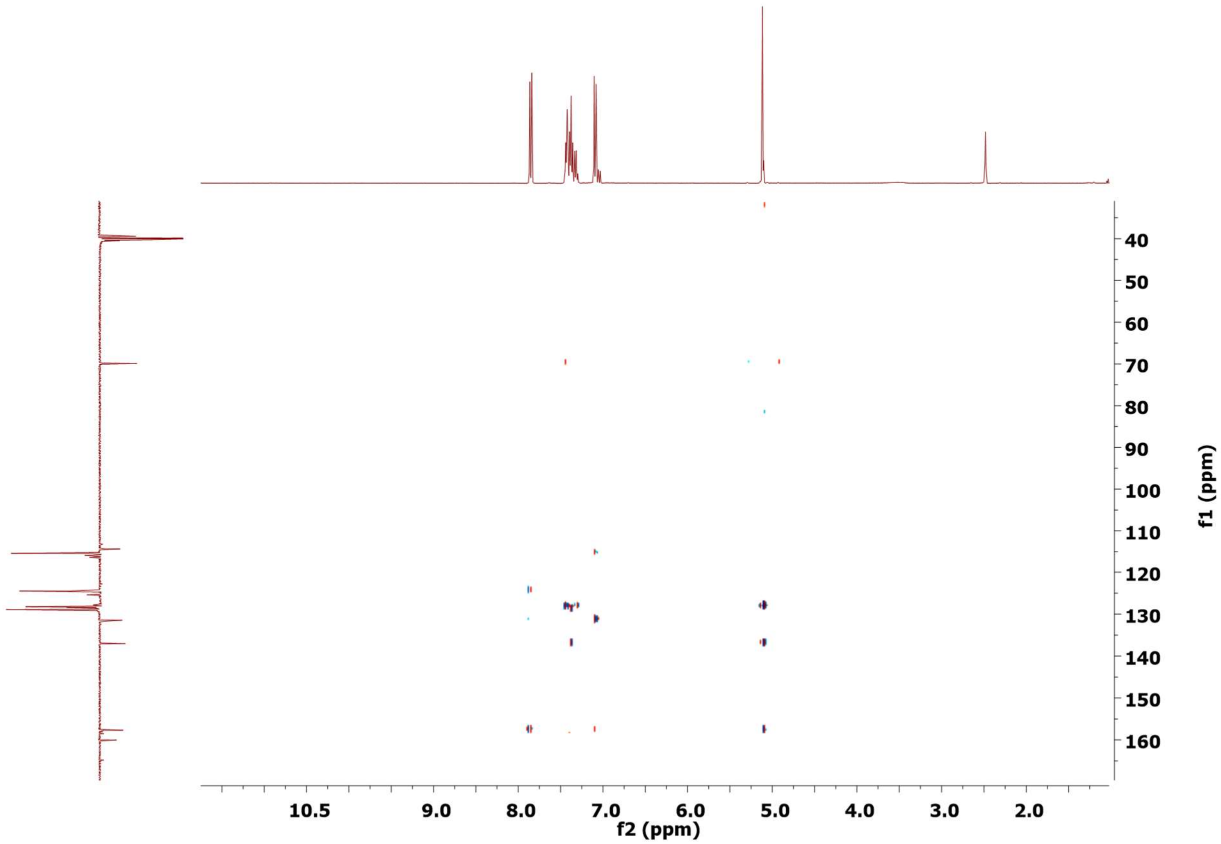

^{1}H NMR ($CDCl_3$) spectrum of (4-(methylthio)phenyl)carbamothioyl cyanide (1:0.19 tautomeric ratio) (1l)

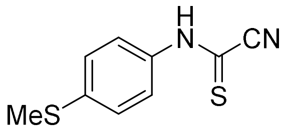

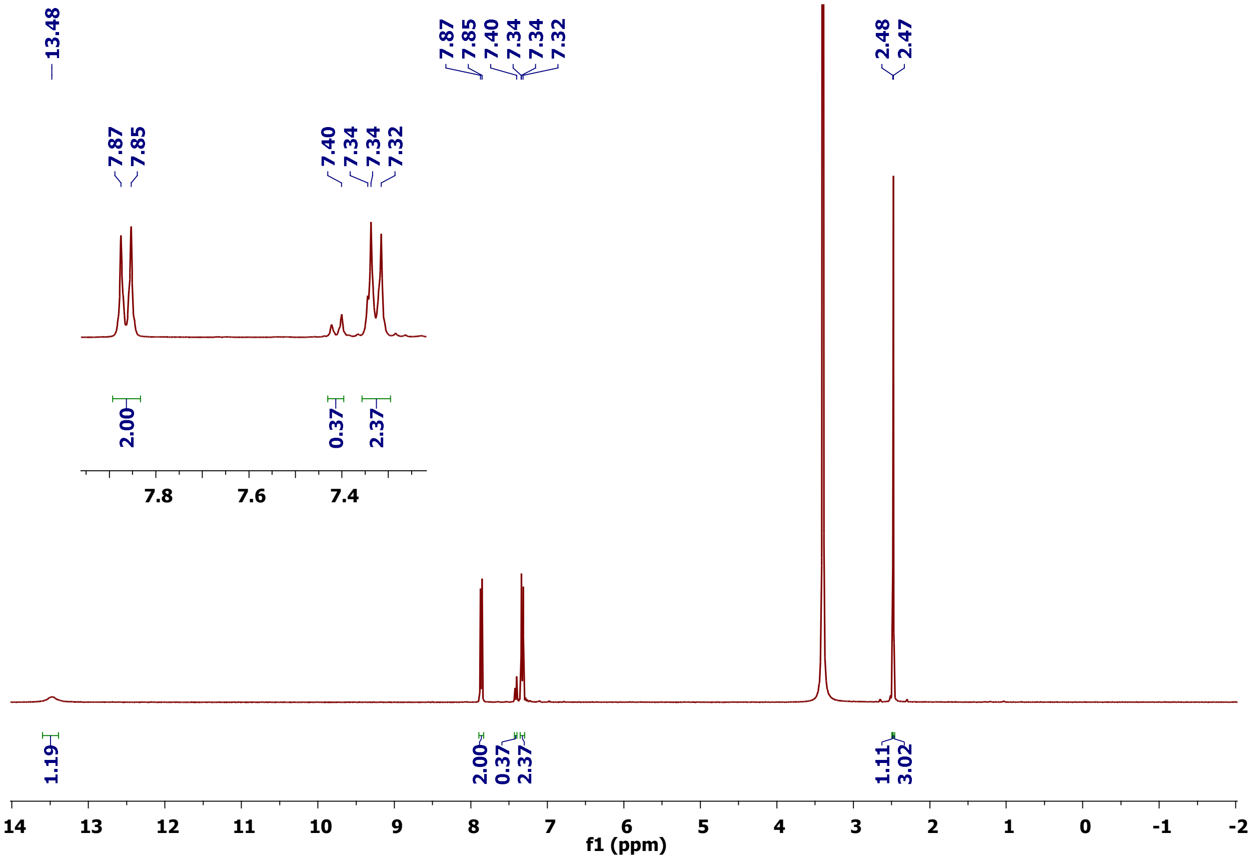

^{13}C NMR ($CDCl_3$) spectrum of (4-(methylthio)phenyl)carbamothioyl cyanide (1:0.19 tautomeric ratio) (11)

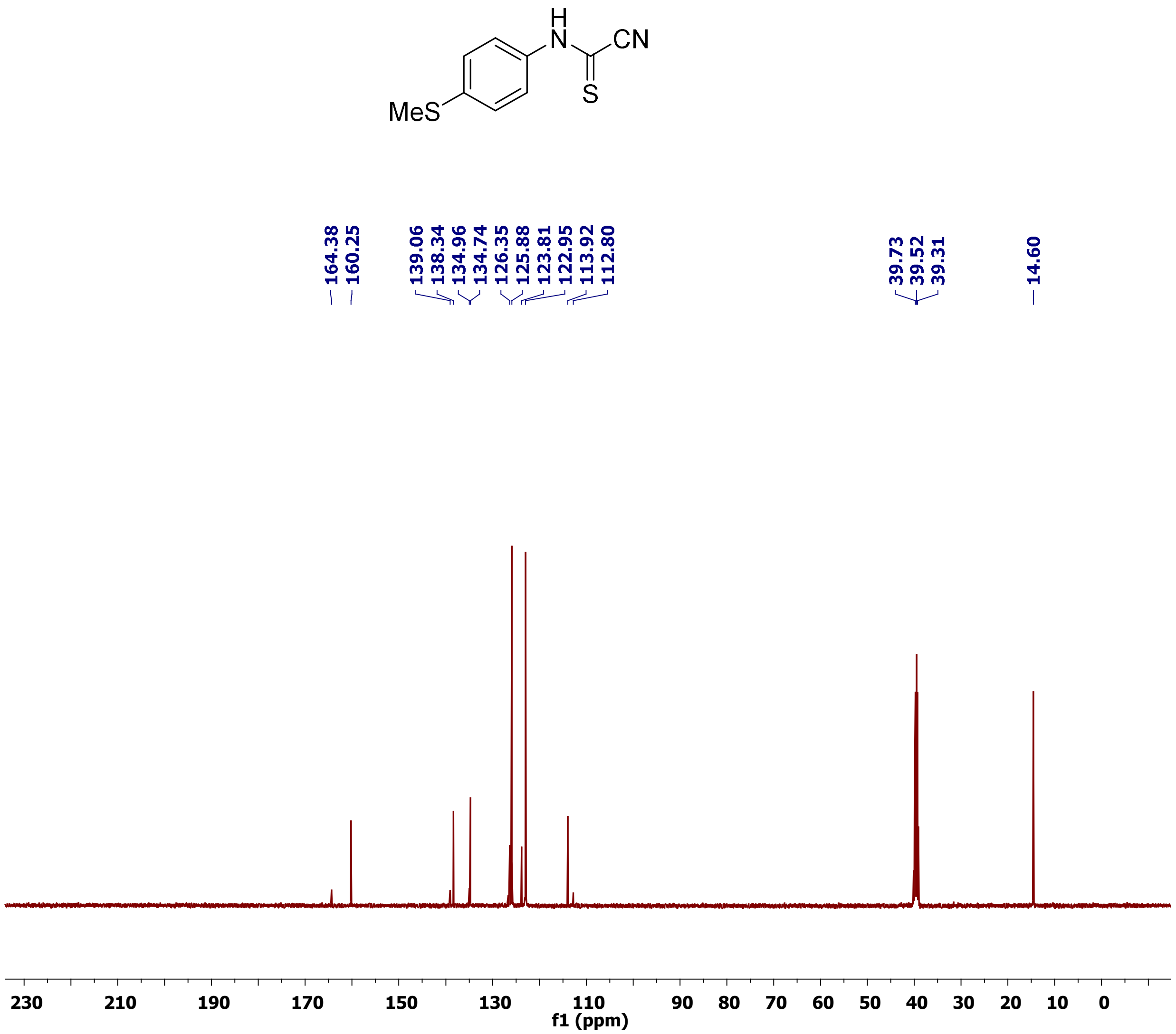

^{13}C-CRAPT NMR ($CDCl_3$) spectrum of (4-(methylthio)phenyl)carbamothioyl cyanide (1:0.19 tautomeric ratio) (1l)

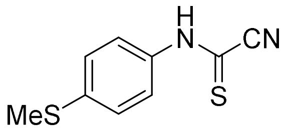

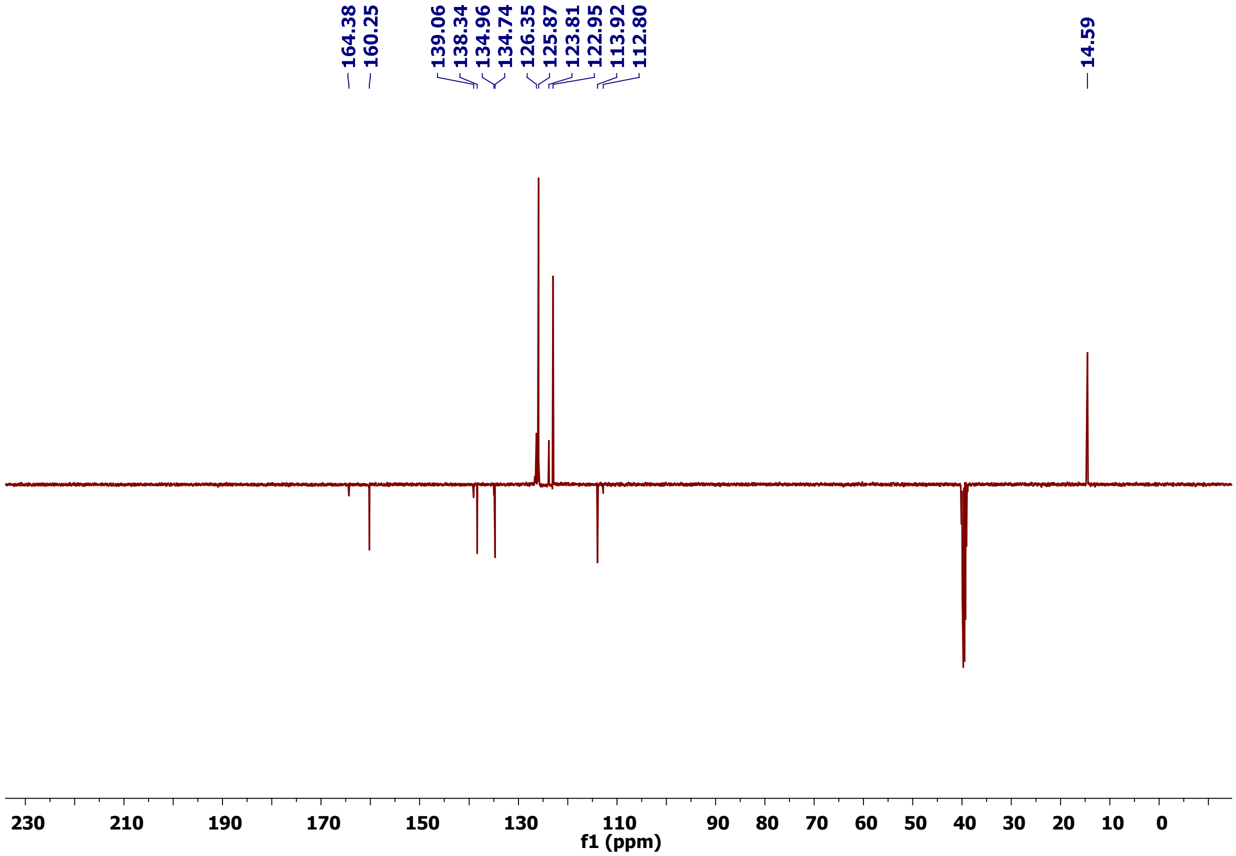

^{1}H-^{1}H -gDQFCOSY NMR ($CDCl_3$) spectrum of (4-(methylthio)phenyl)carbamothioyl cyanide (1:0.19 tautomeric ratio) (1l)

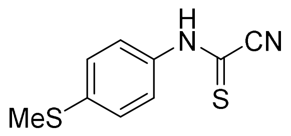

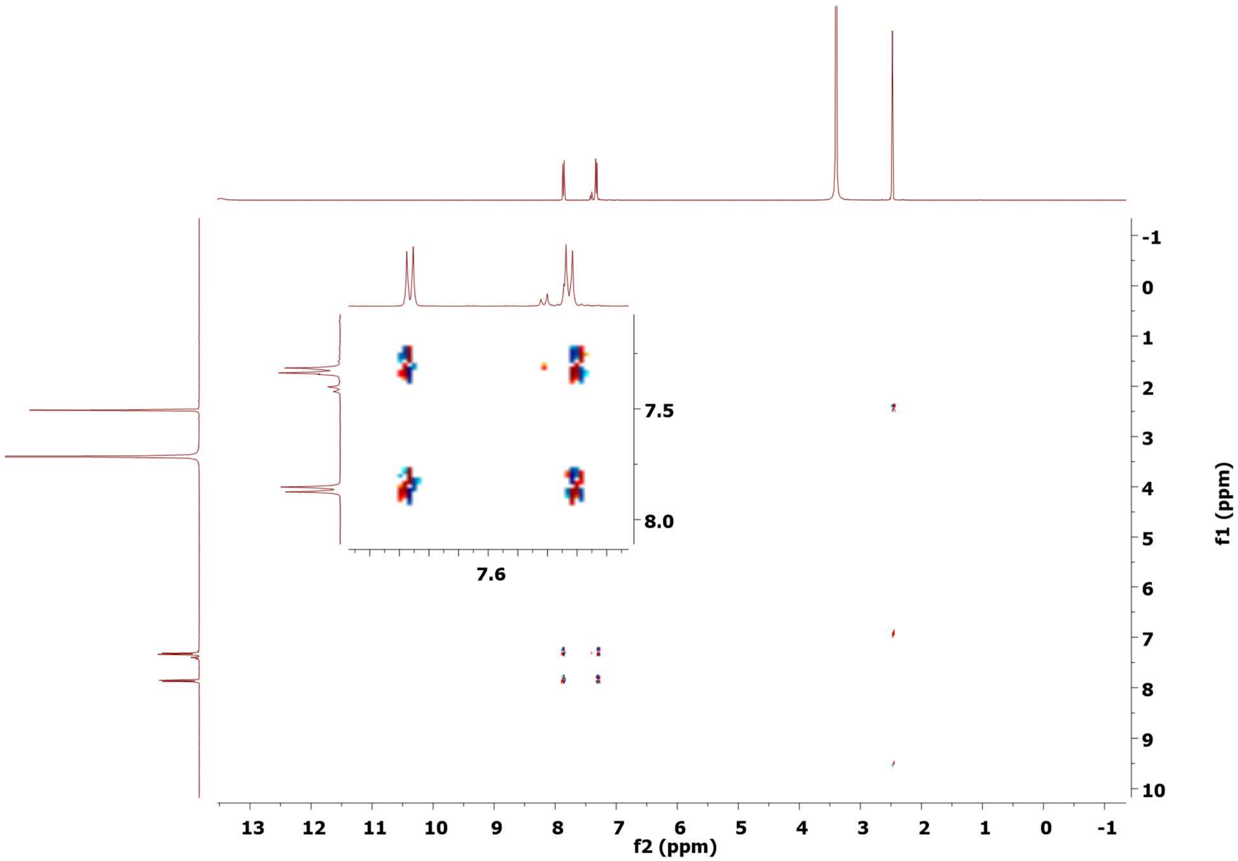

^{1}H-^{13}C-gHSQCAD NMR ($CDCl_3$) spectrum of (4-(methylthio)phenyl)carbamothioyl cyanide (1:0.19 tautomeric ratio) (1l)

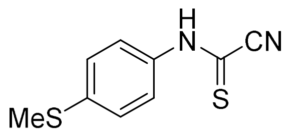

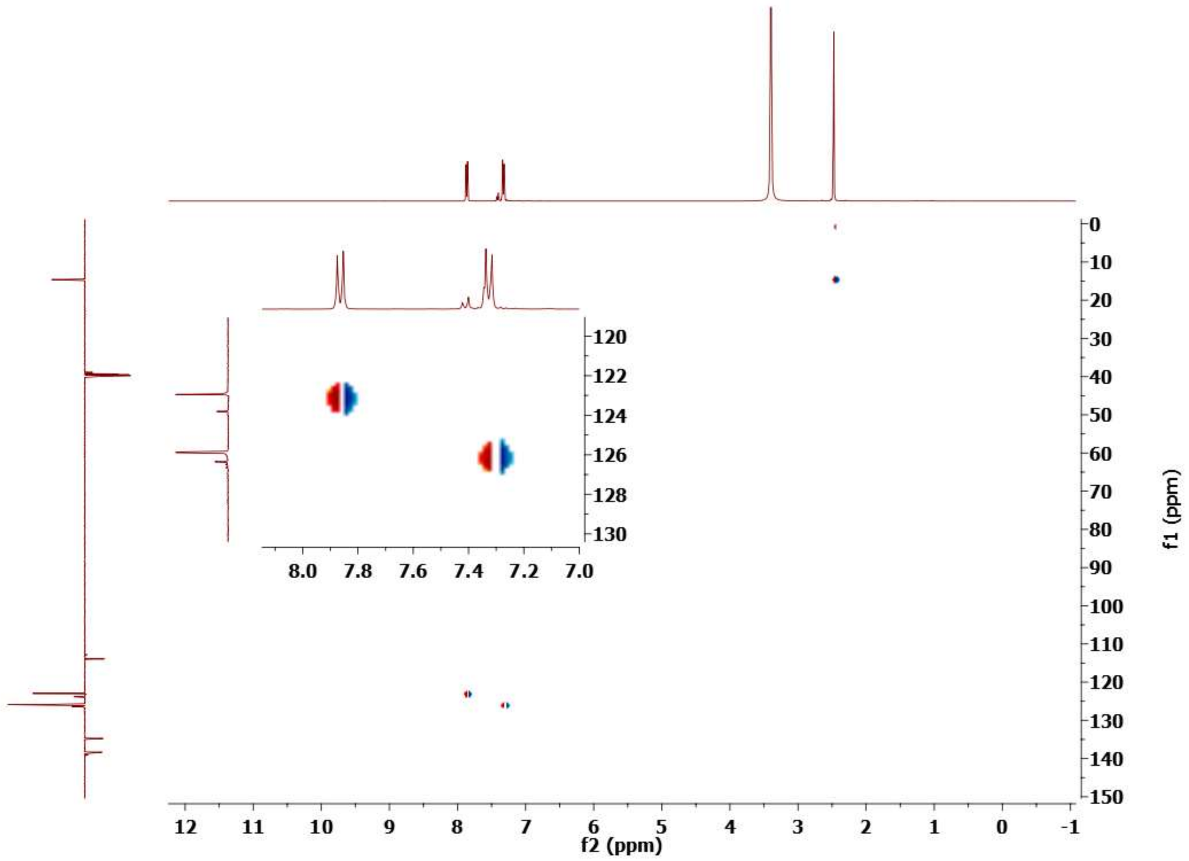

^{1}H-^{13}C-gHMBC NMR ($CDCl_3$) spectrum of (4-(methylthio)phenyl)carbamothioyl cyanide (1:0.19 tautomeric ratio) (1l)

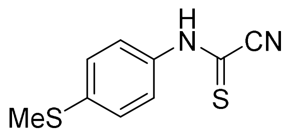

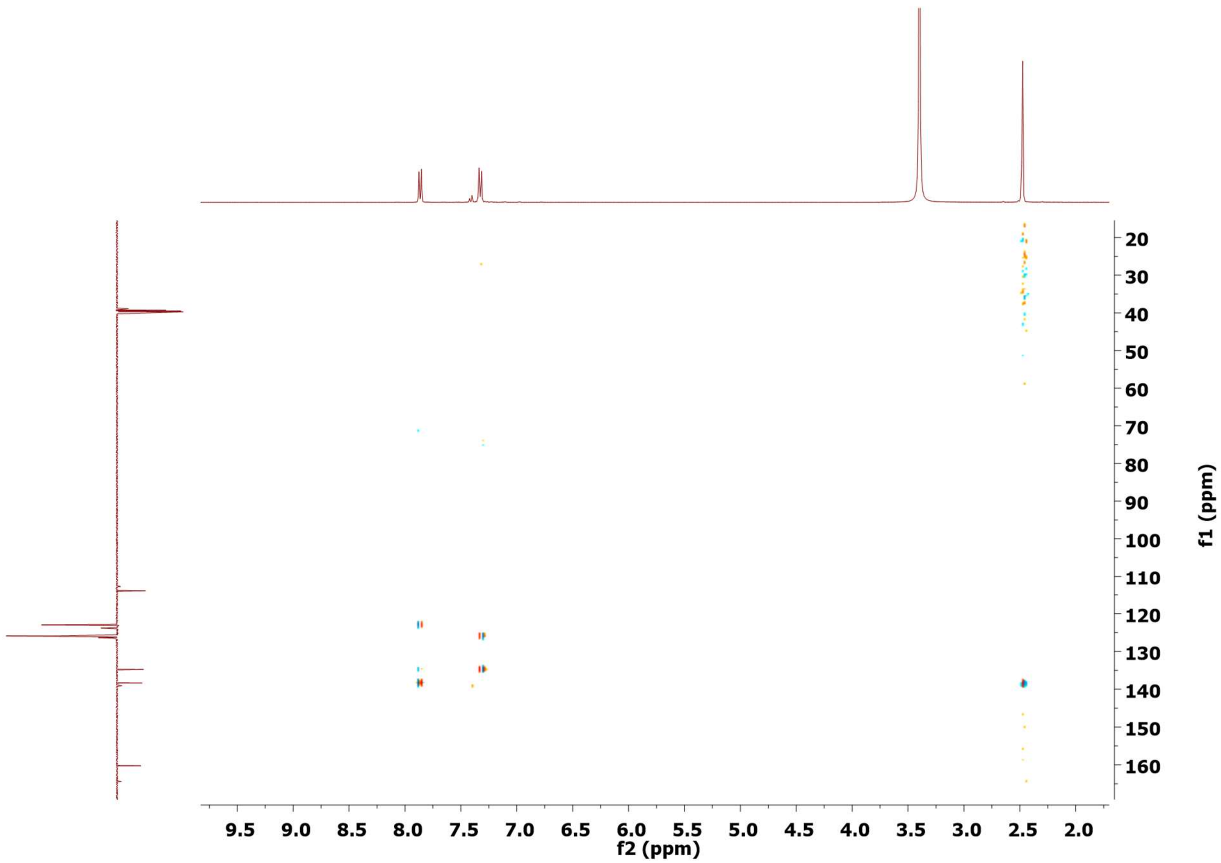

^{1}H NMR (DMSO-d6) spectrum of methyl 4-((cyanocarbonothioyl)amino)benzoate (1:0.13 tautomeric ratio) (1m)

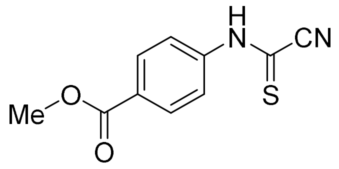

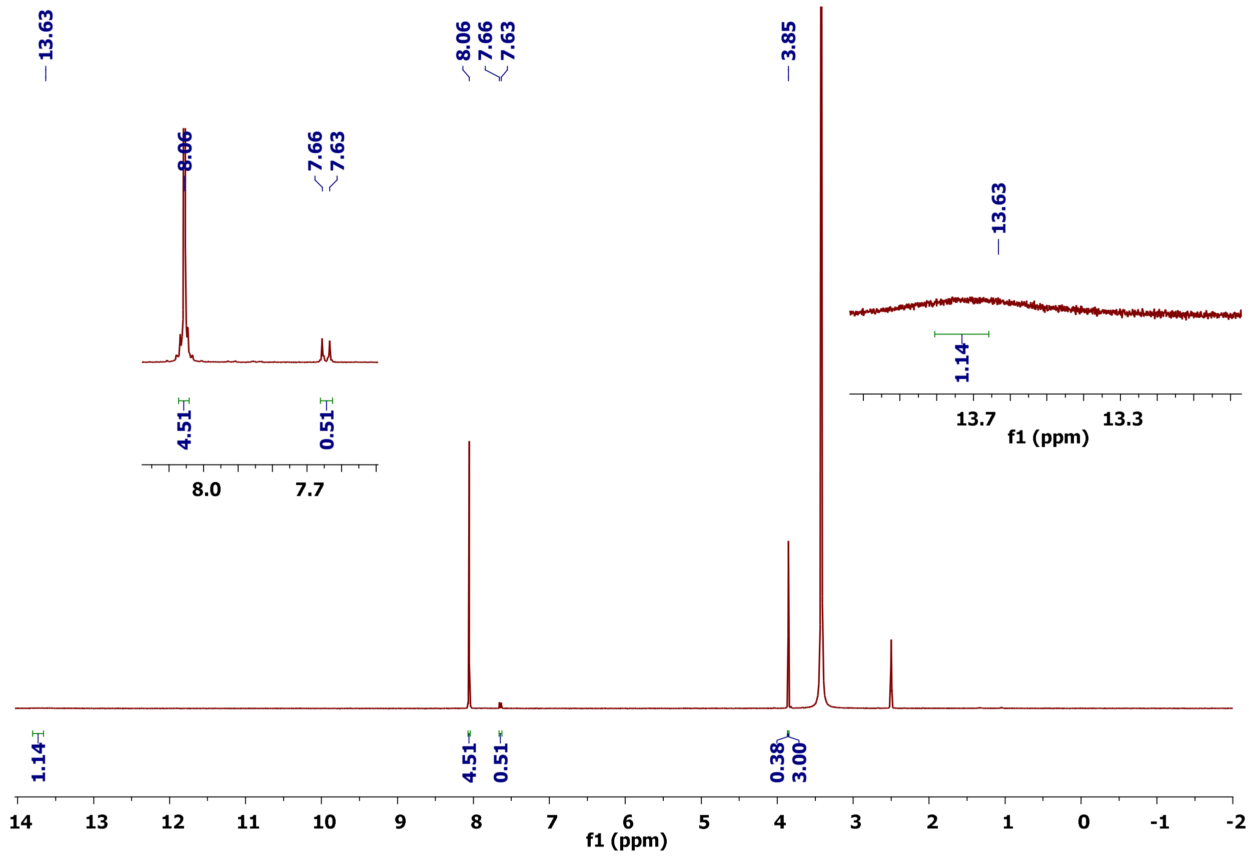

^{13}C NMR (DMSO-d6) spectrum of methyl 4-((cyanocarbonothioyl)amino)benzoate (1:0.13 tautomeric ratio) (1m)

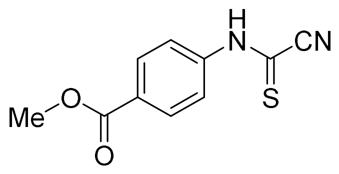

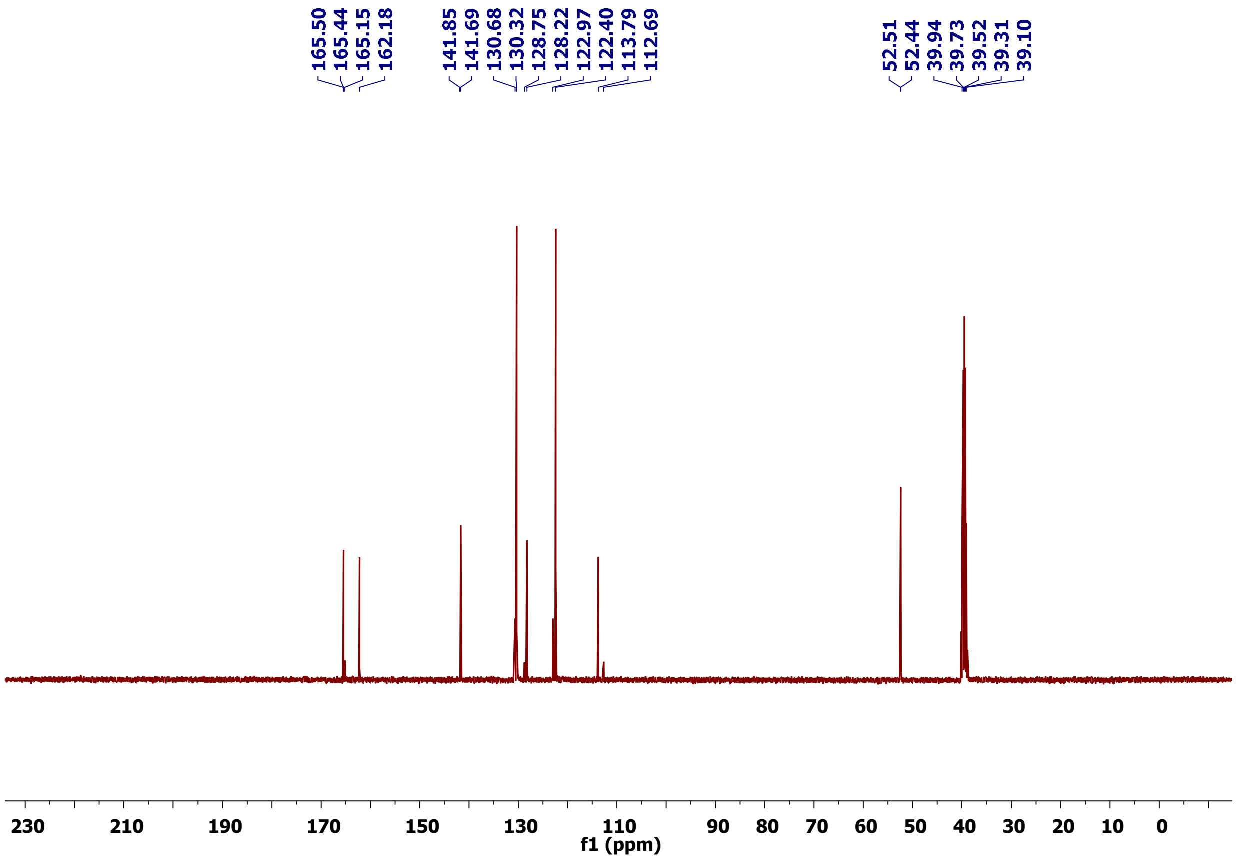

^{13}C-CRAPT NMR (DMSO-d6) spectrum of methyl 4-((cyanocarbonothioyl)amino)benzoate (1:0.13 tautomeric ratio) (1m)

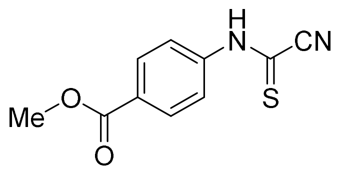

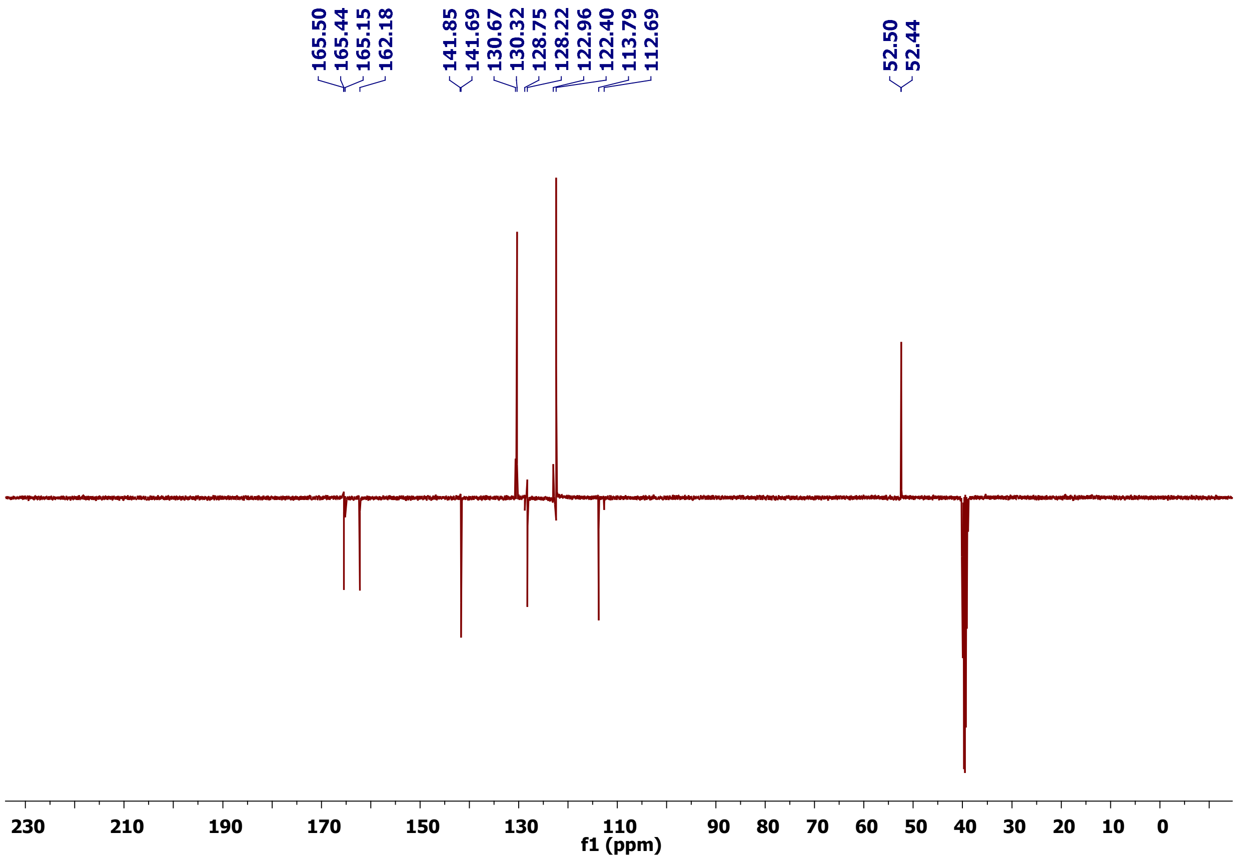

^{1}H-^{1}H -gDQFCOSY NMR (DMSO-d6) spectrum of methyl 4-((cyanocarbonothioyl)amino)benzoate (1:0.13 tautomeric ratio) (1m)

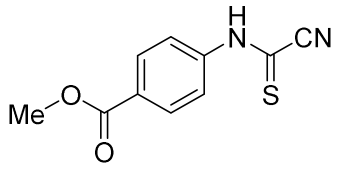

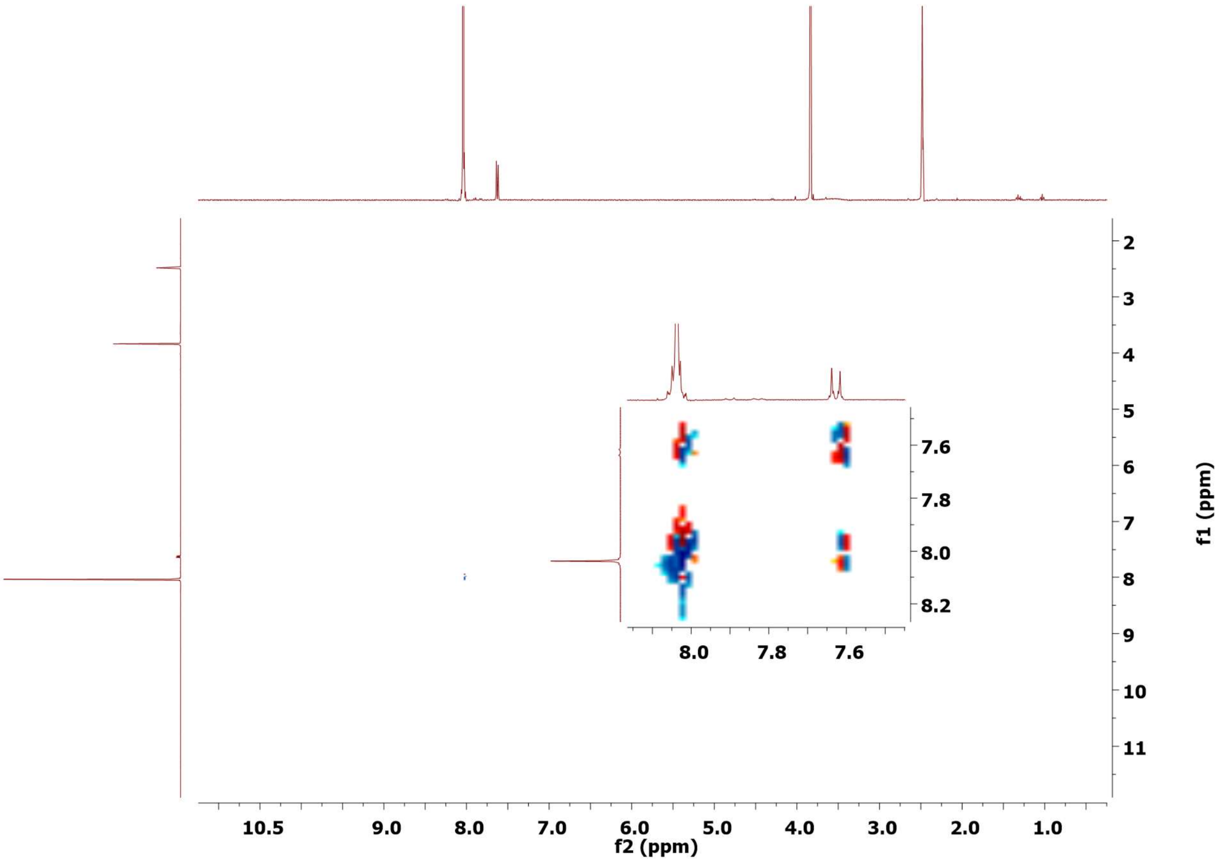

^{1}H-^{13}C-gHSQC NMR (DMSO-d6) spectrum of methyl 4-((cyanocarbonothioyl)amino)benzoate (1:0.13 tautomeric ratio) (1m)

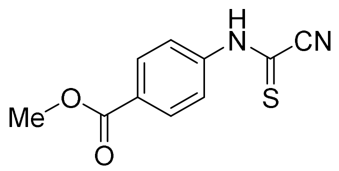

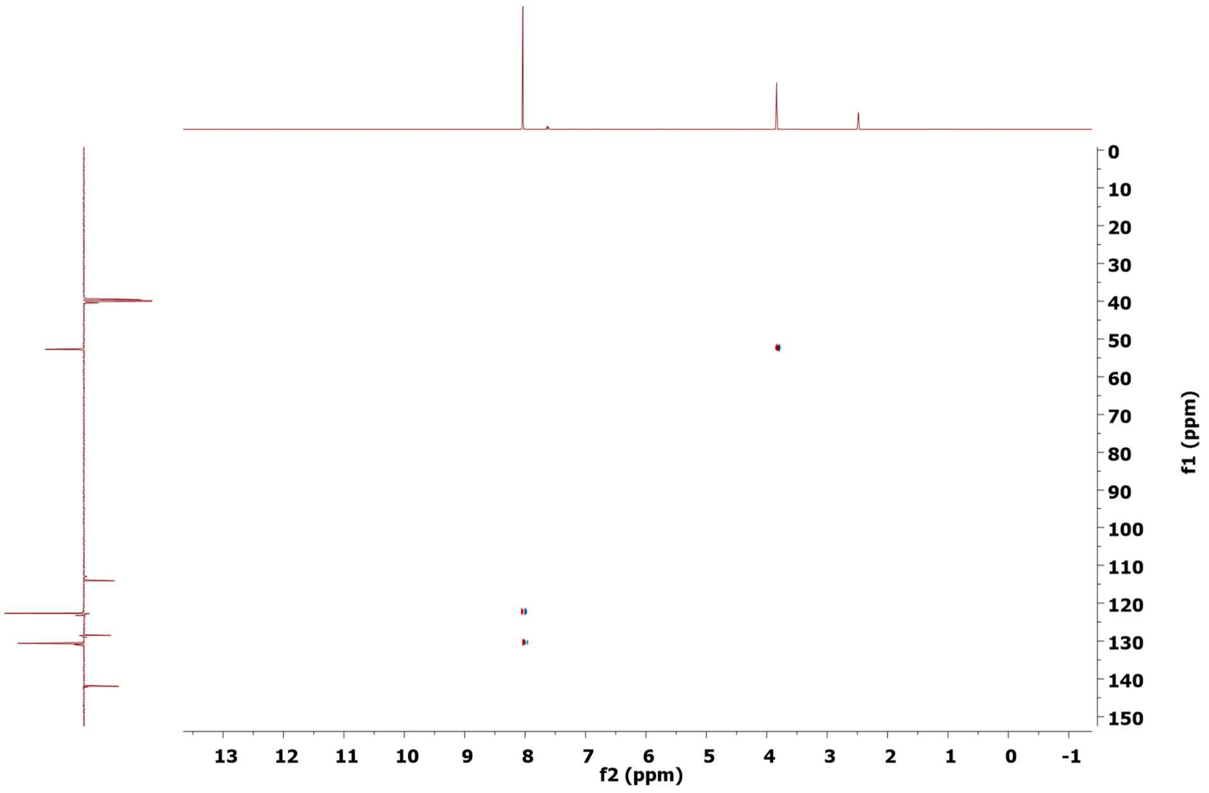

^{1}H-^{13}C-gHMBC NMR (DMSO-d6) spectrum of methyl 4-((cyanocarbonothioyl)amino)benzoate (1:0.13 tautomeric ratio) (1m)

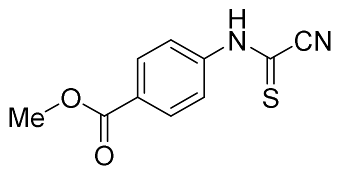

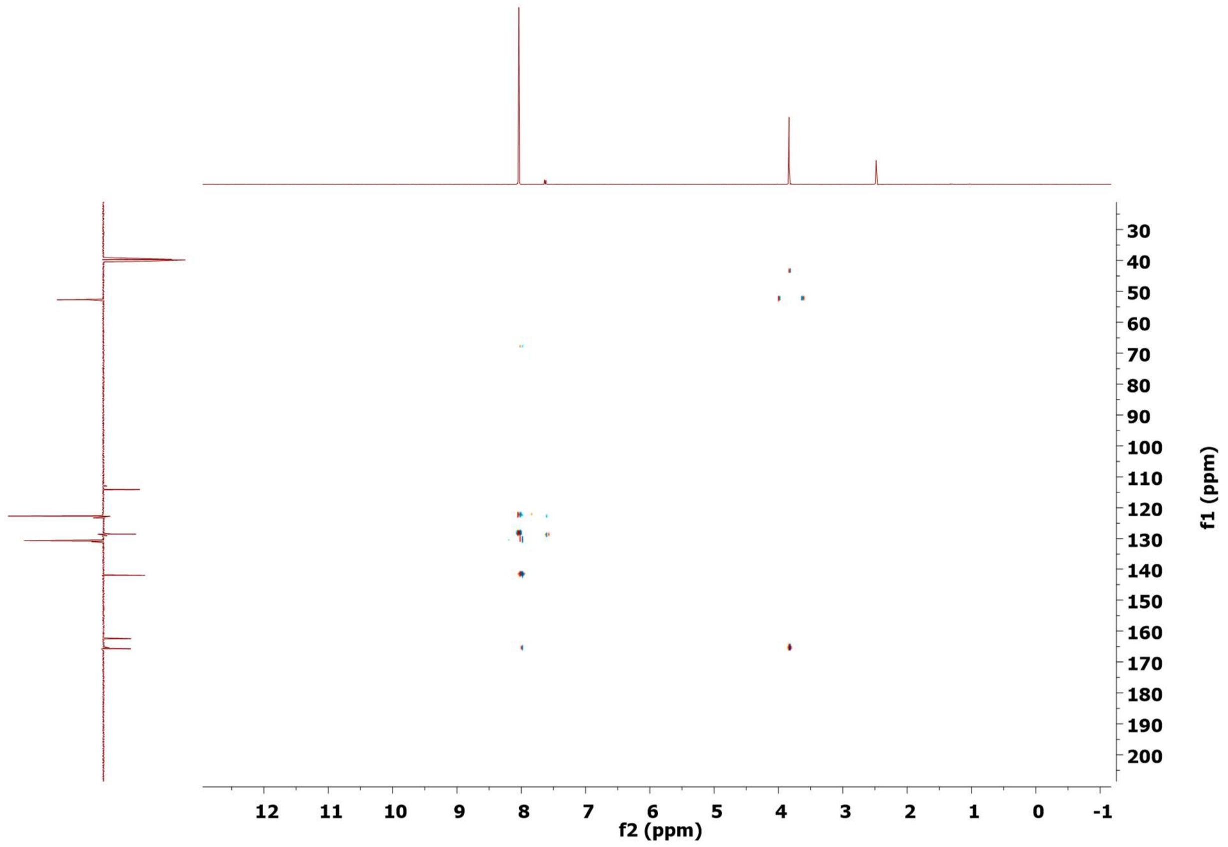

^1H NMR (DMSO-d6) spectrum of ethyl 4-((cyanocarbonothioyl)amino)benzoate (1:0.09 tautomeric ratio) (1n)

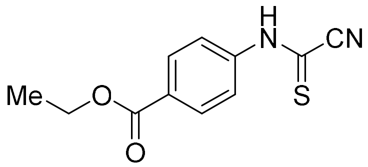

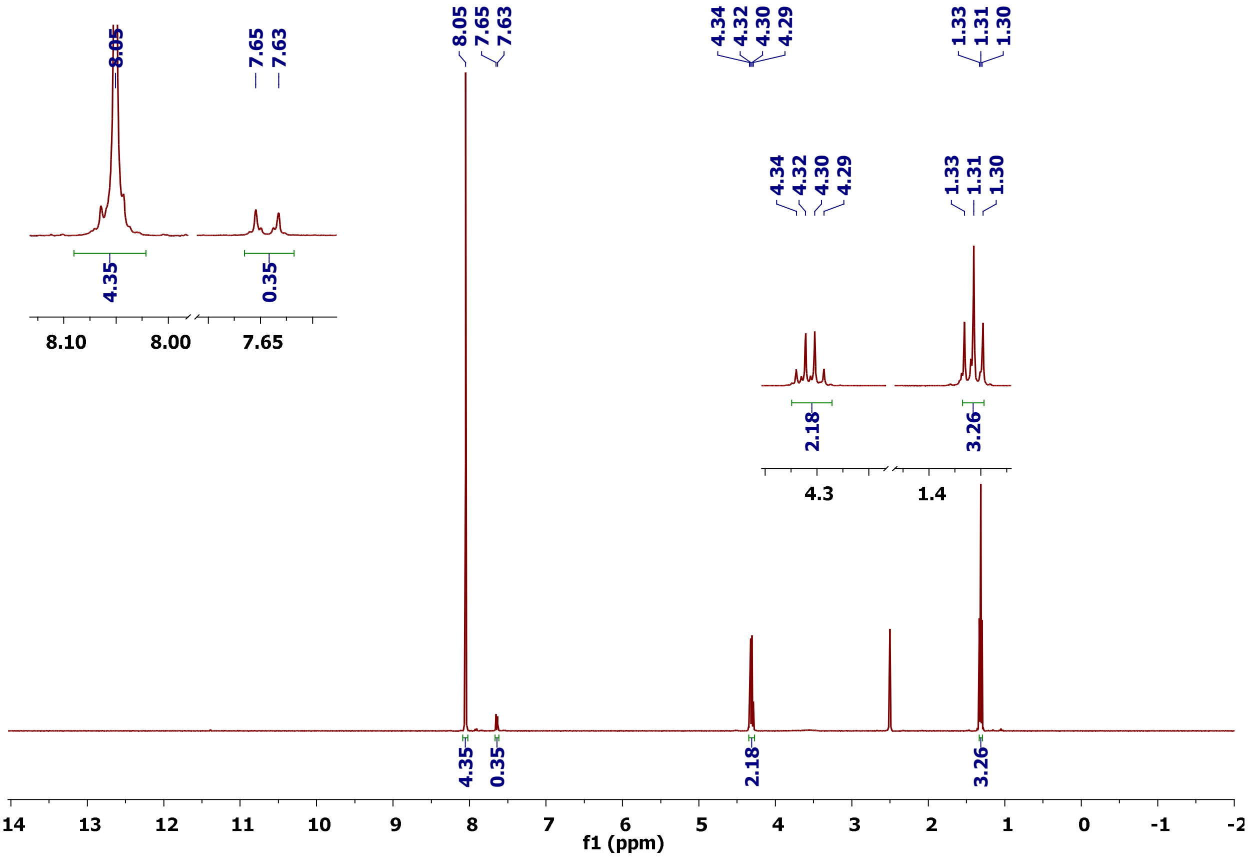

^{13}C NMR (DMSO-d6) spectrum of ethyl 4-((cyanocarbonothioyl)amino)benzoate (1:0.09 tautomeric ratio) (1n)

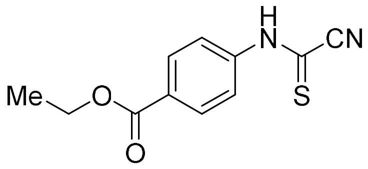

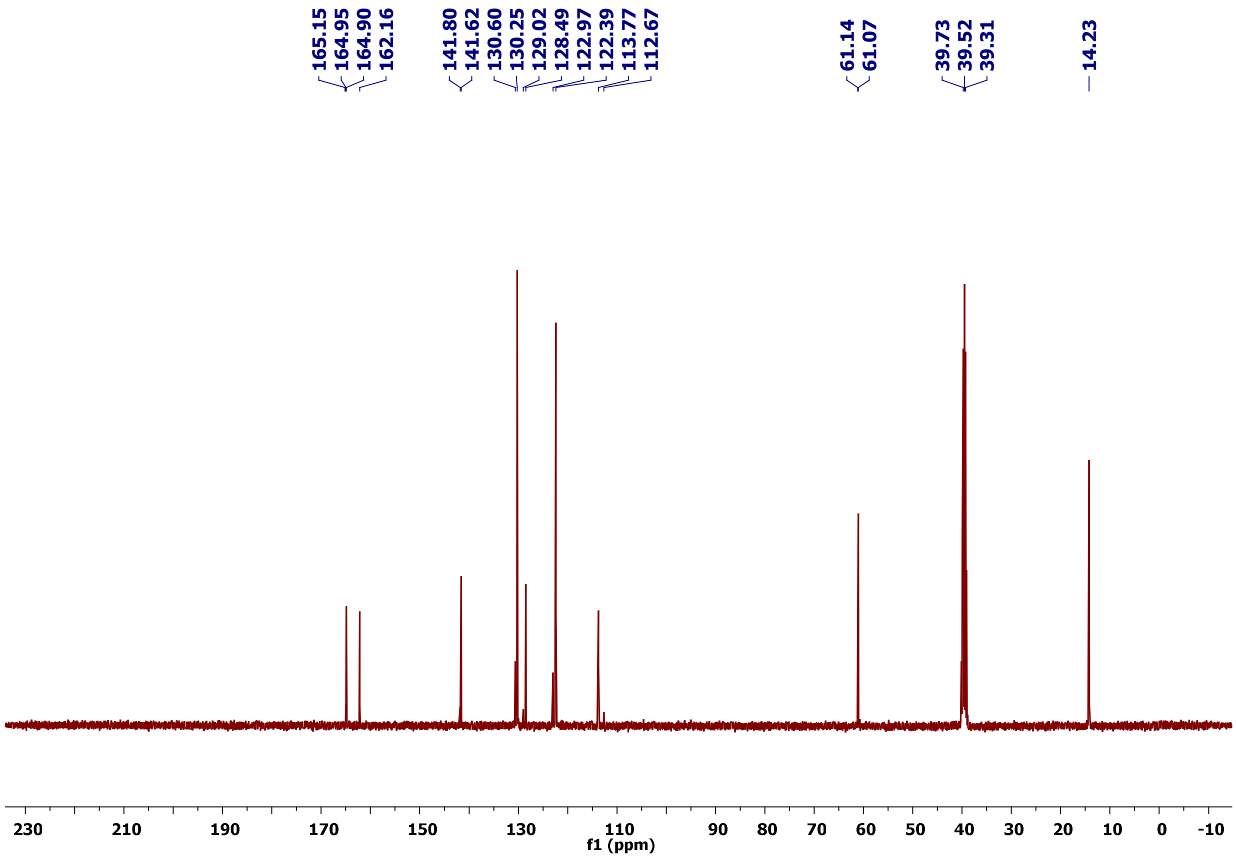

^{13}C-CRAPT NMR (DMSO-d6) spectrum of ethyl 4-((cyanocarbonothioyl)amino)benzoate (1:0.09 tautomeric ratio) (1n)

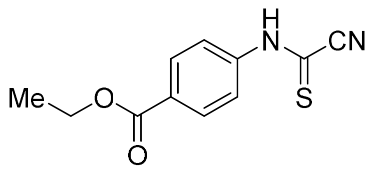

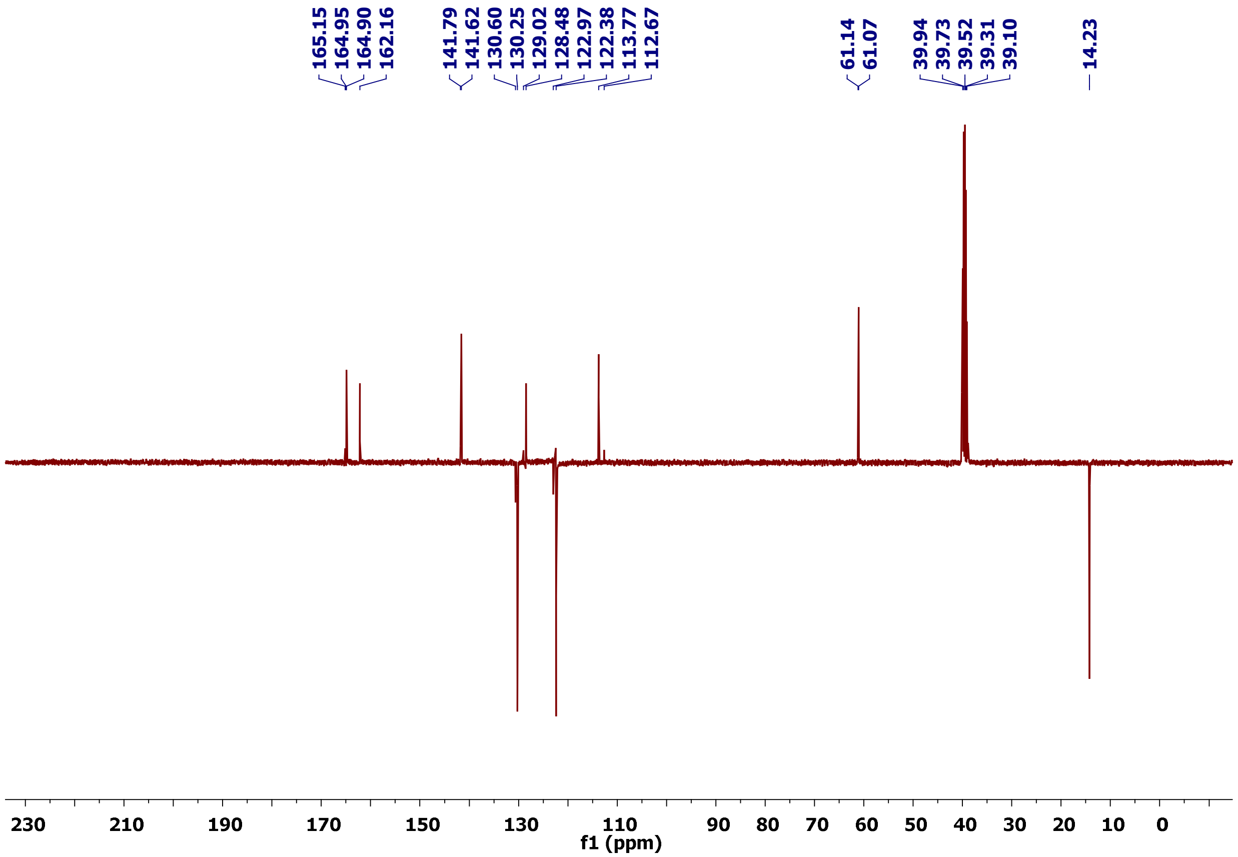

^{1}H-^{1}H -gDQFCOSY NMR (DMSO-d6) spectrum of ethyl 4-((cyanocarbonothioyl)amino)benzoate (1:0.09 tautomeric ratio) (1n)

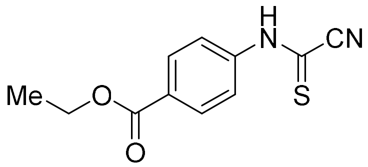

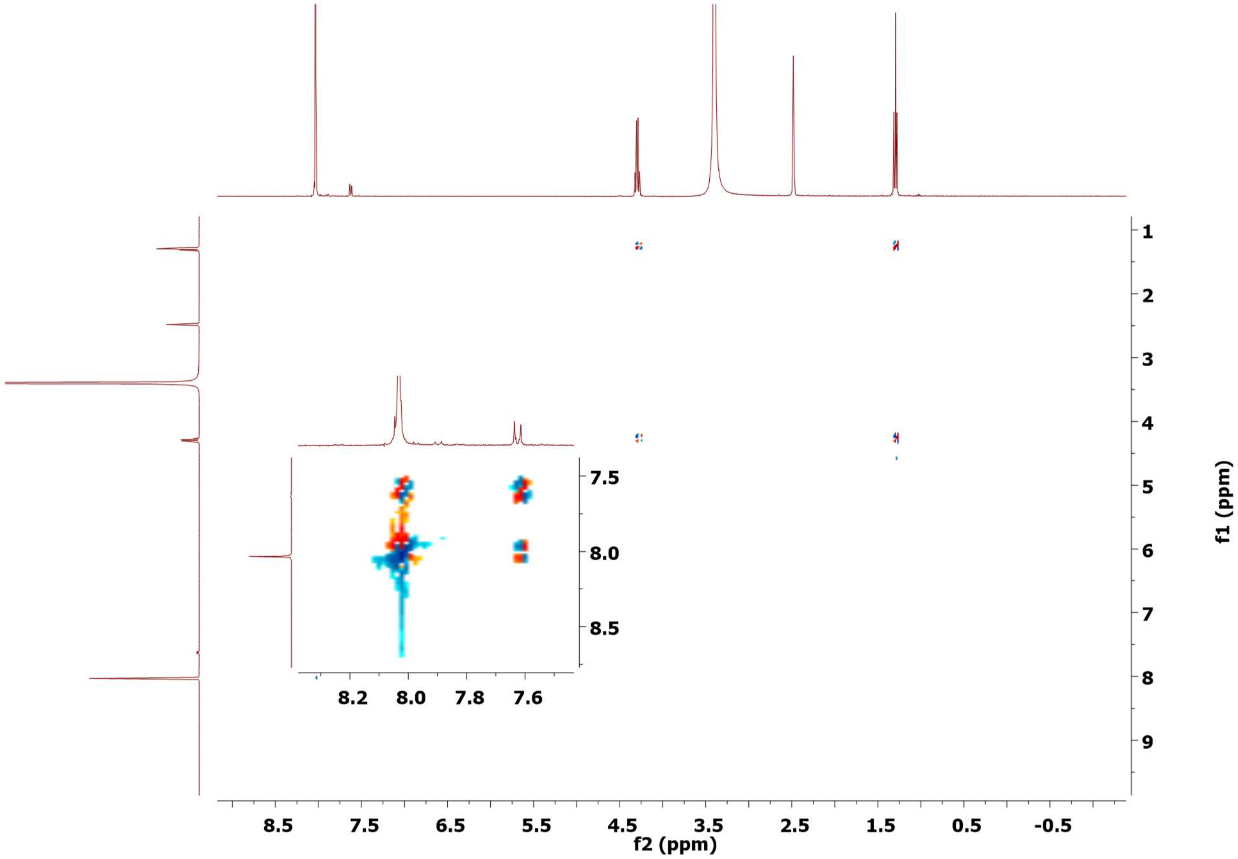

^{1}H-^{13}C-gHSQC NMR (DMSO-d6) spectrum of ethyl 4-((cyanocarbonothioyl)amino)benzoate (1:0.09 tautomeric ratio) (1n)

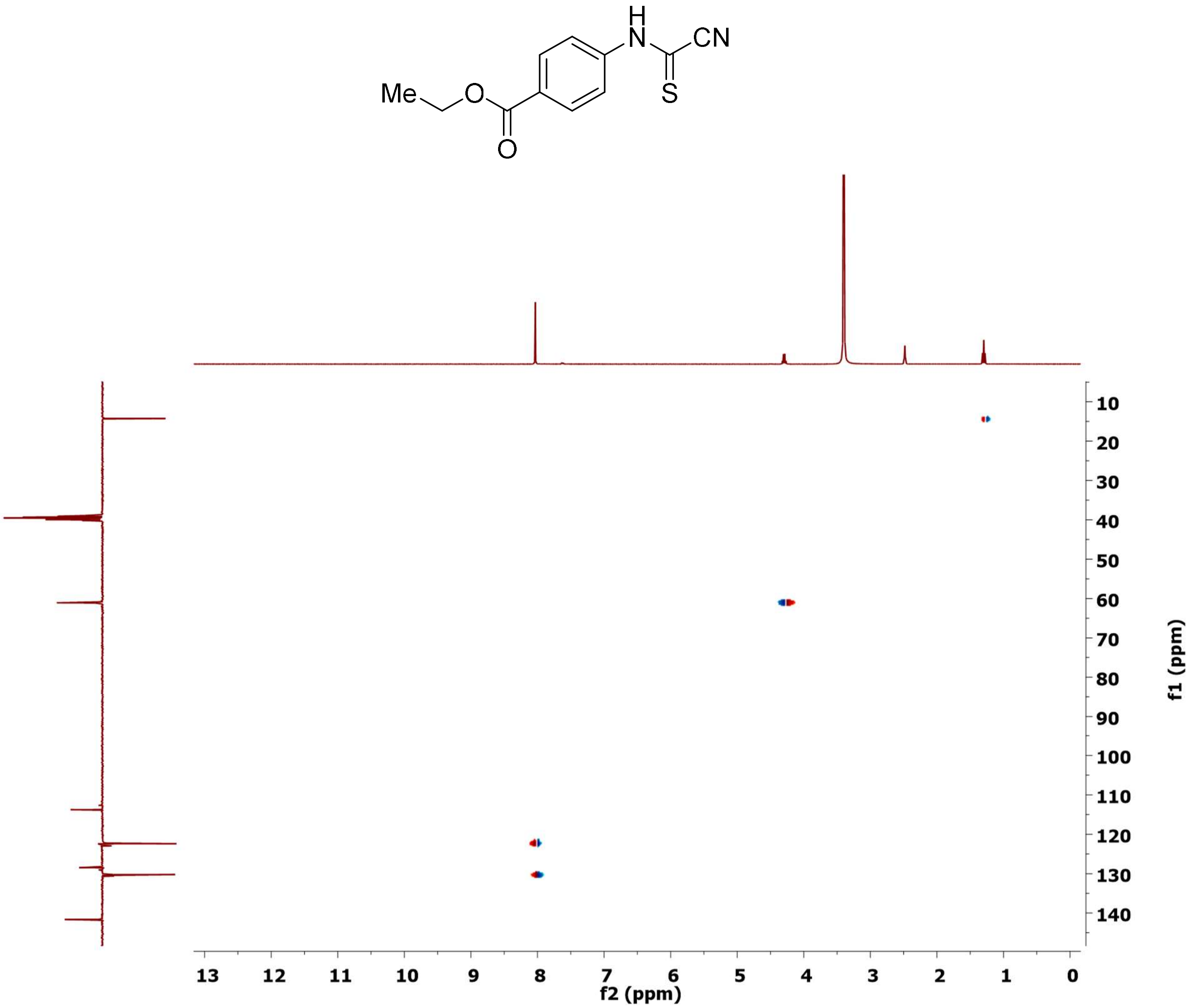

^{1}H-^{13}C-gHMBC NMR (DMSO-d6) spectrum of ethyl 4-((cyanocarbonothioyl)amino)benzoate (1:0.09 tautomeric ratio) (1n)

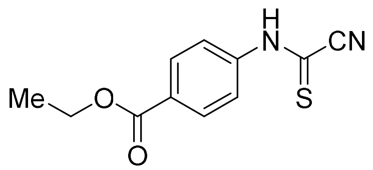

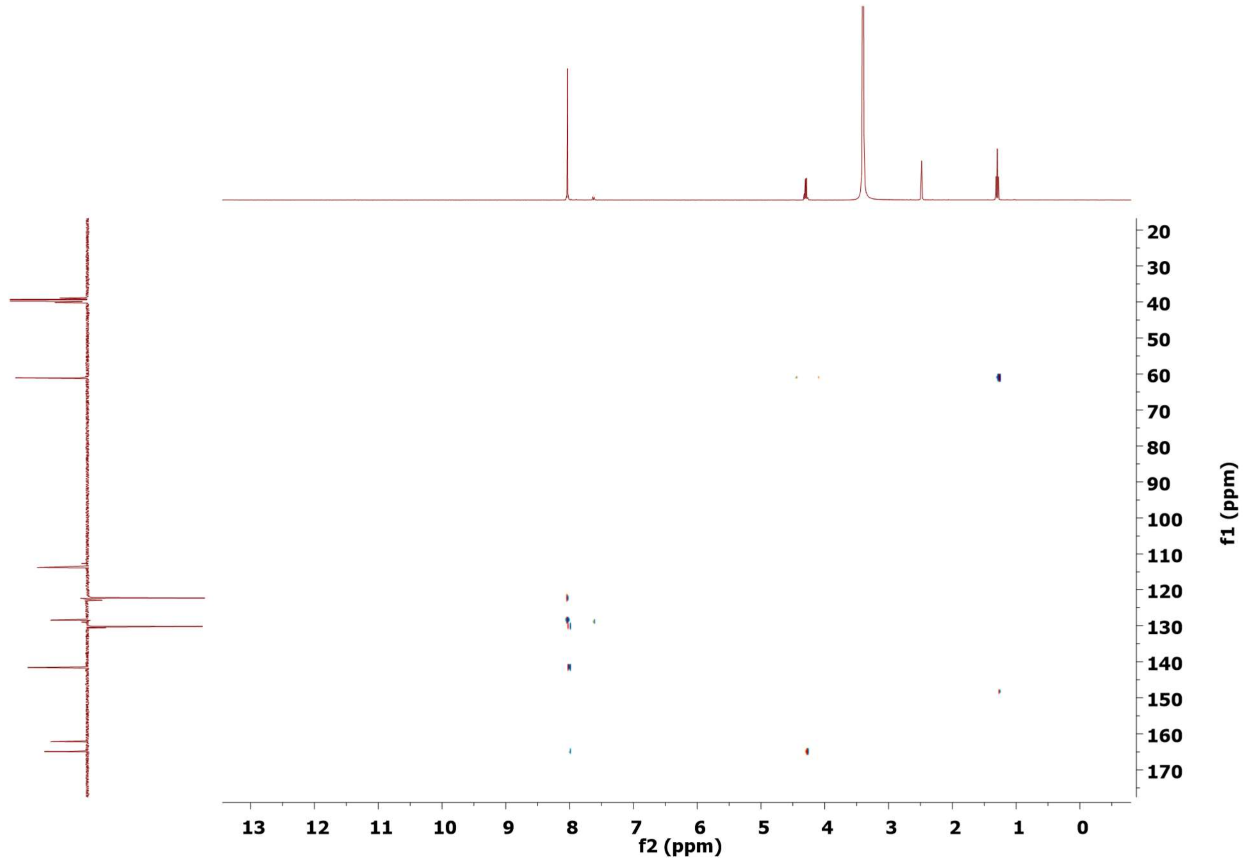

^1H NMR ($CDCl_3$) spectrum of (4-(trifluoromethyl)phenyl)carbamothioyl cyanide (1:0.37 tautomeric ratio) (1o)

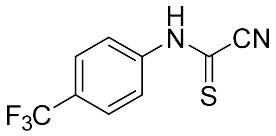

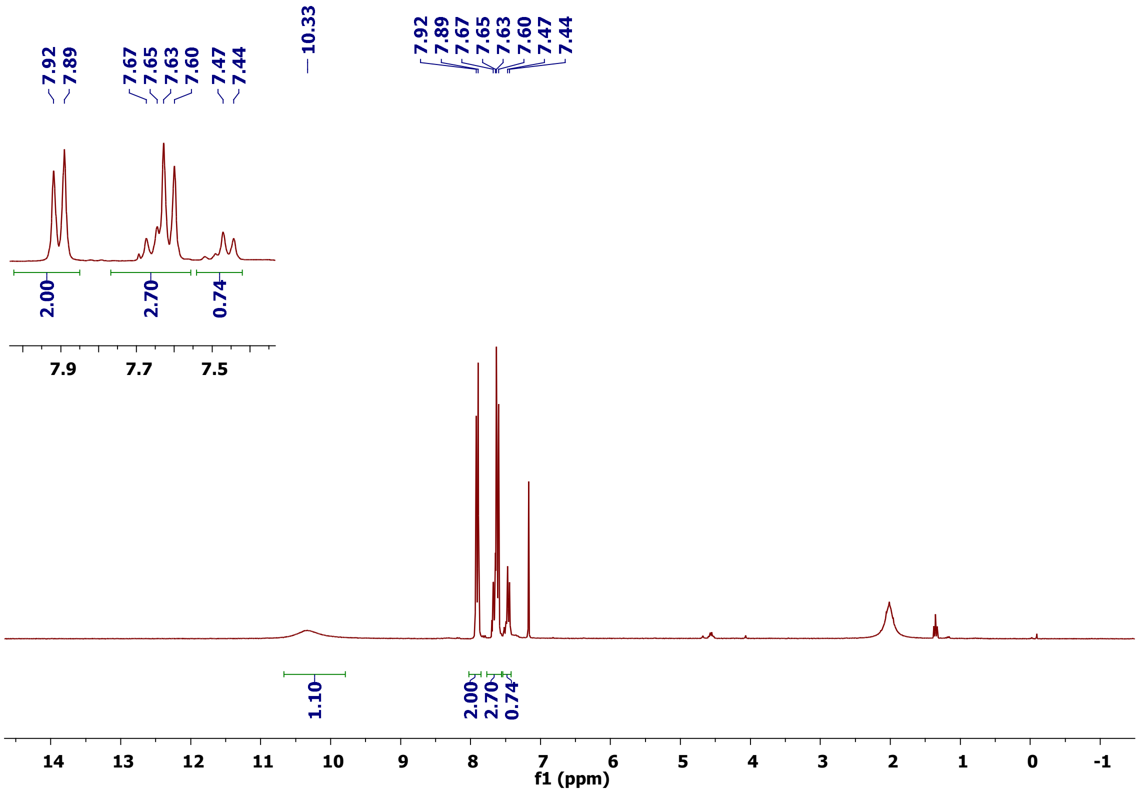

^{13}C-DEPT 135 NMR ($CDCl_3$) spectrum of (4-(trifluoromethyl)phenyl)carbamothioyl cyanide (1:0.37 tautomeric ratio) (1o)

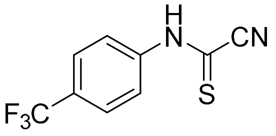

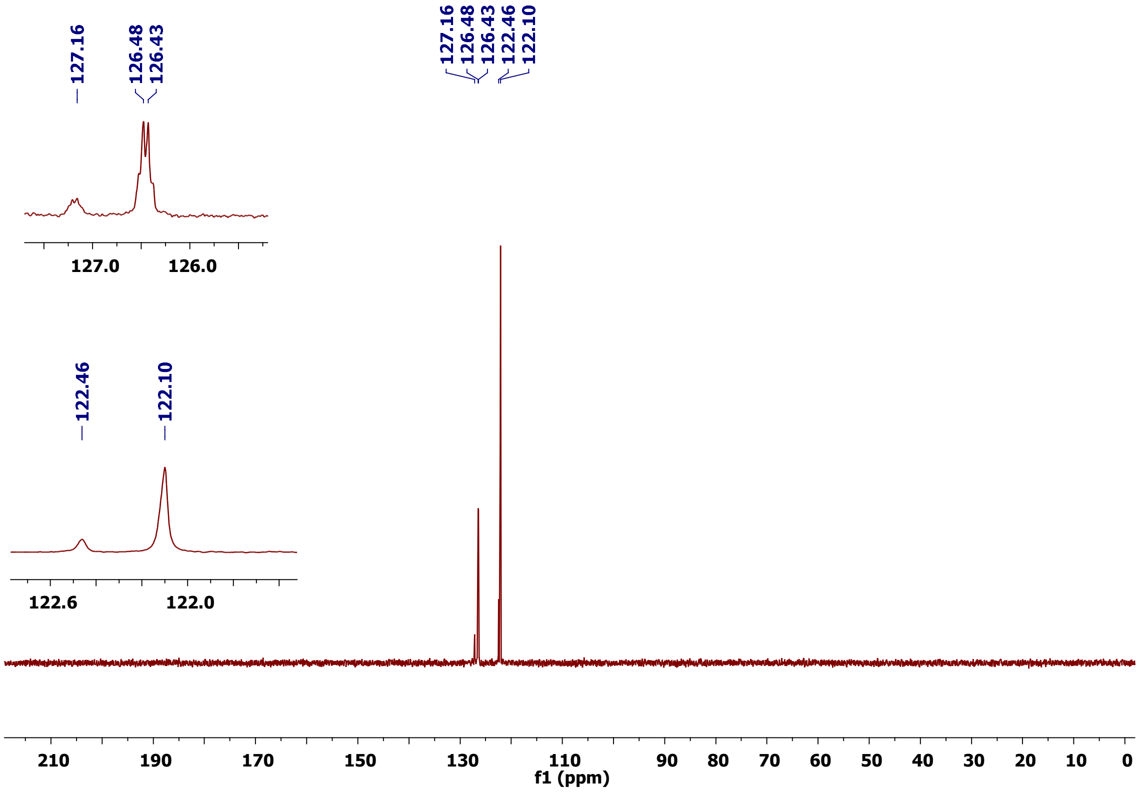

^{13}C NMR ($CDCl_3$) spectrum of (4-(trifluoromethyl)phenyl)carbamothioyl cyanide (1:0.37 tautomeric ratio) (1o)

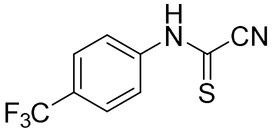

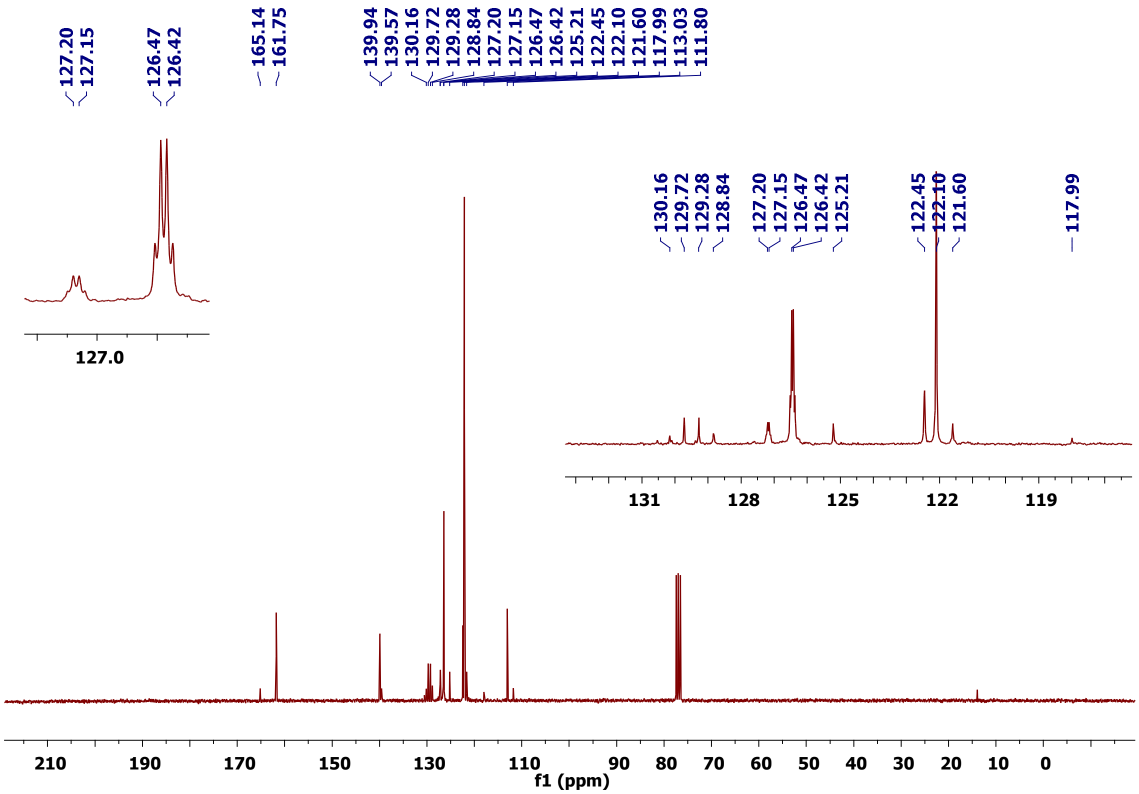

^{1}H NMR (DMSO-d6) spectrum of (4-ethylphenyl)carbamothioyl cyanide (1p)

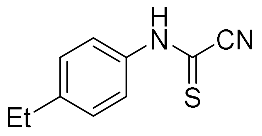

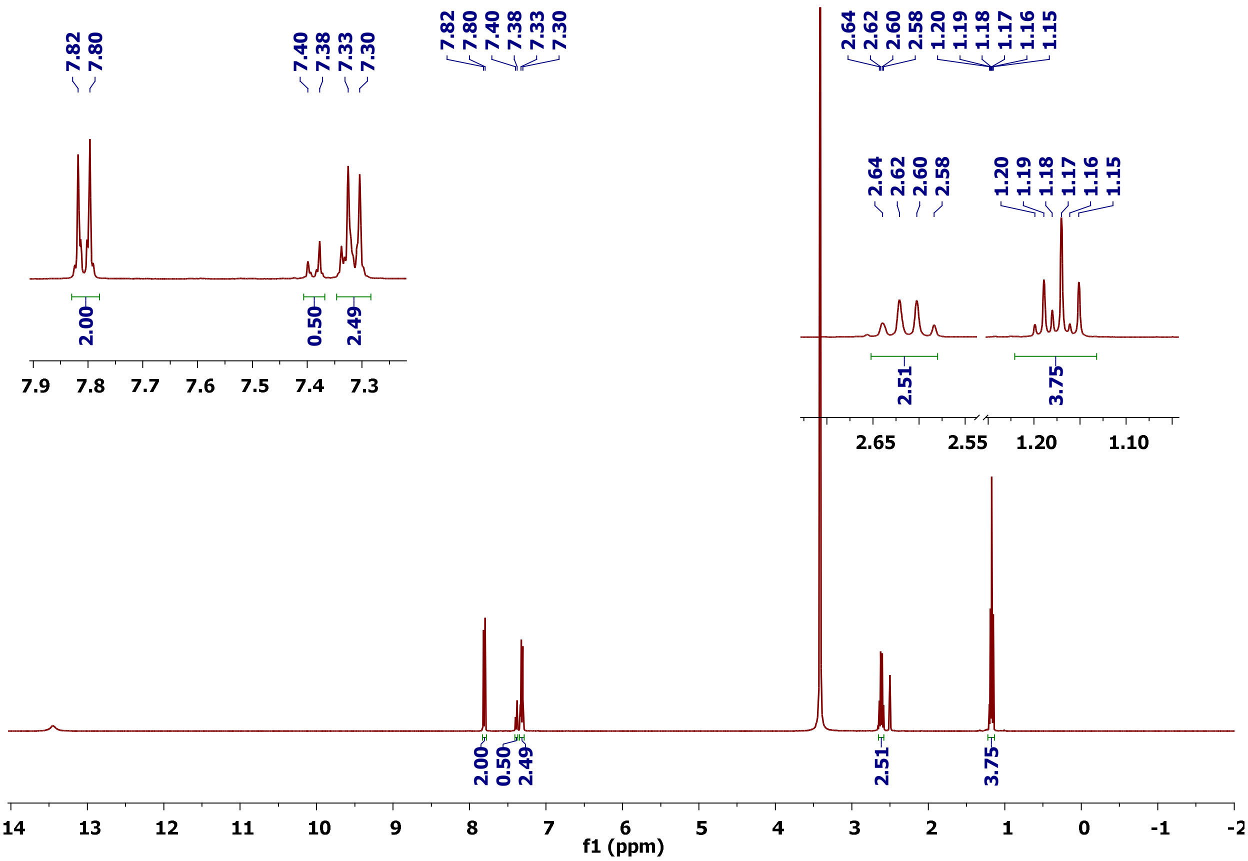

^{13}C NMR (DMSO-d6) spectrum of (4-ethylphenyl)carbamothioyl cyanide (1p)

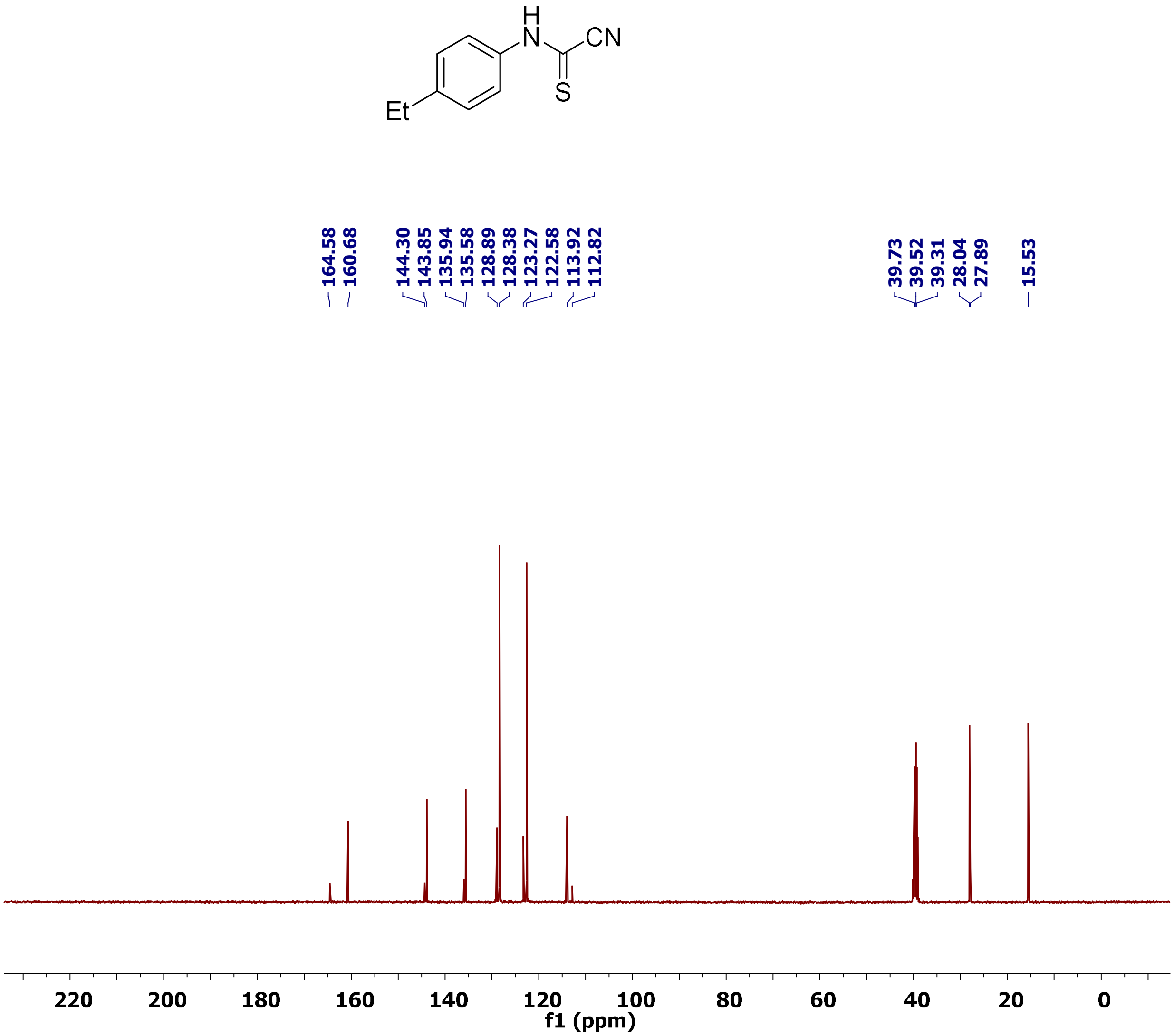

^{13}C CRAPT NMR (DMSO-d6) spectrum of (4-ethylphenyl)carbamothioyl cyanide (1p)

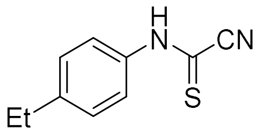

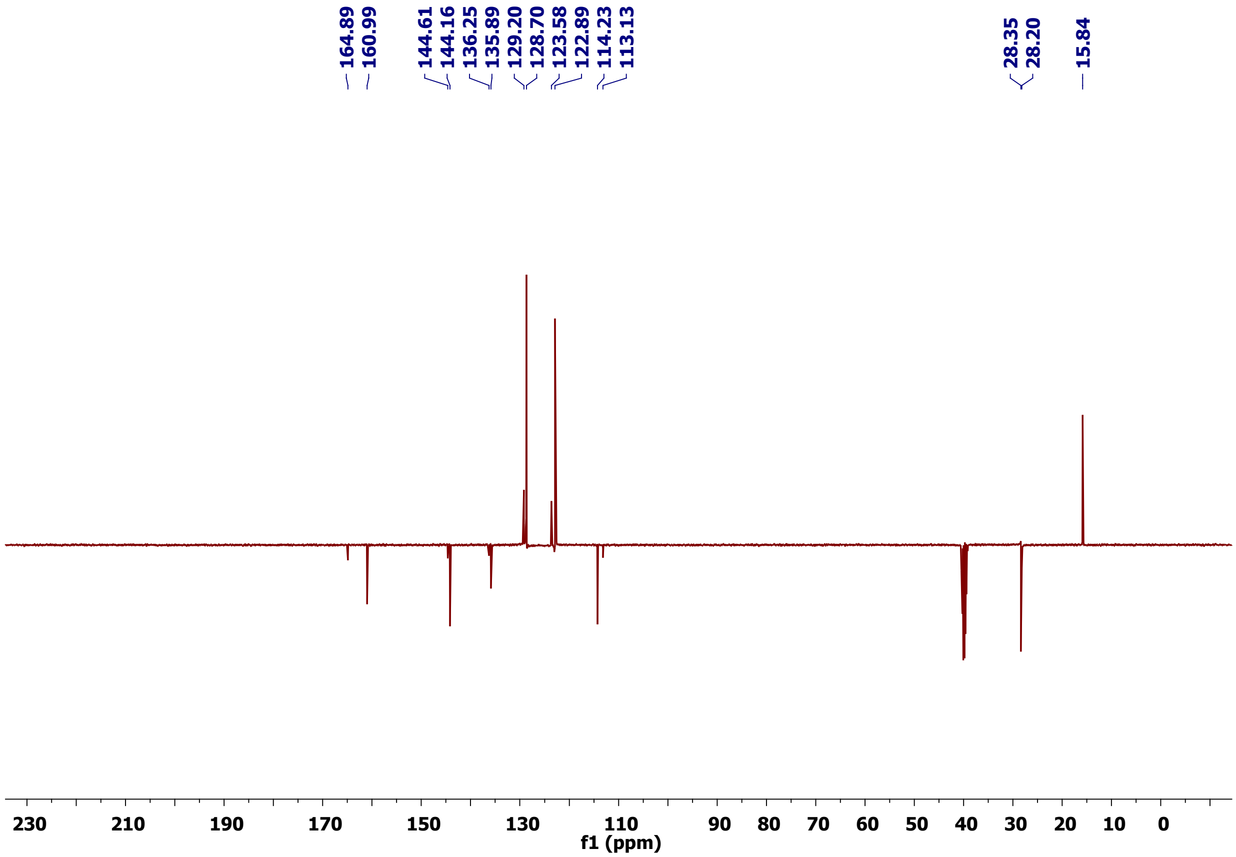

^{1}H-^{1}H gDQCOSY NMR (DMSO-d6) spectrum of (4-ethylphenyl)carbamothioyl cyanide (1p)

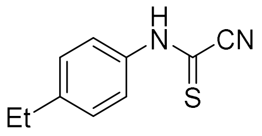

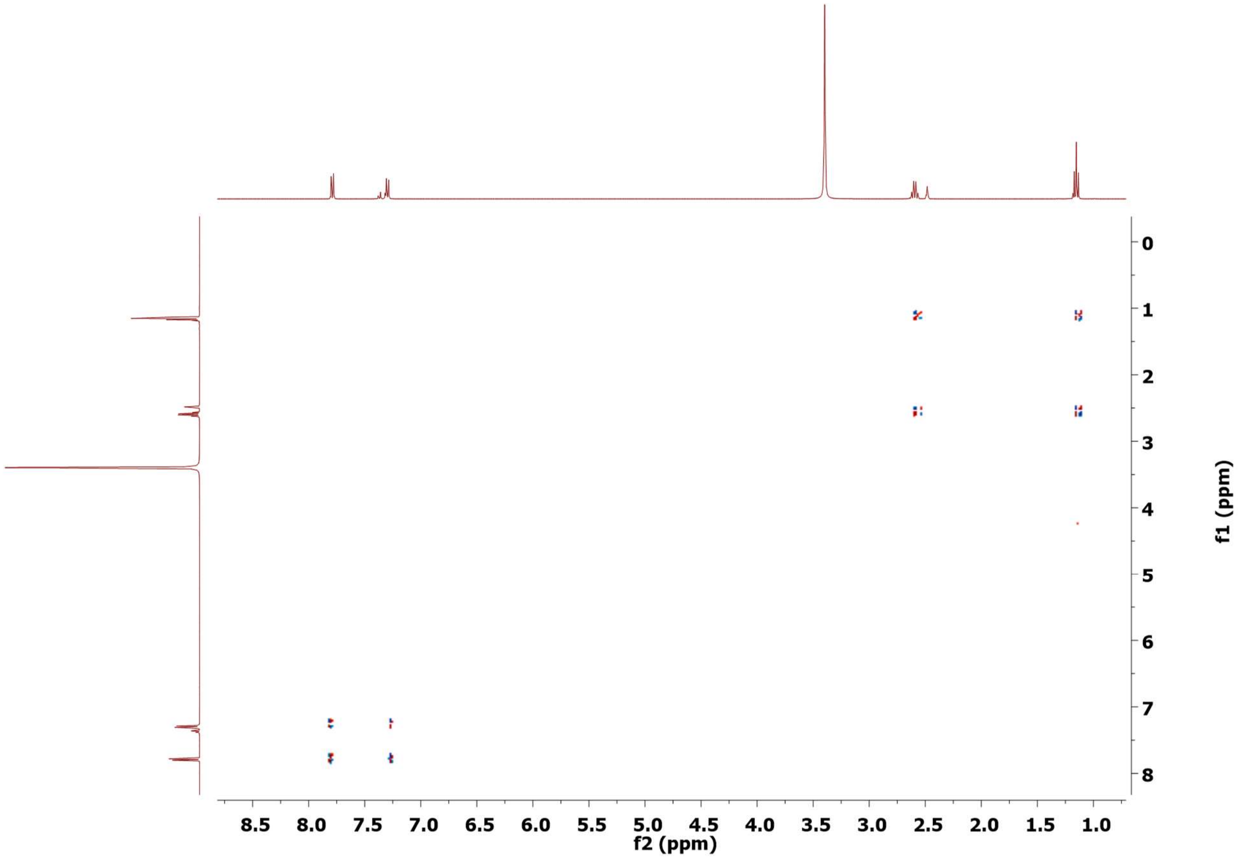

^{1}H-^{13}C-gHSQC NMR (DMSO-d6) spectrum of (4-ethylphenyl)carbamothioyl cyanide (1p)

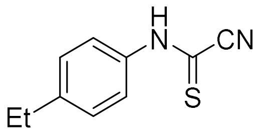

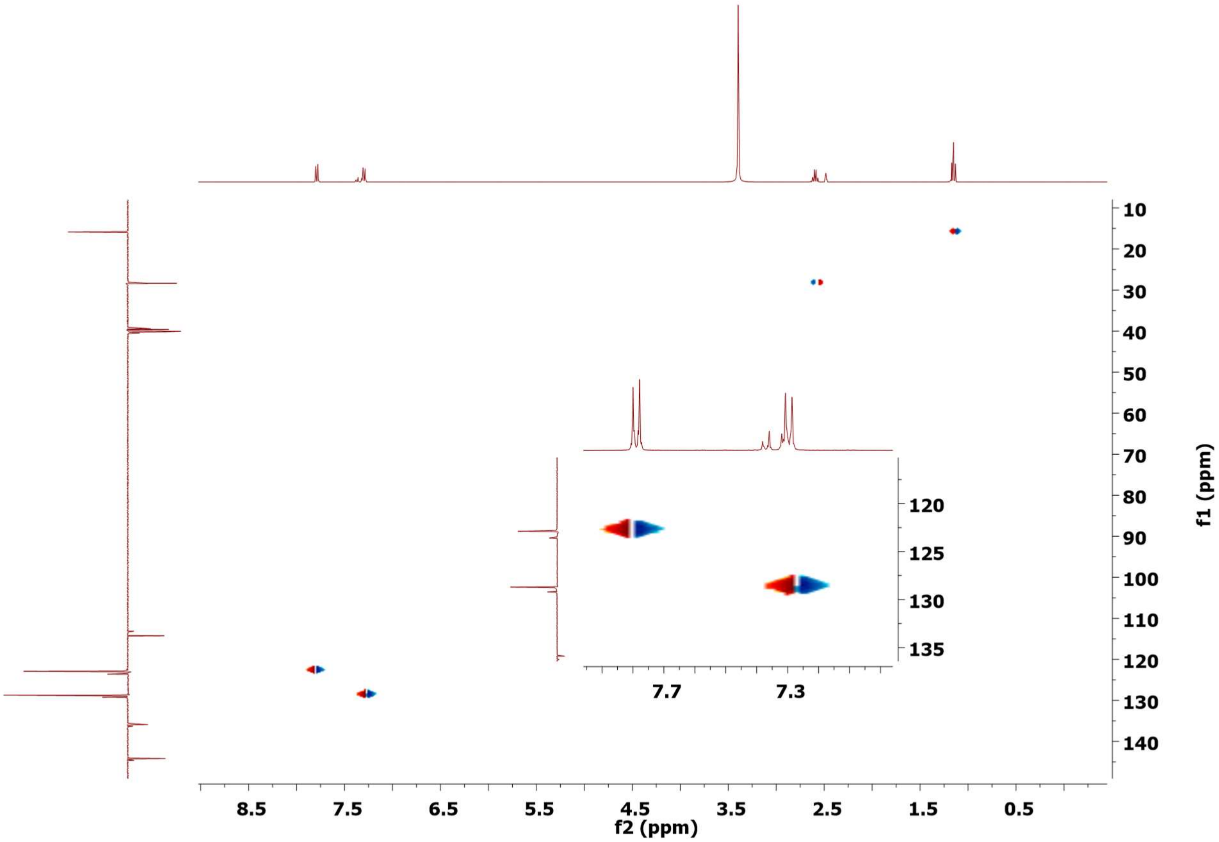

^{1}H-^{13}C-gHMBC NMR (DMSO-d6) spectrum of (4-ethylphenyl)carbamothioyl cyanide (1p)

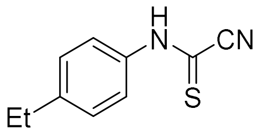

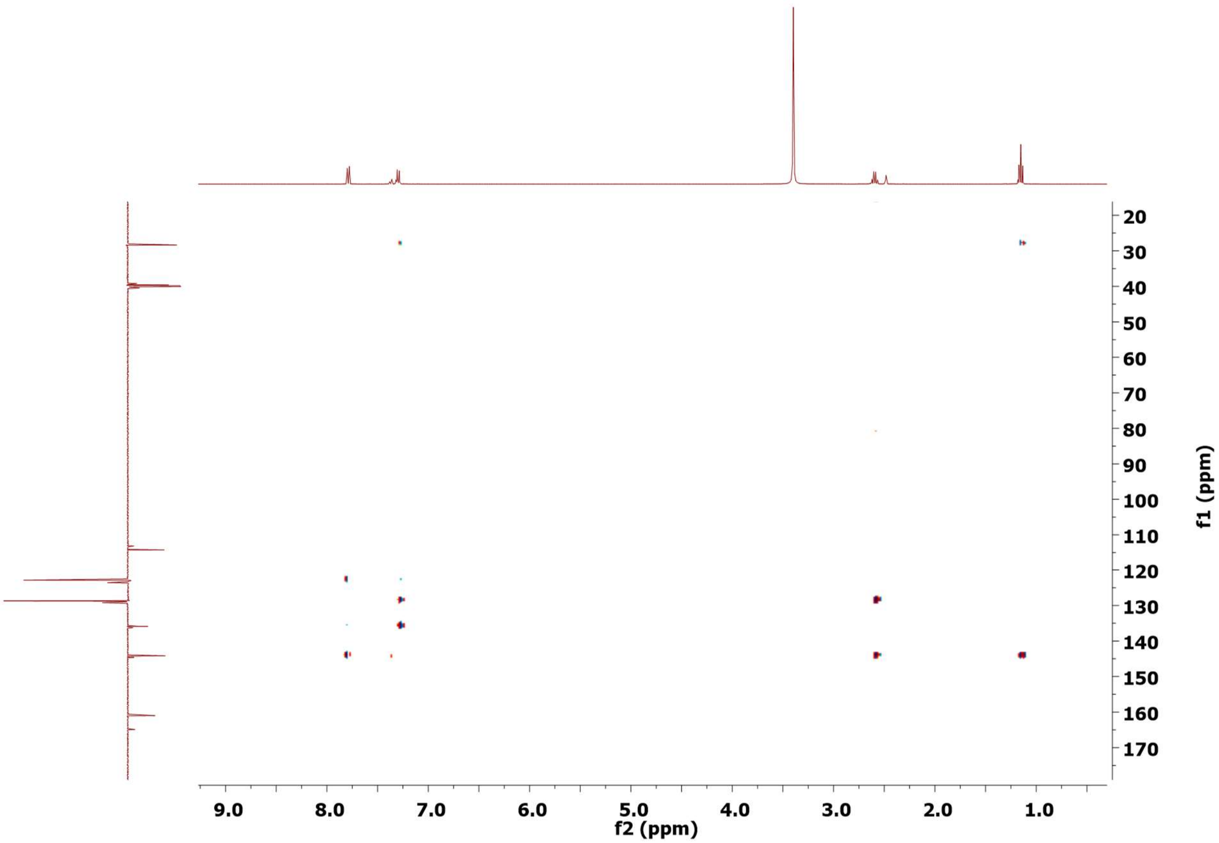

^{1}H NMR (DMSO-d6) spectrum of (4-iodophenyl)carbamothioyl cyanide (1q)

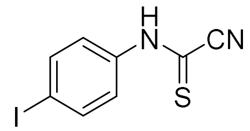

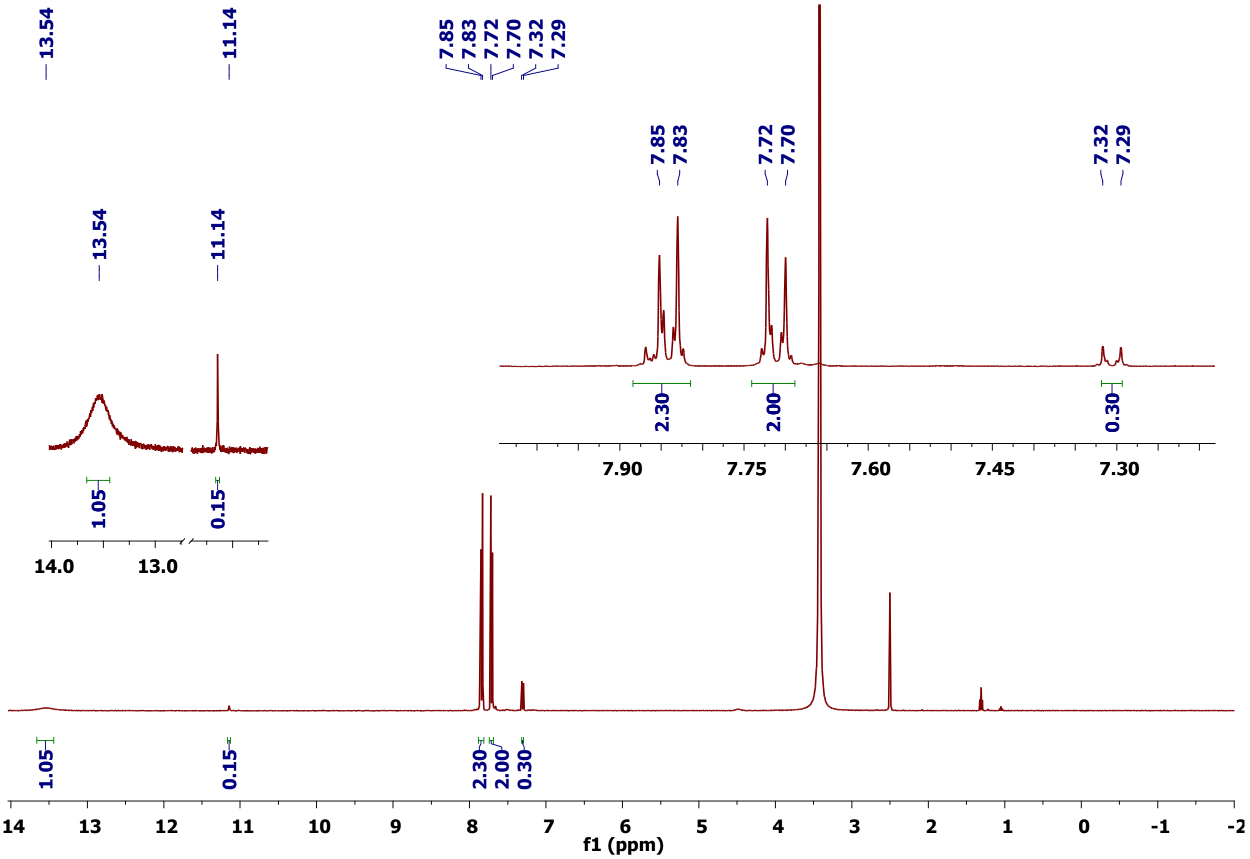

^{13}C NMR (DMSO-d6) spectrum of (4-iodophenyl)carbamothioyl cyanide (1q)

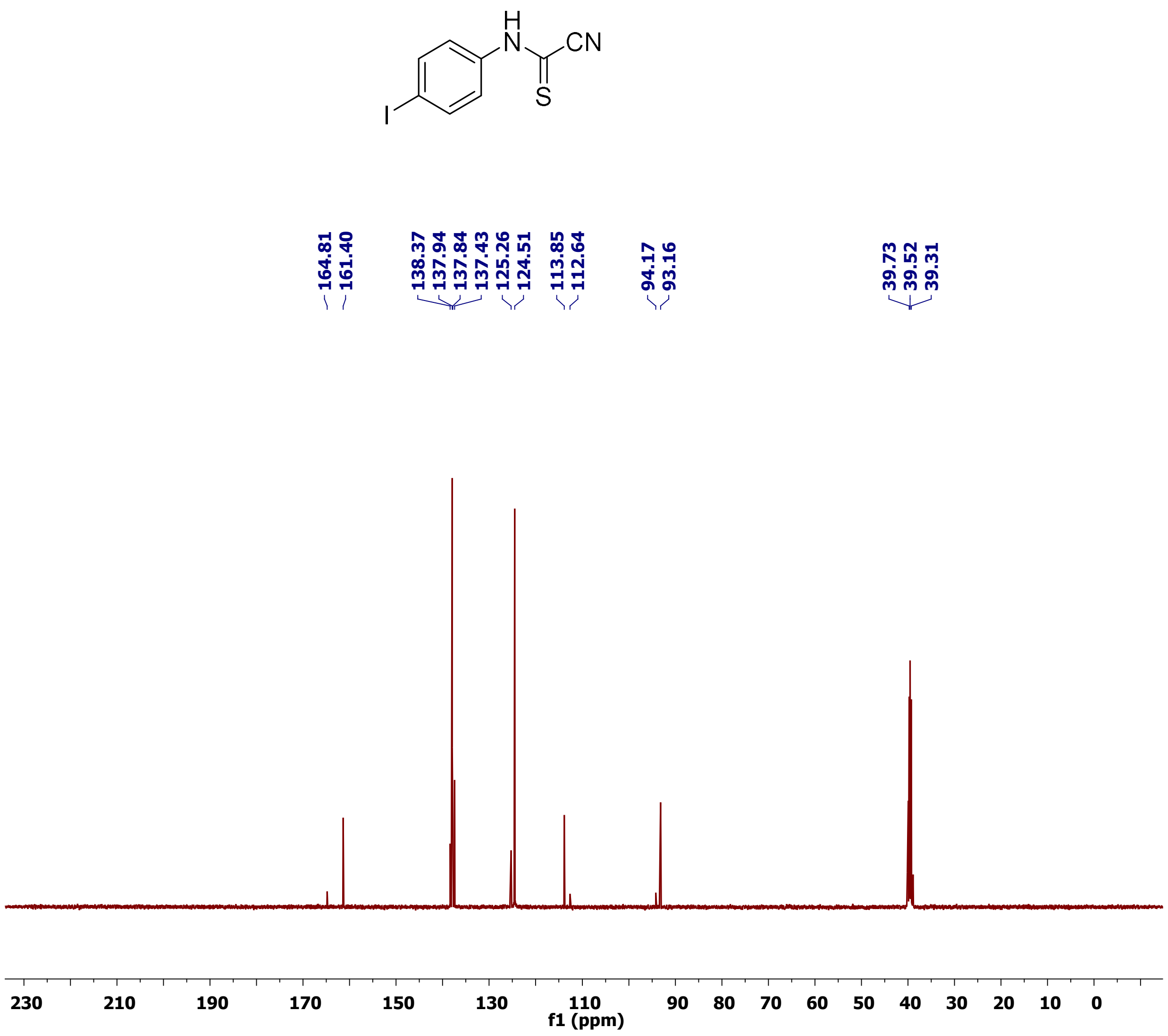

^{13}C CRAPT NMR (DMSO-d6) spectrum of (4-iodophenyl)carbamothioyl cyanide (1q)

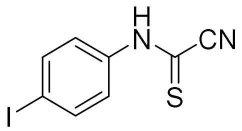

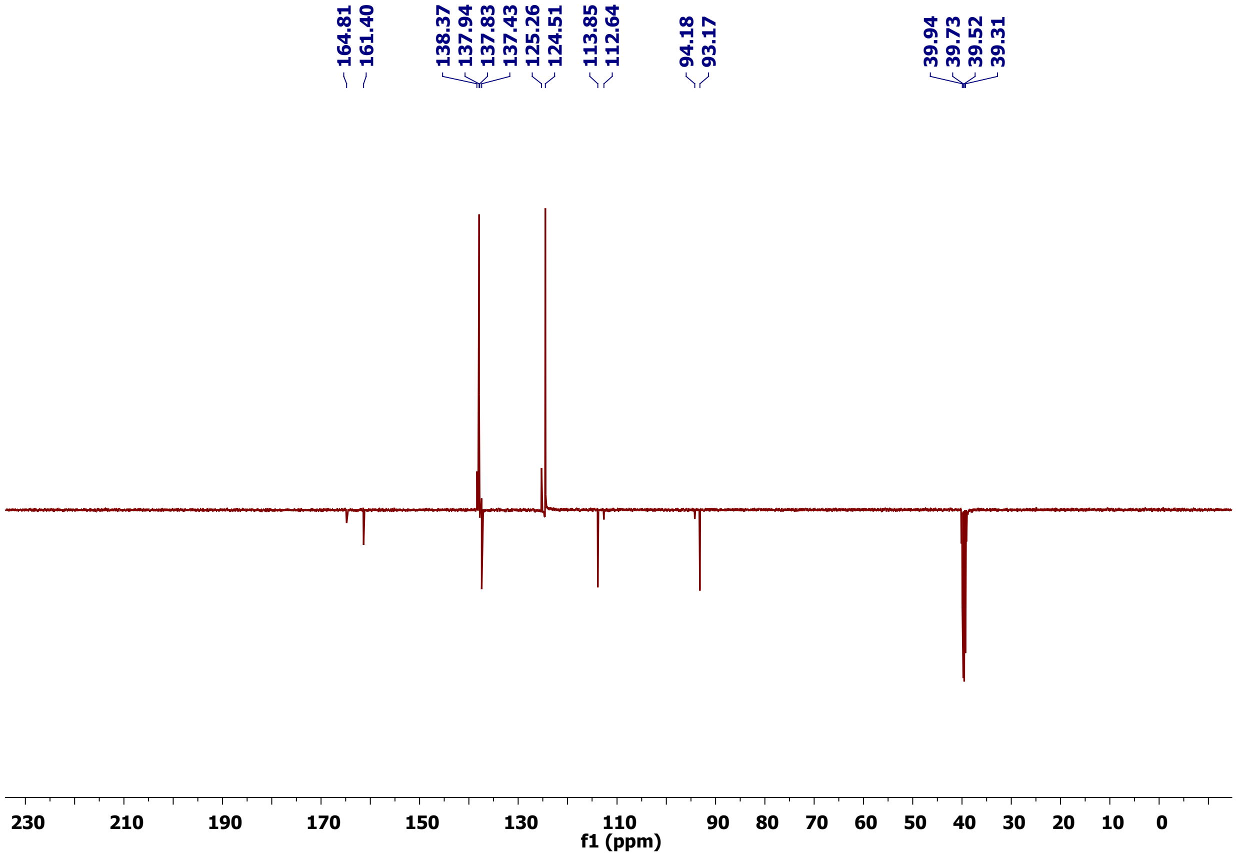

^{1}H-^{1}H gDQCOSY NMR (DMSO-d6) spectrum of (4-iodophenyl)carbamothioyl cyanide (1q)

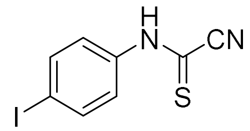

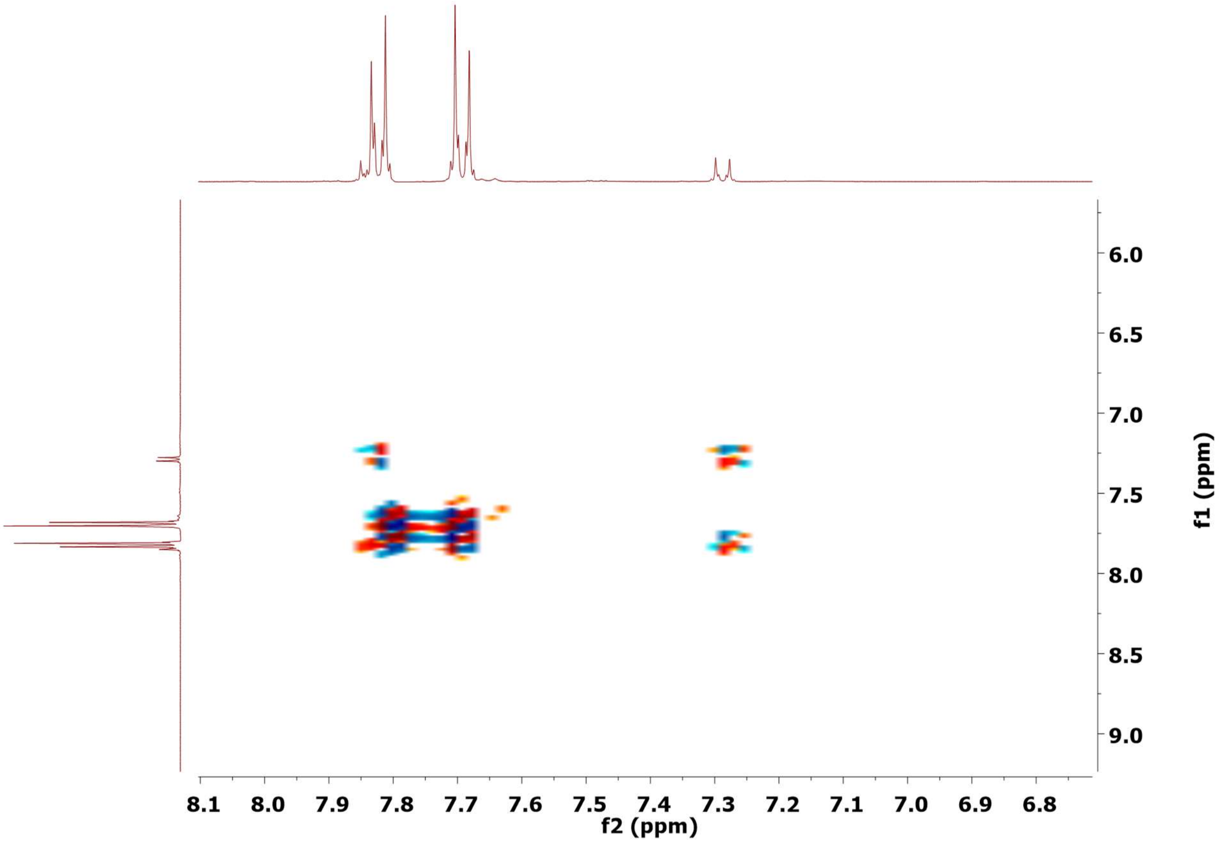

^{1}H-^{13}C-gHSQC NMR (DMSO-d6) spectrum of (4-iodophenyl)carbamothioyl cyanide (1q)

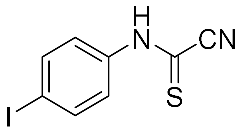

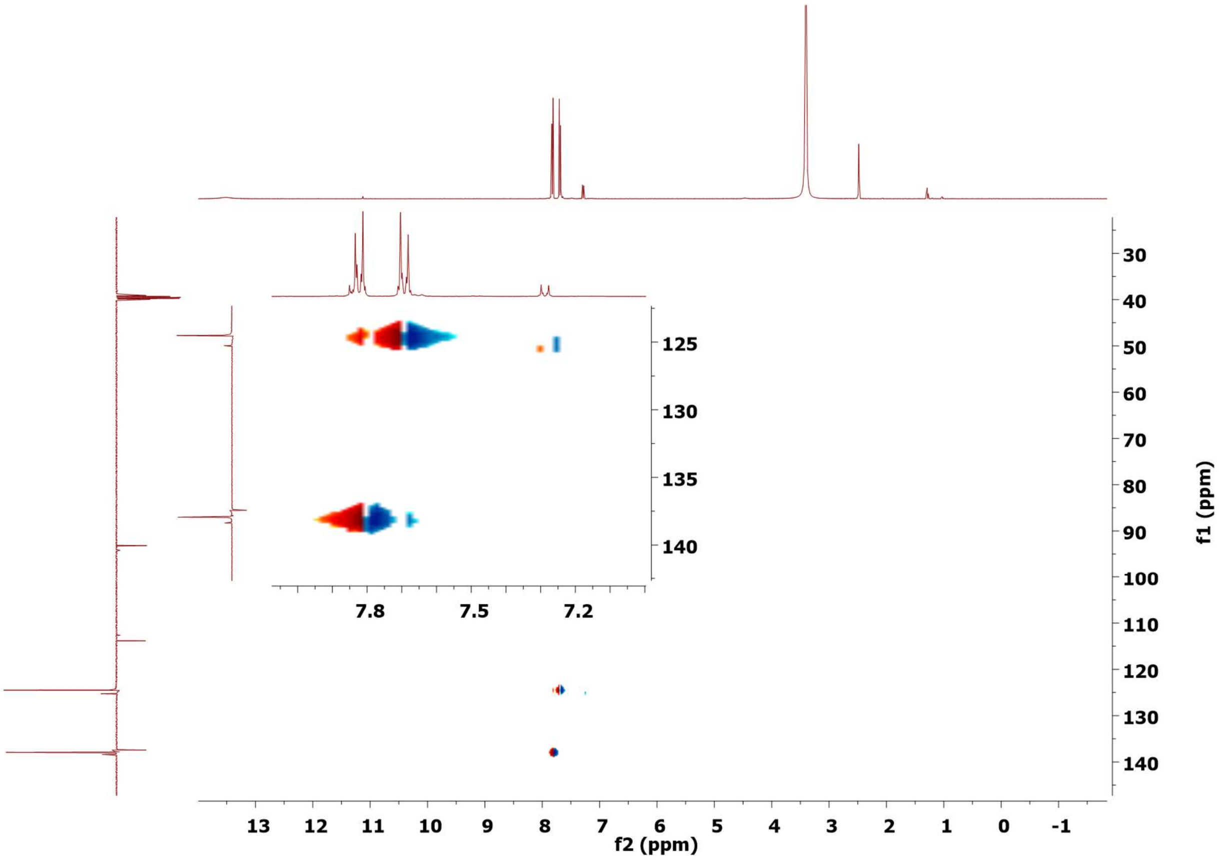

^1H-^{13}C-gHMBC NMR (DMSO-d6) spectrum of (4-iodophenyl)carbamothioyl cyanide (1q)

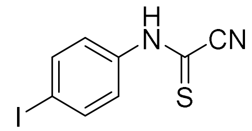

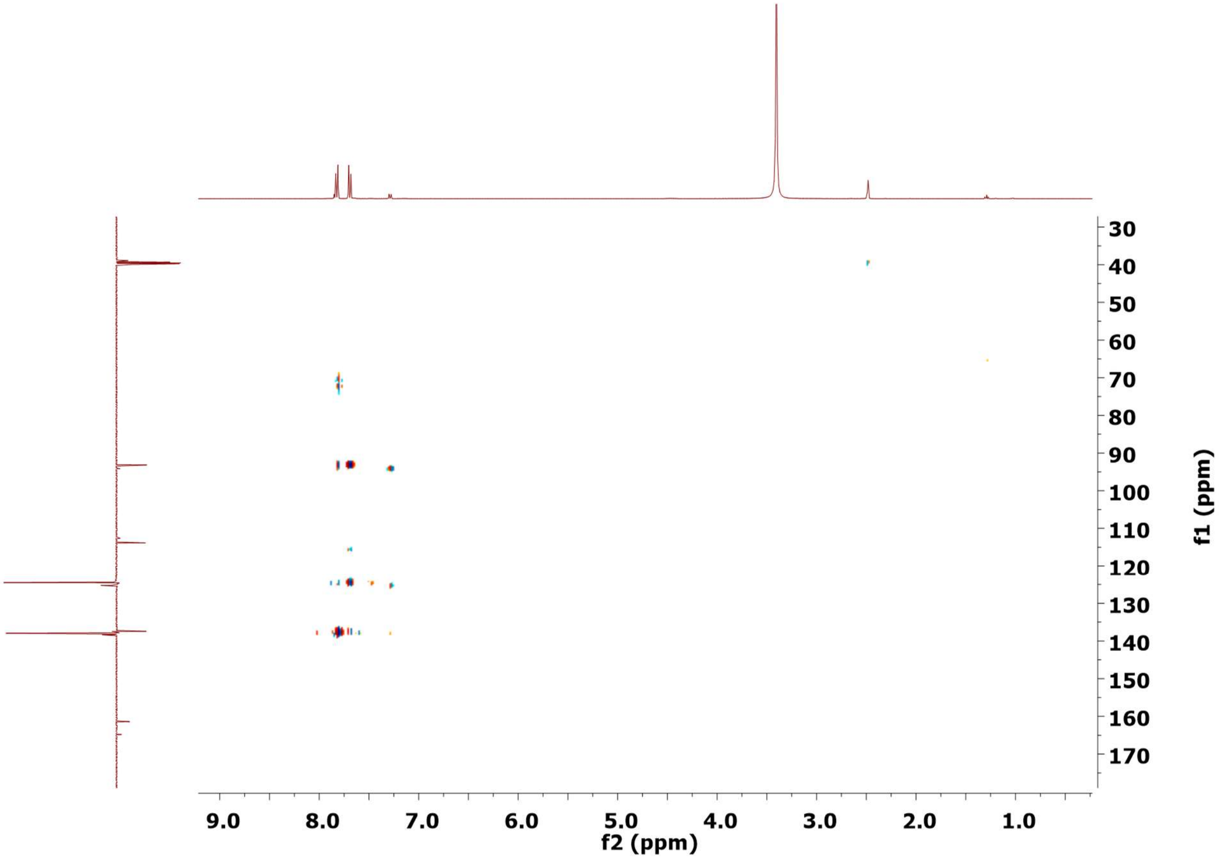

^{1}H NMR ($CDCl_3$) spectrum of (3-chlorophenyl)carbamothioyl cyanide (1:0.28 tautomeric ratio) (1r)

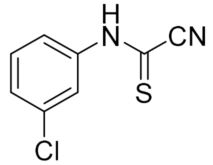

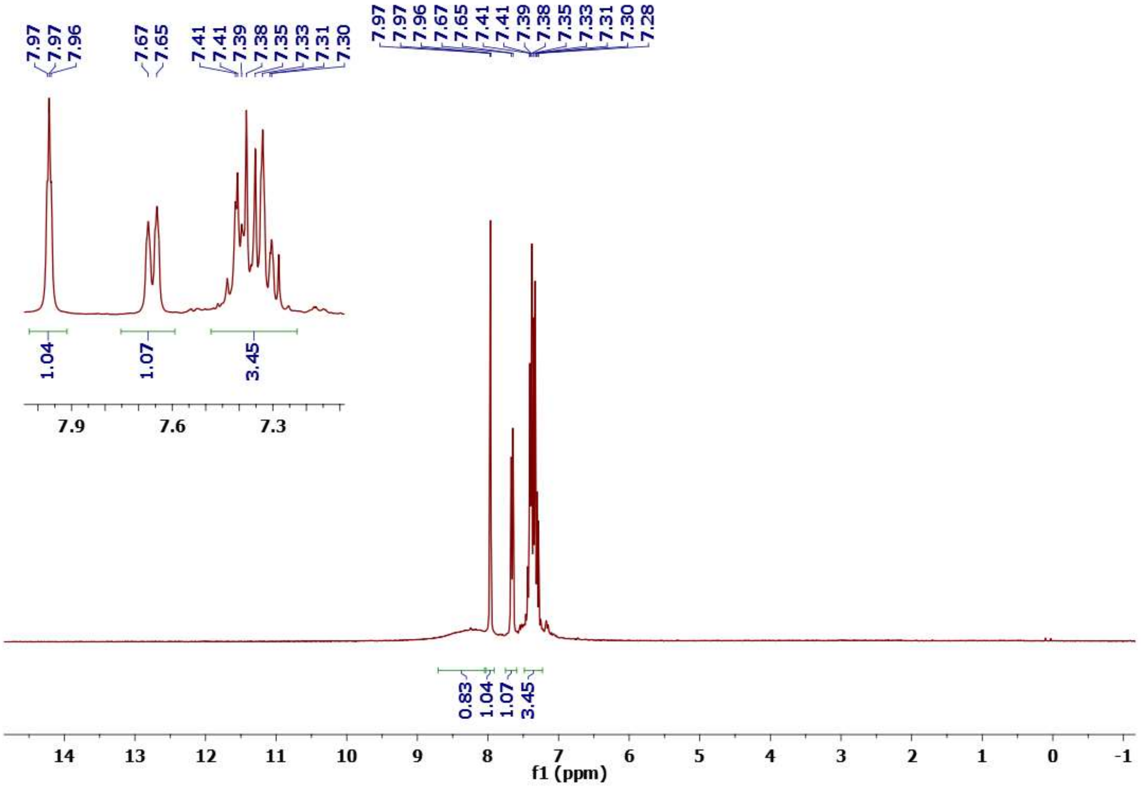

^{13}C-DEPT 90 NMR ($CDCl_3$) spectrum of (3-chlorophenyl)carbamothioyl cyanide (1:0.37 tautomeric ratio) (1r)

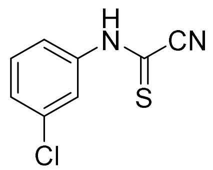

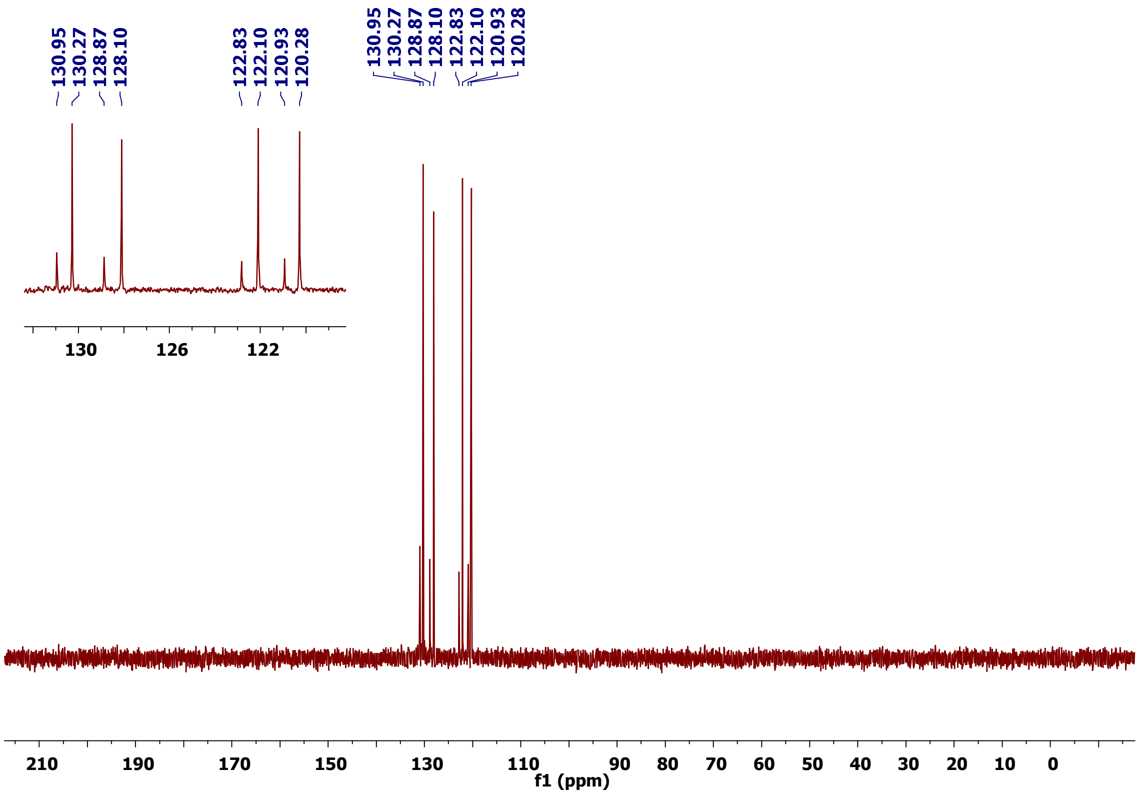

^{13}C NMR ($CDCl_3$) spectrum of (3-chlorophenyl)carbamothioyl cyanide (1:0.37 tautomeric ratio) (1r)

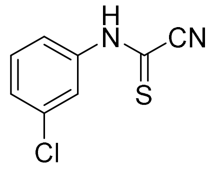

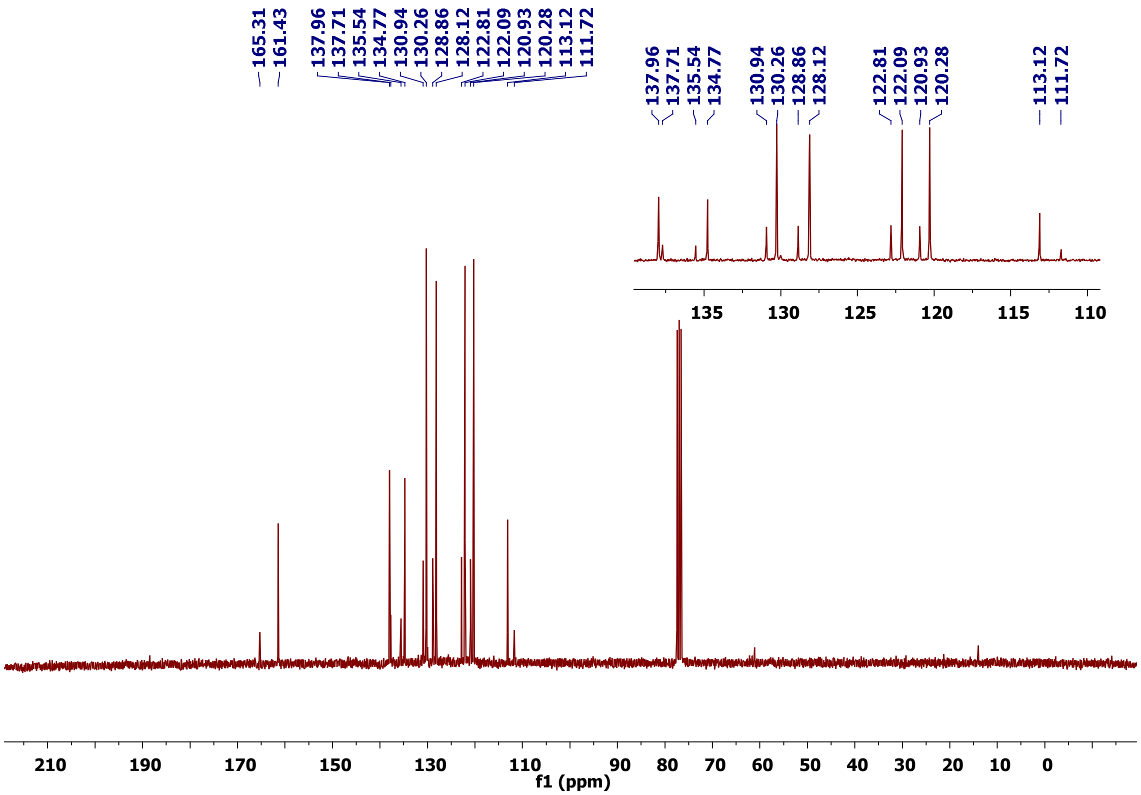

^{1}H NMR ($CDCl_3$) spectrum of (3-bromophenyl)carbamothioyl cyanide (1:0.32 tautomeric ratio) (1s)

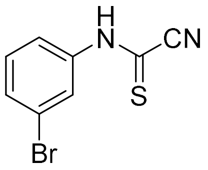

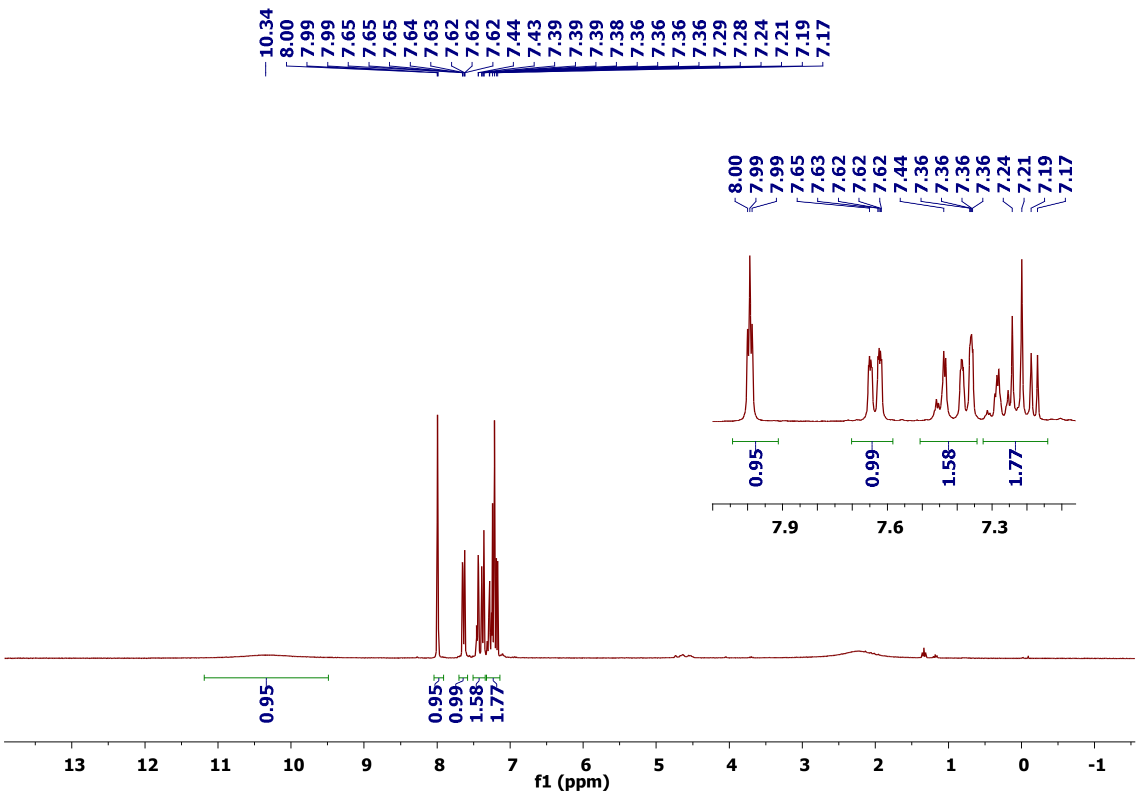

^{13}C-DEPT 135 NMR ($CDCl_3$) spectrum of (3-bromophenyl)carbamothioyl cyanide (1:0.32 tautomeric ratio) (1s)

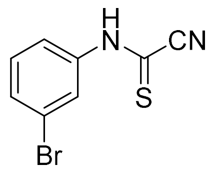

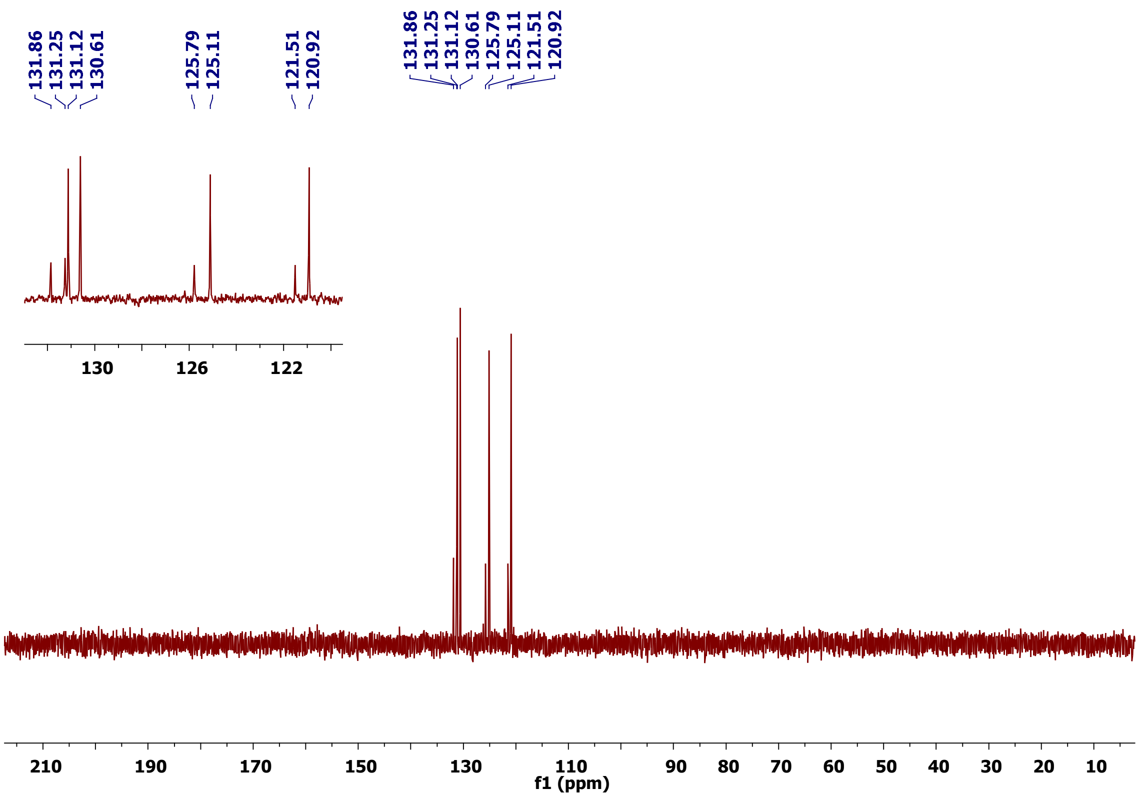

^{13}C NMR ($CDCl_3$) spectrum of (3-bromophenyl)carbamothioyl cyanide (1:0.32 tautomeric ratio) (1s)

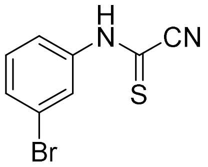

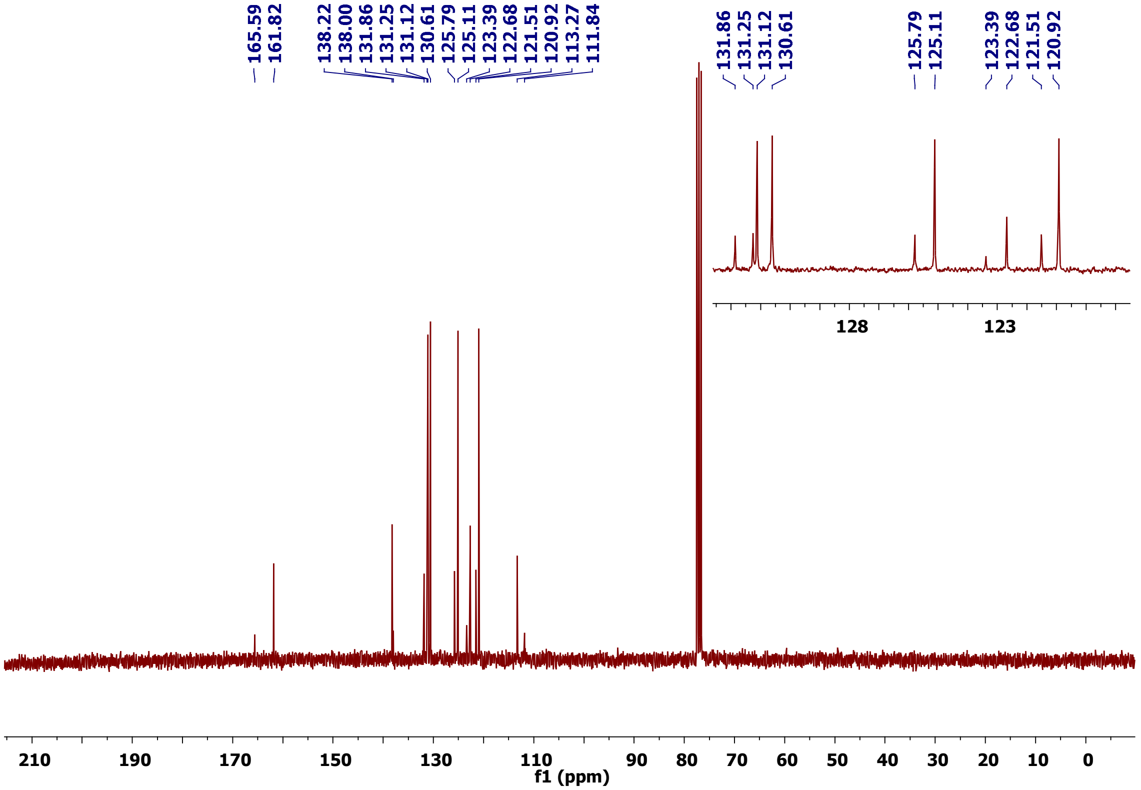

^{1}H NMR (DMSO-d6) spectrum of (3-iodophenyl)carbamothioyl cyanide (1t)

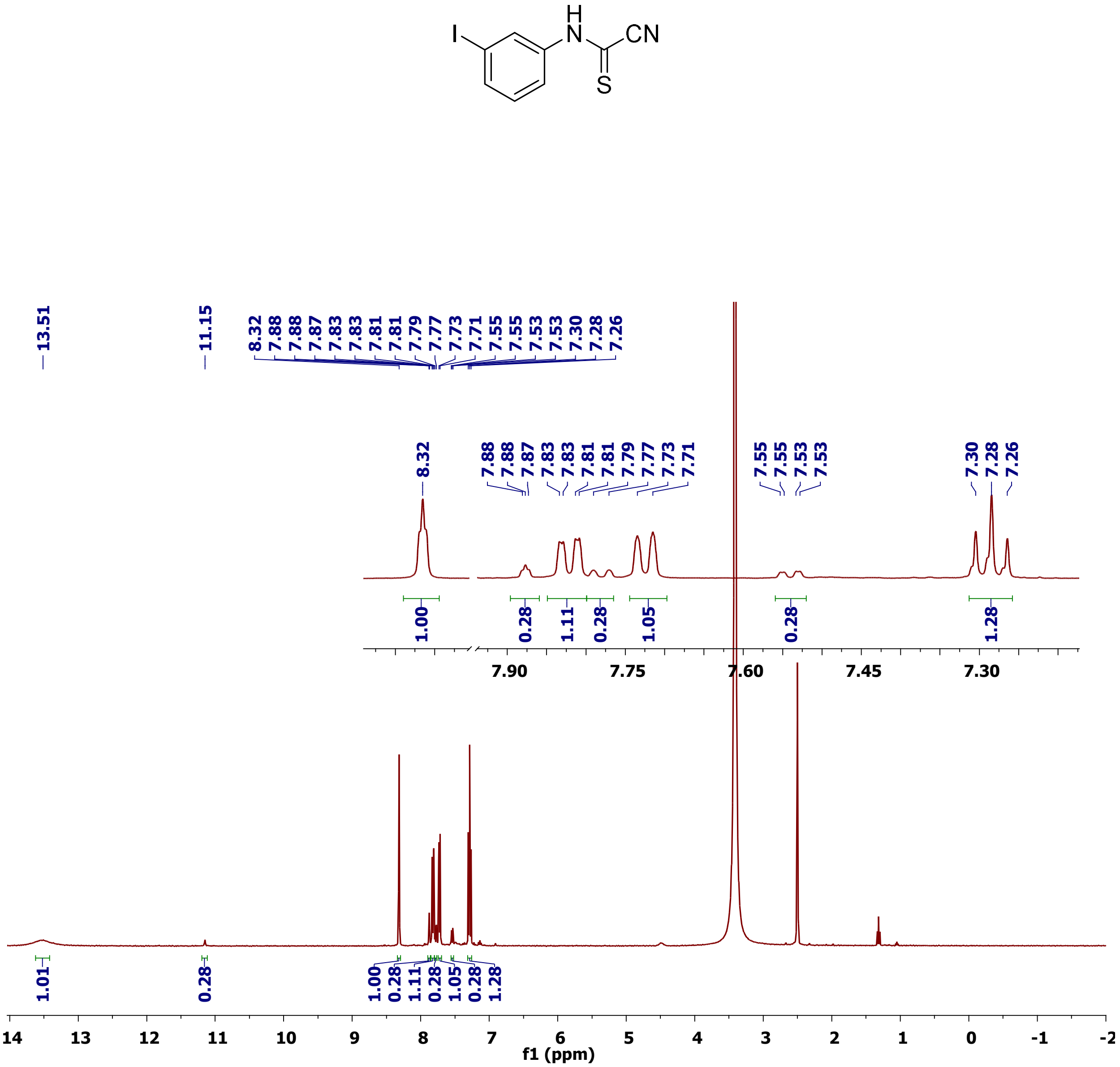

^{13}C NMR (DMSO-d6) spectrum of (3-iodophenyl)carbamothioyl cyanide (1t)

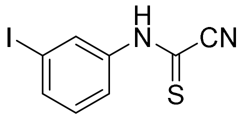

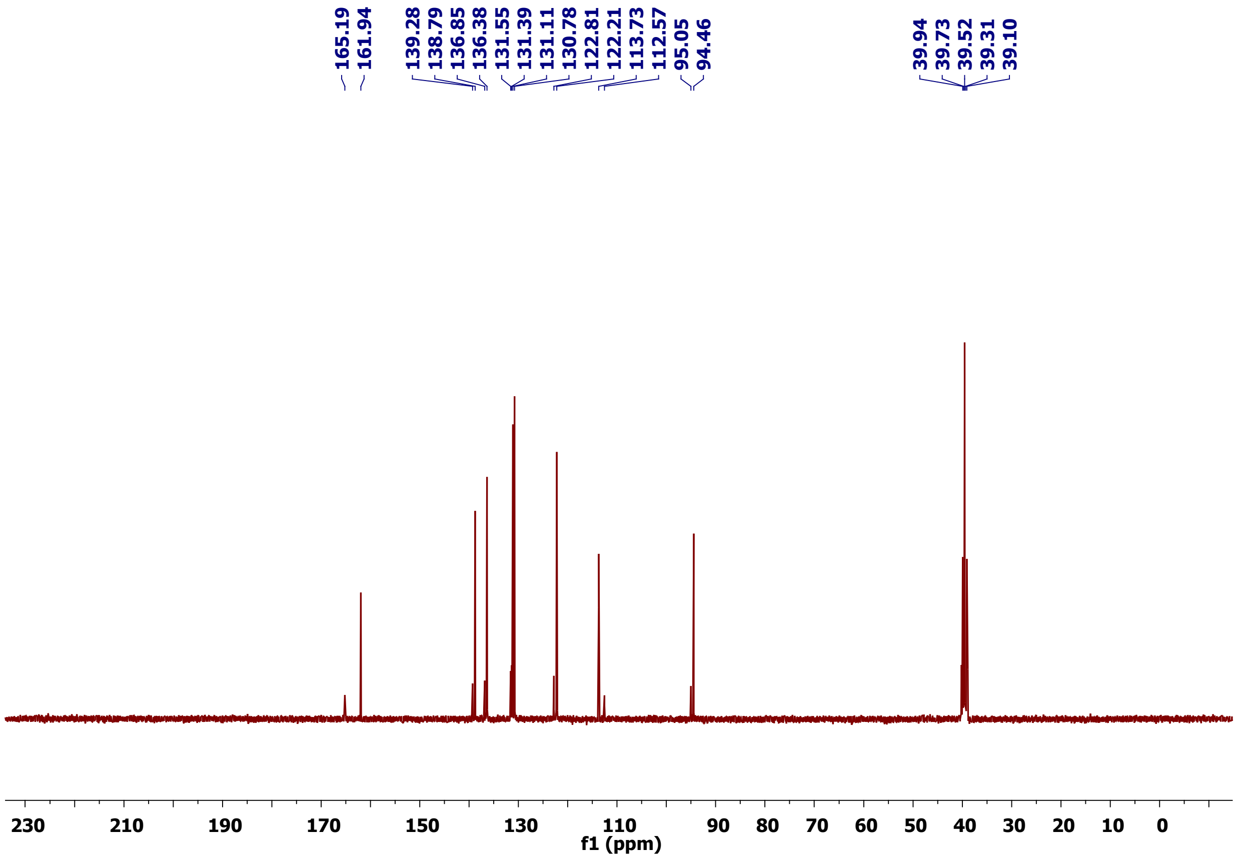

^{13}C CRAPT NMR (DMSO-d6) spectrum of (3-iodophenyl)carbamothioyl cyanide (1t)

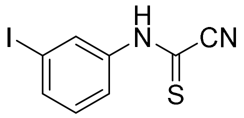

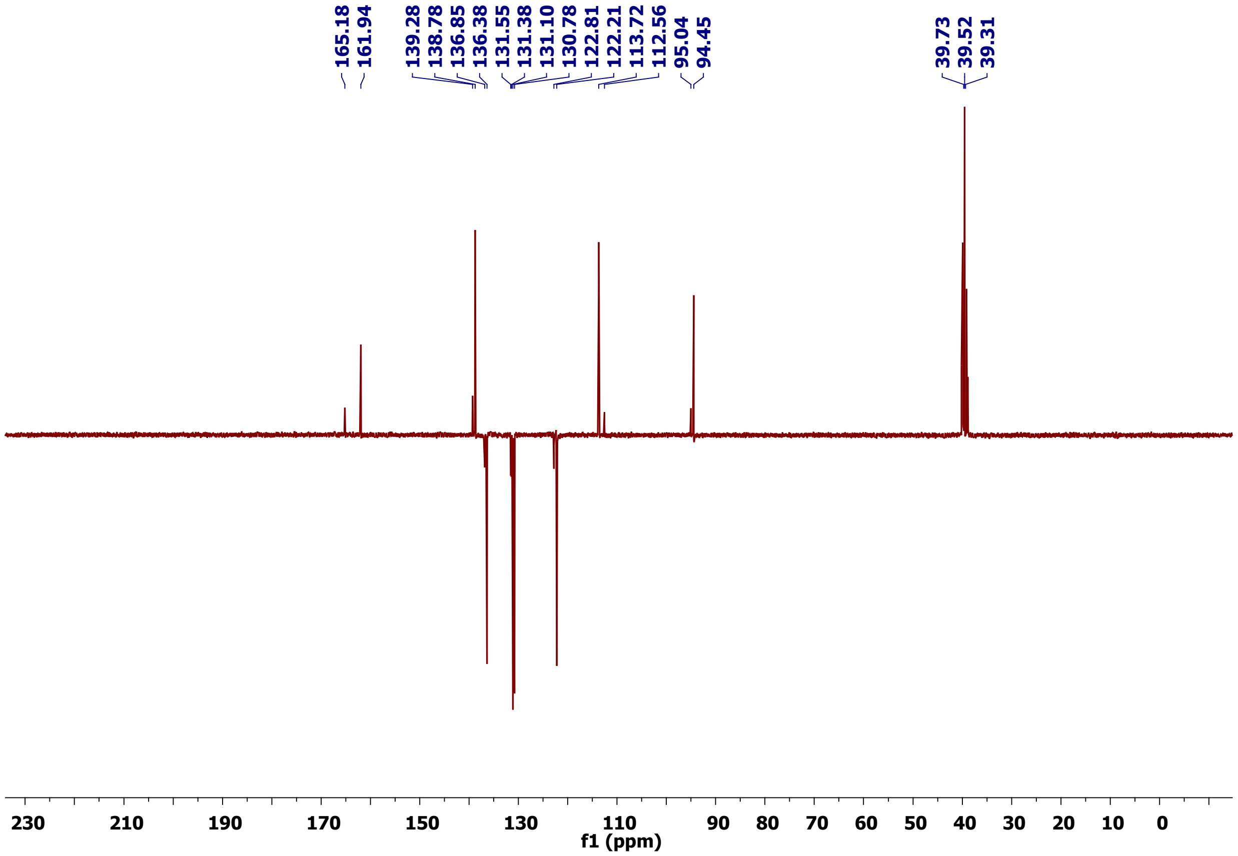

^{1}H-^{1}H gDQCOSY NMR (DMSO-d6) spectrum of (3-iodophenyl)carbamothioyl cyanide (1t)

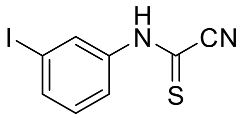

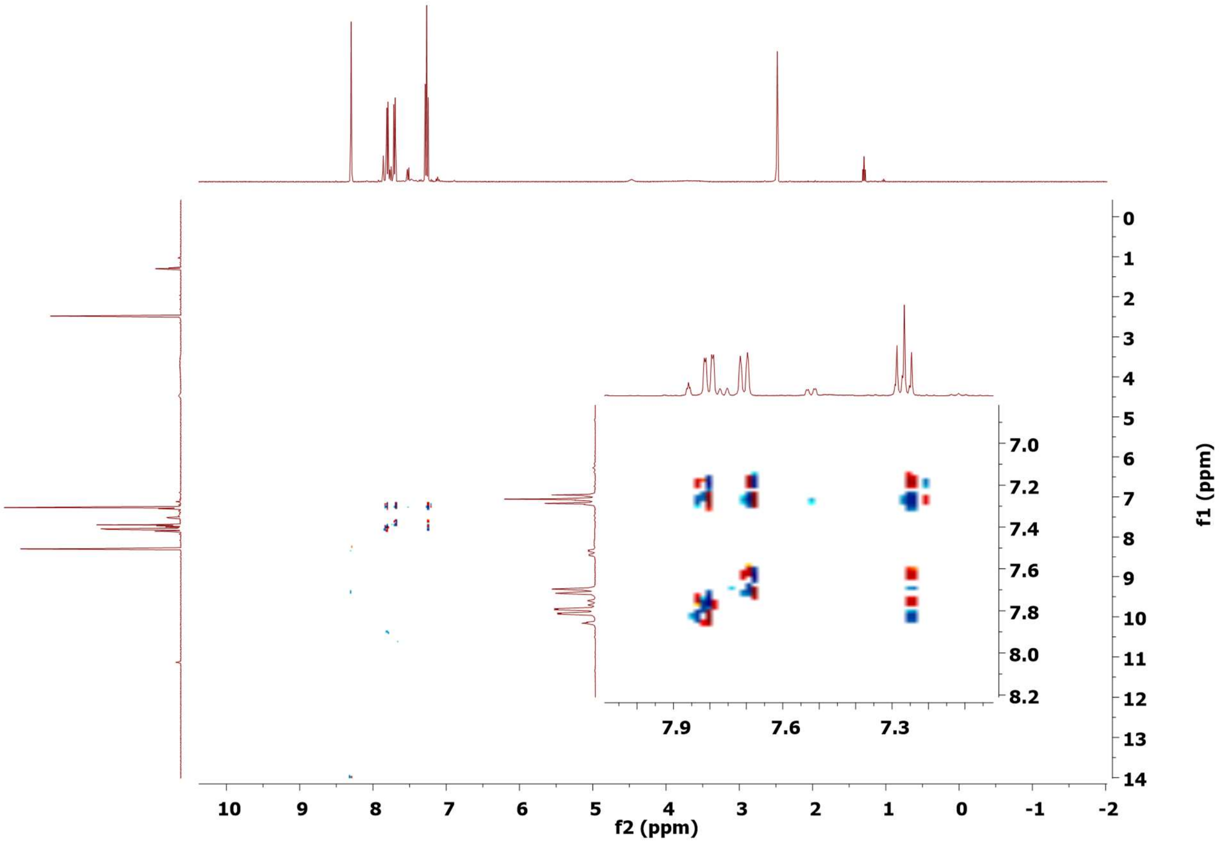

^{1}H-^{13}C-gHSQC NMR (DMSO-d6) spectrum of (3-iodophenyl)carbamothioyl cyanide (1t)

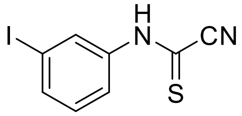

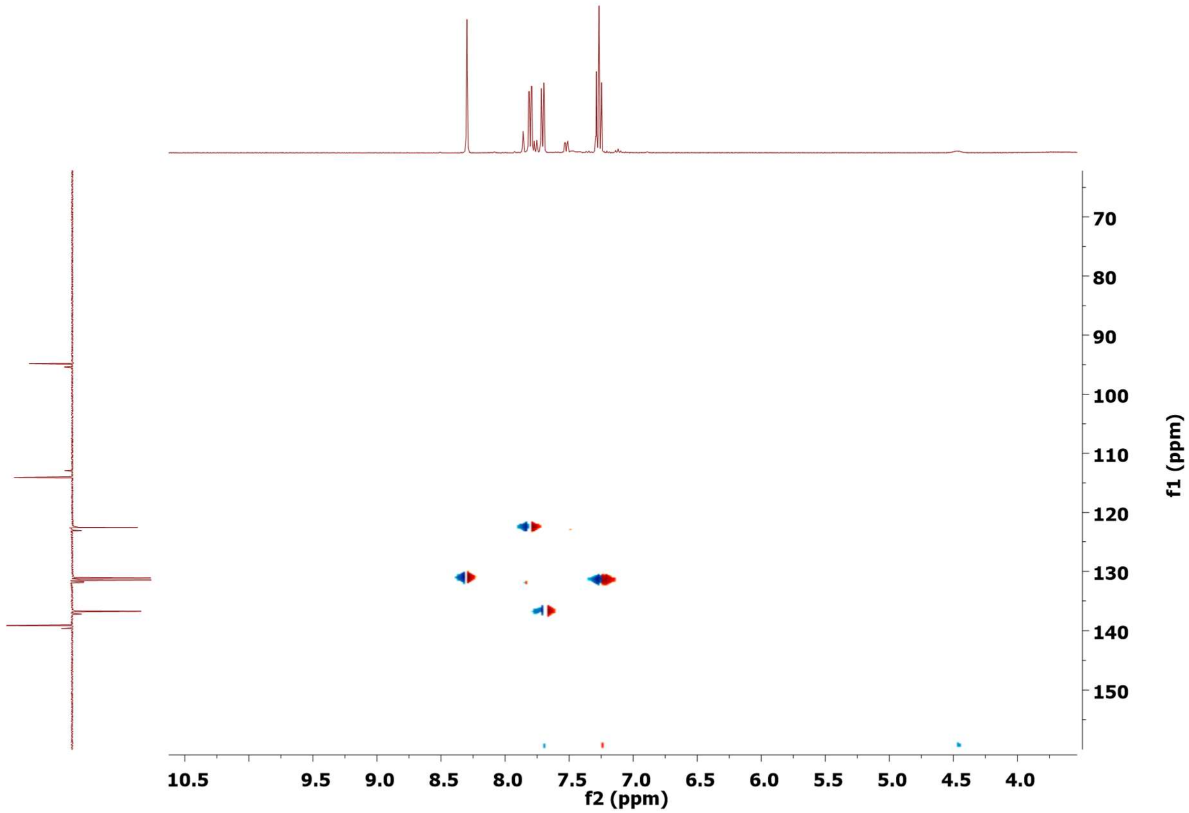

^{1}H-^{13}C-gHMBC NMR (DMSO-d6) spectrum of (3-iodophenyl)carbamothioyl cyanide (1t)

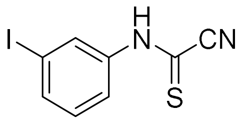

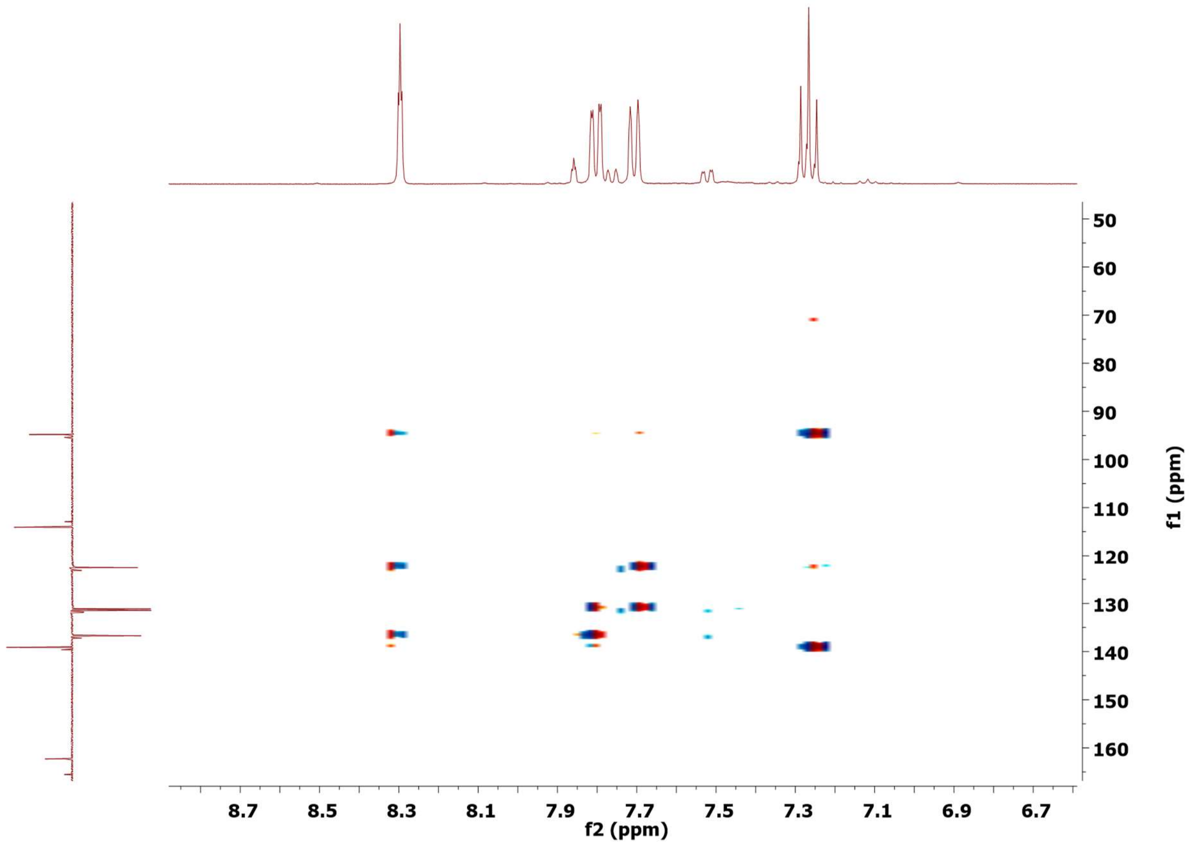

^{1}H NMR (DMSO-d6) spectrum of (3-cyanophenyl)carbamothioyl cyanide (1u)

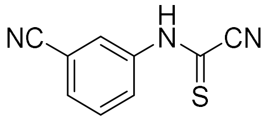

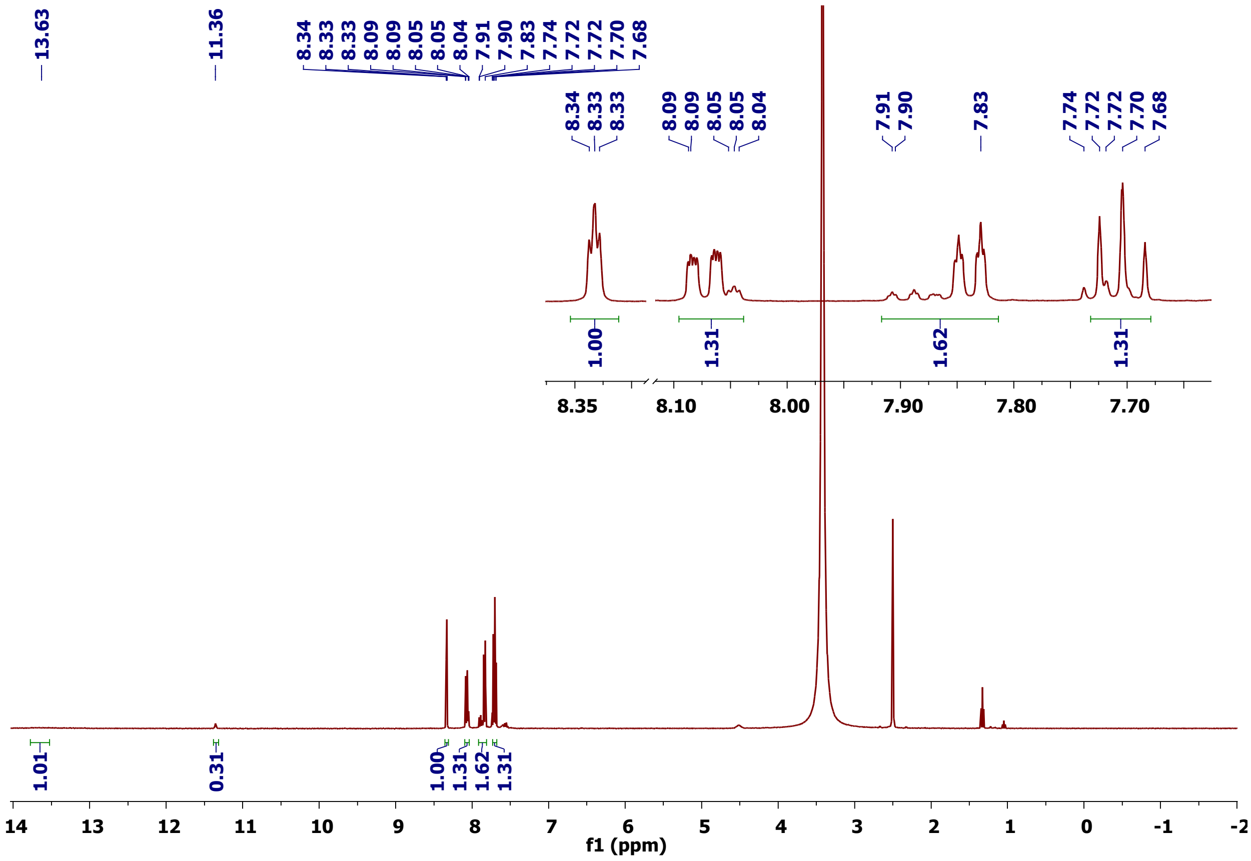

^{13}C NMR (DMSO-d6) spectrum of (3-cyanophenyl)carbamothioyl cyanide (1u)

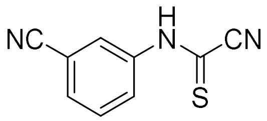

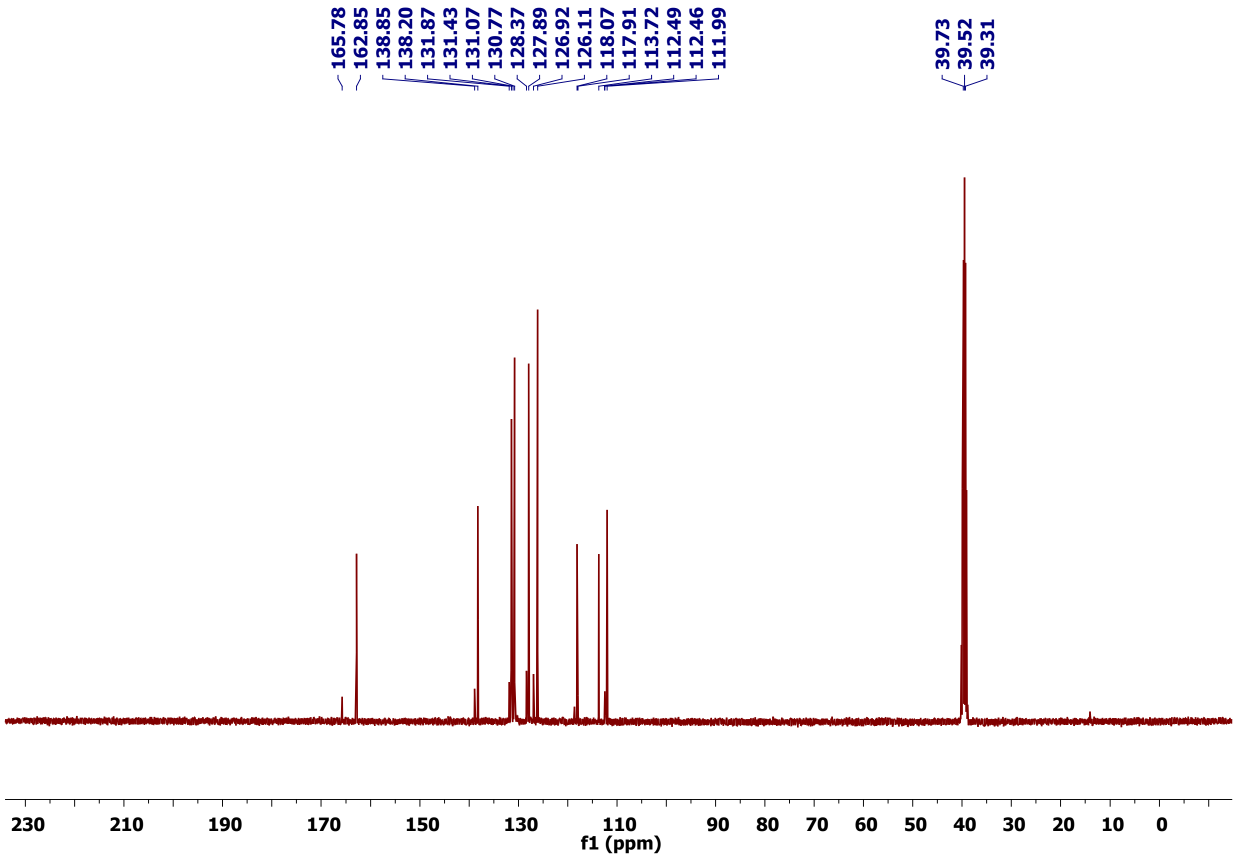

^{13}C CRAPT NMR (DMSO-d6) spectrum of (3-cyanophenyl)carbamothioyl cyanide (1u)

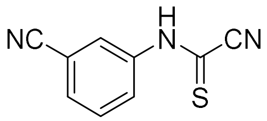

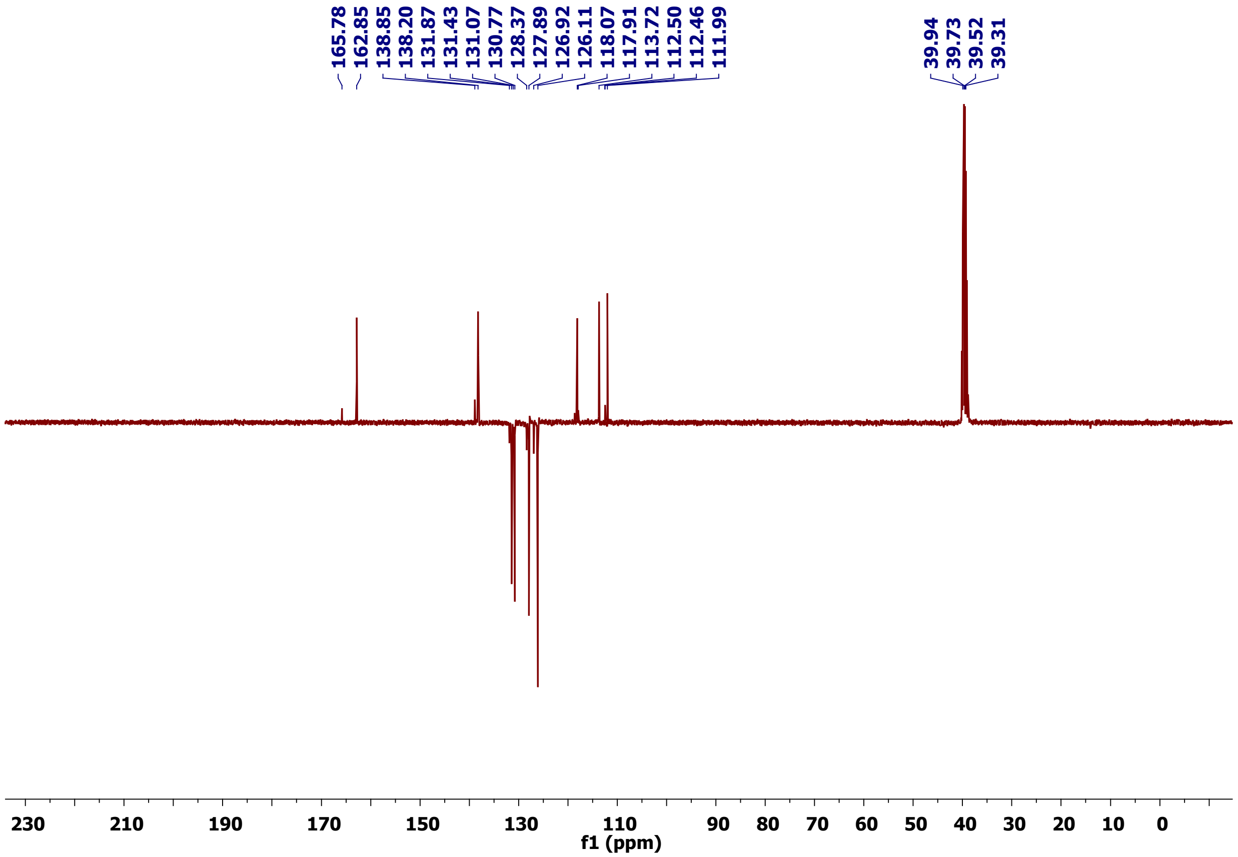

^{1}H-^{1}H gDQCOSY NMR (DMSO-d6) spectrum of (3-cyanophenyl)carbamothioyl cyanide (1u)

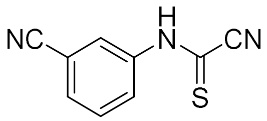

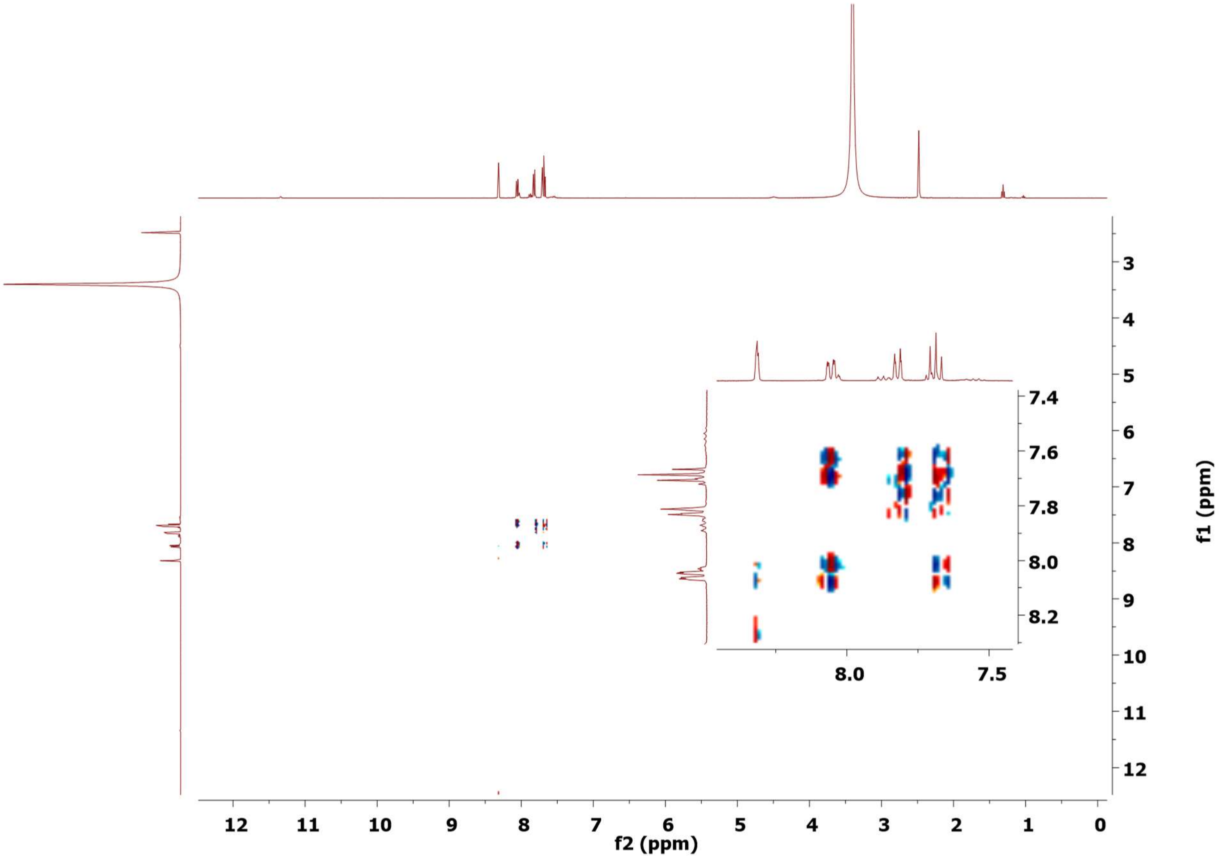

^{1}H-^{13}C-gHSQC NMR (DMSO-d6) spectrum of (3-cyanophenyl)carbamothioyl cyanide (1u)

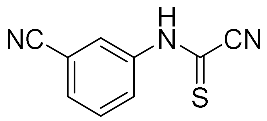

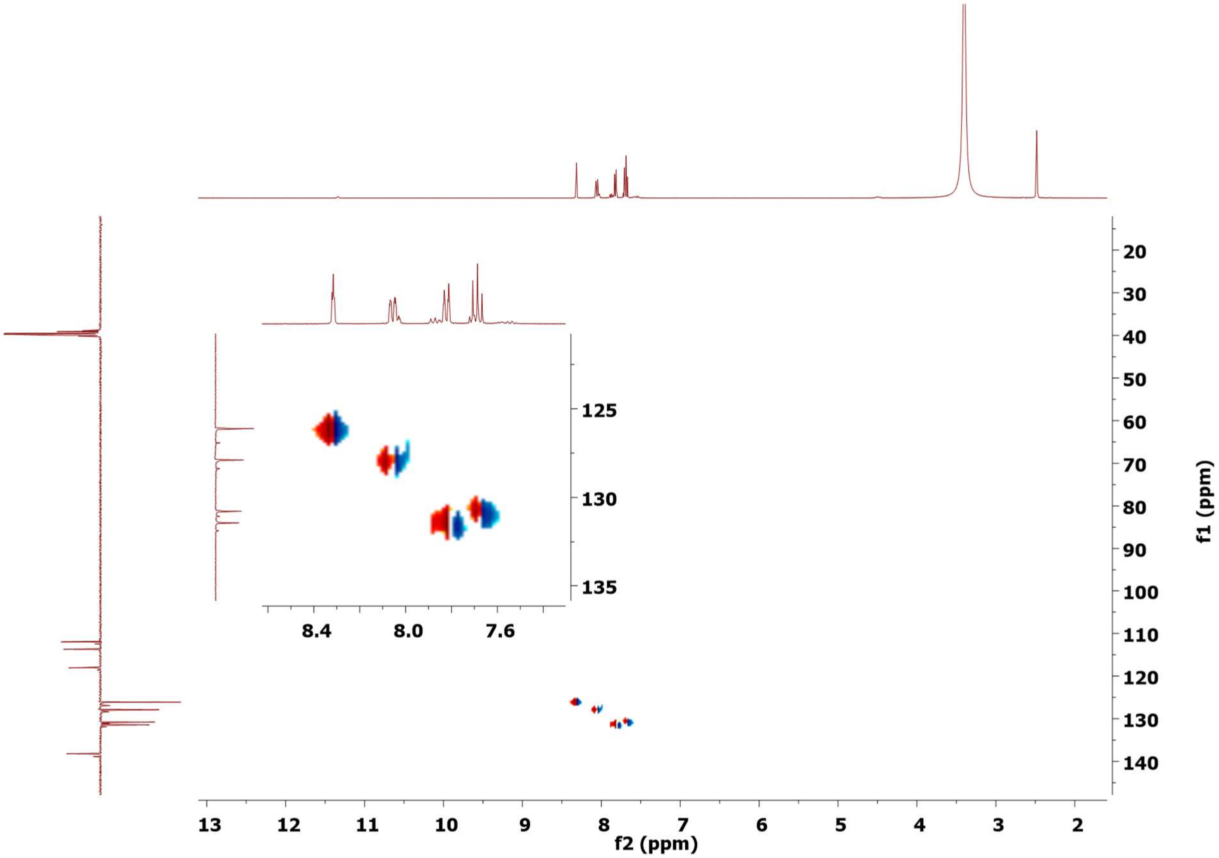

^{1}H-^{13}C-gHMBC NMR (DMSO-d6) spectrum of (3-cyanophenyl)carbamothioyl cyanide (1u)

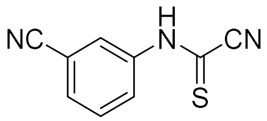

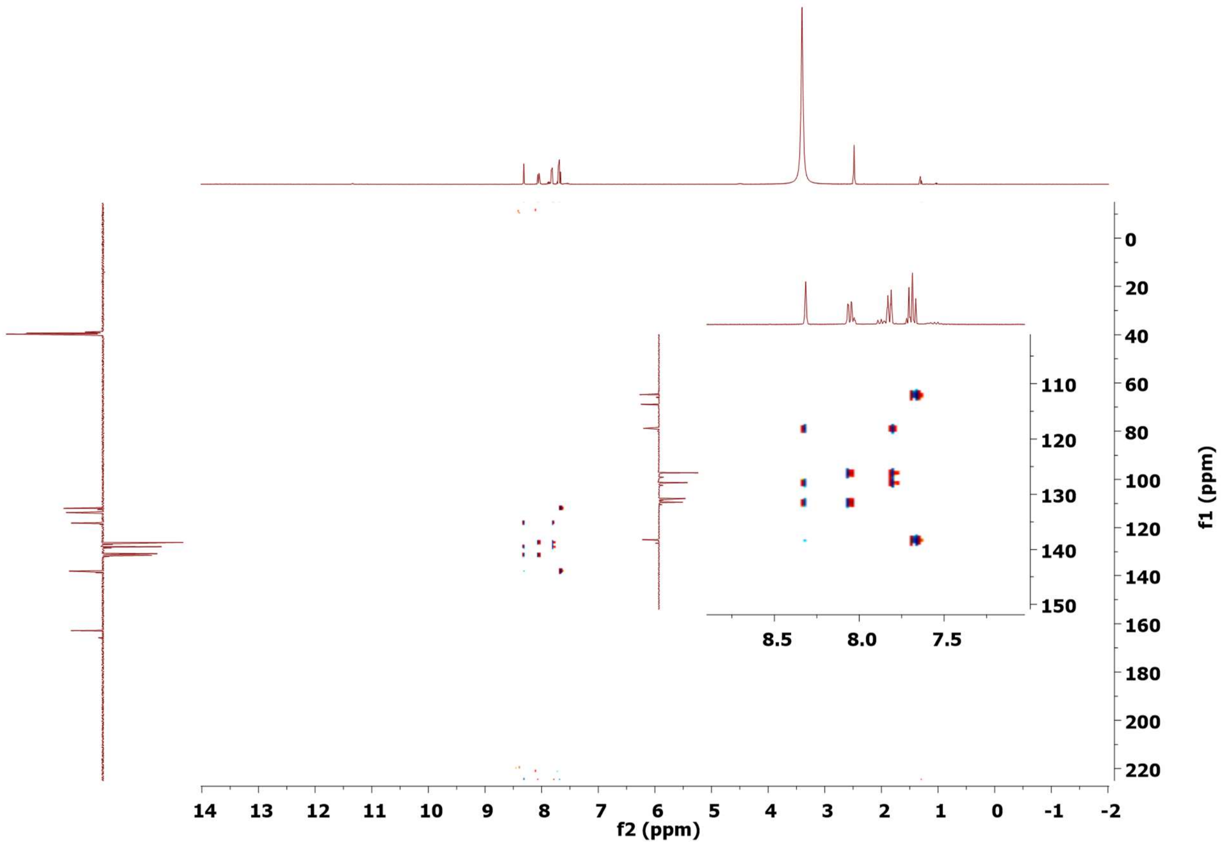

^{1}H NMR (DMSO-d6) spectrum of (3-(trifluoromethyl)phenyl)carbamothioyl cyanide (1v)

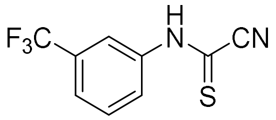

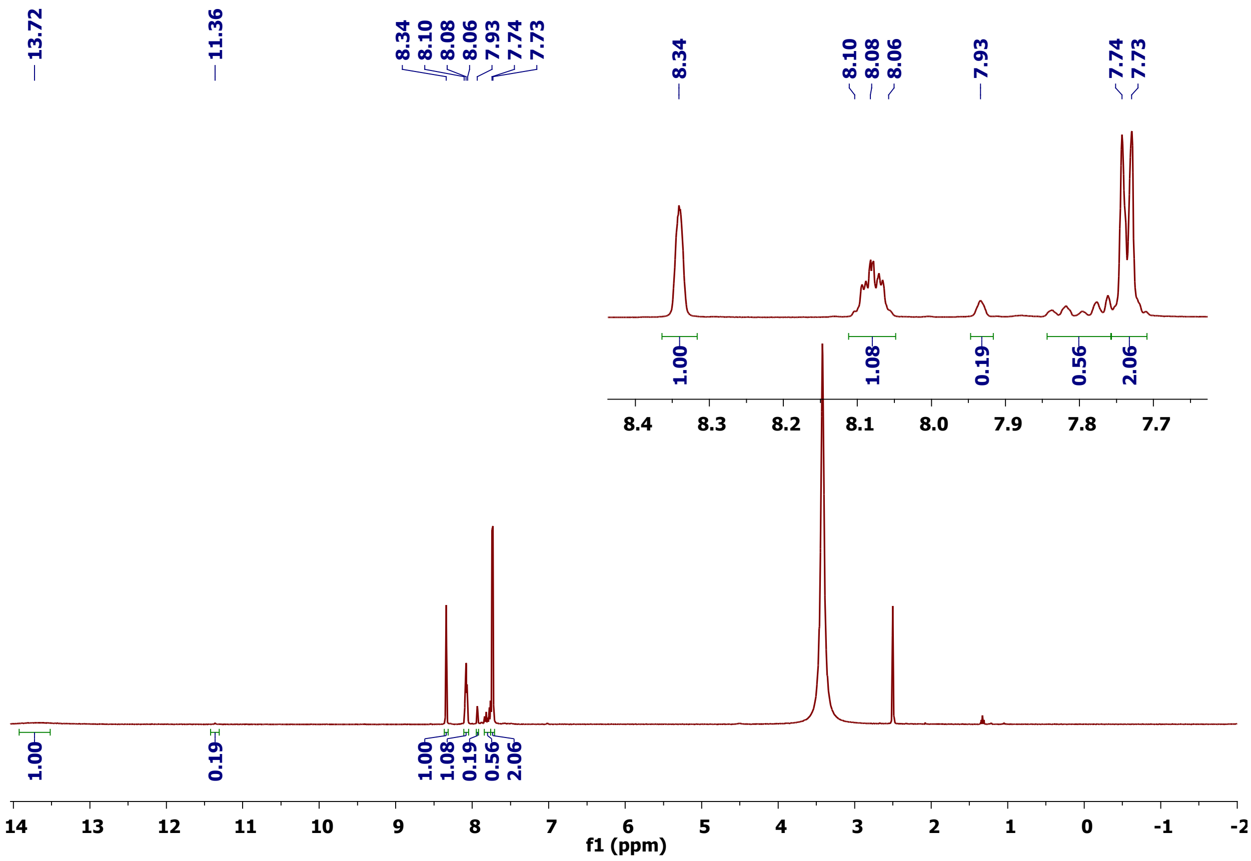

^{13}C NMR (DMSO-d6) spectrum of (3-(trifluoromethyl)phenyl)carbamothioyl cyanide (1v)

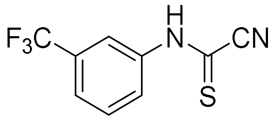

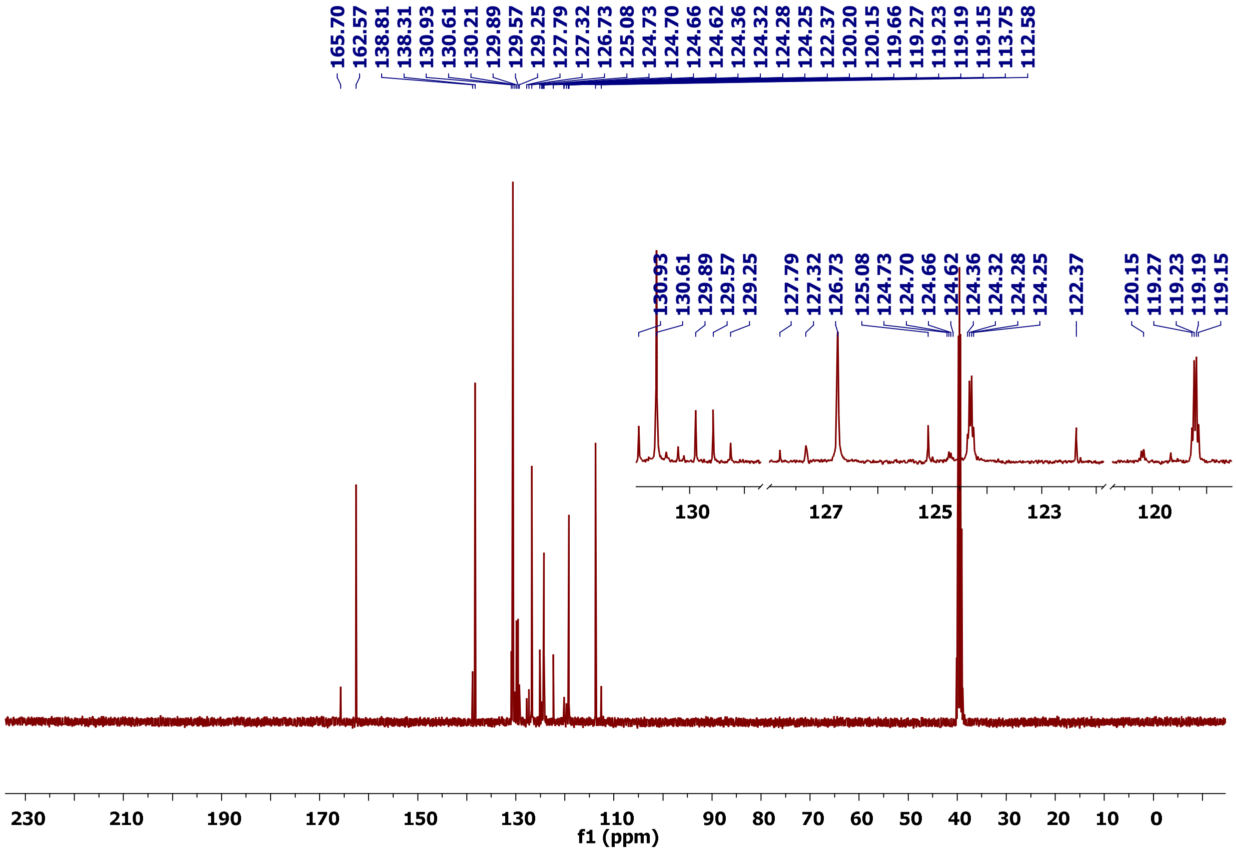

^{13}C CRAPT NMR (DMSO-d6) spectrum of (3-(trifluoromethyl)phenyl)carbamothioyl cyanide (1v)

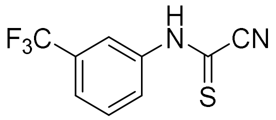

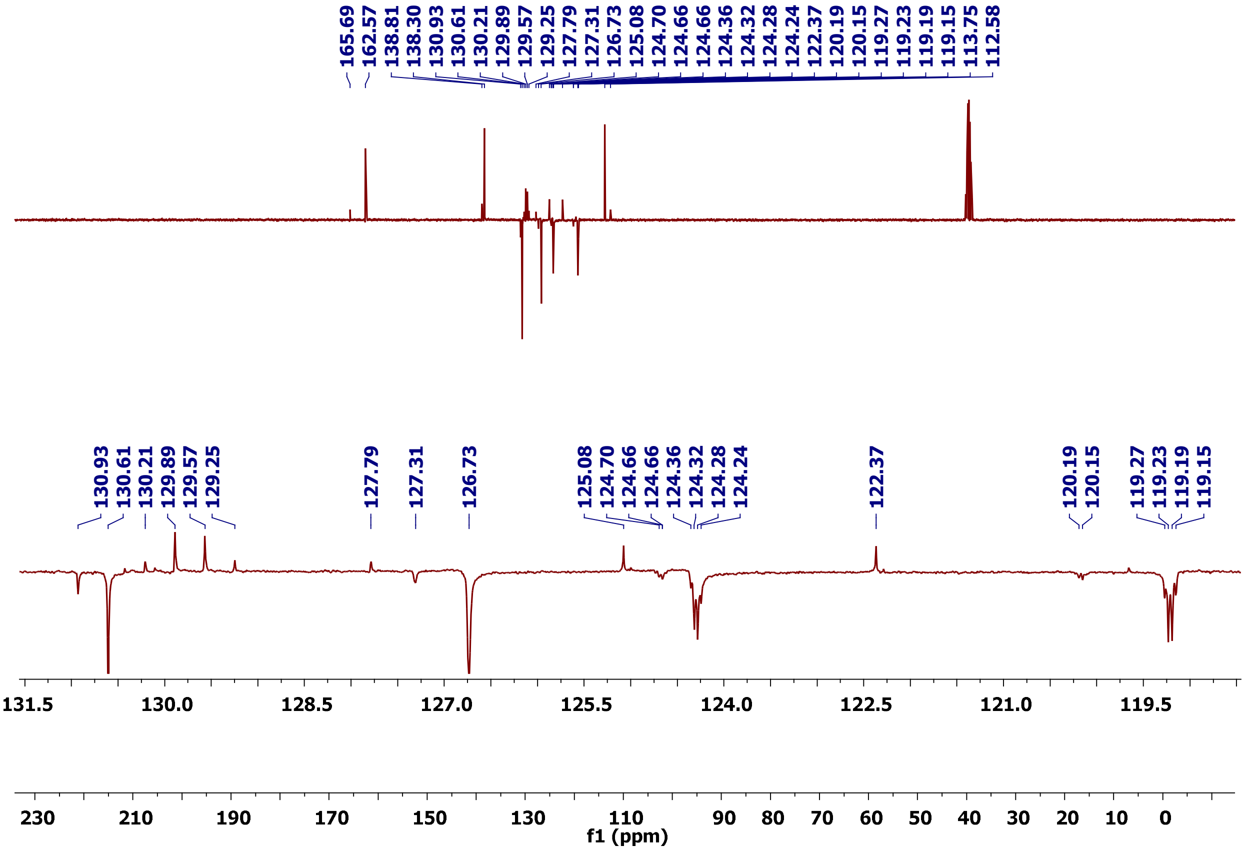

^1H-^1H gDQCOSY NMR (DMSO-d6) spectrum of (3-(trifluoromethyl)phenyl)carbamothioyl cyanide (1v)

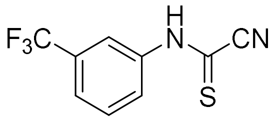

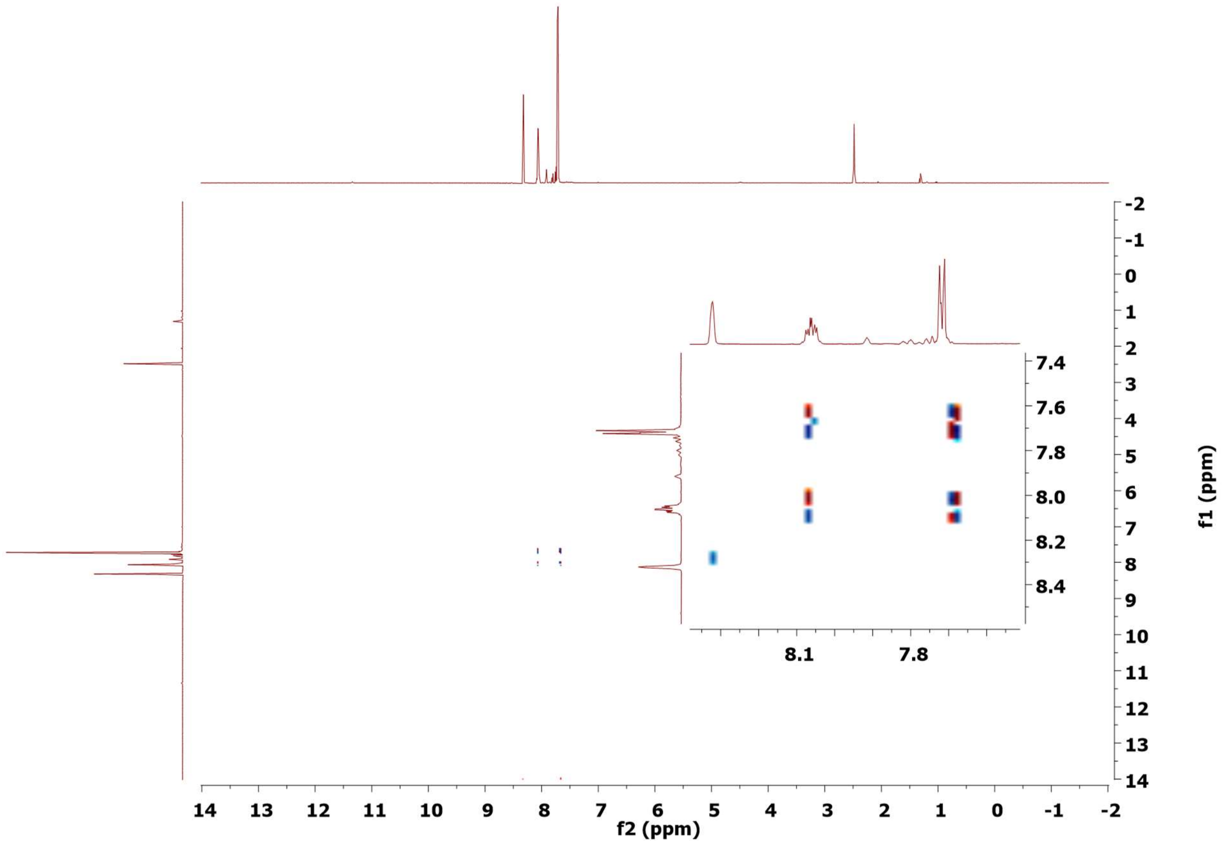

^{1}H-^{13}C-gHSQC NMR (DMSO-d6) spectrum of (3-(trifluoromethyl)phenyl)carbamothioyl cyanide (1v)

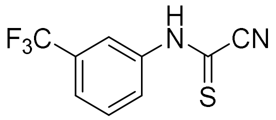

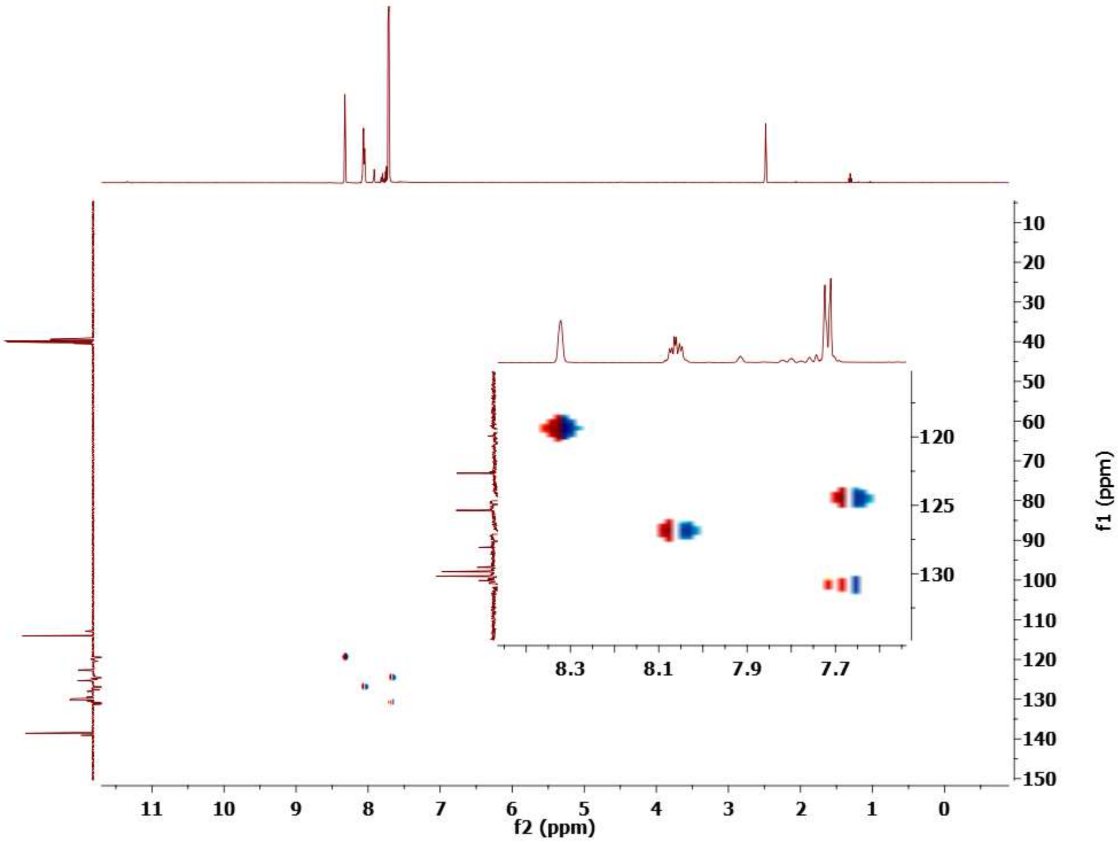

^{1}H-^{13}C-gHMBC NMR (DMSO-d6) spectrum of (3-(trifluoromethyl)phenyl)carbamothioyl cyanide (1v)

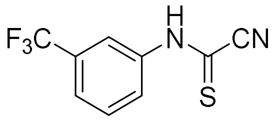

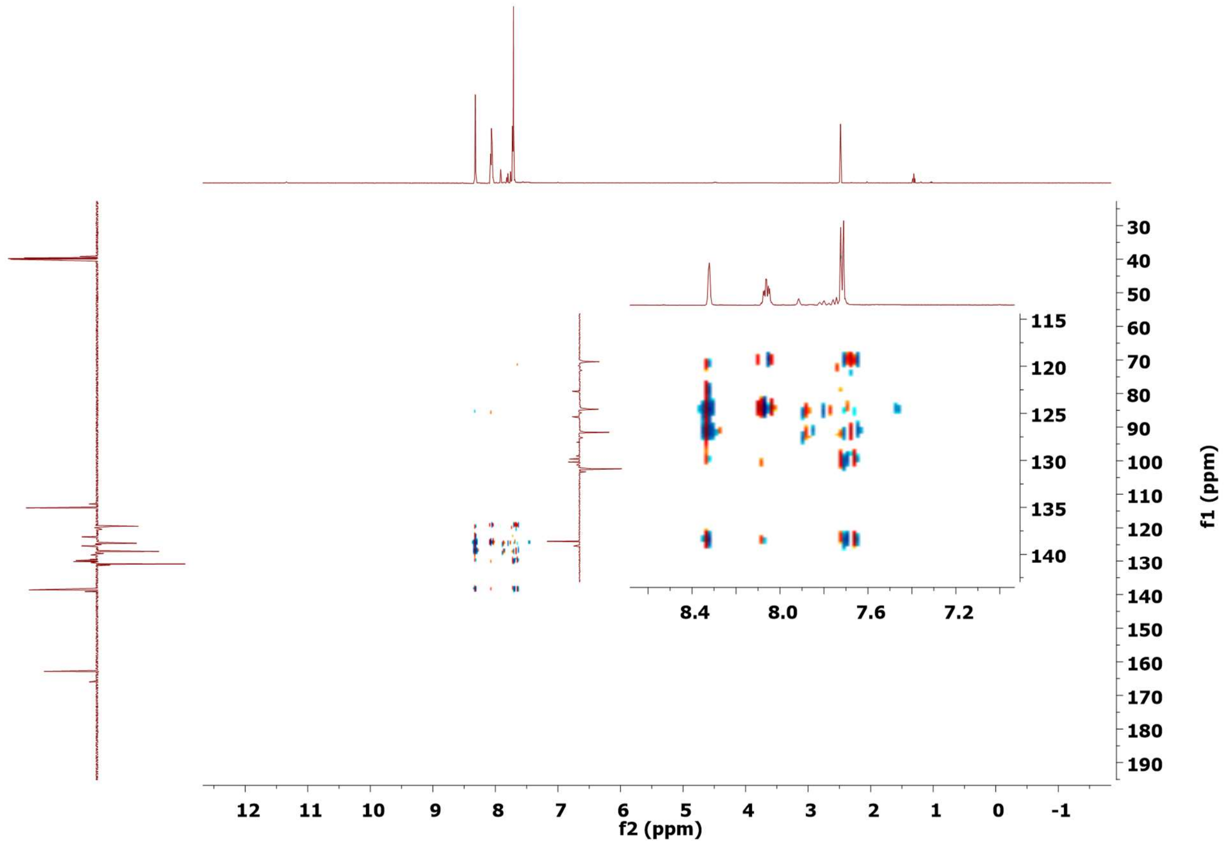

^{1}H NMR (DMSO-d6) spectrum of (4-methyl-1,3-phenylene)dicarbamothioyl cyanide (1w)

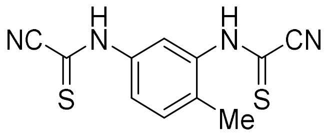

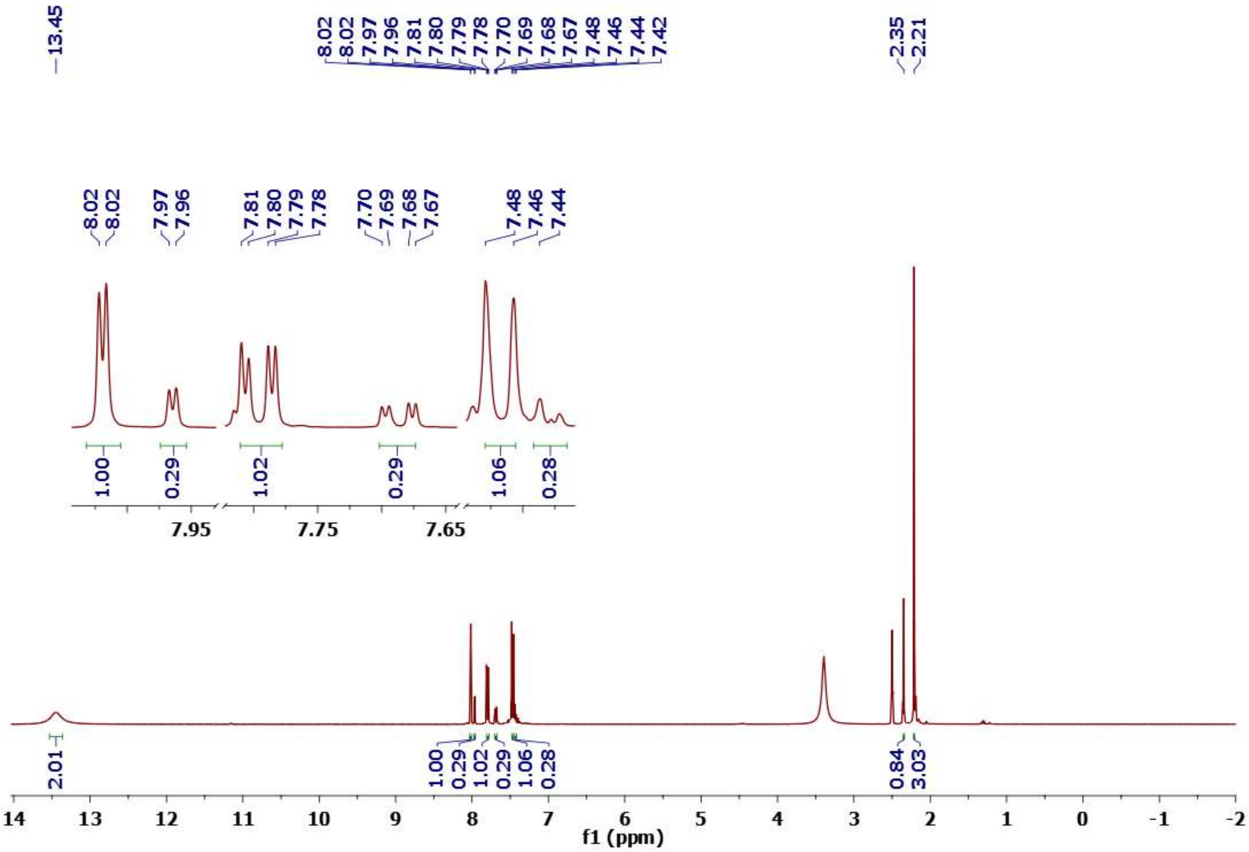

^{13}C CRAPT NMR (DMSO-d6) spectrum of (4-methyl-1,3-phenylene)dicarbamothioyl cyanide (1w)

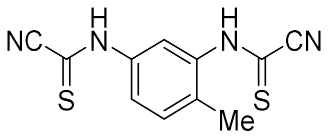

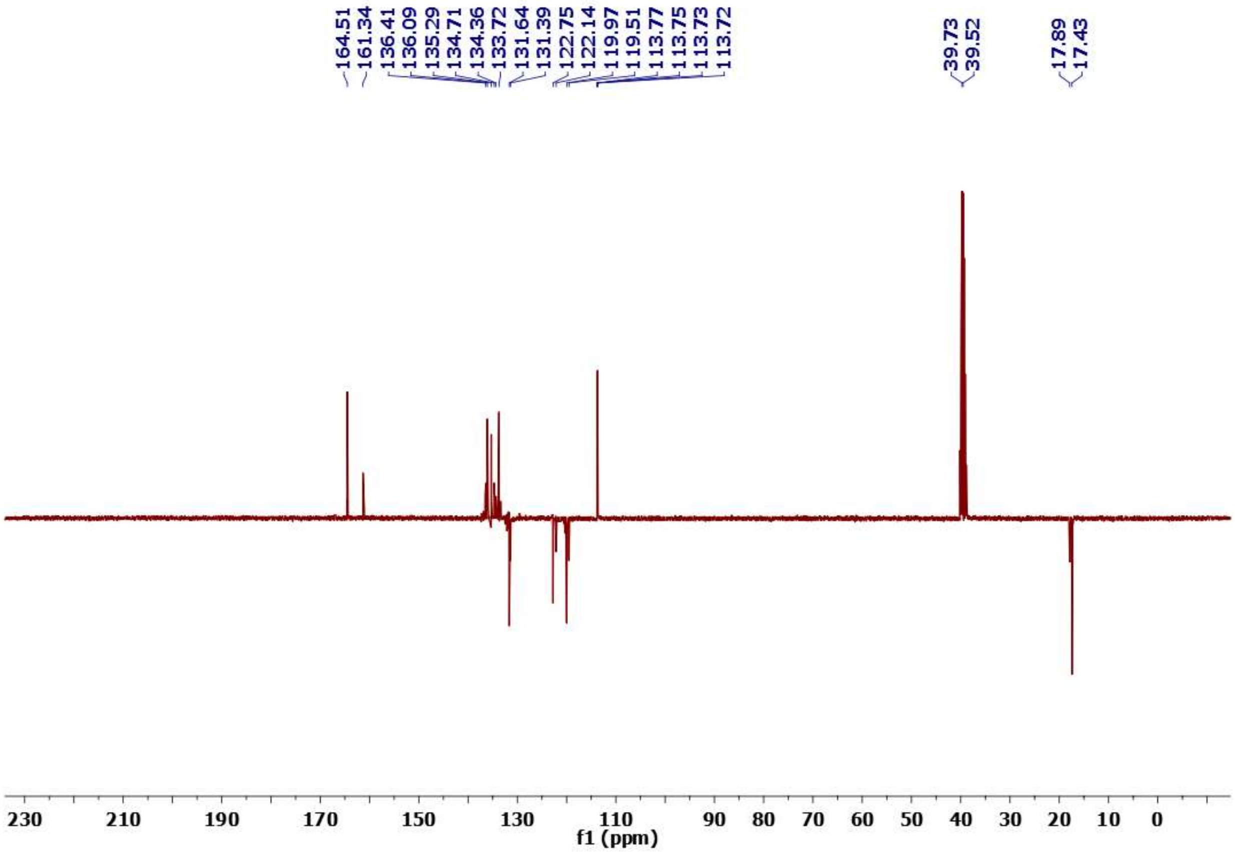

^{1}H NMR (DMSO-d6) spectrum of 1,4-phenylenedicarbamothioyl cyanide (1x)

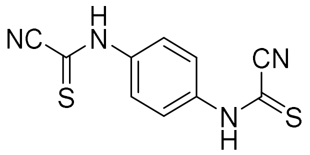

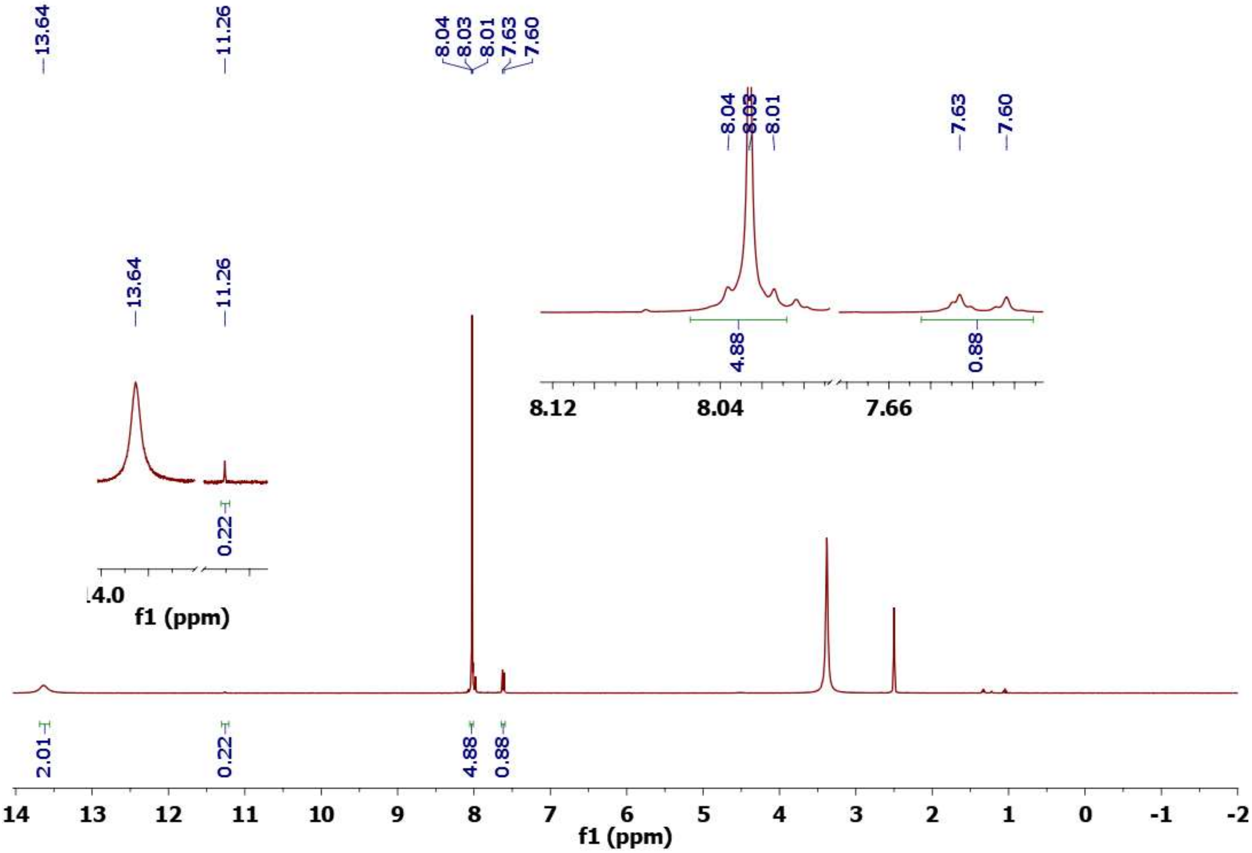

^{13}C NMR (DMSO-d6) spectrum of 1,4-phenylenedicarbamothioyl cyanide (1x)

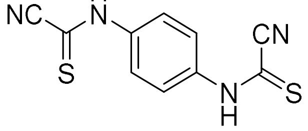

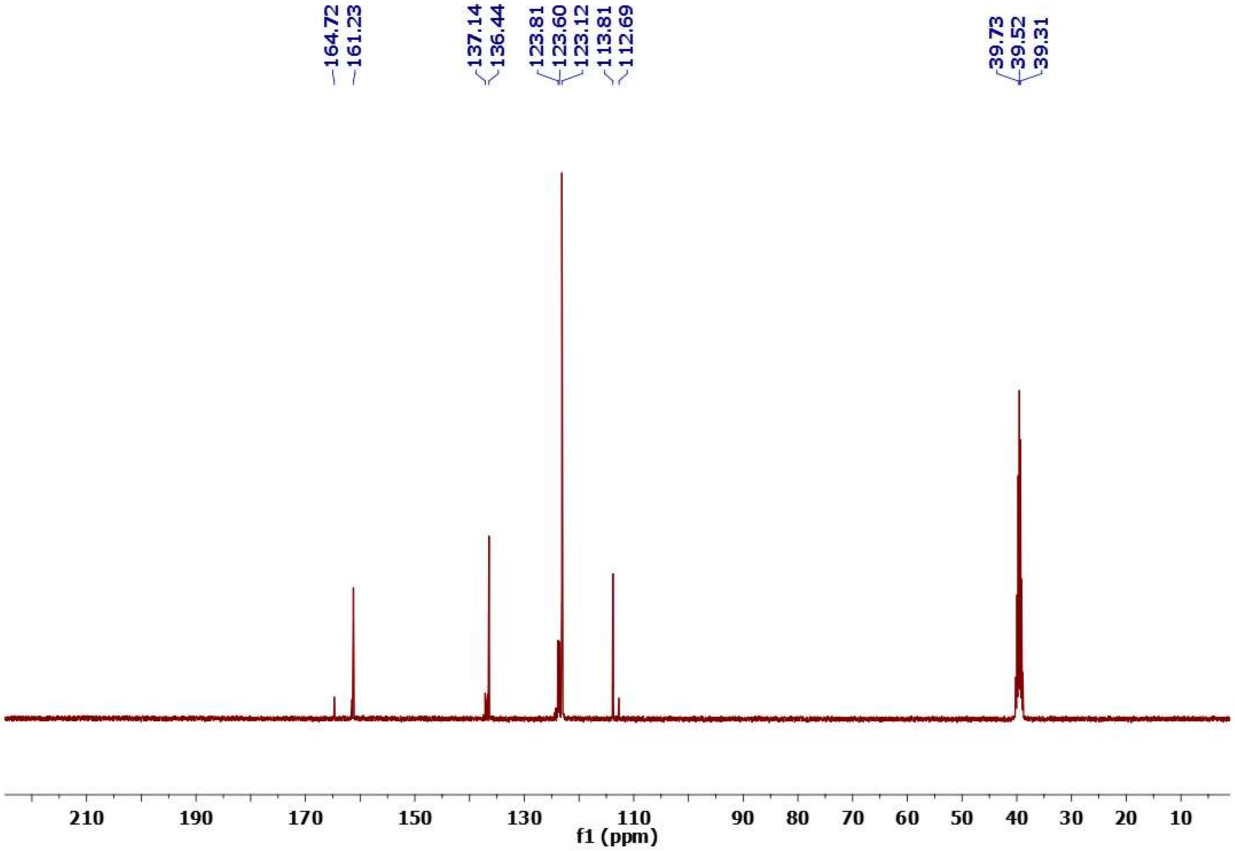

^{13}C CRAPT NMR (DMSO-d6) spectrum of 1,4-phenylenedicarbamothioyl cyanide (1x)

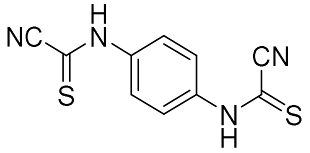

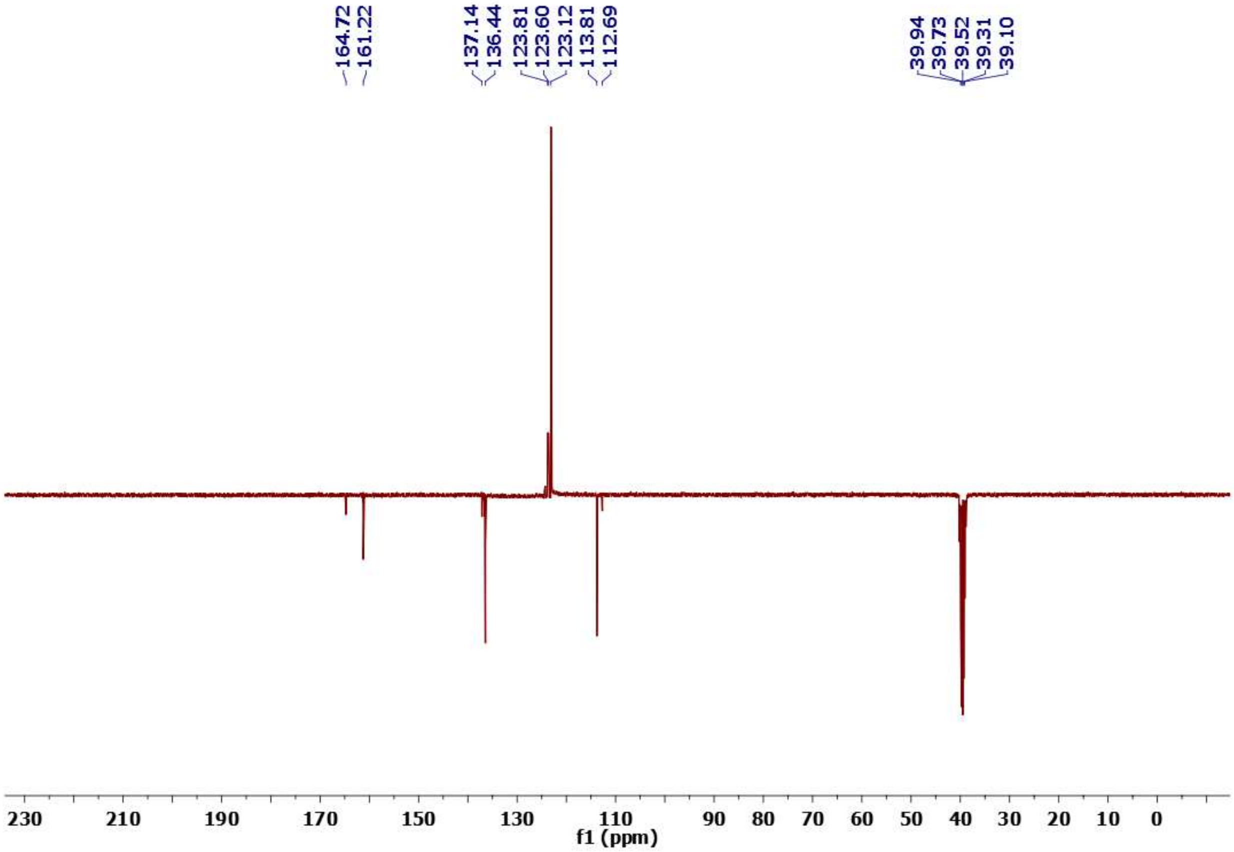

^{1}H NMR (DMSO-d6) spectrum of (2-bromophenyl)carbamothioyl cyanide (1y)

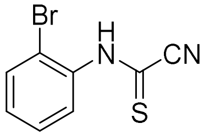

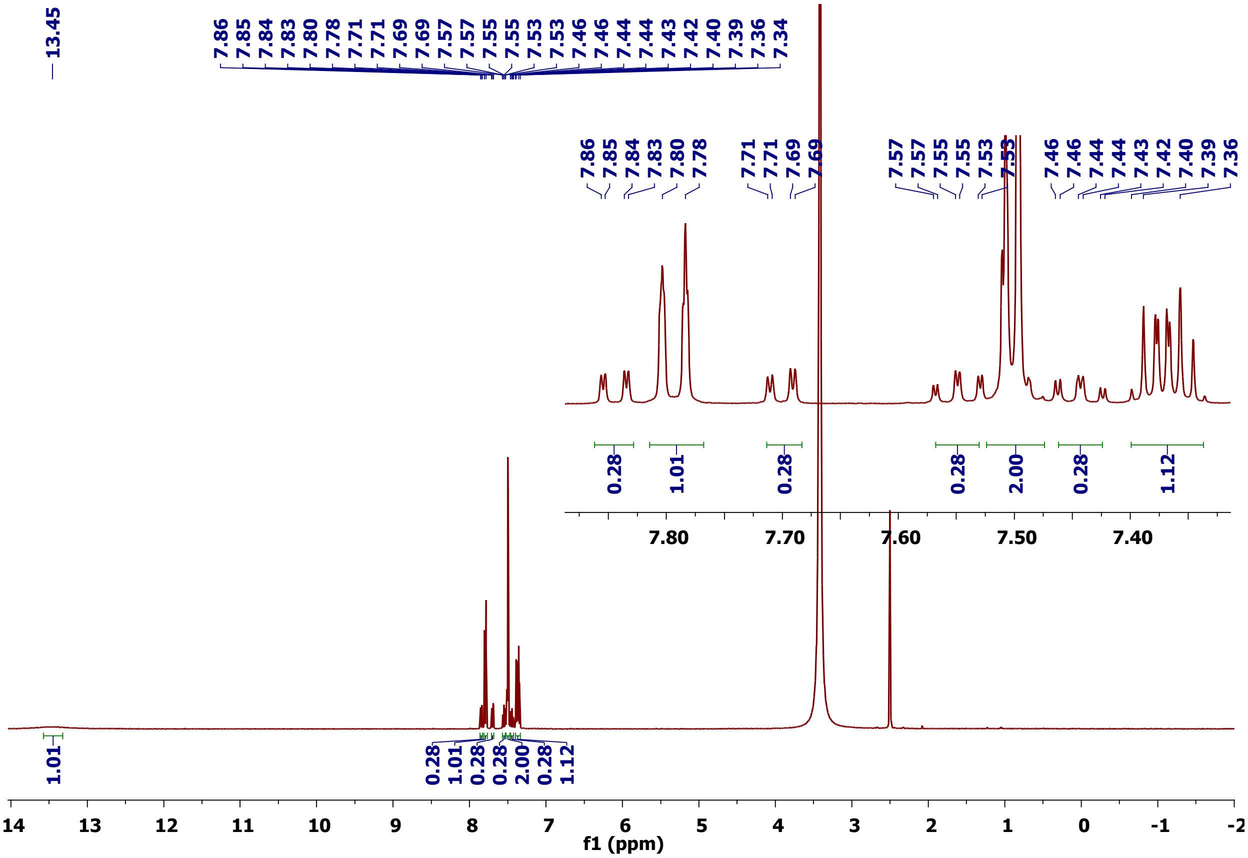

^{13}C NMR (DMSO-d6) spectrum of (2-bromophenyl)carbamothioyl cyanide (1y)

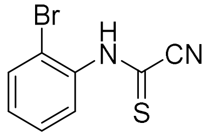

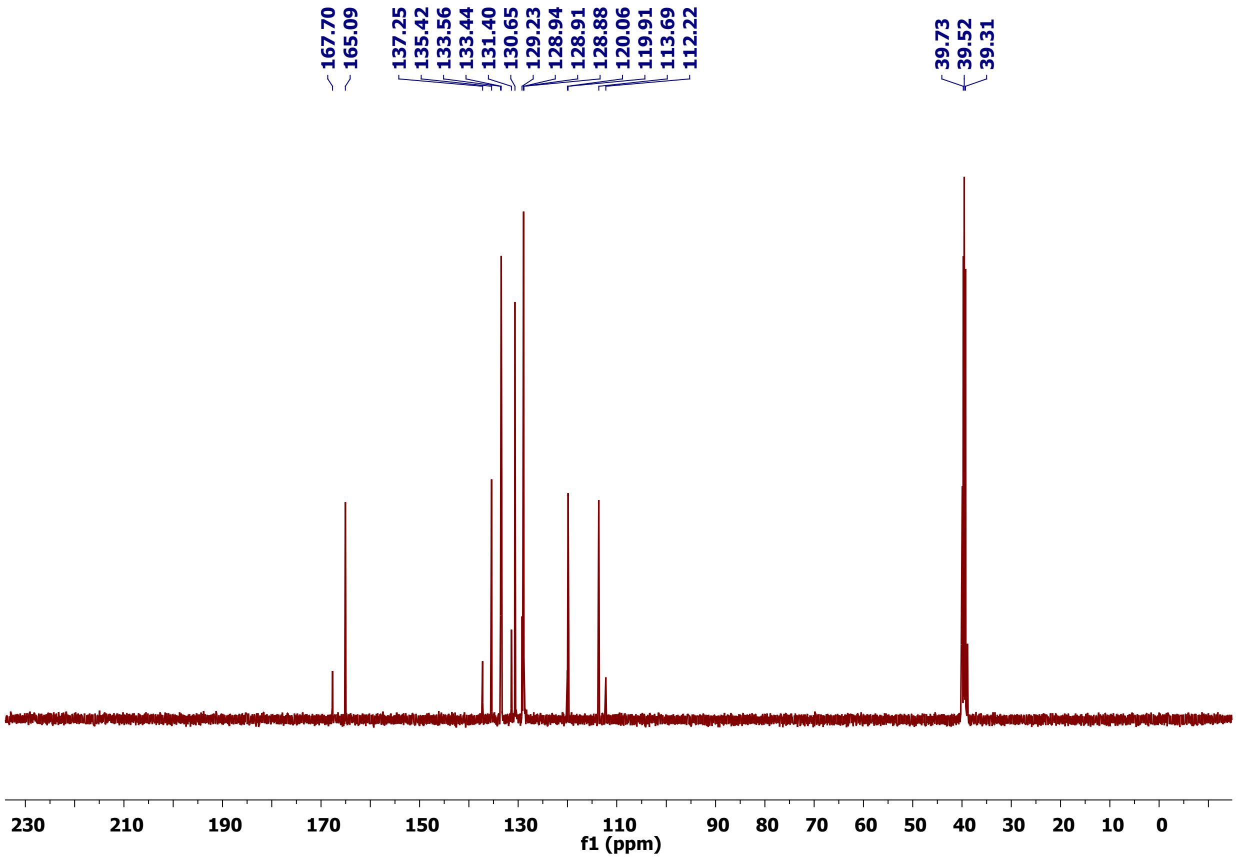

^{13}C CRAPT NMR (DMSO-d6) spectrum of (2-bromophenyl)carbamothioyl cyanide (1y)

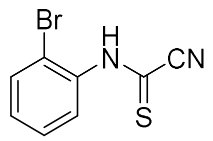

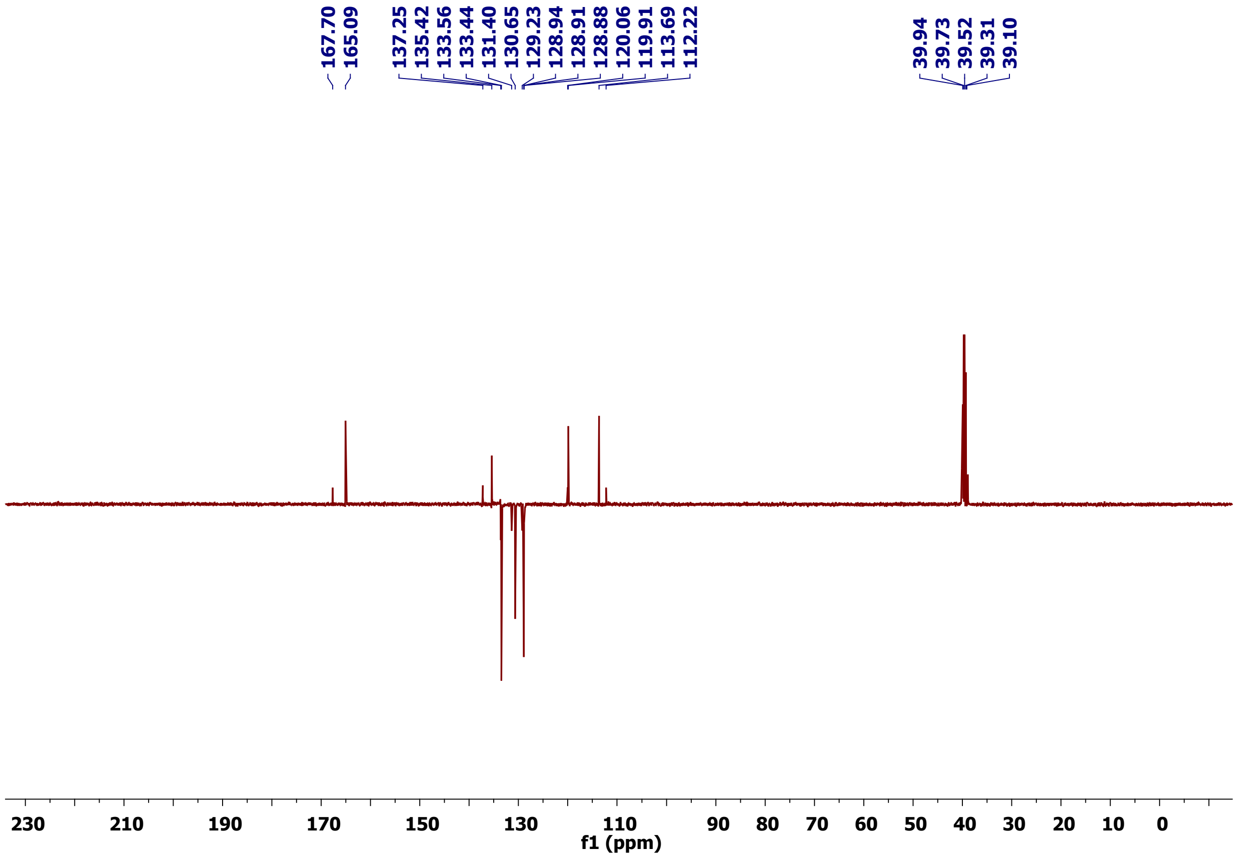

^{1}H-^{1}H gDQCOSY NMR (DMSO-d6) spectrum of (2-bromophenyl)carbamothioyl cyanide (1y)

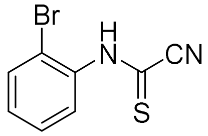

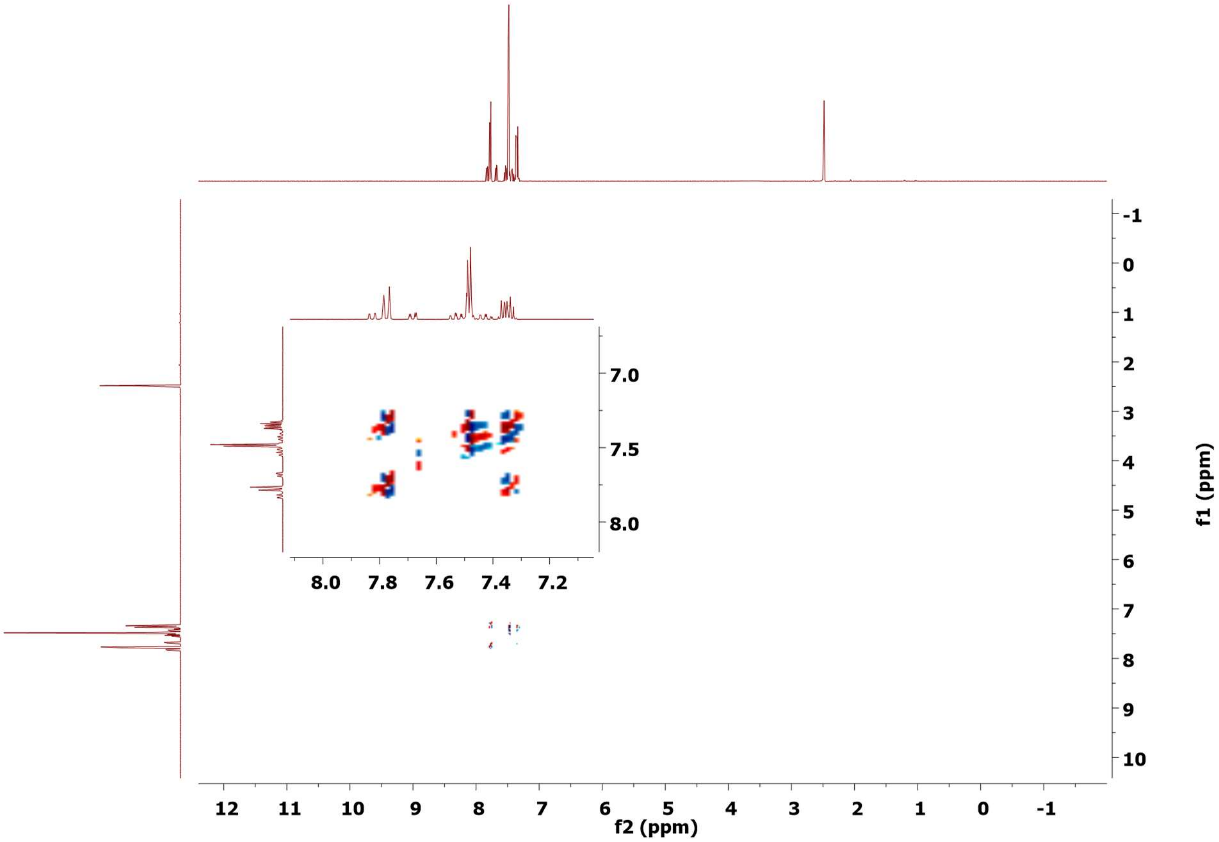

^{1}H-^{13}C-gHSQC NMR (DMSO-d6) spectrum of (2-bromophenyl)carbamothioyl cyanide (1y)

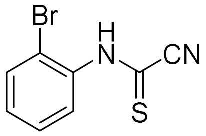

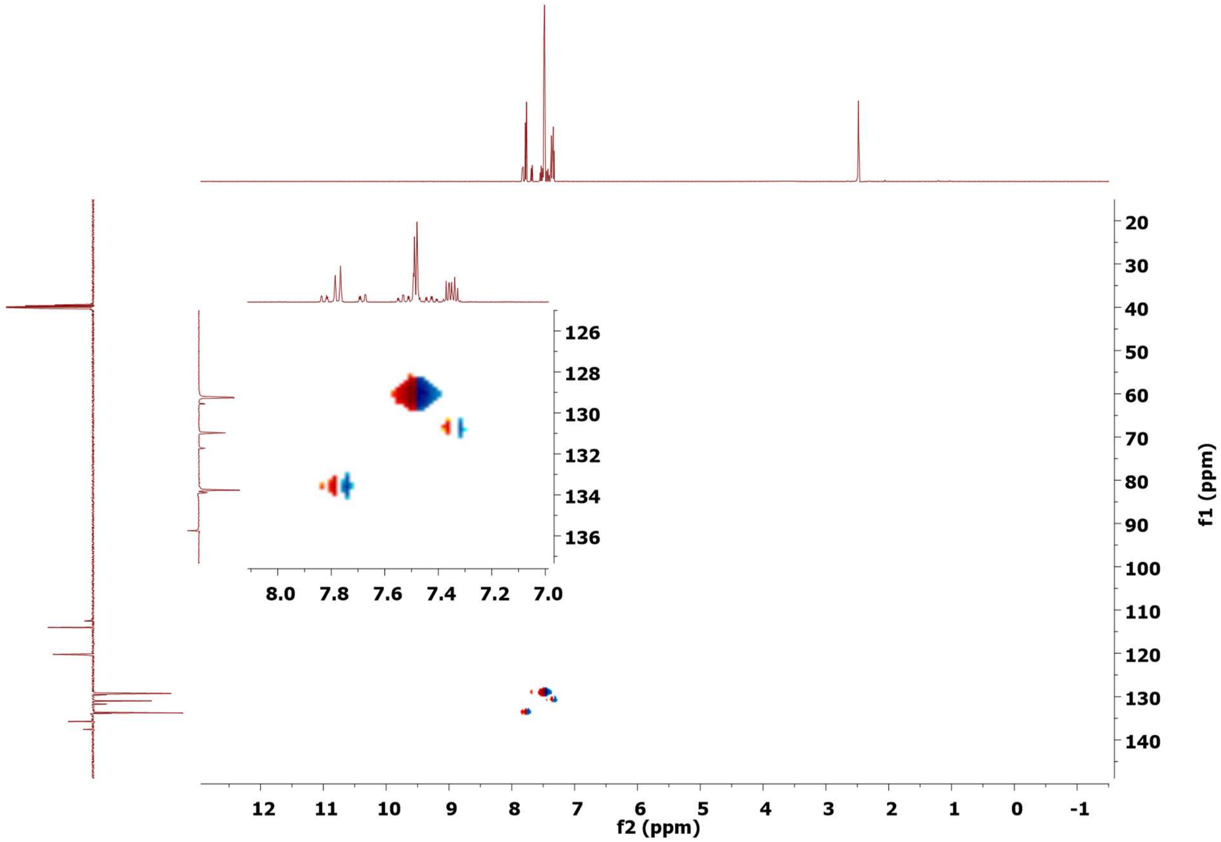

^{1}H-^{13}C-gHMBC NMR (DMSO-d6) spectrum of (2-bromophenyl)carbamothioyl cyanide (1y)

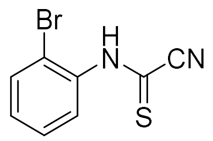

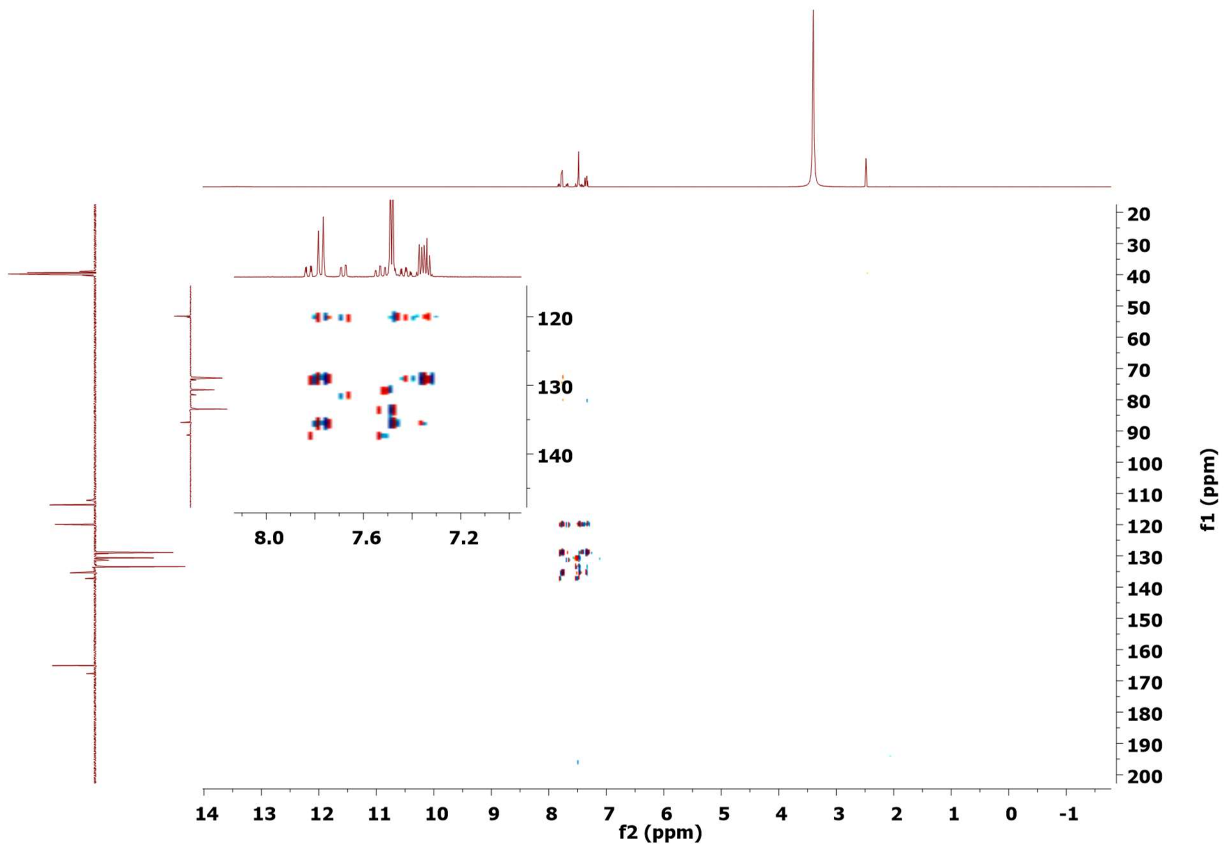

^{1}H NMR (DMSO-d6) spectrum of (2,4-dichlorophenyl)carbamothioyl cyanide (1z)

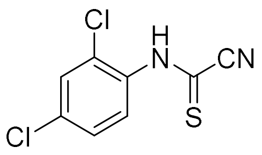

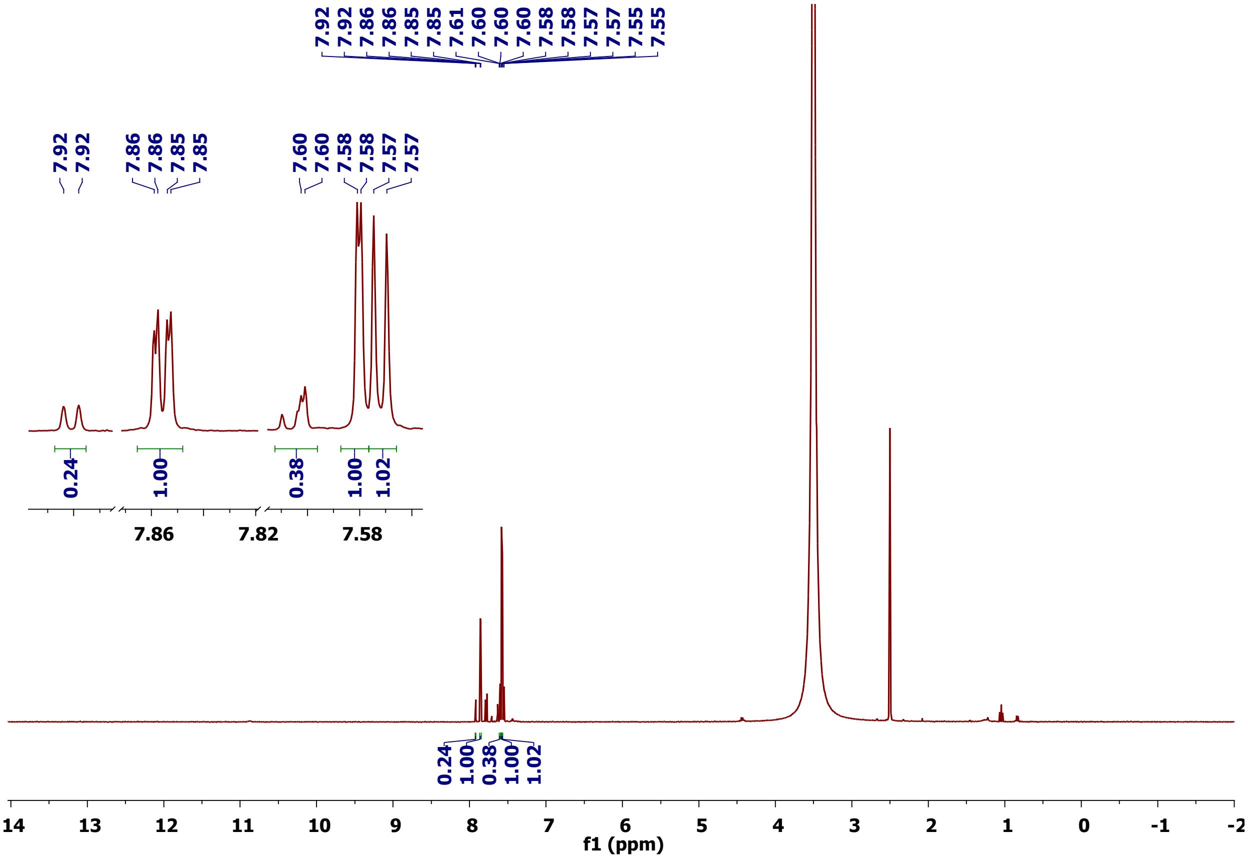

^{13}C NMR (DMSO-d6) spectrum of (2,4-dichlorophenyl)carbamothioyl cyanide (1z)

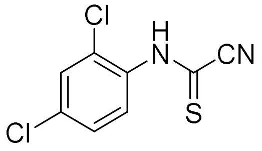

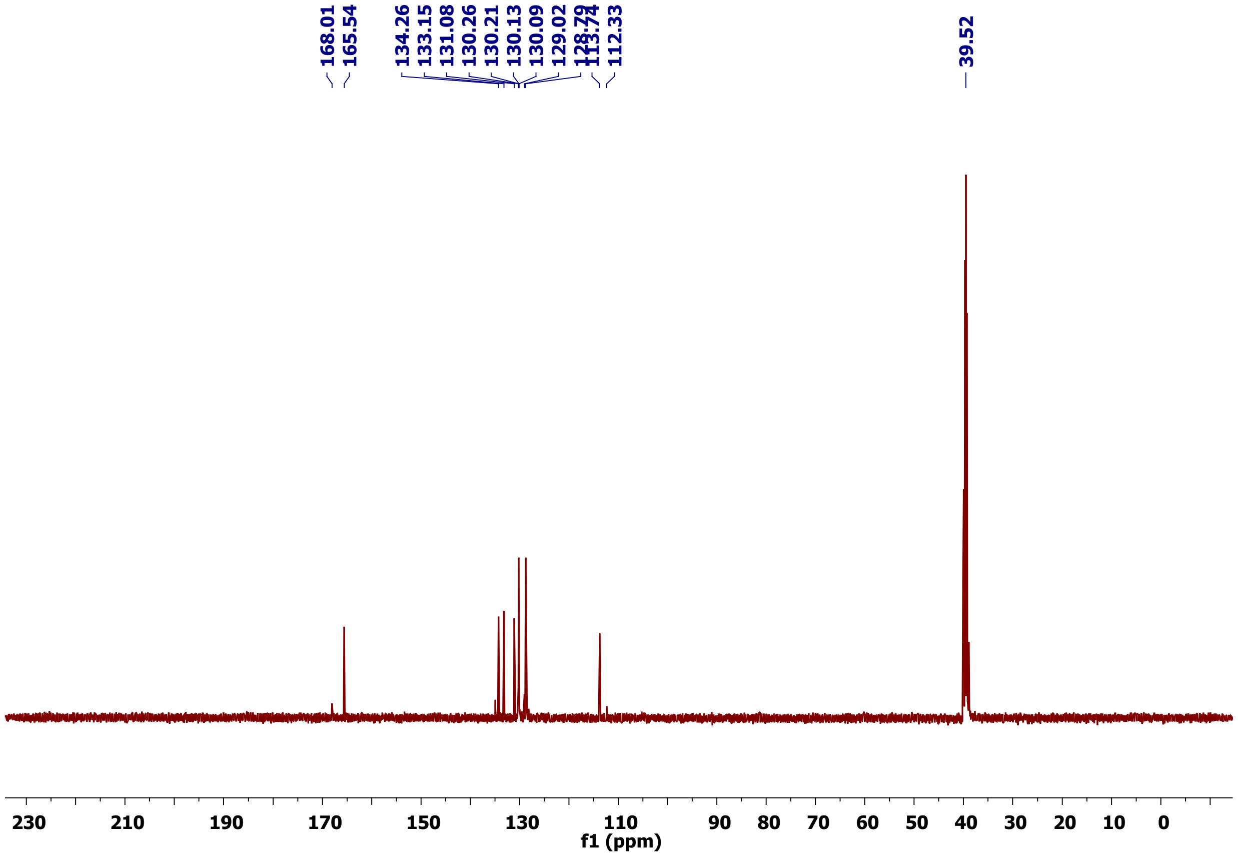

^{13}C CRAPT NMR (DMSO-d6) spectrum of (2,4-dichlorophenyl)carbamothioyl cyanide (1z)

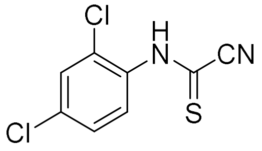

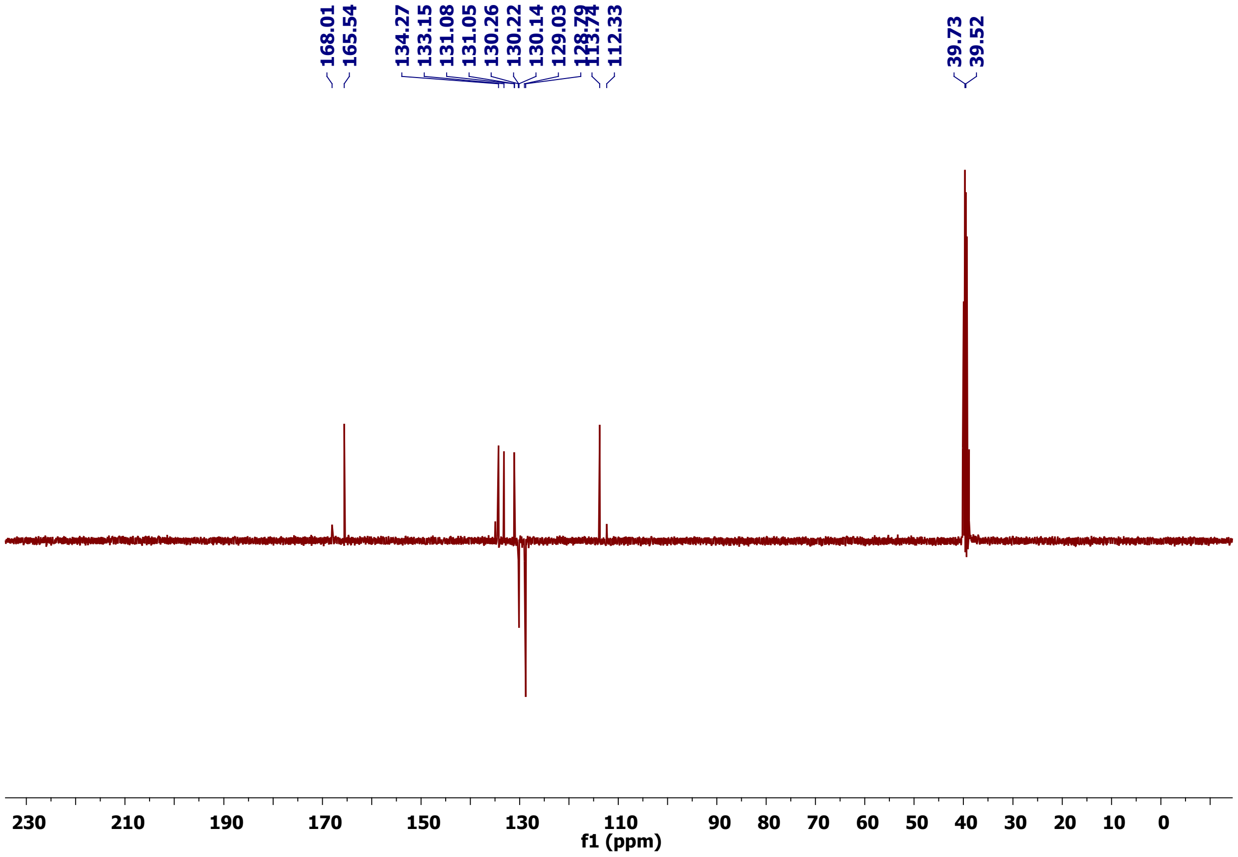

^{1}H NMR (DMSO-d6) spectrum of (5-chloro-2-methylphenyl)carbamothioyl cyanide (1a')

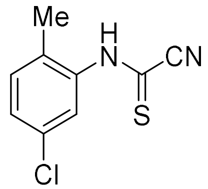

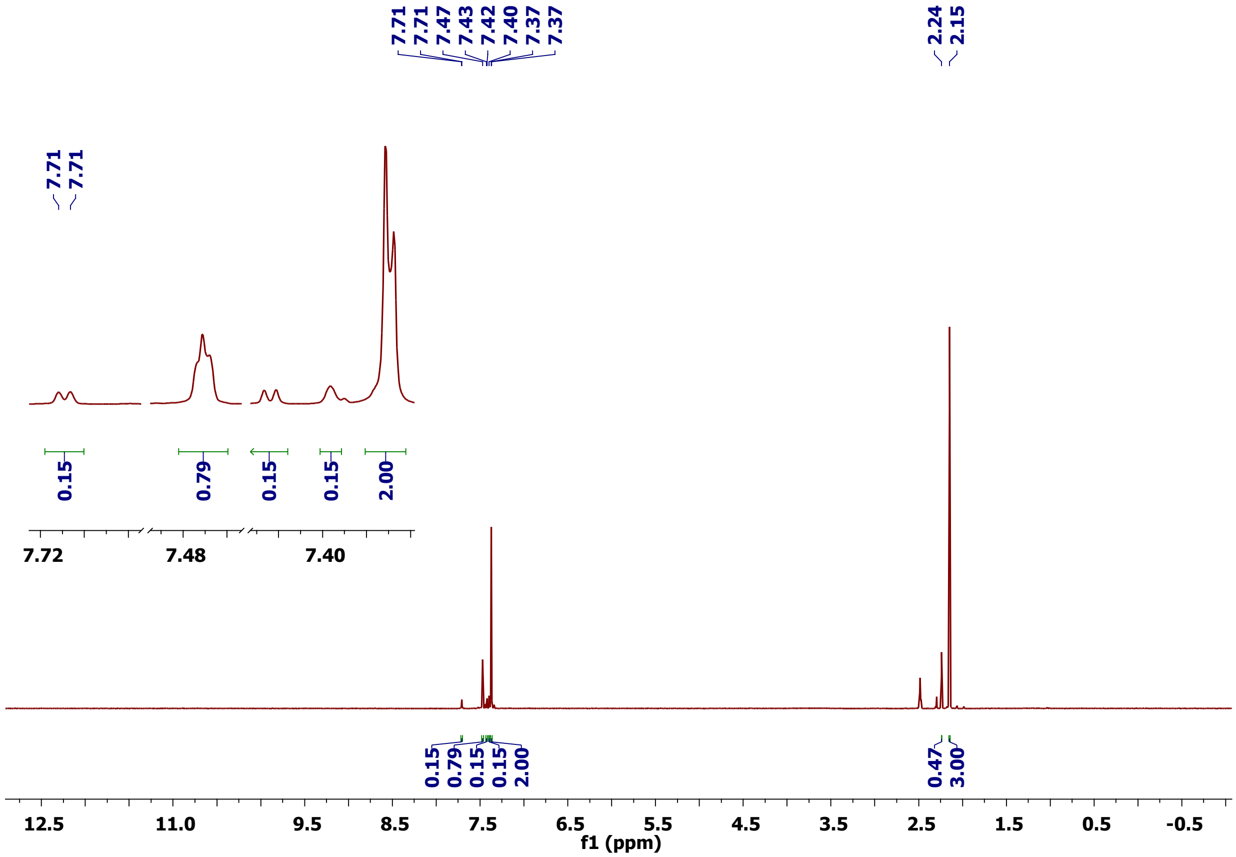

^{13}C NMR (DMSO-d6) spectrum of (5-chloro-2-methylphenyl)carbamothioyl cyanide (1a’)

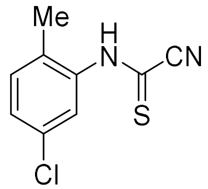

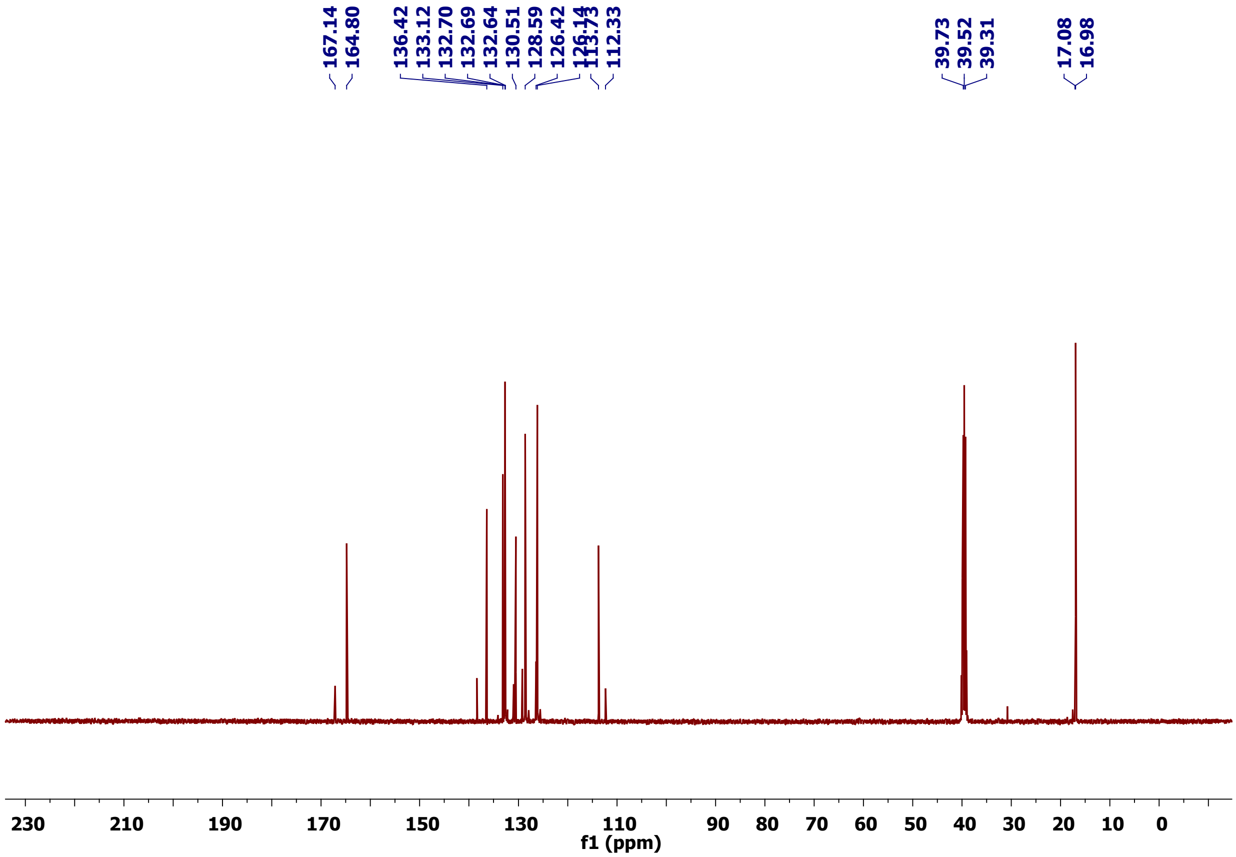

^{13}C CRAPT NMR (DMSO-d6) spectrum of (5-chloro-2-methylphenyl)carbamothioyl cyanide (1a’)

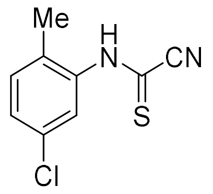

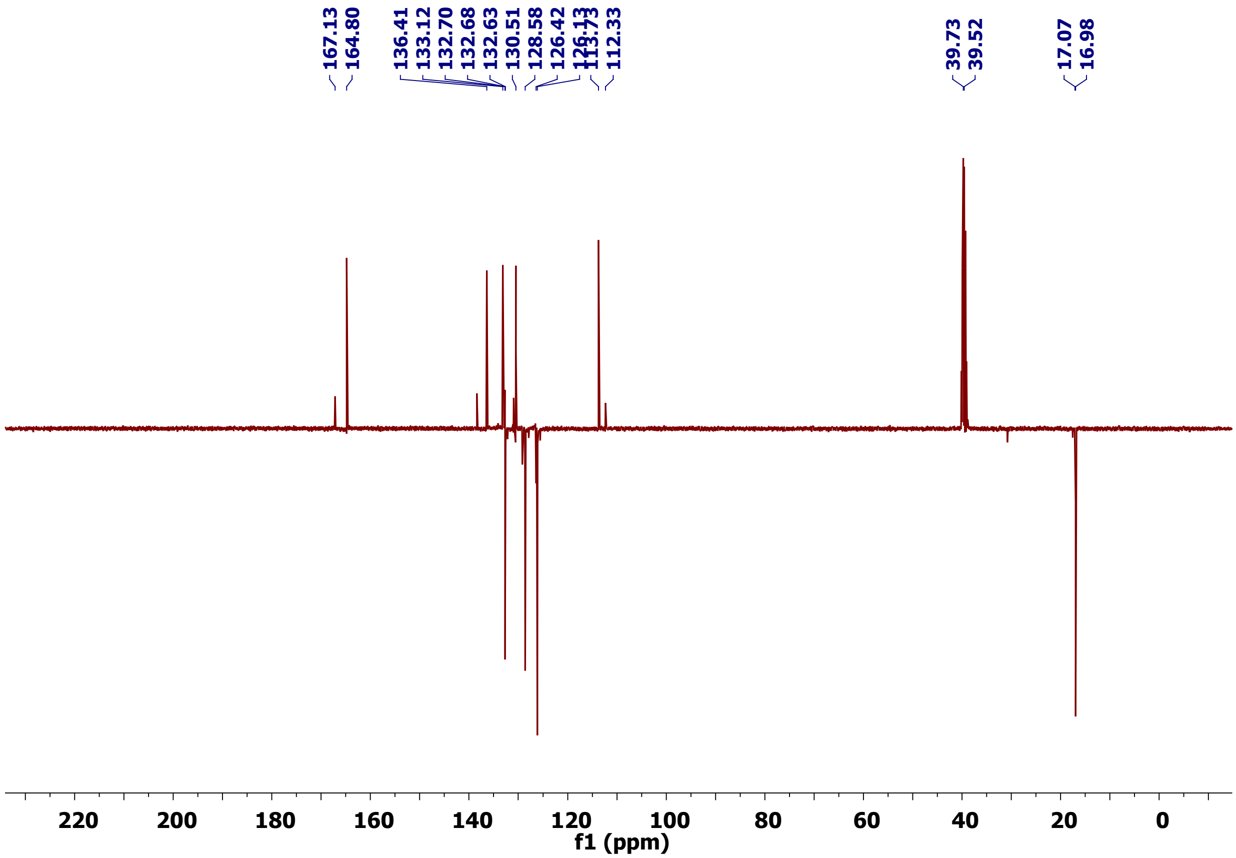

^{1}H-^{1}H-gDQCOSY NMR (DMSO-d6) spectrum of (5-chloro-2-methylphenyl)carbamothioyl cyanide (1a’)

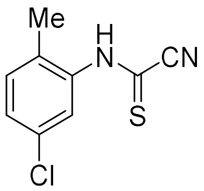

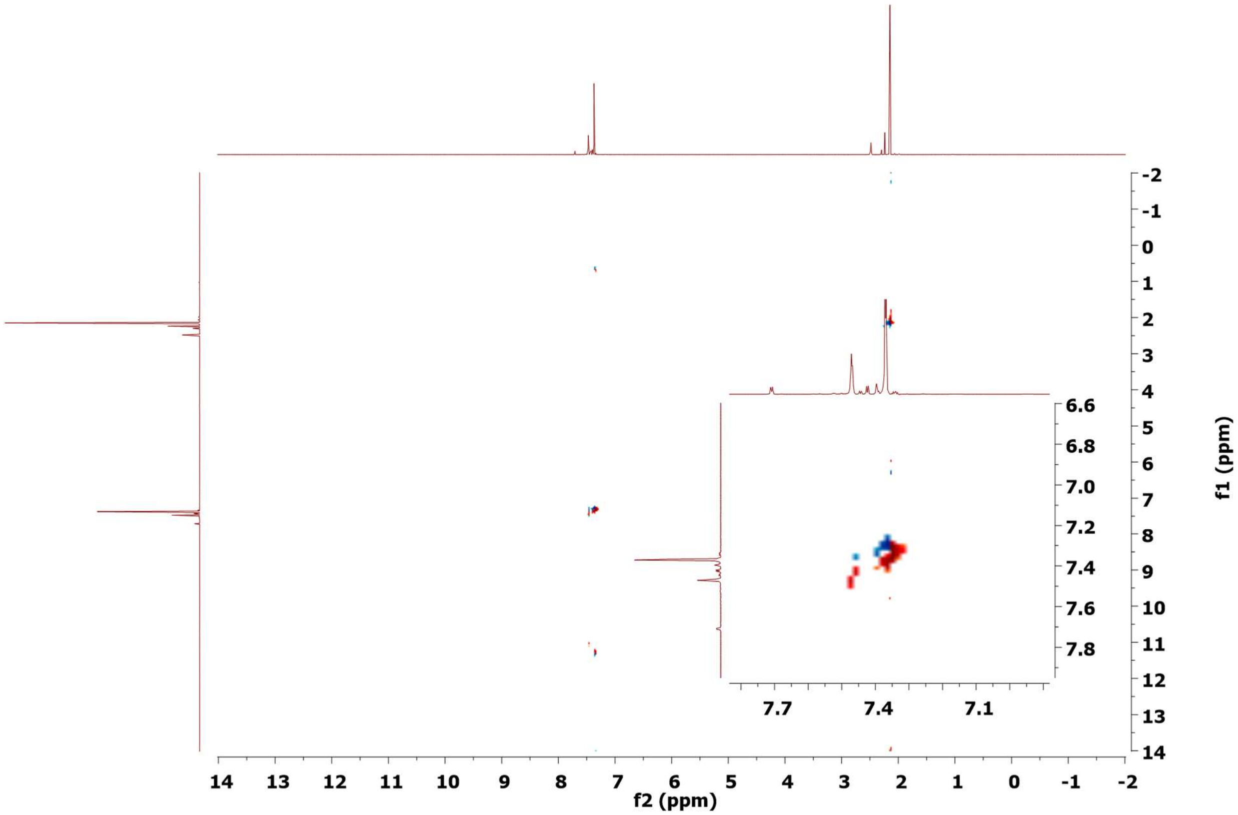

^{1}H-^{13}C-gHSQC NMR (DMSO-d6) spectrum of (5-chloro-2-methylphenyl)carbamothioyl cyanide (1a’)

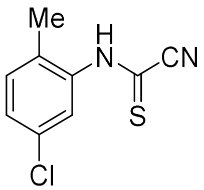

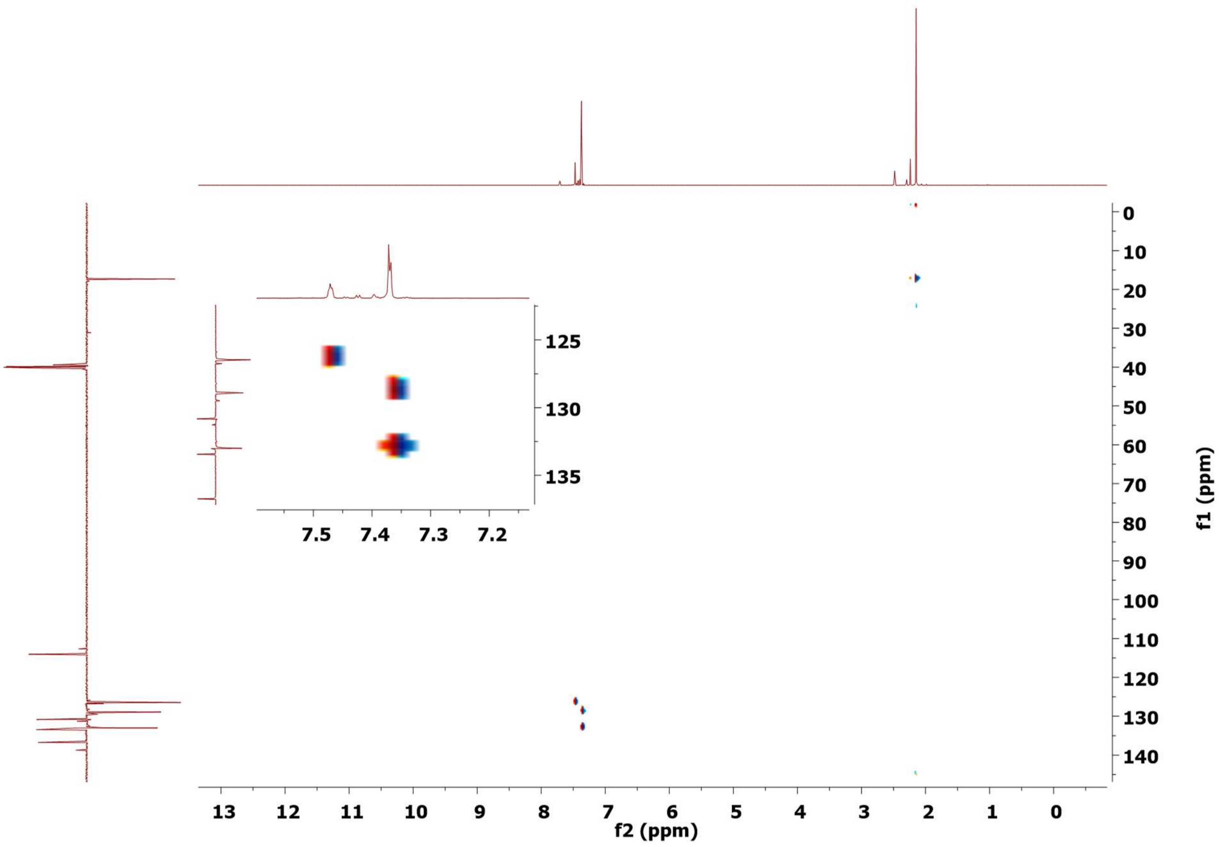

^{1}H-^{13}C-gHMBC NMR (DMSO-d6) spectrum of (5-chloro-2-methylphenyl)carbamothioyl cyanide (1a’)

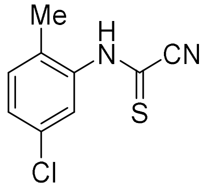

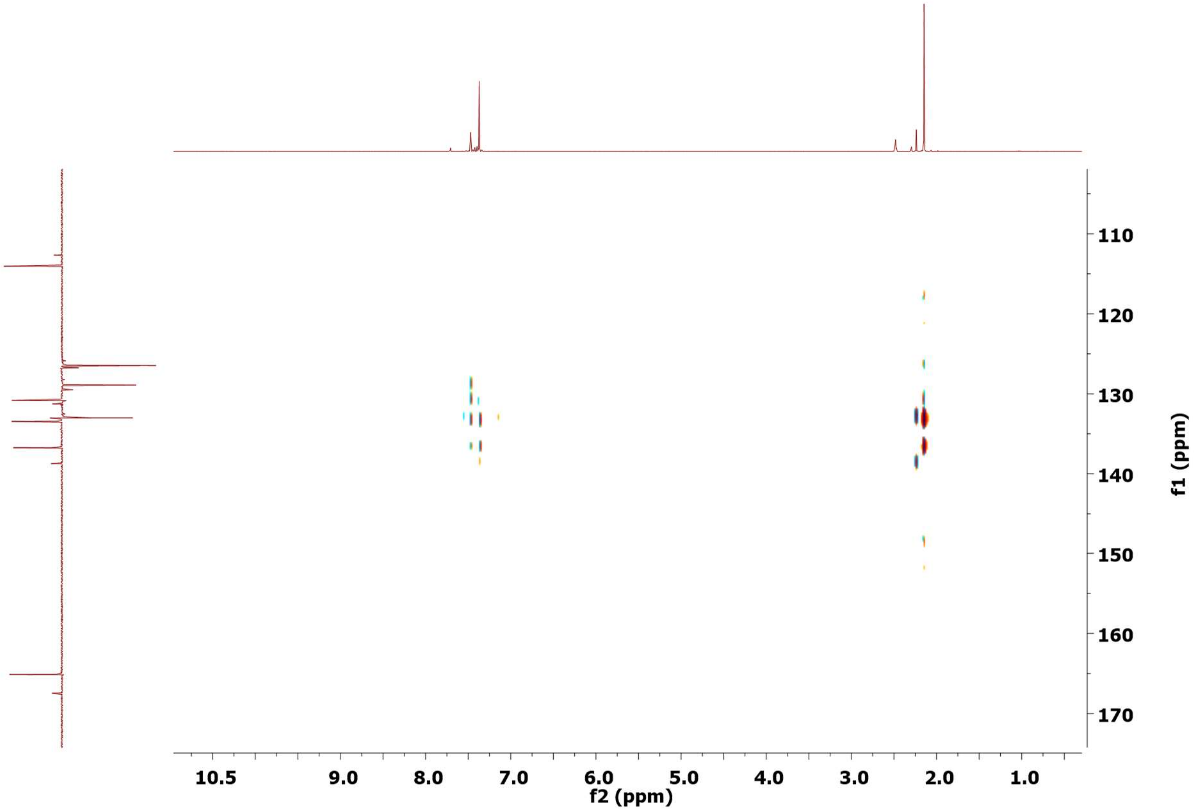

^{1}H NMR (DMSO-d6) spectrum of (2,4-dimethylphenyl)carbamothioyl cyanide (1b')

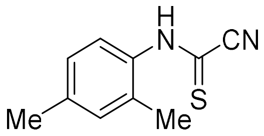

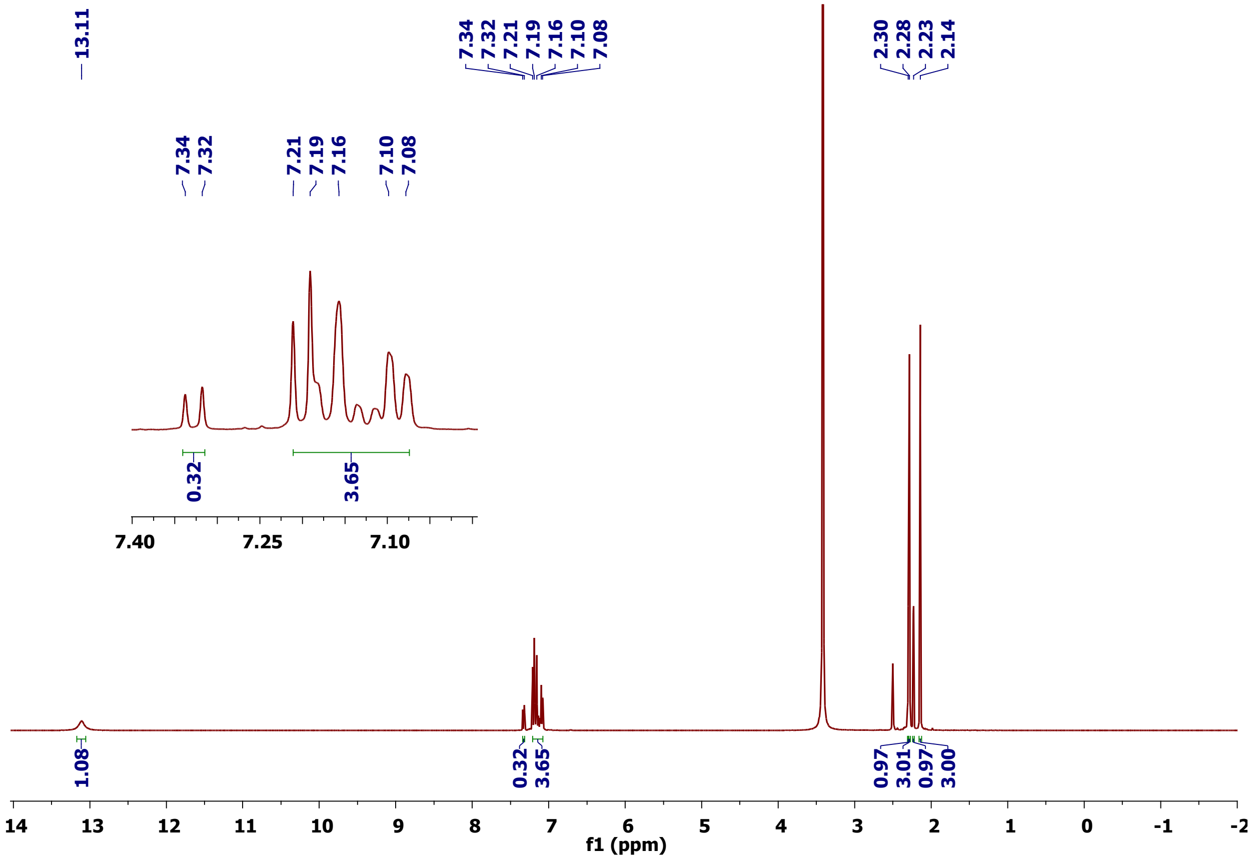

^{13}C NMR (DMSO-d6) spectrum of (2,4-dimethylphenyl)carbamothioyl cyanide (1b')

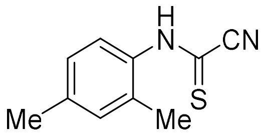

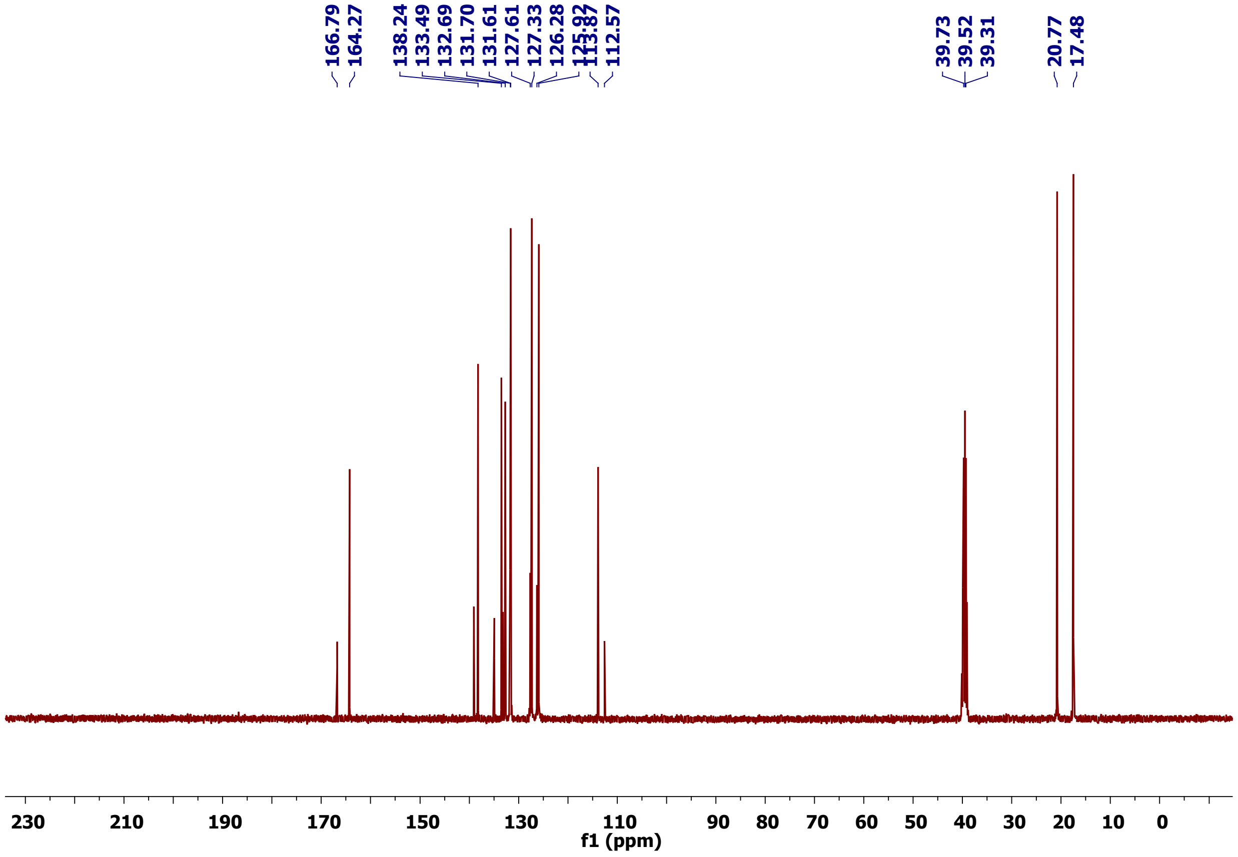

^{13}C CRAPT NMR (DMSO-d6) spectrum of (2,4-dimethylphenyl)carbamothioyl cyanide (1b')

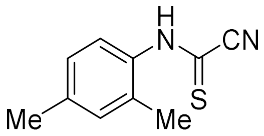

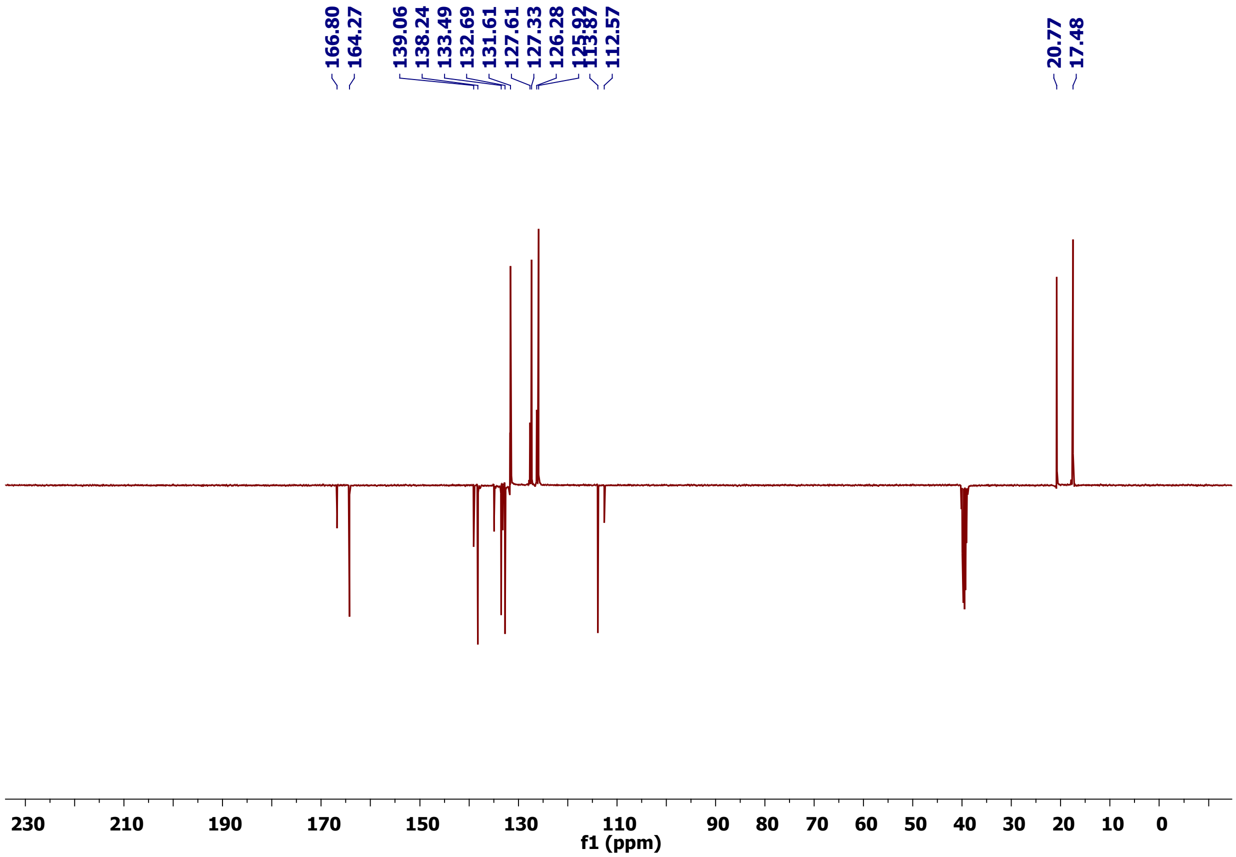

^{1}H-^{1}H gDQCOSY NMR (DMSO-d6) spectrum of (2,4-dimethylphenyl)carbamothioyl cyanide (1b')

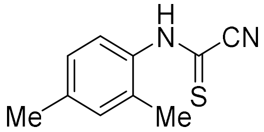

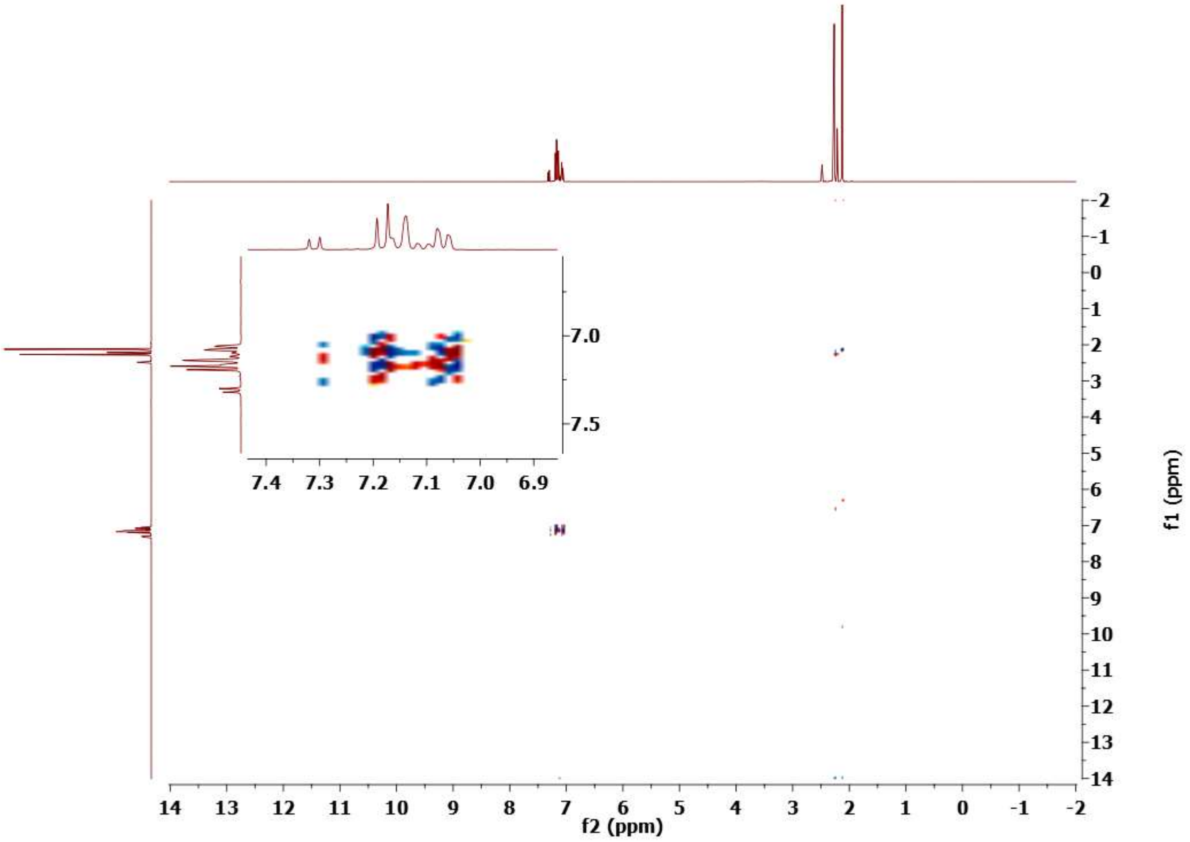

^{1}H-^{13}C-gHSQC NMR (DMSO-d6) spectrum of (2,4-dimethylphenyl)carbamothioyl cyanide (1b’)

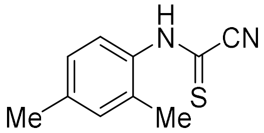

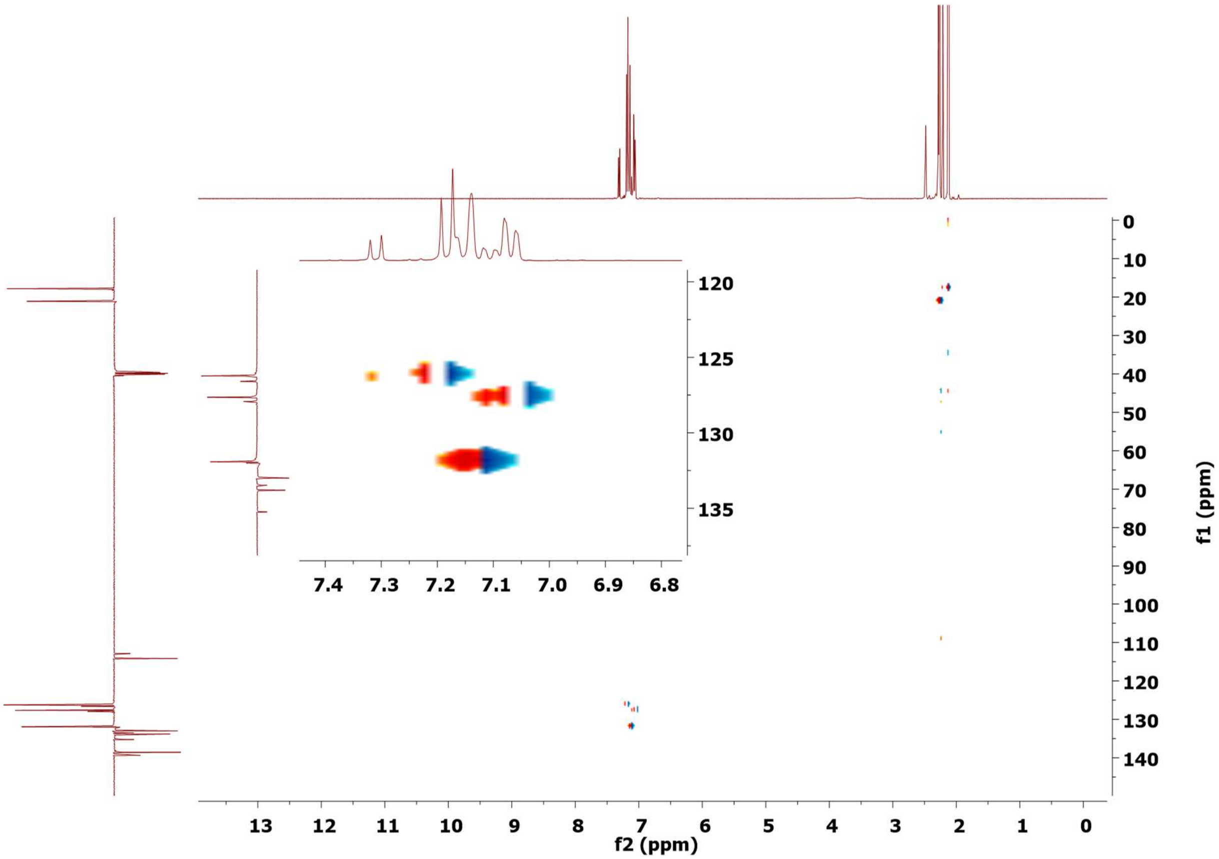

^{1}H-^{13}C-gHMBC NMR (DMSO-d6) spectrum of (2,4-dimethylphenyl)carbamothioyl cyanide (1b’)

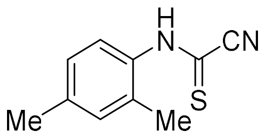

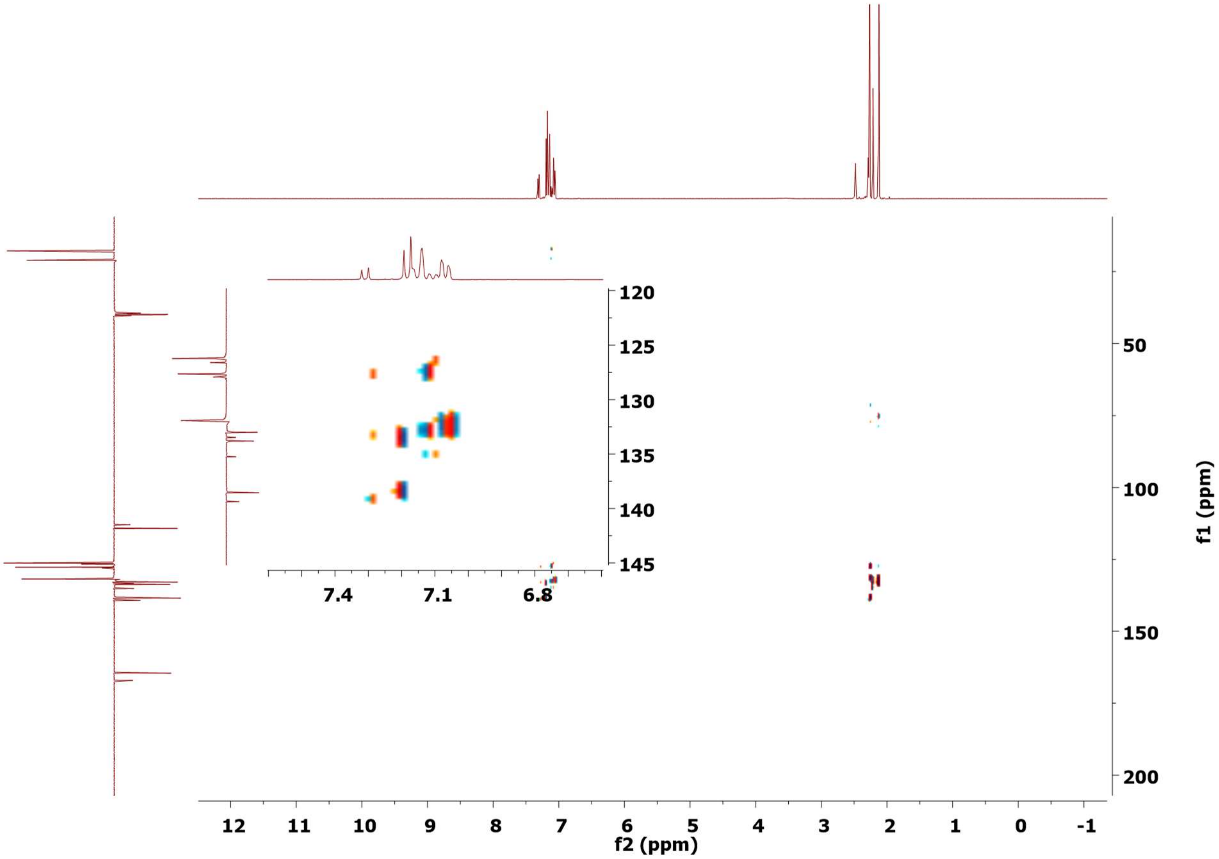

^{1}H NMR (DMSO-d6) spectrum of mesitylcarbamothioyl cyanide (1c’)

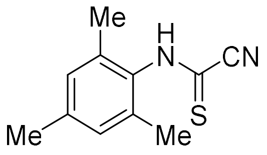

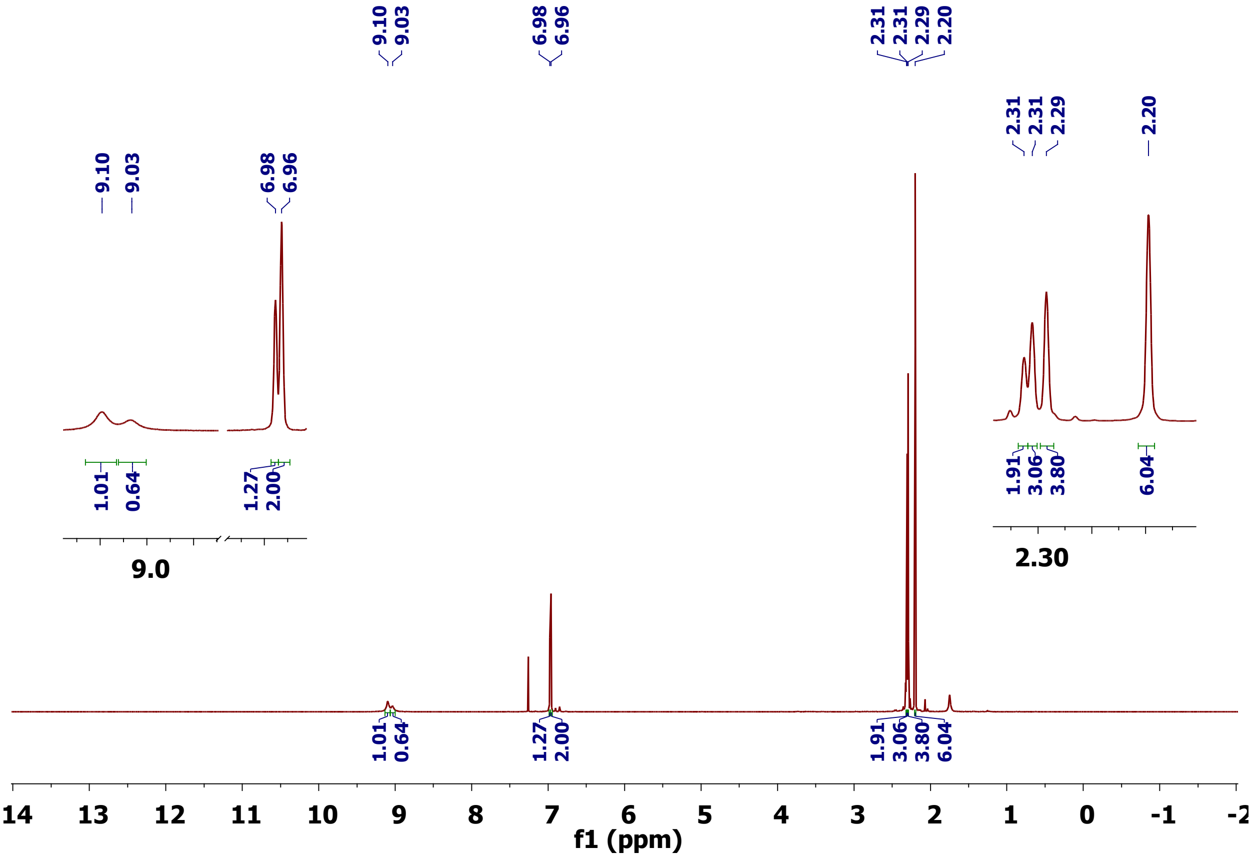

^{13}C NMR (DMSO-d6) spectrum of mesitylcarbamothioyl cyanide (1c')

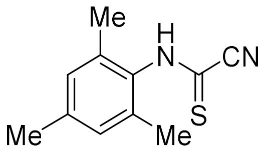

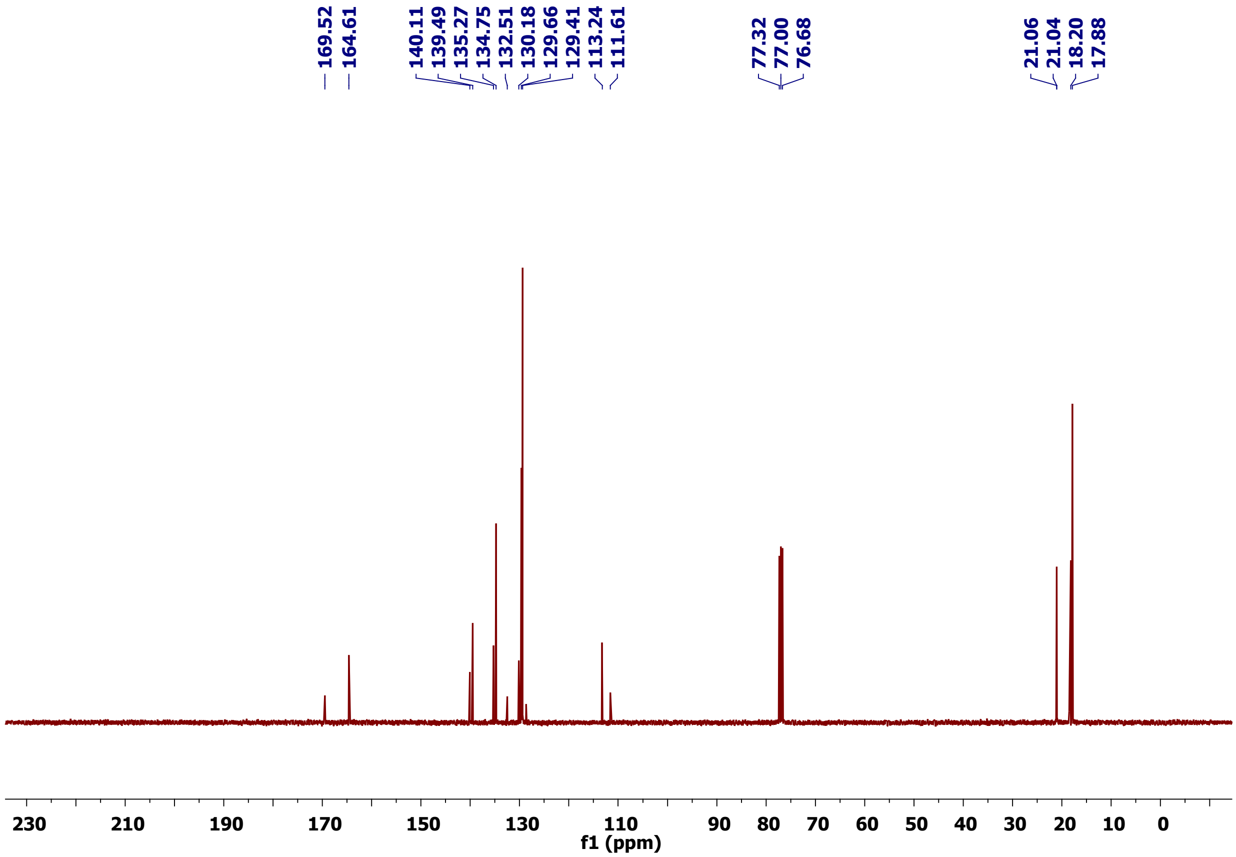

^{13}C CRAPT NMR (DMSO-d6) spectrum of mesitylcarbamothioyl cyanide (1c')

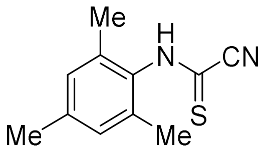

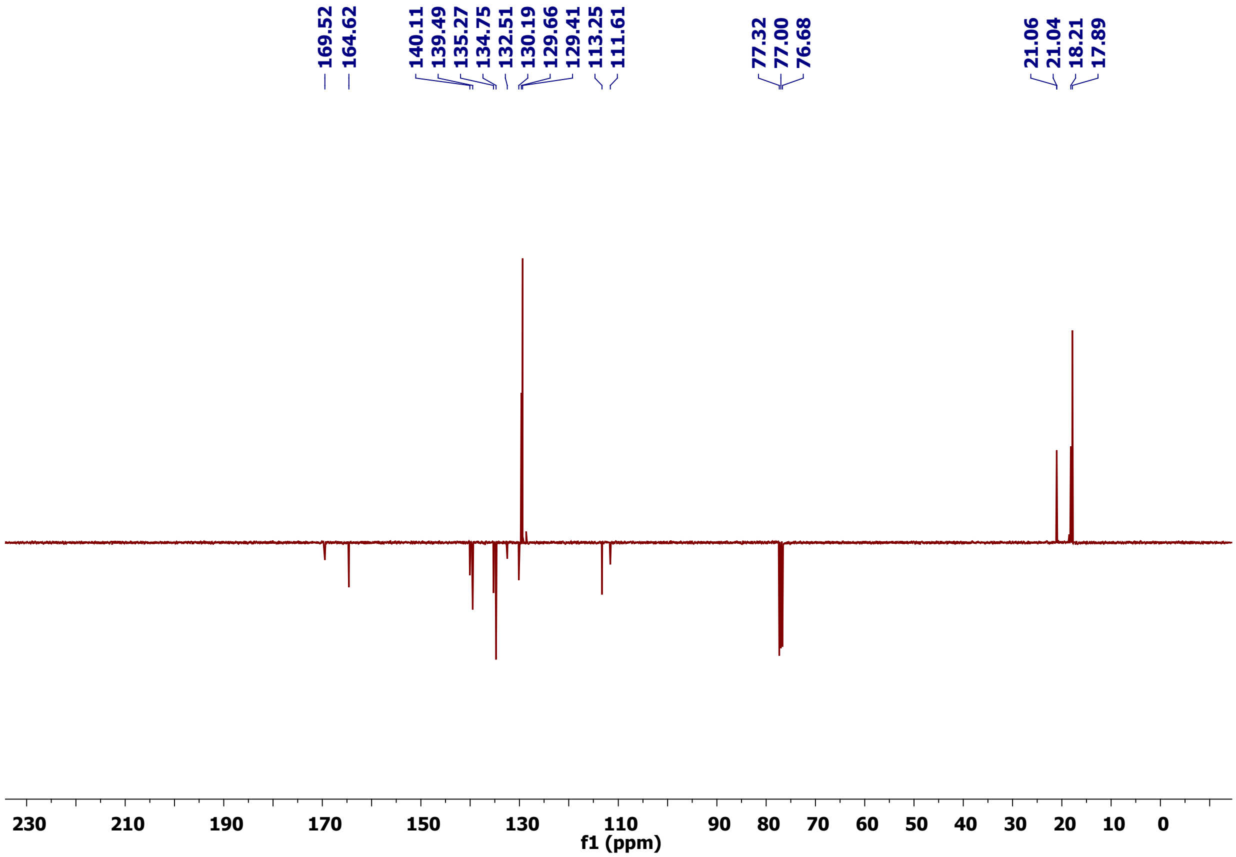

^{1}H-^{13}C-gHSQC NMR (DMSO-d6) spectrum of mesitylcarbamothioyl cyanide (1c')

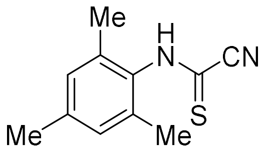

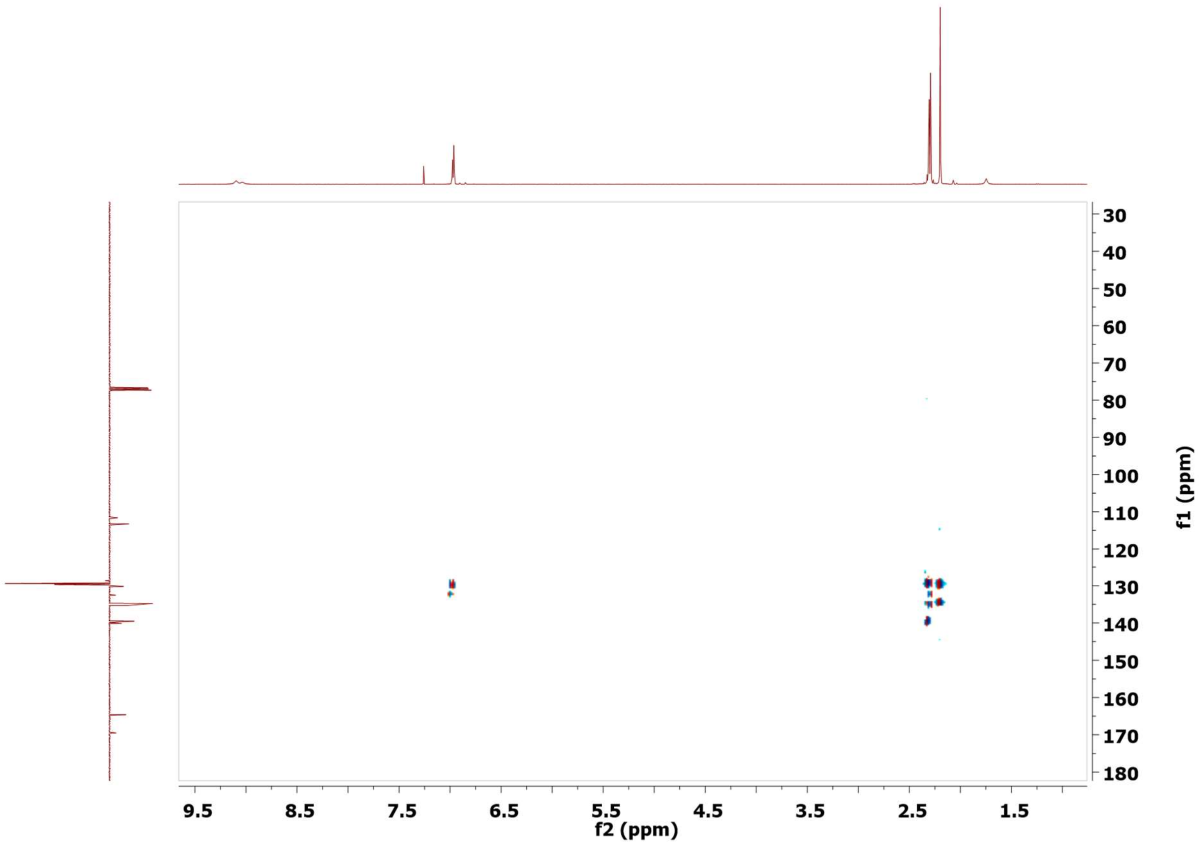

^{1}H-^{13}C-gHMBC NMR (DMSO-d6) spectrum of mesitylcarbamothioyl cyanide (1c')

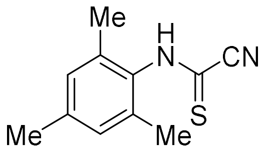

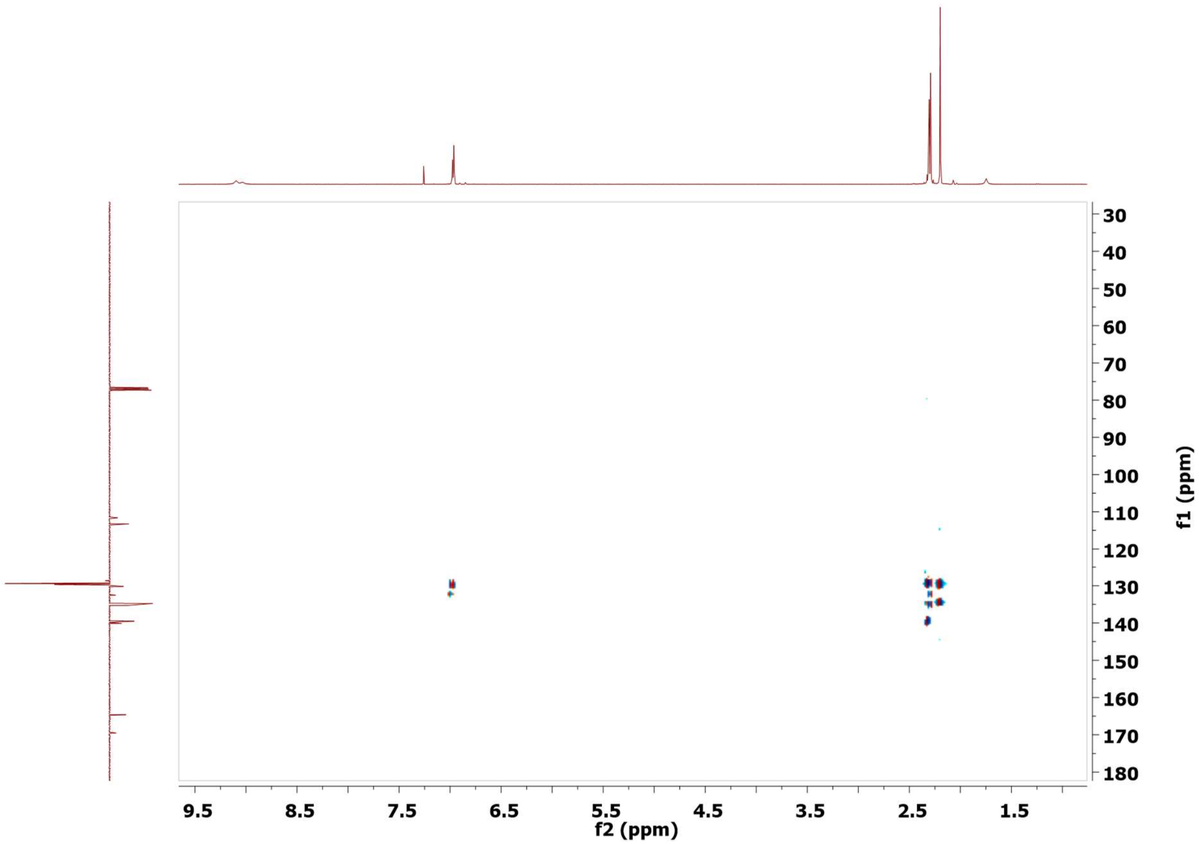

^{1}H NMR (DMSO-d6) spectrum of (2,3-dichlorophenyl)carbamothioyl cyanide (1d')

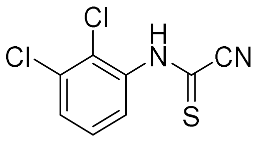

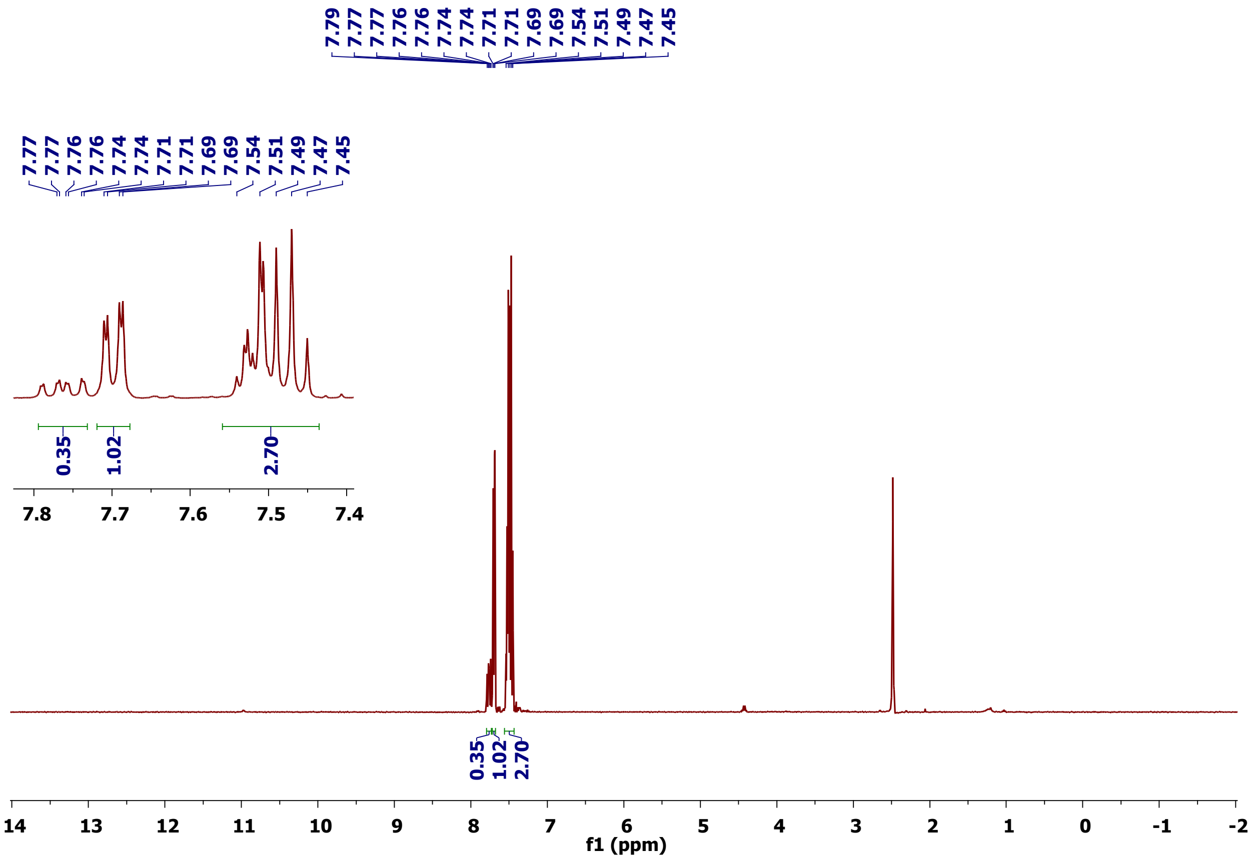

^{13}C NMR (DMSO-d6) spectrum of (2,3-dichlorophenyl)carbamothioyl cyanide (1d')

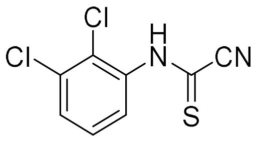

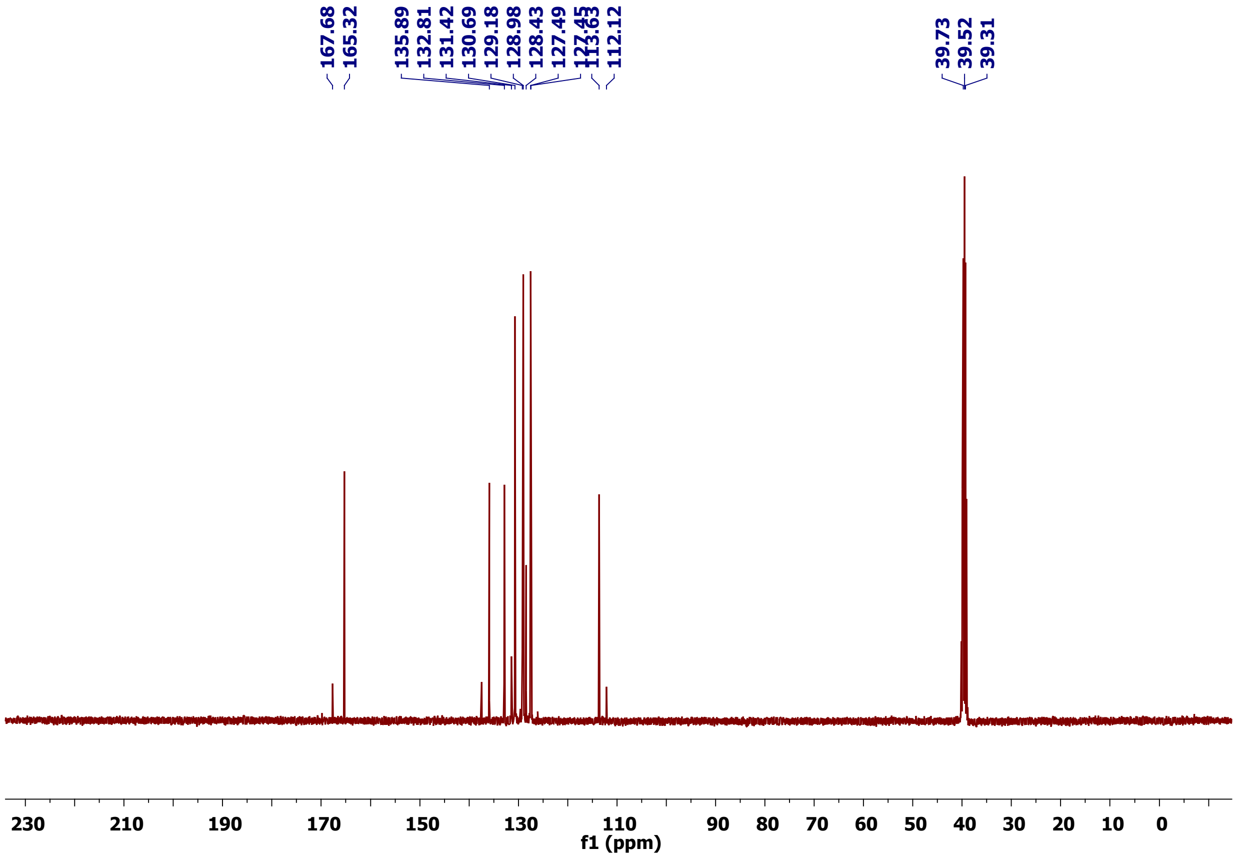

^{13}C CRAPT NMR (DMSO-d6) spectrum of (2,3-dichlorophenyl)carbamothioyl cyanide (1d')

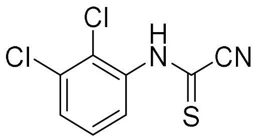

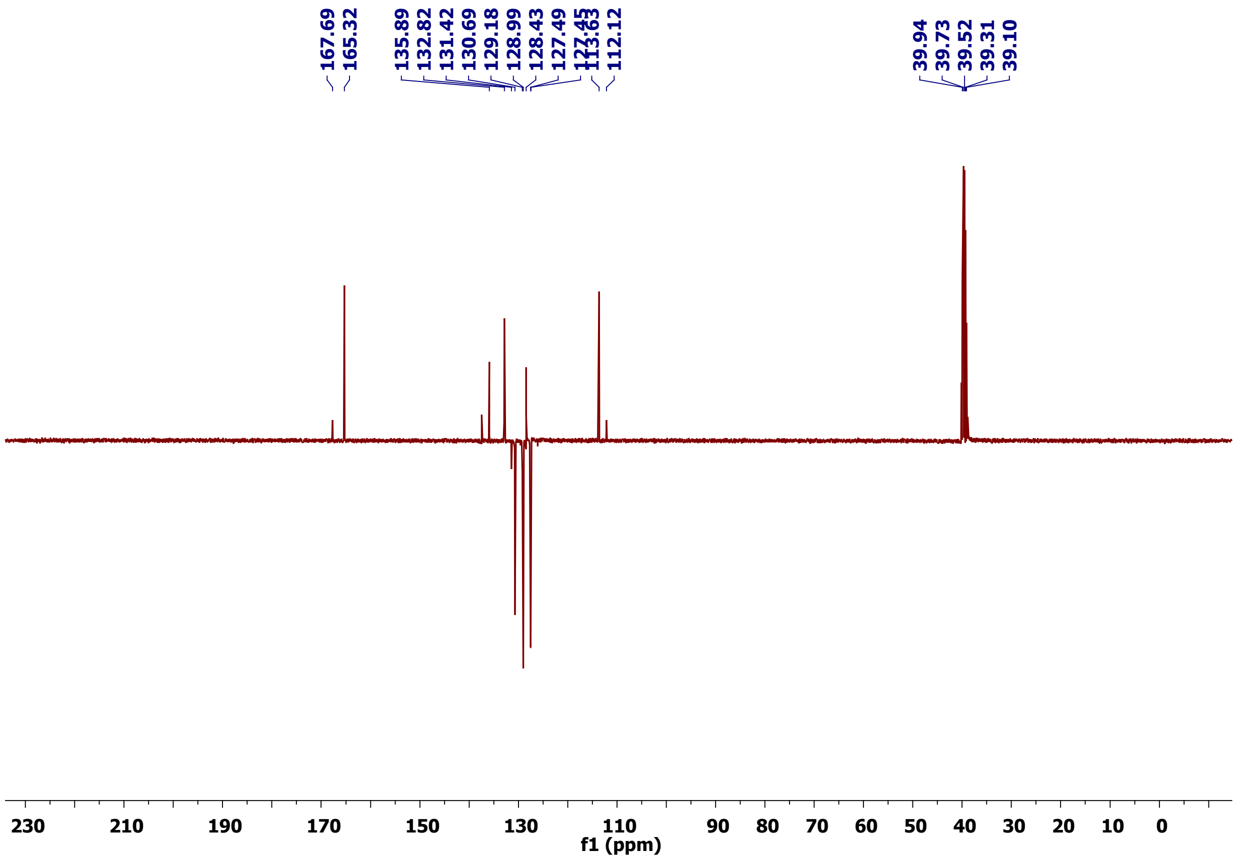

^{1}H-^{1}H-gDQCOSY NMR (DMSO-d6) spectrum of (2,3-dichlorophenyl)carbamothioyl cyanide (1d')

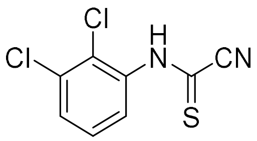

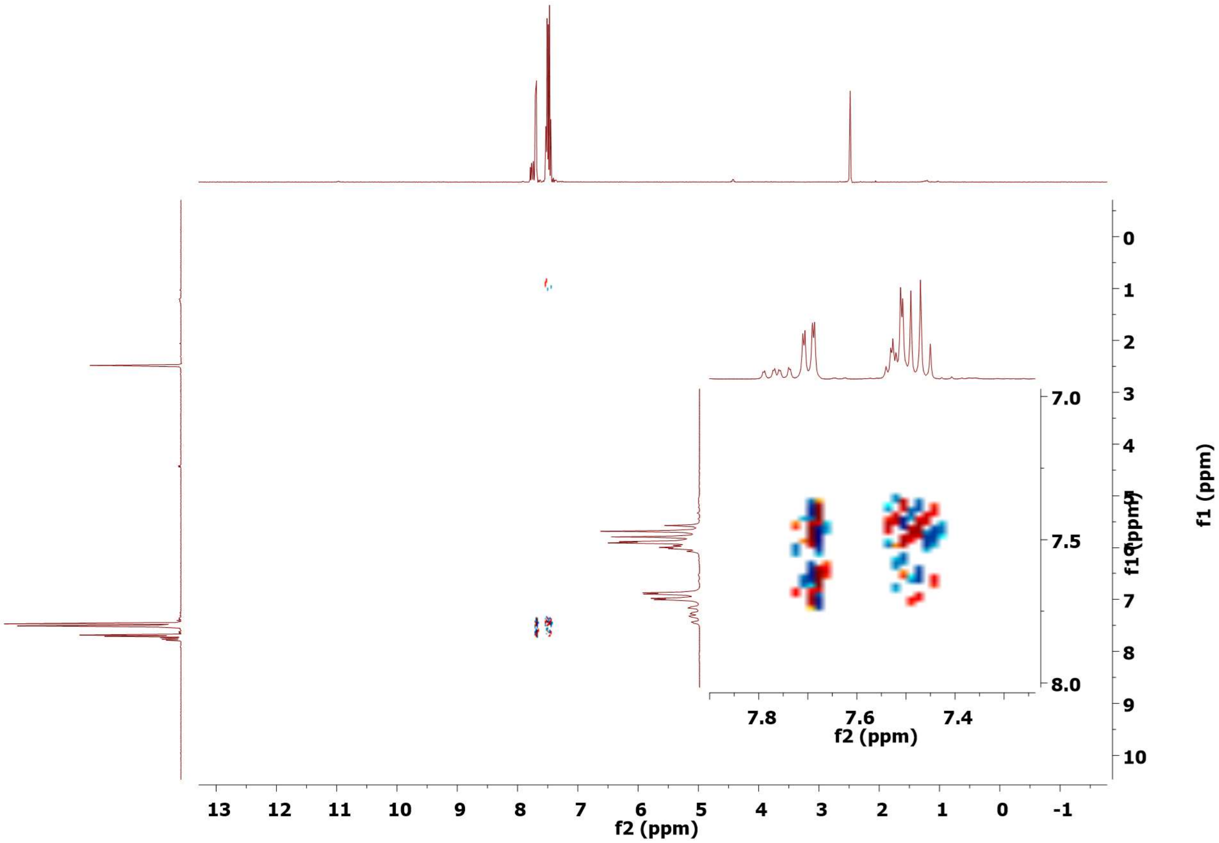

^{1}H-^{13}C-gHSQC NMR (DMSO-d6) spectrum of (2,3-dichlorophenyl)carbamothioyl cyanide (1d')

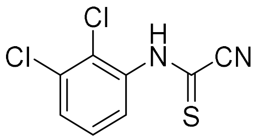

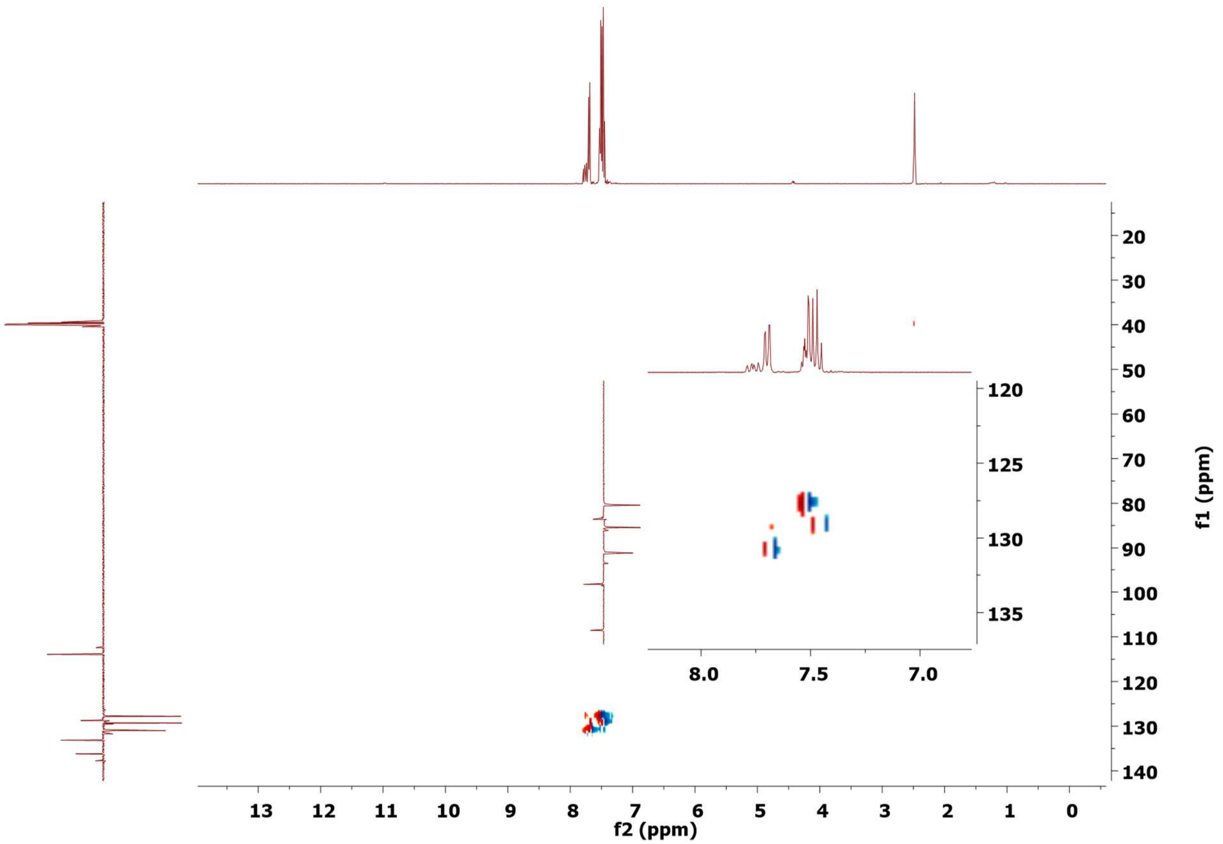

^{1}H-^{13}C-gHMBC NMR (DMSO-d6) spectrum of (2,3-dichlorophenyl)carbamothioyl cyanide (1d')

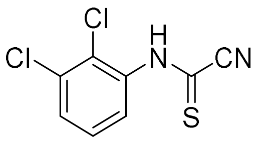

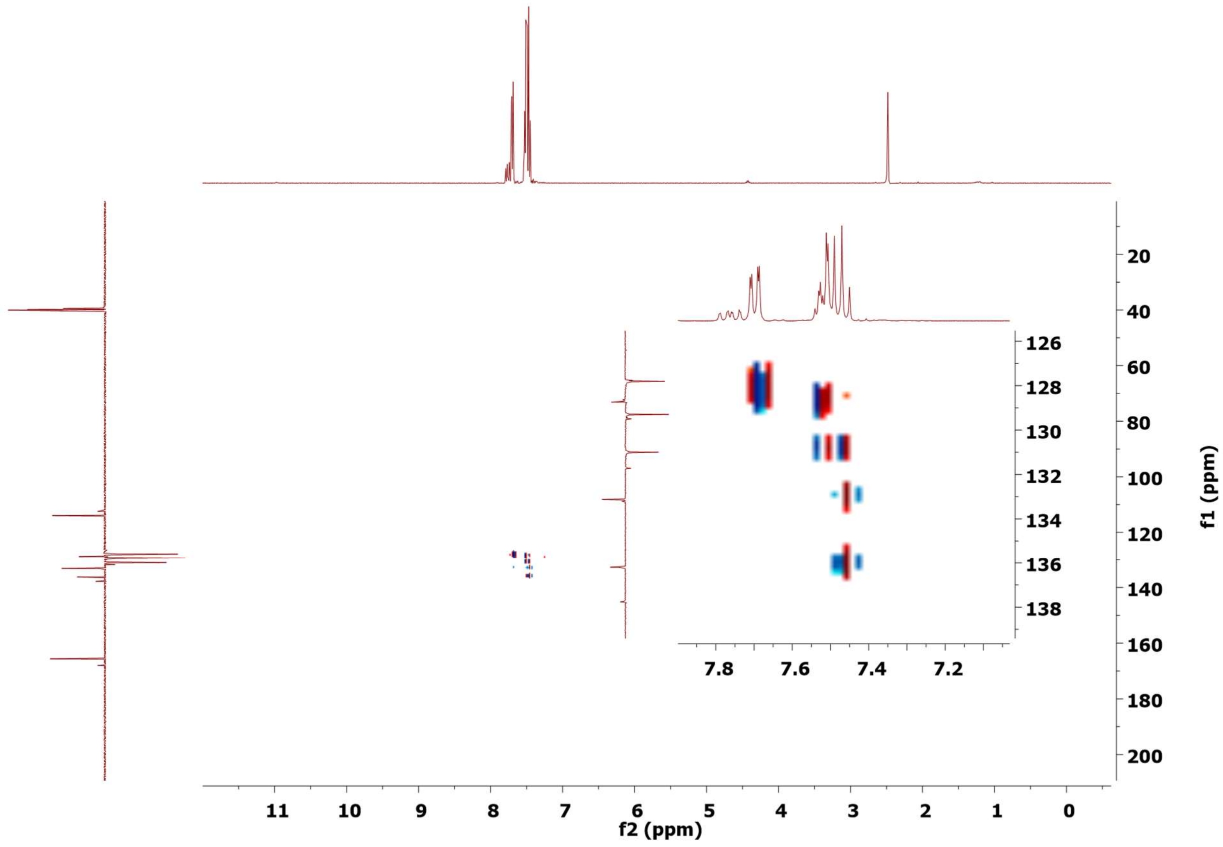

^{1}H NMR (DMSO-d6) spectrum of (2-chloro-5-(trifluoromethyl)phenyl)carbamothioyl cyanide (1:0.17 tautomeric ratio) (1e')

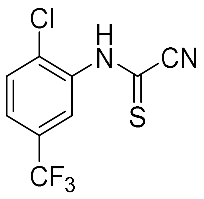

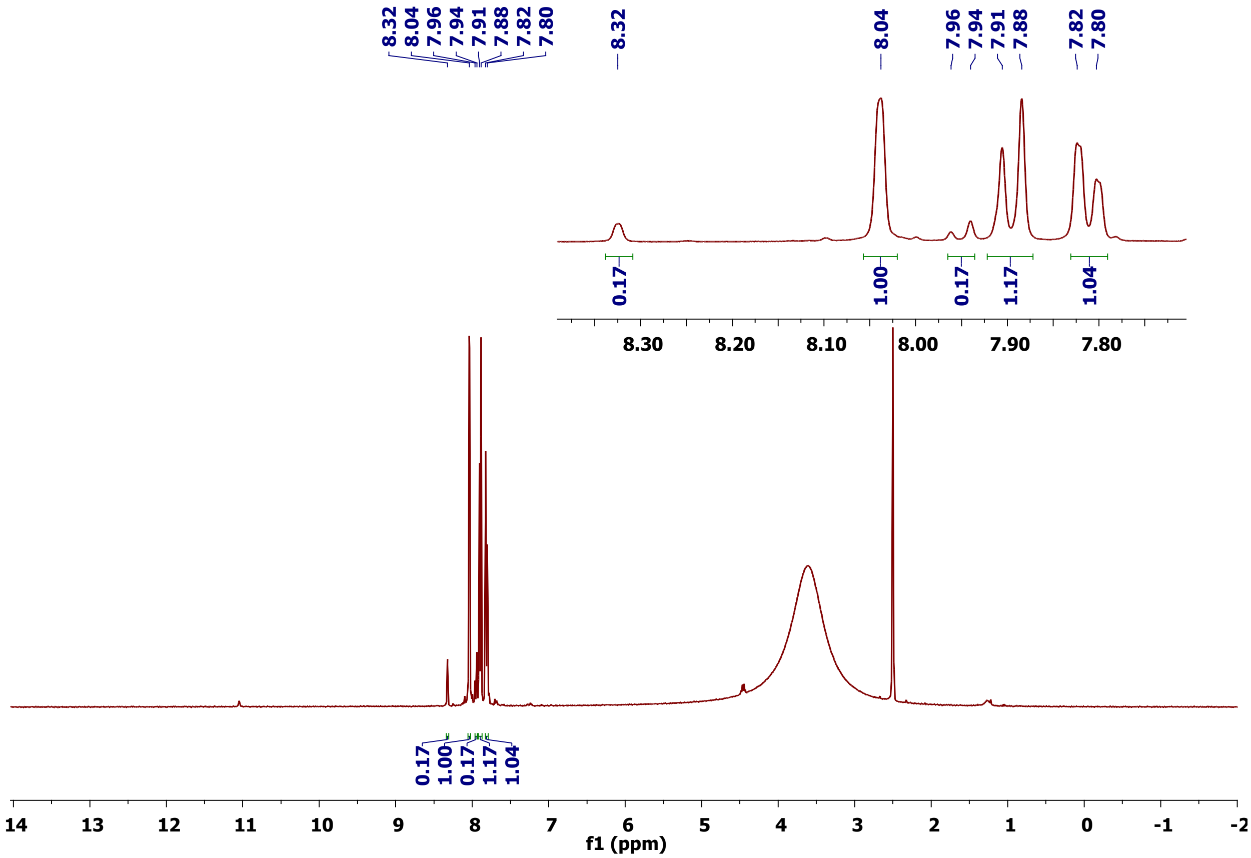

^{13}C NMR (DMSO-d6) spectrum of (2-chloro-5-(trifluoromethyl)phenyl)carbamothioyl cyanide (1:0.17 tautomeric ratio) (1e')

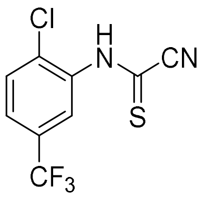

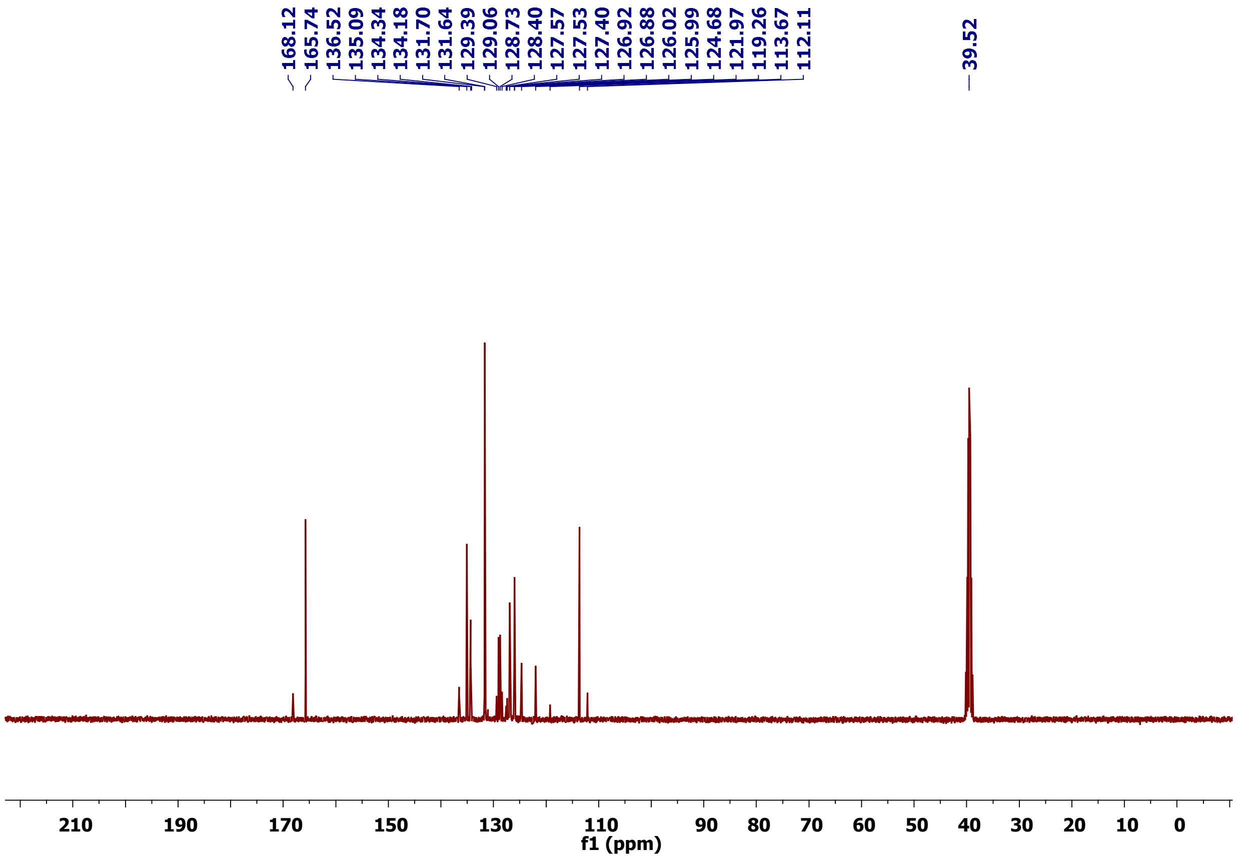

^{13}C CRAPT NMR (DMSO-d6) spectrum of (2-chloro-5-(trifluoromethyl)phenyl)carbamothioyl cyanide (1:0.17 tautomeric ratio) (1e’)

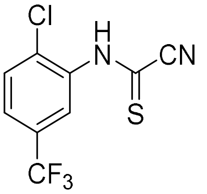

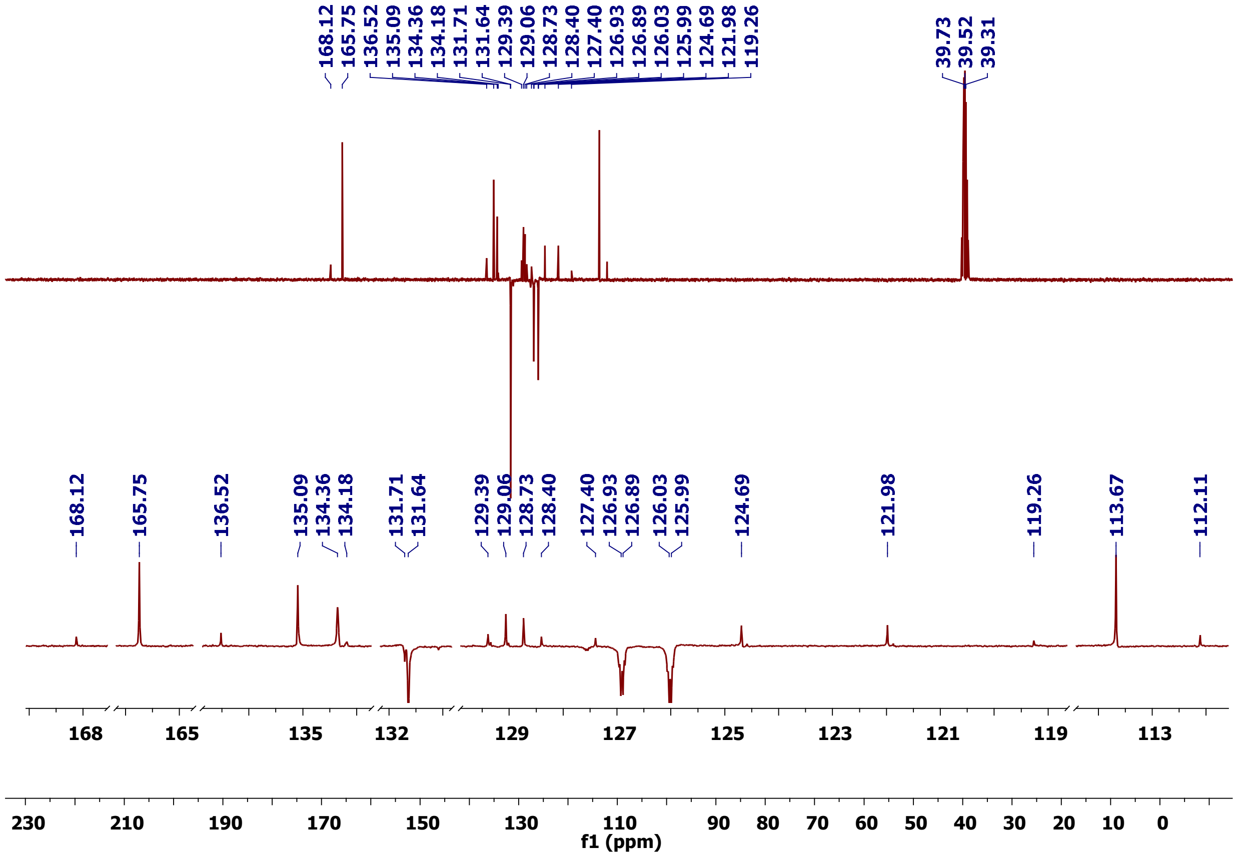

^{1}H-^{1}H-gDQCOSY NMR (DMSO-d6) spectrum of (2-chloro-5-(trifluoromethyl)phenyl)carbamothioyl cyanide (1:0.17 tautomeric ratio) (1e')

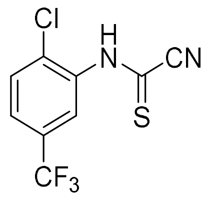

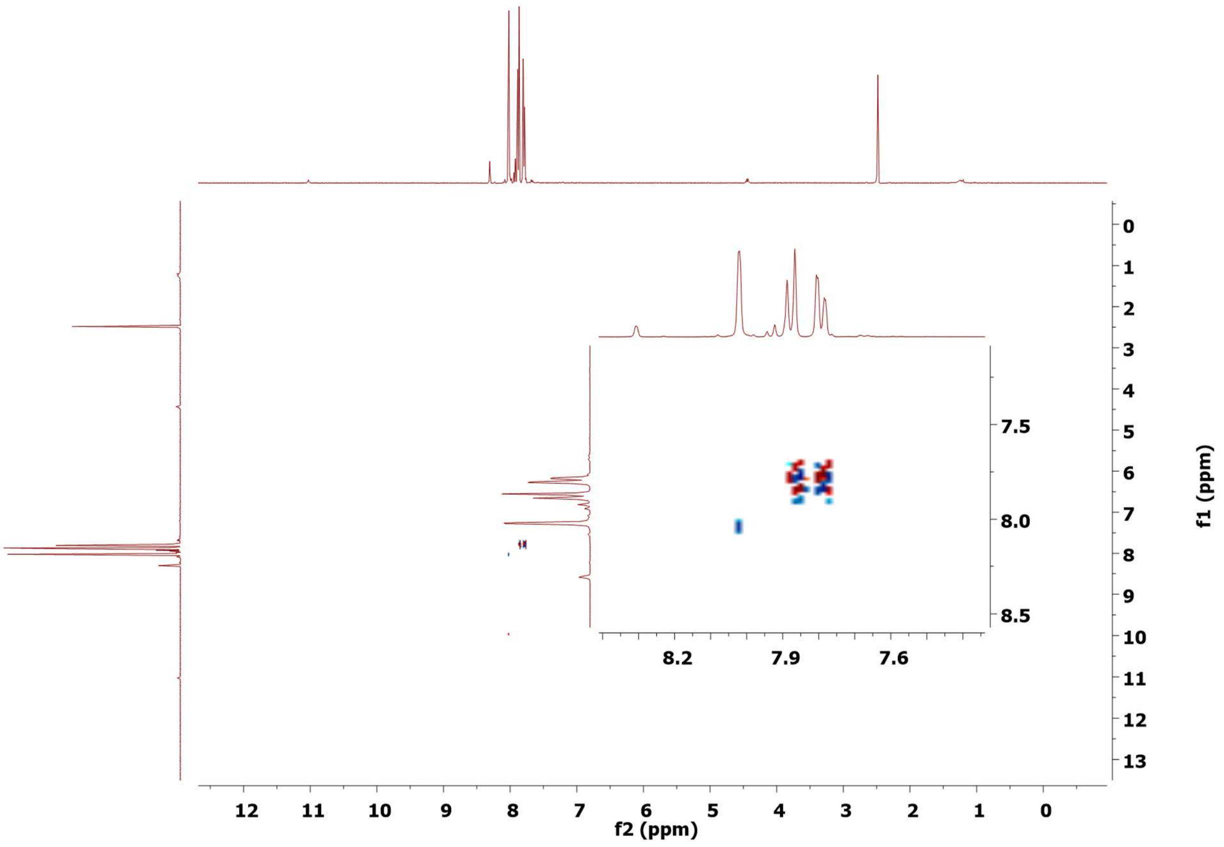

^{1}H-^{13}C-gHSQC NMR (DMSO-d6) spectrum of (2-chloro-5-(trifluoromethyl)phenyl)carbamothioyl cyanide (1:0.17 tautomeric ratio) (1e')

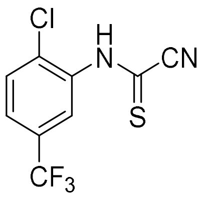

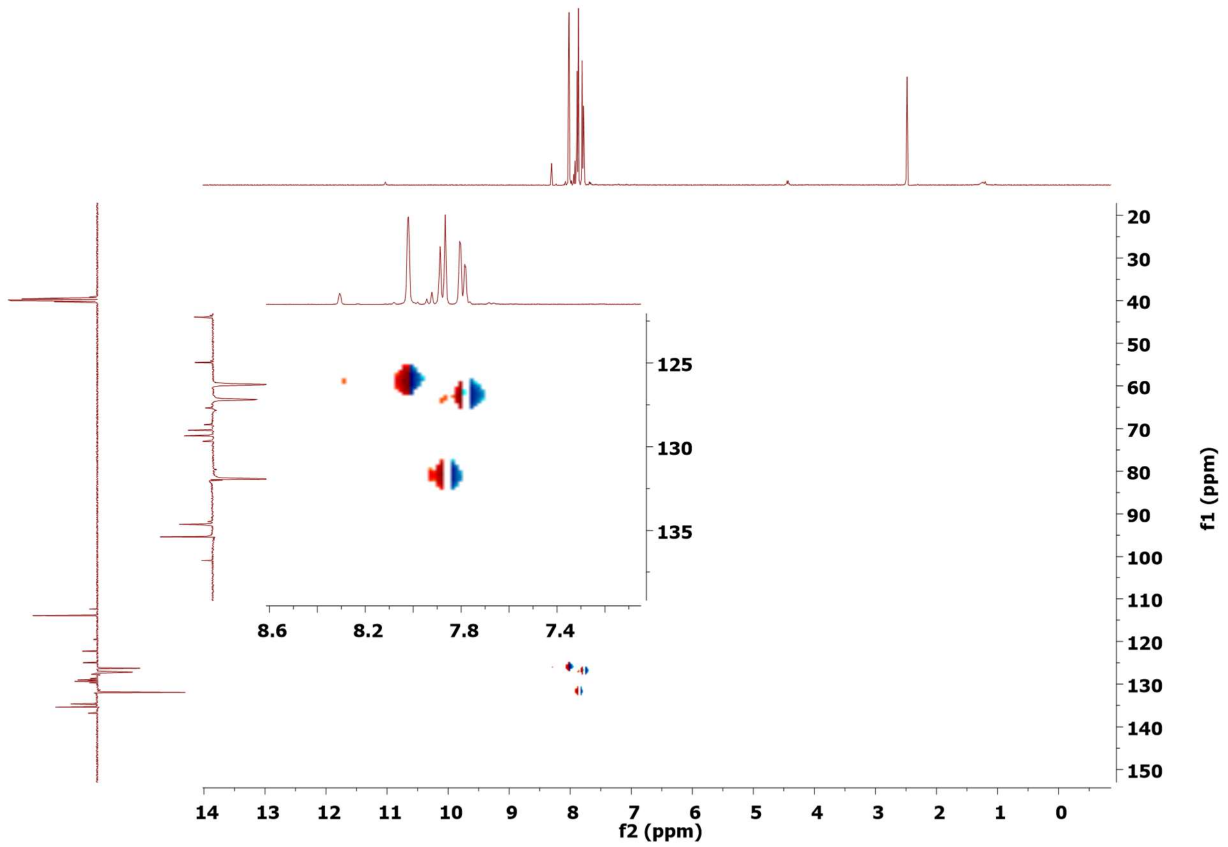

^{1}H-^{13}C-gHMBC NMR (DMSO-d6) spectrum of (2-chloro-5-(trifluoromethyl)phenyl)carbamothioyl cyanide (1:0.17 tautomeric ratio) (1e')

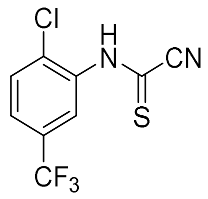

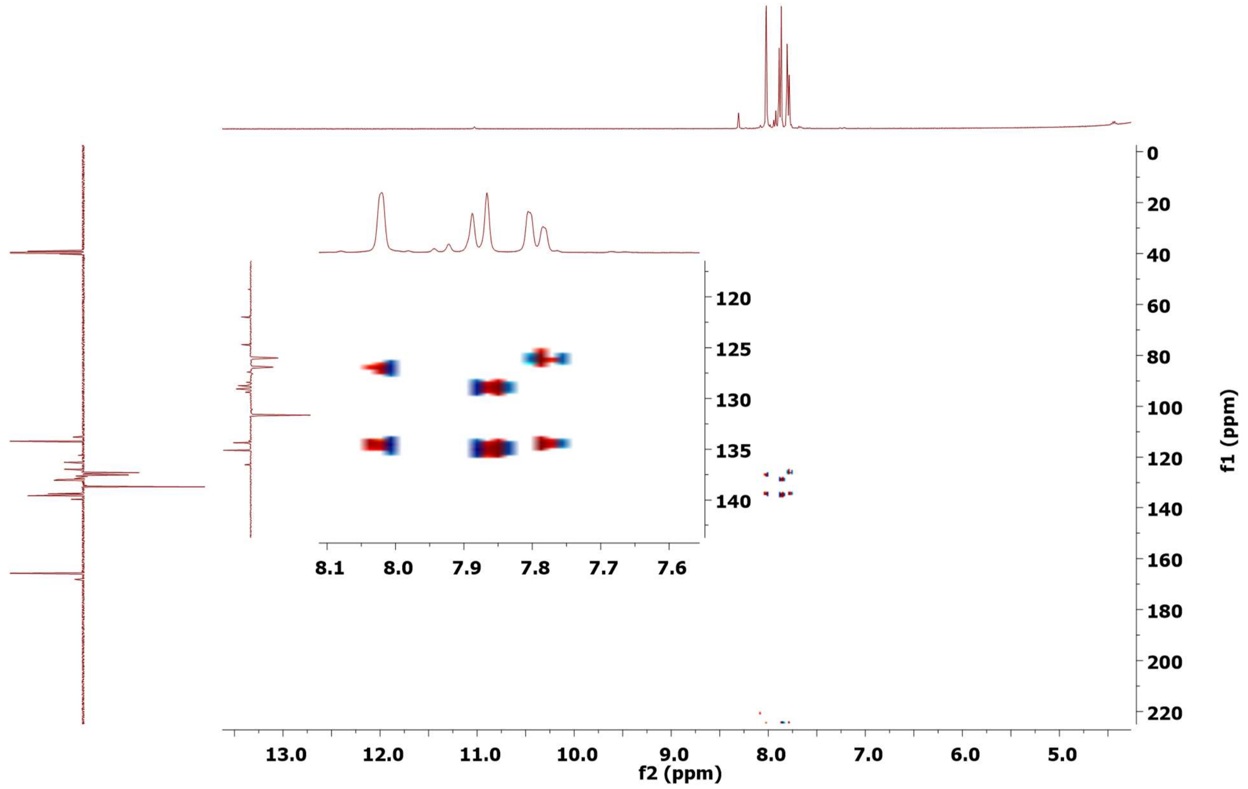

^{1}H NMR (DMSO-d6) spectrum of (3,4-dichlorophenyl)carbamothioyl cyanide (1:0.22 tautomeric ratio) (1j')

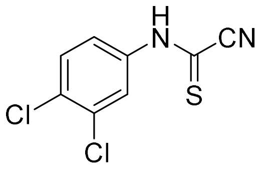

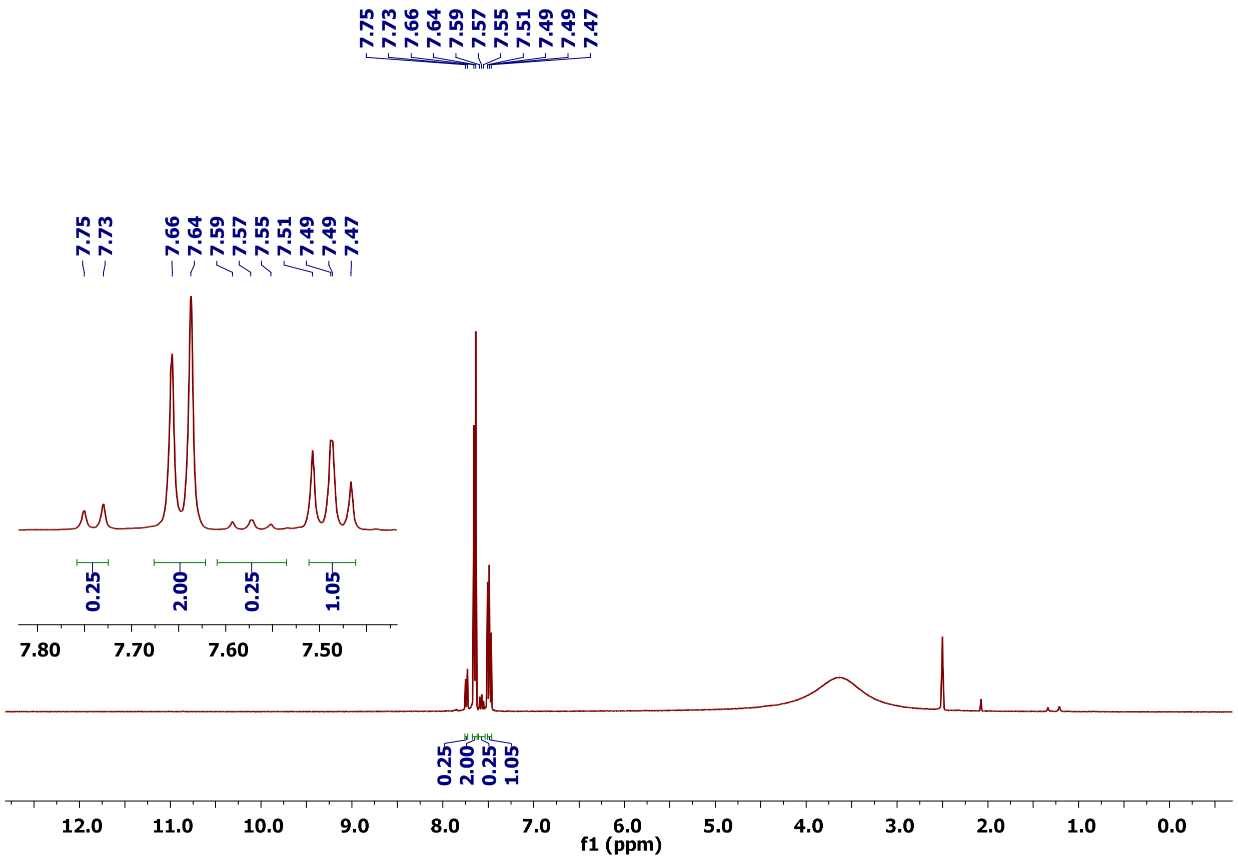

^{13}C NMR (DMSO-d6) spectrum of (2,6-dichlorophenyl)carbamothioyl cyanide (1:0.13 tautomeric ratio) (1f')

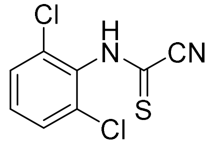

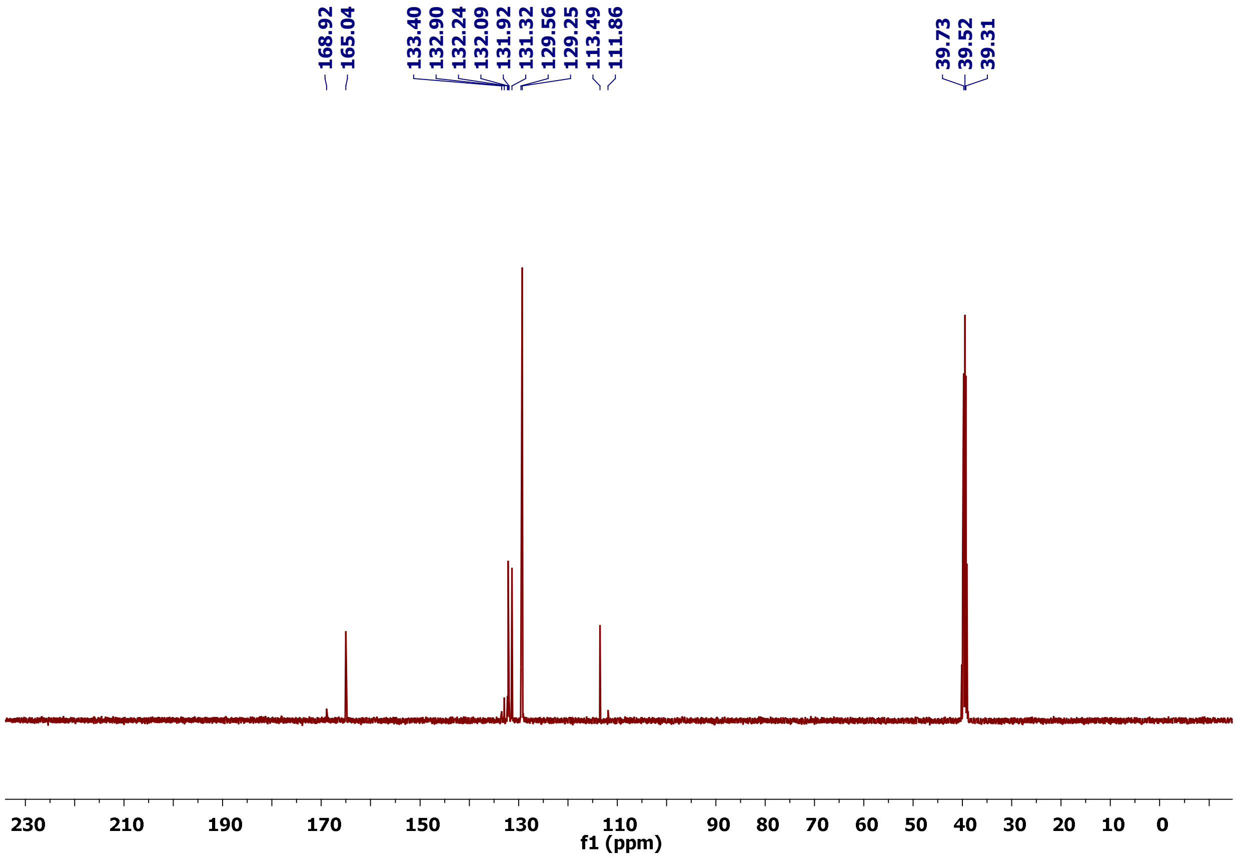

^{13}C CRAPT NMR (DMSO-d6) spectrum of (2,6-dichlorophenyl)carbamothioyl cyanide (1:0.13 tautomeric ratio) (1f’)

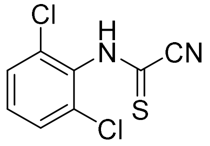

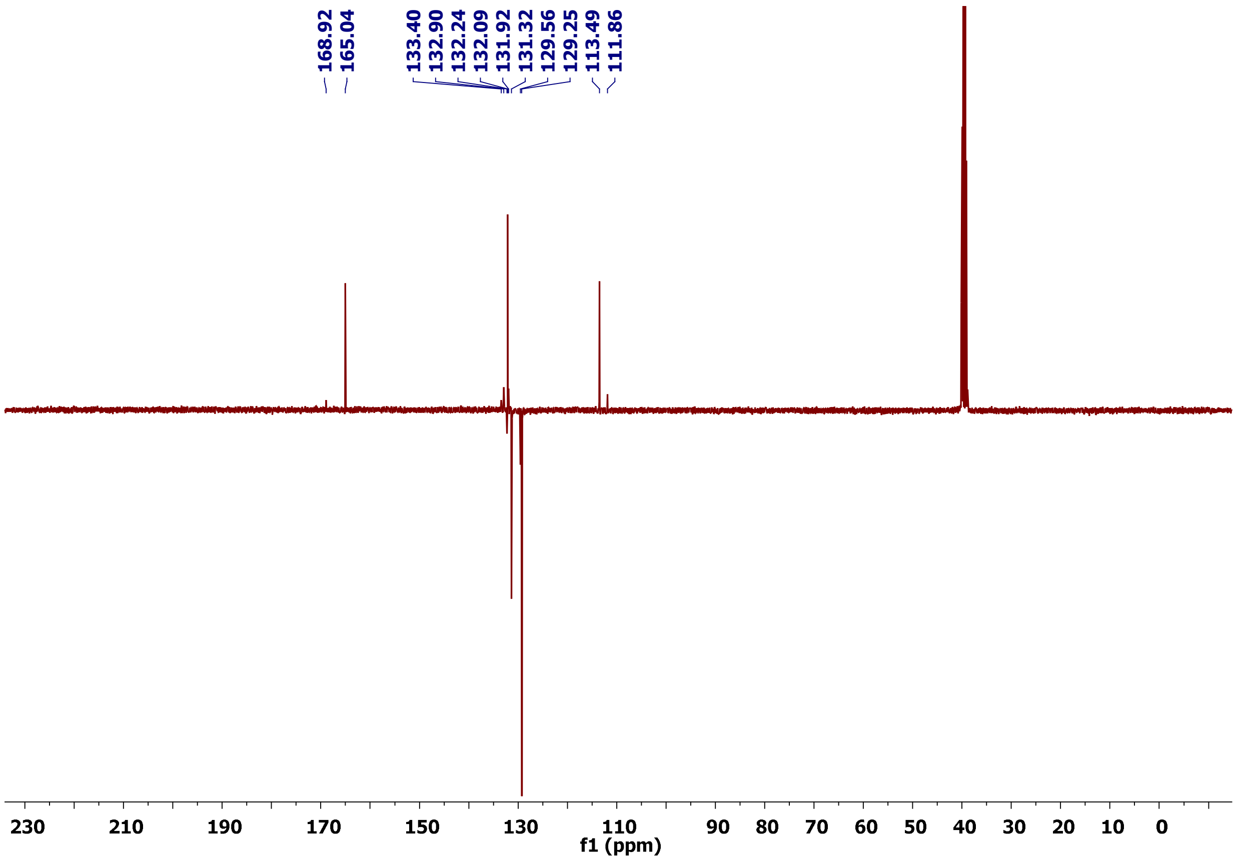

^{1}H-^{1}H-gDQCOSY NMR (DMSO-d6) spectrum of (2,6-dichlorophenyl)carbamothioyl cyanide (1:0.13 tautomeric ratio) (1f')

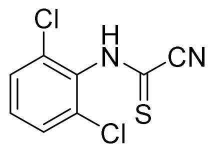

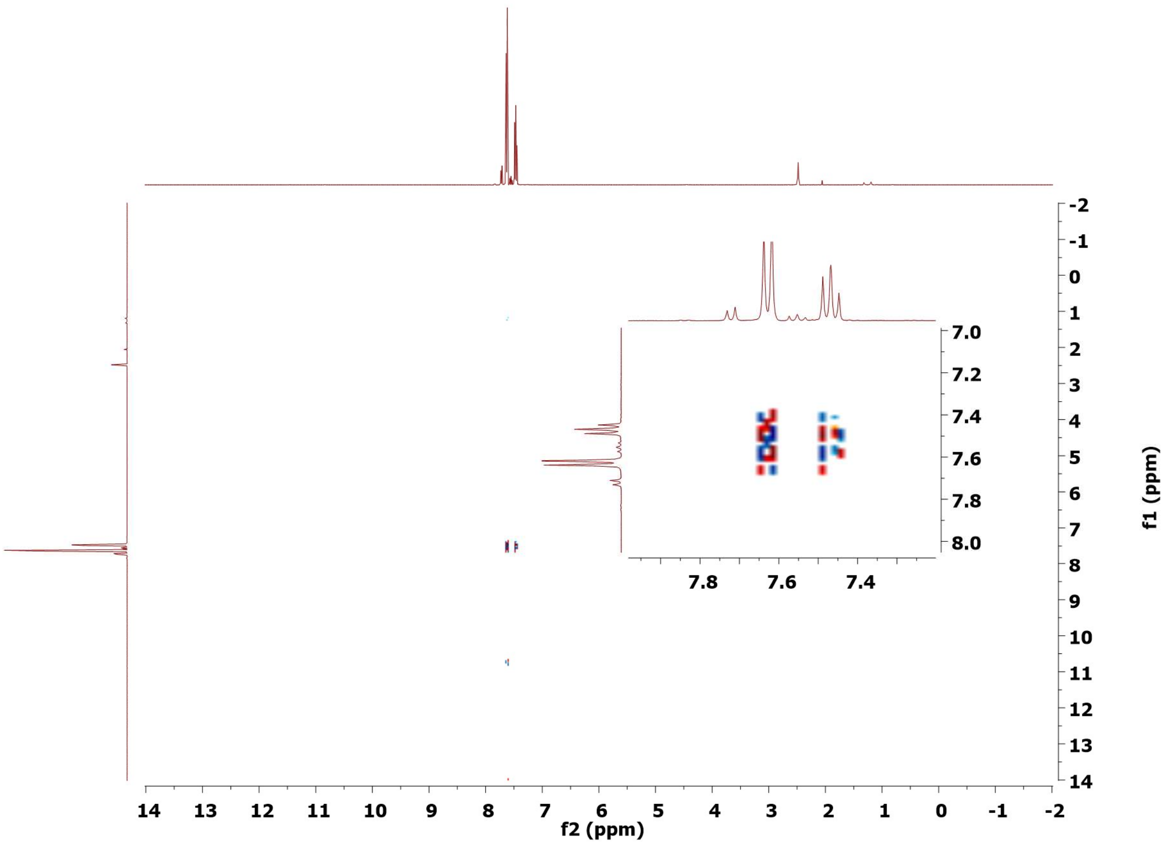

^{1}H-^{13}C-gHSQC NMR (DMSO-d6) spectrum of (2,6-dichlorophenyl)carbamothioyl cyanide (1:0.13 tautomeric ratio) (1f')

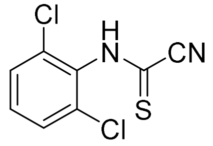

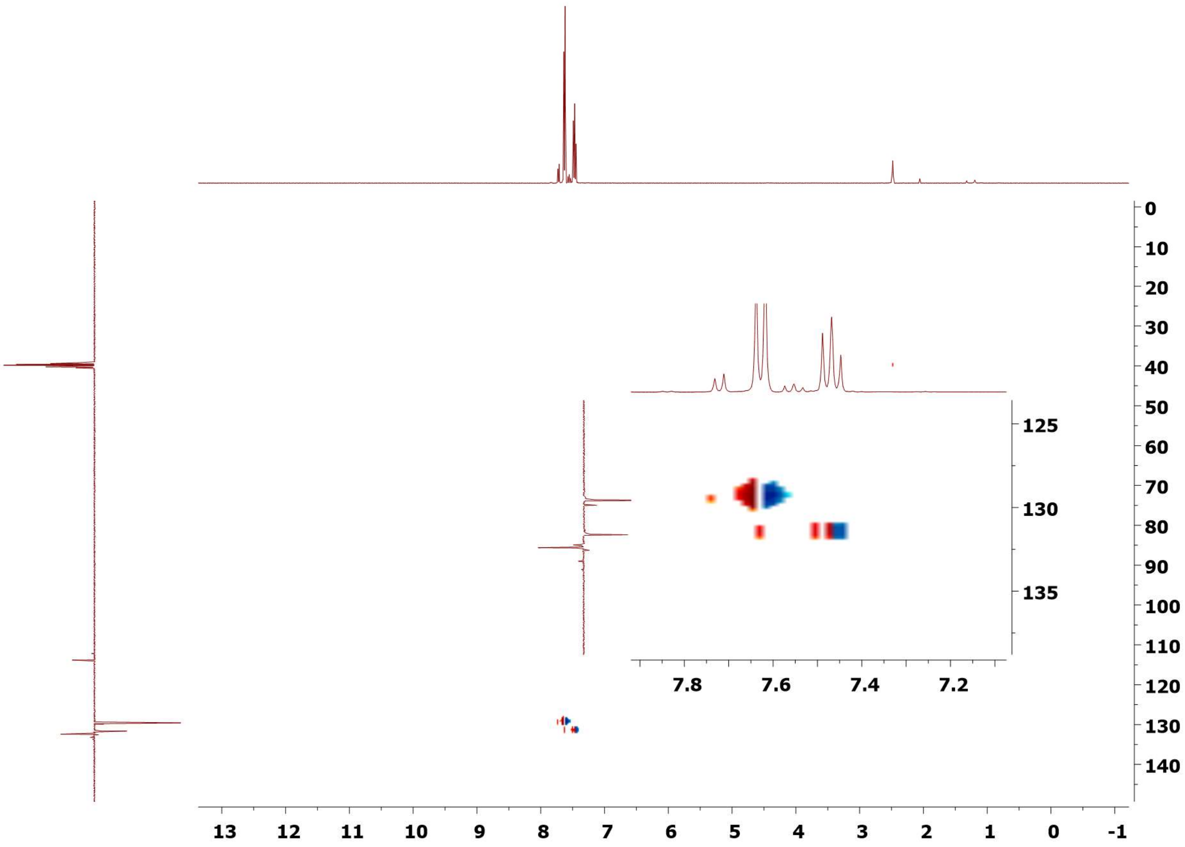

^{1}H-^{13}C-gHMBC NMR (DMSO-d6) spectrum of (2,6-dichlorophenyl)carbamothioyl cyanide (1:0.13 tautomeric ratio) (1f')

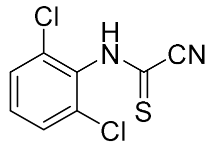

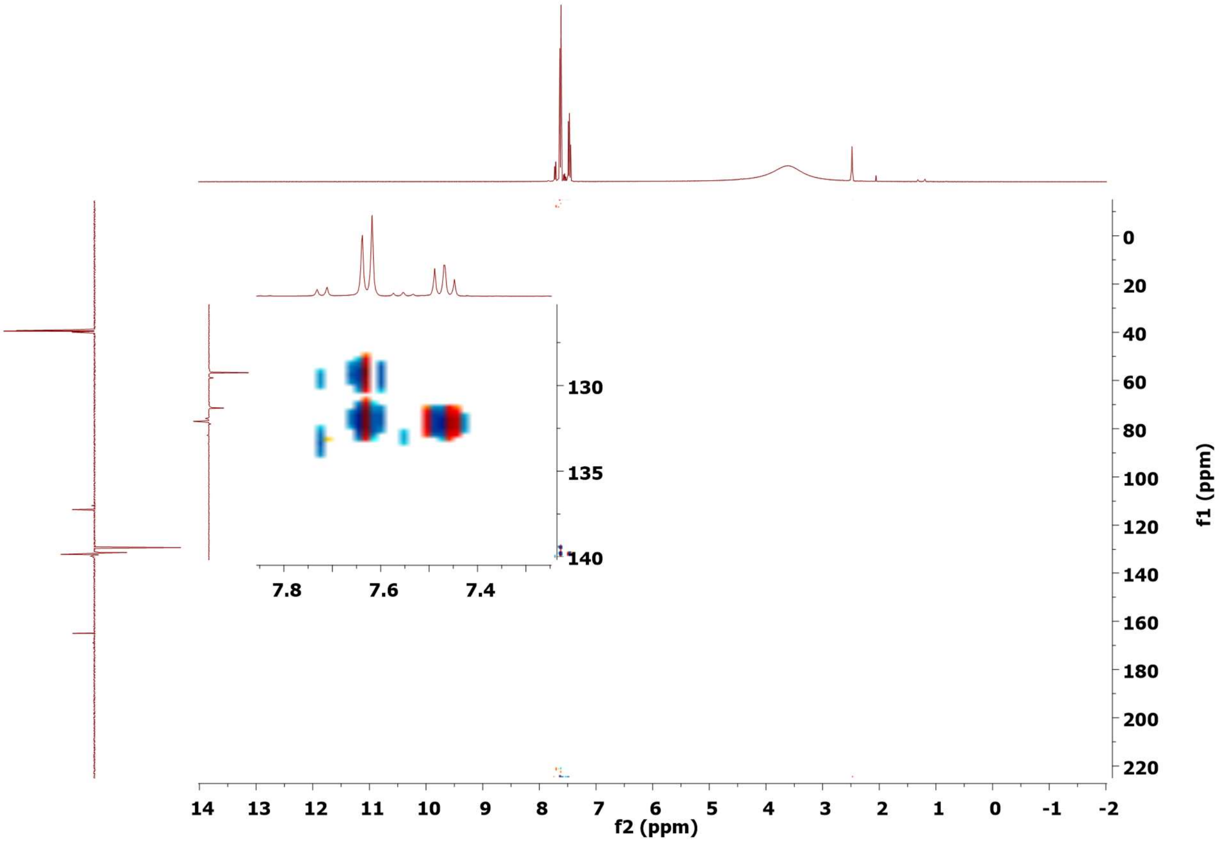

^{1}H NMR (CDCl$_3$) spectrum of (4-bromophenyl)thiocarbamoyl cyanide (1g')

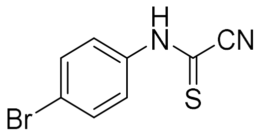

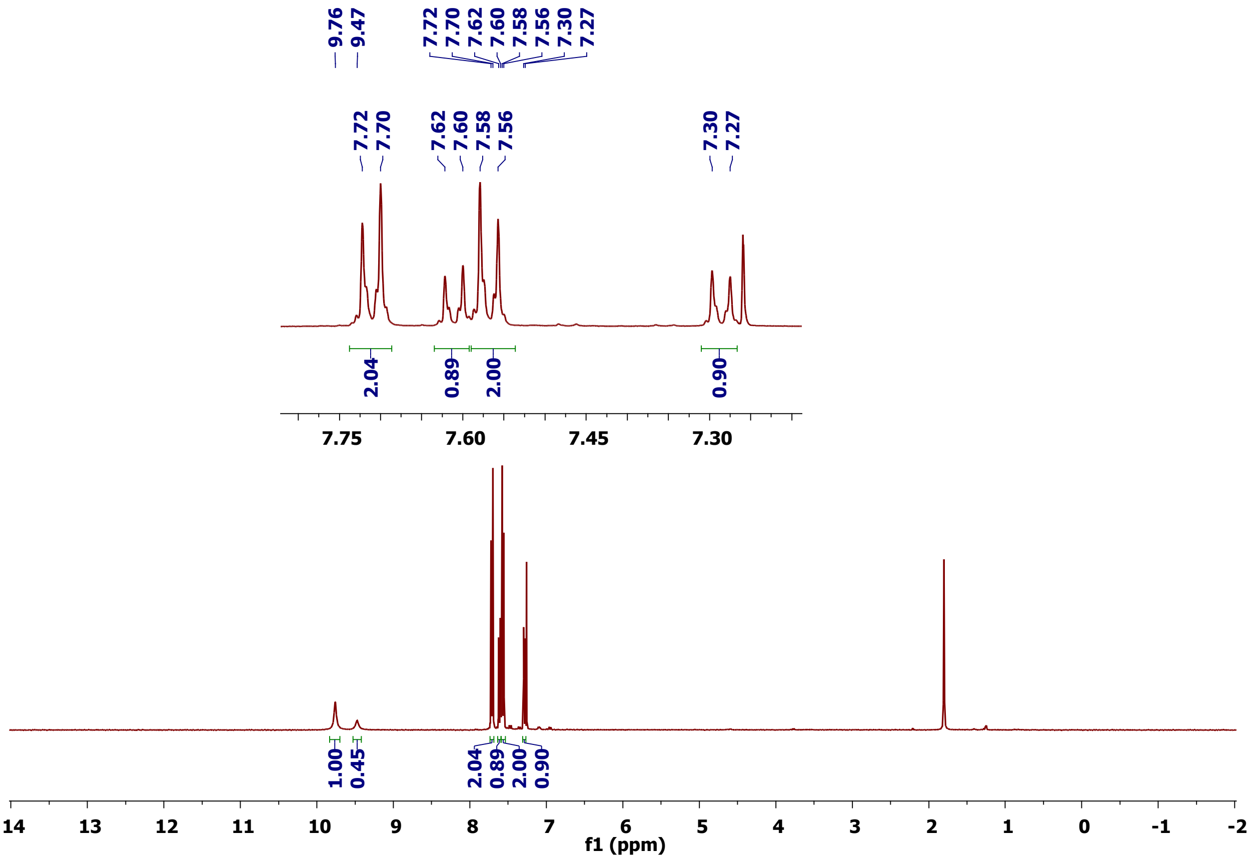

^{13}C NMR ($CDCl_3$) spectrum of (4-bromophenyl)thiocarbamoyl cyanide (1g')

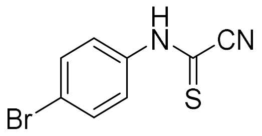

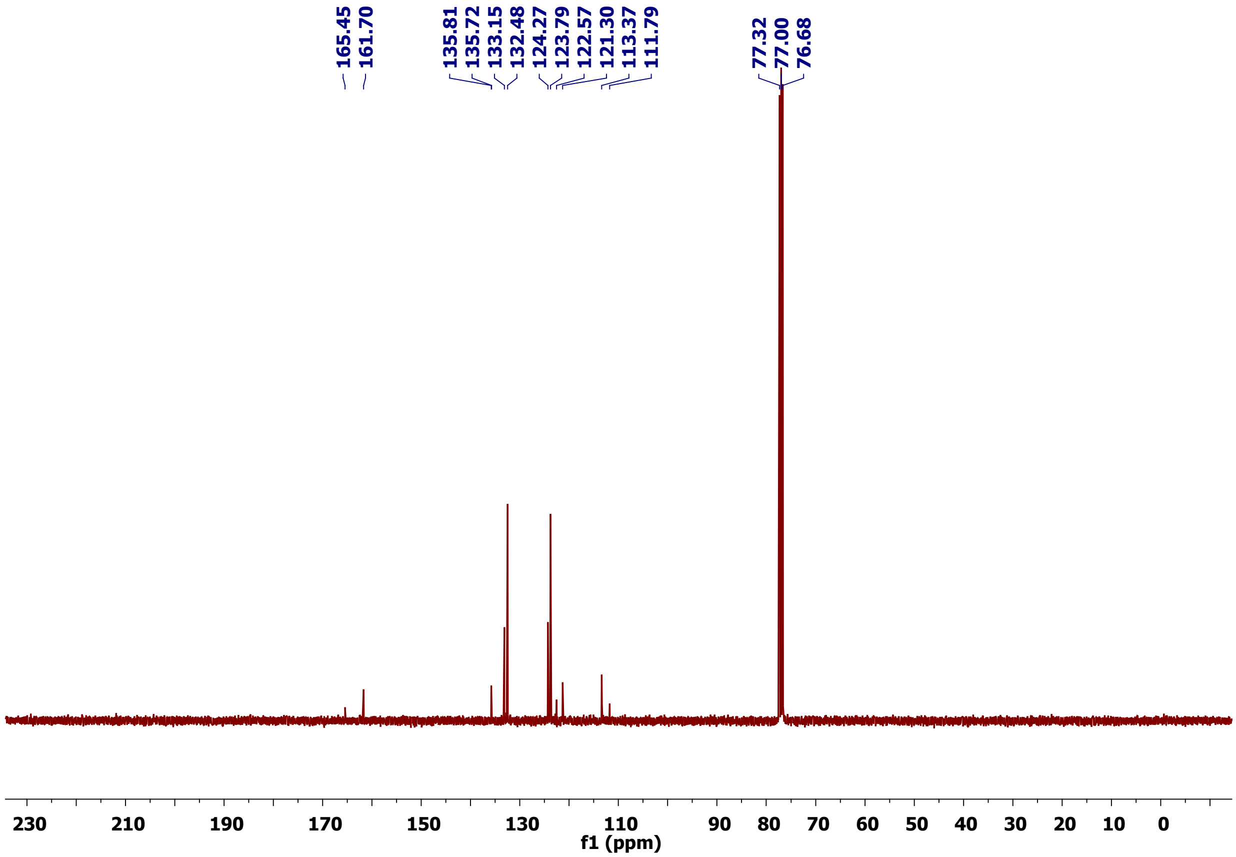

^{13}C CRAPT NMR ($CDCl_3$) spectrum of (4-bromophenyl)thiocarbamoyl cyanide (1g')

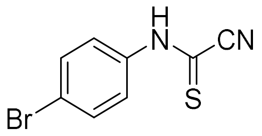

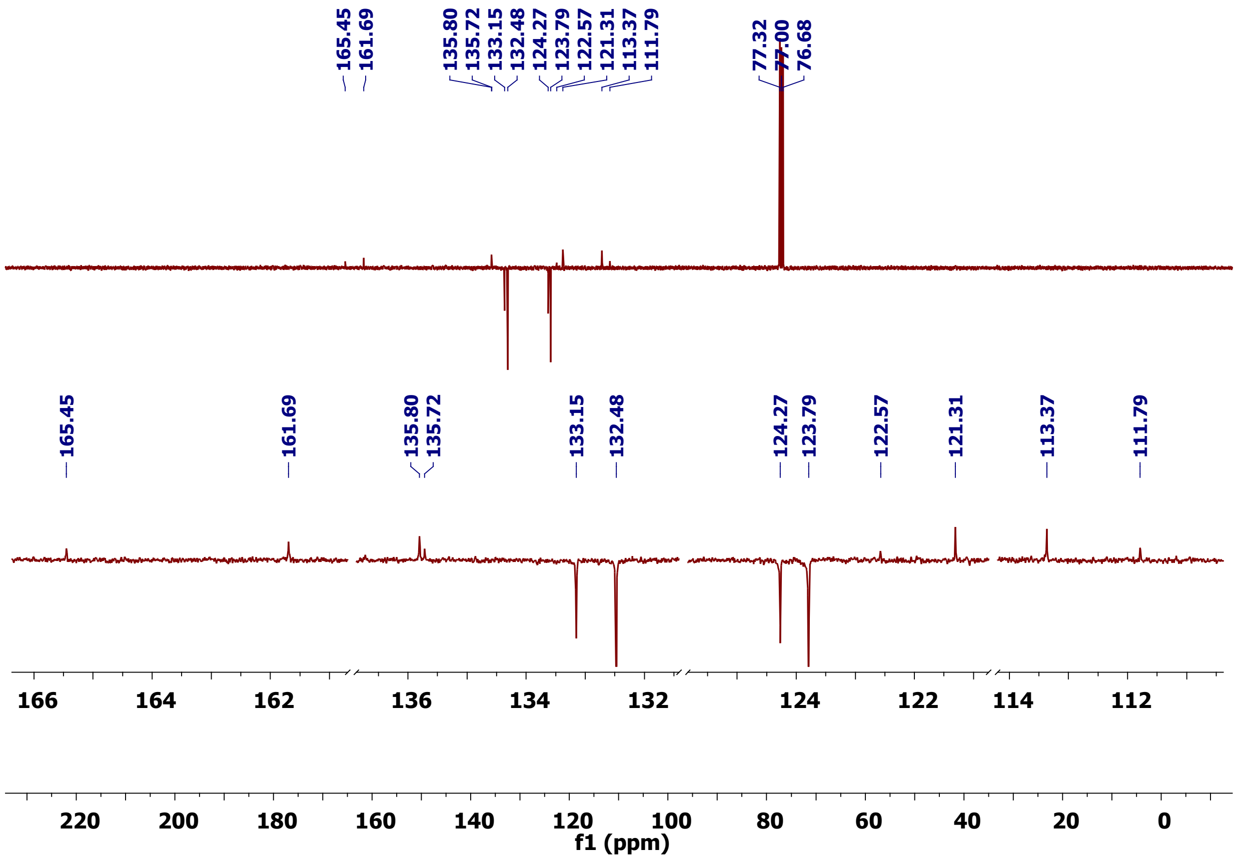

^{1}H-^{1}H gDQCOSY NMR ($CDCl_3$) spectrum of (4-bromophenyl)thiocarbamoyl cyanide (1g')

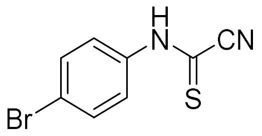

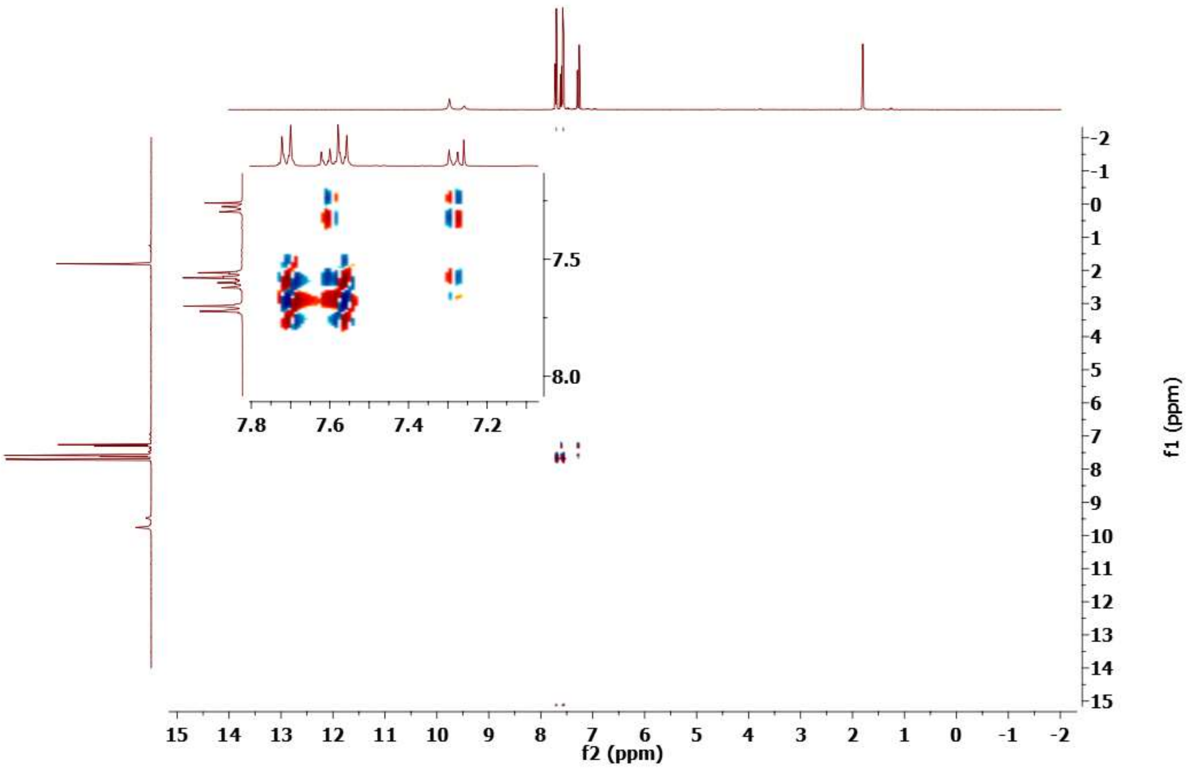

^1H-^{13}C-gHSQC NMR ($CDCl_3$) spectrum of (4-bromophenyl)thiocarbamoyl cyanide (1g')

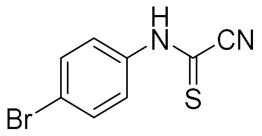

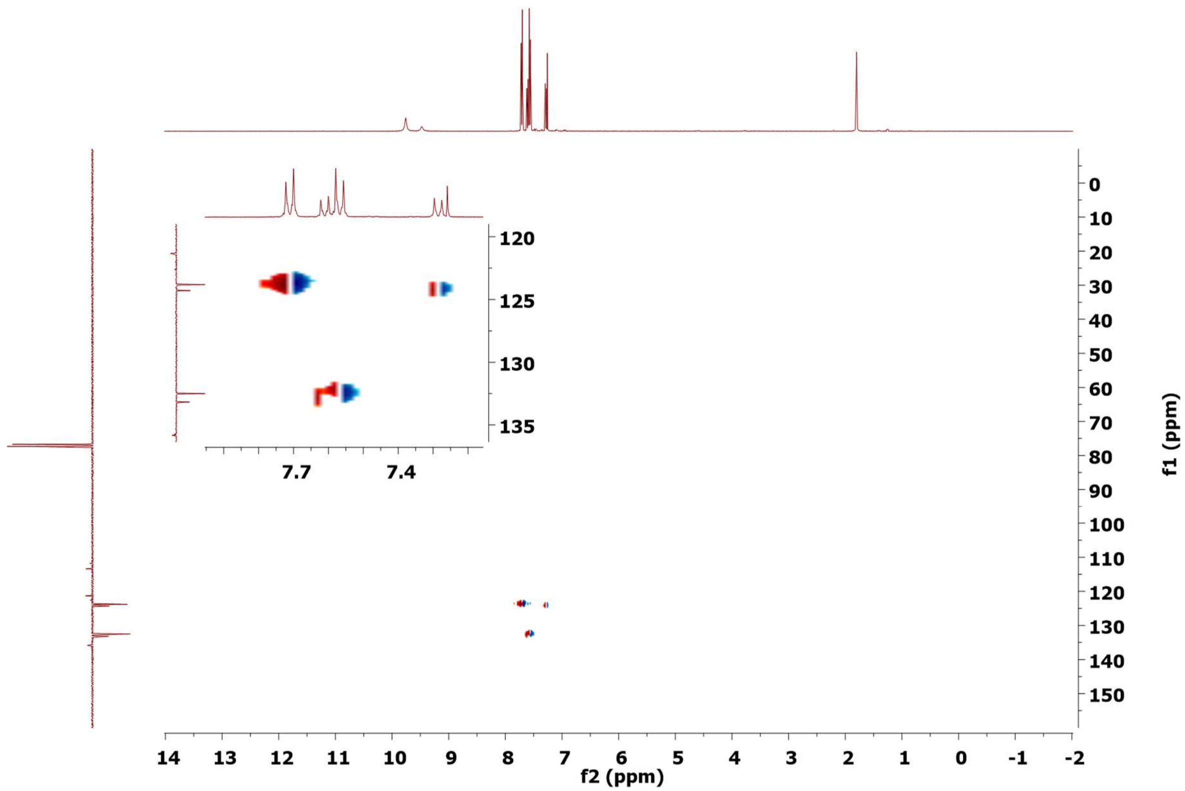

^{1}H-^{13}C-gHMBC NMR ($CDCl_3$) spectrum of (4-bromophenyl)thiocarbamoyl cyanide (1g')

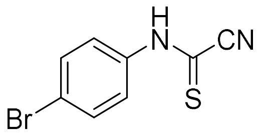

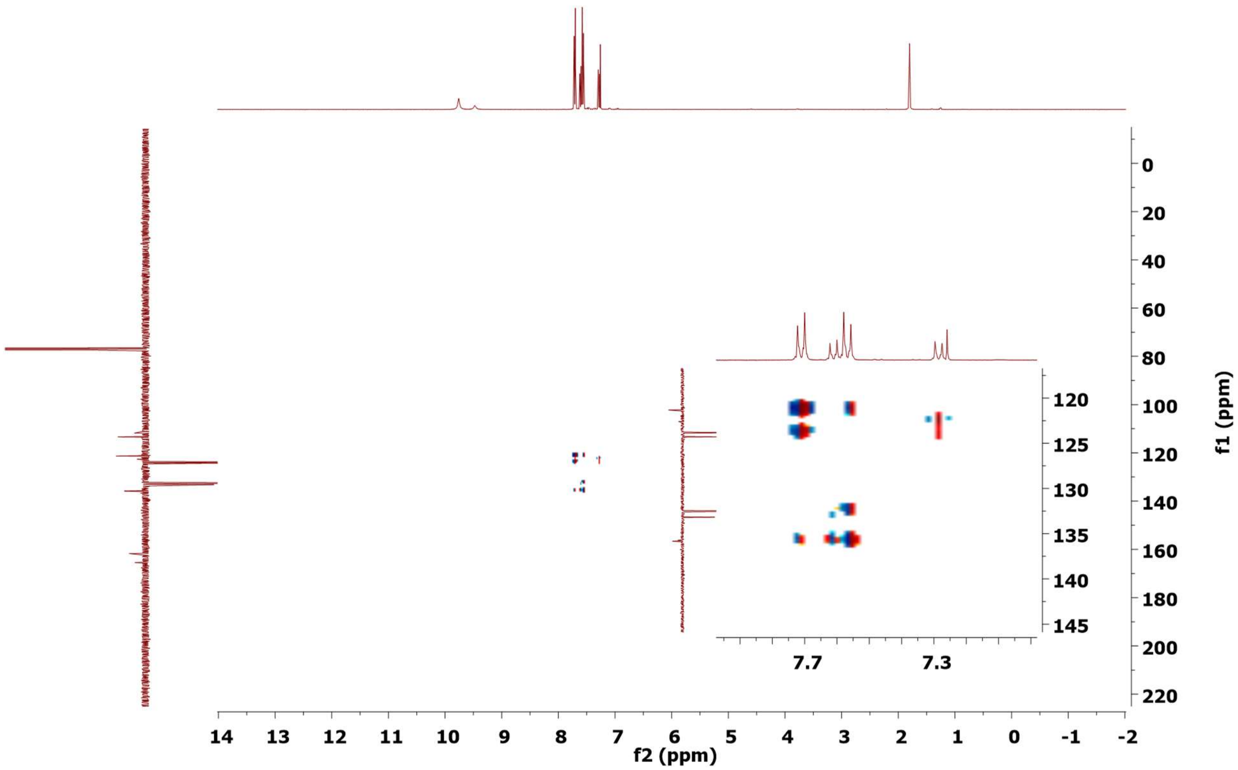

^{1}H NMR (DMSO-d6) spectrum of (2-methoxy-5-methylphenyl)carbamothioyl cyanide (1:0.52 tautomeric ratio) (1h’)

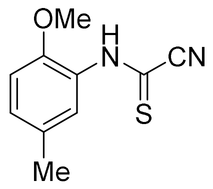

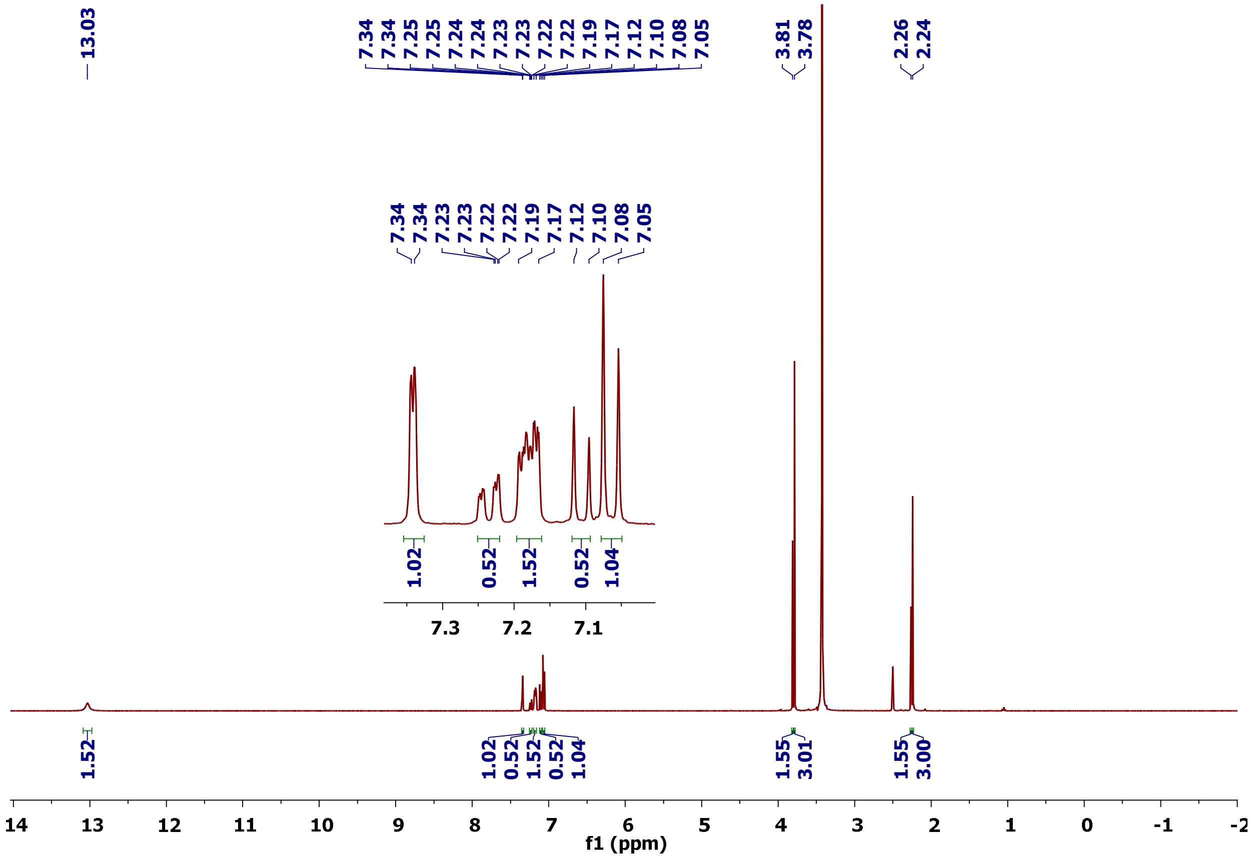

^{13}C NMR (DMSO-d6) spectrum of (2-methoxy-5-methylphenyl)carbamothioyl cyanide (1:0.52 tautomeric ratio) (1h')

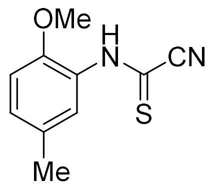

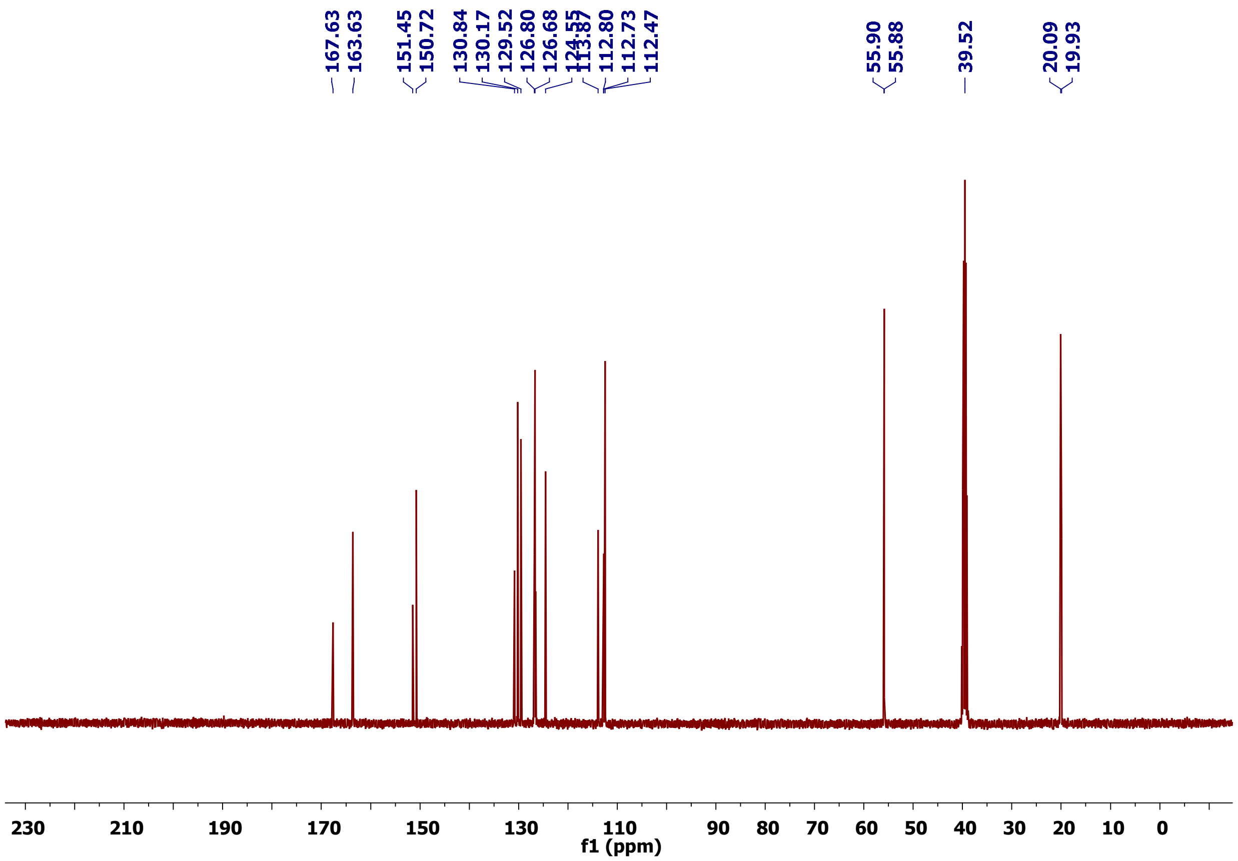

^{13}C CRAPT NMR (DMSO-d6) spectrum of (2-methoxy-5-methylphenyl)carbamothioyl cyanide (1:0.52 tautomeric ratio) (1h')

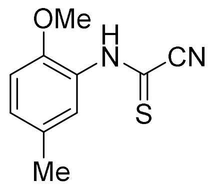

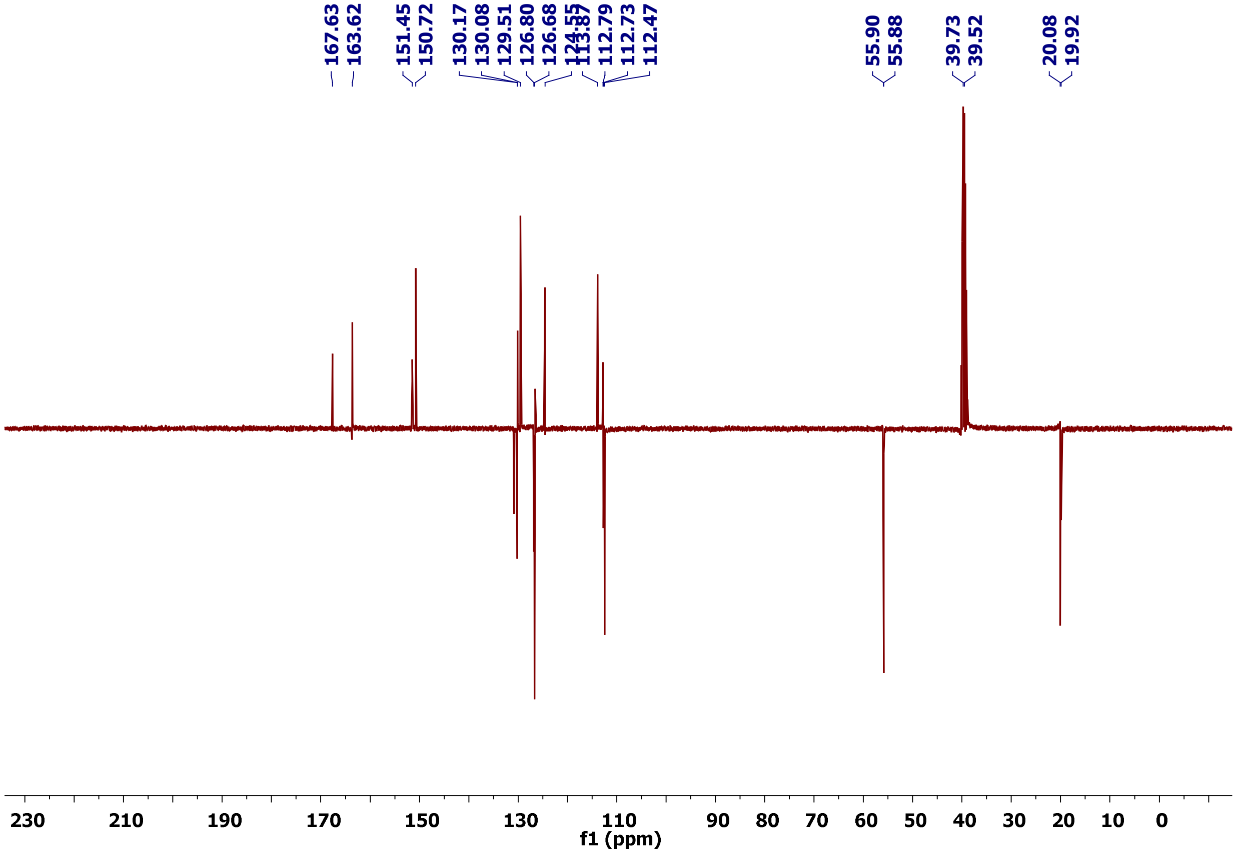

^{1}H-^{1}H-gDQCOSY NMR (DMSO-d6) spectrum of (2-methoxy-5-methylphenyl)carbamothioyl cyanide (1:0.52 tautomeric ratio) (1h')

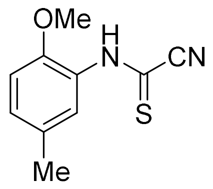

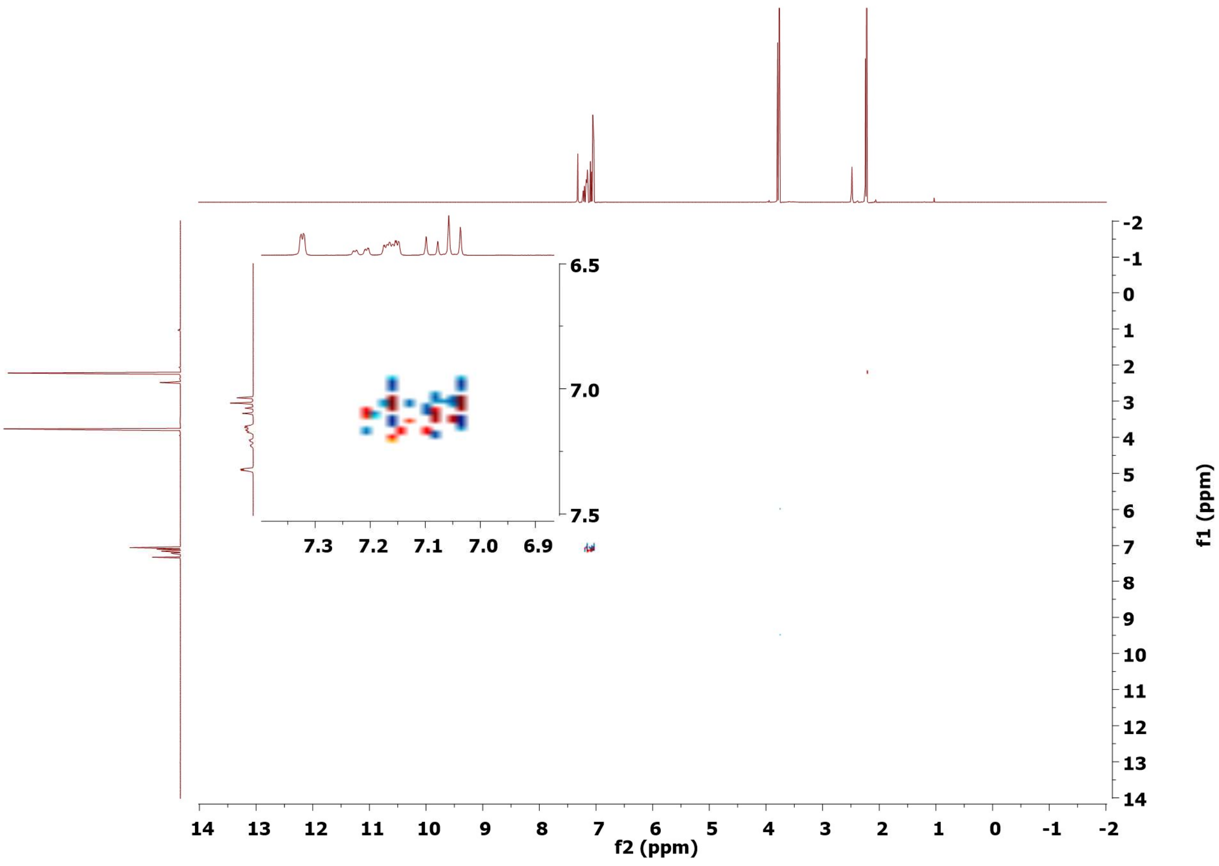

^{1}H-^{13}C-gHSQC NMR (DMSO-d6) spectrum of (2-methoxy-5-methylphenyl)carbamothioyl cyanide (1:0.52 tautomeric ratio) (1h')

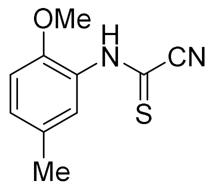

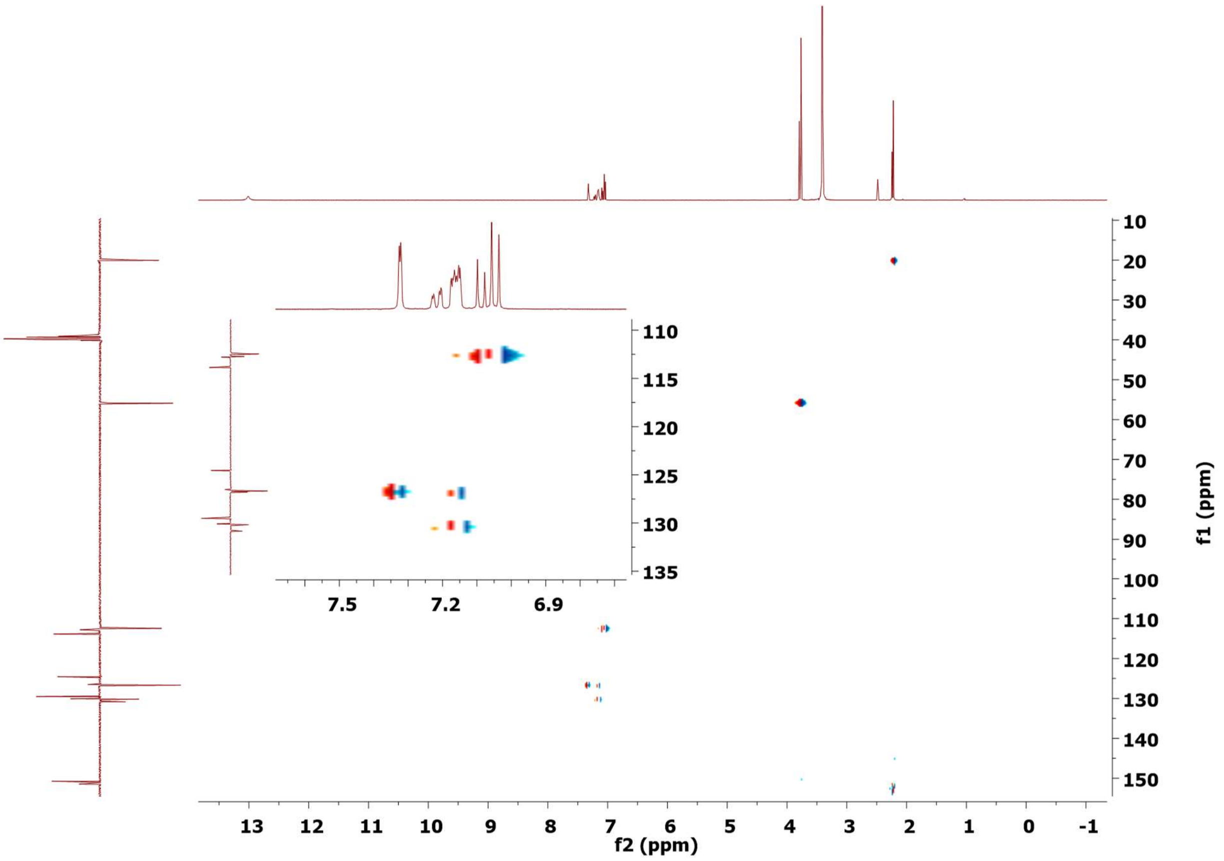

^{1}H-^{13}C-gHMBC NMR (DMSO-d6) spectrum of (2-methoxy-5-methylphenyl)carbamothioyl cyanide (1:0.52 tautomeric ratio) (1h')

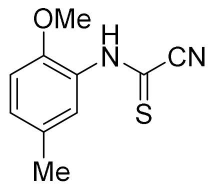

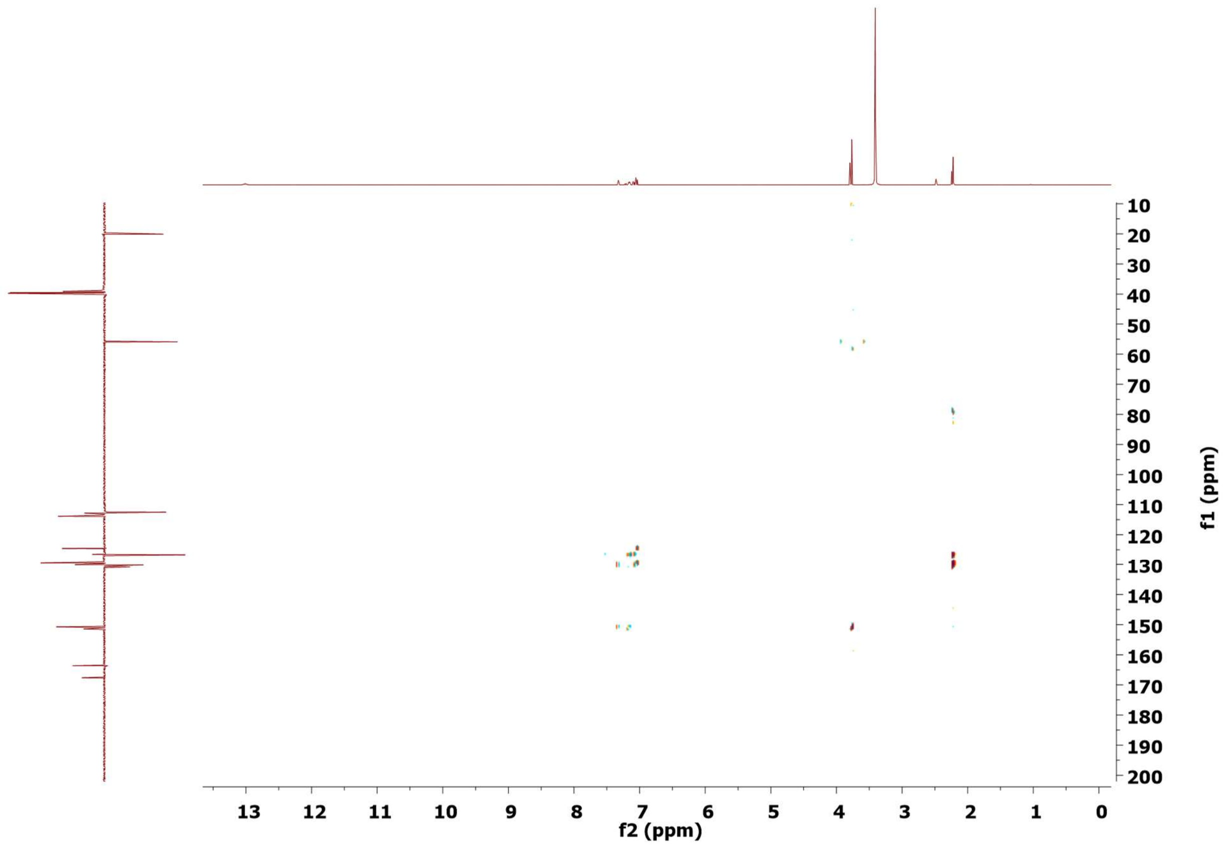

^{1}H NMR (DMSO-d6) spectrum of (3,5-dichlorophenyl)carbamothioyl cyanide (1:0.23 tautomeric ratio) (1i’)

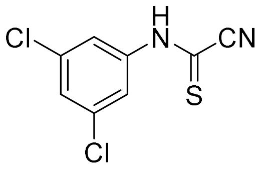

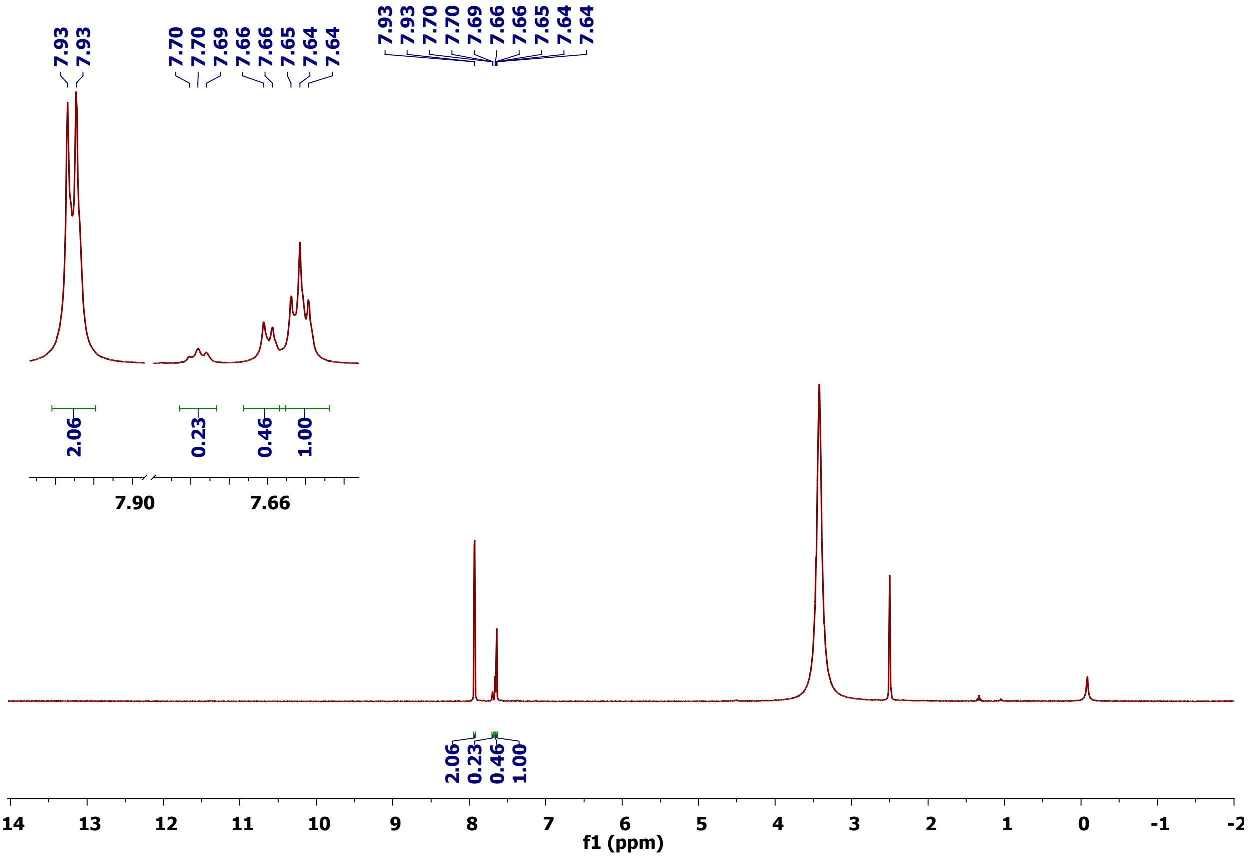

^{13}C NMR (DMSO-d6) spectrum of (3,5-dichlorophenyl)carbamothioyl cyanide (1:0.23 tautomeric ratio) (1i’)

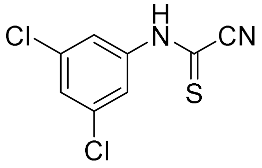

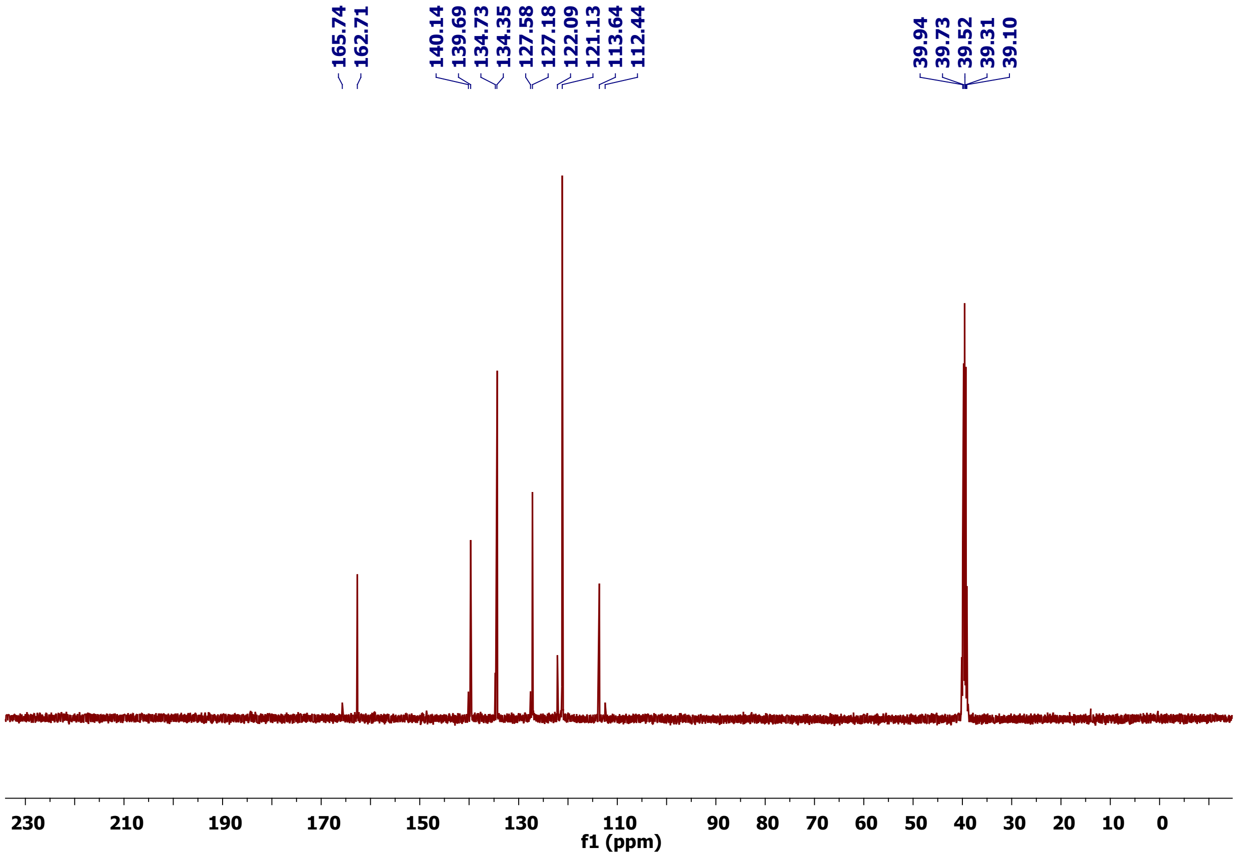

^{13}C CRAPT NMR (DMSO-d6) spectrum of (3,5-dichlorophenyl)carbamothioyl cyanide (1:0.23 tautomeric ratio) (1i')

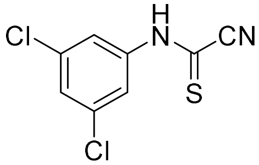

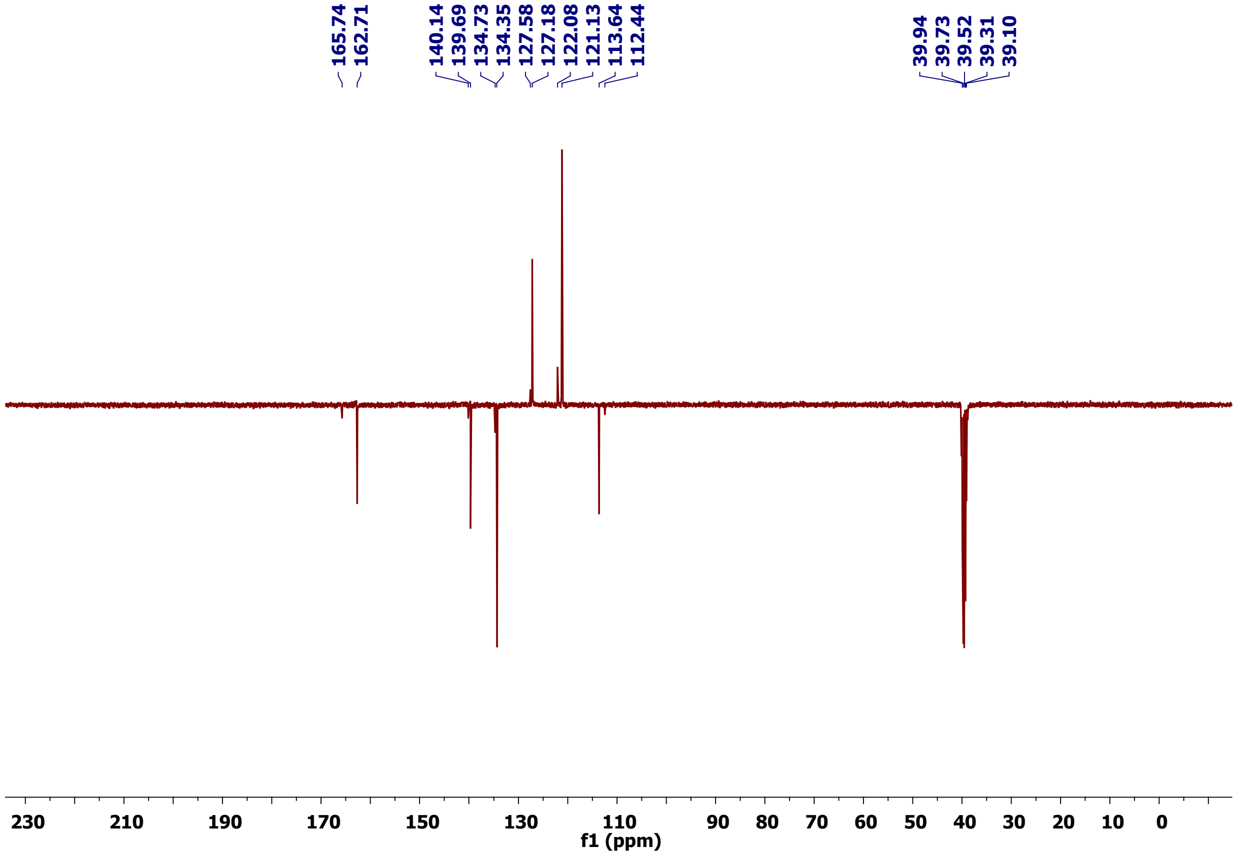

^{1}H-^{1}H-gDQCOSY NMR (DMSO-d6) spectrum of (3,5-dichlorophenyl)carbamothioyl cyanide (1:0.23 tautomeric ratio) (1i')

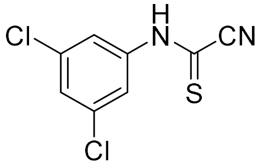

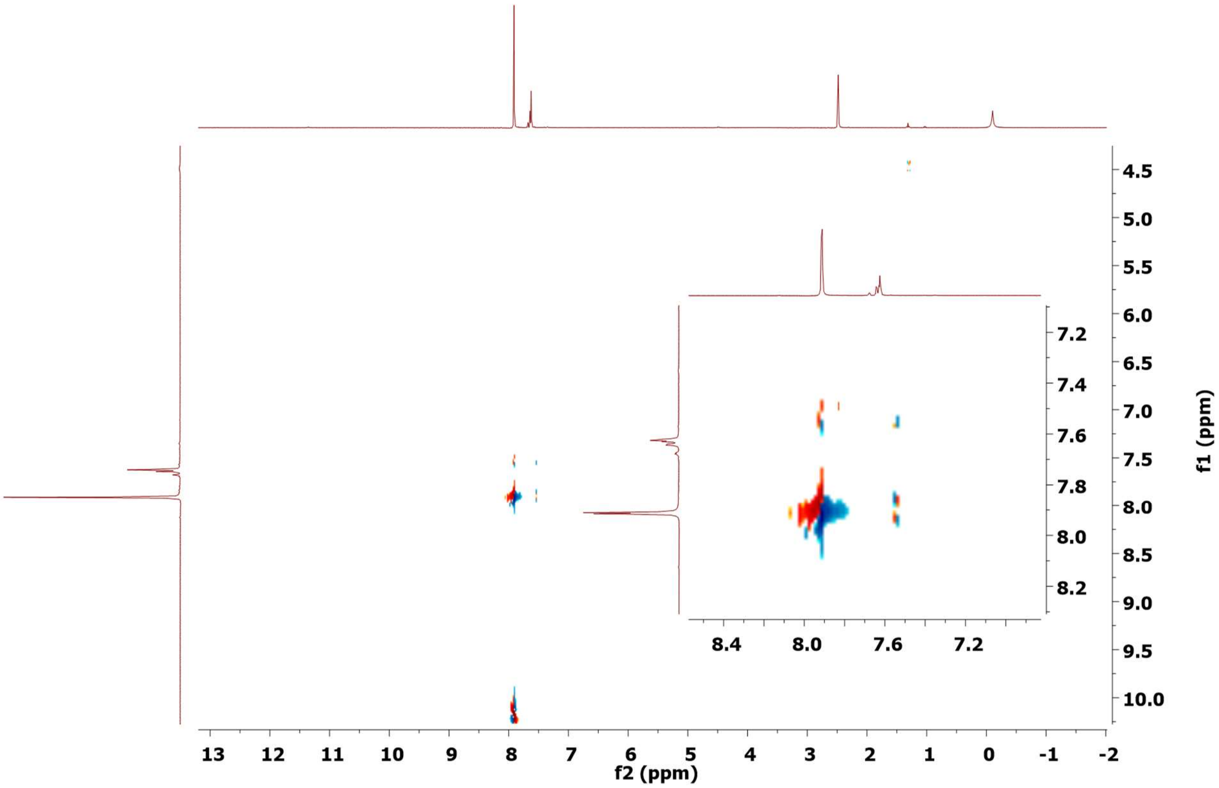

^{1}H-^{13}C-gHSQC NMR (DMSO-d6) spectrum of (3,5-dichlorophenyl)carbamothioyl cyanide (1:0.23 tautomeric ratio) (1i')

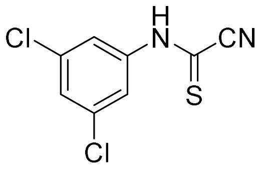

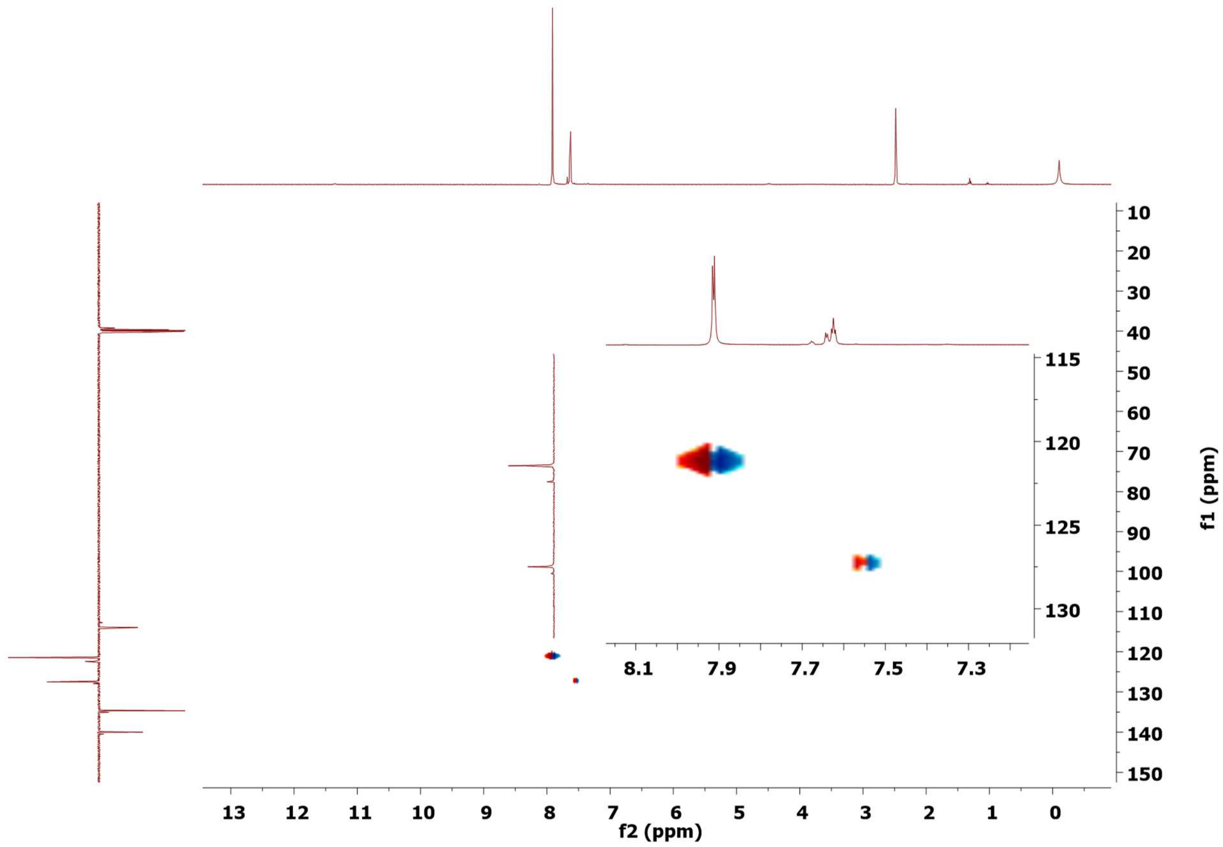

^1H-^{13}C-gHMBC NMR (DMSO-d6) spectrum of (3,5-dichlorophenyl)carbamothioyl cyanide (1:0.23 tautomeric ratio) (1i')

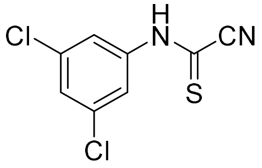

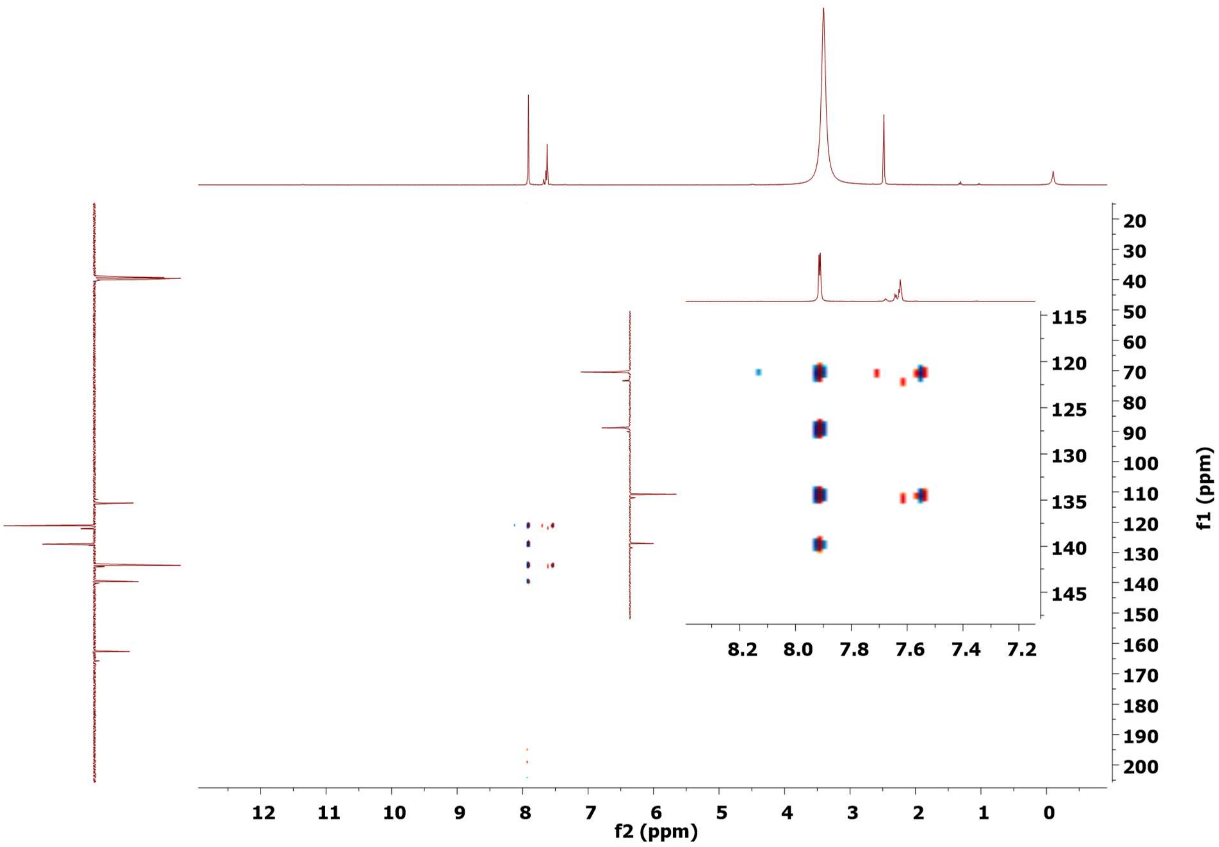

^{1}H NMR (DMSO-d6) spectrum of (3,4-dichlorophenyl)carbamothioyl cyanide (1:0.22 tautomeric ratio) (1j')

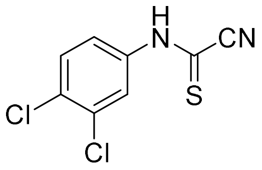

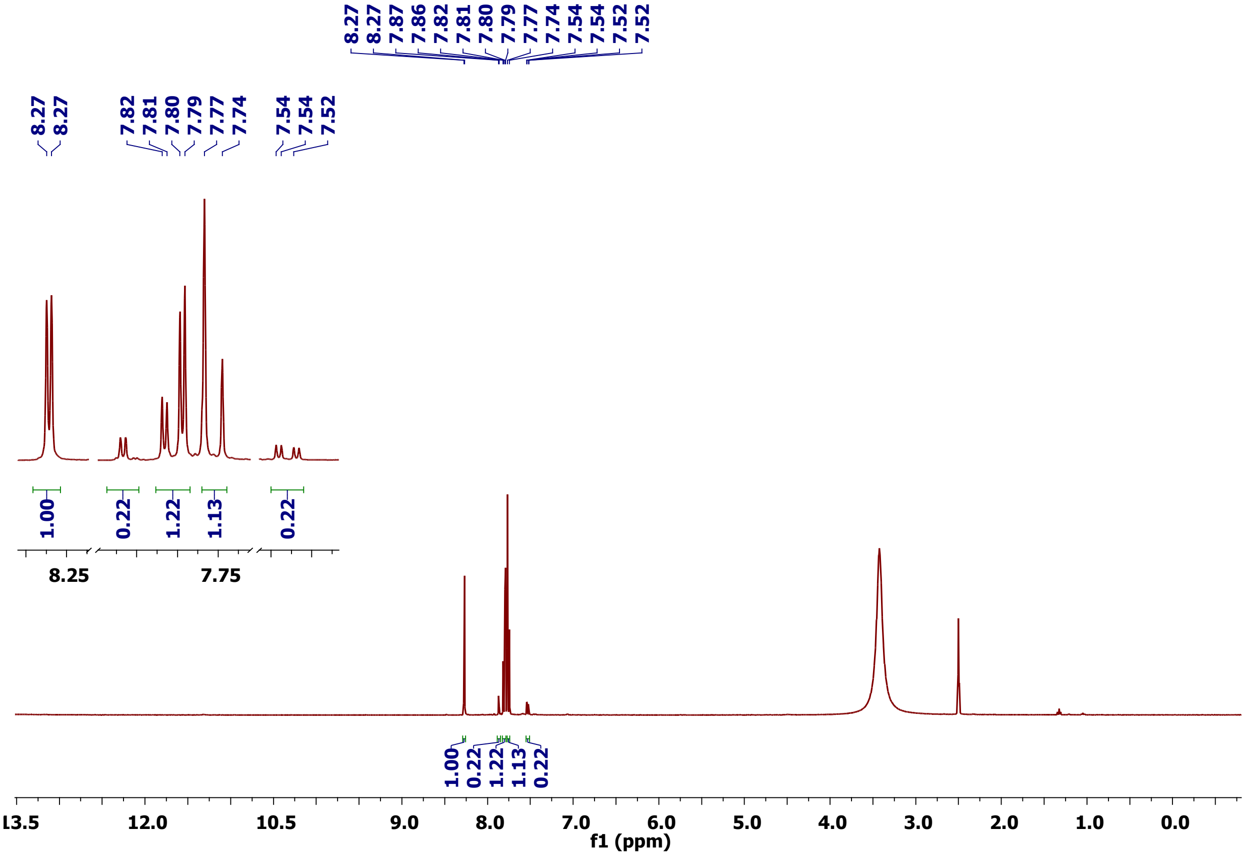

^{13}C NMR (DMSO-d6) spectrum of (3,4-dichlorophenyl)carbamothioyl cyanide (1:0.22 tautomeric ratio) (1j')

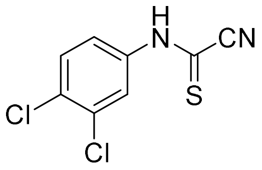

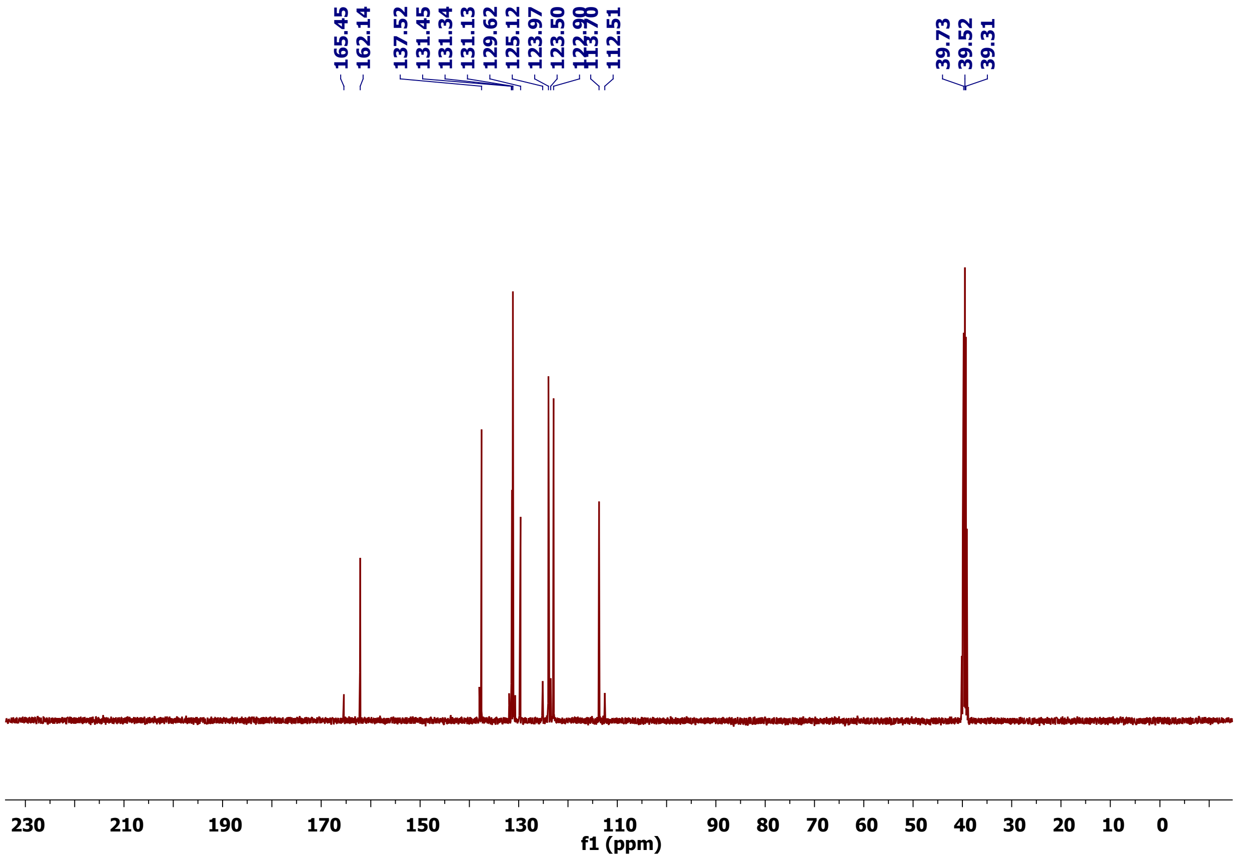

^{13}C CRAPT NMR (DMSO-d6) spectrum of (3,4-dichlorophenyl)carbamothioyl cyanide (1:0.22 tautomeric ratio) (1j')

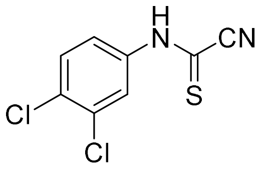

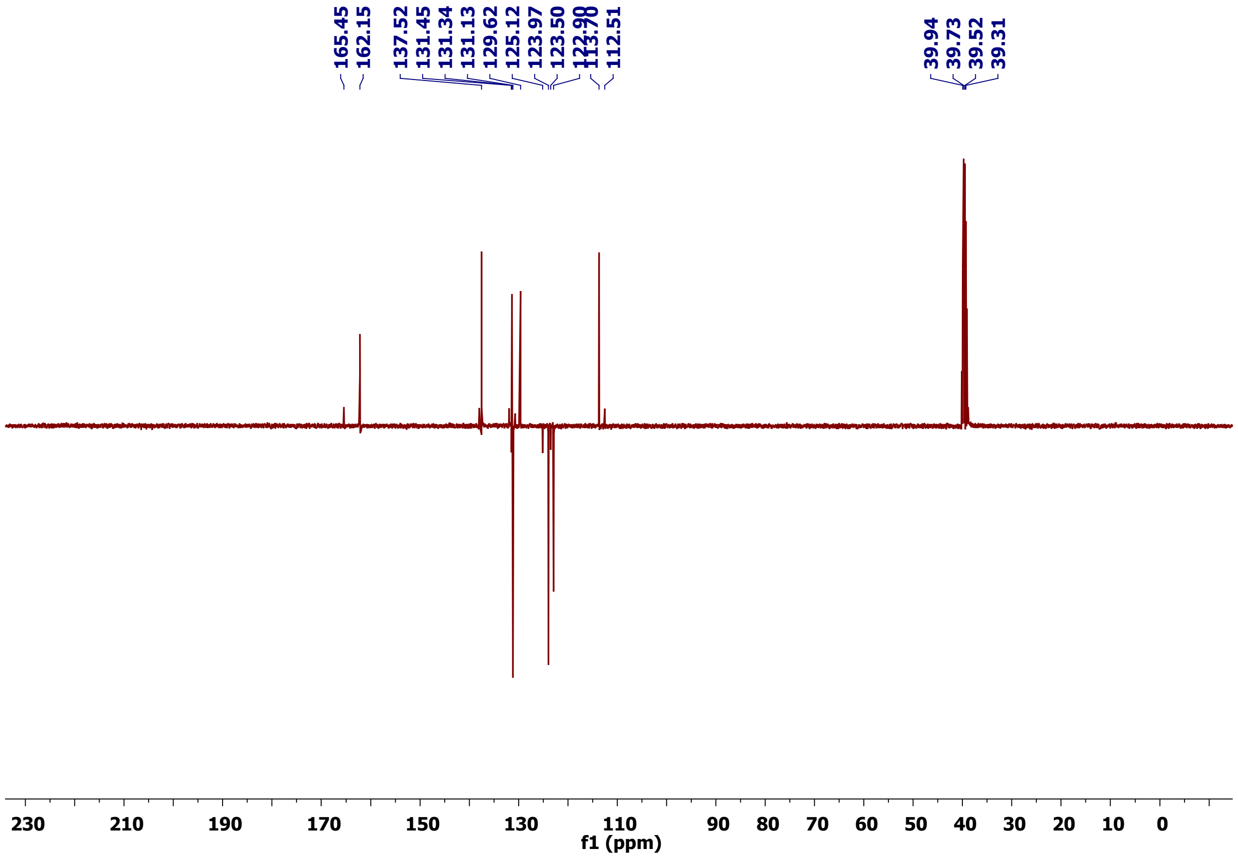

^{1}H-^{1}H-gDQCOSY NMR (DMSO-d6) spectrum of (3,4-dichlorophenyl)carbamothioyl cyanide (1:0.22 tautomeric ratio) (1j')

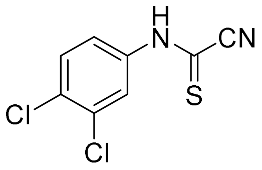

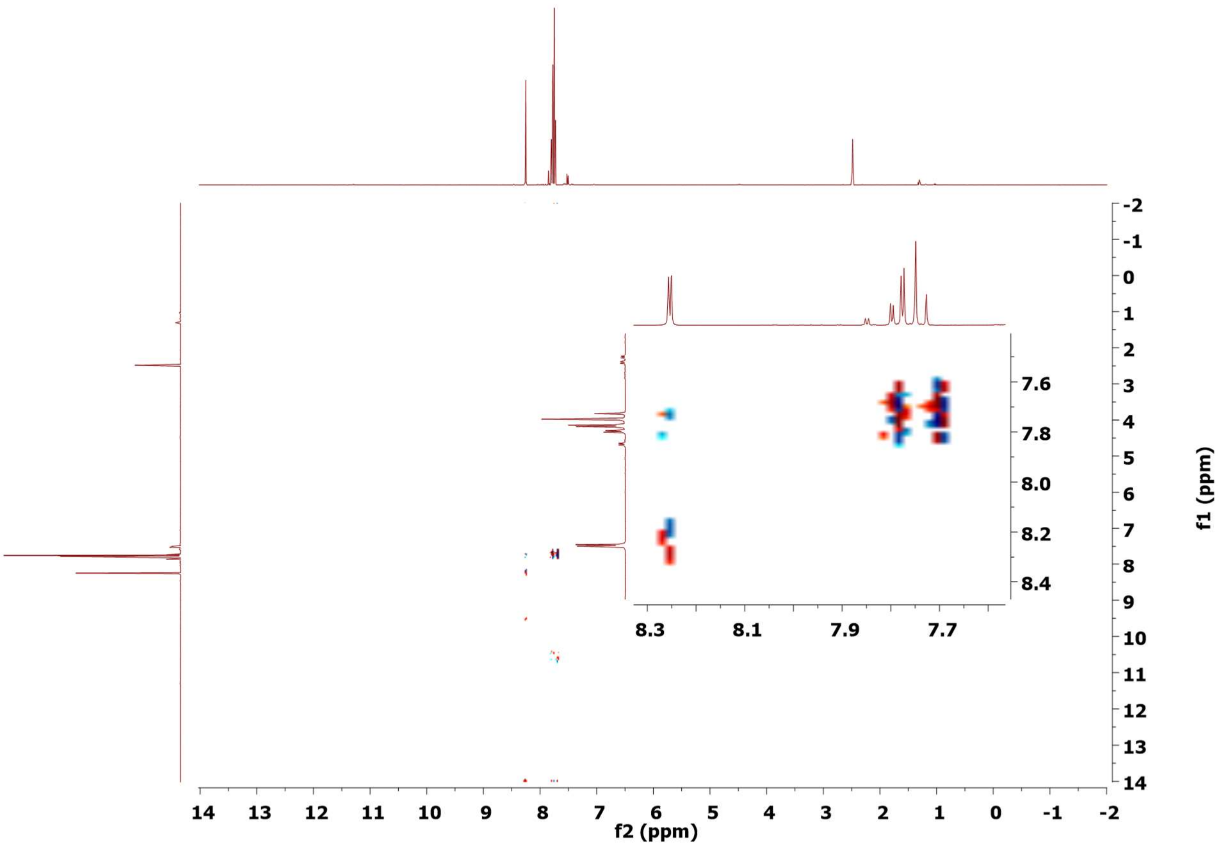

^{1}H-^{13}C-gHSQC NMR (DMSO-d6) spectrum of (3,4-dichlorophenyl)carbamothioyl cyanide (1:0.22 tautomeric ratio) (1j')

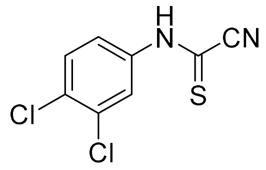

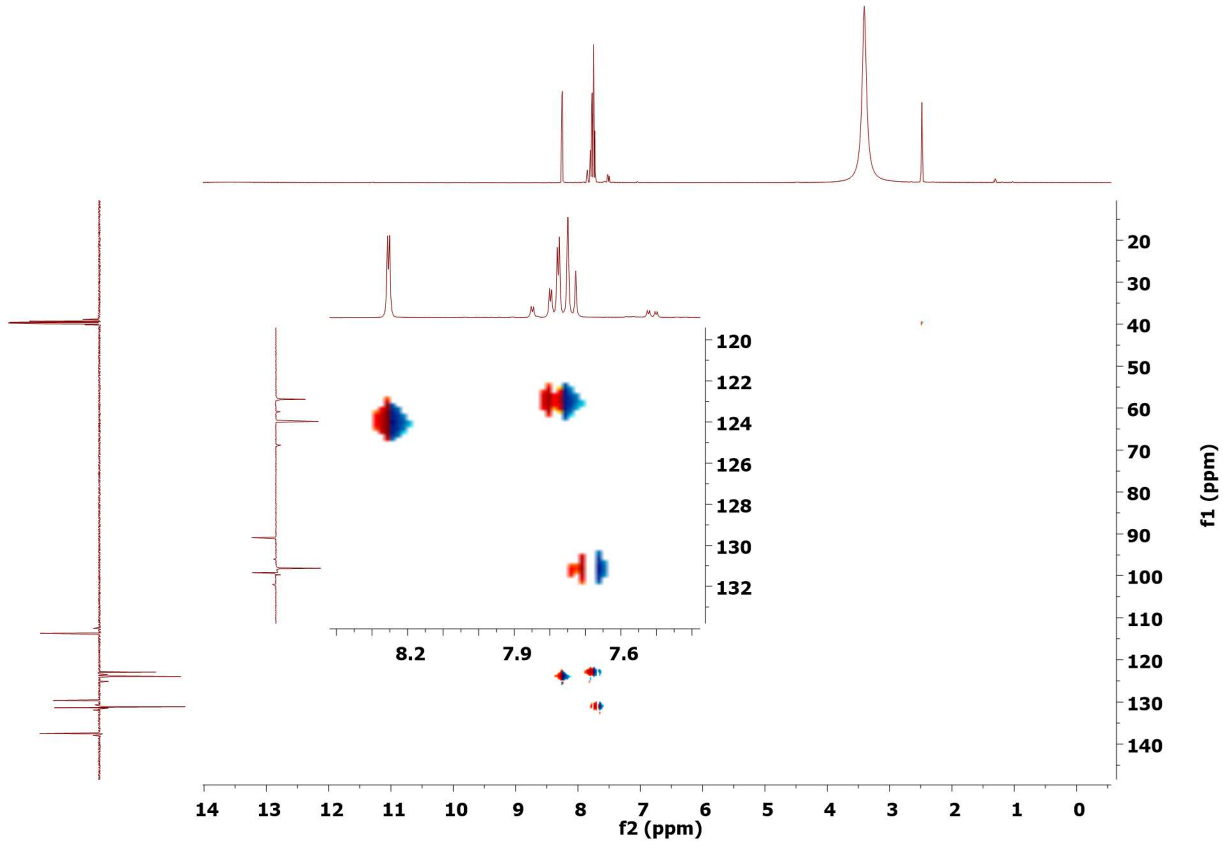

^1H-^{13}C-gHMBC NMR (DMSO-d6) spectrum of (3,4-dichlorophenyl)carbamothioyl cyanide (1:0.22 tautomeric ratio) (1j')

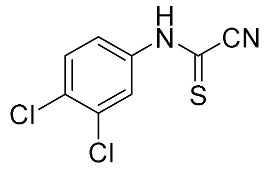

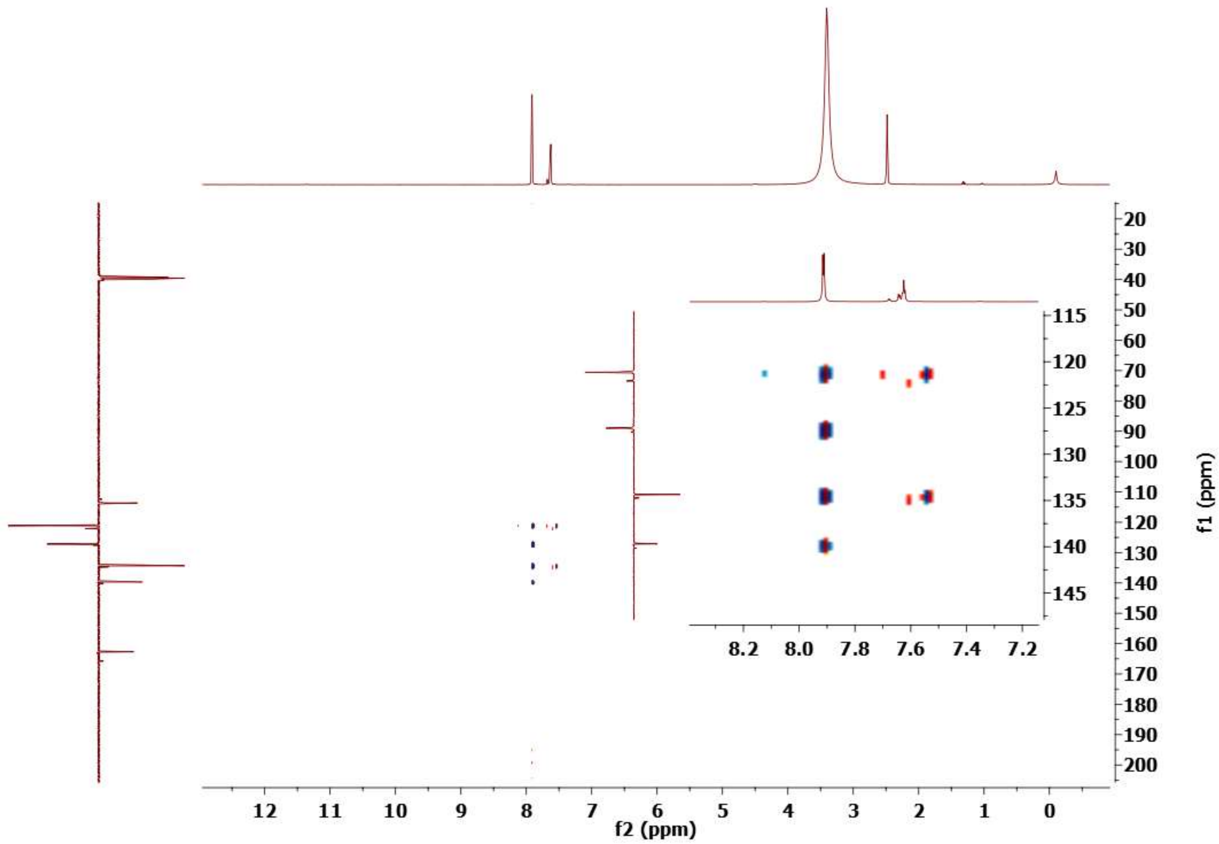

^{1}H NMR (DMSO-d6) spectrum of (2,6-fluorophenyl)carbamothioyl cyanide (1:0.27 tautomeric ratio) (1k')

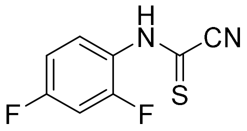

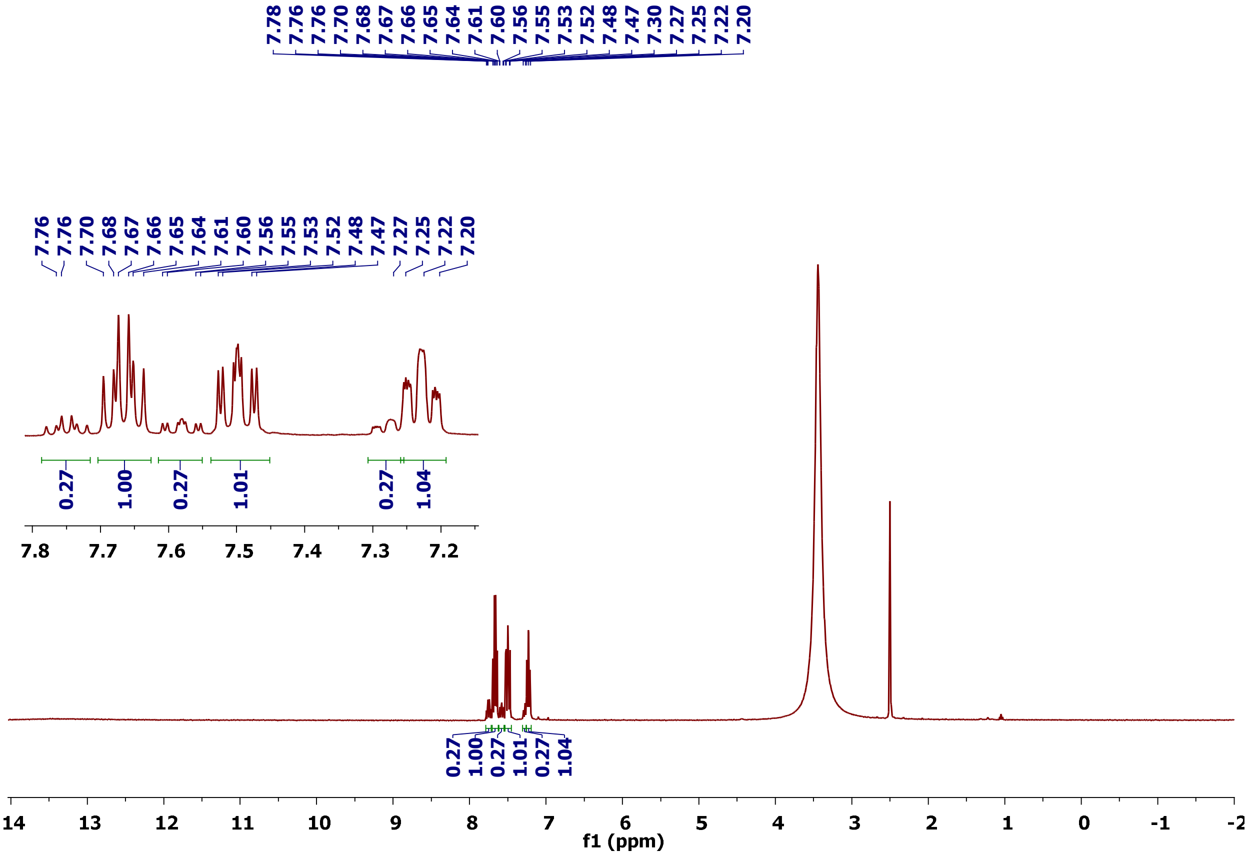

^{13}C NMR (DMSO-d6) spectrum of (2,6-fluorophenyl)carbamothioyl cyanide (1:0.27 tautomeric ratio) (1k')

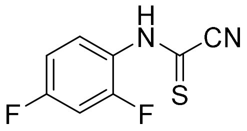

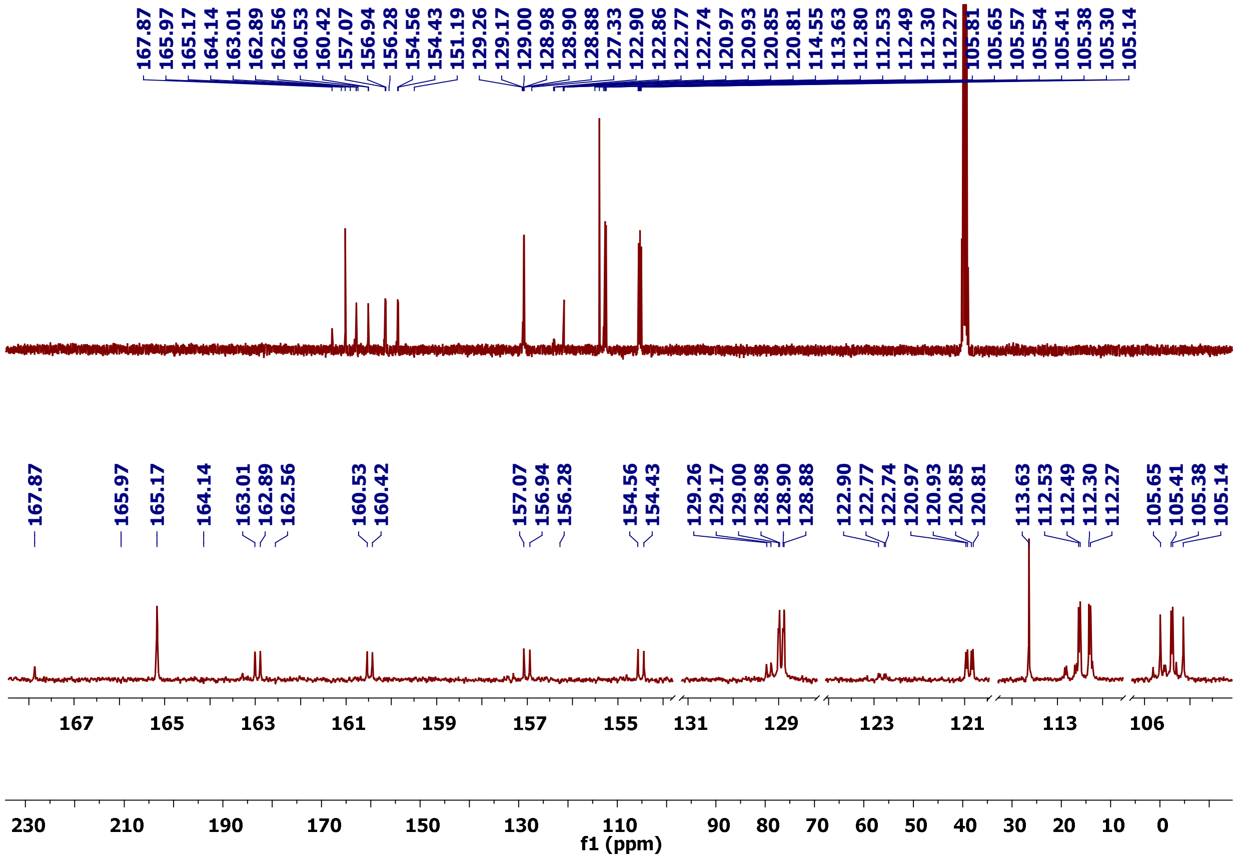

^{13}C CRAPT NMR (DMSO-d6) spectrum of (2,6-fluorophenyl)carbamothioyl cyanide (1:0.27 tautomeric ratio) (1k')

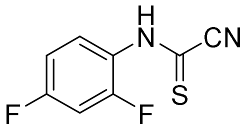

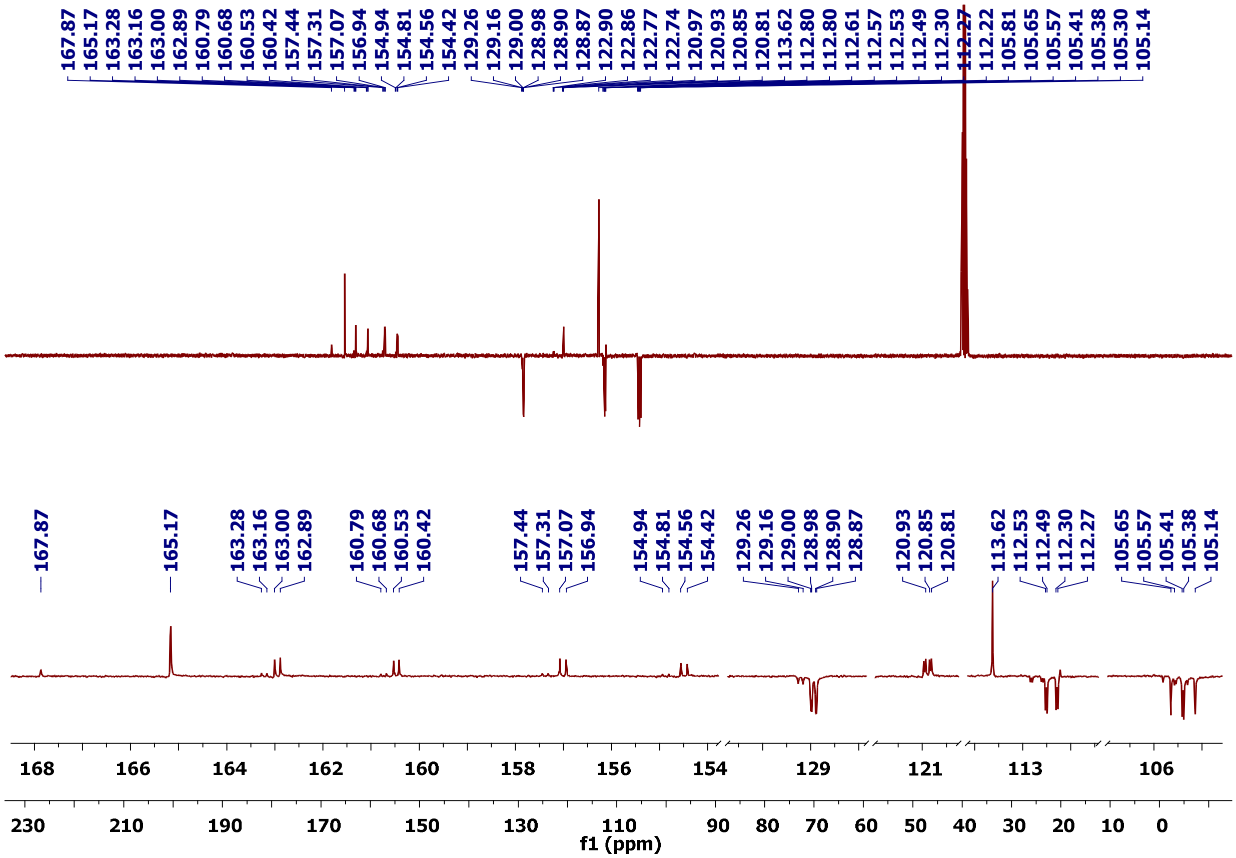

^1H-^1H-gDQCOSY NMR (DMSO-d6) spectrum of (2,6-fluorophenyl)carbamothioyl cyanide (1:0.27 tautomeric ratio) (1k')

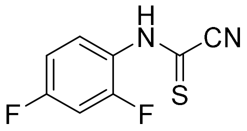

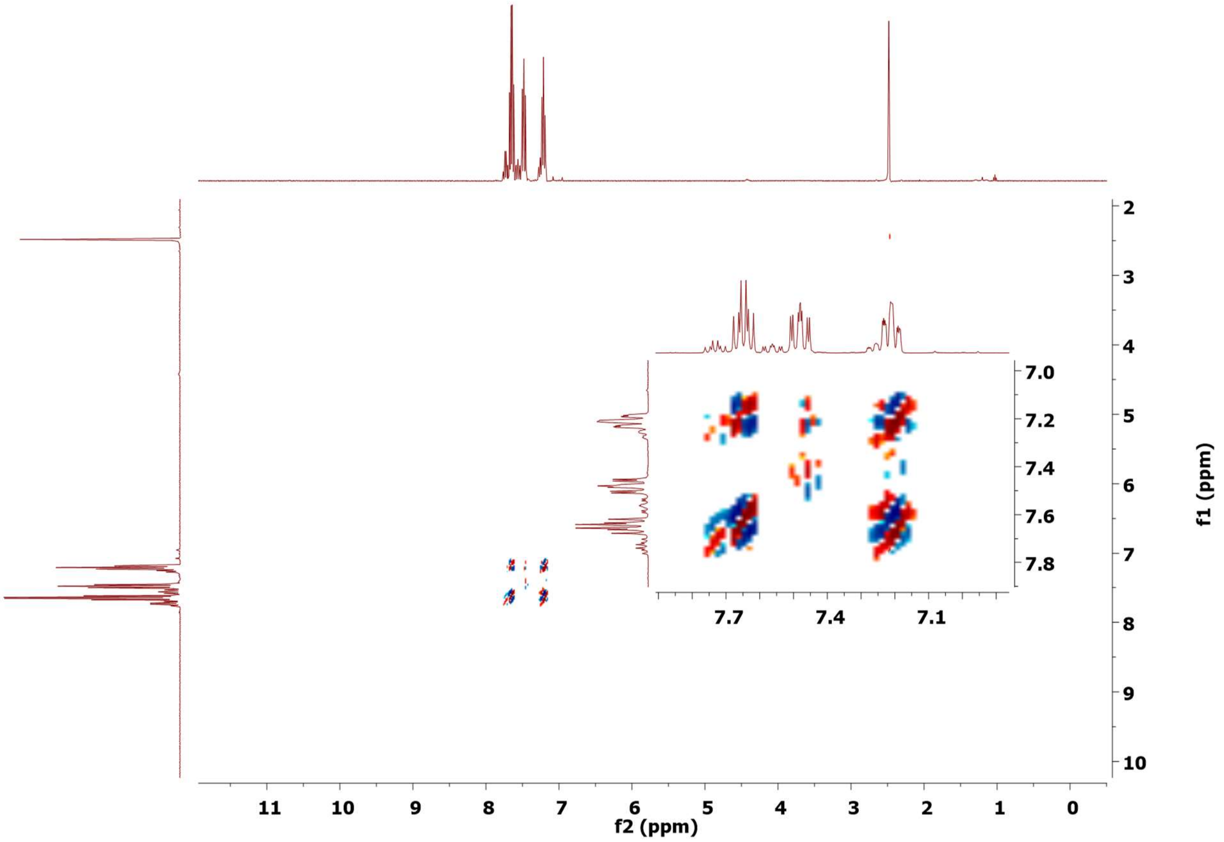

^{1}H-^{13}C-gHSQC NMR (DMSO-d6) spectrum of (2,6-fluorophenyl)carbamothioyl cyanide (1:0.27 tautomeric ratio) (1k')

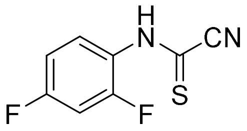

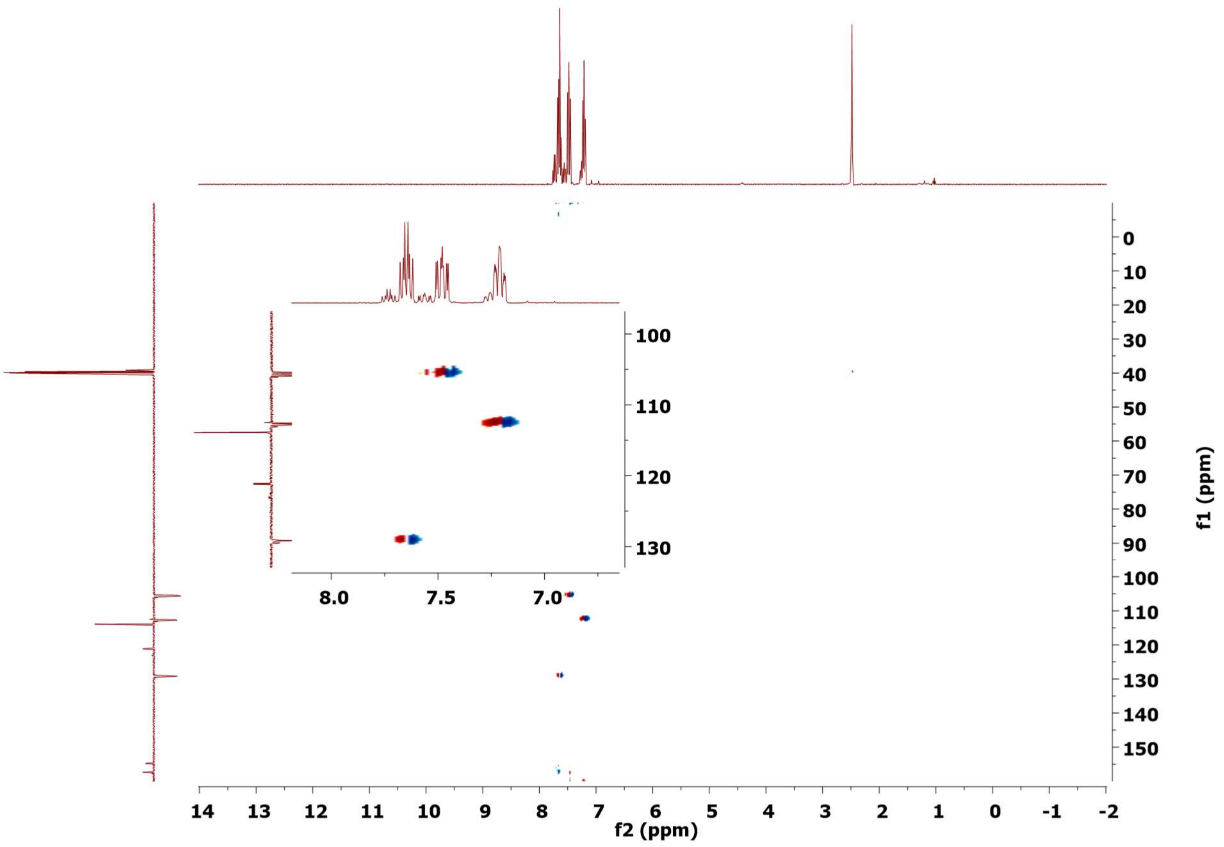

^{1}H-^{13}C-gHMBC NMR (DMSO-d6) spectrum of (2,6-fluorophenyl)carbamothioyl cyanide (1:0.27 tautomeric ratio) (1k')

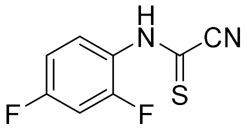

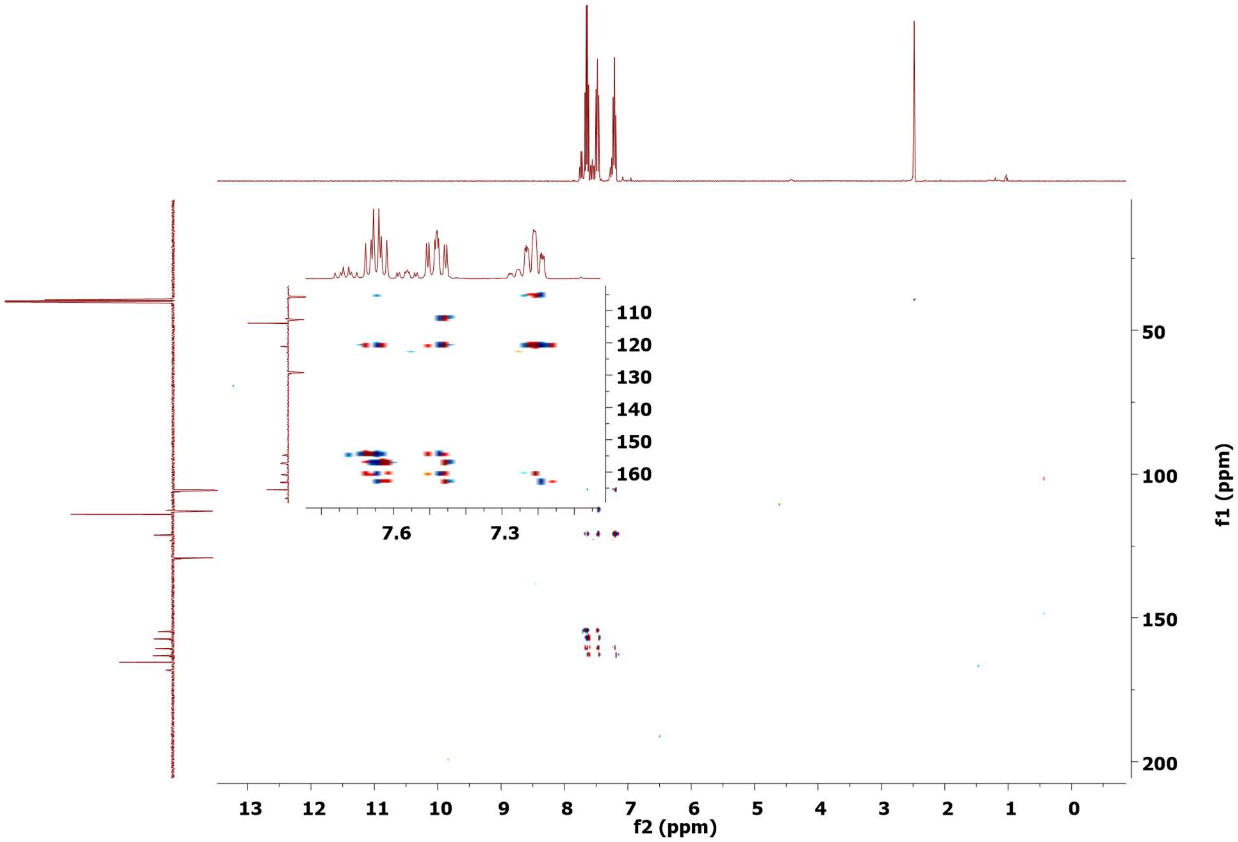

^{1}H NMR (DMSO-d6) spectrum of naphthalen-1-ylcarbamothioyl cyanide (1l')

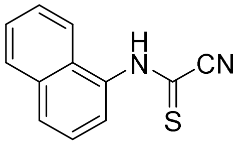

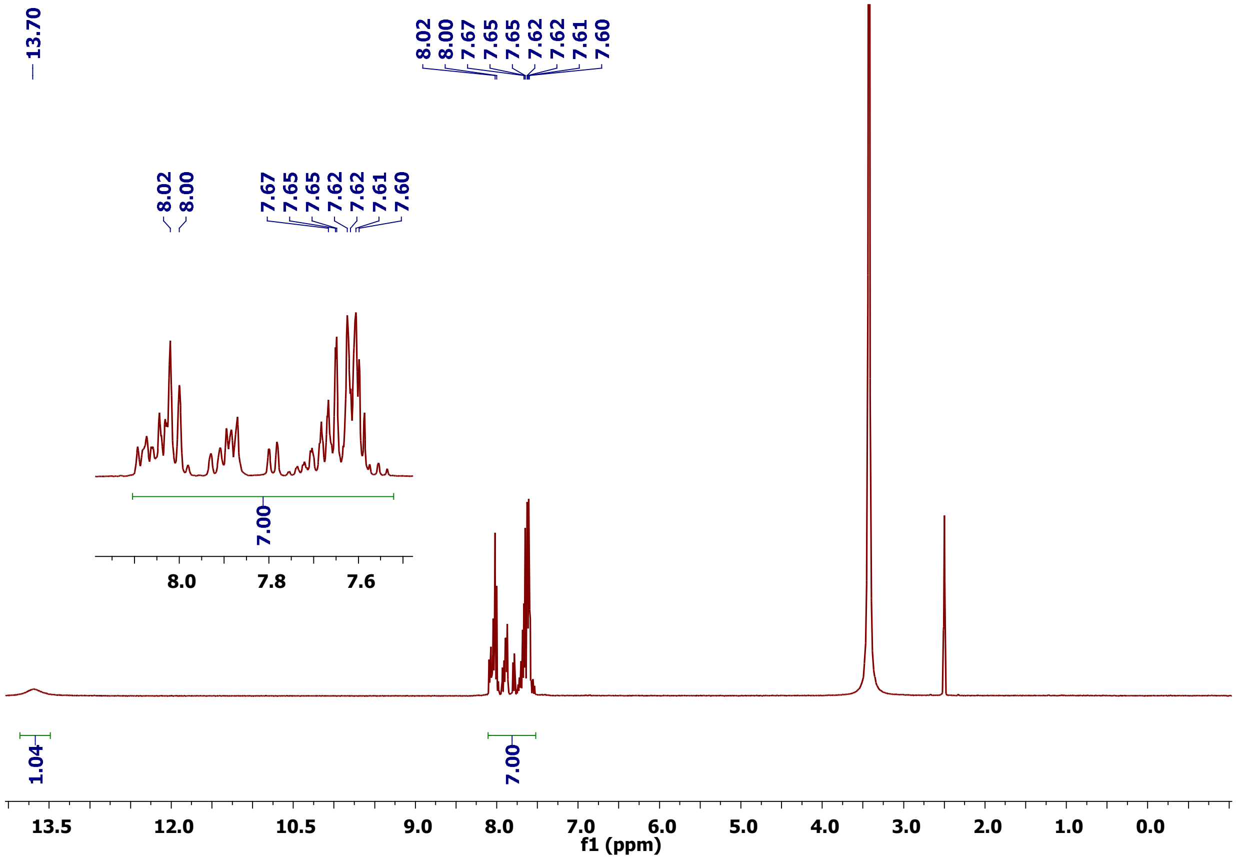

^{13}C NMR (DMSO-d6) spectrum of naphthalen-1-ylcarbamothioyl cyanide (11’)

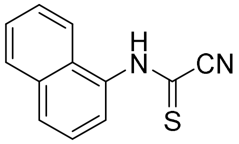

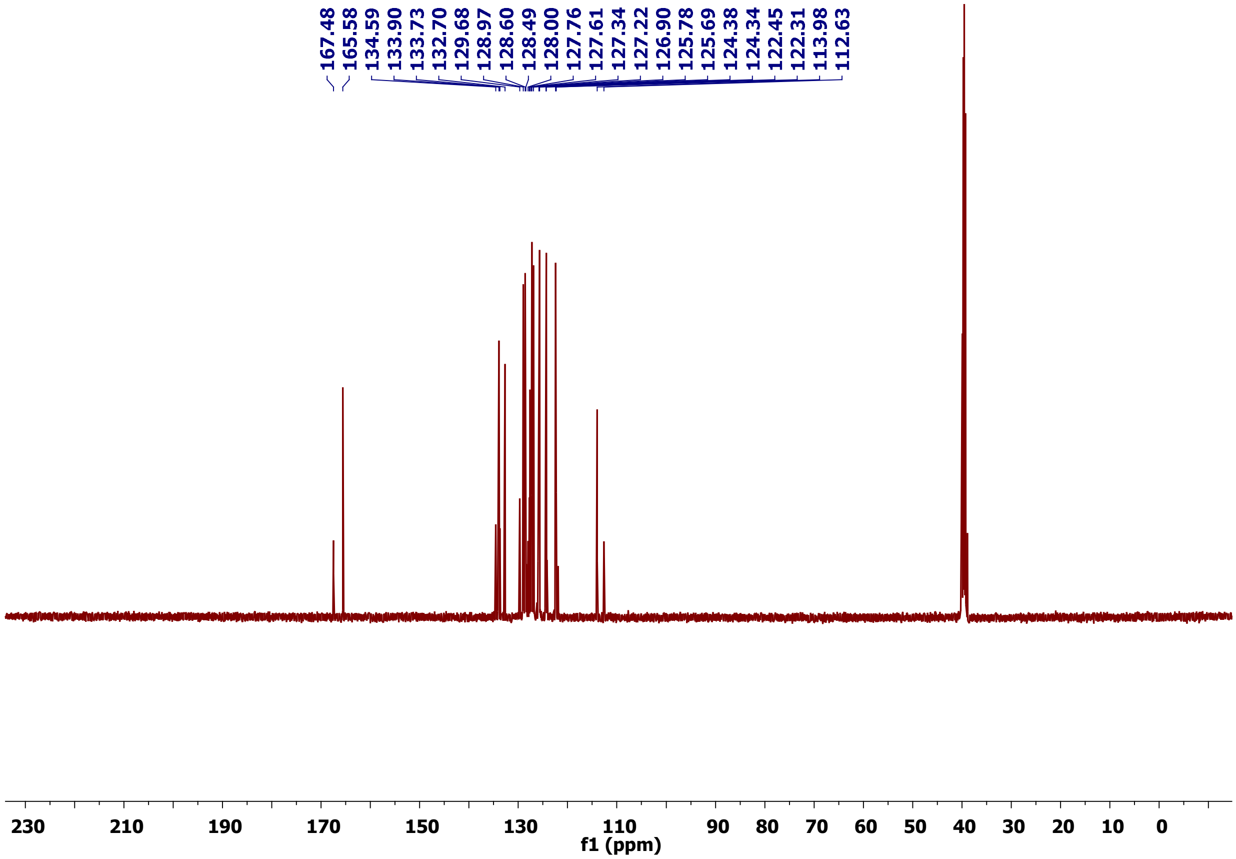

^{13}C CRAPT NMR (DMSO-d6) spectrum of naphthalen-1-ylcarbamothioyl cyanide (1l')

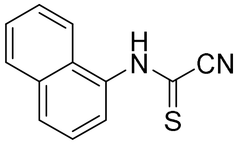

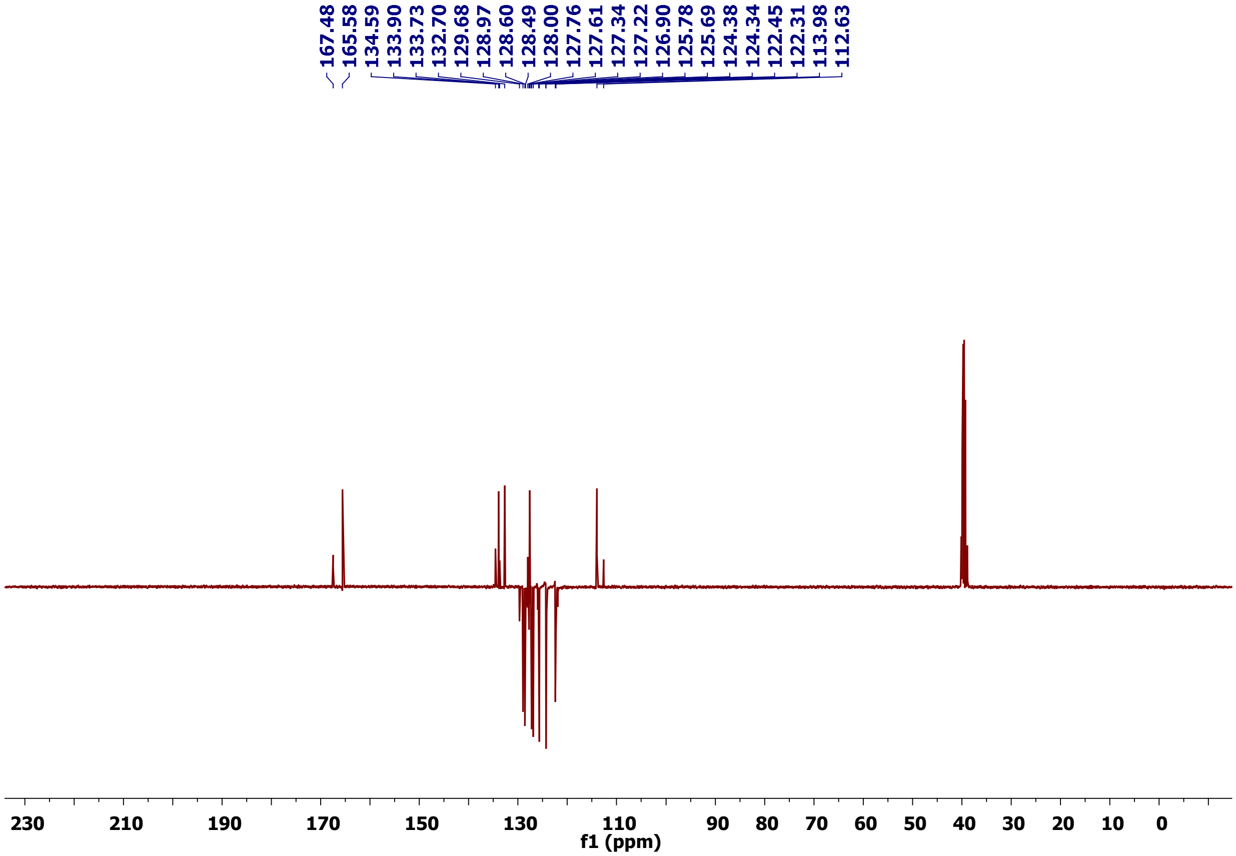

^{1}H-^{13}C-gHSQC NMR (DMSO-d6) spectrum of naphthalen-1-ylcarbamothioyl cyanide (1l')

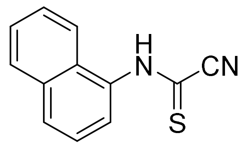

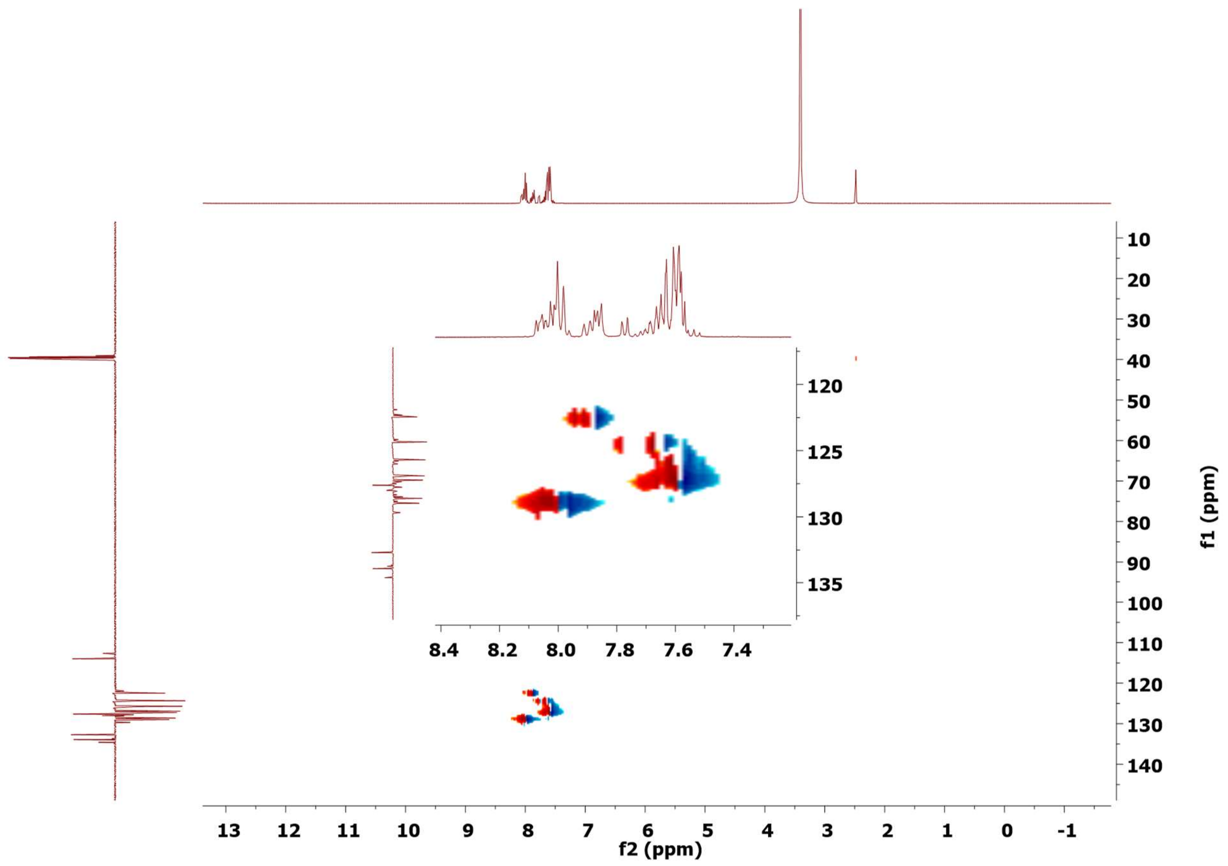

^{1}H-^{13}C-gHMBC NMR (DMSO-d6) spectrum of naphthalen-1-ylcarbamothioyl cyanide (1l’)

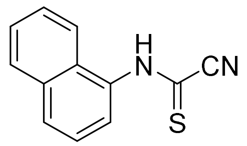

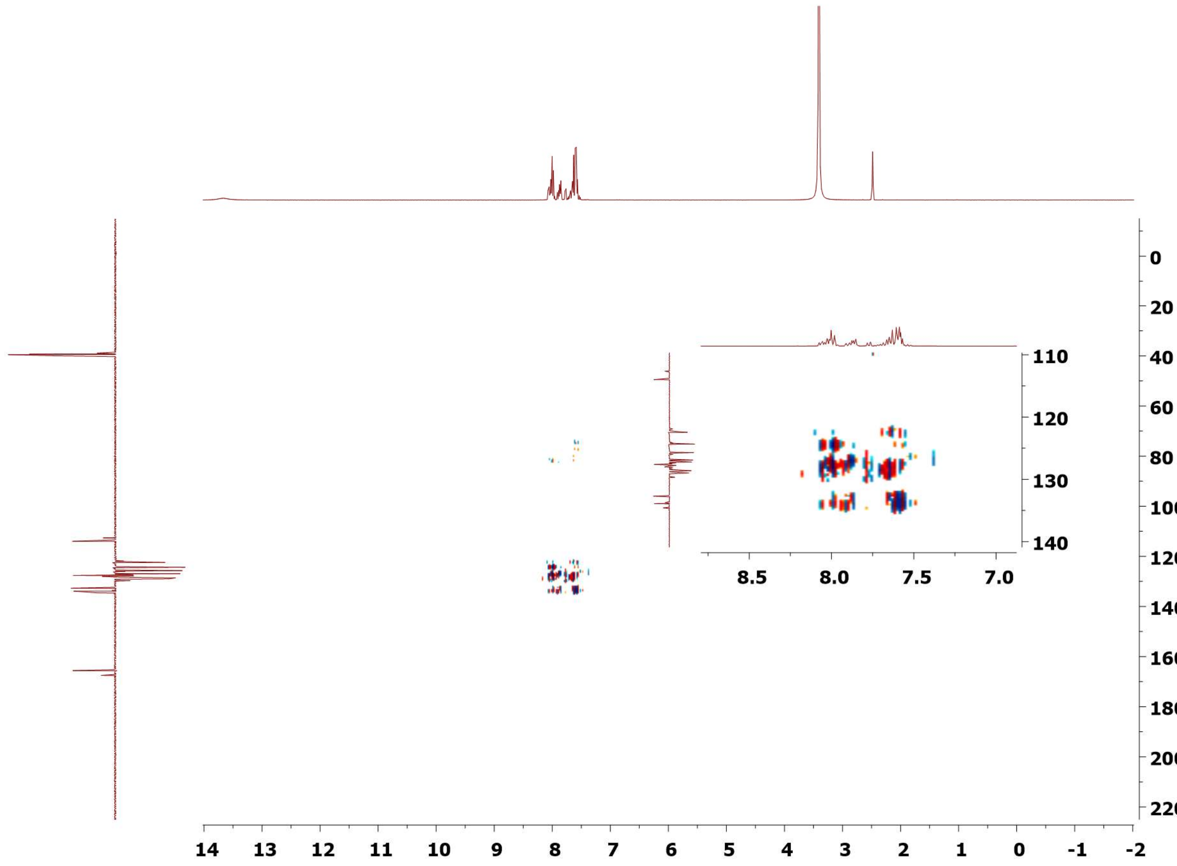

^{1}H NMR ($CDCl_3$) spectrum of (3-methoxyphenyl)carbamothioyl cyanide (1.91:1tautomeric ratio) (1m')

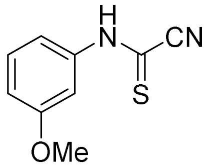

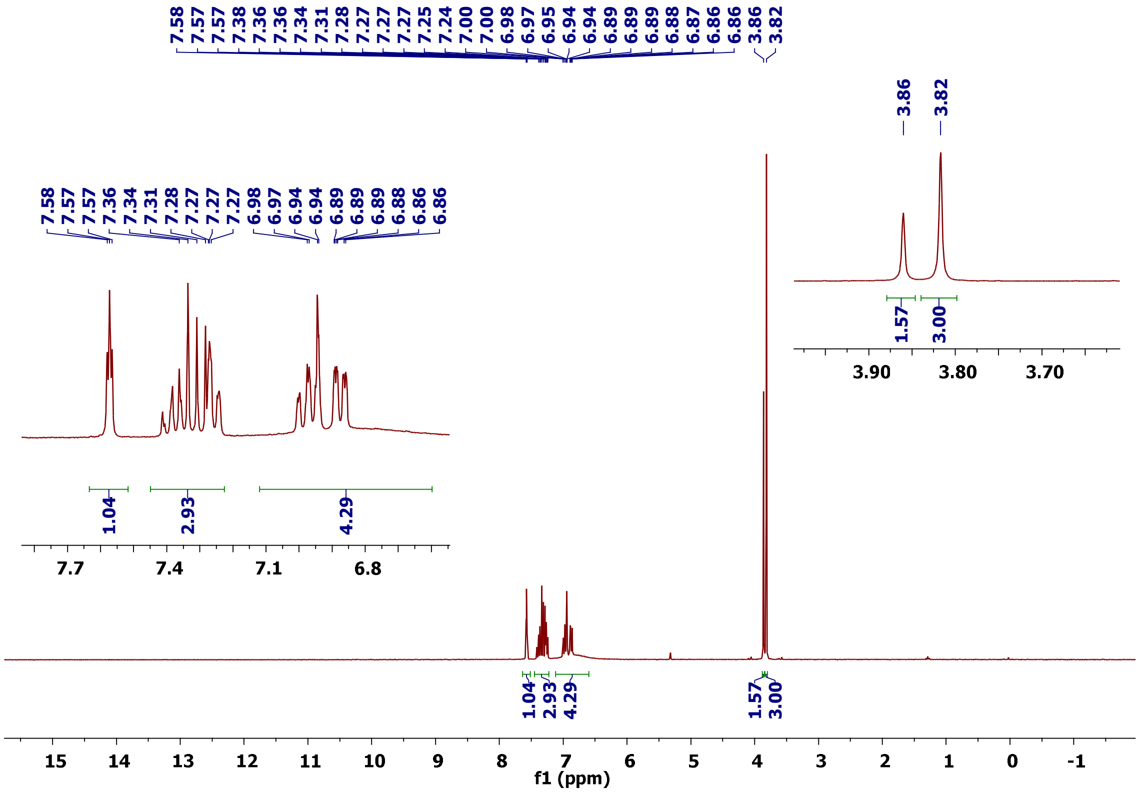

^{13}C-DEPT 90 NMR ($CDCl_3$) spectrum of (3-methoxyphenyl)carbamothioyl cyanide (1.91:1 tautomeric ratio) (1m')

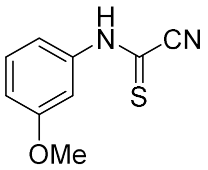

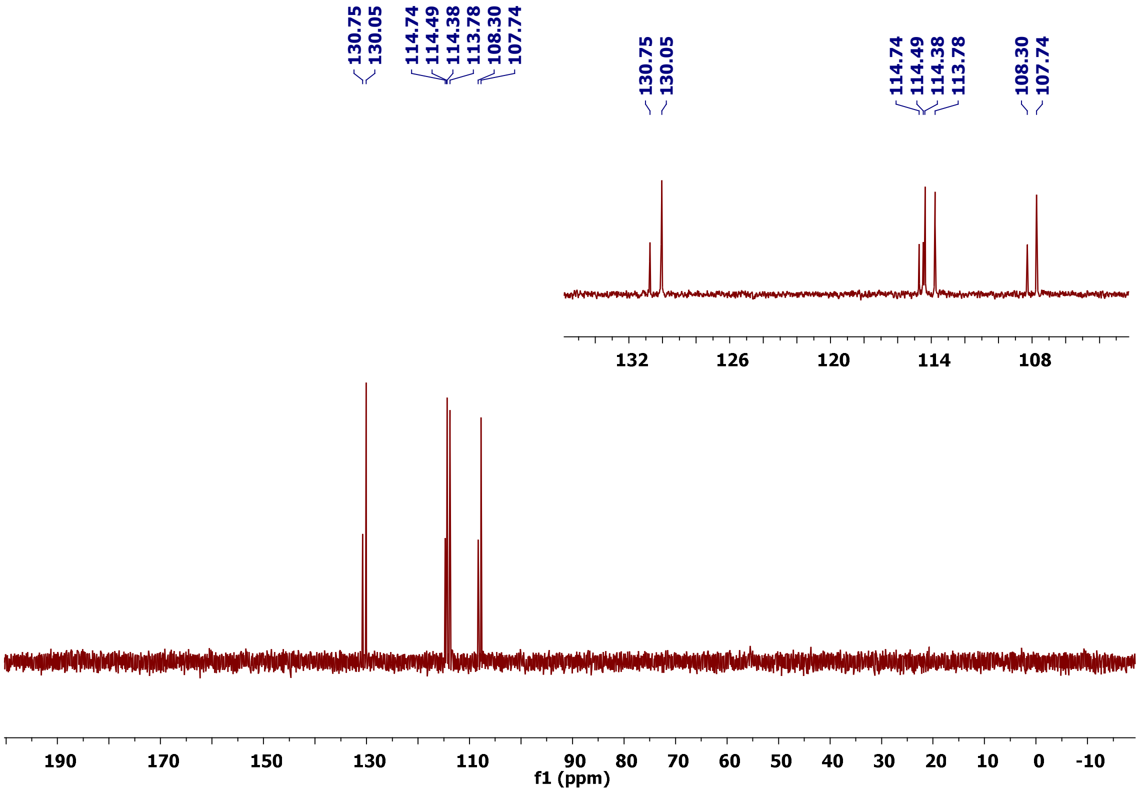

^{13}C-DEPT 135 NMR ($CDCl_3$) spectrum of (3-methoxyphenyl)carbamothioyl cyanide (1.91:1 tautomeric ratio) (1m')

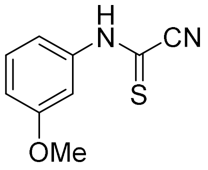

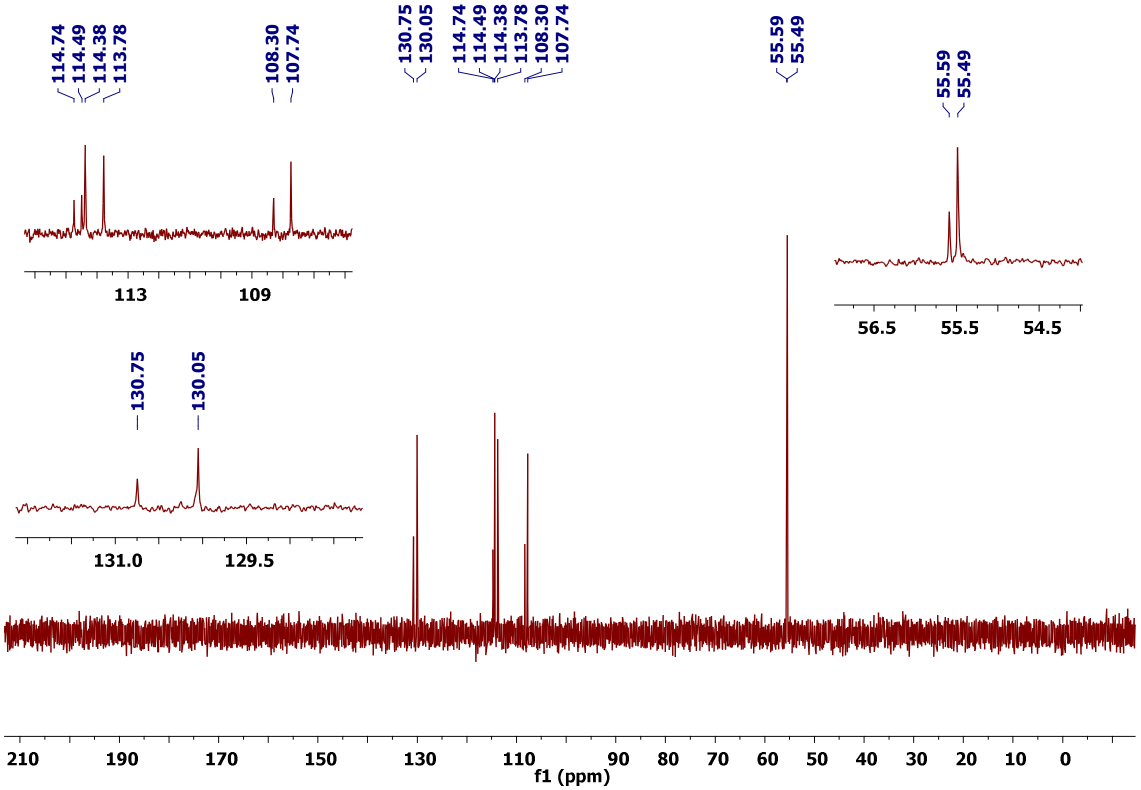

^{13}C NMR ($CDCl_3$) spectrum of (3-methoxyphenyl)carbamothioyl cyanide (1.91:1 tautomeric ratio) (1m')

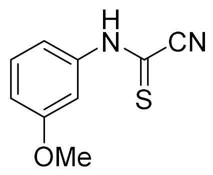

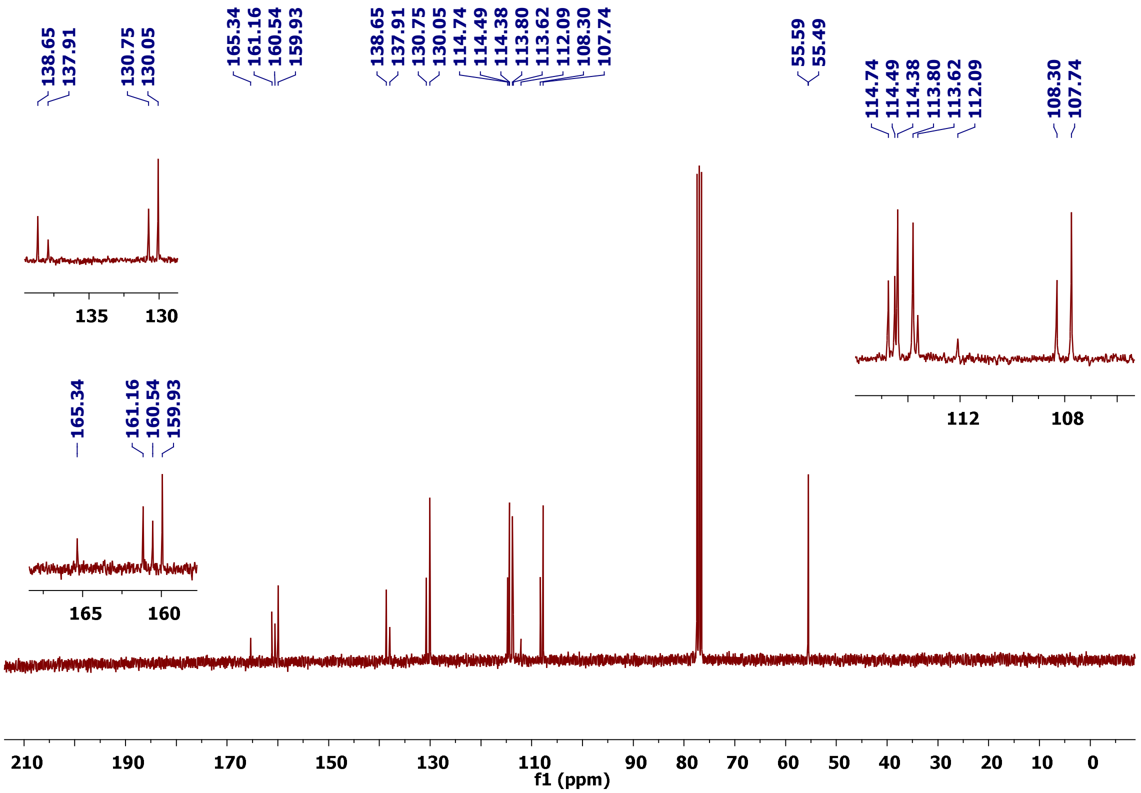

^{1}H NMR (DMSO-d6) spectrum of (3-(benzyloxy)phenyl)carbamothioyl cyanide (1:0.24 tautomeric ratio) (1n')

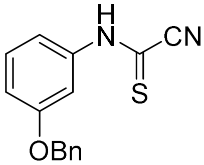

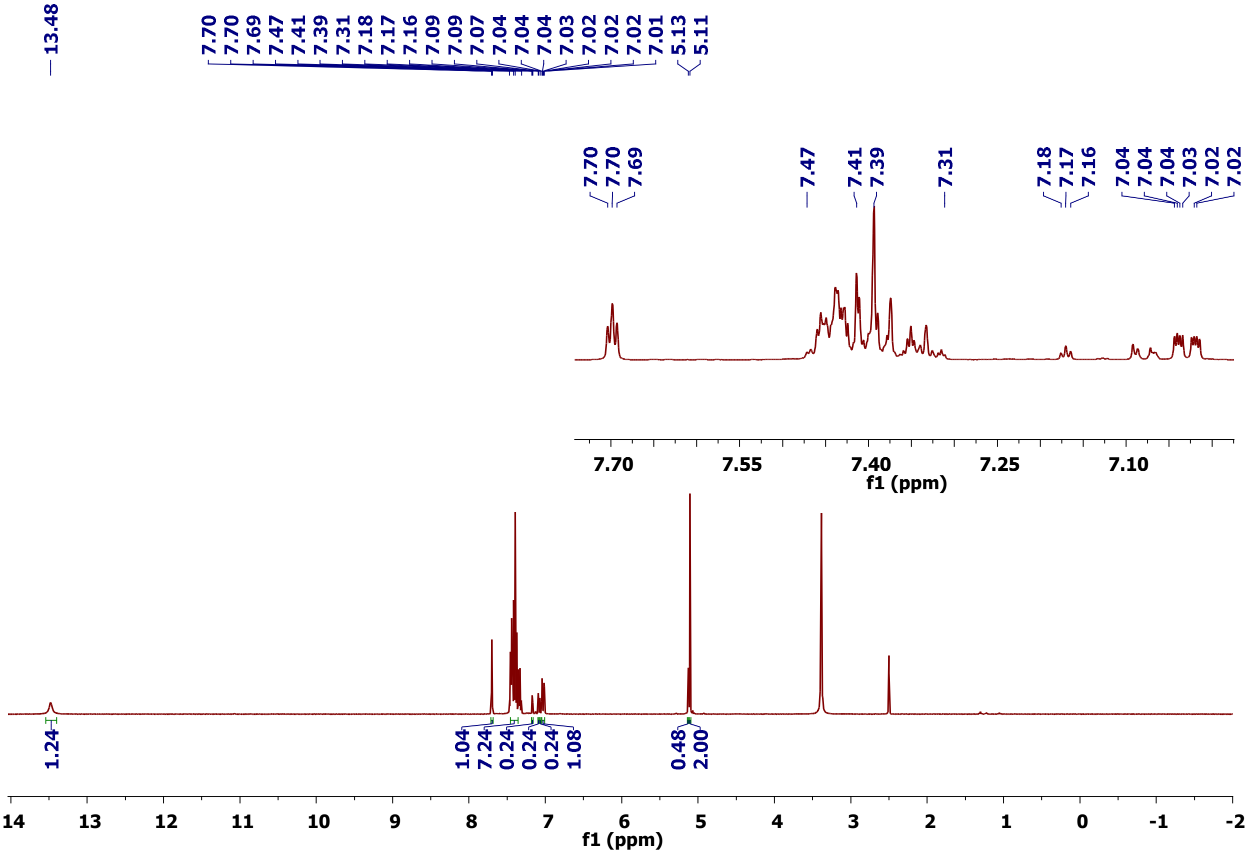

^{13}C NMR (DMSO-d6) spectrum of (3-(benzyloxy)phenyl)carbamothioyl cyanide (1:0.24 tautomeric ratio) (1n')

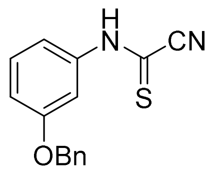

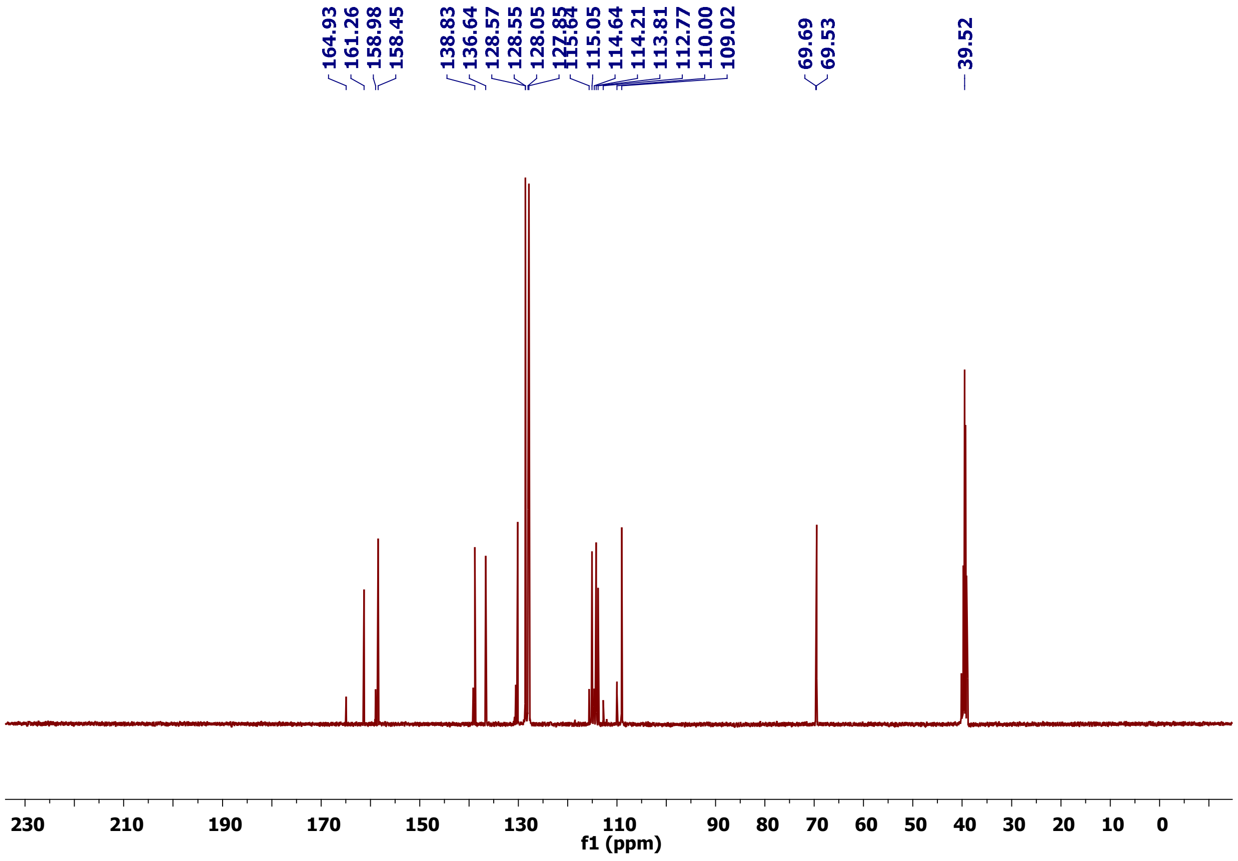

^{13}C CRAPT NMR (DMSO-d6) spectrum of (3-(benzyloxy)phenyl)carbamothioyl cyanide (1:0.24 tautomeric ratio) (1n’)

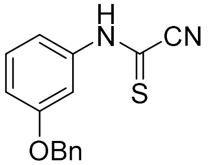

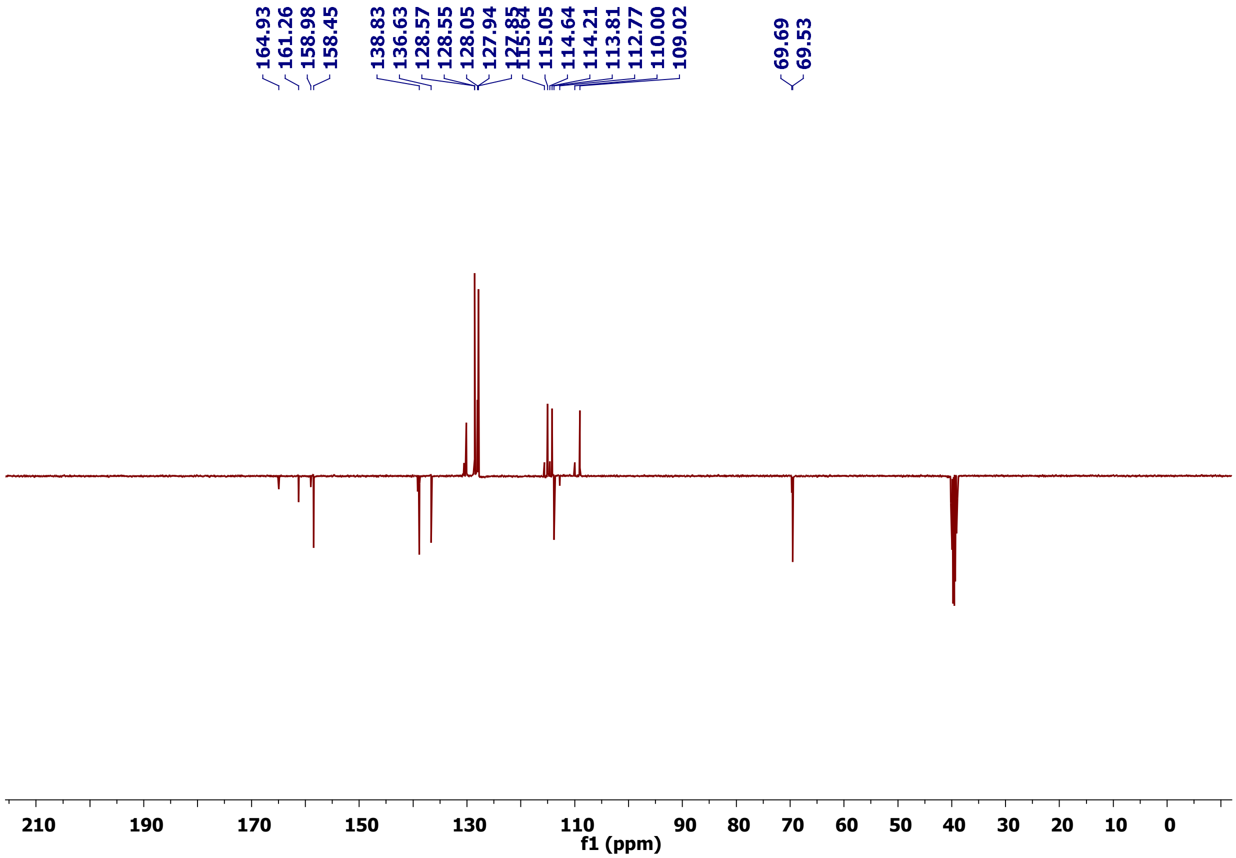

^{1}H-^{1}H-gDQCOSY NMR (DMSO-d6) spectrum of (3-(benzyloxy)phenyl)carbamothioyl cyanide (1:0.24 tautomeric ratio) (1n')

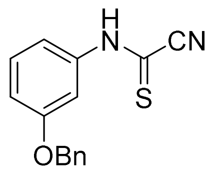

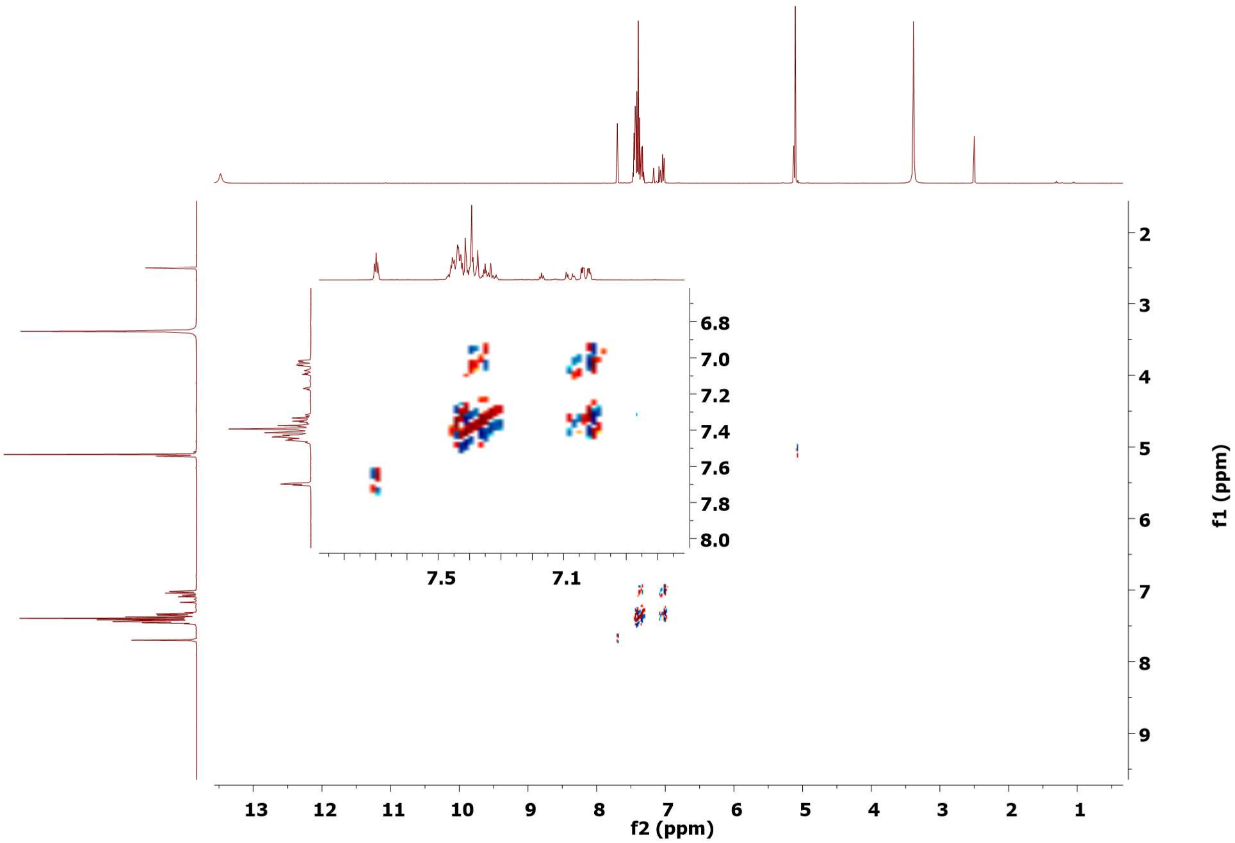

^{1}H-^{13}C-gHSQC NMR (DMSO-d6) spectrum of (3-(benzyloxy)phenyl)carbamothioyl cyanide (1:0.24 tautomeric ratio) (1n')

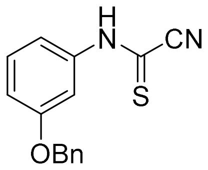

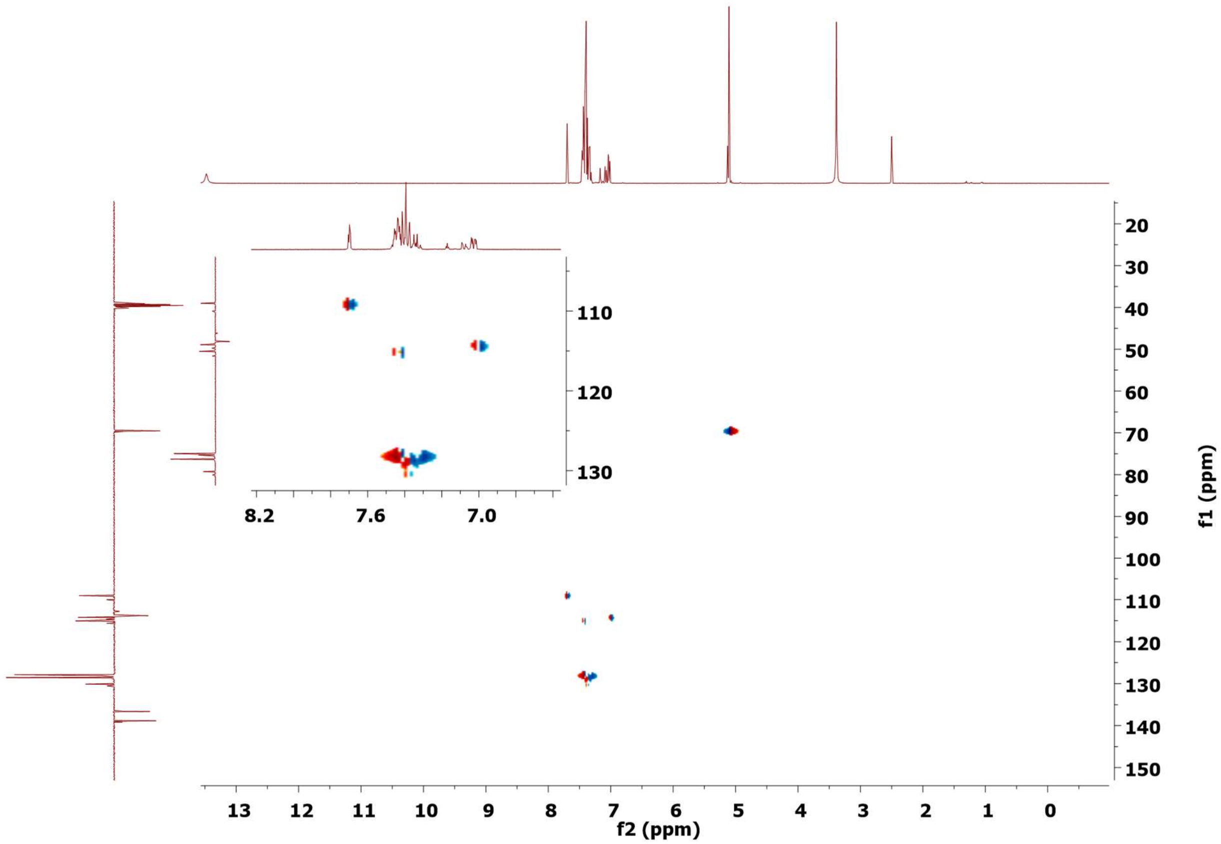

^{1}H-^{13}C-gHMBC NMR (DMSO-d6) spectrum of (3-(benzyloxy)phenyl)carbamothioyl cyanide (1:0.24 tautomeric ratio) (1n')

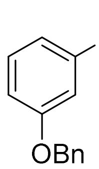

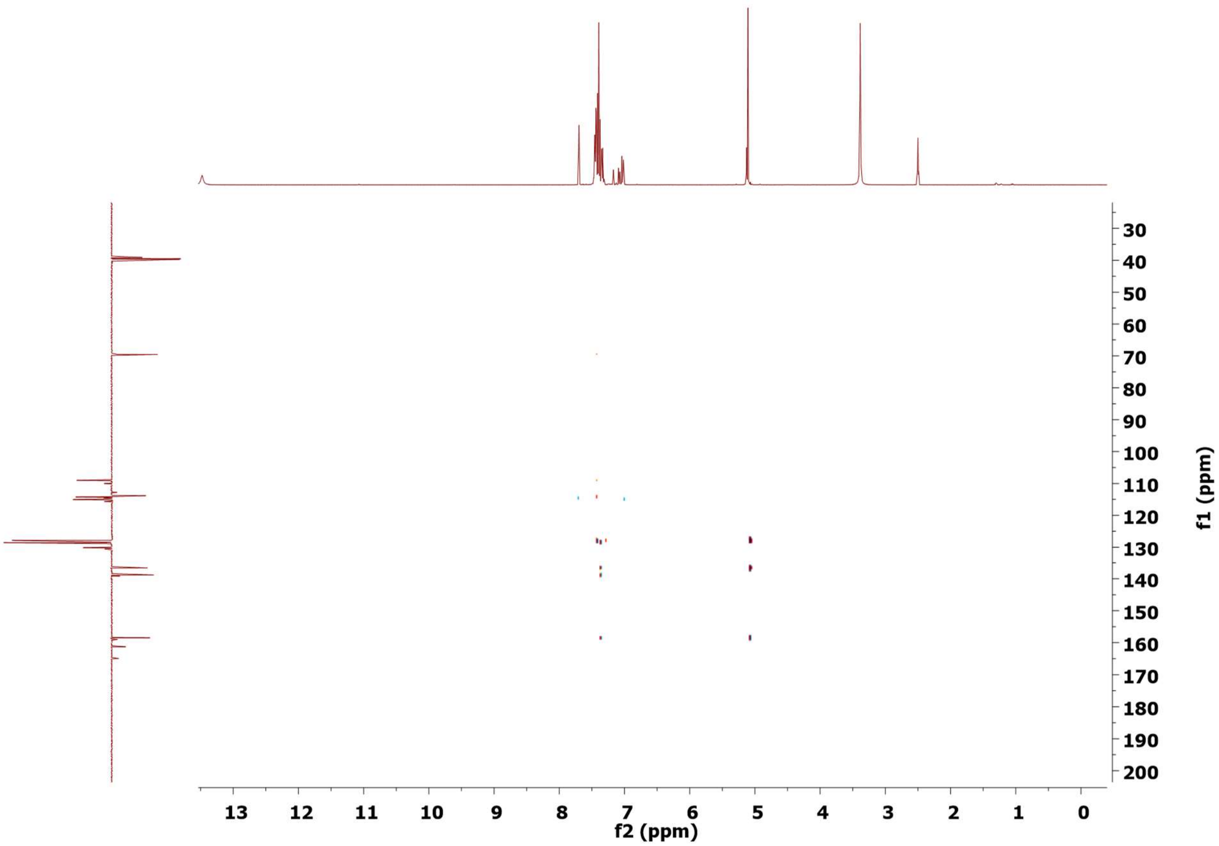

^{1}H NMR (DMSO-d6) spectrum of (3-(methylthio)phenyl)carbamothioyl cyanide (1:0.30 tautomeric ratio) (1o')

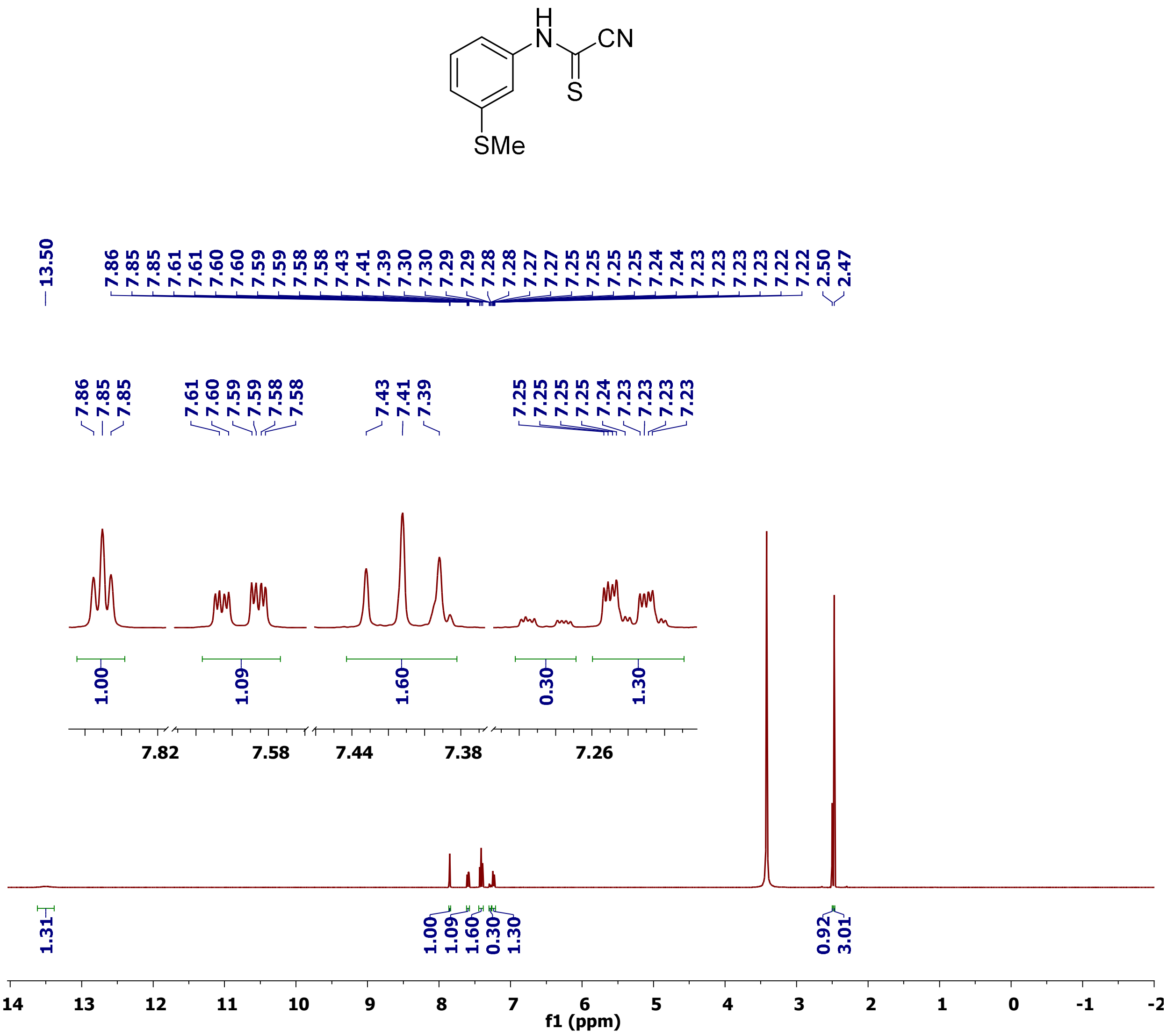

^{13}C NMR (DMSO-d6) spectrum of (3-(methylthio)phenyl)carbamothioyl cyanide (1:0.30 tautomeric ratio) (1o')

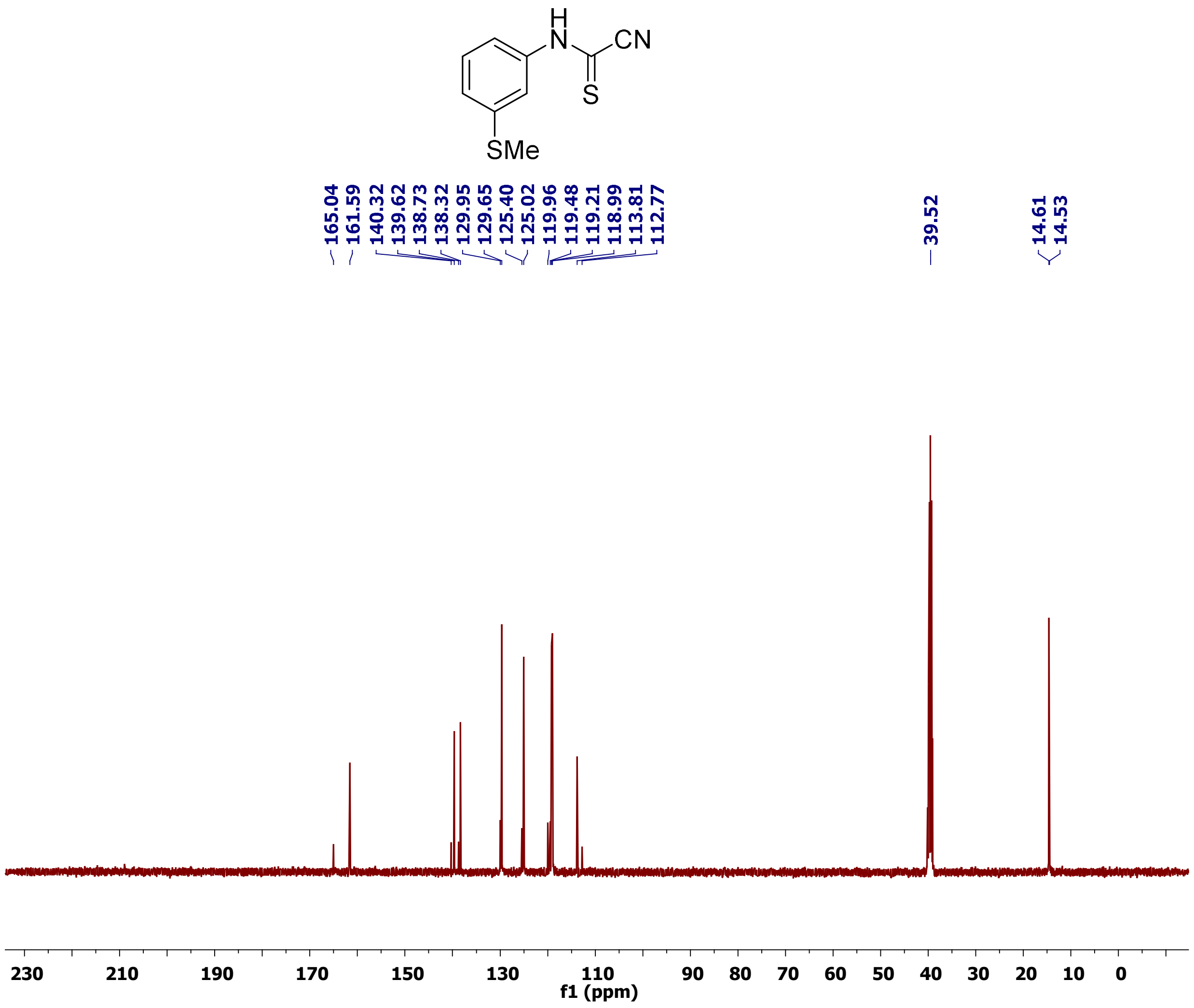

^{13}C CRAPT NMR (DMSO-d6) spectrum of (3-(methylthio)phenyl)carbamothioyl cyanide (1:0.30 tautomeric ratio) (1o’)

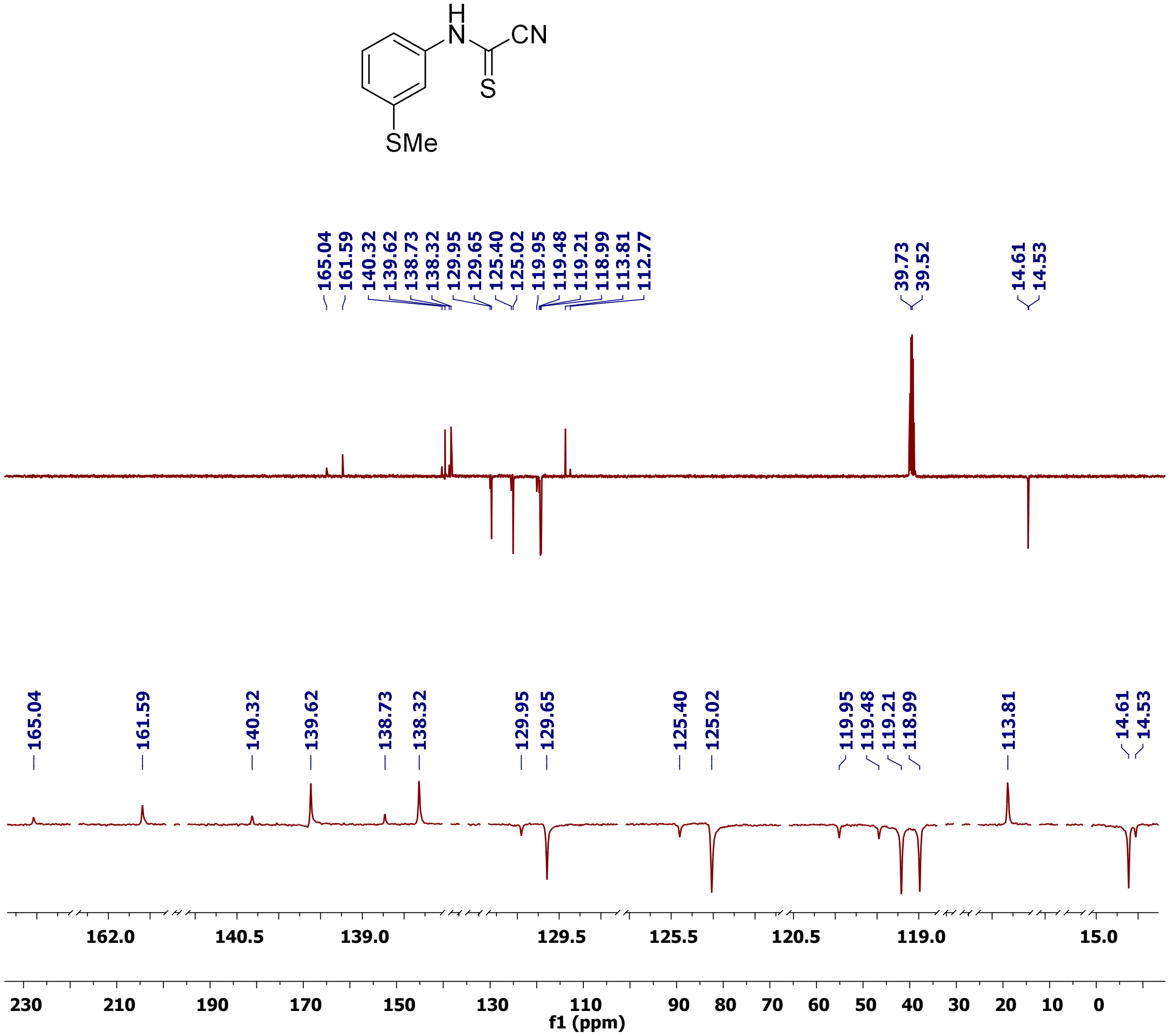

^{1}H-^{1}H-gDQCOSY NMR (DMSO-d6) spectrum of (3-(methylthio)phenyl)carbamothioyl cyanide (1:0.30 tautomeric ratio) (1o’)

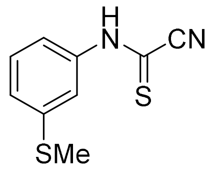

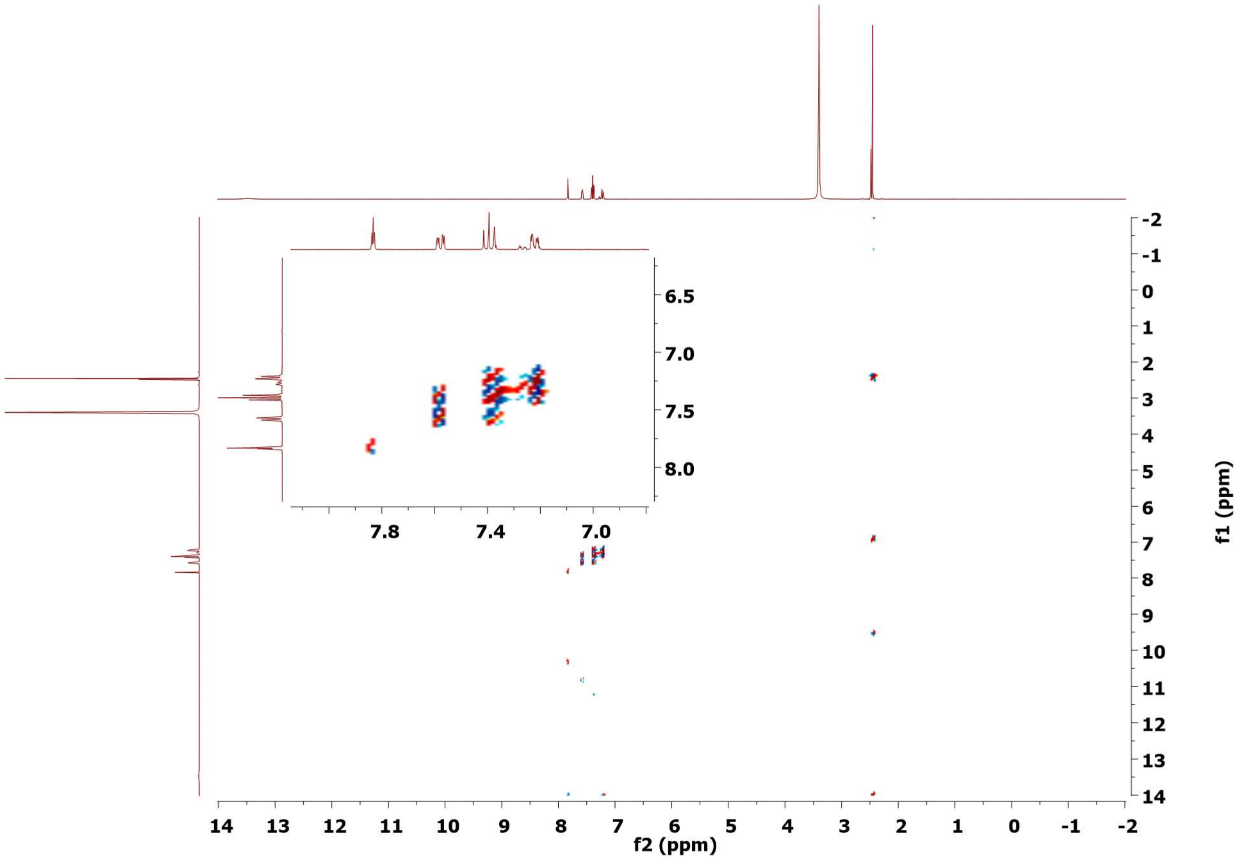

^{1}H-^{13}C-gHSQC NMR (DMSO-d6) spectrum of (3-(methylthio)phenyl)carbamothioyl cyanide (1:0.30 tautomeric ratio) (1o')

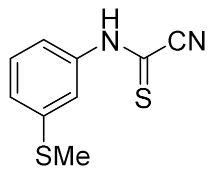

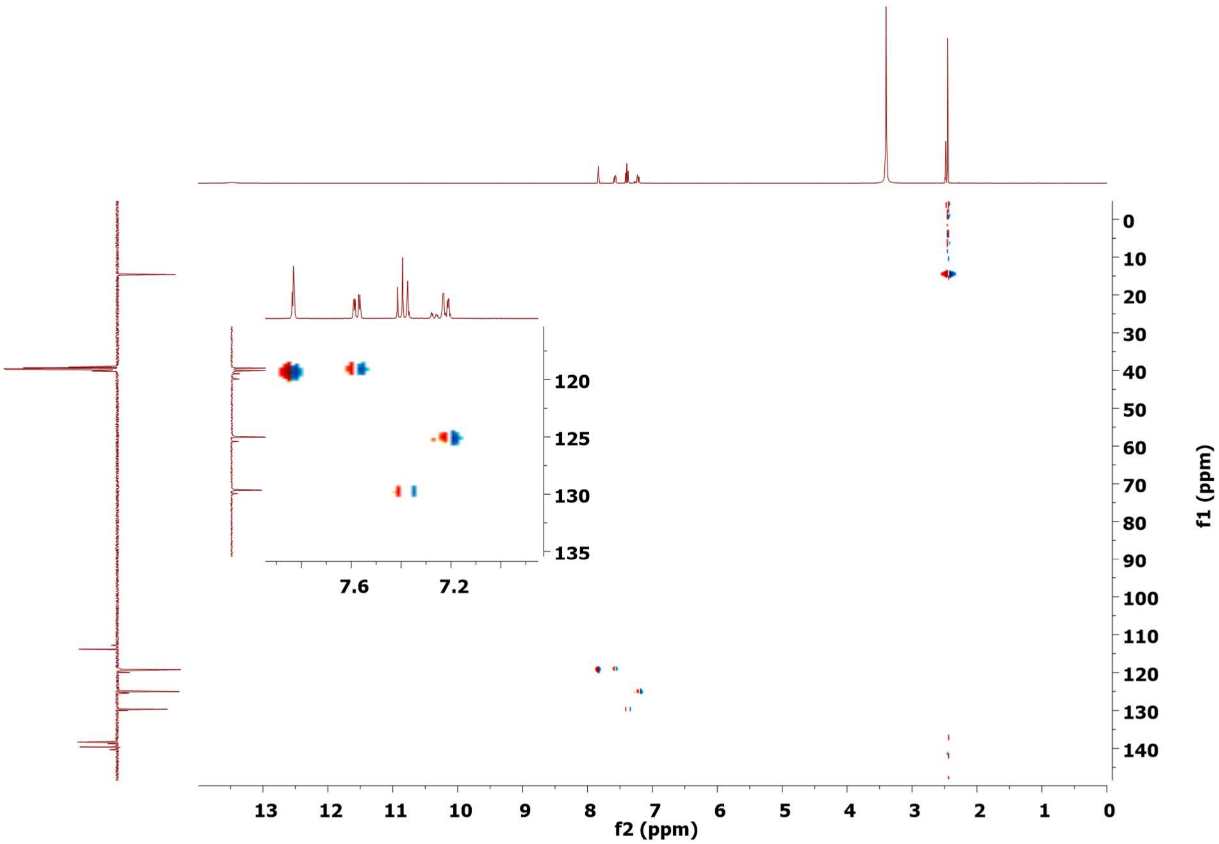

^{1}H-^{13}C-gHMBC NMR (DMSO-d6) spectrum of (3-(methylthio)phenyl)carbamothioyl cyanide (1:0.30 tautomeric ratio) (1o’)

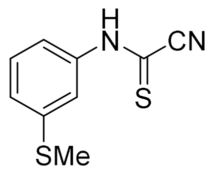

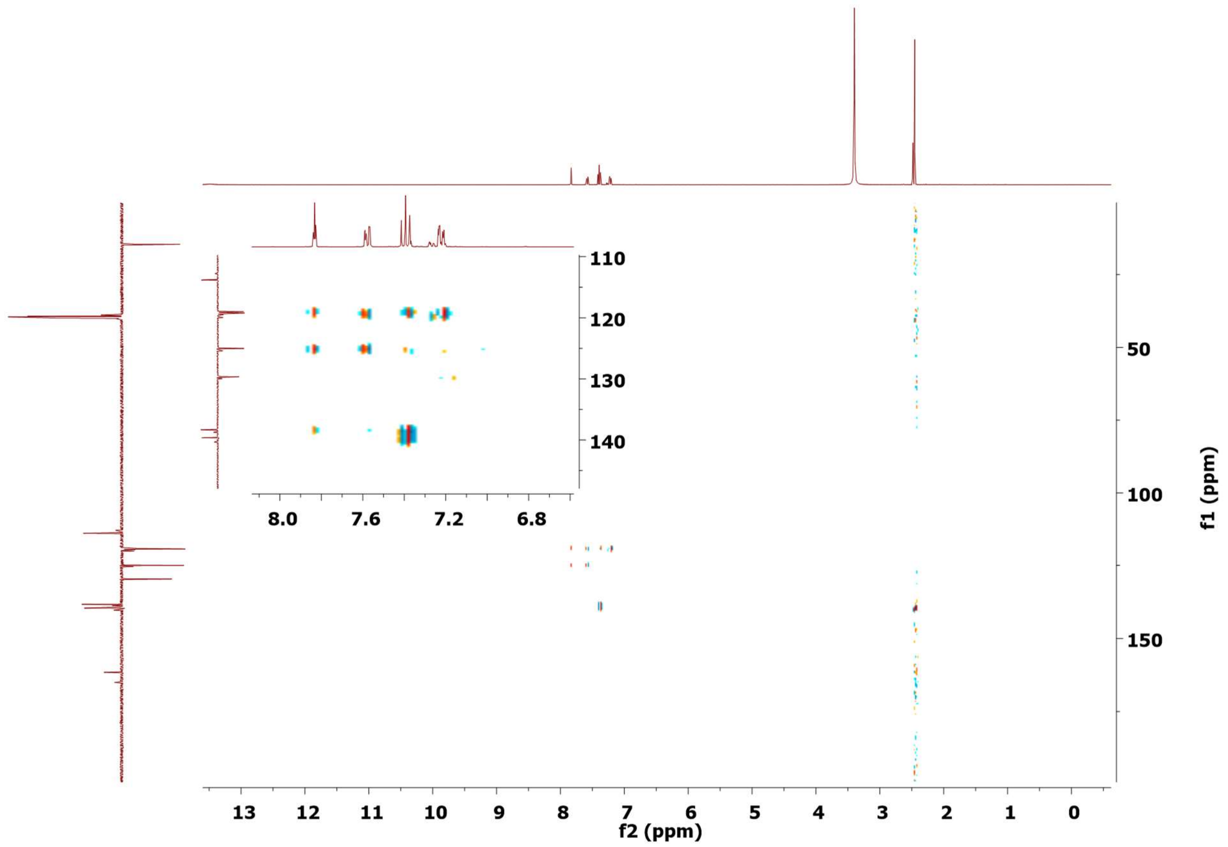

^{1}H NMR (DMSO-d6) spectrum of (3,4-dimethoxyphenyl)carbamothioyl cyanide (1:0.23 tautomeric ratio) (1p’)

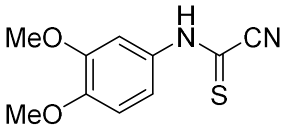

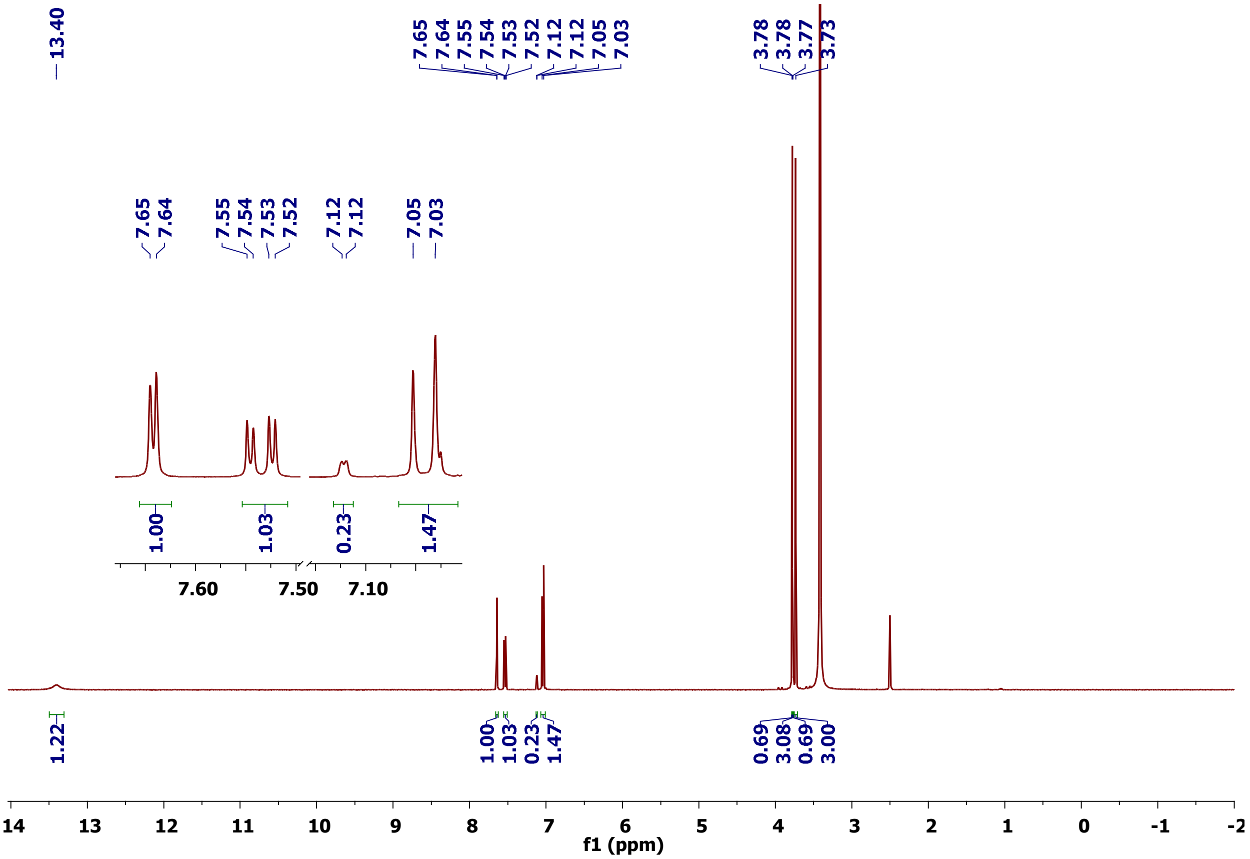

^{13}C NMR (DMSO-d6) spectrum of (3,4-dimethoxyphenyl)carbamothioyl cyanide (1:0.23 tautomeric ratio) (1p')

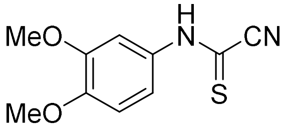

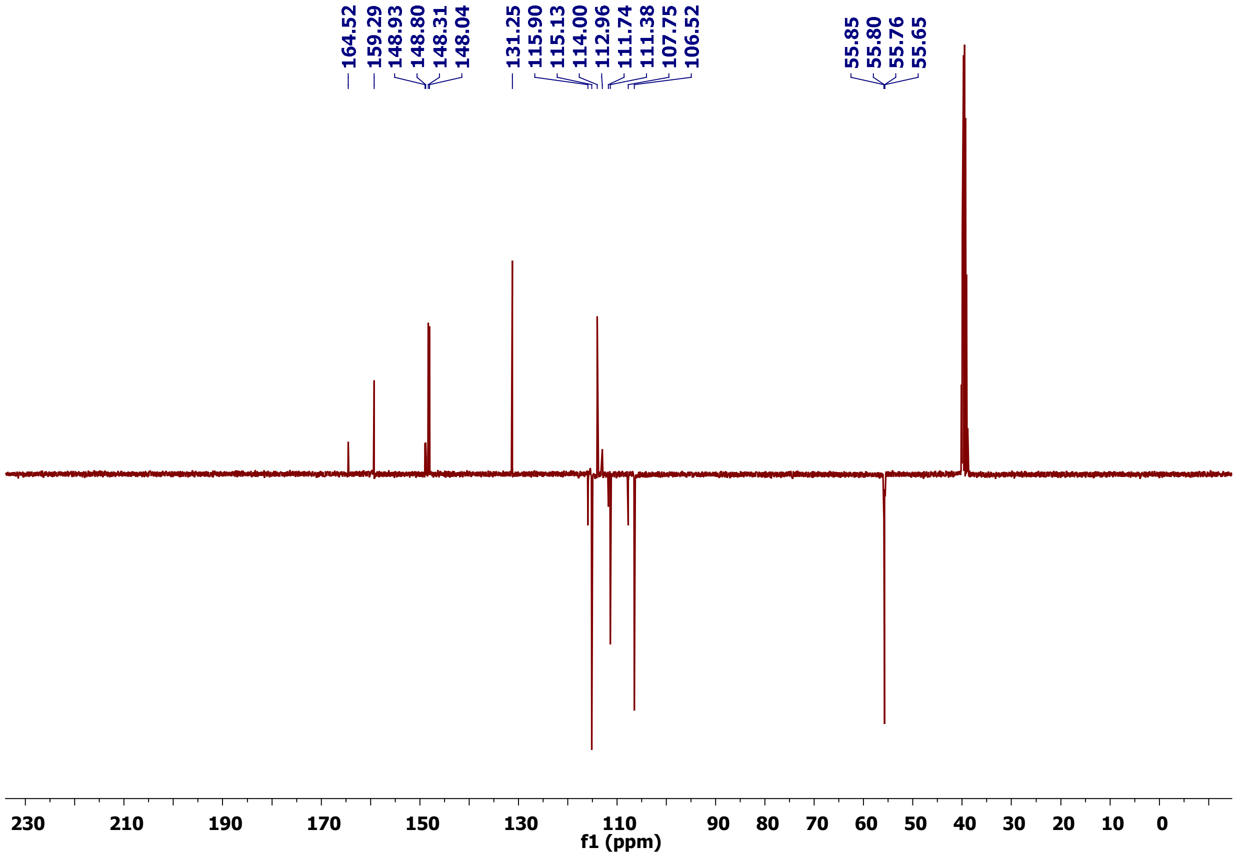

^{13}C CRAPT NMR (DMSO-d6) spectrum of (3,4-dimethoxyphenyl)carbamothioyl cyanide (1:0.23 tautomeric ratio) (1p')

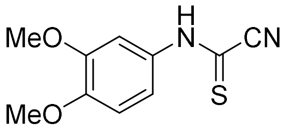

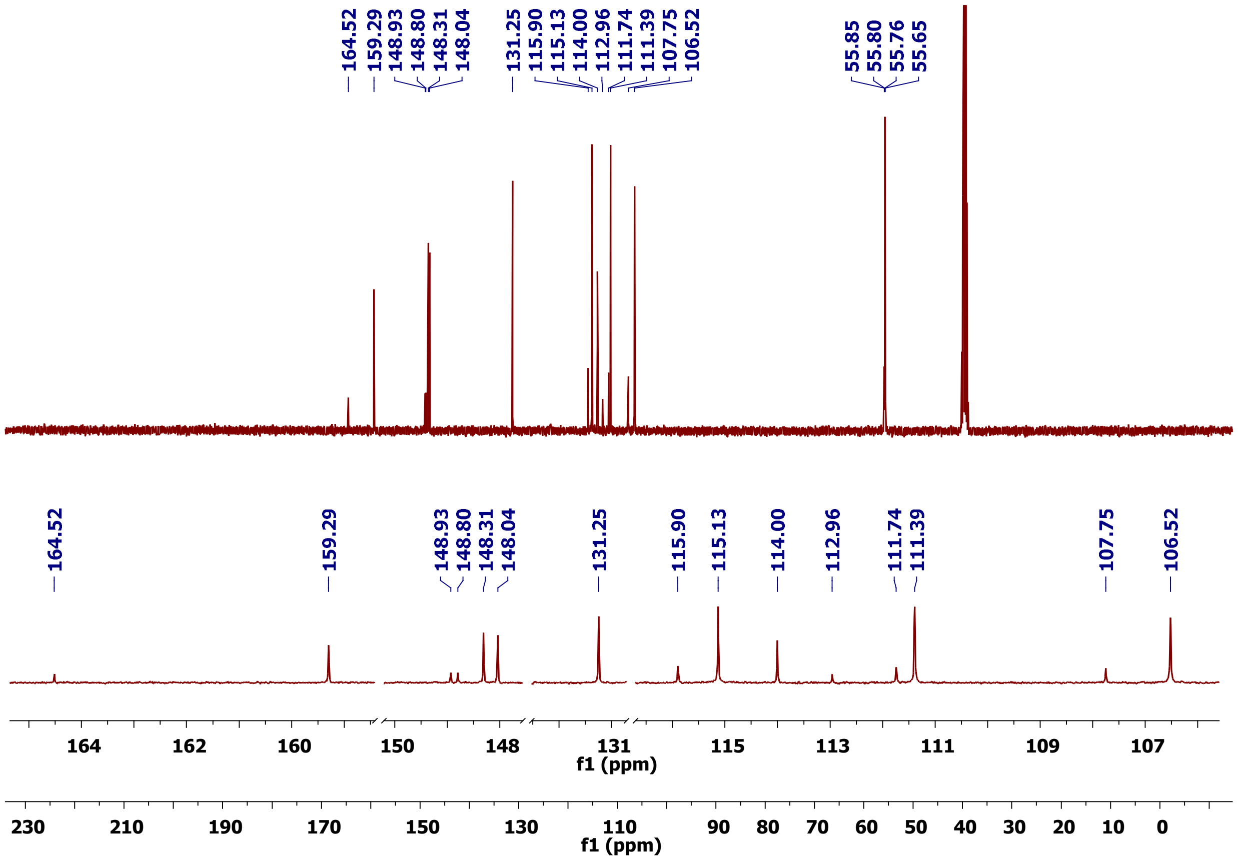

^{1}H-^{1}H-gDQCOSY NMR (DMSO-d6) spectrum of (3,4-dimethoxyphenyl)carbamothioyl cyanide (1:0.23 tautomeric ratio) (1p')

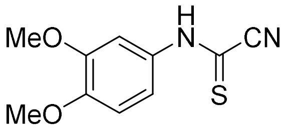

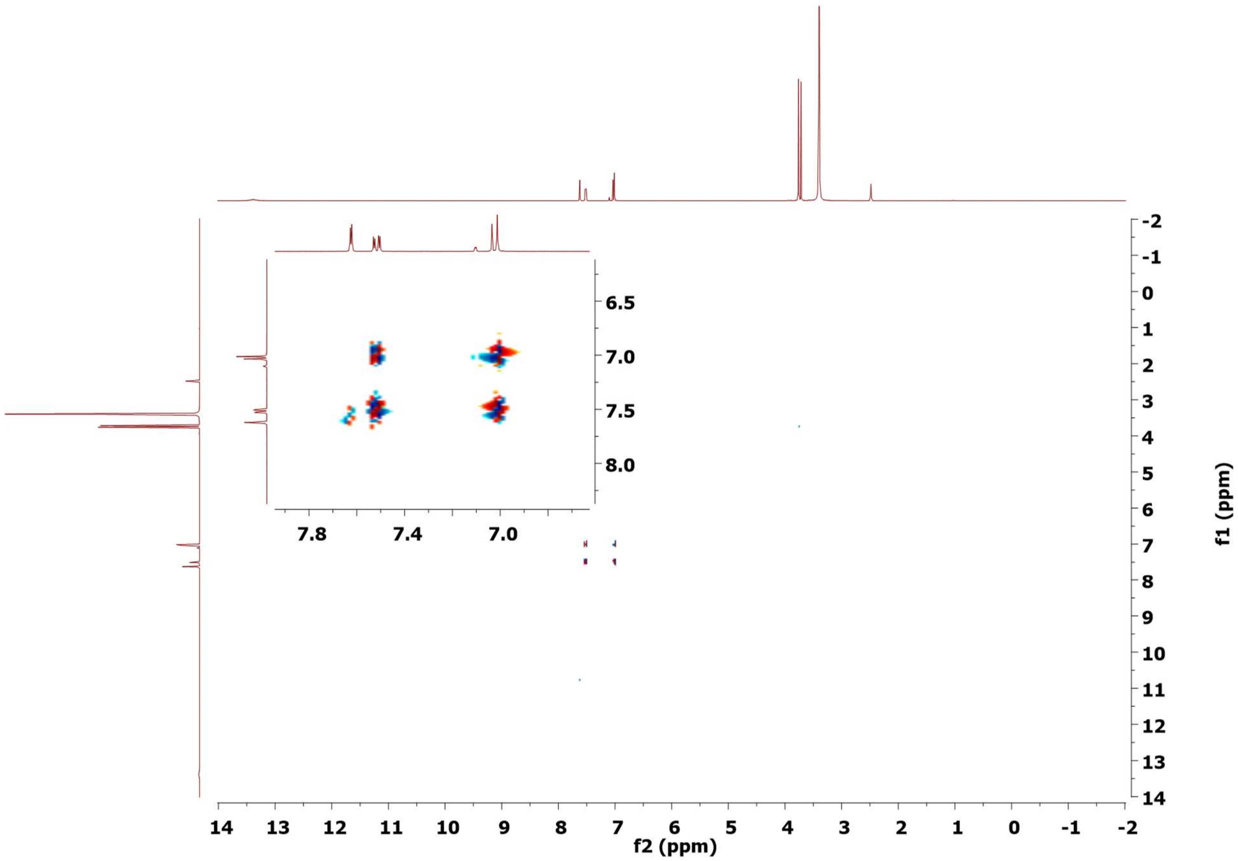

^1H-^{13}C-gHSQC NMR (DMSO-d6) spectrum of (3,4-dimethoxyphenyl)carbamothioyl cyanide (1:0.23 tautomeric ratio) (1p')

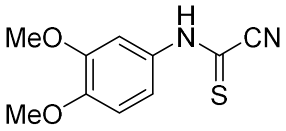

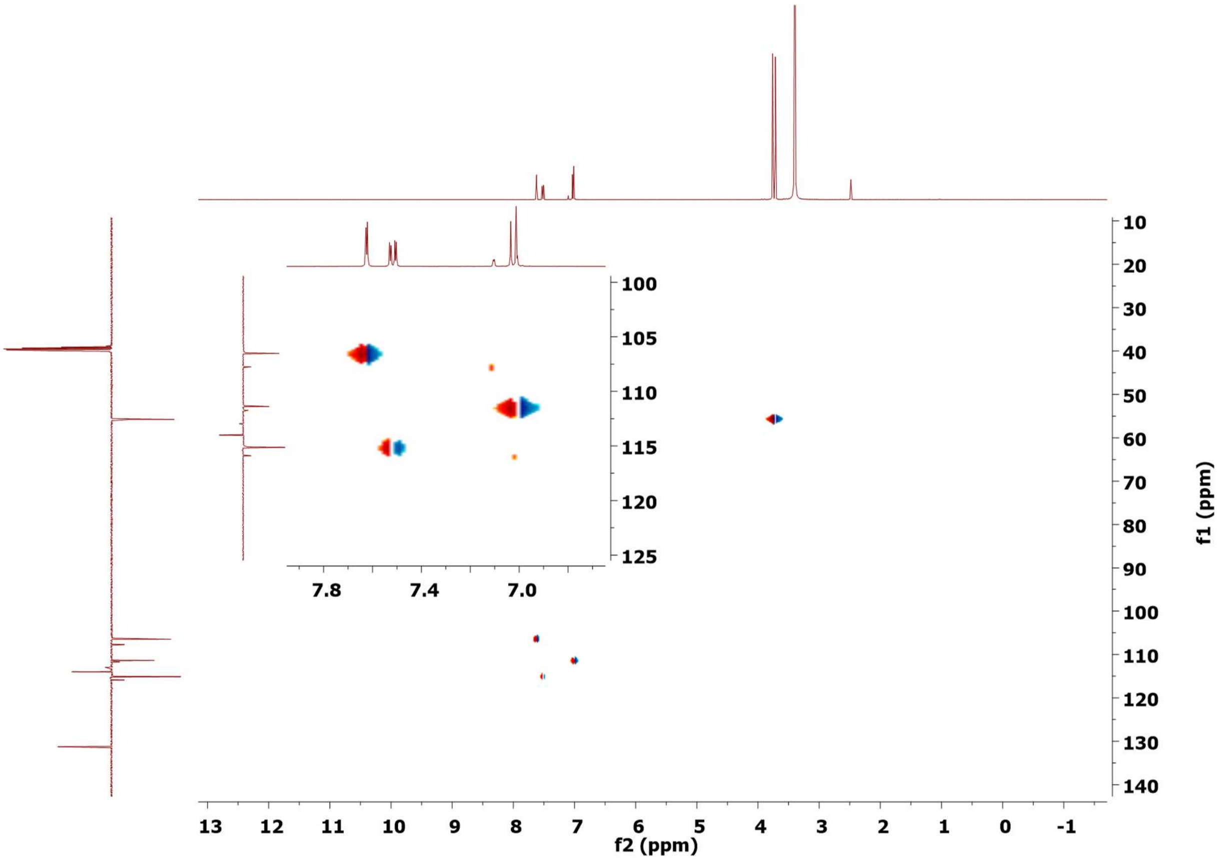

^{1}H-^{13}C-gHMBC NMR (DMSO-d6) spectrum of (3,4-dimethoxyphenyl)carbamothioyl cyanide (1:0.23 tautomeric ratio) (1p’)

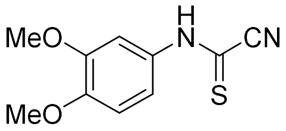

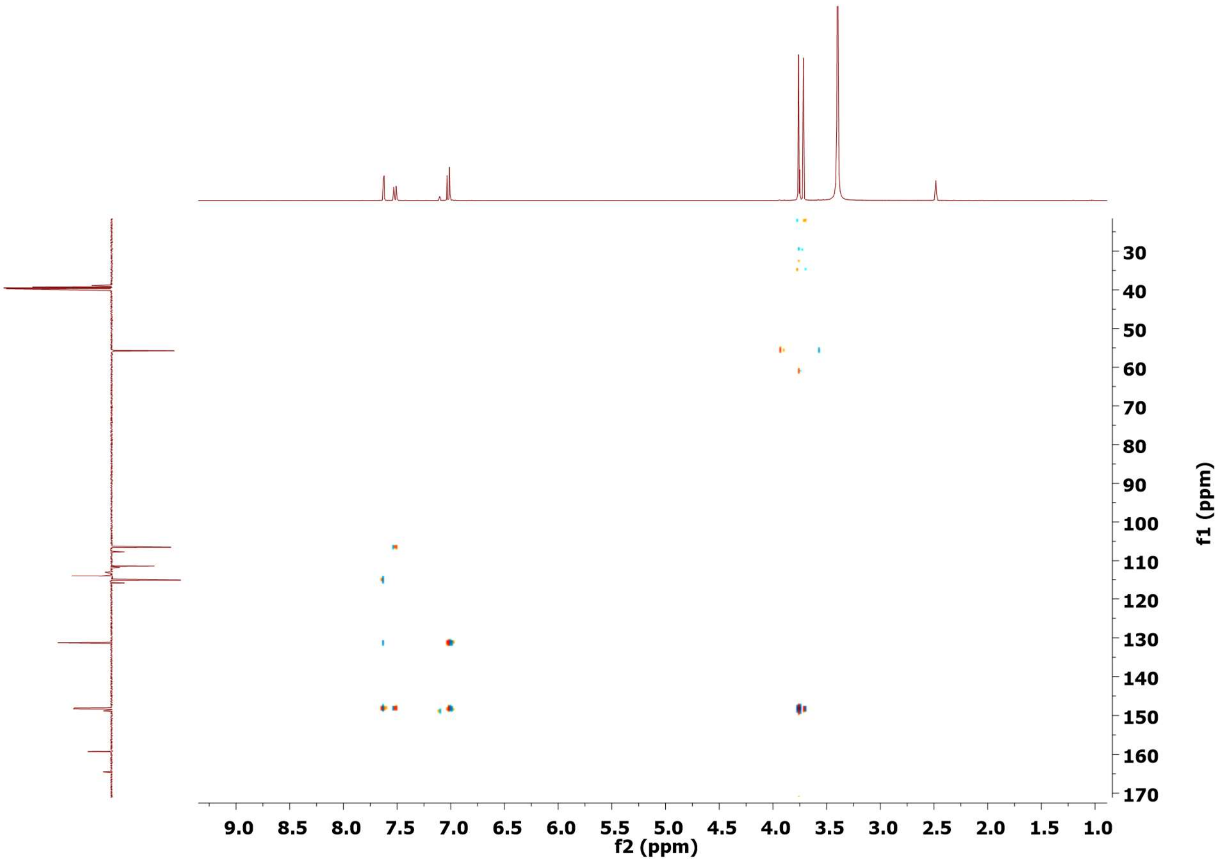

^{1}H NMR (DMSO-d6) spectrum of (2,5-dimethoxyphenyl)carbamothioyl cyanide (1:0.52 tautomeric ratio) (1q')

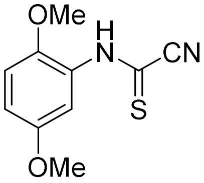

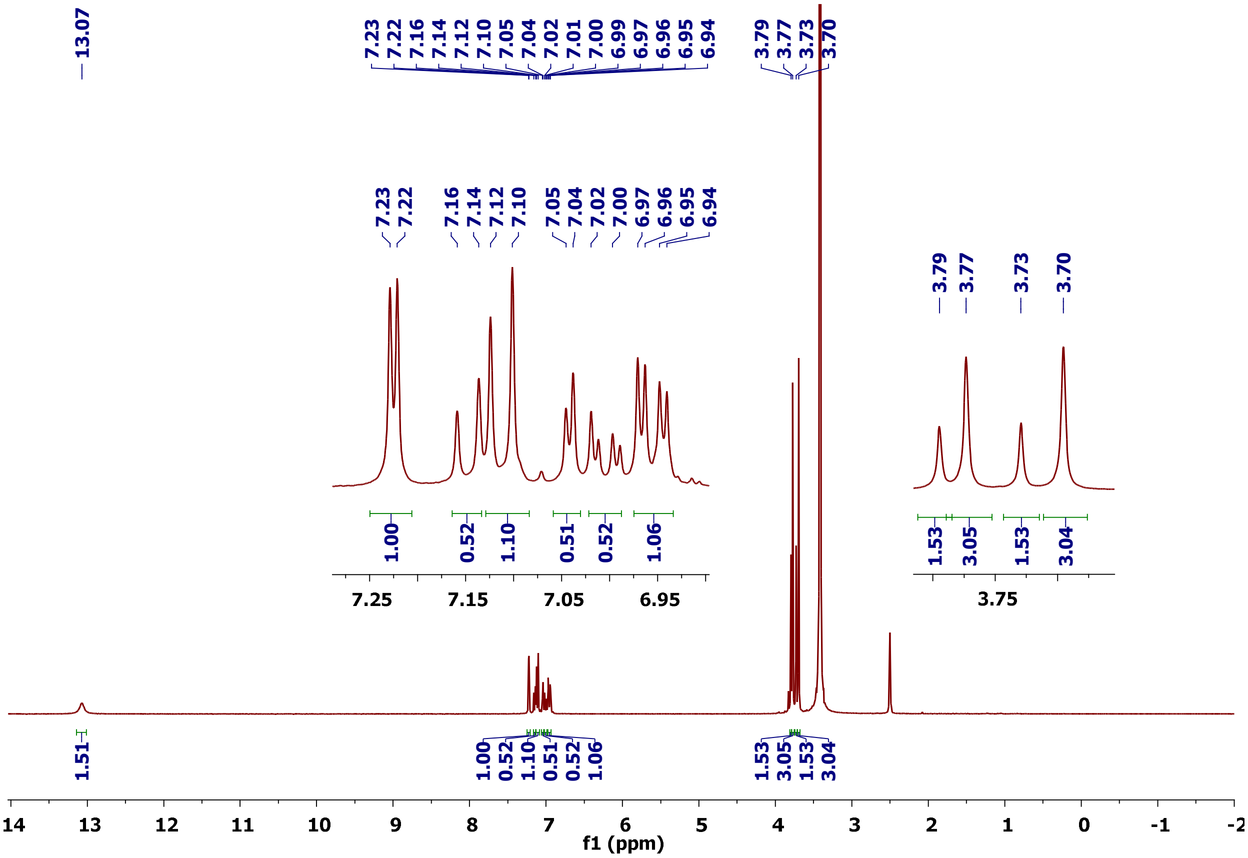

^{13}C NMR (DMSO-d6) spectrum of (2,5-dimethoxyphenyl)carbamothioyl cyanide (1:0.52 tautomeric ratio) (1q')

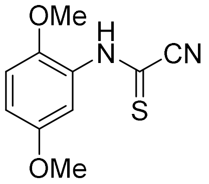

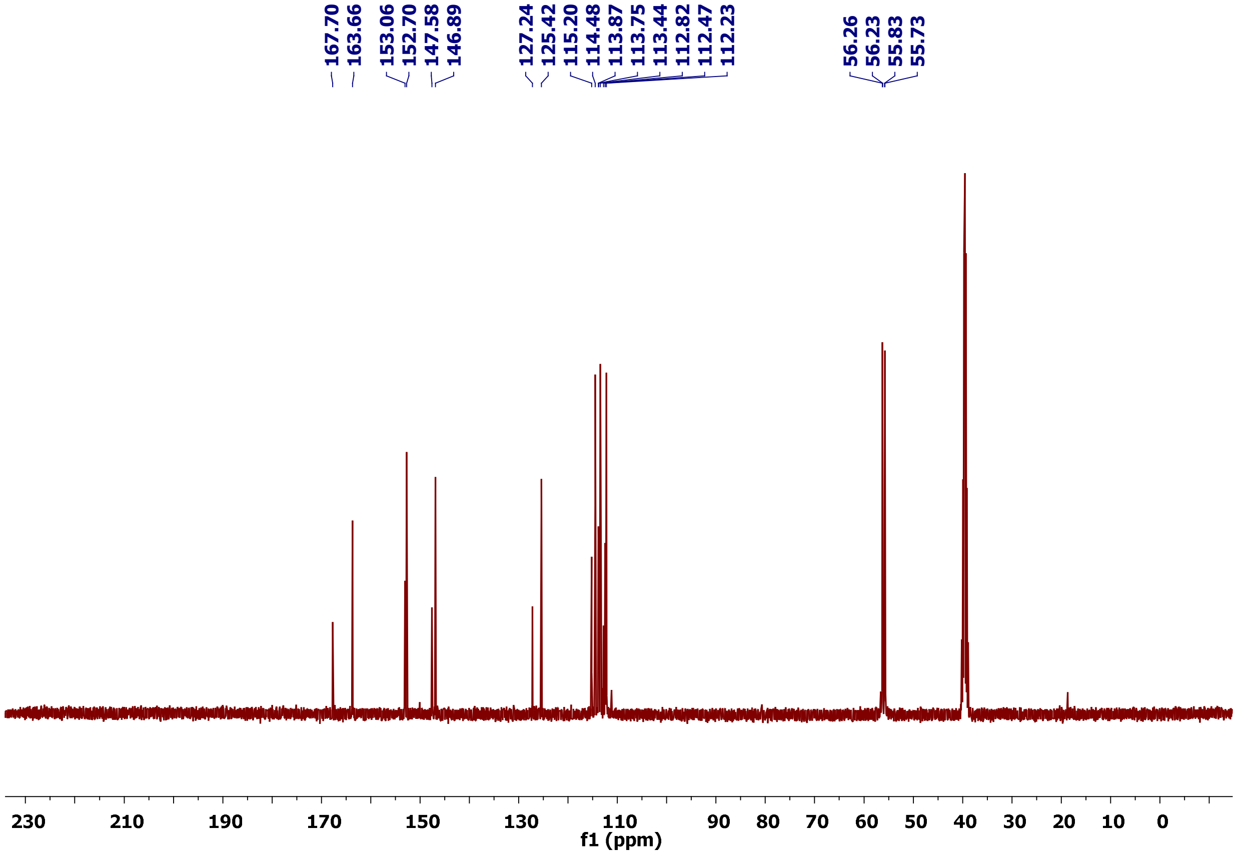

^{13}C CRAPT NMR (DMSO-d6) spectrum of (2,5-dimethoxyphenyl)carbamothioyl cyanide (1:0.52 tautomeric ratio) (1q')

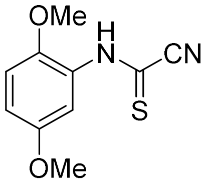

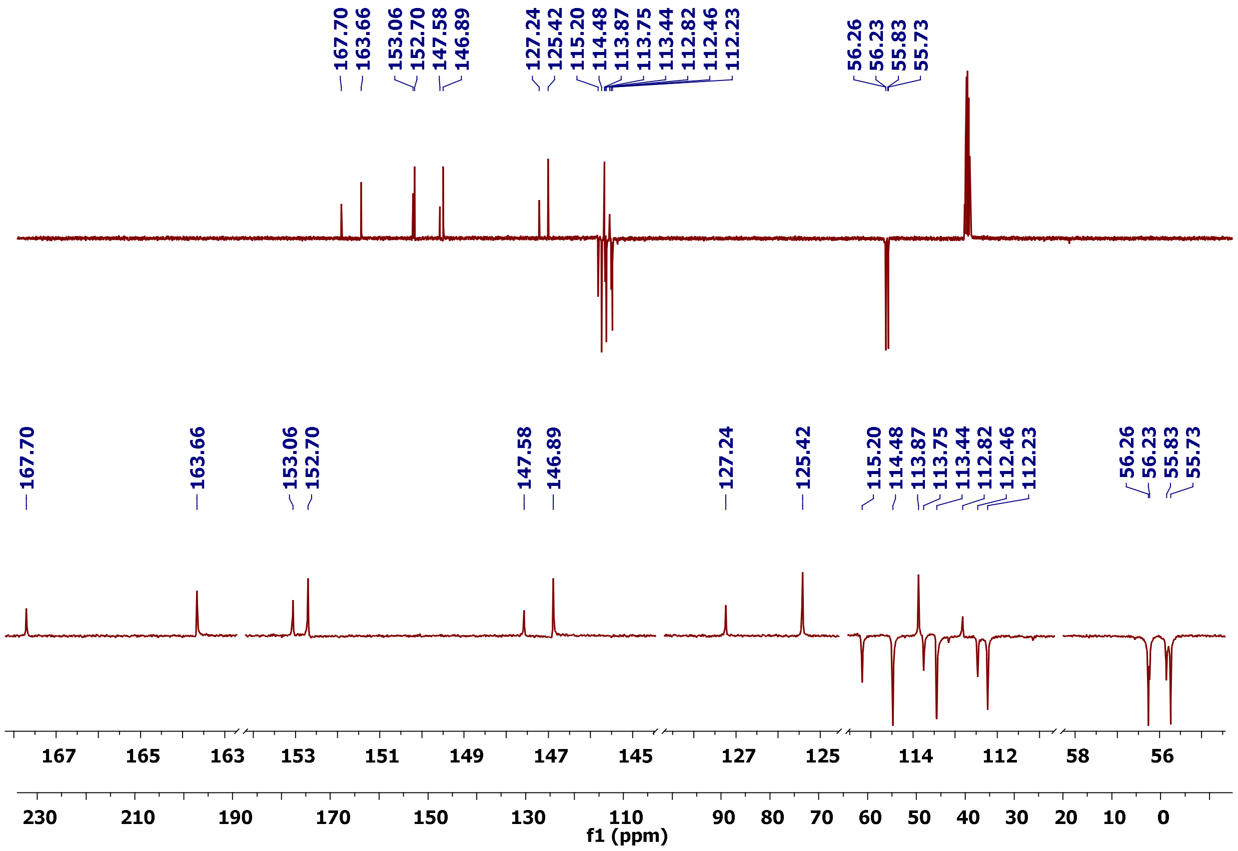

^{1}H-^{1}H-gDQCOSY NMR (DMSO-d6) spectrum of (2,5-dimethoxyphenyl)carbamothioyl cyanide (1:0.52 tautomeric ratio) (1q')

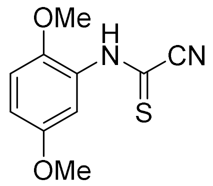

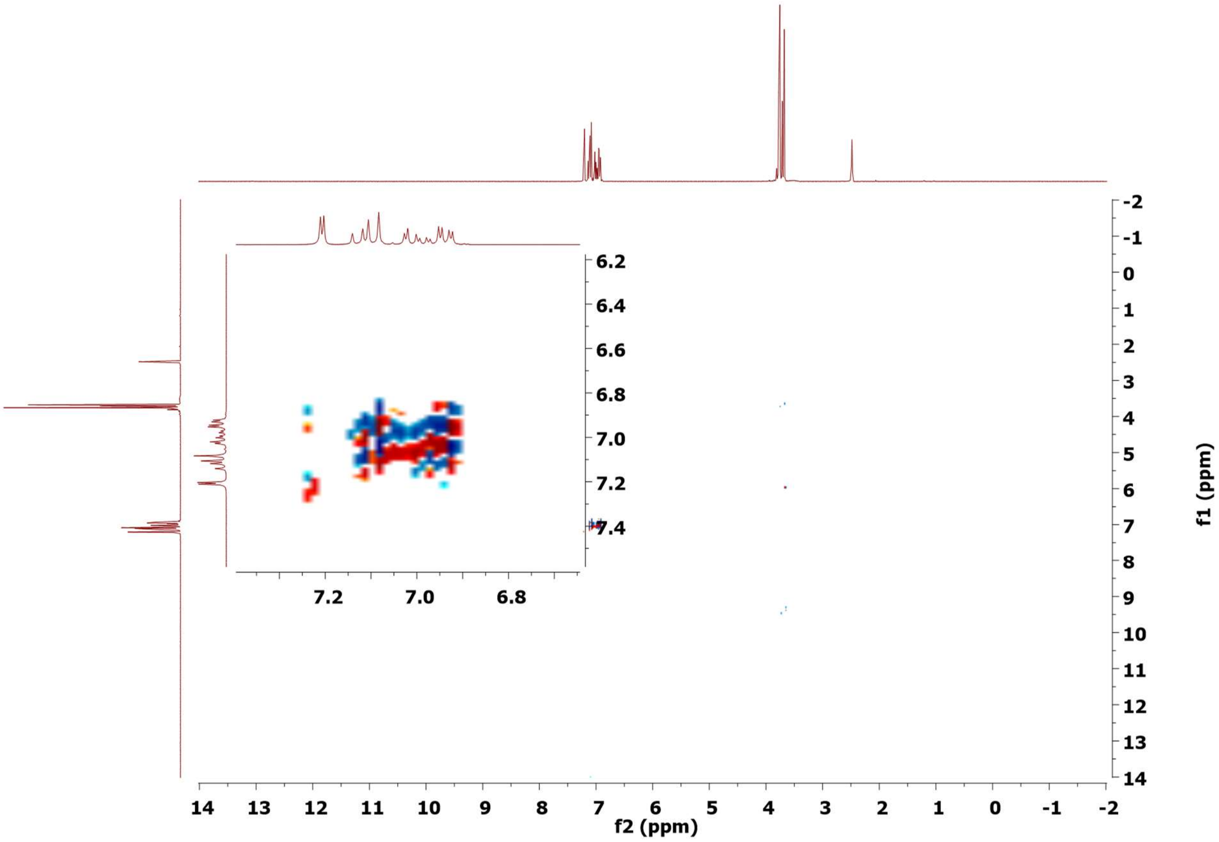

^{1}H-^{13}C-gHSQC NMR (DMSO-d6) spectrum of (2,5-dimethoxyphenyl)carbamothioyl cyanide (1:0.52 tautomeric ratio) (1q')

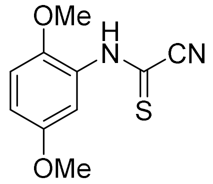

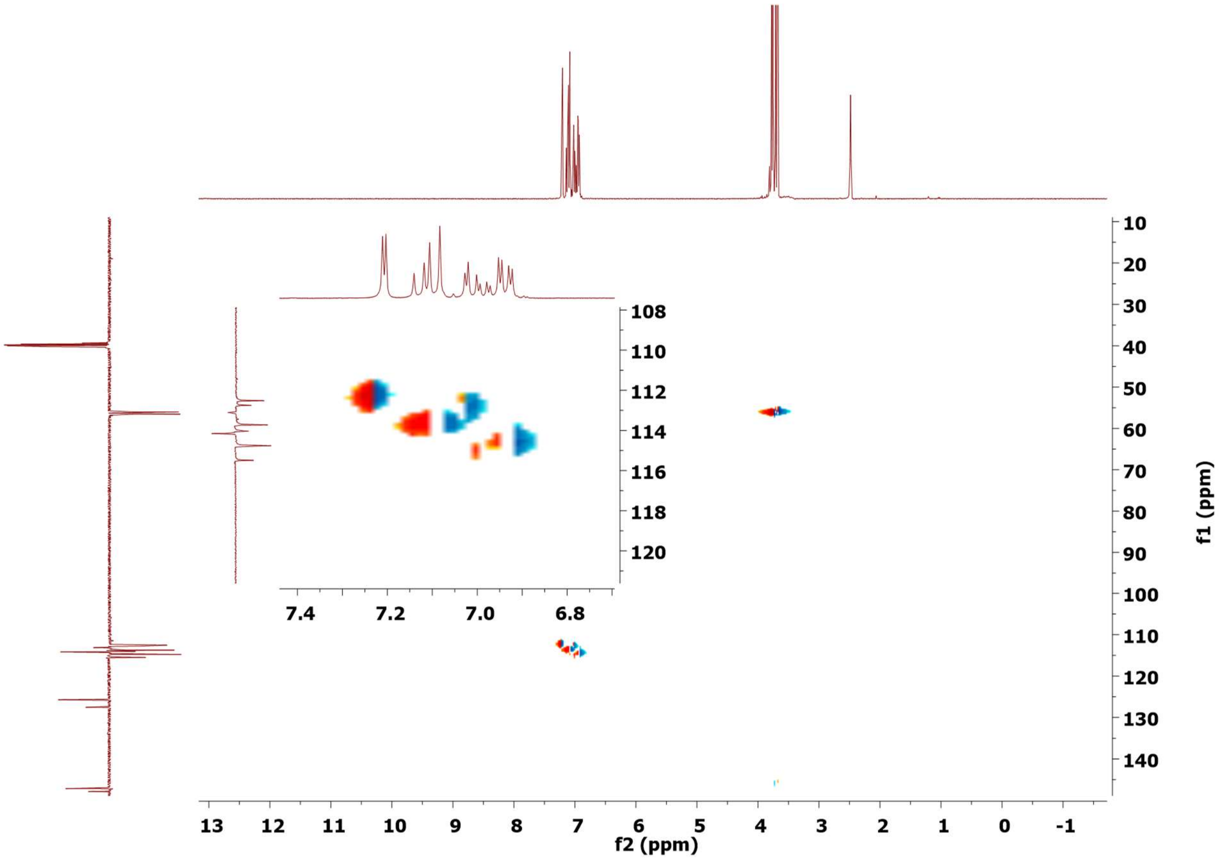

^{1}H-^{13}C-gHMBC NMR (DMSO-d6) spectrum of (2,5-dimethoxyphenyl)carbamothioyl cyanide (1:0.52 tautomeric ratio) (1q')

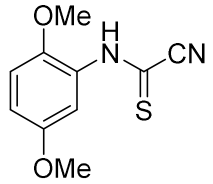

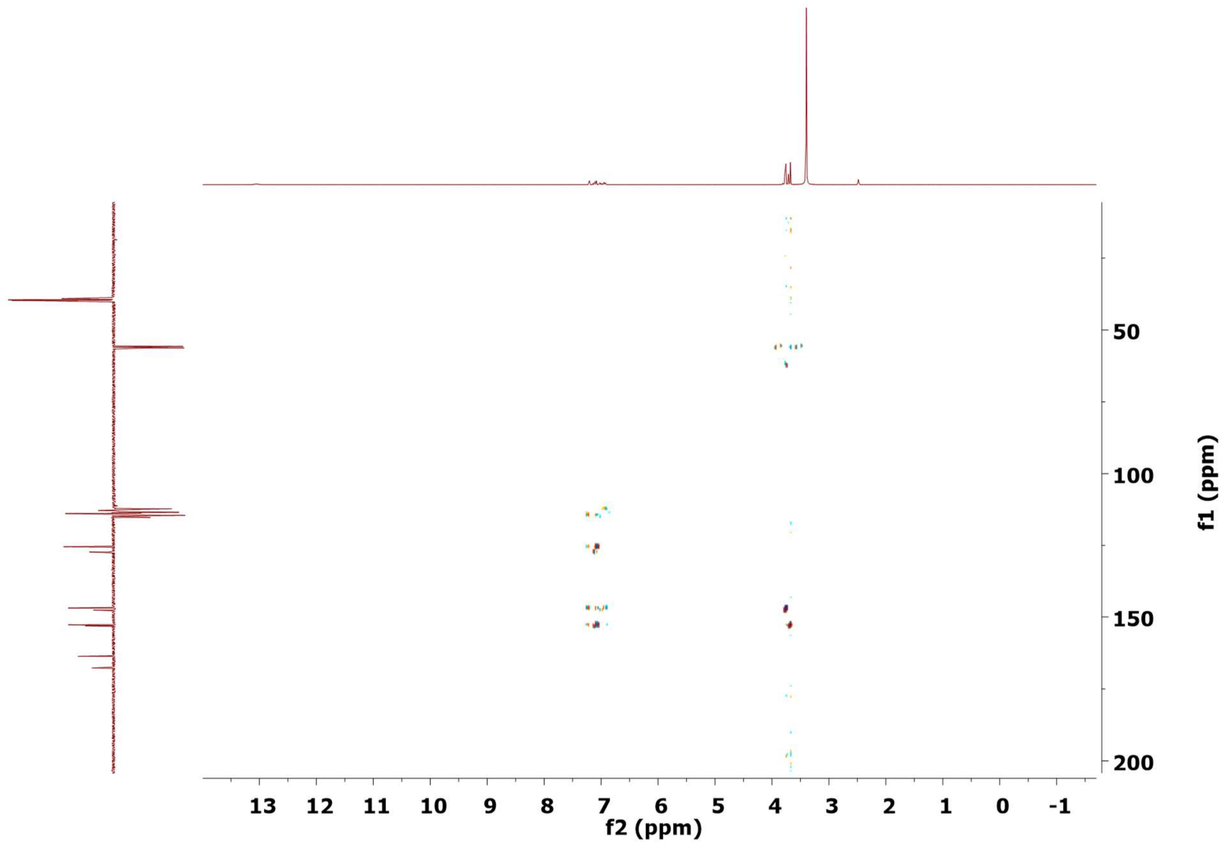

^{1}H NMR (DMSO-d6) spectrum of (2,4-dimethoxyphenyl)carbamothioyl cyanide (1:0.52 tautomeric ratio) (1r')

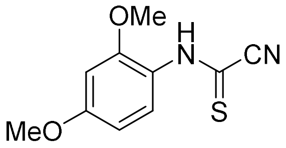

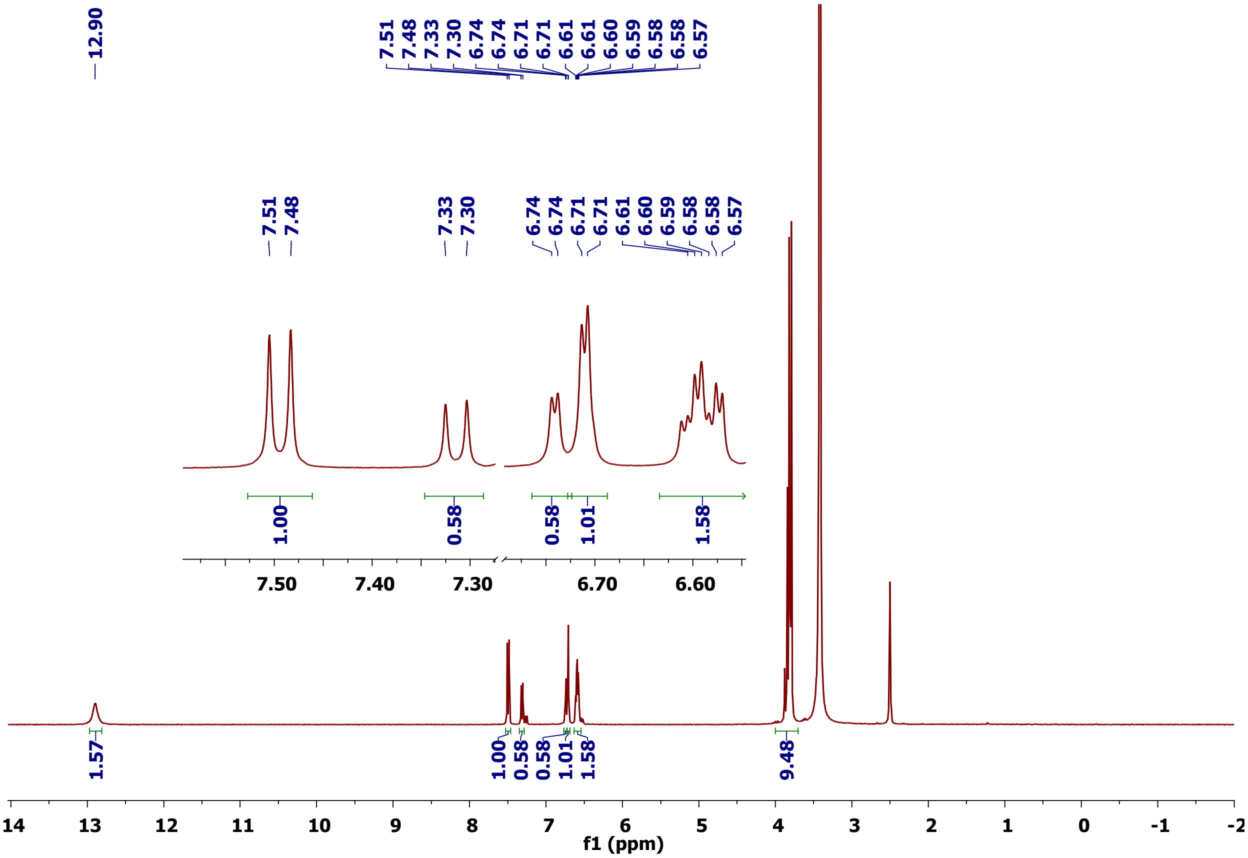

^{13}C NMR (DMSO-d6) spectrum of (2,4-dimethoxyphenyl)carbamothioyl cyanide (1:0.52 tautomeric ratio) (1r')

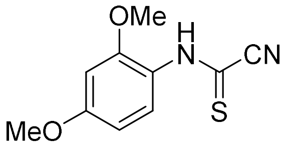

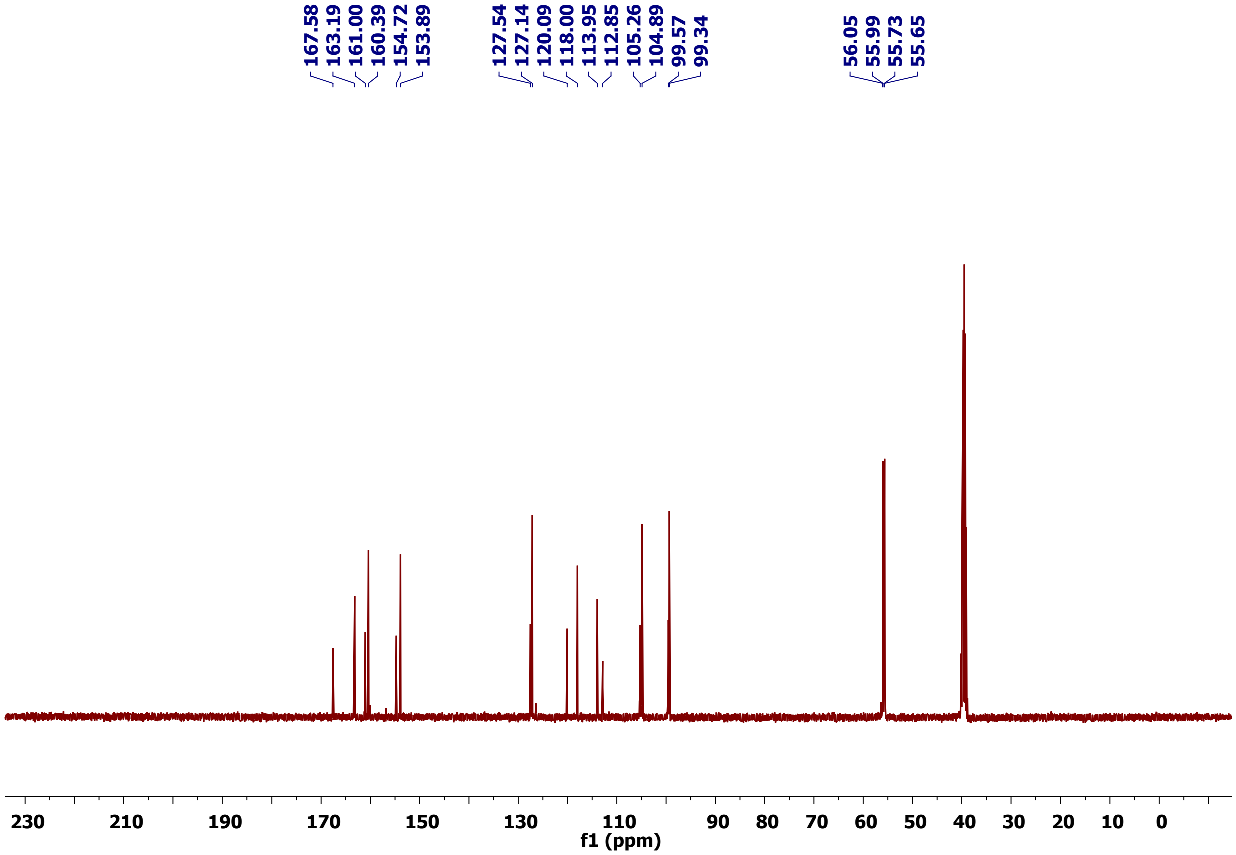

^{13}C CRAPT NMR (DMSO-d6) spectrum of (2,4-dimethoxyphenyl)carbamothioyl cyanide (1:0.52 tautomeric ratio) (1r')

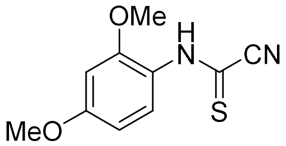

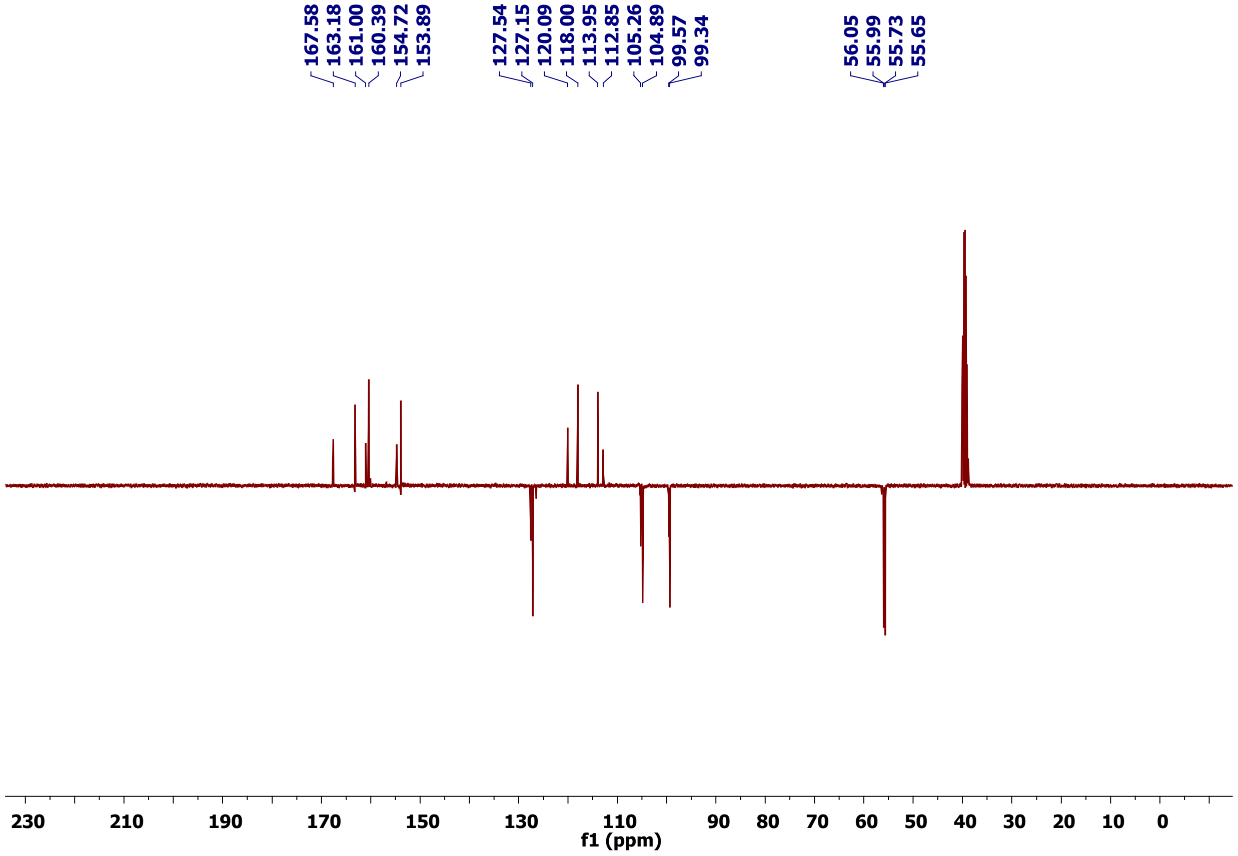

^{1}H-^{1}H-gDQCOSY NMR (DMSO-d6) spectrum of (2,4-dimethoxyphenyl)carbamothioyl cyanide (1:0.52 tautomeric ratio) (1r')

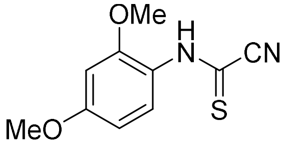

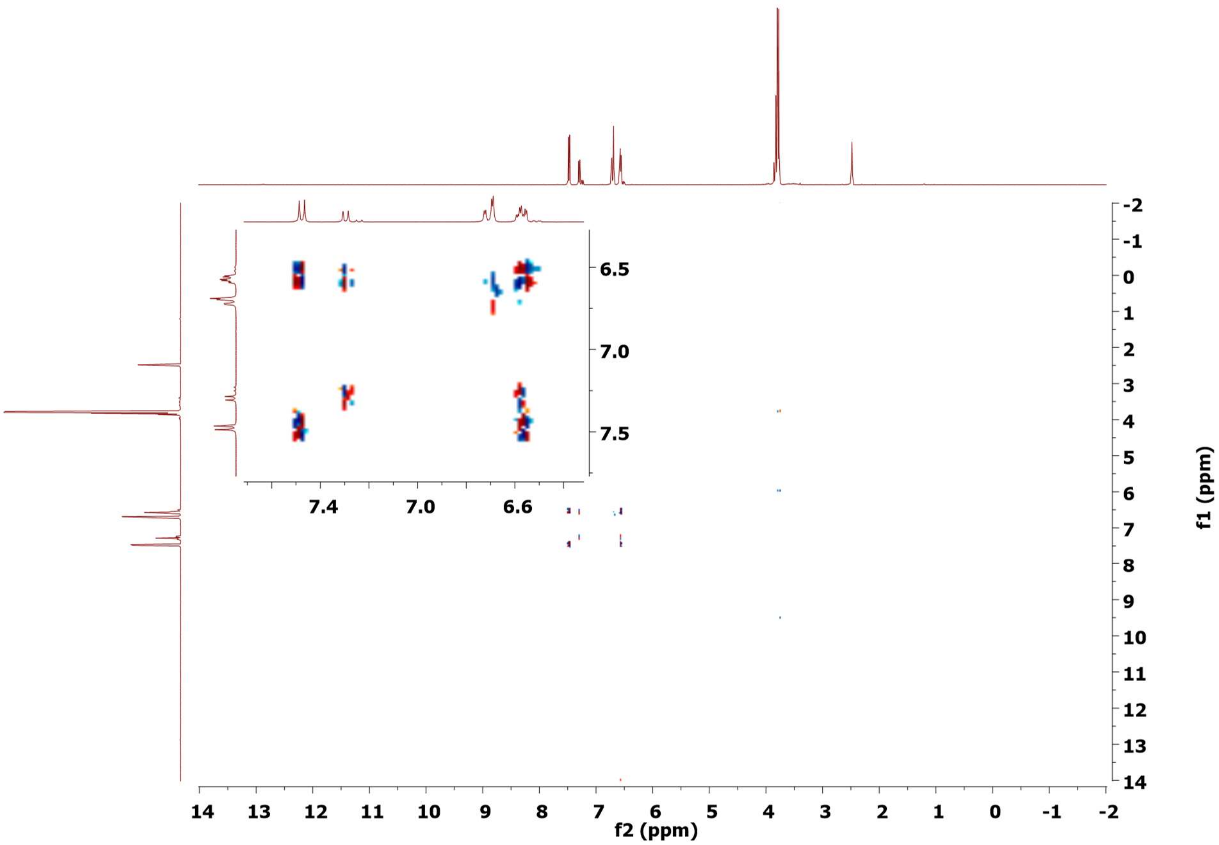

^{1}H-^{13}C-gHSQC NMR (DMSO-d6) spectrum of (2,4-dimethoxyphenyl)carbamothioyl cyanide (1:0.52 tautomeric ratio) (1r')

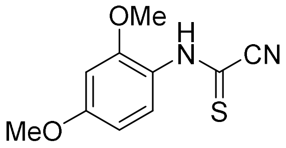

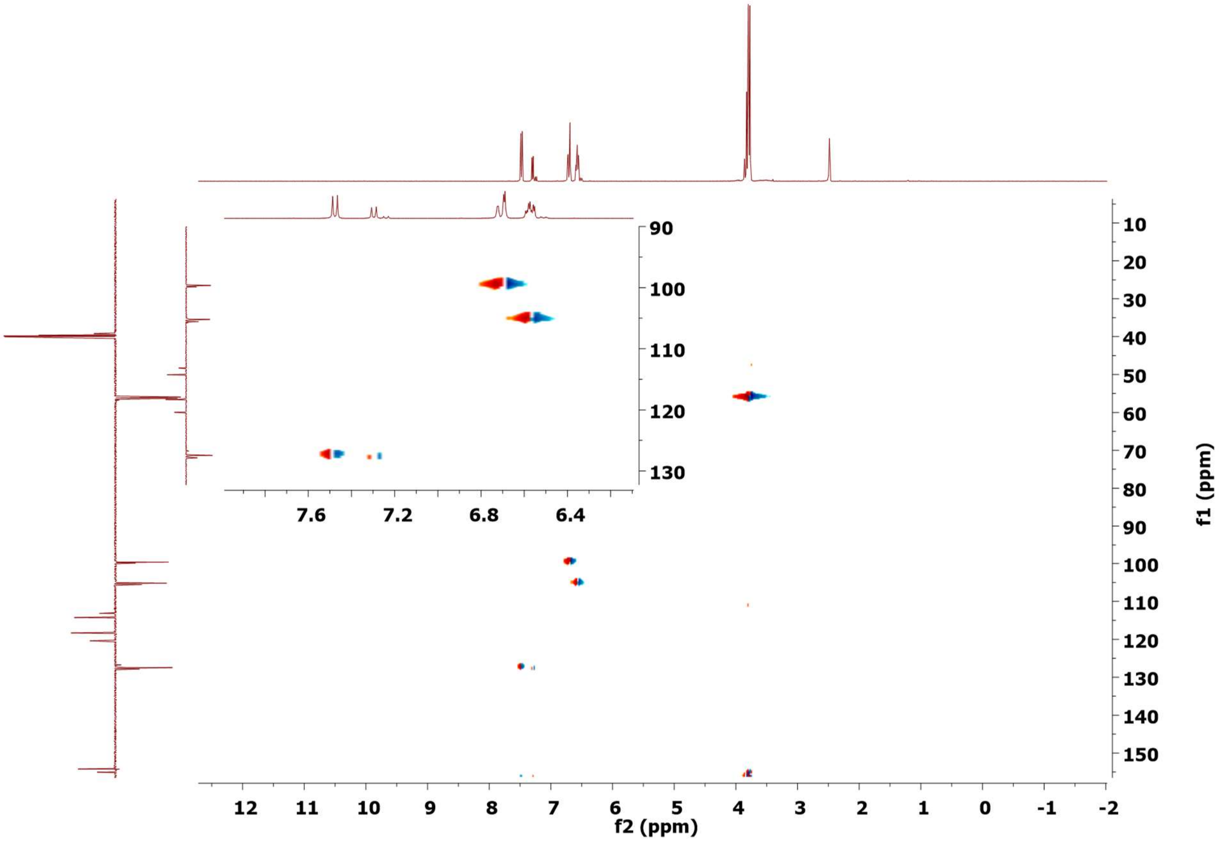

^{1}H-^{13}C-gHMBC NMR (DMSO-d6) spectrum of (2,4-dimethoxyphenyl)carbamothioyl cyanide (1:0.52 tautomeric ratio) (1r')

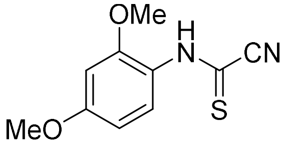

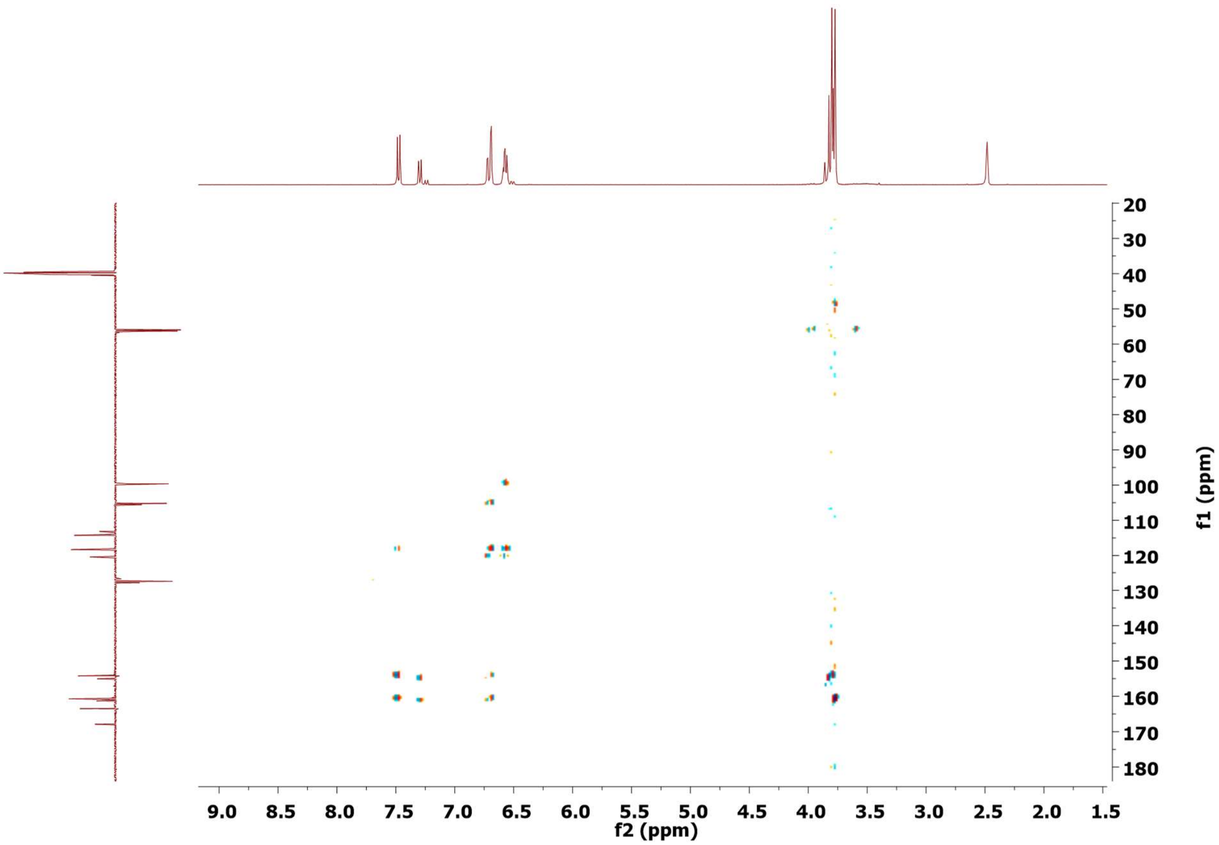

^{1}H NMR (DMSO-d6) spectrum of (3-acetylphenyl)carbamothioyl cyanide (1s')

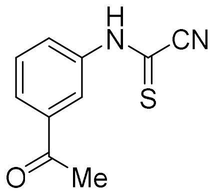

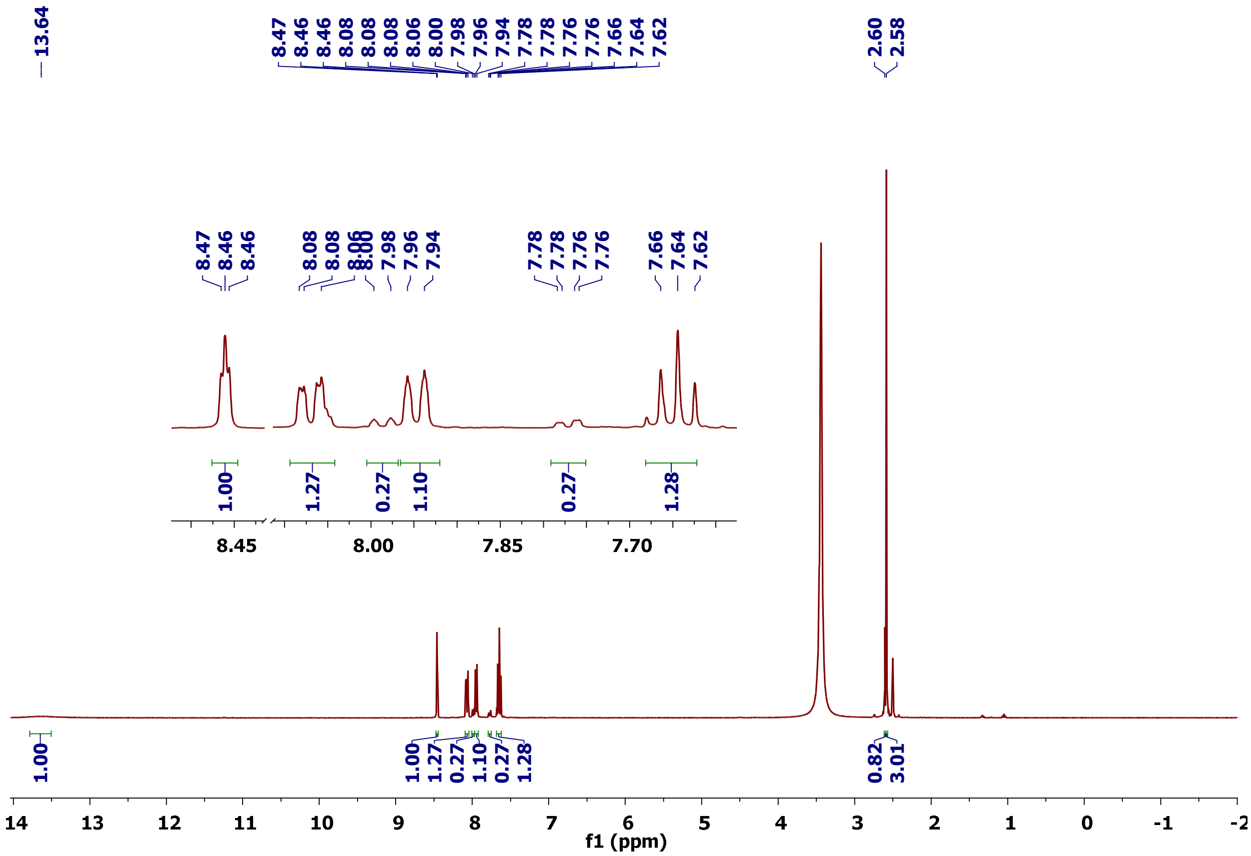

^{13}C NMR (DMSO-d6) spectrum of (3-acetylphenyl)carbamothioyl cyanide (1s’)

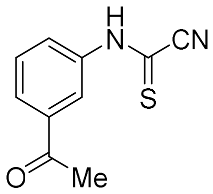

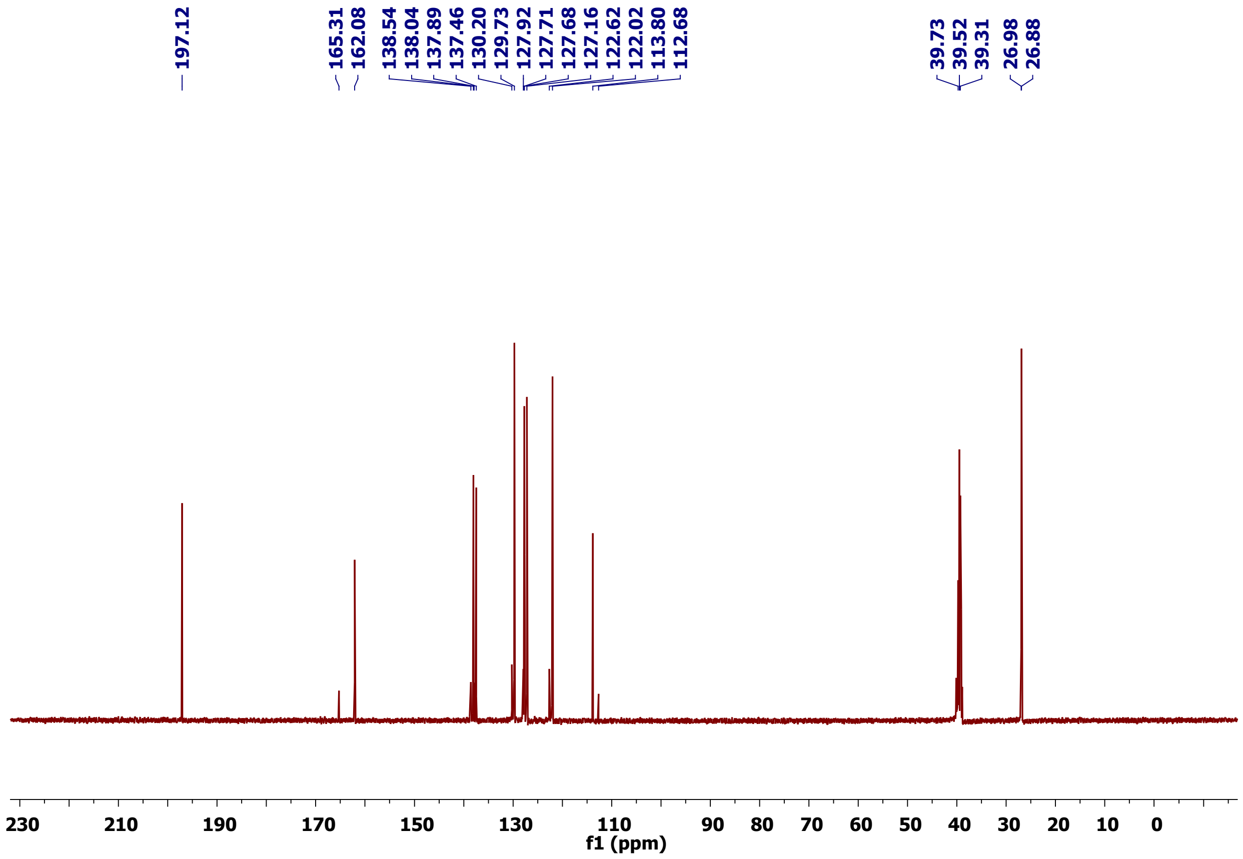

^{13}C CRAPT NMR (DMSO-d6) spectrum of (3-acetylphenyl)carbamothioyl cyanide (1s')

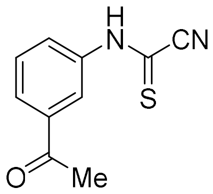

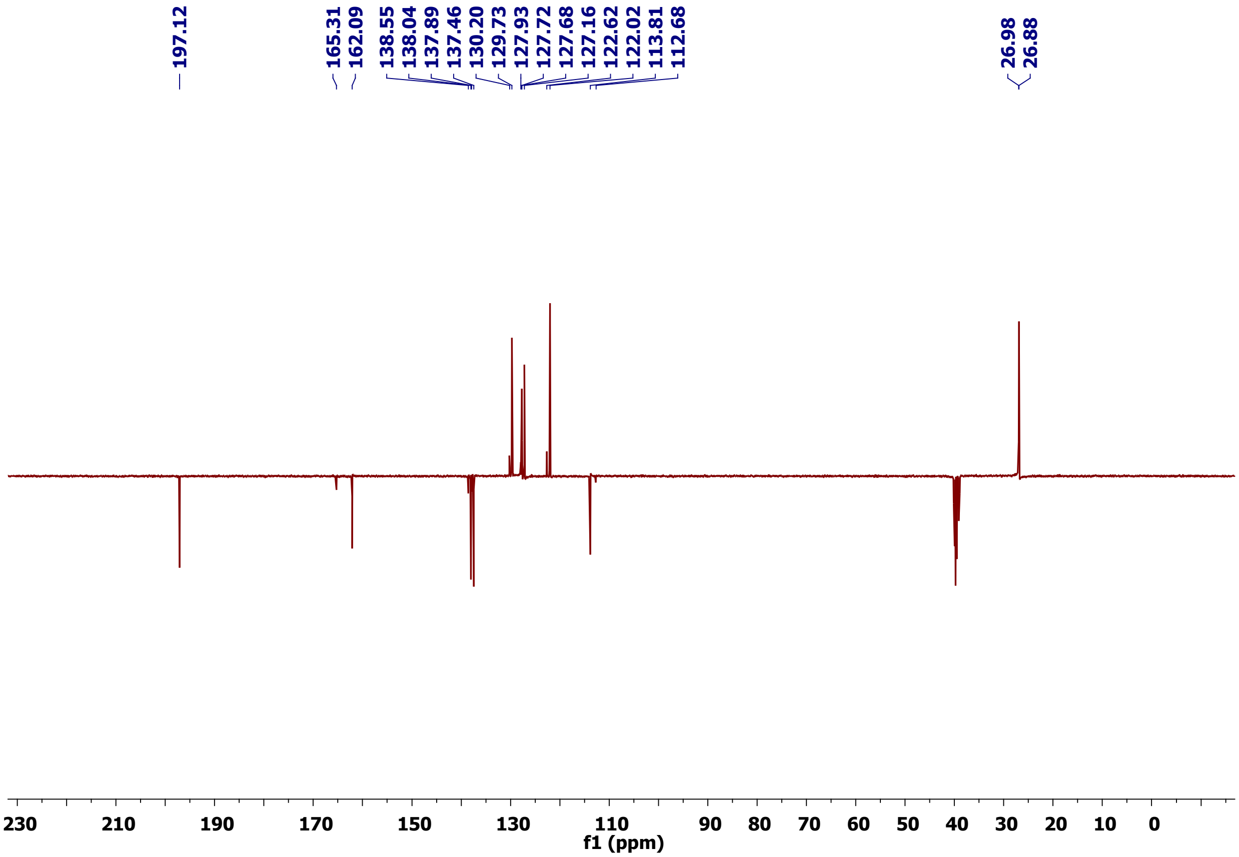

^{1}H-^{1}H gDQCOSY NMR (DMSO-d6) spectrum of (3-acetylphenyl)carbamothioyl cyanide (1s')

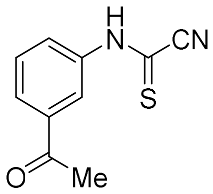

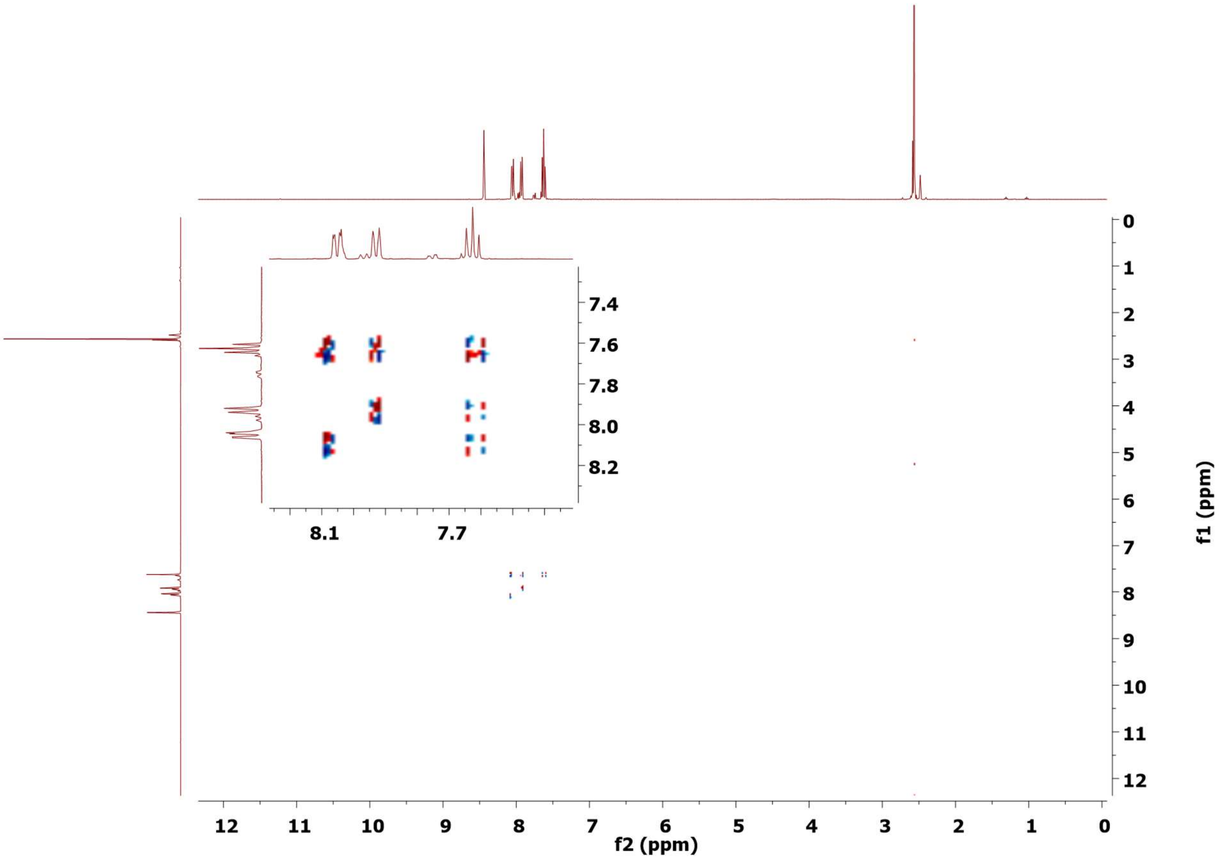

^{1}H-^{13}C-gHSQC NMR (DMSO-d6) spectrum of (3-acetylphenyl)carbamothioyl cyanide (1s')

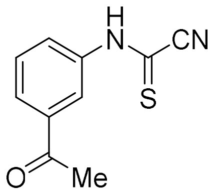

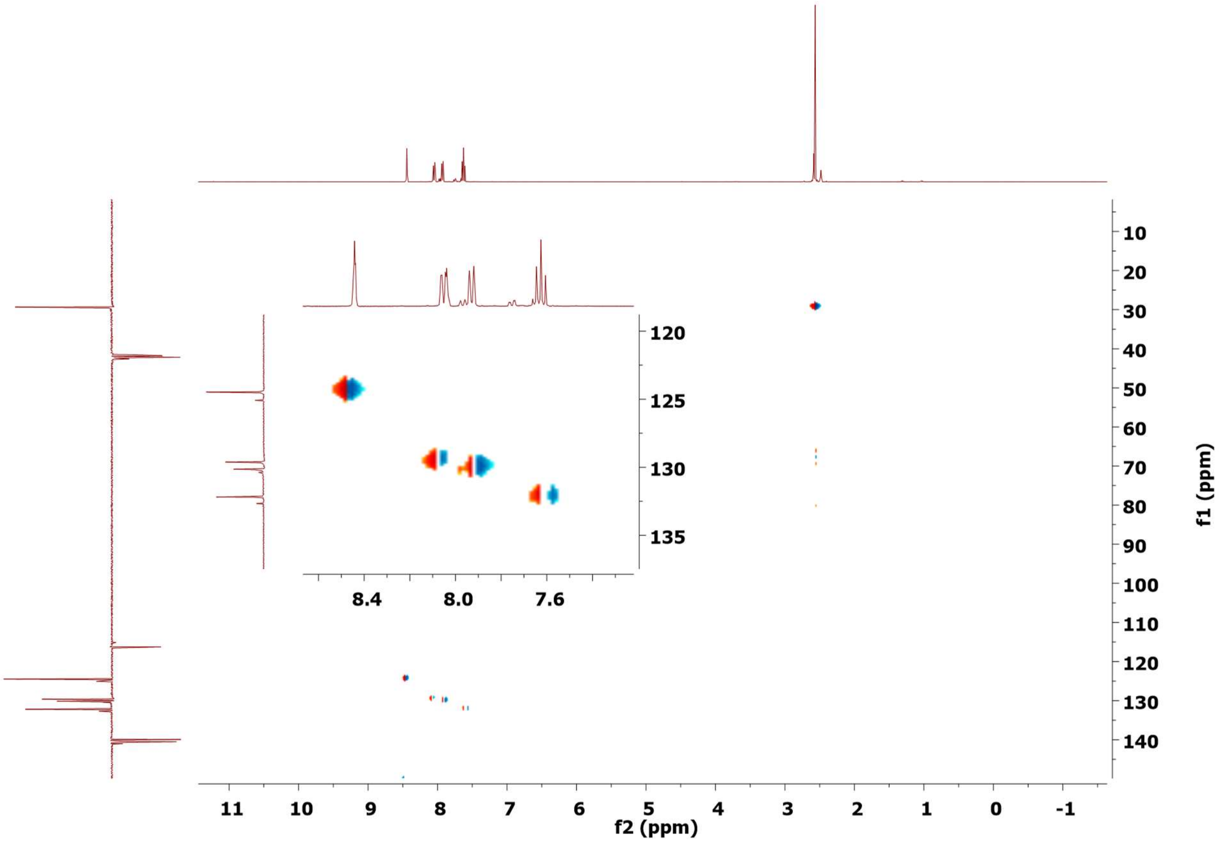

^{1}H-^{13}C-gHMBC NMR (DMSO-d6) spectrum of (3-acetylphenyl)carbamothioyl cyanide (1s')

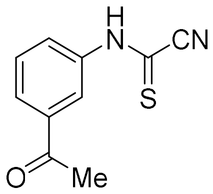

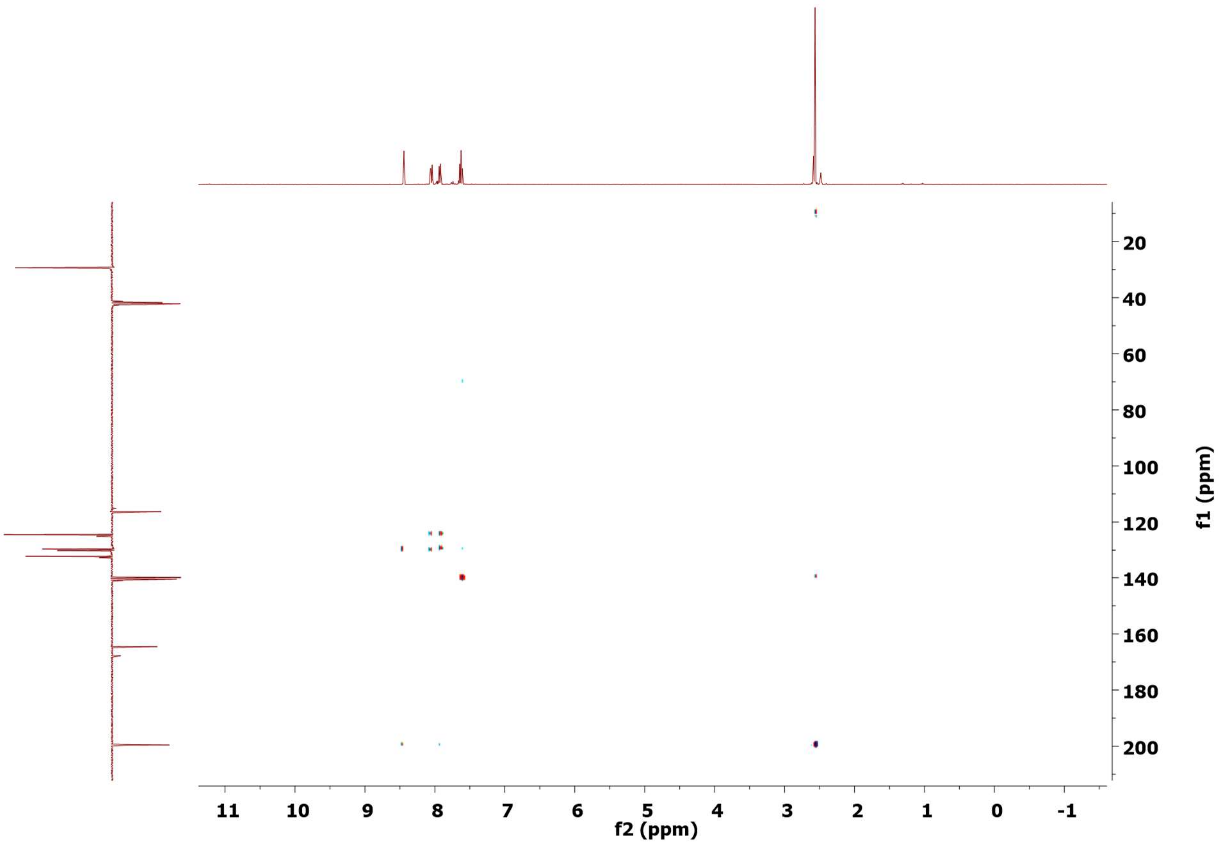

^{1}H NMR (DMSO-d6) spectrum of (2-(methylthio)phenyl)carbamothioyl cyanide (1t')

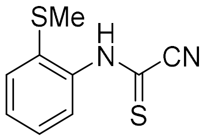

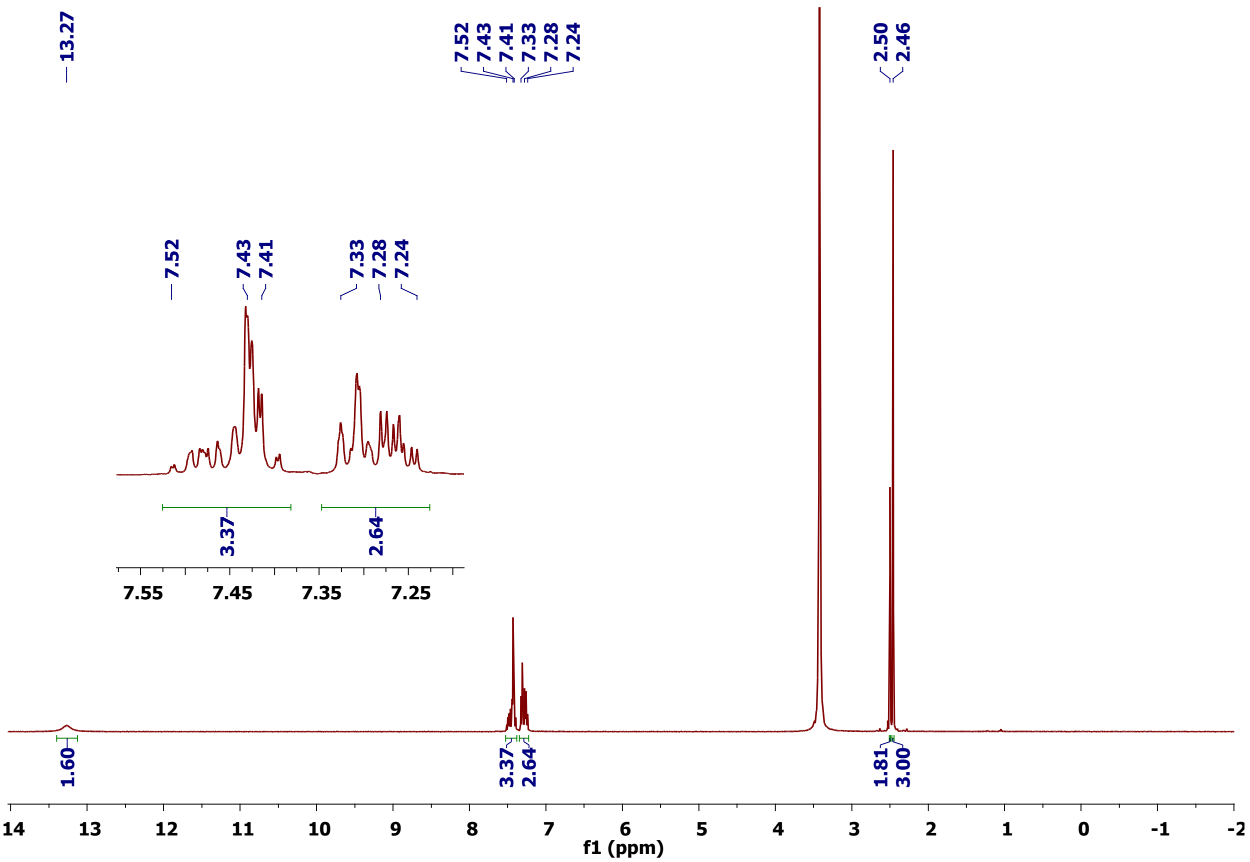

^{13}C NMR (DMSO-d6) spectrum of (2-(methylthio)phenyl)carbamothioyl cyanide (1t')

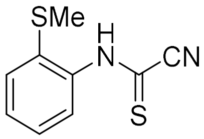

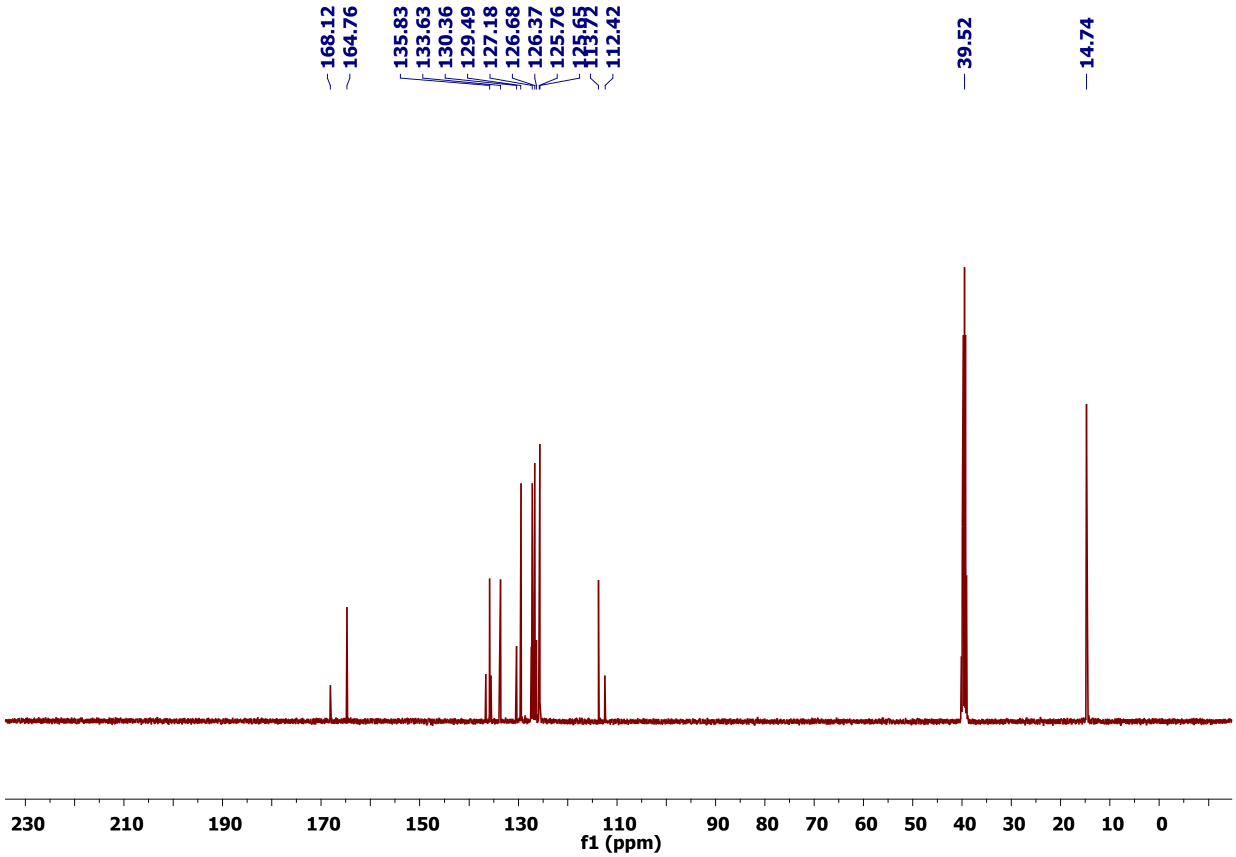

^{13}C CRAPT NMR (DMSO-d6) spectrum of (2-(methylthio)phenyl)carbamothioyl cyanide (1t')

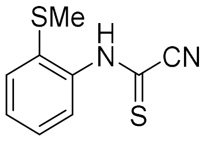

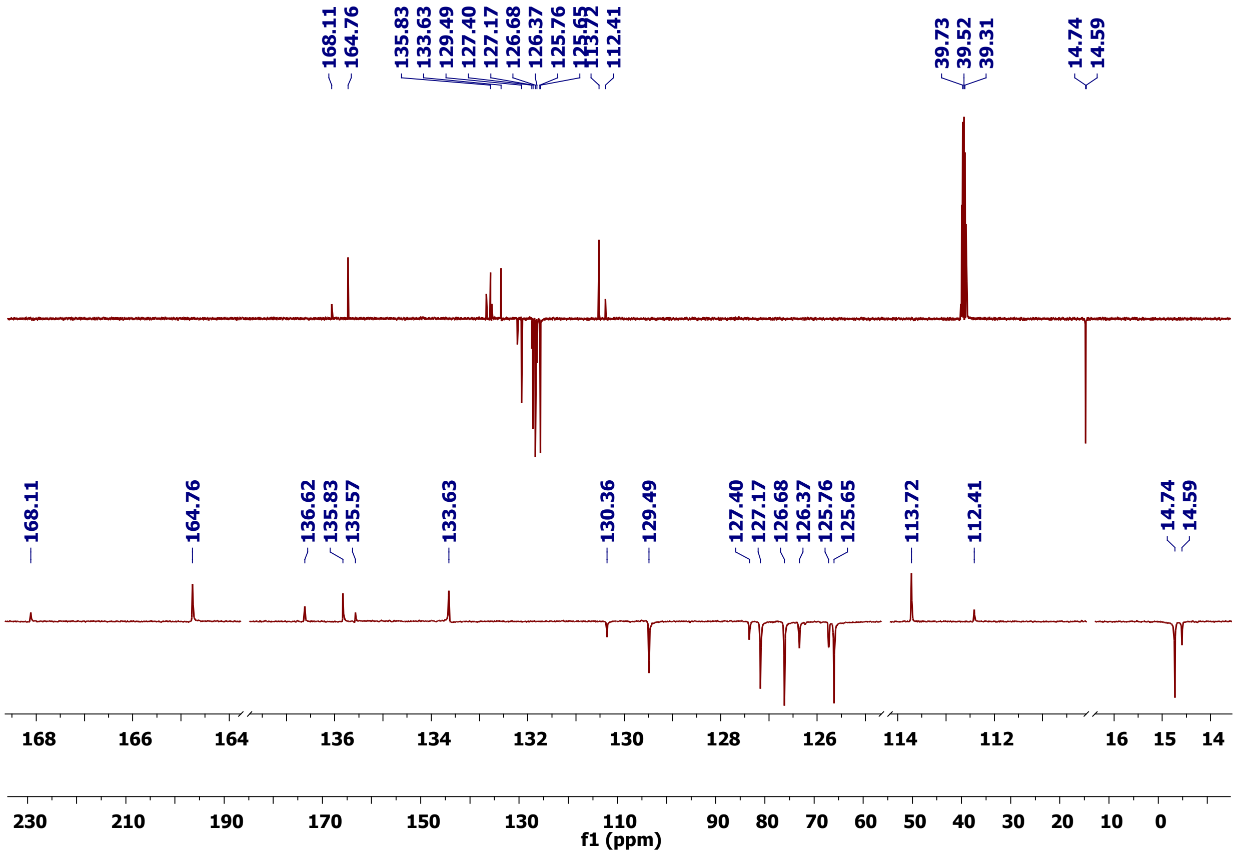

^{1}H-^{1}H gDQCOSY NMR (DMSO-d6) spectrum of (2-(methylthio)phenyl)carbamothioyl cyanide (1t')

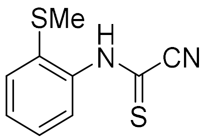

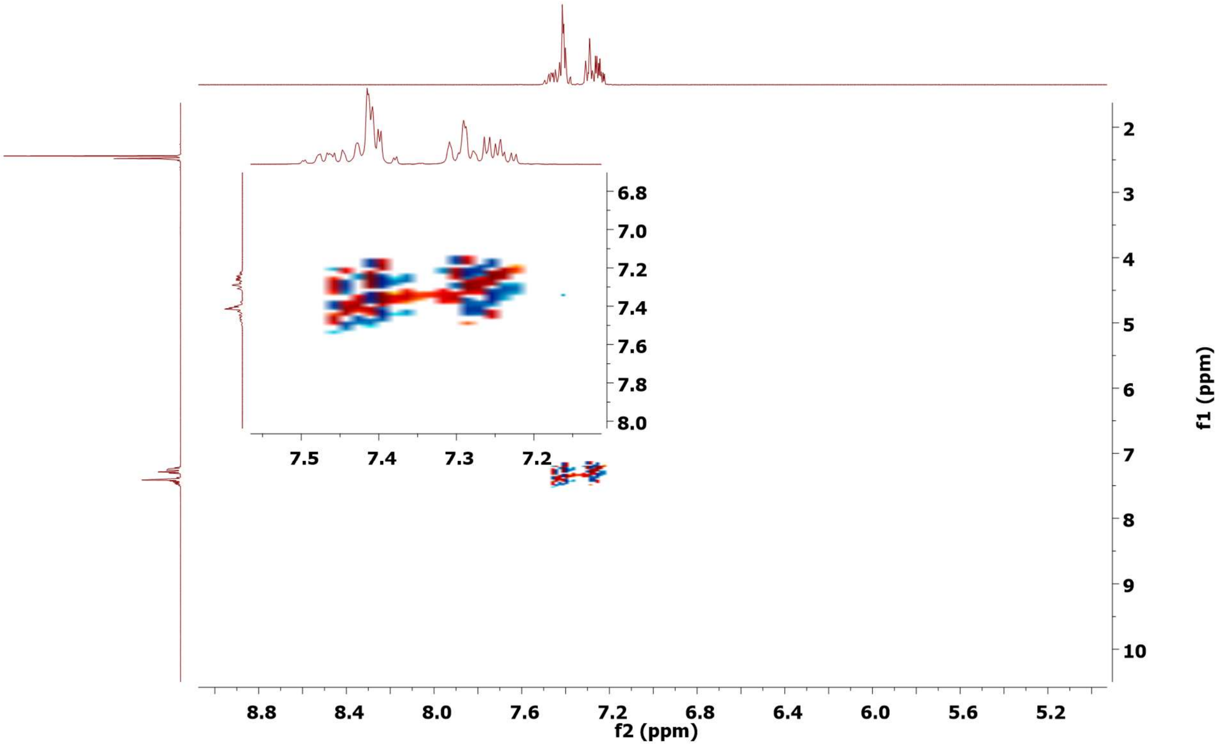

^{1}H-^{13}C-gHSQC NMR (DMSO-d6) spectrum of (2-(methylthio)phenyl)carbamothioyl cyanide (1t')

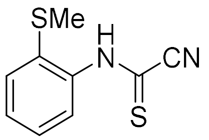

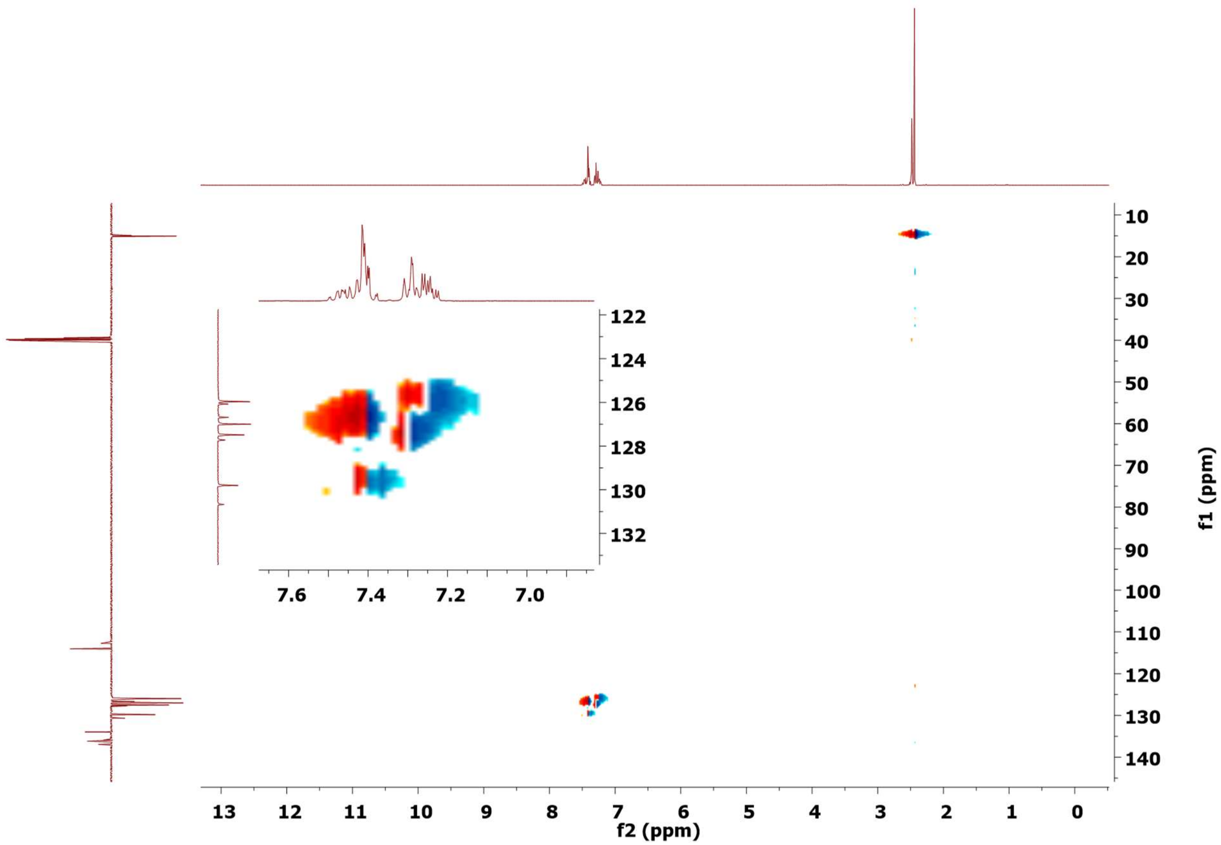

^{1}H-^{13}C-gHMBC NMR (DMSO-d6) spectrum of (2-(methylthio)phenyl)carbamothioyl cyanide (1t')

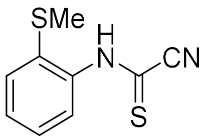

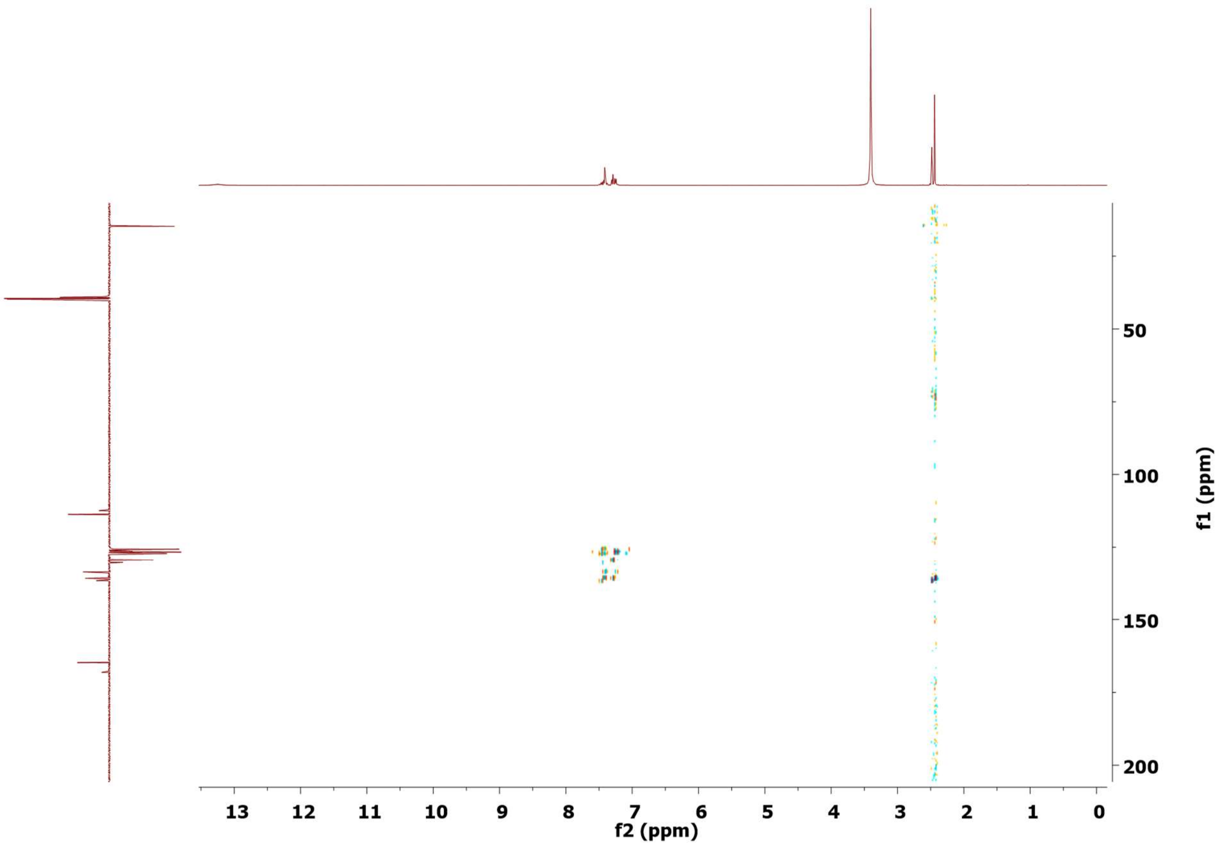

^{1}H NMR (DMSO-d6) spectrum of the oxidation product of (2-(methylthio)phenyl)carbamothioyl cyanide with iodine-dmso

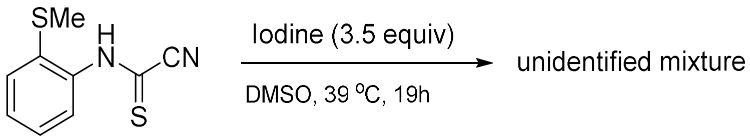

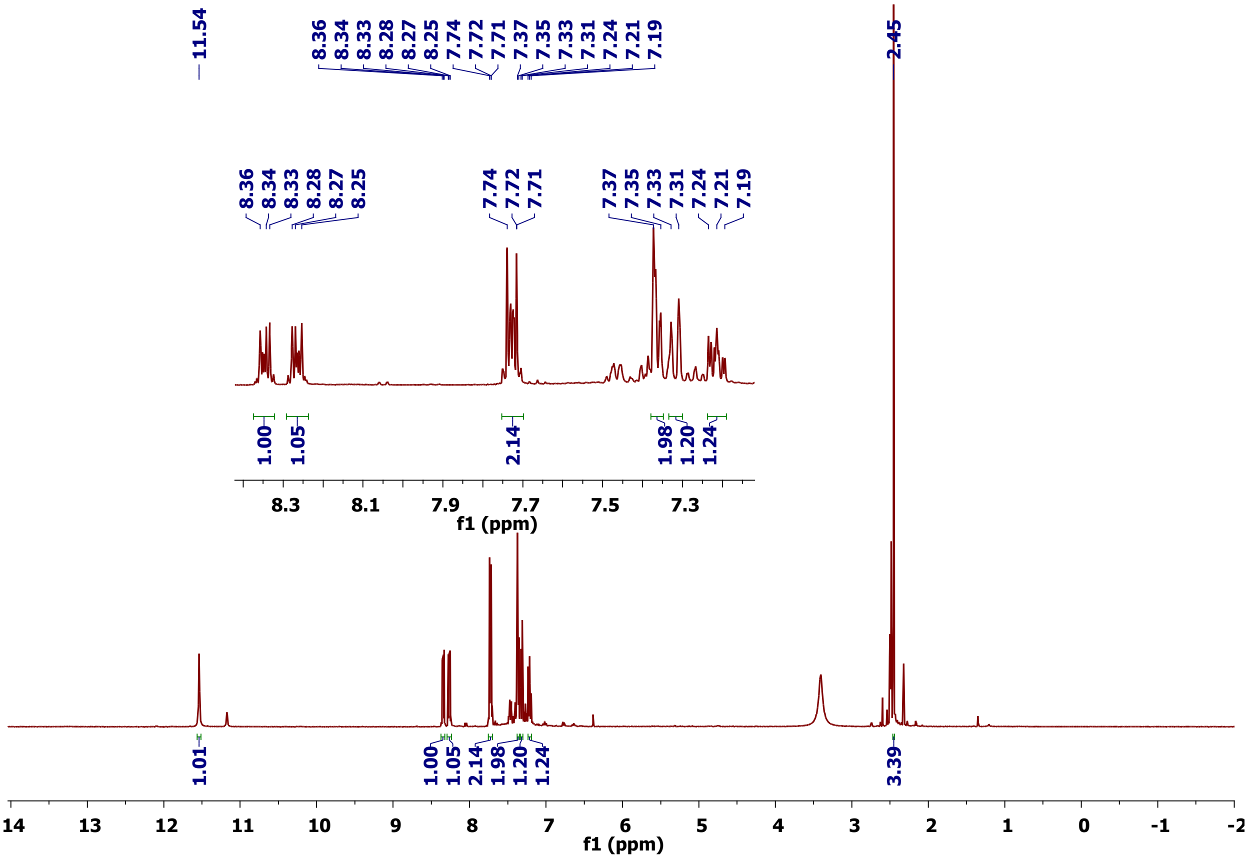

^{13}C NMR (DMSO-d6) spectrum of the oxidation product of (2-(methylthio)phenyl)carbamothioyl cyanide with iodine-dmso

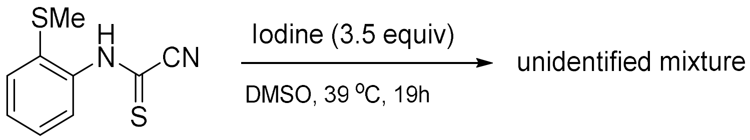

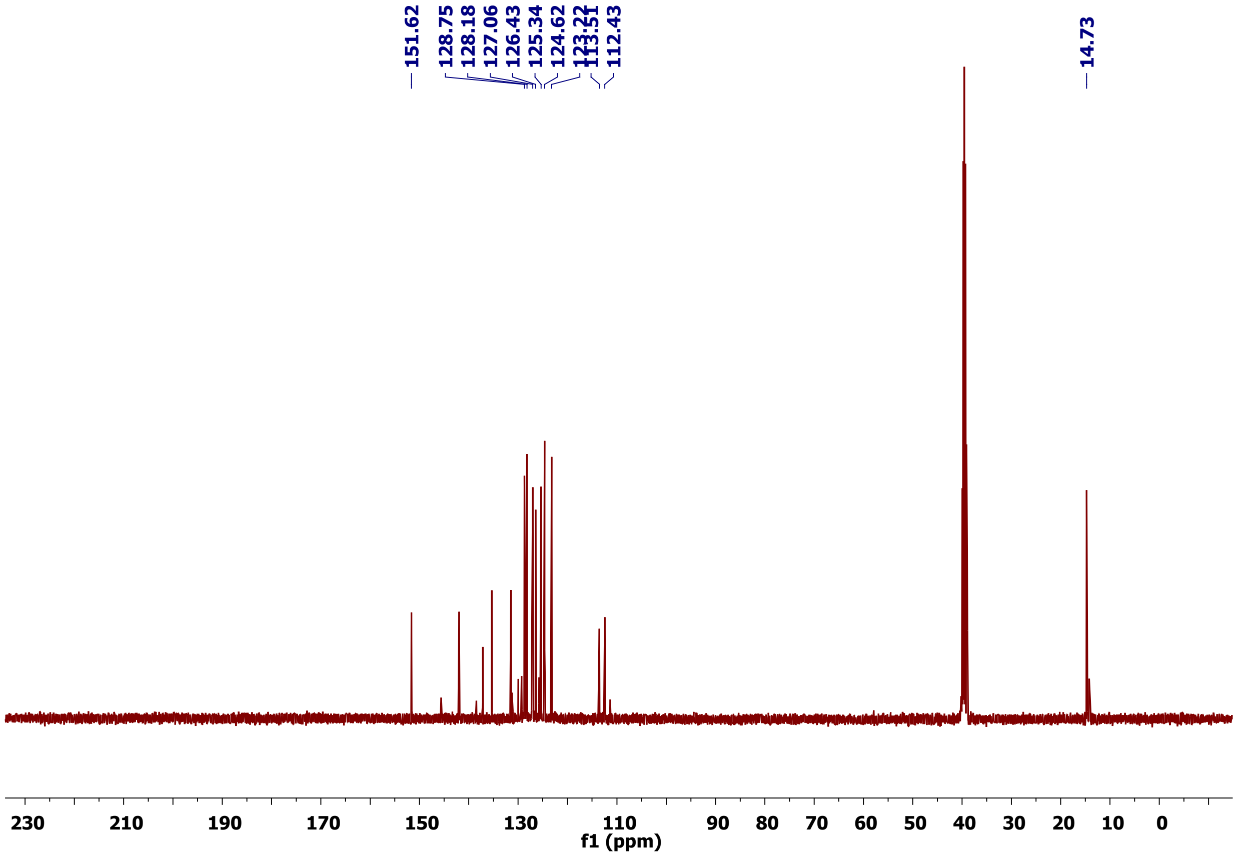

^{13}C CRAPT NMR (DMSO-d6) spectrum of the oxidation product of (2-(methylthio)phenyl)carbamothioyl cyanide with iodine-dmso

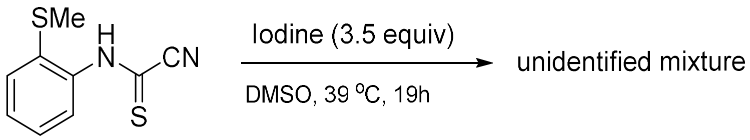

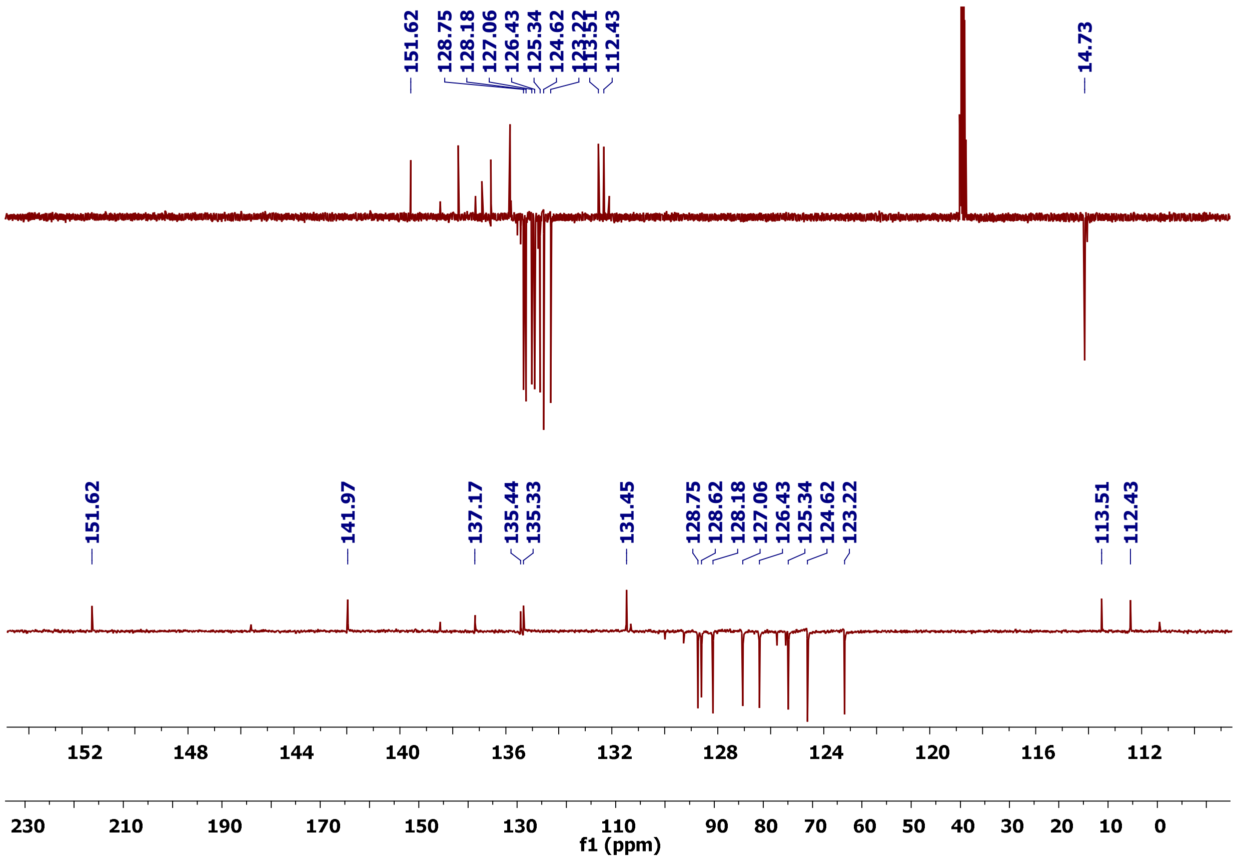

^{1}H-^{1}H gDQCOSY NMR (DMSO-d6) spectrum of the oxidation product of (2-(methylthio)phenyl)carbamothioyl cyanide with iodine-dmso

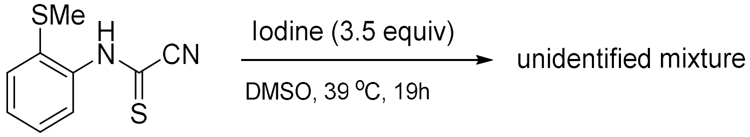

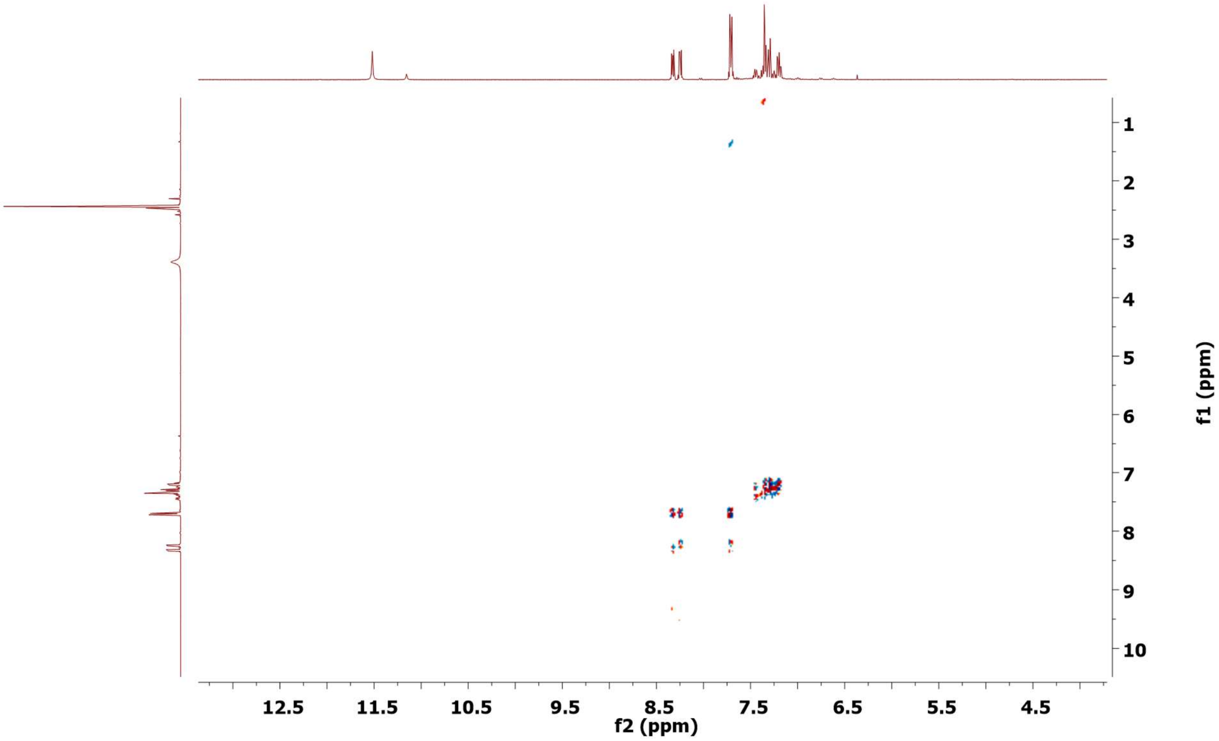

^{1}H-^{13}C-gHSQC NMR (DMSO-d6) spectrum of the oxidation product of (2-(methylthio)phenyl)carbamothioyl cyanide with iodine-dmso

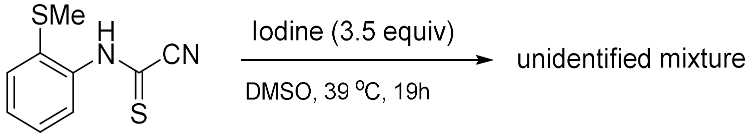

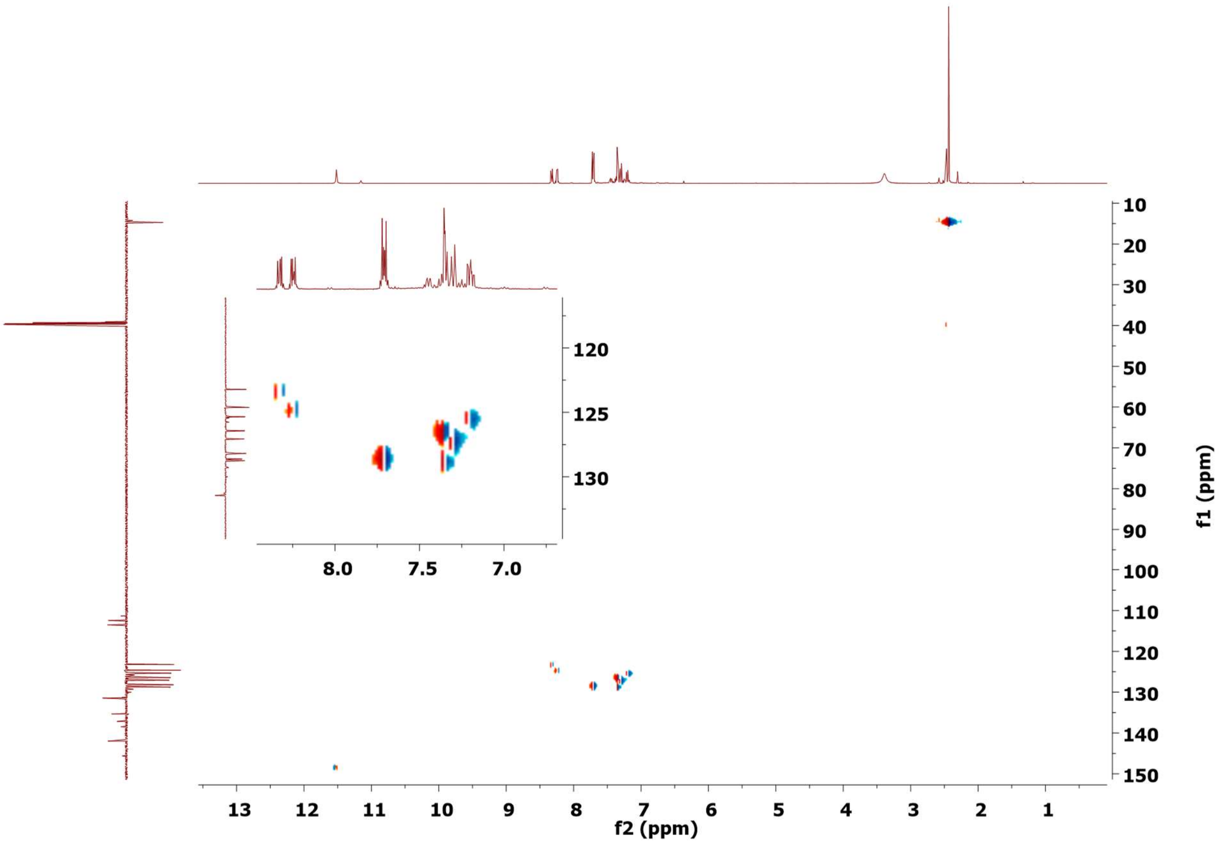

^{1}H-^{13}C-gHMBC NMR (DMSO-d6) spectrum of the oxidation product of (2-(methylthio)phenyl)carbamothioyl cyanide with iodine-dmso

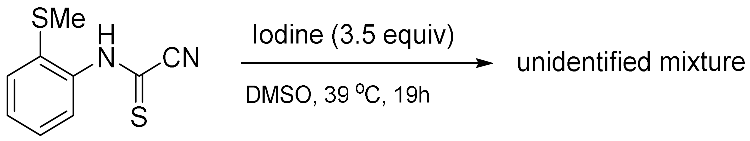

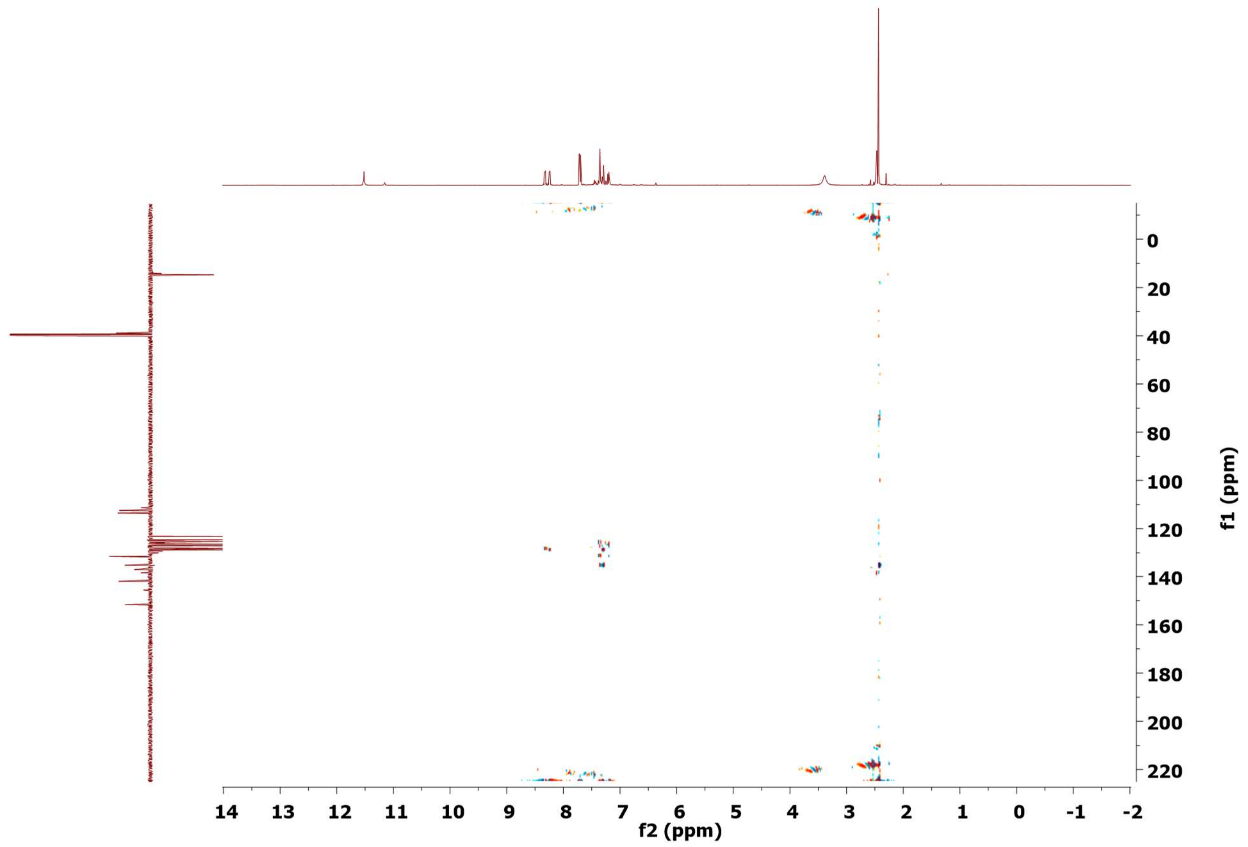

^{1}H NMR (DMSO-d6) spectrum of p-tolylcarbamoyl cyanide (2a)

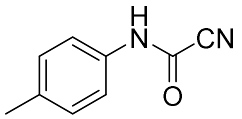

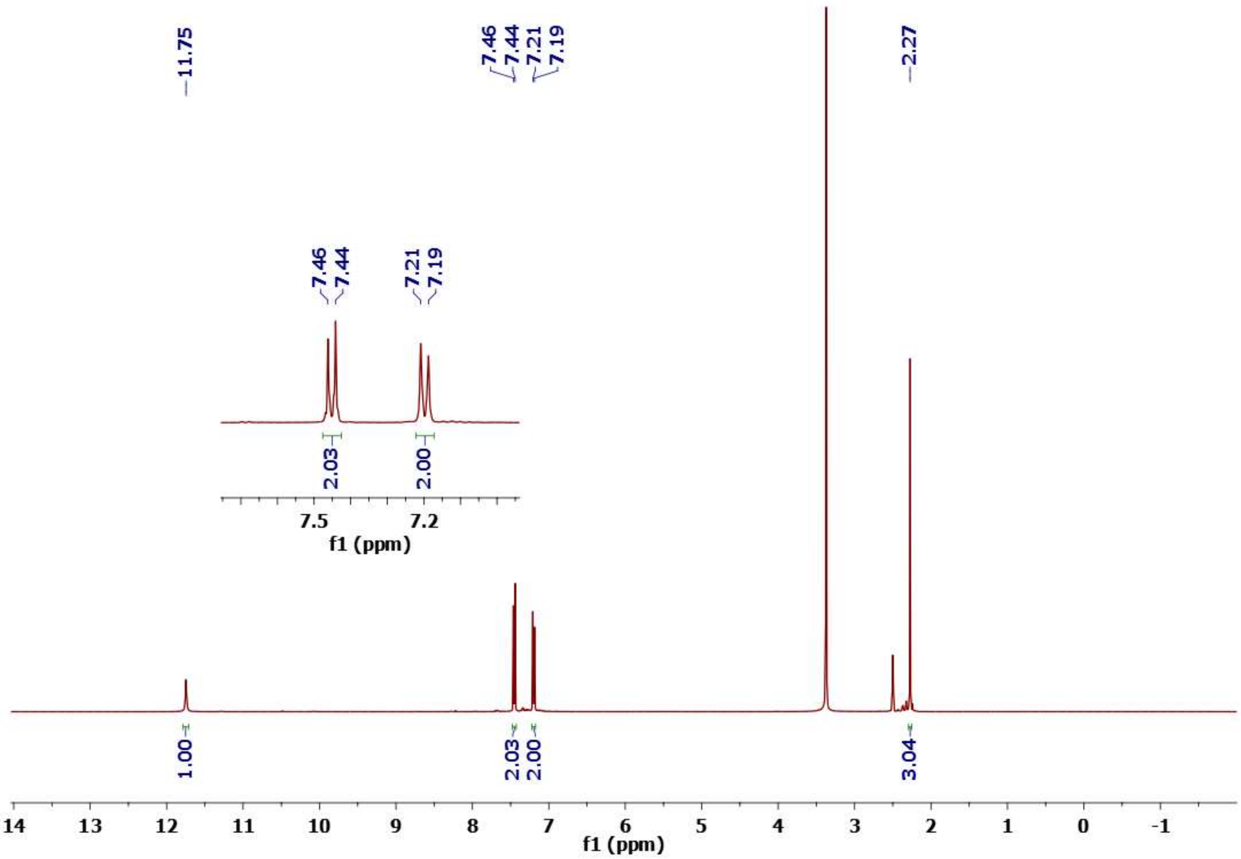

^{13}C NMR (DMSO-d6) spectrum of p-tolylcarbamoyl cyanide (2a)

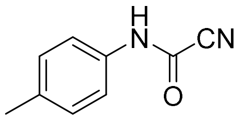

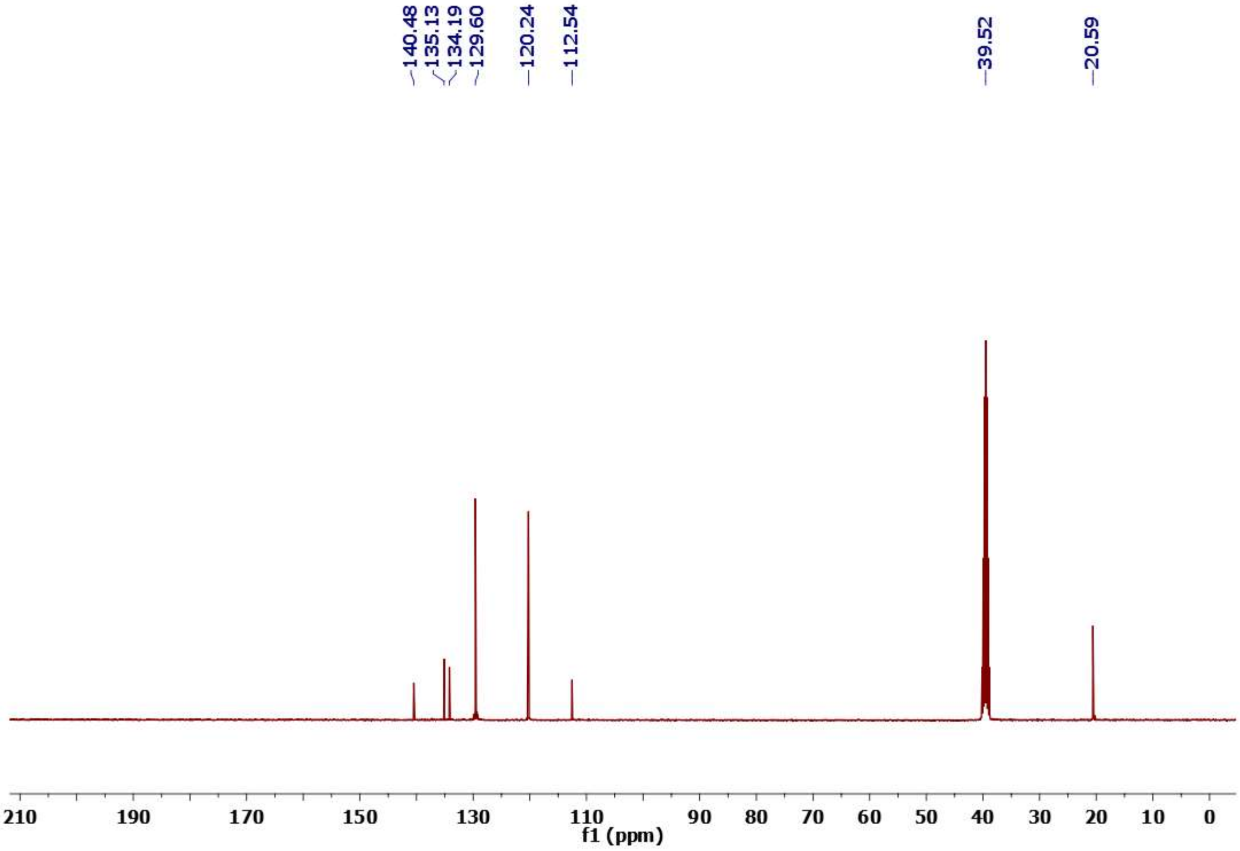

^{13}C CRAPT NMR (DMSO-d6) spectrum of p-tolylcarbamoyl cyanide (2a)

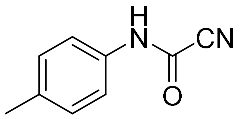

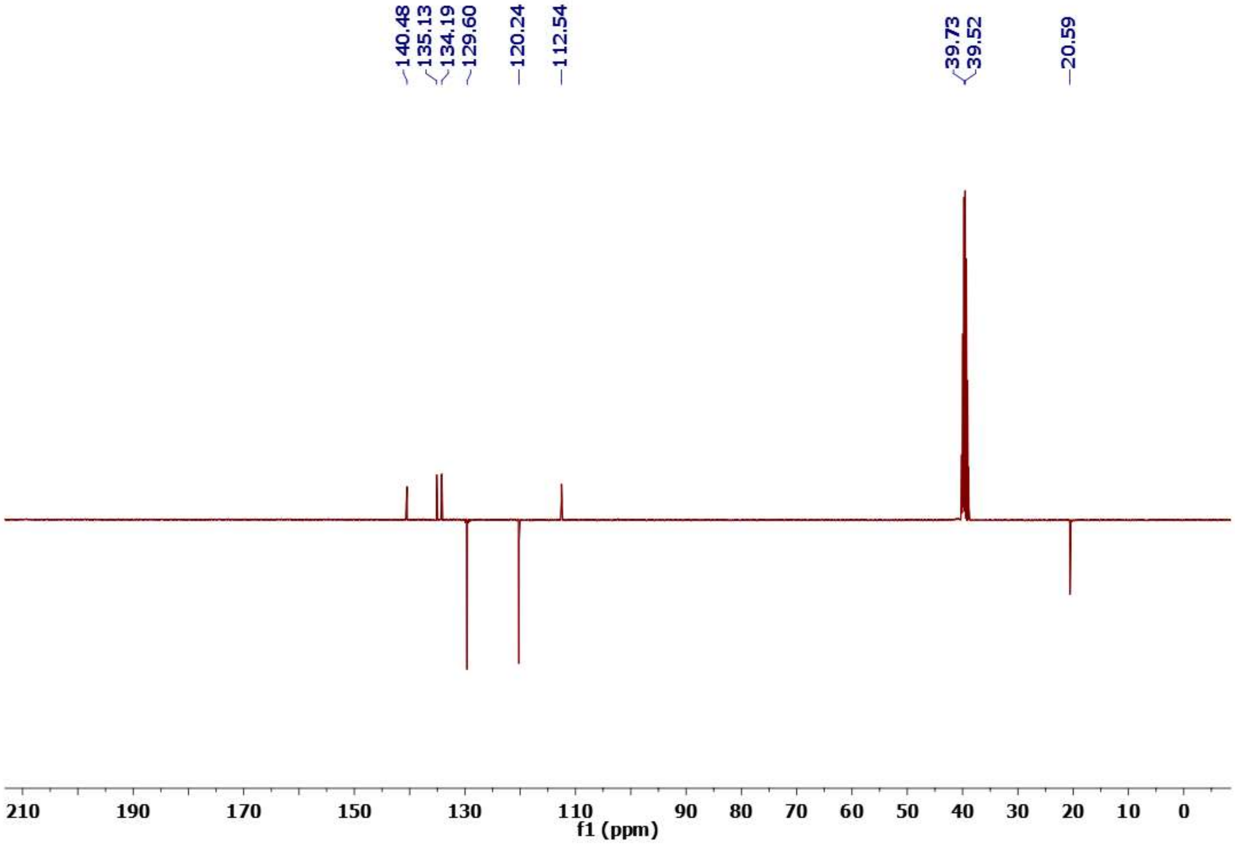

^{1}H-^{1}H gDQCOSY NMR (DMSO-d6) spectrum of p-tolylcarbamoyl cyanide (2a)

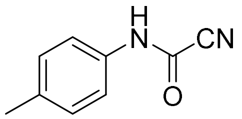

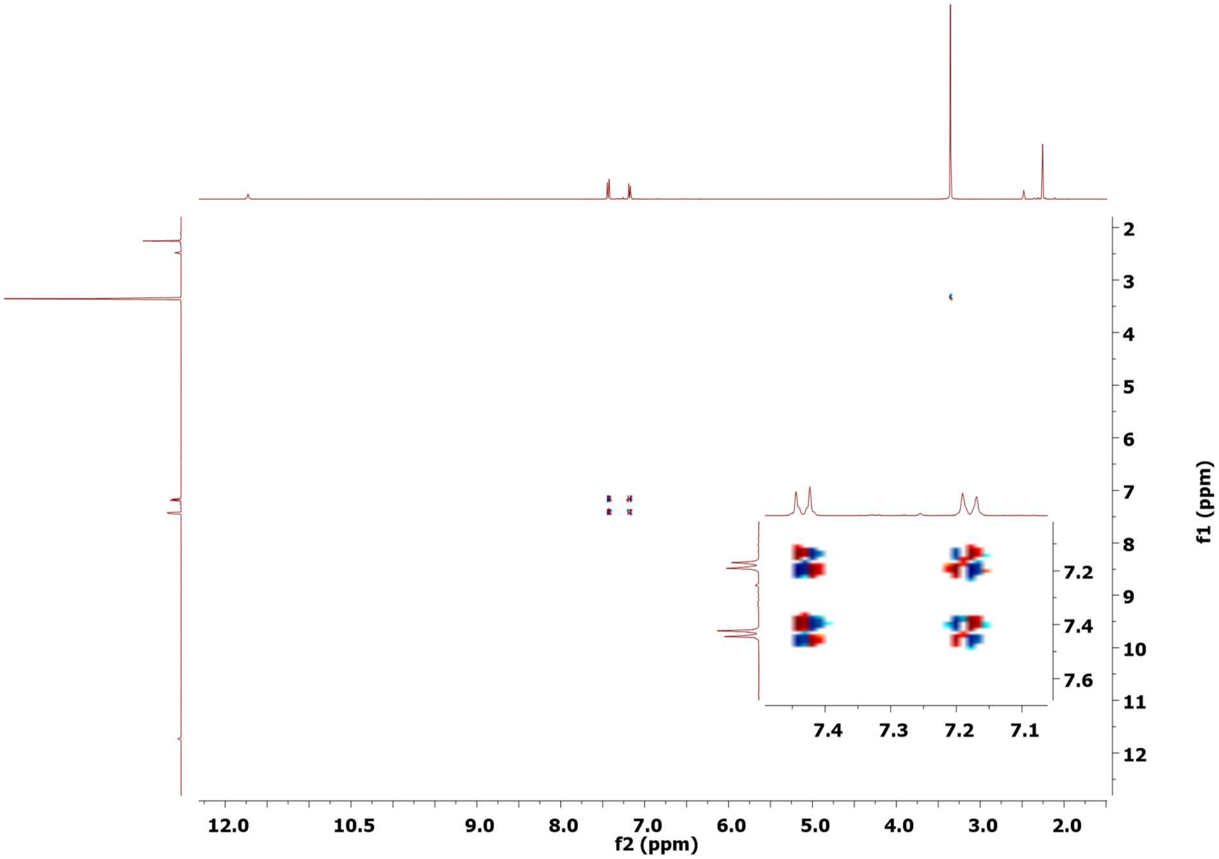

^{1}H NMR (DMSO-d6) spectrum of phenylcarbamothioyl cyanide (2b)

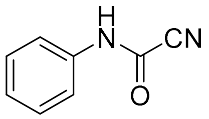

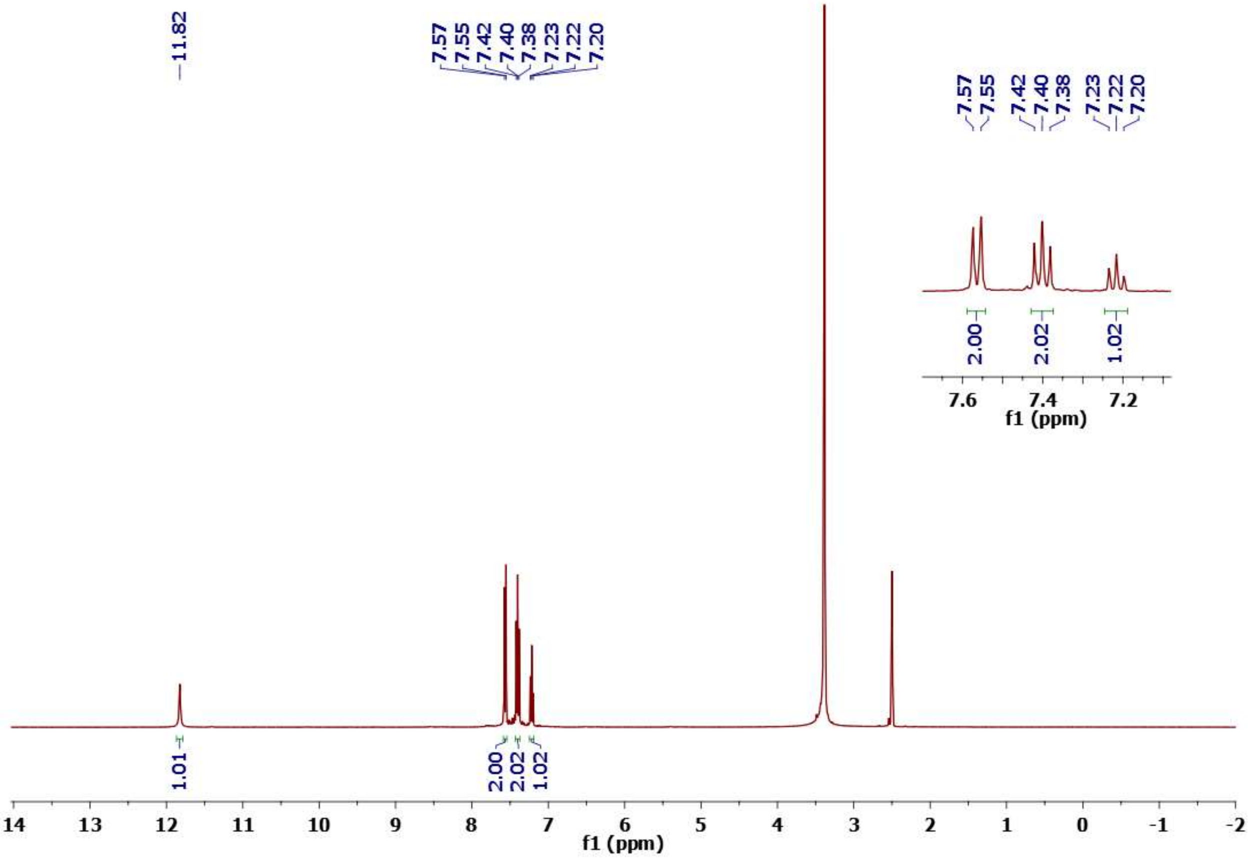

^{13}C NMR (DMSO-d6) spectrum of phenylcarbamothioyl cyanide (2b)

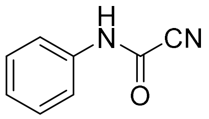

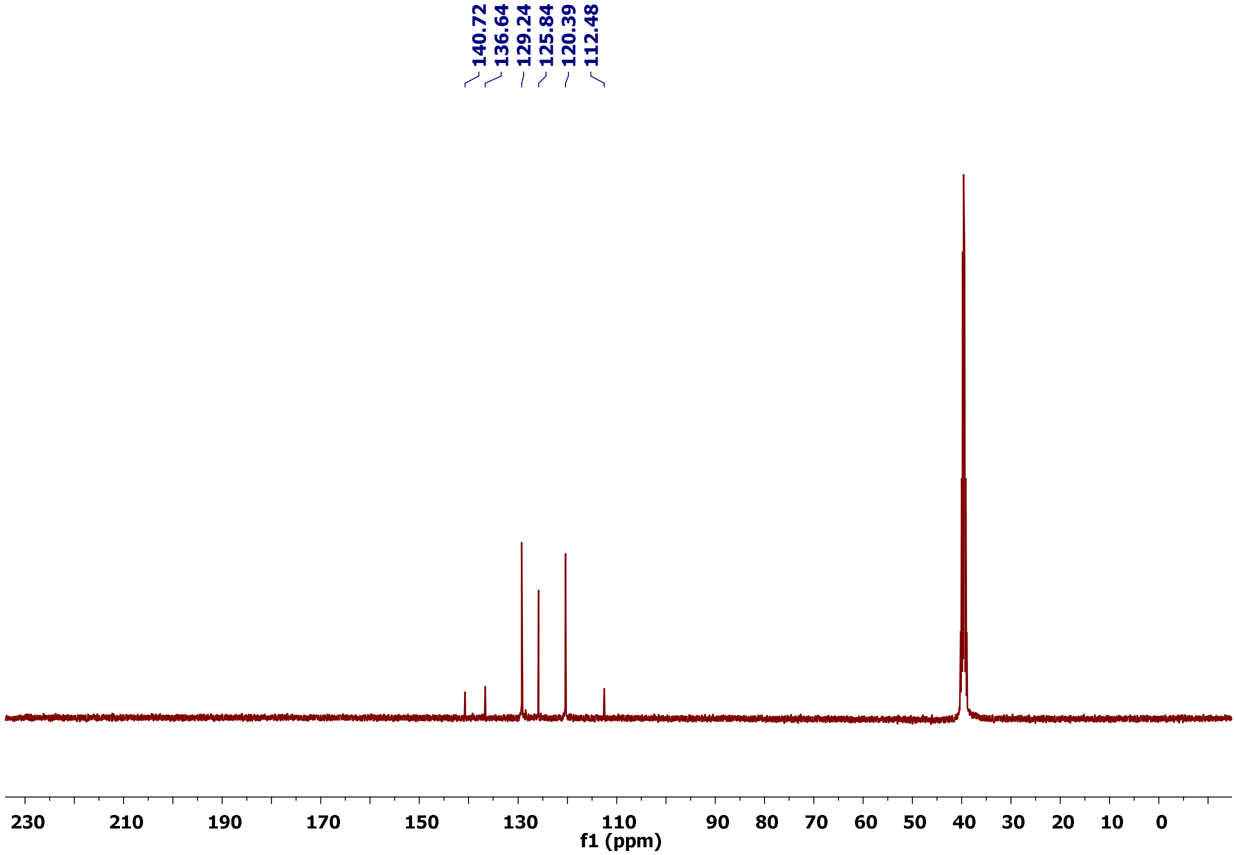

^{13}C CRAPT NMR (DMSO-d6) spectrum of phenylcarbamothioyl cyanide (2b)

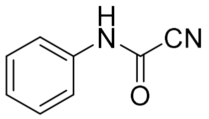

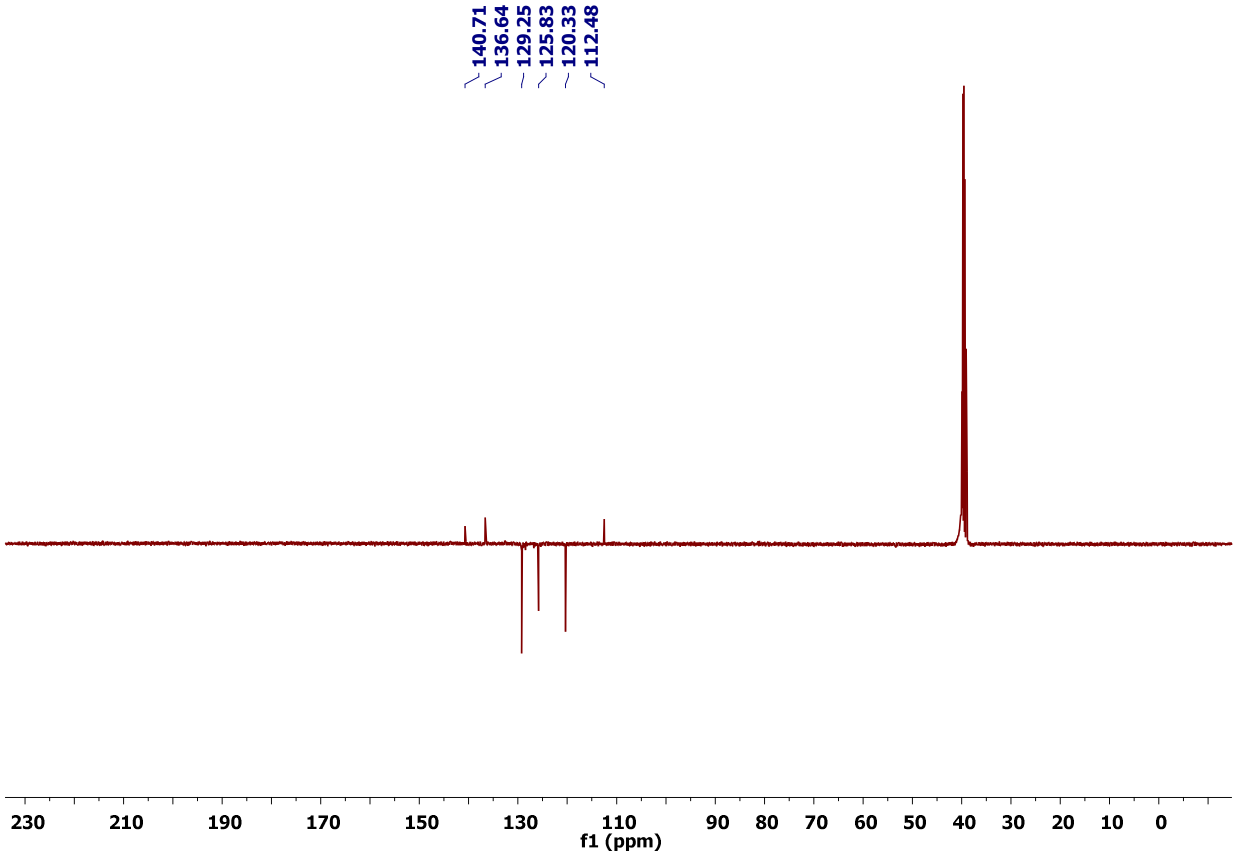

^{1}H NMR (DMSO-d6) spectrum of (4-chlorophenyl)carbamoyl cyanide (2c)

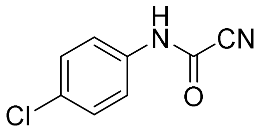

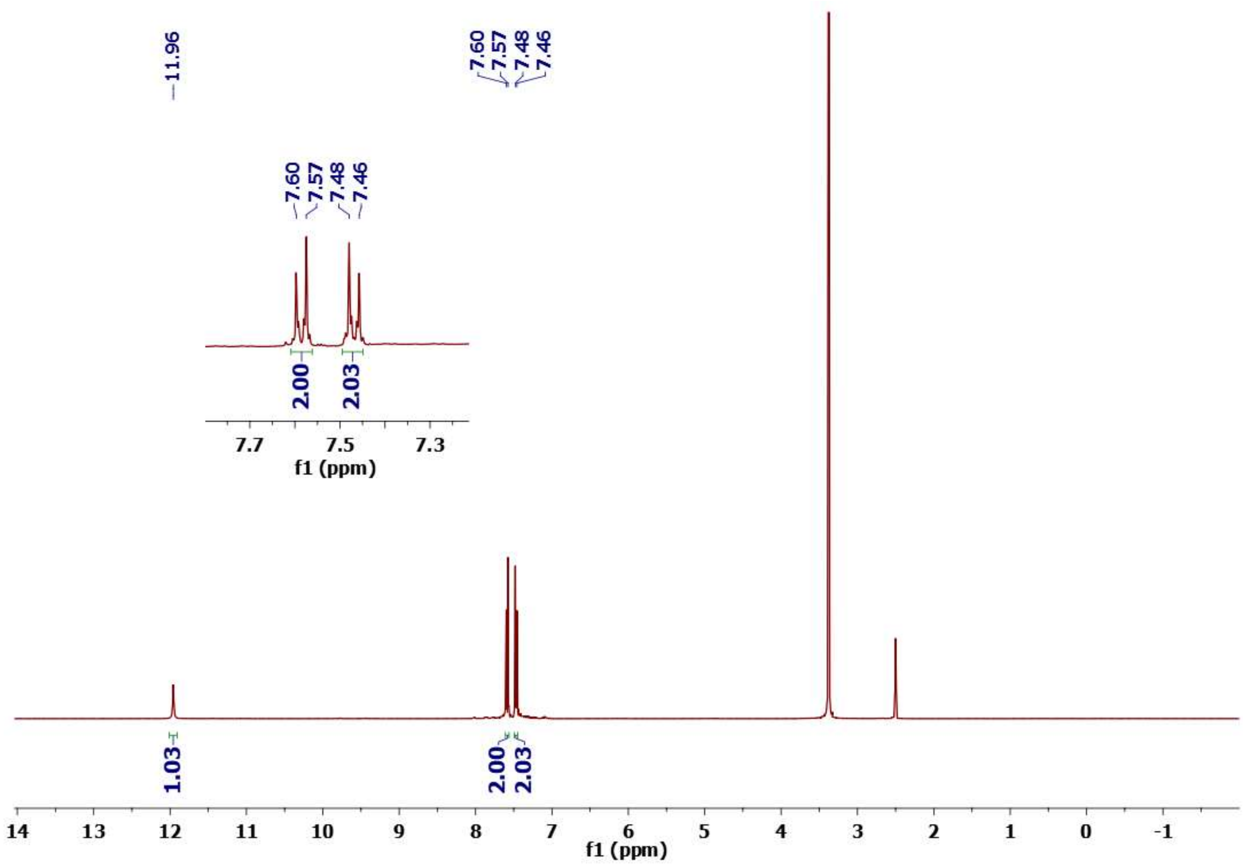

^{13}C NMR (DMSO-d6) spectrum of (4-chlorophenyl)carbamoyl cyanide (2c)

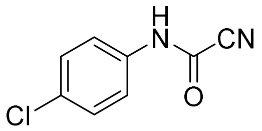

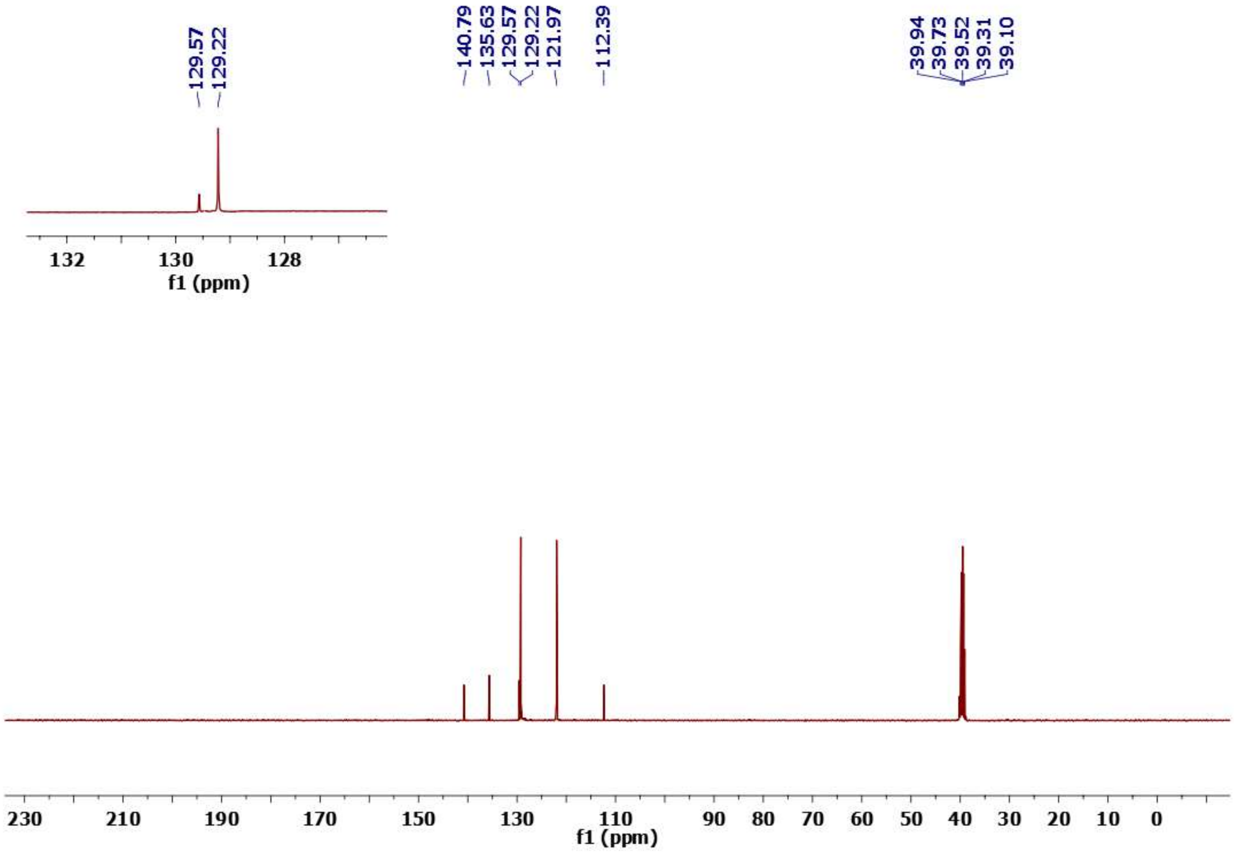

^{13}C CRAPT NMR (DMSO-d6) spectrum of (4-chlorophenyl)carbamoyl cyanide (2c)

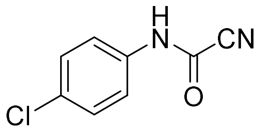

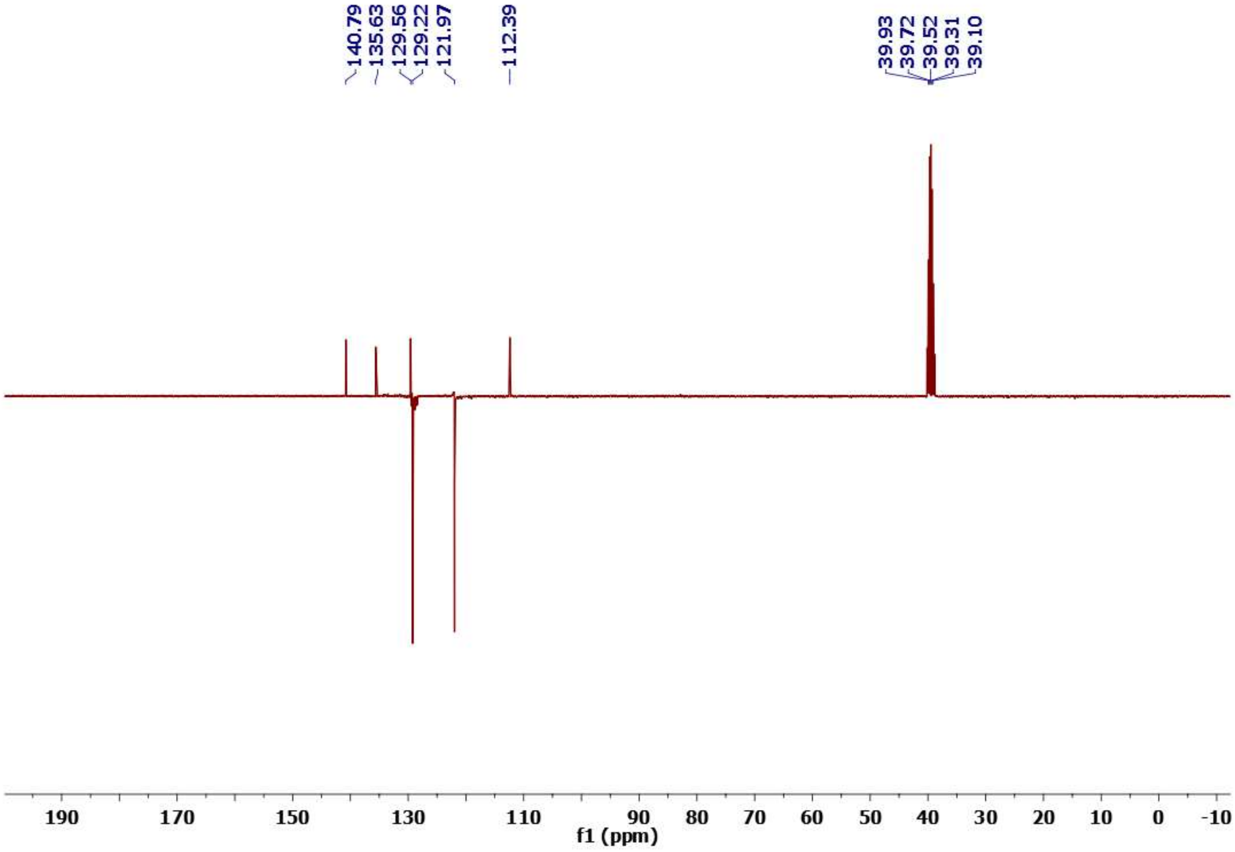

^{1}H-^{1}H gDQCOSY NMR (DMSO-d6) spectrum of (4-chlorophenyl)carbamoyl cyanide (2c)

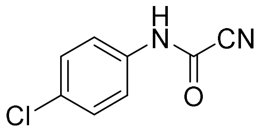

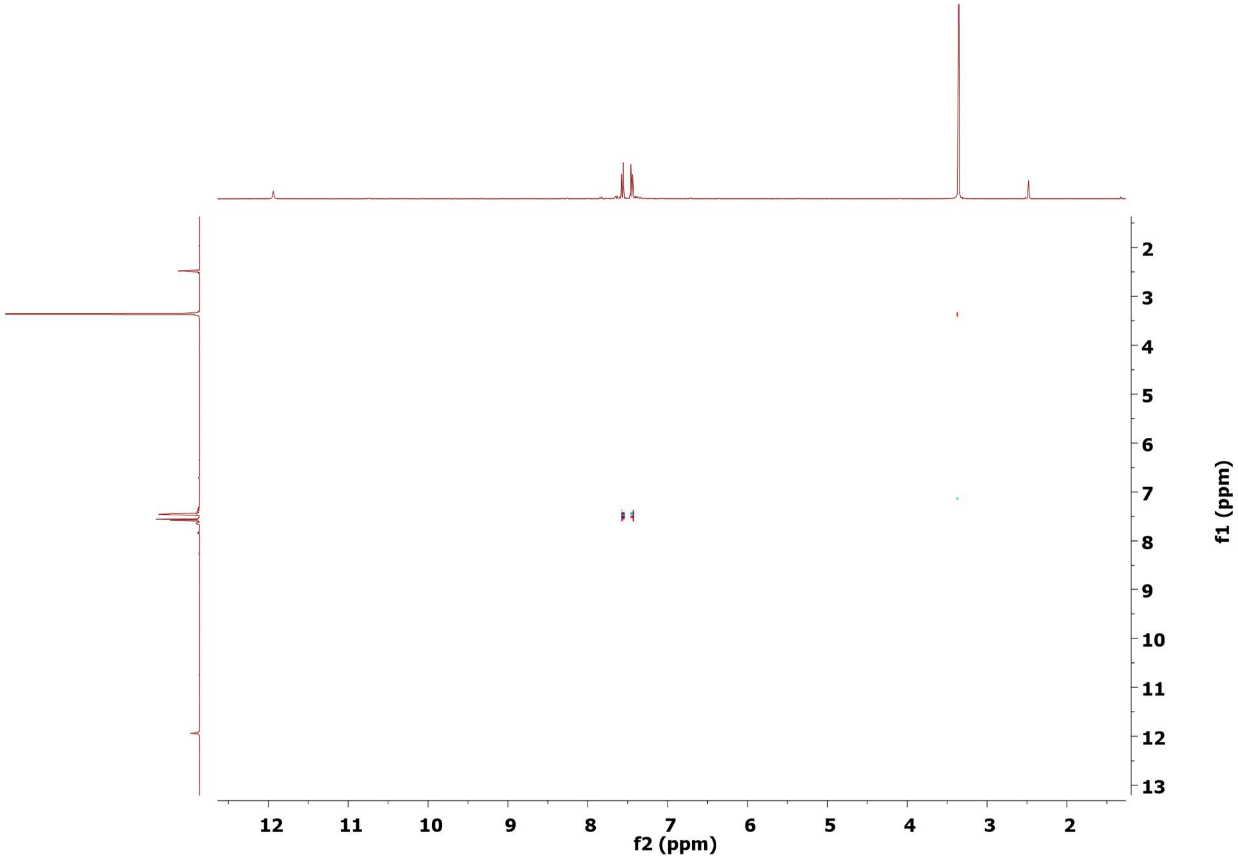

^{1}H NMR (DMSO-d6) spectrum of (2-fluorophenyl)carbamoyl cyanide (2d)

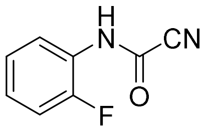

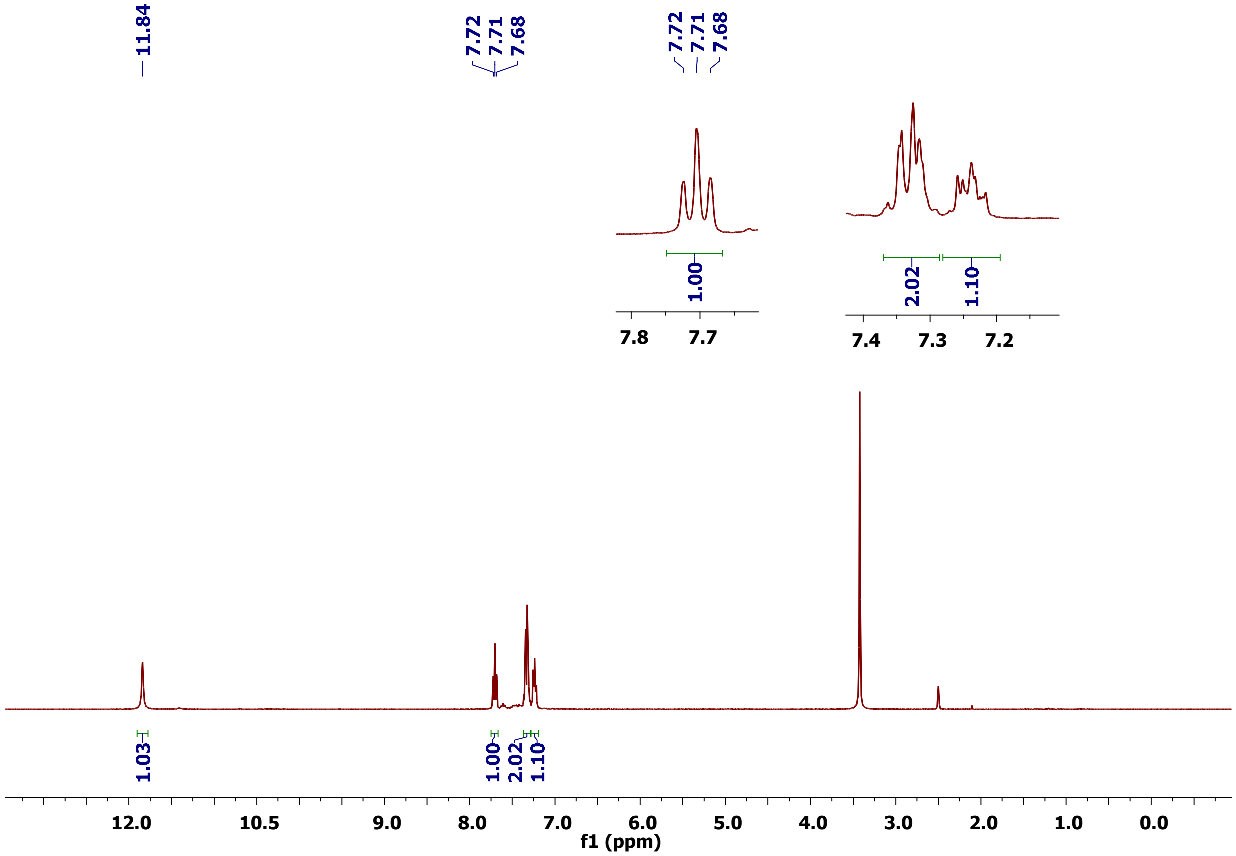

^{13}C NMR (DMSO-d6) spectrum of (2-fluorophenyl)carbamoyl cyanide (2d)

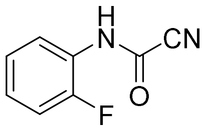

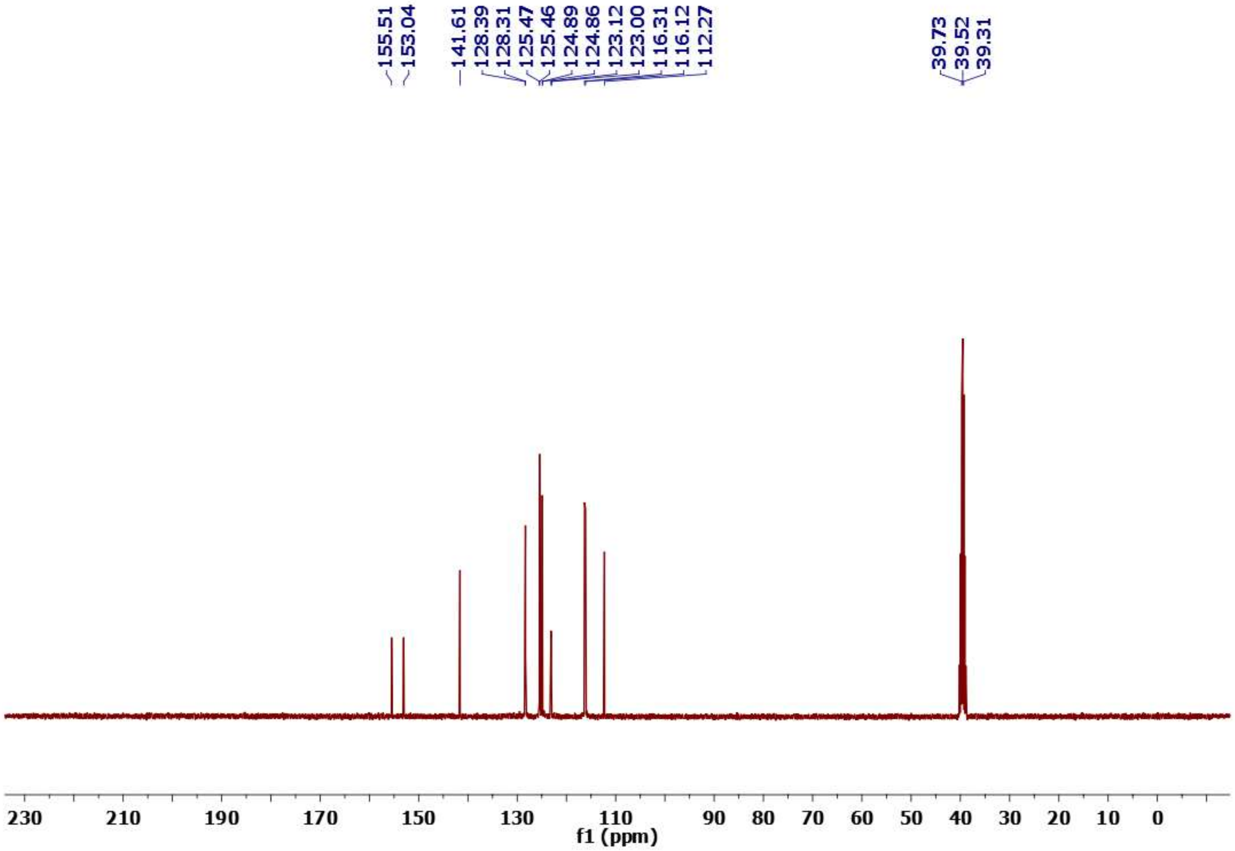

^{13}C CRAPT NMR (DMSO-d6) spectrum of (2-fluorophenyl)carbamoyl cyanide (2d)

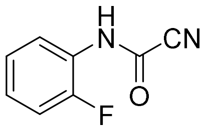

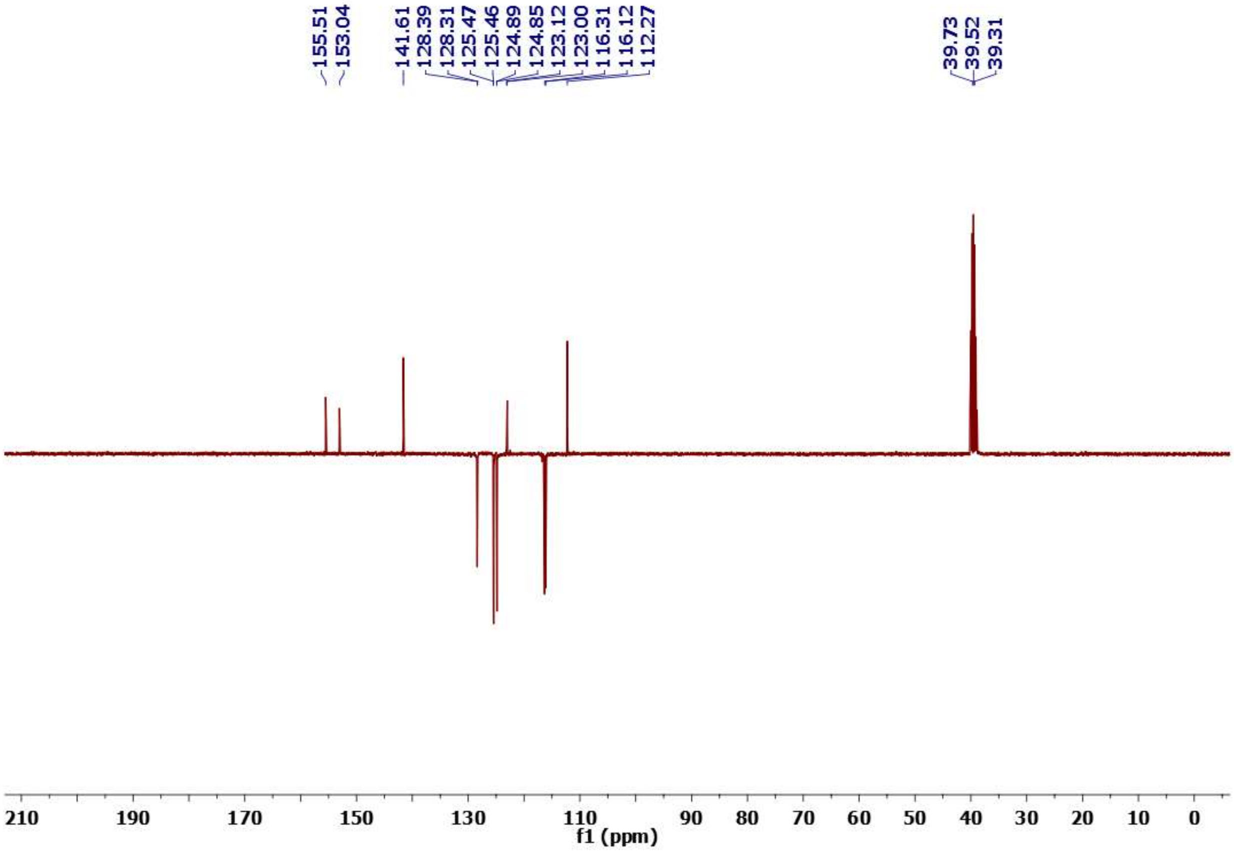

^{1}H-^{1}H gDQCOSY NMR (DMSO-d6) spectrum of (3-fluorophenyl)carbamoyl cyanide (2d)

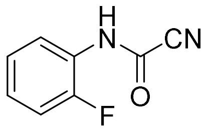

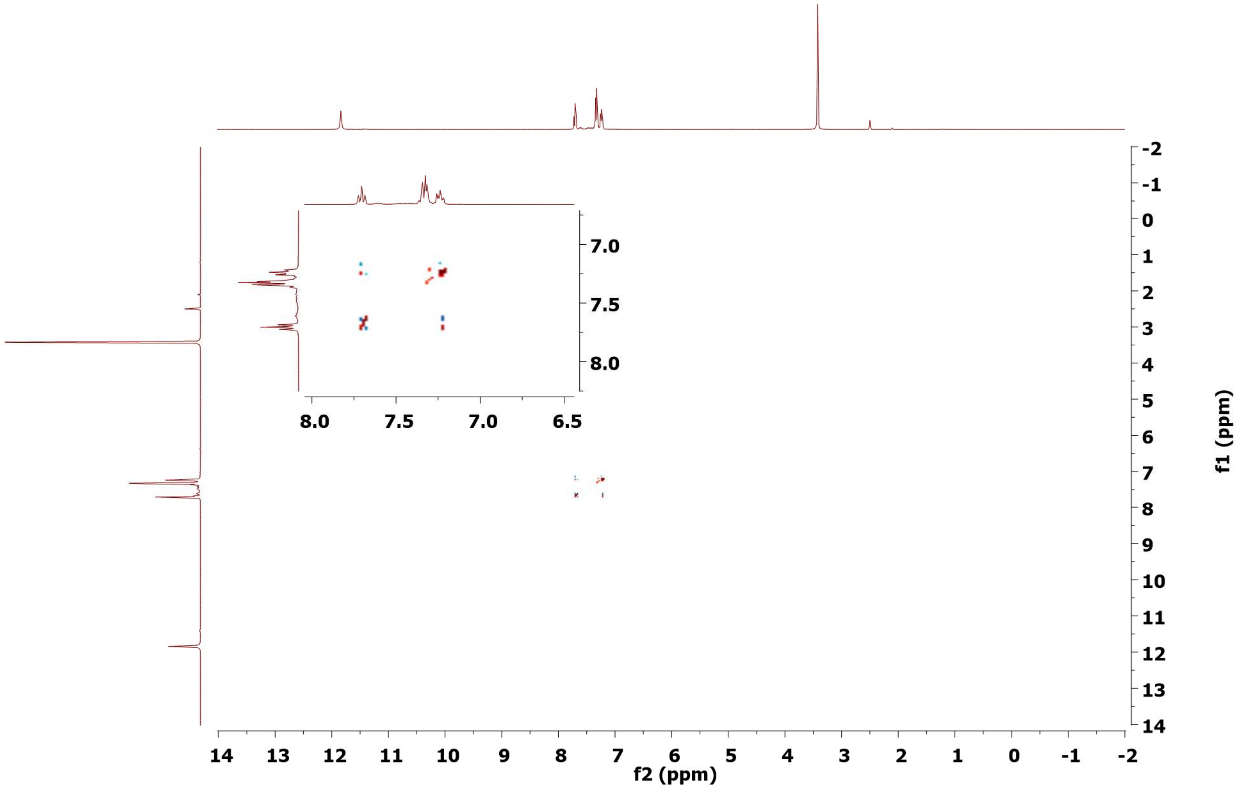

^{1}H-^{13}C-gHSQC NMR (DMSO-d6) spectrum of (3-fluorophenyl)carbamoyl cyanide (2d)

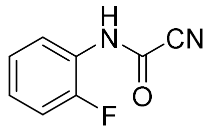

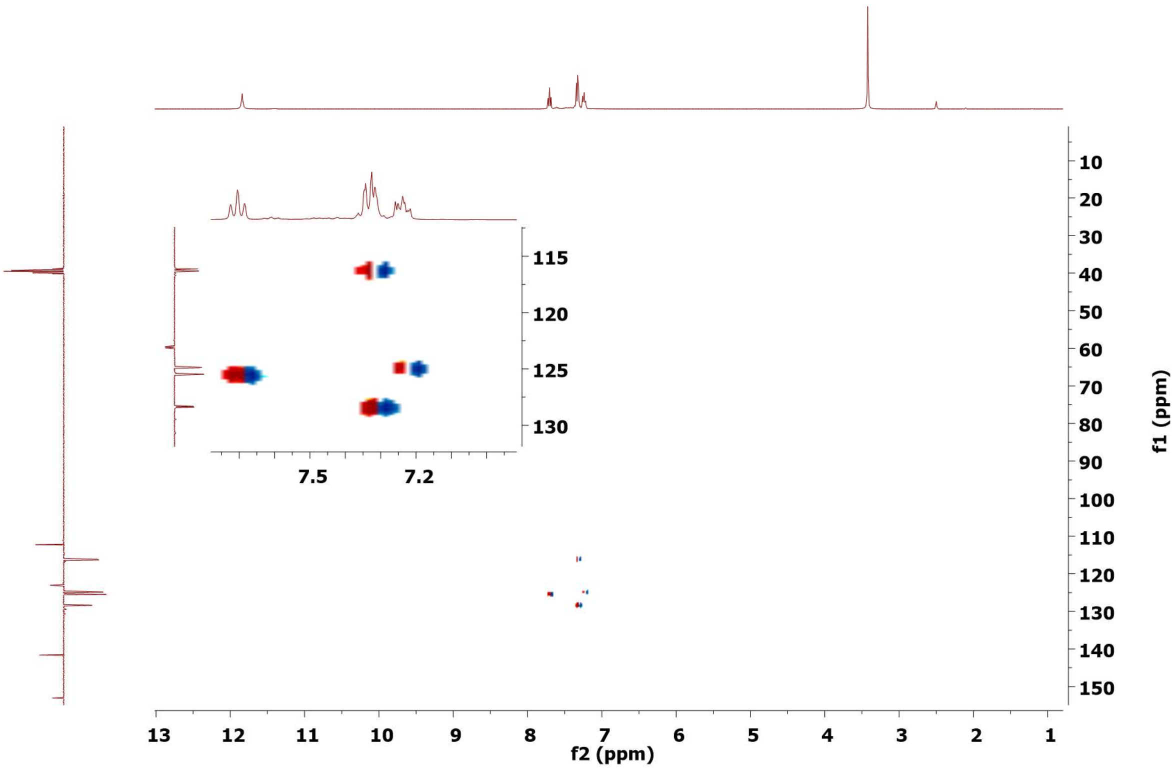

^{1}H-^{13}C-gHMBC NMR (DMSO-d6) spectrum of (3-fluorophenyl)carbamoyl cyanide (2d)

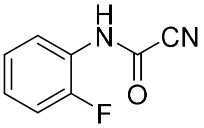

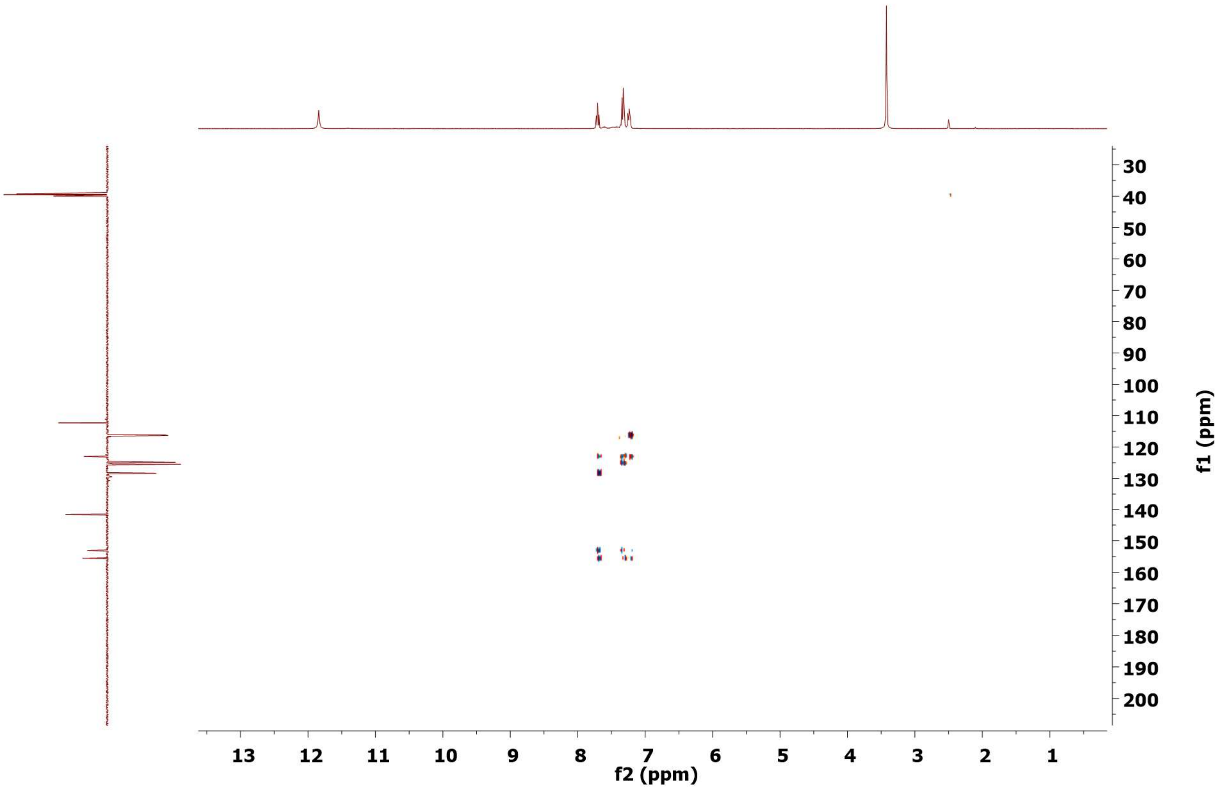

^{1}H NMR (DMSO-d6) spectrum of (4-fluorophenyl)carbamoyl cyanide (2e)

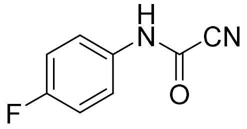

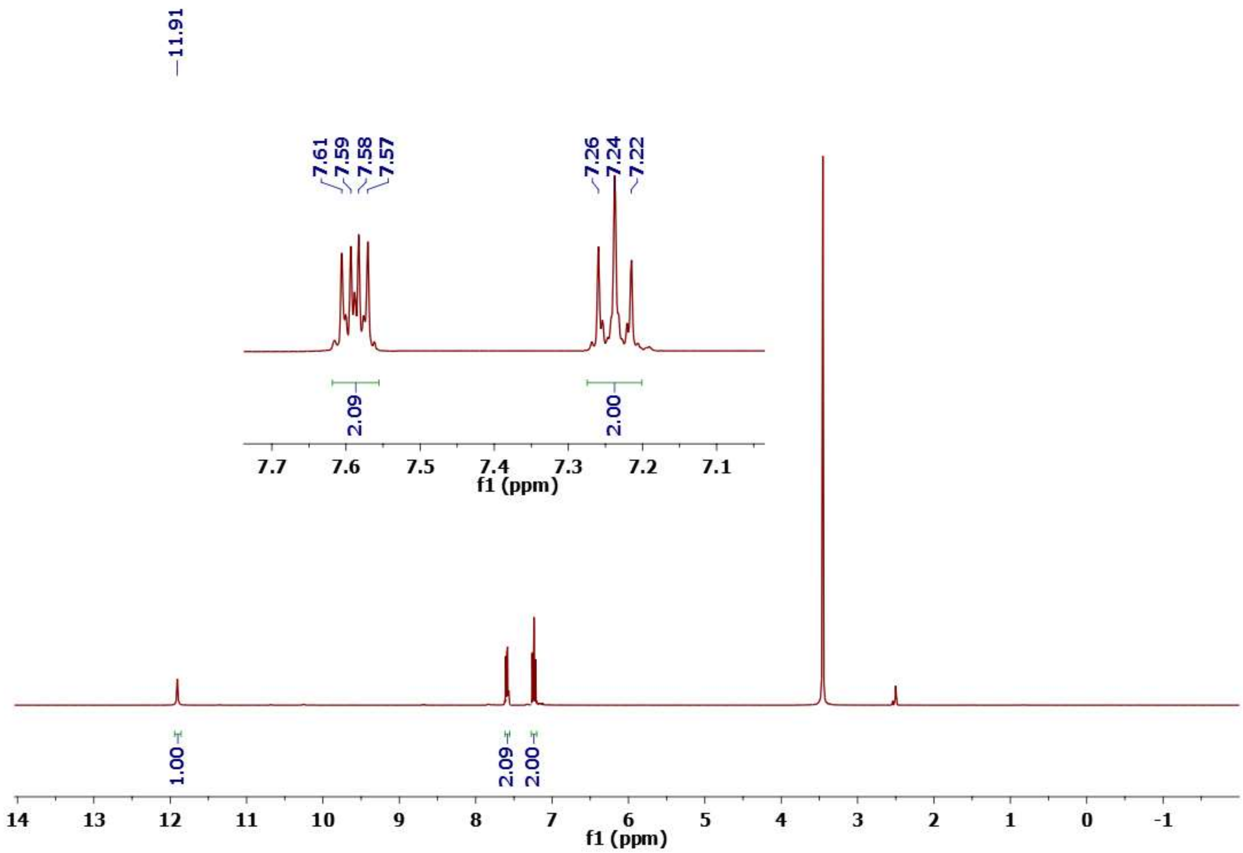

^{13}C NMR (DMSO-d6) spectrum spectrum of (4-fluorophenyl)carbamoyl cyanide (2e)

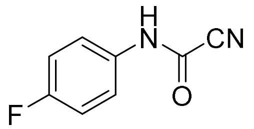

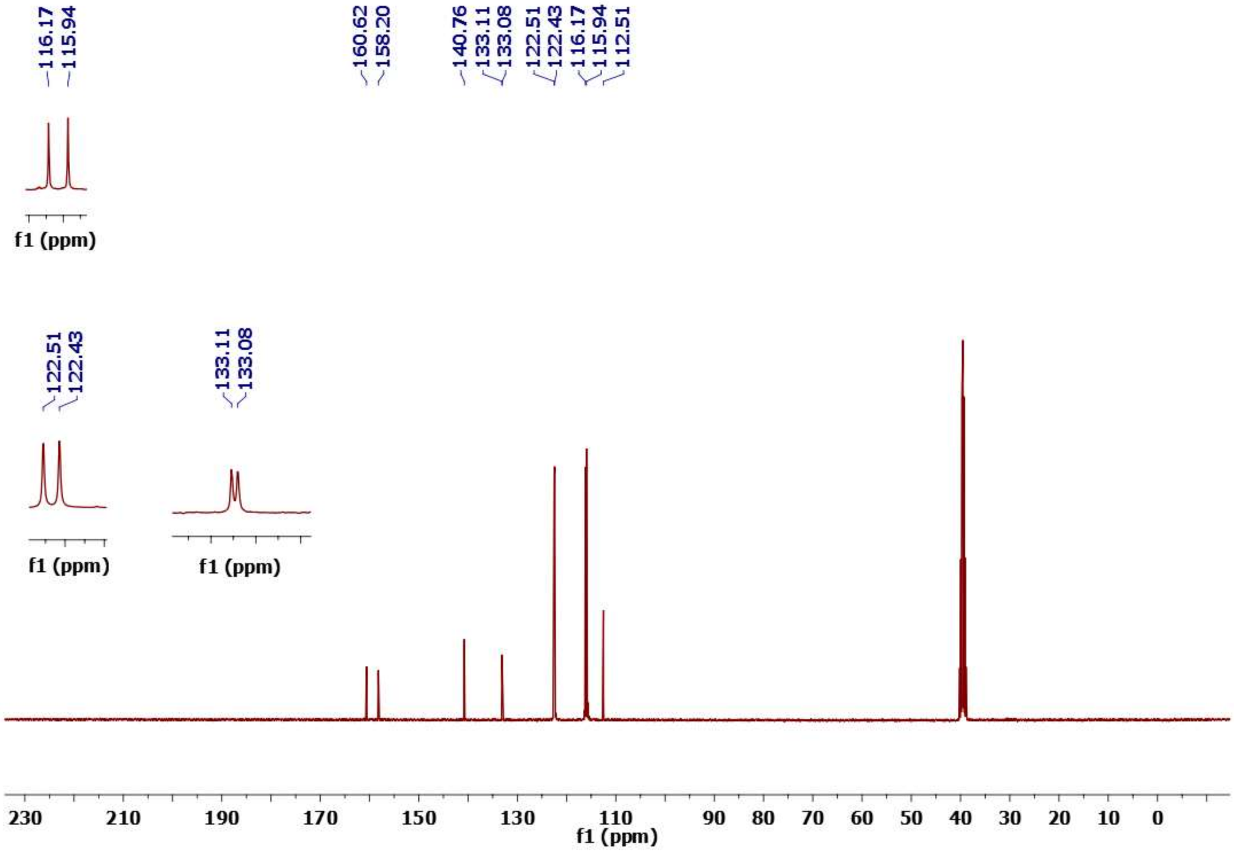

^{13}C CRAPT NMR (DMSO-d6) spectrum of spectrum of (4-fluorophenyl)carbamoyl cyanide (2e)

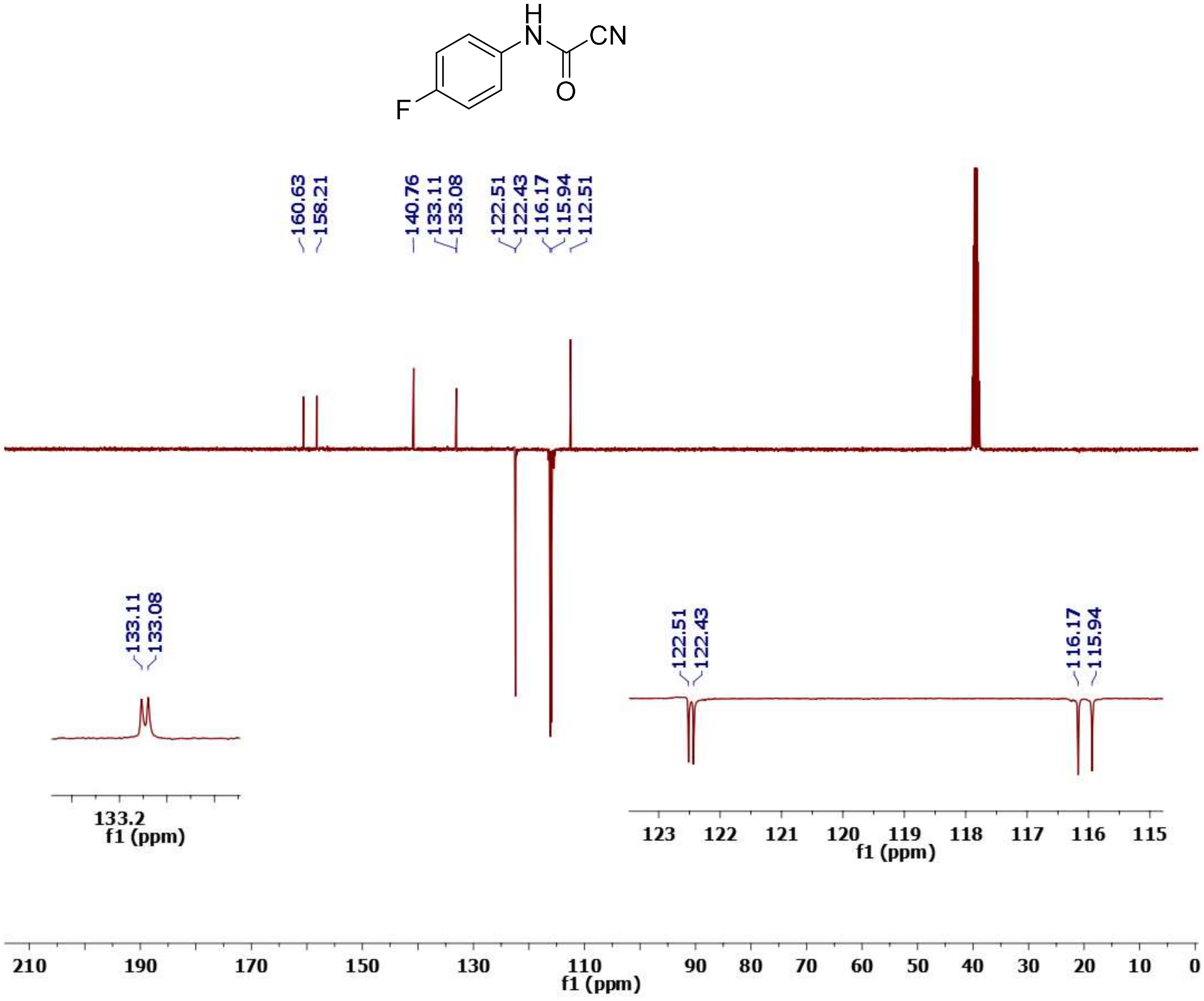

^{1}H NMR (DMSO-d6) spectrum of (3-fluorophenyl)carbamoyl cyanide (2f)

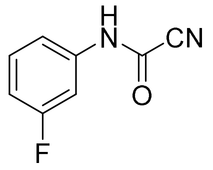

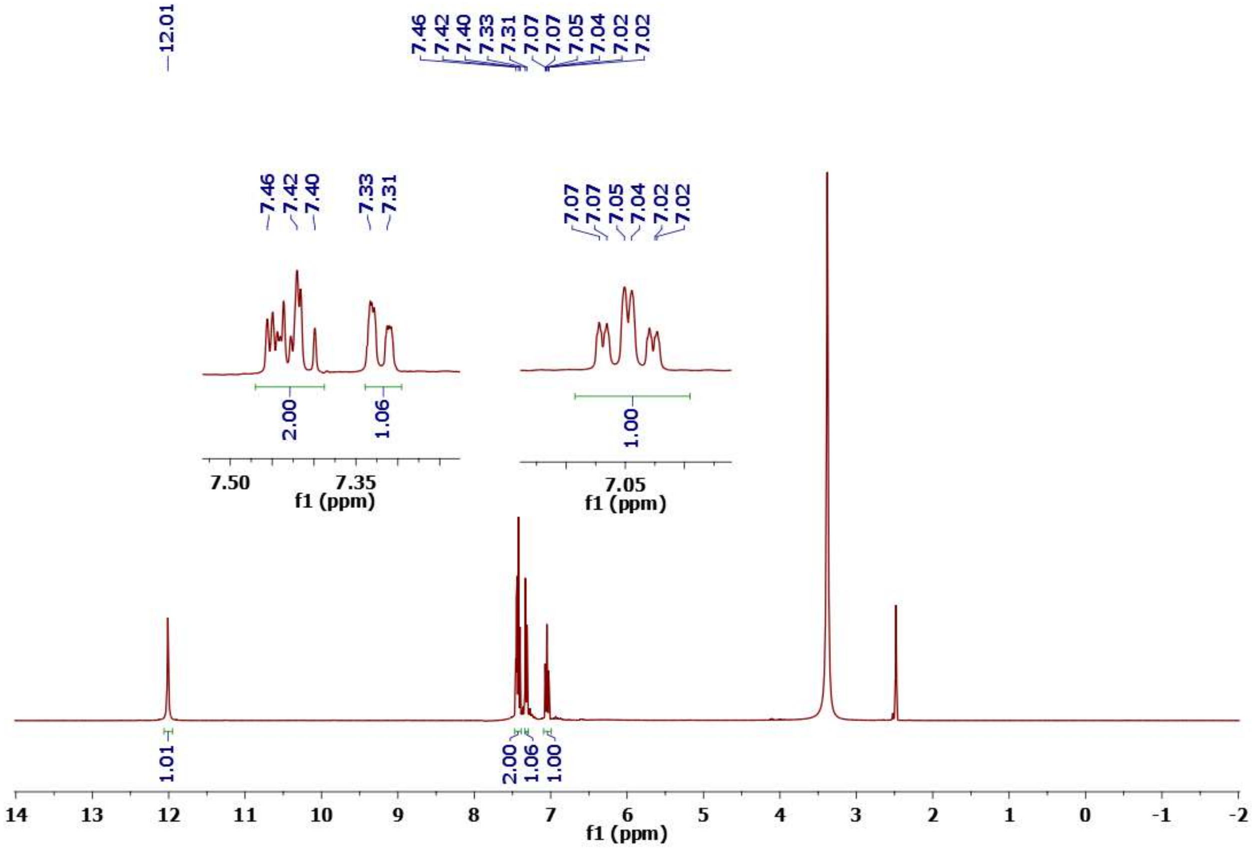

^{13}C NMR (DMSO-d6) spectrum of (3-fluorophenyl)carbamoyl cyanide (2f)

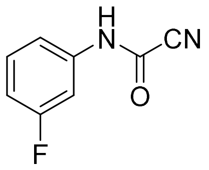

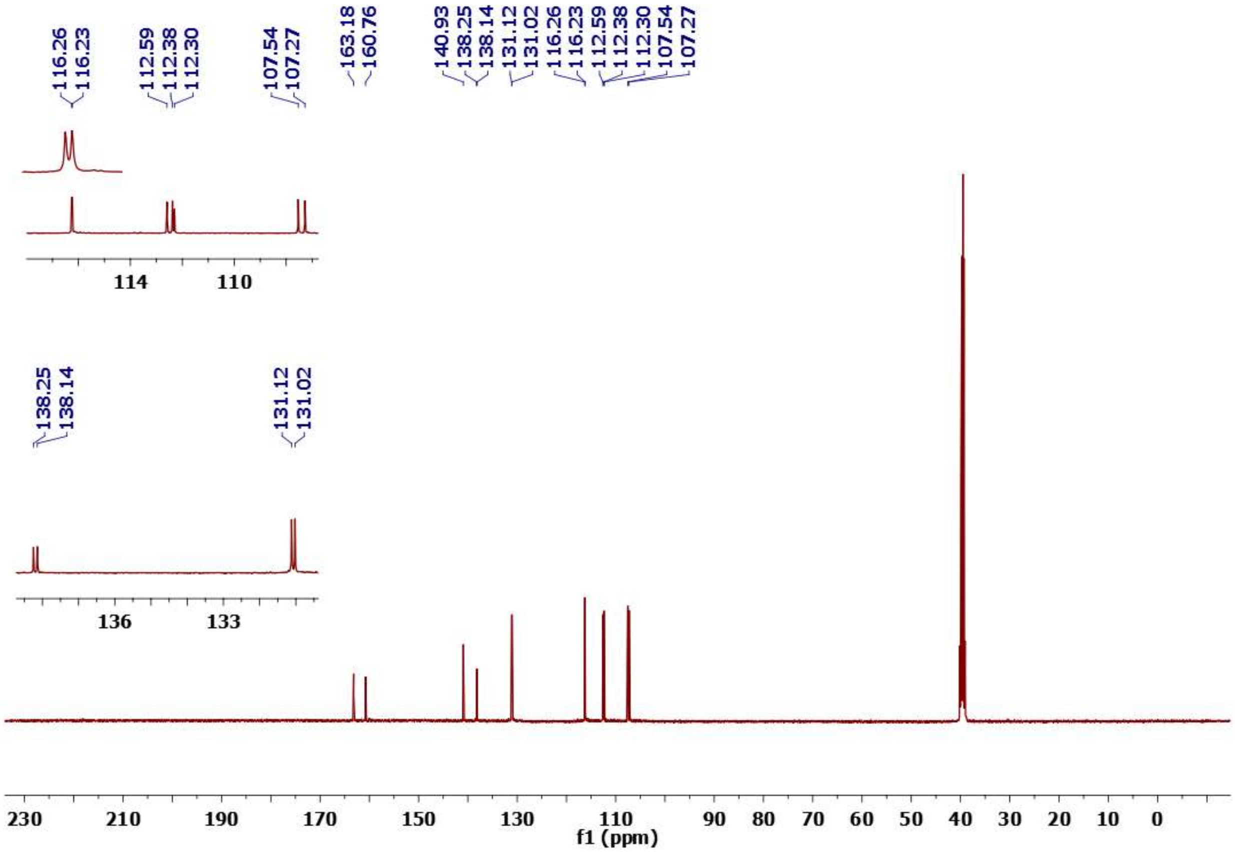

^{13}C CRAPT NMR (DMSO-d6) spectrum of (3-fluorophenyl)carbamoyl cyanide (2f)

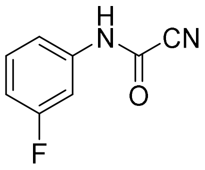

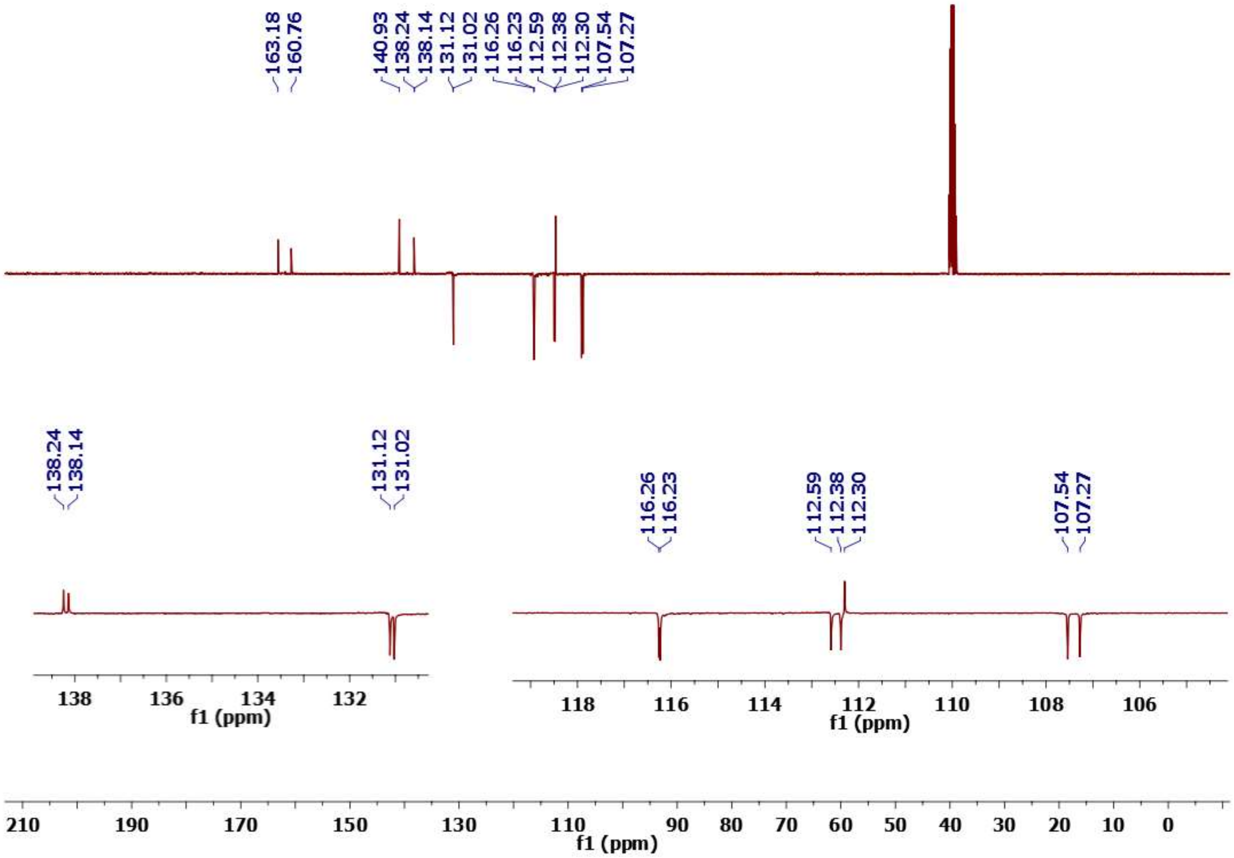

^1H-^1H gDQCOSY NMR (DMSO-d6) spectrum of (3-fluorophenyl)carbamoyl cyanide (2f)

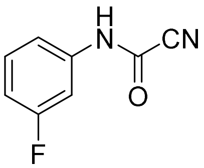

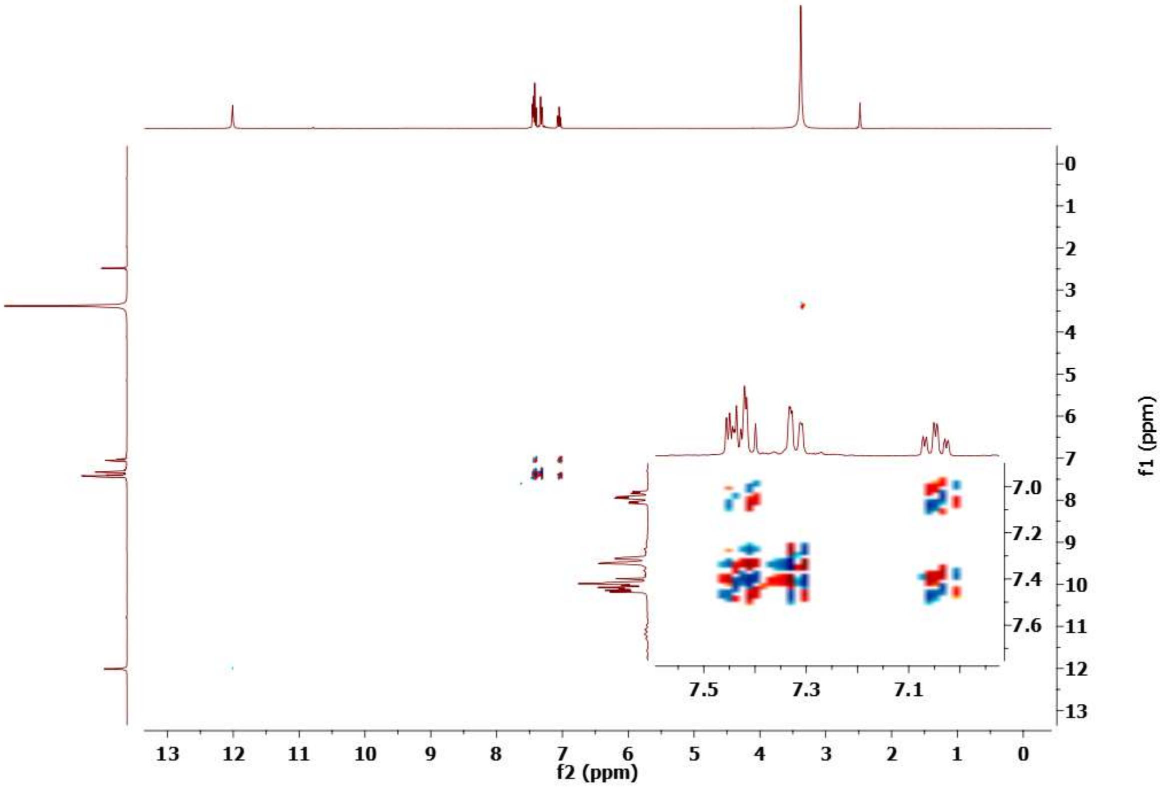

^{1}H NMR (DMSO-d6) spectrum of (4-nitrophenyl)carbamoyl cyanide (2g)

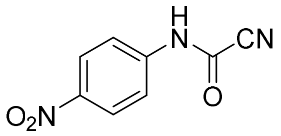

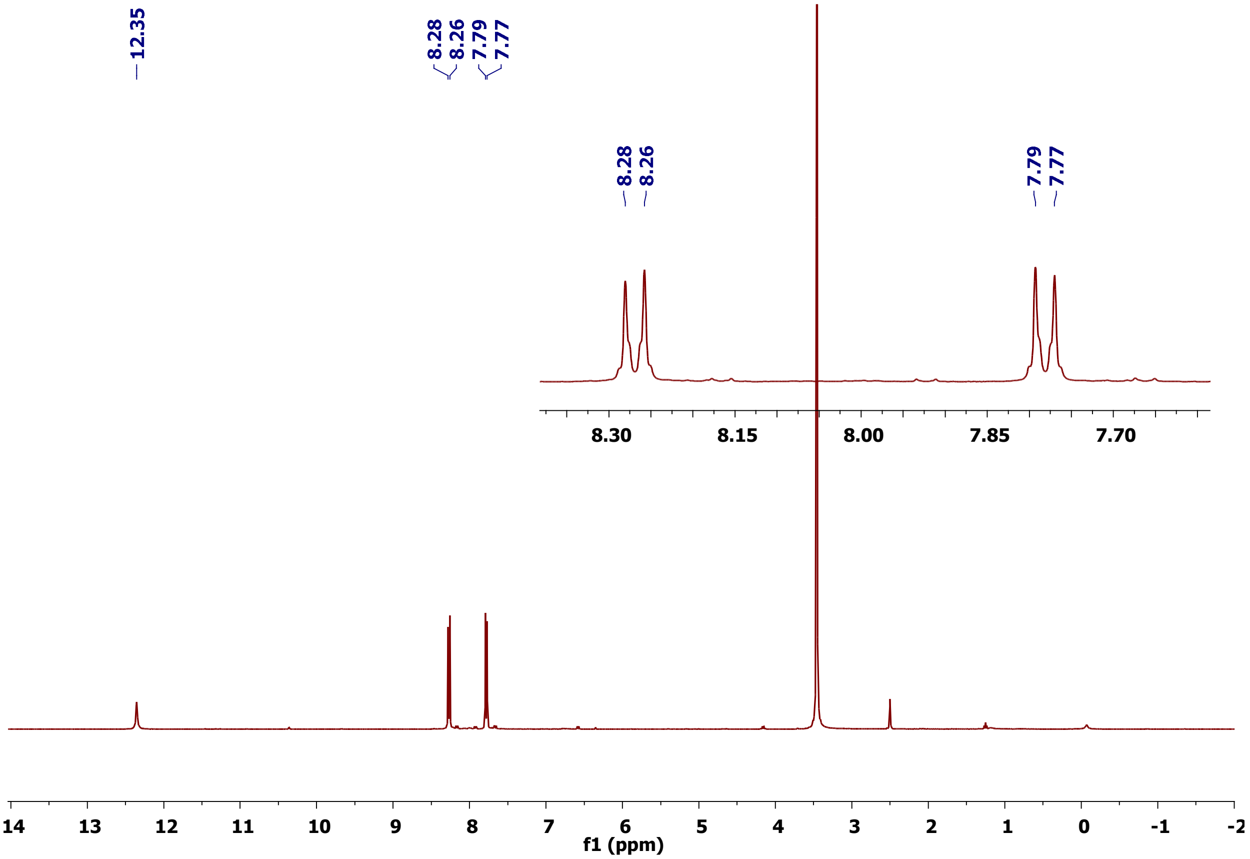

^{13}C NMR (DMSO-d6) spectrum of (4-nitrophenyl)carbamoyl cyanide (2g)

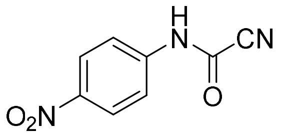

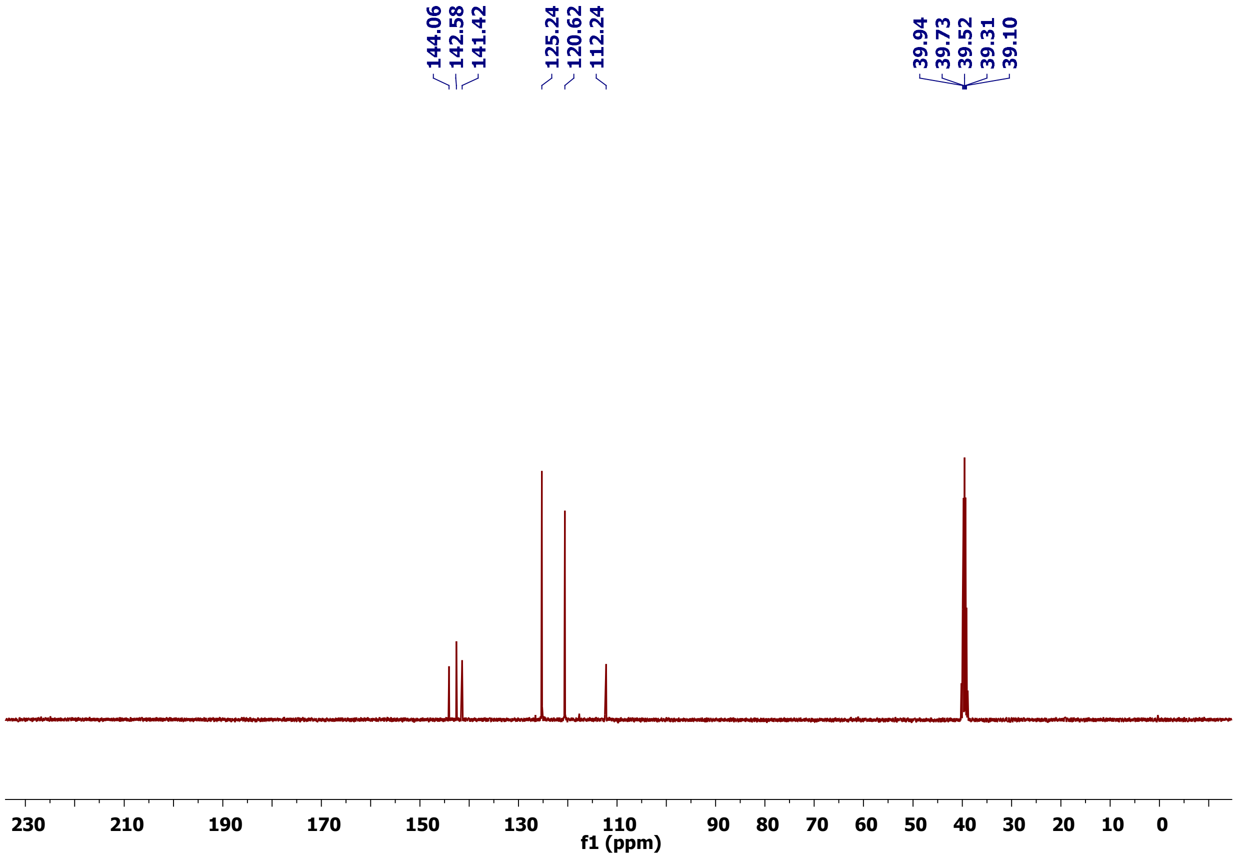

^{13}C CRAPT NMR (DMSO-d6) spectrum of (4-nitrophenyl)carbamoyl cyanide (2g)

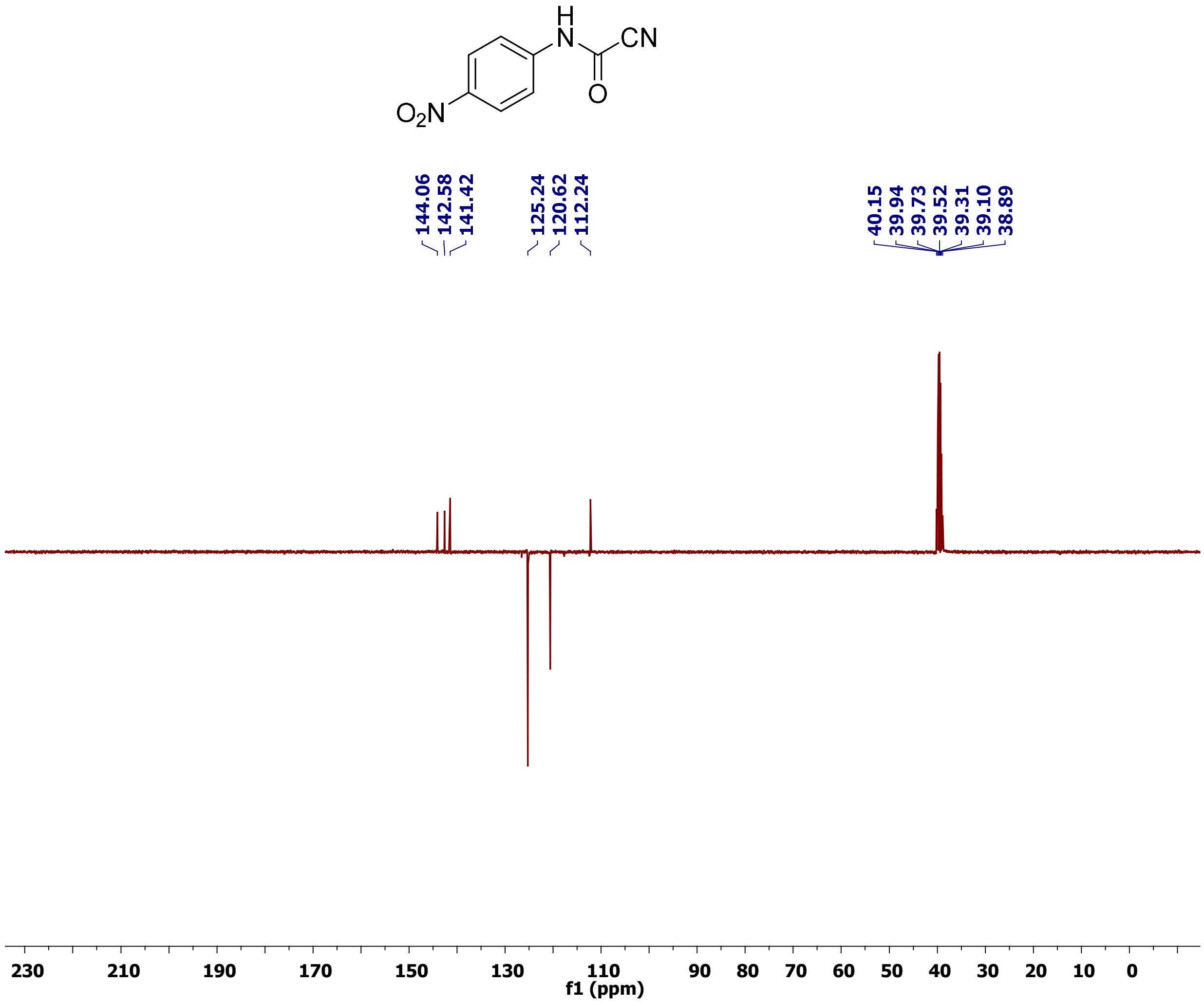

^1H-^1H gDQCOSY NMR (DMSO-d6) spectrum of (4-nitrophenyl)carbamoyl cyanide (2g)

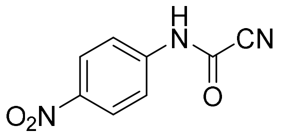

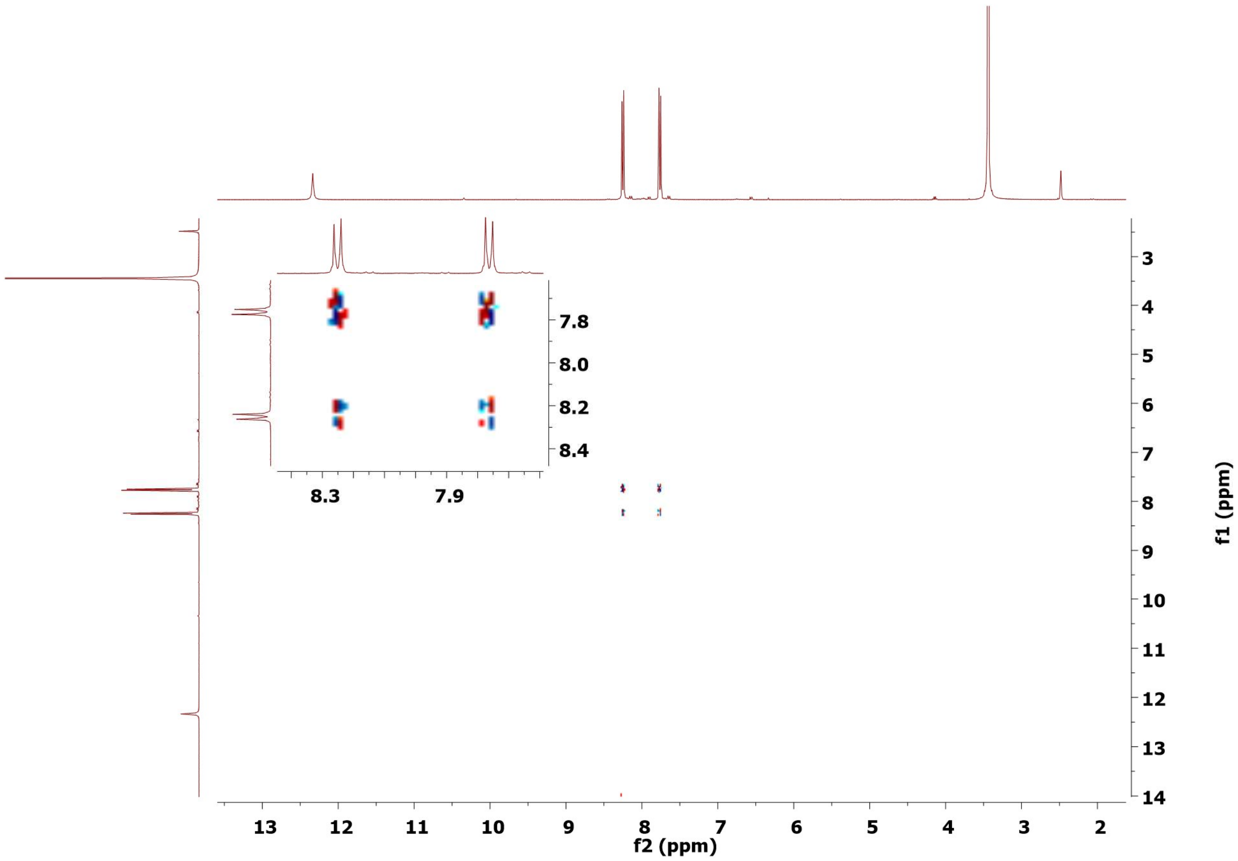

^{1}H-^{13}C-gHSQC NMR (DMSO-d6) spectrum of (4-nitrophenyl)carbamoyl cyanide (2g)

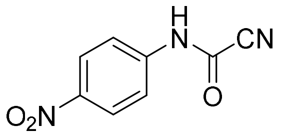

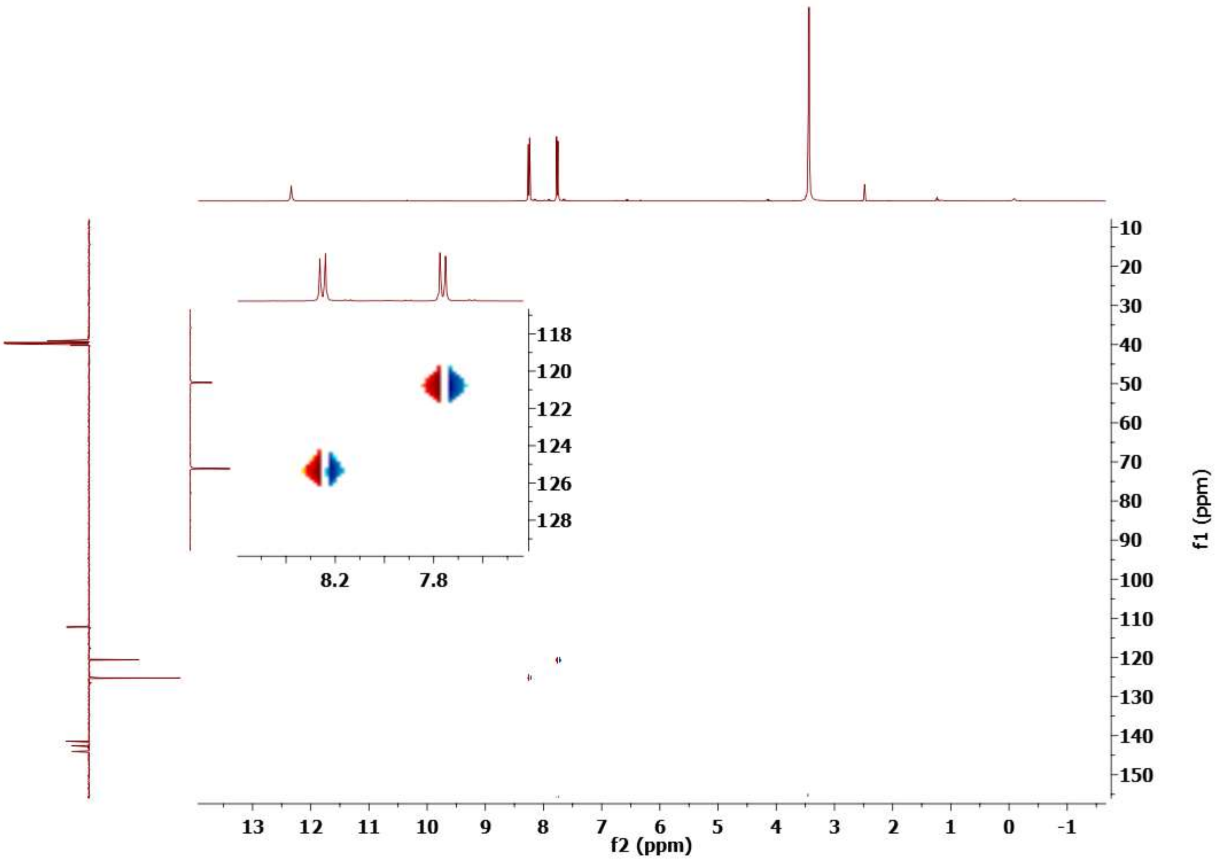

^{1}H-^{13}C-gHMBC NMR (DMSO-d6) spectrum of (4-nitrophenyl)carbamoyl cyanide (2g)

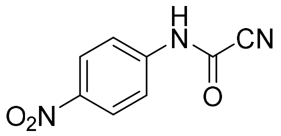

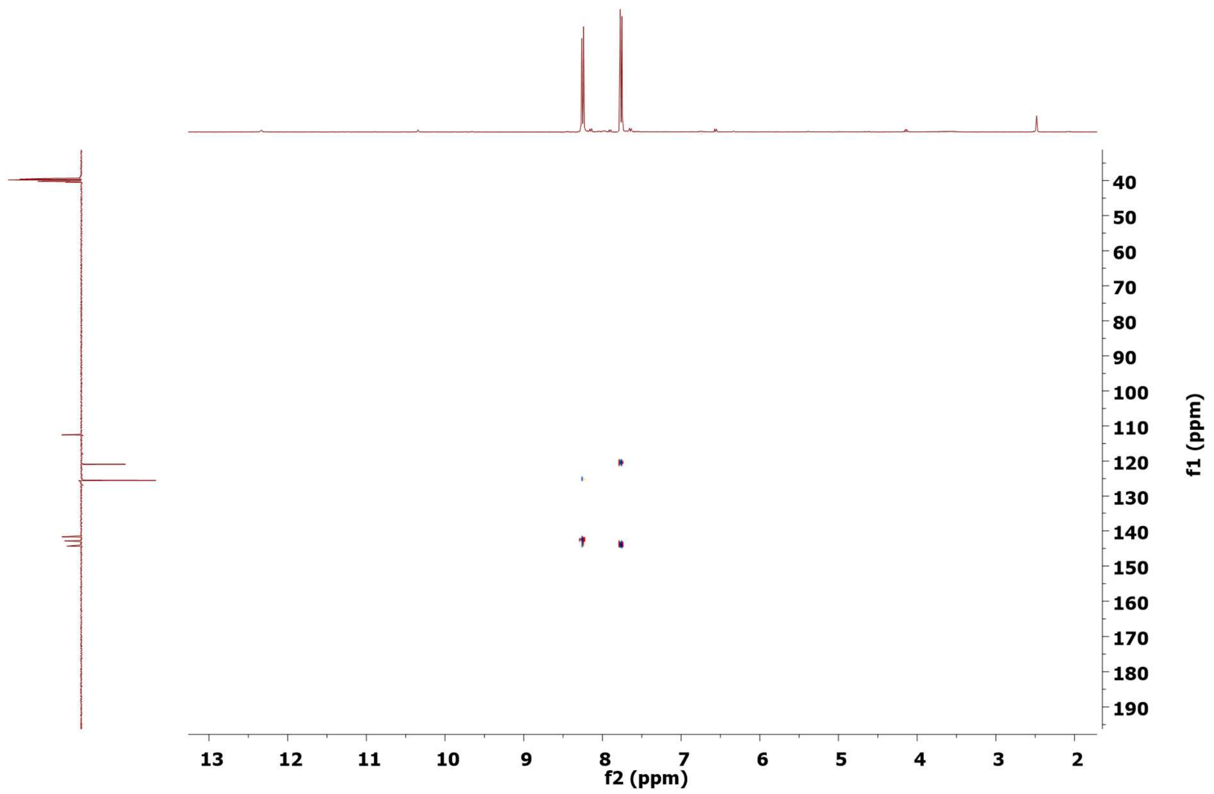

^{1}H NMR (DMSO-d6) spectrum of (3-nitrophenyl)carbamoyl cyanide (2h)

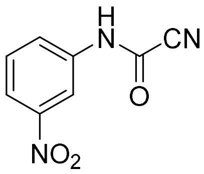

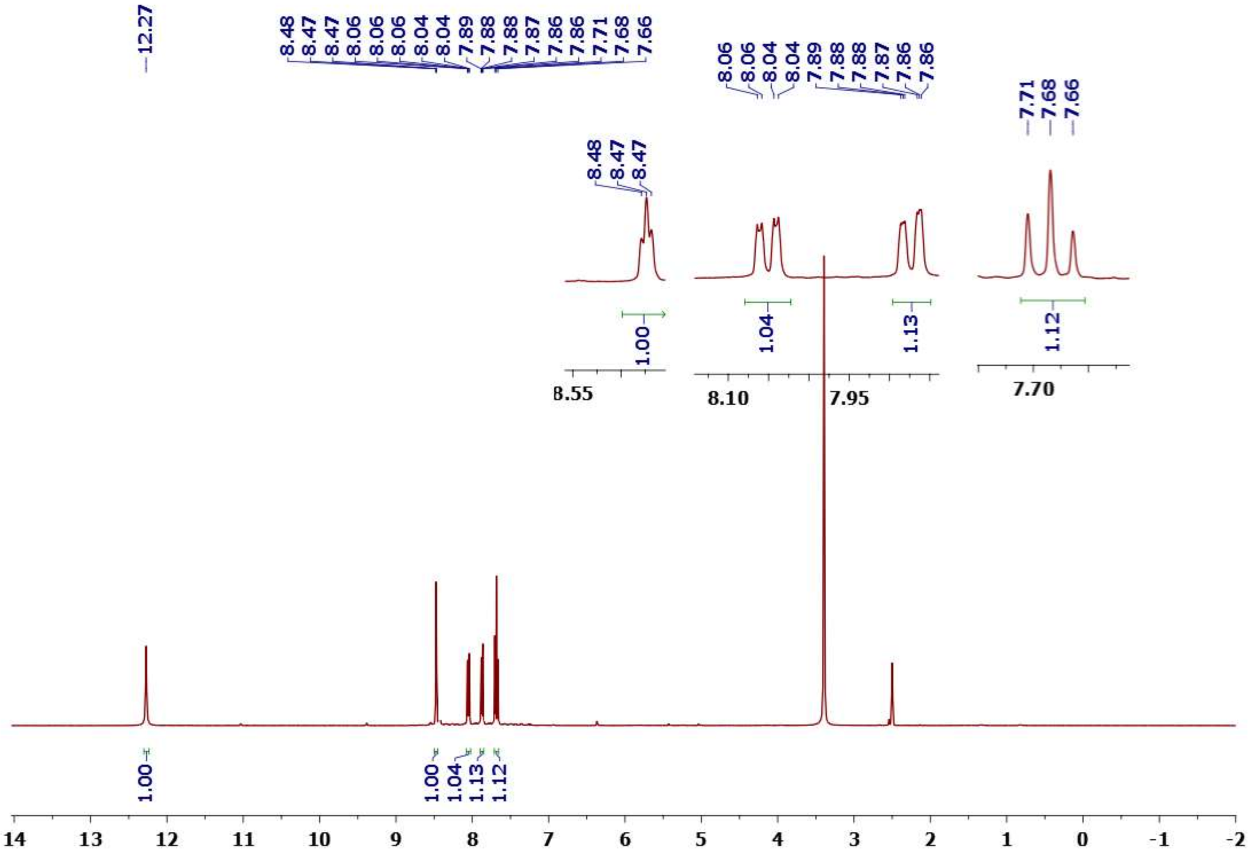

^{13}C NMR (DMSO-d6) spectrum of (3-nitrophenyl)carbamoyl cyanide (2h)

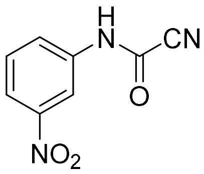

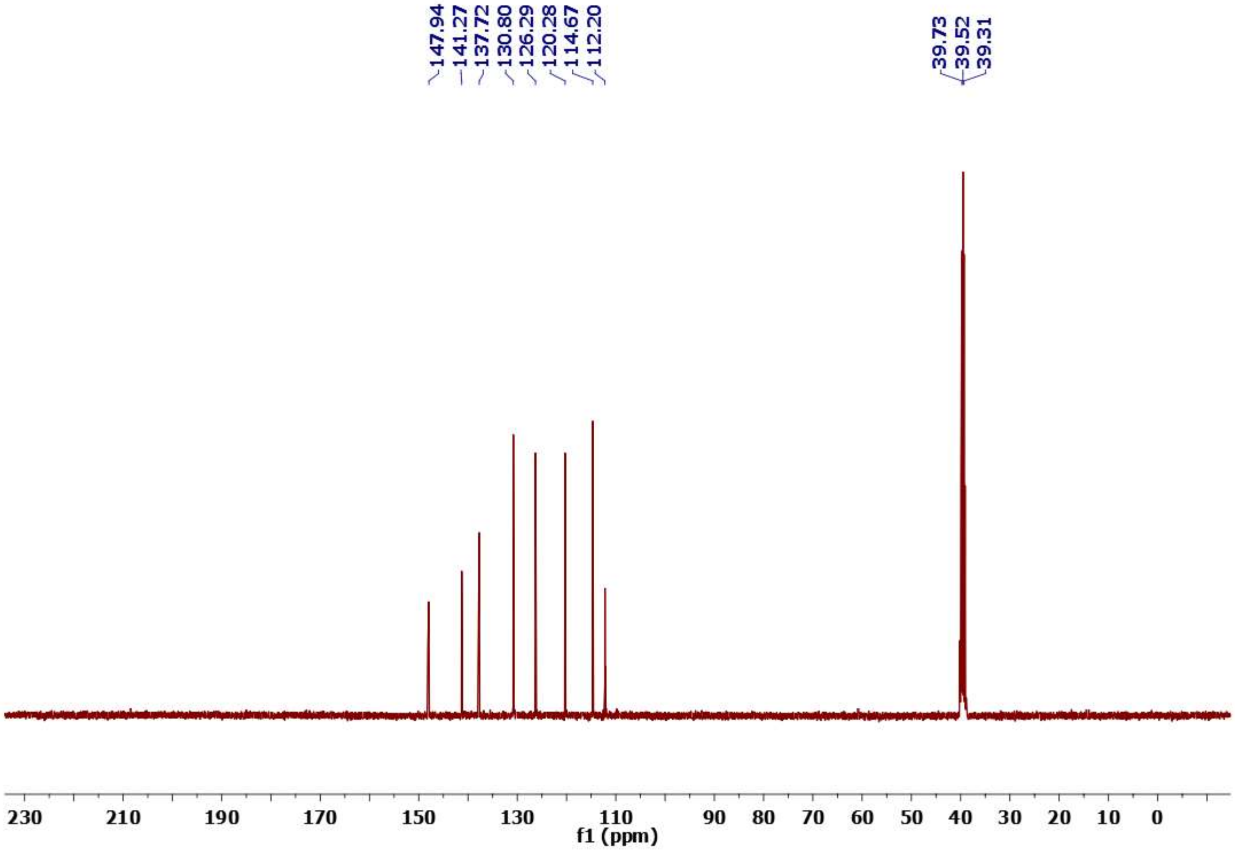

^{13}C CRAPT NMR (DMSO-d6) spectrum of (3-nitrophenyl)carbamoyl cyanide (2h)

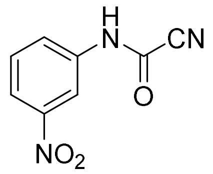

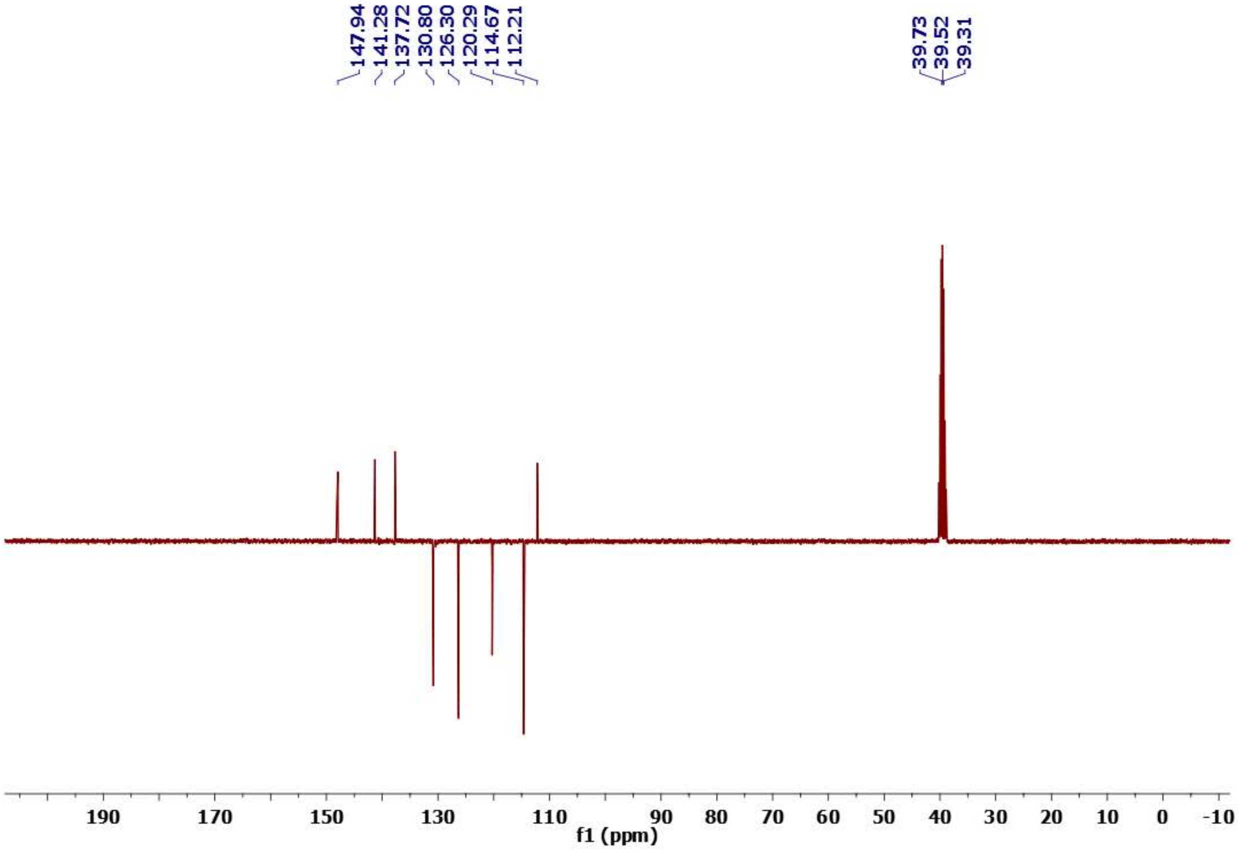

^{1}H NMR (DMSO-d6) spectrum of (4-methoxyphenyl)carbamoyl cyanide (2i)

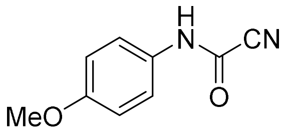

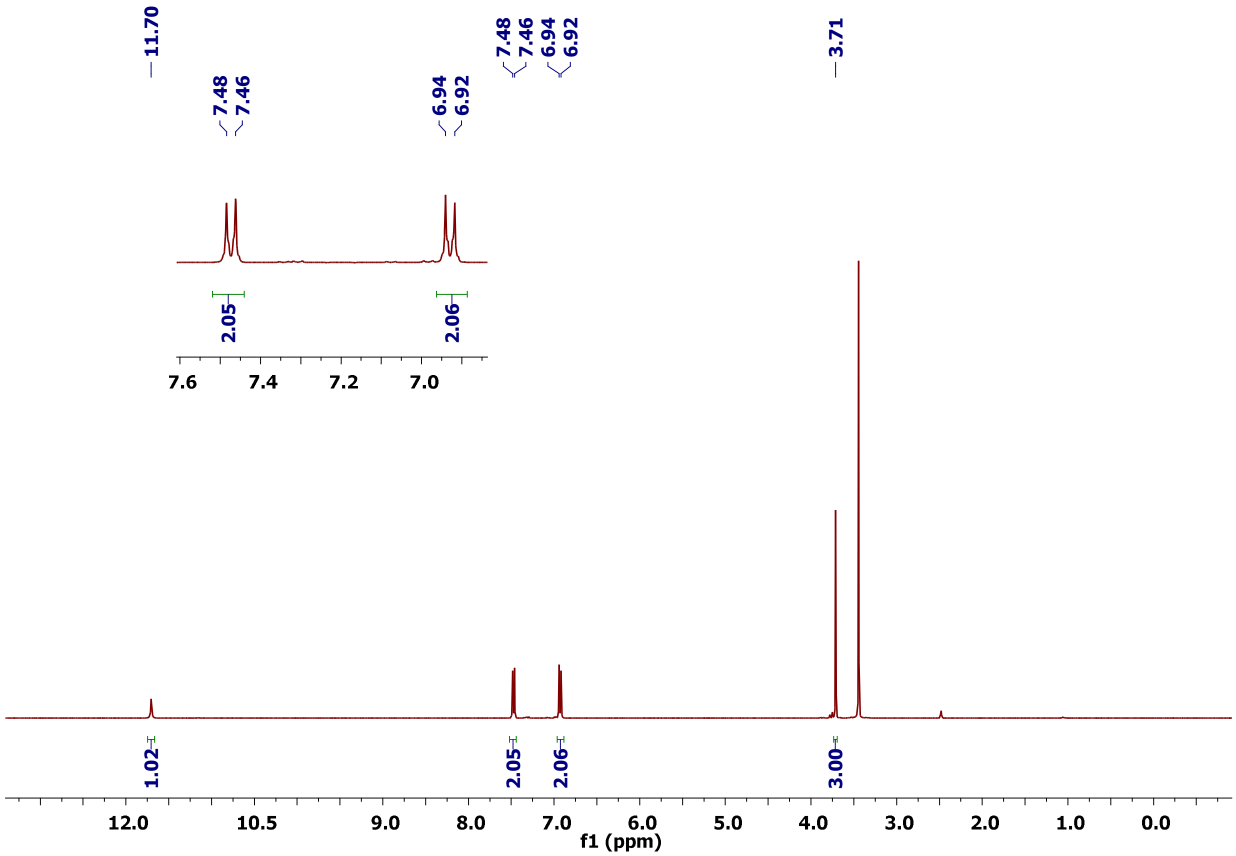

^{13}C NMR (DMSO-d6) spectrum of (4-methoxyphenyl)carbamoyl cyanide (2i)

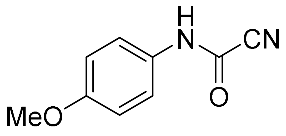

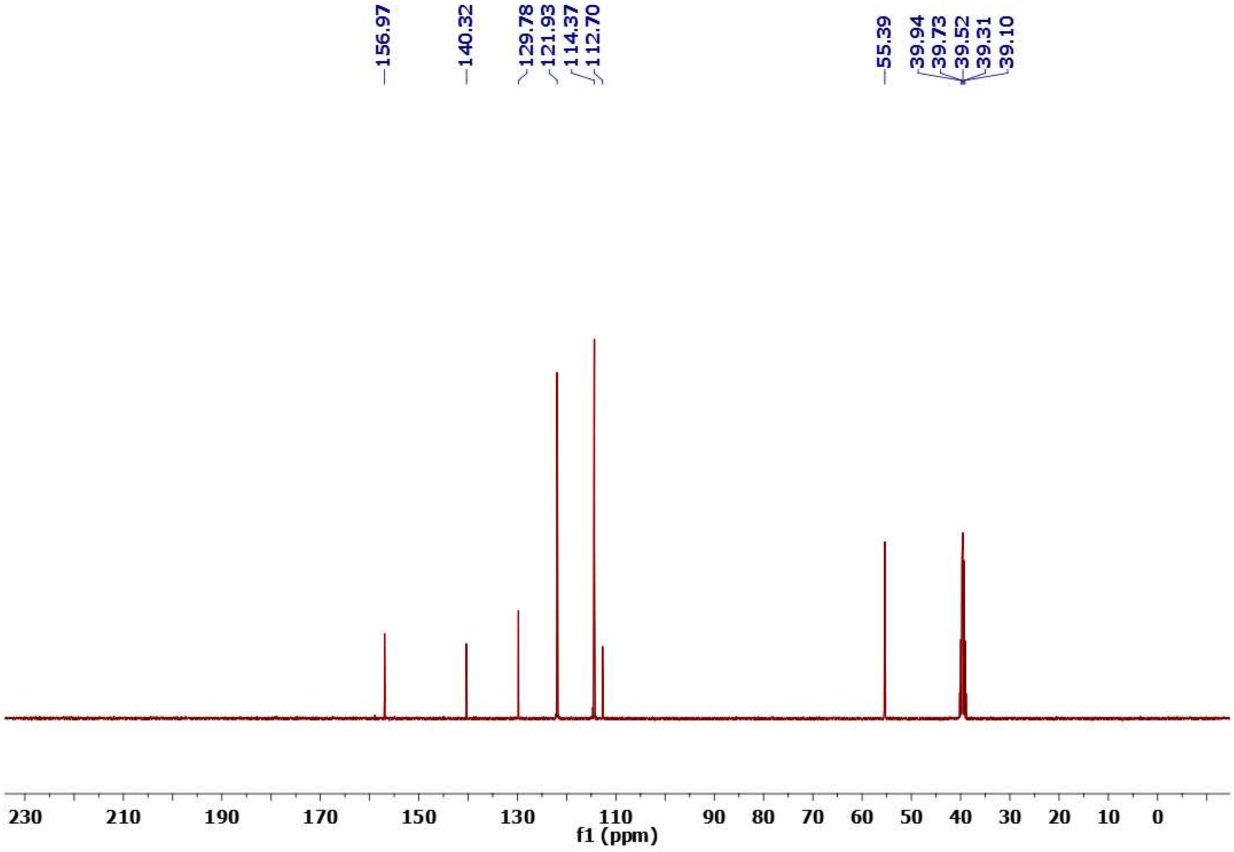

^{13}C CRAPT NMR (DMSO-d6) spectrum of (4-methoxyphenyl)carbamoyl cyanide (2i)

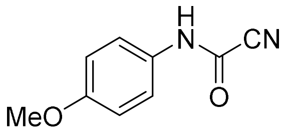

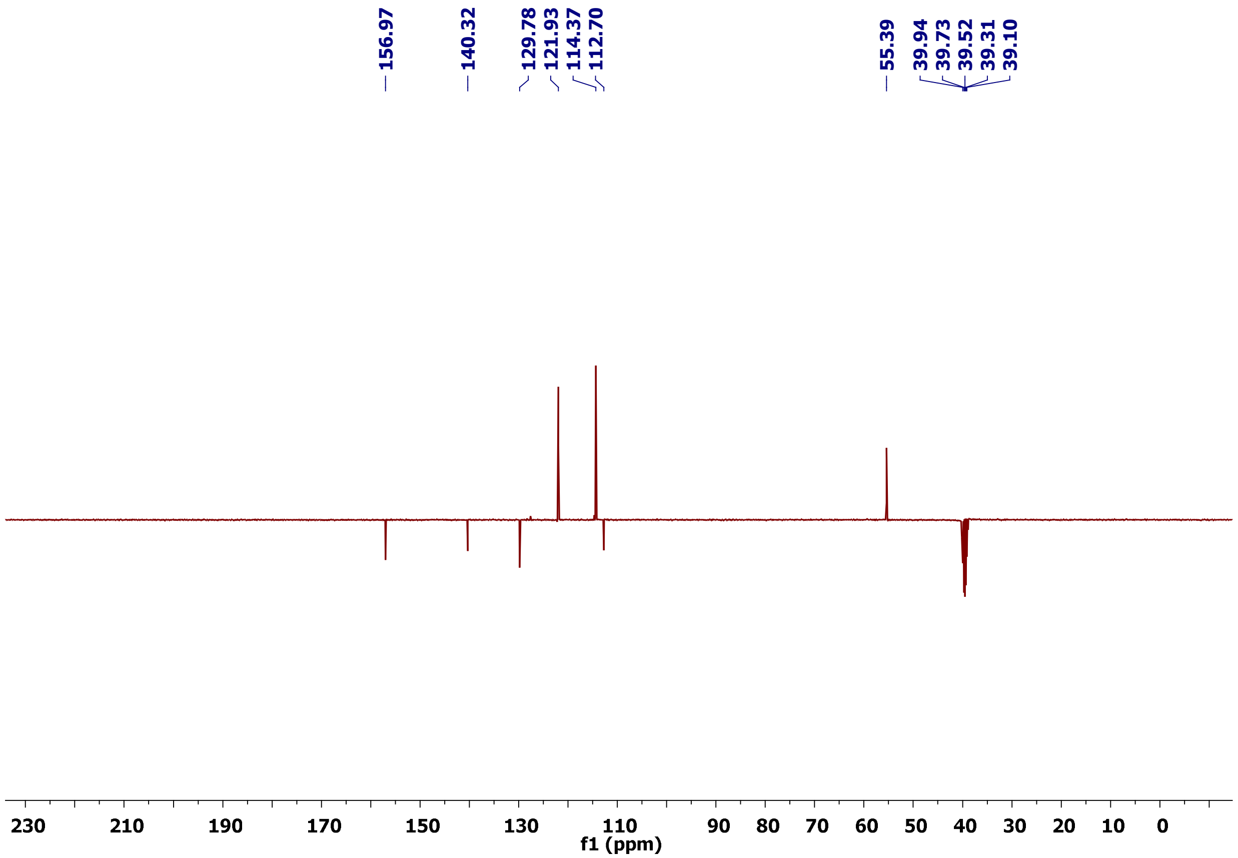

^{1}H-^{1}H gDQCOSY NMR (DMSO-d6) spectrum of (4-methoxyphenyl)carbamoyl cyanide (2i)

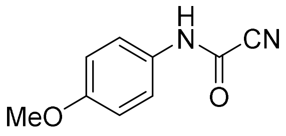

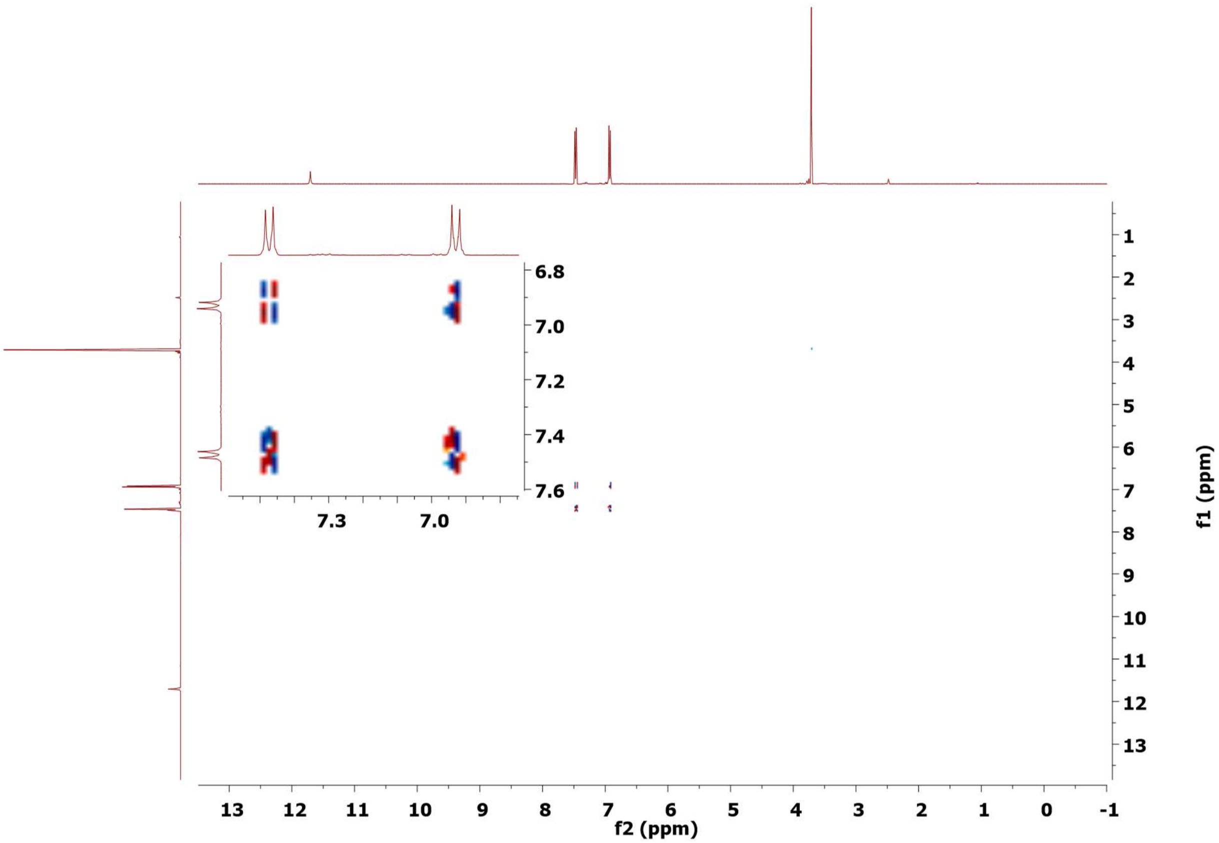

^1H-^{13}C-gHSQC NMR (DMSO-d6) spectrum of (4-methoxyphenyl)carbamoyl cyanide (2i)

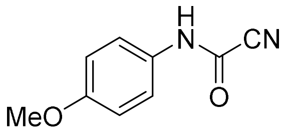

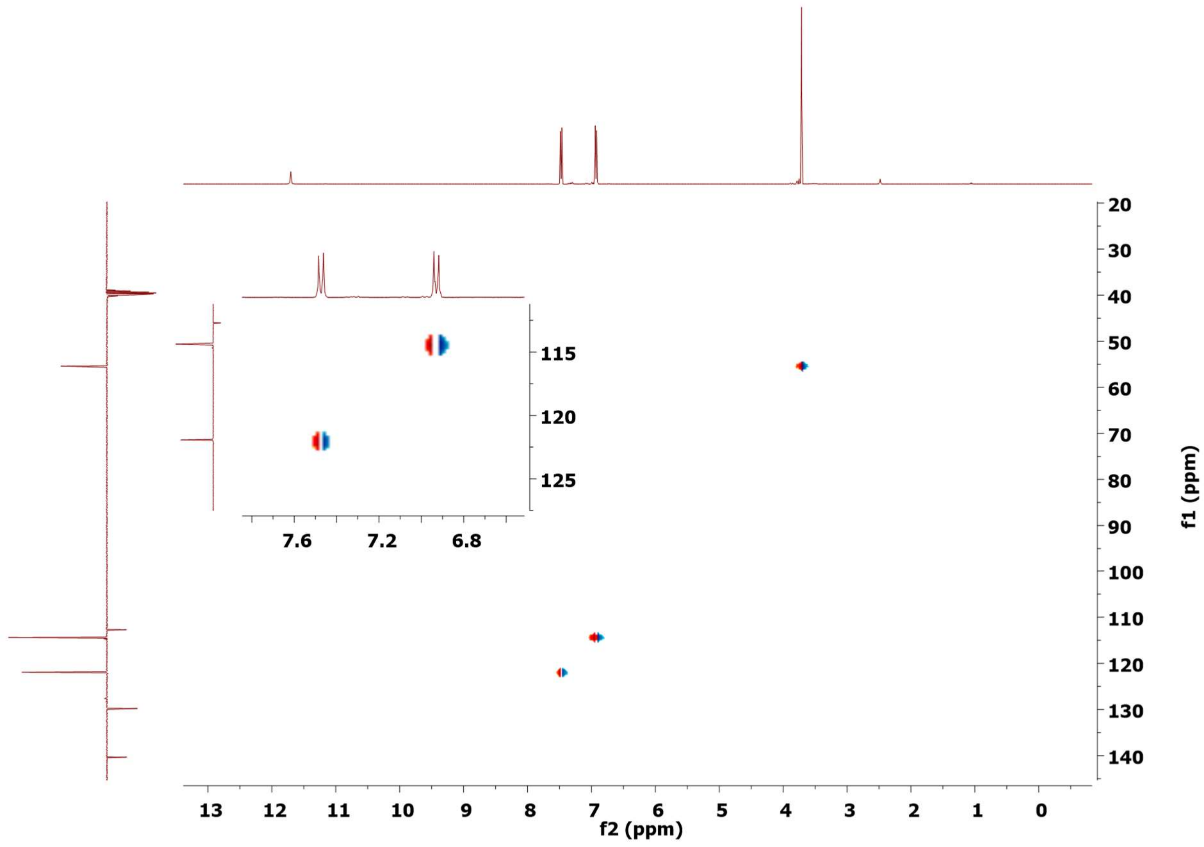

^{1}H-^{13}C-gHMBC NMR (DMSO-d6) spectrum of (4-methoxyphenyl)carbamoyl cyanide (2i)

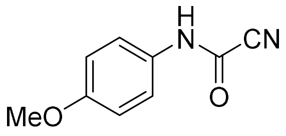

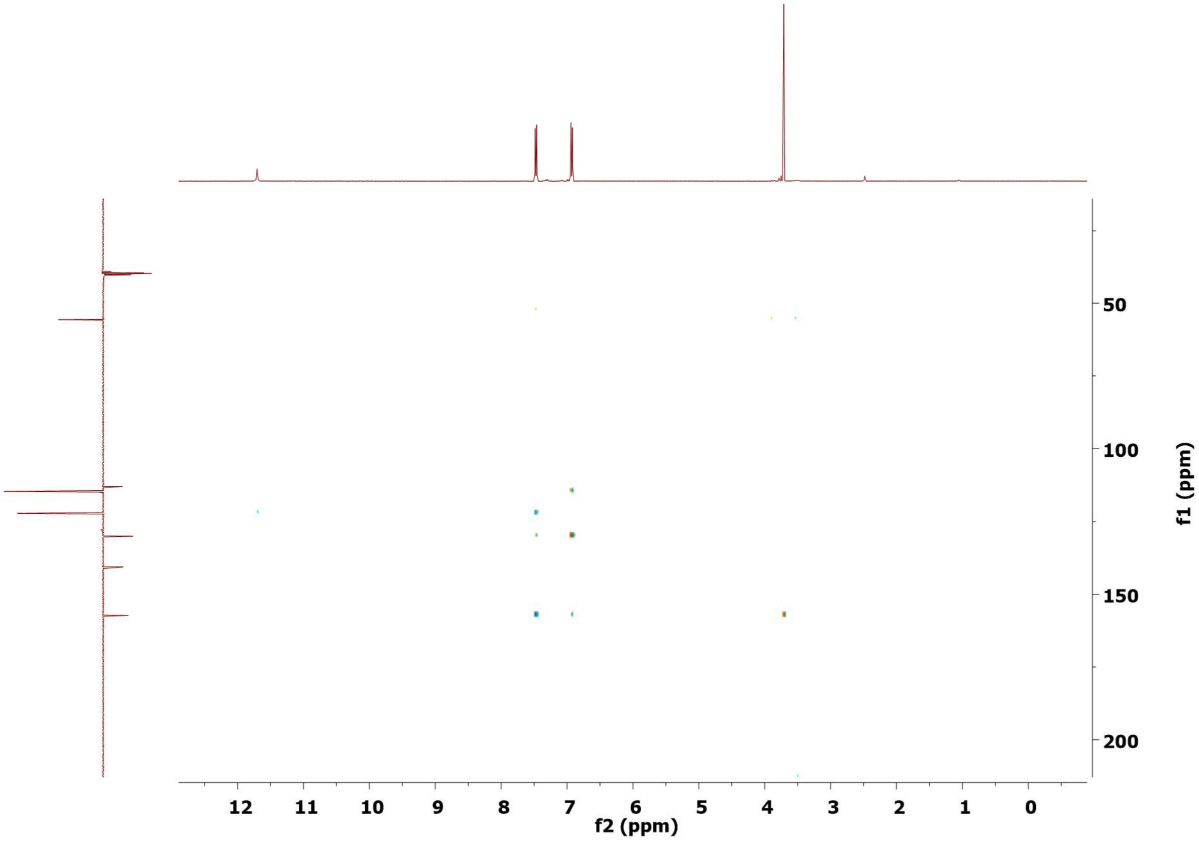

^{1}H NMR (DMSO-d6) spectrum of (4-ethoxyphenyl)carbamoyl cyanide (2j)

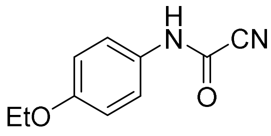

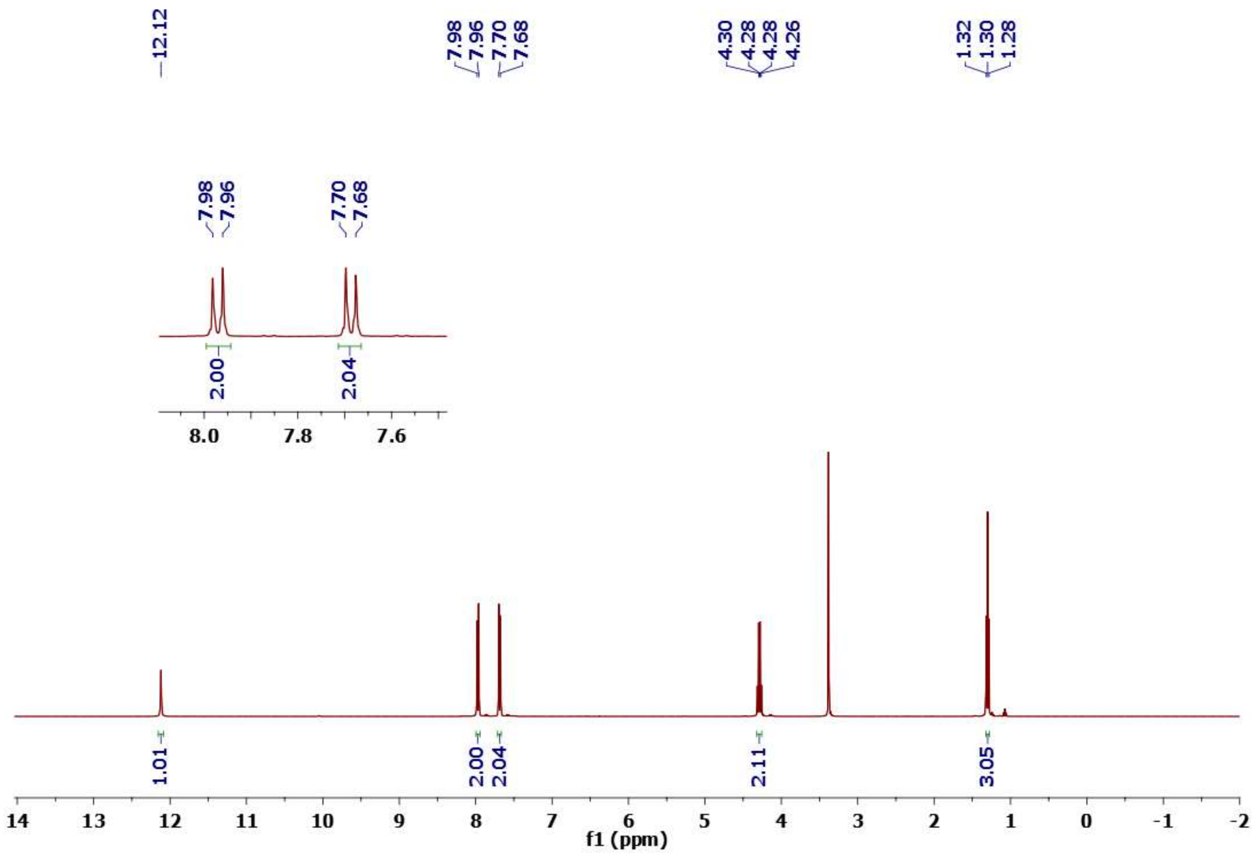

^{13}C NMR (DMSO-d6) spectrum of (4-ethoxyphenyl)carbamoyl cyanide (2j)

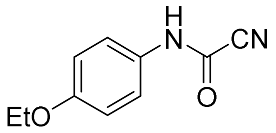

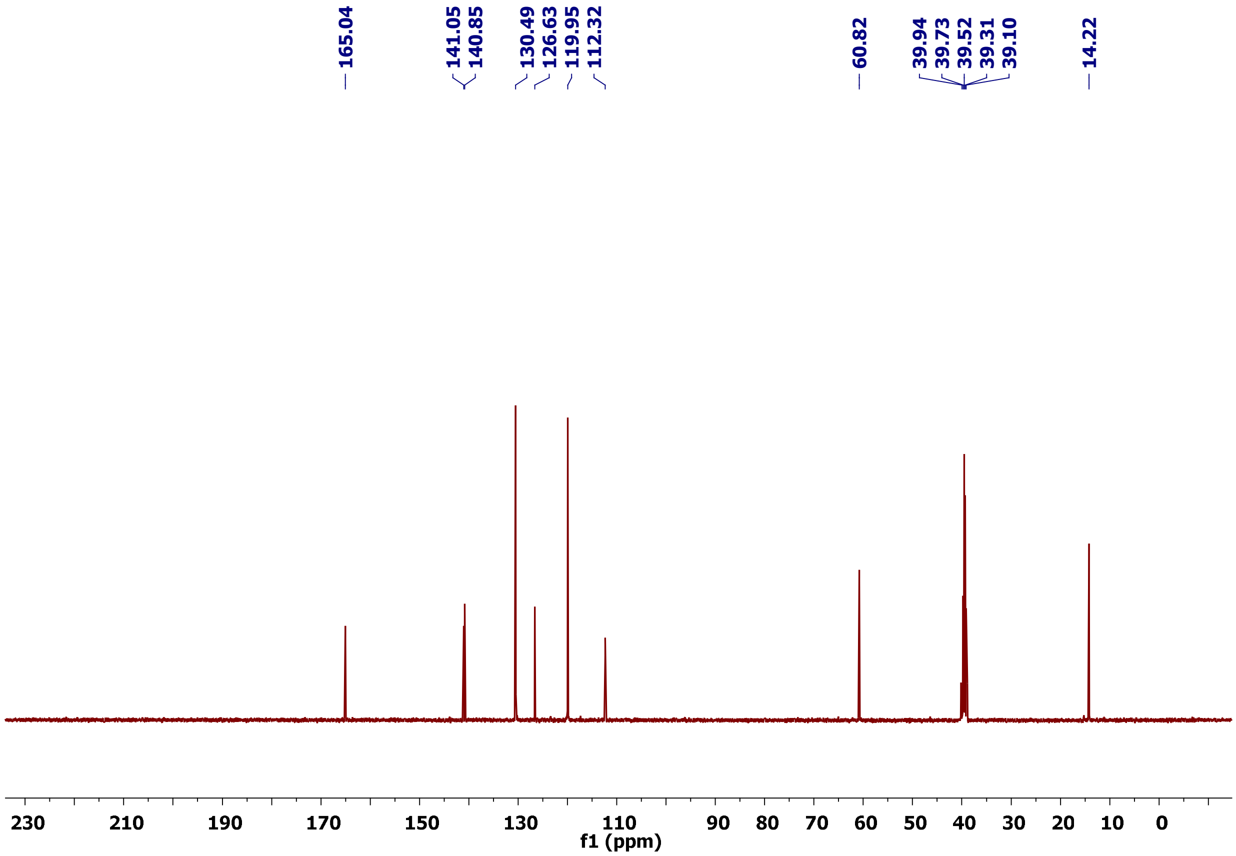

^{13}C CRAPT NMR (DMSO-d6) spectrum of (4-ethoxyphenyl)carbamoyl cyanide (2j)

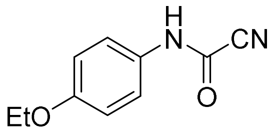

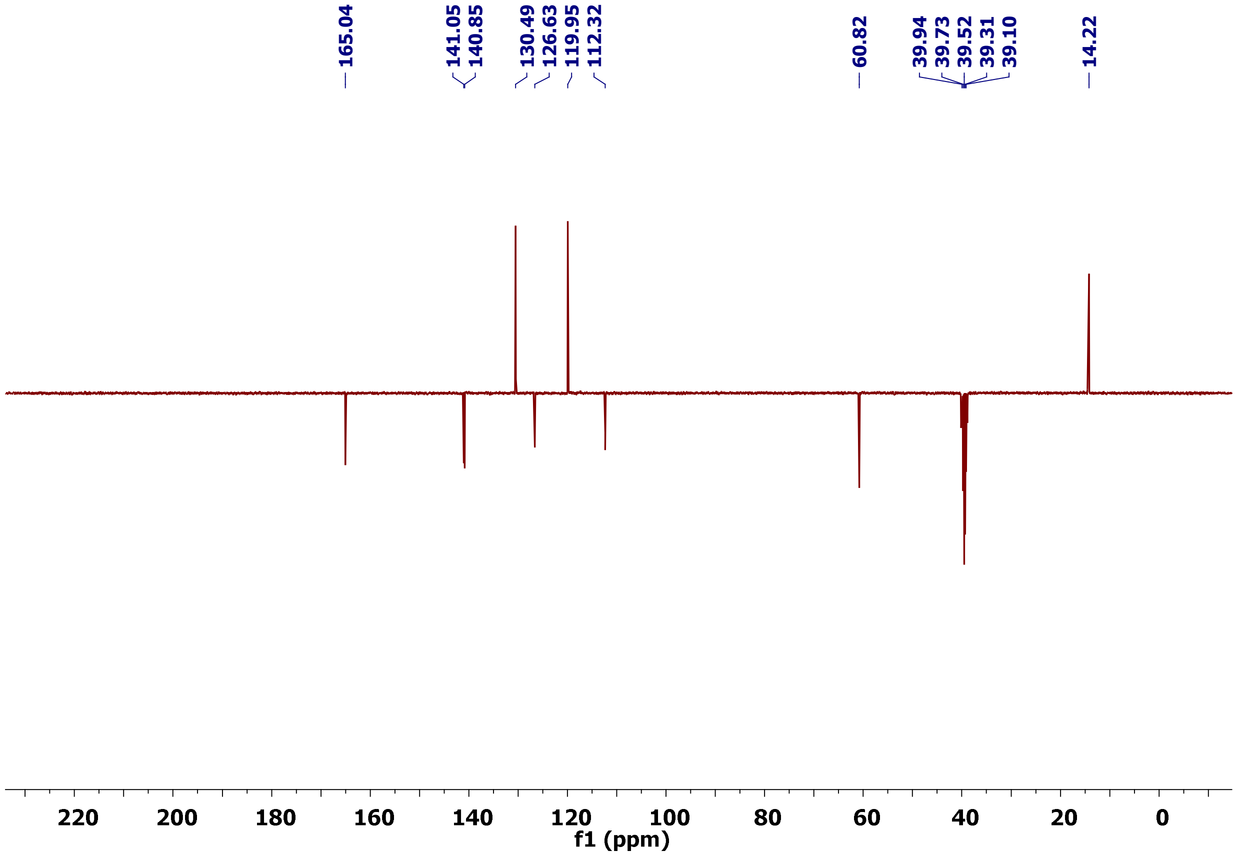

^{1}H-^{1}H gDQCOSY NMR (DMSO-d6) spectrum of (4-ethoxyphenyl)carbamoyl cyanide (2j)

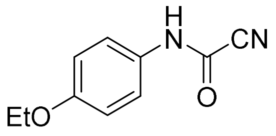

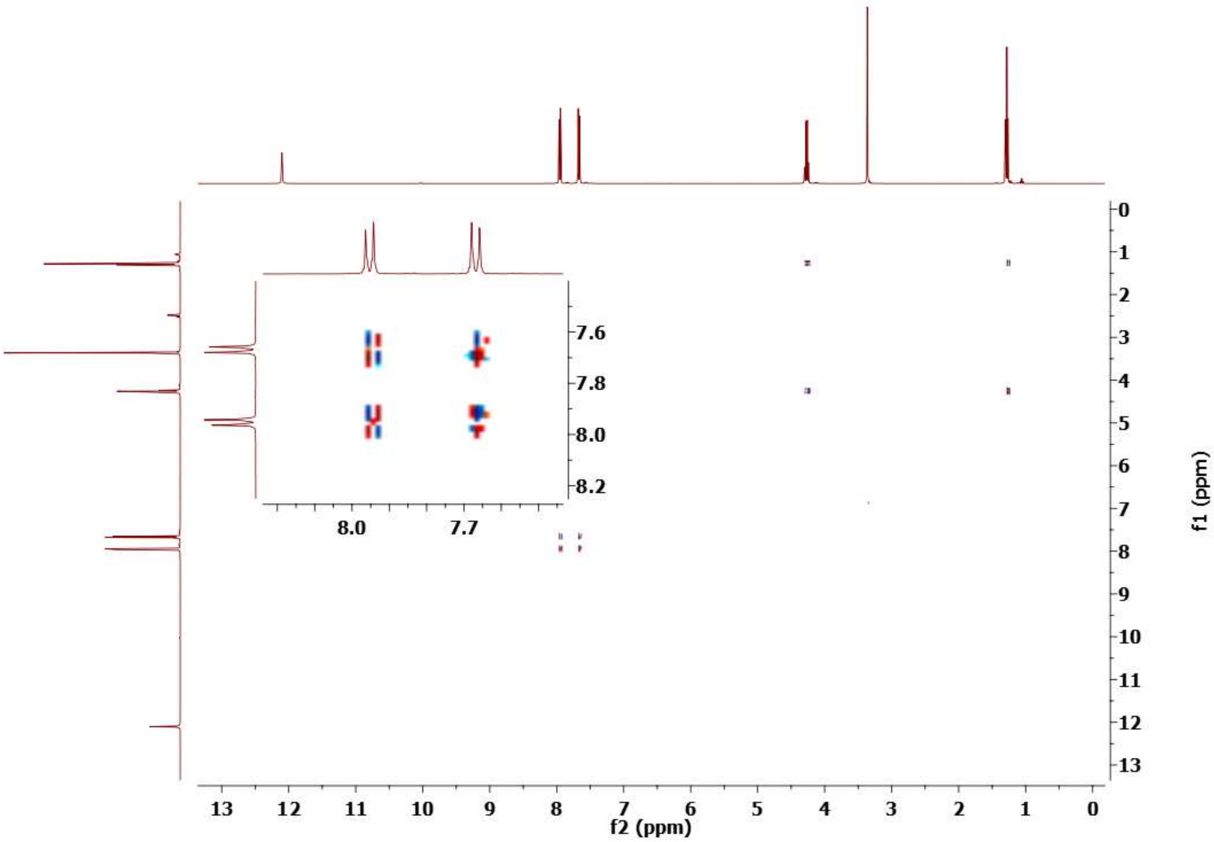

^{1}H-^{13}C-gHSQC NMR (DMSO-d6) spectrum of (4-ethoxyphenyl)carbamoyl cyanide (2j)

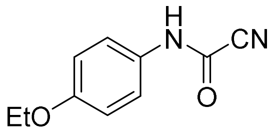

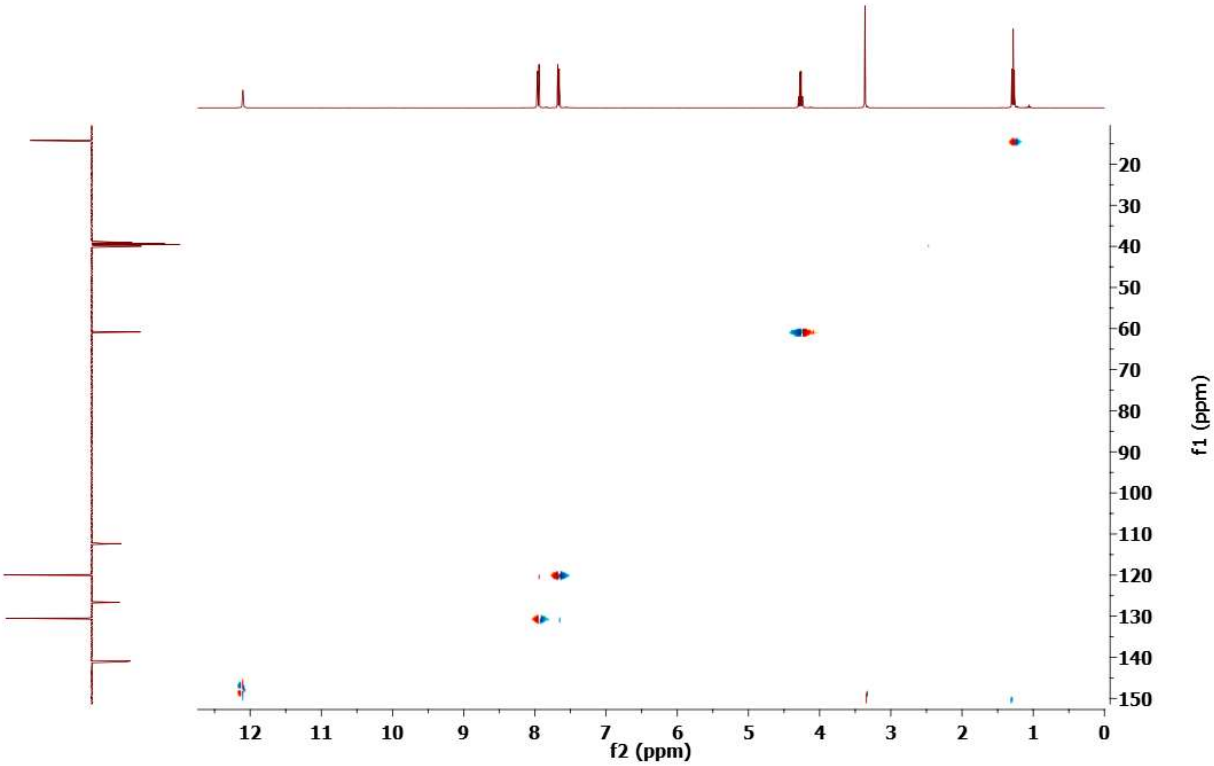

^{1}H-^{13}C-gHMBC NMR (DMSO-d6) spectrum of (4-ethoxyphenyl)carbamoyl cyanide (2j)

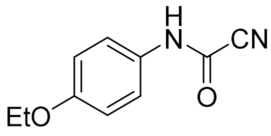

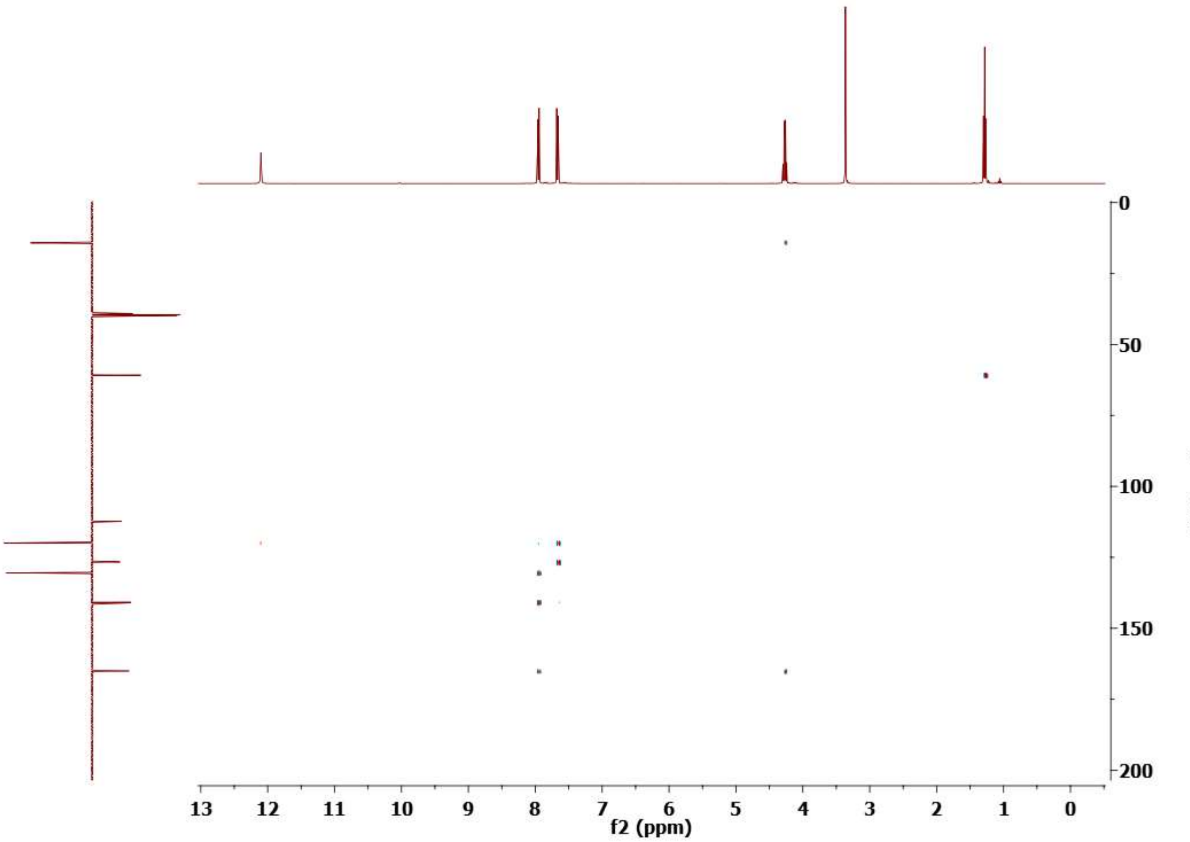

^{1}H NMR (DMSO-d6) spectrum of (4-(benzyloxy)phenyl)carbamoyl cyanide (2k)

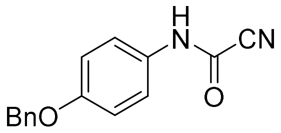

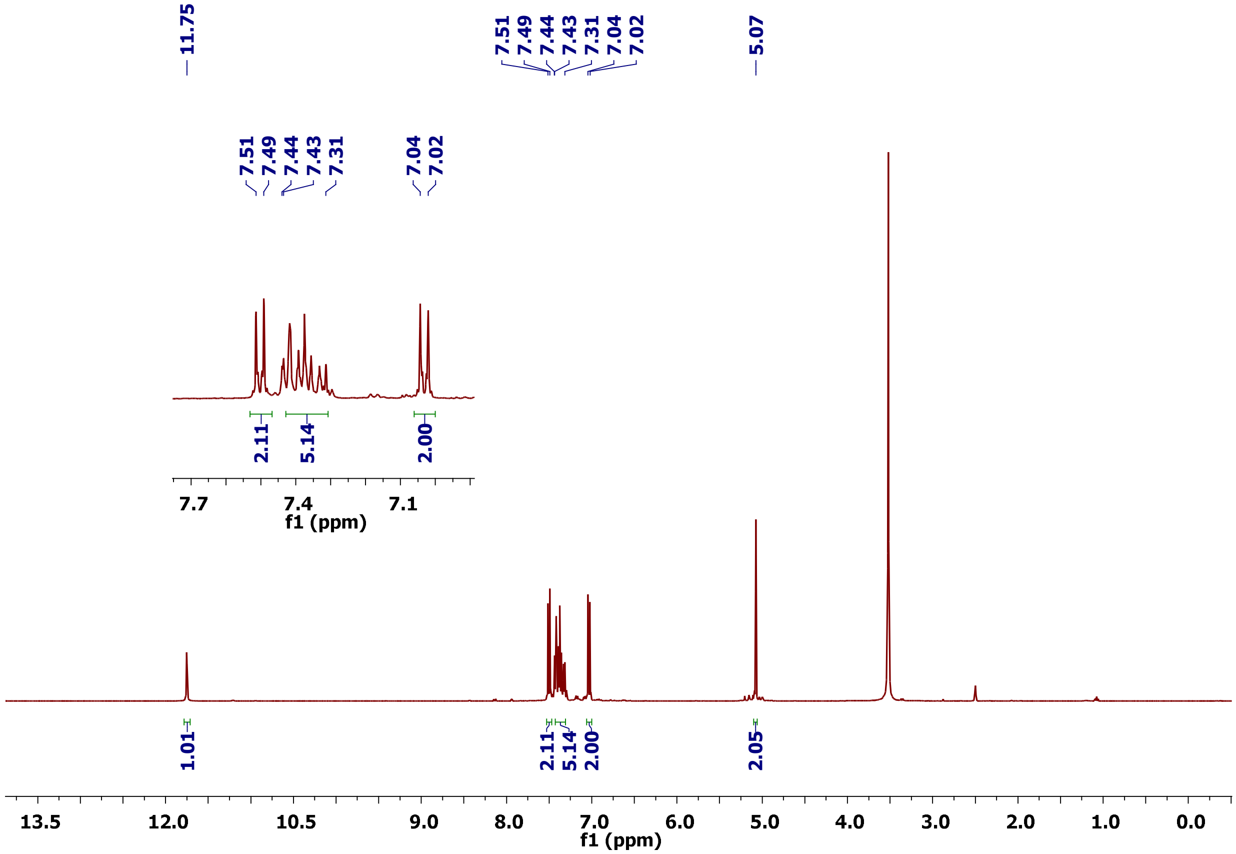

^{13}C NMR (DMSO-d6) spectrum of (4-(benzyloxy)phenyl)carbamoyl cyanide (2k)

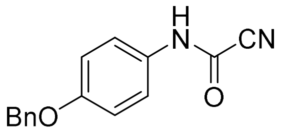

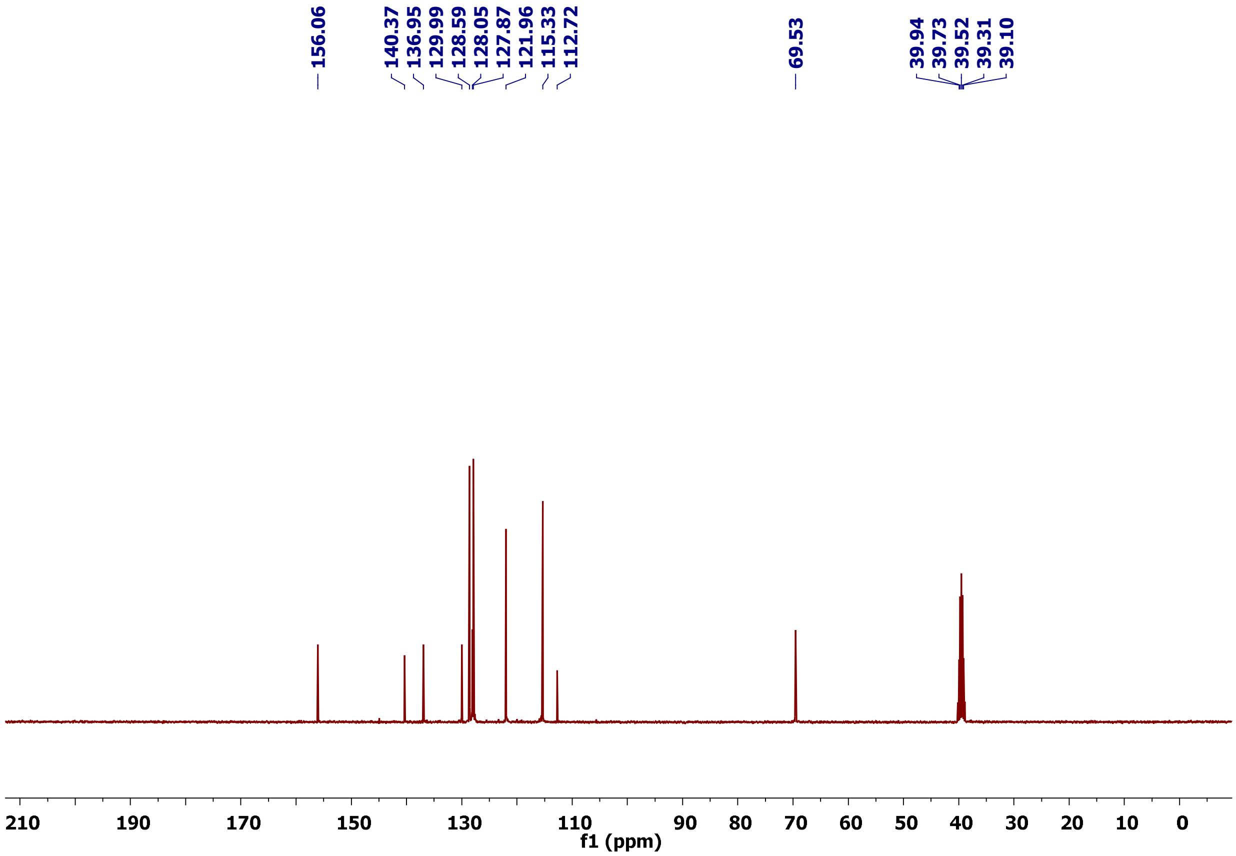

^{13}C CRAPT NMR (DMSO-d6) spectrum of (4-(benzyloxy)phenyl)carbamoyl cyanide (2k)

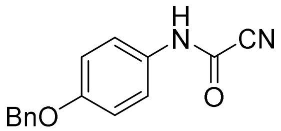

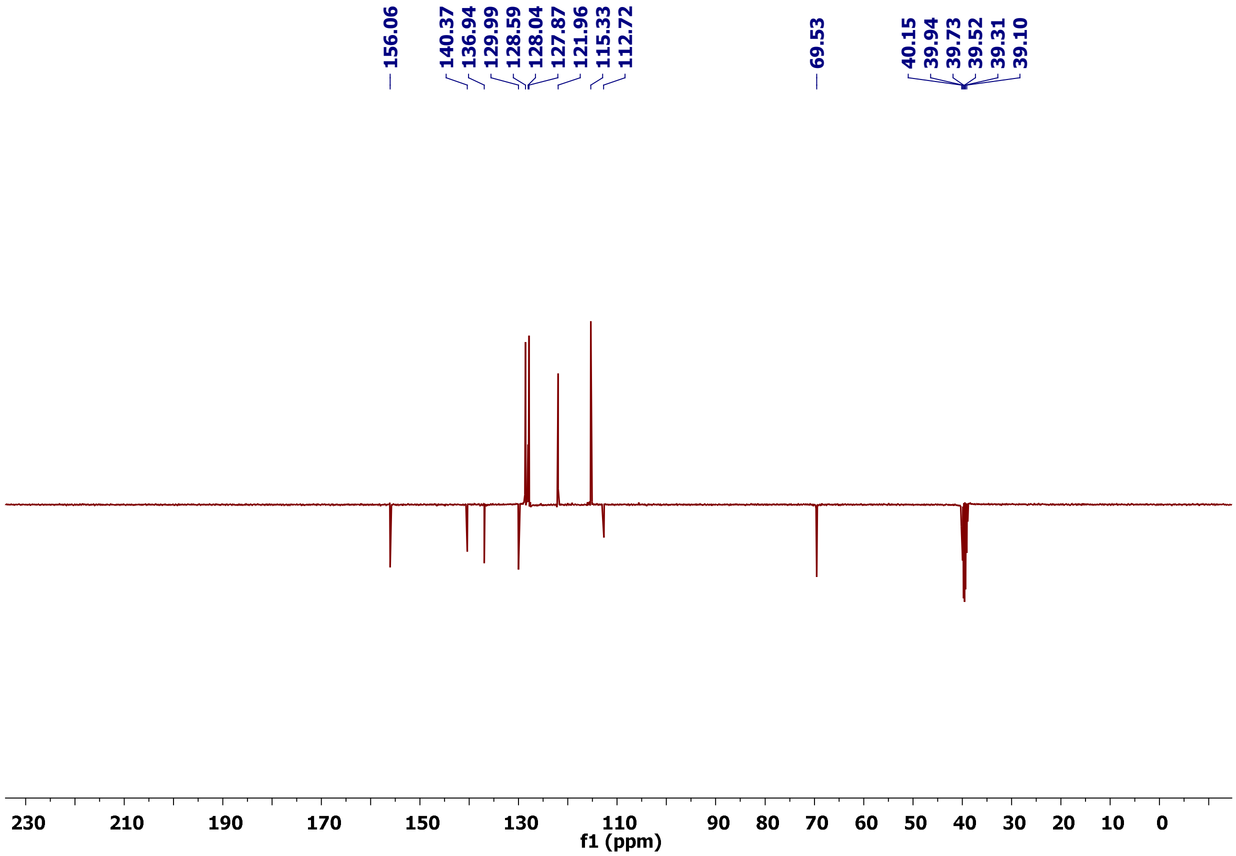

^{1}H-^{1}H gDQCOSY NMR (DMSO-d6) of (4-(benzyloxy)phenyl)carbamoyl cyanide (2k)

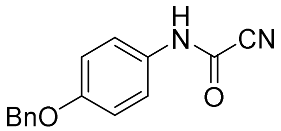

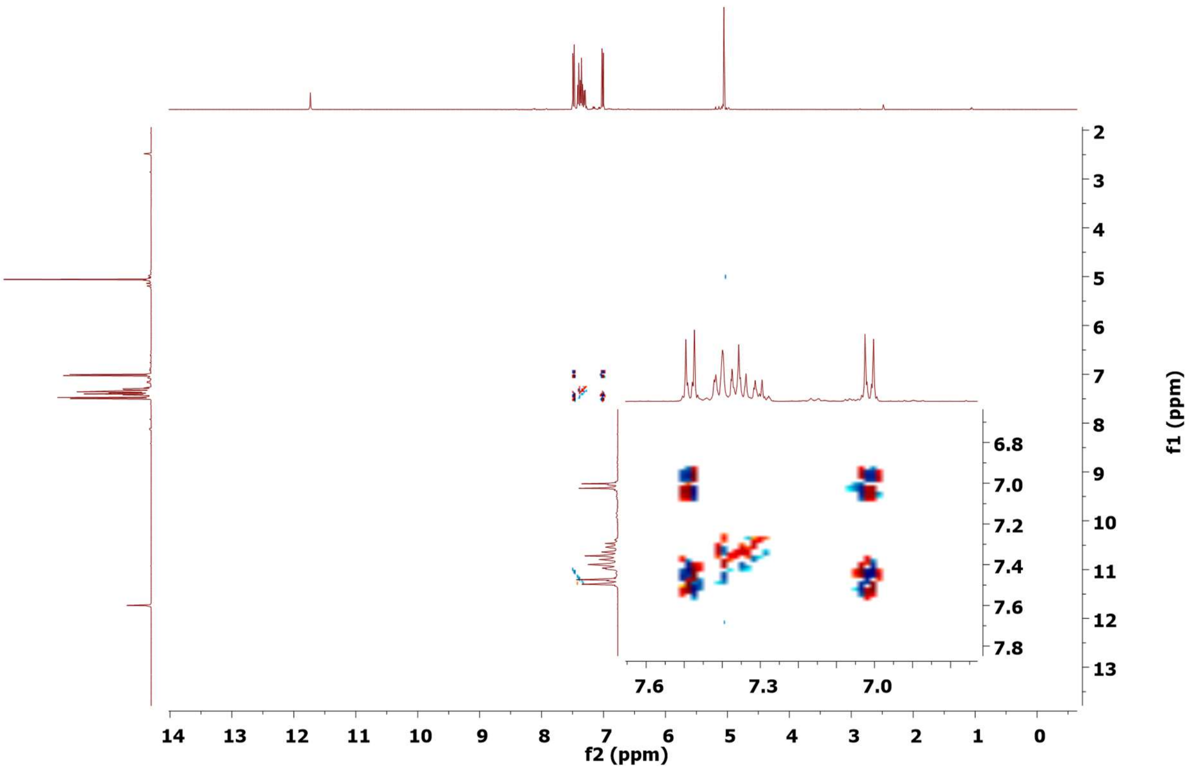

^{1}H-^{13}C-gHSQC NMR (DMSO-d6) spectrum of (4-(benzyloxy)phenyl)carbamoyl cyanide (2k)

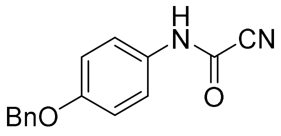

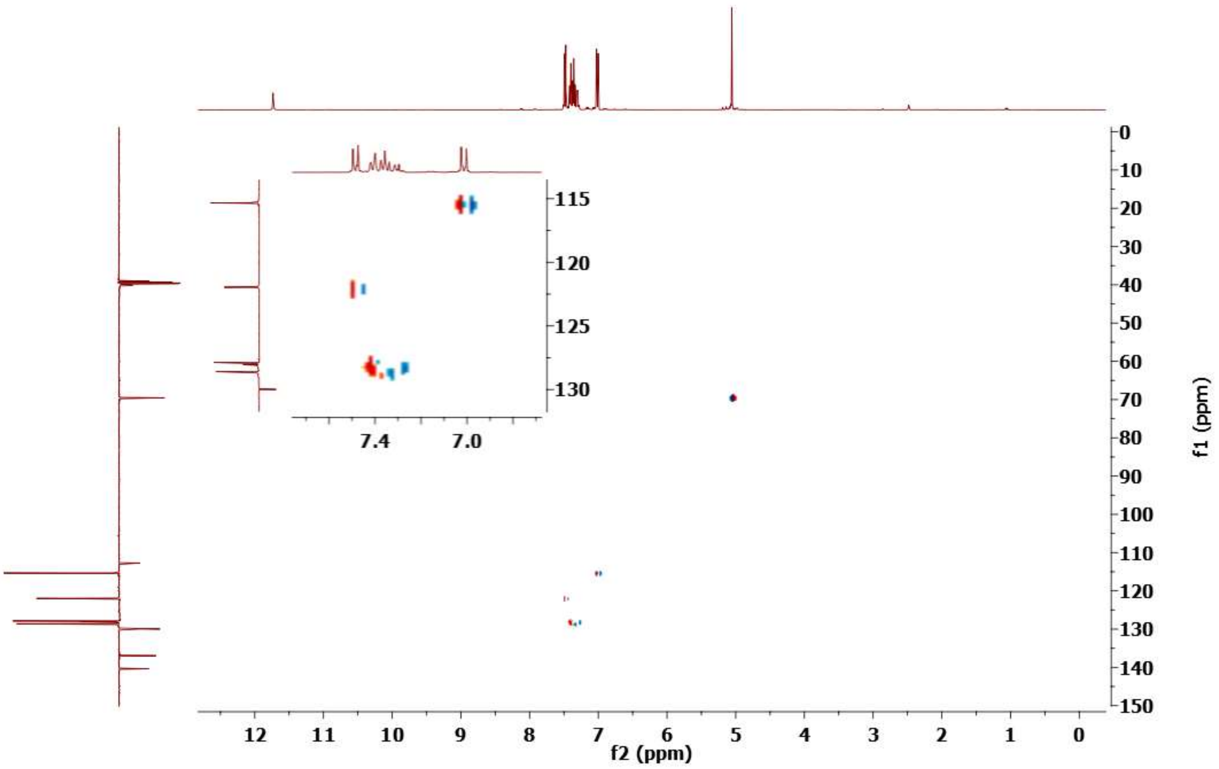

^{1}H-^{13}C-gHMBC NMR (DMSO-d6) spectrum of (4-(benzyloxy)phenyl)carbamoyl cyanide (2k)

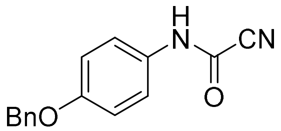

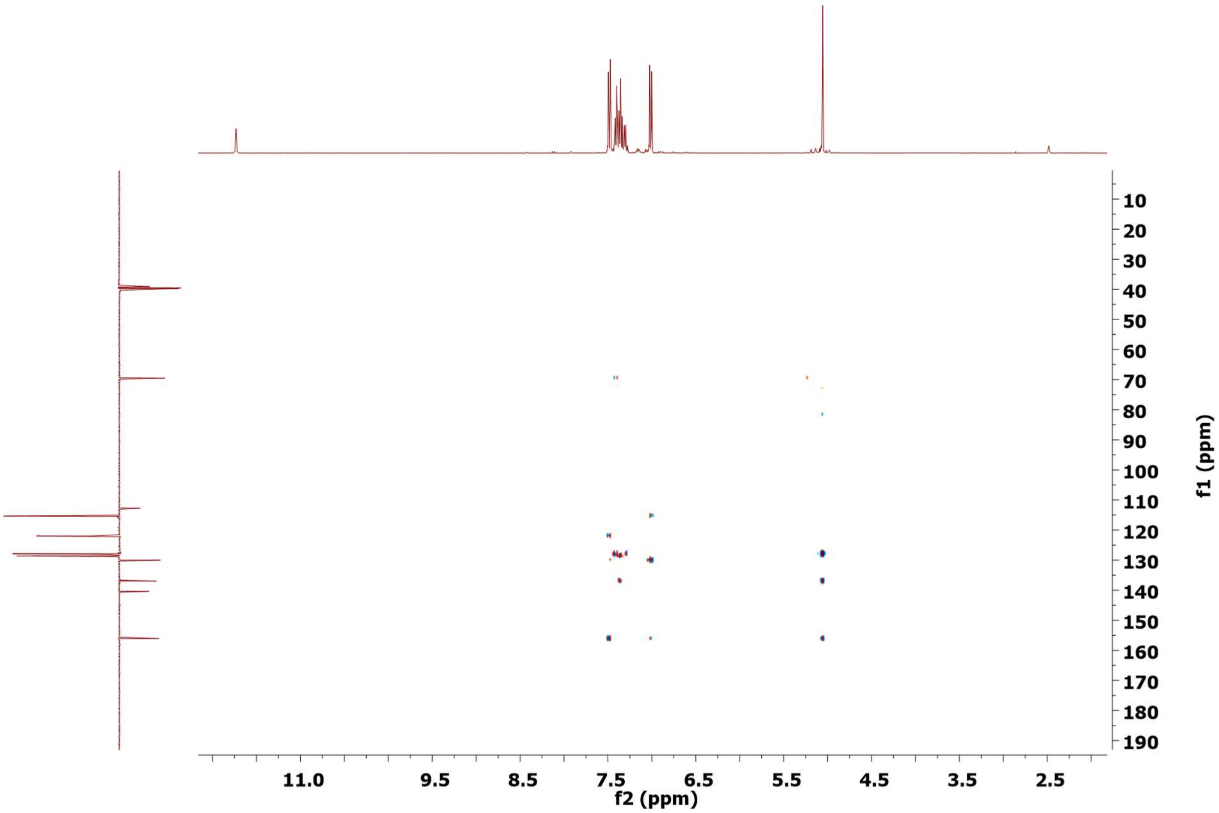

^{1}H NMR (DMSO-d6) spectrum of (4-(methylthio)phenyl)carbamoyl cyanide (2l)

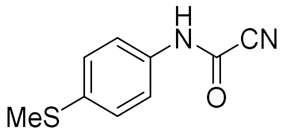

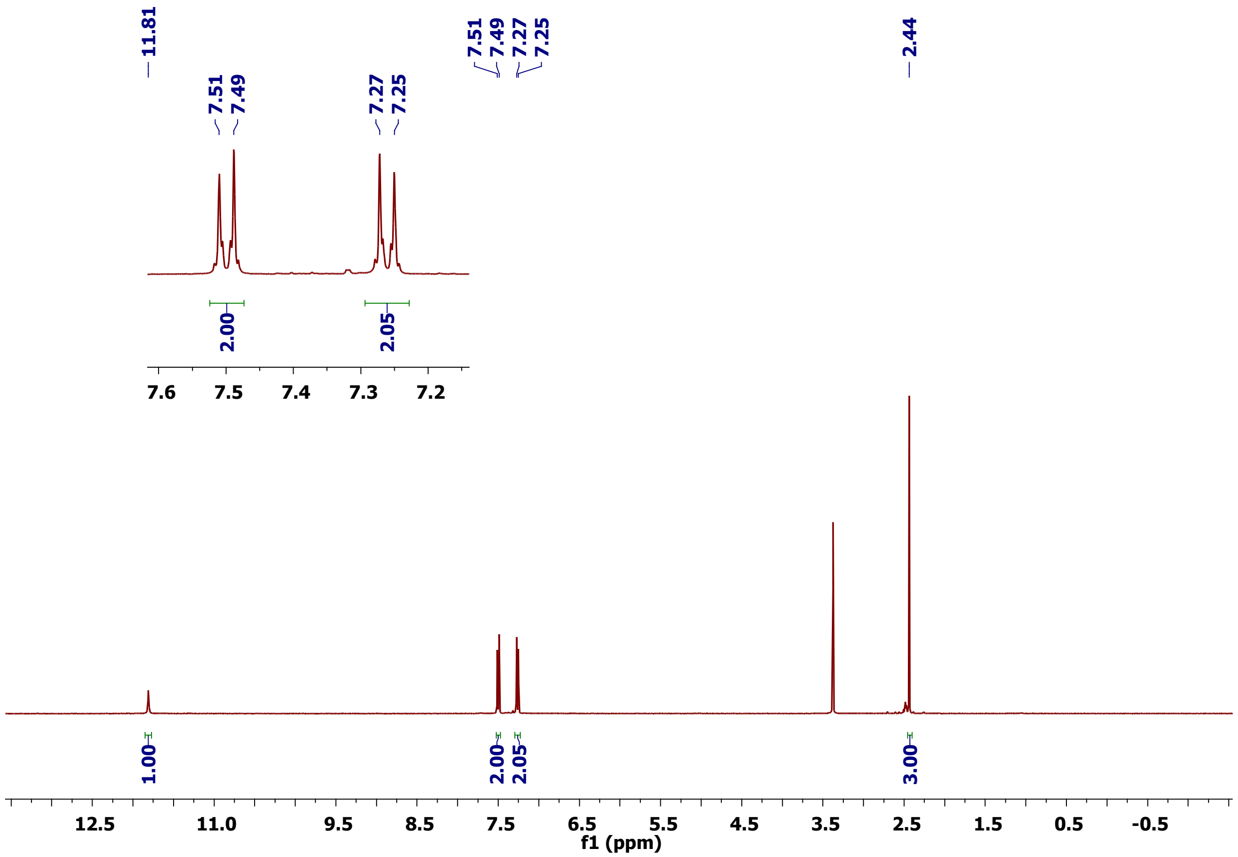

^{13}C NMR (DMSO-d6) spectrum of (4-(methylthio)phenyl)carbamoyl cyanide (2l)

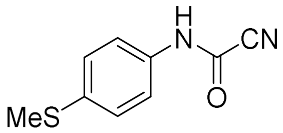

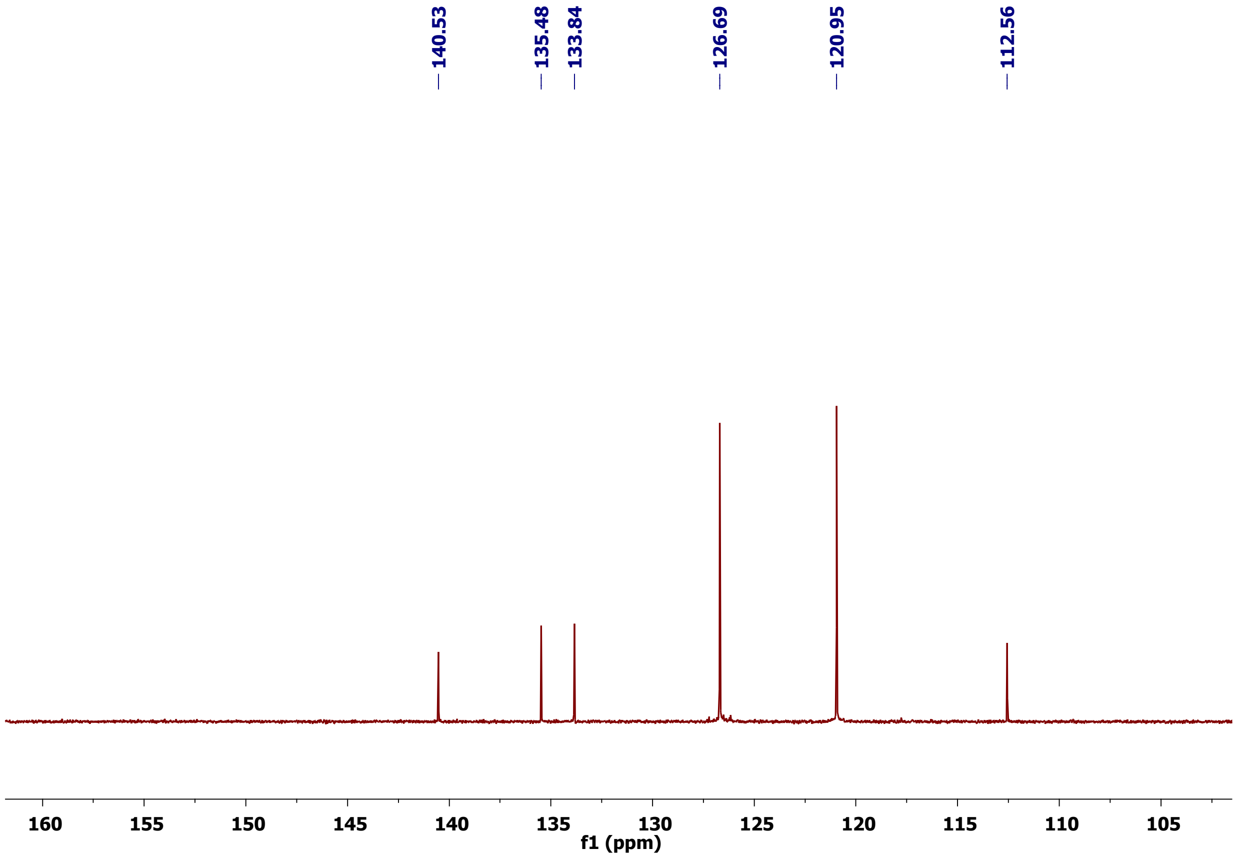

^{13}C CRAPT NMR (DMSO-d6) spectrum of (4-(methylthio)phenyl)carbamoyl cyanide (2l)

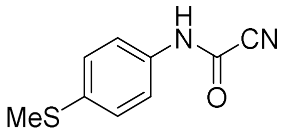

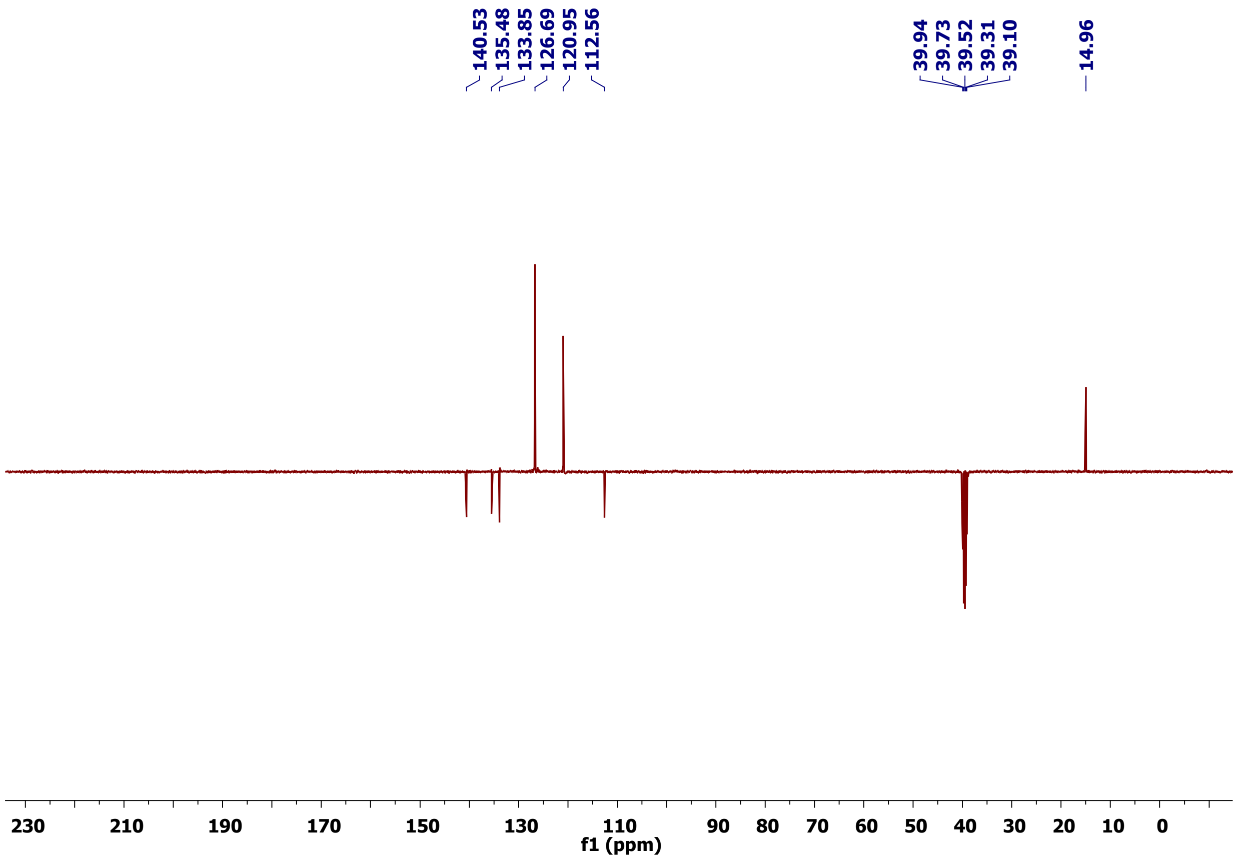

^{1}H-^{1}H gDQCOSY NMR (DMSO-d6) spectrum of (4-(methylthio)phenyl)carbamoyl cyanide (2l)

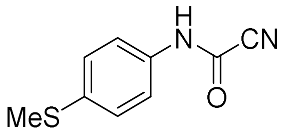

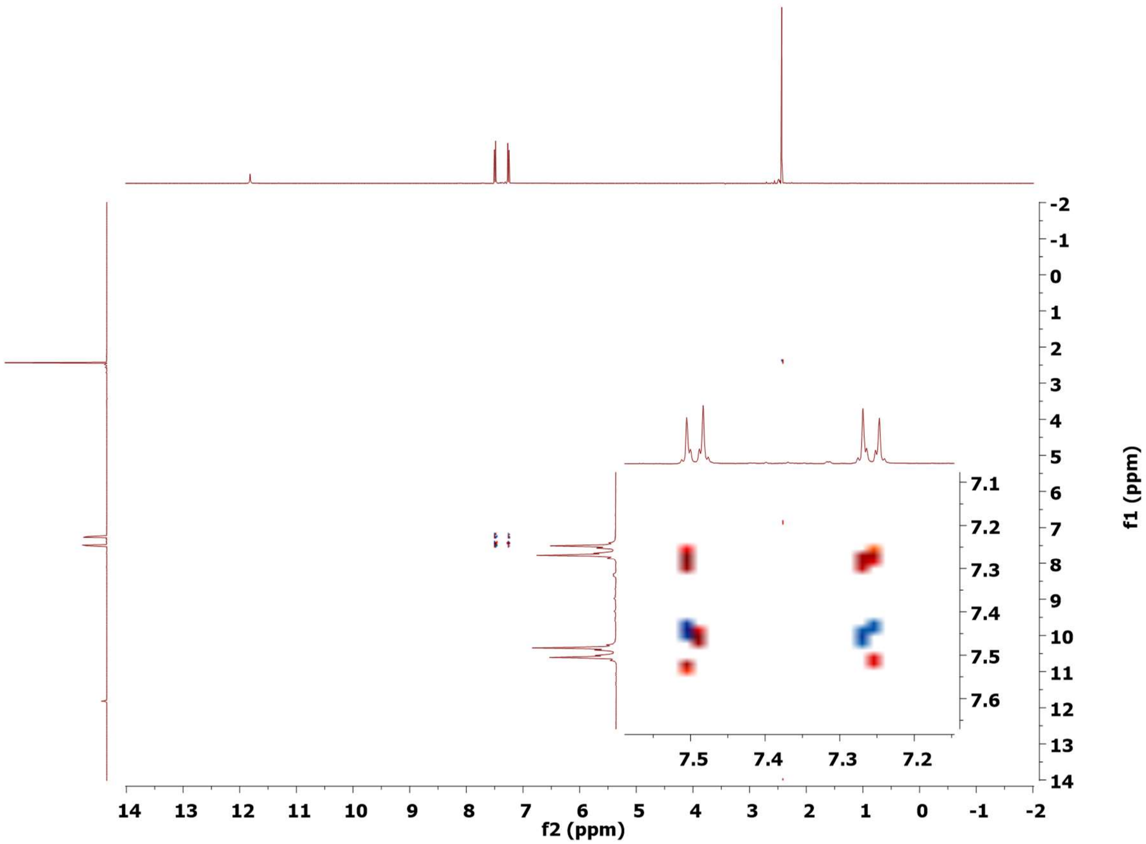

^{1}H-^{13}C-gHSQC NMR (DMSO-d6) spectrum of (4-(methylthio)phenyl)carbamoyl cyanide (2l)

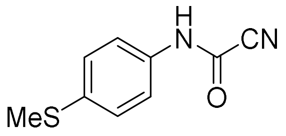

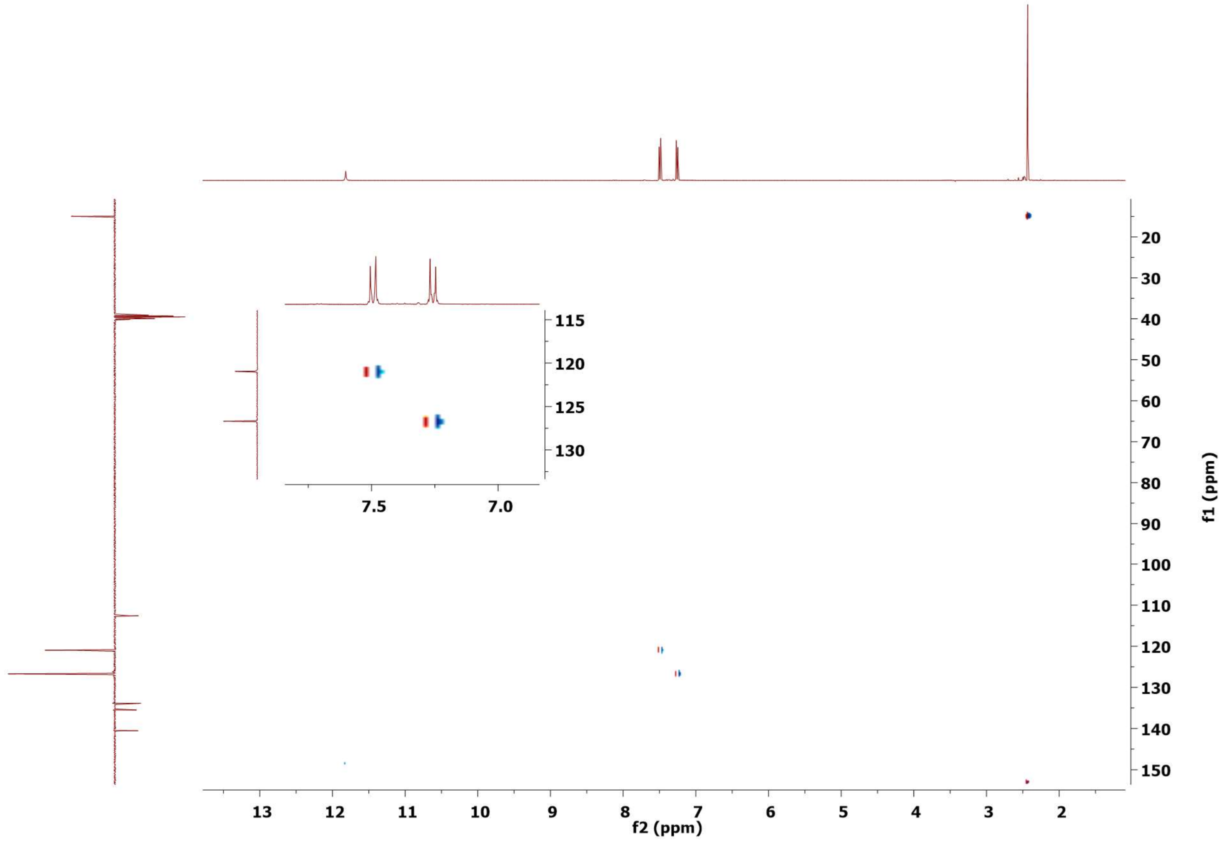

^1H-^{13}C-gHMBC NMR (DMSO-d6) spectrum of (4-(methylthio)phenyl)carbamoyl cyanide (2l)

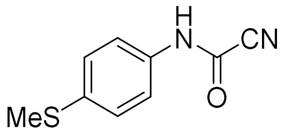

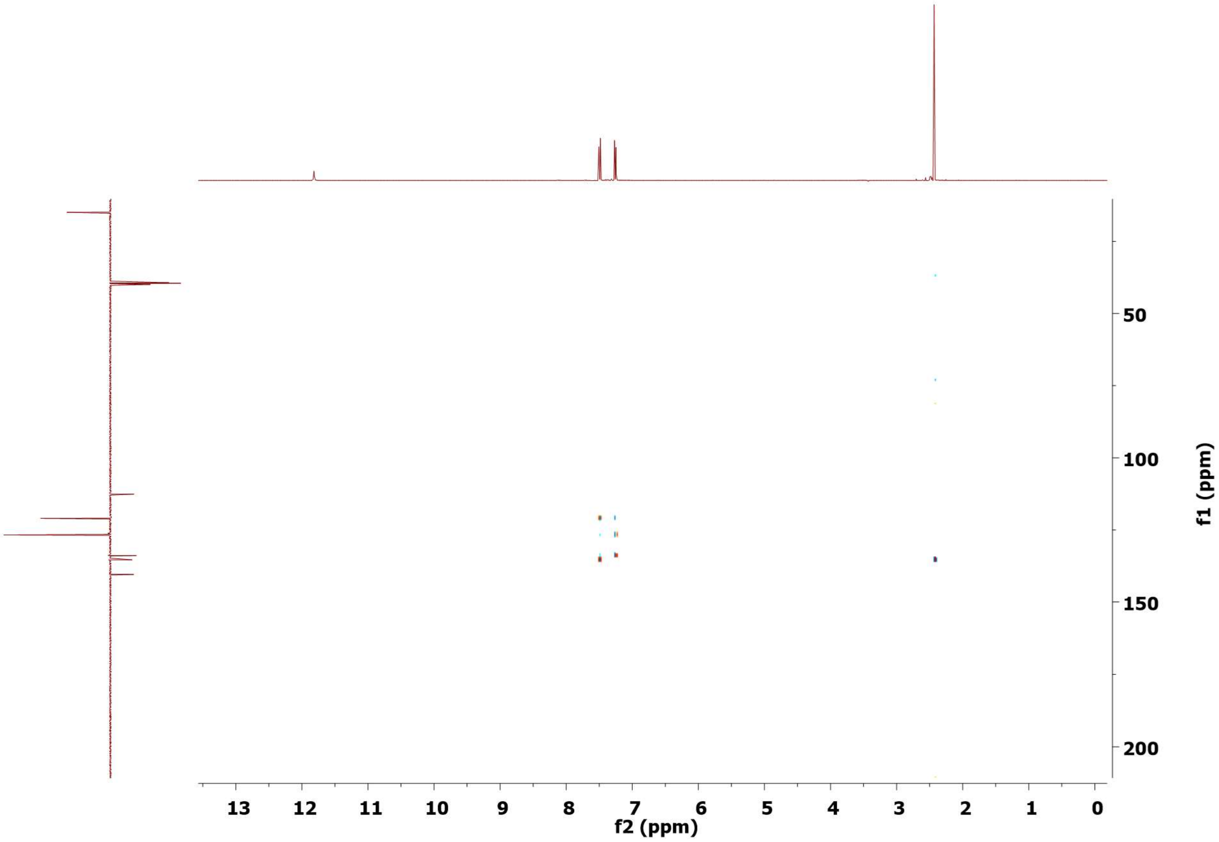

^{1}H NMR (DMSO-d6) spectrum of methyl 4-((cyanocarbonyl)amino)benzoate (2m)

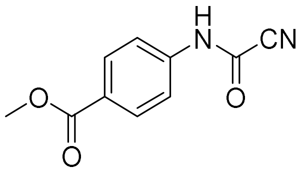

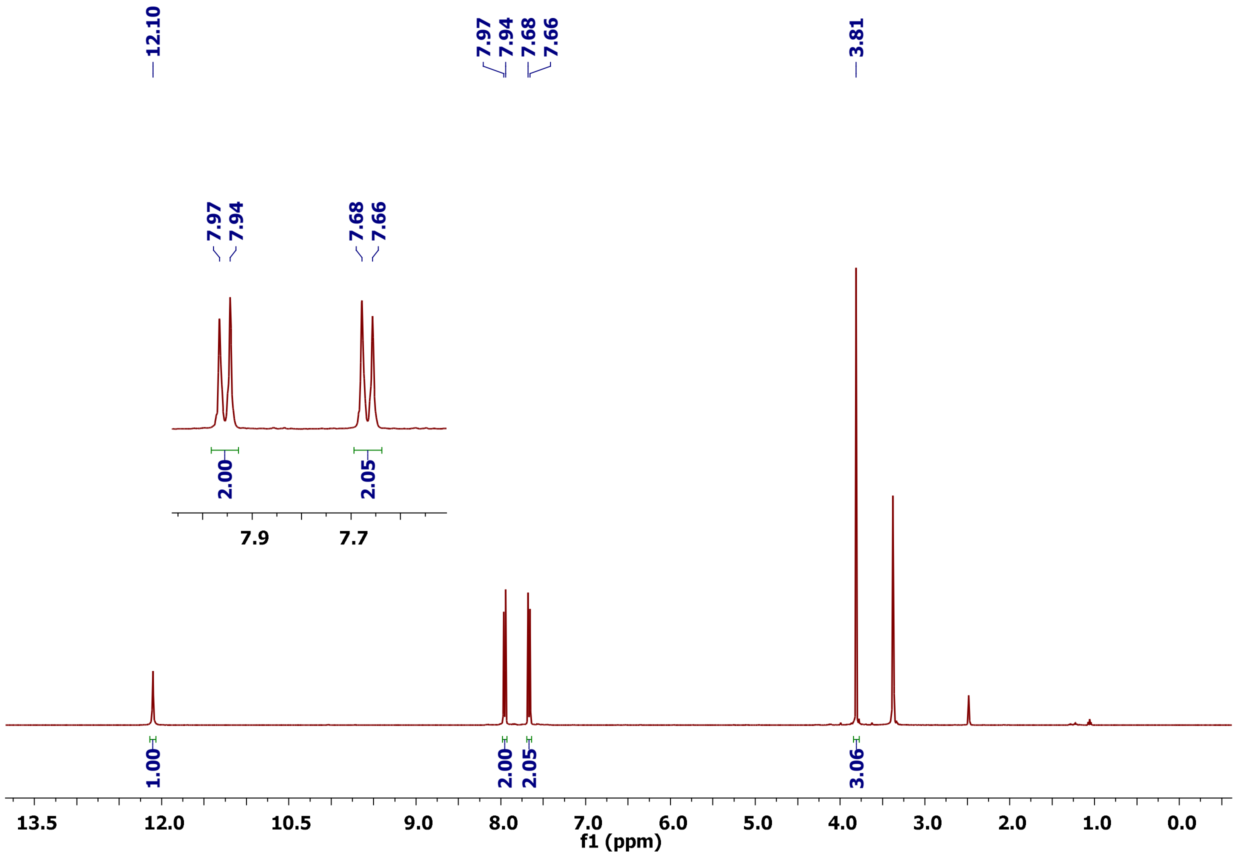

^{13}C NMR (DMSO-d6) spectrum of methyl 4-((cyanocarbonyl)amino)benzoate (2m)

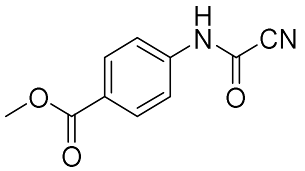

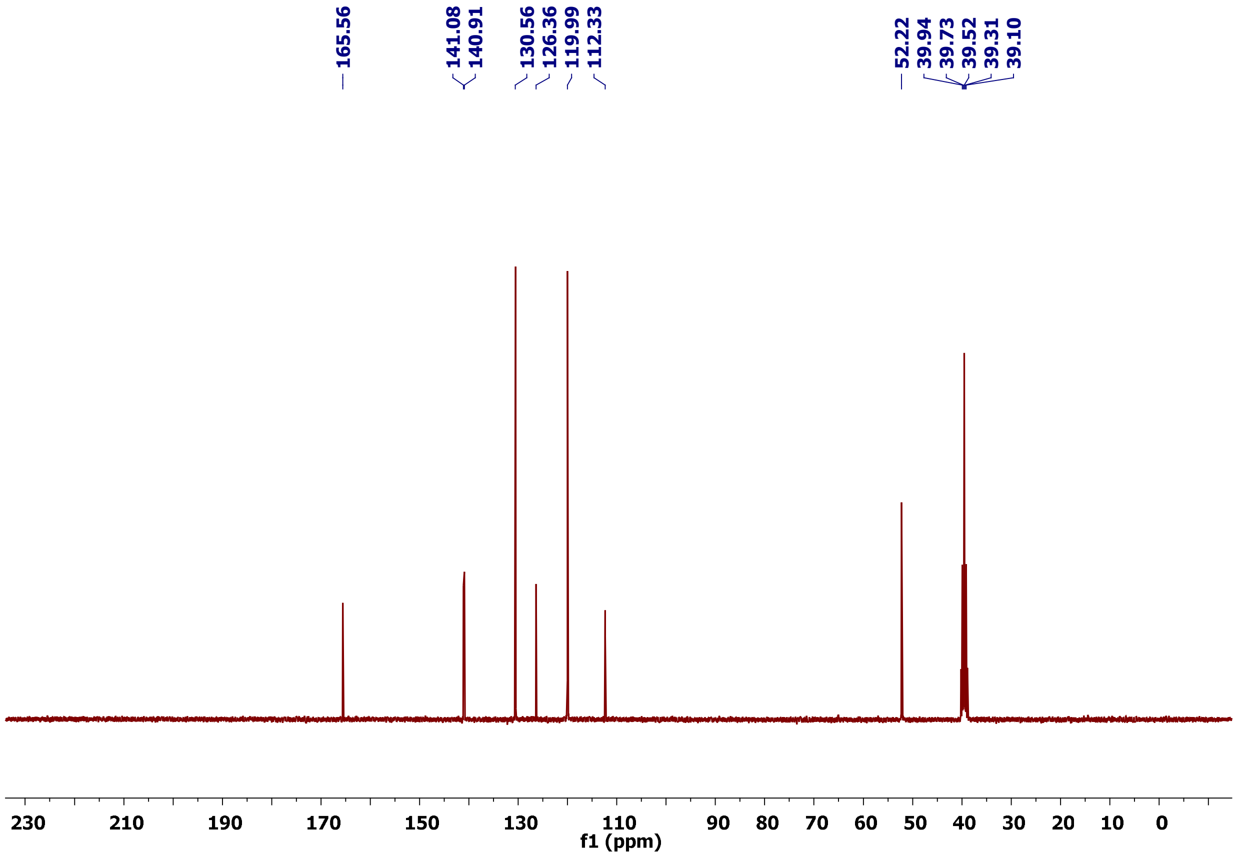

^{13}C CRAPT NMR (DMSO-d6) spectrum of methyl 4-((cyanocarbonyl)amino)benzoate (2m)

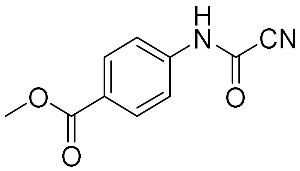

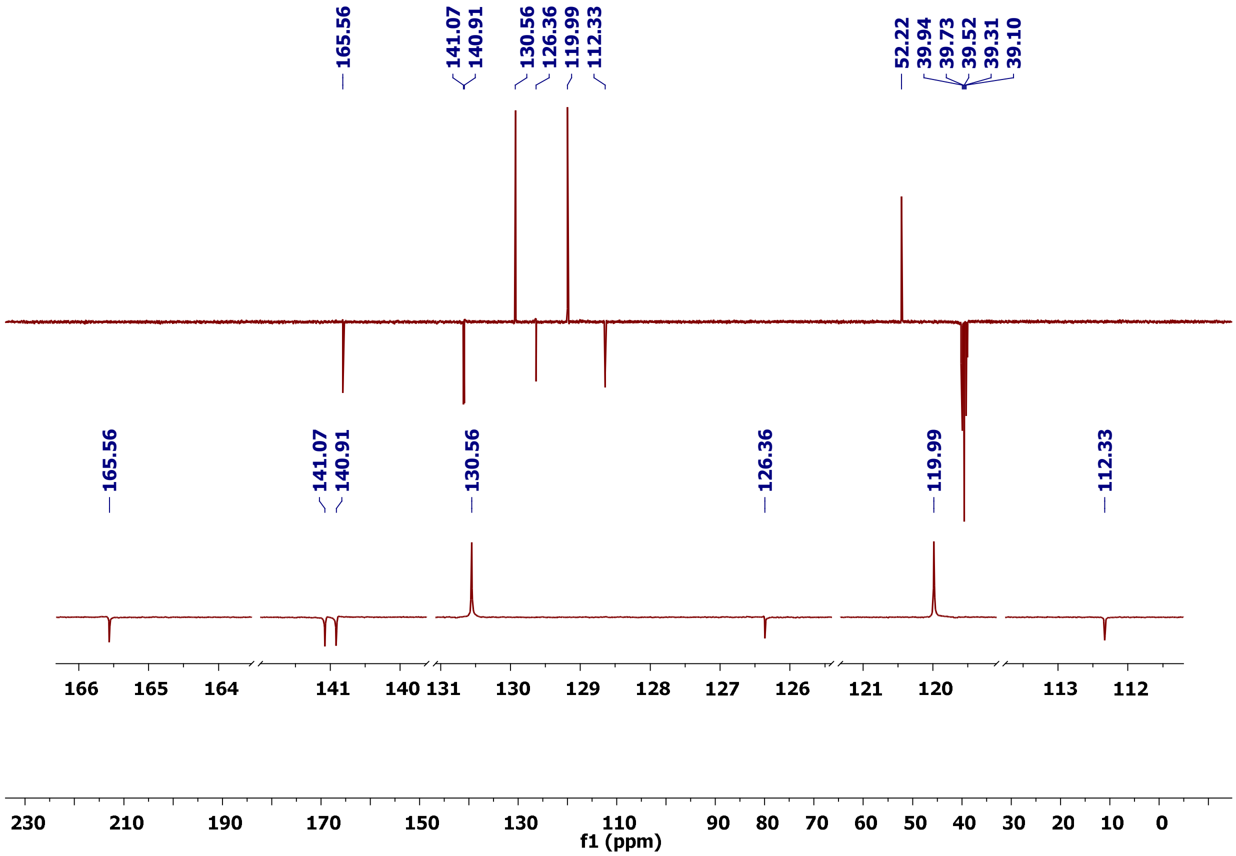

^{1}H-^{1}H gDQCOSY NMR (DMSO-d6) spectrum of methyl 4-((cyanocarbonyl)amino)benzoate (2m)

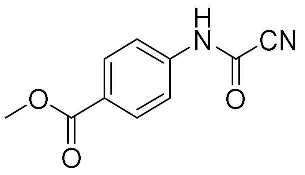

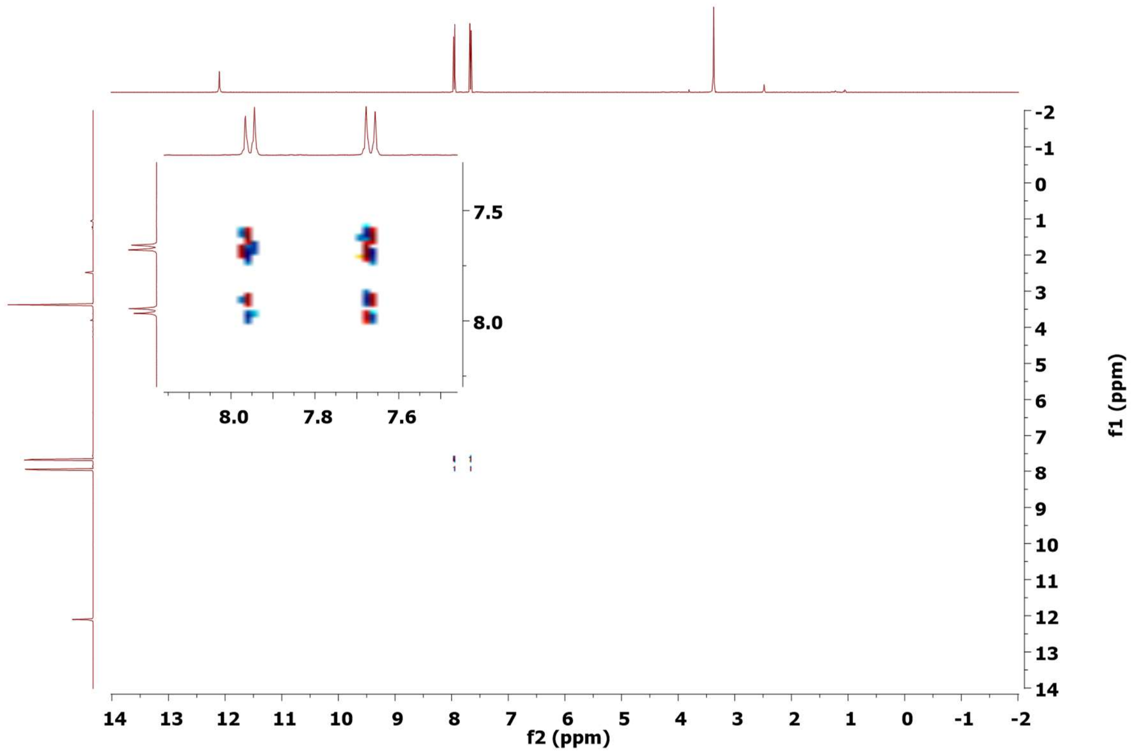

^{1}H-^{13}C-gHSQC NMR (DMSO-d6) spectrum of methyl 4-((cyanocarbonyl)amino)benzoate (2m)

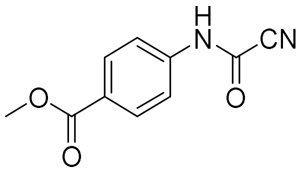

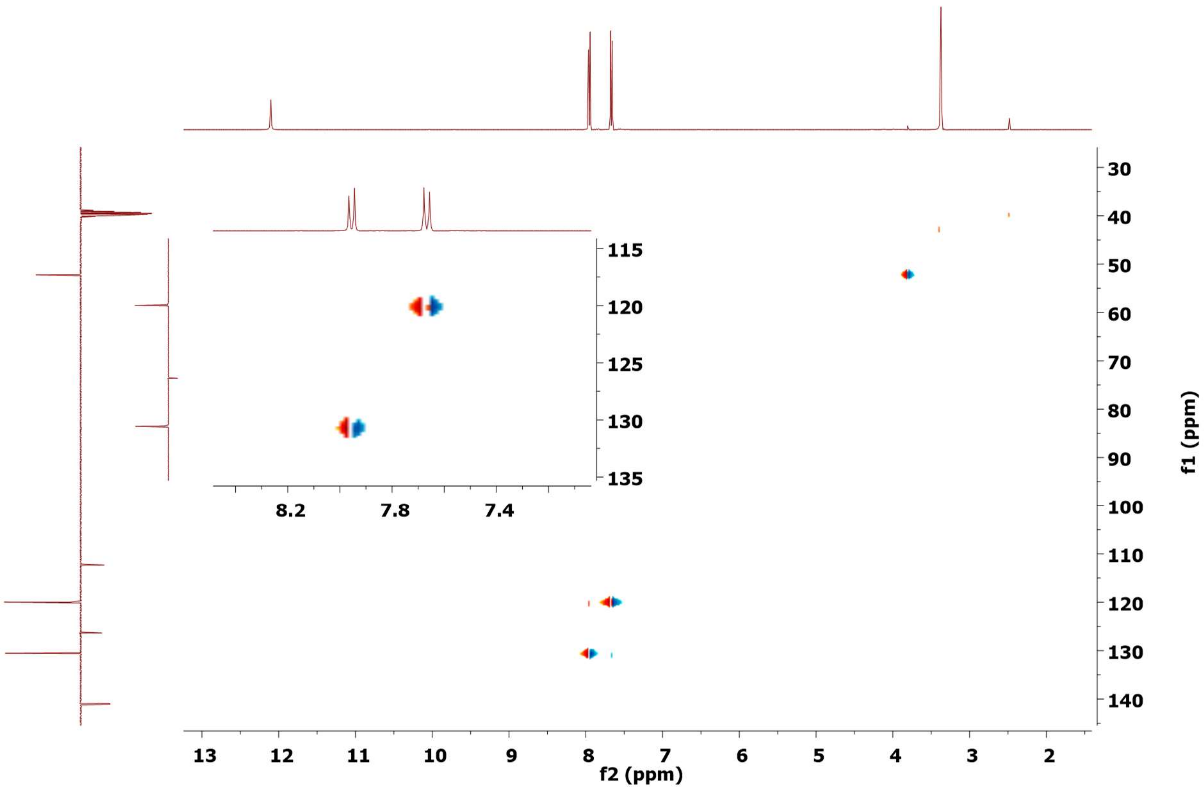

^{1}H-^{13}C-gHMBC NMR (DMSO-d6) spectrum of methyl 4-((cyanocarbonyl)amino)benzoate (2m)

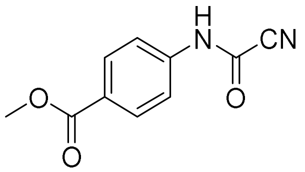

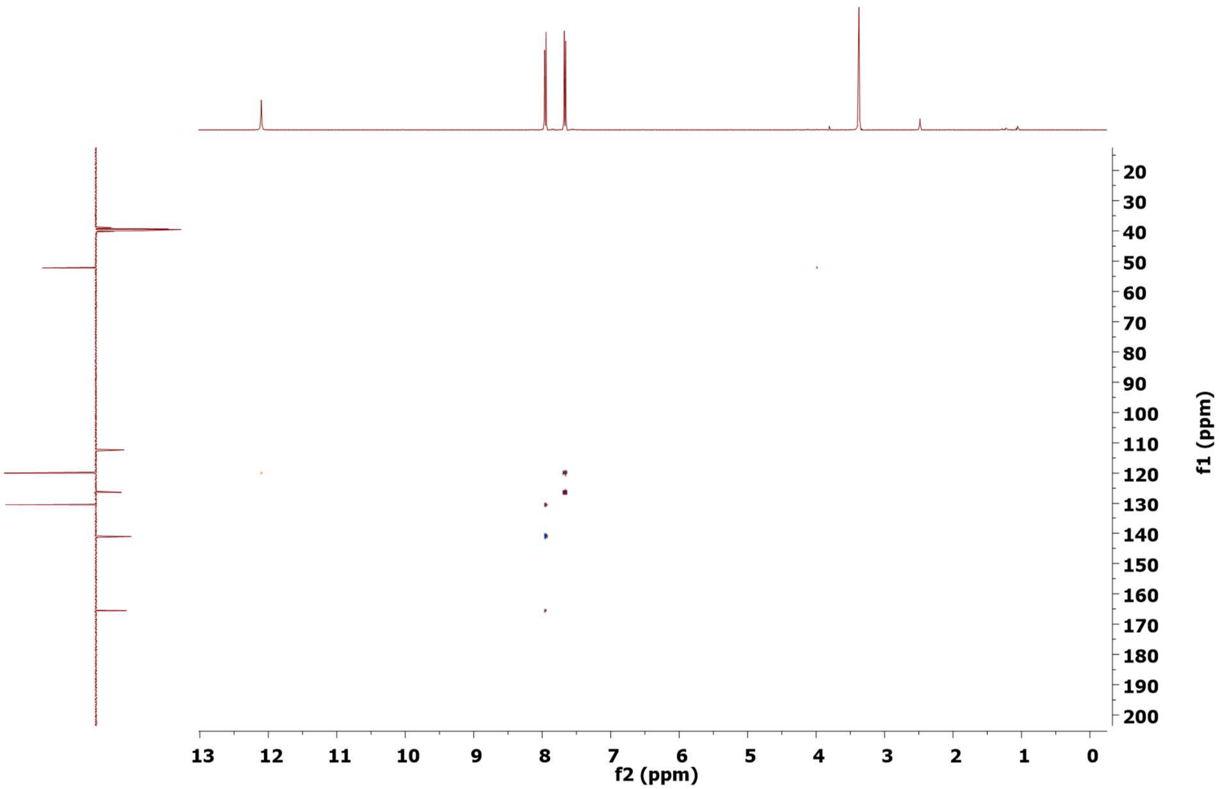

^{1}H NMR (DMSO-d6) spectrum of ethyl 4-((cyanocarbonyl)amino)benzoate (2n)

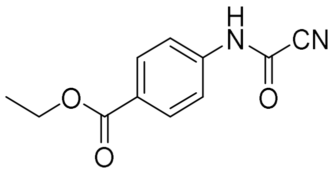

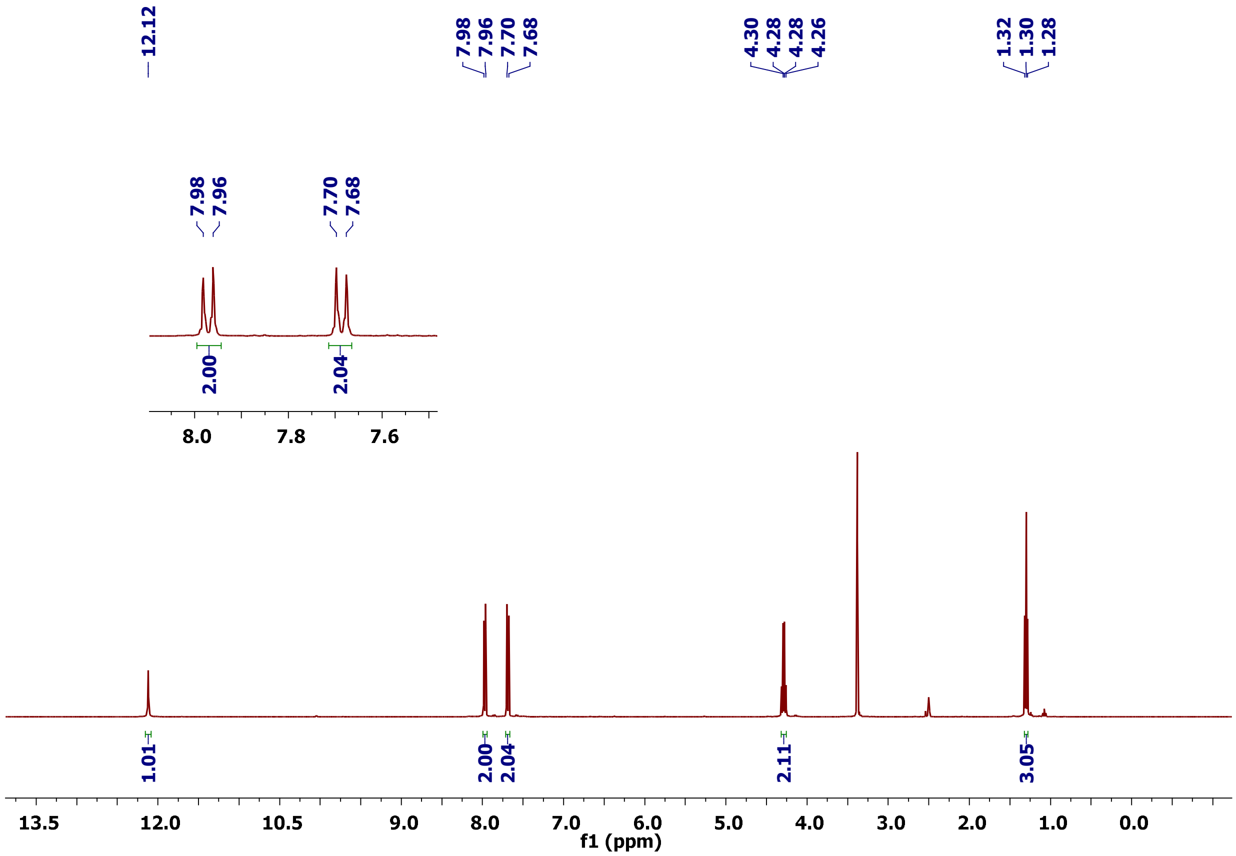

^{13}C NMR (DMSO-d6) spectrum of ethyl 4-((cyanocarbonyl)amino)benzoate (2n)

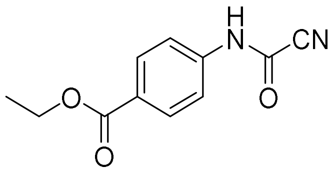

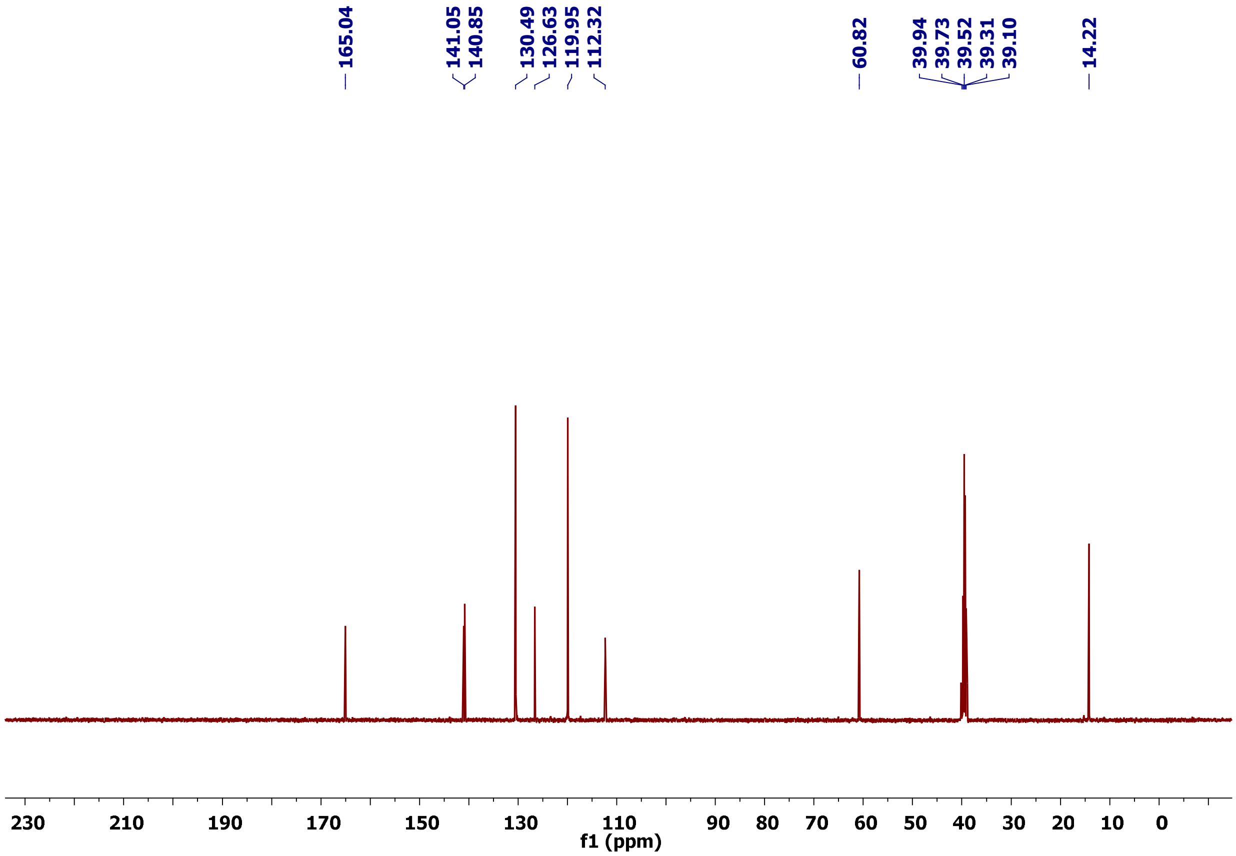

^{13}C CRAPT NMR (DMSO-d6) spectrum of ethyl 4-((cyanocarbonyl)amino)benzoate (2n)

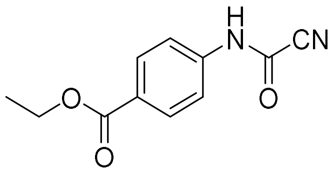

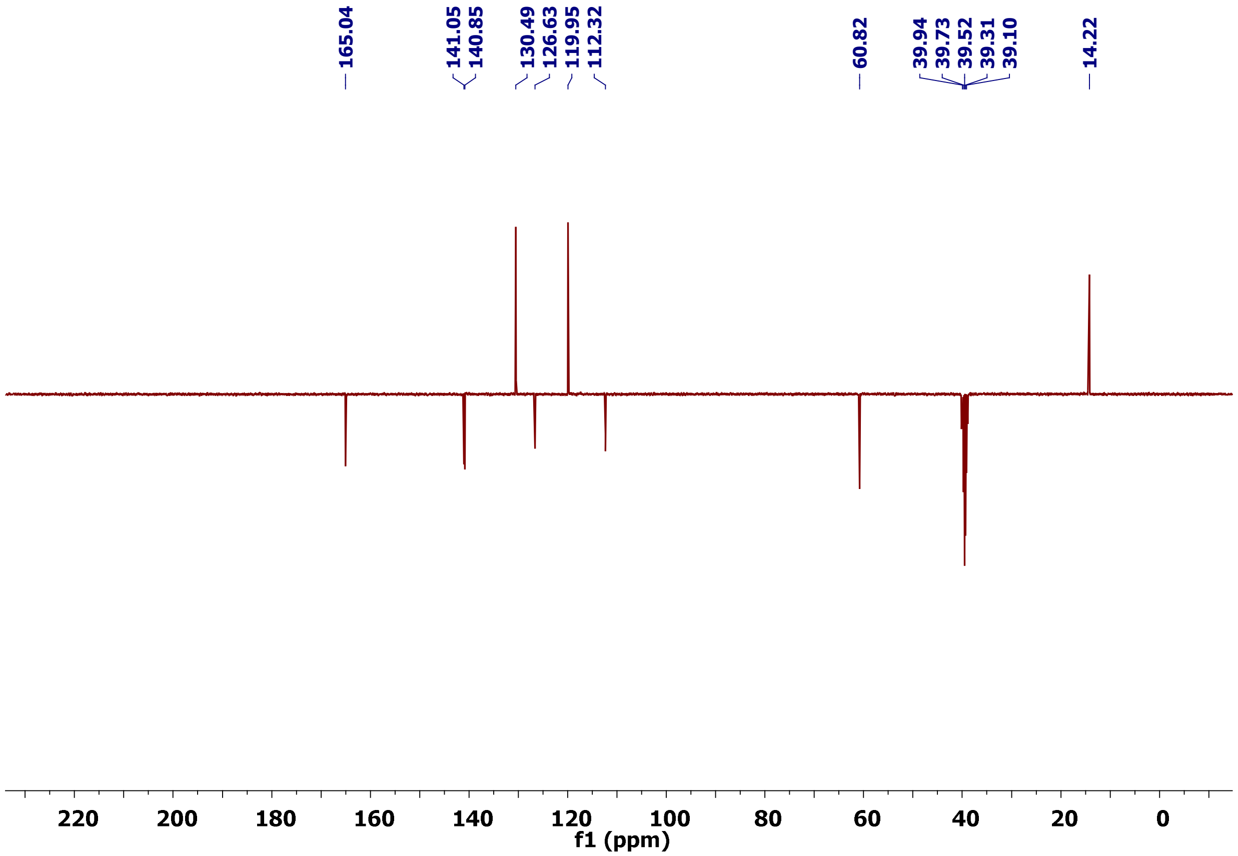

^1H-^1H gDQCOSY NMR (DMSO-d6) spectrum of ethyl 4-((cyanocarbonyl)amino)benzoate (2n)

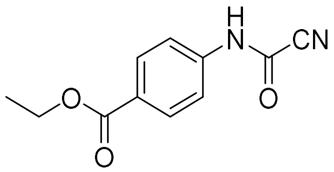

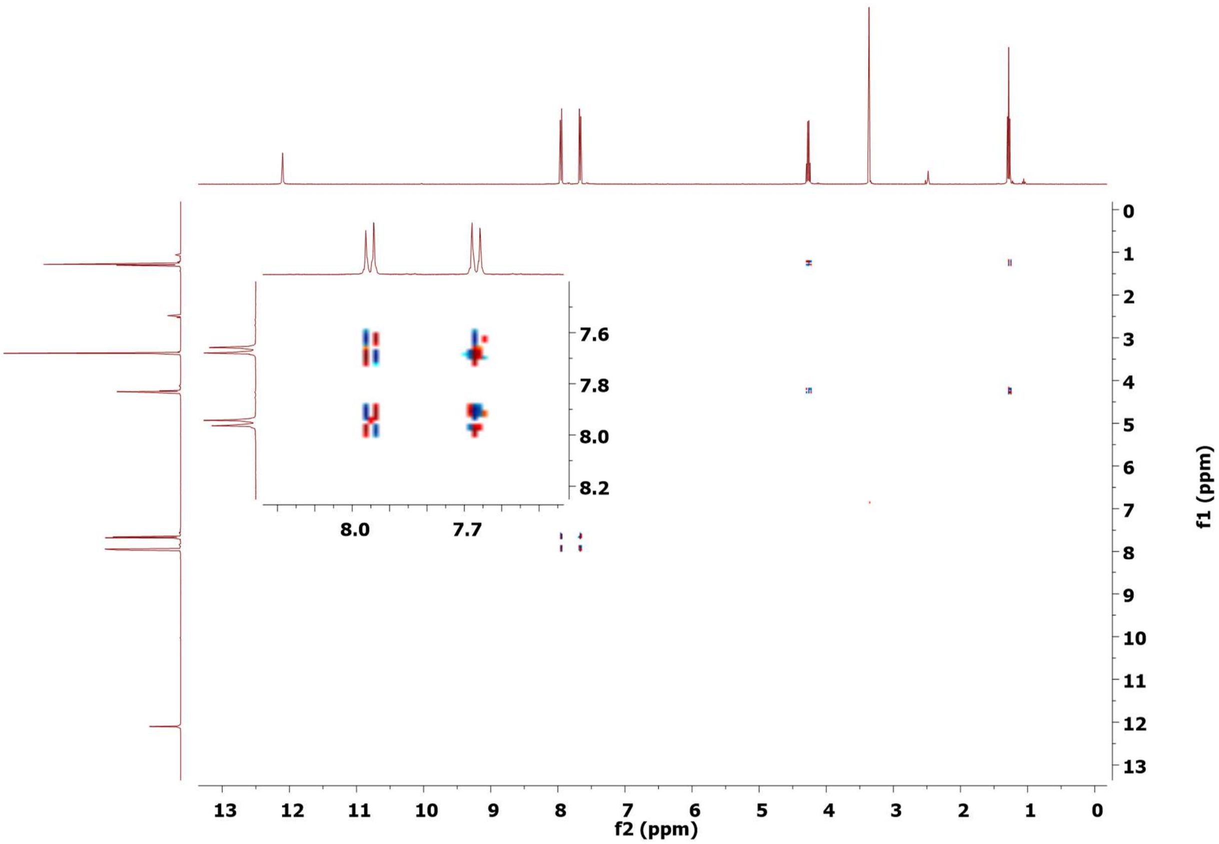

^{1}H-^{13}C-gHSQC NMR (DMSO-d6) spectrum of ethyl 4-((cyanocarbonyl)amino)benzoate (2n)

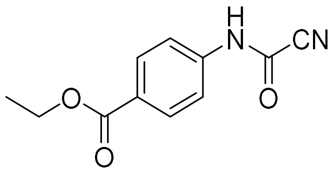

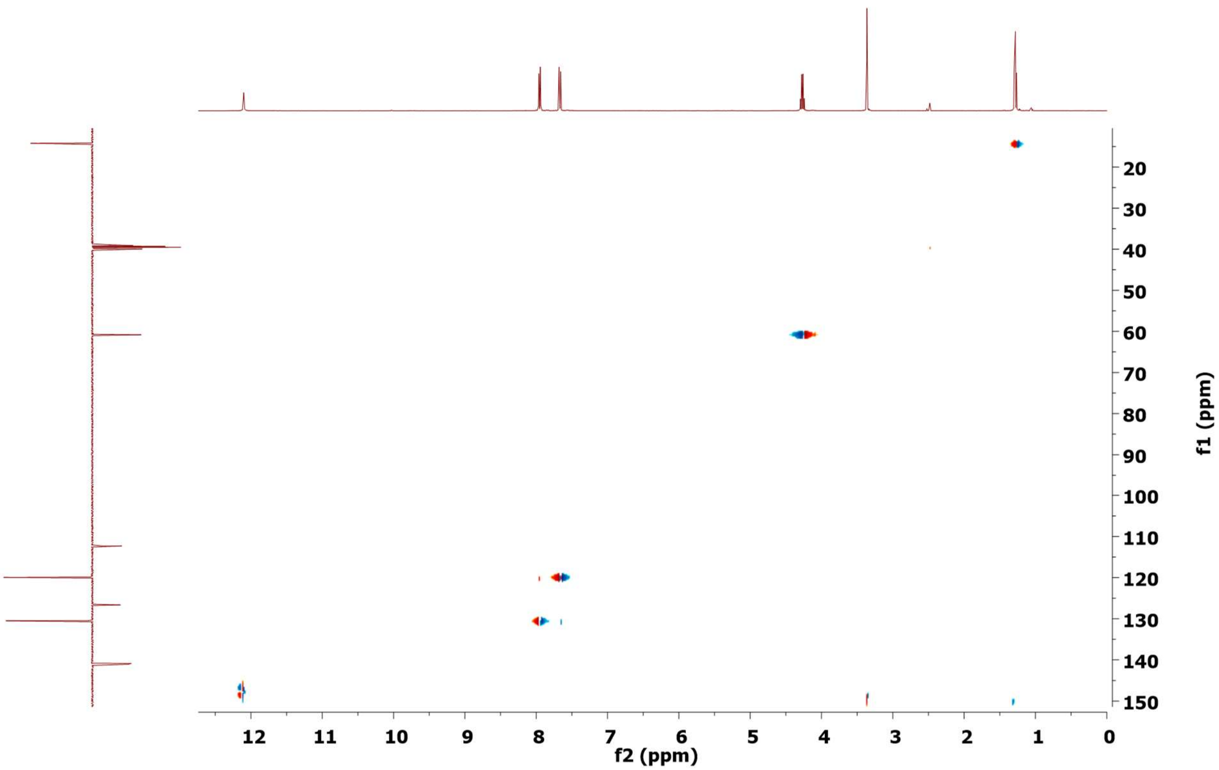

^1H-^{13}C-gHMBC NMR (DMSO-d6) spectrum of ethyl 4-((cyanocarbonyl)amino)benzoate (2n)

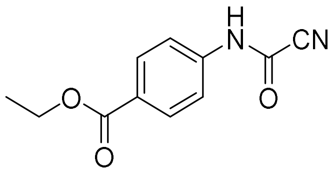

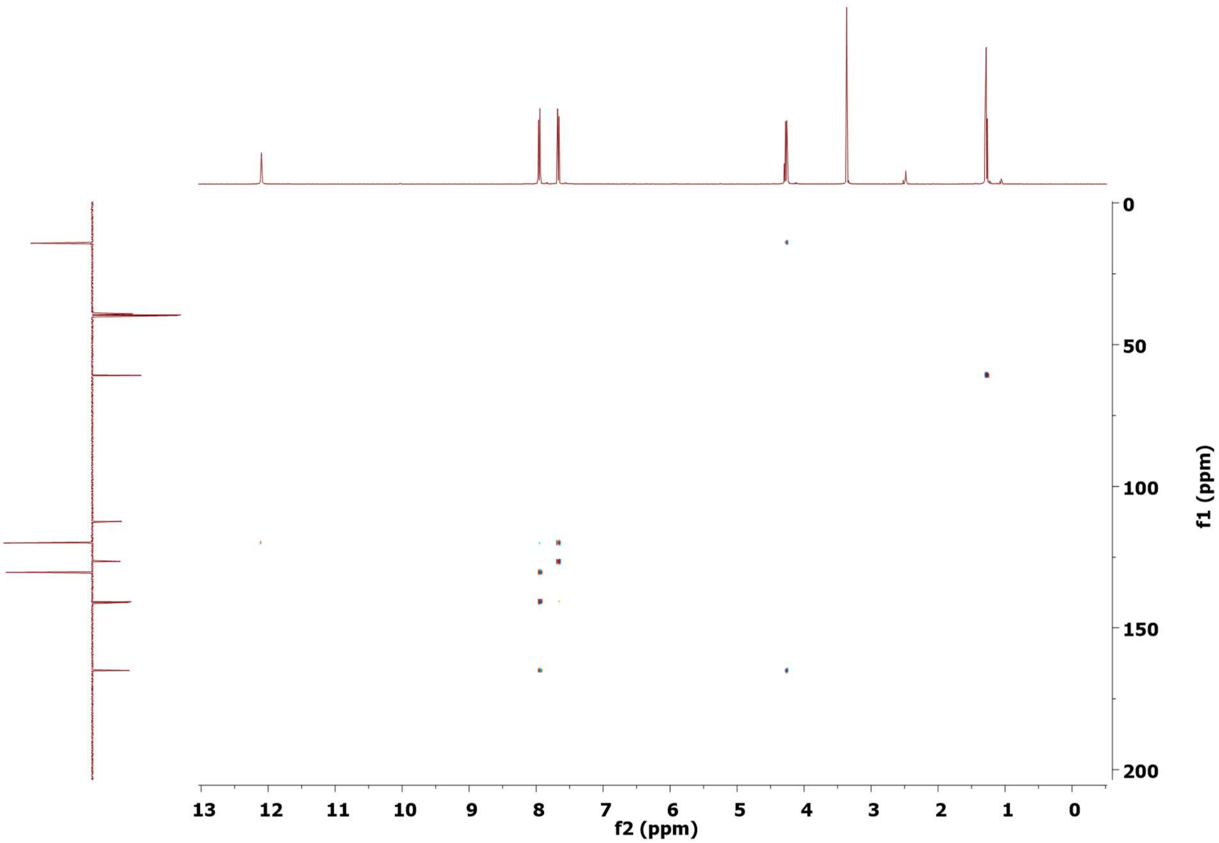

^{1}H NMR (DMSO-d6) spectrum of (4-(trifluoromethyl)phenyl)carbamoyl cyanide (2o)

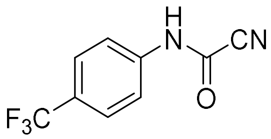

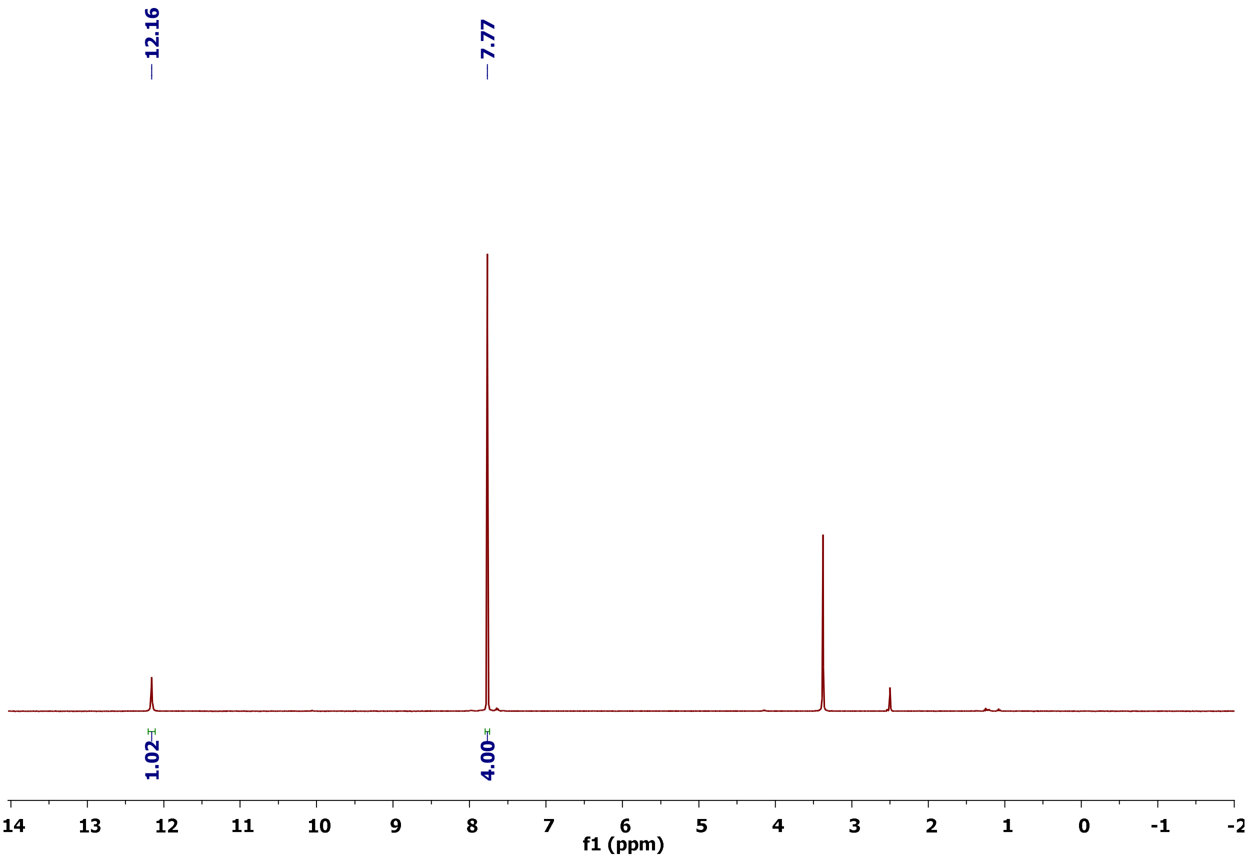

^{13}C NMR (DMSO-d6) spectrum of (4-(trifluoromethyl)phenyl)carbamoyl cyanide (2o)

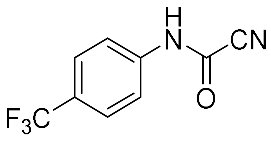

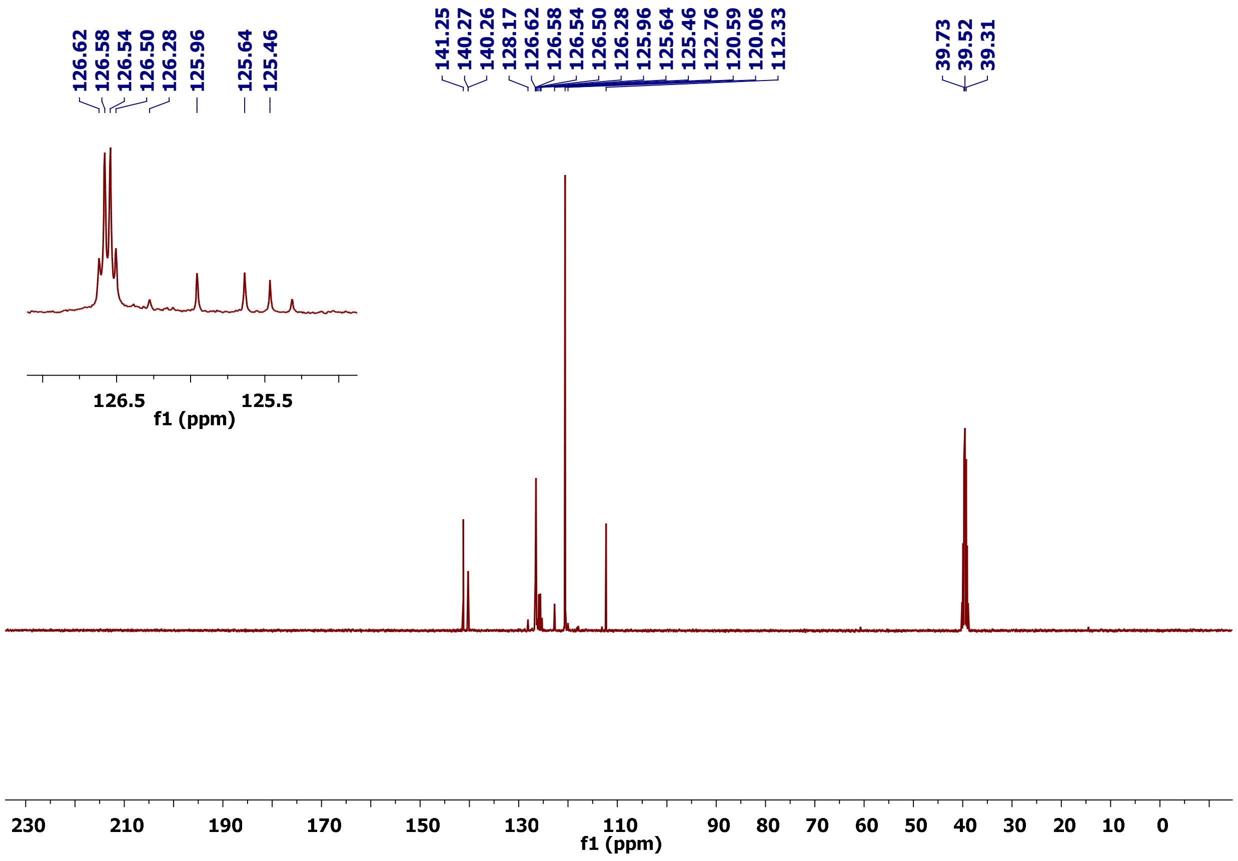

^{13}C CRAPT NMR (DMSO-d6) spectrum of (4-(trifluoromethyl)phenyl)carbamoyl cyanide (2o)

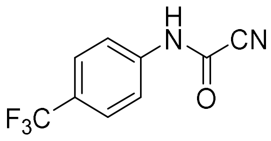

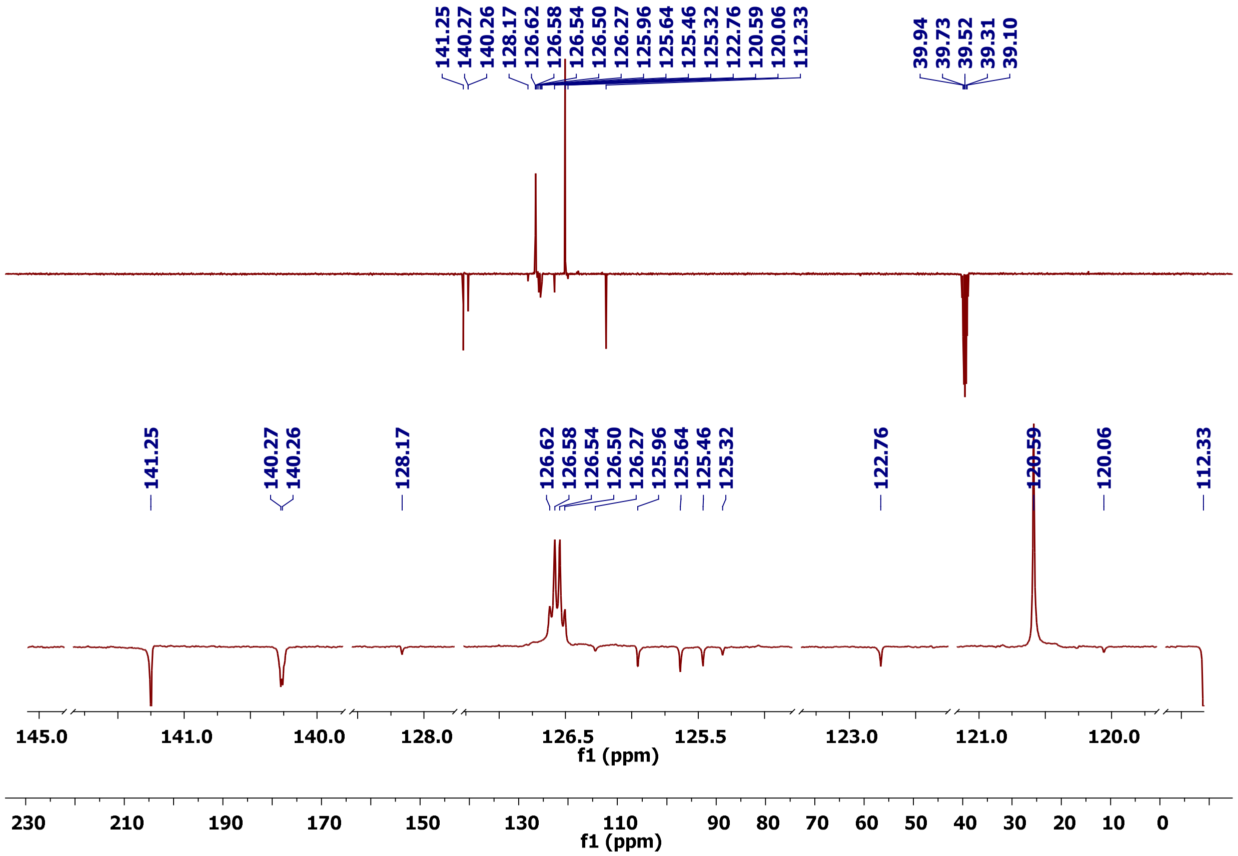

^{1}H-^{13}C-gHSQC NMR (DMSO-d6) spectrum of (4-(trifluoromethyl)phenyl)carbamoyl cyanide (2o)

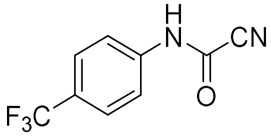

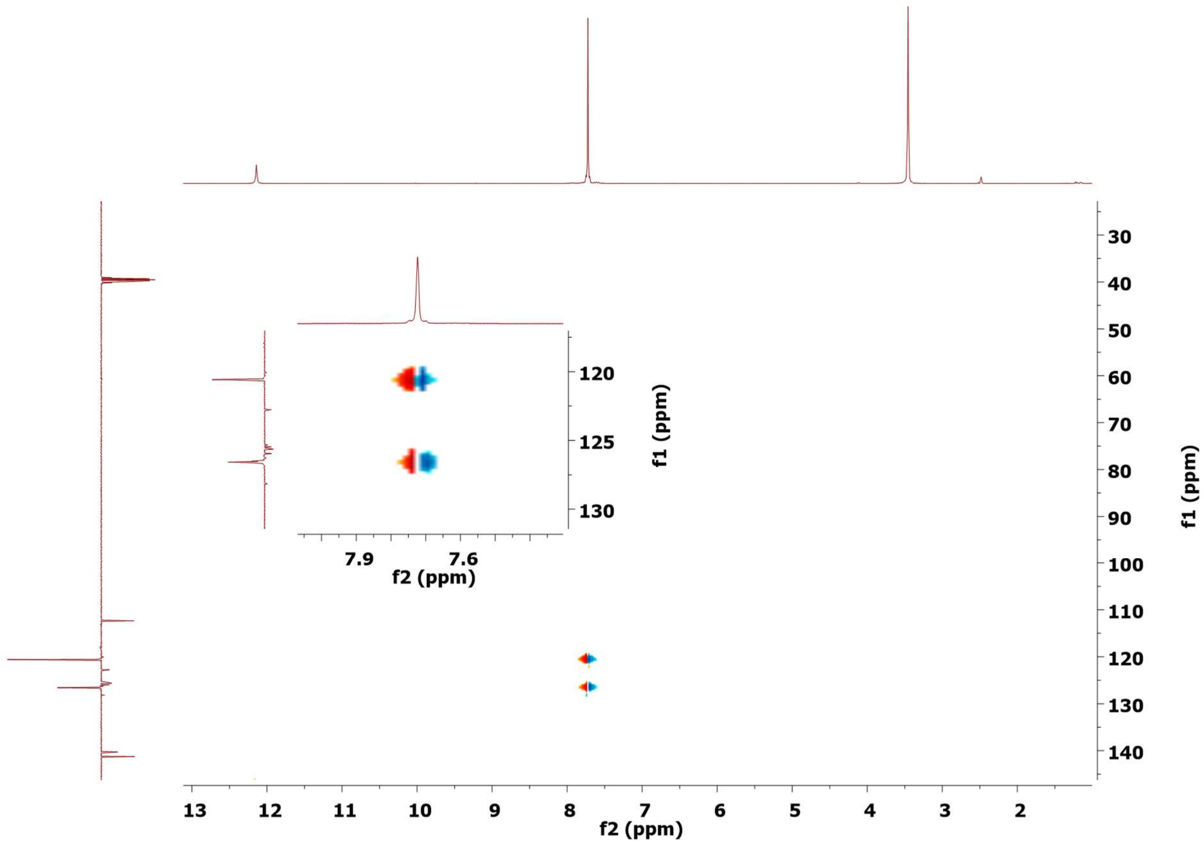

^{1}H-^{13}C-gHMBC NMR (DMSO-d6) spectrum of (4-(trifluoromethyl)phenyl)carbamoyl cyanide (2o)

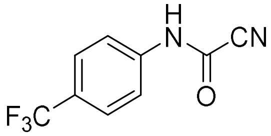

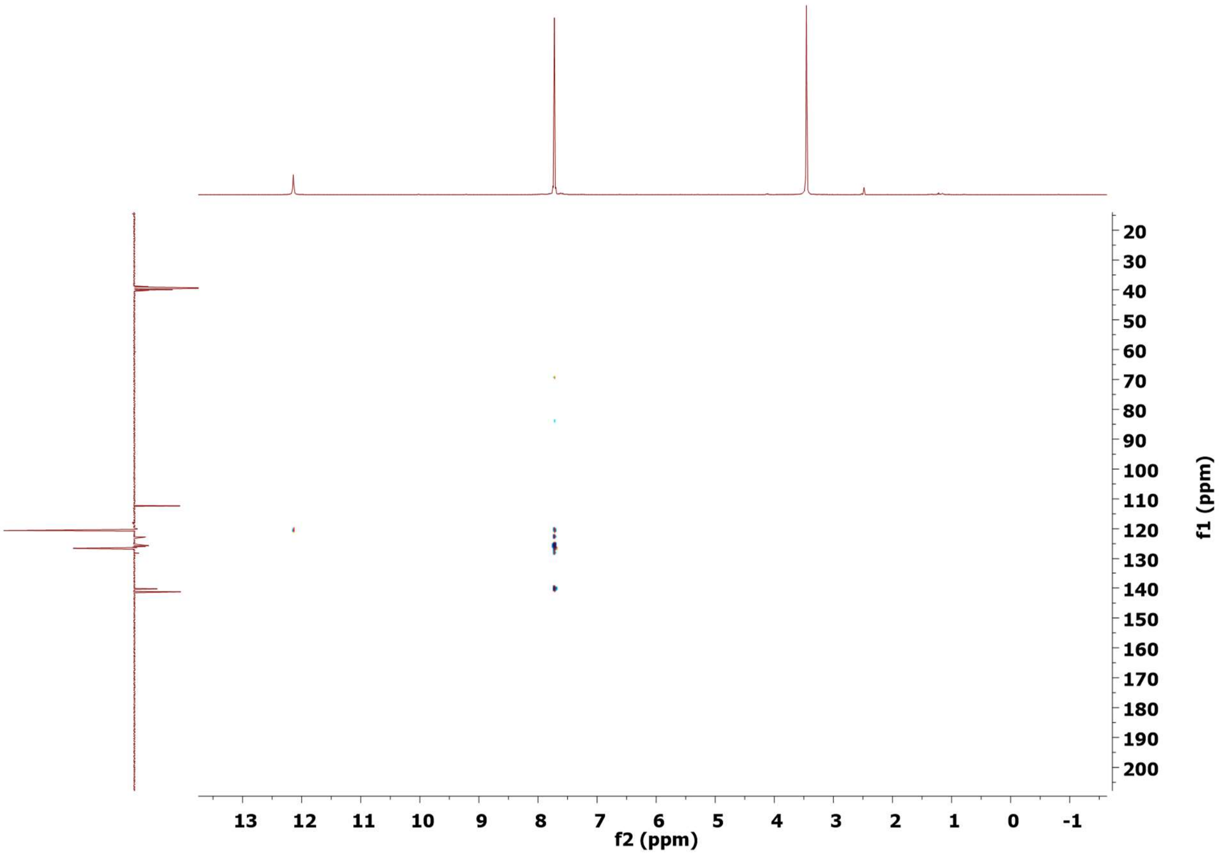

^{1}H NMR (DMSO-d6) spectrum of (4-ethylphenyl)carbamoyl cyanide (2p)

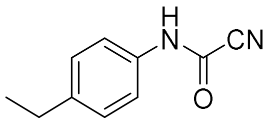

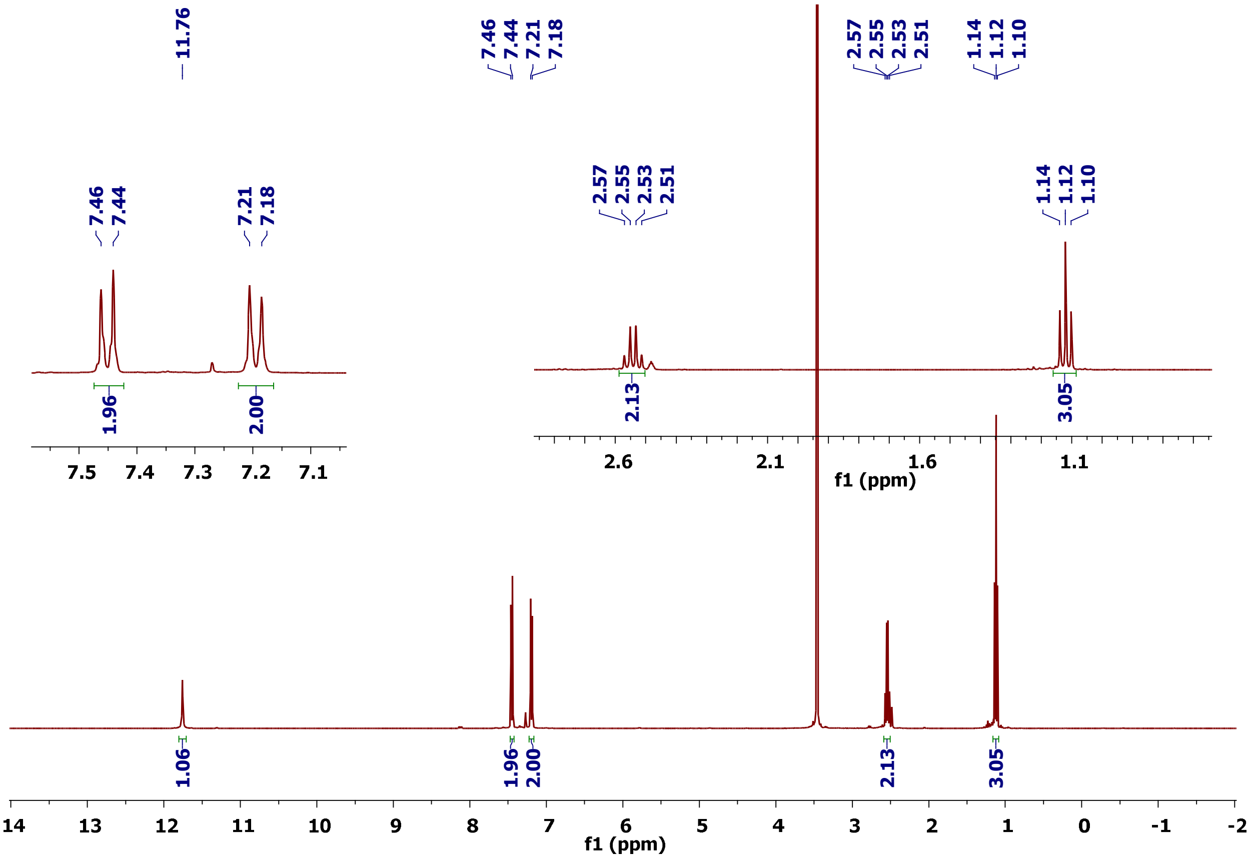

^{13}C NMR (DMSO-d6) spectrum of (4-ethylphenyl)carbamoyl cyanide (2p)

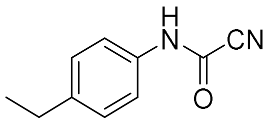

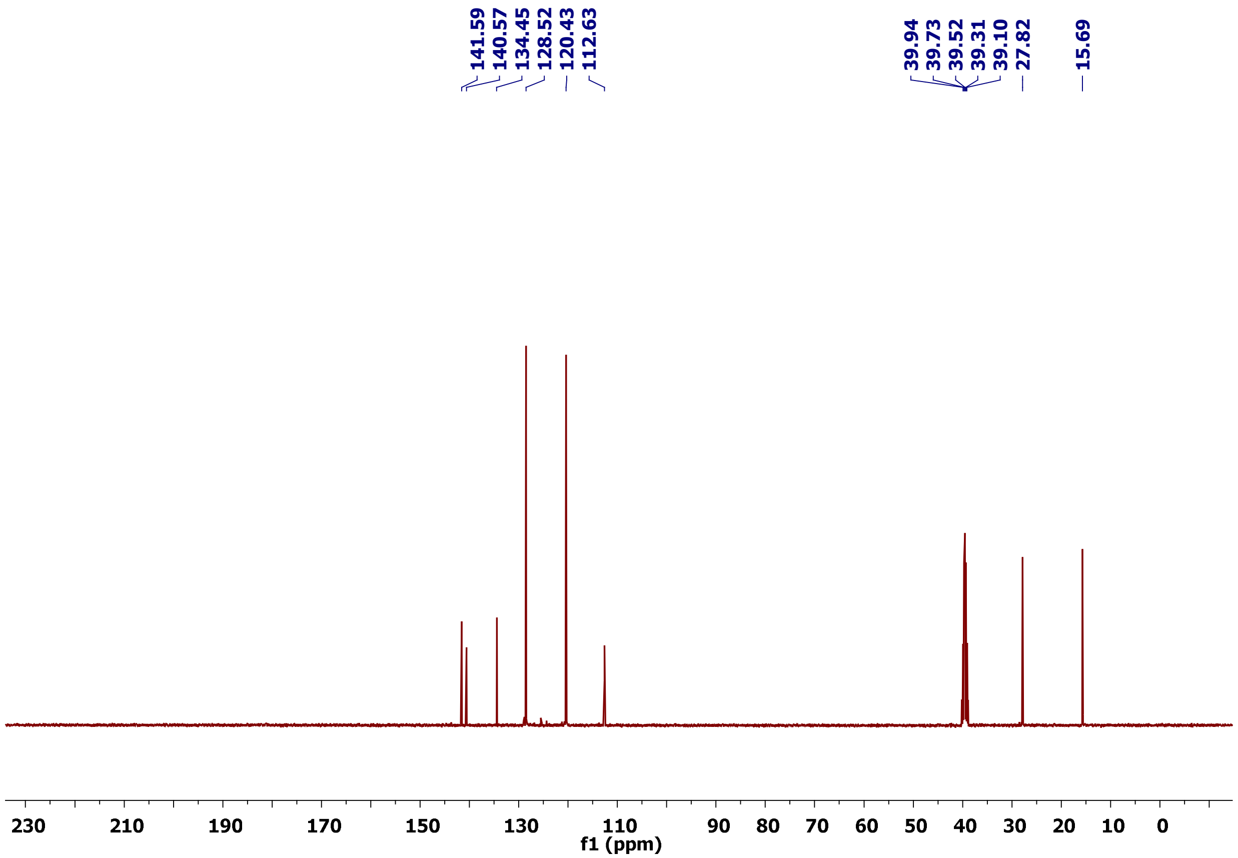

^{13}C CRAPT NMR (DMSO-d6) spectrum of (4-ethylphenyl)carbamoyl cyanide (2p)

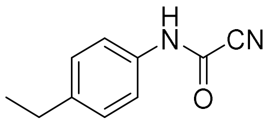

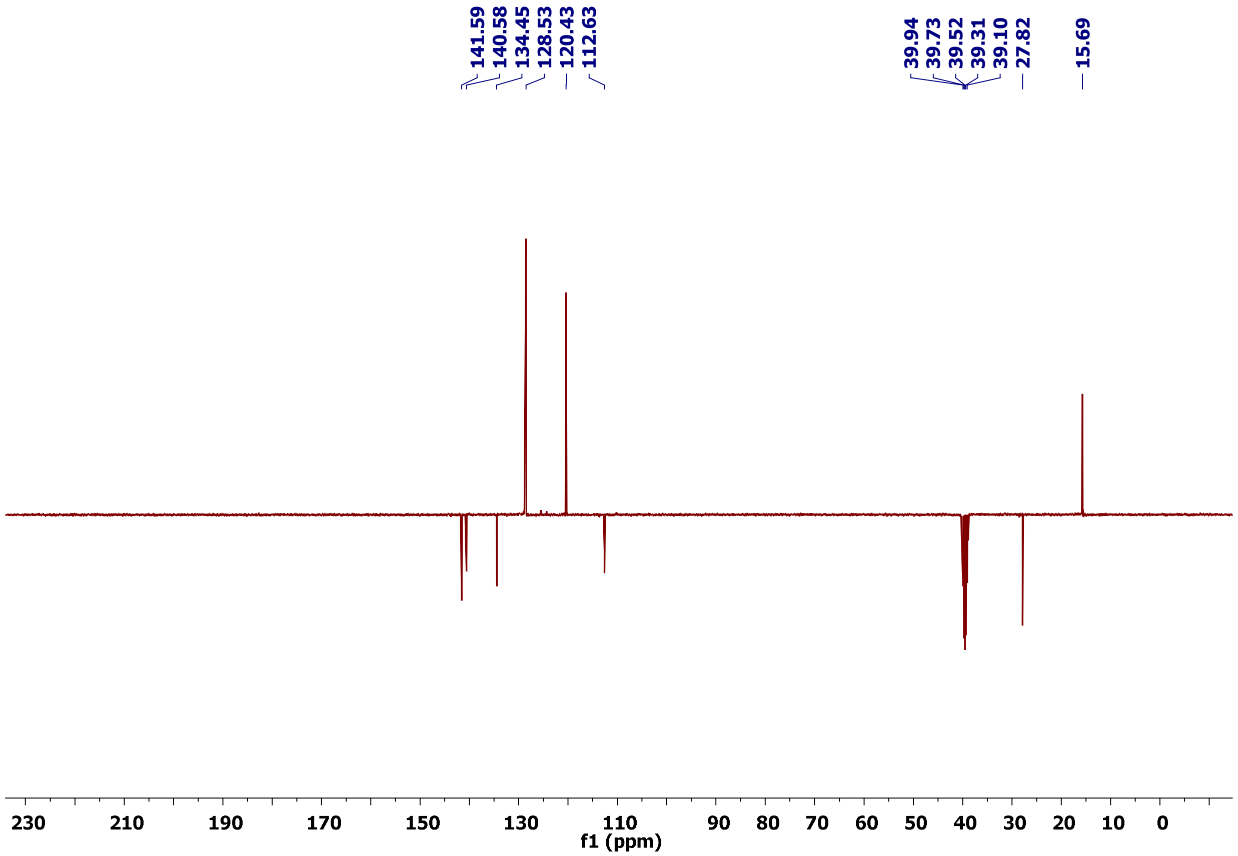

^{1}H-^{1}H gDQCOSY NMR (DMSO-d6) spectrum of (4-ethylphenyl)carbamoyl cyanide (2p)

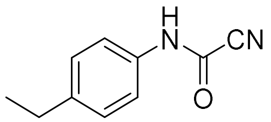

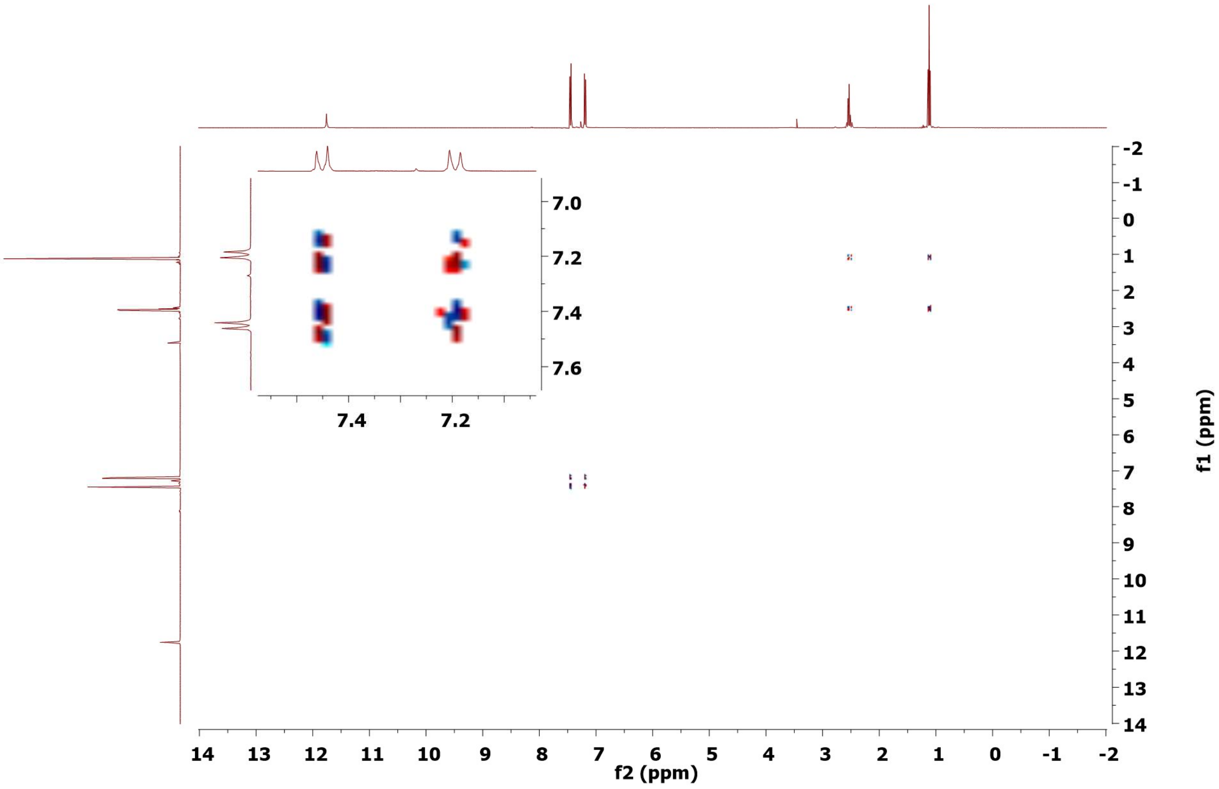

^{1}H-^{13}C-gHSQC NMR (DMSO-d6) spectrum of (4-ethylphenyl)carbamoyl cyanide (2p)

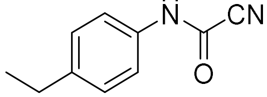

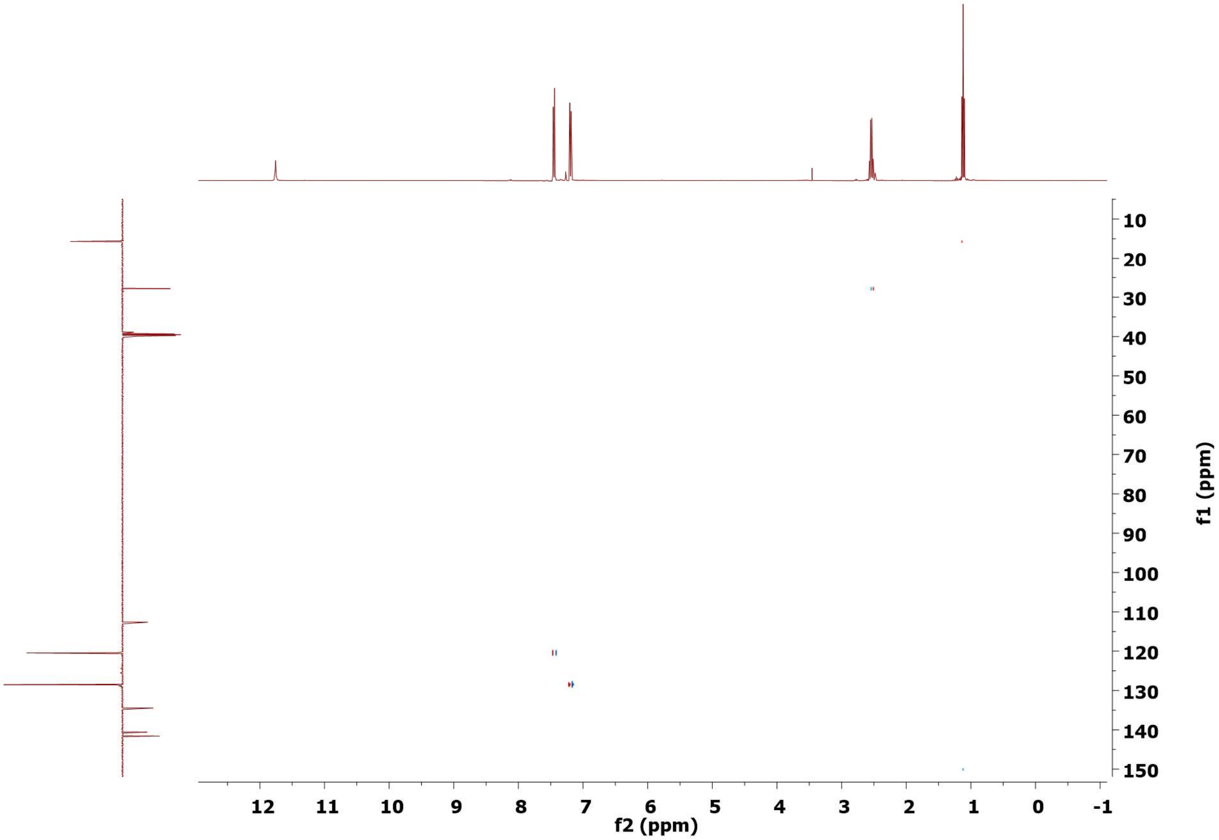

^{1}H-^{13}C-gHMBC NMR (DMSO-d6) spectrum of (4-ethylphenyl)carbamoyl cyanide (2p)

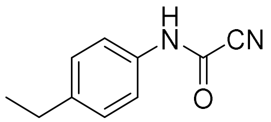

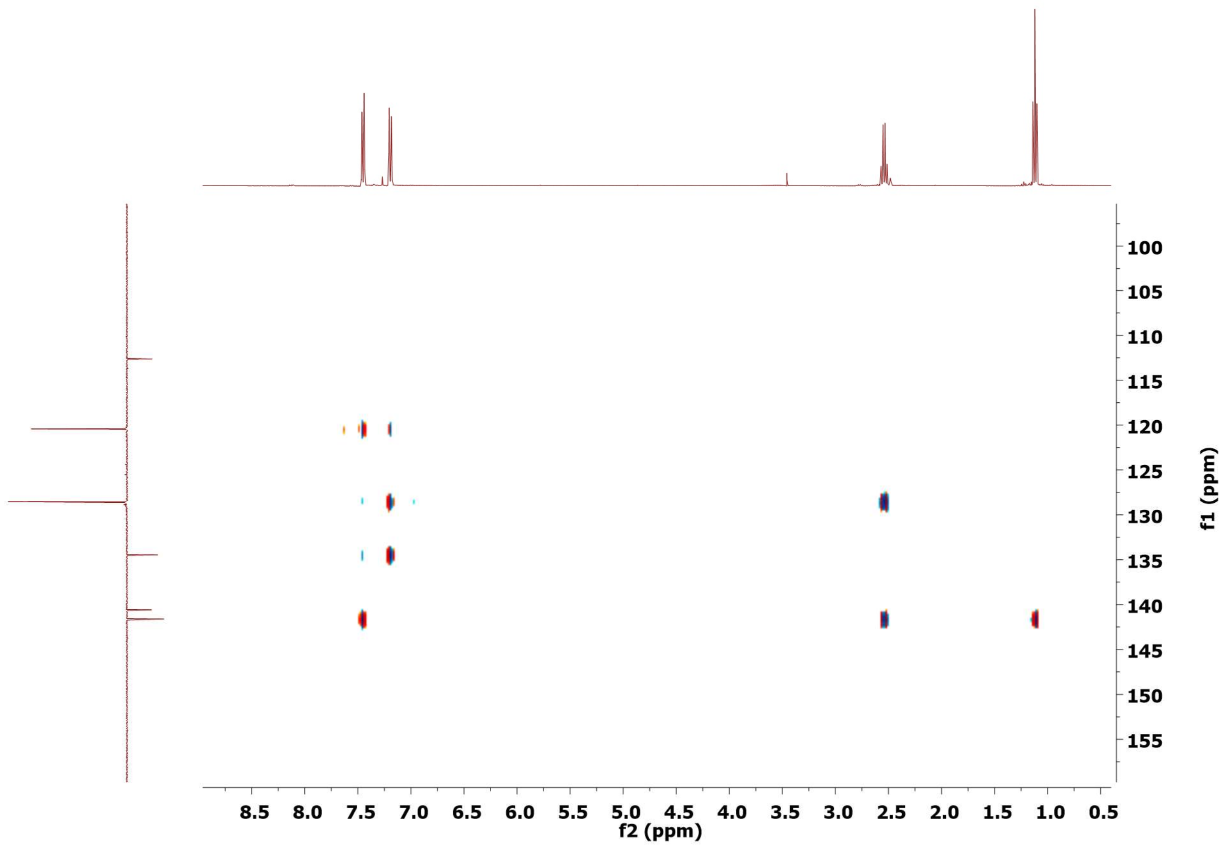

^{1}H NMR (DMSO-d6) spectrum of (4-iodophenyl)carbamoyl cyanide (2q)

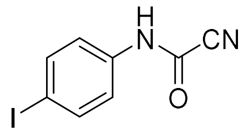

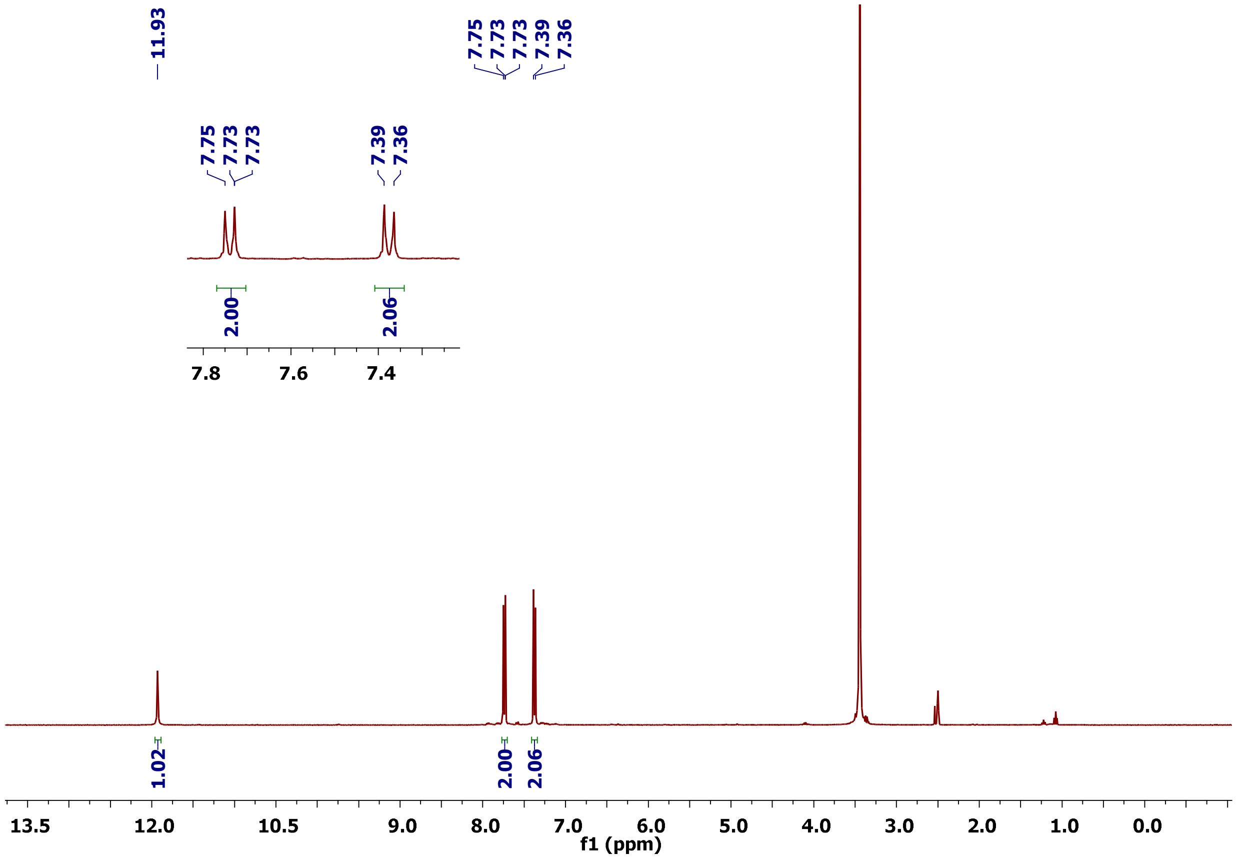

^{13}C NMR (DMSO-d6) spectrum of (4-iodophenyl)carbamoyl cyanide (2q)

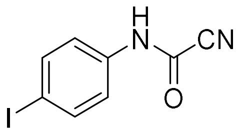

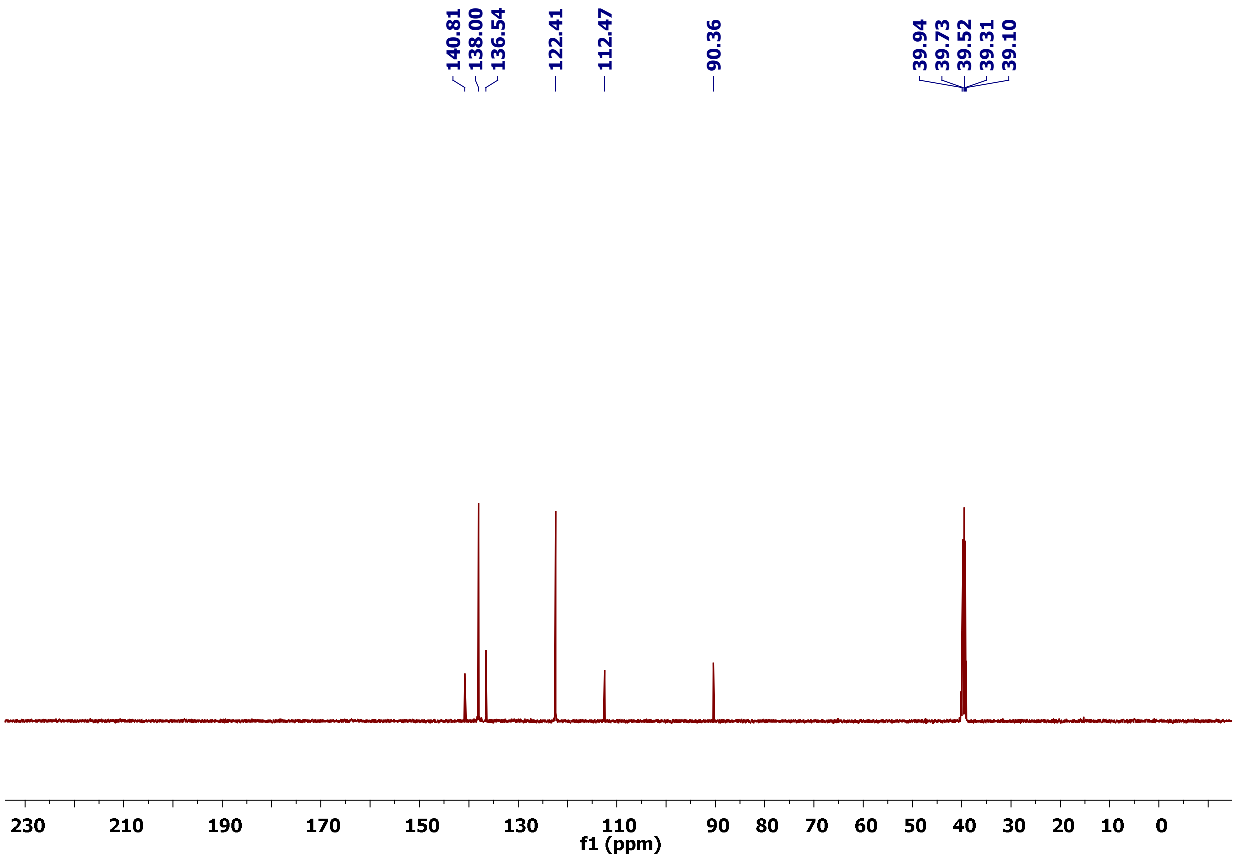

^{13}C CRAPT NMR (DMSO-d6) spectrum of (4-iodophenyl)carbamoyl cyanide (2q)

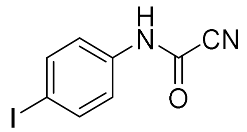

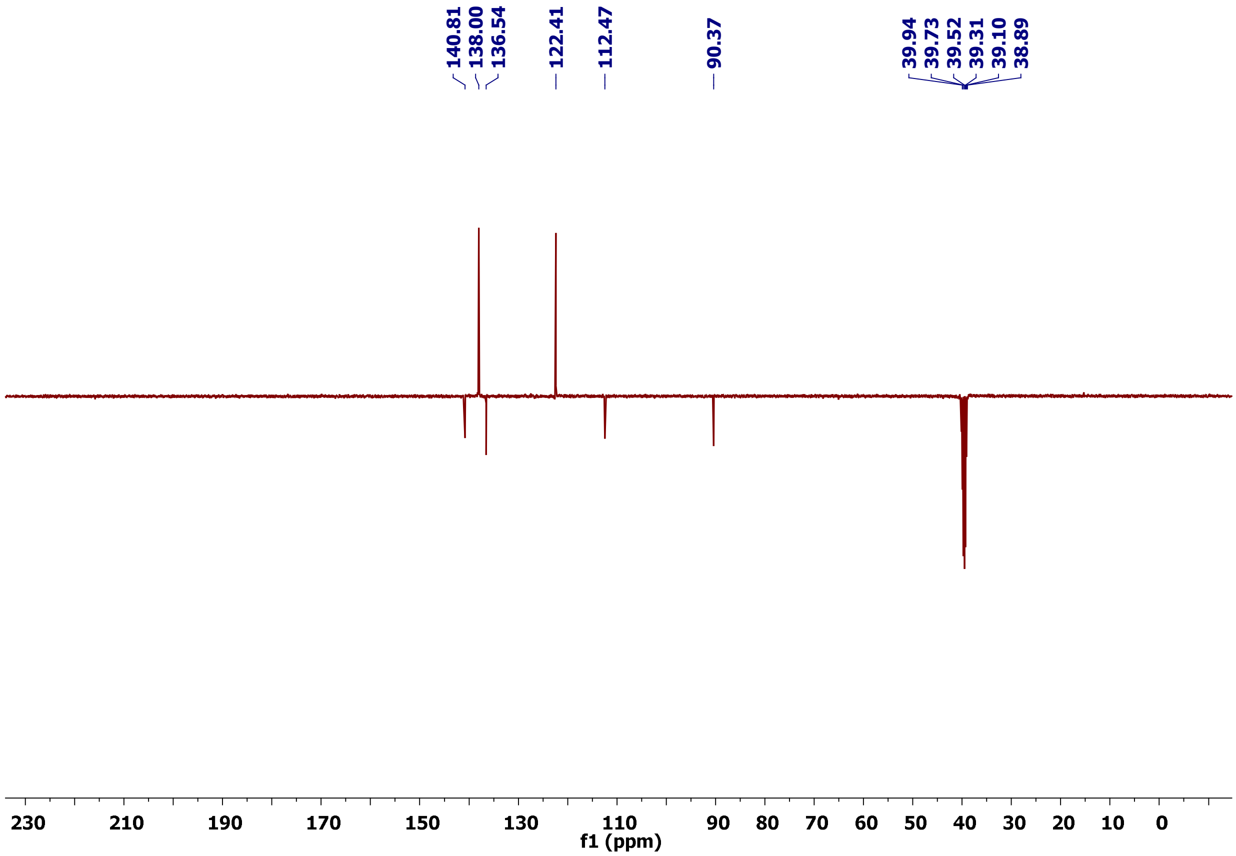

^{1}H-^{1}H gDQCOSY NMR (DMSO-d6) spectrum of (4-iodophenyl)carbamoyl cyanide (2q)

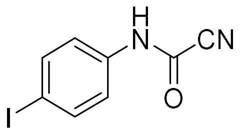

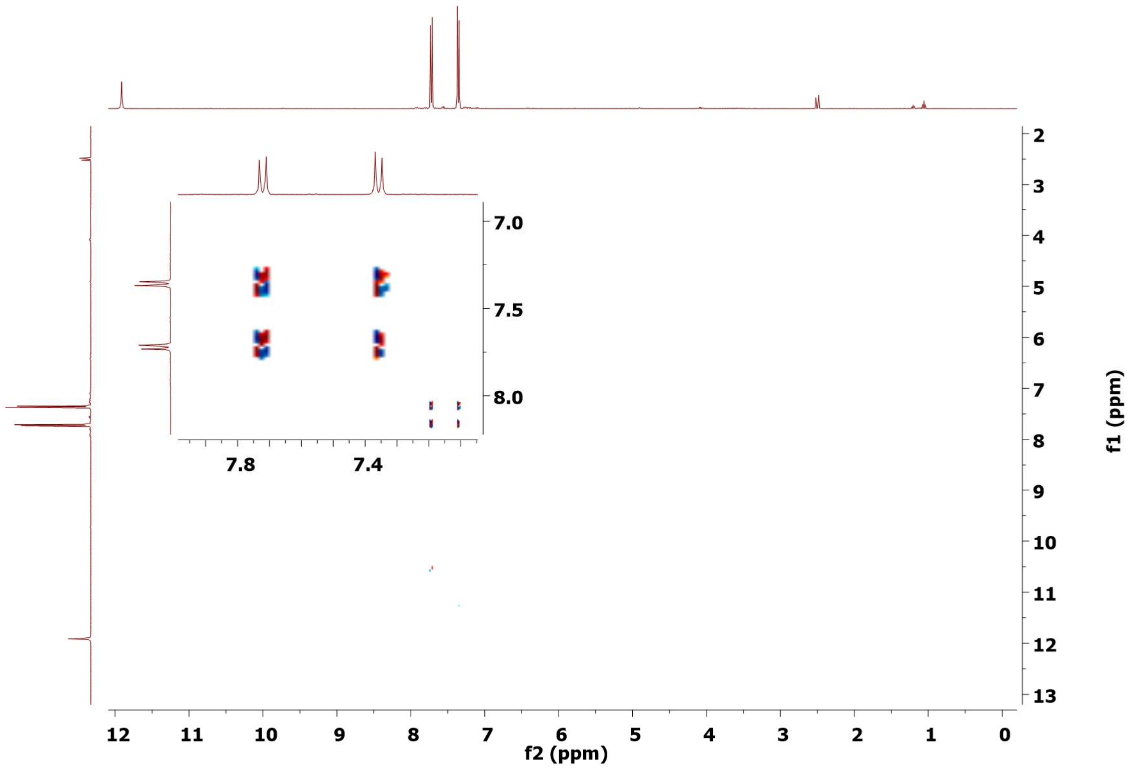

^{1}H-^{13}C-gHSQC NMR (DMSO-d6) spectrum of (4-iodophenyl)carbamoyl cyanide (2q)

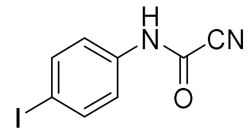

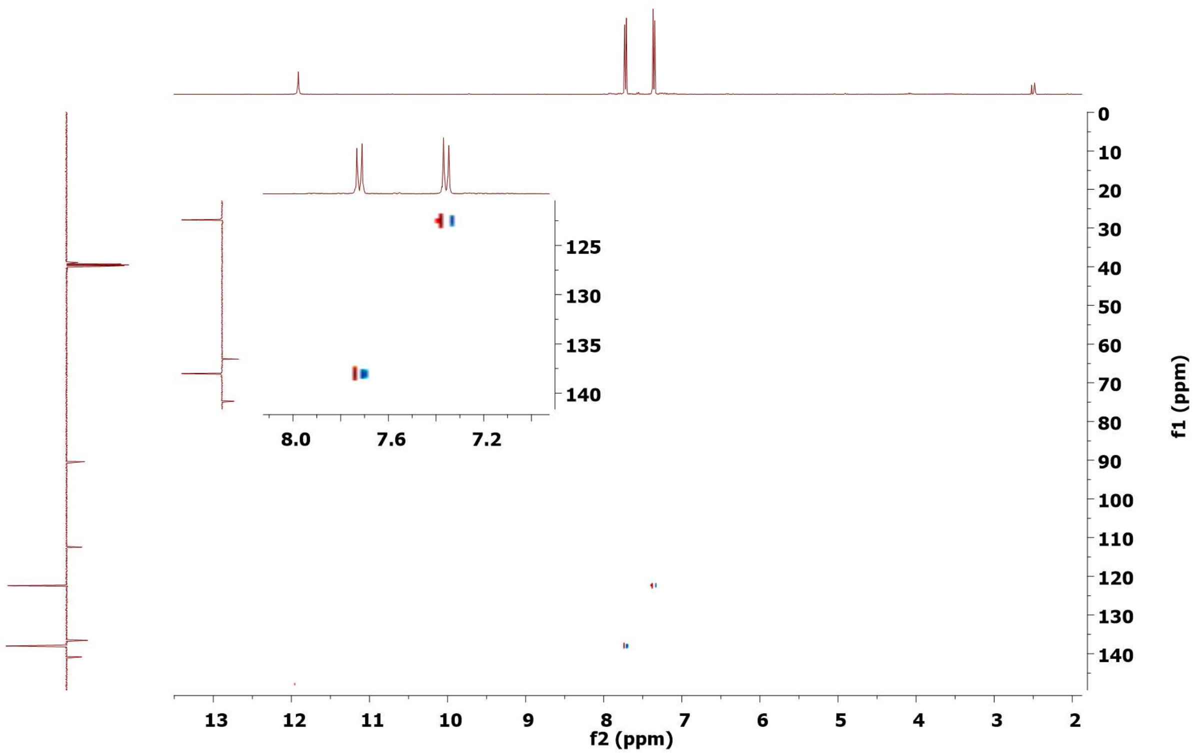

^{1}H-^{13}C-gHMBC NMR (DMSO-d6) spectrum of (4-iodophenyl)carbamoyl cyanide (2q)

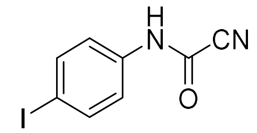

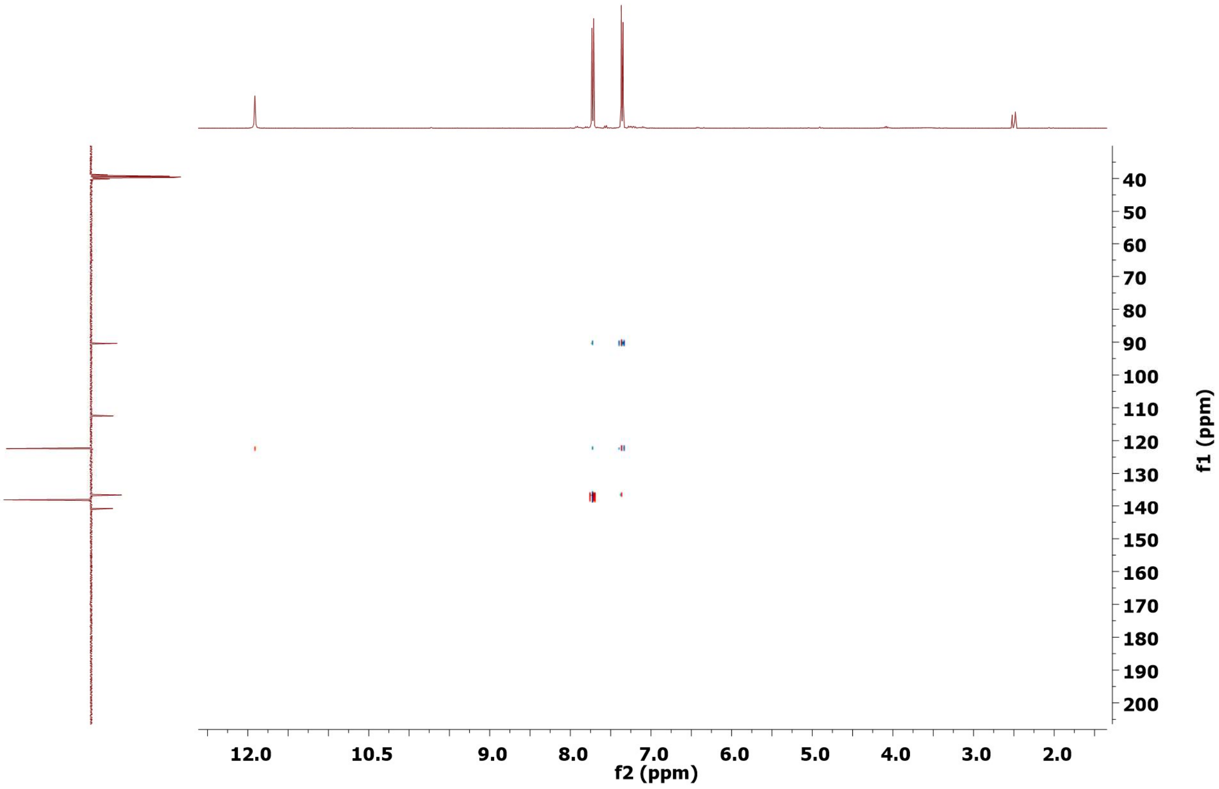

^{1}H NMR (DMSO-d6) spectrum of (3-chlorophenyl)carbamoyl cyanide (2r)

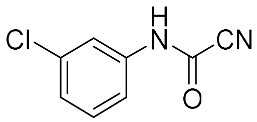

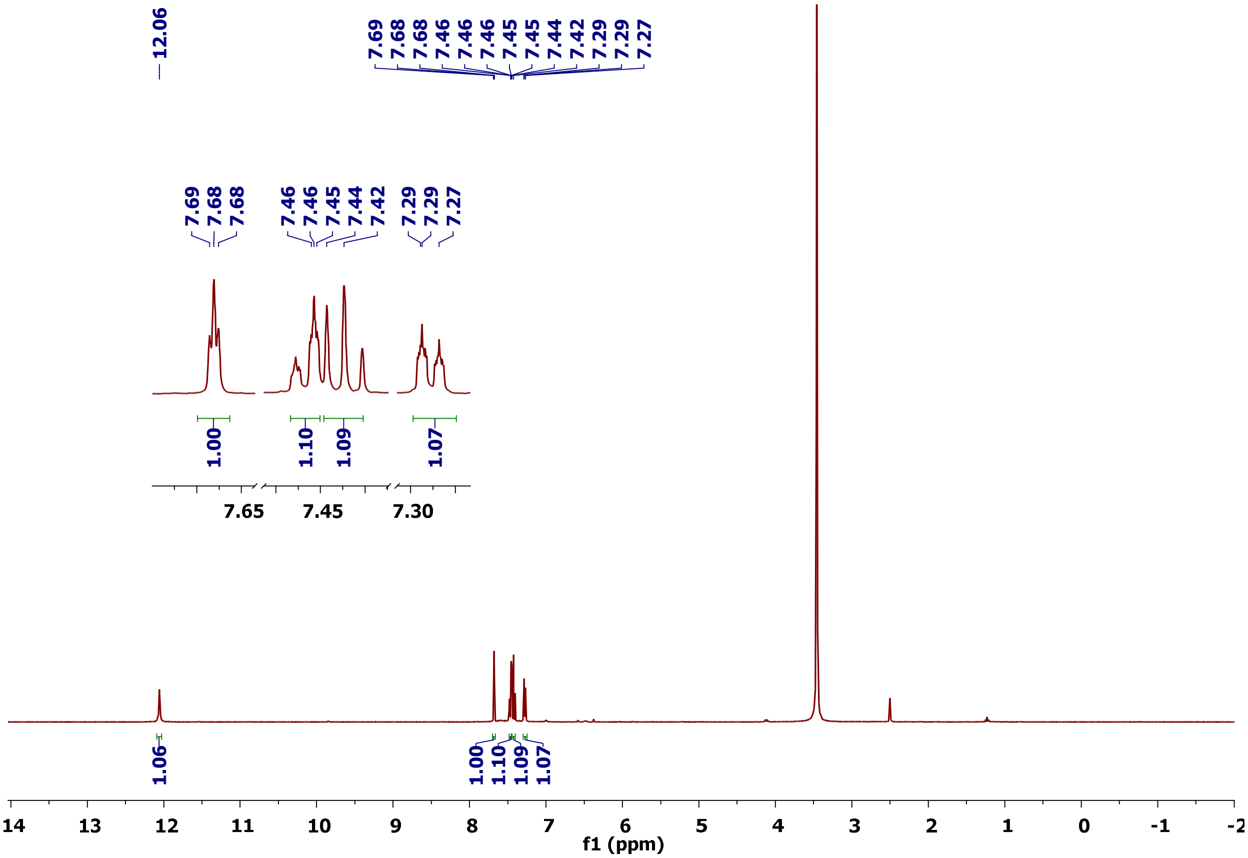

^{13}C NMR (DMSO-d6) spectrum of (3-chlorophenyl)carbamoyl cyanide (2r)

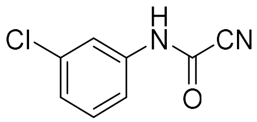

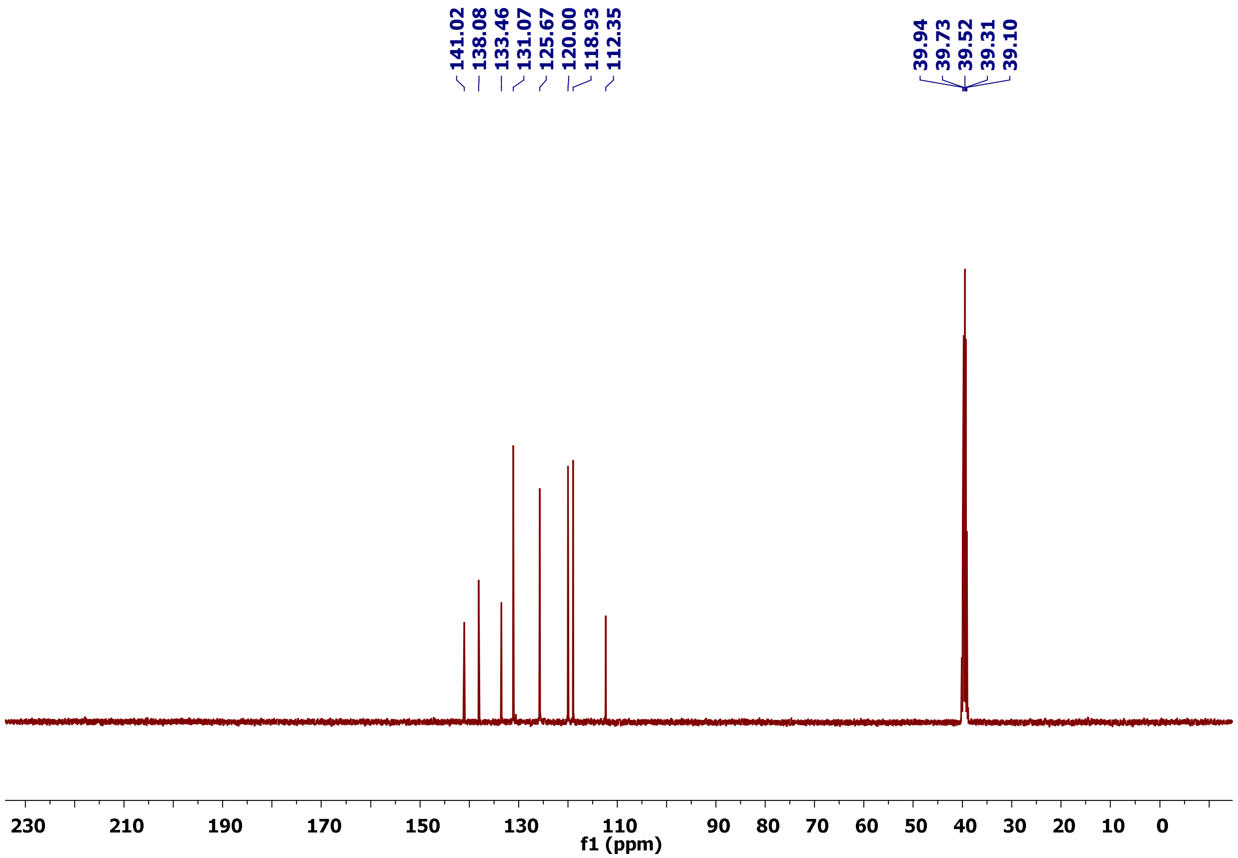

^{13}C CRAPT NMR (DMSO-d6) spectrum of (3-chlorophenyl)carbamoyl cyanide (2r)

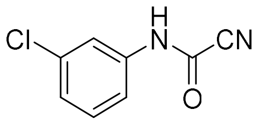

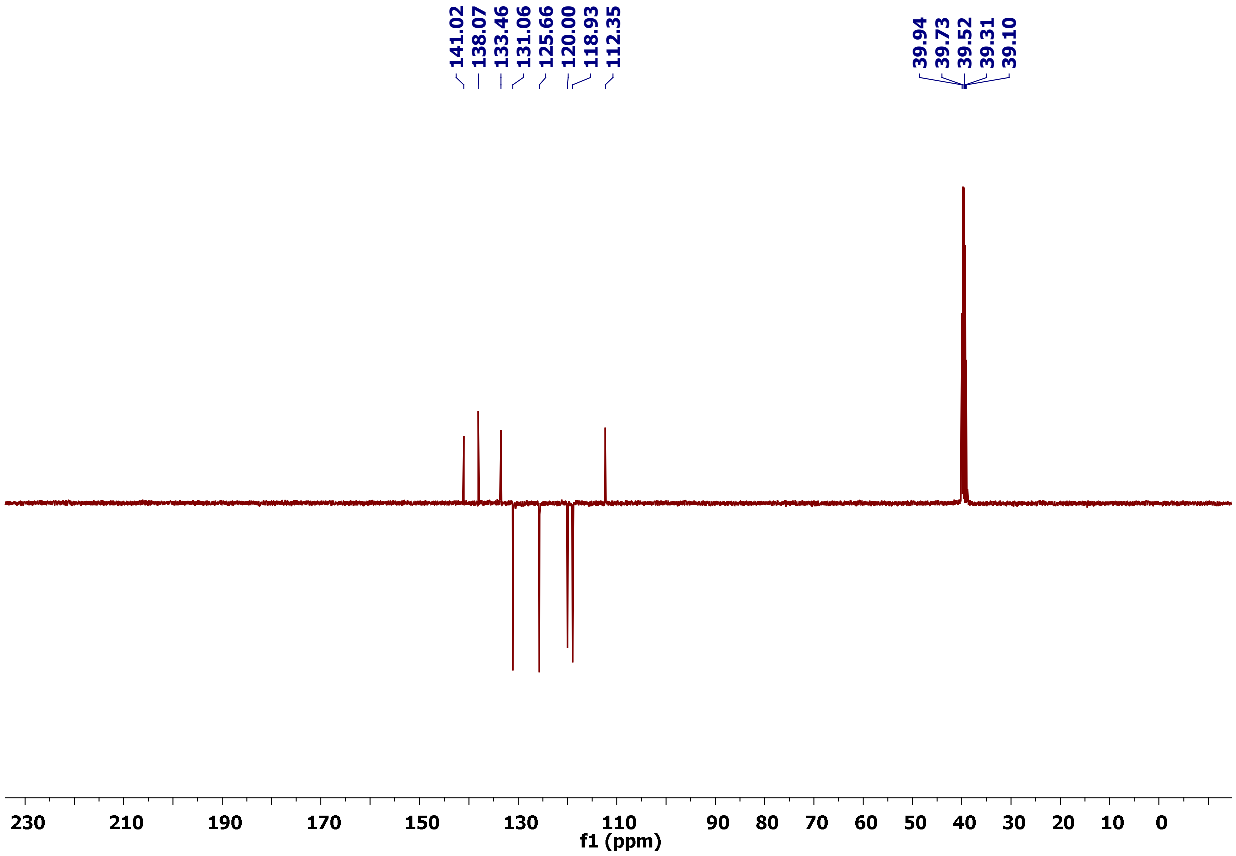

^{1}H-^{1}H gDQCOSY NMR (DMSO-d6) spectrum of (3-chlorophenyl)carbamoyl cyanide (2r)

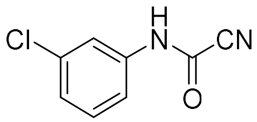

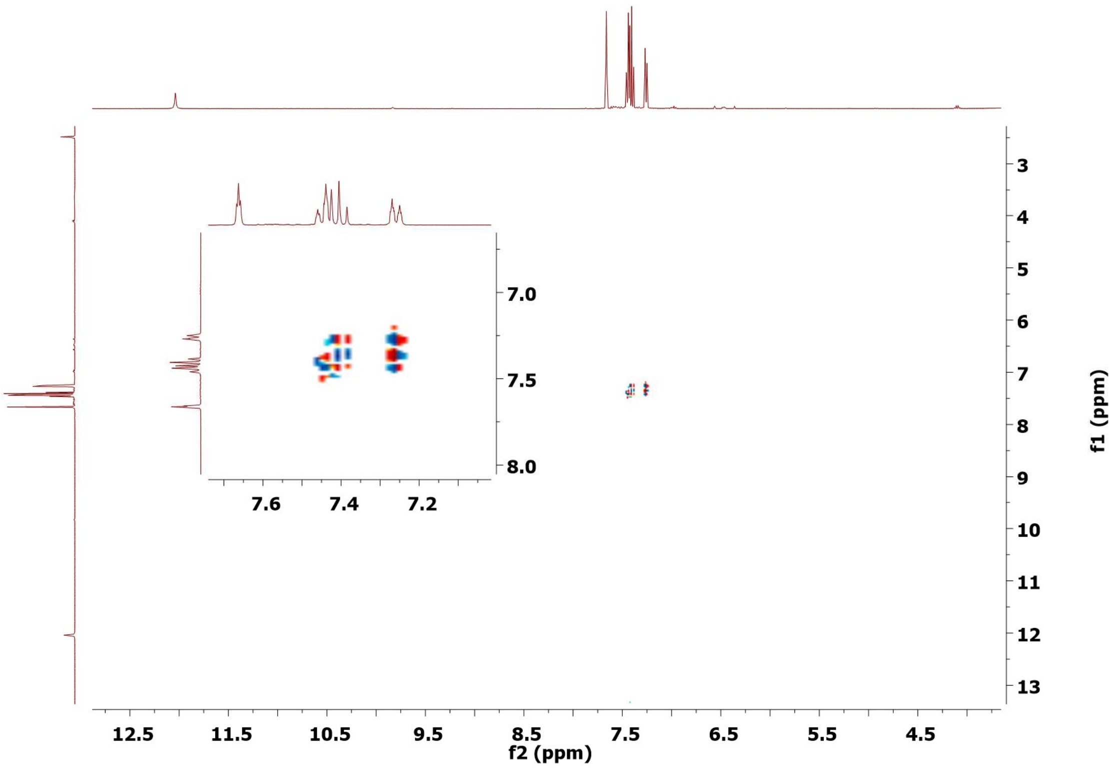

^{1}H-^{13}C-gHSQC NMR (DMSO-d6) spectrum of (3-chlorophenyl)carbamoyl cyanide (2r)

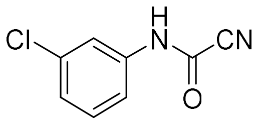

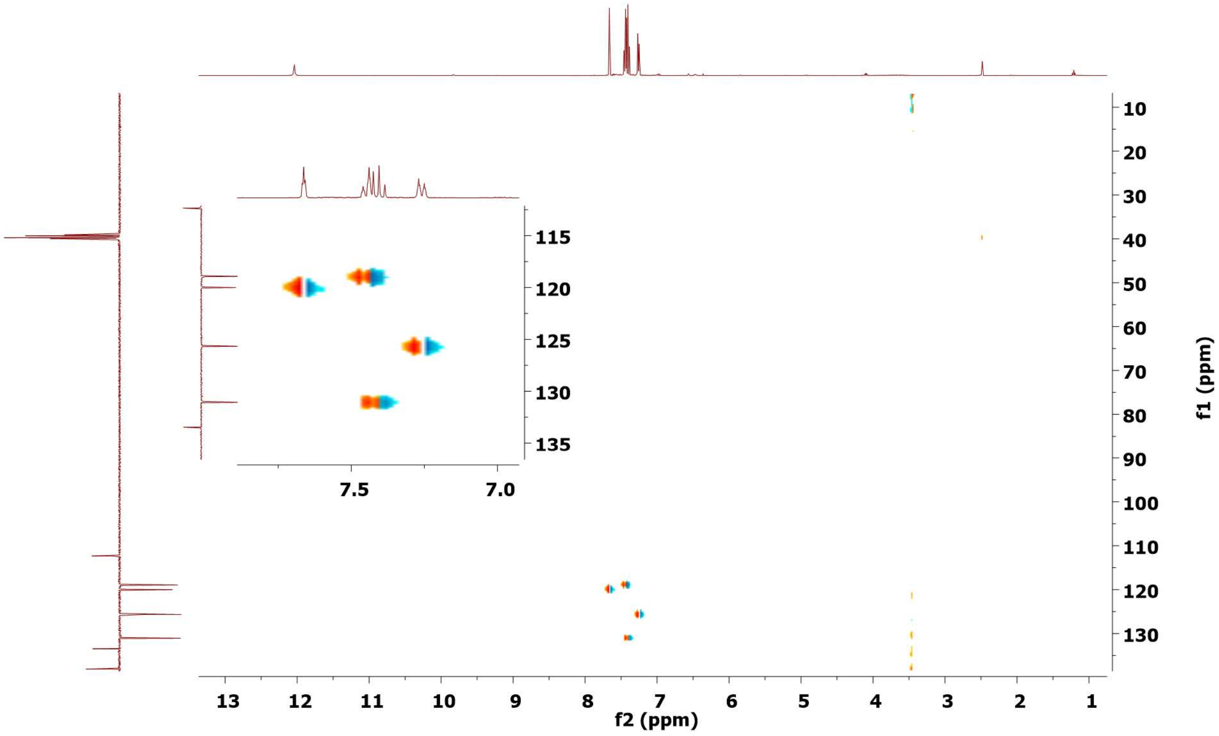

^1H-^{13}C-gHMBC NMR (DMSO-d6) spectrum of (3-chlorophenyl)carbamoyl cyanide (2r)

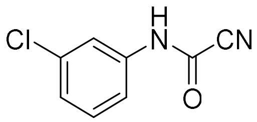

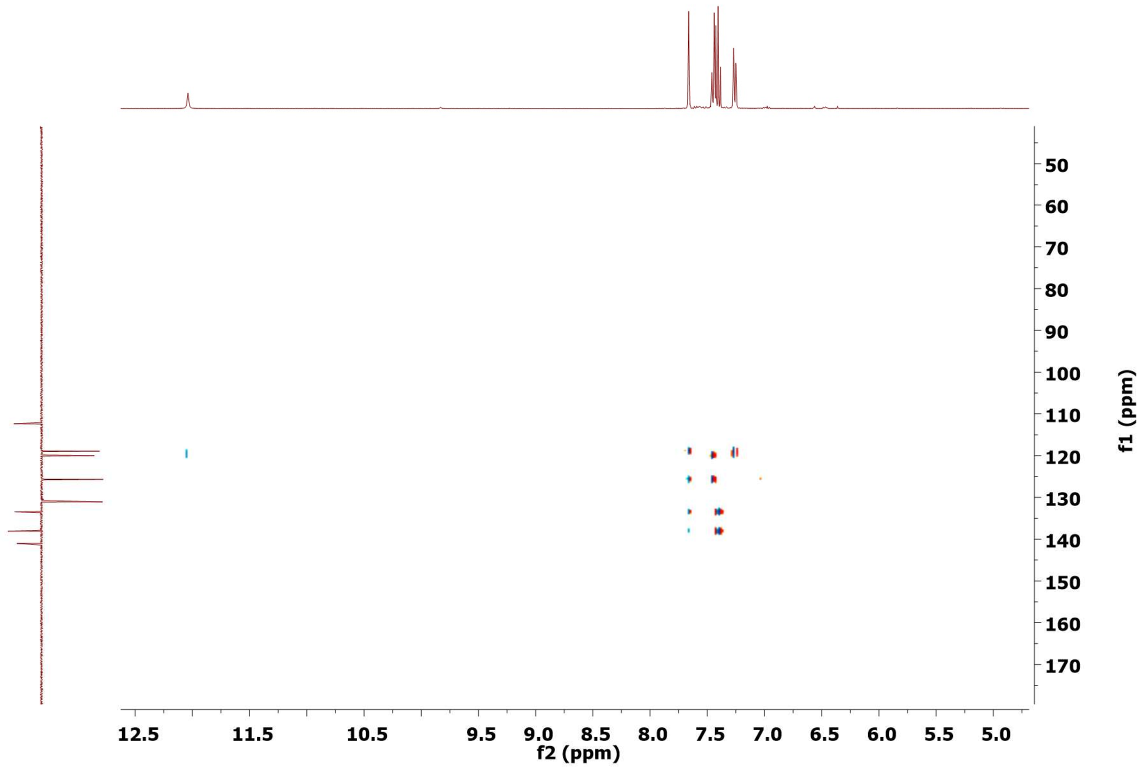

^{1}H NMR (DMSO-d6) spectrum of (3-bromophenyl)carbamoyl cyanide (2s)

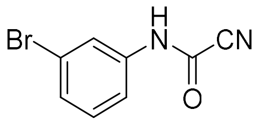

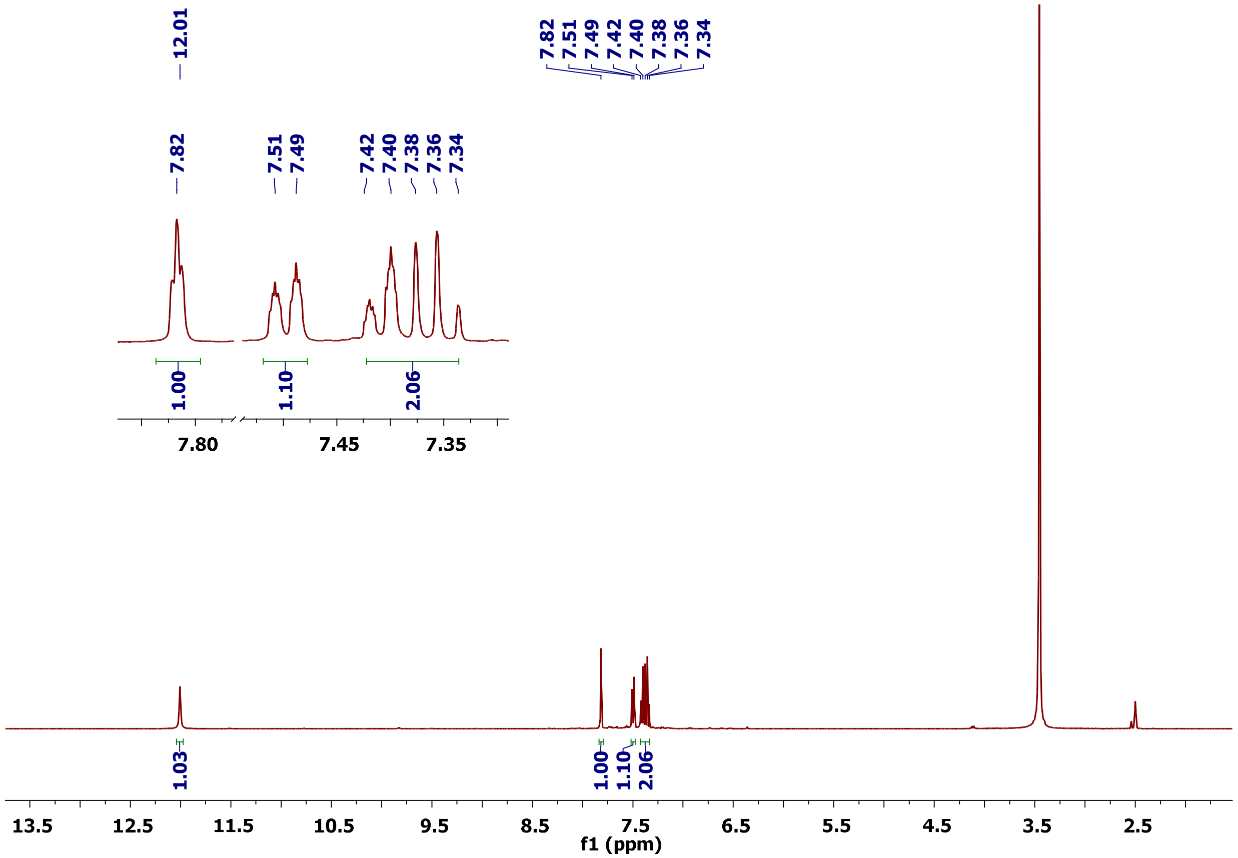

^{13}C NMR (DMSO-d6) spectrum of (3-bromophenyl)carbamoyl cyanide (2s)

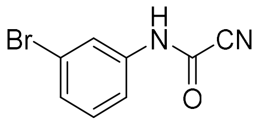

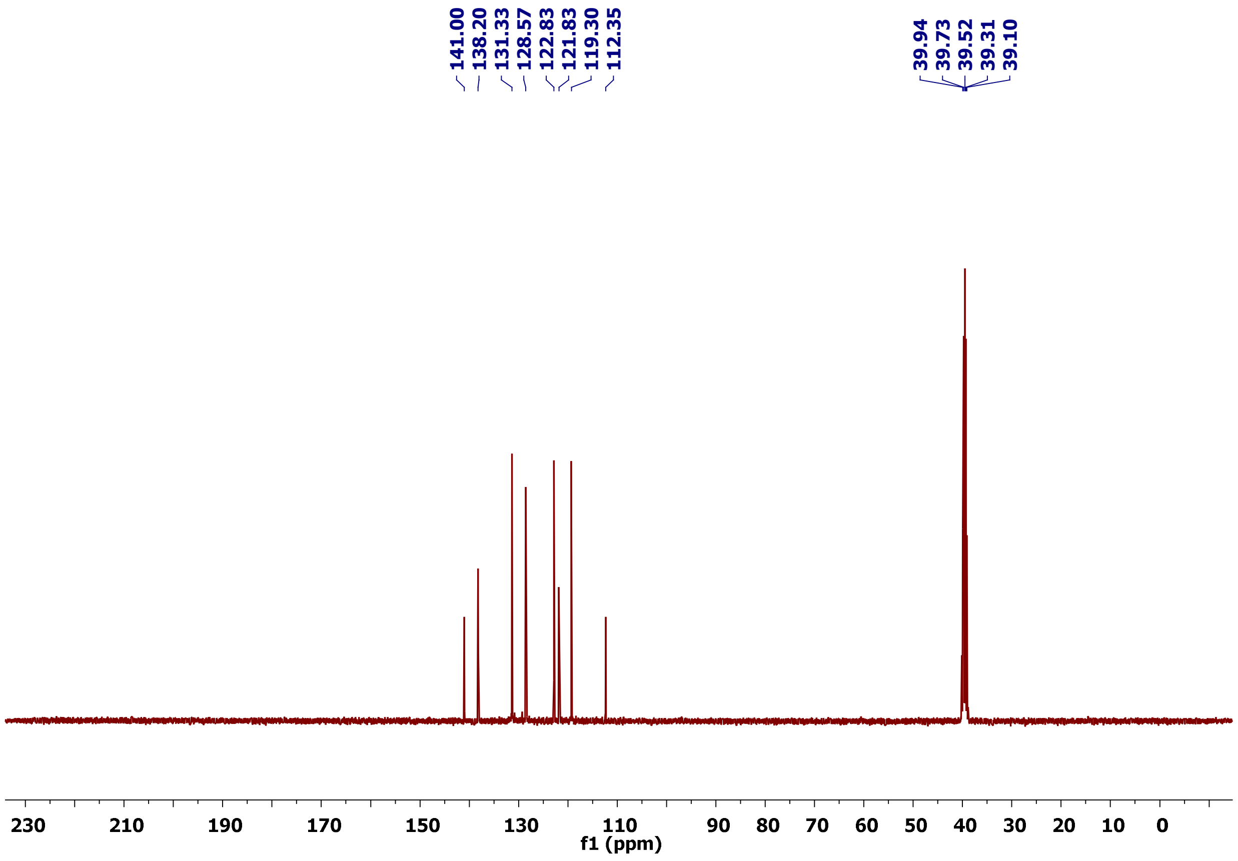

^{13}C CRAPT NMR (DMSO-d6) spectrum of (3-bromophenyl)carbamoyl cyanide (2s)

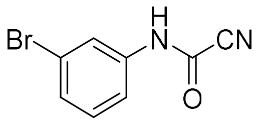

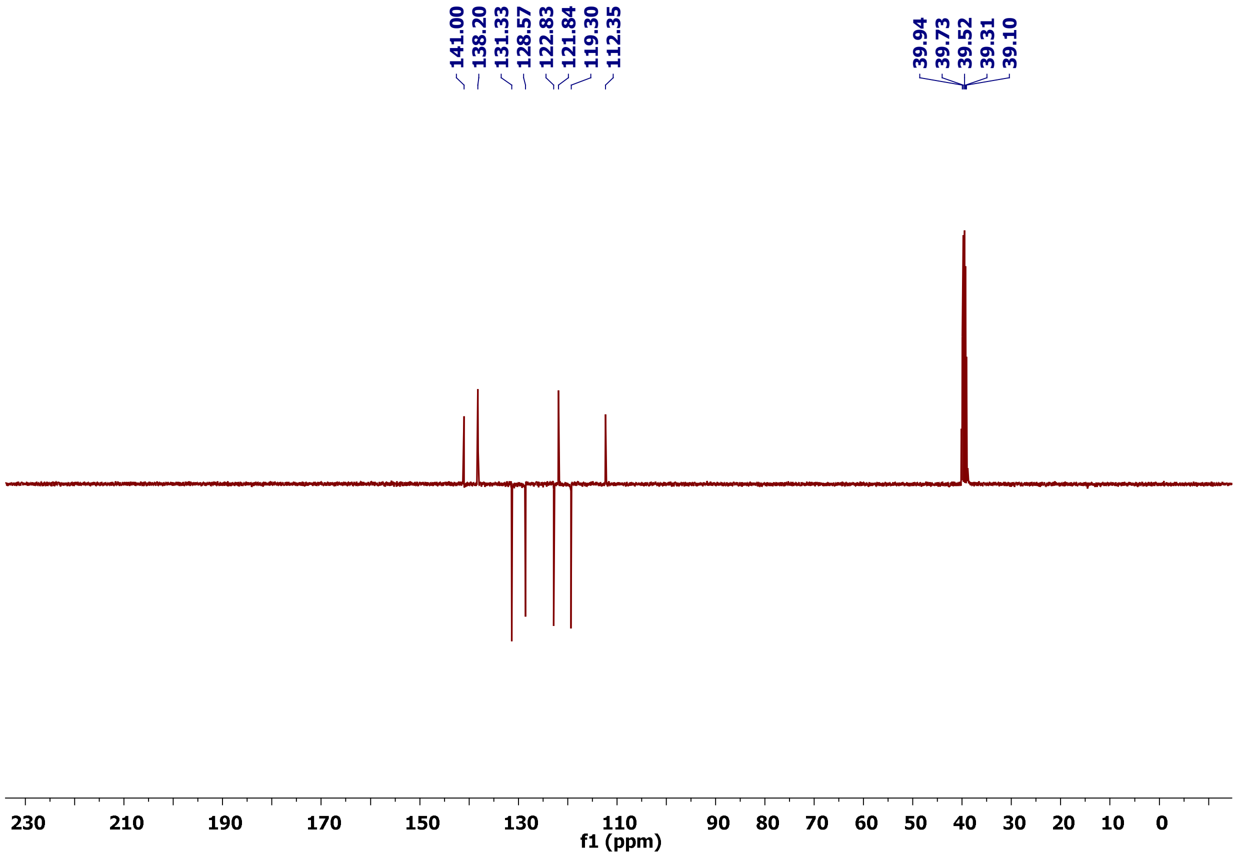

^{1}H-^{1}H gDQCOSY NMR (DMSO-d6) spectrum of (3-bromophenyl)carbamoyl cyanide (2s)

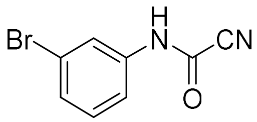

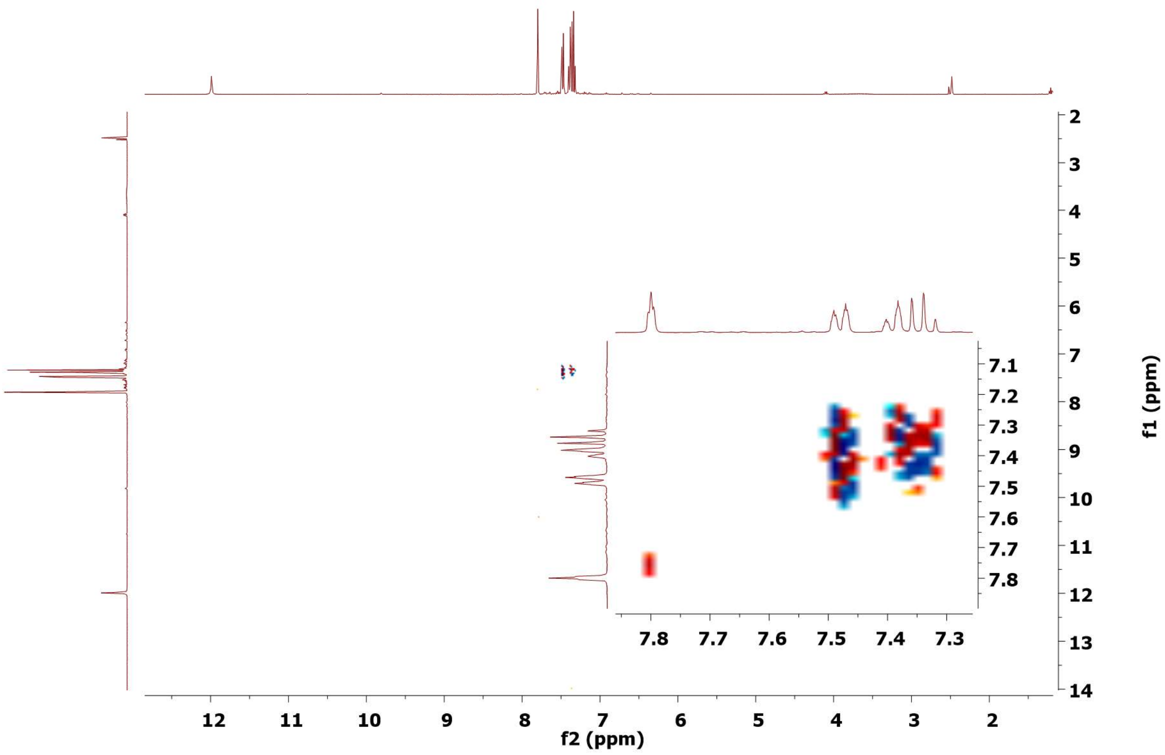

^{1}H-^{13}C-gHSQC NMR (DMSO-d6) spectrum of (3-bromophenyl)carbamoyl cyanide (2s)

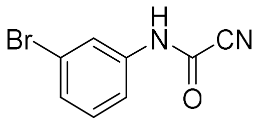

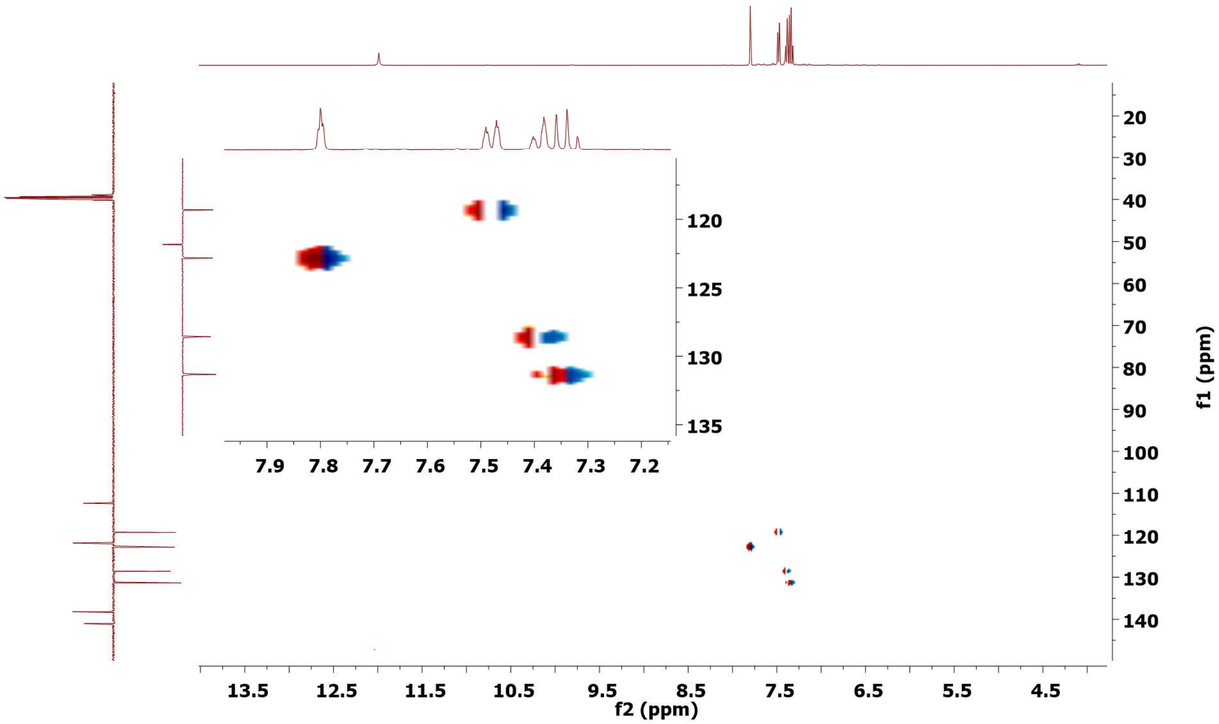

^{1}H-^{13}C-gHMBC NMR (DMSO-d6) spectrum of (3-bromophenyl)carbamoyl cyanide (2s)

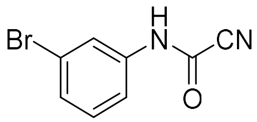

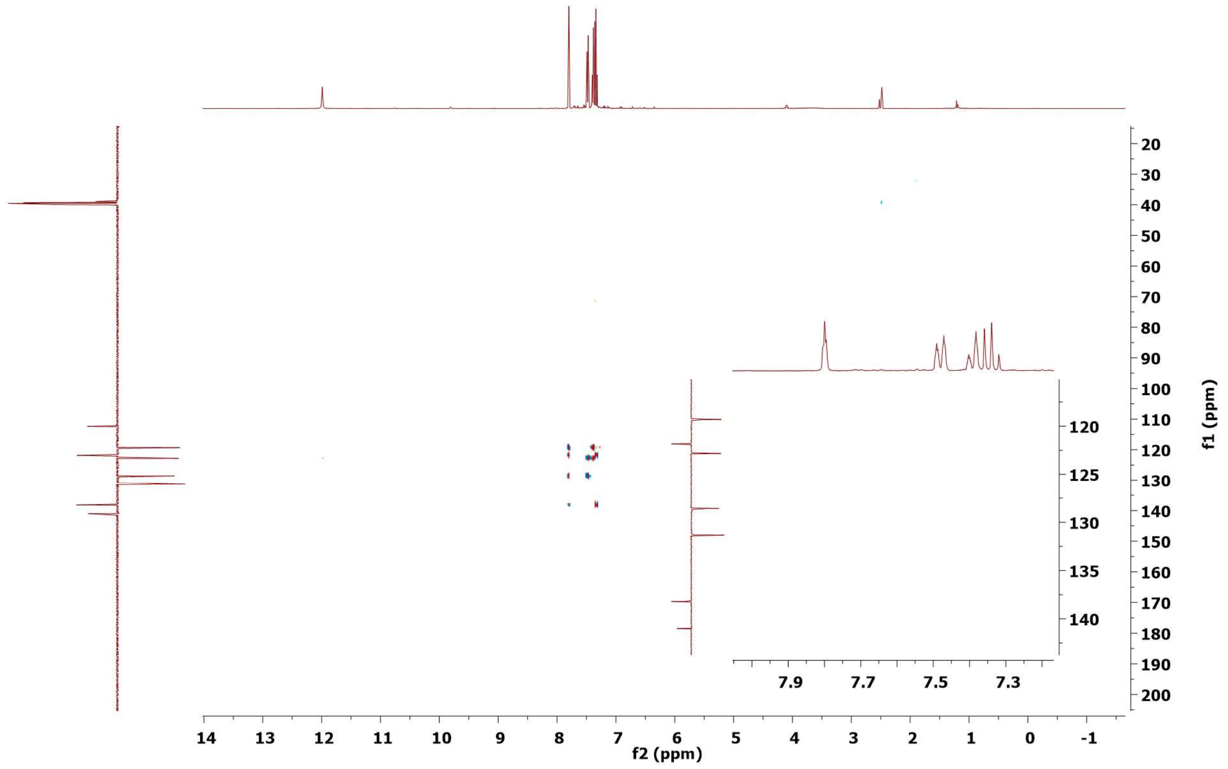

^{1}H NMR (DMSO-d6) spectrum of (3-iodophenyl)carbamoyl cyanide (2t)

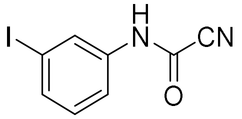

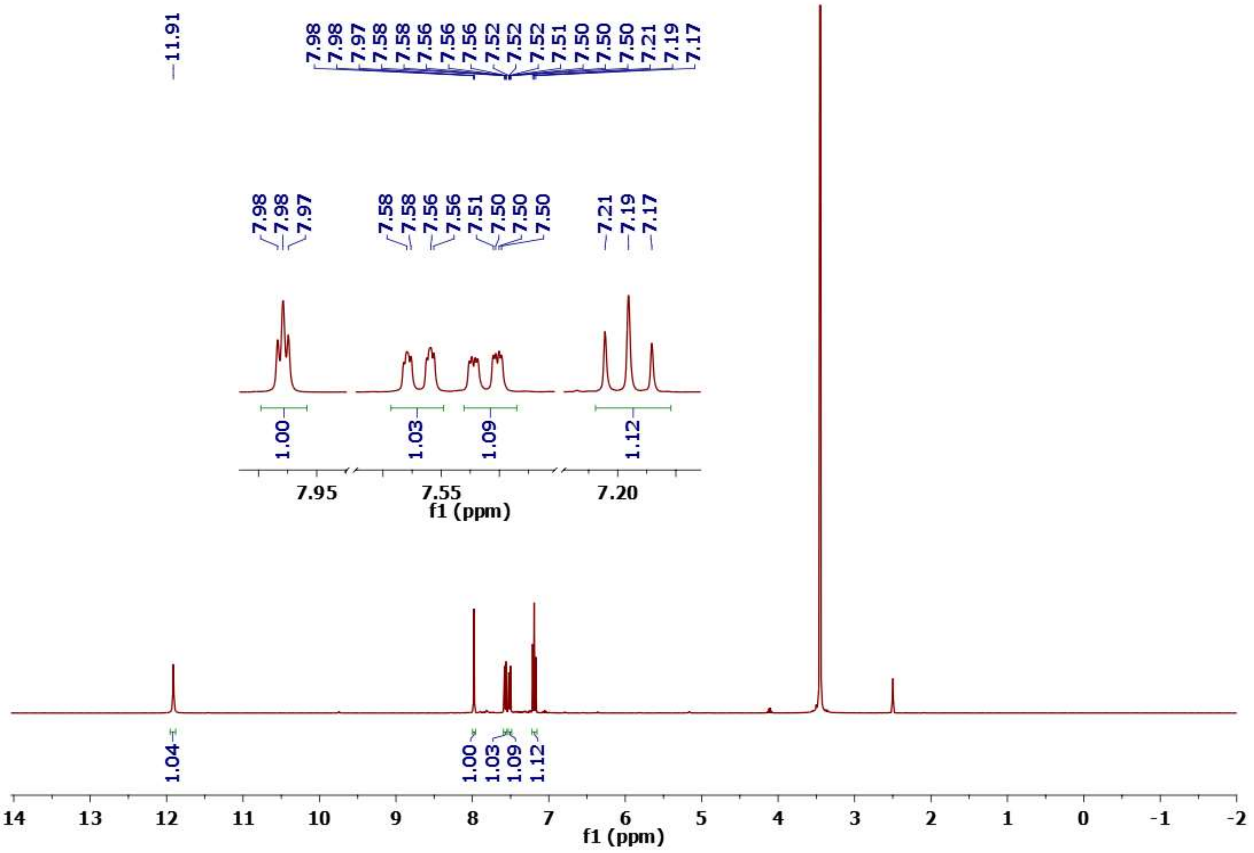

^{13}C NMR (DMSO-d6) spectrum of (3-iodophenyl)carbamoyl cyanide (2t)

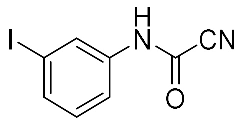

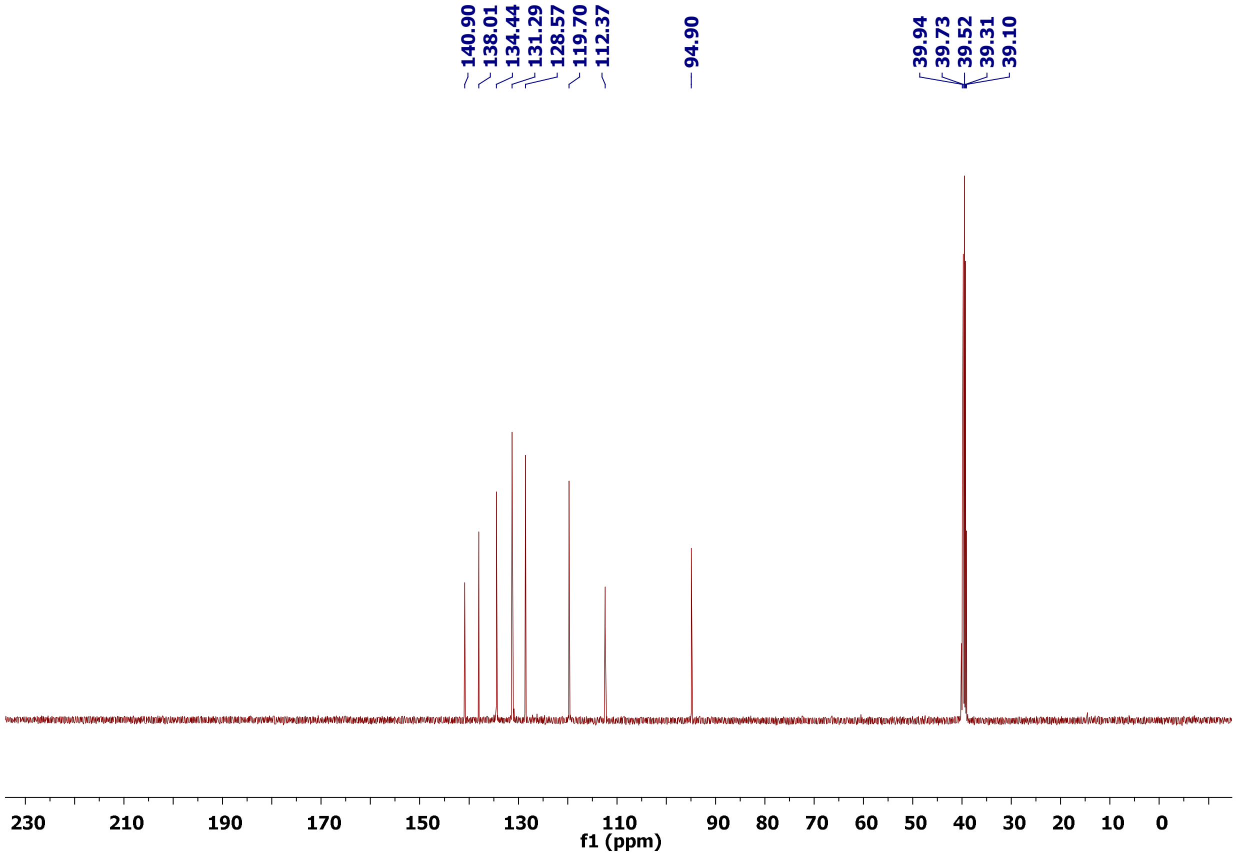

^{13}C CRAPT NMR (DMSO-d6) spectrum of (3-iodophenyl)carbamoyl cyanide (2t)

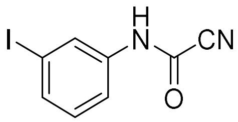

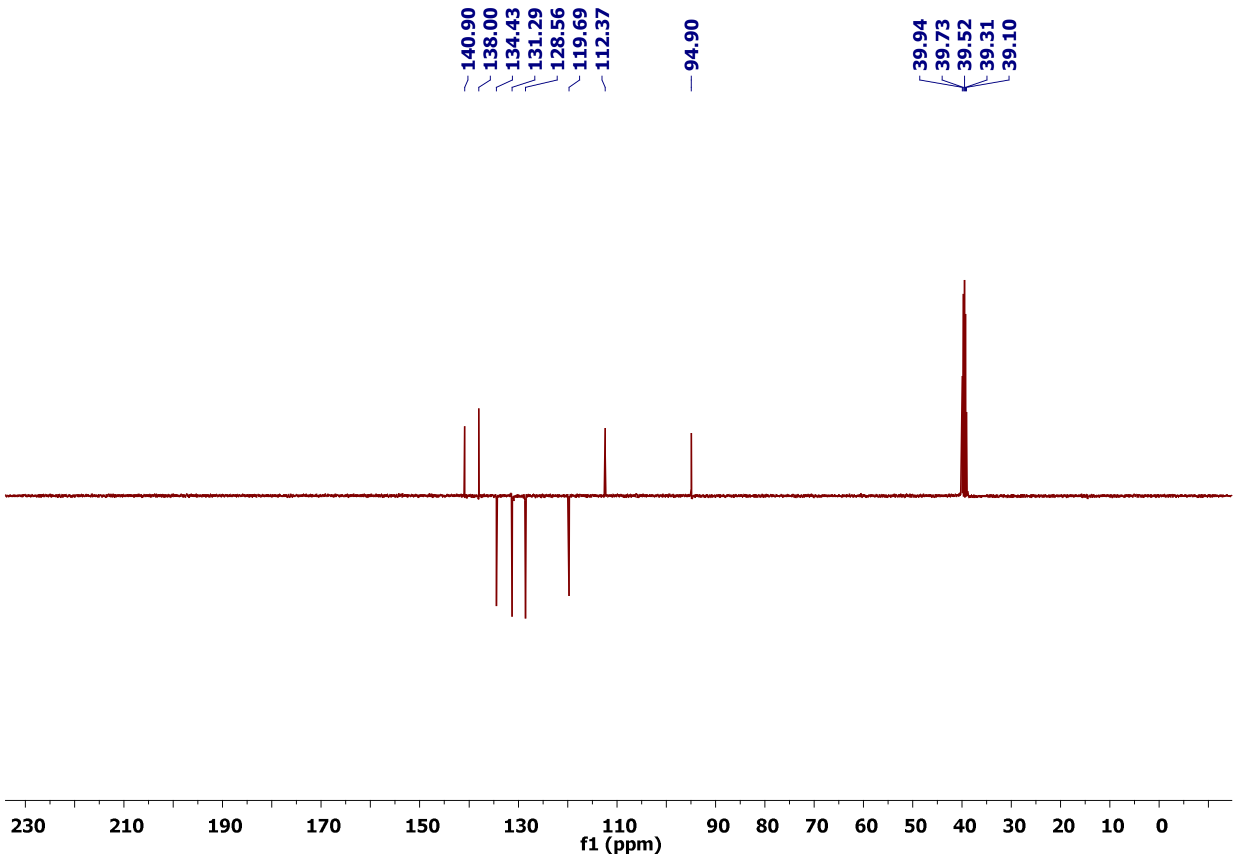

^{1}H-^{1}H gDQCOSY NMR (DMSO-d6) spectrum of (3-iodophenyl)carbamoyl cyanide (2t)

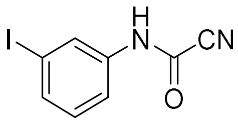

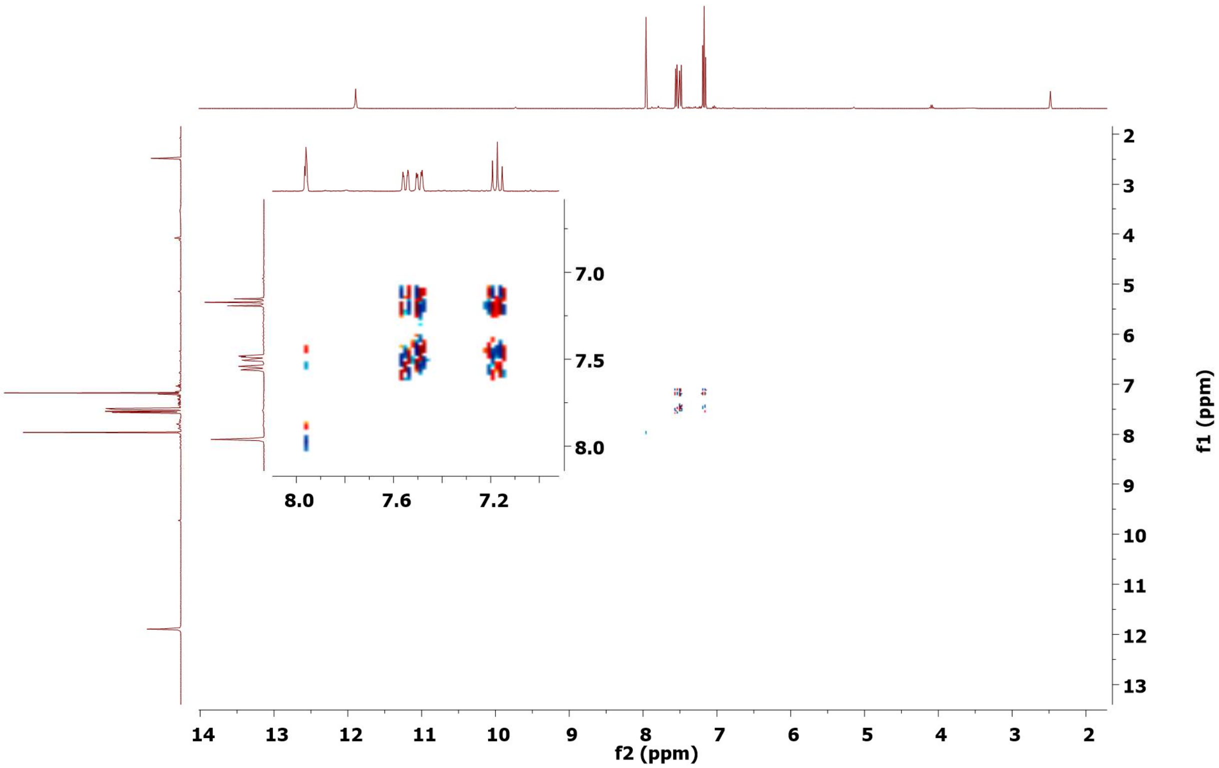

^{1}H-^{13}C-gHSQC NMR (DMSO-d6) spectrum of (3-iodophenyl)carbamoyl cyanide (2t)

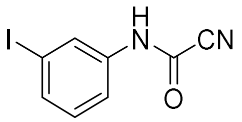

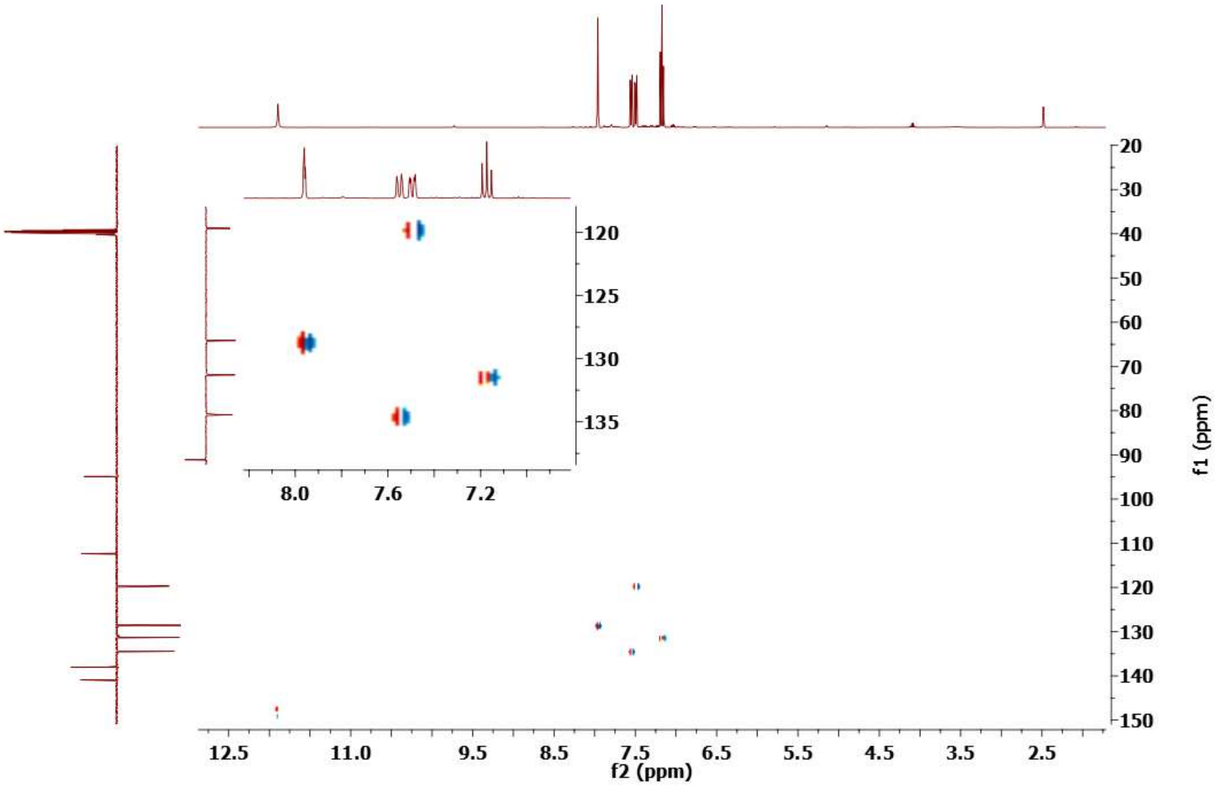

^{1}H-^{13}C-gHMBC NMR (DMSO-d6) spectrum of (3-iodophenyl)carbamoyl cyanide (2t)

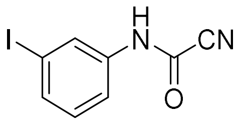

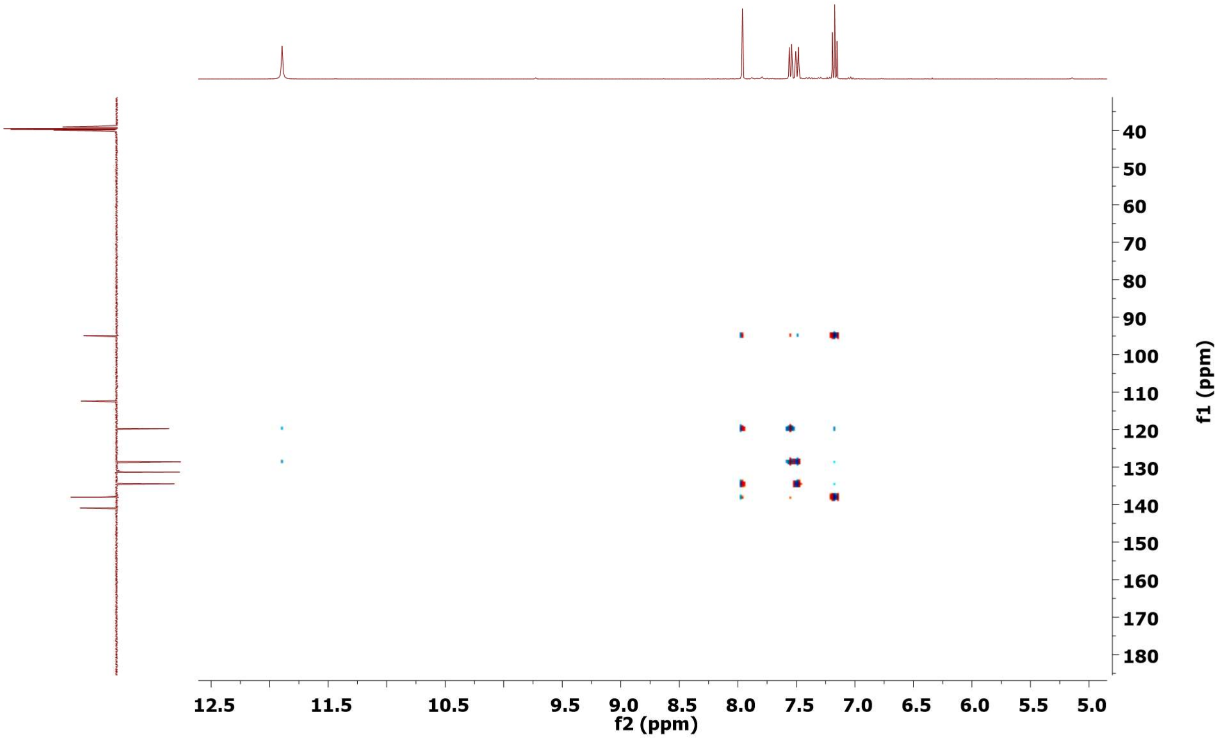

^{1}H NMR (DMSO-d6) spectrum of (3-cyanophenyl)carbamoyl cyanide (2u)

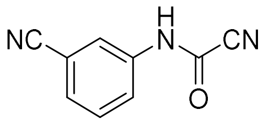

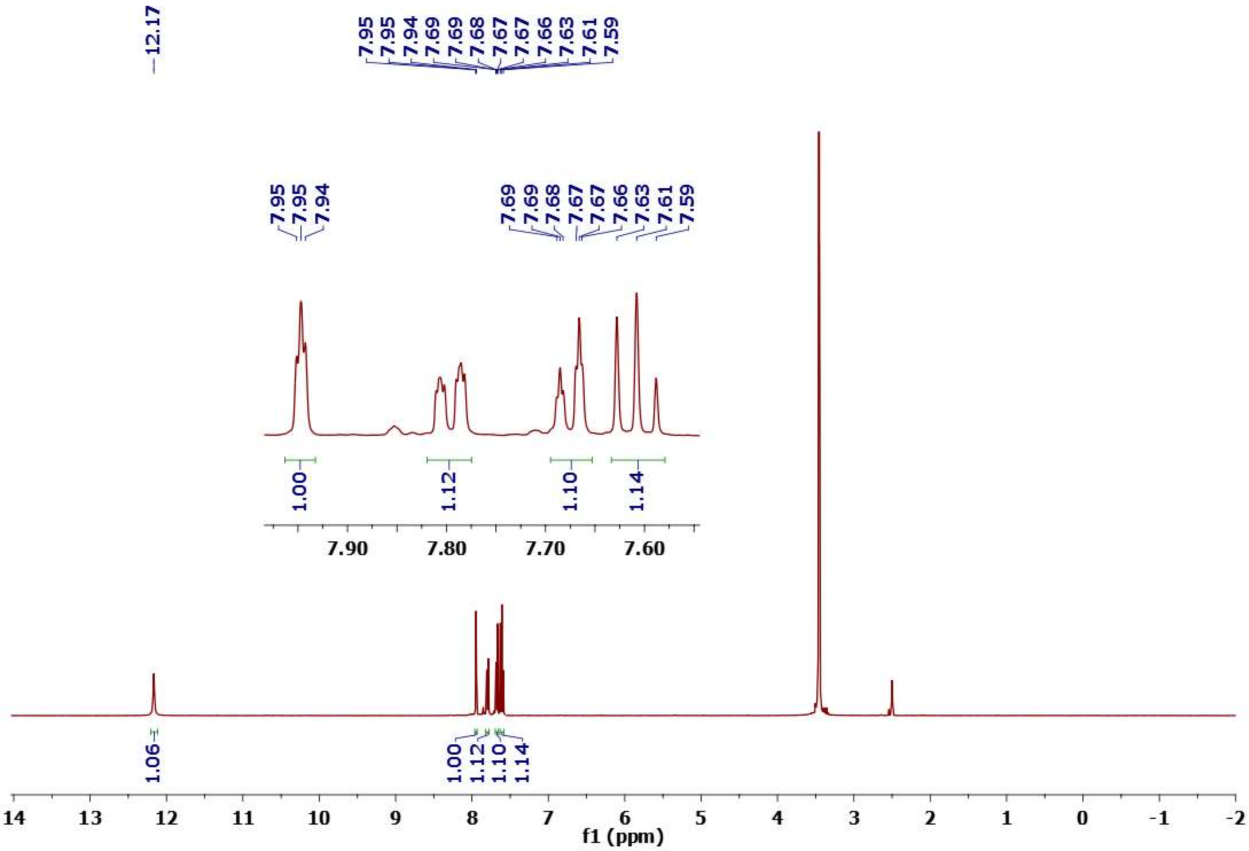

^{13}C NMR (DMSO-d6) spectrum of (3-cyanophenyl)carbamoyl cyanide (2u)

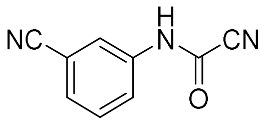

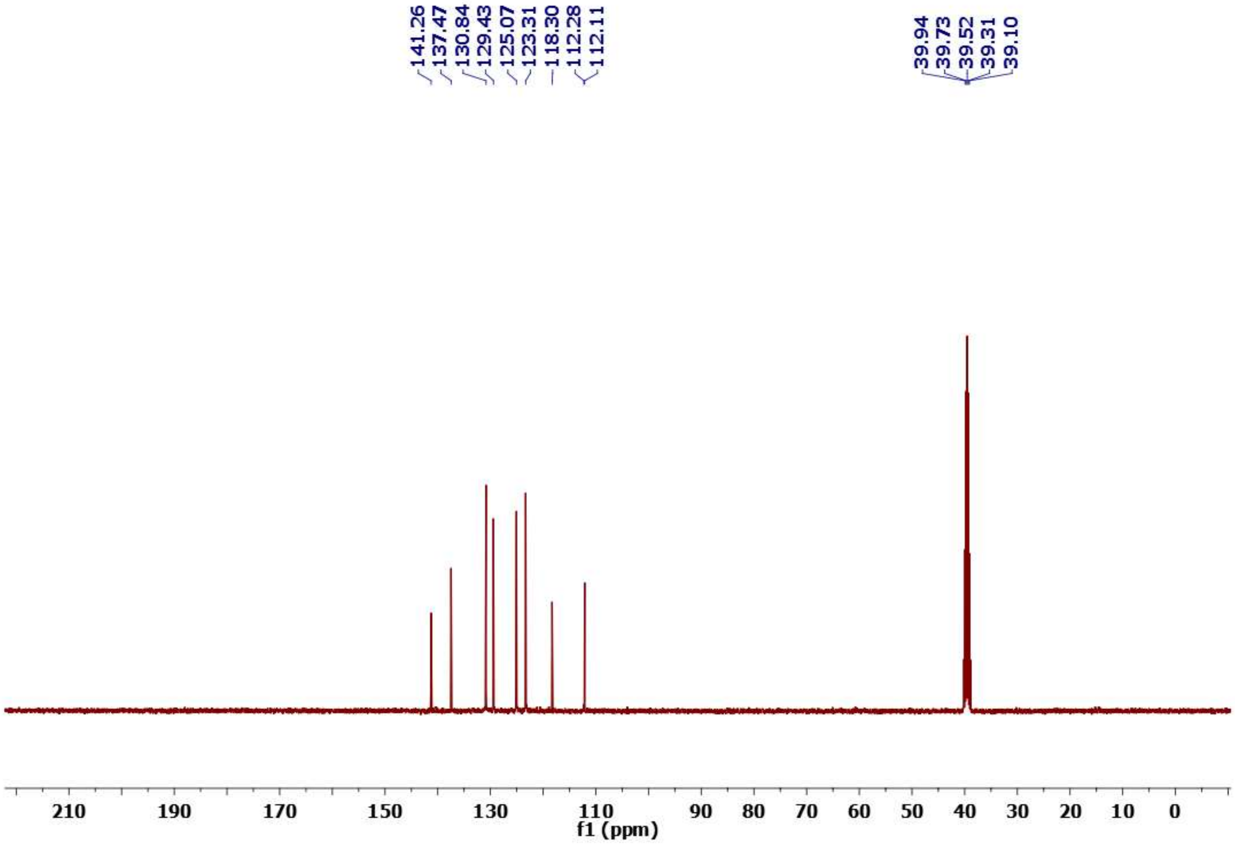

^{13}C CRAPT NMR (DMSO-d6) spectrum of (3-cyanophenyl)carbamoyl cyanide (2u)

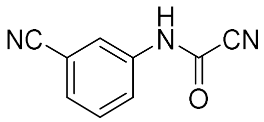

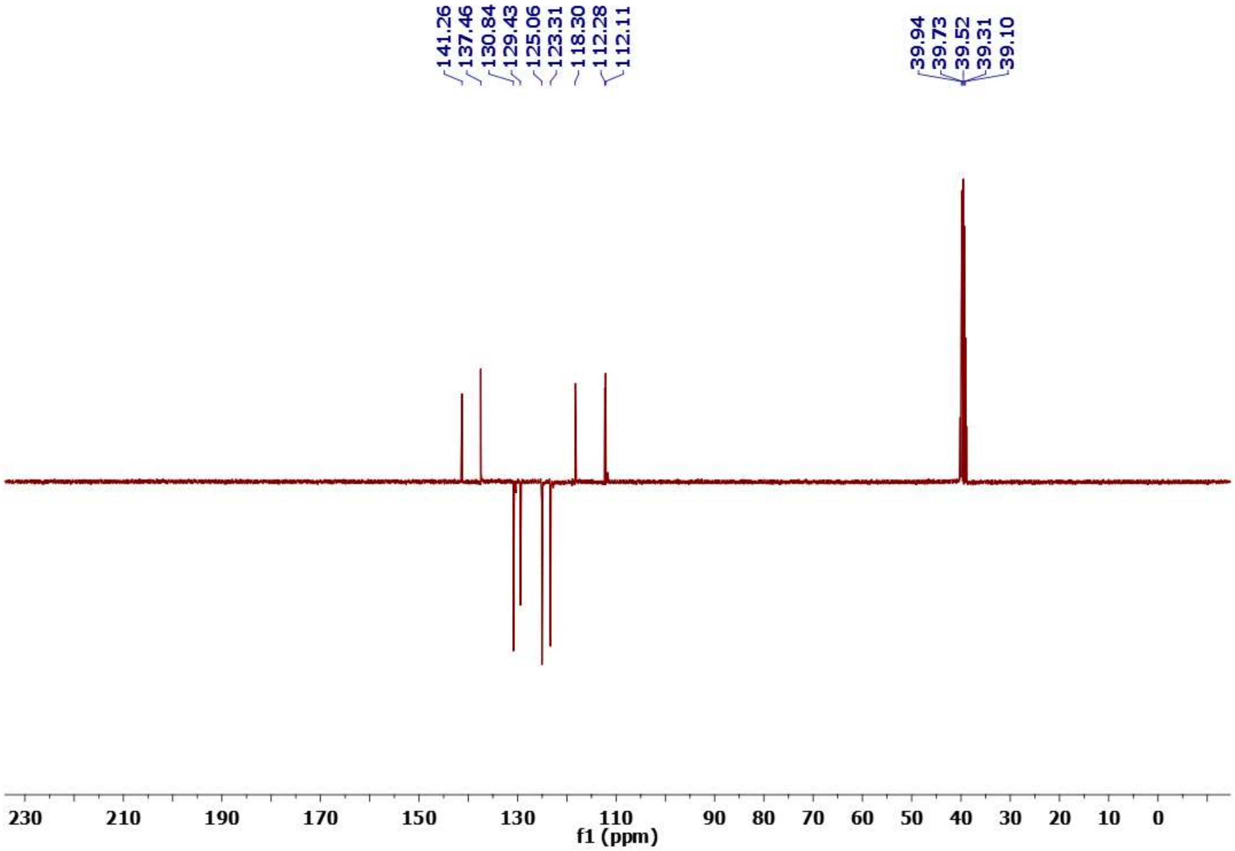

^{1}H-^{1}H gDQCOSY NMR (DMSO-d6) spectrum of (3-cyanophenyl)carbamoyl cyanide (2u)

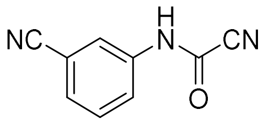

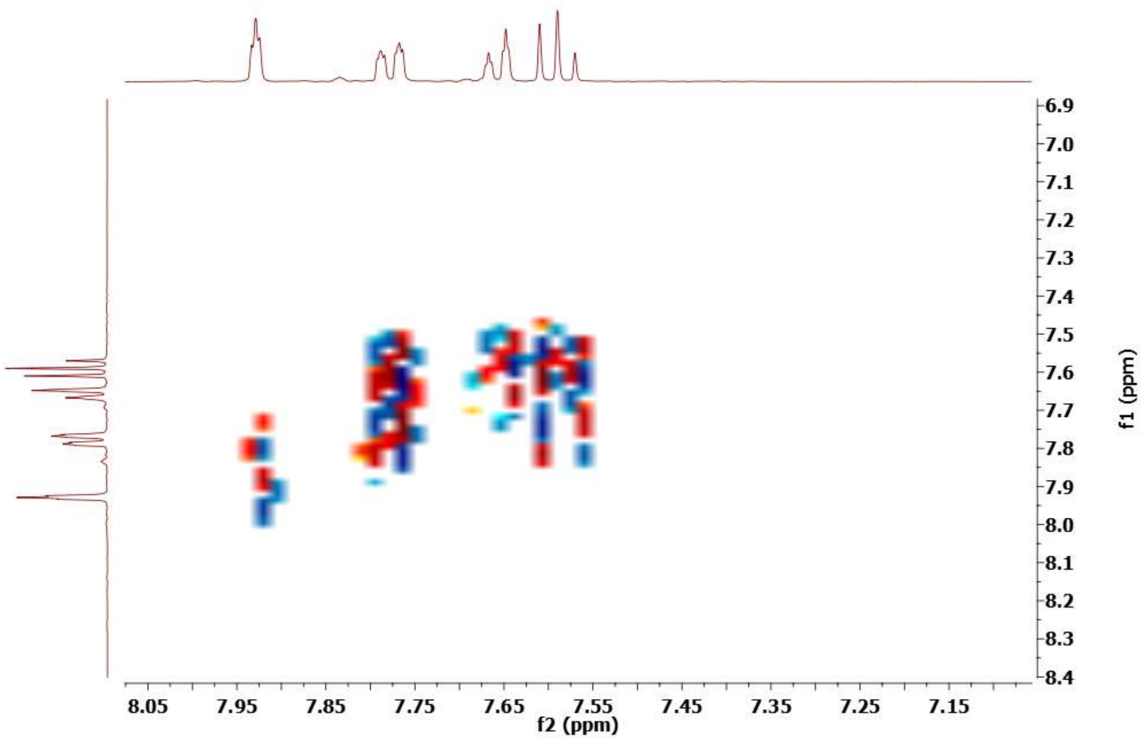

^{1}H-^{13}C-gHSQC NMR (DMSO-d6) spectrum of (3-cyanophenyl)carbamoyl cyanide (2u)

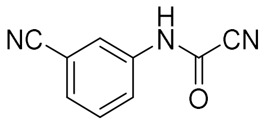

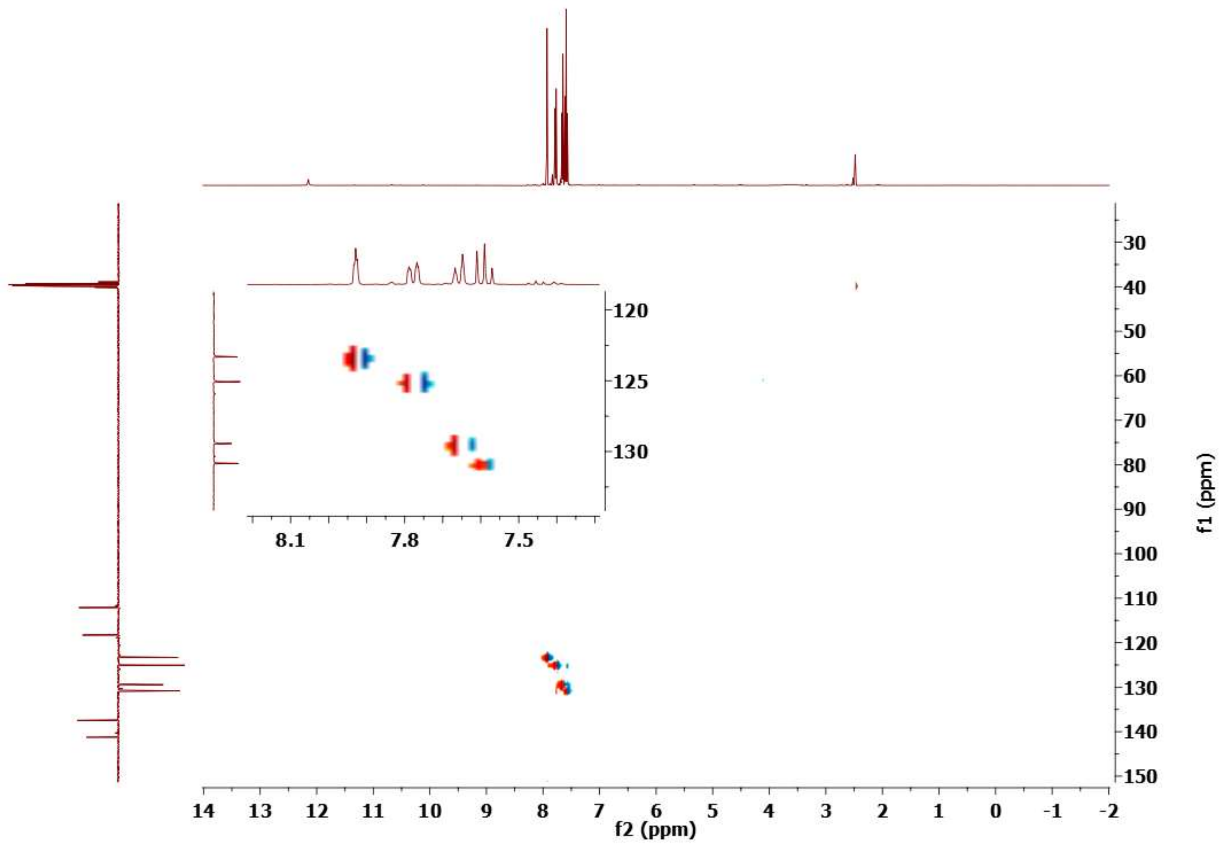

^{1}H-^{13}C-gHMBC NMR (DMSO-d6) spectrum of (3-cyanophenyl)carbamoyl cyanide (2u)

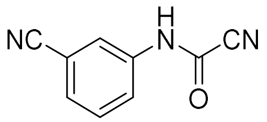

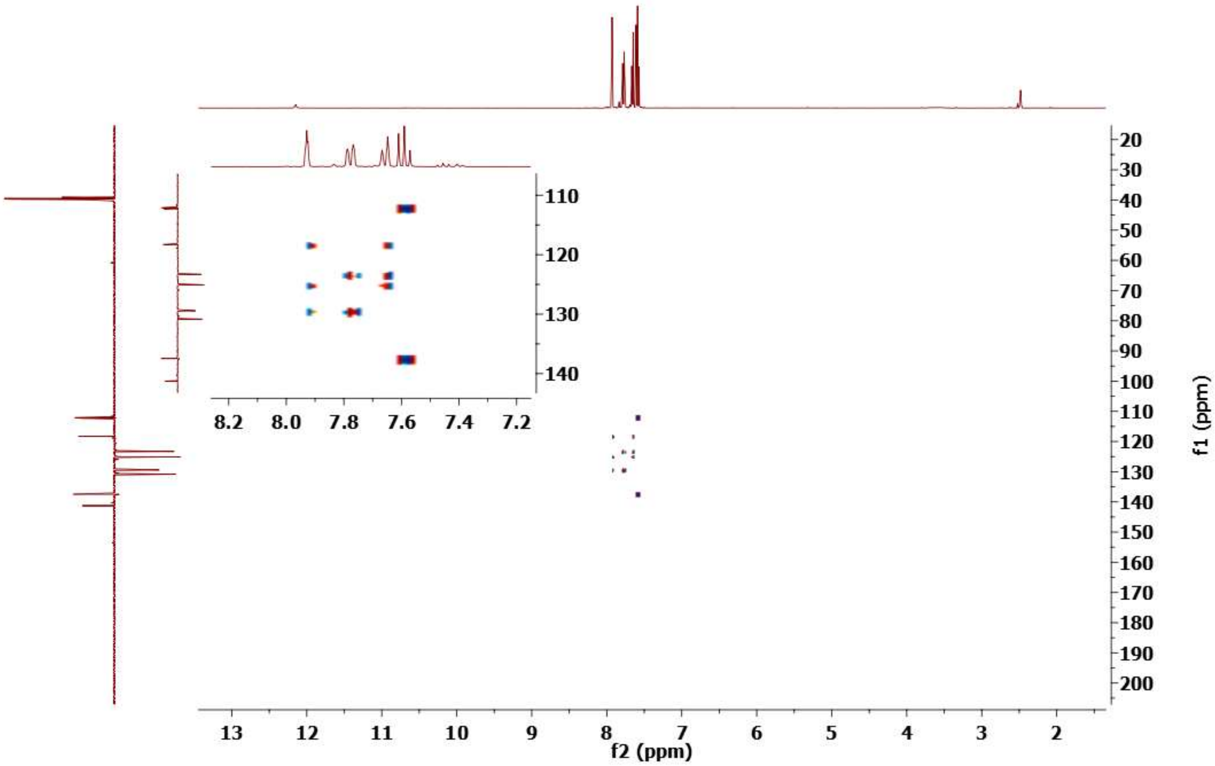

^{1}H NMR (DMSO-d6) spectrum of (3-(trifluoromethyl)phenyl)carbamoyl cyanide (2v)

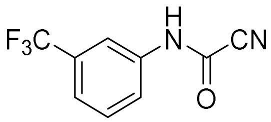

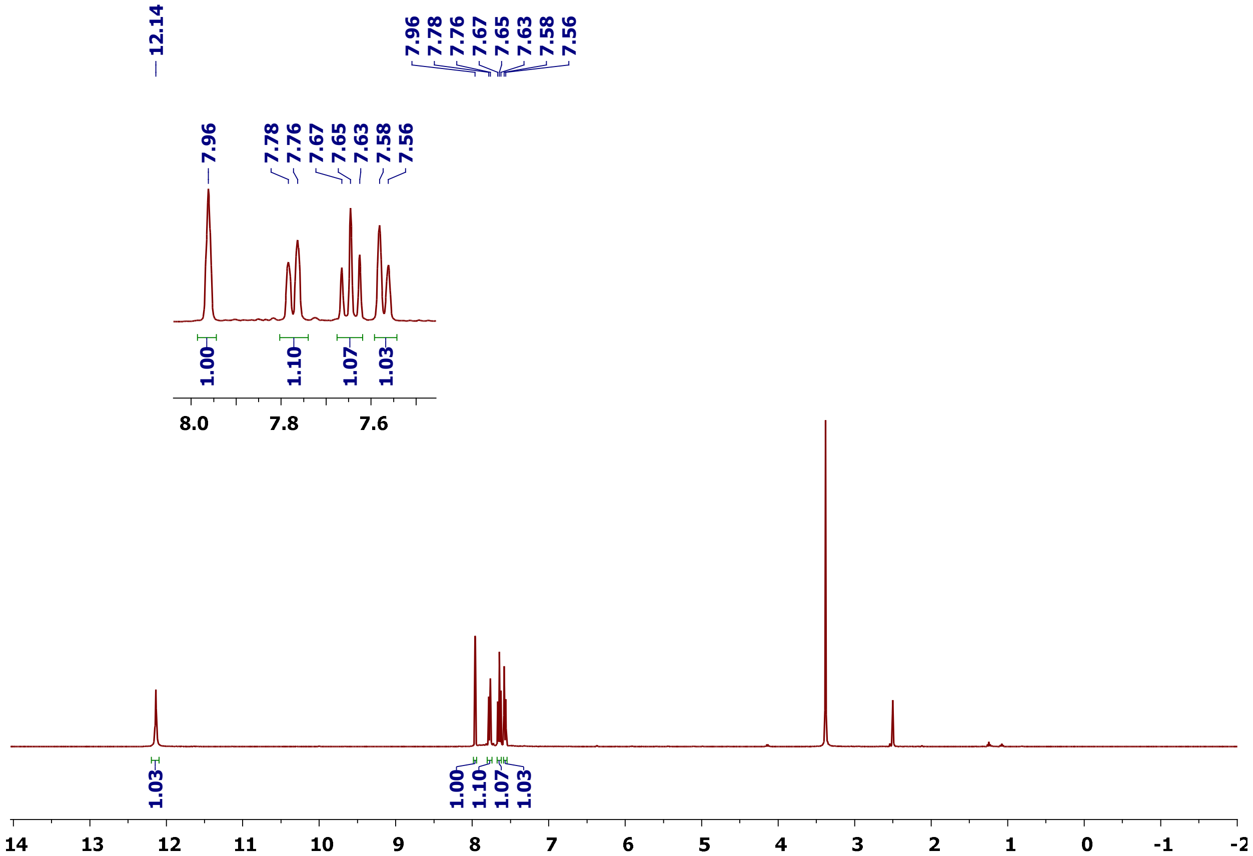

^{13}C NMR (DMSO-d6) spectrum of (3-(trifluoromethyl)phenyl)carbamoyl cyanide (2v)

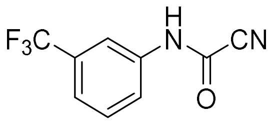

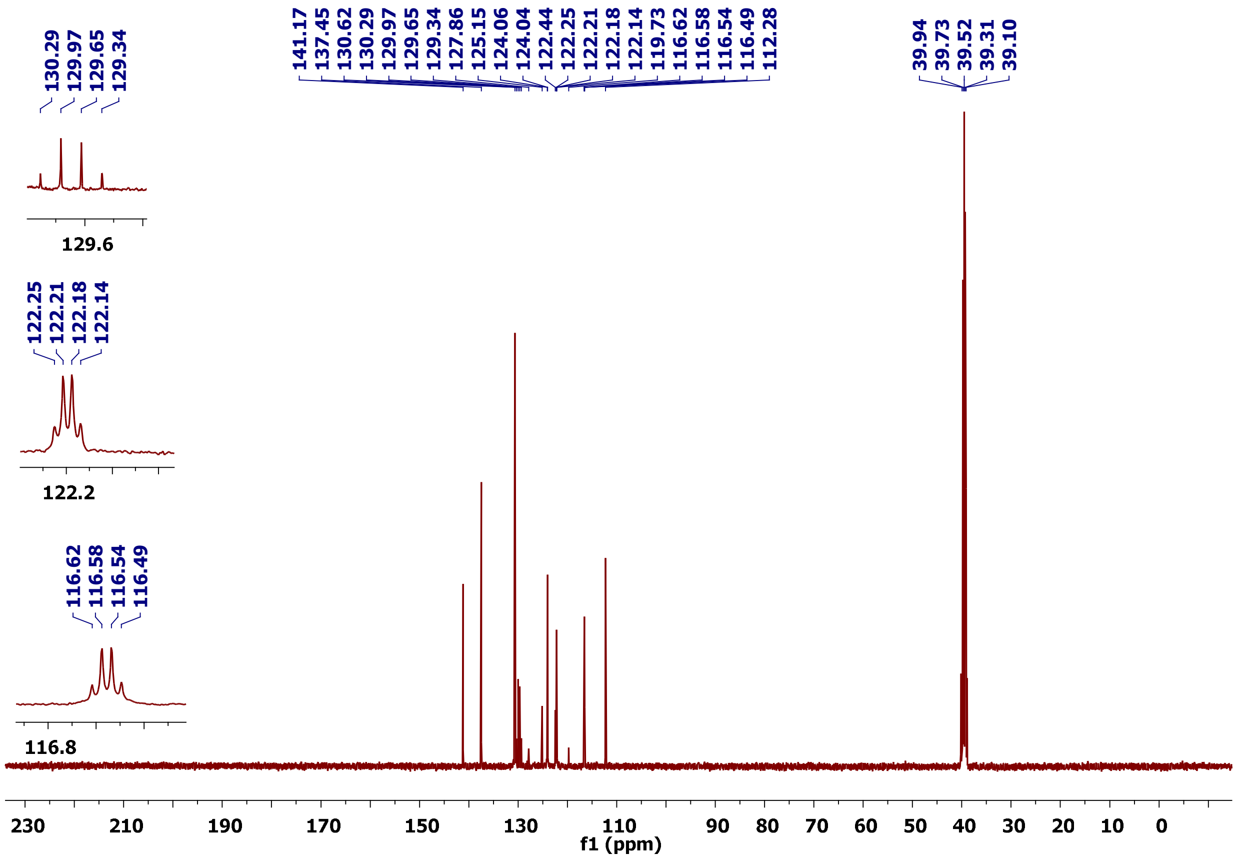

^{13}C CRAPT NMR (DMSO-d6) spectrum of (3-(trifluoromethyl)phenyl)carbamoyl cyanide (2v)

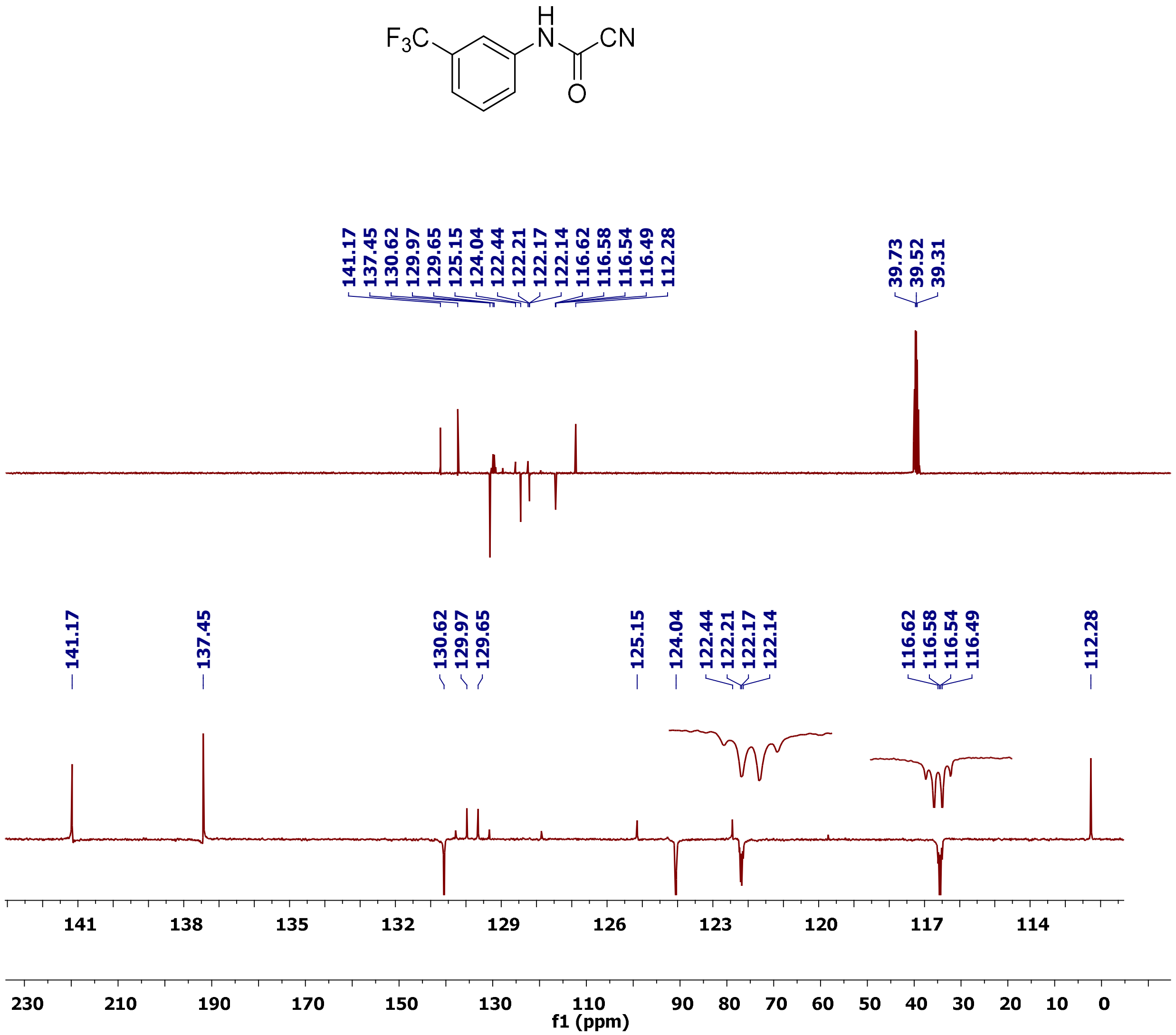

^{1}H-^{1}H gDQCOSY NMR (DMSO-d6) spectrum of (3-(trifluoromethyl)phenyl)carbamoyl cyanide (2v)

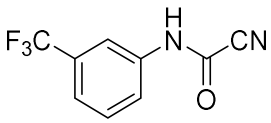

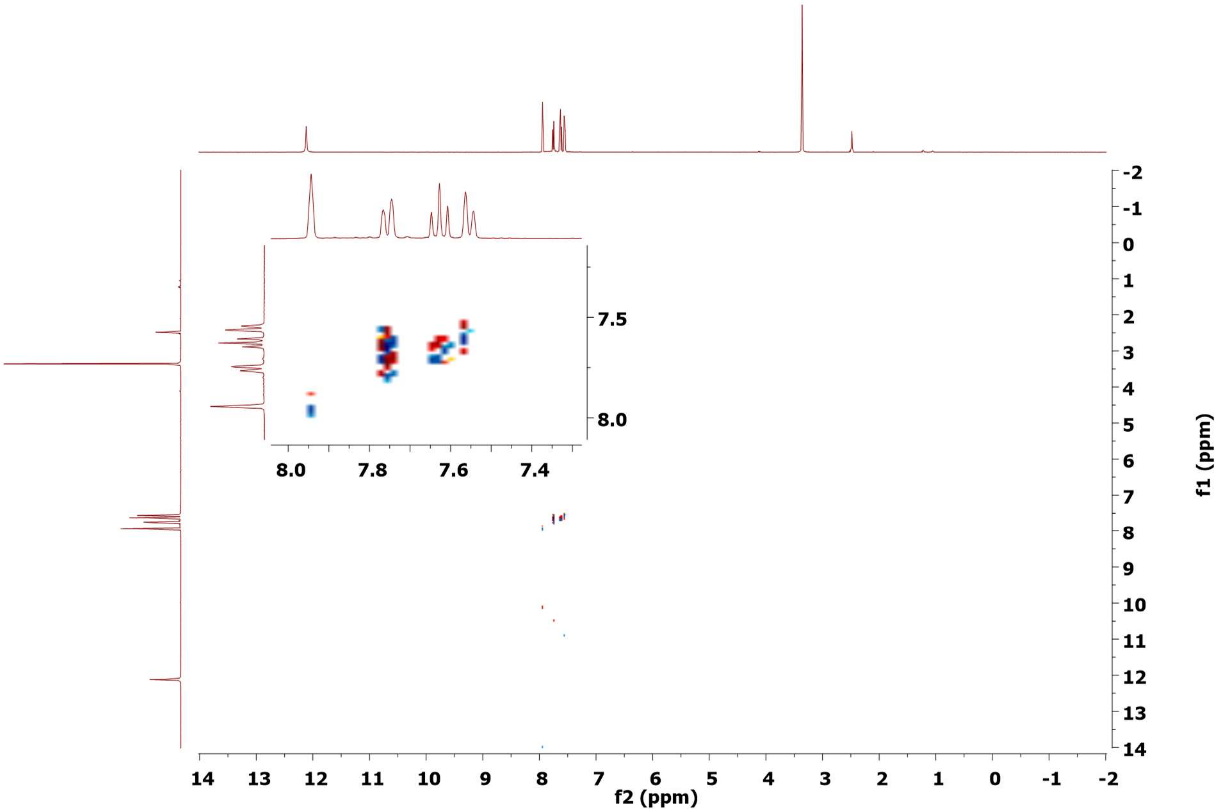

^{1}H-^{13}C-gHSQC NMR (DMSO-d6) spectrum of (3-(trifluoromethyl)phenyl)carbamoyl cyanide (2v)

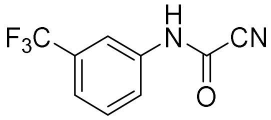

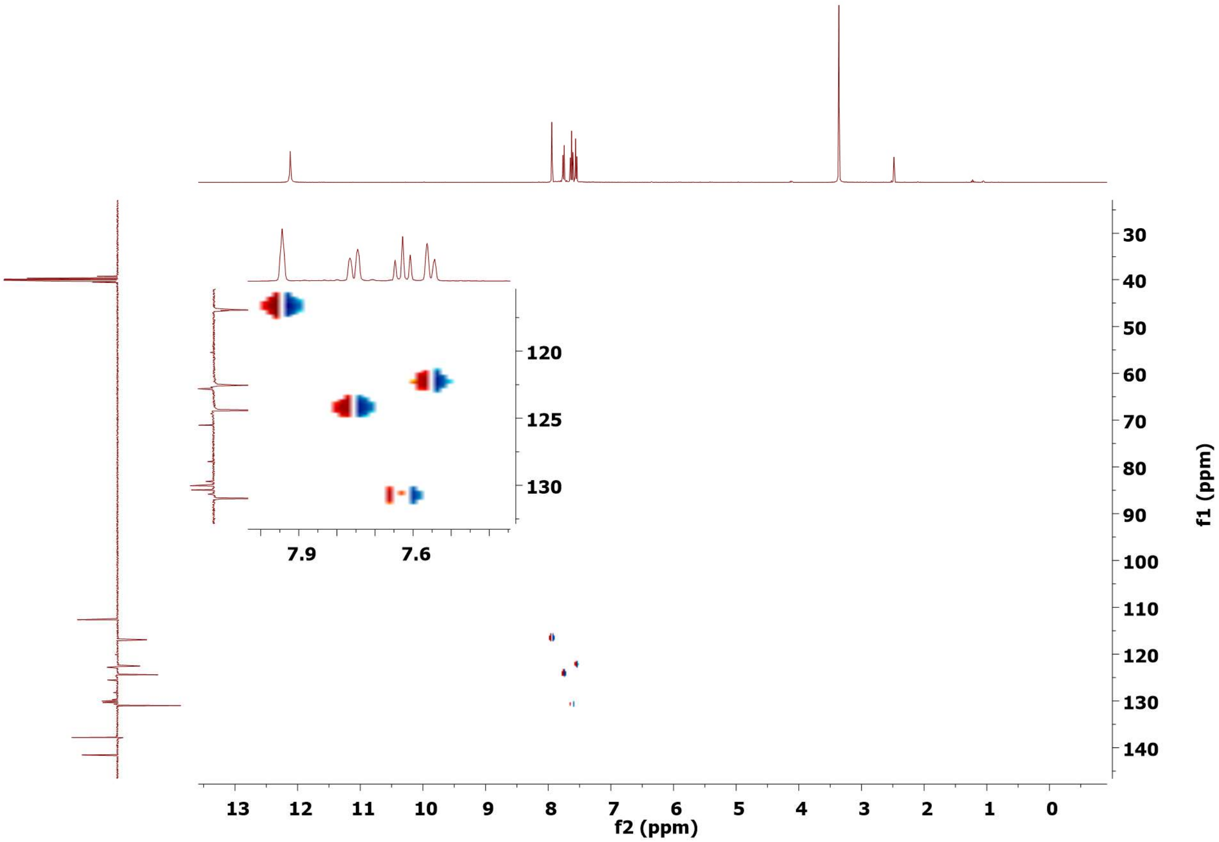

^{1}H-^{13}C-gHMBC NMR (DMSO-d6) spectrum of (3-(trifluoromethyl)phenyl)carbamoyl cyanide (2v)

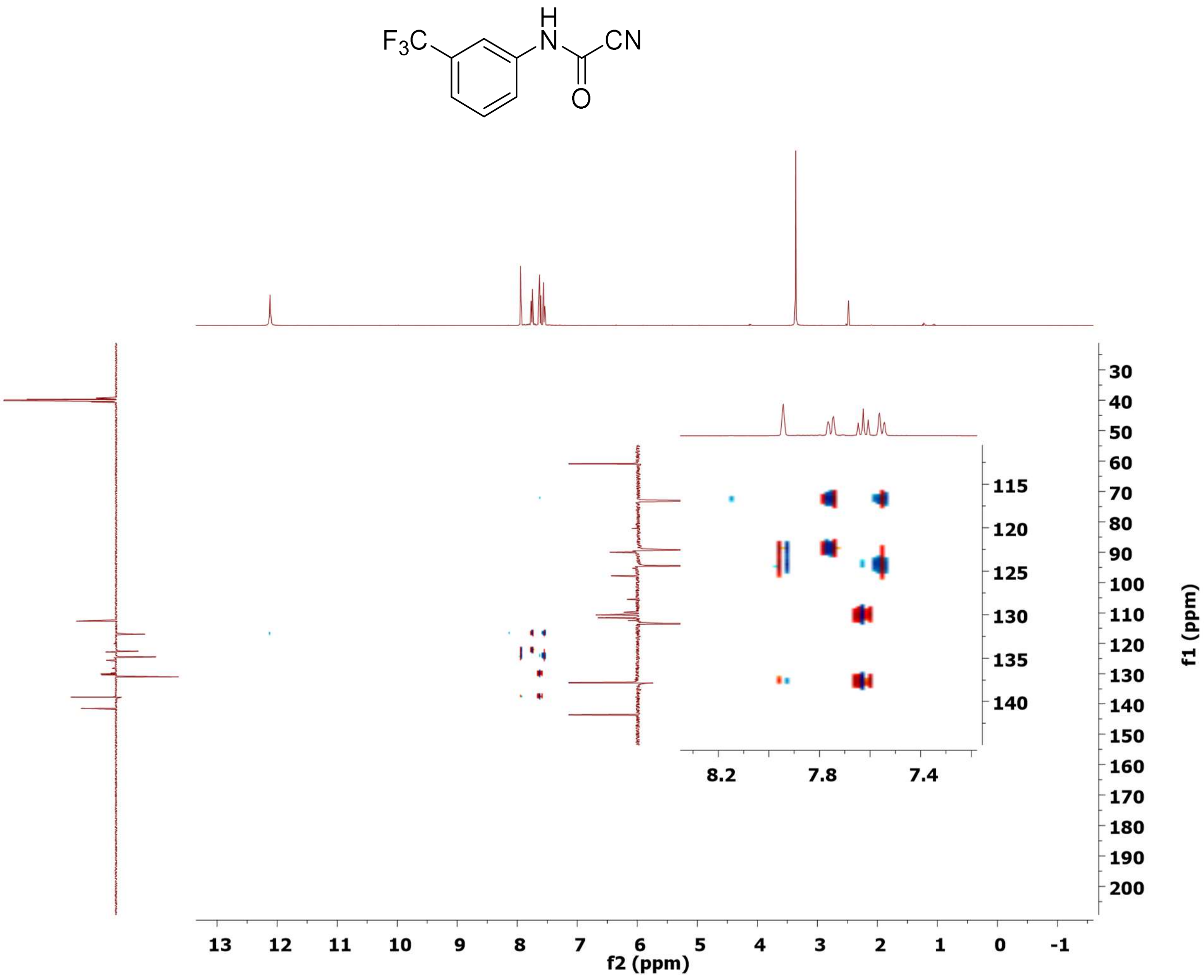

^{1}H NMR (DMSO-d6) spectrum of (4-methyl-1,3-phenylene)dicarbamoyl cyanide (2w)

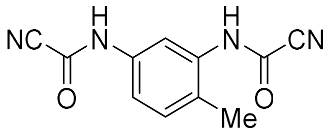

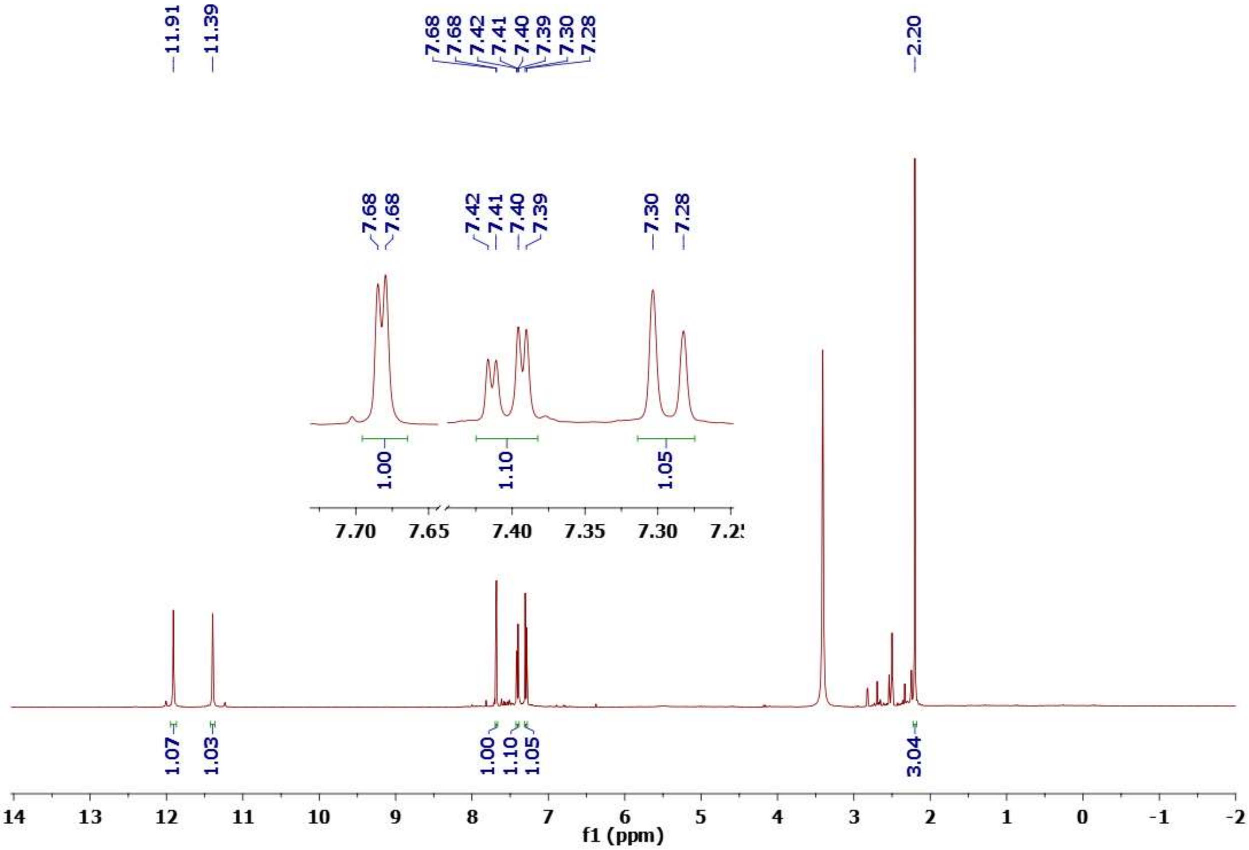

^{13}C NMR (DMSO-d6) spectrum of (4-methyl-1,3-phenylene)dicarbamoyl cyanide (2w)

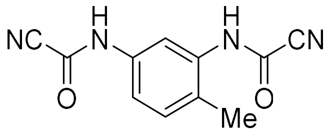

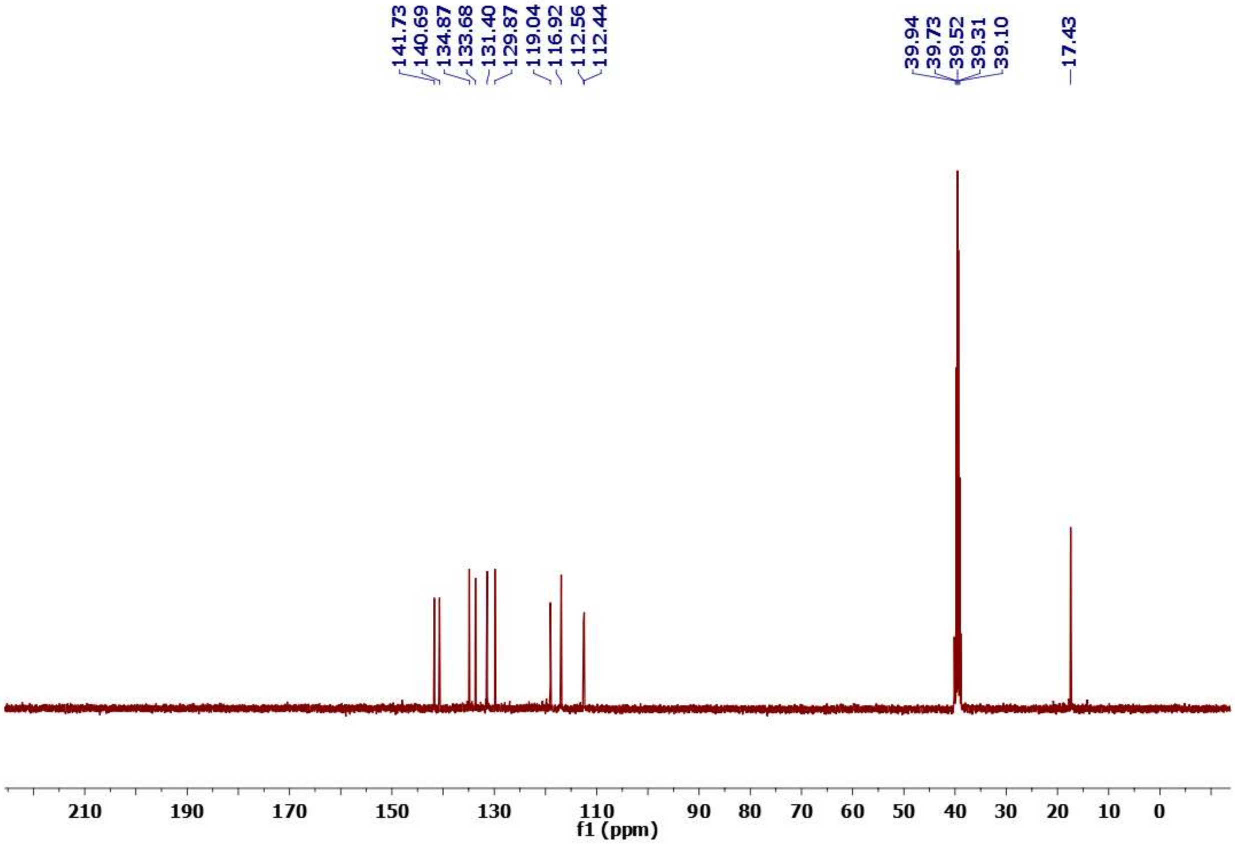

^{13}C CRAPT NMR (DMSO-d6) spectrum of (4-methyl-1,3-phenylene)dicarbamoyl cyanide (2w)

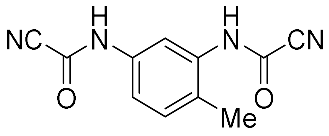

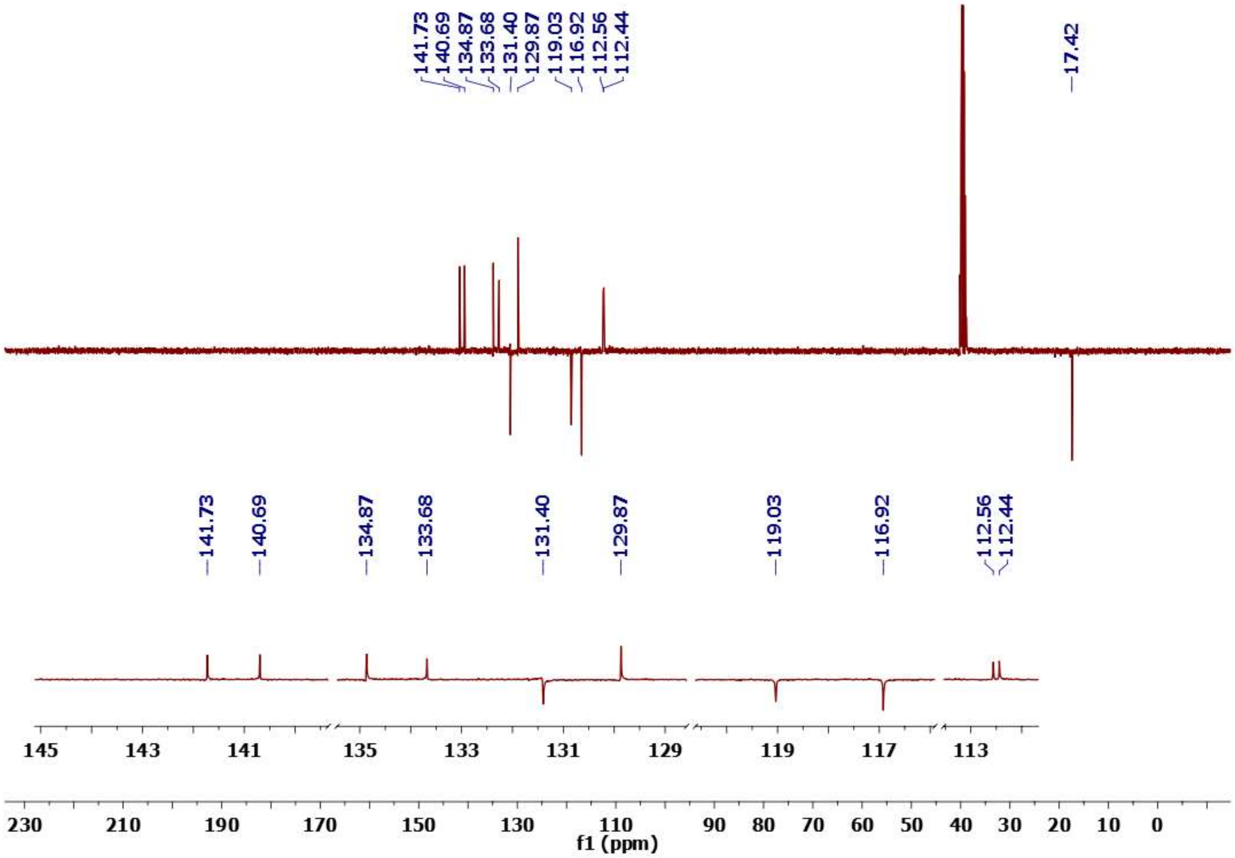

^{1}H-^{1}H gDQCOSY NMR (DMSO-d6) spectrum of (4-methyl-1,3-phenylene)dicarbamoyl cyanide (2w)

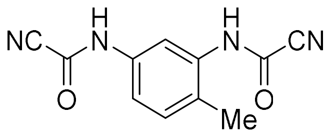

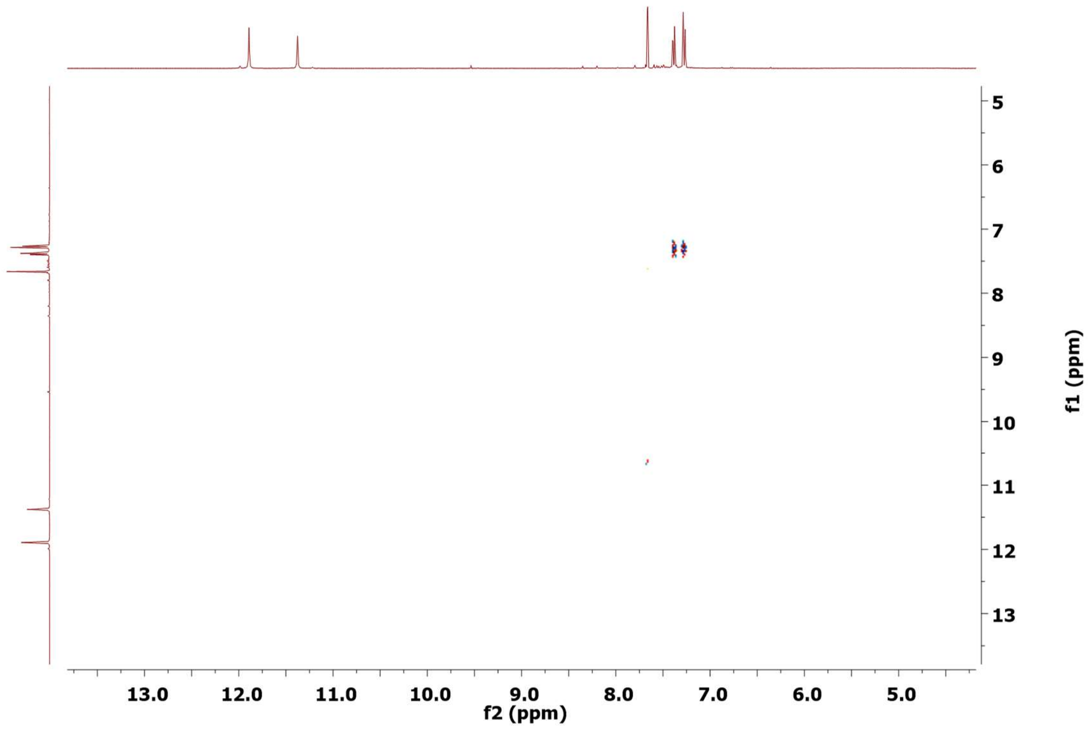

^{1}H-^{13}C-gHSQC NMR (DMSO-d6) spectrum of (4-methyl-1,3-phenylene)dicarbamoyl cyanide (2w)

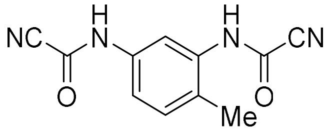

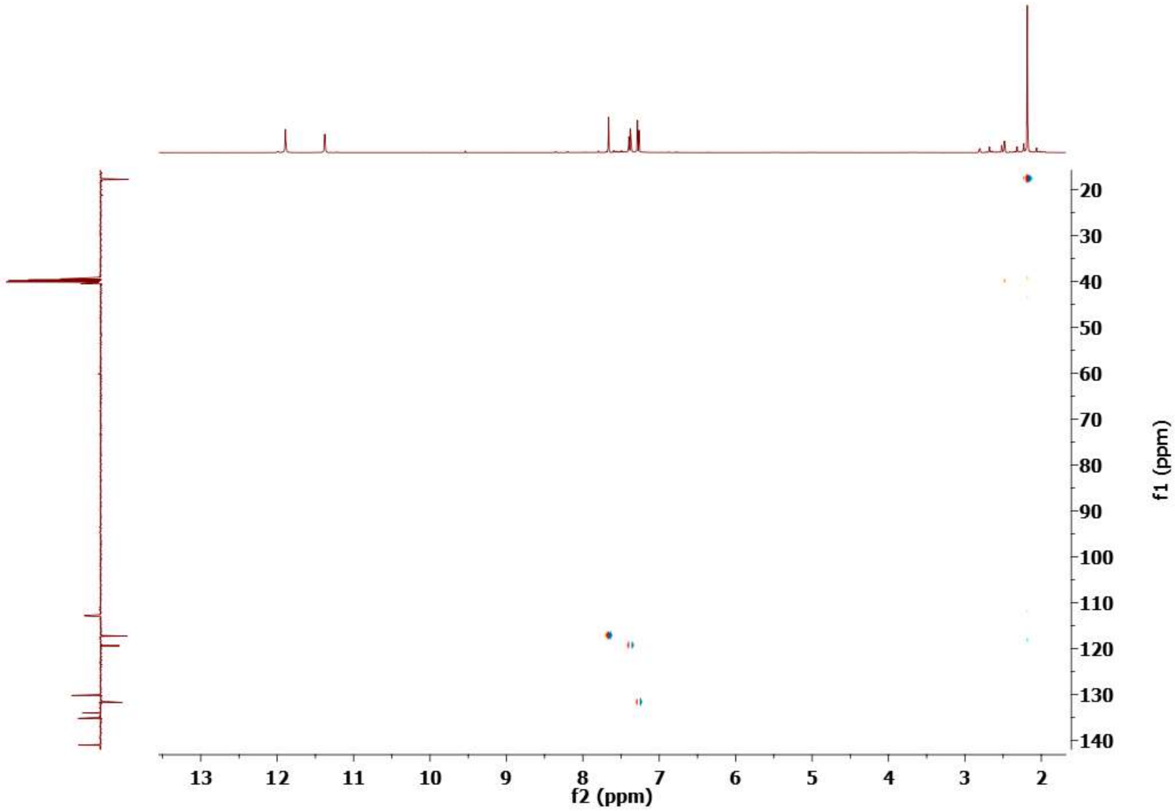

^{1}H-^{13}C-gHMBC NMR (DMSO-d6) spectrum of (4-methyl-1,3-phenylene)dicarbamoyl cyanide (2w)

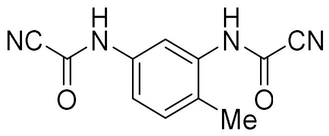

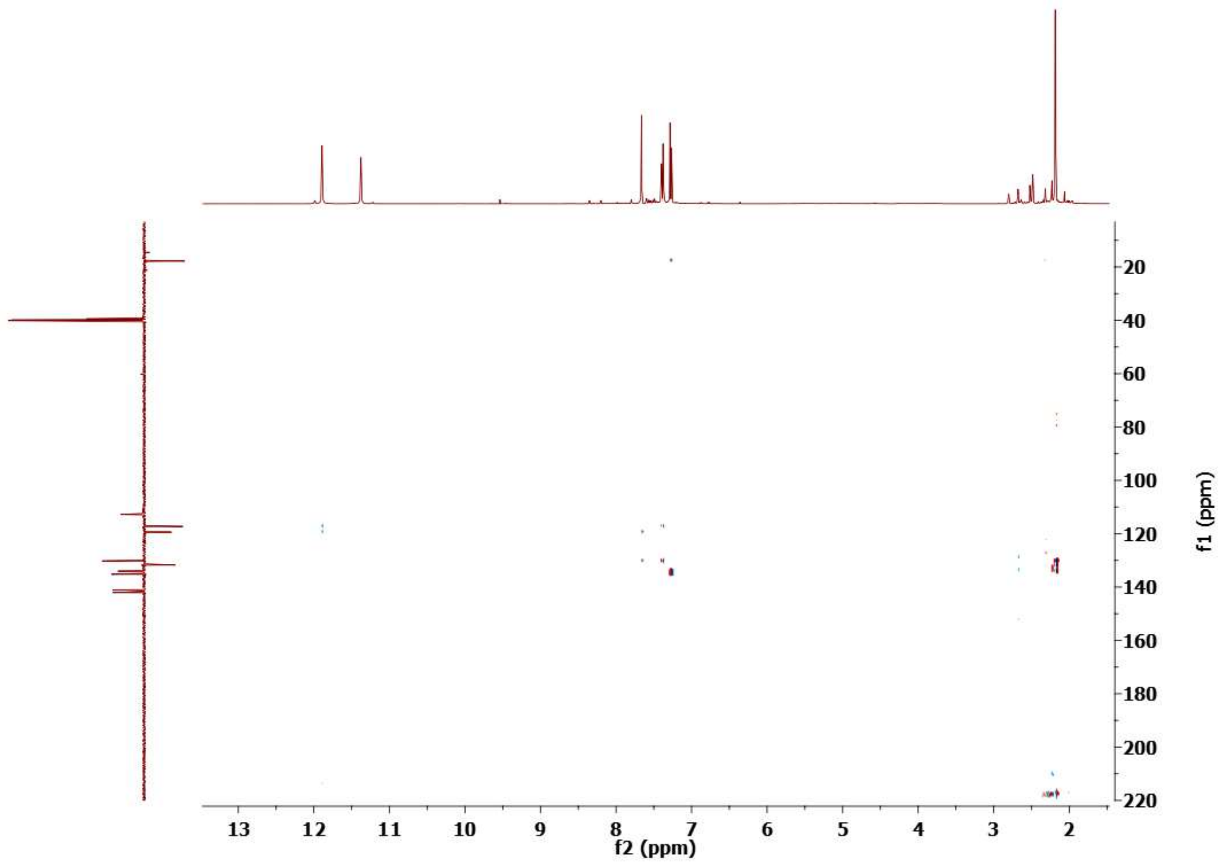

^{1}H NMR (DMSO-d6) spectrum of 1,4-phenylenedicarbamoyl cyanide (2x)

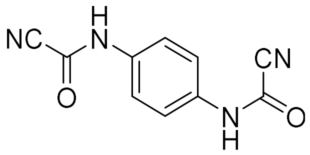

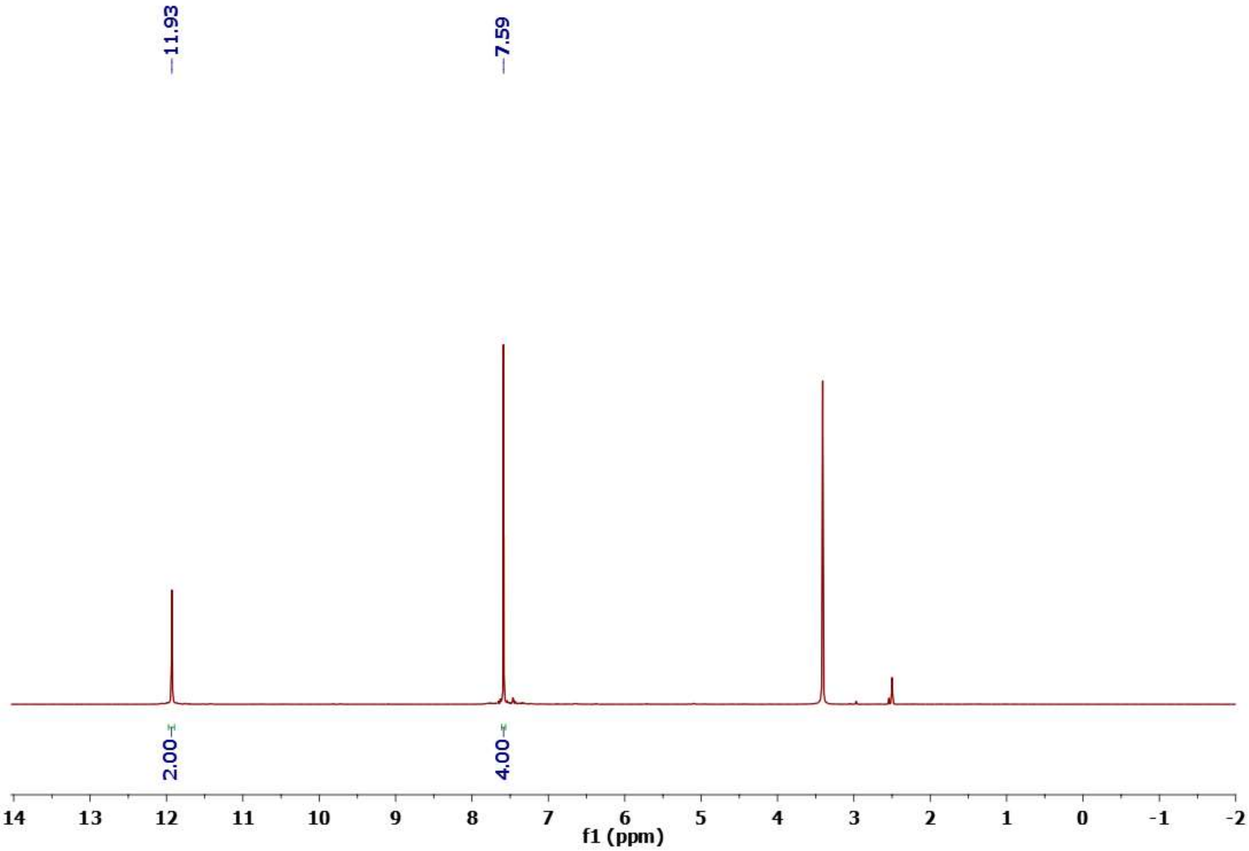

^{13}C NMR (DMSO-d6) spectrum of 1,4-phenylenedicarbamoyl cyanide (2x)

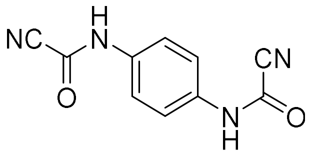

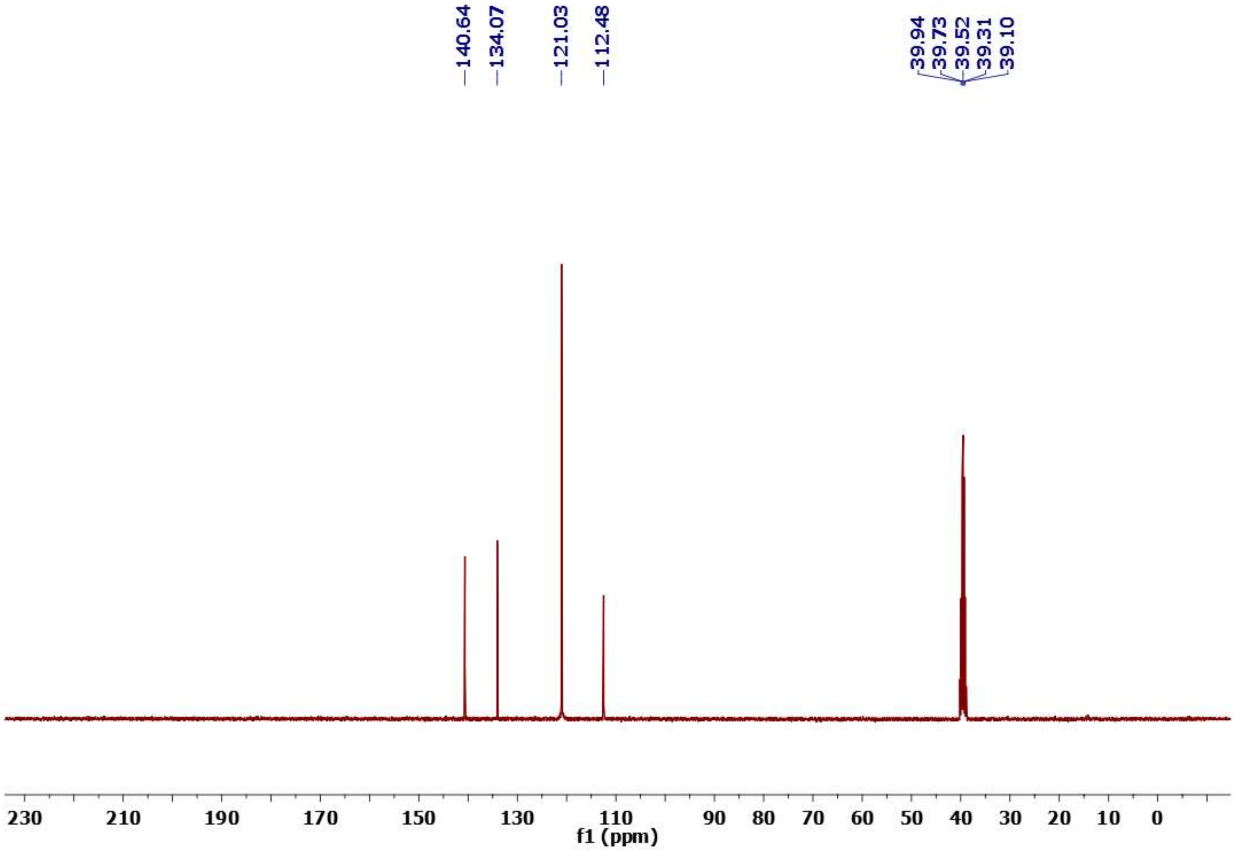

^{13}C CRAPT NMR (DMSO-d6) spectrum of 1,4-phenylenedicarbamoyl cyanide (2x)

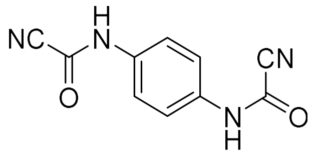

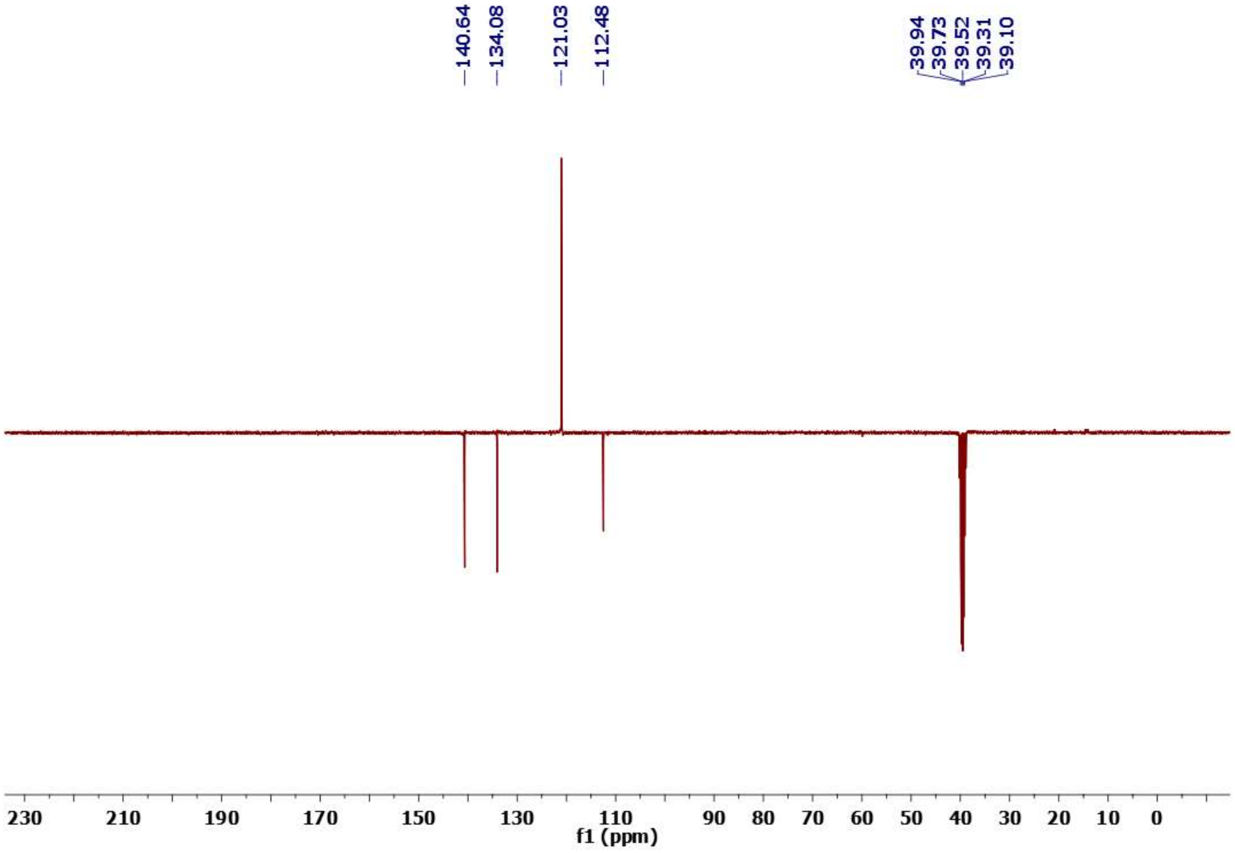

^{1}H-^{13}C-gHSQC NMR (DMSO-d6) spectrum of 1,4-phenylenedicarbamoyl cyanide (2x)

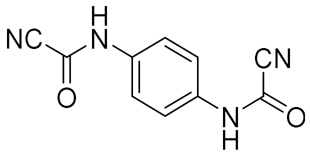

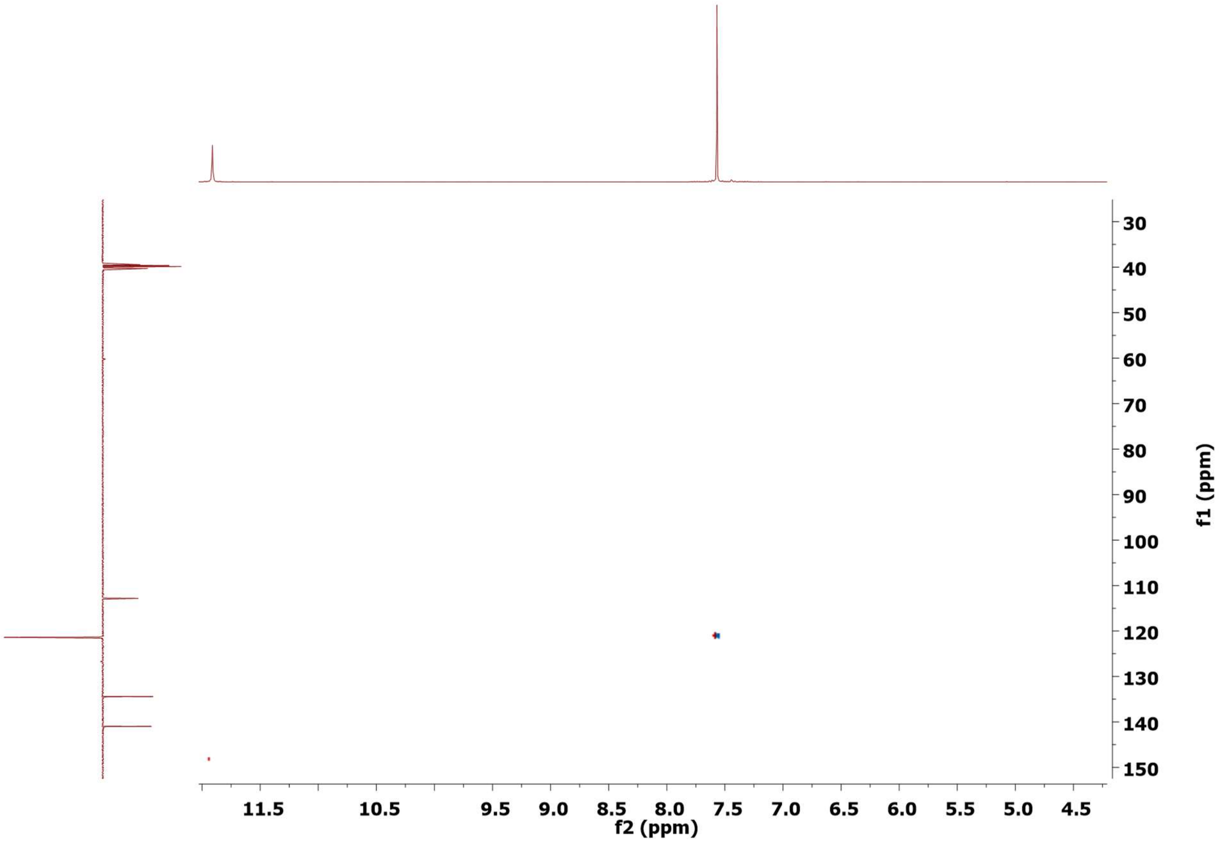

^1H-^{13}C-gHMBC NMR (DMSO-d6) spectrum of 1,4-phenylenedicarbamoyl cyanide (2x)

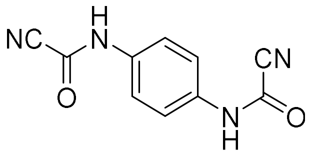

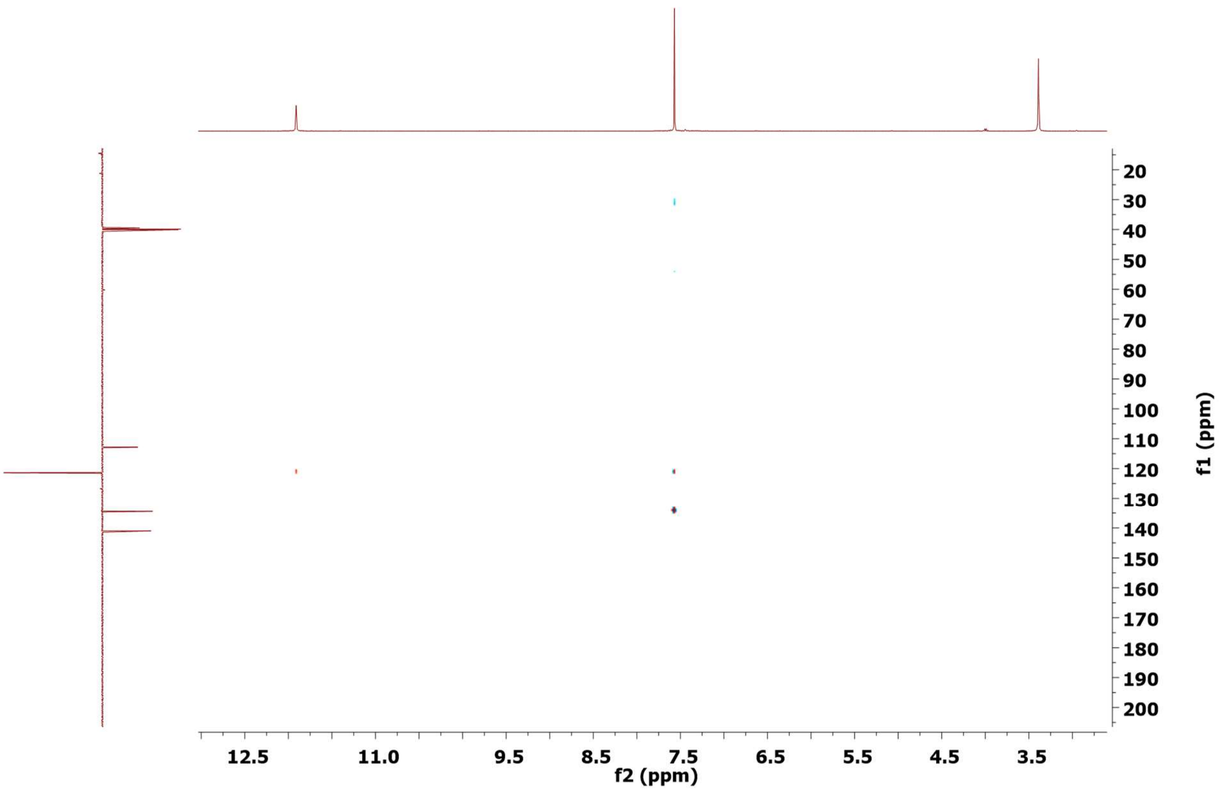

^{1}H NMR (DMSO-d6) spectrum of (2-bromophenyl)carbamoyl cyanide (2y)

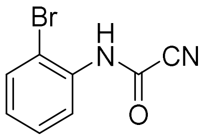

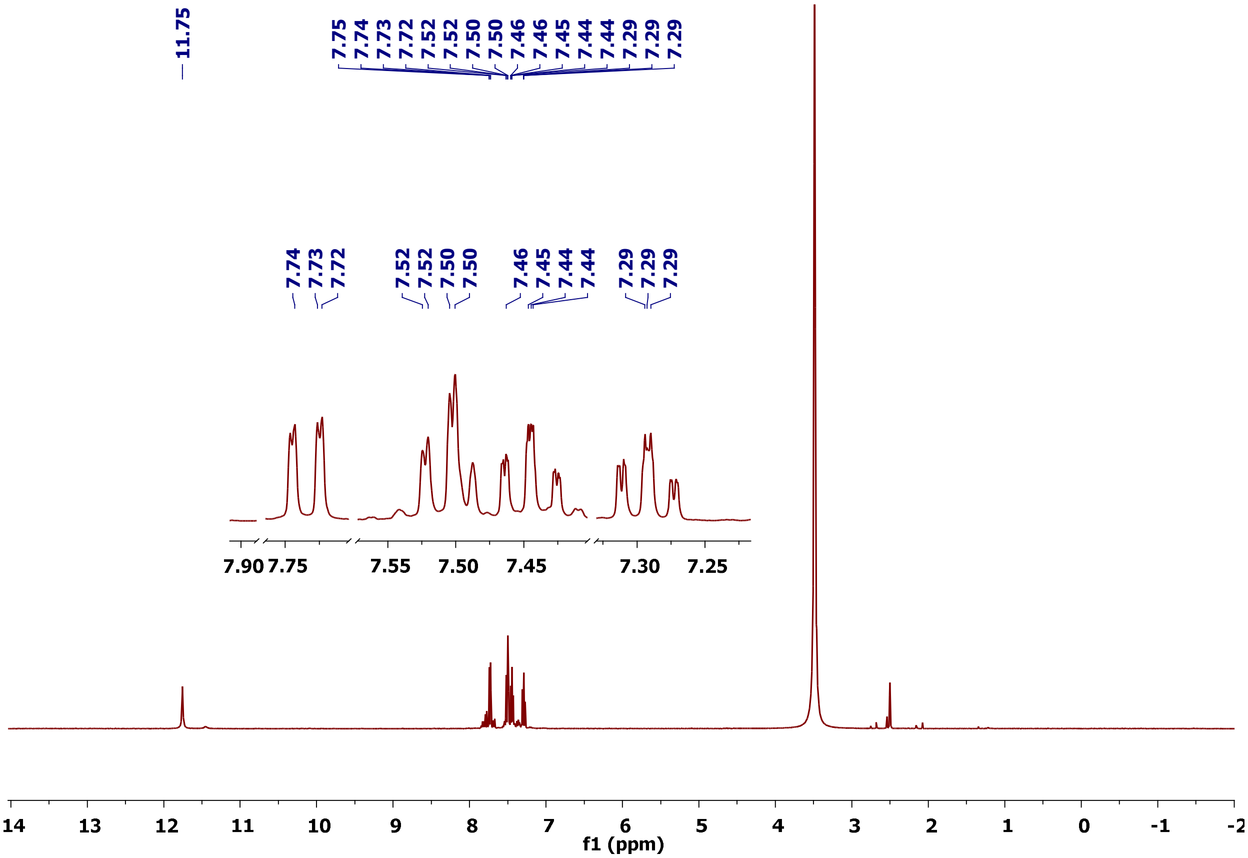

^{13}C NMR (DMSO-d6) spectrum of (2-bromophenyl)carbamoyl cyanide (2y)

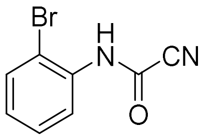

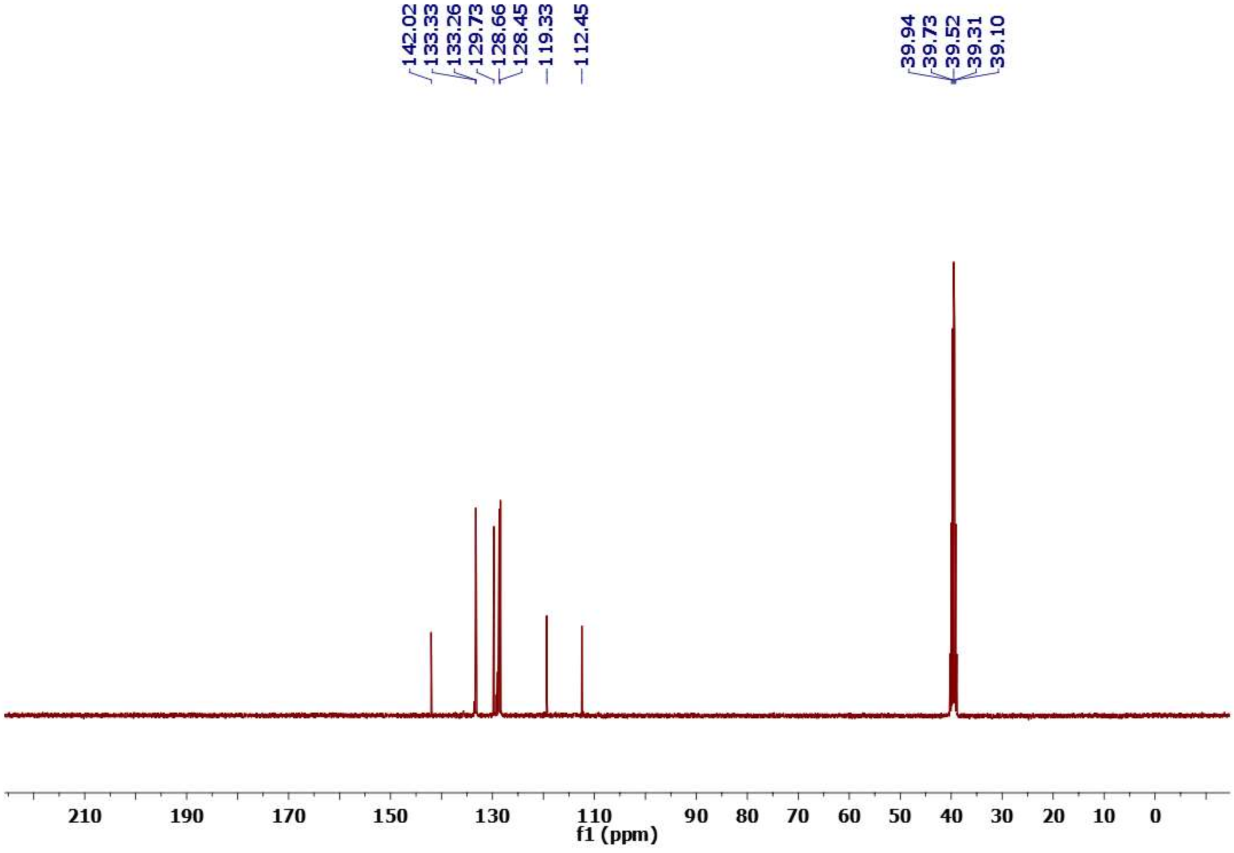

^{13}C CRAPT NMR (DMSO-d6) spectrum of (2-bromophenyl)carbamoyl cyanide (2y)

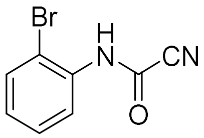

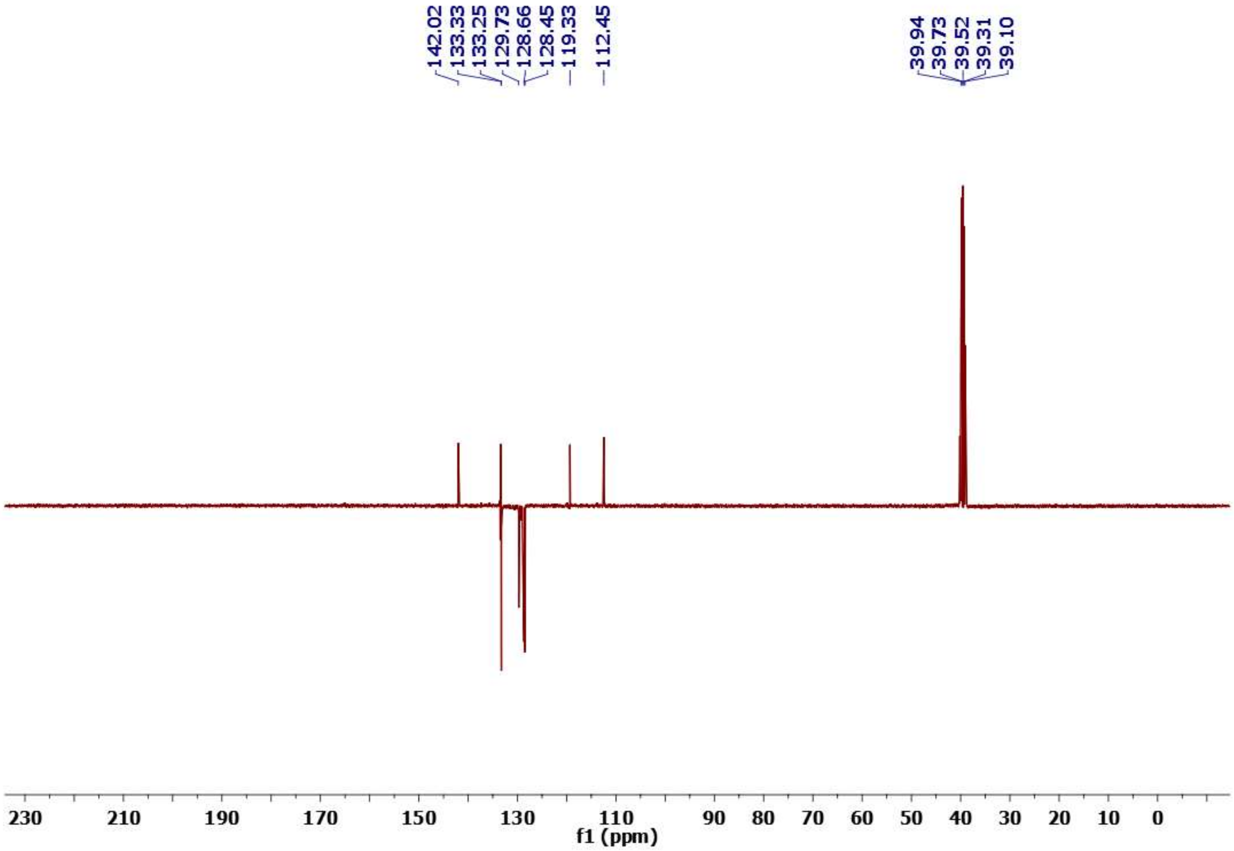

^{1}H-^{1}H gDQCOSY NMR (DMSO-d6) spectrum of (2-bromophenyl)carbamoyl cyanide (2y)

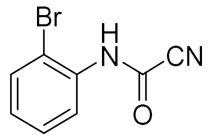

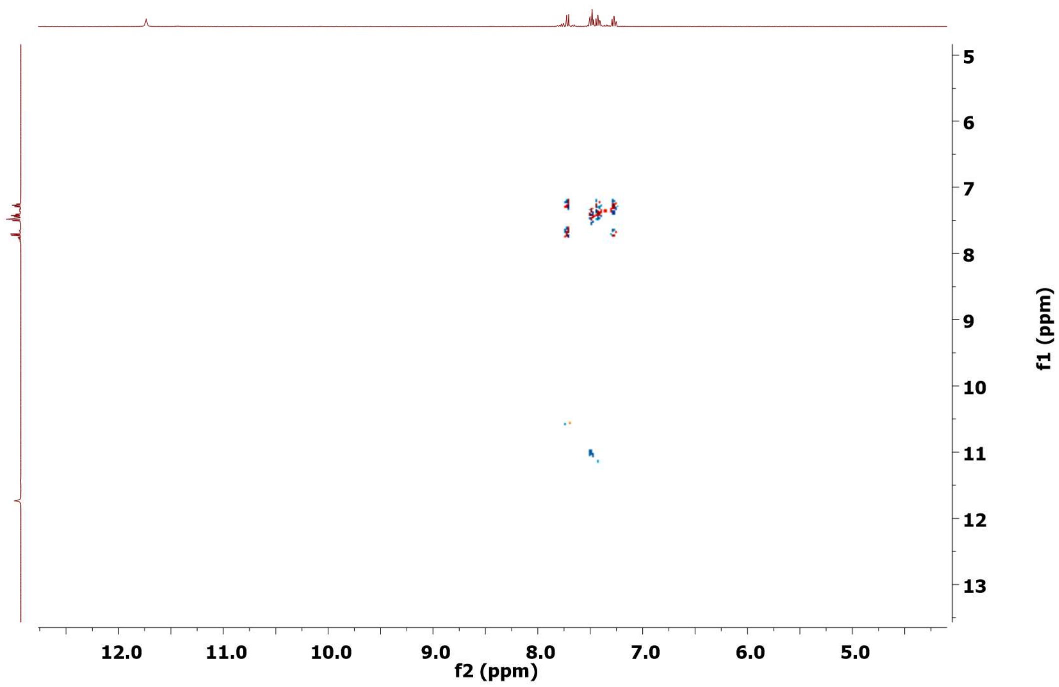

^{1}H-^{13}C-gHSQC NMR (DMSO-d6) spectrum of (2-bromophenyl)carbamoyl cyanide (2y)

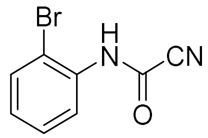

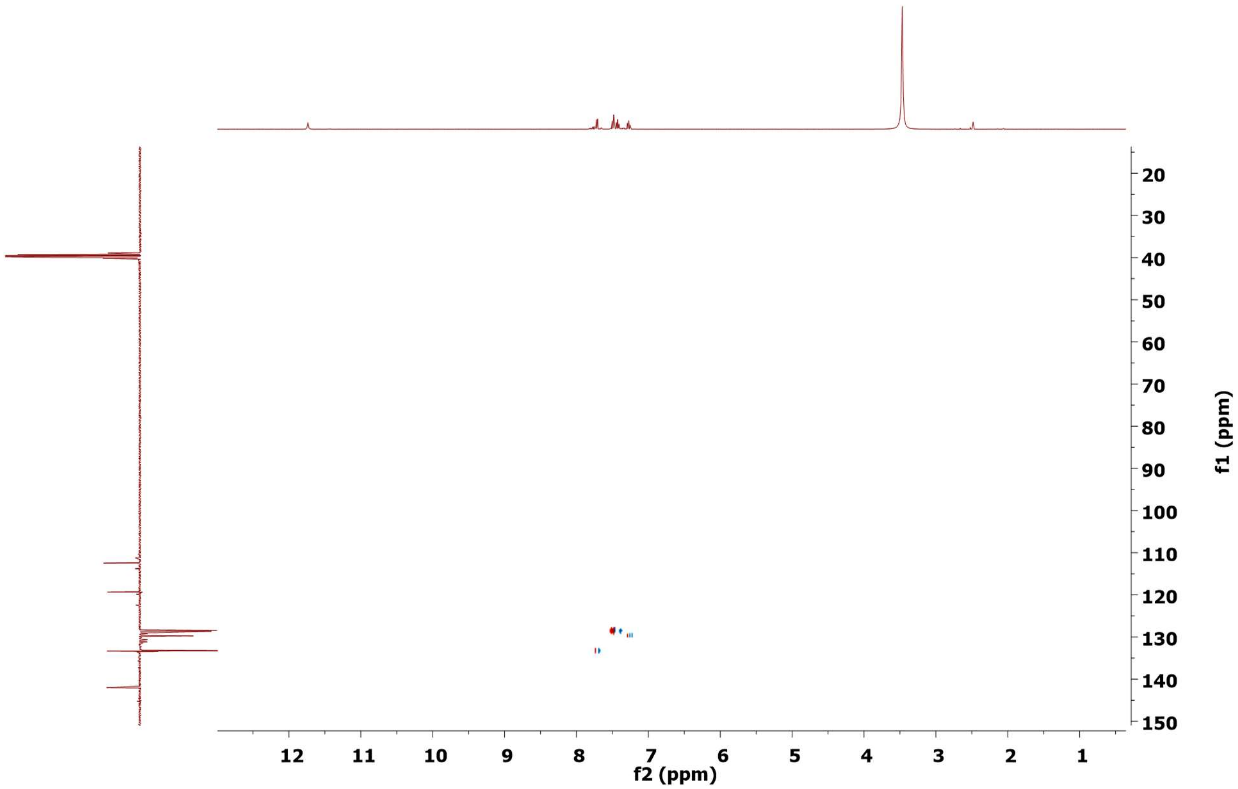

^{1}H-^{13}C-gHMBC NMR (DMSO-d6) spectrum of (2-bromophenyl)carbamoyl cyanide (2y)

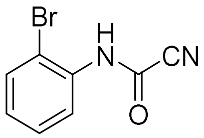

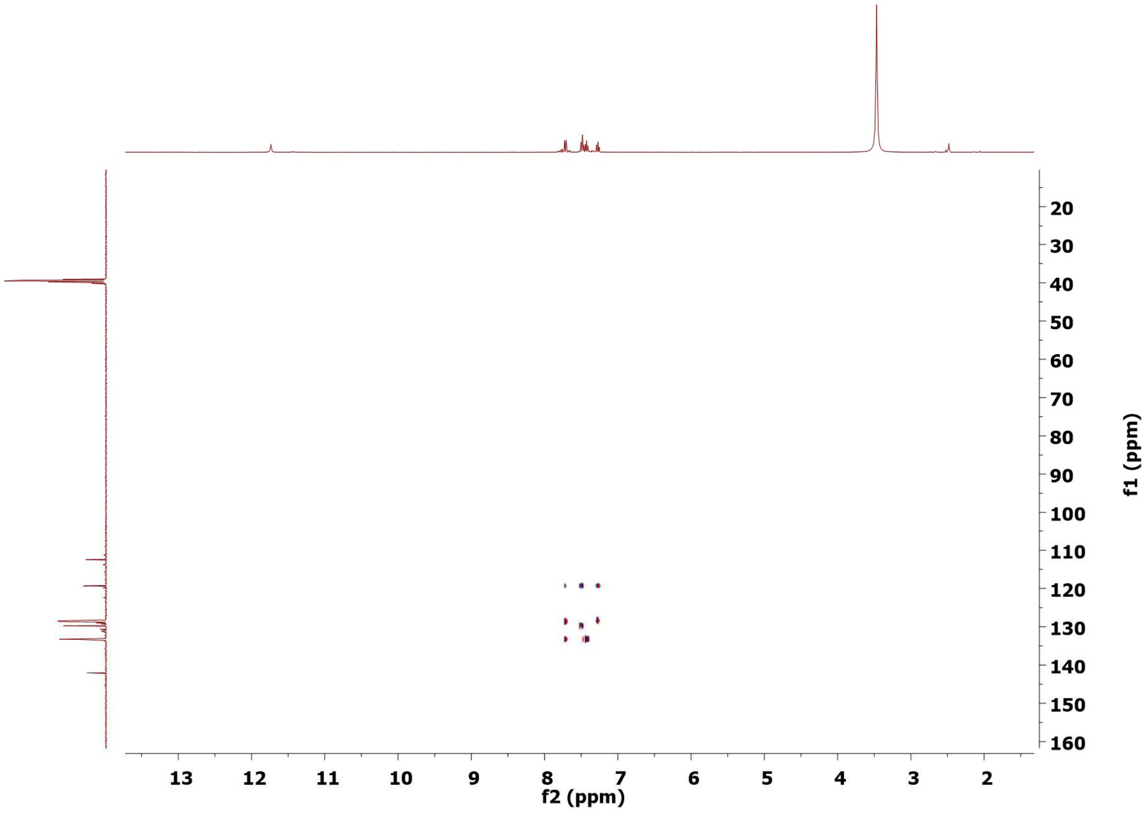

^{1}H NMR (DMSO-d6) spectrum of (2,4-dichlorophenyl)carbamoyl cyanide after 1h (2z)

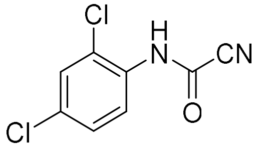

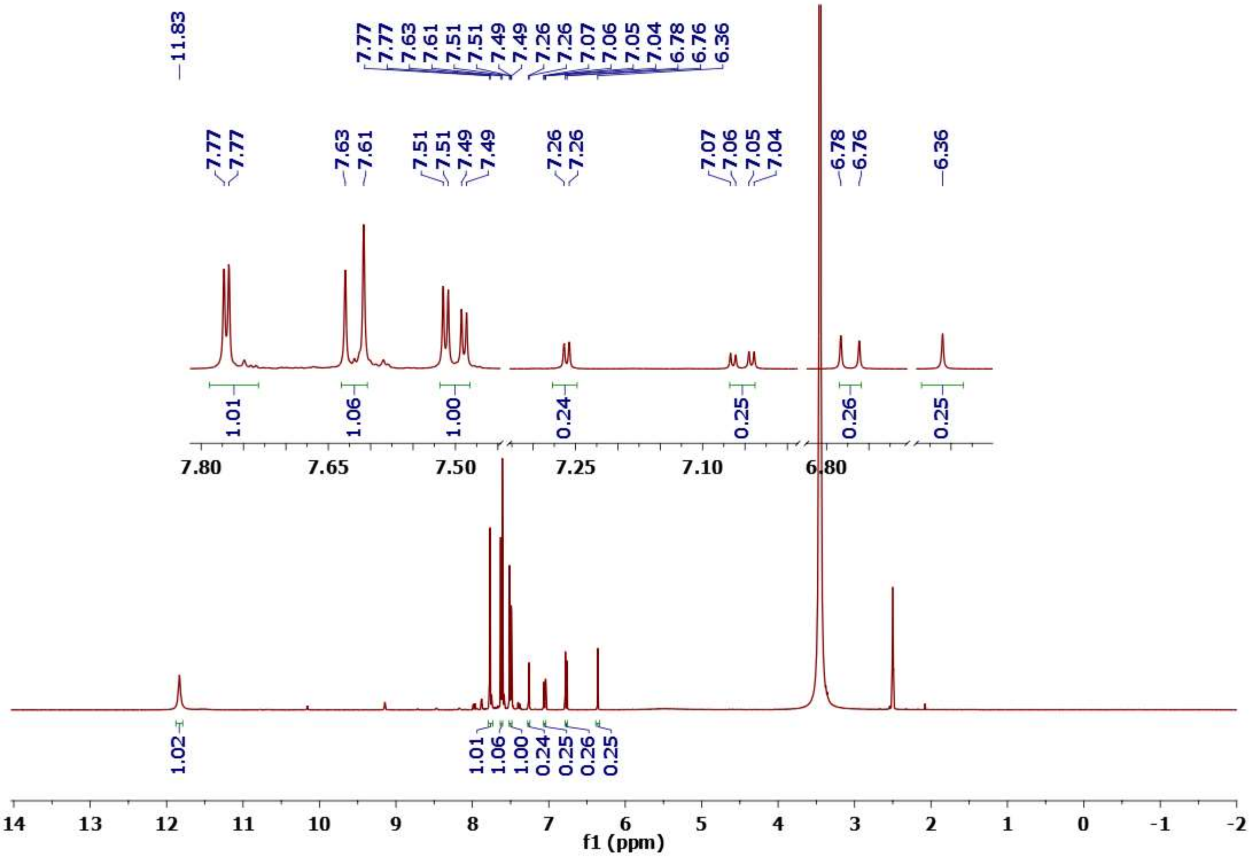

^{13}C NMR (DMSO-d6) spectrum of (2,4-dichlorophenyl)carbamoyl cyanide after 8h (2z)

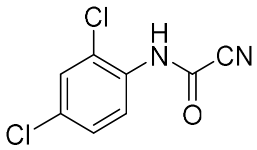

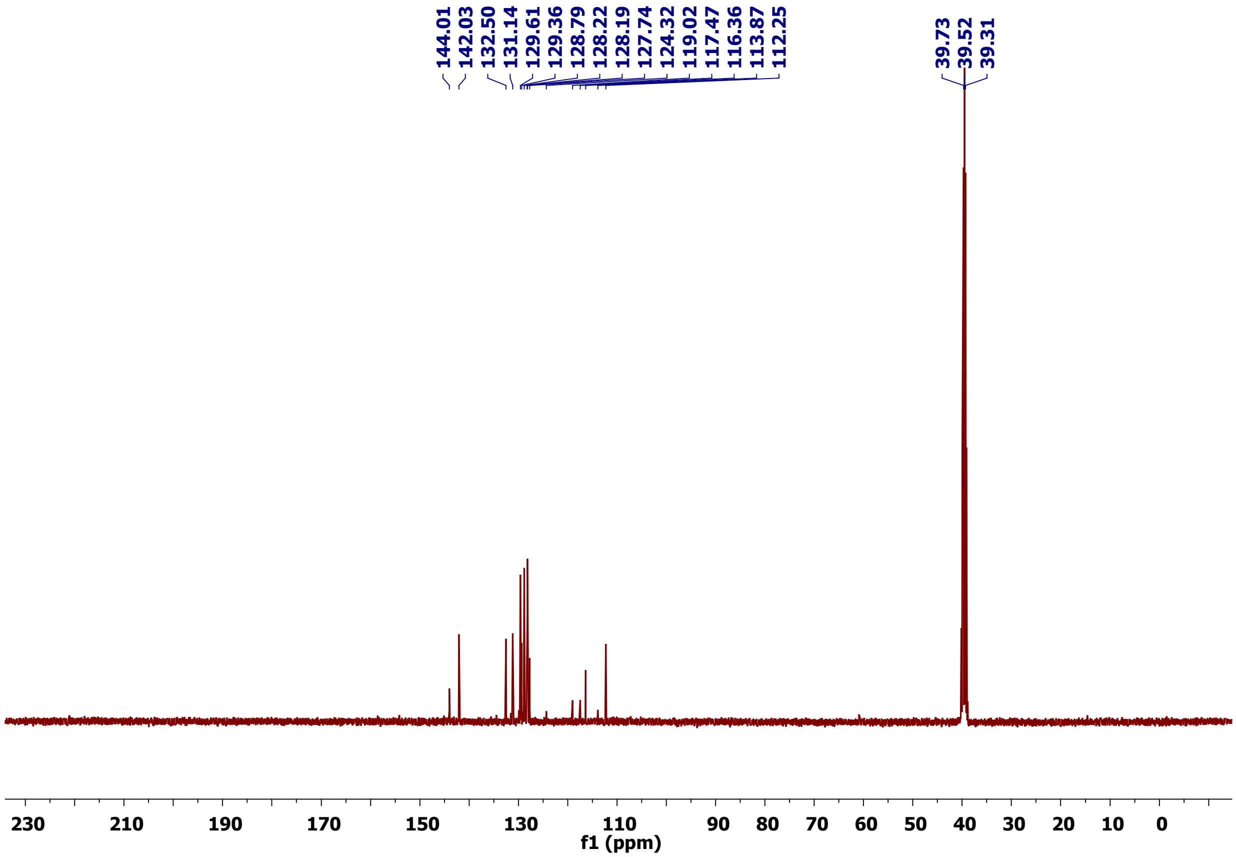

^{13}C CRAPT NMR (DMSO-d6) spectrum of (2,4-dichlorophenyl)carbamoyl cyanide after 8h (2z)

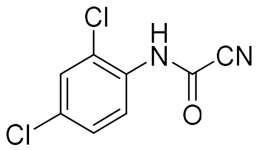

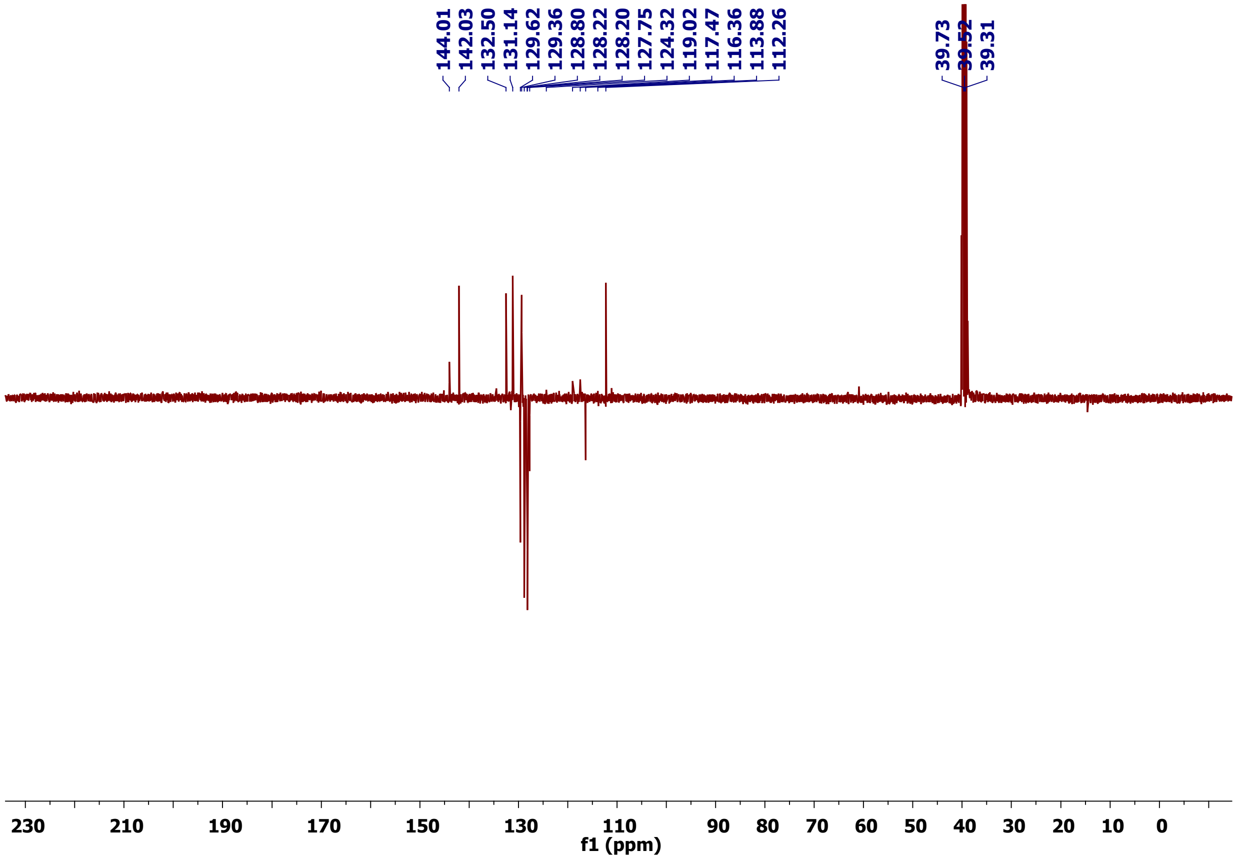

^{1}H NMR (DMSO-d6) spectrum of (2,4-dichlorophenyl)carbamoyl cyanide after 8h (2z)

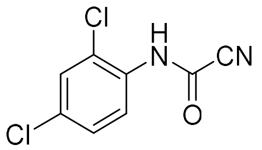

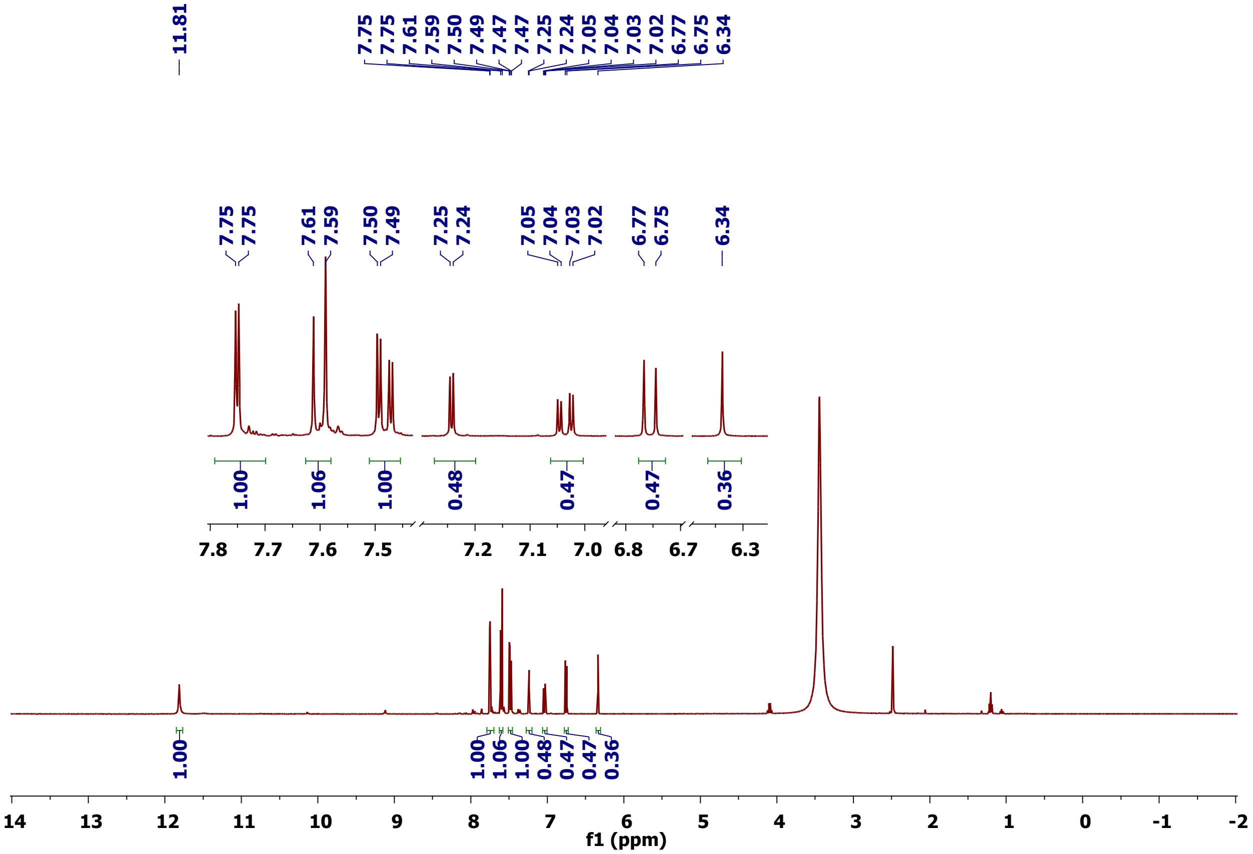

^{13}C NMR (DMSO-d6) spectrum of (2,4-dichlorophenyl)carbamoyl cyanide after 8h (2z)

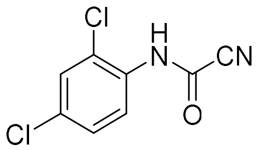

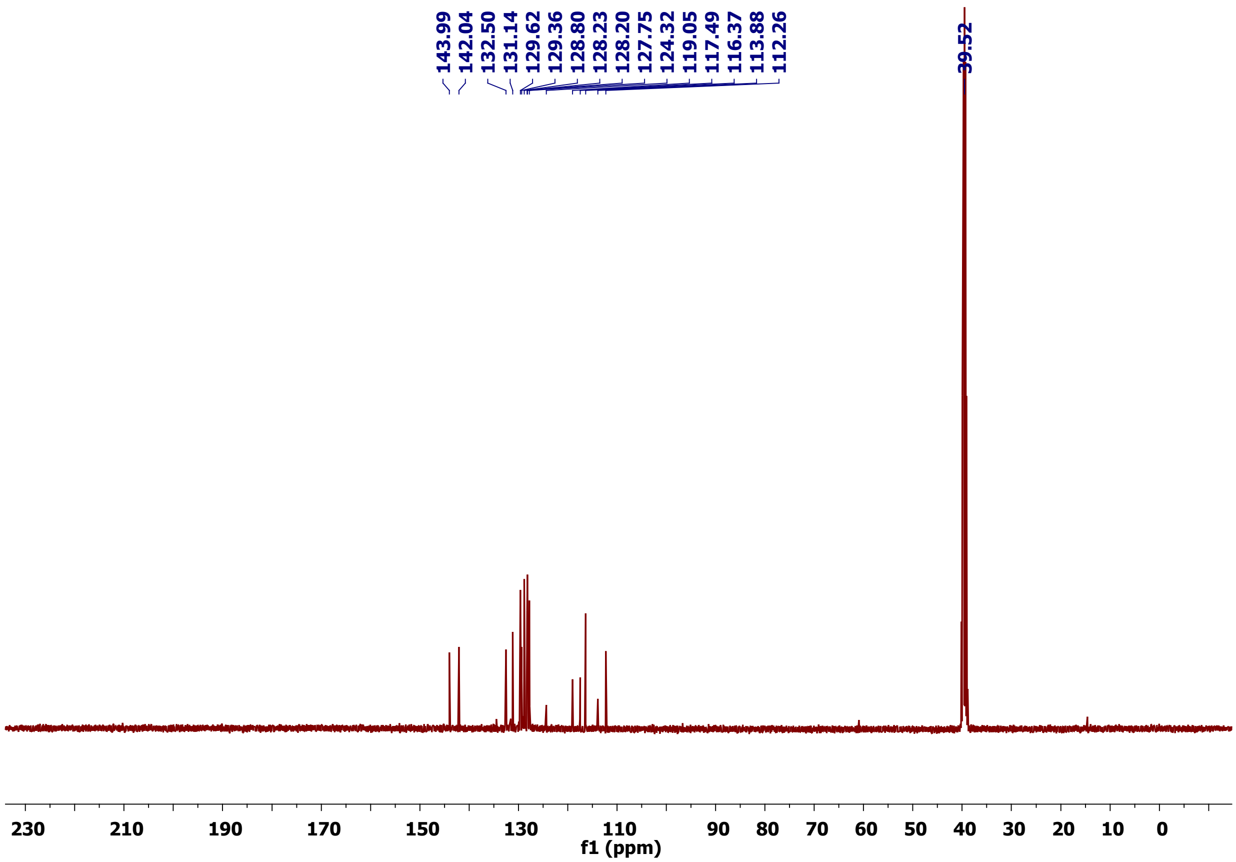

^{13}C CRAPT NMR (DMSO-d6) spectrum of (2,4-dichlorophenyl)carbamoyl cyanide after 8h (2z)

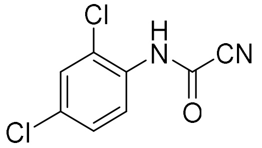

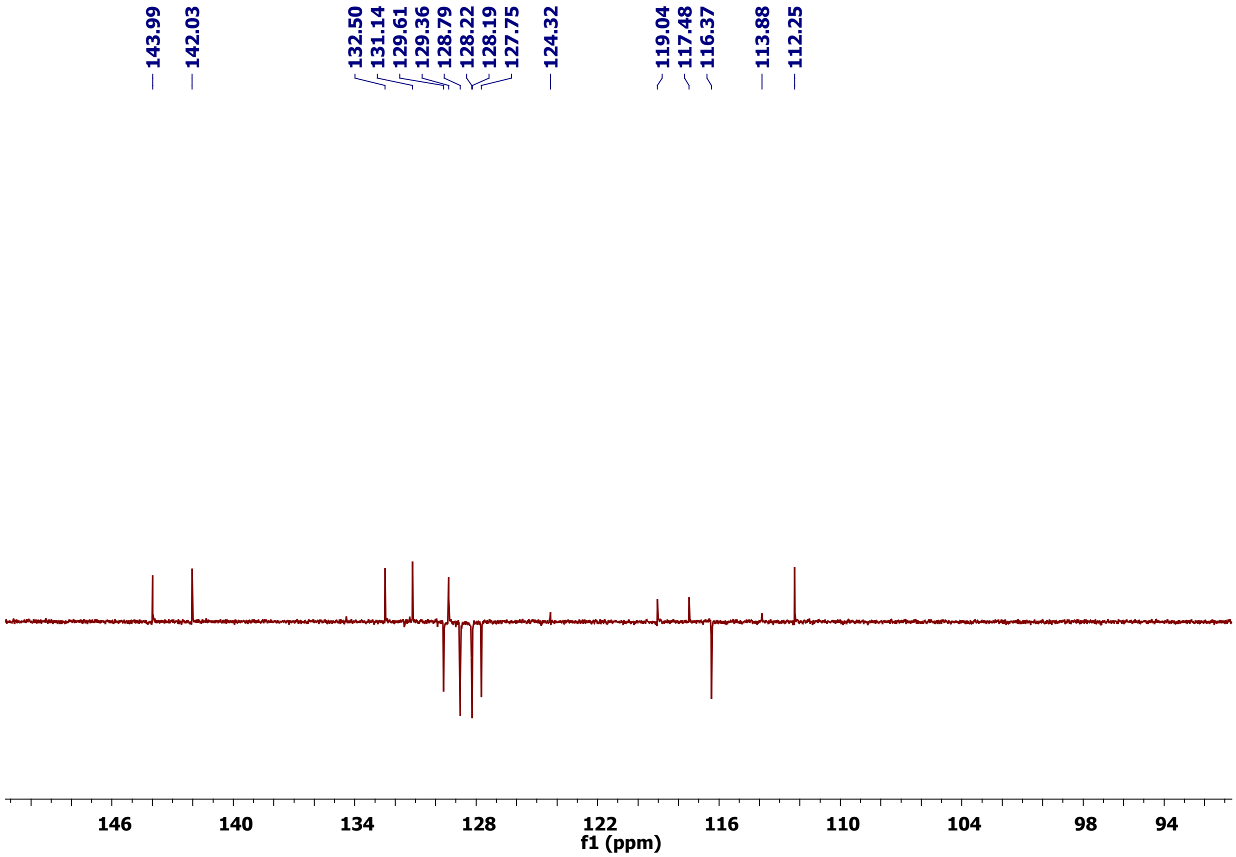

^{1}H-^{1}H gDQCOSY NMR (DMSO-d6) spectrum of (2,4-dichlorophenyl)carbamoyl cyanide after 8h (2z)

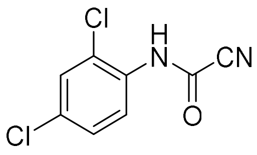

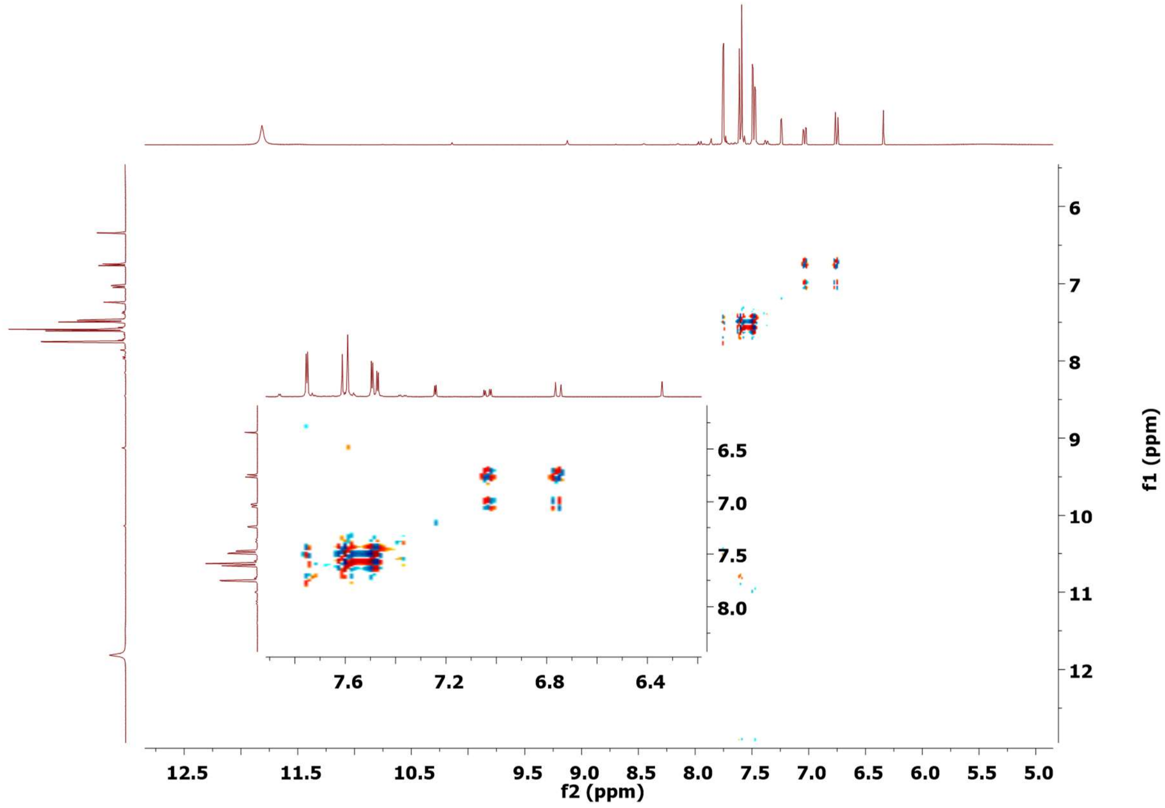

^{1}H-^{13}C-gHSQC NMR (DMSO-d6) spectrum of (2,4-dichlorophenyl)carbamoyl cyanide after 8h (2z)

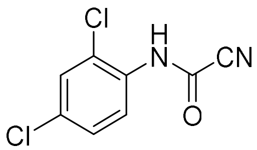

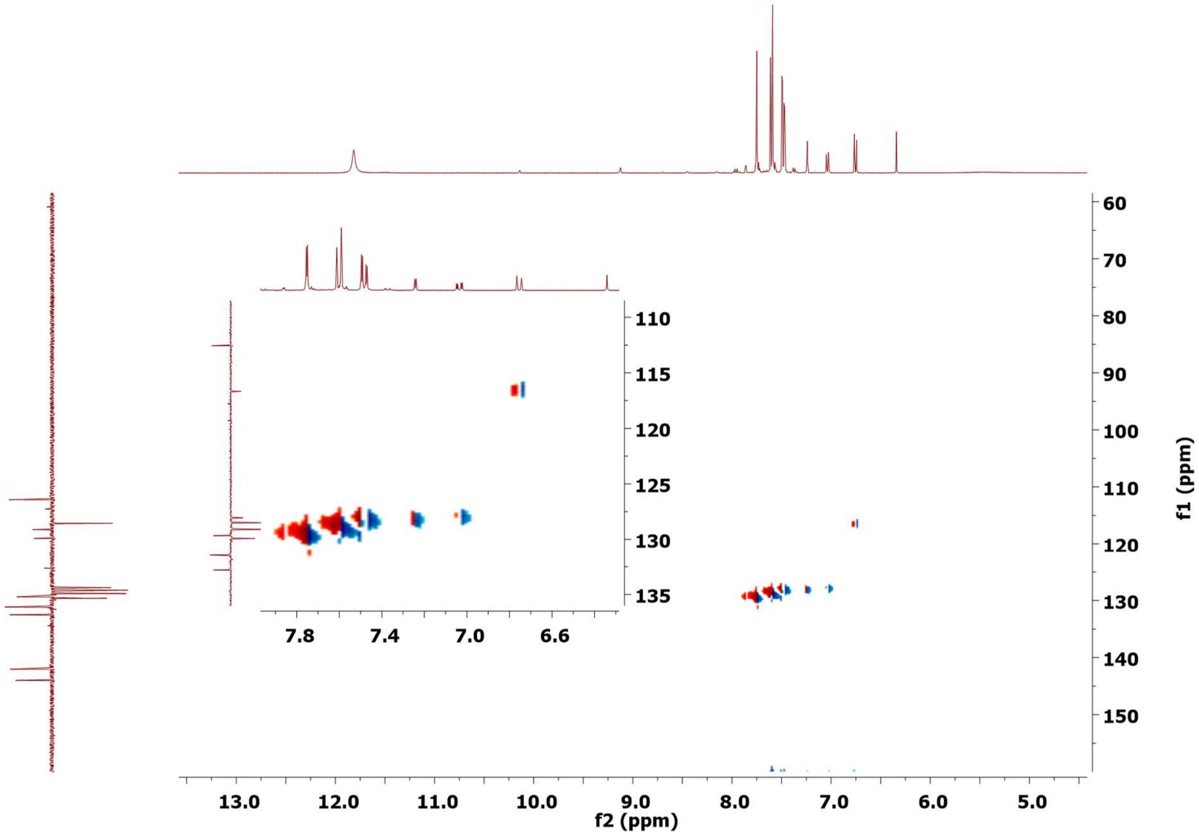

^1H-^{13}C-gHMBC NMR (DMSO-d6) spectrum of (2,4-dichlorophenyl)carbamoyl cyanide after 8h (2z)

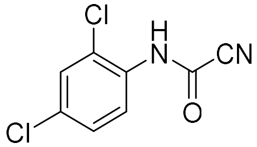

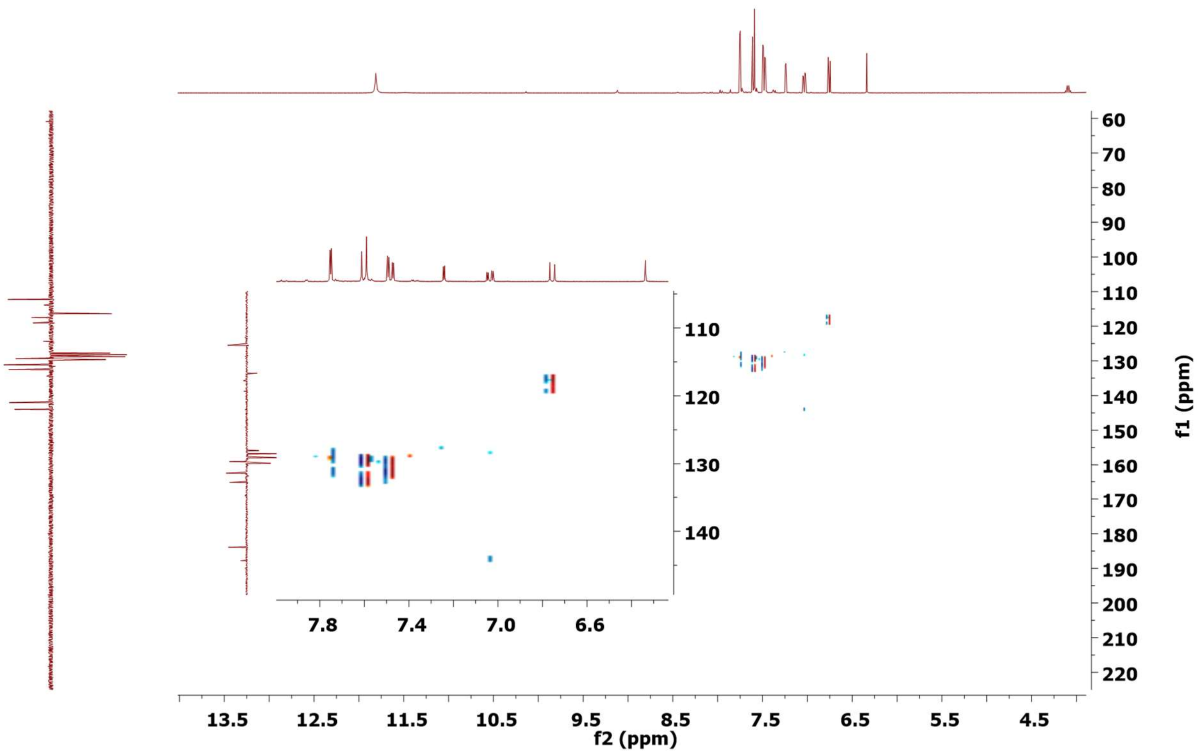

^{1}H NMR (DMSO-d6) spectrum of (5-chloro-2-methylphenyl)carbamoyl cyanide (2a')

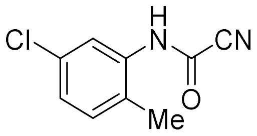

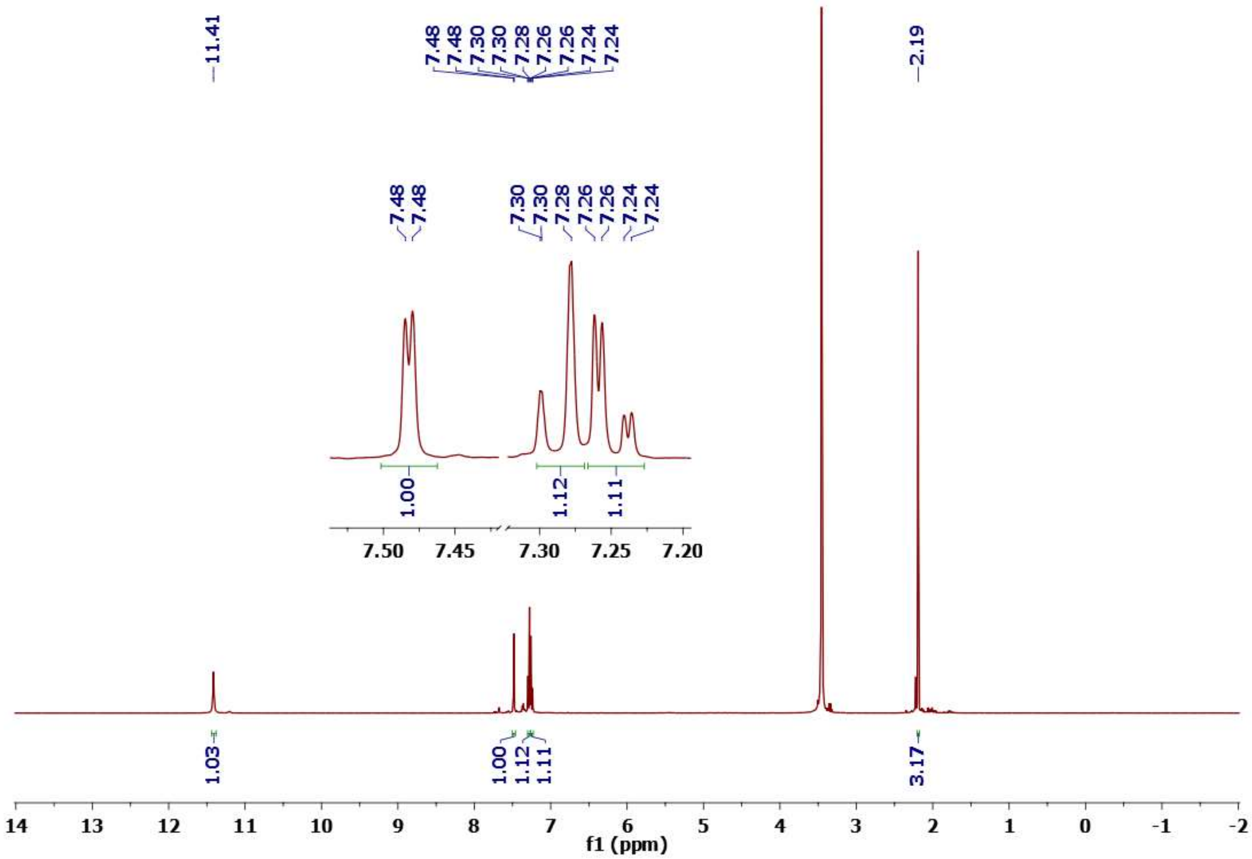

^{13}C NMR (DMSO-d6) spectrum of (5-chloro-2-methylphenyl)carbamoyl cyanide (2a')

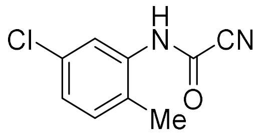

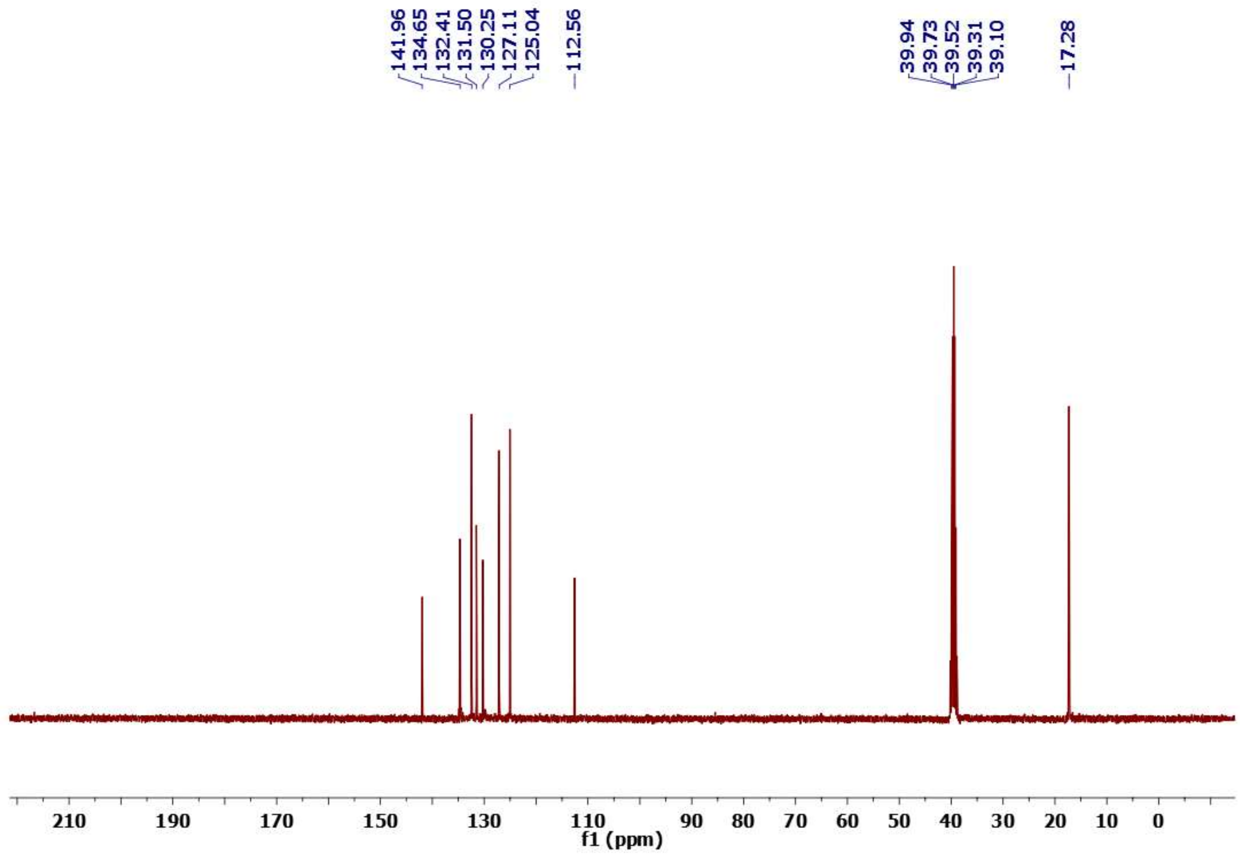

^{13}C CRAPT NMR (DMSO-d6) spectrum of (5-chloro-2-methylphenyl)carbamoyl cyanide (2a')

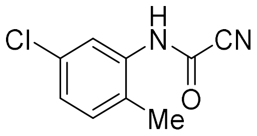

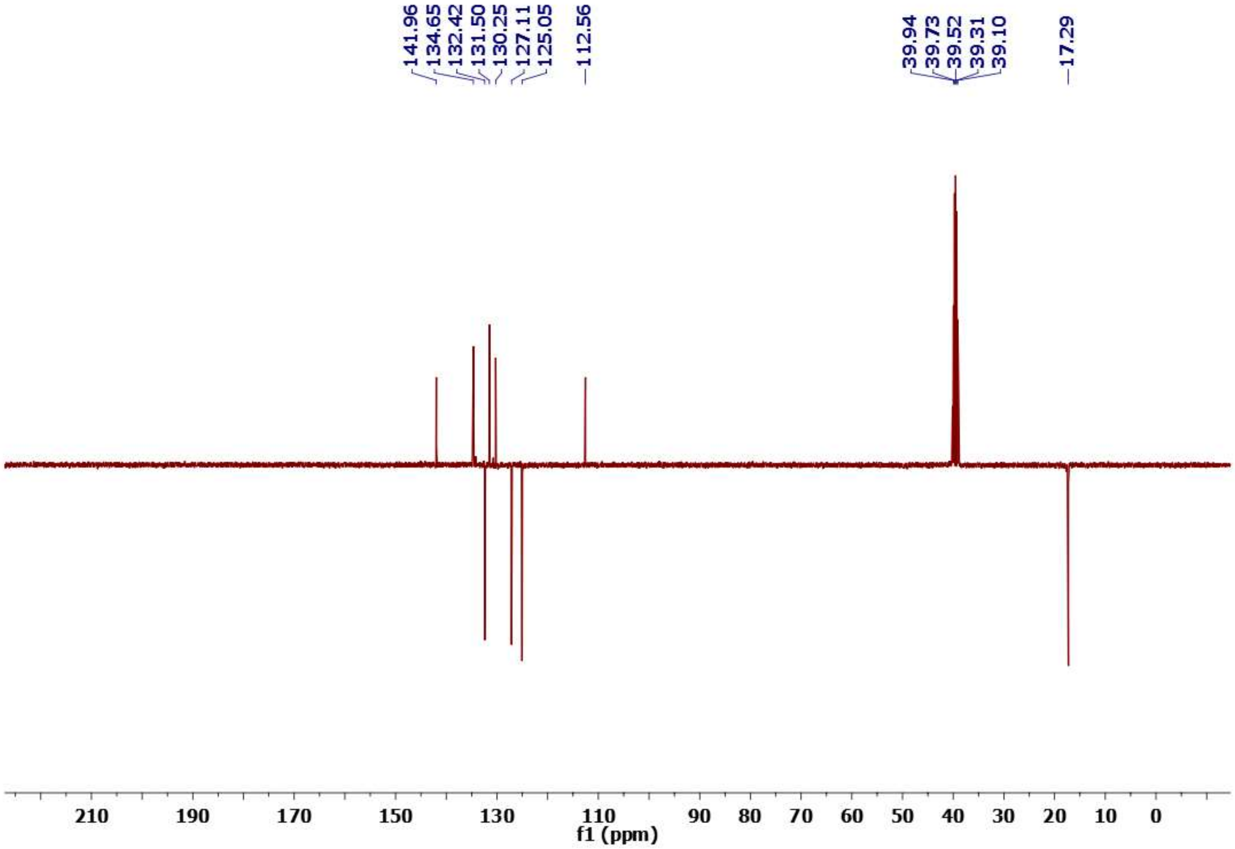

^{1}H-^{1}H gDQCOSY NMR (DMSO-d6) spectrum of (5-chloro-2-methylphenyl)carbamoyl cyanide (2a')

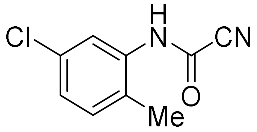

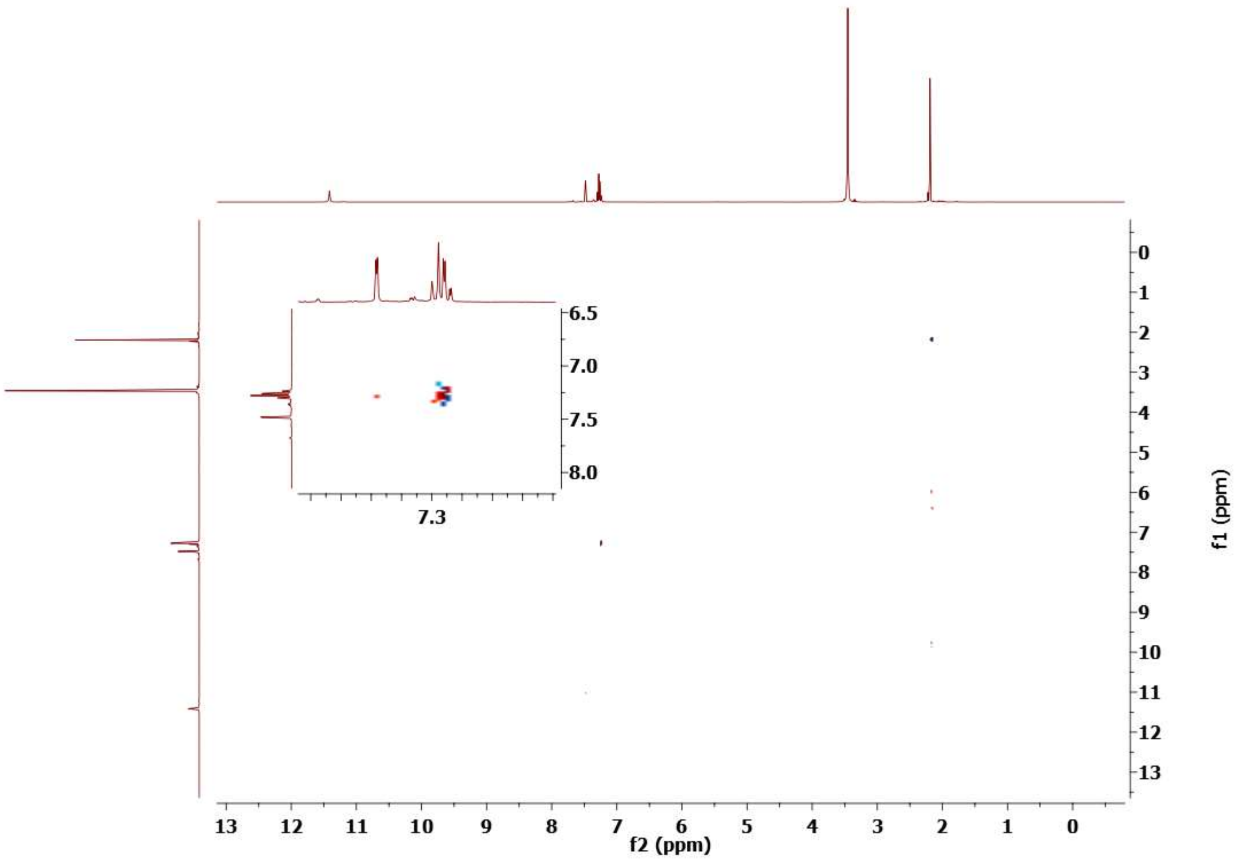

^{1}H-^{13}C-gHSQC NMR (DMSO-d6) spectrum of (5-chloro-2-methylphenyl)carbamoyl cyanide (2a')

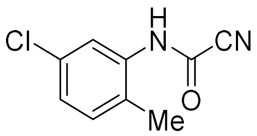

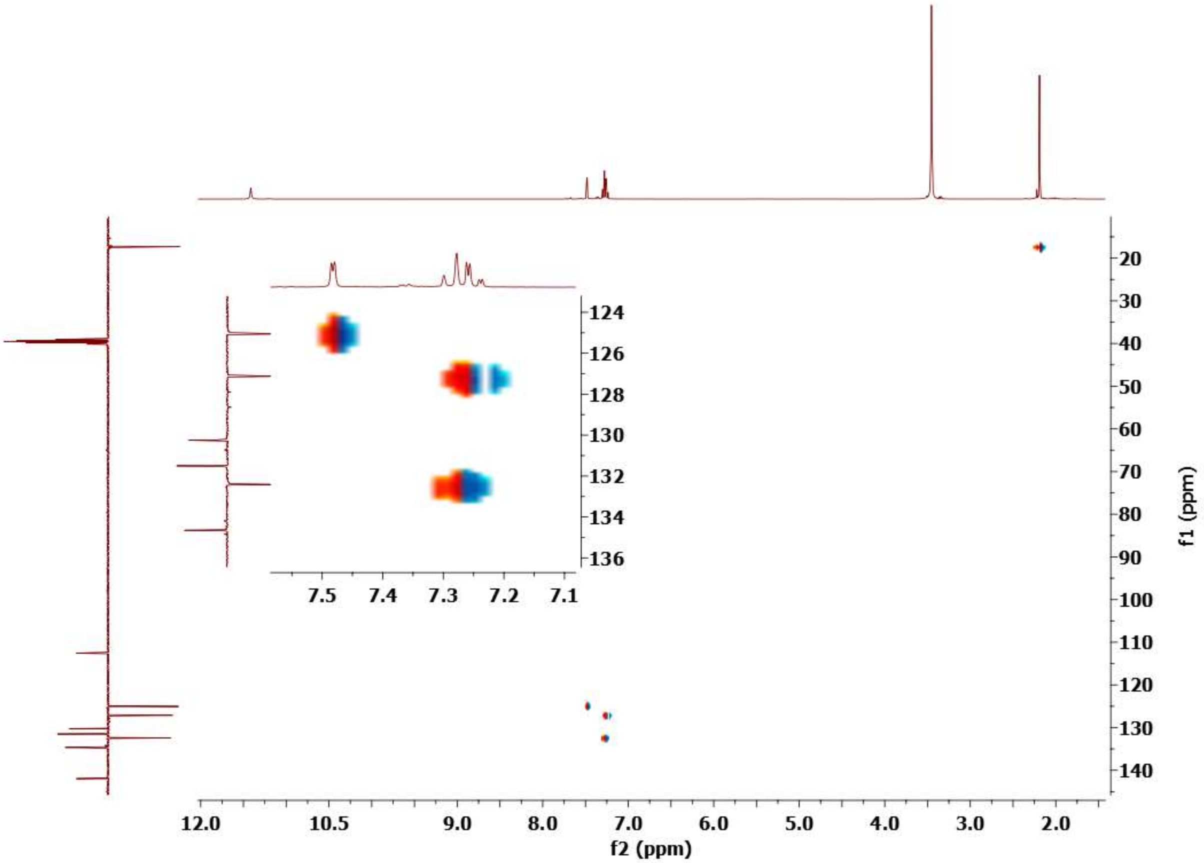

^{1}H-^{13}C-gHMBC NMR (DMSO-d6) spectrum of (5-chloro-2-methylphenyl)carbamoyl cyanide (2a')

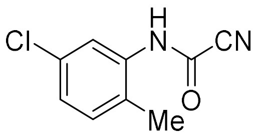

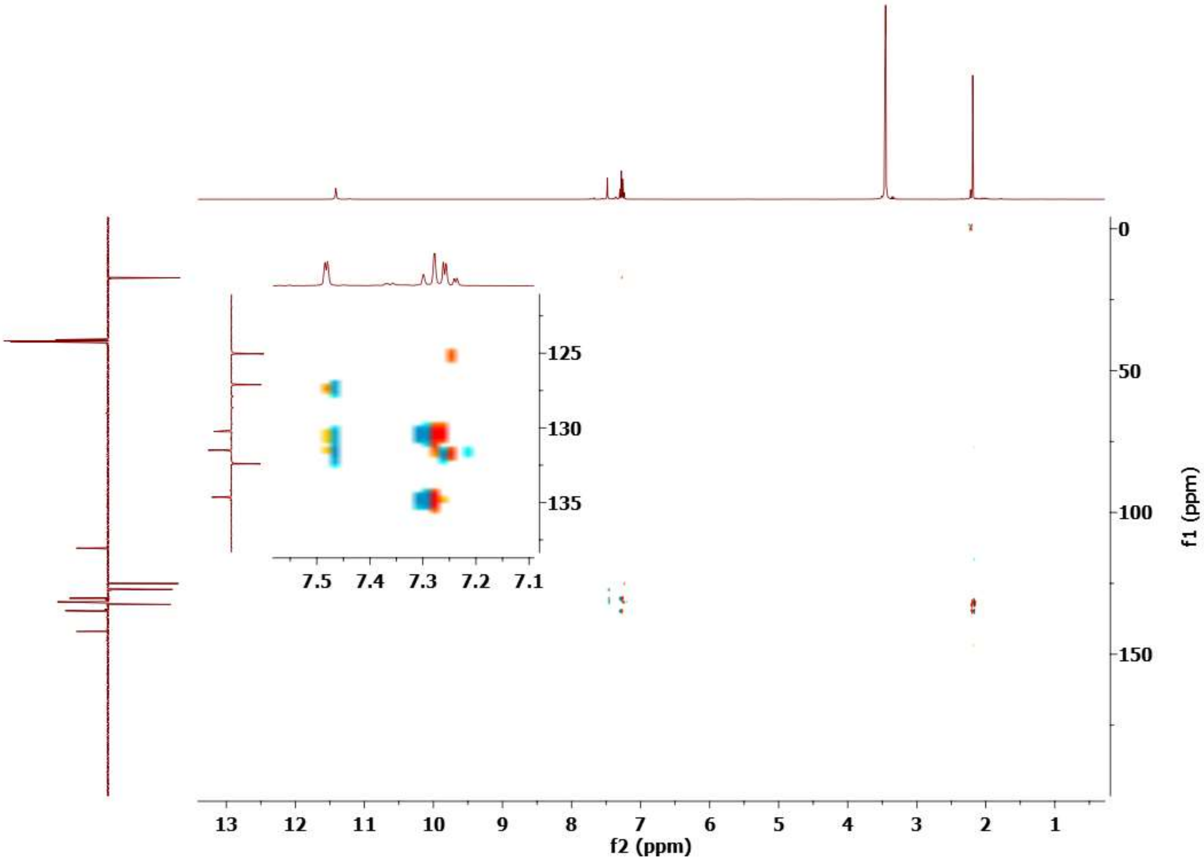

^{1}H NMR (DMSO-d6) spectrum of (2,4-dimethylphenyl)carbamoyl cyanide (2b’)

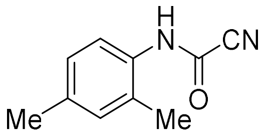

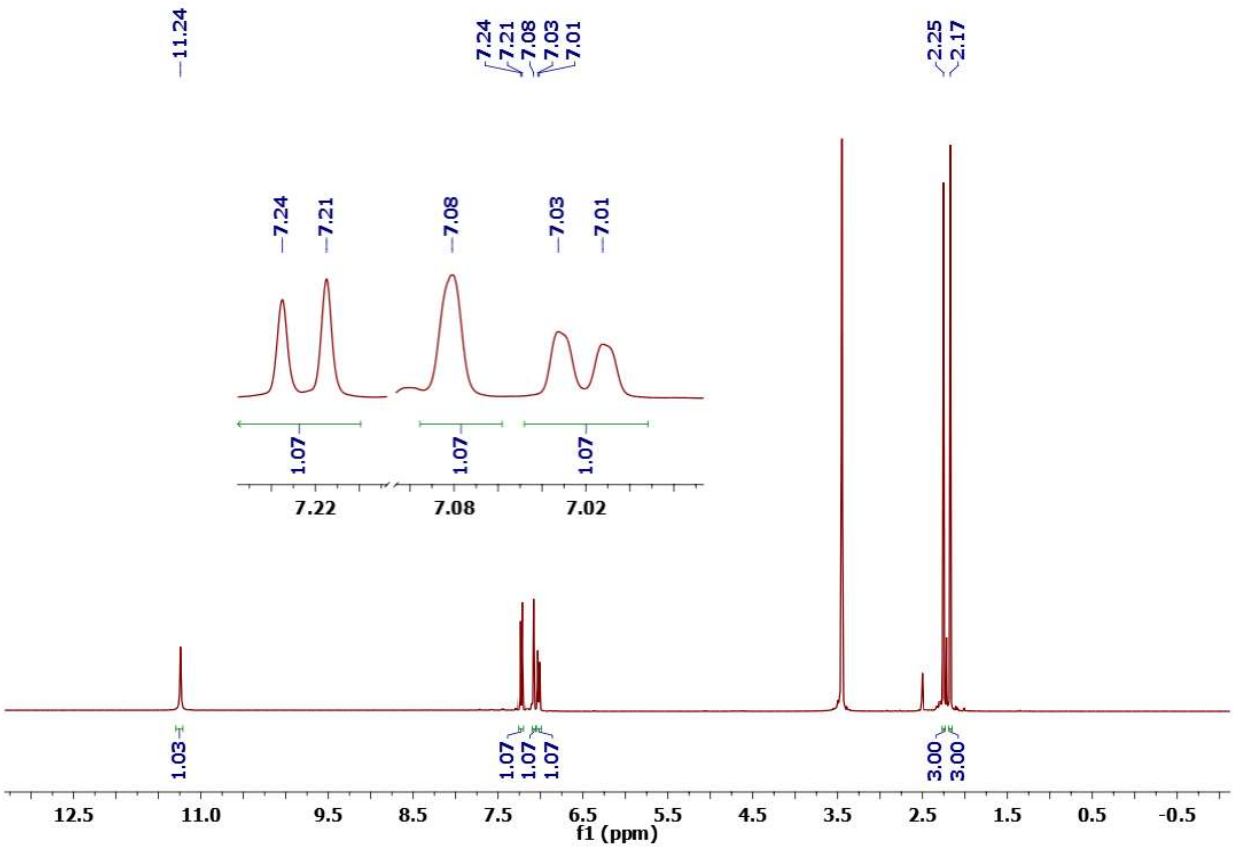

^{13}C NMR (DMSO-d6) spectrum of (2,4-dimethylphenyl)carbamoyl cyanide (2b')

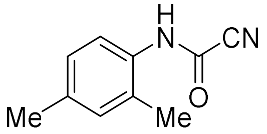

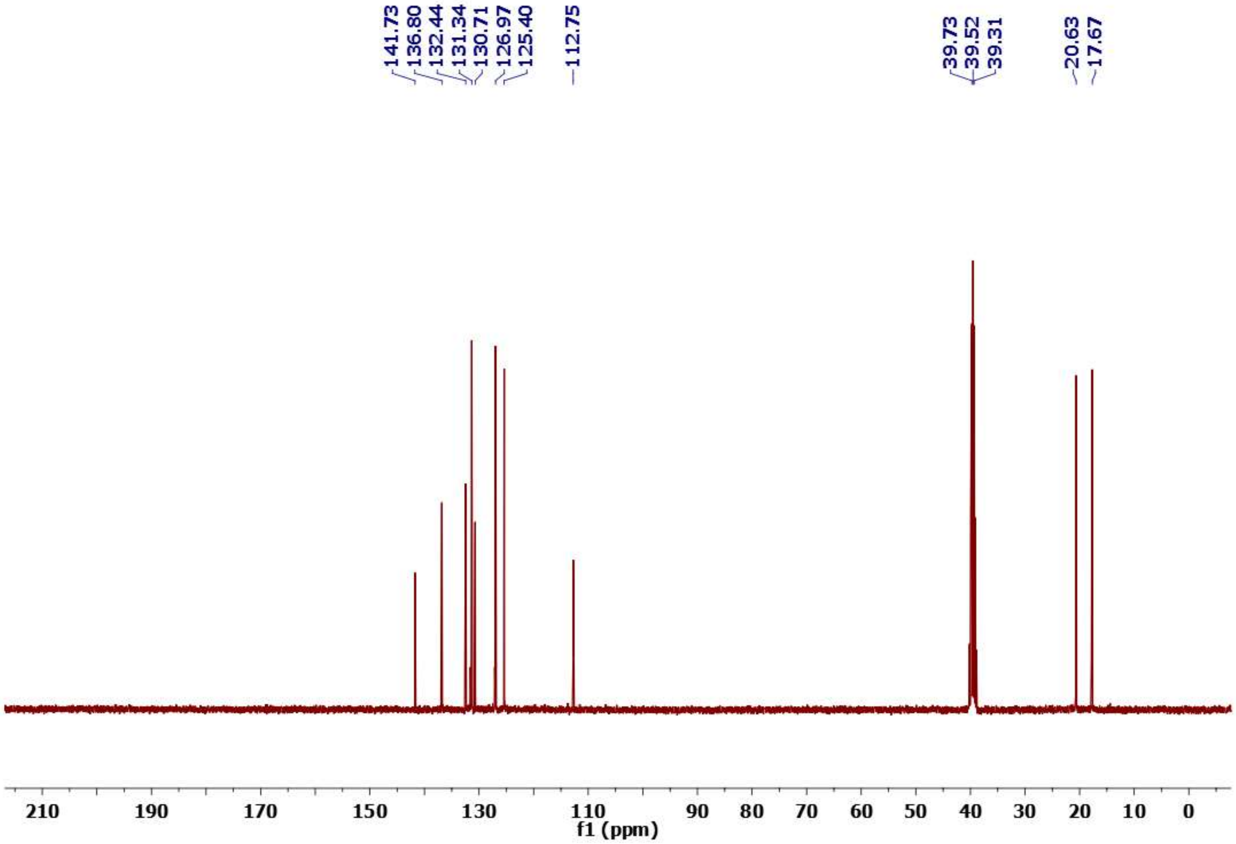

^{13}C CRAPT NMR (DMSO-d6) spectrum of (2,4-dimethylphenyl)carbamoyl cyanide (2b')

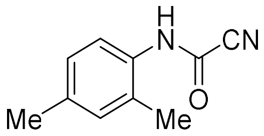

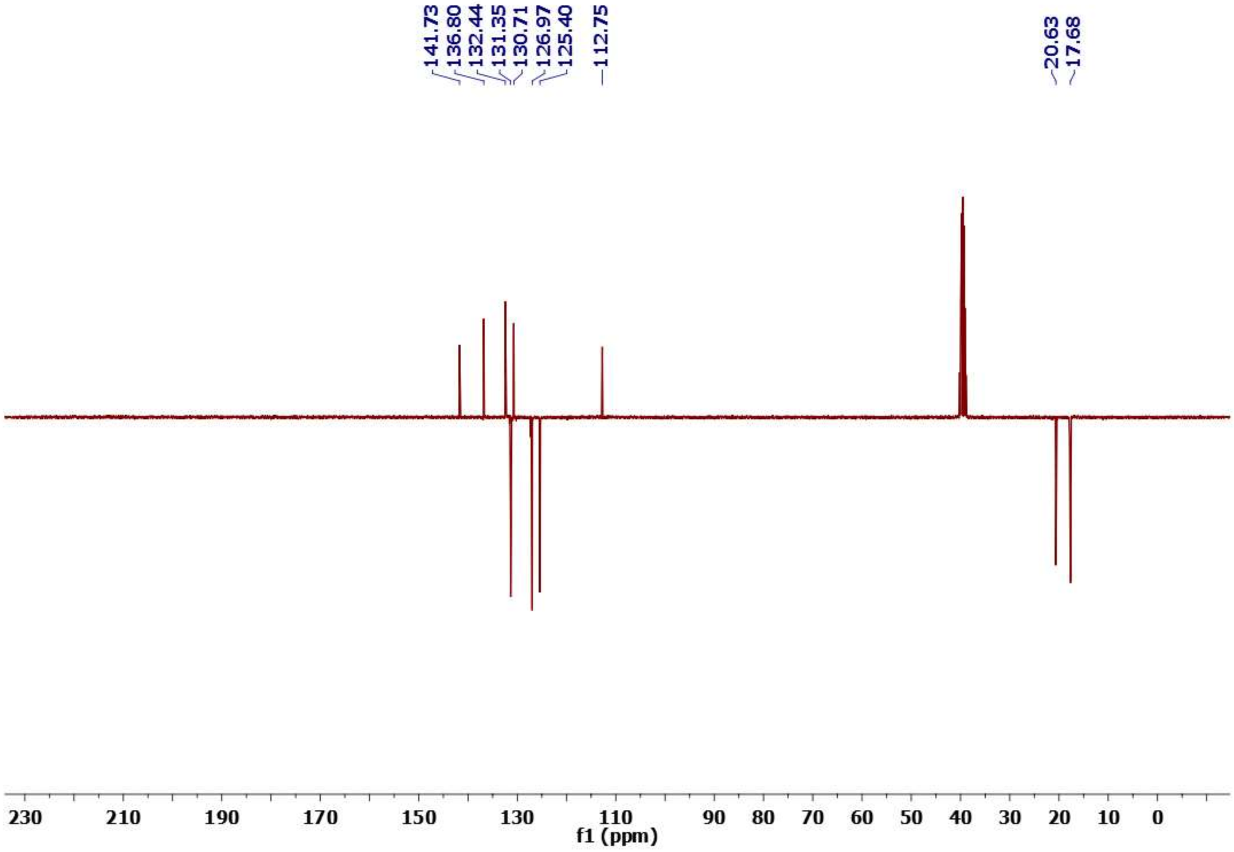

^{1}H-^{1}H gDQCOSY NMR (DMSO-d6) spectrum of (2,4-dimethylphenyl)carbamoyl cyanide (2b')

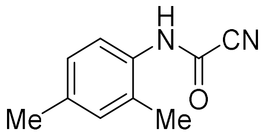

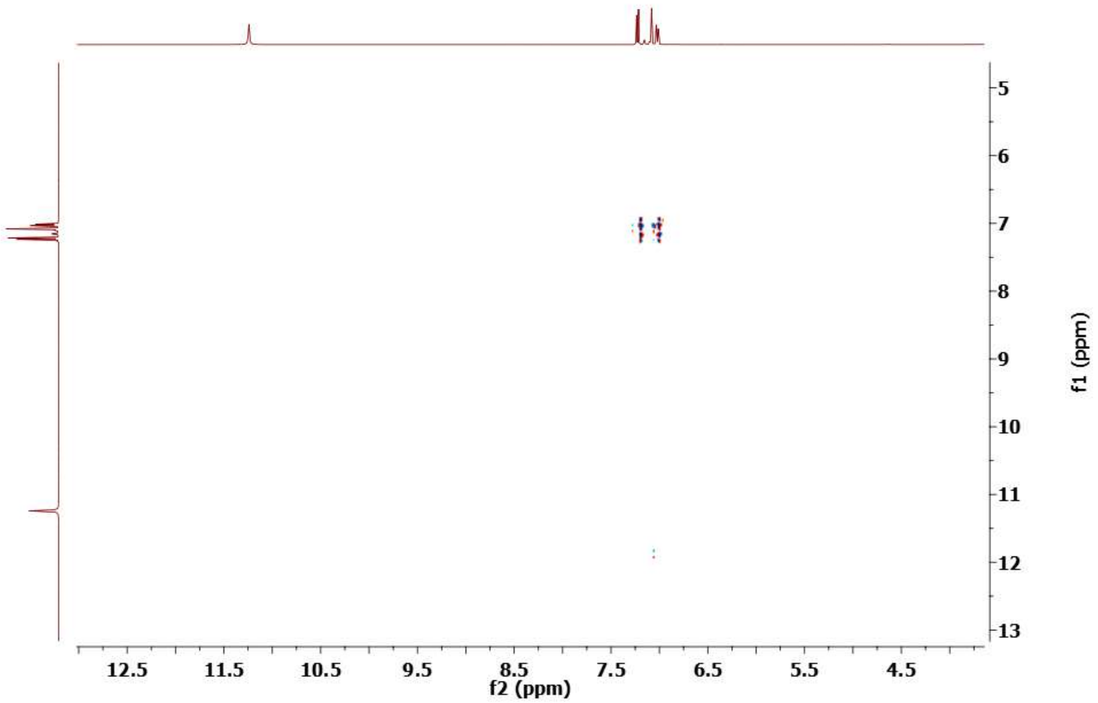

^{1}H-^{13}C-gHSQC NMR (DMSO-d6) spectrum of (2,4-dimethylphenyl)carbamoyl cyanide (2b')

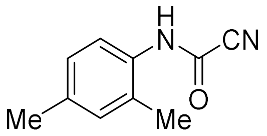

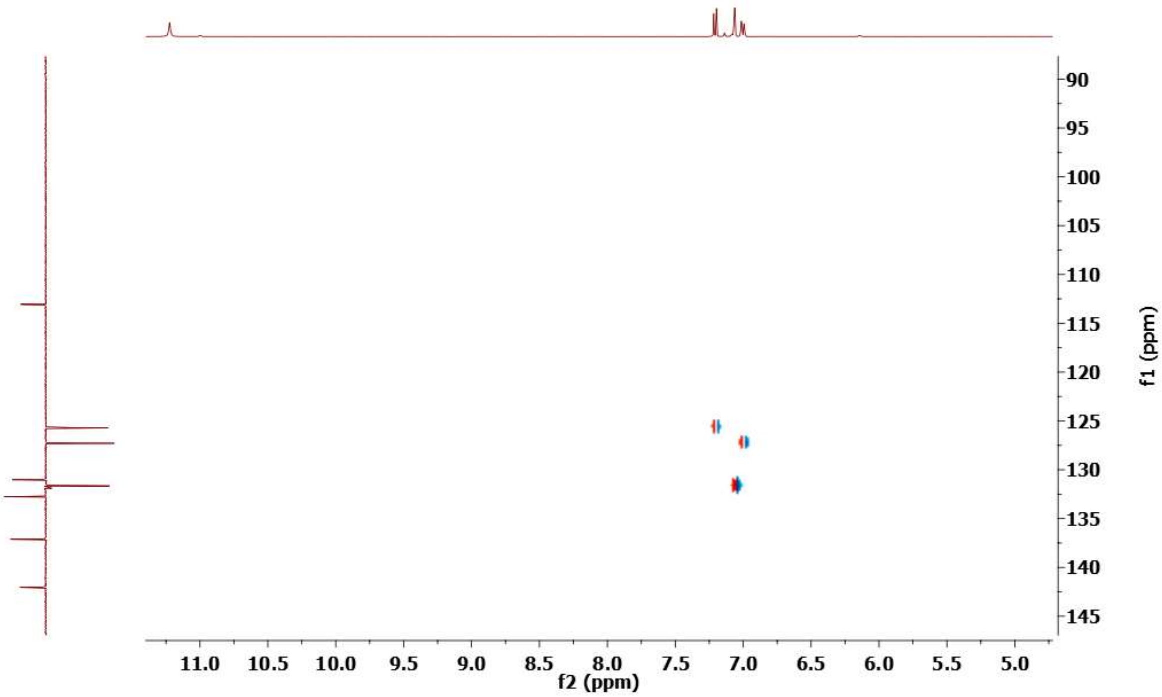

^{1}H NMR ($CDCl_3$) spectrum of mesitylcarbamoyl cyanide (2c')

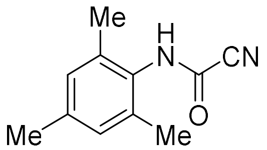

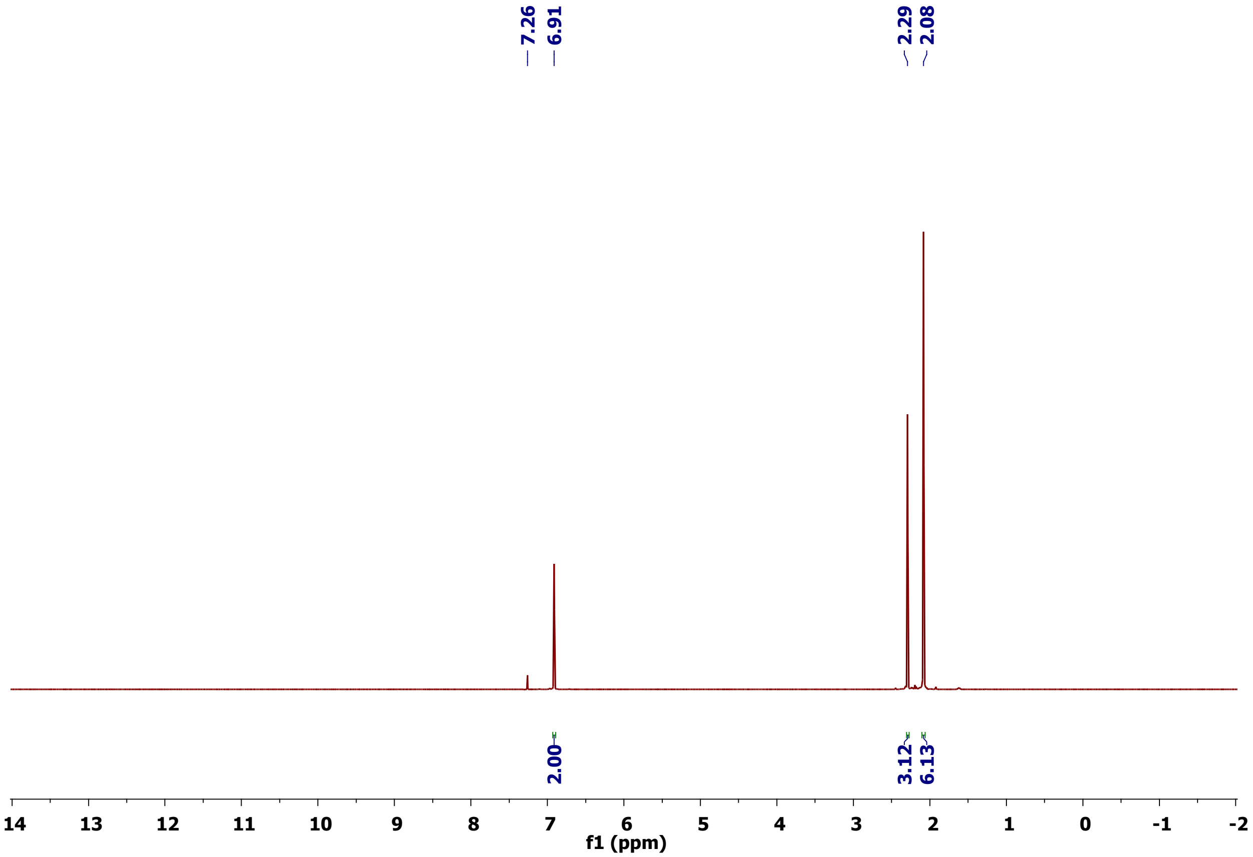

^{13}C NMR ($CDCl_3$) spectrum of mesitylcarbamoyl cyanide (2c')

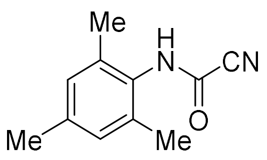

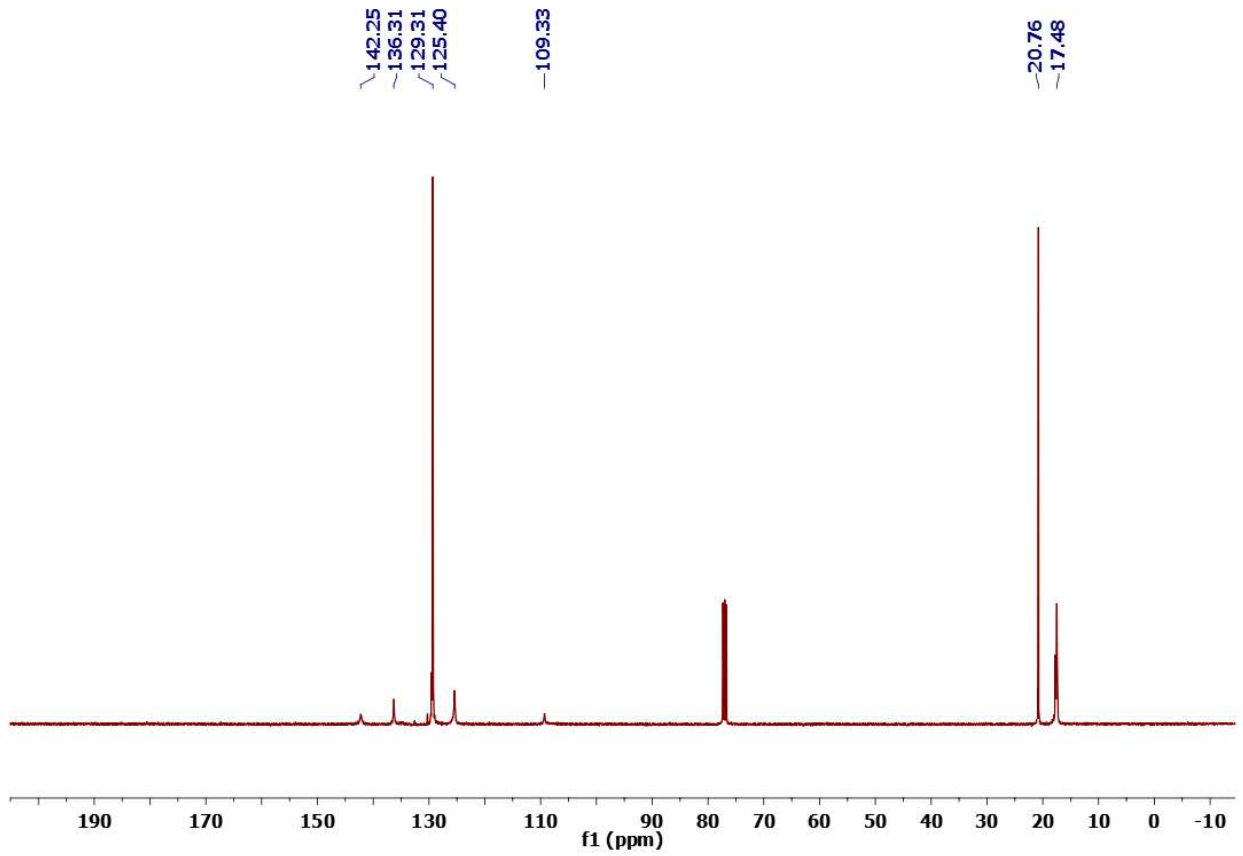

^{13}C CRAPT NMR ($CDCl_3$) spectrum of mesitylcarbamoyl cyanide (2c')

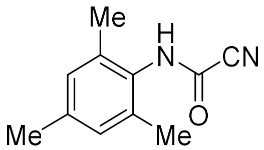

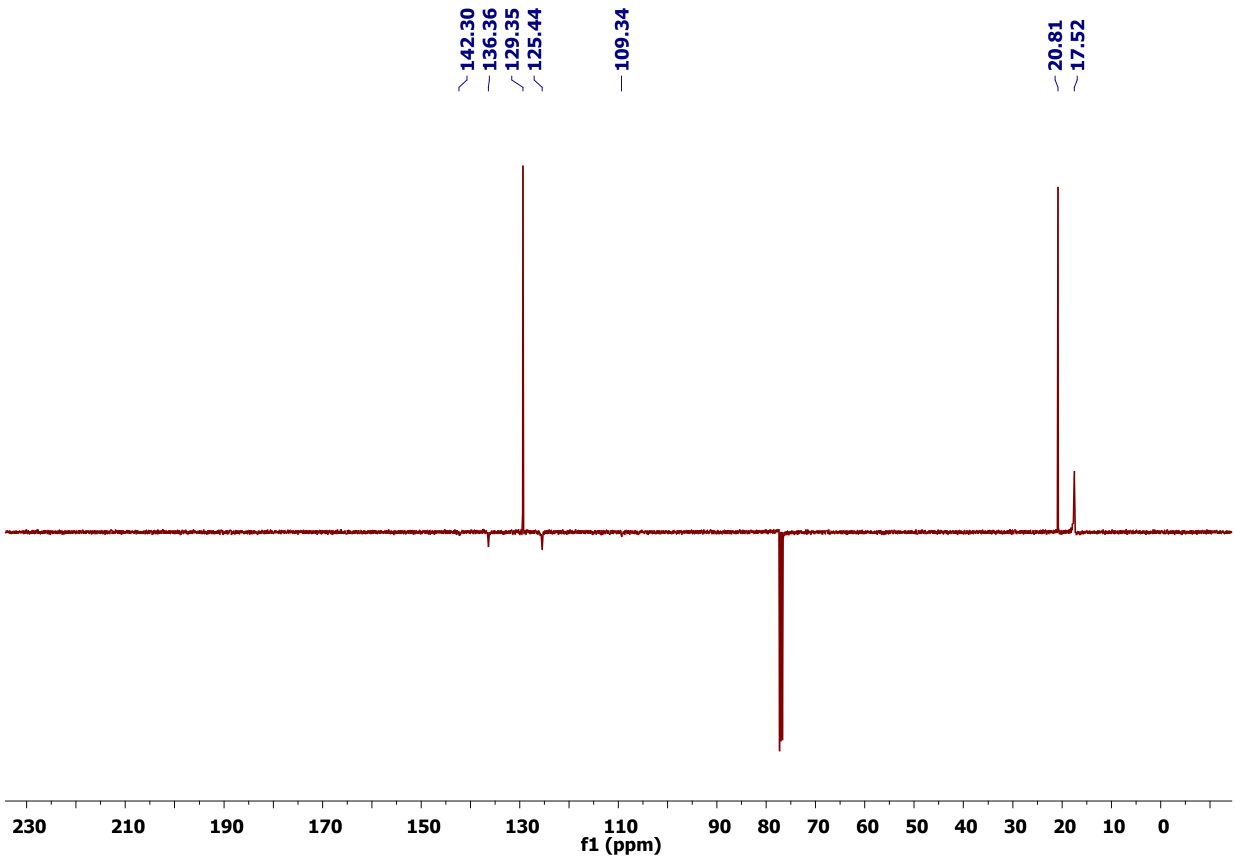

^{1}H-^{13}C-gHSQC NMR ($CDCl_3$) spectrum of mesitylcarbamoyl cyanide (2c’)

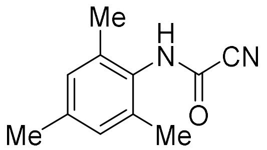

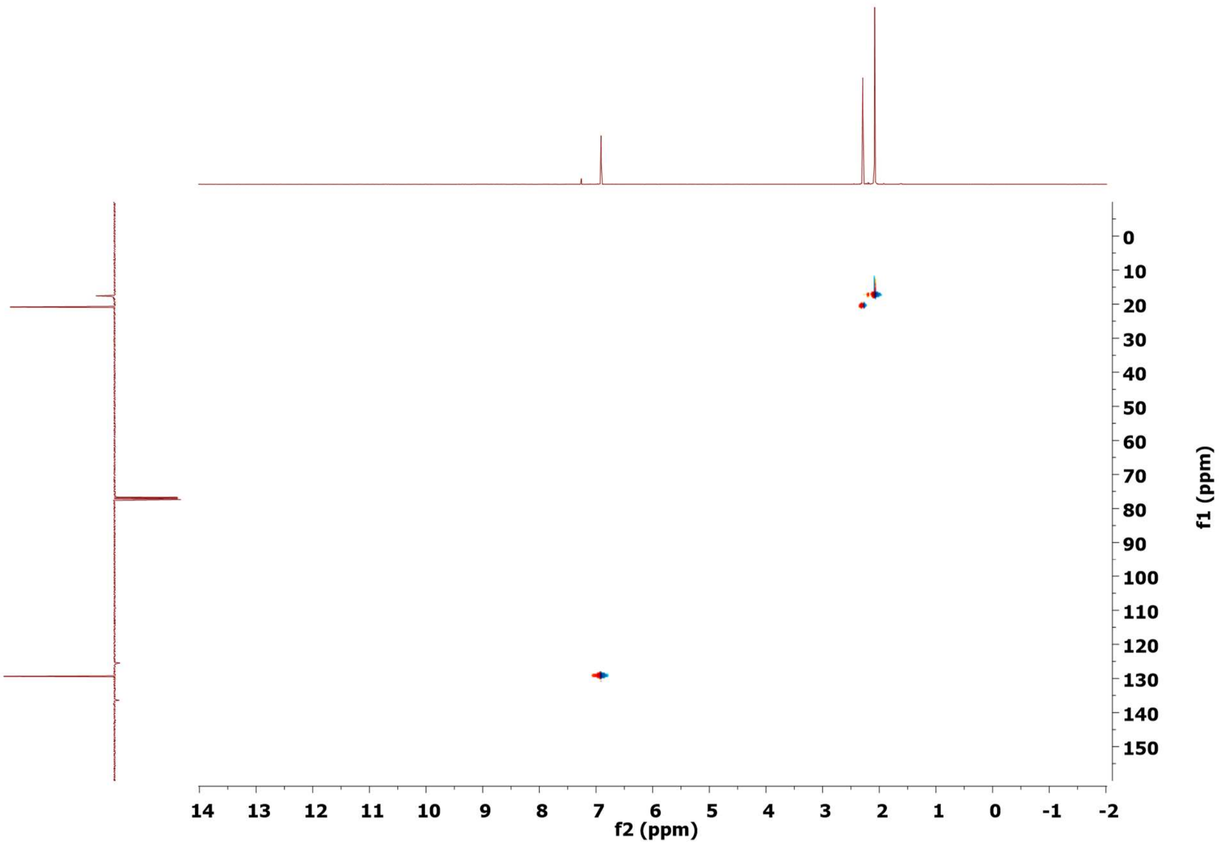

^1H-^{13}C-gHMBC NMR ($CDCl_3$) spectrum of mesitylcarbamoyl cyanide (2c')

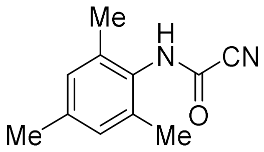

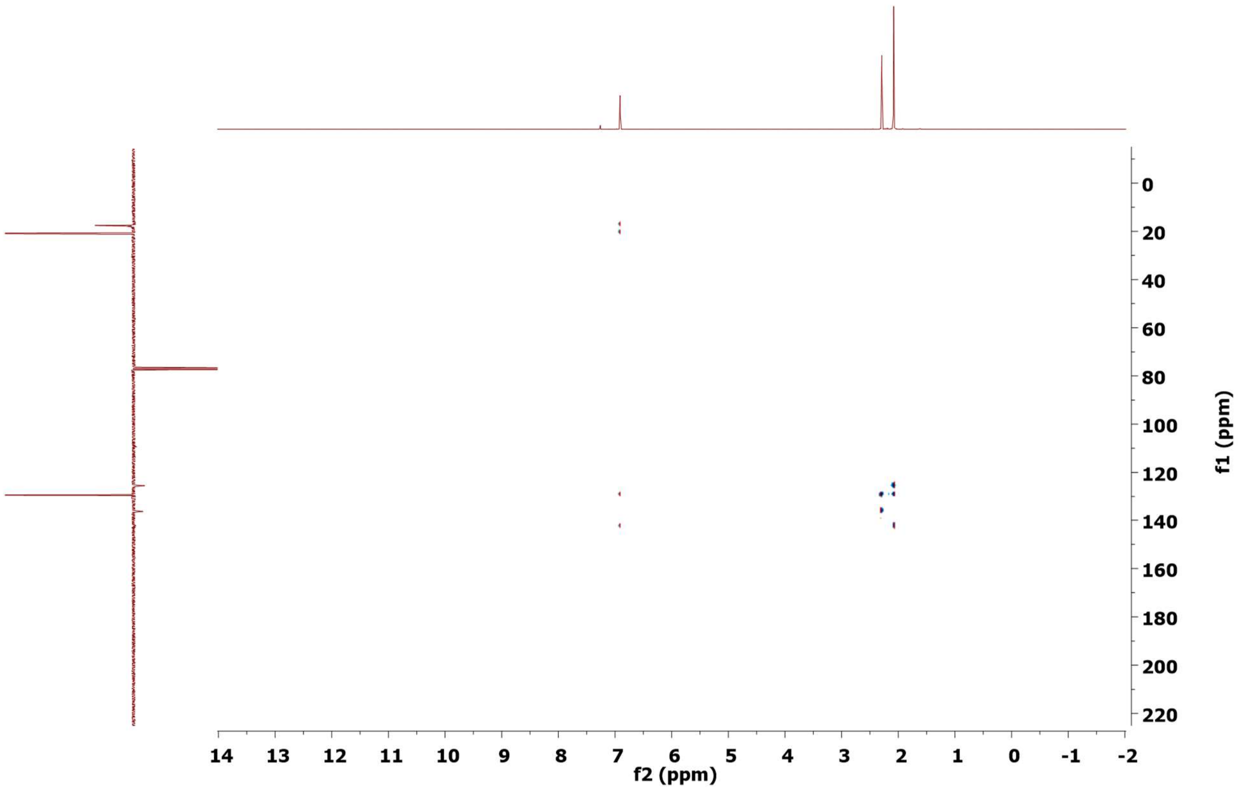

^{1}H NMR (DMSO-d6) spectrum of (2,3-dichlorophenyl)carbamoyl cyanide (2d')

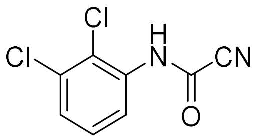

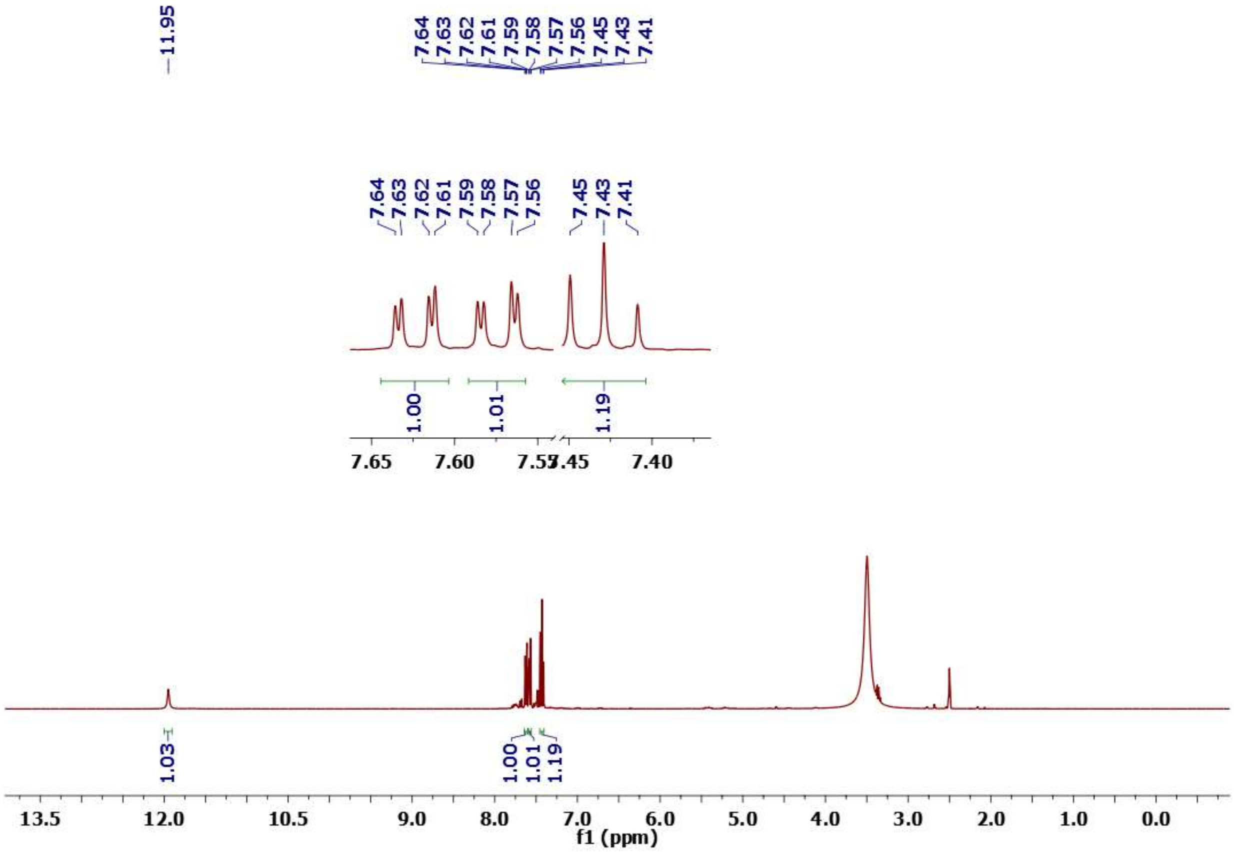

^{13}C NMR (DMSO-d6) spectrum of (2,3-dichlorophenyl)carbamoyl cyanide (2d')

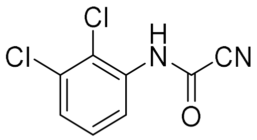

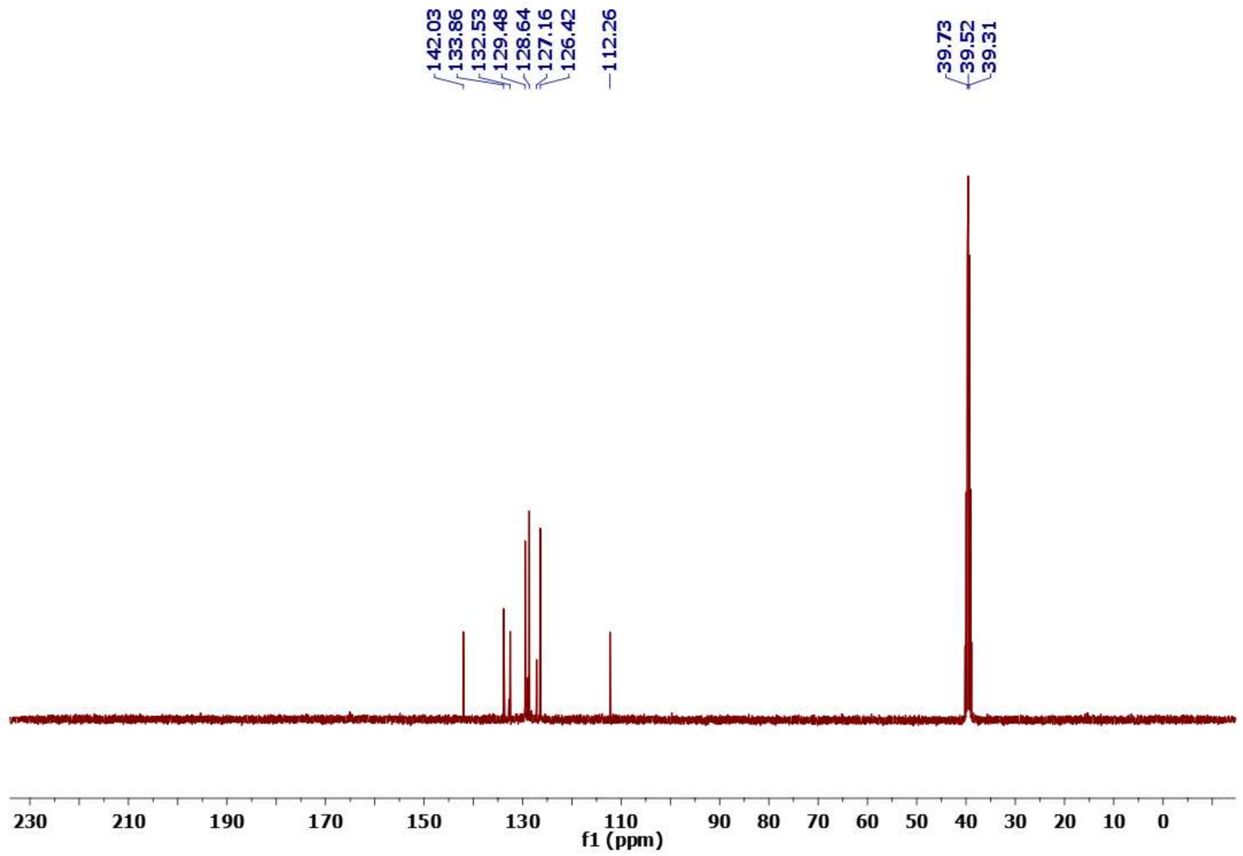

^{13}C CRAPT NMR (DMSO-d6) spectrum of (2,3-dichlorophenyl)carbamoyl cyanide (2d')

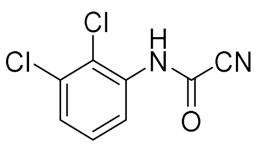

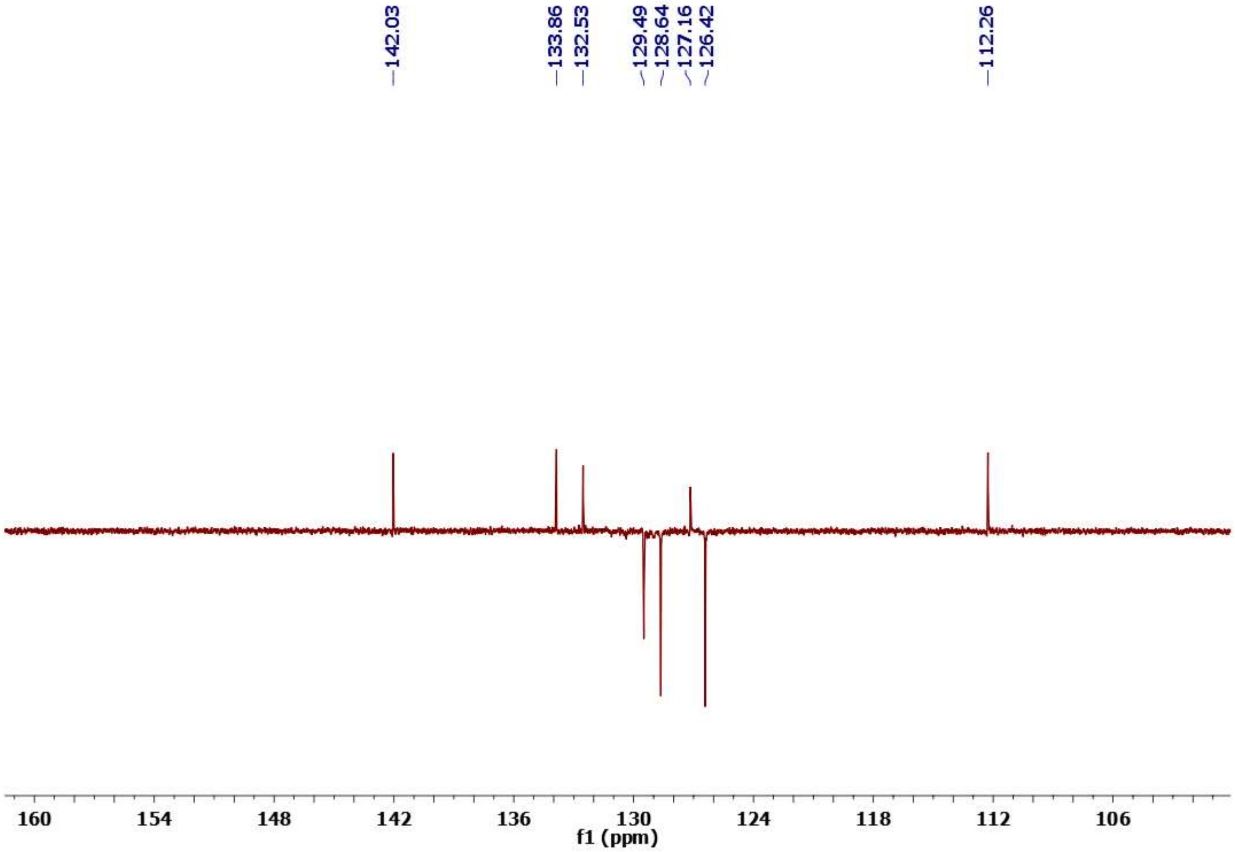

^{1}H-^{1}H-gDQCOSY NMR (DMSO-d6) spectrum of (2,3-dichlorophenyl)carbamoyl cyanide (2d’)

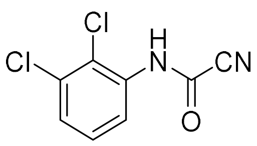

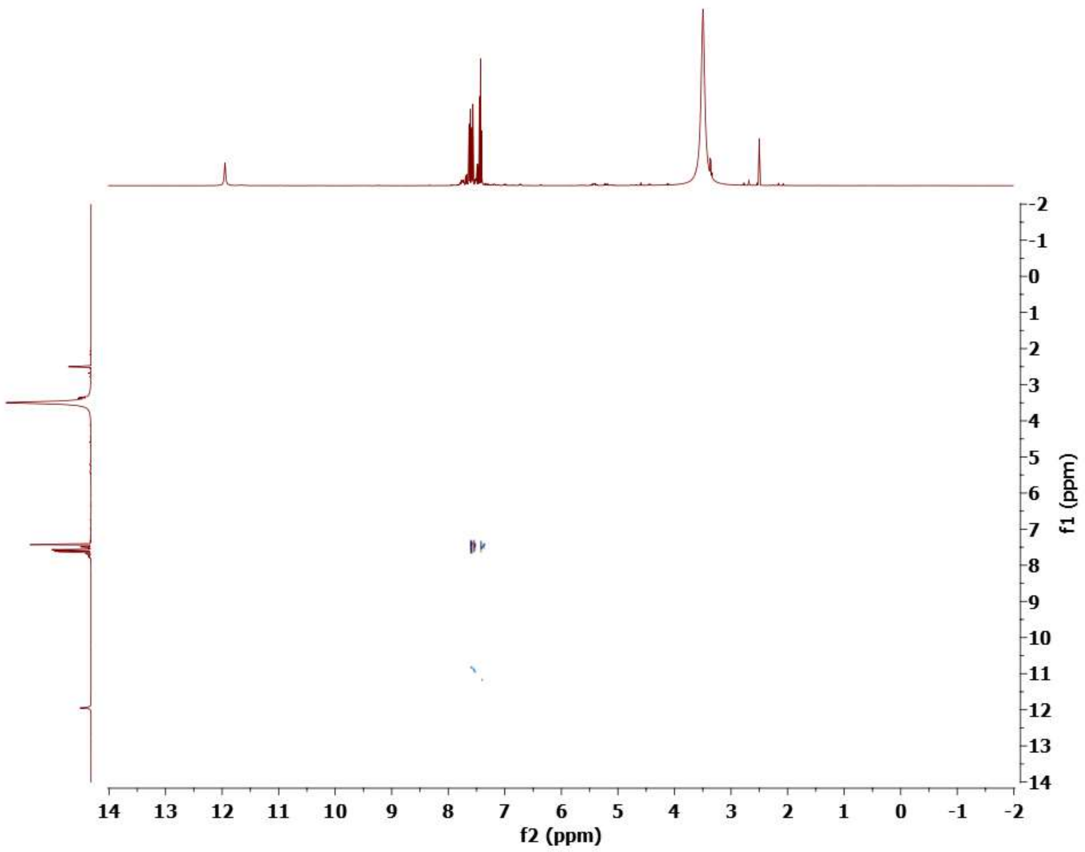

^1H-^{13}C-gHSQC NMR (DMSO-d6) spectrum of (2,3-dichlorophenyl)carbamoyl cyanide (2d')

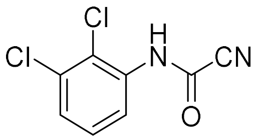

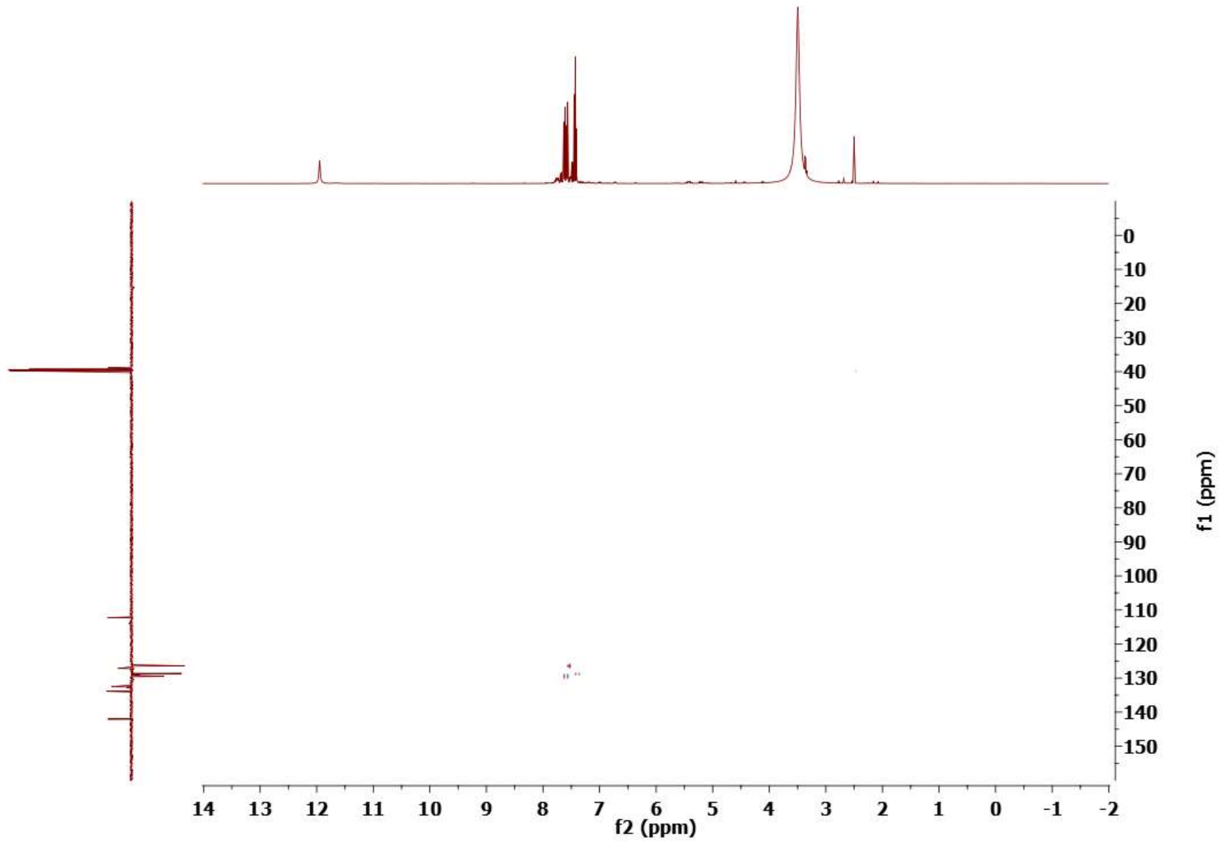

^{1}H-^{13}C-gHMBC NMR (DMSO-d6) spectrum of (2,3-dichlorophenyl)carbamoyl cyanide (2d')

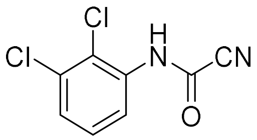

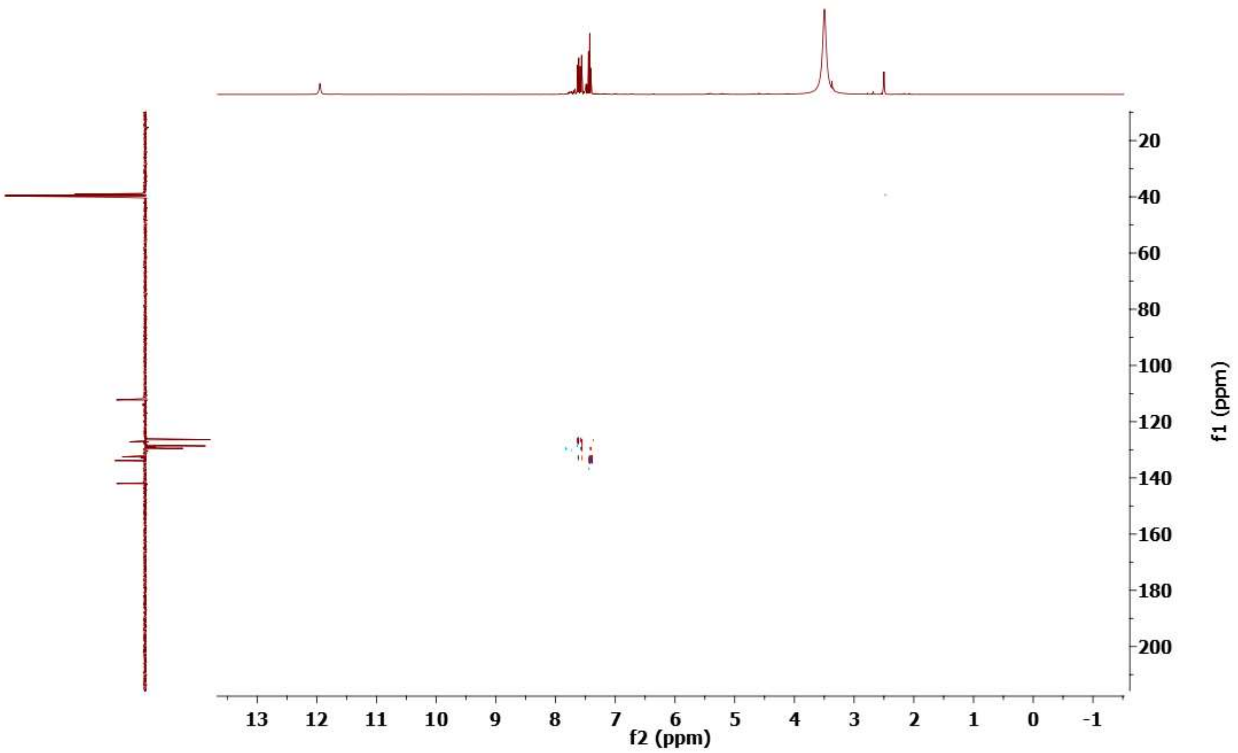

^{1}H NMR (DMSO-d6) spectrum of (2-chloro-5-(trifluoromethyl)phenyl)carbamoyl cyanide (2e')

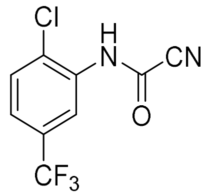

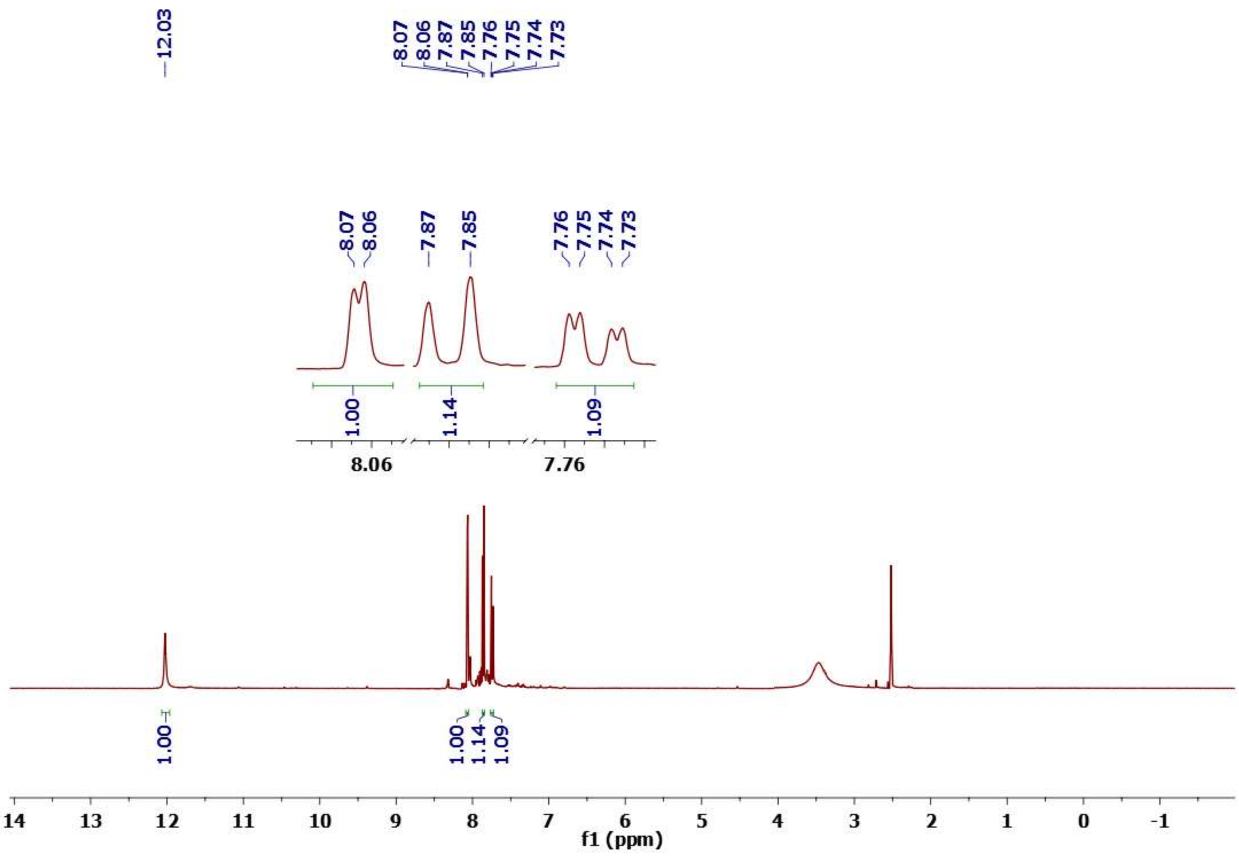

^{13}C NMR (DMSO-d6) spectrum of (2-chloro-5-(trifluoromethyl)phenyl)carbamoyl cyanide (2e')

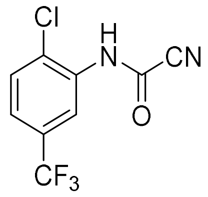

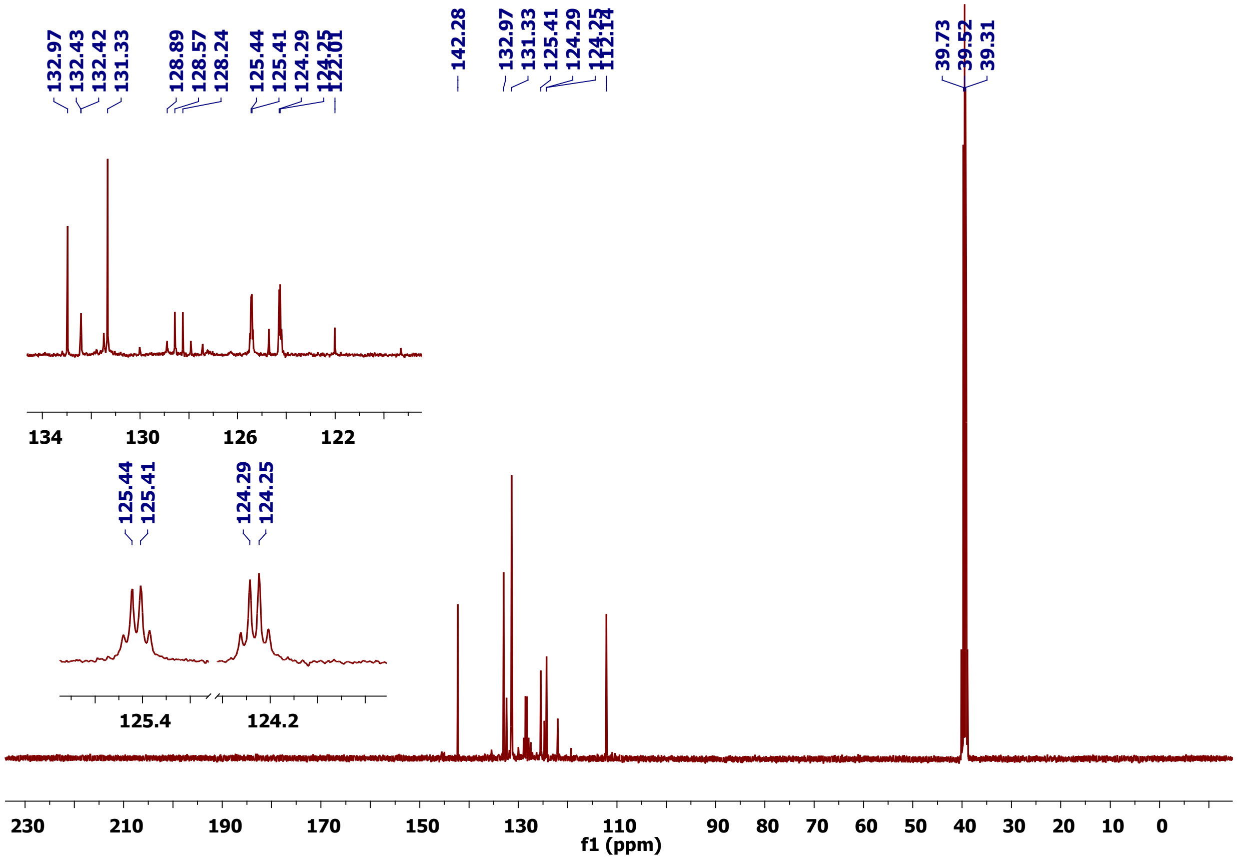

^{13}C CRAPT NMR (DMSO-d6) spectrum of (2-chloro-5-(trifluoromethyl)phenyl)carbamoyl cyanide (2e')

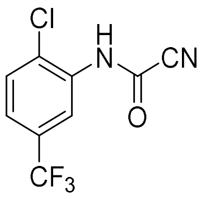

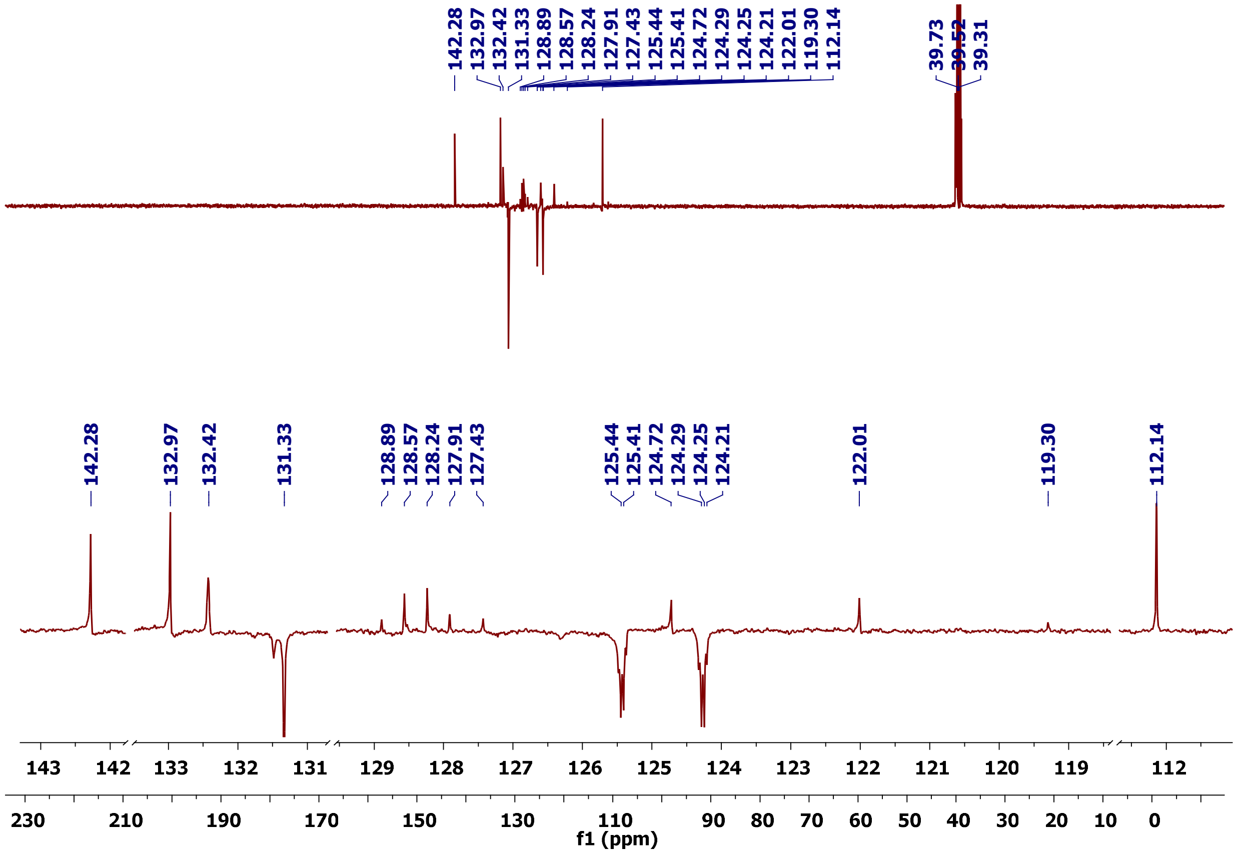

^{1}H-^{1}H-gDQCOSY NMR (DMSO-d6) spectrum of (2-chloro-5-(trifluoromethyl)phenyl)carbamoyl cyanide (2e')

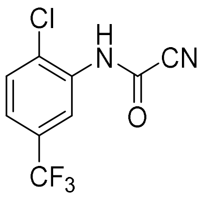

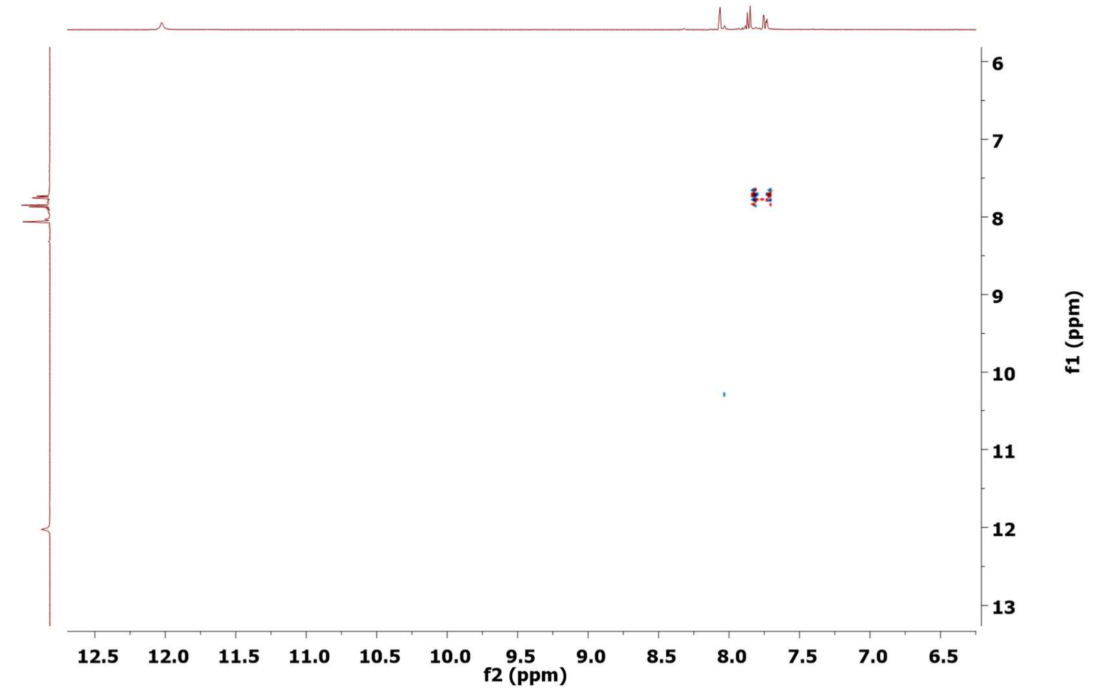

^{1}H-^{13}C-gHSQC NMR (DMSO-d6) spectrum of (2-chloro-5-(trifluoromethyl)phenyl)carbamoyl cyanide (2e')

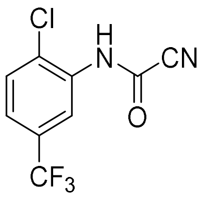

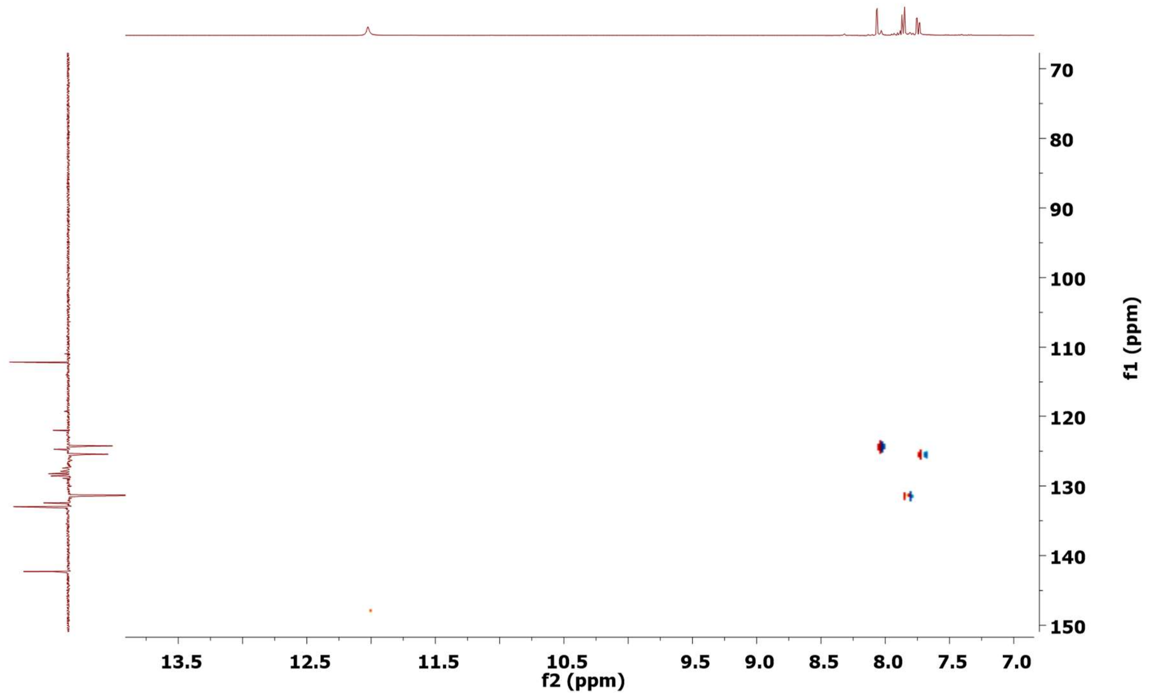

^{1}H-^{13}C-gHMBC NMR (DMSO-d6) spectrum of (2-chloro-5-(trifluoromethyl)phenyl)carbamoyl cyanide (2e')

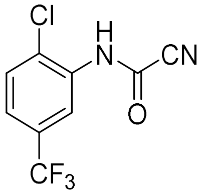

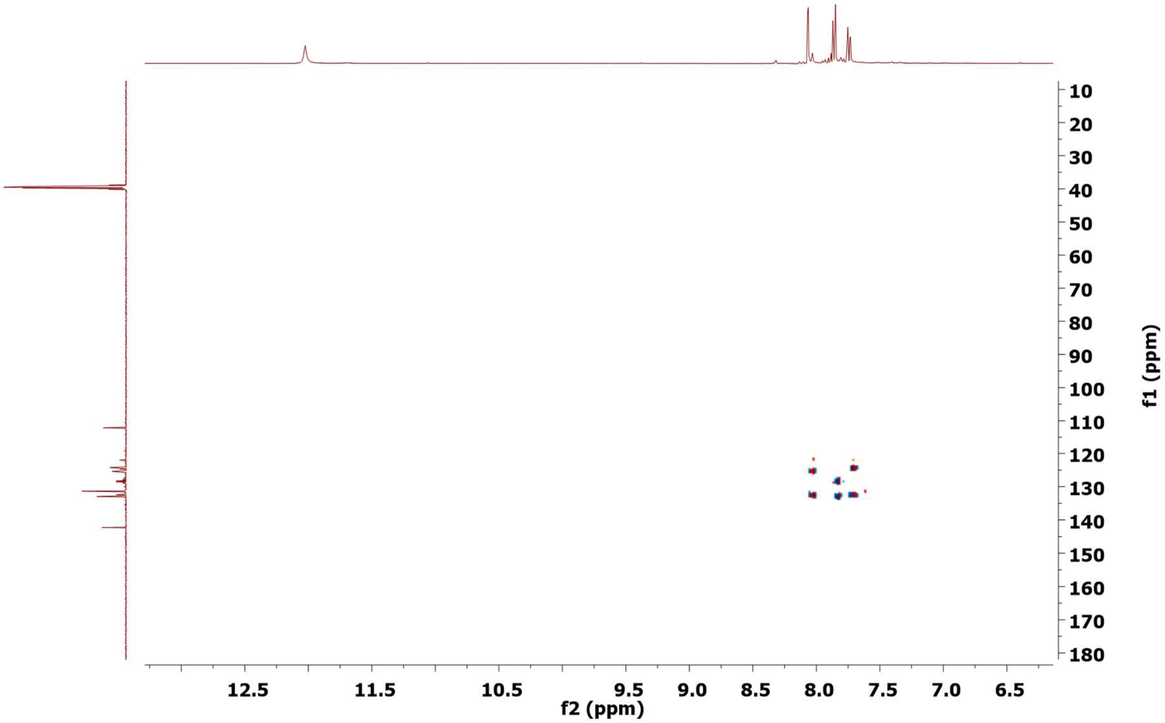

^{1}H NMR (DMSO-d6) spectrum of (2,6-dichlorophenyl)carbamoyl cyanide (2f')

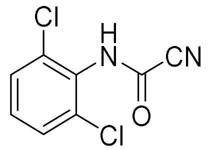

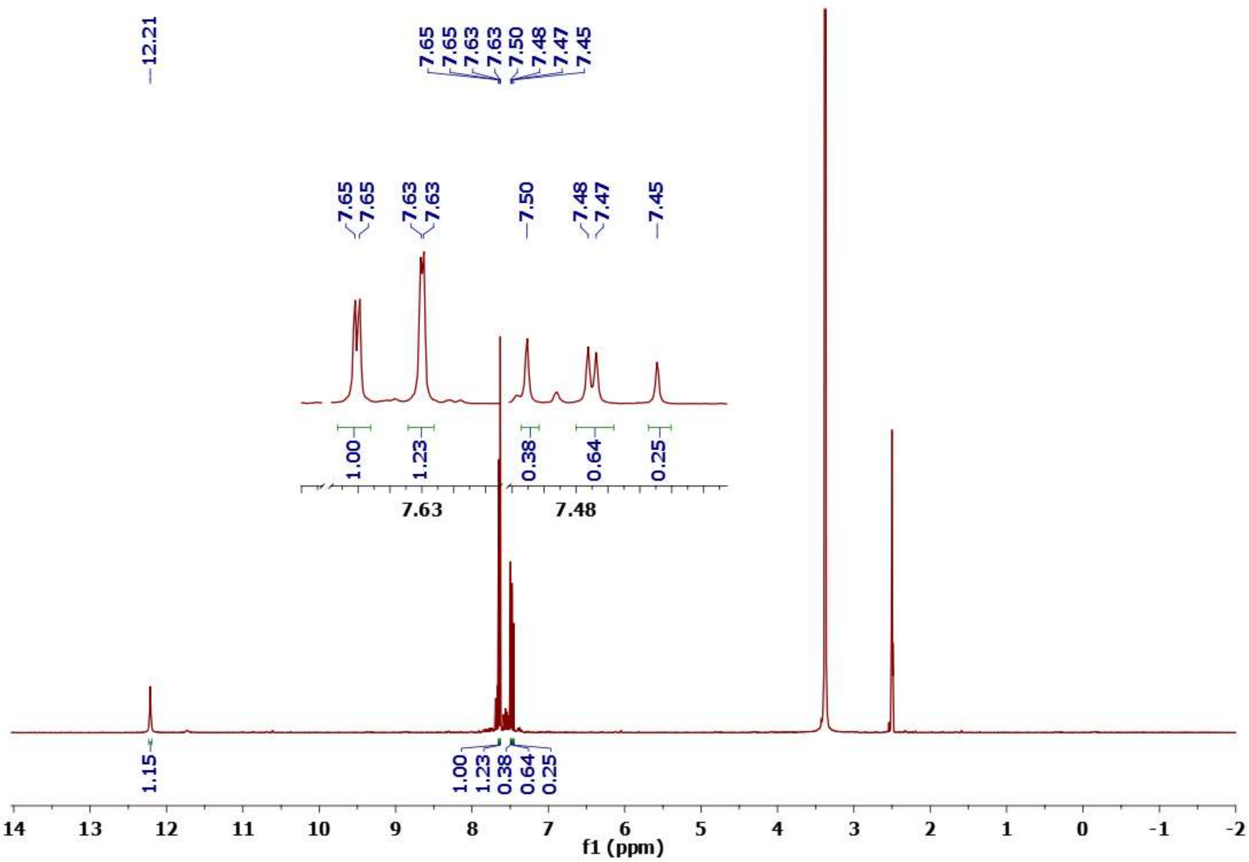

^{13}C CRAPT NMR (DMSO-d6) spectrum of (2,6-dichlorophenyl)carbamoyl cyanide (2f')

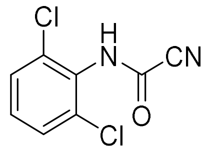

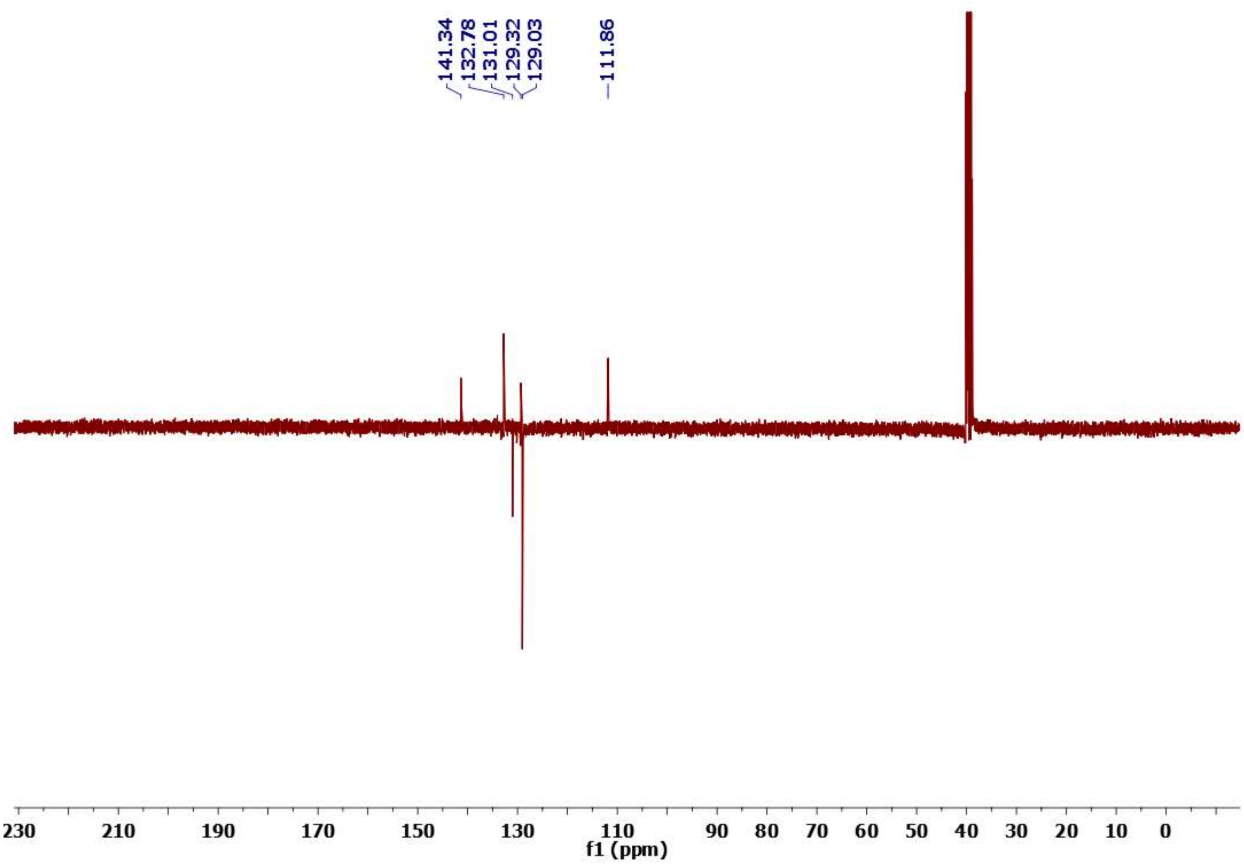

^{1}H NMR (DMSO-d6) spectrum of (4-bromophenyl)carbamoyl cyanide (2g')

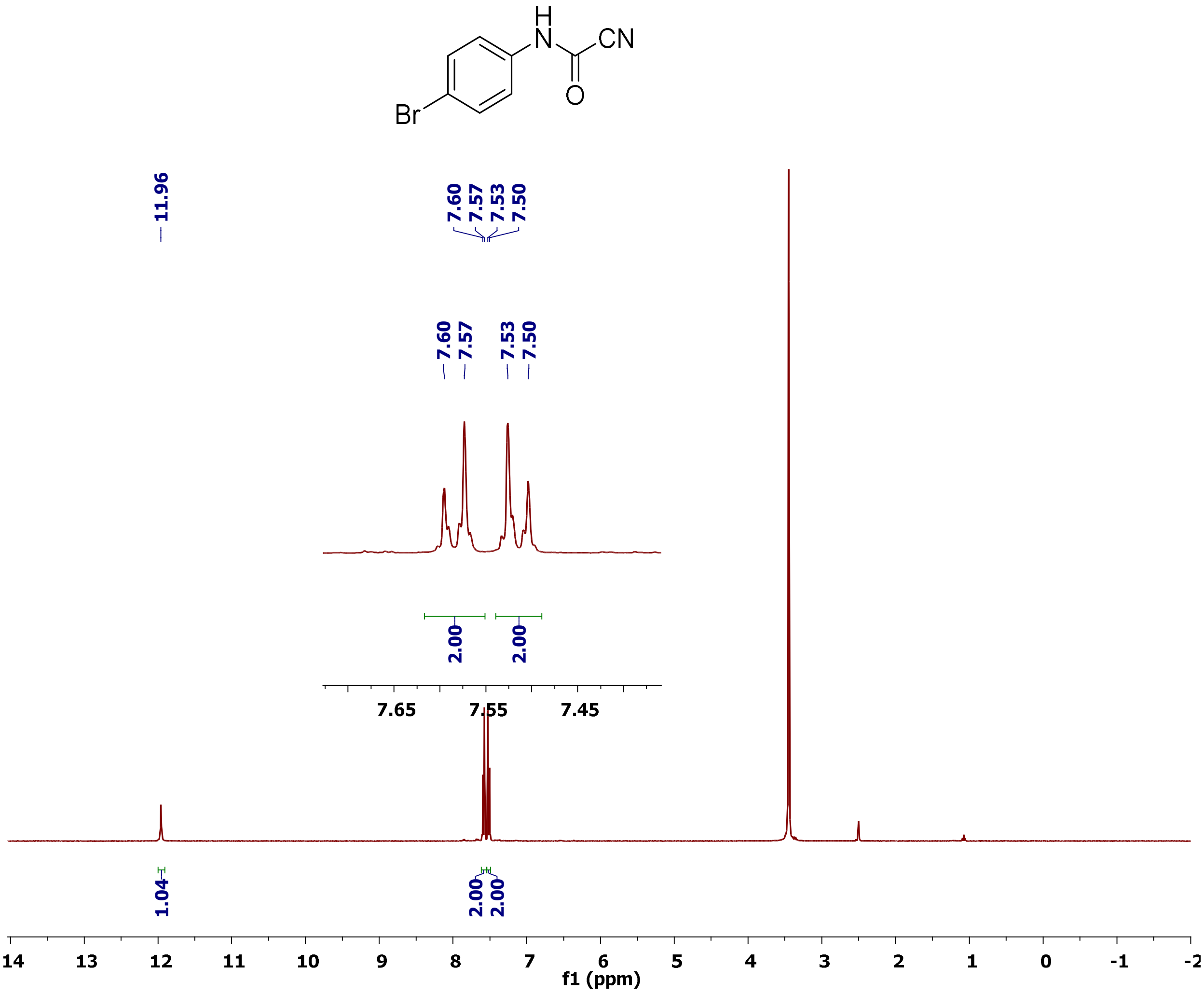

^{13}C NMR (DMSO-d6) spectrum of (4-bromophenyl)carbamoyl cyanide (2g’)

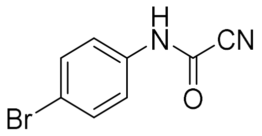

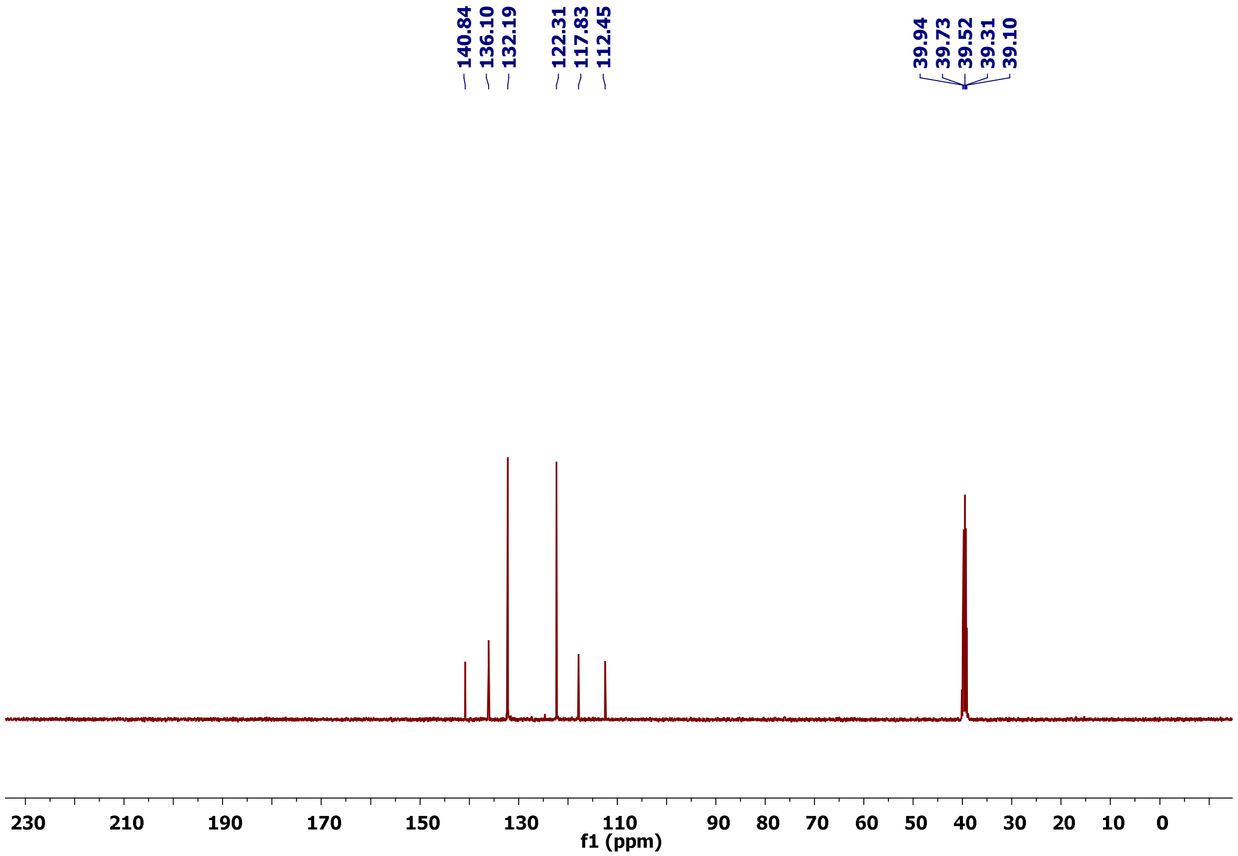

^{13}C CRAPT NMR (DMSO-d6) spectrum of (4-bromophenyl)carbamoyl cyanide (2g’)

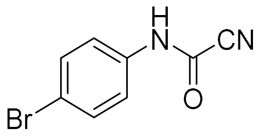

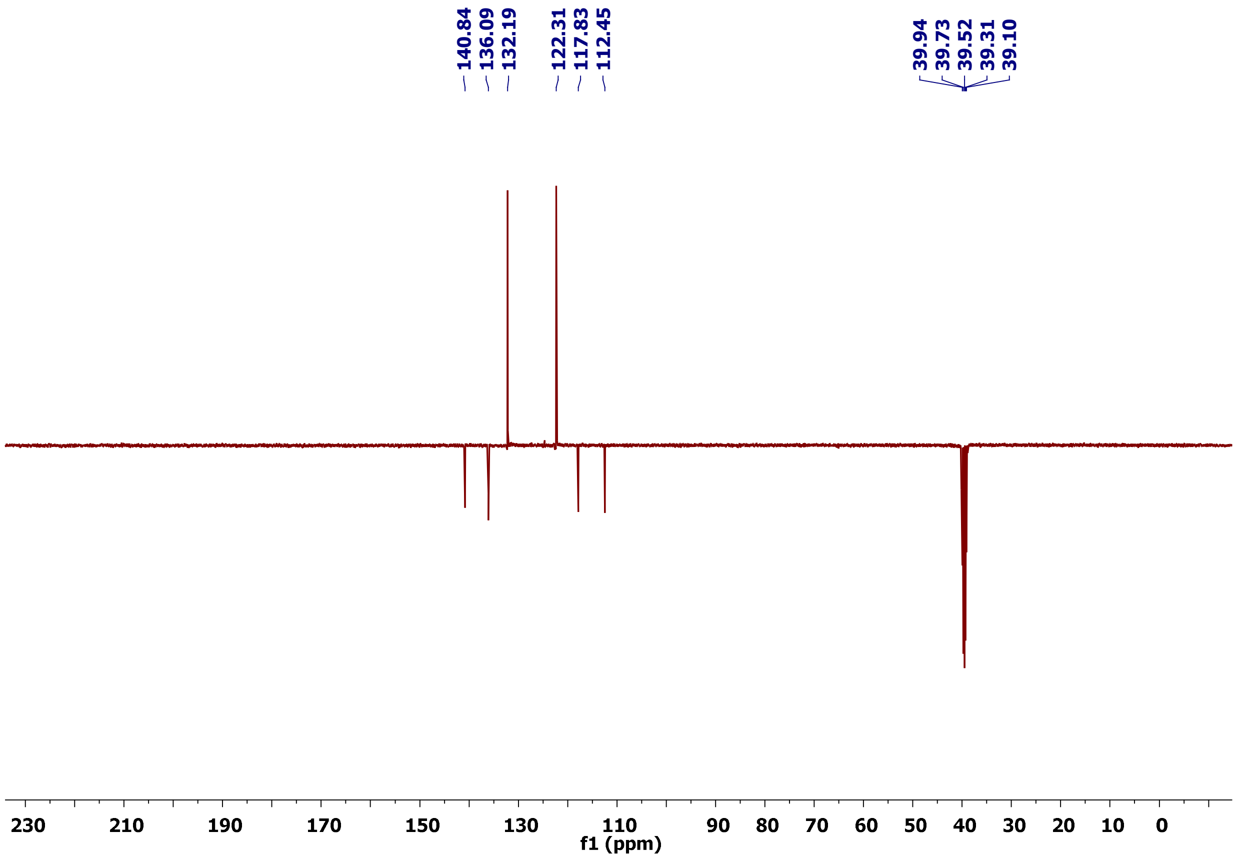

^{1}H-^{1}H gDQCOSY NMR (DMSO-d6) spectrum of (4-bromophenyl)carbamoyl cyanide (2g')

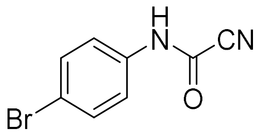

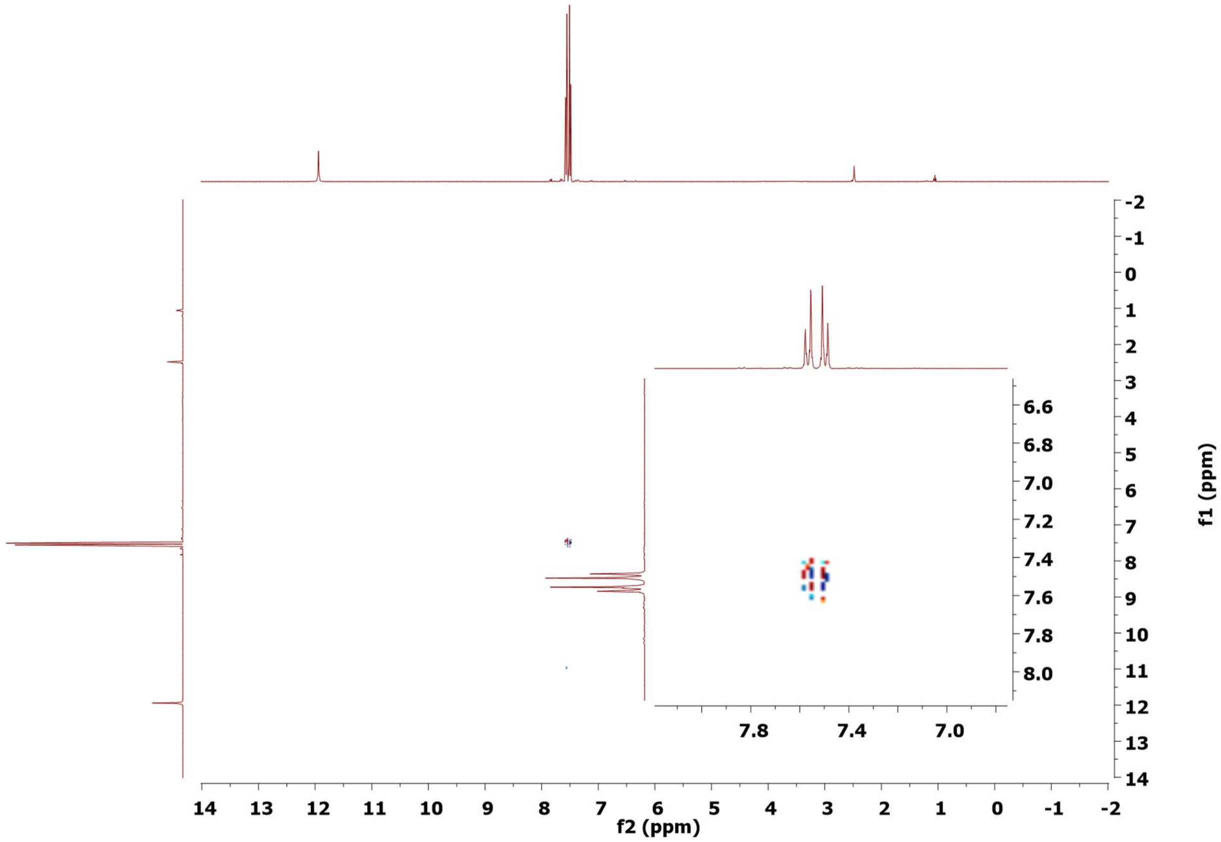

^{1}H-^{13}C-gHSQC NMR (DMSO-d6) spectrum of (4-bromophenyl)carbamoyl cyanide (2g')

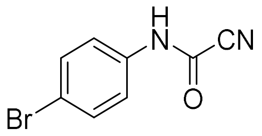

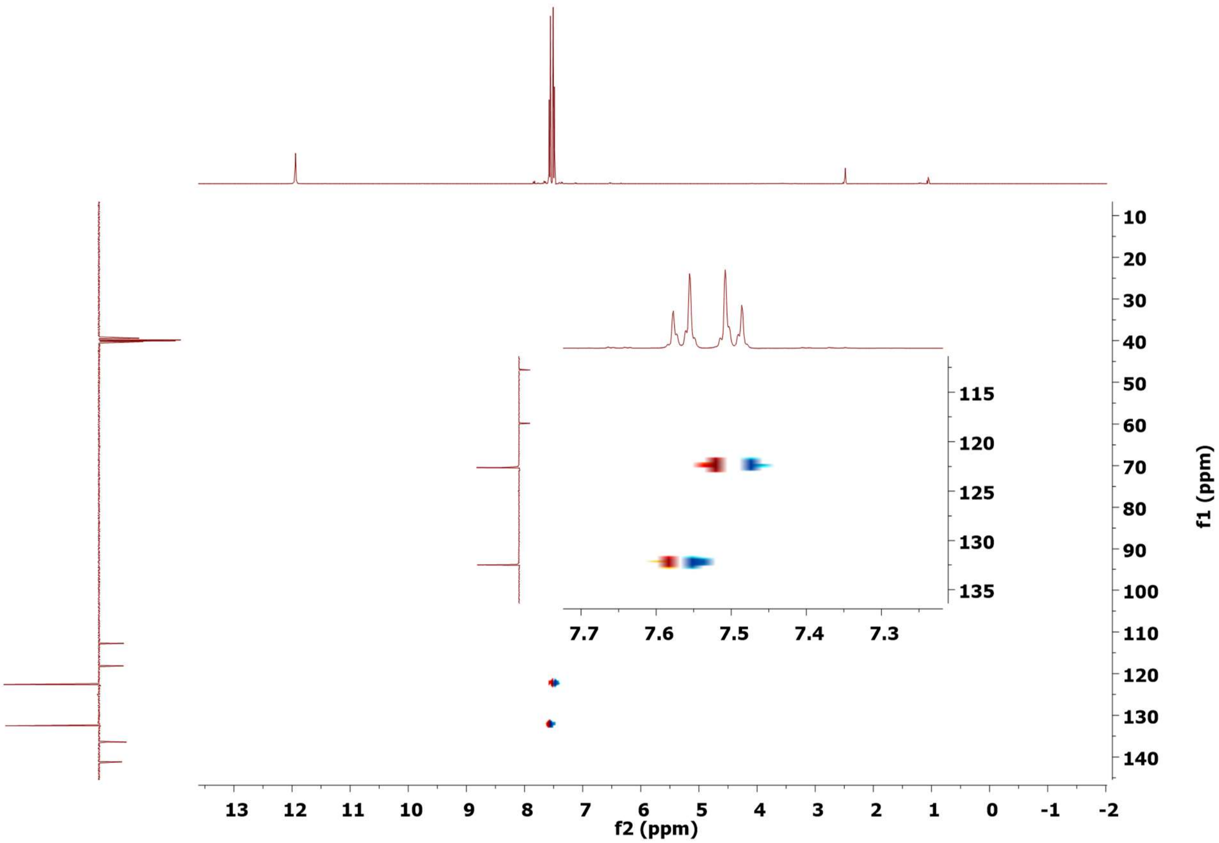

^{1}H-^{13}C-gHMBC NMR (DMSO-d_6) spectrum of (4-bromophenyl)carbamoyl cyanide (2g')

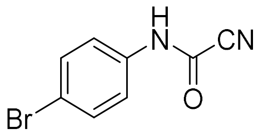

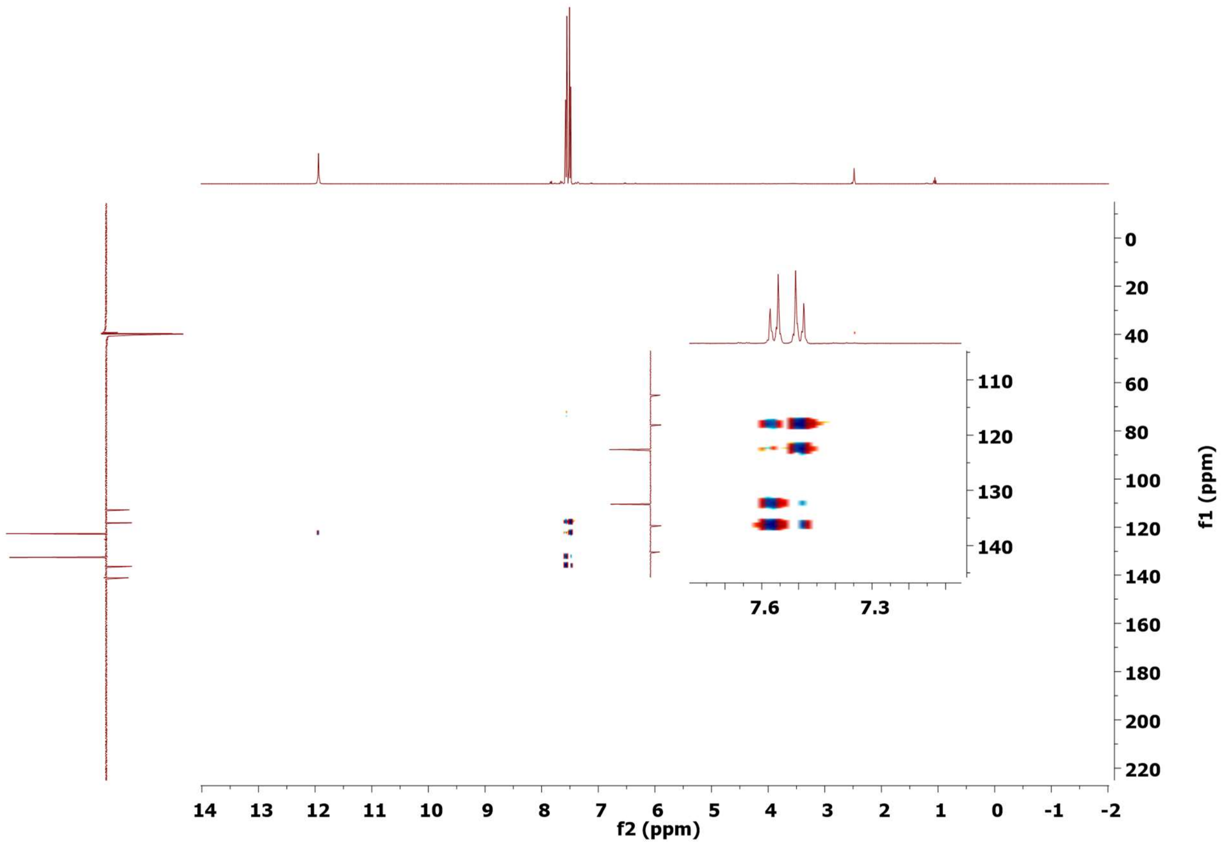

^{1}H NMR (DMSO-d6) spectrum of (2-methoxy-5-methylphenyl)carbamoyl cyanide (2h')

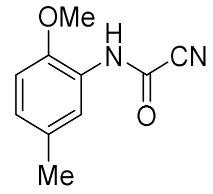

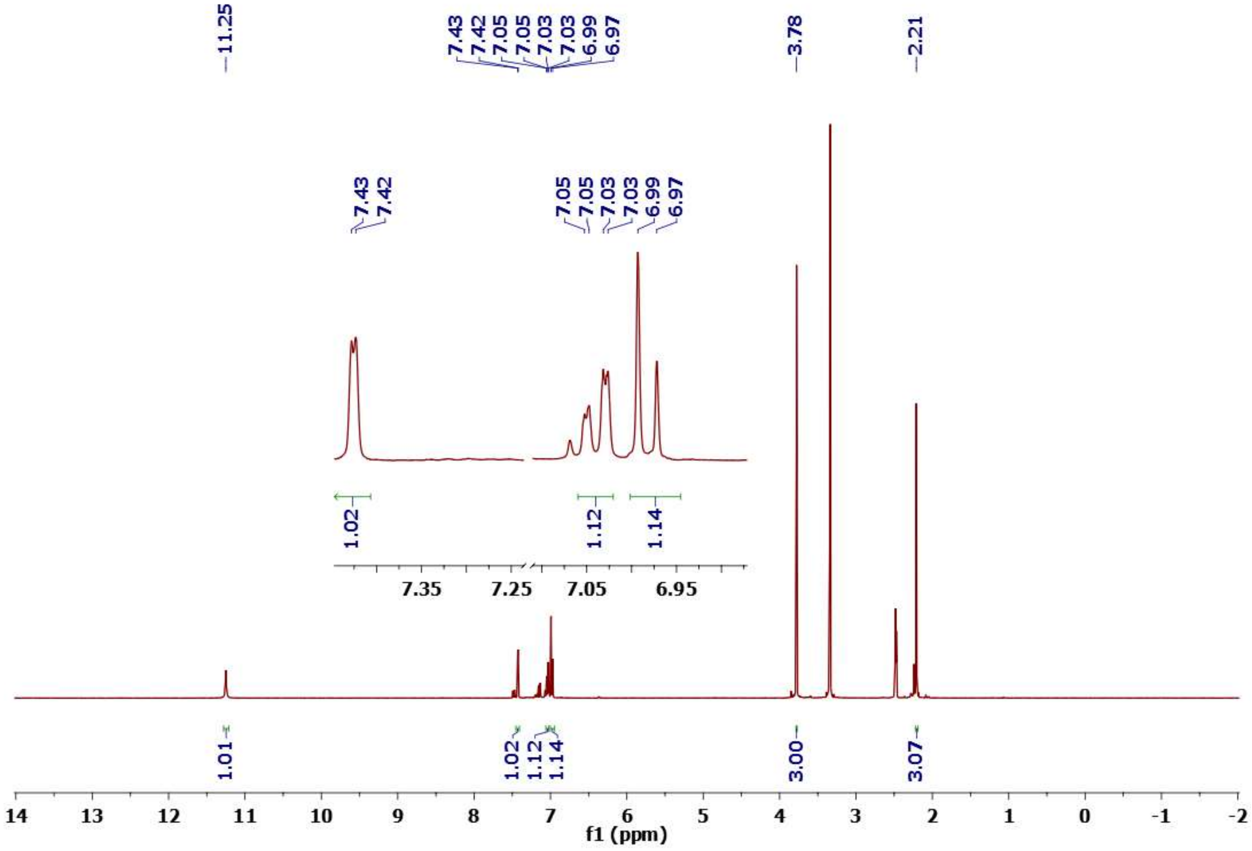

^{13}C NMR (DMSO-d6) spectrum of (2-methoxy-5-methylphenyl)carbamoyl cyanide (2h’)

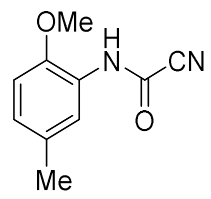

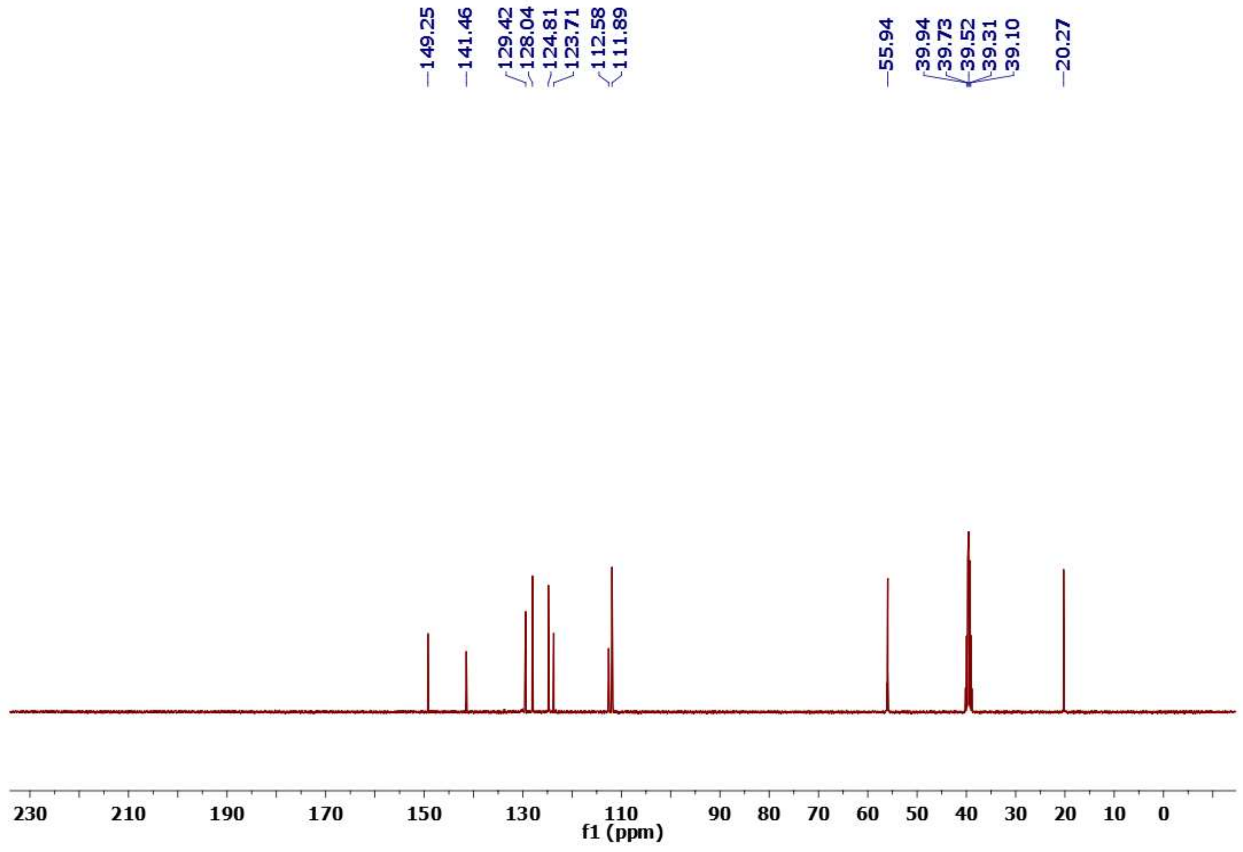

^{13}C CRAPT NMR (DMSO-d6) spectrum of (2-methoxy-5-methylphenyl)carbamoyl cyanide (2h')

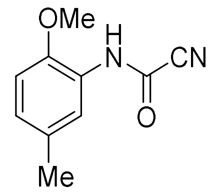

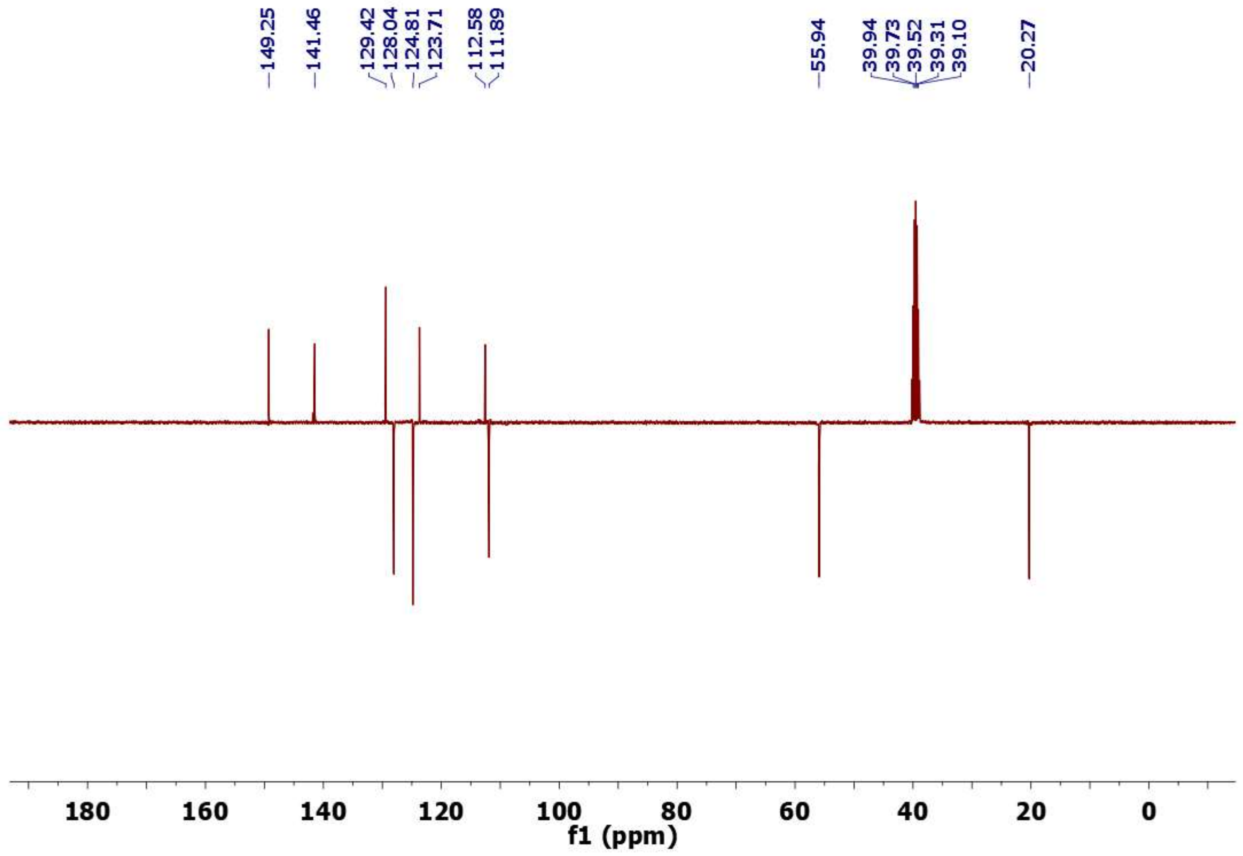

^{1}H-^{1}H-gDQCOSY NMR (DMSO-d6) spectrum of (2-methoxy-5-methylphenyl)carbamoyl cyanide (2h')

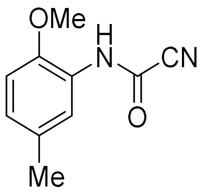

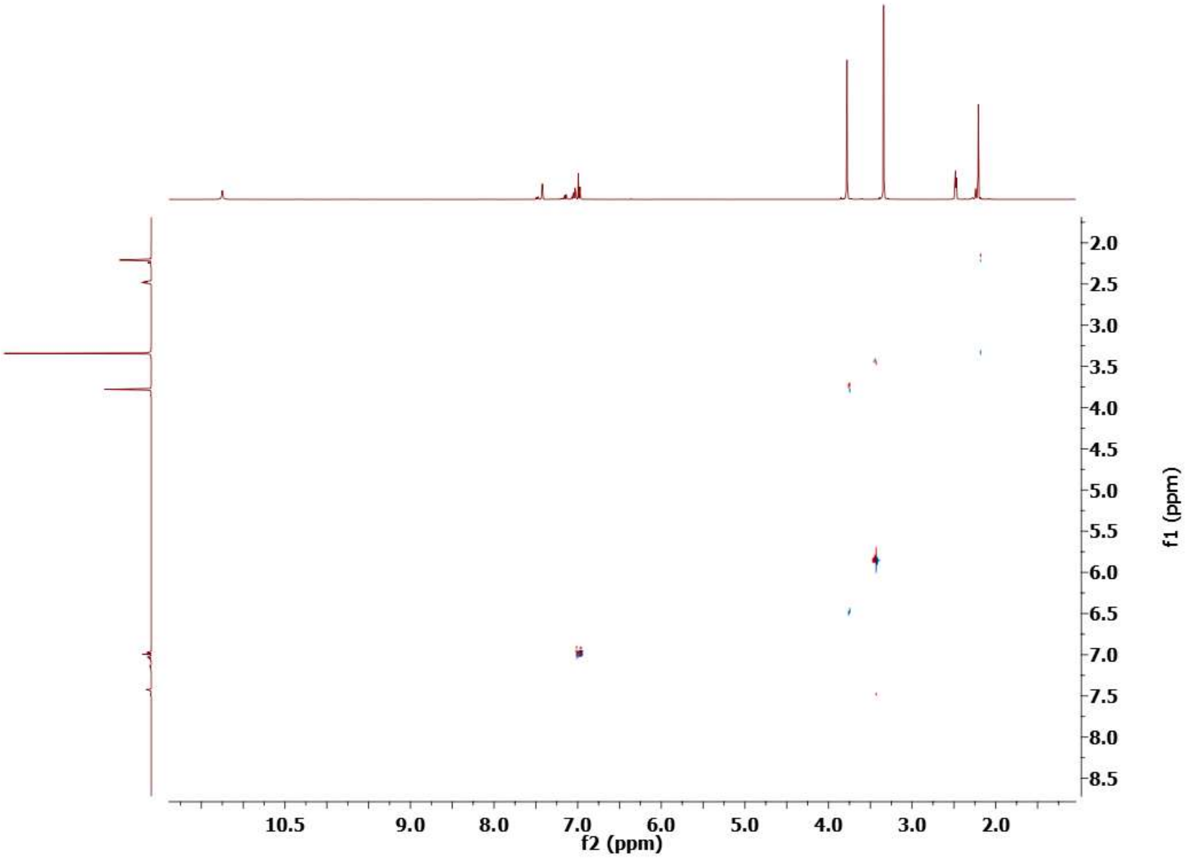

^{1}H-^{13}C-gHSQC NMR (DMSO-d6) spectrum of (2-methoxy-5-methylphenyl)carbamoyl cyanide (2h')

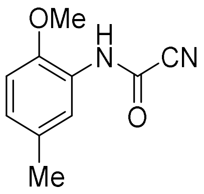

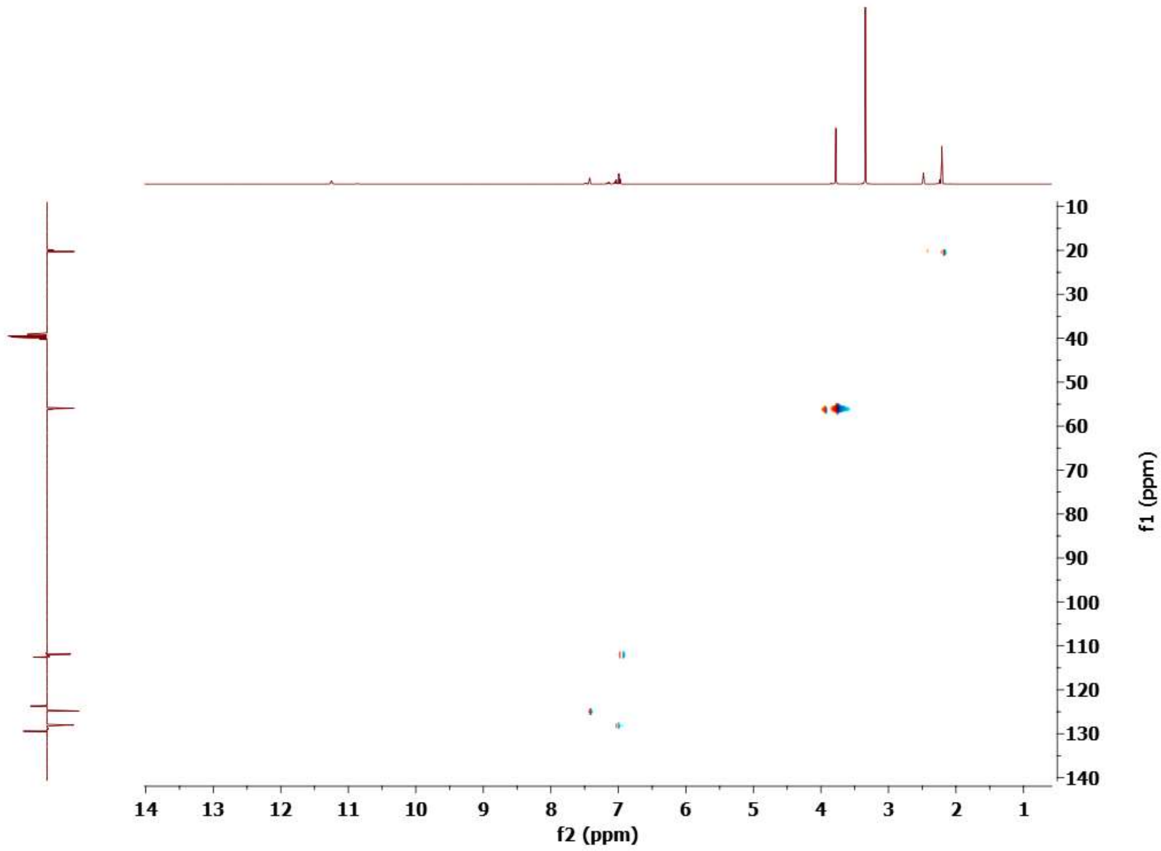

^{1}H NMR (DMSO-d6) spectrum of (3,5-dichlorophenyl)carbamoyl cyanide (2i')

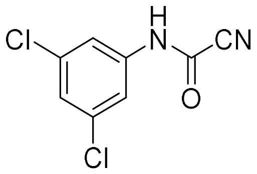

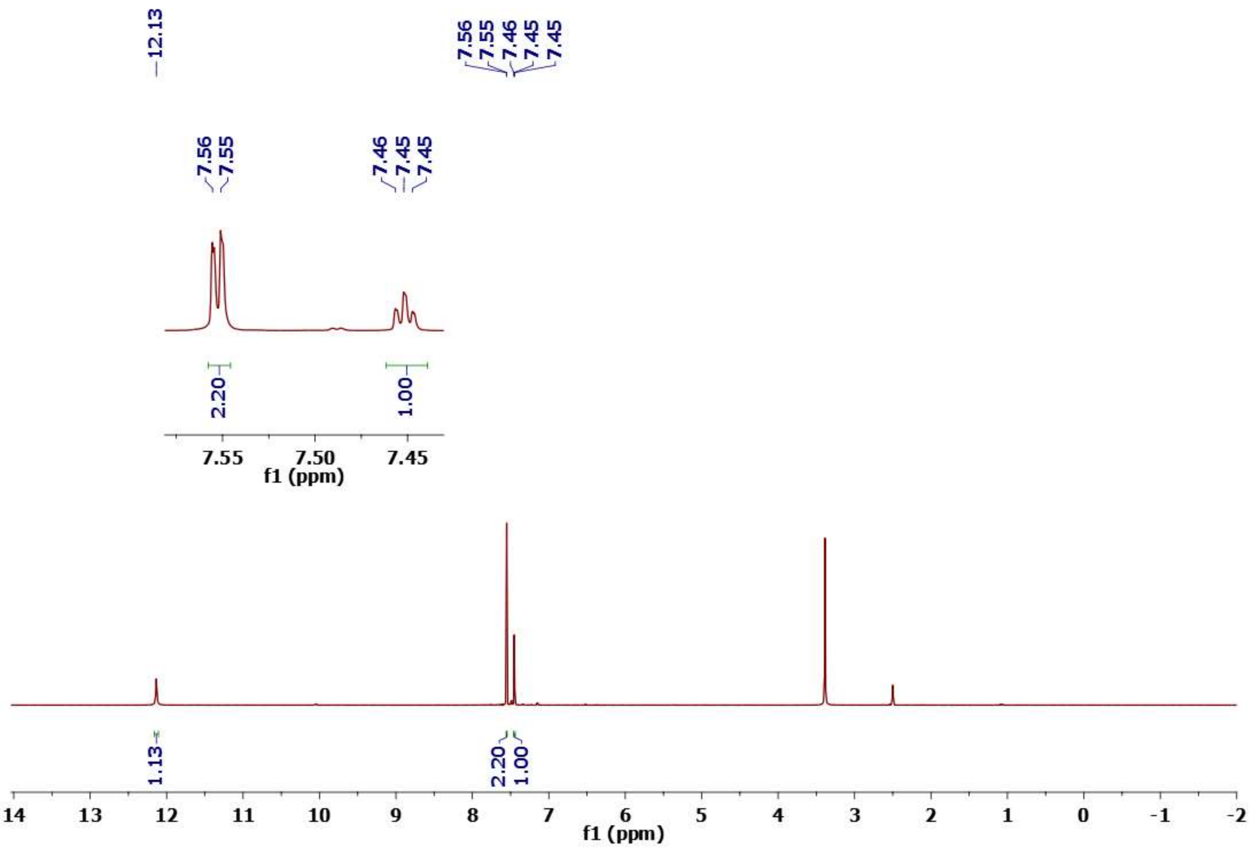

^{13}C NMR (DMSO-d6) spectrum of (3,5-dichlorophenyl)carbamoyl cyanide (2i')

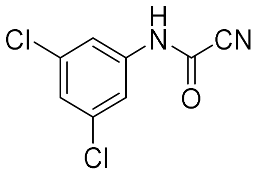

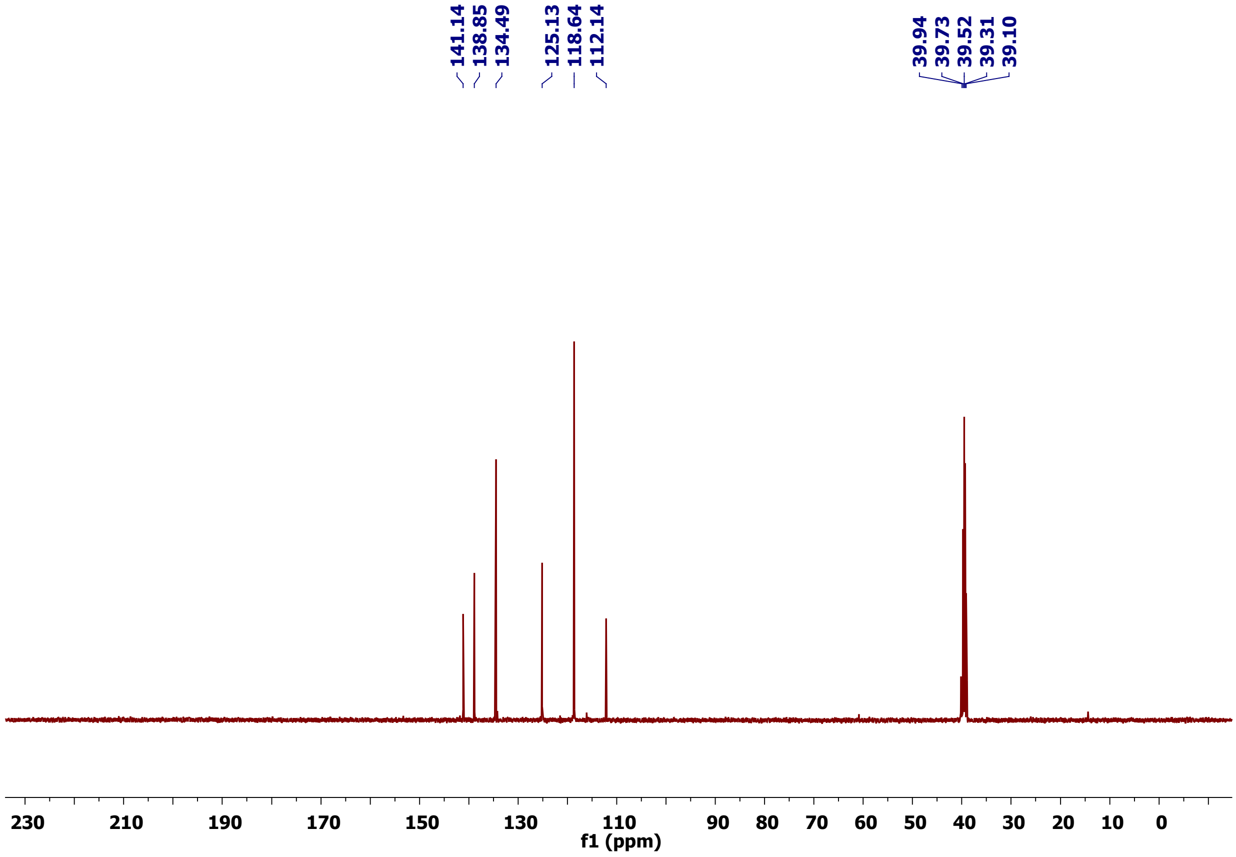

^{13}C CRAPT NMR (DMSO-d6) spectrum of (3,5-dichlorophenyl)carbamoyl cyanide (2i’)

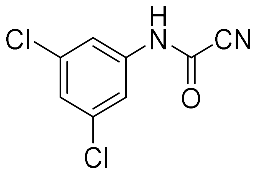

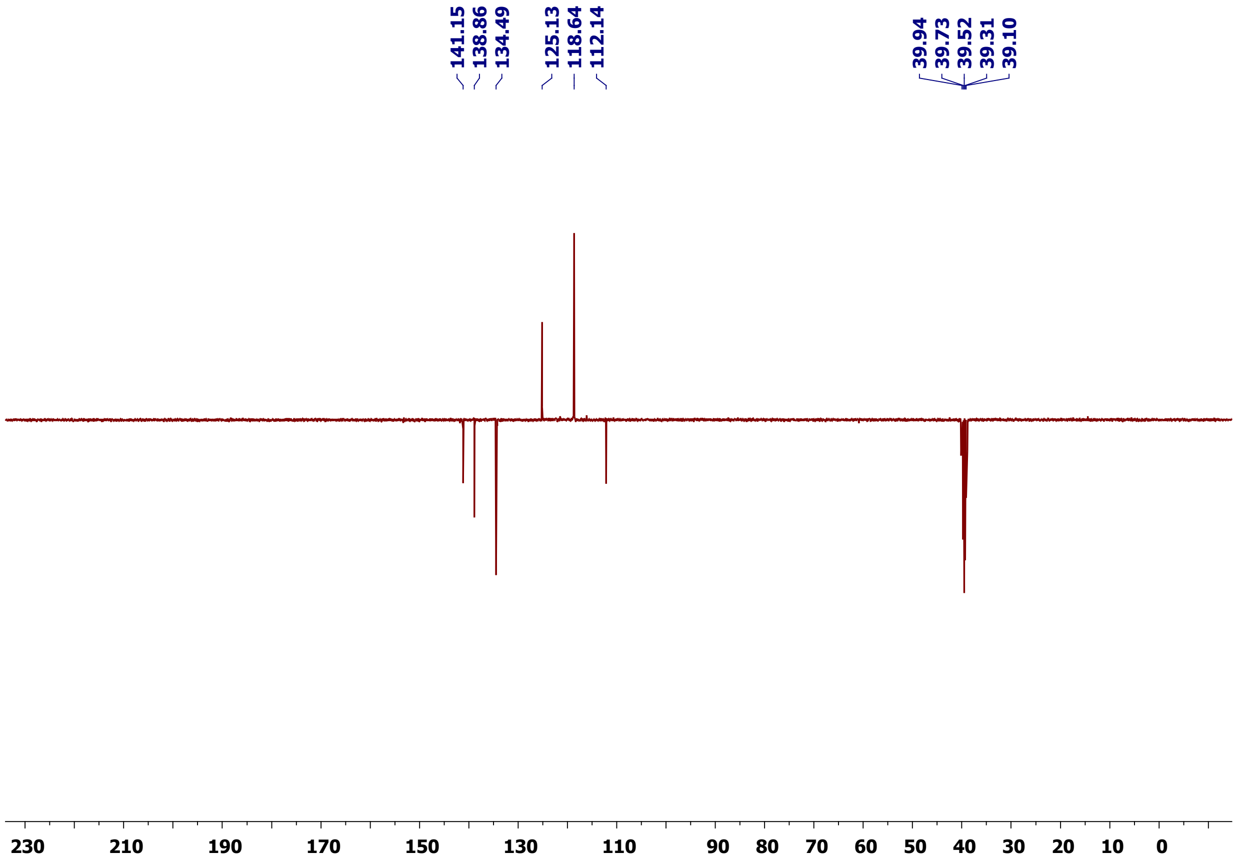

^1H-^1H-gDQCOSY NMR (DMSO-d6) spectrum of (3,5-dichlorophenyl)carbamoyl cyanide (2i')

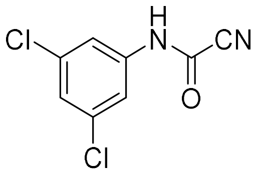

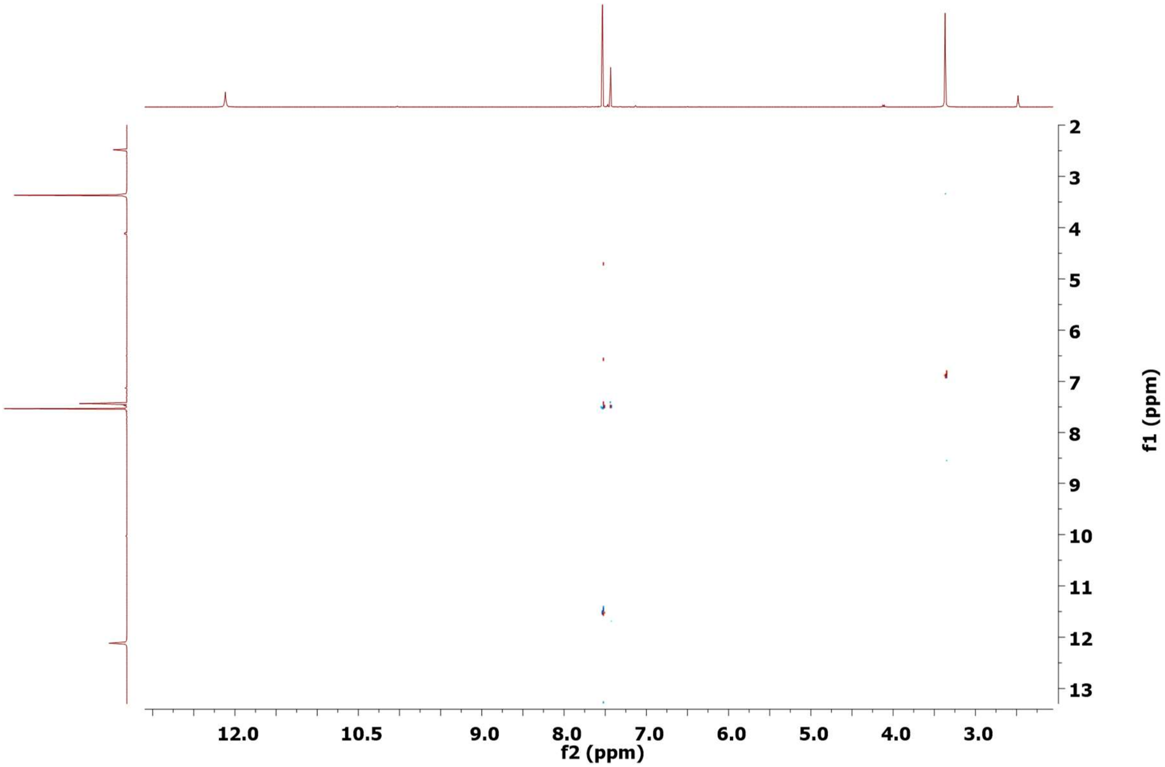

^{1}H-^{13}C-gHSQC NMR (DMSO-d6) spectrum of (3,5-dichlorophenyl)carbamoyl cyanide (2i')

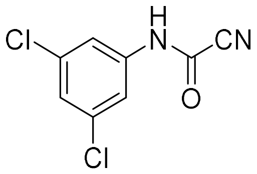

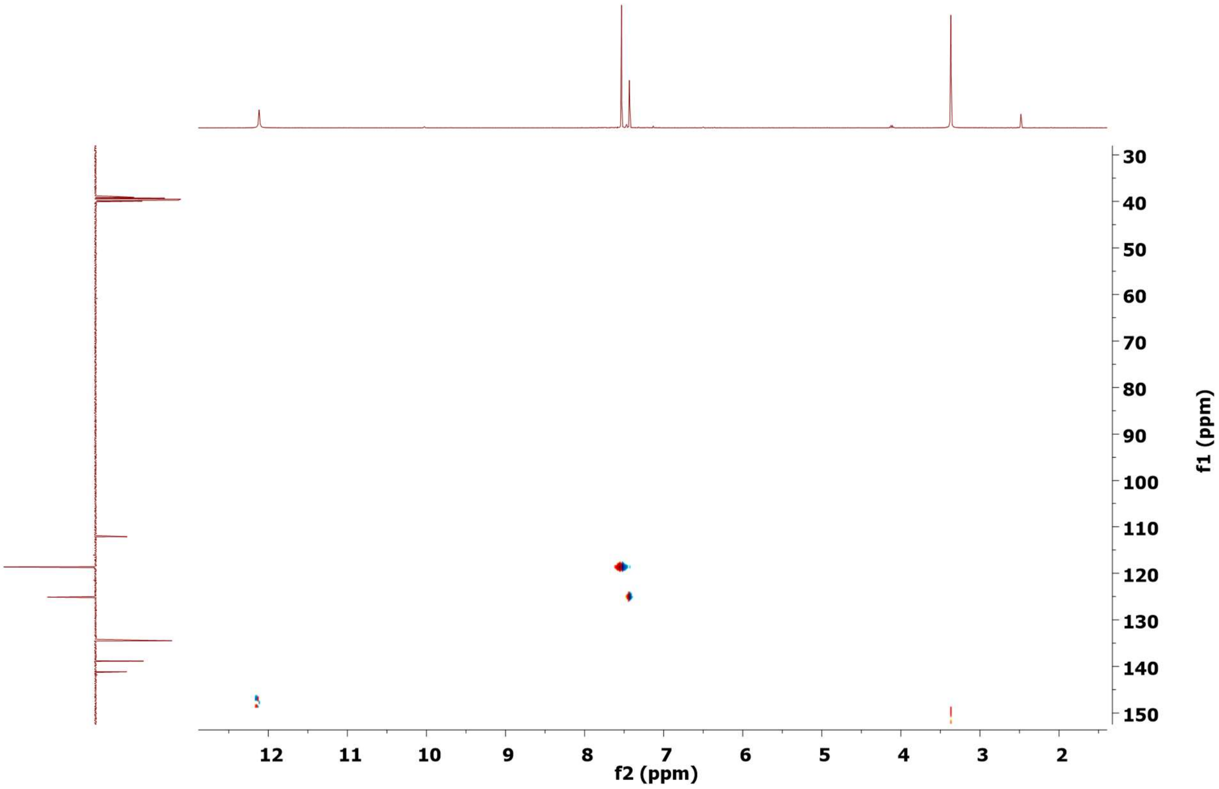

^{1}H-^{13}C-gHMBC NMR (DMSO-d6) spectrum of (3,5-dichlorophenyl)carbamoyl cyanide (2i')

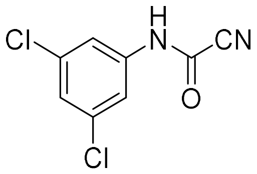

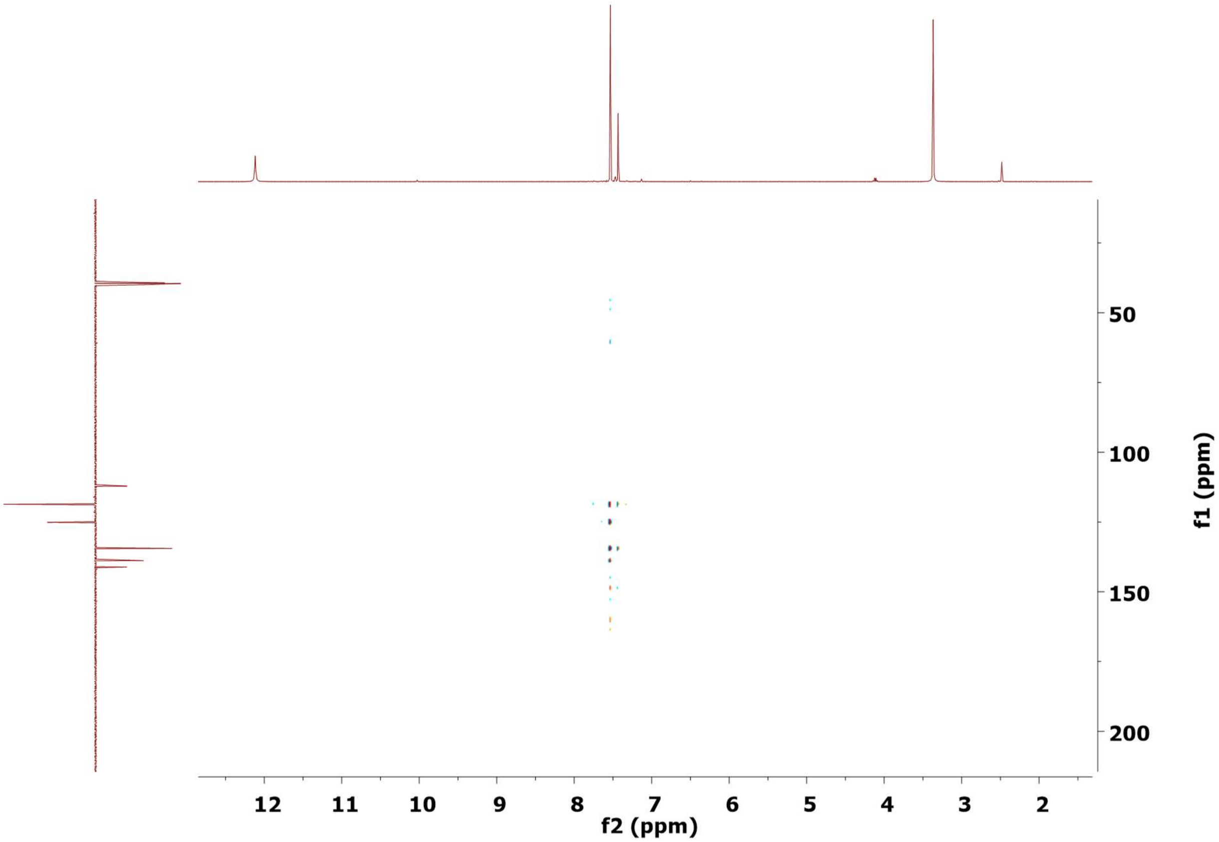

^{1}H NMR (DMSO-d6) spectrum of (3,4-dichlorophenyl)carbamoyl cyanide (2j’)

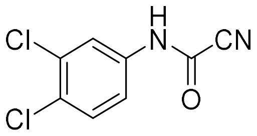

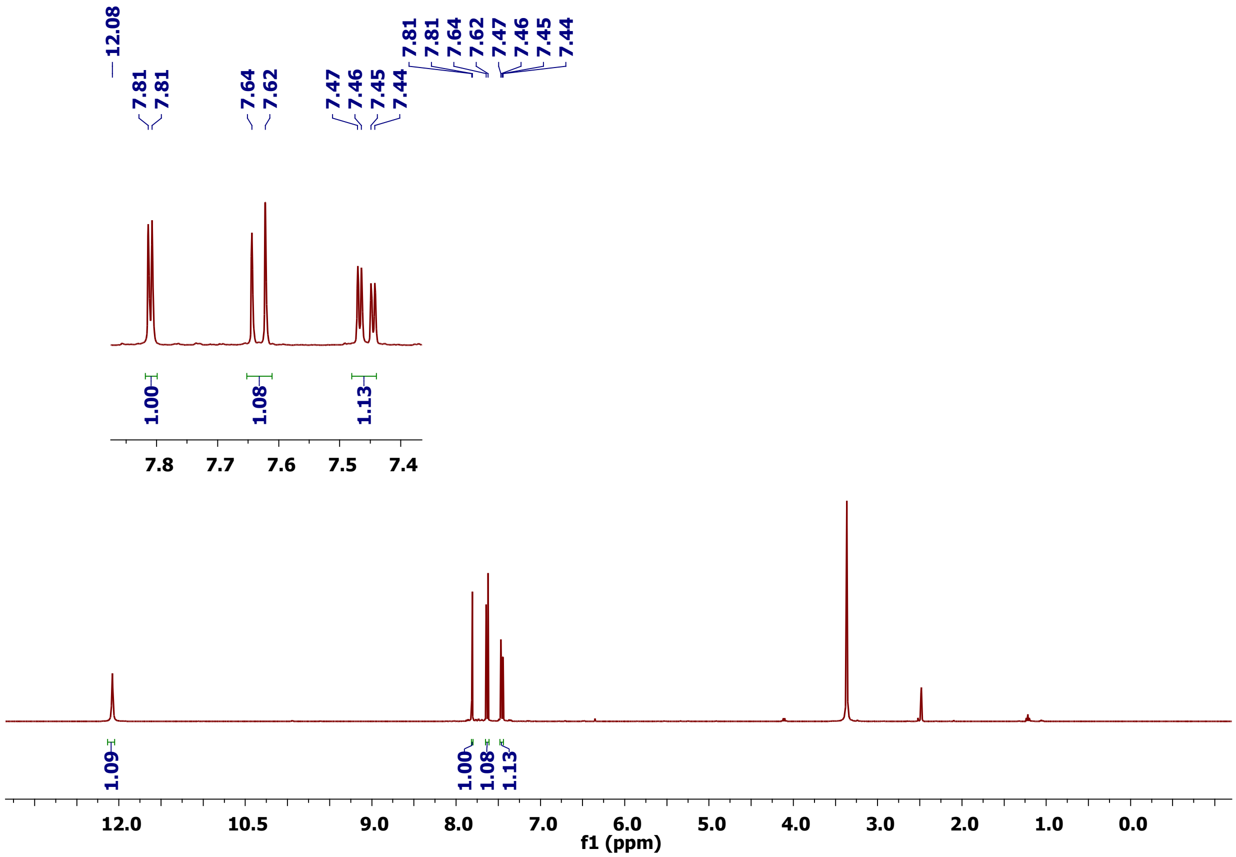

^{13}C NMR (DMSO-d6) spectrum of (3,4-dichlorophenyl)carbamoyl cyanide (2j')

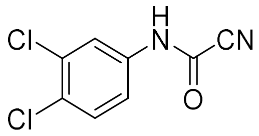

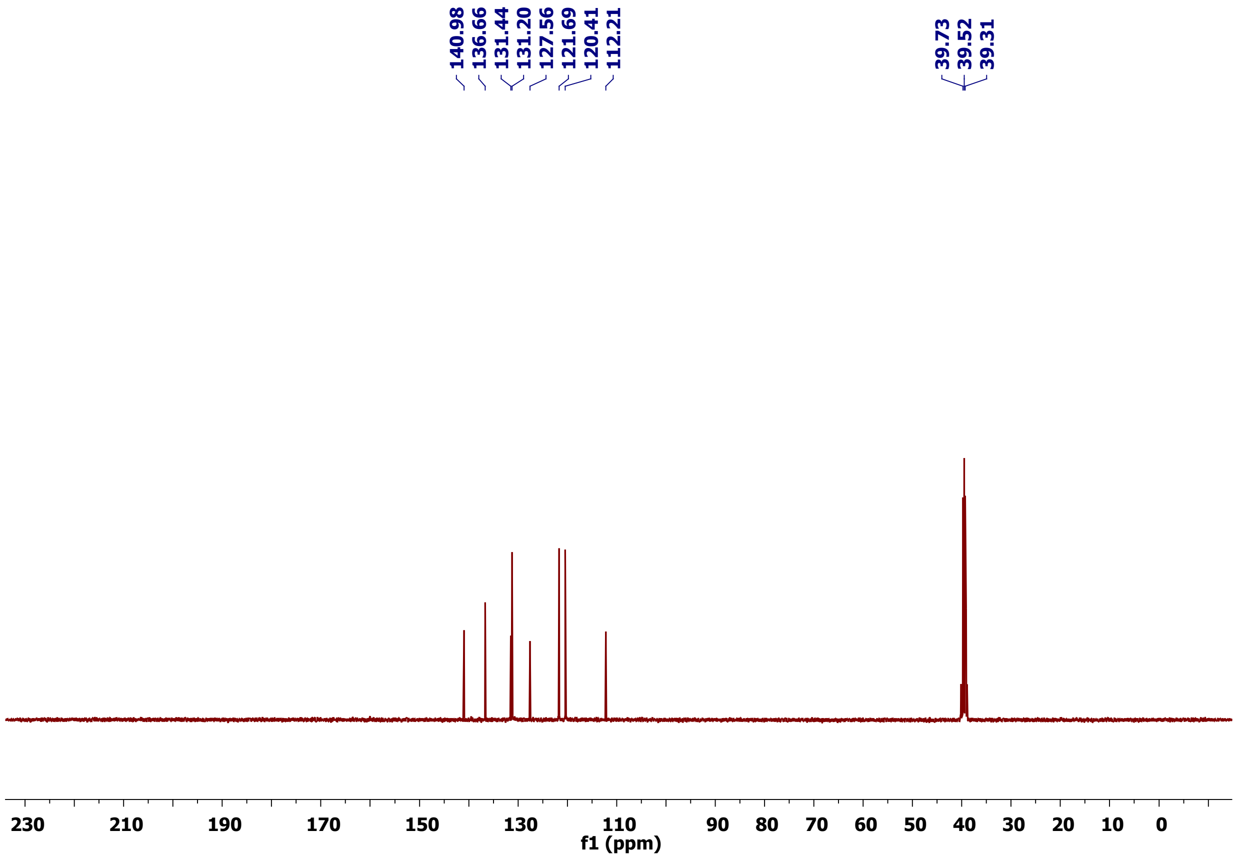

^{13}C CRAPT NMR (DMSO-d6) spectrum of (3,4-dichlorophenyl)carbamoyl cyanide (2j')

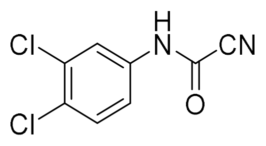

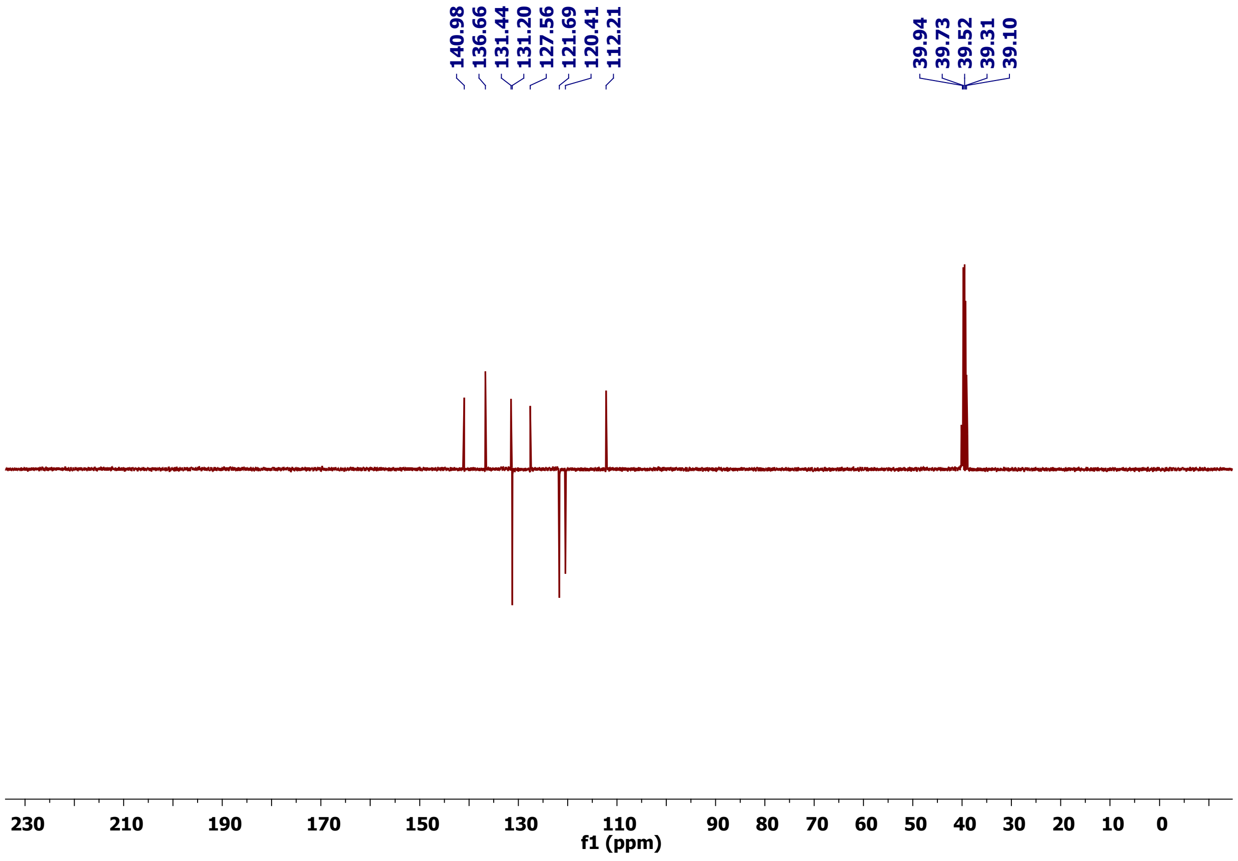

^{1}H-^{1}H-gDQCOSY NMR (DMSO-d6) spectrum of (3,4-dichlorophenyl)carbamoyl cyanide (2j')

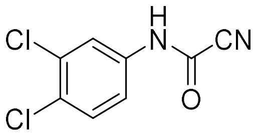

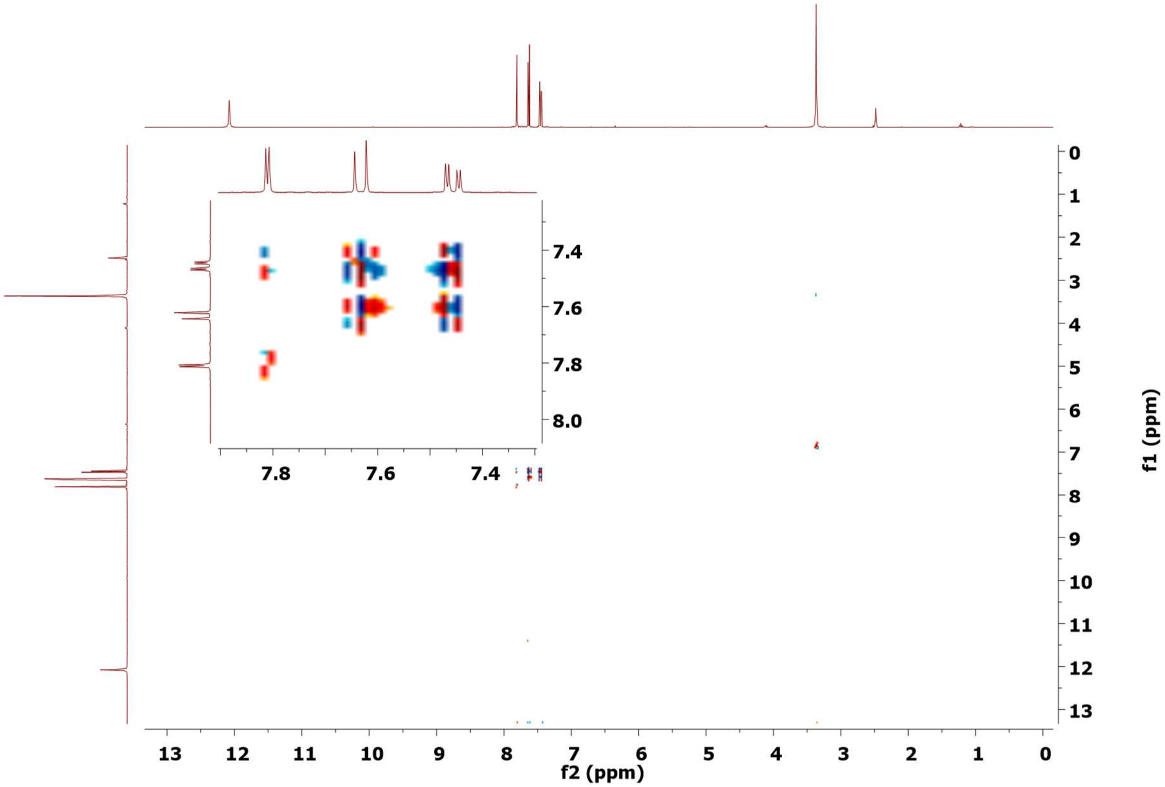

^1H-^{13}C-gHSQC NMR (DMSO-d6) spectrum of (3,4-dichlorophenyl)carbamoyl cyanide (2j')

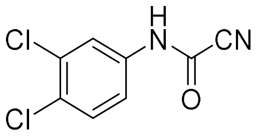

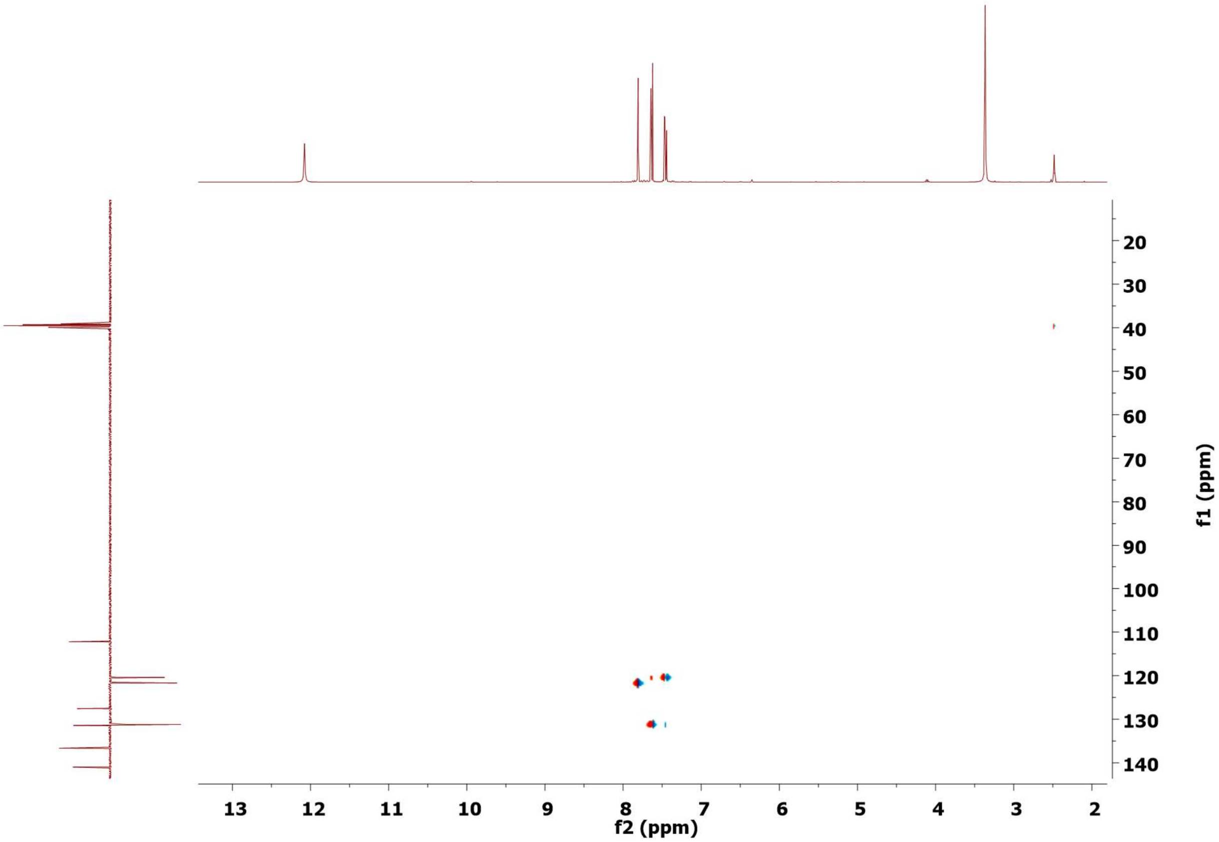

^{1}H-^{13}C-gHMBC NMR (DMSO-d6) spectrum of (3,4-dichlorophenyl)carbamoyl cyanide (2j')

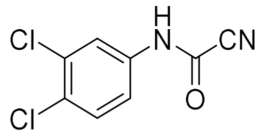

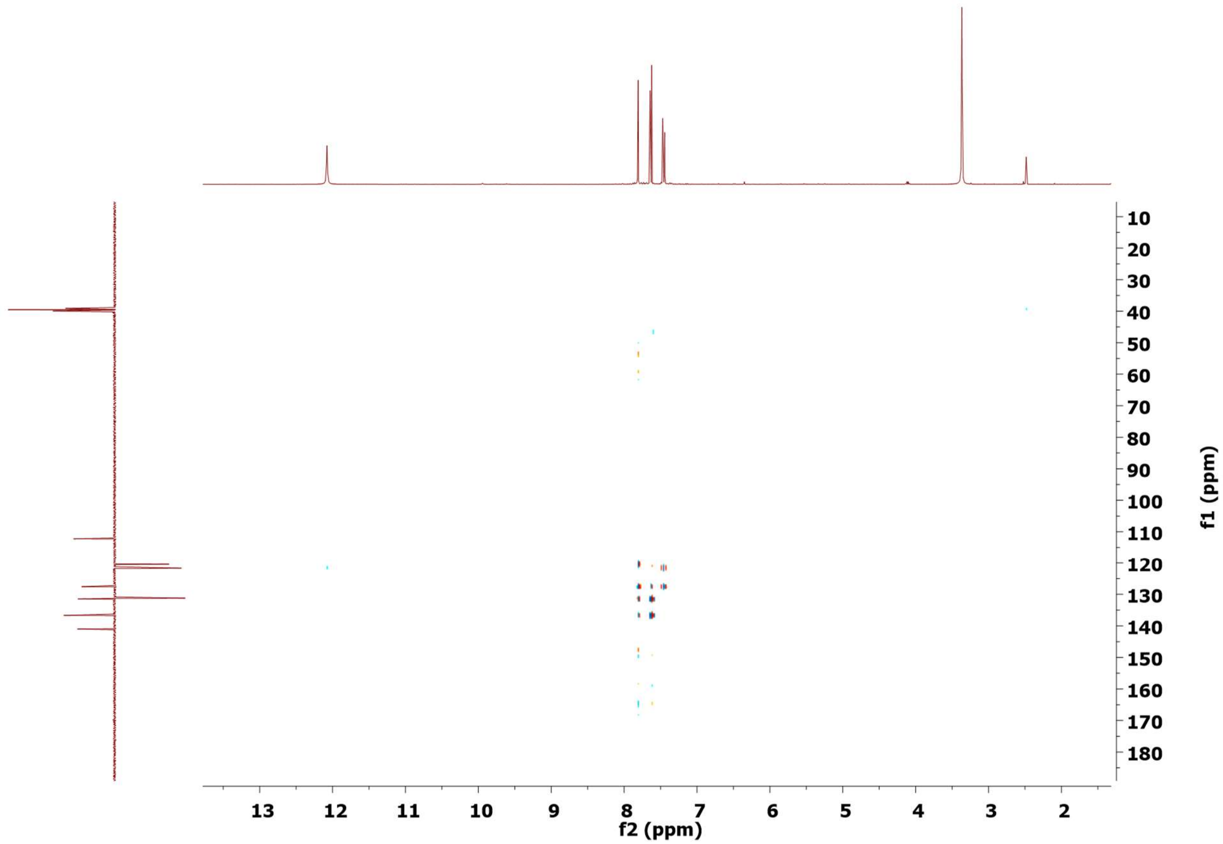

^{1}H NMR (DMSO-d6) spectrum of (2,4-difluorophenyl)carbamoyl cyanide (2k')

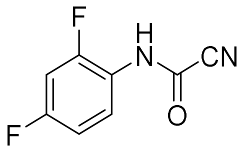

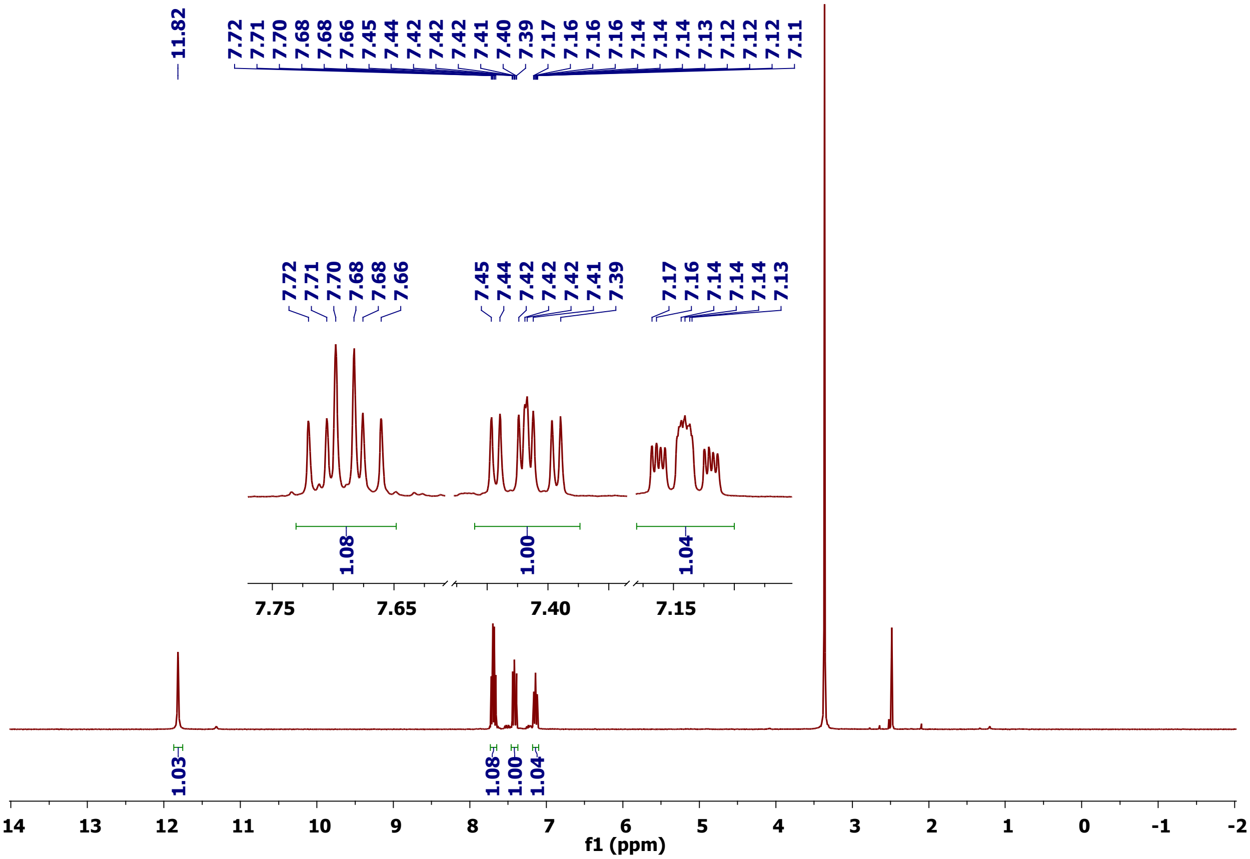

^{13}C NMR (DMSO-d6) spectrum of (2,4-difluorophenyl)carbamoyl cyanide (2k')

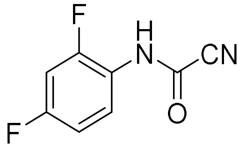

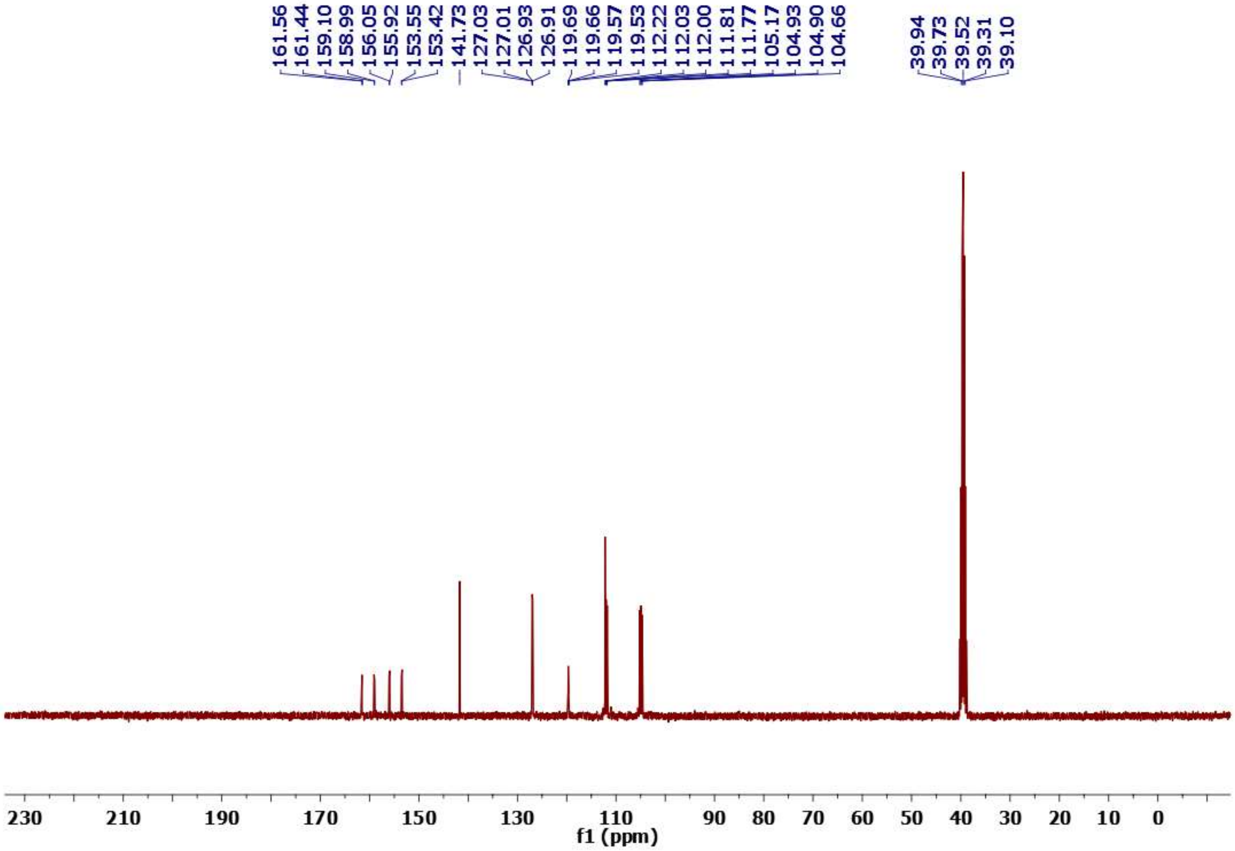

^{13}C CRAPT NMR (DMSO-d6) spectrum of (2,4-difluorophenyl)carbamoyl cyanide (2k')

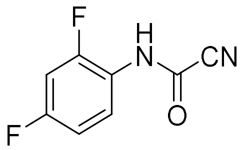

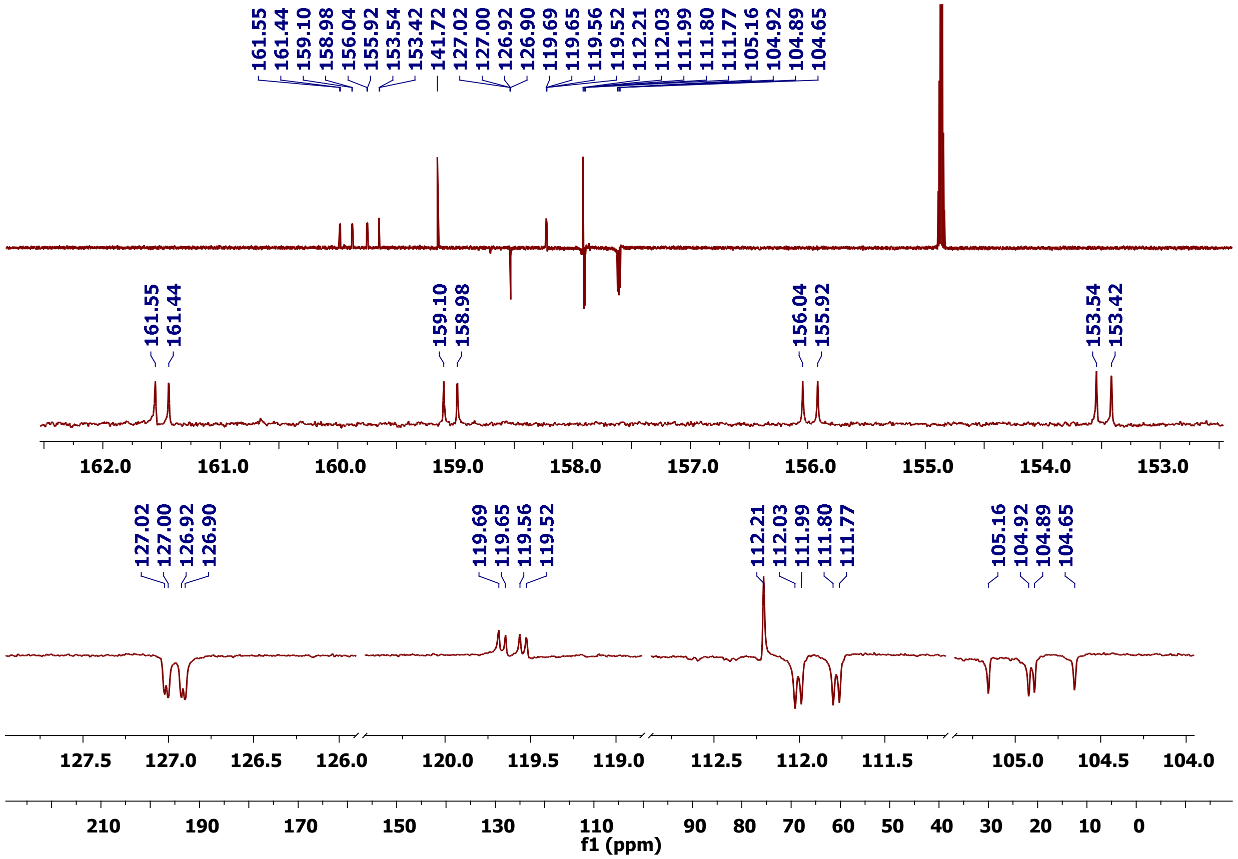

^{1}H-^{1}H-gDQCOSY NMR (DMSO-d6) spectrum of (2,4-difluorophenyl)carbamoyl cyanide (2k')

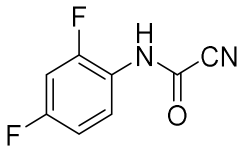

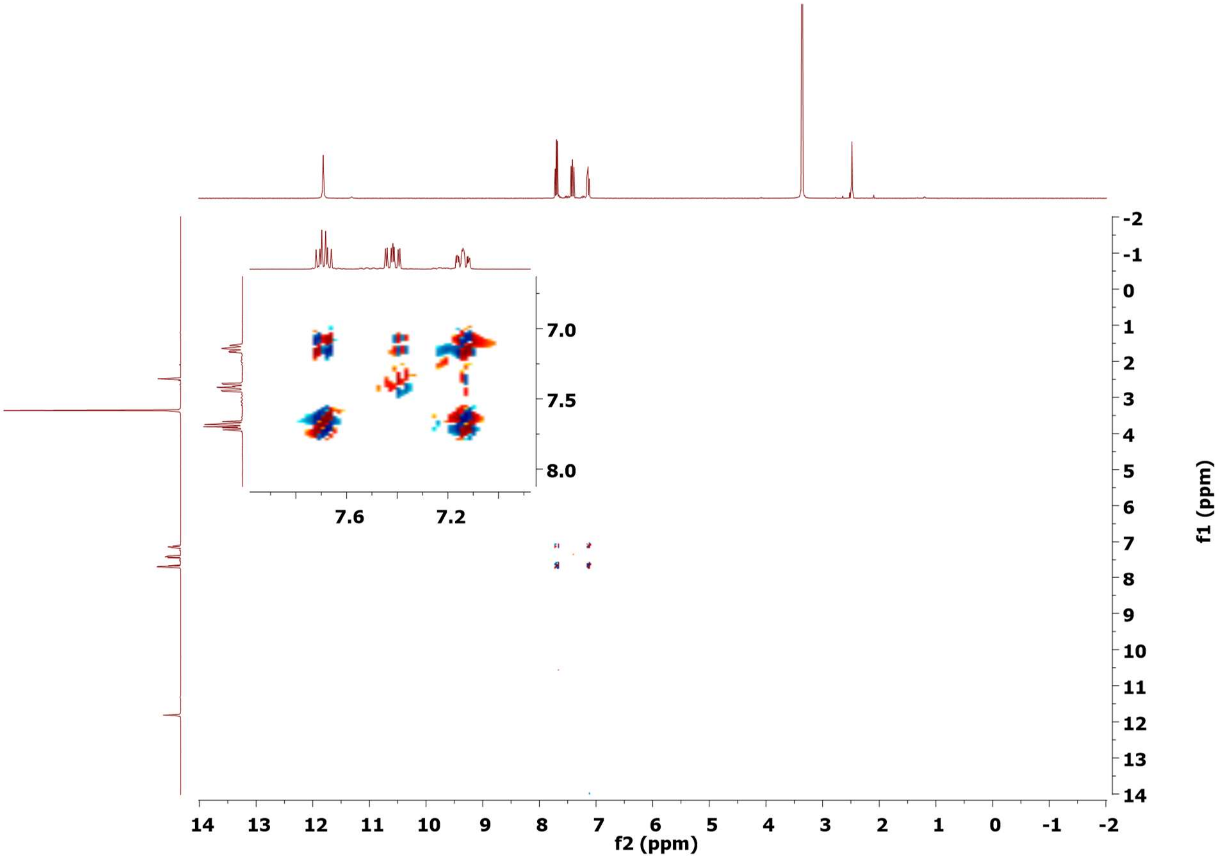

^{1}H-^{13}C-gHSQC NMR (DMSO-d6) spectrum of (2,4-difluorophenyl)carbamoyl cyanide (2k')

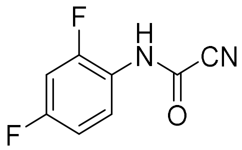

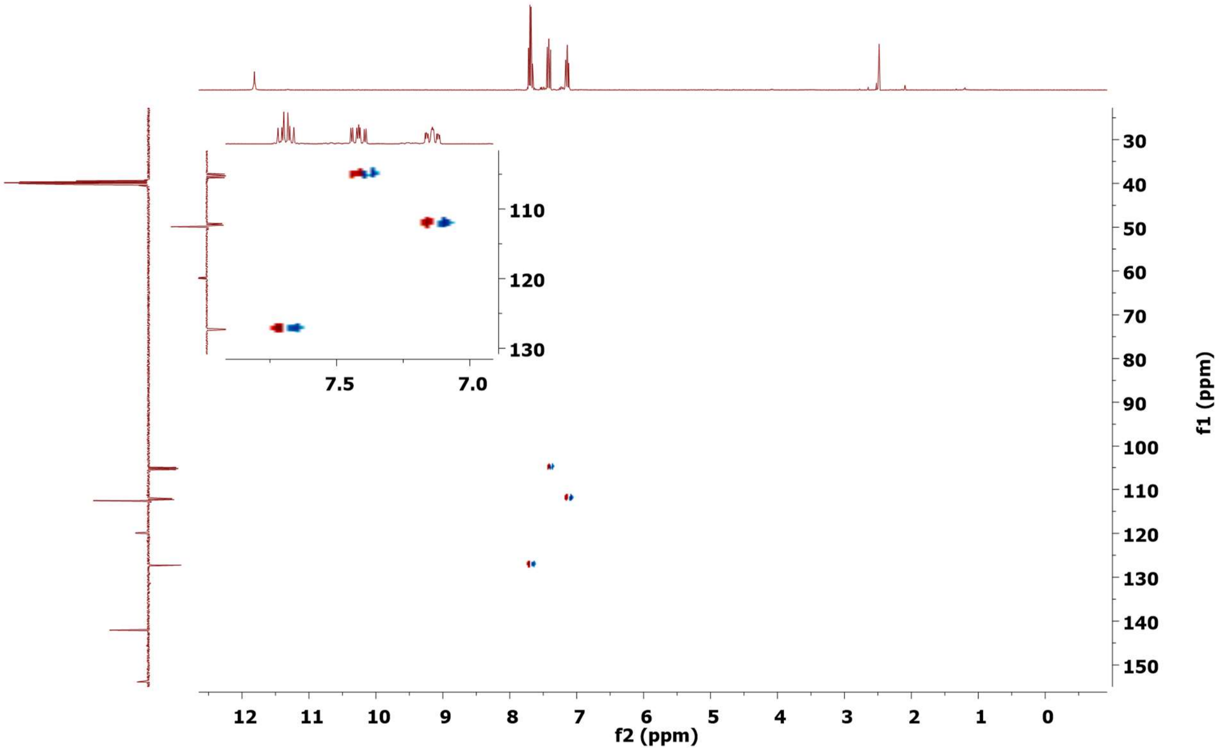

^{1}H-^{13}C-gHMBC NMR (DMSO-d6) spectrum of (2,4-difluorophenyl)carbamoyl cyanide (2k')

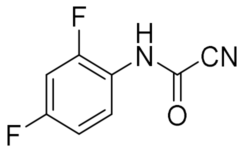

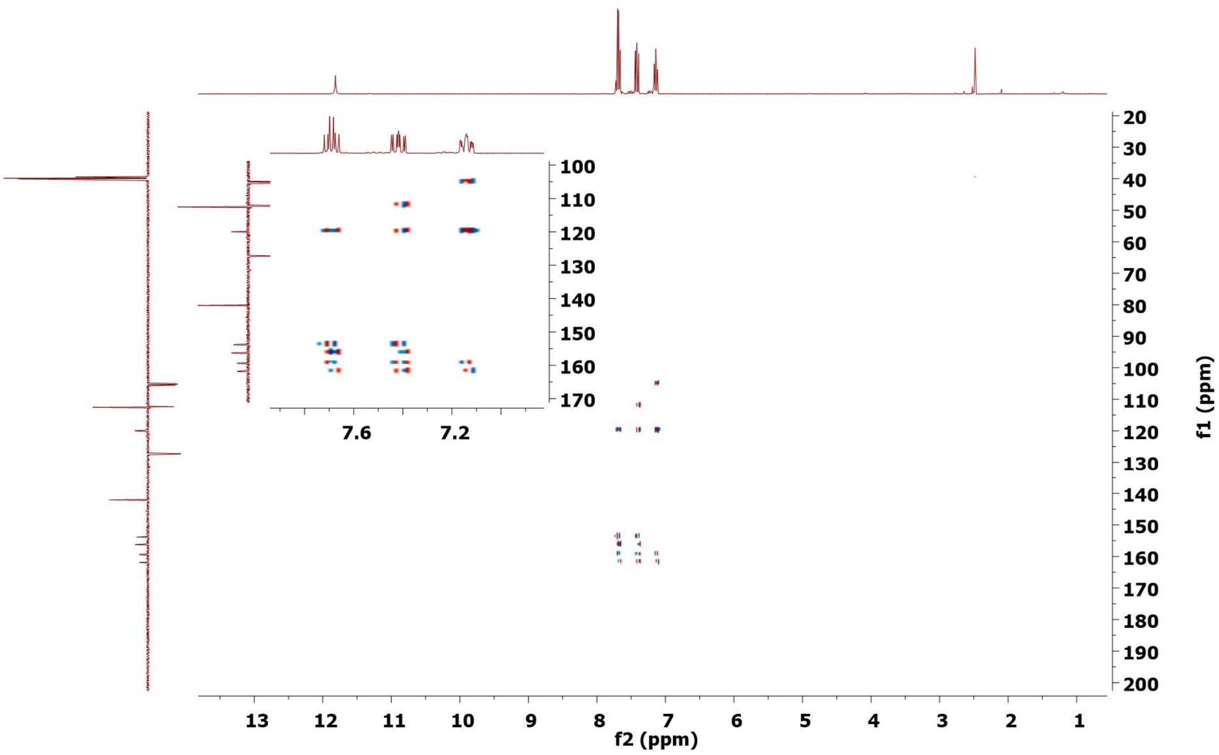

^{1}H NMR (DMSO-d6) spectrum of naphtho[1,2-d]thiazole-2-carbonitrile (3a)

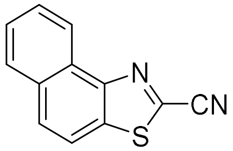

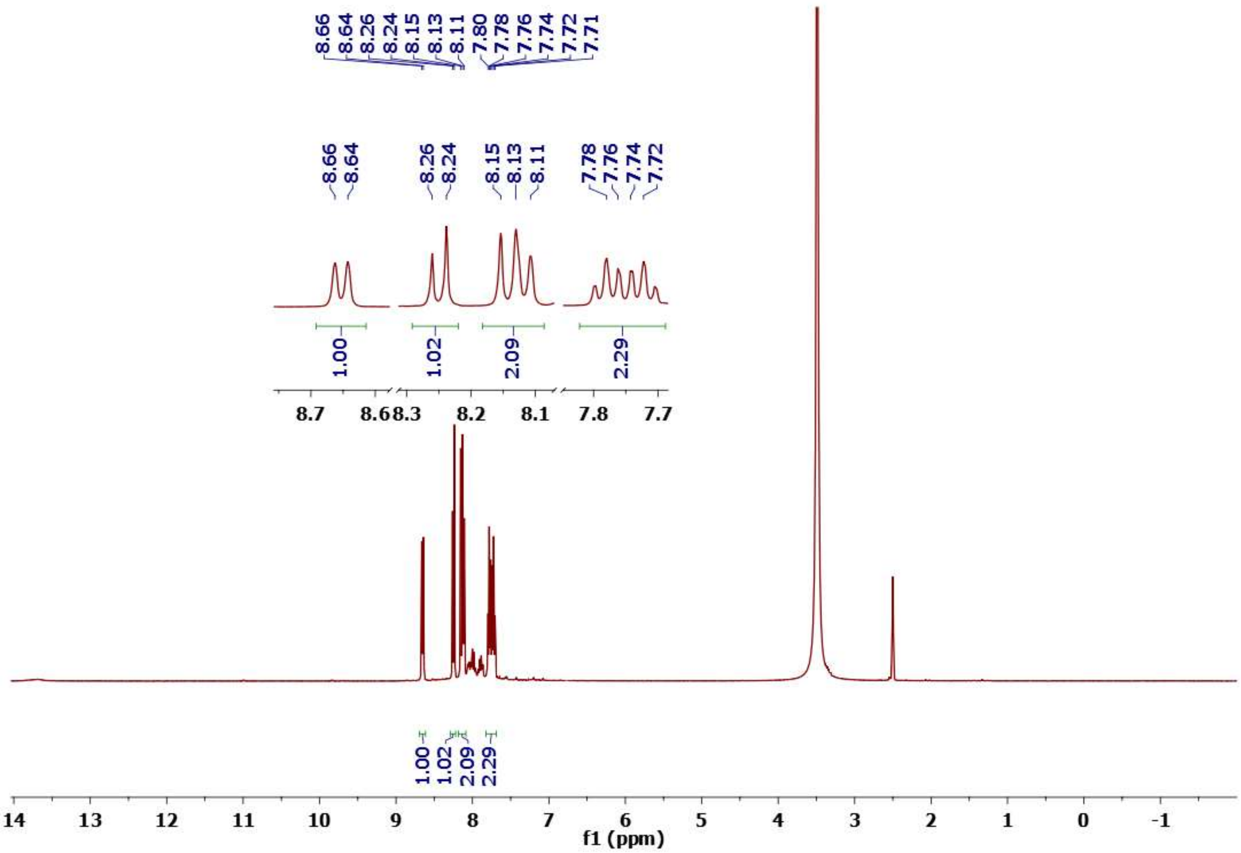

^{13}C NMR (DMSO-d6) spectrum of naphtho[1,2-d]thiazole-2-carbonitrile (3a)

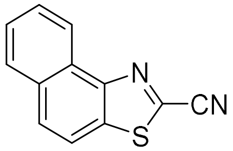

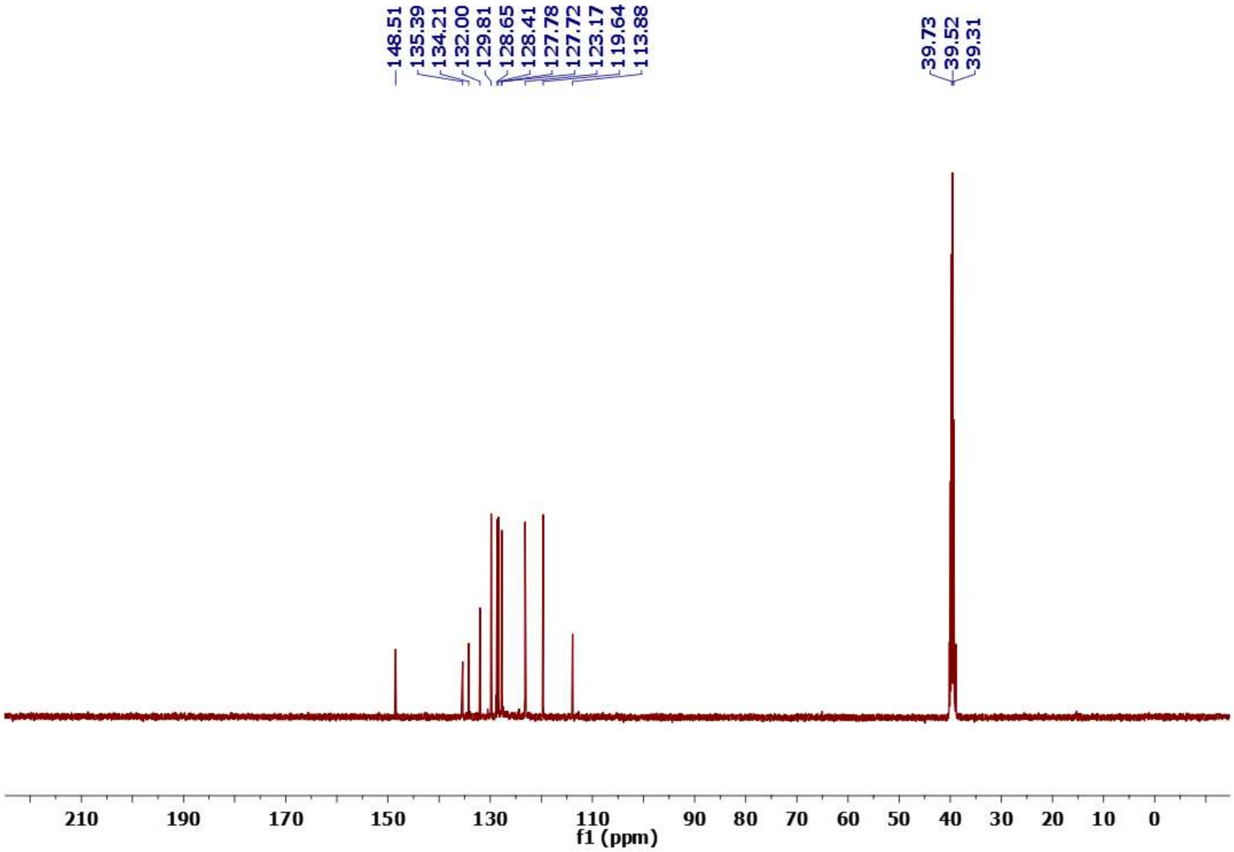

^{13}C CRAPT NMR (DMSO-d6) spectrum of naphtho[1,2-d]thiazole-2-carbonitrile (3a)

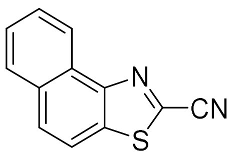

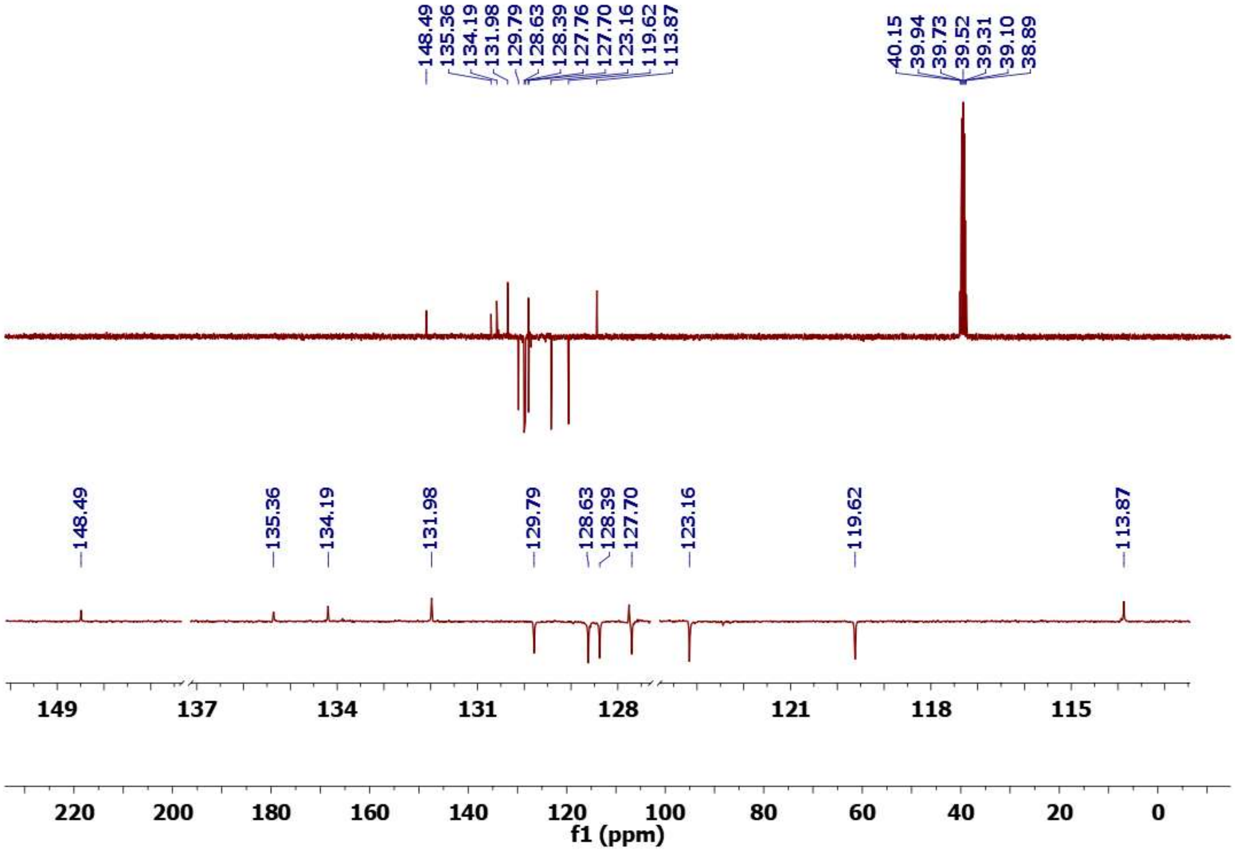

^{1}H-^{1}H-gDQCOSY NMR (DMSO-d6) spectrum of naphtho[1,2-d]thiazole-2-carbonitrile (3a)

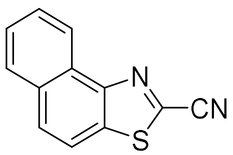

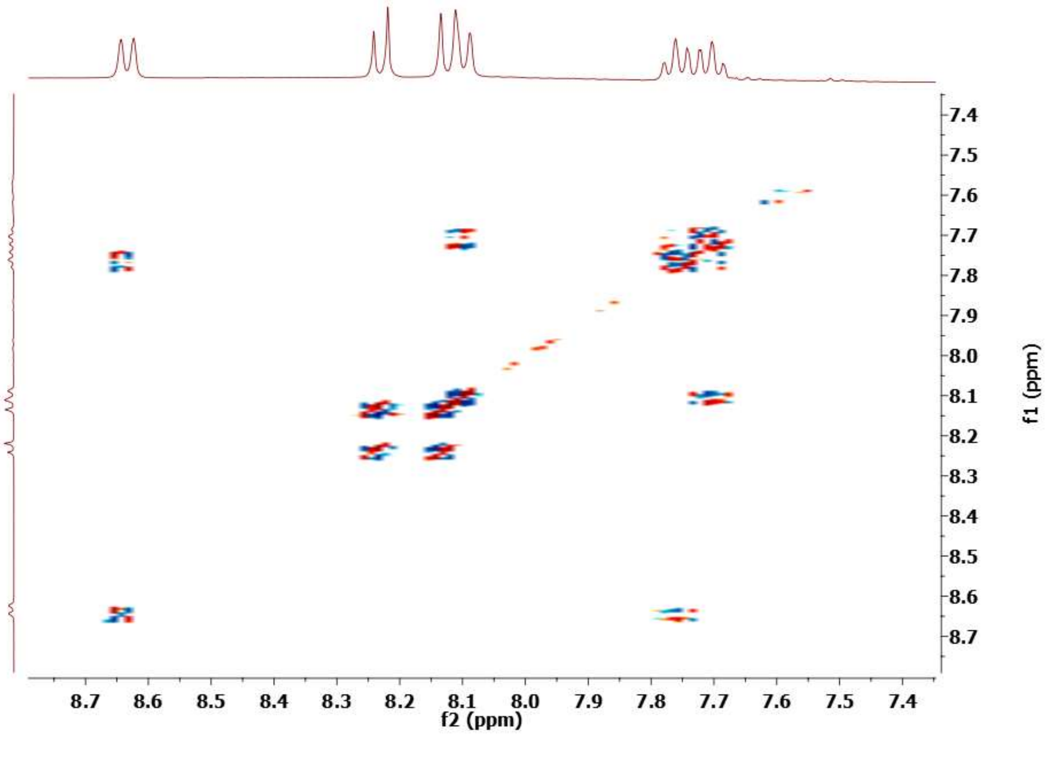

^{1}H-^{13}C-gHSQC NMR (DMSO-d6) spectrum of naphtho[1,2-d]thiazole-2-carbonitrile (3a)

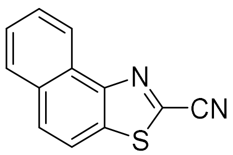

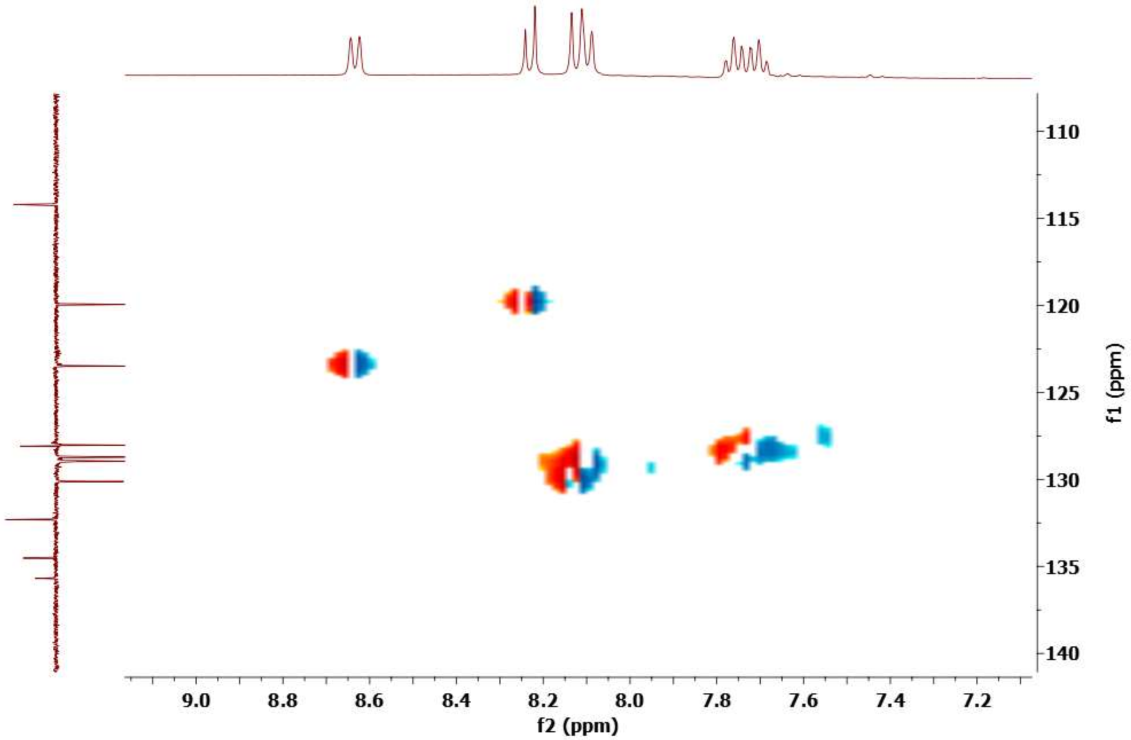

^{1}H-^{13}C-gHMBC NMR (DMSO-d6) spectrum of naphtho[1,2-d]thiazole-2-carbonitrile (3a)

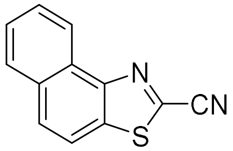

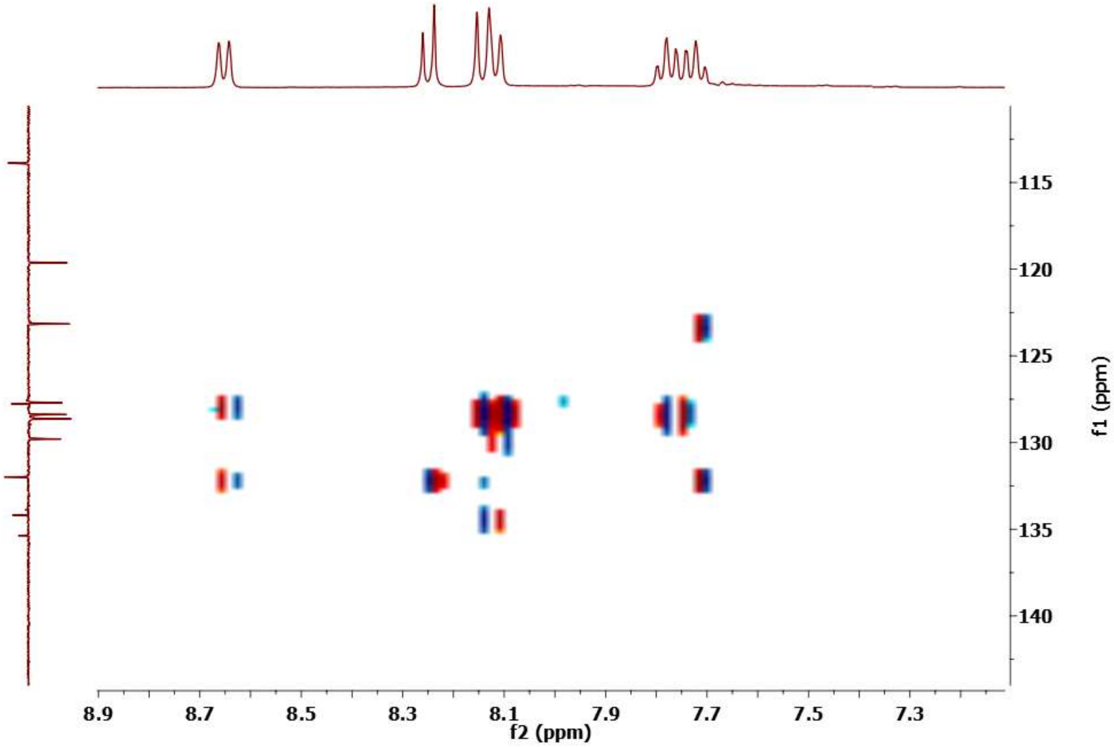

^{1}H NMR (DMSO-d6) spectrum of 5-methoxybenzo[d]thiazole-2-carbonitrile (3b)

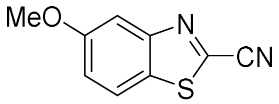

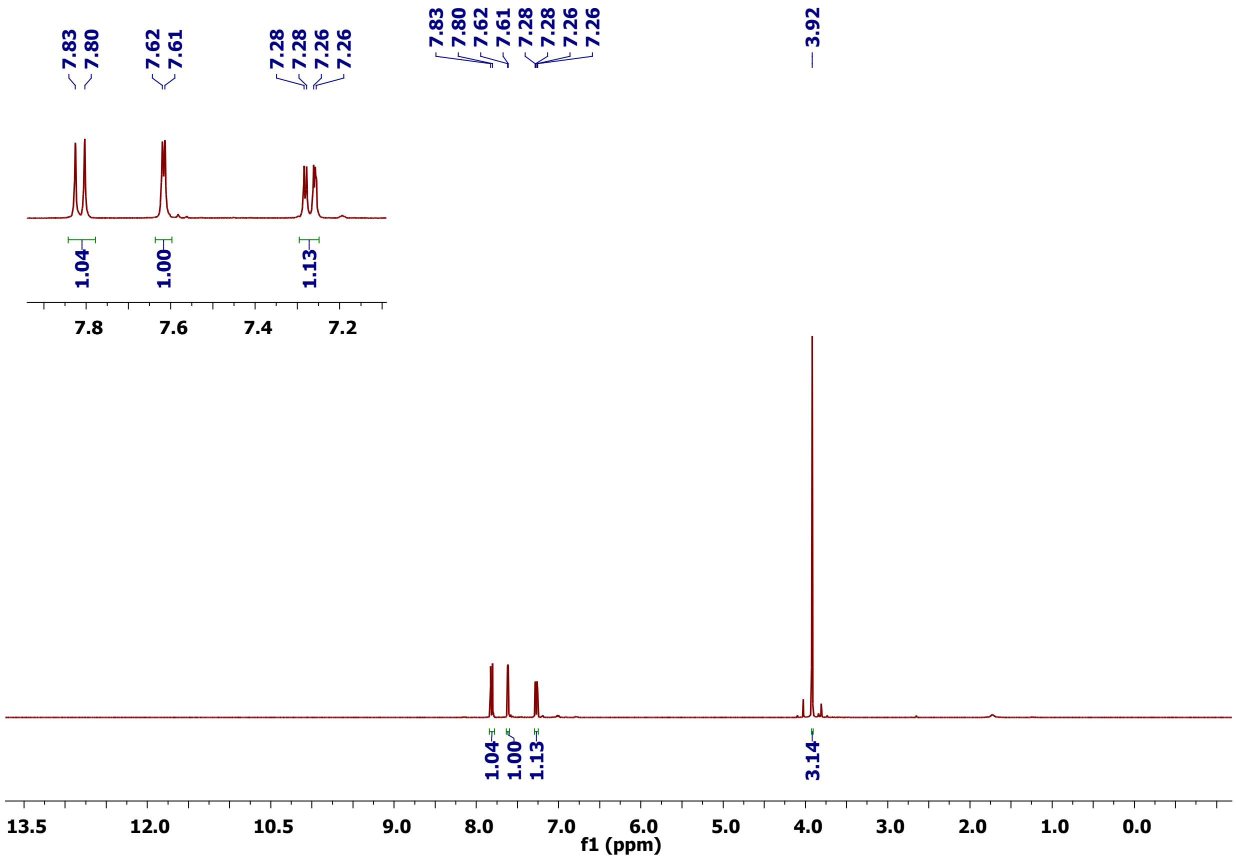

^{13}C NMR (DMSO-d6) spectrum of 5-methoxybenzo[d]thiazole-2-carbonitrile (3b)

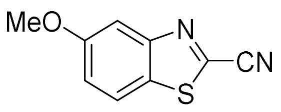

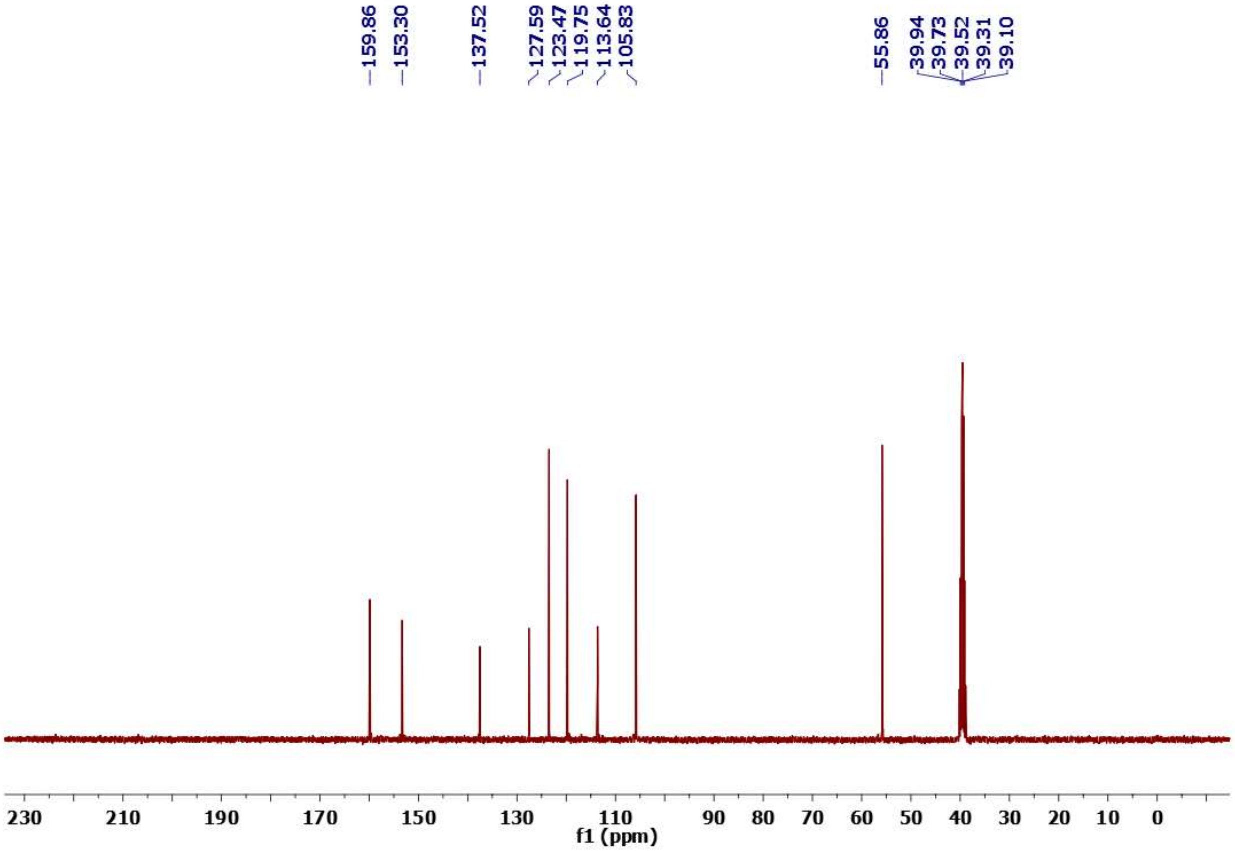

^{13}C CRAPT NMR (DMSO-d6) spectrum of 5-methoxybenzo[d]thiazole-2-carbonitrile (3b)

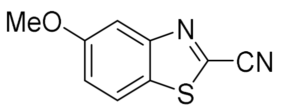

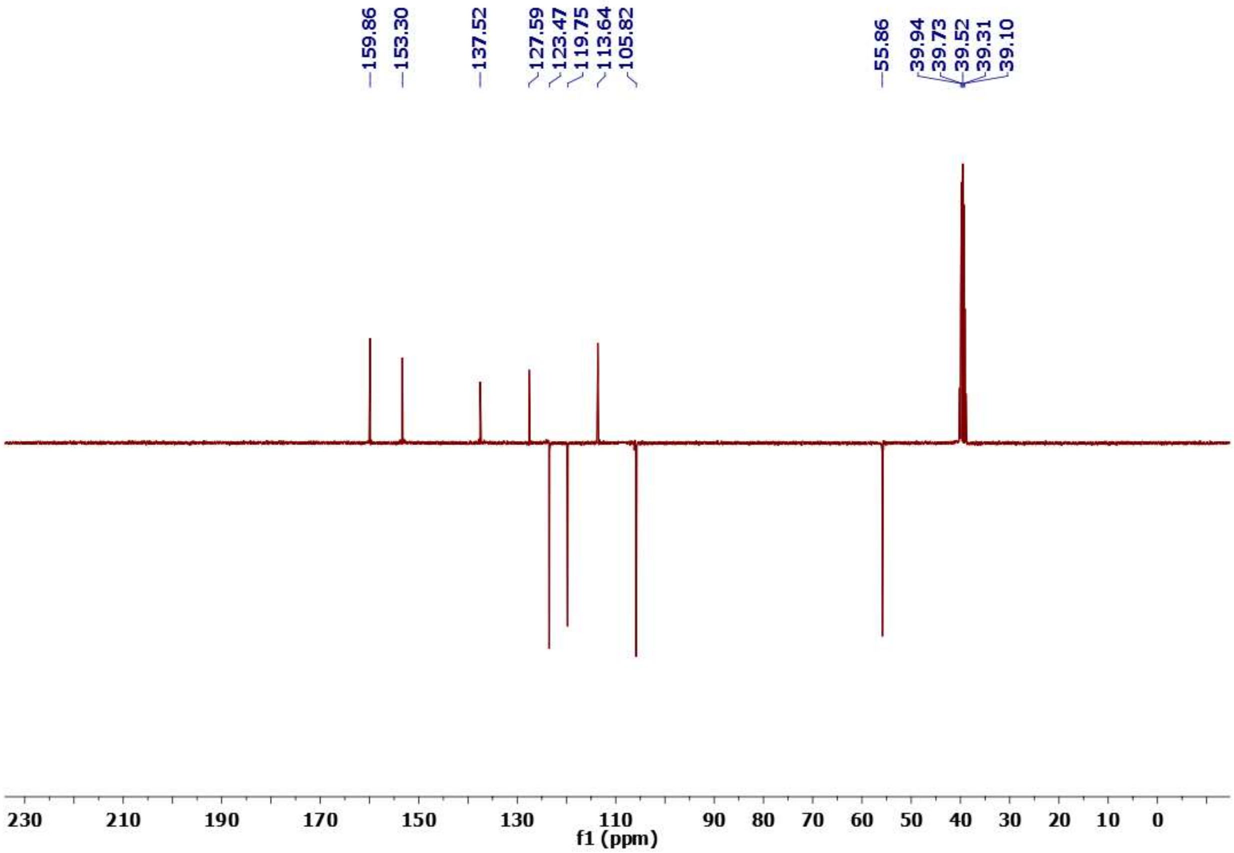

^{1}H-^{1}H-gDQCOSY NMR (DMSO-d6) spectrum of 5-methoxybenzo[d]thiazole-2-carbonitrile (3b)

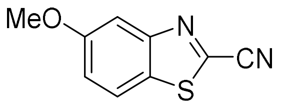

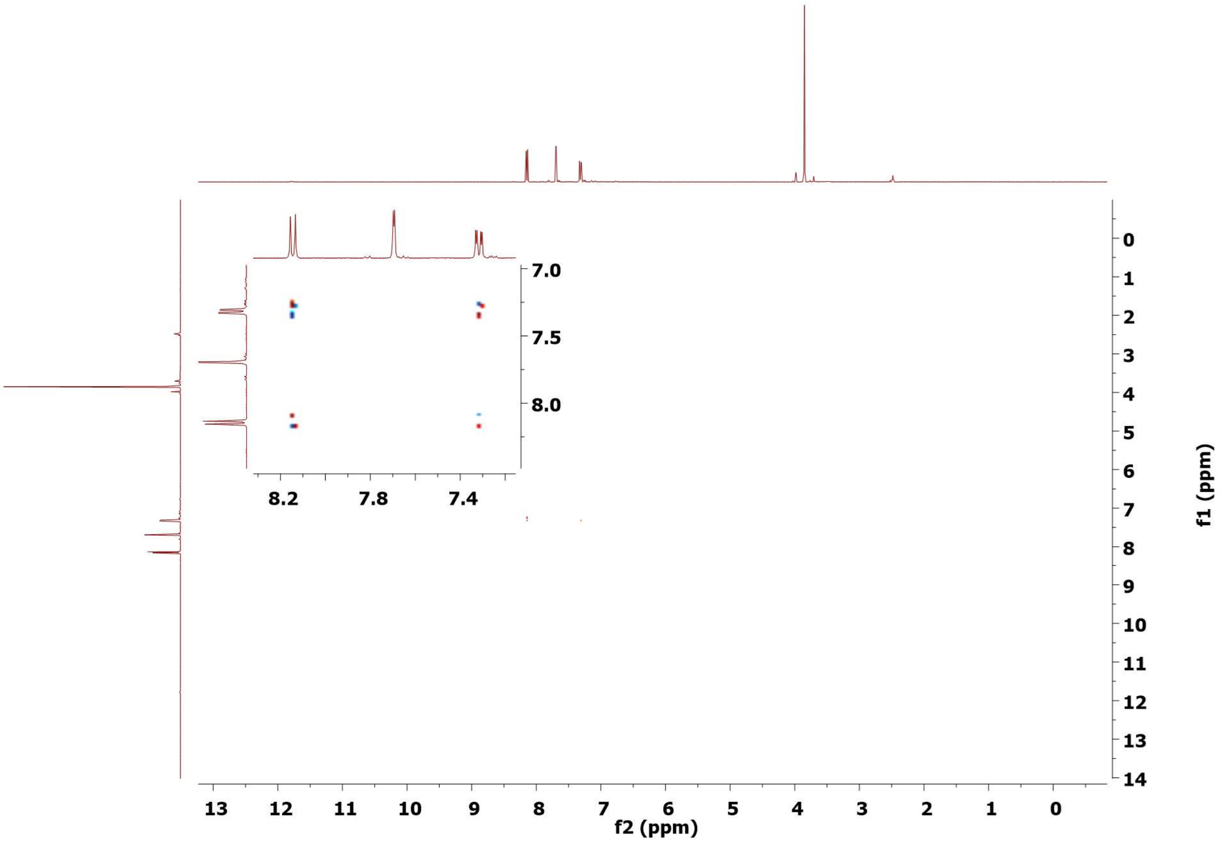

^{1}H-^{13}C-gHSQC NMR (DMSO-d6) spectrum of 5-methoxybenzo[d]thiazole-2-carbonitrile (3b)

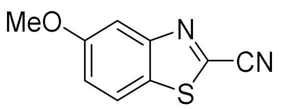

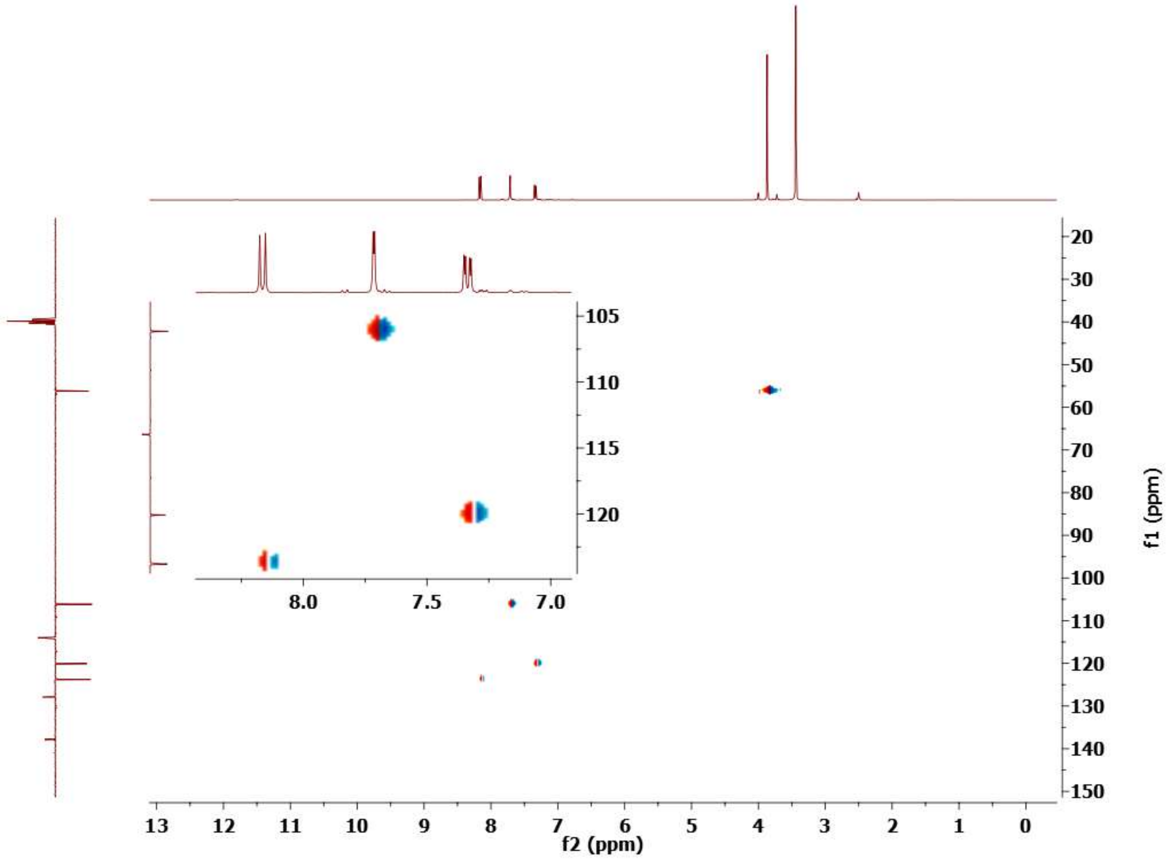

^{1}H-^{13}C-gHMBC NMR (DMSO-d6) spectrum of 5-methoxybenzo[d]thiazole-2-carbonitrile (3b)

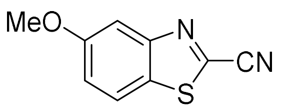

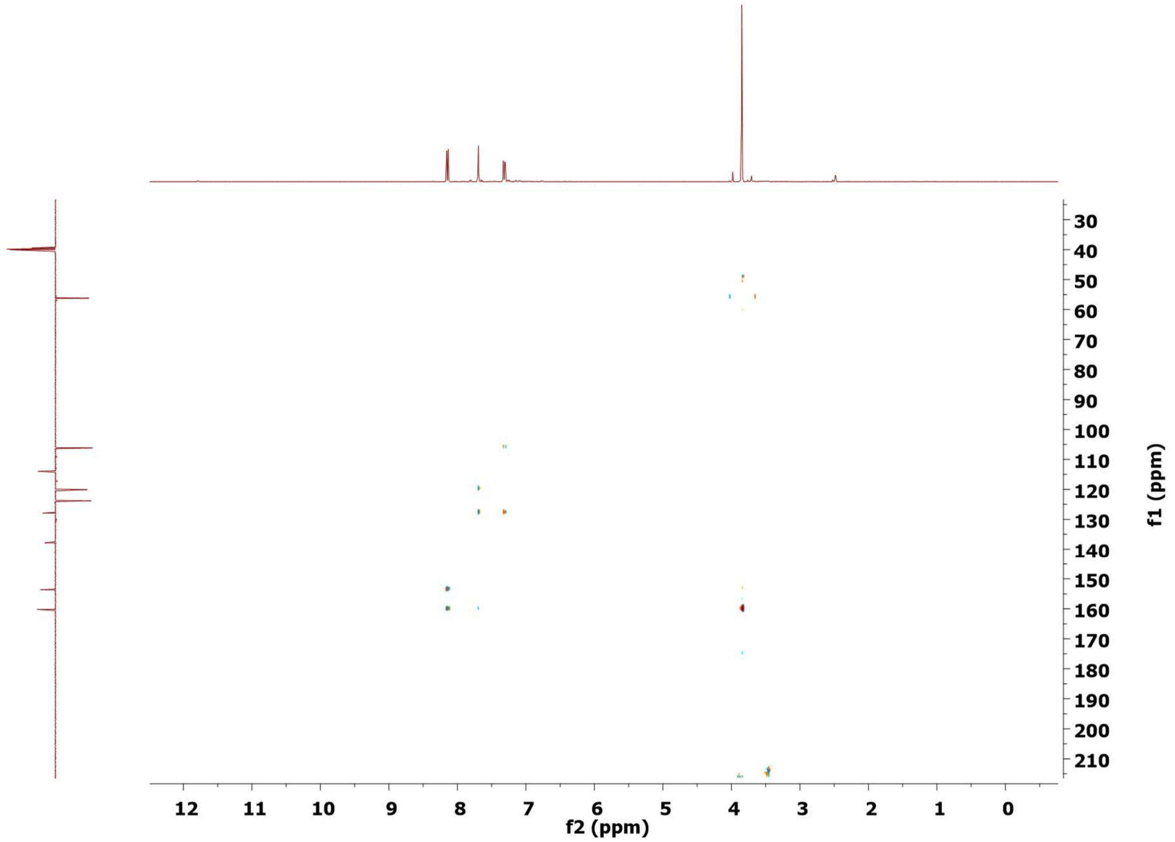

^{1}H NMR ($CDCl_3$) spectrum of 5-methoxybenzo[d]thiazole-2-carbonitrile (3b)

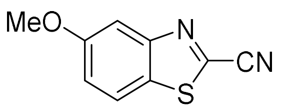

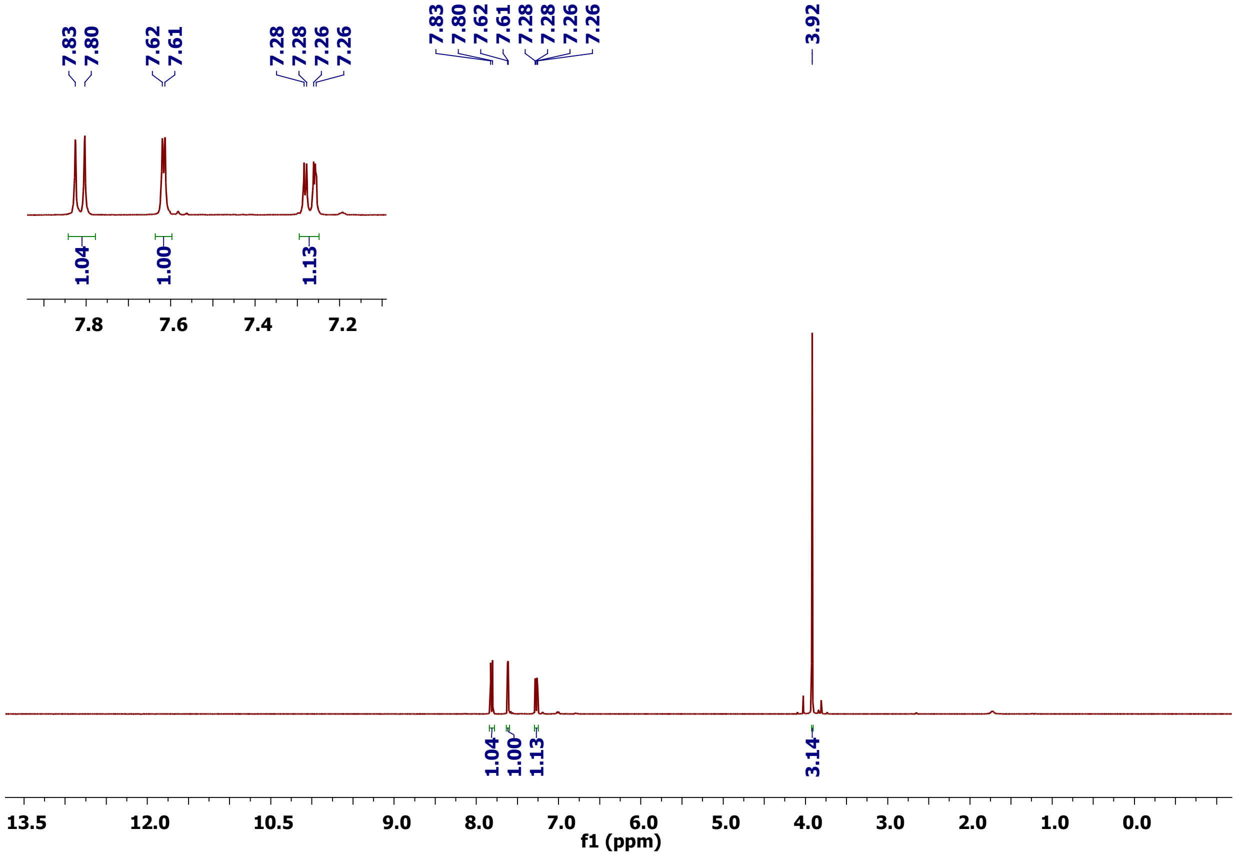

^{13}C CRAPT NMR ($CDCl_3$) spectrum of 5-methoxybenzo[d]thiazole-2-carbonitrile (3b)

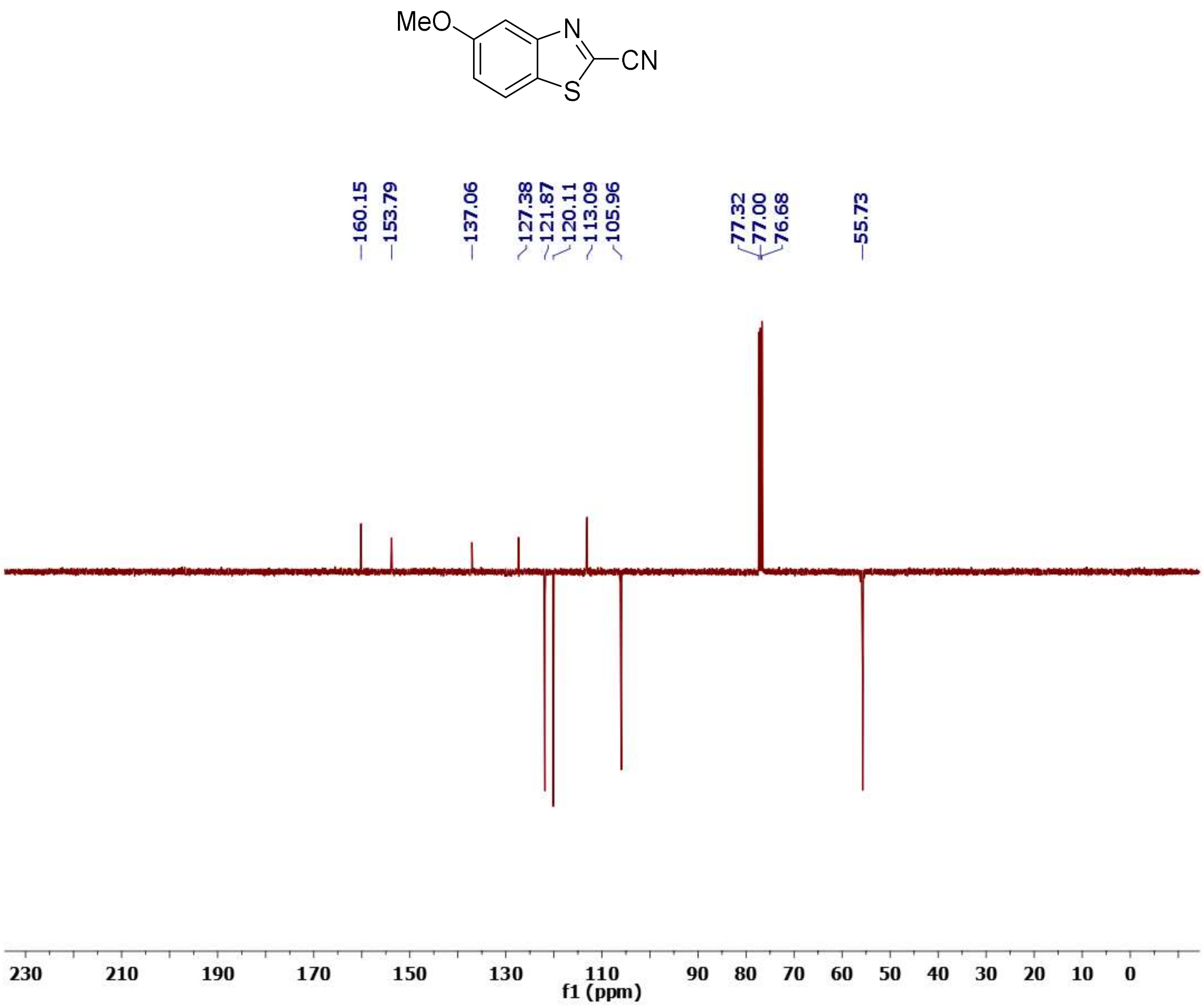

^{1}H NMR (DMSO-d6) spectrum of 5-(benzyloxy)benzo[d]thiazole-2-carbonitrile (3c)

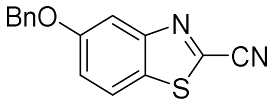

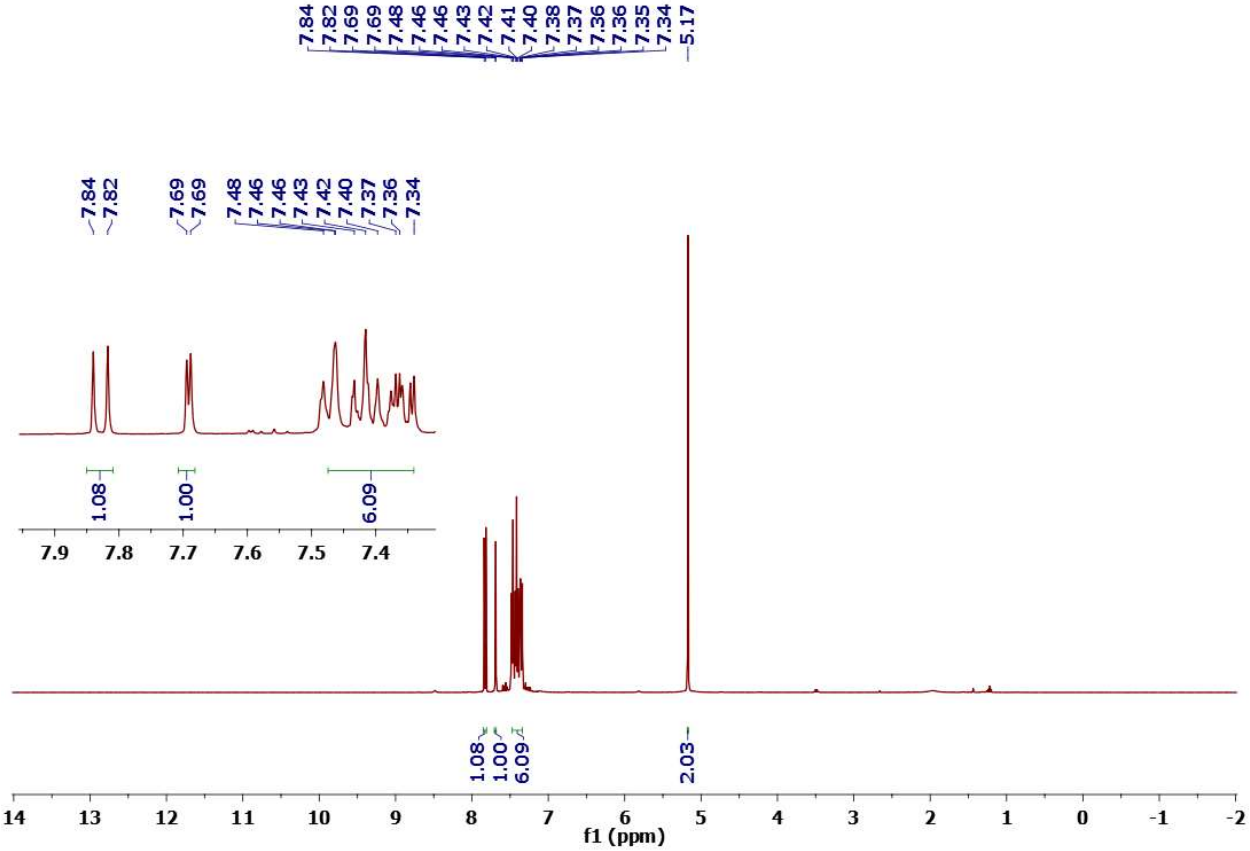

^{13}C NMR (DMSO-d6) spectrum of 5-(benzyloxy)benzo[d]thiazole-2-carbonitrile (3c)

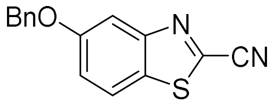

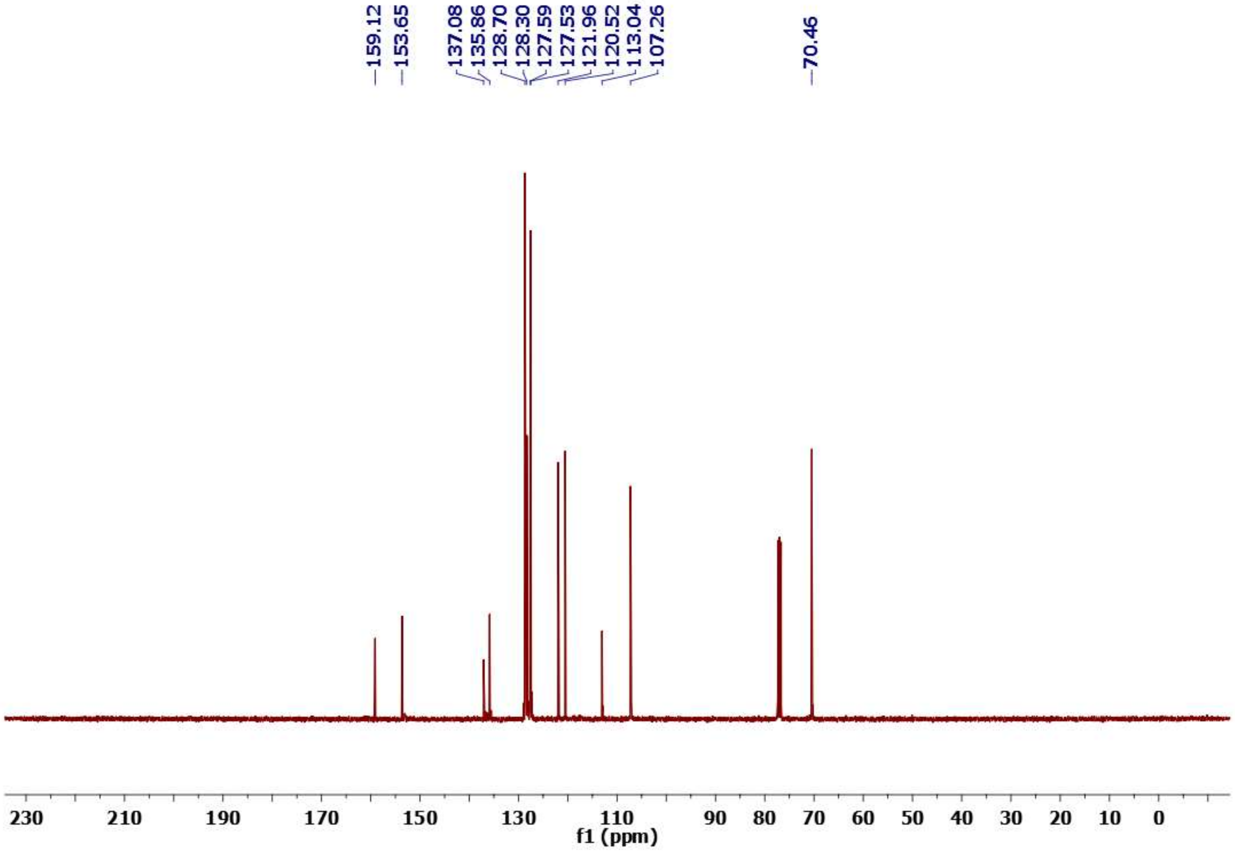

^{13}C CRAPT NMR (DMSO-d6) spectrum of 5-(benzyloxy)benzo[d]thiazole-2-carbonitrile (3c)

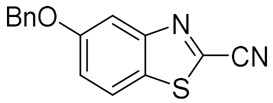

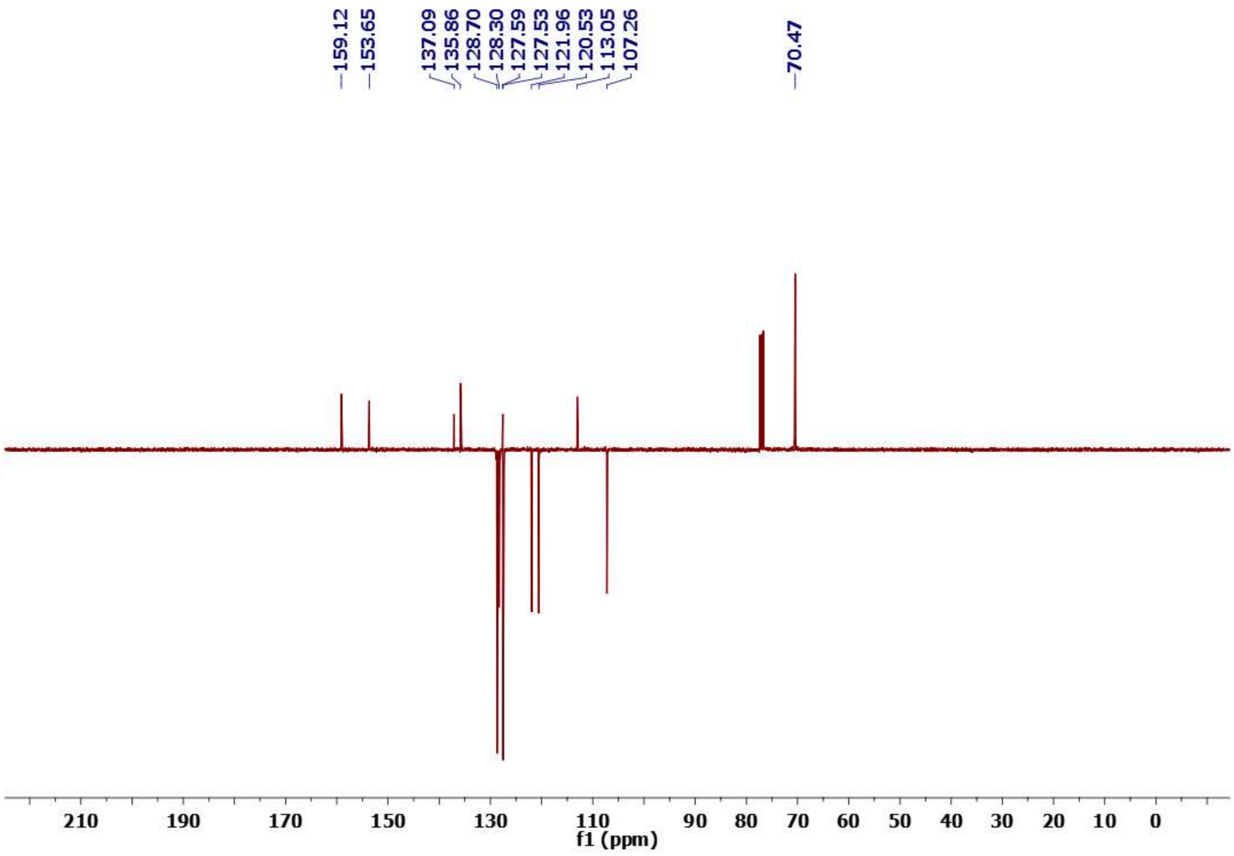

^{1}H-^{1}H-gDQCOSY NMR (DMSO-d6) spectrum of 5-(benzyloxy)benzo[d]thiazole-2-carbonitrile (3c)

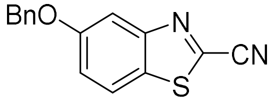

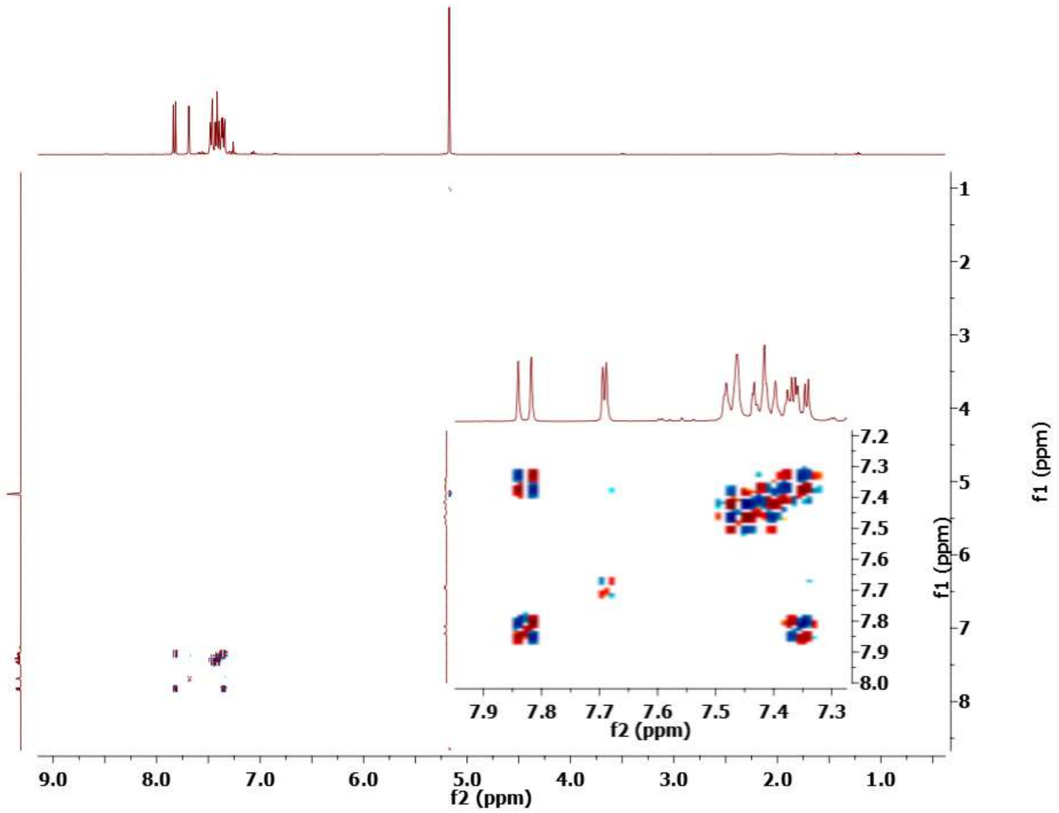

^{1}H-^{13}C-gHSQC NMR (DMSO-d6) spectrum of 5-(benzyloxy)benzo[d]thiazole-2-carbonitrile (3c)

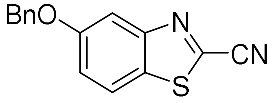

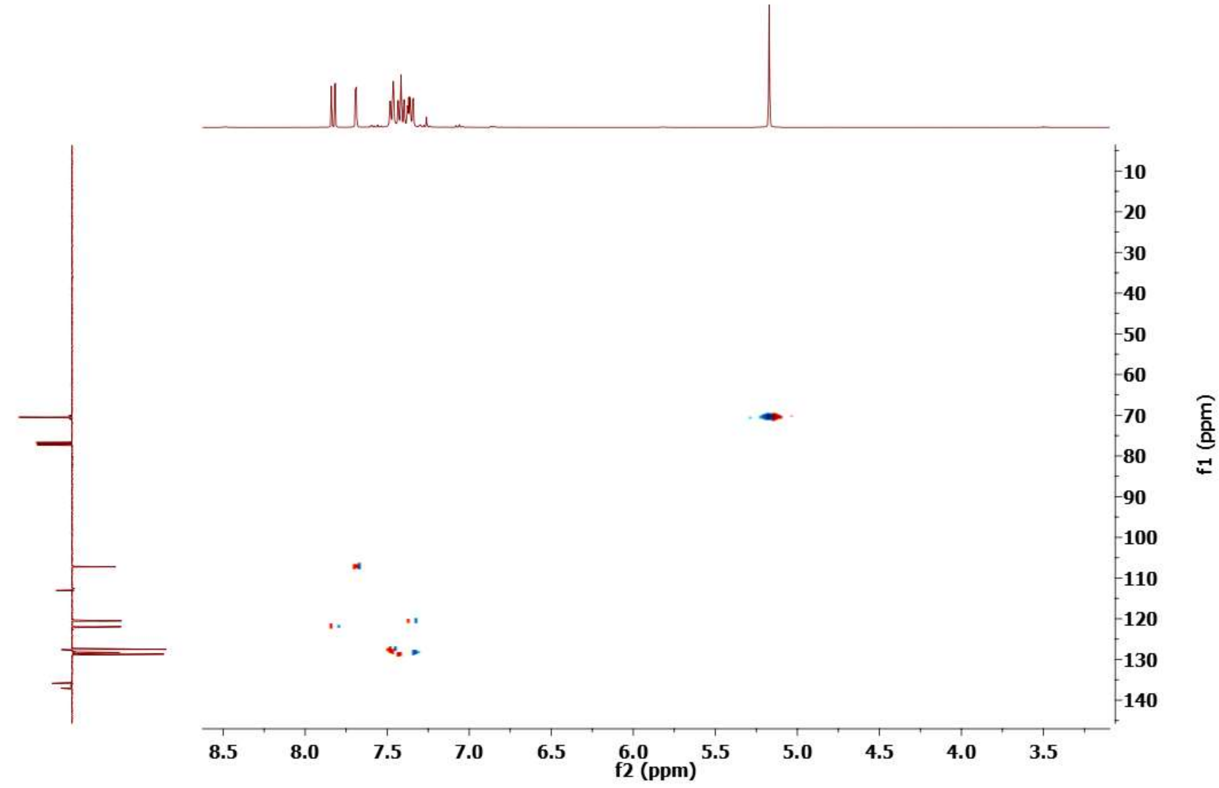

^{1}H-^{13}C-gHMBC NMR (DMSO-d6) spectrum of 5-(benzyloxy)benzo[d]thiazole-2-carbonitrile (3c)

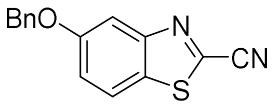

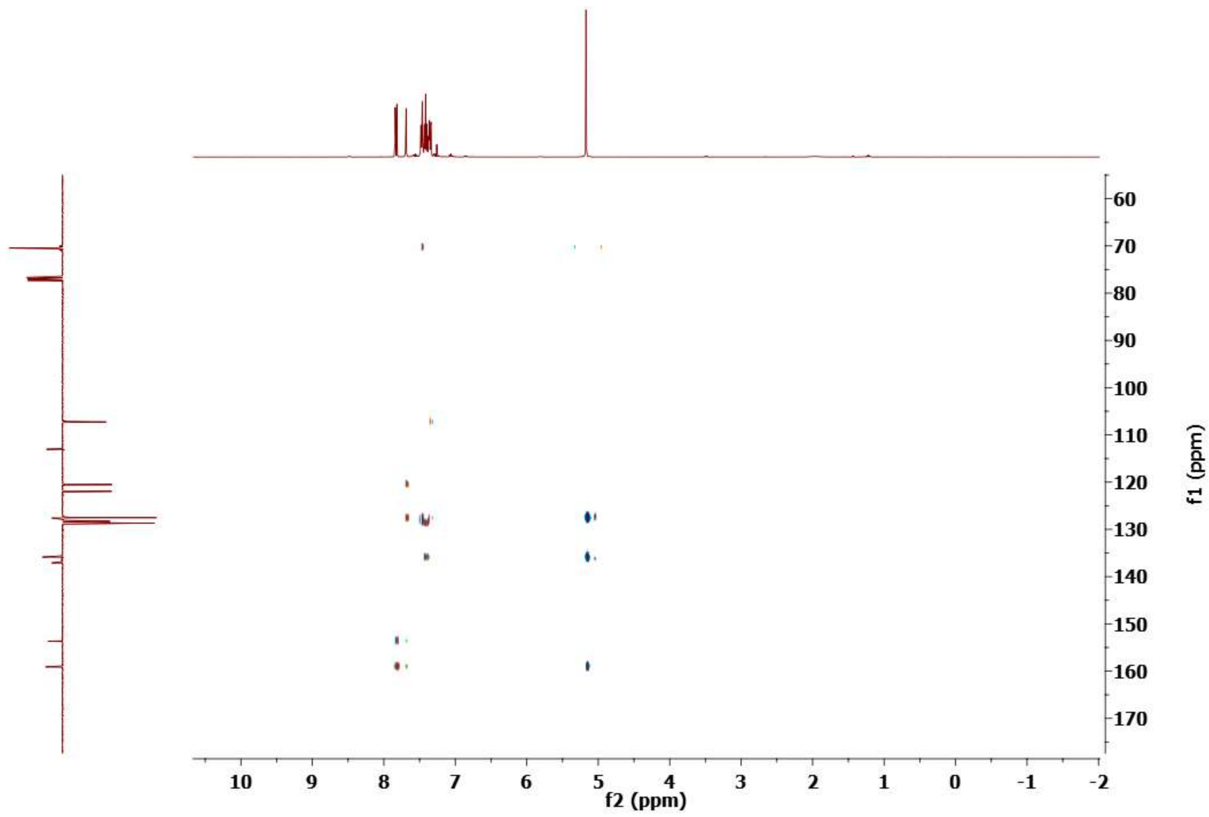

^{1}H NMR (DMSO-d6) spectrum of 5-(methylthio)benzo[d]thiazole-2-carbonitrile (3d)

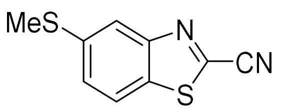

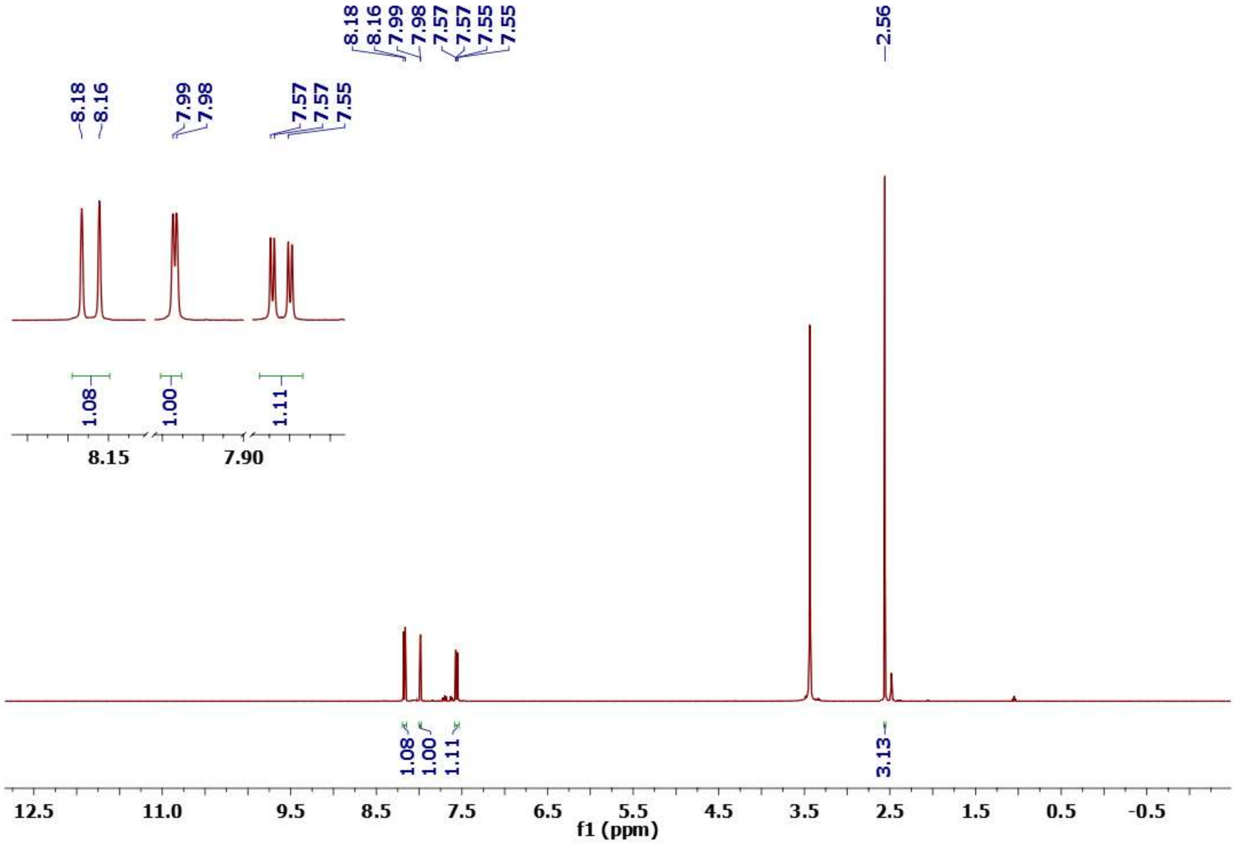

^{13}C NMR (DMSO-d6) spectrum of 5-(methylthio)benzo[d]thiazole-2-carbonitrile (3d)

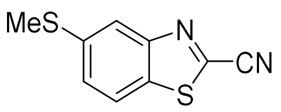

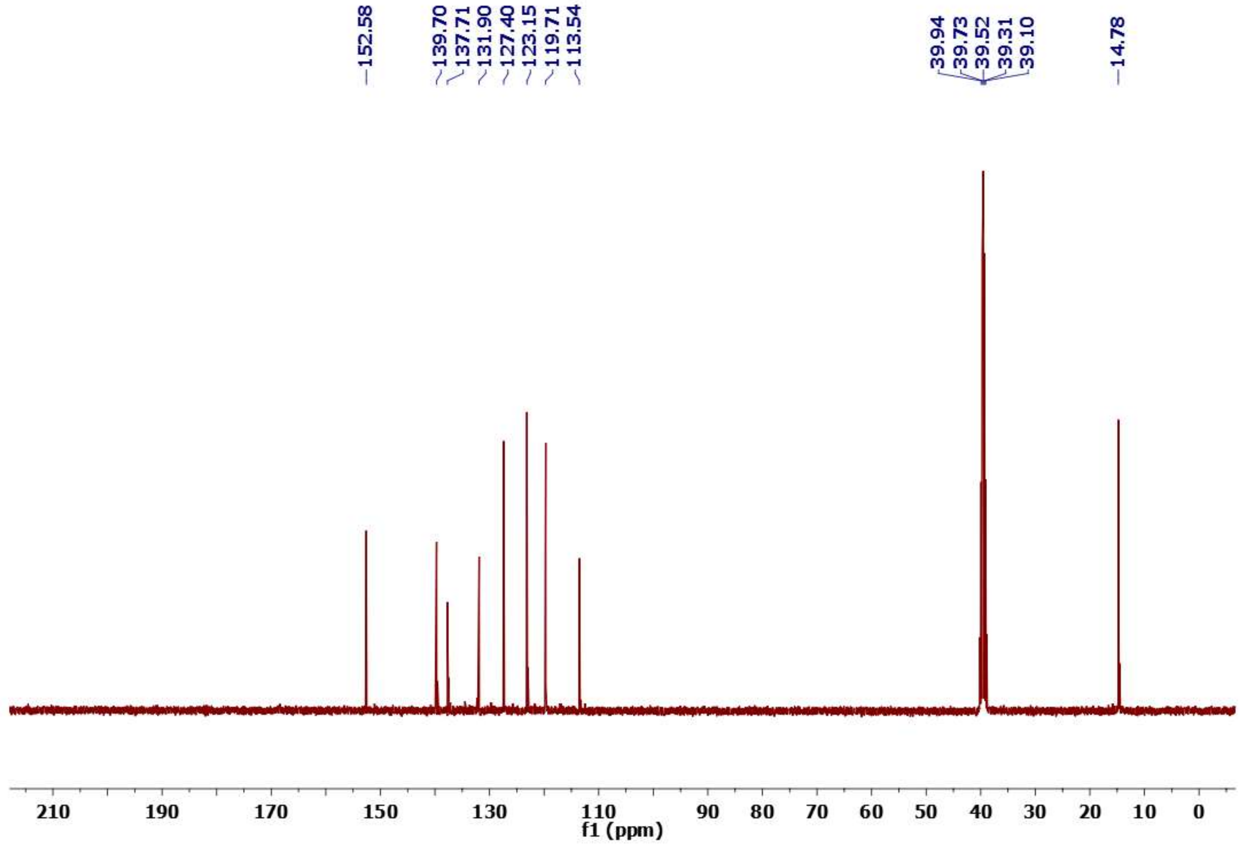

^{13}C CRAPT NMR (DMSO-d6) spectrum of 5-(methylthio)benzo[d]thiazole-2-carbonitrile (3d)

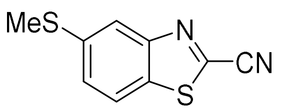

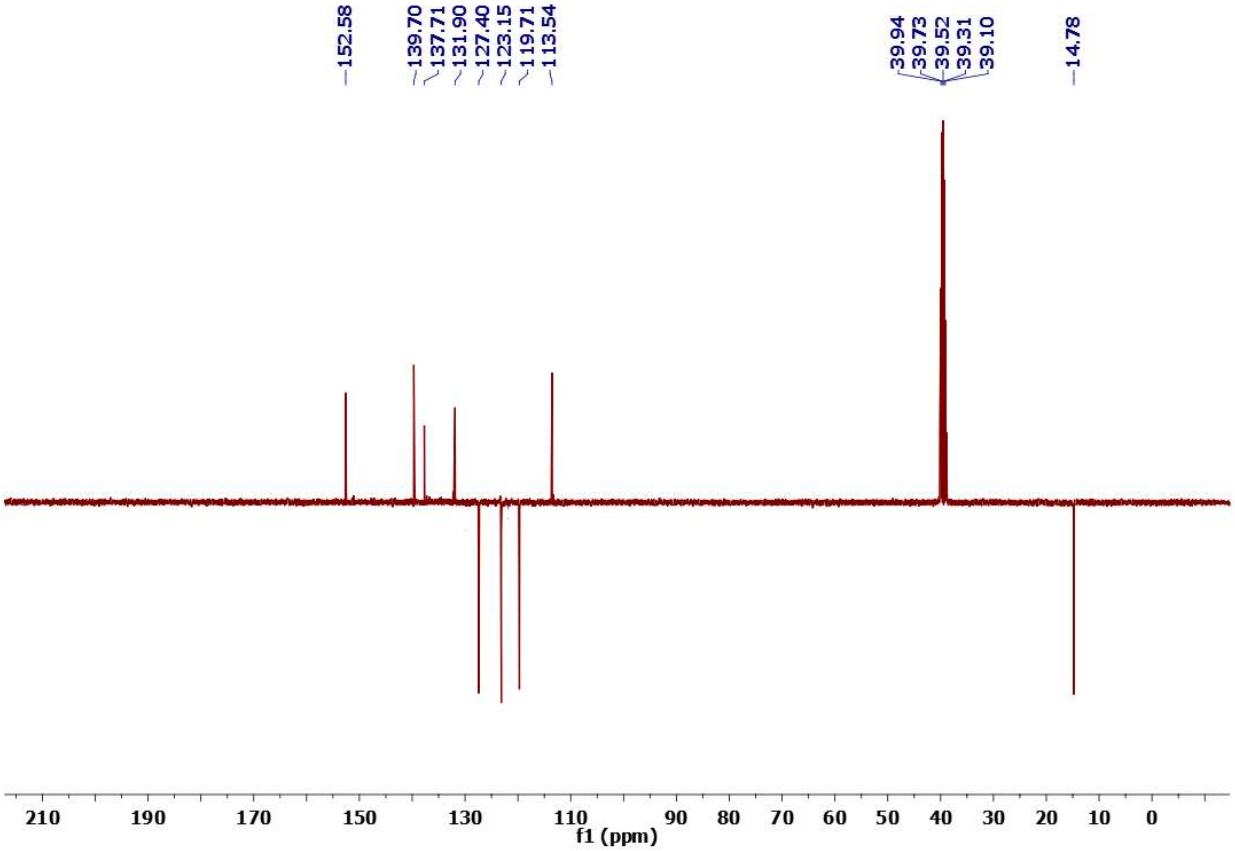

^{1}H-^{1}H-gDQCOSY NMR (DMSO-d6) spectrum of 5-(methylthio)benzo[d]thiazole-2-carbonitrile (3d)

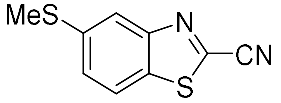

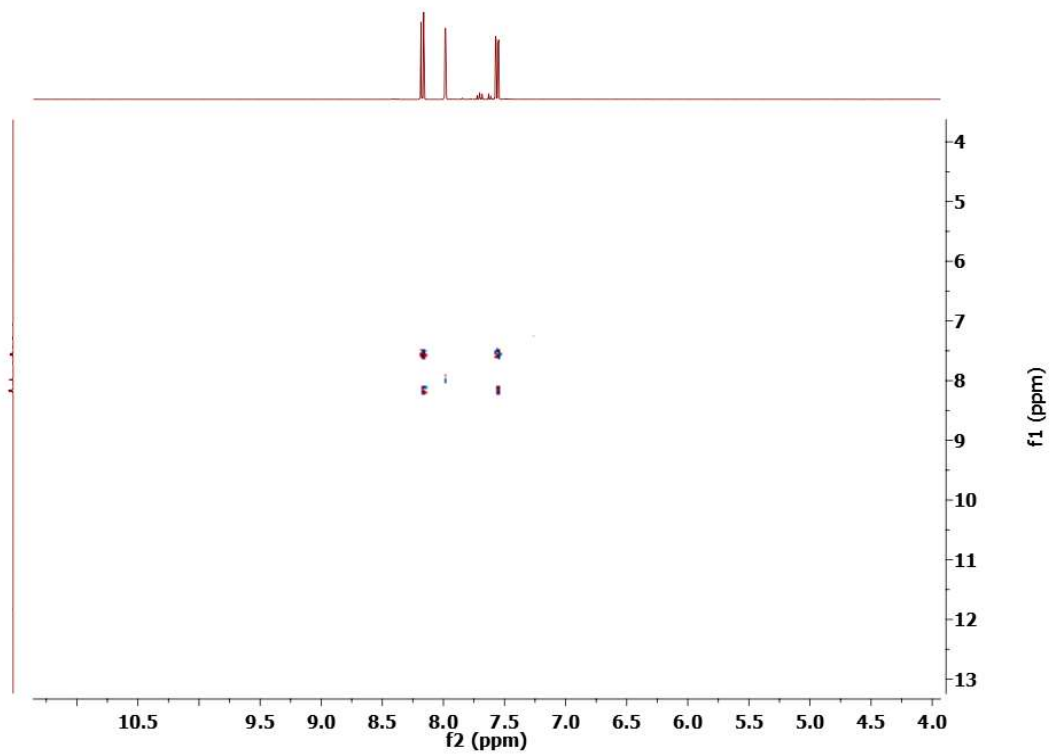

^{1}H-^{13}C-gHSQC NMR (DMSO-d6) spectrum of 5-(methylthio)benzo[d]thiazole-2-carbonitrile (3d)

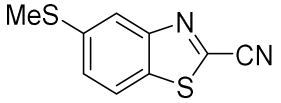

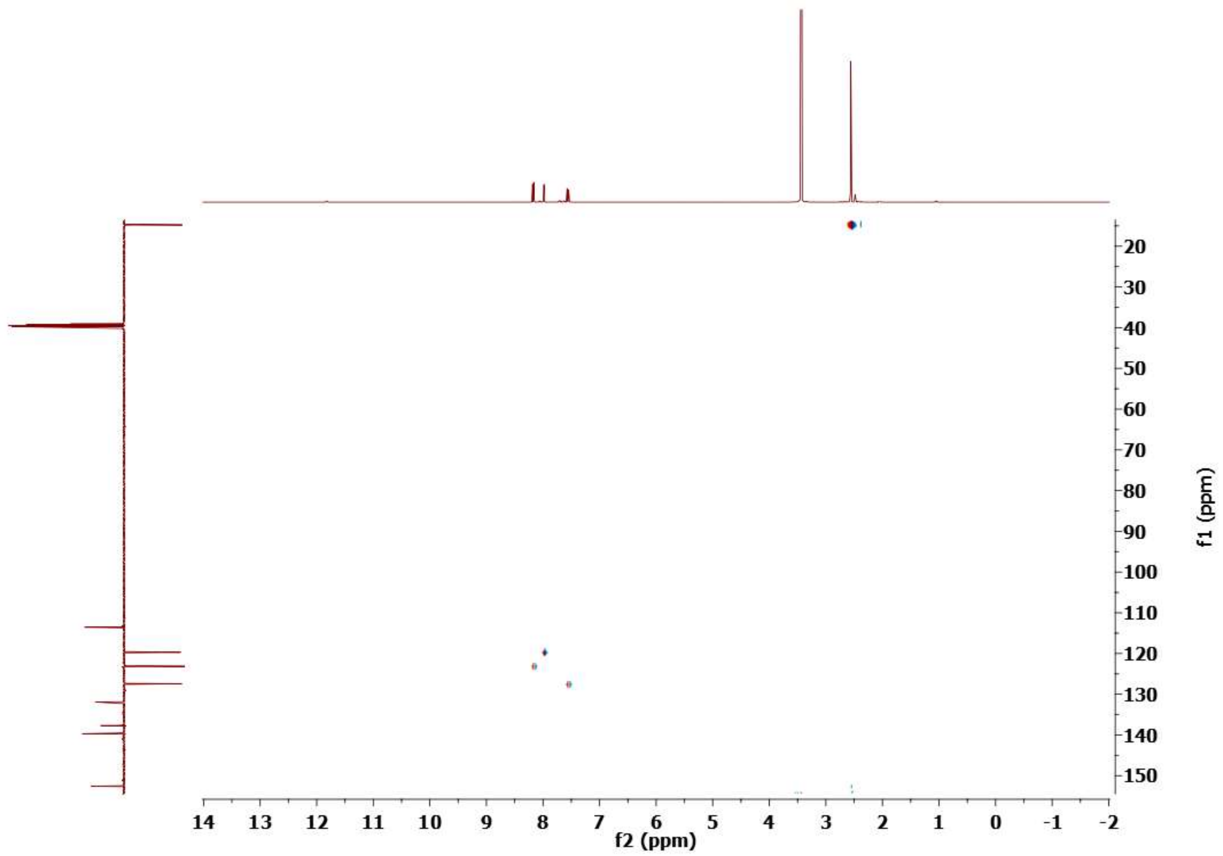

^{1}H-^{13}C-gHMBC NMR (DMSO-d6) spectrum of 5-(methylthio)benzo[d]thiazole-2-carbonitrile (3d)

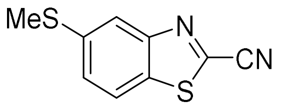

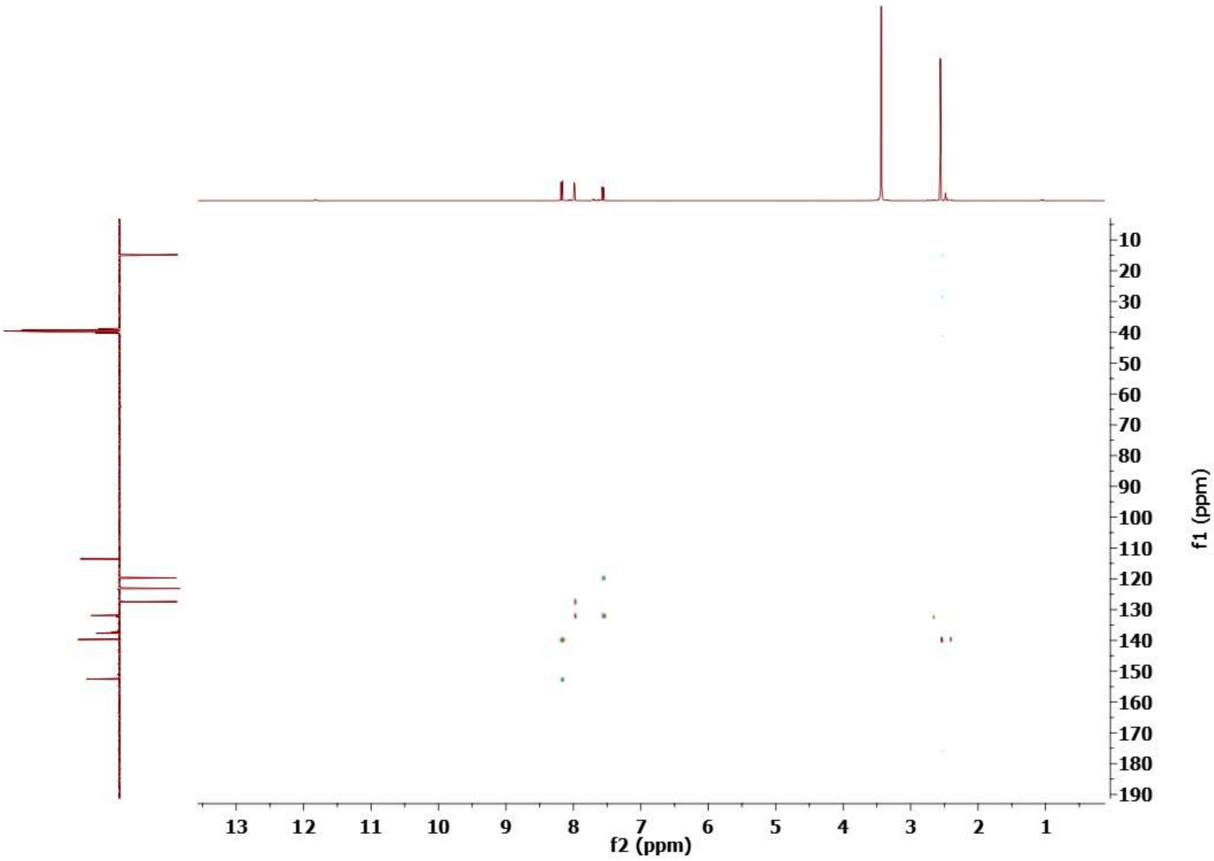

^{1}H NMR (DMSO-d6) spectrum of 5,6-dimethoxybenzo[d]thiazole-2-carbonitrile (3e)

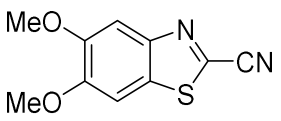

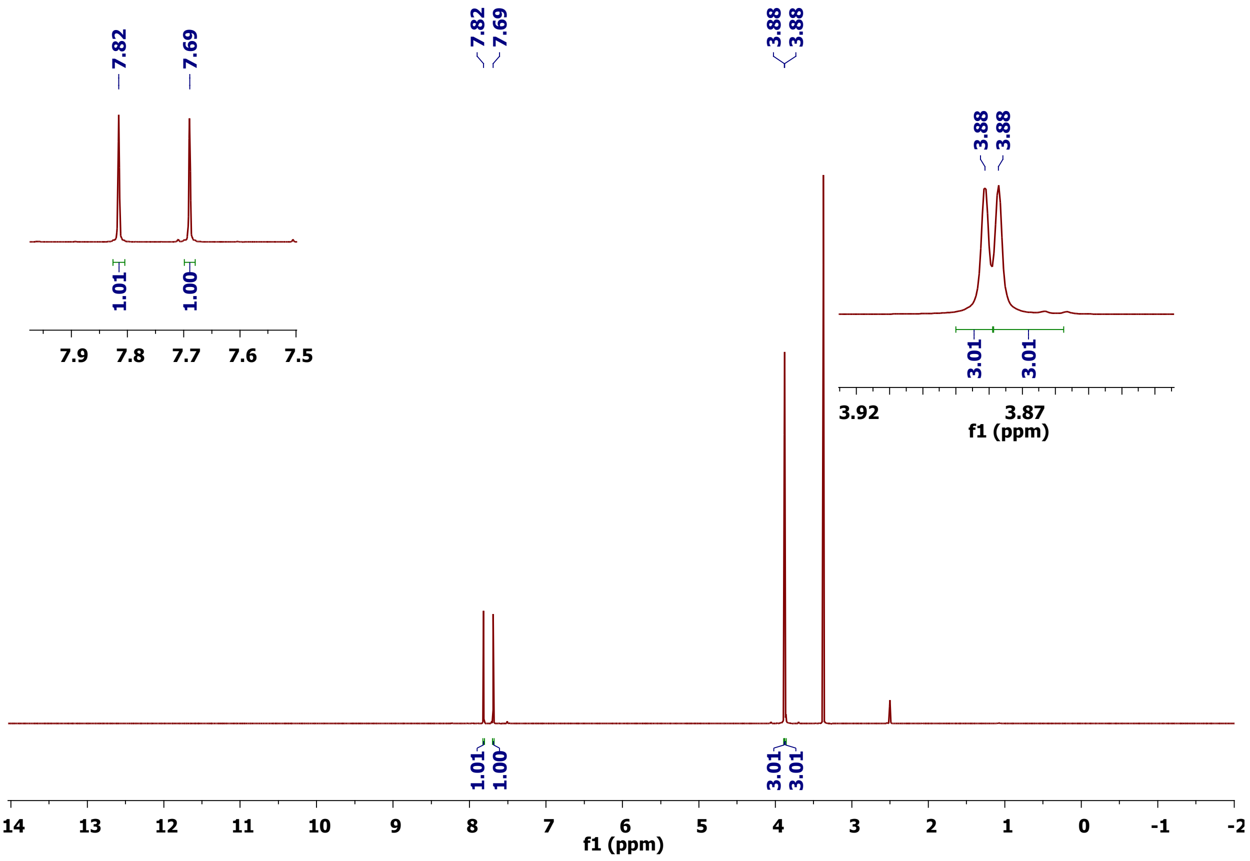

^{13}C NMR (DMSO-d6) spectrum of 5,6-dimethoxybenzo[d]thiazole-2-carbonitrile (3e)

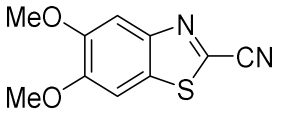

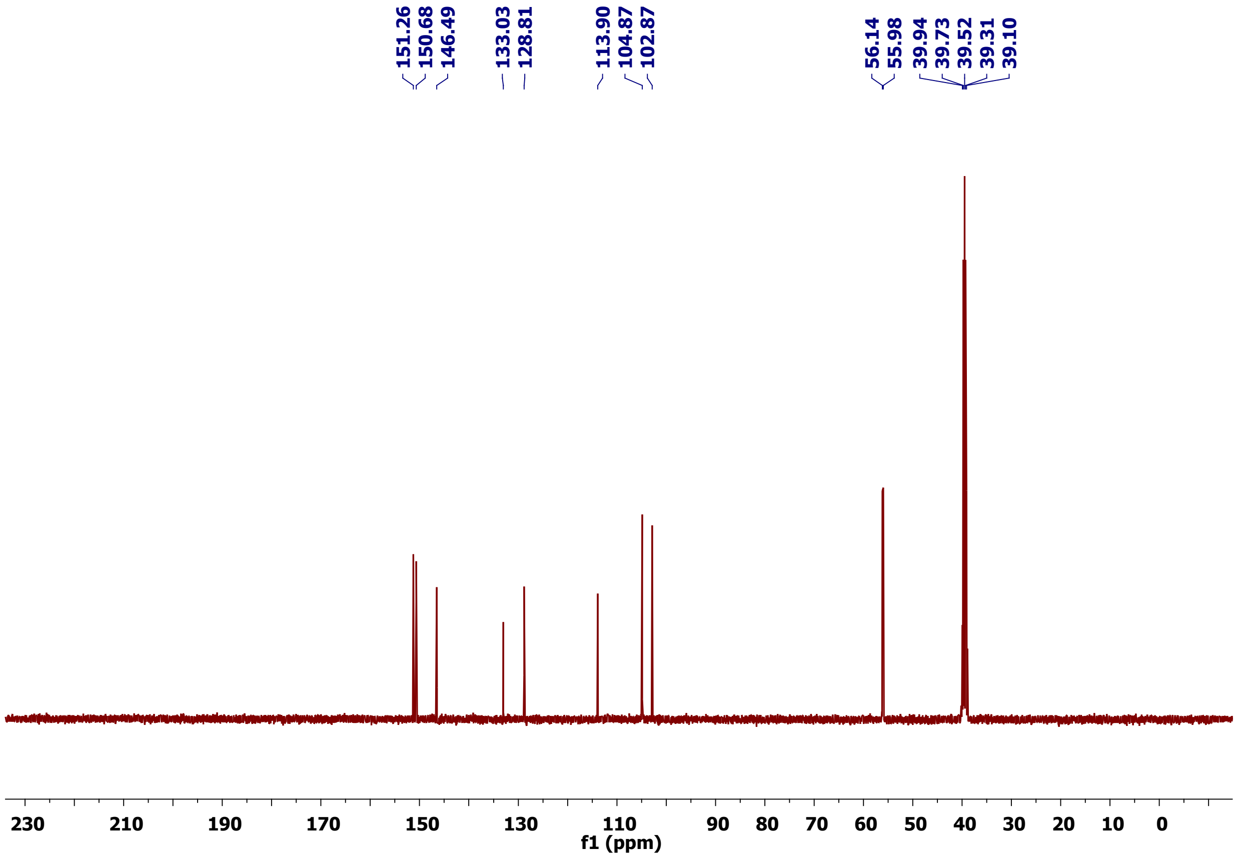

^{13}C CRAPT NMR (DMSO-d6) spectrum of 5,6-dimethoxybenzo[d]thiazole-2-carbonitrile (3e)

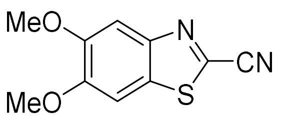

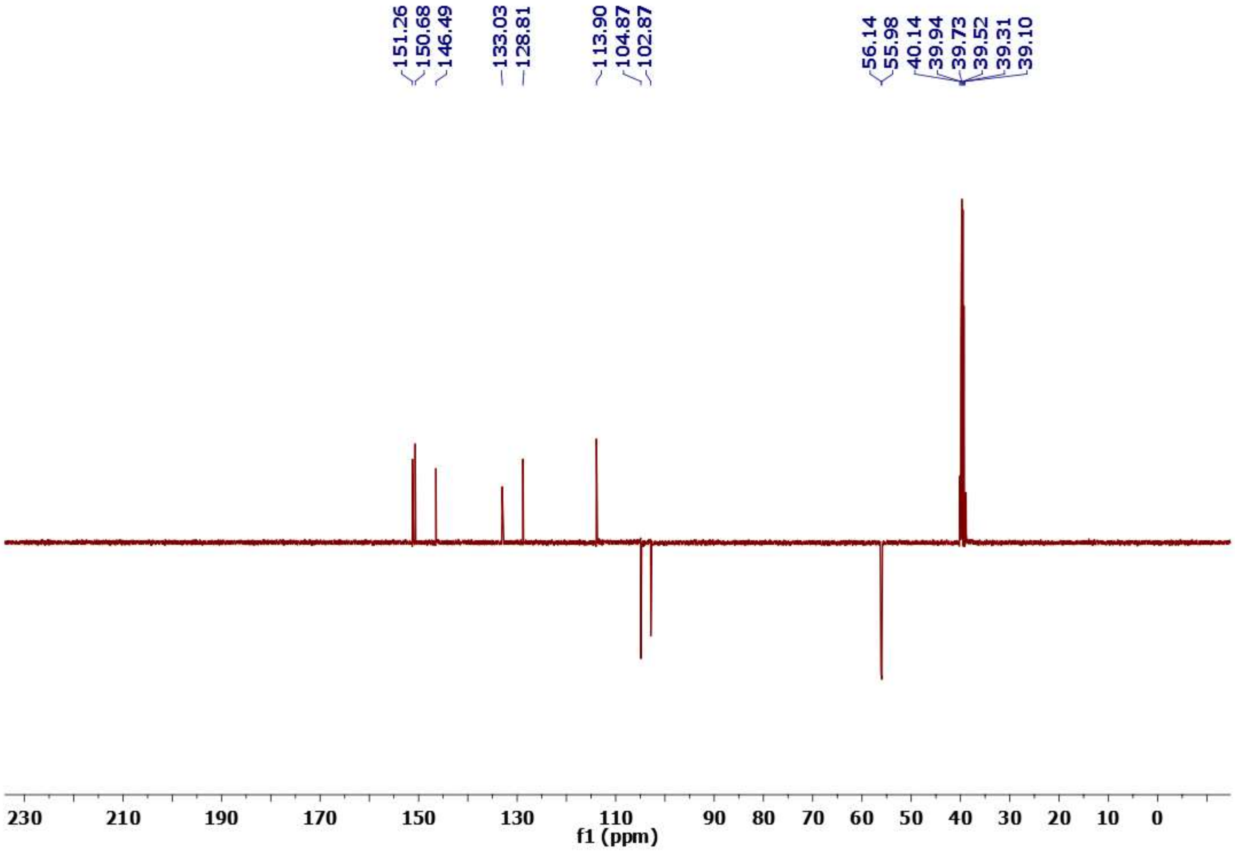

^{1}H-^{1}H-gDQCOSY NMR (DMSO-d6) spectrum of 5,6-dimethoxybenzo[d]thiazole-2-carbonitrile (3e)

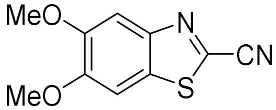

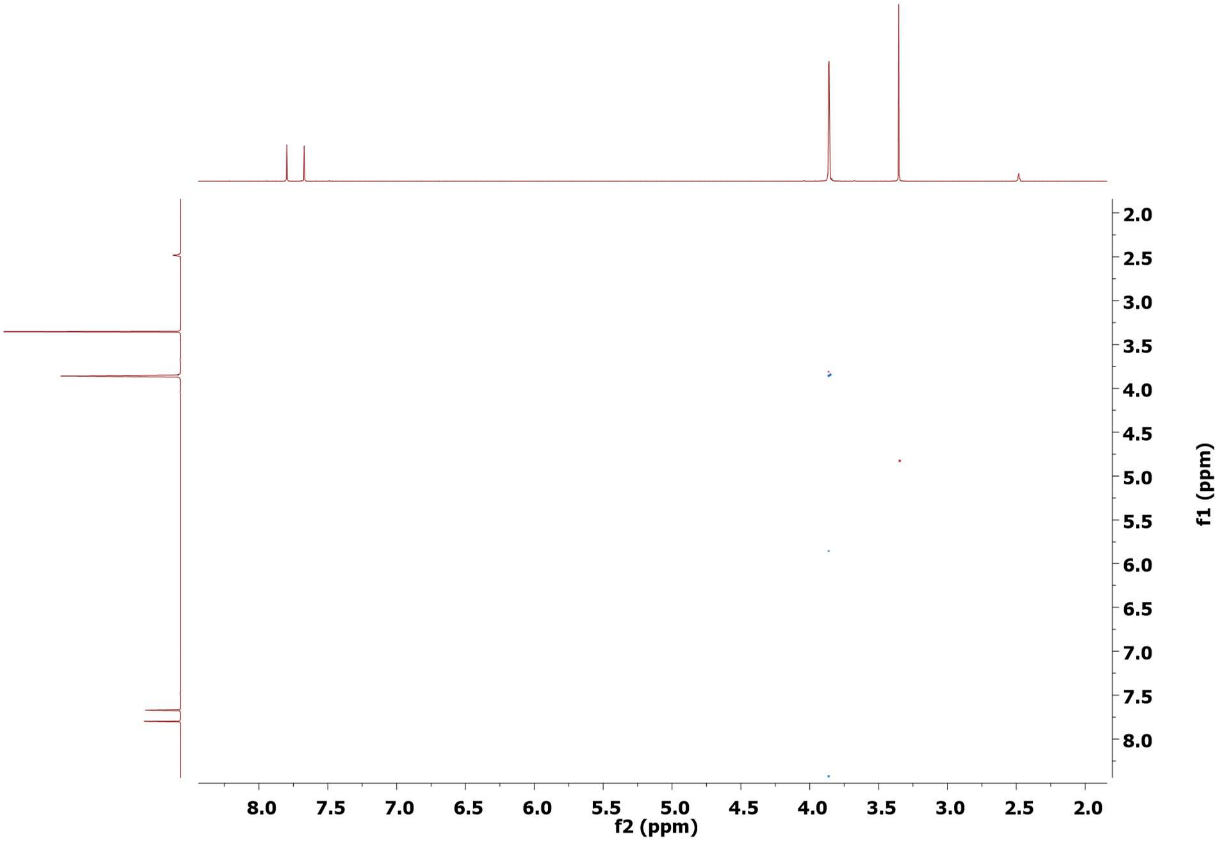

^{1}H-^{13}C-gHSQC NMR (DMSO-d6) spectrum of 5,6-dimethoxybenzo[d]thiazole-2-carbonitrile (3e)

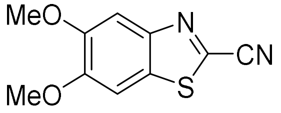

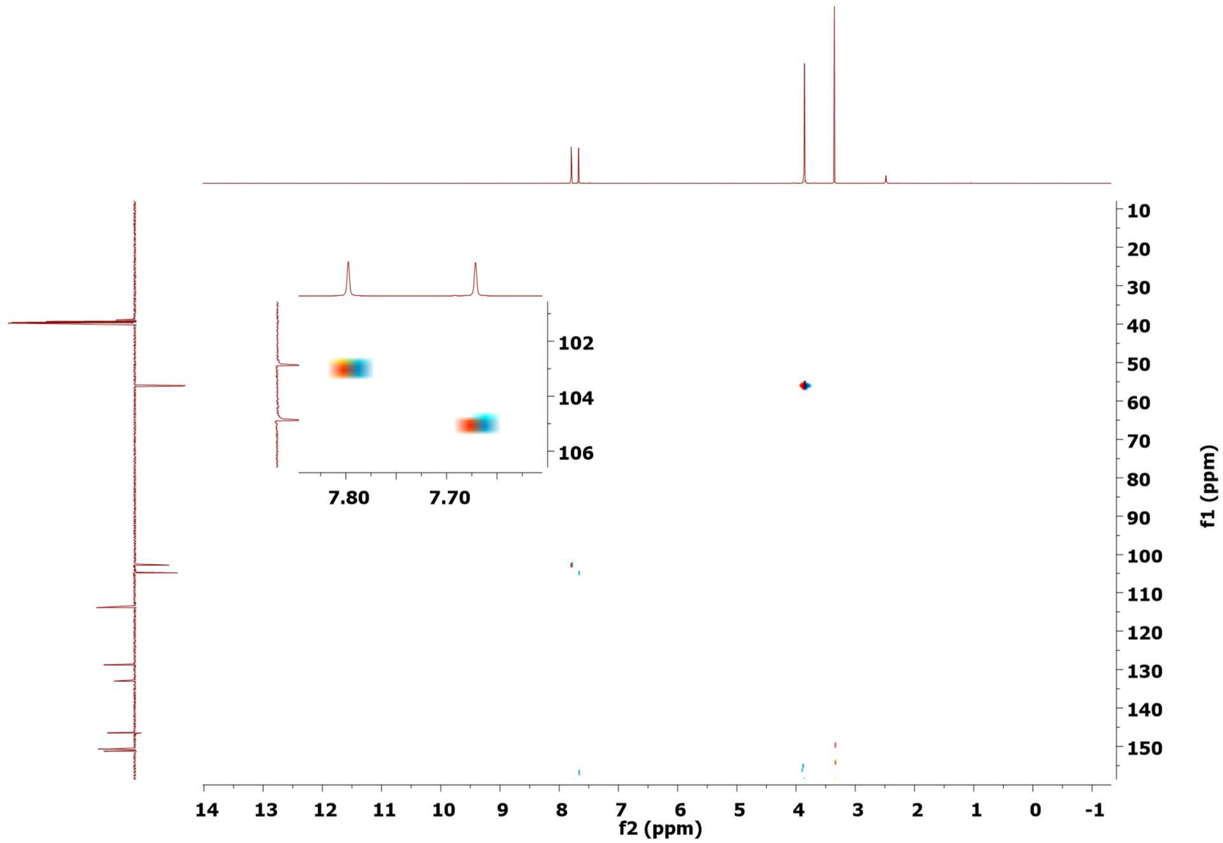

^{1}H-^{13}C-gHMBC NMR (DMSO-d6) spectrum of 5,6-dimethoxybenzo[d]thiazole-2-carbonitrile (3e)

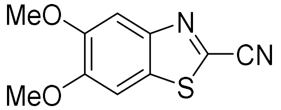

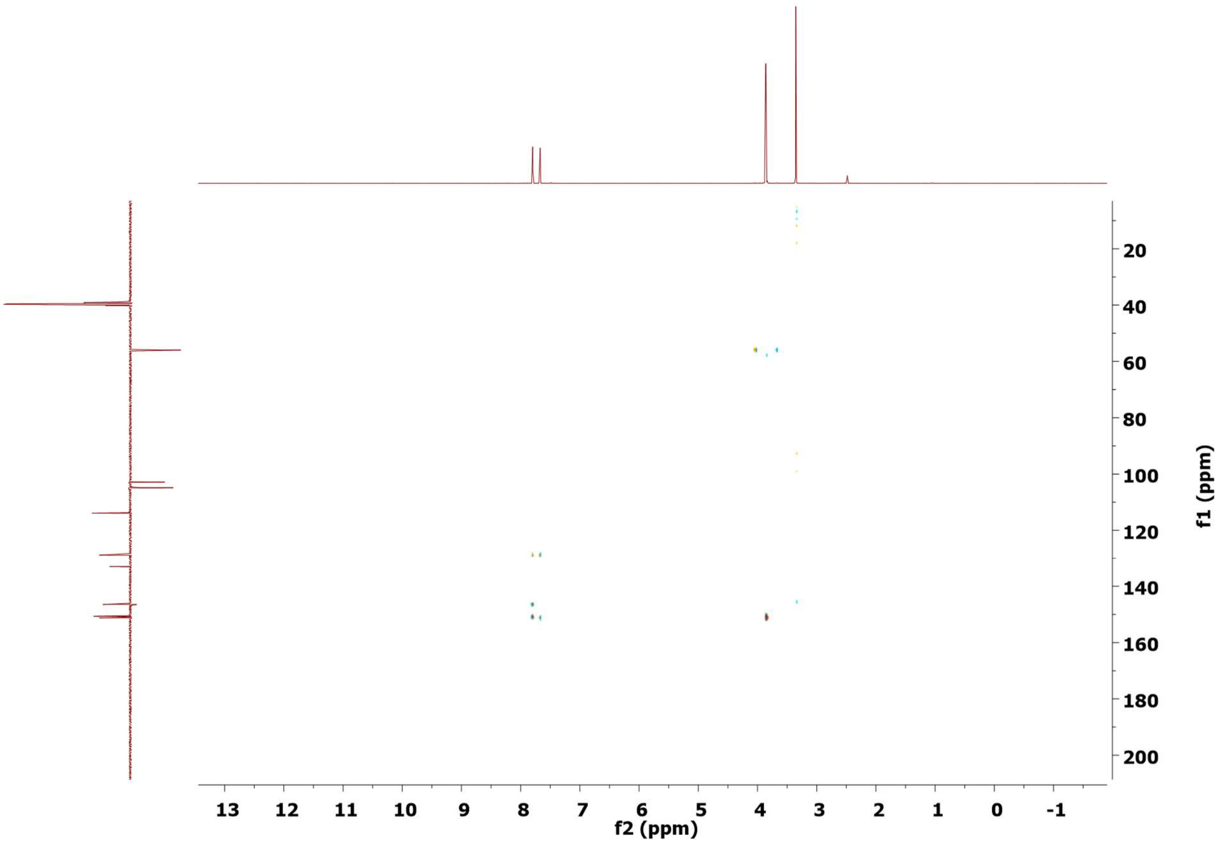

^{1}H NMR (DMSO-d6) spectrum of 4,7-dimethoxybenzo[d]thiazole-2-carbonitrile (3f)

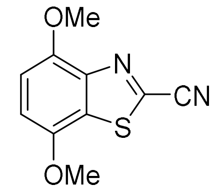

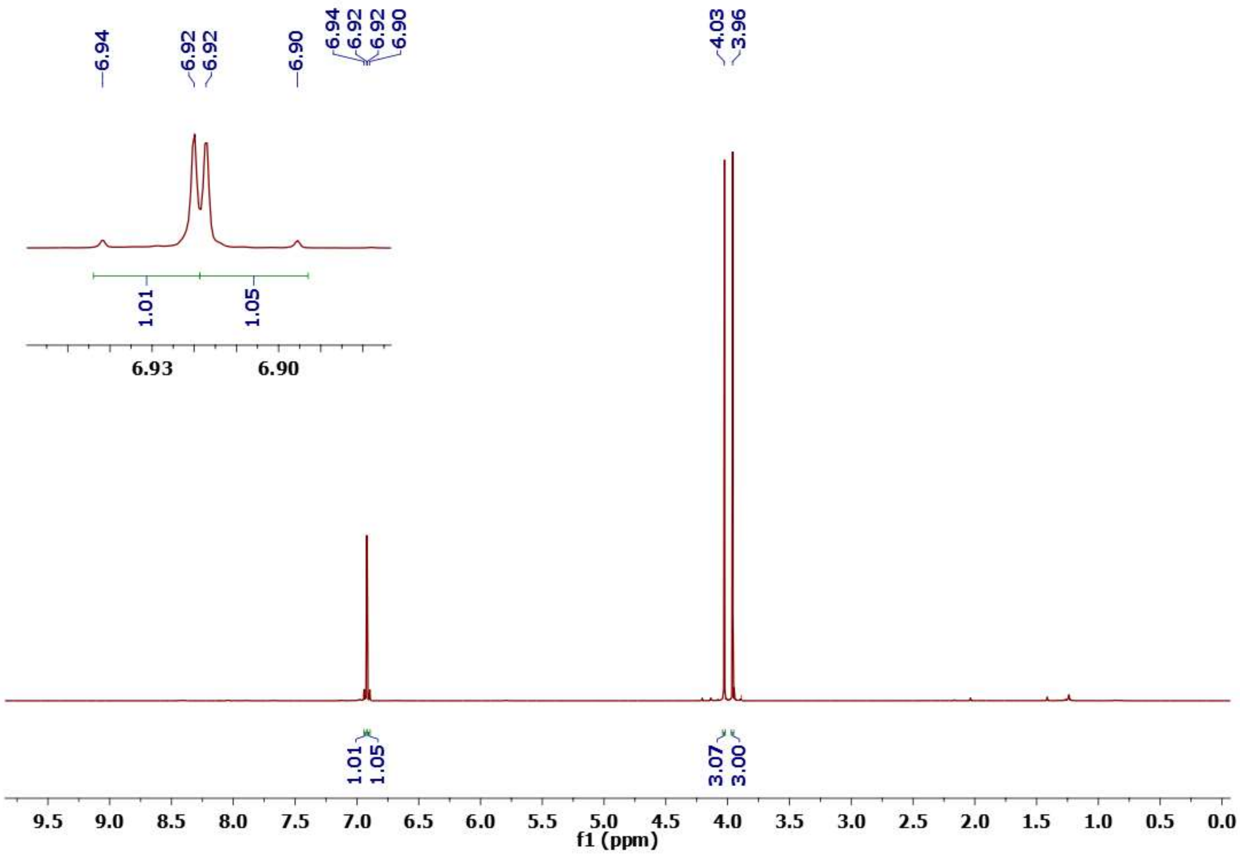

^{13}C NMR (DMSO-d6) spectrum of 4,7-dimethoxybenzo[d]thiazole-2-carbonitrile (3f)

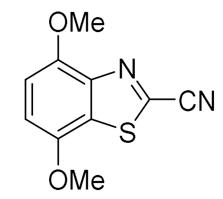

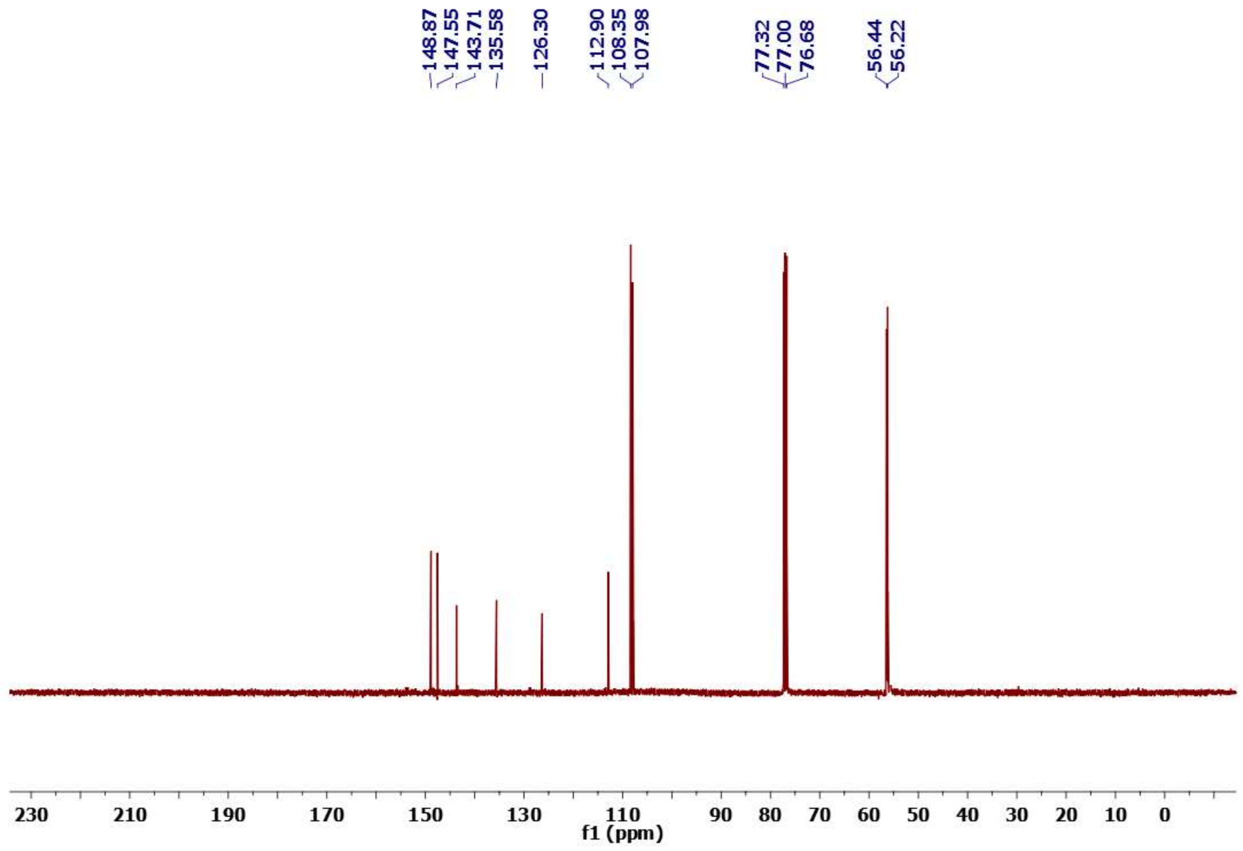

^{13}C CRAPT NMR (DMSO-d6) spectrum of 4,7-dimethoxybenzo[d]thiazole-2-carbonitrile (3f)

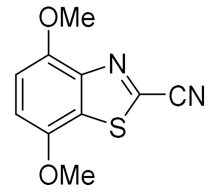

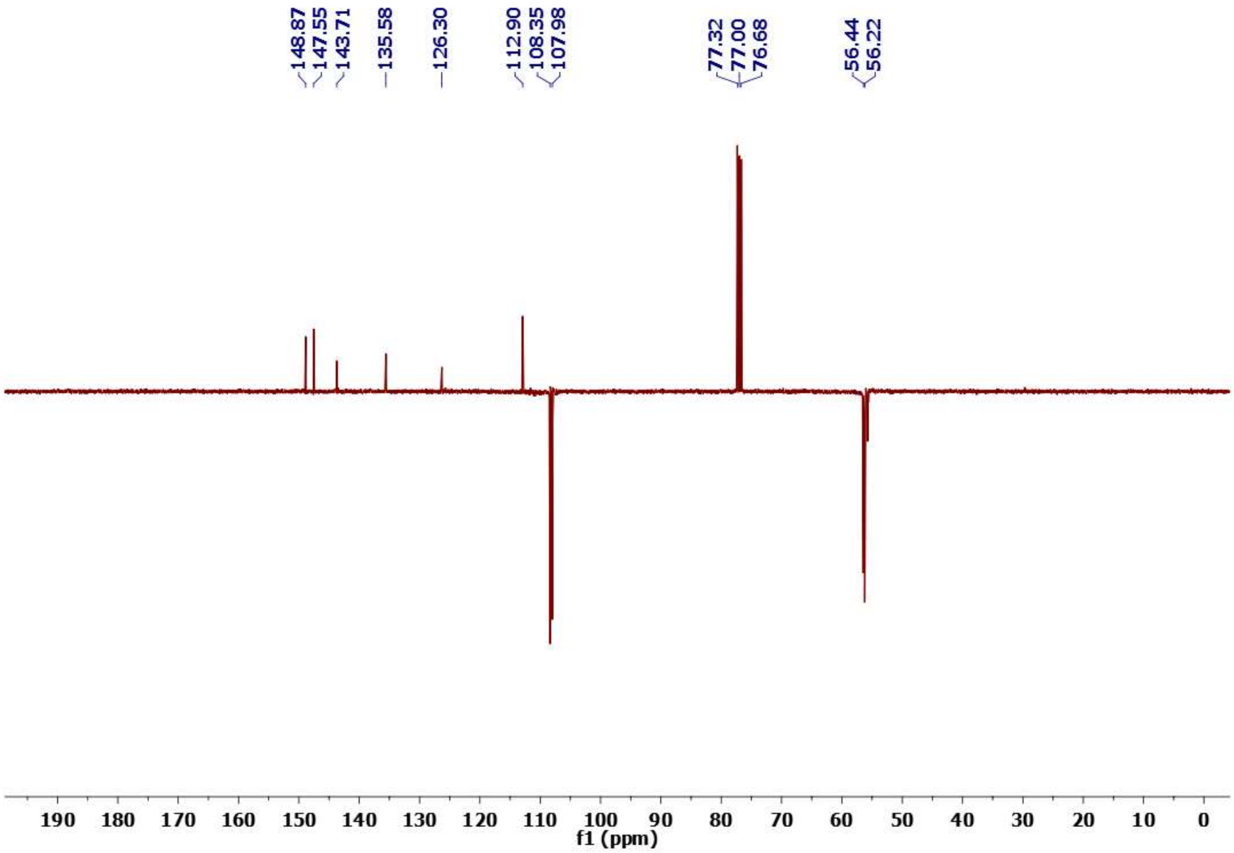

^{1}H-^{1}H-gDQCOSY NMR (DMSO-d6) spectrum of 4,7-dimethoxybenzo[d]thiazole-2-carbonitrile (3f)

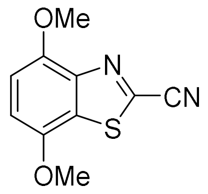

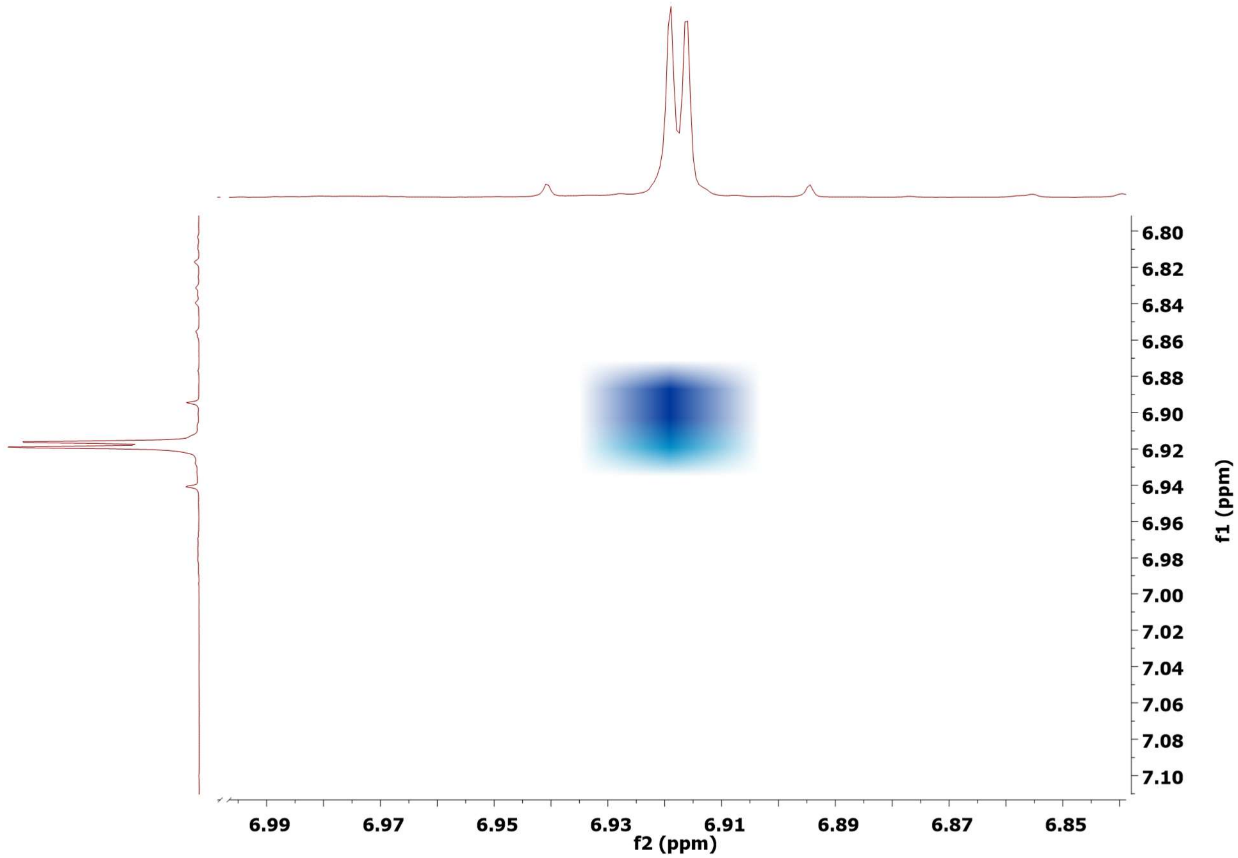

^{1}H-^{13}C-gHSQC NMR (DMSO-d6) spectrum of 4,7-dimethoxybenzo[d]thiazole-2-carbonitrile (3f)

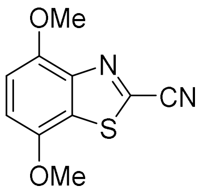

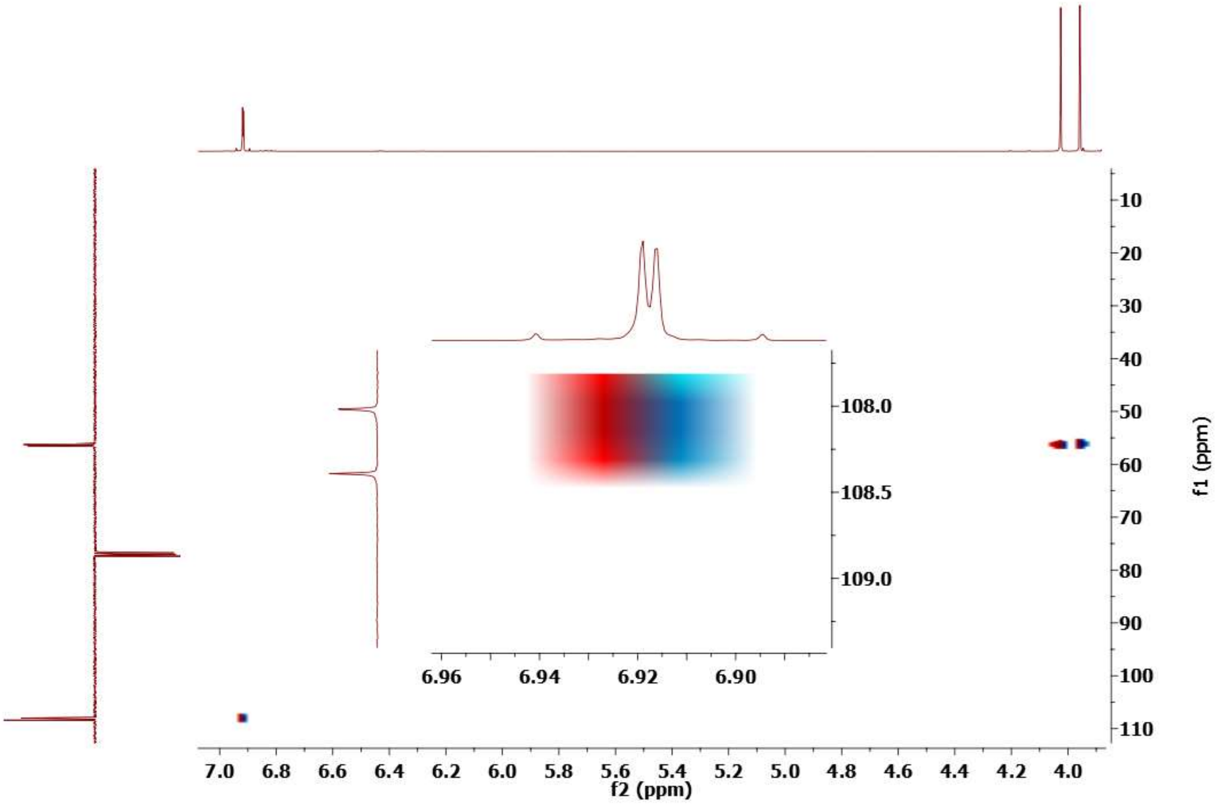

^{1}H NMR (DMSO-d6) spectrum of (5-iodo-2,4-dimethoxyphenyl)carbamoyl cyanide (3g)

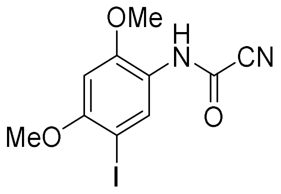

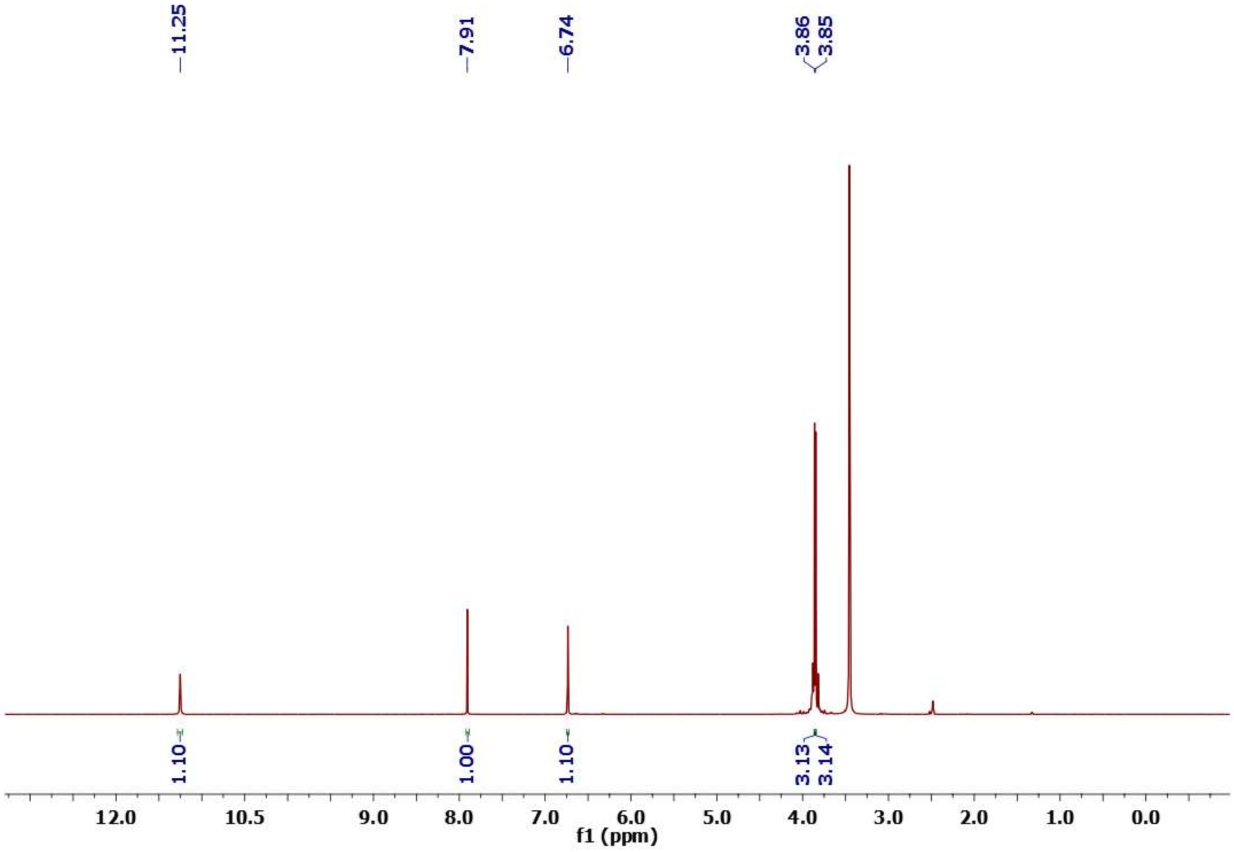

^{13}C NMR (DMSO-d6) spectrum of (5-iodo-2,4-dimethoxyphenyl)carbamoyl cyanide (3g)

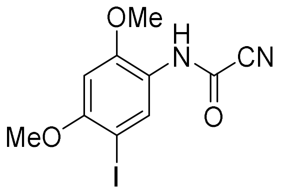

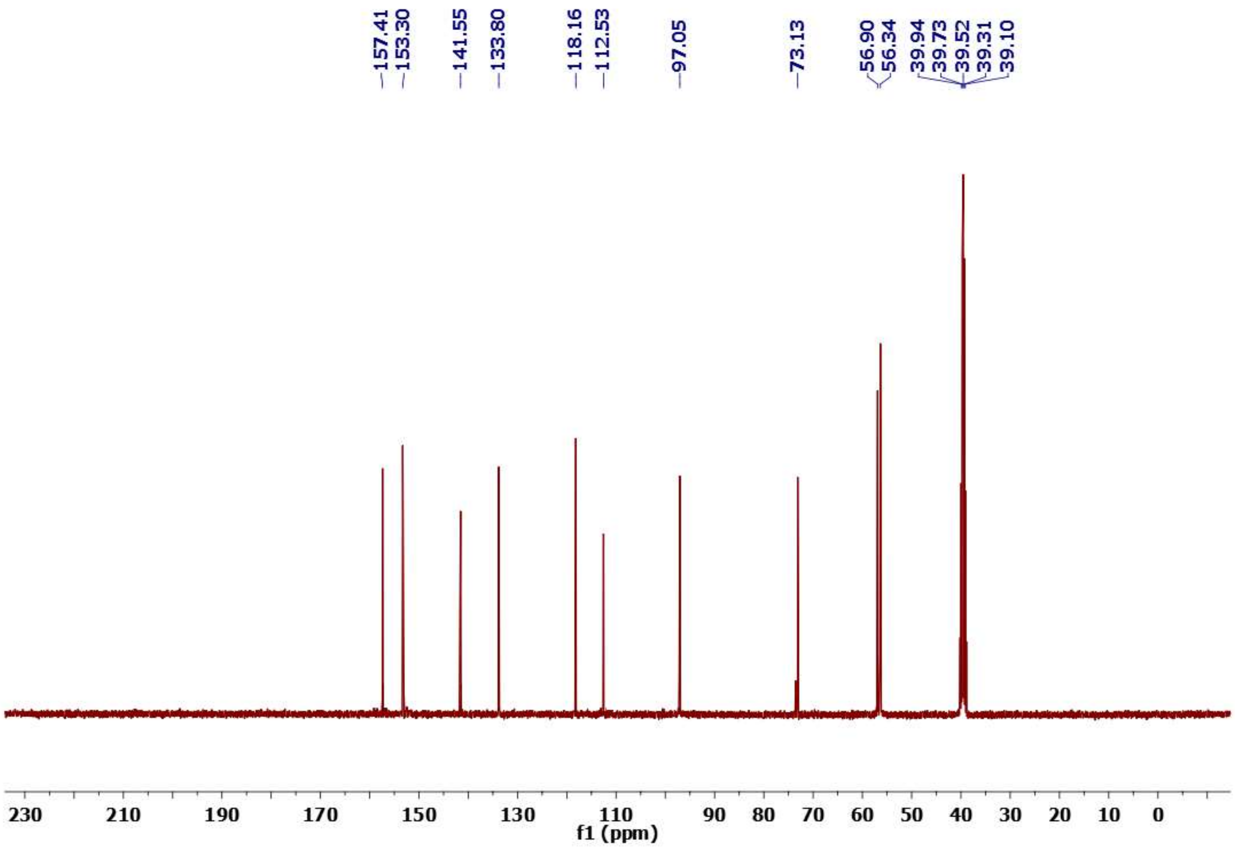

^{13}C CRAPT NMR (DMSO-d6) spectrum of (5-iodo-2,4-dimethoxyphenyl)carbamoyl cyanide (3g)

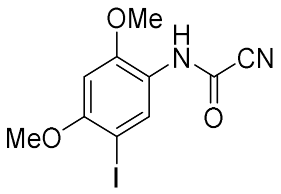

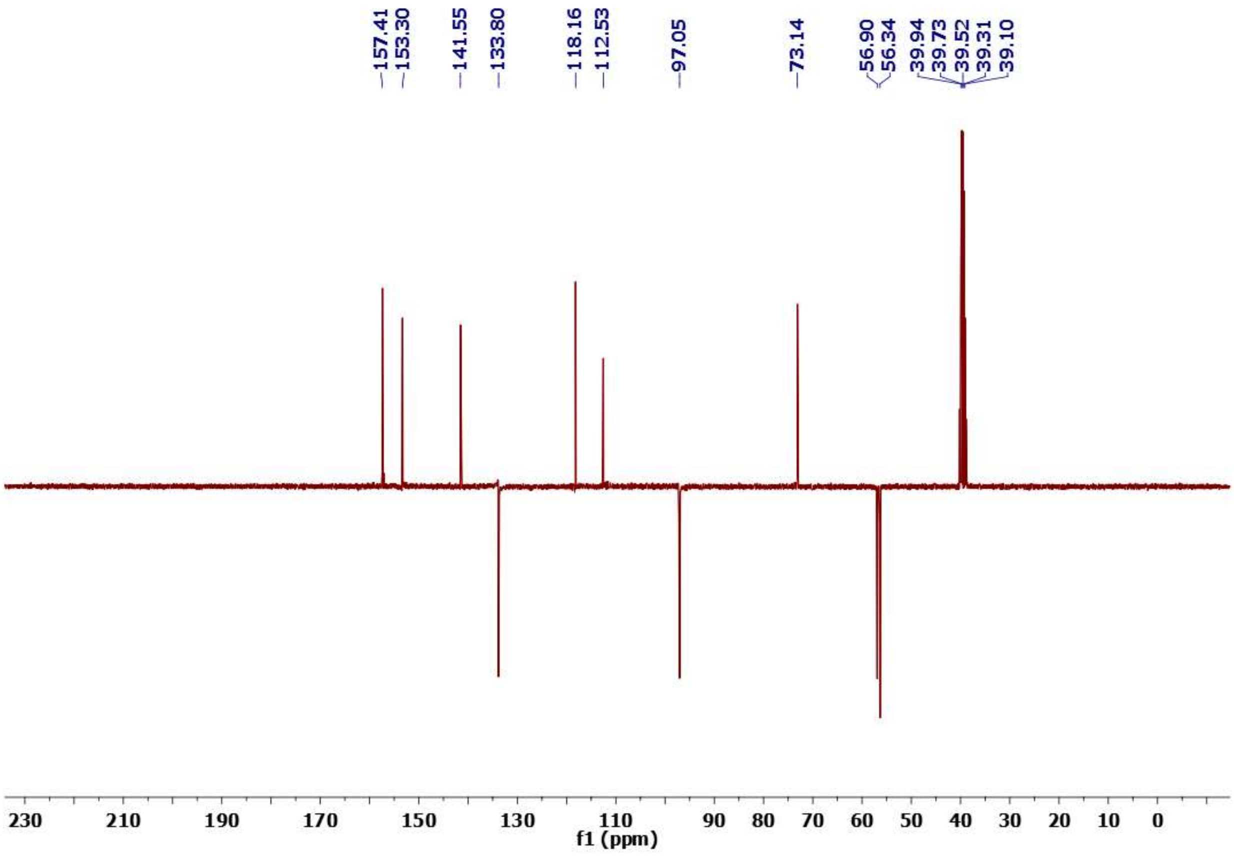

^{1}H-^{1}H-gDQCOSY NMR (DMSO-d6) spectrum of (5-iodo-2,4-dimethoxyphenyl)carbamoyl cyanide (3g)

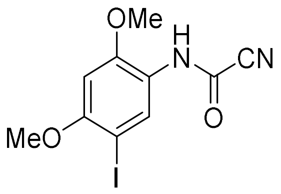

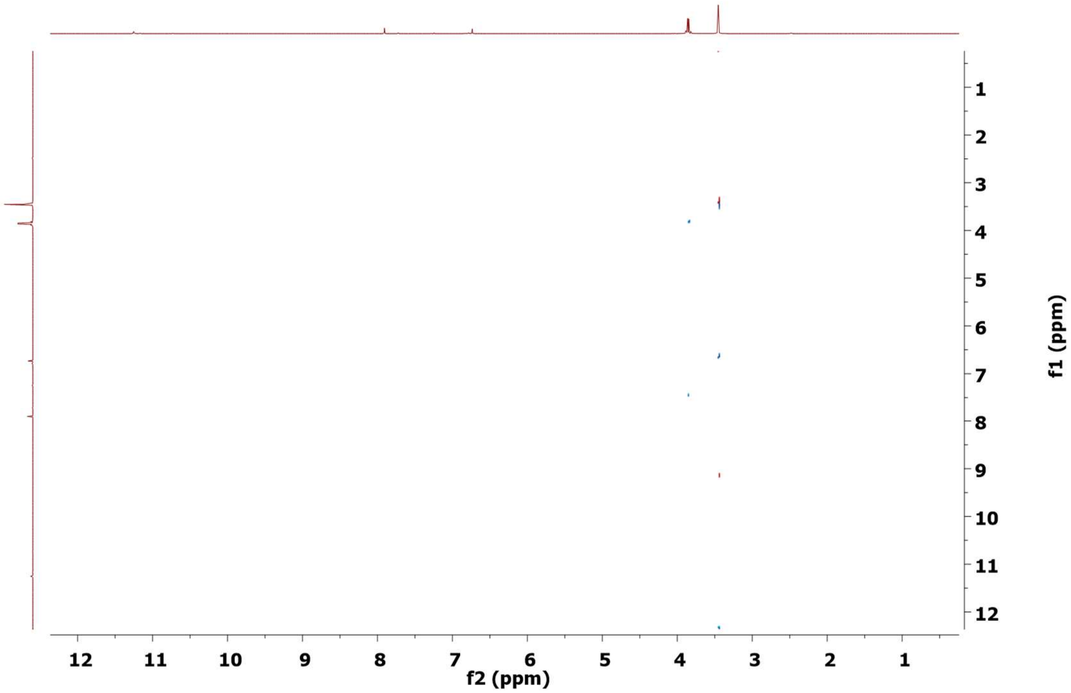

^{1}H-^{13}C-gHSQC NMR (DMSO-d6) spectrum of (5-iodo-2,4-dimethoxyphenyl)carbamoyl cyanide (3g)

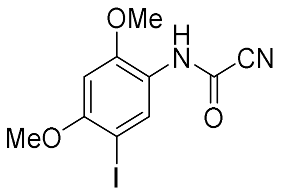

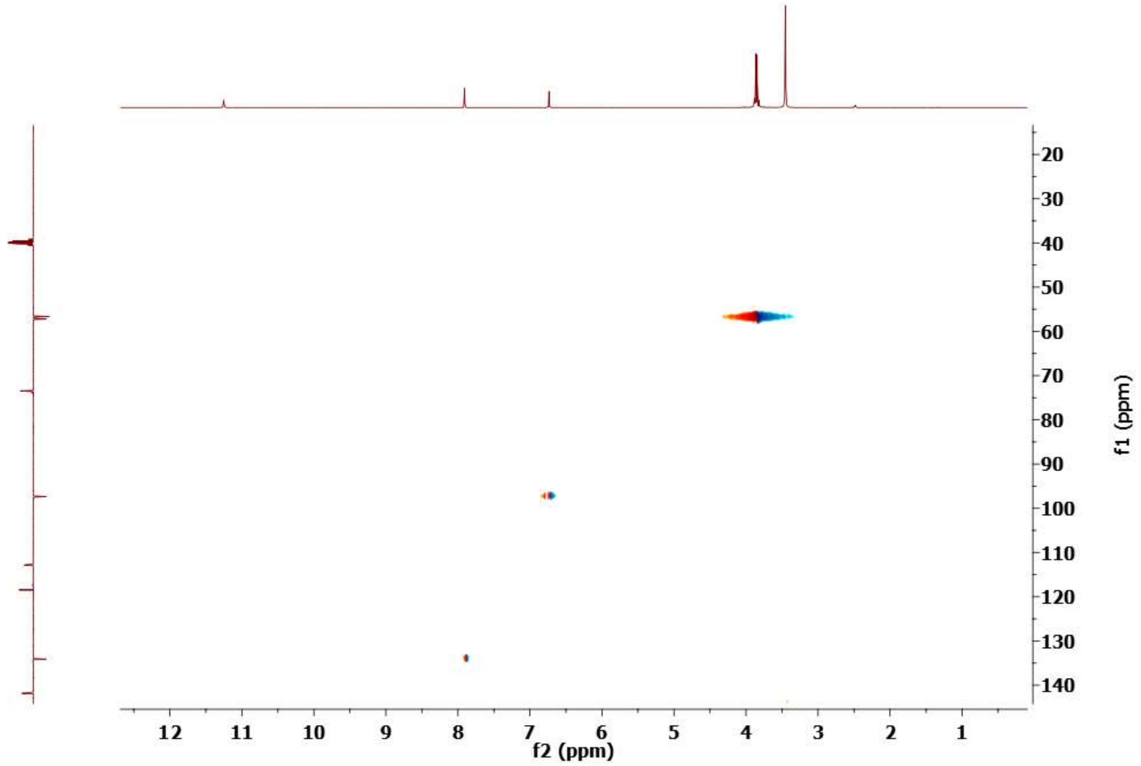

^{1}H-^{13}C-gHMBC NMR (DMSO-d6) spectrum of (5-iodo-2,4-dimethoxyphenyl)carbamoyl cyanide (3g)

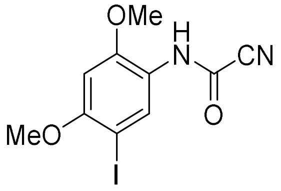

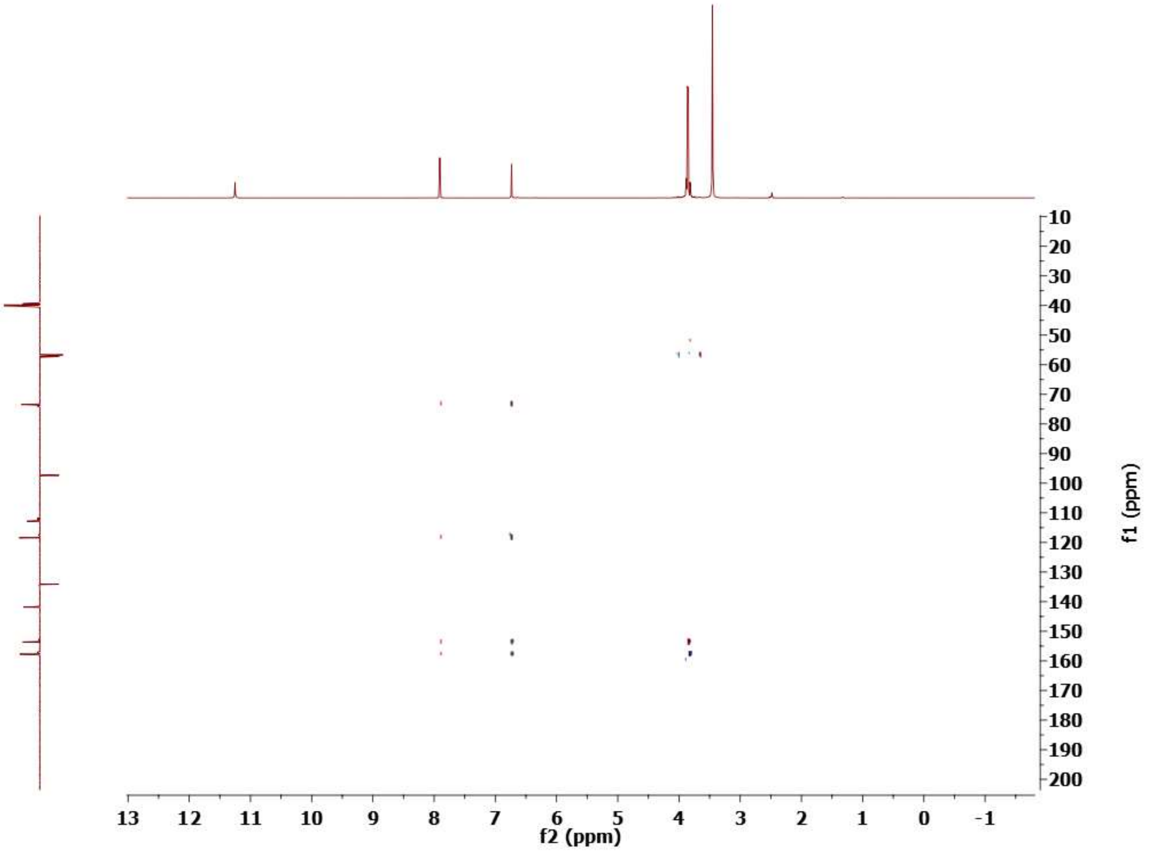

^{1}H NMR (DMSO-d6) spectrum of ethyl phenylcarbamate

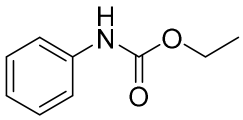

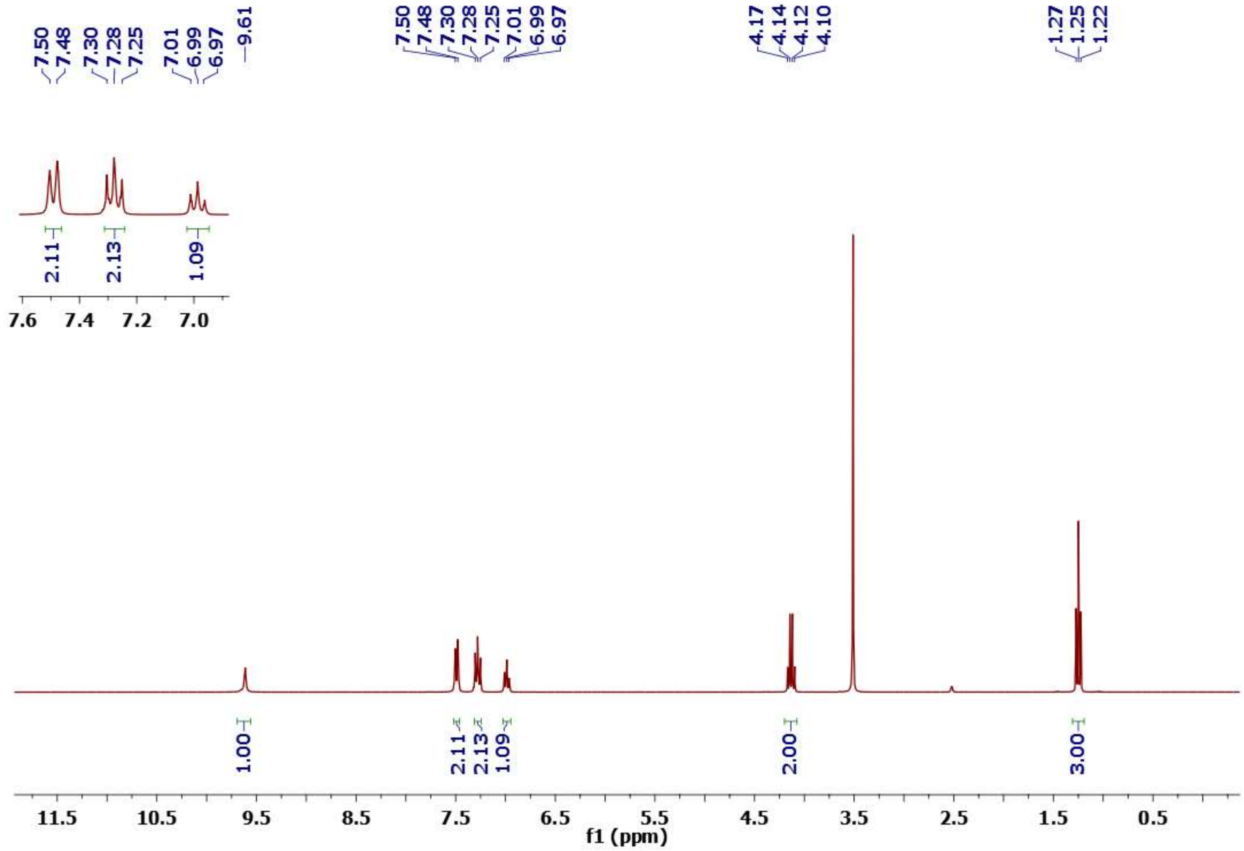

^{13}C NMR (DMSO-d6) spectrum of spectrum of ethyl phenylcarbamate

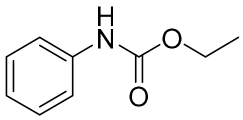

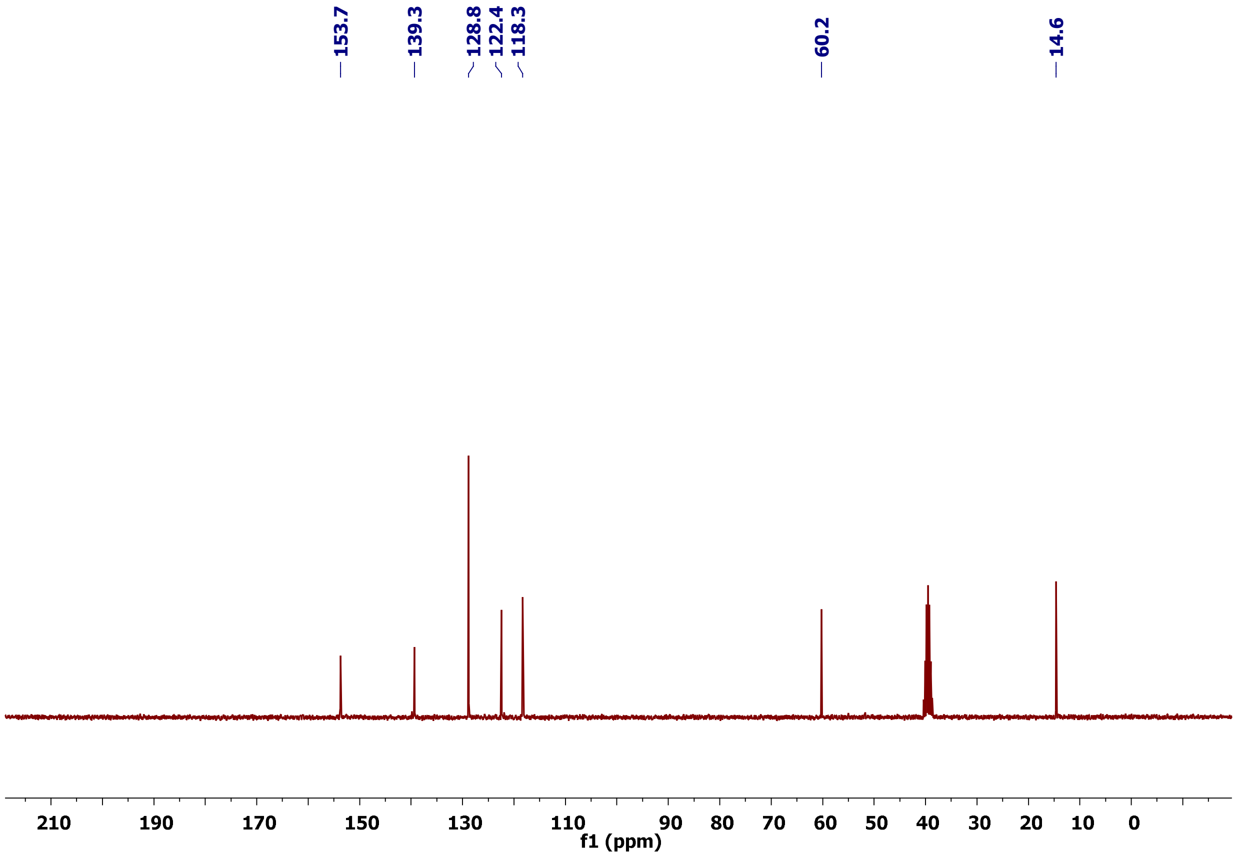

^{13}C DEPT-90 NMR (DMSO-d6) spectrum of ethyl phenylcarbamate

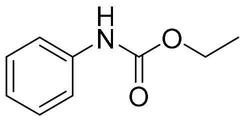

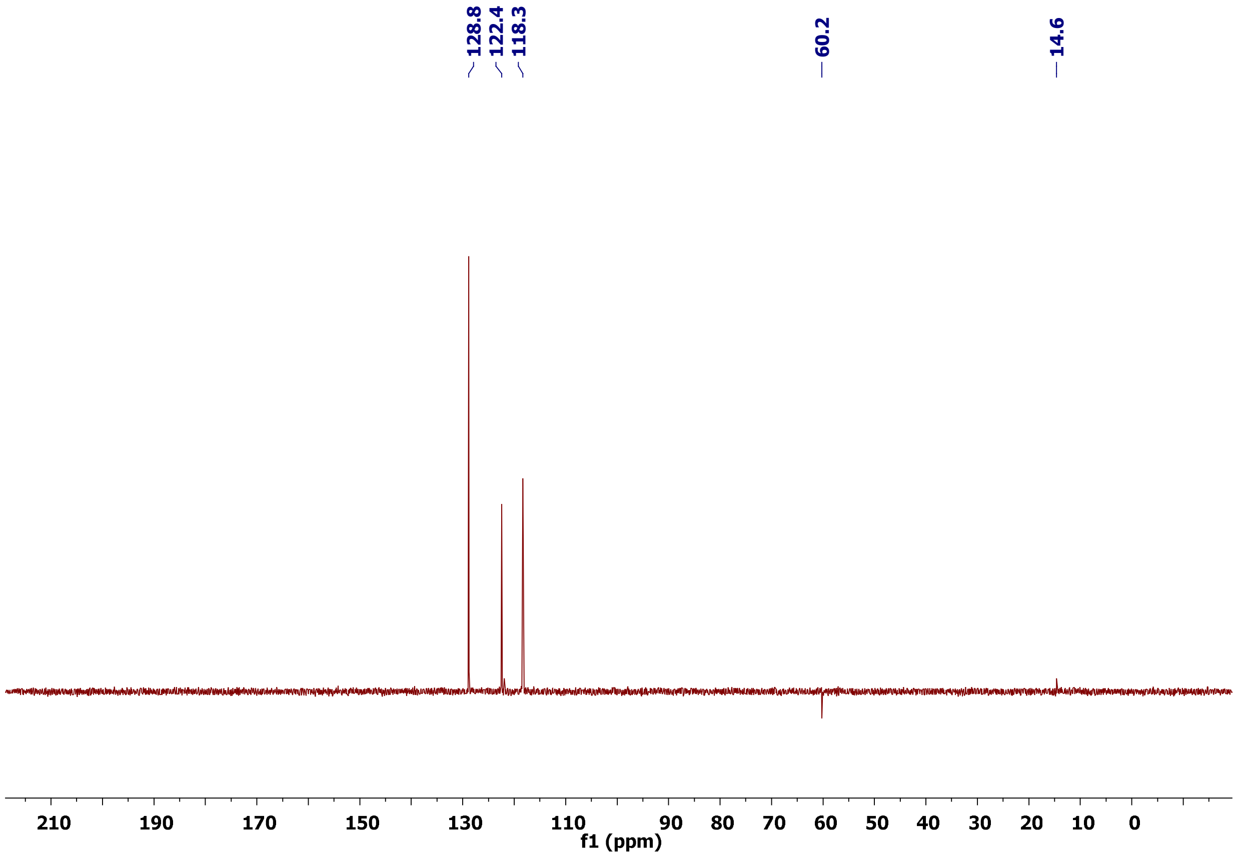

Single crystal X-ray diffraction data for compound 2g’

Table 1 Crystal data and structure refinement for exp_218_auto.

Identification code	exp_218_auto
Empirical formula	$C_8H_5BrN_2O$
Formula weight	225.05
Temperature/K	297.00(10)
Crystal system	triclinic
Space group	P-1
a/Å	8.65259(19)
b/Å	9.7551(2)
c/Å	10.0315(3)
α/°	82.229(2)
β/°	85.282(2)
γ/°	79.1919(19)
Volume/Å^3	822.66(3)
Z	4
ρ_{calc}g/cm^3	1.817
μ/mm^{-1}	6.405
F(000)	440.0
Crystal size/mm^3	0.182 × 0.1 × 0.075
Radiation	Cu Kα (λ = 1.54184)
2Θ range for data collection/°	8.912 to 154.918
Index ranges	-10 ≤ h ≤ 10, -11 ≤ k ≤ 12, -11 ≤ l ≤ 12
Reflections collected	14176
Independent reflections	3283 [R_{int} = 0.0349, R_{sigma} = 0.0237]
Data/restraints/parameters	3283/0/225
Goodness-of-fit on F^2	1.071
Final R indexes [I>=2σ (I)]	R_1 = 0.0301, wR_2 = 0.0786
Final R indexes [all data]	R_1 = 0.0333, wR_2 = 0.0807
Largest diff. peak/hole / e Å^{-3}	0.35/-0.50

Table 2 Fractional Atomic Coordinates (×10^4) and Equivalent Isotropic Displacement Parameters (Å^2×10^3) for exp_218_auto. U_{eq} is defined as 1/3 of the trace of the orthogonalised U_{IJ} tensor.

Atom	*x*	*y*	*z*	U(eq)
Br11	614.4(3)	6188.0(3)	12038.4(3)	53.32(11)
Br1	11432.9(4)	1225.3(3)	961.3(3)	56.64(11)

Table 2 Fractional Atomic Coordinates (×10⁴) and Equivalent Isotropic Displacement Parameters (Å²×10³) for exp_218_auto. U_{eq} is defined as 1/3 of the trace of the orthogonalised U_{IJ} tensor.

Atom	*x*	*y*	*z*	U(eq)
O11	5706(2)	4262.8(19)	6452.0(19)	53.6(4)
O1	6050(2)	-793.6(18)	6233.2(19)	54.7(5)
N13	5277(3)	6435(2)	7185(2)	44.3(5)
N2	6753(3)	1386(2)	5814(2)	45.2(5)
C12	5936(3)	5468(3)	6386(3)	44.2(5)
N1	4045(3)	1279(3)	8460(3)	73.8(8)
C17	2091(3)	6213(3)	10522(3)	42.3(5)
C14	4199(3)	6297(2)	8311(2)	40.4(5)
C6	9971(3)	1234(3)	2486(3)	44.4(5)
C3	7851(3)	1259(2)	4690(2)	40.7(5)
N11	7917(4)	6332(3)	4492(3)	72.4(8)
C2	5968(3)	422(3)	6464(3)	44.8(5)
C18	2413(3)	5105(3)	9778(3)	49.4(6)
C11	7038(3)	5995(3)	5327(3)	51.7(6)
C1	4892(3)	944(3)	7585(3)	53.2(6)
C5	9667(3)	2381(3)	3184(3)	53.2(6)
C7	9245(3)	89(3)	2889(3)	50.5(6)
C4	8609(3)	2399(3)	4287(3)	51.3(6)
C19	3467(3)	5134(3)	8664(3)	48.7(6)
C8	8184(3)	97(3)	4002(3)	48.9(6)
C16	2812(3)	7372(3)	10187(3)	52.4(6)
C15	3868(3)	7402(3)	9082(3)	51.0(6)

Table 3 Anisotropic Displacement Parameters (Å²×10³) for exp_218_auto. The Anisotropic displacement factor exponent takes the form: $-2\pi^2[h^2a^{*2}U_{11}+2hka^*b^*U_{12}+\ldots]$.

Atom	U_{11}	U_{22}	U_{33}	U_{23}	U_{13}	U_{12}
Br11	55.58(19)	52.91(18)	49.10(18)	-4.28(12)	11.98(13)	-11.57(13)
Br1	60.1(2)	53.66(18)	50.56(19)	-2.87(13)	14.71(13)	-6.09(13)
O11	63.7(11)	37.8(9)	60.6(11)	-14.8(8)	13.2(9)	-13.4(8)
O1	69.1(12)	38.0(9)	58.4(11)	-5.4(8)	12.3(9)	-20.3(8)
N13	51.8(12)	32.9(10)	48.3(12)	-6.3(9)	11.2(9)	-13.0(9)
N2	54.1(12)	31.7(10)	49.6(12)	-8.0(9)	10.1(10)	-10.9(9)
C12	46.2(13)	40.5(12)	46.1(13)	-8.0(10)	5.0(11)	-9.5(10)
N1	77.4(19)	77(2)	68.4(19)	-20.4(16)	30.9(16)	-24.9(16)
C17	43.5(13)	38.8(12)	42.5(13)	-2.3(10)	2.5(10)	-6.3(10)
C14	44.7(13)	33.1(11)	42.6(13)	-3.0(10)	2.9(10)	-8.6(9)

Table 3 Anisotropic Displacement Parameters (Å^2×10^3) for exp_218_auto. The Anisotropic displacement factor exponent takes the form: $-2\pi^2[h^2a^{*2}U_{11}+2hka^*b^*U_{12}+\ldots]$.

Atom	U_{11}	U_{22}	U_{33}	U_{23}	U_{13}	U_{12}
C6	46.4(13)	40.2(12)	43.5(13)	-0.9(10)	4.0(10)	-6.0(10)
C3	46.1(13)	34.9(11)	40.9(13)	-2.9(10)	3.2(10)	-10.3(10)
N11	82.9(19)	71.6(18)	63.7(16)	-15.2(14)	26.5(15)	-24.3(15)
C2	48.6(14)	41.7(13)	43.8(13)	-3.9(10)	5.6(11)	-11.8(10)
C18	57.6(15)	39.2(12)	52.8(15)	-5.4(11)	9.1(12)	-17.9(11)
C11	59.7(16)	44.6(14)	51.5(15)	-13.2(12)	10.1(13)	-11.6(12)
C1	58.4(16)	48.9(14)	54.6(16)	-5.8(12)	9.0(13)	-21.1(12)
C5	63.0(16)	37.7(12)	59.8(16)	-5.7(11)	14.5(13)	-19.0(11)
C7	59.8(16)	37.3(12)	53.8(15)	-11.2(11)	11.0(12)	-8.9(11)
C4	63.6(16)	34.7(12)	56.4(16)	-9.9(11)	15.1(13)	-16.0(11)
C19	62.5(16)	34.9(12)	50.9(14)	-10.1(10)	10.3(12)	-16.5(11)
C8	56.2(15)	34.3(12)	56.8(15)	-7.6(11)	7.3(12)	-13.0(10)
C16	63.2(16)	36.7(12)	57.9(16)	-14.6(11)	16.6(13)	-12.8(11)
C15	59.9(16)	33.2(12)	61.3(16)	-10.7(11)	14.1(13)	-16.4(11)

Table 4 Bond Lengths for exp_218_auto.

Atom	Atom	Length/Å	Atom	Atom	Length/Å
Br11	C17	1.905(2)	C14	C19	1.392(3)
Br1	C6	1.903(3)	C14	C15	1.384(3)
O11	C12	1.220(3)	C6	C5	1.372(4)
O1	C2	1.227(3)	C6	C7	1.381(4)
N13	C12	1.337(3)	C3	C4	1.393(3)
N13	C14	1.413(3)	C3	C8	1.379(3)
N2	C3	1.416(3)	N11	C11	1.141(4)
N2	C2	1.331(3)	C2	C1	1.481(4)
C12	C11	1.474(4)	C18	C19	1.384(4)
N1	C1	1.139(4)	C5	C4	1.377(4)
C17	C18	1.369(4)	C7	C8	1.385(4)
C17	C16	1.381(3)	C16	C15	1.378(4)

Table 5 Bond Angles for exp_218_auto.

Atom	Atom	Atom	Angle/°	Atom	Atom	Atom	Angle/°
C12	N13	C14	128.5(2)	C8	C3	N2	123.9(2)
C2	N2	C3	128.3(2)	C8	C3	C4	120.0(2)
O11	C12	N13	127.3(2)	O1	C2	N2	127.6(2)

Table 5 Bond Angles for exp_218_auto.

Atom	Atom	Atom	Angle/°	Atom	Atom	Atom	Angle/°
O11	C12	C11	120.0(2)	O1	C2	C1	119.3(2)
N13	C12	C11	112.8(2)	N2	C2	C1	113.2(2)
C18	C17	Br11	120.26(19)	C17	C18	C19	120.2(2)
C18	C17	C16	121.1(2)	N11	C11	C12	176.3(3)
C16	C17	Br11	118.69(19)	N1	C1	C2	176.5(3)
C19	C14	N13	123.7(2)	C6	C5	C4	119.7(2)
C15	C14	N13	116.7(2)	C6	C7	C8	119.9(2)
C15	C14	C19	119.6(2)	C5	C4	C3	120.1(2)
C5	C6	Br1	119.23(19)	C18	C19	C14	119.4(2)
C5	C6	C7	120.7(2)	C3	C8	C7	119.5(2)
C7	C6	Br1	120.04(19)	C15	C16	C17	118.9(2)
C4	C3	N2	116.1(2)	C16	C15	C14	120.9(2)

Table 6 Torsion Angles for exp_218_auto.

A	B	C	D	Angle/°	A	B	C	D	Angle/°
Br11	C17	C18	C19	179.0(2)	C6	C5	C4	C3	-0.1(5)
Br11	C17	C16	C15	-179.3(2)	C6	C7	C8	C3	0.6(4)
Br1	C6	C5	C4	179.9(2)	C3	N2	C2	O1	1.1(5)
Br1	C6	C7	C8	179.8(2)	C3	N2	C2	C1	-179.0(2)
N13	C14	C19	C18	-179.2(2)	C2	N2	C3	C4	-174.2(3)
N13	C14	C15	C16	178.9(3)	C2	N2	C3	C8	5.9(4)
N2	C3	C4	C5	-178.4(3)	C18	C17	C16	C15	0.1(4)
N2	C3	C8	C7	178.1(3)	C5	C6	C7	C8	0.6(4)
C12	N13	C14	C19	-9.4(4)	C7	C6	C5	C4	-0.9(4)
C12	N13	C14	C15	171.1(3)	C4	C3	C8	C7	-1.7(4)
C17	C18	C19	C14	0.2(4)	C19	C14	C15	C16	-0.6(4)
C17	C16	C15	C14	0.4(4)	C8	C3	C4	C5	1.4(4)
C14	N13	C12	O11	1.8(5)	C16	C17	C18	C19	-0.4(4)
C14	N13	C12	C11	-178.6(2)	C15	C14	C19	C18	0.3(4)

Table 7 Hydrogen Atom Coordinates (Å×10^4) and Isotropic Displacement Parameters (Å^2×10^3) for exp_218_auto.

Atom	*x*	*y*	*z*	U(eq)
H18	1920.86	4330.82	10021.18	59
H5	10172.21	3143.01	2913.63	64
H7	9467.3	-687.16	2414.14	61

Table 7 Hydrogen Atom Coordinates (Å×10^4) and Isotropic Displacement Parameters (Å^2×10^3) for exp_218_auto.

Atom	*x*	*y*	*z*	U(eq)
H4	8400.44	3173.65	4764.43	62
H19	3685.08	4383.51	8155.28	58
H8	7699.47	-676.32	4282.26	59
H16	2587.94	8120.18	10698.53	63
H15	4364.97	8175.22	8851.48	61
H2	6500(30)	2140(30)	6060(30)	47(8)
H13	5470(40)	7150(40)	7020(30)	60(10)

Single crystal X-ray diffraction data for compound 3c

Table 1 Crystal data and structure refinement for exp_229_auto.

Identification code	exp_229_auto
Empirical formula	$C_{15}H_{10}N_2OS$
Formula weight	266.31
Temperature/K	297(2)
Crystal system	triclinic
Space group	P-1
a/Å	7.0053(3)
b/Å	8.4517(3)
c/Å	11.6853(6)
α/°	86.836(3)
β/°	77.121(4)
γ/°	71.464(4)
Volume/Å^3	639.36(5)
Z	2
ρ_{calc}g/cm^3	1.383
μ/mm^{-1}	2.182
F(000)	276.0
Crystal size/mm^3	0.362 × 0.147 × 0.104
Radiation	CuKα (λ = 1.54184)
2Θ range for data collection/°	7.762 to 154.926
Index ranges	$-8 \le h \le 8$, $-10 \le k \le 6$, $-14 \le l \le 14$
Reflections collected	12094
Independent reflections	2576 [R_{int} = 0.0332, R_{sigma} = 0.0228]
Data/restraints/parameters	2576/0/172
Goodness-of-fit on F^2	1.062
Final R indexes [I>=2σ (I)]	R_1 = 0.0389, wR_2 = 0.1062
Final R indexes [all data]	R_1 = 0.0417, wR_2 = 0.1089
Largest diff. peak/hole / e Å^{-3}	0.14/-0.33

Table 2 Fractional Atomic Coordinates (×10^4) and Equivalent Isotropic Displacement Parameters (Å^2×10^3) for exp_229_auto. U_{eq} is defined as 1/3 of the trace of the orthogonalised U_{IJ} tensor.

Atom	*x*	*y*	*z*	U(eq)
S6	4117.6(7)	398.9(5)	2882.3(4)	58.01(16)
O10	-134.9(17)	4548.1(15)	7100.4(11)	61.1(3)
N4	5846(2)	1956.2(16)	3992.2(12)	51.0(3)
C12	2412(2)	4342.9(18)	8300.4(14)	49.8(3)
C3	3826(2)	2301.2(18)	4603.0(13)	45.7(3)
C7	2639(2)	1561.1(18)	4126.9(14)	47.9(3)
C1	926(2)	3567.3(19)	6132.6(14)	49.6(4)
C2	2971(2)	3314.9(19)	5612.3(14)	50.1(4)
C5	6163(3)	990.1(19)	3091.8(14)	52.0(4)
C8	570(3)	1821(2)	4661.4(16)	55.4(4)
C17	1846(3)	3213(2)	9101.5(15)	57.2(4)
C9	-250(2)	2806(2)	5651.0(16)	55.7(4)
C11	923(3)	5435(2)	7612.1(16)	58.6(4)
N19	9697(3)	148(3)	1650.5(18)	88.3(6)
C13	4363(3)	4479(2)	8173.9(16)	59.7(4)
C15	5126(3)	2409(2)	9617.2(17)	66.0(5)
C16	3202(3)	2249(2)	9752.3(16)	62.9(4)
C18	8133(3)	484(2)	2282.7(17)	62.0(4)
C14	5710(3)	3522(2)	8828.1(18)	69.4(5)

Table 3 Anisotropic Displacement Parameters (Å^2×10^3) for exp_229_auto. The Anisotropic displacement factor exponent takes the form: $-2\pi^2[h^2a^{*2}U_{11}+2hka^*b^*U_{12}+\ldots]$.

Atom	U_{11}	U_{22}	U_{33}	U_{23}	U_{13}	U_{12}
S6	65.2(3)	61.7(3)	55.8(3)	-2.07(18)	-19.4(2)	-27.0(2)
O10	47.7(6)	72.1(7)	59.2(7)	-4.0(6)	-8.6(5)	-14.1(5)
N4	47.9(7)	59.3(7)	49.3(7)	0.2(6)	-10.9(5)	-21.2(6)
C12	53.9(8)	46.7(7)	44.6(8)	-6.5(6)	-5.2(6)	-12.7(6)
C3	46.0(7)	49.2(8)	46.7(8)	8.3(6)	-16.2(6)	-18.9(6)
C7	54.0(8)	49.1(8)	49.3(8)	10.7(6)	-22.6(7)	-21.9(6)
C1	46.5(8)	52.8(8)	48.5(8)	8.2(6)	-13.9(6)	-13.2(6)
C2	47.8(8)	57.1(9)	51.0(9)	1.9(7)	-15.4(7)	-21.2(6)
C5	55.0(9)	54.1(8)	49.7(9)	4.3(7)	-13.6(7)	-20.0(7)
C8	52.8(8)	61.8(9)	62.9(10)	11.6(8)	-24.6(8)	-27.2(7)
C17	53.9(9)	57.8(9)	56.3(10)	-2.0(7)	-3.2(7)	-18.1(7)

Table 3 Anisotropic Displacement Parameters (Å^2×10^3) for exp_229_auto. The Anisotropic displacement factor exponent takes the form: $-2\pi^2[h^2a^{*2}U_{11}+2hka^*b^*U_{12}+\ldots]$.

Atom	U_{11}	U_{22}	U_{33}	U_{23}	U_{13}	U_{12}
C9	44.1(8)	64.9(9)	62.5(10)	14.4(8)	-17.3(7)	-21.1(7)
C11	60.5(10)	53.4(9)	55.9(10)	-3.7(7)	-10.7(8)	-10.1(7)
N19	74.2(12)	95.5(13)	87.0(13)	-19.8(10)	8.2(10)	-29.4(10)
C13	62.1(10)	61.5(9)	58.0(10)	4.1(7)	-9.0(8)	-25.9(8)
C15	68.2(11)	67.3(11)	57.3(10)	-1.1(8)	-20.3(9)	-9.3(8)
C16	73.1(11)	57.1(9)	51.6(10)	5.2(7)	-6.1(8)	-16.7(8)
C18	64.4(11)	64.2(10)	59.1(10)	-6.9(8)	-9.7(9)	-23.7(8)
C14	59.3(10)	81.8(12)	72.1(12)	-1.7(10)	-17.5(9)	-26.5(9)

Table 4 Bond Lengths for exp_229_auto.

Atom	Atom	Length/Å	Atom	Atom	Length/Å
S6	C7	1.7299(17)	C7	C8	1.396(2)
S6	C5	1.7282(16)	C1	C2	1.378(2)
O10	C1	1.362(2)	C1	C9	1.412(2)
O10	C11	1.438(2)	C5	C18	1.439(2)
N4	C3	1.381(2)	C8	C9	1.366(2)
N4	C5	1.303(2)	C17	C16	1.381(3)
C12	C17	1.387(2)	N19	C18	1.136(2)
C12	C11	1.506(2)	C13	C14	1.377(3)
C12	C13	1.382(2)	C15	C16	1.370(3)
C3	C7	1.403(2)	C15	C14	1.372(3)
C3	C2	1.398(2)			

Table 5 Bond Angles for exp_229_auto.

Atom	Atom	Atom	Angle/°	Atom	Atom	Atom	Angle/°
C5	S6	C7	87.96(7)	C2	C1	C9	120.18(15)
C1	O10	C11	118.23(13)	C1	C2	C3	118.25(14)
C5	N4	C3	109.38(13)	N4	C5	S6	117.73(13)
C17	C12	C11	121.03(15)	N4	C5	C18	120.76(15)
C13	C12	C17	118.28(15)	C18	C5	S6	121.48(13)
C13	C12	C11	120.65(15)	C9	C8	C7	118.27(15)
N4	C3	C7	114.75(14)	C16	C17	C12	120.65(16)
N4	C3	C2	124.14(13)	C8	C9	C1	121.96(15)
C2	C3	C7	121.11(14)	O10	C11	C12	113.64(13)
C3	C7	S6	110.18(12)	C14	C13	C12	120.95(16)

Table 5 Bond Angles for exp_229_auto.

Atom	Atom	Atom	Angle/°	Atom	Atom	Atom	Angle/°
C8	C7	S6	129.58(12)	C16	C15	C14	119.96(17)
C8	C7	C3	120.24(15)	C15	C16	C17	120.11(16)
O10	C1	C2	125.14(14)	N19	C18	C5	177.1(2)
O10	C1	C9	114.68(14)	C15	C14	C13	120.05(18)

Table 6 Torsion Angles for exp_229_auto.

A	B	C	D	Angle/°	A	B	C	D	Angle/°
S6	C7	C8	C9	-179.96(12)	C2	C3	C7	C8	-0.3(2)
O10	C1	C2	C3	-178.45(14)	C2	C1	C9	C8	-0.7(2)
O10	C1	C9	C8	178.20(14)	C5	S6	C7	C3	-0.18(11)
N4	C3	C7	S6	0.20(16)	C5	S6	C7	C8	179.73(15)
N4	C3	C7	C8	-179.72(13)	C5	N4	C3	C7	-0.10(18)
N4	C3	C2	C1	179.53(13)	C5	N4	C3	C2	-179.49(14)
C12	C17	C16	C15	0.5(3)	C17	C12	C11	O10	-46.7(2)
C12	C13	C14	C15	0.2(3)	C17	C12	C13	C14	-0.2(3)
C3	N4	C5	S6	-0.05(17)	C9	C1	C2	C3	0.3(2)
C3	N4	C5	C18	177.96(14)	C11	O10	C1	C2	1.8(2)
C3	C7	C8	C9	-0.1(2)	C11	O10	C1	C9	-177.03(13)
C7	S6	C5	N4	0.14(13)	C11	C12	C17	C16	-178.22(15)
C7	S6	C5	C18	-177.85(14)	C11	C12	C13	C14	177.92(16)
C7	C3	C2	C1	0.2(2)	C13	C12	C17	C16	-0.1(2)
C7	C8	C9	C1	0.5(2)	C13	C12	C11	O10	135.24(16)
C1	O10	C11	C12	-75.38(18)	C16	C15	C14	C13	0.2(3)
C2	C3	C7	S6	179.61(11)	C14	C15	C16	C17	-0.5(3)

Table 7 Hydrogen Atom Coordinates (Å×10^4) and Isotropic Displacement Parameters (Å^2×10^3) for exp_229_auto.

Atom	*x*	*y*	*z*	U(eq)
H2	3761.29	3806.14	5924.34	60
H8	-230.04	1336.51	4351.94	66
H17	539.35	3103.3	9201.47	69

Table 7 Hydrogen Atom Coordinates (Å×10^4) and Isotropic Displacement Parameters (Å^2×10^3) for exp_229_auto.

Atom	*x*	*y*	*z*	U(eq)
H9	-1624.01	2980.28	6018.48	67
H11A	-89.81	6313.02	8127.4	70
H11B	1670.88	5959.76	6990.82	70
H13	4772.47	5228.09	7639.33	72
H15	6034.08	1765.1	10060.14	79
H16	2810.24	1488.7	10283.09	75
H14	7016.16	3629.23	8734.82	83

Single crystal X-ray diffraction data for compound 3f

Table 1 Crystal data and structure refinement for exp_240_auto.

Identification code	exp_240_auto
Empirical formula	$C_{10}H_8N_2O_2S$
Formula weight	220.24
Temperature/K	297.00(10)
Crystal system	orthorhombic
Space group	$Pna2_1$
a/Å	13.7218(16)
b/Å	18.260(2)
c/Å	3.9580(7)
α/°	90
β/°	90
γ/°	90
Volume/Å^3	991.7(2)
Z	4
ρ_{calc}g/cm^3	1.475
μ/mm^{-1}	2.754
F(000)	456.0
Crystal size/mm^3	0.254 × 0.135 × 0.048
Radiation	Cu Kα (λ = 1.54184)
2Θ range for data collection/°	8.06 to 153.842
Index ranges	$-16 \leq h \leq 12$, $-20 \leq k \leq 23$, $-4 \leq l \leq 4$
Reflections collected	4966
Independent reflections	1696 [R_{int} = 0.0826, R_{sigma} = 0.0595]
Data/restraints/parameters	1696/1/138
Goodness-of-fit on F^2	1.122
Final R indexes [I>=2σ (I)]	R_1 = 0.0704, wR_2 = 0.1981
Final R indexes [all data]	R_1 = 0.0894, wR_2 = 0.2219
Largest diff. peak/hole / e Å^{-3}	0.47/-0.39

Flack parameter -0.02(7)

Table 2 Fractional Atomic Coordinates (×10⁴) and Equivalent Isotropic Displacement Parameters (Å²×10³) for exp_240_auto. U_{eq} is defined as 1/3 of the trace of the orthogonalised U_{IJ} tensor.

Atom	*x*	*y*	*z*	U(eq)
S8	-5544.4(13)	-5766.4(10)	-5385(7)	64.9(6)
O12	-5709(4)	-8449(3)	-6091(19)	72(2)
O10	-3605(4)	-6015(3)	-1870(20)	73.1(16)
N6	-6390(4)	-7001(3)	-7040(20)	58.9(16)
C5	-5512(5)	-7171(4)	-5520(30)	55.6(16)
C1	-4051(6)	-6653(5)	-2810(20)	61(2)
C9	-4948(6)	-6577(4)	-4430(20)	60(2)
C7	-6464(5)	-6293(4)	-7120(20)	57.0(18)
C4	-5145(6)	-7891(4)	-5040(30)	66(2)
N15	-7934(6)	-5635(4)	-9840(30)	89(3)
C3	-4258(6)	-7959(5)	-3400(30)	70(2)
C2	-3723(6)	-7348(5)	-2370(30)	70(2)
C14	-7284(7)	-5933(5)	-8590(30)	69(2)
C13	-5382(7)	-9169(4)	-5620(40)	79(2)
C11	-2685(6)	-6085(5)	-270(30)	82(2)

Table 3 Anisotropic Displacement Parameters (Å²×10³) for exp_240_auto. The Anisotropic displacement factor exponent takes the form: $-2\pi^2[h^2a^{*2}U_{11}+2hka^*b^*U_{12}+\ldots]$.

Atom	U_{11}	U_{22}	U_{33}	U_{23}	U_{13}	U_{12}
S8	61.0(11)	61.4(10)	72.2(13)	-0.9(13)	-7.3(13)	-1.3(8)
O12	68(3)	55(3)	93(6)	2(3)	-10(3)	-1(2)
O10	59(3)	74(3)	86(4)	-4(3)	-14(3)	-2(3)
N6	49(3)	66(4)	62(4)	-1(3)	1(3)	0(3)
C5	50(4)	65(4)	52(4)	-4(5)	-3(4)	-4(3)
C1	51(4)	72(5)	60(5)	0(4)	-1(4)	1(4)
C9	62(5)	59(4)	60(5)	2(3)	-1(4)	2(3)
C7	45(4)	66(4)	59(5)	-6(4)	-7(3)	2(3)
C4	71(5)	58(4)	69(6)	1(5)	13(5)	-7(3)
N15	74(5)	73(4)	119(9)	5(5)	-17(6)	4(3)
C3	64(5)	70(5)	75(6)	9(5)	-6(5)	10(4)
C2	59(5)	77(5)	74(6)	4(5)	-9(5)	2(4)
C14	62(5)	67(5)	78(6)	0(4)	1(5)	1(4)

Table 3 Anisotropic Displacement Parameters (Å^2×10^3) for exp_240_auto. The Anisotropic displacement factor exponent takes the form: $-2\pi^2[h^2a^{*2}U_{11}+2hka^*b^*U_{12}+\ldots]$.

Atom	U_{11}	U_{22}	U_{33}	U_{23}	U_{13}	U_{12}
C13	79(5)	62(4)	96(7)	12(6)	-5(7)	-3(4)
C11	62(5)	103(6)	80(6)	-2(7)	-19(6)	-17(4)

Table 4 Bond Lengths for exp_240_auto.

Atom	Atom	Length/Å	Atom	Atom	Length/Å
S8	C9	1.732(8)	C5	C9	1.400(11)
S8	C7	1.729(7)	C5	C4	1.421(11)
O12	C4	1.345(10)	C1	C9	1.395(12)
O12	C13	1.403(9)	C1	C2	1.357(11)
O10	C1	1.369(10)	C7	C14	1.427(12)
O10	C11	1.418(10)	C4	C3	1.385(13)
N6	C5	1.381(10)	N15	C14	1.156(12)
N6	C7	1.298(9)	C3	C2	1.396(13)

Table 5 Bond Angles for exp_240_auto.

Atom	Atom	Atom	Angle/°	Atom	Atom	Atom	Angle/°
C7	S8	C9	87.5(4)	C1	C9	S8	127.1(6)
C4	O12	C13	119.0(7)	C1	C9	C5	123.4(7)
C1	O10	C11	116.2(7)	N6	C7	S8	119.1(5)
C7	N6	C5	107.6(6)	N6	C7	C14	122.1(7)
N6	C5	C9	116.2(7)	C14	C7	S8	118.8(6)
N6	C5	C4	125.0(7)	O12	C4	C5	117.1(8)
C9	C5	C4	118.7(7)	O12	C4	C3	125.7(7)
O10	C1	C9	115.7(7)	C3	C4	C5	117.2(7)
C2	C1	O10	127.8(8)	C4	C3	C2	121.8(8)
C2	C1	C9	116.5(8)	C1	C2	C3	122.3(8)
C5	C9	S8	109.5(6)	N15	C14	C7	178.3(11)

Table 6 Torsion Angles for exp_240_auto.

A	B	C	D	Angle/°	A	B	C	D	Angle/°
O12	C4	C3	C2	-179.5(10)	C9	C1	C2	C3	-1.5(14)
O10	C1	C9	S8	0.4(13)	C7	S8	C9	C5	-0.3(7)
O10	C1	C9	C5	-178.9(9)	C7	S8	C9	C1	-179.7(8)

Table 6 Torsion Angles for exp_240_auto.

A	B	C	D	Angle/°	A	B	C	D	Angle/°
O10	C1	C2	C3	178.8(10)	C7	N6	C5	C9	1.0(11)
N6	C5	C9	S8	-0.3(11)	C7	N6	C5	C4	-178.0(9)
N6	C5	C9	C1	179.1(9)	C4	C5	C9	S8	178.7(8)
N6	C5	C4	O12	-1.5(15)	C4	C5	C9	C1	-1.8(14)
N6	C5	C4	C3	-178.7(10)	C4	C3	C2	C1	2.2(17)
C5	N6	C7	S8	-1.3(10)	C2	C1	C9	S8	-179.3(8)
C5	N6	C7	C14	179.0(9)	C2	C1	C9	C5	1.4(13)
C5	C4	C3	C2	-2.5(15)	C13	O12	C4	C5	-179.0(10)
C9	S8	C7	N6	1.0(8)	C13	O12	C4	C3	-2.0(17)
C9	S8	C7	C14	-179.3(8)	C11	O10	C1	C9	-179.0(8)
C9	C5	C4	O12	179.6(8)	C11	O10	C1	C2	0.7(15)
C9	C5	C4	C3	2.3(13)					

Table 7 Hydrogen Atom Coordinates (Å×10^4) and Isotropic Displacement Parameters (Å^2×10^3) for exp_240_auto.

Atom	*x*	*y*	*z*	U(eq)
H3	-4011.94	-8424.26	-2972.3	84
H2	-3120.22	-7419.31	-1348.5	84
H13A	-5304.79	-9263.39	-3246.69	119
H13B	-4766.96	-9233.06	-6738.32	119
H13C	-5849.31	-9504.71	-6545.85	119
H11A	-2767.8	-6309.01	1902.21	123
H11B	-2397.62	-5609.38	-1.17	123
H11C	-2266.19	-6384.81	-1639.13	123

Single crystal X-ray diffraction data for compound 3e

Table 1 Crystal data and structure refinement for exp_235_auto.

Identification code	exp_235_auto
Empirical formula	$C_{10}H_8N_2O_2S$
Formula weight	220.24
Temperature/K	297.00(10)
Crystal system	monoclinic
Space group	$P2_1/n$
a/Å	3.9979(2)
b/Å	15.5449(11)
c/Å	15.9729(8)

α/°	90
β/°	90.509(4)
γ/°	90
Volume/Å^3	992.63(10)
Z	4
ρ_{calc}g/cm^3	1.474
μ/mm^{-1}	2.752
F(000)	456.0
Crystal size/mm^3	0.278 × 0.031 × 0.026
Radiation	Cu Kα (λ = 1.54184)
2Θ range for data collection/°	11.08 to 154.728
Index ranges	-4 ≤ h ≤ 2, -18 ≤ k ≤ 18, -20 ≤ l ≤ 19
Reflections collected	7605
Independent reflections	1900 [R_{int} = 0.0609, R_{sigma} = 0.0368]
Data/restraints/parameters	1900/0/138
Goodness-of-fit on F^2	1.063
Final R indexes [I>=2σ (I)]	R_1 = 0.0477, wR_2 = 0.1276
Final R indexes [all data]	R_1 = 0.0607, wR_2 = 0.1349
Largest diff. peak/hole / e Å^{-3}	0.25/-0.23

Table 2 Fractional Atomic Coordinates (×10^4) and Equivalent Isotropic Displacement Parameters (Å^2×10^3) for exp_235_auto. U_{eq} is defined as 1/3 of the trace of the orthogonalised U_{IJ} tensor.

Atom	*x*	*y*	*z*	U(eq)
S5	3631.7(15)	3123.8(4)	7320.9(4)	56.6(2)
O10	4994(4)	3085.4(11)	3691.2(10)	58.5(5)
O12	7434(5)	1807.8(11)	4466.0(11)	59.8(5)
N7	1504(5)	4352.3(14)	6339.0(13)	52.8(5)
C8	2953(5)	3739.6(15)	5833.1(14)	47.8(5)
C1	4629(6)	3127.8(15)	4533.9(15)	48.4(5)
C4	4284(6)	3024.1(16)	6257.1(15)	49.1(5)
C2	6012(5)	2405.7(16)	4973.8(15)	49.0(6)
C3	5815(5)	2345.7(16)	5827.7(15)	50.4(6)
C9	3124(6)	3790.0(15)	4958.9(15)	49.3(5)
C6	1722(6)	4104.1(17)	7117.1(15)	53.4(6)
N15	-474(8)	5052.9(19)	8316.1(16)	83.0(8)
C14	490(7)	4626.6(19)	7791.9(17)	62.6(7)
C11	3420(7)	3745.7(19)	3205.8(16)	59.9(6)
C13	8818(7)	1059.1(18)	4855.4(18)	60.5(7)

Table 3 Anisotropic Displacement Parameters (Å^2×10^3) for exp_235_auto. The Anisotropic displacement factor exponent takes the form: $-2\pi^2[h^2a^{*2}U_{11}+2hka^*b^*U_{12}+\ldots]$.

Atom	U_{11}	U_{22}	U_{33}	U_{23}	U_{13}	U_{12}
S5	65.1(4)	55.4(4)	49.3(4)	3.7(3)	8.7(2)	3.5(3)
O10	75.0(11)	54.1(11)	46.6(9)	1.6(8)	7.2(8)	12.3(8)
O12	75.3(11)	47.0(10)	57.3(10)	-1.0(8)	10.9(8)	13.2(8)
N7	57.6(11)	48.5(12)	52.3(11)	-3.4(9)	8.6(8)	1.9(9)
C8	49.2(12)	41.4(13)	53.1(13)	-2.9(10)	6.2(9)	-2.2(9)
C1	53.6(12)	45.5(13)	46.1(12)	0.4(10)	5.9(9)	-2.1(10)
C4	48.7(11)	46.4(13)	52.2(13)	0.8(10)	5.9(9)	-3.5(10)
C2	50.8(12)	41.7(13)	54.5(14)	-1.0(10)	6.6(9)	1.6(10)
C3	54.6(13)	43.8(14)	53.0(13)	3.3(10)	4.5(10)	2.7(10)
C9	56.0(12)	40.4(13)	51.6(13)	2.2(10)	5.0(9)	2.8(10)
C6	57.0(13)	53.2(15)	50.1(13)	-4.5(11)	9.1(10)	-0.4(11)
N15	110.6(19)	80.9(19)	57.6(15)	-4.2(13)	14.8(13)	23.3(16)
C14	71.7(16)	62.0(18)	54.2(14)	0.1(13)	8.0(12)	6.5(14)
C11	70.7(15)	57.3(17)	51.8(13)	4.6(12)	1.0(11)	4.0(12)
C13	64.7(15)	49.0(15)	67.8(16)	-1.4(12)	3.6(11)	13.2(12)

Table 4 Bond Lengths for exp_235_auto.

Atom	Atom	Length/Å	Atom	Atom	Length/Å
S5	C4	1.728(2)	C8	C4	1.405(3)
S5	C6	1.734(3)	C8	C9	1.401(3)
O10	C1	1.357(3)	C1	C2	1.433(3)
O10	C11	1.429(3)	C1	C9	1.375(3)
O12	C2	1.361(3)	C4	C3	1.402(3)
O12	C13	1.429(3)	C2	C3	1.370(3)
N7	C8	1.380(3)	C6	C14	1.440(4)
N7	C6	1.303(3)	N15	C14	1.138(4)

Table 5 Bond Angles for exp_235_auto.

Atom	Atom	Atom	Angle/°	Atom	Atom	Atom	Angle/°
C4	S5	C6	87.95(12)	C3	C4	S5	128.2(2)
C1	O10	C11	116.92(18)	C3	C4	C8	121.7(2)
C2	O12	C13	117.3(2)	O12	C2	C1	113.8(2)
C6	N7	C8	109.2(2)	O12	C2	C3	125.1(2)
N7	C8	C4	115.1(2)	C3	C2	C1	121.1(2)
N7	C8	C9	124.7(2)	C2	C3	C4	117.7(2)

Table 5 Bond Angles for exp_235_auto.

Atom	Atom	Atom	Angle/°	Atom	Atom	Atom	Angle/°
C9	C8	C4	120.2(2)	C1	C9	C8	118.4(2)
O10	C1	C2	113.84(19)	N7	C6	S5	117.69(18)
O10	C1	C9	125.3(2)	N7	C6	C14	121.8(2)
C9	C1	C2	120.9(2)	C14	C6	S5	120.5(2)
C8	C4	S5	110.07(17)	N15	C14	C6	178.7(3)

Table 6 Torsion Angles for exp_235_auto.

A	B	C	D	Angle/°	A	B	C	D	Angle/°
S5	C4	C3	C2	179.20(18)	C4	C8	C9	C1	-0.3(3)
O10	C1	C2	O12	-0.1(3)	C2	C1	C9	C8	-0.4(3)
O10	C1	C2	C3	-179.2(2)	C9	C8	C4	S5	-178.58(18)
O10	C1	C9	C8	-179.8(2)	C9	C8	C4	C3	0.2(3)
O12	C2	C3	C4	179.6(2)	C9	C1	C2	O12	-179.6(2)
N7	C8	C4	S5	0.8(3)	C9	C1	C2	C3	1.3(4)
N7	C8	C4	C3	179.6(2)	C6	S5	C4	C8	-0.81(18)
N7	C8	C9	C1	-179.6(2)	C6	S5	C4	C3	-179.4(2)
C8	N7	C6	S5	-0.4(3)	C6	N7	C8	C4	-0.3(3)
C8	N7	C6	C14	178.4(2)	C6	N7	C8	C9	179.1(2)
C8	C4	C3	C2	0.7(3)	C11	O10	C1	C2	174.7(2)
C1	C2	C3	C4	-1.4(3)	C11	O10	C1	C9	-5.9(3)
C4	S5	C6	N7	0.7(2)	C13	O12	C2	C1	-178.9(2)
C4	S5	C6	C14	-178.1(2)	C13	O12	C2	C3	0.2(3)

Table 7 Hydrogen Atom Coordinates (Å×10[4]) and Isotropic Displacement Parameters (Å[2]×10[3]) for exp_235_auto.

Atom	*x*	*y*	*z*	U(eq)
H3	6668.99	1871.19	6112.6	61
H9	2240.27	4260.27	4672.91	59
H11A	3856.54	3651.25	2622.97	90
H11B	4294.63	4295.84	3370.74	90
H11C	1050.3	3733.44	3297.59	90
H13A	9887.58	709.87	4440.58	91
H13B	7063.79	735.5	5114.85	91
H13C	10431.3	1228.73	5272.3	91

Single crystal X-ray diffraction data for compound 3g

Table 1 Crystal data and structure refinement for exp_233_auto.

Identification code	exp_233_auto
Empirical formula	$C_{10}H_9IN_2O_3$
Formula weight	332.09
Temperature/K	297.00(10)
Crystal system	monoclinic
Space group	$P2_1/c$
a/Å	14.7562(4)
b/Å	4.73210(10)
c/Å	16.9571(4)
α/°	90
β/°	101.469(2)
γ/°	90
Volume/Å^3	1160.43(5)
Z	4
ρ_{calc}g/cm^3	1.901
μ/mm^{-1}	21.669
F(000)	640.0
Crystal size/mm^3	0.1 × 0.04 × 0.02
Radiation	Cu Kα (λ = 1.54184)
2Θ range for data collection/°	6.112 to 155.32
Index ranges	-18 ≤ h ≤ 18, -5 ≤ k ≤ 4, -20 ≤ l ≤ 21
Reflections collected	11384
Independent reflections	2300 [R_{int} = 0.0338, R_{sigma} = 0.0224]
Data/restraints/parameters	2300/0/147
Goodness-of-fit on F^2	1.051
Final R indexes [I>=2σ (I)]	R_1 = 0.0288, wR_2 = 0.0742
Final R indexes [all data]	R_1 = 0.0316, wR_2 = 0.0761
Largest diff. peak/hole / e Å^{-3}	0.83/-0.49

Table 2 Fractional Atomic Coordinates (×10^4) and Equivalent Isotropic Displacement Parameters (Å^2×10^3) for exp_233_auto. U_{eq} is defined as 1/3 of the trace of the orthogonalised U_{IJ} tensor.

Atom	*x*	*y*	*z*	U(eq)
I7	9107.1(2)	11591.2(5)	6840.8(2)	60.12(12)
O13	8972.4(19)	12367(7)	4999.6(17)	64.2(7)
O15	6515(2)	5696(6)	3980.2(14)	59.8(7)
N8	6374(2)	4260(6)	5422.5(16)	44.1(6)
O12	6523(2)	3931(6)	6790.9(15)	69.9(8)

Table 2 Fractional Atomic Coordinates (×10⁴) and Equivalent Isotropic Displacement Parameters (Å²×10³) for exp_233_auto. U_{eq} is defined as 1/3 of the trace of the orthogonalised U_{IJ} tensor.

Atom	*x*	*y*	*z*	U(eq)
N11	4971(3)	-832(8)	5927(2)	68.8(9)
C6	7602(2)	7695(7)	5993.1(19)	42.6(7)
C2	8315(2)	10408(8)	5077(2)	48.8(8)
C5	7033(2)	6377(6)	5350.9(19)	41.6(7)
C3	7737(3)	9139(7)	4433(2)	50.6(8)
C10	5487(3)	931(8)	5981(2)	52.0(8)
C1	8238(2)	9696(7)	5858(2)	46.4(7)
C9	6191(3)	3231(7)	6113(2)	47.6(8)
C4	7096(2)	7133(7)	4562.5(19)	45.7(7)
C14	9135(3)	12893(10)	4213(3)	76.2(13)
C16	6458(4)	6495(11)	3160(2)	77.4(14)

Table 3 Anisotropic Displacement Parameters (Å²×10³) for exp_233_auto. The Anisotropic displacement factor exponent takes the form: $-2\pi^2[h^2a^{*2}U_{11}+2hka^*b^*U_{12}+\ldots]$.

Atom	U_{11}	U_{22}	U_{33}	U_{23}	U_{13}	U_{12}
I7	58.24(17)	59.73(18)	56.28(17)	-4.8(1)	-3.41(11)	-0.11(11)
O13	67.1(16)	63.2(16)	65.8(17)	2.4(14)	21.7(13)	-16.3(14)
O15	87.9(18)	56.9(14)	33.2(11)	-1.5(11)	8.5(11)	-15.9(14)
N8	56.4(16)	39.4(14)	36.6(13)	-0.3(11)	9.6(11)	-3.9(12)
O12	101(2)	71.3(18)	39.2(13)	-1.1(12)	18.9(14)	-26.2(16)
N11	73(2)	67(2)	70(2)	0.3(18)	24.3(18)	-17.3(19)
C6	51.4(17)	40.3(16)	36.2(15)	2.2(13)	9.0(13)	4.8(14)
C2	53.8(19)	43.3(18)	52.4(18)	2.2(15)	17.7(15)	3.3(15)
C5	53.5(18)	34.3(16)	37.8(15)	1.2(12)	11.3(13)	3.6(13)
C3	66(2)	46.5(18)	43.3(17)	4.3(15)	20.1(15)	1.3(17)
C10	64(2)	49(2)	45.7(18)	2.7(15)	18.2(16)	0.8(18)
C1	47.8(17)	42.4(17)	47.2(17)	-1.3(14)	5.3(13)	3.9(14)
C9	61(2)	42.6(18)	41.3(17)	1.2(14)	16.2(15)	-1.1(15)
C4	63(2)	39.3(17)	35.3(15)	-0.2(13)	10.2(14)	1.7(15)
C14	82(3)	78(3)	79(3)	7(2)	42(3)	-11(3)
C16	106(4)	94(4)	32.1(17)	-2.0(19)	13.1(19)	-15(3)

Table 4 Bond Lengths for exp_233_auto.

Atom	Atom	Length/Å	Atom	Atom	Length/Å
I7	C1	2.091(3)	N11	C10	1.121(5)
O13	C2	1.367(4)	C6	C5	1.384(5)
O13	C14	1.424(5)	C6	C1	1.384(5)
O15	C4	1.355(4)	C2	C3	1.382(5)
O15	C16	1.428(4)	C2	C1	1.394(5)
N8	C5	1.418(4)	C5	C4	1.405(4)
N8	C9	1.344(4)	C3	C4	1.387(5)
O12	C9	1.203(4)	C10	C9	1.490(5)

Table 5 Bond Angles for exp_233_auto.

Atom	Atom	Atom	Angle/°	Atom	Atom	Atom	Angle/°
C2	O13	C14	117.6(3)	N11	C10	C9	176.1(4)
C4	O15	C16	118.9(3)	C6	C1	I7	119.4(2)
C9	N8	C5	126.2(3)	C6	C1	C2	120.5(3)
C1	C6	C5	120.3(3)	C2	C1	I7	120.1(3)
O13	C2	C3	123.9(3)	N8	C9	C10	112.9(3)
O13	C2	C1	116.6(3)	O12	C9	N8	128.2(3)
C3	C2	C1	119.5(3)	O12	C9	C10	118.9(3)
C6	C5	N8	124.8(3)	O15	C4	C5	114.4(3)
C6	C5	C4	119.3(3)	O15	C4	C3	125.5(3)
C4	C5	N8	116.0(3)	C3	C4	C5	120.1(3)
C2	C3	C4	120.4(3)				

Table 6 Torsion Angles for exp_233_auto.

A	B	C	D	Angle/°	A	B	C	D	Angle/°
O13	C2	C3	C4	178.9(3)	C5	C6	C1	C2	0.0(5)
O13	C2	C1	I7	-0.2(4)	C3	C2	C1	I7	180.0(3)
O13	C2	C1	C6	-178.9(3)	C3	C2	C1	C6	1.3(5)
N8	C5	C4	O15	-0.5(4)	C1	C6	C5	N8	178.6(3)
N8	C5	C4	C3	-178.5(3)	C1	C6	C5	C4	-1.3(5)
C6	C5	C4	O15	179.4(3)	C1	C2	C3	C4	-1.2(5)
C6	C5	C4	C3	1.3(5)	C9	N8	C5	C6	0.8(5)
C2	C3	C4	O15	-177.9(3)	C9	N8	C5	C4	-179.4(3)
C2	C3	C4	C5	-0.1(5)	C14	O13	C2	C3	-7.0(6)
C5	N8	C9	O12	2.1(6)	C14	O13	C2	C1	173.1(3)
C5	N8	C9	C10	-177.5(3)	C16	O15	C4	C5	173.9(4)

Table 6 Torsion Angles for exp_233_auto.

A	B	C	D	Angle/°	A	B	C	D	Angle/°
C5	C6	C1	I7	-178.7(2)	C16	O15	C4	C3	-8.2(6)

Table 7 Hydrogen Atom Coordinates (Å×10^4) and Isotropic Displacement Parameters (Å^2×10^3) for exp_233_auto.

Atom	*x*	*y*	*z*	U(eq)
H8	6061.67	3568.71	4982.31	53
H6	7556.47	7234.69	6516.95	51
H3	7777.1	9632.55	3910.41	61
H14A	8587.66	13676.4	3883.62	114
H14B	9289.45	11153.11	3979.86	114
H14C	9637.1	14204.72	4243.92	114
H16A	5947.91	5534.62	2827.67	116
H16B	7021.49	5987.92	2993.31	116
H16C	6366.46	8499.8	3106.39	116

^{1}H NMR ($CDCl_3$) spectrum of (3-methoxy-[1,1'-biphenyl]-4-yl)carbamoyl cyanide

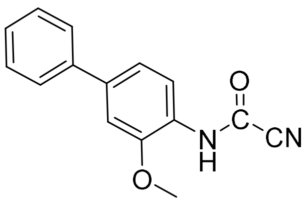

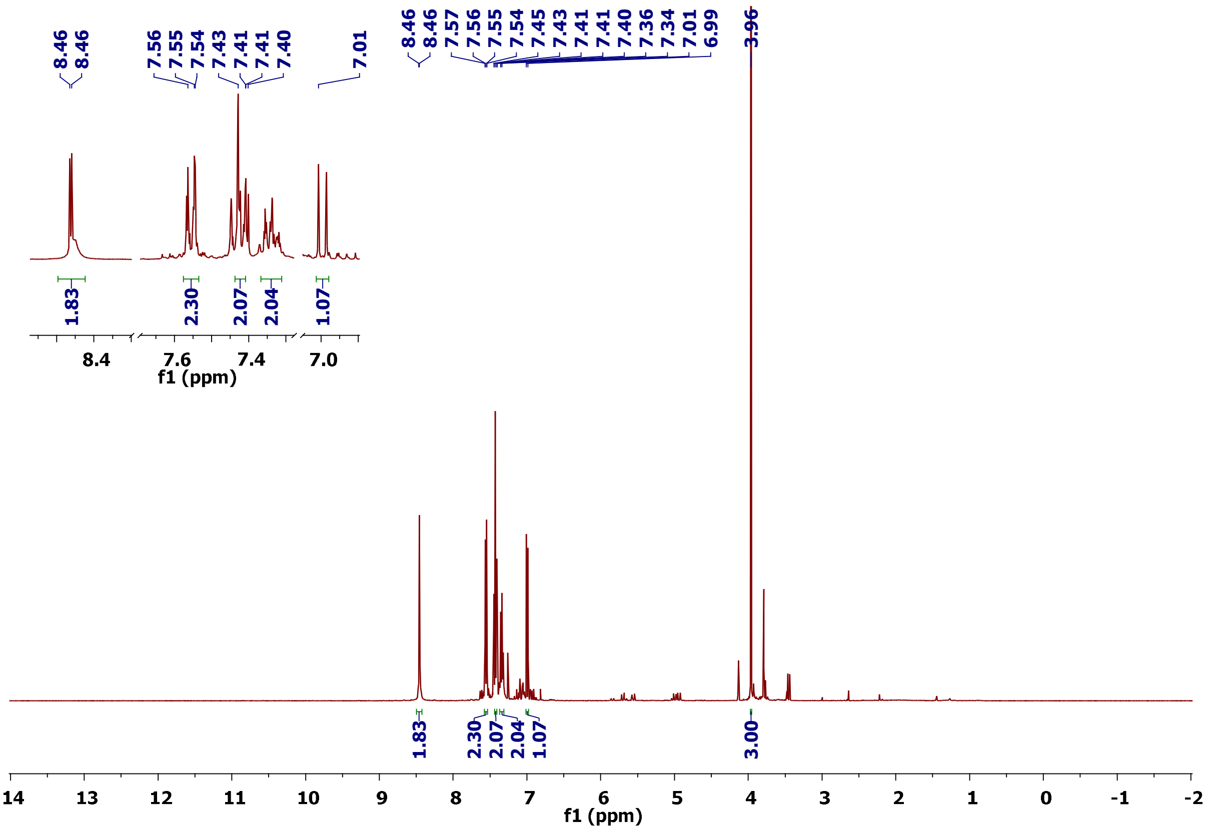

^{13}C NMR ($CDCl_3$) spectrum of (3-methoxy-[1,1'-biphenyl]-4-yl)carbamoyl cyanide

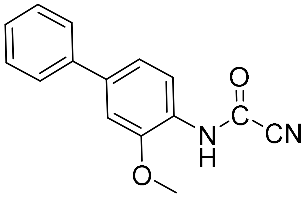

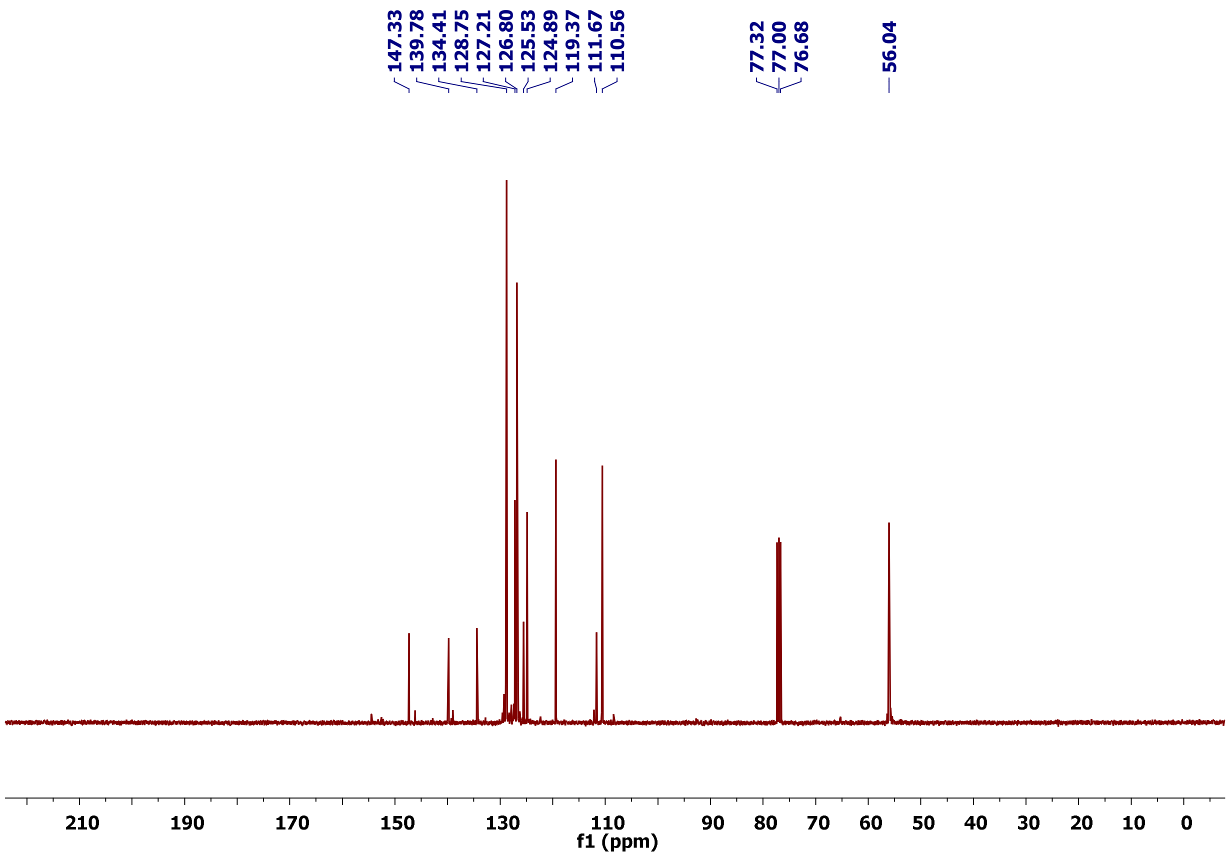

^{13}C-CRAPT NMR ($CDCl_3$) spectrum of (3-methoxy-[1,1'-biphenyl]-4-yl)carbamoyl cyanide

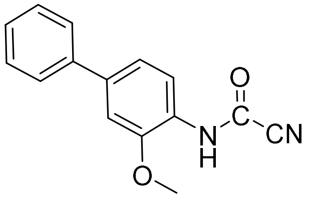

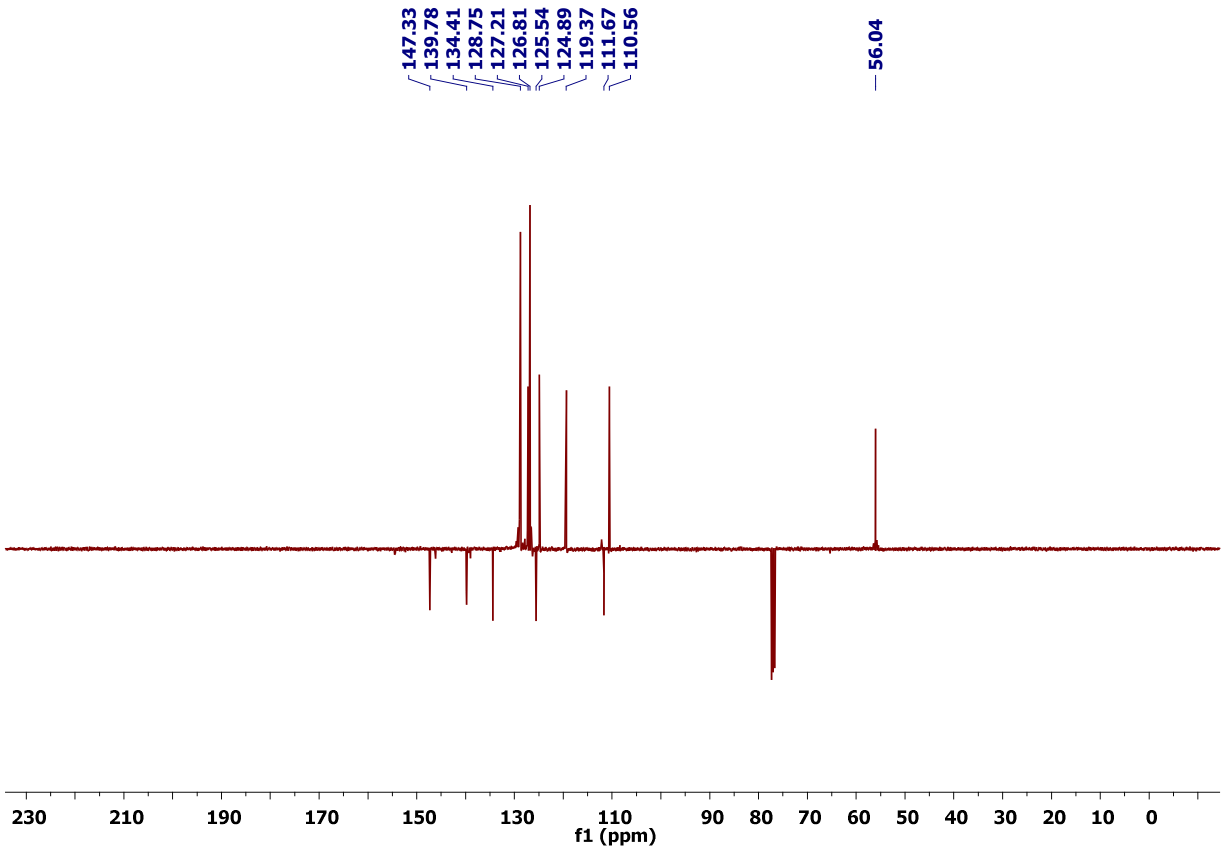

^{1}H-^{1}H-gDQFCOSY NMR ($CDCl_3$) spectrum of (3-methoxy-[1,1'-biphenyl]-4-yl)carbamoyl cyanide

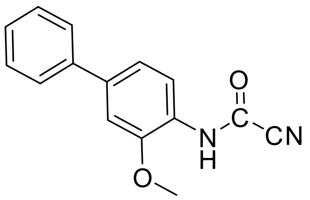

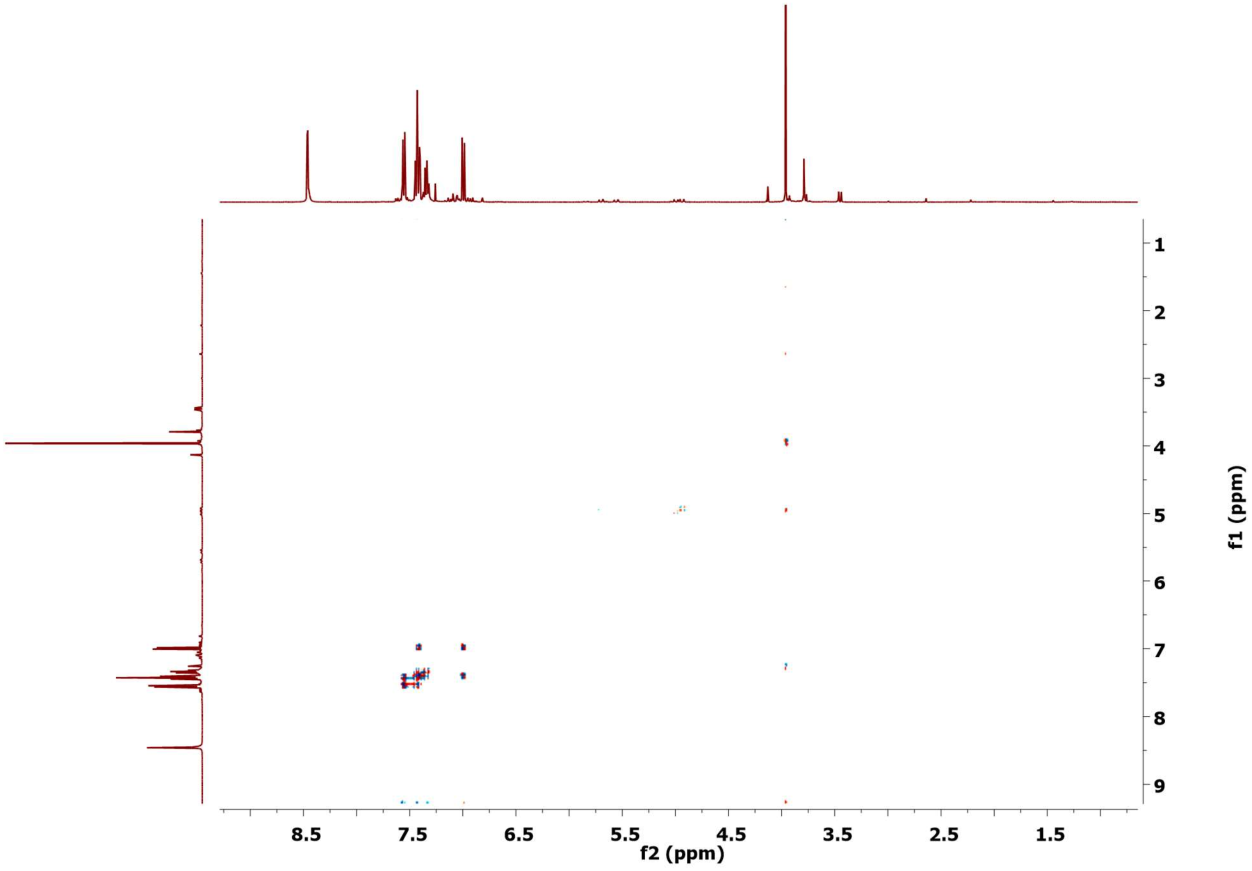

^{1}H-^{13}C-gHSQCAD NMR ($CDCl_3$) spectrum of (3-methoxy-[1,1'-biphenyl]-4-yl)carbamoyl cyanide

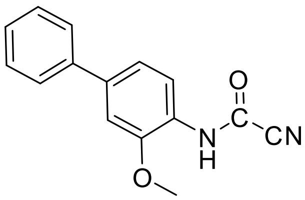

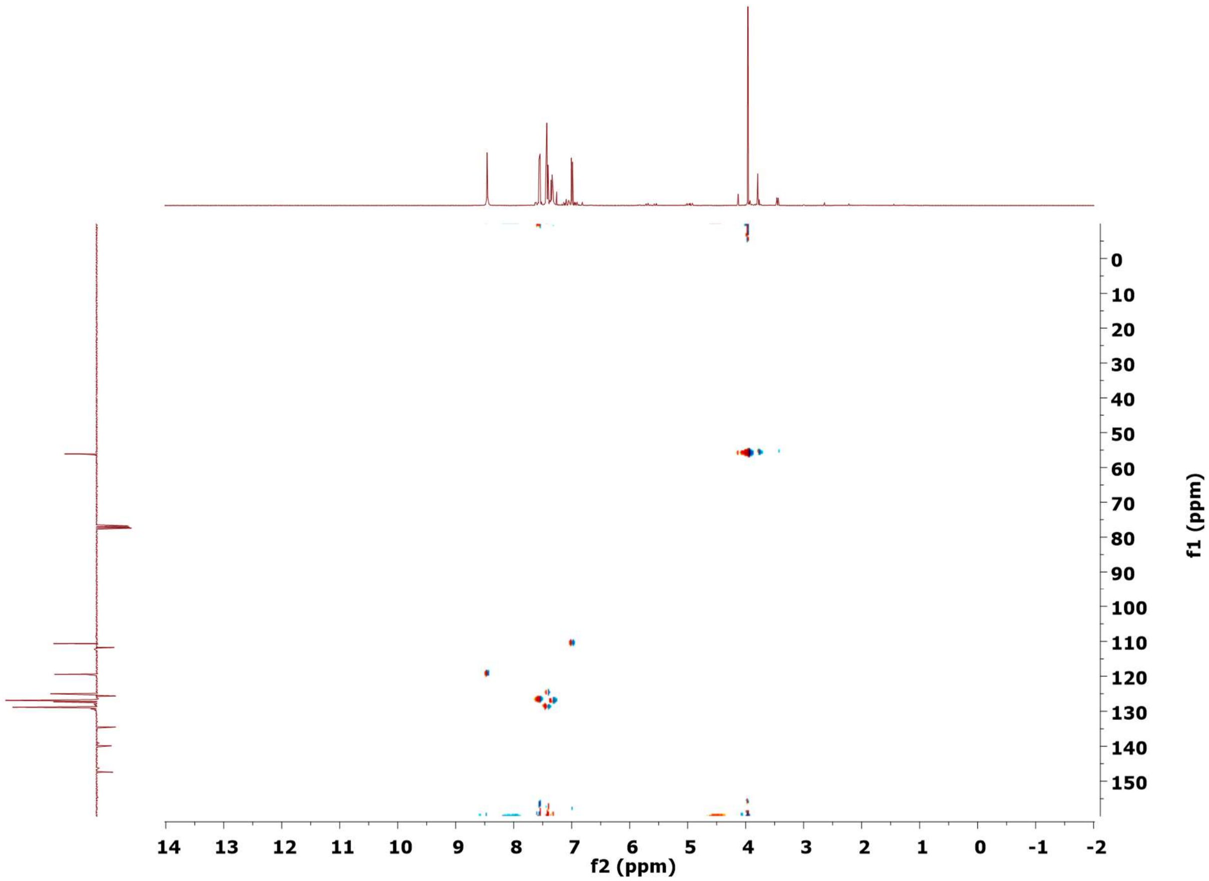

^{1}H-^{13}C-gHMBC NMR ($CDCl_3$) spectrum of (3-methoxy-[1,1'-biphenyl]-4-yl)carbamoyl cyanide

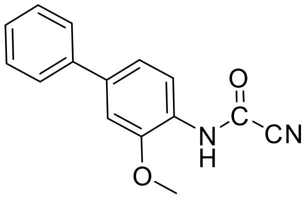

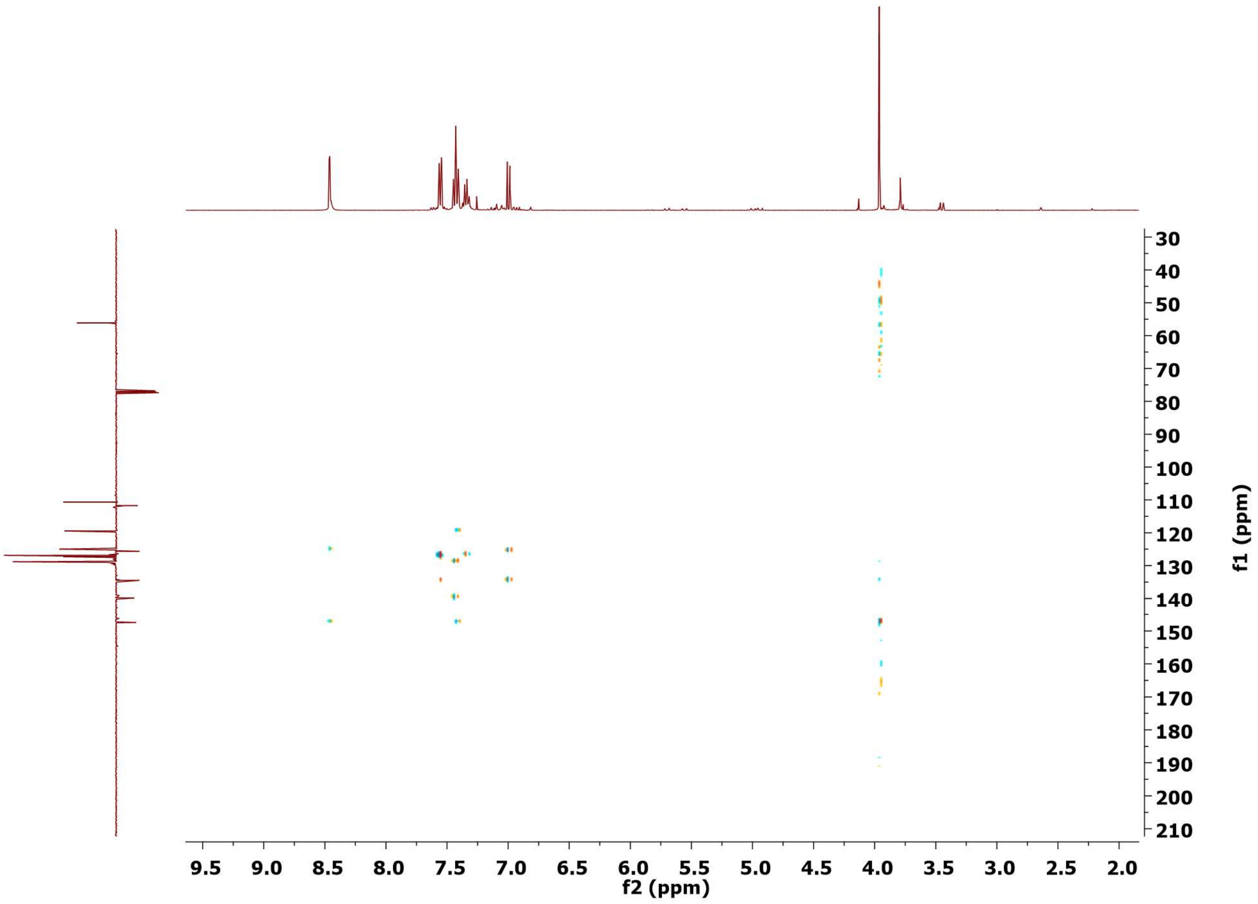

^{1}H NMR ($CDCl_3$) spectrum of (2-iodophenyl)carbamothioyl cyanide

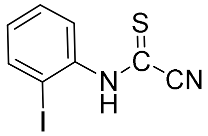

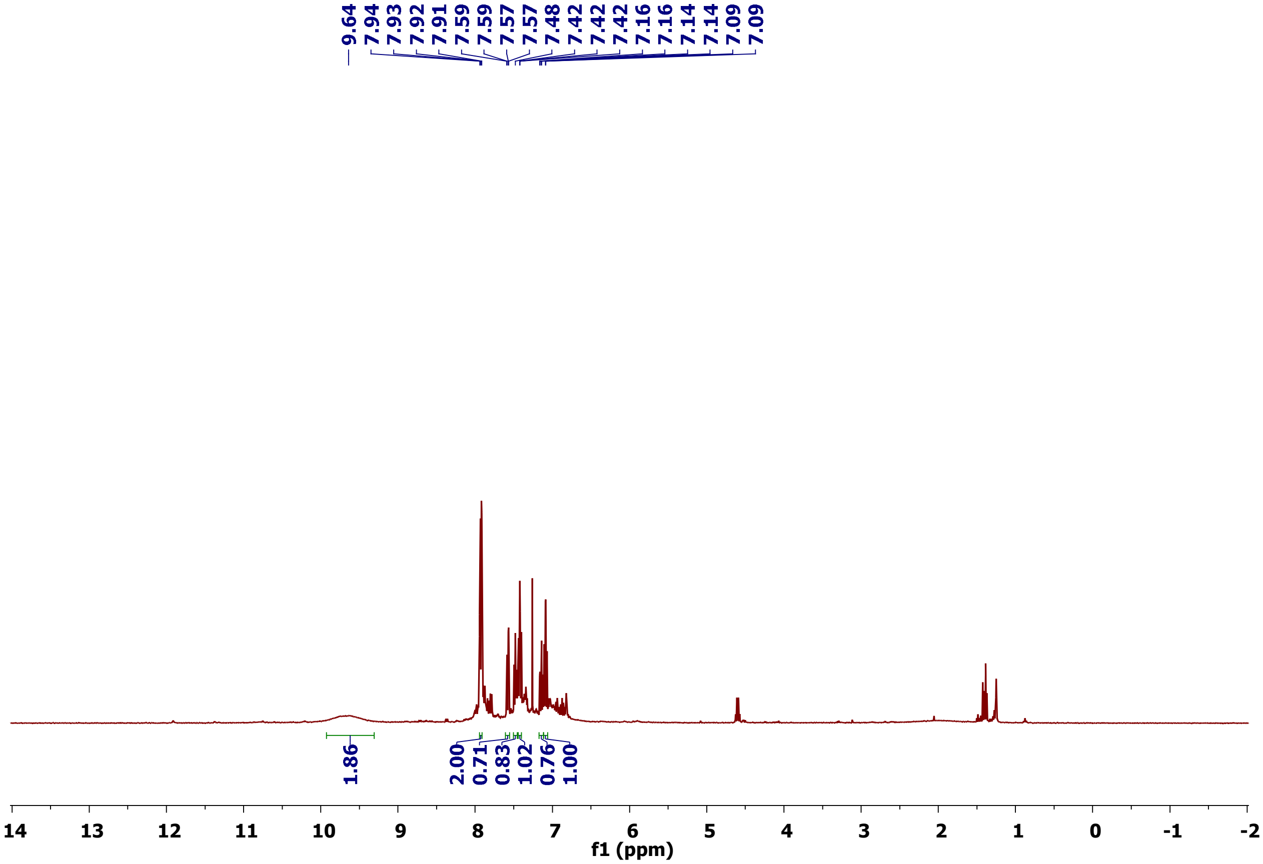

^{13}C NMR ($CDCl_3$) spectrum of (2-iodophenyl)carbamothioyl cyanide

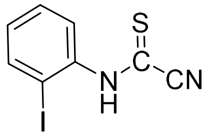

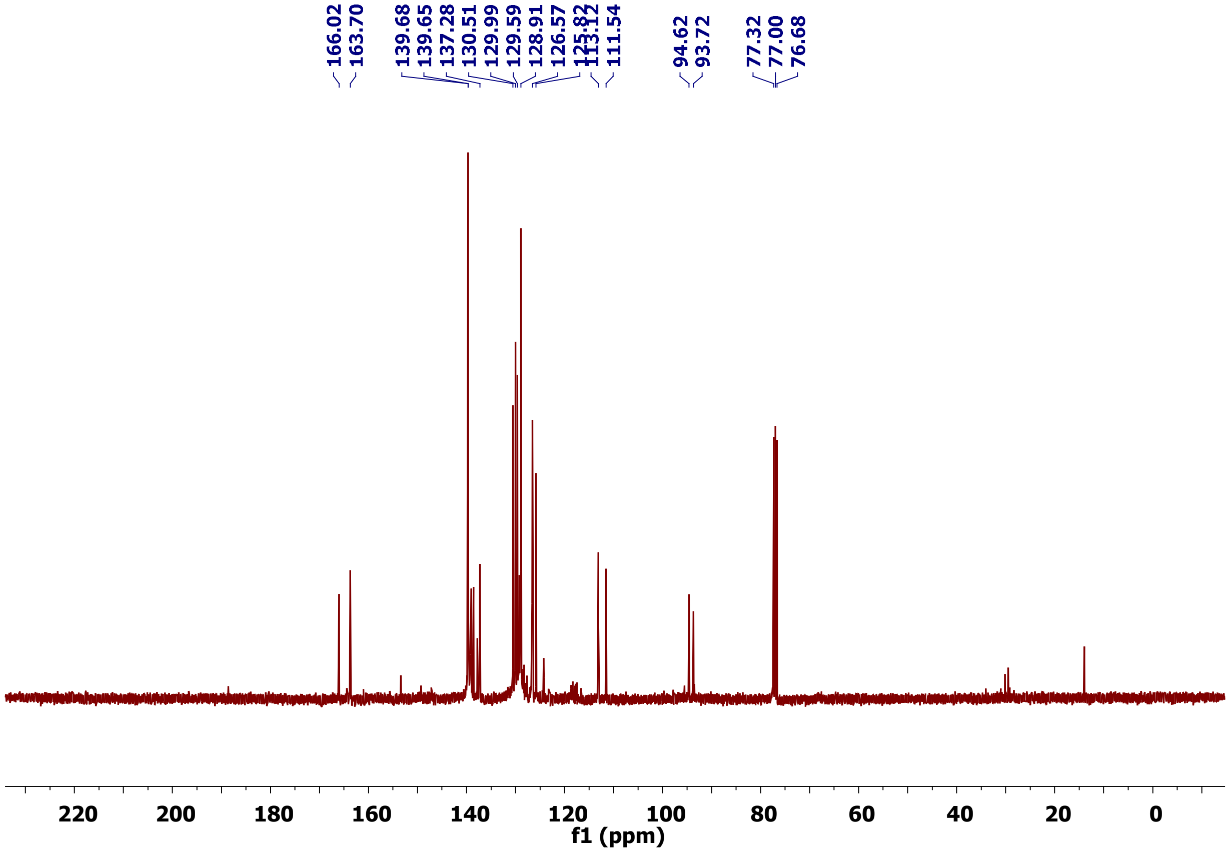

^{13}C-CRAPT NMR ($CDCl_3$) spectrum of (2-iodophenyl)carbamothioyl cyanide

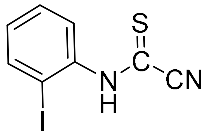

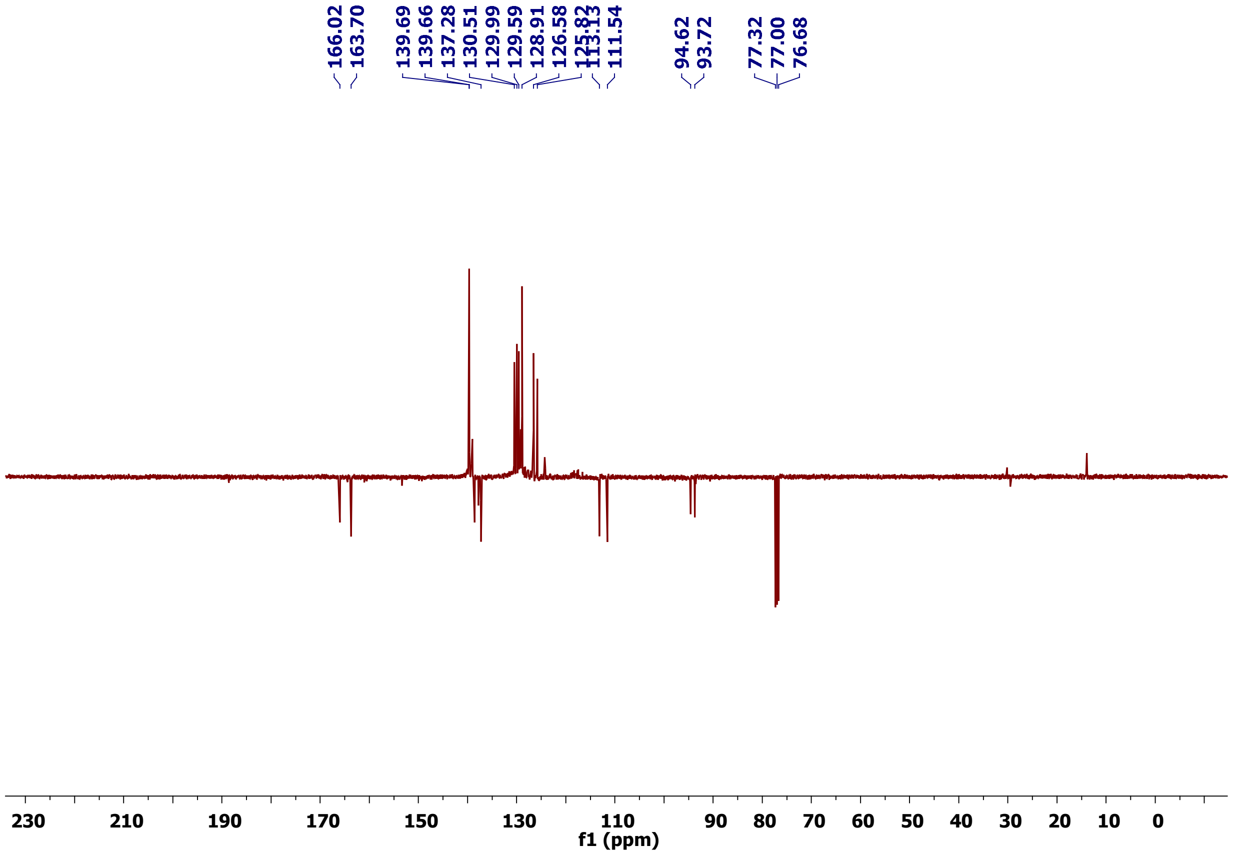

^{1}H-^{1}H-gDQFCOSY NMR ($CDCl_3$) spectrum of (2-iodophenyl)carbamothioyl cyanide

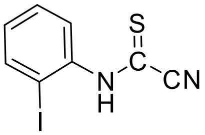

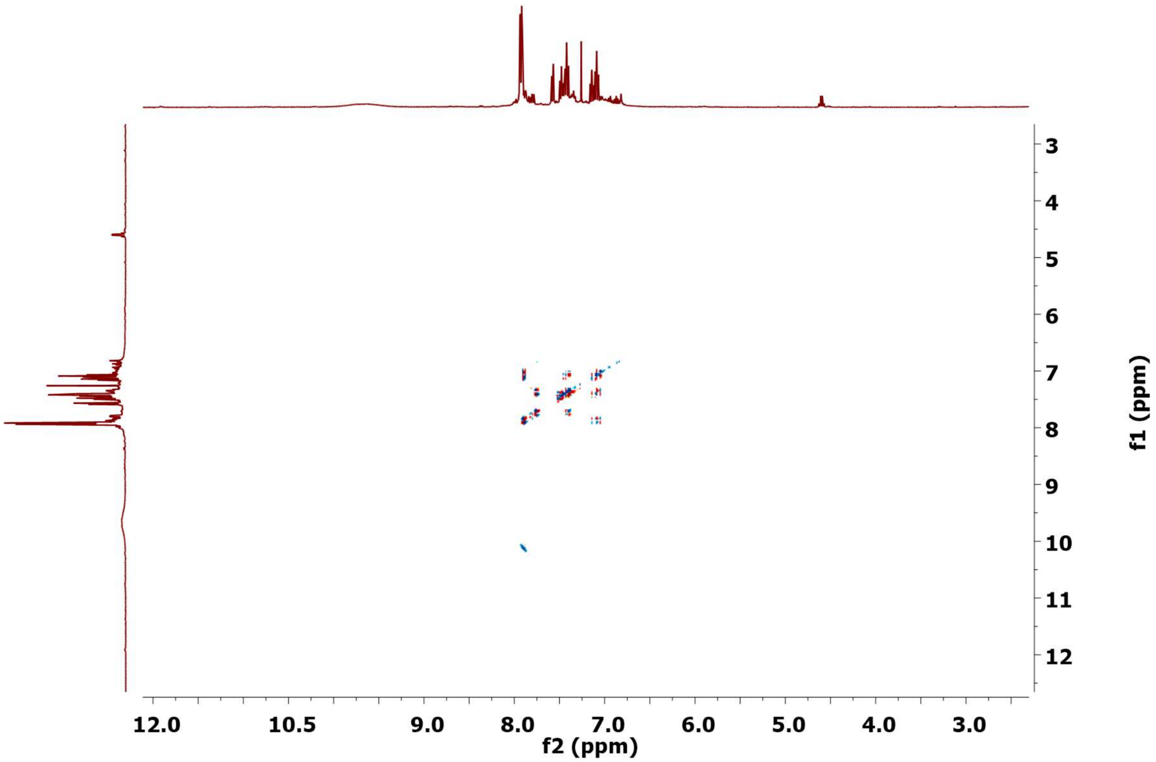

^{1}H-^{13}C-gHSQCAD NMR ($CDCl_3$) spectrum of (2-iodophenyl)carbamothioyl cyanide

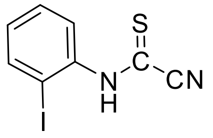

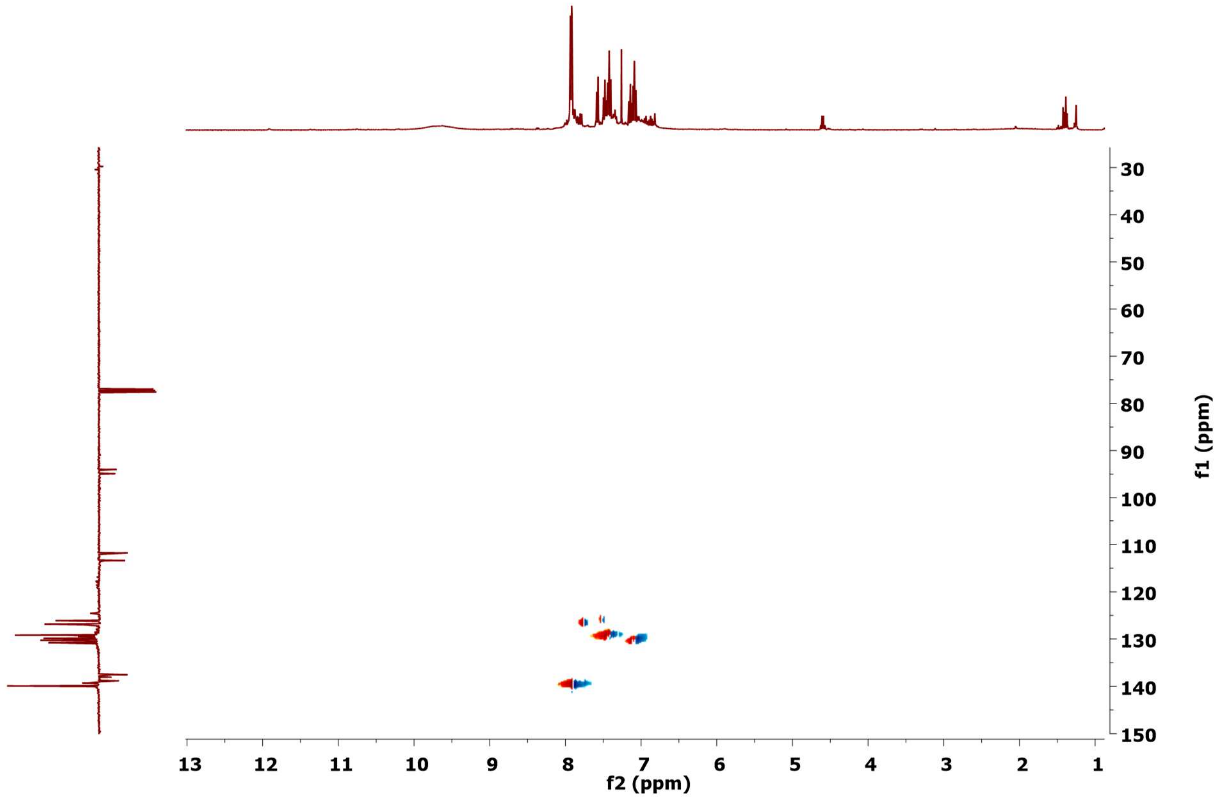

^{1}H-^{13}C-gHMBC NMR ($CDCl_3$) spectrum of (2-iodophenyl)carbamothioyl cyanide

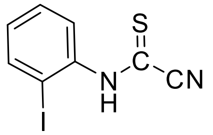

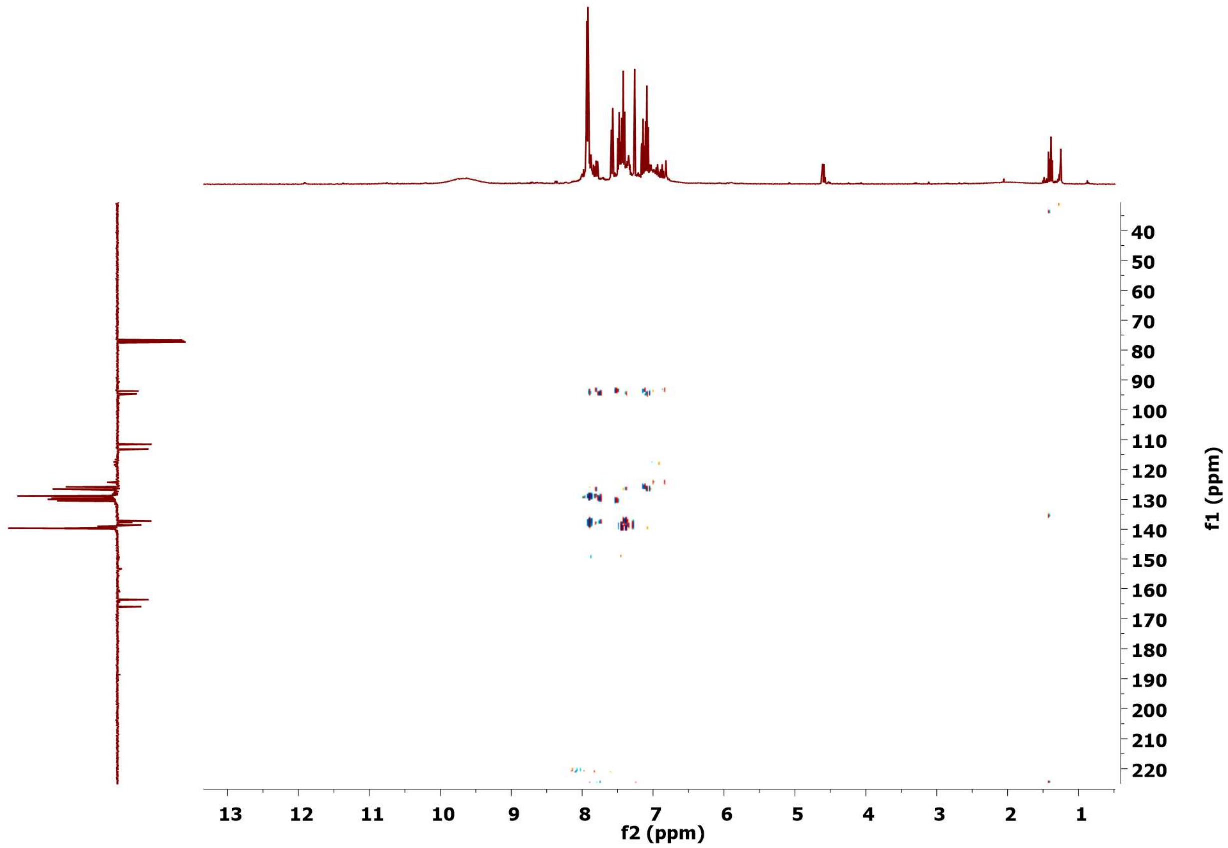

Supplement: RA-012-D2RA00049K-s001 [file RA-012-D2RA00049K-s001.pdf]
